# Coping with living in the soil: the genome of the parthenogenetic springtail *Folsomia candida*

**DOI:** 10.1186/s12864-017-3852-x

**Published:** 2017-06-28

**Authors:** Anna Faddeeva-Vakhrusheva, Ken Kraaijeveld, Martijn F. L. Derks, Seyed Yahya Anvar, Valeria Agamennone, Wouter Suring, Andries A. Kampfraath, Jacintha Ellers, Giang Le Ngoc, Cornelis A. M. van Gestel, Janine Mariën, Sandra Smit, Nico M. van Straalen, Dick Roelofs

**Affiliations:** 1 Department of Ecological Science, Vrije Universiteit Amsterdam, Amsterdam, The Netherlands; 2 Department of Animal Sciences, Animal Breeding and Genetics, Wageningen University, Wageningen, The Netherlands; 3 Department of Human Genetics, Leiden University Medical Center, Leiden, The Netherlands; 4 Leiden Genome Technology Center, Leiden University Medical Center, Leiden, The Netherlands; 5 Department of Plant Sciences, Bioinformatics Group, Wageningen University, Wageningen, The Netherlands; 6 Institute of Biotechnology, Vietnam Academy of Science and Technology, Hanoi, Vietnam

**Keywords:** Collembola, Intragenomic rearrangement, Gene family expansions, *Hox* genes, Horizontal gene transfer, Genome collinearity, Carbohydrate metabolism, Palindrome

## Abstract

**Background:**

*Folsomia candida* is a model in soil biology, belonging to the family of Isotomidae, subclass Collembola. It reproduces parthenogenetically in the presence of *Wolbachia*, and exhibits remarkable physiological adaptations to stress. To better understand these features and adaptations to life in the soil, we studied its genome in the context of its parthenogenetic lifestyle.

**Results:**

We applied Pacific Bioscience sequencing and assembly to generate a reference genome for *F. candida* of 221.7 Mbp, comprising only 162 scaffolds. The complete genome of its endosymbiont *Wolbachia*, was also assembled and turned out to be the largest strain identified so far. Substantial gene family expansions and lineage-specific gene clusters were linked to stress response. A large number of genes (809) were acquired by horizontal gene transfer. A substantial fraction of these genes are involved in lignocellulose degradation. Also, the presence of genes involved in antibiotic biosynthesis was confirmed. Intra-genomic rearrangements of collinear gene clusters were observed, of which 11 were organized as palindromes. The *Hox* gene cluster of *F. candida* showed major rearrangements compared to arthropod consensus cluster, resulting in a disorganized cluster.

**Conclusions:**

The expansion of stress response gene families suggests that stress defense was important to facilitate colonization of soils. The large number of HGT genes related to lignocellulose degradation could be beneficial to unlock carbohydrate sources in soil, especially those contained in decaying plant and fungal organic matter. Intra- as well as inter-scaffold duplications of gene clusters may be a consequence of its parthenogenetic lifestyle. This high quality genome will be instrumental for evolutionary biologists investigating deep phylogenetic lineages among arthropods and will provide the basis for a more mechanistic understanding in soil ecology and ecotoxicology.

**Electronic supplementary material:**

The online version of this article (doi:10.1186/s12864-017-3852-x) contains supplementary material, which is available to authorized users.

## Background

The soil environment harbors communities that are functionally important and abundant, as well as biologically extremely diverse. In addition, the greater part of soil microbes and invertebrates remains unknown and has been qualified as the “unseen majority” [1]. To reveal the functionalities of soil organisms, genome information may be of help. Until now such information is mostly limited to nematodes, representatives of the (microscopic) microfauna and a few macrofaunal species, such as the earthworm *Lumbricus rubellus* [2]. Previously, we studied the genome sequence of a representative of the mesofauna, the springtail *Orchesella cincta*, which is an obligate sexual reproducing species belonging to the family of Entomobryidae [3]. In this paper, we focus on the parthenogenetic species *Folsomia candida,* belonging to the family of Isotomidae.

**Fig. 1:**
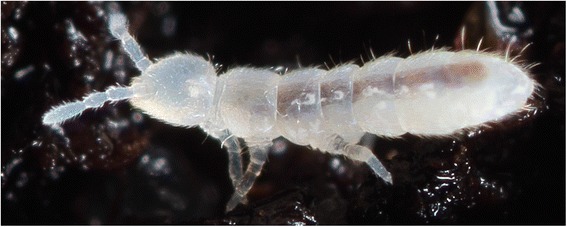
A specimen of the parthenogenetic species *Folsomia candida*. It has a slender, white body shape on average 2.5 mm long. It has a fully developed furca that is used for jumping. The chewing type of mandibula has a molar plate present. *F. candida* is a euedaphic species, thriving inside the soil. Photo by Jan van Duinen

*F. candida* (Fig. 1) is a soil-dwelling invertebrate that belongs to the hexapod subclass Collembola (springtails), which shares the most recent common ancestor with insects [4]. Collembola are one of the most abundant arthropods. They inhabit soil and leaf litter layers and they have radiated into many niches [5]. For many years, *F. candida* has been used as a model organism for standardized toxicity tests [6, 7]. *F. candida* has a high rate of reproduction and is easy to culture, which makes it suitable for laboratory testing.

The genome of *F. candida* is diploid and consists of seven chromosome pairs [8]. It has been suggested that the sex determination is diplo-diploid (XX/XO) [8, 9], although definite proof of this is still lacking. Overall, *F. candida* reproduces through parthenogenesis, although bisexual populations have been observed [10]. Riparbelli et al. [11] have shown that the oocyte is able to self-assemble microtubule-based asters to support centrosome formation and successfully complete meiosis I. The highly unusual spindle structures in subsequent rounds of mitosis may cause maintenance of diploidy in the absence of fertilization [11]. Parthenogenesis is most likely imposed by *Wolbachia*, an endosymbiotic bacterium located in the ovaries and brain of the animal [10]. In fact, the presence of *Wolbachia* is essential for reproduction: animals cured of *Wolbachia* by antibiotic treatment lay eggs that fail to hatch and develop [12, 13]. Despite these observations, causal evidence of *Wolbachia*-mediated induction of parthenogenesis is currently lacking [14].

In the soil ecosystem, desiccation stress is an important selective factor [15]. *F. candida* is adapted to cope with moderately dry soil conditions through a unique system of water vapor absorption. The animal maintains all body fluids hyperosmotic to its surroundings to allow net water uptake from the atmosphere by passive diffusion along the gradient in water potential. Below a relative air humidity of 95% the animal actively increases the osmotic pressure of its body fluids by a production of sugars and polyols [16]. Indeed, a transcriptome study performed by Timmermans et al. [17] confirmed the involvement of sugar metabolism in drought adaptation.

Collembolans are a globally significant group of organisms that play a major role in soil functioning [18], particularly through their effect on the rate of litter decomposition and nutrient fluxes [19]. *F. candida* prefers grazing on fungi growing on organic surfaces rather than on soil particles [5], which has been suggested to stimulate the spread of fungal infections among plants [20]. These plants benefit from a moderate amount of grazing activity by having an increased transfer of spores of essential arbuscular mycorrhizal fungi (AMF) from other plants [21]. More recently, plants have been shown to transfer feeding repellents to their mycorrhizae when the fungal hyphae of AMF are grazed by *F. candida* [22]. This indicates a tritrophic interaction between plant, AMF, and *F. candida* potentially improving soil fertility and plant production.

In this study, we describe the first high-quality reference genome of *F. candida*, assembled from Pacific Bioscience SMRT long sequence reads. We pay special attention to features of the genome that may be associated with a parthenogenetic lifestyle inside the soil. We focus on expanded and lineage-specific gene families as well as horizontal gene transfer (HGTs), and genes related to stress tolerance. We also investigated intragenomic rearrangements and discuss potential consequences of parthenogenesis on genome structure and variation. Where possible, we compare data to the already available genome sequence of *O. cincta*. The presented genome will be highly instrumental for evolutionary biologists investigating deep phylogenetic lineages among arthropods and will provide the basis for a more mechanistic understanding in soil ecotoxicology.

## Results and discussion

### *F. candida* Genome features

A total of 1.43 million PacBio reads were generated from 20 SMRT cells with an N50 of 20,147 bp. As such, we used 18.85 Gbp of raw data to assemble a high-quality reference genome of 221.7 Mbp in only 162 scaffolds with an N50 of 6.5 Mbp and a maximum uninterrupted sequence length of 28.5 Mbp (Table 1). Gaps occupied only 0.1% of all bases. In total, 28,734 gene models could be predicted, which were supported by various experimental data (see section below). We identified 254,312 repeat segments that cover 23.3% (bp) of the genome. The most abundant types of repeats were simple repeats, unclassified, low complexity repeats, and TcMar DNA transposons (Additional file 1). Single-nucleotide polymorphisms (SNP) density was very low and predicted to be 0.148 per kbp. The GC content of the *F. candida* nuclear genome was 37.5%, which is slightly higher than in the other published collembolan genome of *Orchesella cincta* [3].

**Table 1:** *Folsomia candida* genome properties

Assembly	
Total sequences	162
Total bases (Mbp)	221.7
Min sequence length (bp)	2433
Max sequence length (Mbp)	28.5
N50 length (Mbp)	6.5
GC %	37.5
Structural Annotation	
Genes	28,734
Mean gene length (bp)	4615
Exon (%)	31.9
Intron (%)	28.0
Repeats (%)	23.3
Functional annotation	
Swiss	16,528
TrEMBL	19,592
InterPro	16,840
Gene Ontology	15,883
Enzyme Codes	5535
Validation	
CEGMA complete	245 (98.8%)
CEGMA complete + partial	246 (99.2%)
BUSCO complete	363 (84%)
BUSCO complete + partial	377 (87%)

Blobology analysis revealed 13 contigs with deviating GC content, which could be the result of contamination. The GC content in scaffold 160 was only 29.9%. Blast searches in the NCBI non-redundant database yielded no significant hits to non-metazoan sequences for this scaffold, suggesting that this scaffold is not a result of contamination. Instead, visual inspection revealed stretches of low base pair calling. (N stretches) along this scaffold, which resulted in a biased GC estimate. The remaining 12 scaffolds show GC contents of approximately 50%. Blast searches indicated that these scaffolds comprised long stretches of GC-rich repeat sequences without any indication for contamination.

The high quality of the genome assembly and the 28,734 gene models were supported by various lines of evidence. First, we identified 245 complete genes and one partial gene out of 248 (98.8%) core eukaryotic CEGMA predicted gene models, which is a good indicator of completeness of the assembled gene space. Subsequently, we applied Benchmarking Universal Single-Copy Orthologs (BUSCO) analysis against the eukaryote dataset and identified 363 (84%) complete single copy orthologs, of which 116 (27%) were duplicated, and 14 (3.2%) were fragmented, while 52 (12%) were missing. Also, 37,680 (99.1%) previously assembled de novo transcripts [23] mapped to the genome. Finally, more than 96% of PacBio long reads and Illumina reads mapped to the final genome scaffolds (Table 1).

Gene density in the *F. candida* genome was 129.6 genes per Mbp, which is 1.8 times higher than the gene density in the genome of the recently sequenced Collembola species *O. cincta* [3], but less than in *Daphnia pulex* (192.9 genes per Mbp) [24]. Overall, the coding sequences covered 31.9% of the total genome. Interpro domains were identified for 16,840 (58.6%) predicted proteins and 20,179 (70.2%) predicted proteins showed similarity to proteins from other species in SwissProt or TrEMBL databases. Also, 15,913 (55.4%) genes were supported by RNA-Seq data with a fraction per kilobase of exon per million fragments (FPKM) of at least 0.5. Moreover, gene ontology (GO) terms could be assigned to 15,883 (55.3%) gene models (Additional file 2), of which 5535 (19.3%) were associated with enzyme codes (EC).

The assembly also included a single scaffold for the mitochondrial genome of *F. candida*. It is 15,147 bp long and consists of 22 tRNA genes, 13 protein-coding genes, and 2 rRNA genes (Additional file 1). The mitochondrial (mt) genome of *F. candida* showed the highest sequence identity (75%) and coverage (92%) with the mt genome of the collembolan *Cryptopygus antarcticus*. Moreover, size, AT percentage and gene content were highly comparable to *C. antarcticus* as well as to the mt genome of *O. cincta*, suggesting monophyly of mt genomes within this animal group.

Additionally, we assembled the complete genome of the *Wolbachia* endosymbiont of *F. candida* (*wFol*) in a single scaffold with a size of 1,801,583 bp and GC content of 34.84%. A total of 48 ankyrin (ANK) repeat-containing genes, 35 tRNA genes, 1627 protein coding genes and 3 rRNA genes were identified in the *wFol* genome (Additional file 1). The presence of ANK repeats in facultative intracellular prokaryotes may be explained by their typical involvement in mediating protein-protein and protein-DNA interactions with the host cells. Such interactions can modulate transcriptional regulation, signal transduction, inflammation response and protein transport in such a way that the endosymbiont is retained in the host [25]. The highest number of ANK genes (60) in a prokaryote has been reported for the *Wolbachia* strain residing in *Culex pipiens*, where they show sex-specific expression, implying sex-specific interaction with their host [26]. The authors suggest that ANK genes play a role in the feminization process associated with *Wolbachia* infection. Gene expression profiling of the *wFol* genome will be necessary to elucidate whether ANK genes play such a role in *F. candida*. Interestingly, eight *Wolbachia* ANK genes were identified to be horizontally transferred into the *F. candida* genome (Additional file 1). However, these ANK genes showed low protein identity with the ones identified in *wFol* genome, indicating ancient HGT events.

Recently, Gerth et al. [27] showed that *wFol* belongs to supergroup E, which is a sister group ancestral to all fully assembled *Wolbachia* genomes represented in supergroups A to D, F and H. Here, we show that *wFol* has the largest genome size of all *Wolbachia* genomes analyzed so far. This suggests that there was a genome reduction event in the common ancestor of more recently evolving *Wolbachia* strains. Alternatively, the *wFol* genome expanded in size during evolution in the *F. candida* host. Timmermans and Ellers [13] showed that *F. candida* reproduction (egg development) is dependent on *Wolbachia*, while most of the other sequenced *Wolbachia* strains form a facultative relationship with their host. The genome size and gene repertoire of the *wFol* genome thus represents an exception to the general trend that a strong interdependency between an endosymbiont and its host leads to a reduction in the size of the genome of the endosymbiont [28].

### Gene family analysis

Out of the total number of proteins in *F. candida* 18,654 (64.9%) grouped into 7738 gene clusters and 10,080 remained unassigned, although about 35% of these are actively transcribed. Of all gene clusters, 74 were specific for *F. candida* and 1265 for the collembolan lineage (Additional file 1). In total, we predicted 12,030 gene families in *F. candida*, represented by more than one gene. Orthofinder was used to identify ortholog groups among 12 species, where five were considered to be outgroups species to hexapods (*Strigamia maritima*, *Ixodes scapularis, Tetranychus urticae*, *D. pulex* and *Caenorhabditis elegans)*, while the remaining seven are all hexapod ingroup species (*F. candida*, *O. cincta,* and insects *Tribolium castaneum*, *Pediculus humanus*, *Acyrthosiphon pisum*, *Drosophila melanogaster, Aedes aegypti)*. In total, 2444 gene families were shared between *F. candida* and all other species in our analysis.

We identified 368 gene families that were expanded in the *F. candida* genome in relation to the above mentioned species (Additional file 1). Gene ontology analysis identified diverse biological functions among them (Additional file 2). The top five largest expanded families included Sec14-like proteins (154 genes), zinc-finger proteins (123 genes), putative diacylglycerol O-acyltransferase (116 genes), and group 2 allergens (78 genes). Several genes within each of 19 expanded gene clusters were associated with differential expression in response to stress [29]. Among these, the largest gene families included sec14-like proteins, group 2 allergens, ATP-binding cassette (ABC) transporters, glutathione S-transferases (GSTs), and DBH-like monooxygenases/cytochrome p450s. Glutathione S-transferases and ABC transporters participate in phase II xenobiotic metabolism (conjugation of xenobiotic metabolites) and phase III (further modification and excretion of conjugated metabolites). These reactions act to detoxify and remove xenobiotics, plant anti-herbivory toxins, lignocellulose breakdown products, and feeding deterrents. Expansion of these gene families could be an important mechanism of adaptation to the soil environment, where potentially toxic organic compounds persist and accumulate in decaying soil organic matter.

Sec14 proteins are involved in the cytosolic transport of secretory proteins from the Golgi apparatus in yeast [30]. More recent research [31] indicates that Sec14 proteins are essential in membrane trafficking, connecting lipid metabolism with phosphoinositide signaling through transport of phosphatidylinositol. Sec14 proteins contain a CRAL-TRIO lipid-binding domain, and protein families with this domain also show expansion in lepidopteran species [32]. However, the number of Sec14 proteins in both the *F. candida* genome and the genome of a related collembolan, *O. cincta*, exceeds by far the greatest number observed in any hexapod genome (Additional file 1). The major expansion of this gene family in two collembolans suggests an important role for this gene in the evolution of these collembolan species.

Two other expanded families worth mentioning are both ATP-dependent DNA helicase PIF1 families that consisted of 52 and 28 members respectively. ATP-dependent DNA helicase is essential in homologous recombination and DNA repair, which are important functions for successful incorporation and maintenance of DNA after translocation and horizontal gene transfer [33]. Likewise, Spo11, involved in homologous recombination, is expanded in the genome of the bdelloid rotifer *Adineta vaga*, which contains the largest number of horizontally transferred genes of all sequenced metazoans so far [34]. Translocation and horizontal gene transfer seem to have played an important role in *F. candida’s* genome structure (see sections below), and the expanded ATP-dependent DNA helicase PIF1 family could have facilitated abundant horizontal transfer of genes into the *F. candida* genome.

### Horizontal gene transfer analysis

**Fig. 2:**
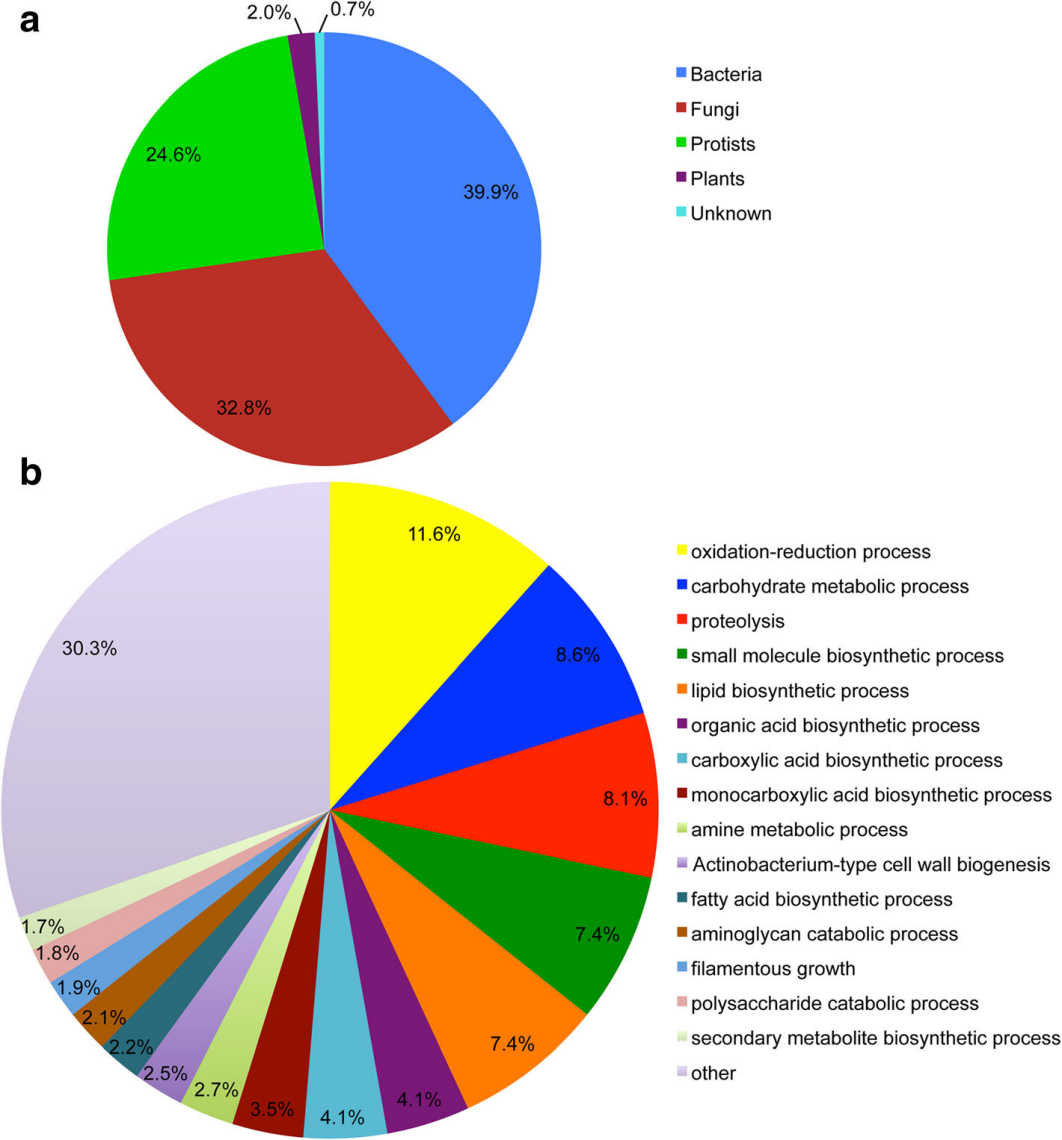
Origin and function of horizontal gene transfer in *Folsomia candida*. **a** Origin of foreign genes. **b** Percentage of enriched gene ontology biological processes associated with foreign genes

Earlier we reported that *F. candida* carries an Isopenicillin N synthase (IPNS) gene most likely acquired by HGT [35]. In order to systematically identify all HGTs in the *F. candida* genome, we assessed 28,734 genes with the horizontal gene transfer index h, calculated as the score difference between the best non-metazoan and the best metazoan match. We identified a total of 809 ‘foreign’ genes in the *F. candida* genome (2.8%), which were validated by physical linkage with native genes and phylogenetic inference (Additional file 3). This number is among the highest found in metazoan genomes, being only exceeded in rotifers and some nematode species [34, 36]. Interestingly, transposon abundance was significantly correlated with HGT abundance (Spearman rank correlation rho = 0.637, *p* < 2.2e^−16^, Additional file 2). Furthermore, 792 HGTs contain a complete intact open reading frame, while 15 HGTs only miss an ATG start codon, and two miss a 3′ stop codon. In total, 466 HGTs (57.6%) were supported by RNA-Seq data with an FPKM value of more than 0.5, suggesting that a large fraction of HGTs are functional and are being transcribed. The majority of HGTs originated from bacteria (39.9%), fungi (32.8%) and protists (24.6%) (Fig. 2a). A subset of 330 *F. candida* HGTs each share an orthologous HGT in the *O. cincta* genome, suggesting that a proportion of these genes have been horizontally transferred before divergence of the two species. The evolutionary split between *F. candida* and *O. cincta* is substantial. They belong to different families and have evolved very different adaptive traits [37, 38]. The observation that roughly 30% of HGT has persisted in both species after divergence suggests that these genes are important in the ecology of Collembola.

Remarkably, the arbuscular mycorrhizal fungus (AMF) *Rhizophagus irregularis (Glomus intraradices)* is indicated as a source of HGTs in 87 cases. Eighteen of them showed high similarity to heat shock protein (Hsp) 70/90 co-chaperone. Interestingly, three laccases were identified, suggesting the potential of *F. candida* to break down lignins. *F. candida* has an intimate association with AMF like *Rhizophagus*, as it preferably grazes these fungi [22] and the AMF-derived genes may have optimized their grazing capacity. Low springtail abundance is also beneficial for the AMF because grazing induces spreading and fungal inoculation of AMF to other plants [21]. Because AMFs facilitate uptake of phosphorus by plants, while they benefit from the plant’s assimilation products, there is a mutually beneficial tritrophic interaction between *F. candida*, AMF and the plant, which contributes to soil health and agricultural production [20–22].

**Fig. 3:**
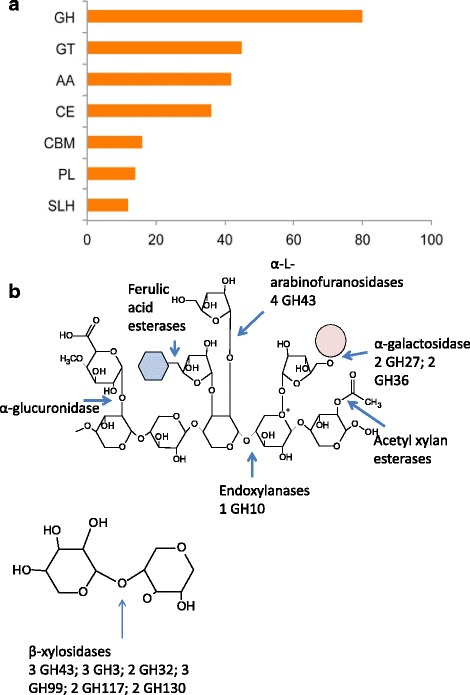
HGT genes with functional domains involved in carbohydrate metabolism as depicted from Carbohydrate-Active enZYmes Database (CAZy). **a** CAZy general categories among HGTs. Horizontal bars represent the number of unique sequences annotated with CAZyme domains. GH – glycoside hydrolases; GT – glycosyltransferases; AA – auxiliary activity module; CE – carbohydrate esterases; CBM – carbohydrate binding module; PL – polysaccharide lyases; SLH – S-layer homology module. **b** Hemicellulose degradation. The numbers represent the number of identified HGT enzymes. Pink circle represents D-Galactose; Blue hexagon represents Furelic acid

Many HGT genes in *F. candida* were shown to be involved in carbohydrate metabolism (Fig. 2b). A similar pattern was observed in the springtail *O. cincta* [3]. Statistical analysis showed that HGTs were enriched for CAZymes (229 out of 809 genes have CAZy domains) and that significantly more HGT genes had assigned CAZy domains when compared to the presence of CAZy domains among native genes (two-tailed *p*-value <0.0001). The largest three families of CAZymes were glycoside hydrolases (GHs), glycosyltransferases (GTs) and auxiliary activity family (AAs) (Fig. 3).

Glycoside hydrolases and carbohydrate esterases (CEs) are known to degrade cell walls, as was shown in a study on plant cell wall disintegration by fungi and bacteria [39]. Moreover, most enzymes in the AA category belong to the AA7 sub category, which are potentially involved in biotransformation/detoxification of lignocellulose compounds [40]. Based on these findings, we hypothesize that HGTs involved in cell wall degradation could be beneficial for life in the soil. These genes may allow access to important food sources in a habitat rich in plant- and fungal degradation products, which are rich in polysaccharides.

#### Secondary metabolite and antibiotic biosynthesis gene clusters

Using the antiSMASH tool, we identified 36 biosynthetic gene clusters in the *F. candida* genome. Most of the genes (52) in these clusters were also identified as HGTs. Functional annotation revealed that 30 clusters were type I polyketide synthases (PKSs), and three were non-ribosomal peptide synthetases (NRPS). Two clusters were related to terpene metabolism and one cluster was unclassified (Additional file 1). Both NRPSs and PKSs are considered to be rare in animals [41].

An interesting feature of *F. candida* is that it is the first animal discovered with penicillin biosynthesis genes in its genome. One single isopenicillin N synthase (*IPNS*) gene was reported previously [35]. Our current systemic HGT analysis re-identified *IPNS*, but the antiSMASH analysis identified a second expressed *IPNS* gene in the genome together with a second amino adipyl-cysteine-valine synthase (*ACVS*, antiSMASH cluster 29, Additional file 1), while cluster 30 contained *ACVS*, and *IPNS* together with *cmcJ* and *cmcI*. This cluster was very recently reconfirmed using Sanger sequencing in another study and is flanked by a retrotransposon [42]. The role of beta-lactam production in the gut epithelium and excretion in the gut lumen could be two-fold. It will allow the animal to control the gut microbial association, stabilizing it compared to its surrounding highly diverse soil microbial community. Indeed, our recent study on gut microbiome diversity in *F. candida* supports this hypothesis, although differences were observed between animals from a laboratory culture and animals from a natural population [43]. Alternatively, internal beta-lactam production could protect the animal from potentially harmful pathogens in the soil and may explain its lack of susceptibility to pathogens as observed in previous studies [44, 45].

### Intragenomic collinearity

**Fig. 4:**
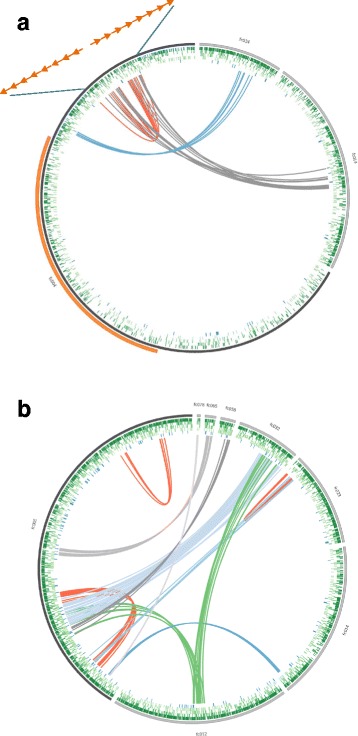
Examples of intragenomic collinearity in the scaffolds 4 (**a**) and 5 (**b**) of the *Folsomia candida* genome. The rings are from outer to inner represent: 1) DNA transposons, 2) LTRs, 3) LINEs, 4) RC (=Helitrons, or rolling circle transposons), 5) HGT genes. The orange bar in Fig. 5a marks the location of the *Hox* gene cluster

Segmental duplications can play a major role in gene and genome evolution because they have been associated with gene innovation and disease-causing rearrangements for instance in humans [46, 47]. To identify syntenic blocks of highly similar gene pairs, a BlastP search was performed using protein sequences within the genome to identify collinear groups of protein-coding gene pairs. We observed 883 (3.2% of all genes) collinear genes, which were organized in 55 syntenic blocks. Most of these were observed on scaffold 4 and 5 (Fig. 4, Additional file 2), while most other large scaffolds (2, 3, 6, and 7) were completely devoid of such structures. These regions were correlated with increased numbers of transposons (Spearman rank correlation rho = 0.637, *p* < 2.2e^−16^, Additional file 2), and were also associated with increased numbers of HGT genes (Spearman rank correlation rho = 0.359, *p* < 1.4e^−10^, Additional file 2). In contrast, no such collinear blocks were observed within the obligate sexual reproducing species *O. cincta*.

**Fig. 5:**
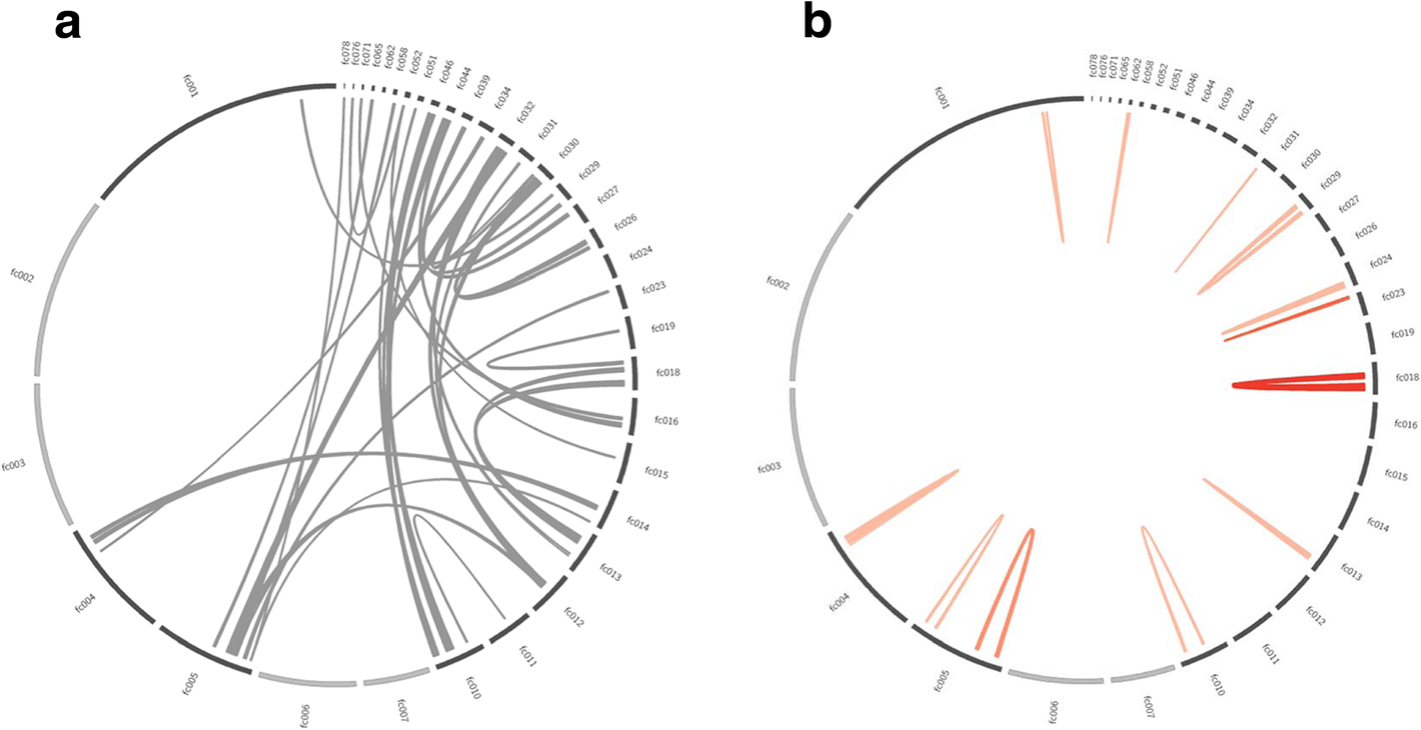
Collinearity of the *Folsomia candida* genes among and within scaffolds. **a** inter-scaffold collinear blocks among the seven largest scaffolds (*gray*); light gray bars depict scaffolds without collinearity. **b** intra-scaffold collinear blocks; palindromes are depicted in pink; tandem repeats are depicted in red

Fourty cases of synteny were observed between collinear blocks across different scaffolds. Some of the collinear blocks suggested allelic variation (scaffold 5 with 58, 65 and 78, Fig. 5a). For instance, scaffold 58 was completely collinear with a gene cluster located on major scaffold 2. Such patterns were previously observed for all collinear gene clusters in the parthenogenetic nematode *Meloidogyne incognita* [48]. The authors suggested that this might be a consequence of the unusual reproduction system of *M. incognita*, which includes only mitotic cell division. As such, homologous chromosomes cannot undergo meiotic recombination and therefore diverge independently from each other. However, this explanation cannot be considered for *F. candida* since it undergoes meiosis I [11]. Moreover, we should observe a division of PacBio coverage between two collinear blocks, if allelic variation is captured in these structures. This was not the case: PacBio coverage did not differ significantly between regions (1 Mb windows) with or without collinear blocks (t_259.81_ = −0.83, *P* = 0.41). For instance highly rearranged scaffolds 4 and 5 show highly similar PacBio read coverage when compared with scaffold 2, 3, 6 and 7, which are void of collinear blocks across other scaffolds (Additional file 2). Moreover, several regions showed collinearity with regions on other scaffolds, but their surrounding sequences did not match among the two scaffolds. This was observed for scaffold 5 and 14 (dark blue lines, Fig. 4b), where 12 genes showed collinearity, but their surrounding sequences did not match at all. Such observations may be better explained by translocation events.

We identified 15 blocks that were physically linked within a scaffold, of which 11 formed palindromes by inverted duplication (Fig. 5b). Flot et al. [34] suggested that such an arrangement does not allow meiotic pairing. Two palindromes showed substantial copy number variation of a particular open reading frame. The palindrome on scaffold 4 was of special interest because manual inspection showed that it contained two times eight immunoglobin superfamily member *Dscam2* genes (schematic representations of *Dscam 2* copies are depicted as arrows in Fig. 4a). *Dscam2* is proposed to be a tiling receptor in lamina 1 neurons of *Drosophila* [49]. *Drosophila’s* visual system is modular with photoreceptor neurons projecting on lamina neurons via a process called tiling. *Dscam2* is essential in this tiling process. It is unclear why this particular gene was amplified in this way, especially since *F. candida* has lost its eye structures as a consequence of evolution in the soil. Another palindrome on scaffold 30 contained two times five copies of a zink finger protein, which is well known to bind DNA and RNA and may be involved in transcriptional regulation.

**Fig. 6:**
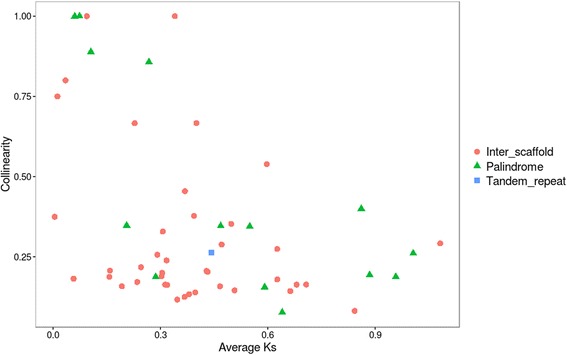
Relation between Ks (synonymous divergence, *x-axis*) and the fraction of collinear genes for collinear regions (*y-axis*). Regions with high collinearity and low divergence are probably due to recent duplication events

Palindromic organization of gene synteny was first discovered in the human Y-chromosome [50]. More recently, 17 instances of the palindromic organization were discovered in the genome of the parthenogenetic rotifer *A. vaga* [34]. Rozen et al. suggested that palindromes are involved in gene conversion, driving the opposite collinear blocks to evolve in concert. This was indeed shown for palindromic sequences in the human Y chromosome [50]. Gene conversion may remove mutations because they are overwritten with the other allelic version. This process can slow down Muller’s ratchet, the irreversible accumulation of detrimental mutations. Alternatively, mutations may become homozygous, and therefore exposed to selection [34]. The *Dscam2* genes organized in a palindrome on scaffold 4 (Fig. 4a) showed a low synonymous substitution rate of 0.2, although this was still one magnitude higher than observed in Y-chromosome genes. Subsequently, we compared Ks for segmental duplications regions with inter collinear regions. Figure 6 shows that both palindromic collinear blocks and tandem repeated collinear blocks show a wide range of Ks values. This indicates that a mechanism of concerted evolution does not explain the occurrence of these blocks, despite previous suggestions that such a process would be advantageous for parthenogenetically reproducing animals to counteract accumulation of deleterious mutations.

### Rearrangement in the *Hox* gene cluster

**Fig. 7:**
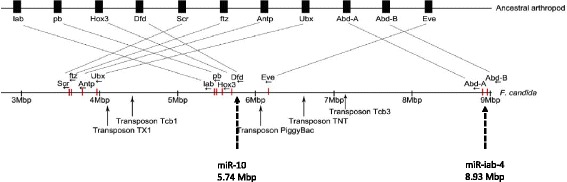
The *Hox* gene cluster of *Folsomia candida* on scaffold 4, as compared to consensus arthropod *Hox* gene cluster. *Lab*, labial; *pb,* proboscipedia; *Dfd*, deformed; *Scr*, sex combs reduced; *ftz*, fugi tarazo; *Antp*, antennapedia; *Ubx*, ultrabithorax; *Abd-A*, abdominal-A; *Abd-B*, abdominal-B; *Eve*, even-skipped; miR, microRNA (miRNA) positions depicted with dashed arrows. Transposon positions depicted with arrows

The homeobox domain-containing genes of the *Hox* gene cluster are important in patterning the anterior-posterior axis during embryonic development. An intact well-ordered *Hox* gene cluster was observed in the *F. candida* genome on scaffold 4, spanning 5.36 Mbp. This is extraordinary long when compared to the *Hox* cluster in a range of invertebrates, which does not exceed 500 Kbp [51]. We checked whether the synteny of *Hox* cluster in *F. candida* was according to the ancestral arthropod synteny [51]. *Hox* genes usually have an anterior-posterior collinearity pattern, where *labial*/*Hox1* coordinates development of anterior/head structures. At the posterior side, Abdominal-B*/Hox 11* coordinates more abdominal body parts. The synteny of the ancestral arthropod *Hox* gene cluster is depicted in Fig. 7.

Both *F. candida* and *O. cincta* contain a full set of 11 functional *Hox* genes (Fig. 7). In *F. candida*, they are identified scaffold 4 and surrounded by 577 predicted genes (www.collembolomics.nl). We observed a major rearrangement of this cluster, while in *O. cincta* the synteny of the *Hox* gene cluster is completely in accordance with the ancestral arthropod synteny [3]. In *F. candida*, the segment containing *Hox* genes Sex combs reduced (*Scr),* Fushi-tarazu *(ftz),* Antennapedia *(Atnp),* and Ultrabithorax *(Ubx)*, (involved in patterning of thoracic segments) were positioned uptream of the *Hox* genes Labial (*lab),* Proboscipedia *(pb), Hox3,* and Deformed *(Dfd)* (involved in anterior/head patterning), while Even-skipped *(Eve)* was positioned in between *lab, pb, Hox3, Dfd* and *Abdominal A* and *B* (*Abd-A, Abd-B* (Fig. 7). This rearrangement can most likely be explained by a translocation event in the region containing *Scr-Ubx* and violates the general principle of collinearity in the *Hox* genes cluster. A meta-analysis of *Hox* genes cluster organization among bilaterian animals by Duboule [52] suggests that collinearity in the *Hox* genes cluster has strictly been consolidated in vertebrates, but to a lesser extent in arthropods. For instance, the *Hox* gene cluster is split and distributed over two chromosomal regions in *D. melanogaster* and *Bombyx mori*. Furthermore, predatory mites show a completely atomized *Hox* gene organization [53]. The *Hox* gene cluster organization of *F. candida* is more comparable to the disorganized structure in the *Hox* gene cluster of sea urchins, where the *Hox* genes 1-3 (*lab*, *pb*, *Hox3*) region is positioned opposite to the posterior *Hox* cluster next to *Hox* gene 13 (*Abd-B*) [54]. This rearrangement was also suggested to be the result of a translocation.

Interestingly, we identified two microRNAs (miR), essential for spatiotemporal *Hox* gene expression, in *F. candida*’s *Hox* gene cluster [55]. MicroRNA *miR-iab-4* was positioned in between *Abd-A* and *Abd-B*. The experimentally verified targets of miR-*iab-4* are *Abd-A* and *Ubx*, which are usually positioned next to each other [52]. In contrast, in *F.candida Ubx* was relocated in front of the anterior gene *labial*. Whether these genes are still under control of *miR-iab-4*, needs to be experimentally verified. The other microRNA (miR-10) is usually positioned between *Scr* and *Dfd* [55]. In the case of *F. candida*, it was situated next to *Dfd* but it seemed disassociated from *Scr* (Fig. 7), while the latter has been shown to be a target of miR-10 transcriptional control. Temporal and spatial gene expression analysis should elucidate whether these unusual microRNA positions have affected timing of *Hox* gene activation. Furthermore, we observe five transposons in the *Hox* cluster, of which two are positioned at break points of the putative translocation event. They could have facilitated the genomic rearrangement of the *Hox* gene cluster. Additionally, they could have provided novel transcription factor binding sites for correct spatio-temporal expression of the *Hox* gene cluster [52, 56]. To conclude, we observed a highly unusual *Hox* gene cluster organization in *F. candida*. The organization of its body plan, however, contains bilaterian antero-posterior and dorso-ventral axes with normal head, thoracic and abdominal appendages. This suggests that the structural organization of the *Hox*-cluster bears little relation to the organization of the body plan.

## Conclusions

We successfully applied PacBio sequencing to assemble high quality nuclear and mitochondrial genomes of *F. candida,* including a genome of its endosymbiont *Wolbachia*. Functional analysis of expanded gene families suggests they are involved in response to environmental stress, recombination and DNA repair, and membrane tracking of lipids. Expansions in such families could be beneficial pre-adaptations to cope with life in the soil as a parthenogenetic species. We also identified a substantial number of HGTs, mainly enriched for carbohydrate active enzymes that may be optimal for unlocking carbohydrates from the plant and fungal decaying matter, which may provide an abundant food source in soil. Moreover, a functional antibiotic biosynthesis cluster was identified, suggesting the production of yet undiscovered antimicrobial compounds in an animal genome.

Analysis of *Hox* genes indicates a disorganized structure as compared to ancestral arthropod *Hox* organization. Despite that, *F. candida* shows a clear anteroposterior axis formation, commonly observed among hexapods. The collinearity analysis of the *F. candida* genome reflects some extraordinary features that may be related to genetic consequences of a parthenogenetic lifestyle.

Finally, all genome information is organized within the *F. candida* genome portal (http://www.collembolomics.nl/folsomia/portal) featuring the data and results of our analyses. It will assist researchers interested in the genome and genes of *F. candida*, and also facilitate further functional studies of this fascinating species.

## Methods

### Sample preparation and sequencing

*F. candida* animals (“Berlin strain”, Vrije Universiteit, Amsterdam) were kept at 15 °C in a climate room [57]. For high molecular DNA isolation, we crushed 16 times 100 animals with CTAB lysis buffer (800 μl per 100 animals) and incubated the lysates for 2 h at 65 °C. We extracted DNA from the lysates with phenol:chloroform:isoamyl alcohol (25:24:1) and chloroform:isoamyl alcohol (24:1). After this step, we pooled the water phases into 4 samples and precipitated the DNA with isopropanol. The pellets were dried and solved in 400 μl H_2_O. To each aliquot, we added 1 μl RNAse (100 mg/ml) and after an incubation of 15 min at 37 °C, we purified the DNA again with chloroform:isoamyl alcohol. We precipitated the DNA with (3 x volume) ethanol 100% and (1/10 x volume) NaAc (3 M, pH = 5.2), washed the pellets with ethanol 70% and after drying we solved the DNA in 200 μl H_2_O. An extra cleanup of the DNA was necessary for downstream procedures. Therefore, we used the MoBio Power Clean® DNA clean up kit and followed the manufacturer’s protocol. *F. candida* genome fragments of more than 20 kbp were sequenced using 12 SMRT cells on the PacBio RS II platform (with P6 chemistry) according to the manufacturer’s protocol at the Leiden Genome Technology Center (LGTC).

At the initial stage of the project, we also generated Illumina genomic data. For that, we isolated DNA from 2 × 50 animals from an isofemale line of *F. candida.* The animals were washed in 70% ethanol and crushed in 200 μl PBS. 200 μl Nuclei Lysis Solution (Promega) and 4 μl Proteinase K (20 mg/ml, Roche) were added before incubation for 15 min at 65 °C. After incubation 340 μl DNA Lysis Buffer (Promega) was added. The lysate was centrifuged for 10 min at 14.000 rpm. DNA was recovered from the supernatant using a DNA spin column (Promega), following the manufacturer’s protocol. DNA of the 2 samples was pooled and precipitated with ice-cold 2,5 x volume 100% ethanol/0,1 x volume NaAc (3 M, pH = 5,2) at −80 °C. After washing with ice-cold 70% ethanol, the DNA pellet was solved in 30 μl H_2_O. For sequencing, we used 3 μl 500 ng/μl DNA. Next-generation sequencing was performed on the Illumina HiSeq2000 platform at the LGTC.

### Assembly of *F. candida* genome

The assembly of *F. candida* genome PacBio reads was done with the Falcon assembler [58]. Reads longer than 8 kbp were assembled with the following parameters (output_multi, min_it = 0.70, local_match_count_threshold = 2, max_n_read = 200, max_diff = 100, max_cov = 50, min_cov = 5, bestn = 10) followed by PBJelly [59] scaffolding and gap filling and polishing with Quiver [60]. Afterwards, we performed another scaffolding with SSPACE-LongRead v.1.1 (l = 2) [61] and polished final scaffolds with Pilon v.1.13 (−-fix bases, −-diploid) [62]. Blobology was applied to identify taxon-annotated GC-coverage plots, possibly indicating contamination from external sources [63]. CEGMA pipeline v.2.4 [64] and Benchmarking Universal Single-Copy Orthologs (BUSCO) tool v.1.1 [65] was used to identify universal single copy orthologs in the assembly as a measure of the completeness and contiguity.. Additionally, we mapped *F. candida* de novo transcripts [23] to the genome scaffolds with Blat and used isoblat v.3.0 [66] to assess assembly completeness. The genome of *Wolbachia* endosymbiont was identified among assembled scaffolds using BlastN [67] against other *Wolbachia* genomes. All reads mapping to these two scaffolds were selected and assembled in Falcon [58] (output_multi, min_it = 0.70, local_match_count_threshold = 2, max_n_read = 100, max_diff = 40, max_cov = 60, min_cov = 2, bestn = 10) followed by PBJelly [59]. Strings of N’s were removed from the scaffolds of the original assembly introducing breaks and the two assemblies were merged using Minimus2 [68]. The remaining two gaps were resolved by blasting the trimmed edges of the scaffolds to the PacBio library in search for reads bridging them. Consensuses of these reads were created with PBdagcon (https://github.com/PacificBiosciences/pbdagcon), after which they were manually inserted into the assembly. Thereafter all reads were mapped back to the assembly and a consensus was created with PBdagcon [69] followed by a polishing with Quiver [60].

To reconstruct a mitochondrial genome of *F. candida*, quality checked Illumina reads were trimmed in Trimmomatic v.0.33 [70] with the following parameters: ILLUMINACLIP:TruSeq2-PE.fa:2:30:10; LEADING:3; TRAILING:3; SLIDINGWINDOW:4:15; HEADCROP:8; MINLEN:50. The reads were then error corrected in Lighter (k = 17; alpha = 0.085) [71]. We used a PacBio read covering the complete mitochondrial genome as a reference for IOGA [72]. The annotation of the mitochondrial genome was performed in MITOS annotation web-tool [73] followed by manual curation.

### Genome annotation

Genome annotation was performed using the MAKER v.2.31.8 pipeline [74]. Augustus v.3.1.0. [75] and GeneMark [76] were used as ab initio gene predictors; they were trained using BRAKER1 [77] with raw RNA-Seq data (17.9 Gbp Illumina, SRR935329 [23], 3.3 Gbp Illumina, SRR921597 [4]). The de novo *F. candida* transcriptome [23], as well as RNA-Seq data mapped with TopHat2 [78] and protein sequences from *D. pulex*, *A. pisum* and *D. melanogaster* from the Ensembl Genomes database [79] were used as transcript-based and protein homology-based evidence, respectively. We used RepeatMasker with RepBase database [80, 81] together with a de novo repeat library, constructed using RepeatModeler [82] to find and mask repeats in the *F. candida* genome. Functional annotation was assigned with BlastP [67] (E-value threshold of 0.1) using SwissProt, TrEMBL databases, and InterProScan analysis [83] against the Superfamily [84] and Pfam [85] protein databases. Also, we used Blast2GO suite v.3.1 to predict gene ontology (GO) terms in our dataset based on InterPro domains and BlastP hits against Swiss-Prot database (E-value threshold of 1e^−5^). To identify secondary metabolite biosynthesis gene clusters, we applied antiSMASH [86].

### Gene family expansion analysis

OrthoFinder with inflation parameter of 1.5 [87] was used to calculate ortholog clusters between *F. candida*, *O. cincta, T. castaneum*, *P. humanus*, *A. pisum*, *D. melanogaster, A. aegypti*, *S. maritima*, *I. scapularis, T. urticae*, *D. pulex* and *C. elegans*. In order to identify expanded gene families in *F. candida,* we calculated z-scores as proposed by Cao et al. [88]. In short, the gene number for each family was subtracted by the average gene number of the family among all species in the analysis. This number was then divided by the standard deviation of the average gene number. Gene families with z-scores ≥2 were assumed to be expanded. Additionally, we performed a BlastN search (with E-value threshold of 1e^−5^) of expanded gene families against genes that were differentially expressed in response to stress [29].

### Horizontal gene transfer

To identify foreign genes in the genome of *F. candida,* we performed calculations of the horizontal gene transfer index h based on the protocol described by Crisp et al. [36] with a number of modifications described by Faddeeva-Vakhrusheva et al. [3]. The final set of foreign genes was predicted based on the h-score, followed by a physical linkage test (PacBio long read evidence of physical linkage of HGT candidate to neighboring native genes). HGT candidates that passed the linkage test, but with a best metazoan Blast bit-score of ≥50 were also subjected to phylogenetic validation with the same parameters as described by Faddeeva-Vakhrusheva et al. [3, 36]. To predict enriched gene ontology biological processes and molecular functions among the foreign genes in *F. candida,* we performed GO enrichment analysis using the topGO package [89] in R (v.3.2.2) with an elim algorithm (with the *p*-value threshold of 0.05). To check whether HGT genes were enriched for carbohydrate metabolism a chi-square test was performed. We additionally checked CAZy (Carbohydrate-Active enZYmes Database) domains [90] in the HGT genes and we compared the number of HGT genes with CAZy (with an e-value <0.1) domains to the number of native genes with CAZy domains with a chi-square test.

### *Hox* gene cluster analysis

We used BlastP to identify *Hox* genes and we assessed the synteny manually using the genomic locations on scaffold 4.

### Collinearity analysis

A BlastP search of all-against-all *F. candida* proteins was performed using an E-value cutoff of 1e^−10^. Collinear blocks of genes were detected using the package MCScanX [91]. Synteny plots were drawn using Circos [92]. A pearson correlation test was performed to statistically test association between collinear blocks, transposons and horizontally transferred genes.

## Additional files

Additional file 1: Tables with associated data. (XLSX 4914 kb)

Additional file 2: Word file containing gene ontology distribution in *Folsomia candida* genome, metabolic map for *Folsomia candida,* correlation plots between HGTs transposons and collinearity; additional collinear scaffolds, distribution of PacBio read coverage along the 7 largest scaffolds. (DOCX 2743 kb)

Additional file 3: Phylogenetic trees for confirmed HGTs in *Folosmia candida* genome. (PDF 1641 kb)

## Abbreviations

ABC: ABC transporter
Abd: Abdominal
ACVS: Amino adipyl-cysteine-valine synthase
AMF: Arbuscular mycorrhizal fungus
ANK: Ankyrin
Antp: Antennapedia
CAZy: Carbohydrate enzyme
Dfd: Deformed
Dscam: Down syndrome cell adhesion molecule
FPKM: fraction per kilobase of exon per million fragments
ftz: Fushi-tarazu
GST: Glutathione S-transferases
HGT: Horizontal gene transfer
IPNS: Isopenicillin N synthase
lab: Labial
miR: microRNA
PacBio: Pacific Biosciences Sequencing
pb: Proboscipedia
Scr: Sex combs reduced
SMRT: Single molecule real-time sequencing
SNP: Single nucleotide polymorphism
Ubx: Ultrabithorax
wFol: Wolbachia endosymbiont of *F. candida*
