# Supplementary material for: Coping with living in the soil: the genome of the parthenogenetic springtail Folsomia candida
Source: BMC Genomics. 2017 Jun 28;18:493. doi: 10.1186/s12864-017-3852-x (PMC5490193; doi:10.1186/s12864-017-3852-x)

Additional file 2

Distribution of GO terms assigned to the 15,883 annotated sequences

The proportion of expanded gene families with GO terms. Blue bars: GO terms relative to the total number of expanded gene families in *Folsomia candida* (n=368); Orange bars: a proportion of lineage-specific gene families annotated with GO terms relative to the total number of lineage-specific gene families in *F. candida* (n=74).


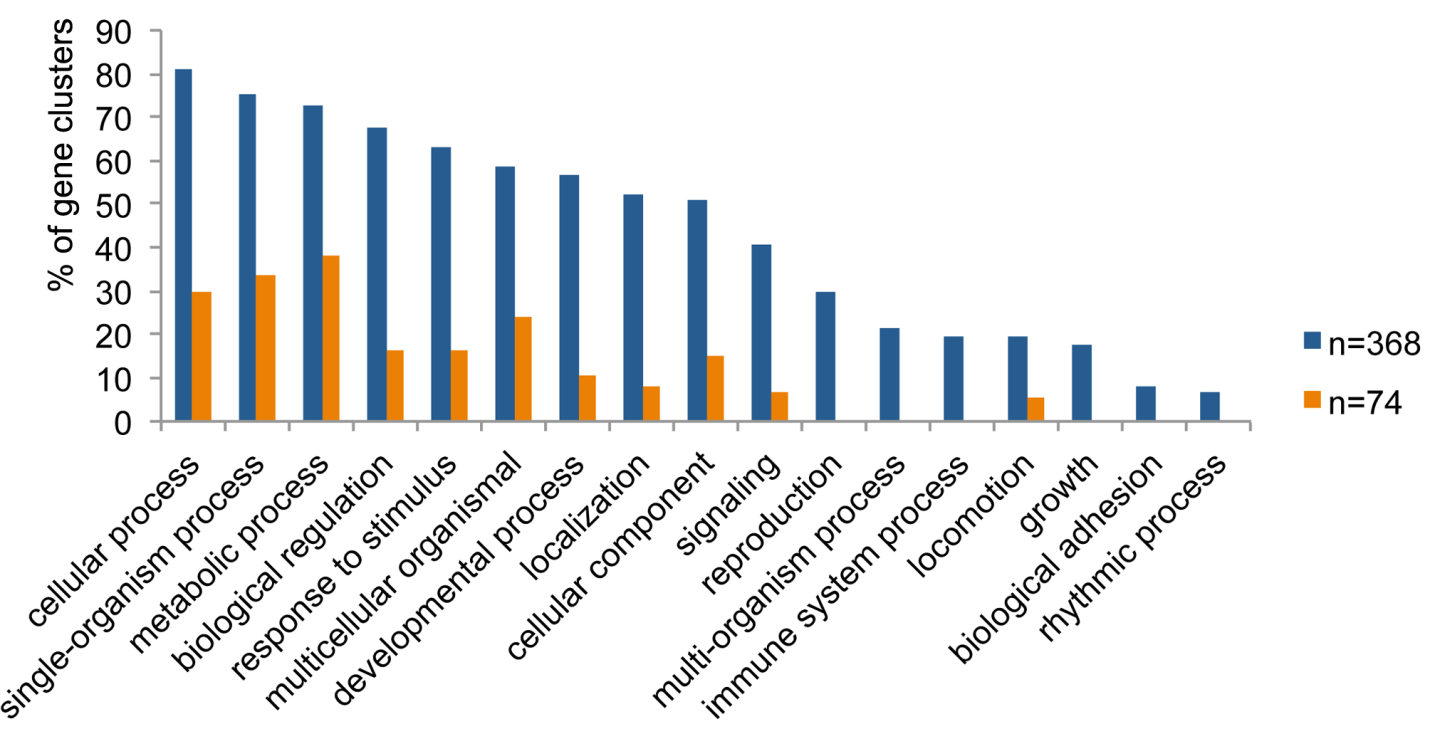


Coverage distribution of Pacific Bioscience reads across the seven largest scaffolds


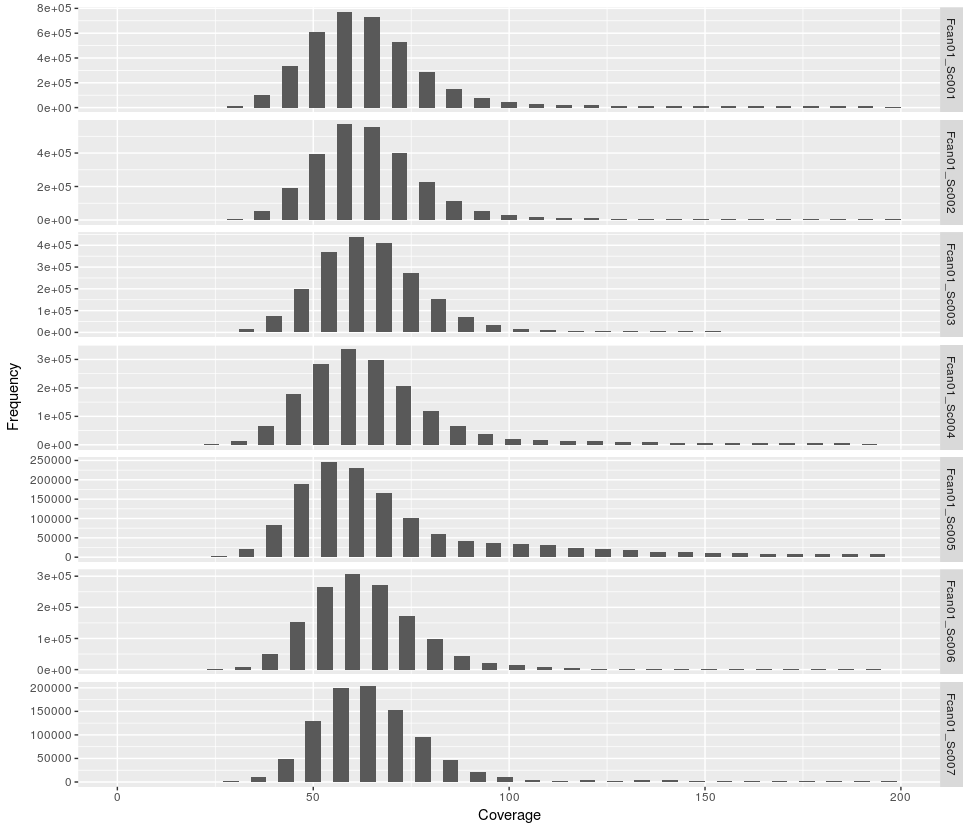


Correlation graphs of transposable element abundance (TE per 1Mb, x-axis) with abundance of horizontally transferred genes (HGTs per 1Mb, left graph) or with abundance of collinear genes (collinear genes per 1Mb, right graph).


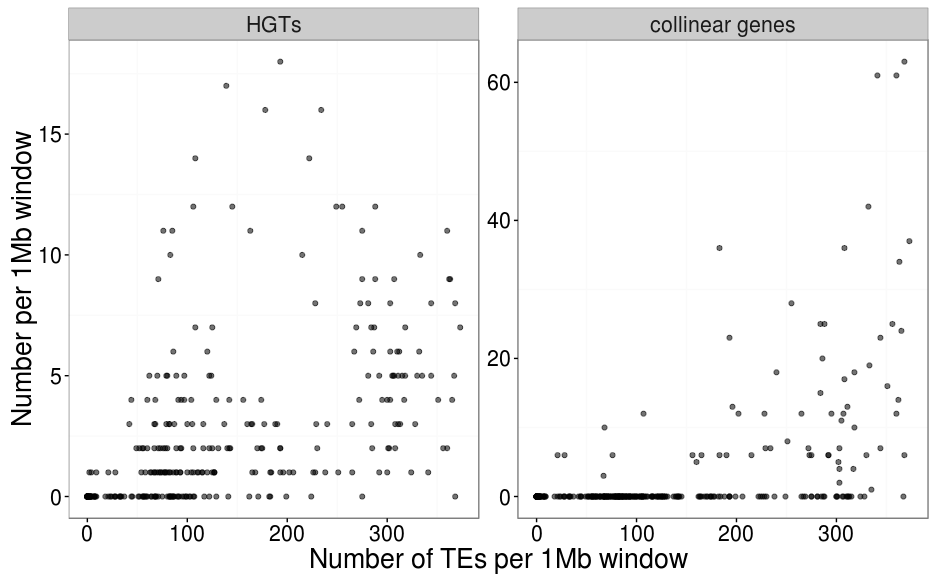


Collinearity graphs

The rings are from outer to inner represent: 1) DNA transposons, 2) LTRs, 3) LINEs, 4) RC (=Helitrons, or rolling circle transposons), 5) HGT genes (blue bars). Inter-scaffold collinear blocks are colored gray; Intra-scaffold collinear blocks are colored orange

Scaffold 1


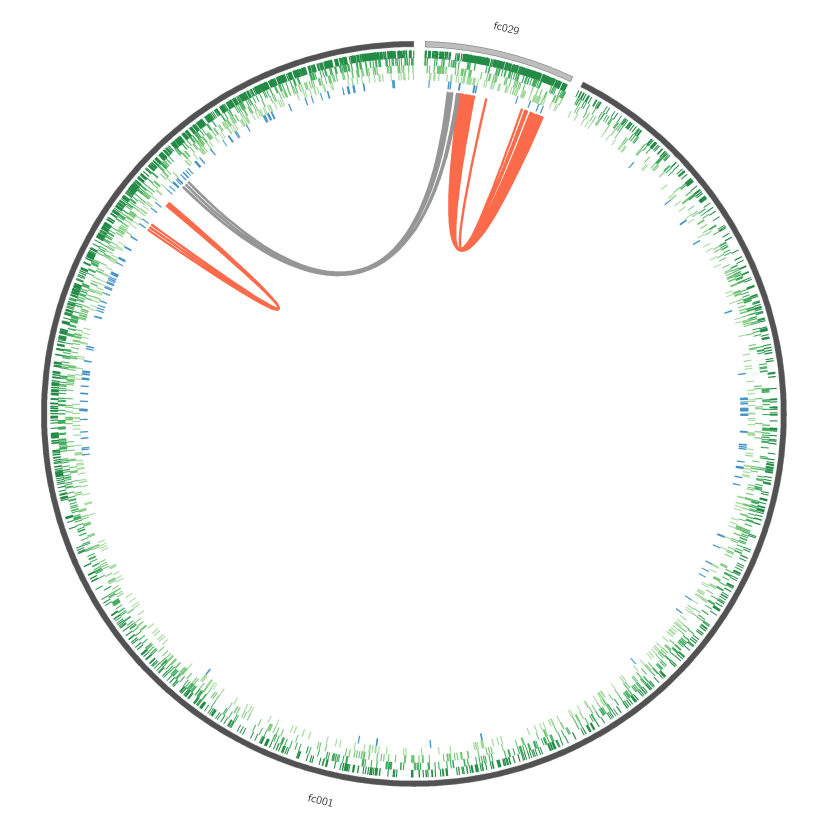


Scaffold 2


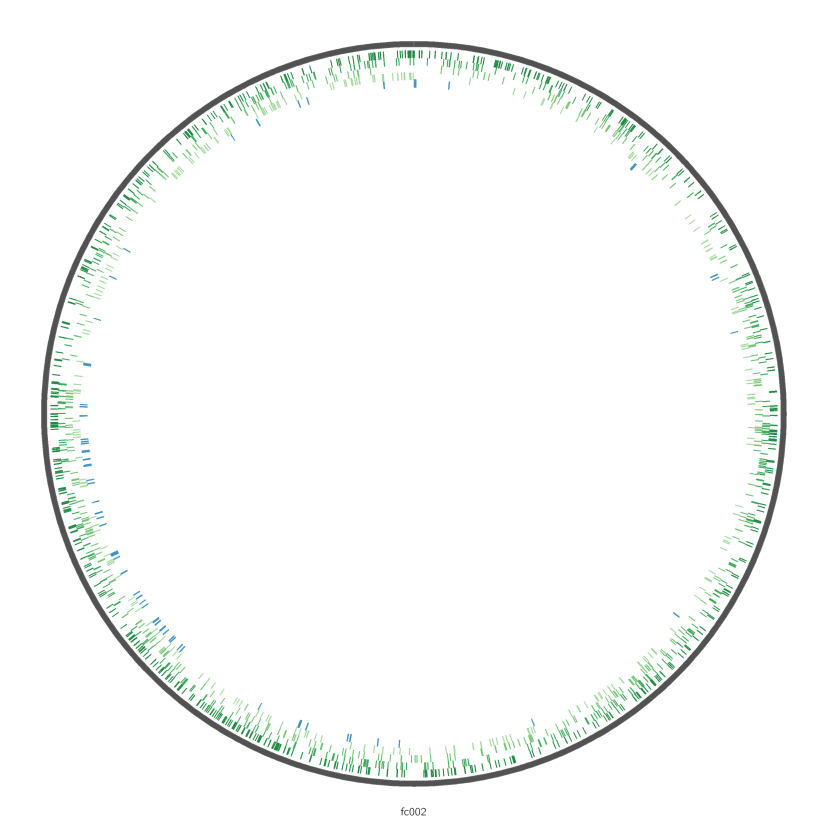


Scaffold 3


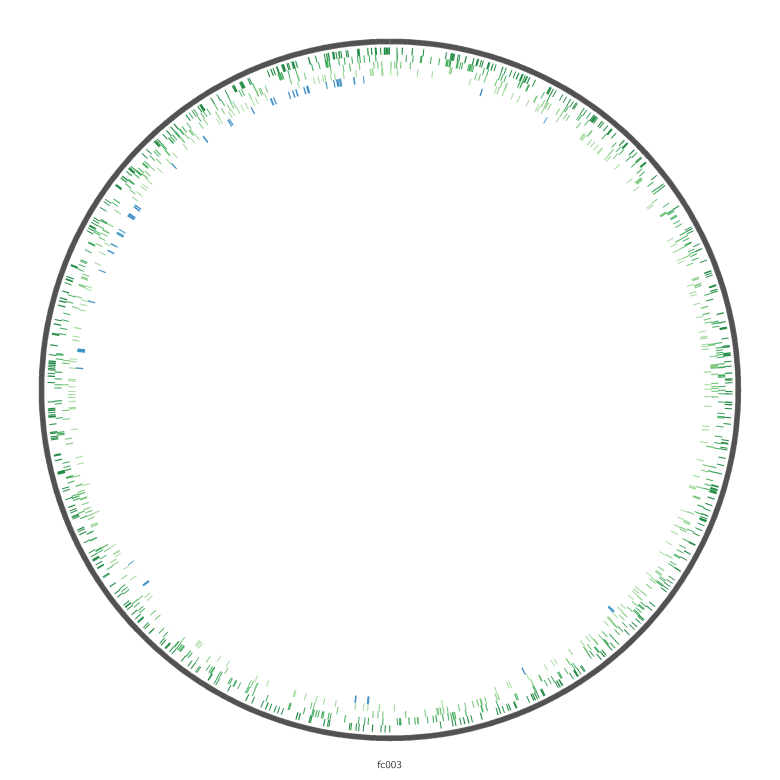


Scaffold 6


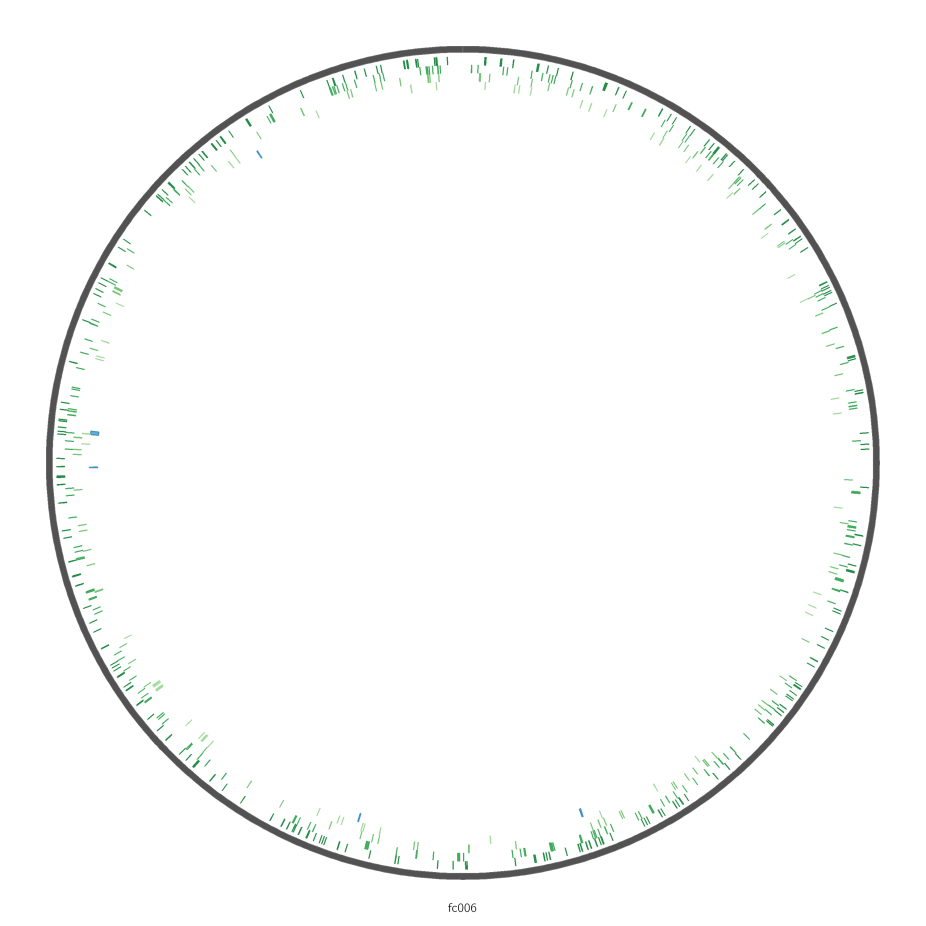


Scaffold 7


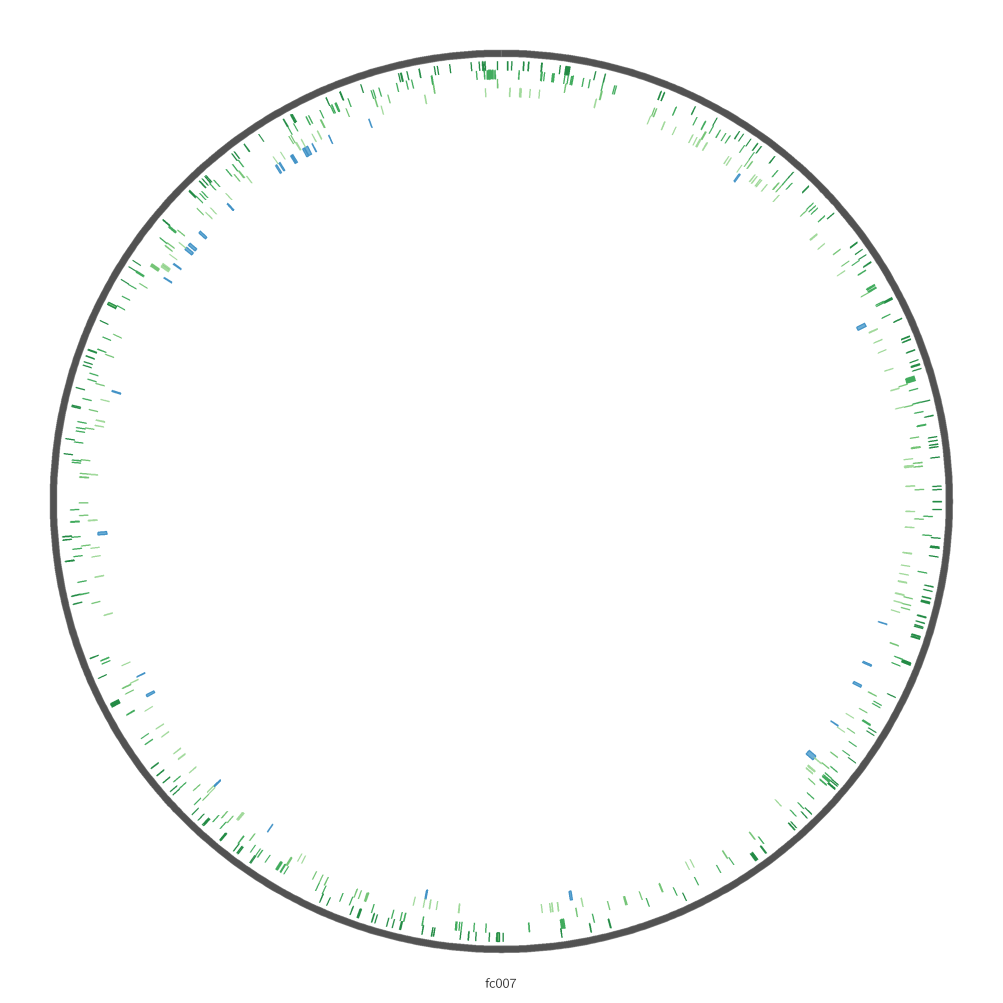

Supplement: Supplementary file 2 — Word file containing gene ontology distribution in Folsomia candida genome, metabolic map for Folsomia candida, correlation plots between HGTs transposons and collinearity; additional collinear scaffolds, distribution of PacBio read coverage along the 7 largest scaffolds. (DOCX 2743 kb) [file 12864_2017_3852_MOESM2_ESM.docx]
