# Supplementary material for: Coping with living in the soil: the genome of the parthenogenetic springtail Folsomia candida
Source: BMC Genomics. 2017 Jun 28;18:493. doi: 10.1186/s12864-017-3852-x (PMC5490193; doi:10.1186/s12864-017-3852-x)
Supplement: Supplementary file 3 — Phylogenetic trees for confirmed HGTs in Folosmia candida genome. (PDF 1641 kb) [file 12864_2017_3852_MOESM3_ESM.pdf]

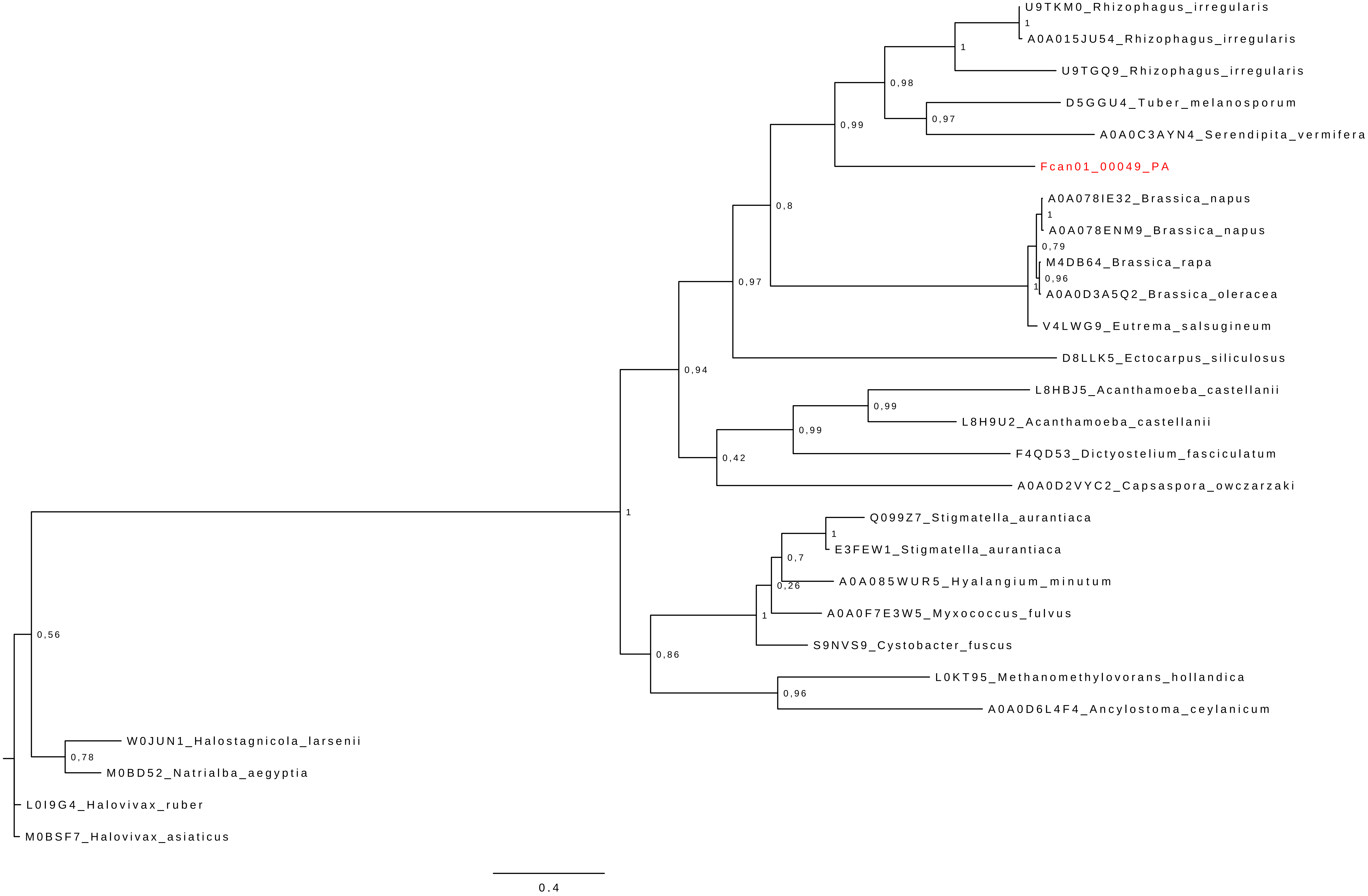
U9TKM0_Rhizophagus_irregularis
1
A0A015JU54_Rhizophagus_irregularis
1
U9TGQ9_Rhizophagus_irregularis
0,98
D5GGU4_Tuber_melanosporum
0,97
A0A0C3AYN4_Serendipita_vermifera
0,99
Fcan01_00049_PA
A0A078IE32_Brassica_napus
1
A0A078ENM9_Brassica_napus
0,79
M4DB64_Brassica_rapa
0,96
0,8
A0A0D3A5Q2_Brassica_oleracea
1
0,97
V4LWG9_Eutrema_salsugineum
D8LLK5_Ectocarpus_siliculosus
0,94
L8HBJ5_Acanthamoeba_castellanii
0,99
L8H9U2_Acanthamoeba_castellanii
0,99
F4QD53_Dictyostelium_fasciculatum
0,42
A0A0D2VYC2_Capsaspora_owczarzaki
1
Q099Z7_Stigmatella_aurantiaca
1
E3FEW1_Stigmatella_aurantiaca
0,7
A0A085WUR5_Hyalangium_minutum
0,26
A0A0F7E3W5_Myxococcus_fulvus
1
S9NVS9_Cystobacter_fuscus
0,56
0,86
L0KT95_Methanomethylovorans_hollandica
0,96
A0A0D6L4F4_Ancylostoma_ceylanicum
W0JUN1_Halostagnicola_larsenii
0,78
M0BD52_Natrialba_aegyptia
L0I9G4_Halovivax_ruber
M0BSF7_Halovivax_asiaticus
0.4

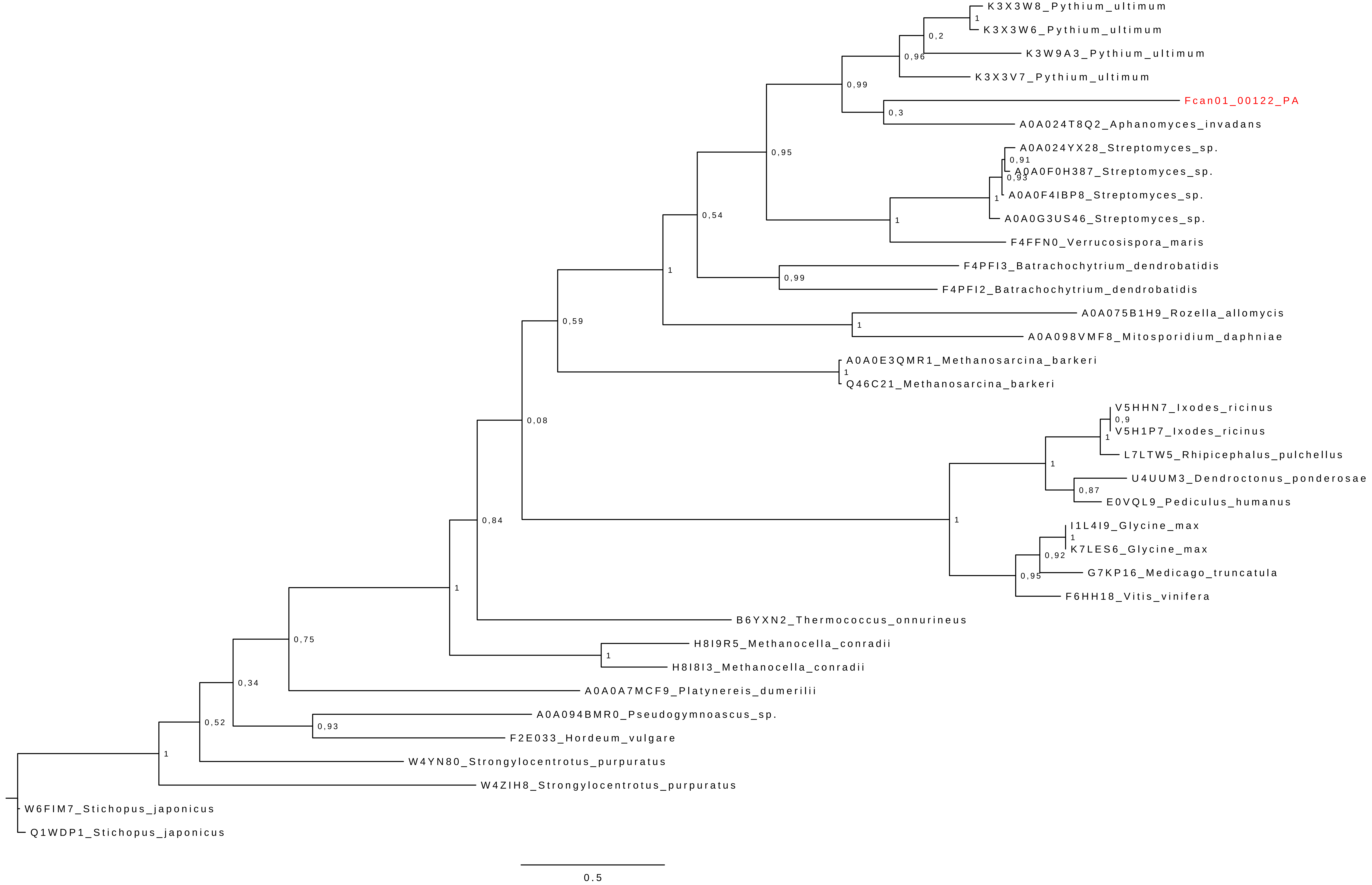
K3X3W8_Pythium_ultimum
K3X3W6_Pythium_ultimum
K3W9A3_Pythium_ultimum
K3X3V7_Pythium_ultimum
Fcan01_00122_PA
A0A024T8Q2_Aphanomyces_invadans
A0A024YX28_Streptomyces_sp.
A0A0F0H387_Streptomyces_sp.
A0A0F4IBP8_Streptomyces_sp.
A0A0G3US46_Streptomyces_sp.
F4FFN0_Verrucosispora_maris
F4PFI3_Batrachochytrium_dendrobatidis
F4PFI2_Batrachochytrium_dendrobatidis
A0A075B1H9_Rozella_allomycis
A0A098VMF8_Mitosporidium_daphniae
A0A0E3QMR1_Methanosarcina_barkeri
Q46C21_Methanosarcina_barkeri
V5HHN7_Ixodes_ricinus
V5H1P7_Ixodes_ricinus
L7LTW5_Rhipicephalus_pulchellus
U4UUM3_Dendroctonus_ponderosae
E0VQL9_Pediculus_humanus
I1L4I9_Glycine_max
K7LES6_Glycine_max
G7KP16_Medicago_truncatula
F6HH18_Vitis_vinifera
B6YXN2_Thermococcus_onnurineus
H8I9R5_Methanocella_conradii
H8I8I3_Methanocella_conradii
A0A0A7MCF9_Platynereis_dumerilii
A0A094BMR0_Pseudogymnoascus_sp.
F2E033_Hordeum_vulgare
W4YN80_Strongylocentrotus_purpuratus
W4ZIH8_Strongylocentrotus_purpuratus
W6FIM7_Stichopus_japonicus
Q1WDP1_Stichopus_japonicus
0.5

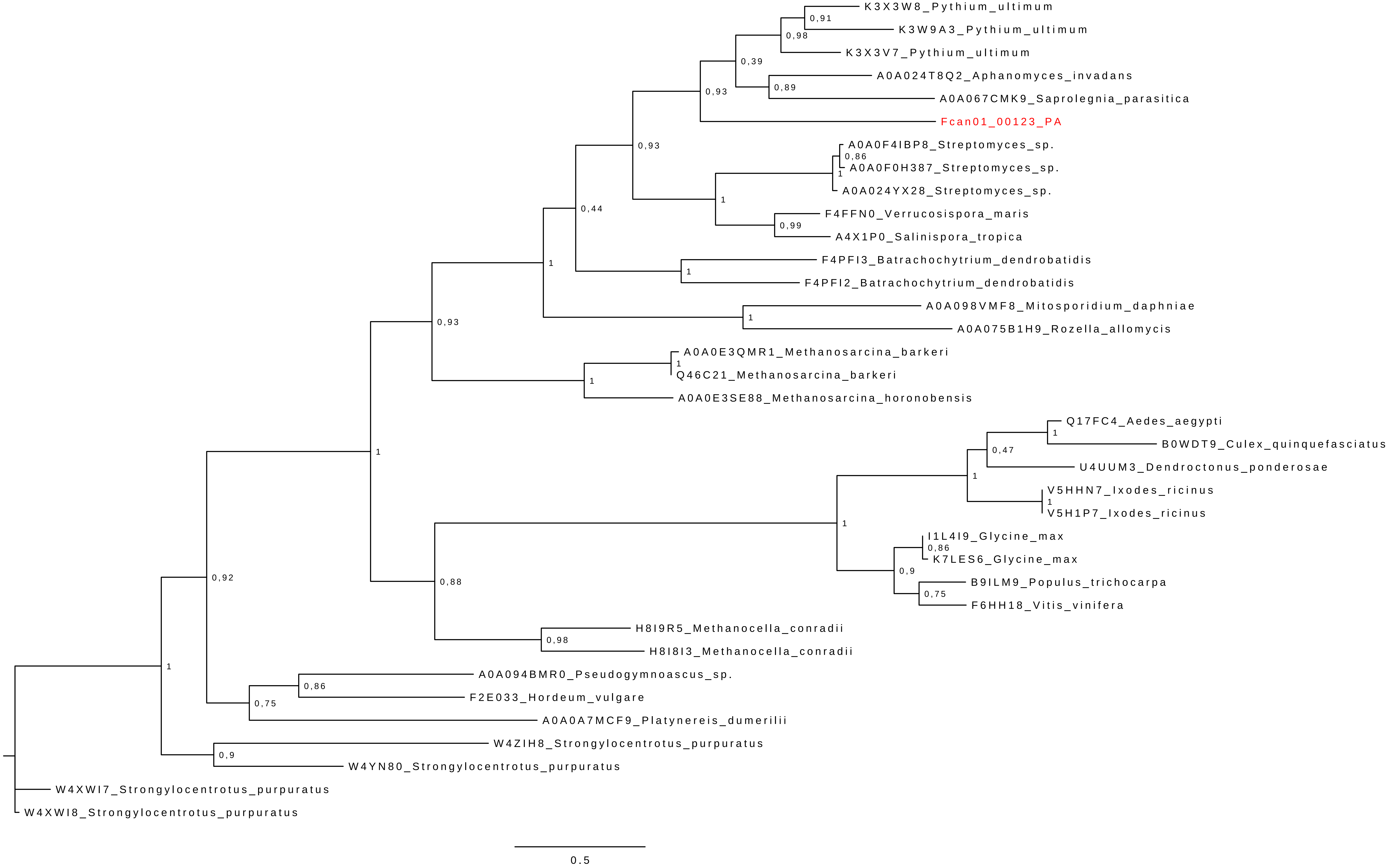
K3X3W8_Pythium_ultimum
0,91
K3W9A3_Pythium_ultimum
0,98
K3X3V7_Pythium_ultimum
0,39
A0A024T8Q2_Aphanomyces_invadans
0,89
A0A067CMK9_Saprolegnia_parasitica
0,93
Fcan01_00123_PA
0,93
A0A0F4IBP8_Streptomyces_sp.
0,86
A0A0F0H387_Streptomyces_sp.
1
A0A024YX28_Streptomyces_sp.
1
F4FFN0_Verrucosispora_maris
0,99
A4X1P0_Salinispora_tropica
0,44
F4PFI3_Batrachochytrium_dendrobatidis
1
1
F4PFI2_Batrachochytrium_dendrobatidis
A0A098VMF8_Mitosporidium_daphniae
1
A0A075B1H9_Rozella_allomycis
0,93
A0A0E3QMR1_Methanosarcina_barkeri
1
Q46C21_Methanosarcina_barkeri
1
A0A0E3SE88_Methanosarcina_horonobensis
1
Q17FC4_Aedes_aegypti
1
B0WDT9_Culex_quinquefasciatus
0,47
U4UUM3_Dendroctonus_ponderosae
1
V5HHN7_Ixodes_ricinus
1
V5H1P7_Ixodes_ricinus
1
I1L4I9_Glycine_max
0,86
K7LES6_Glycine_max
0,9
B9ILM9_Populus_trichocarpa
0,75
F6HH18_Vitis_vinifera
0,92
0,88
H8I9R5_Methanocella_conradii
0,98
H8I8I3_Methanocella_conradii
1
A0A094BMR0_Pseudogymnoascus_sp.
0,86
F2E033_Hordeum_vulgare
0,75
A0A0A7MCF9_Platynereis_dumerilii
W4ZIH8_Strongylocentrotus_purpuratus
0,9
W4YN80_Strongylocentrotus_purpuratus
W4XWI7_Strongylocentrotus_purpuratus
W4XWI8_Strongylocentrotus_purpuratus
0.5

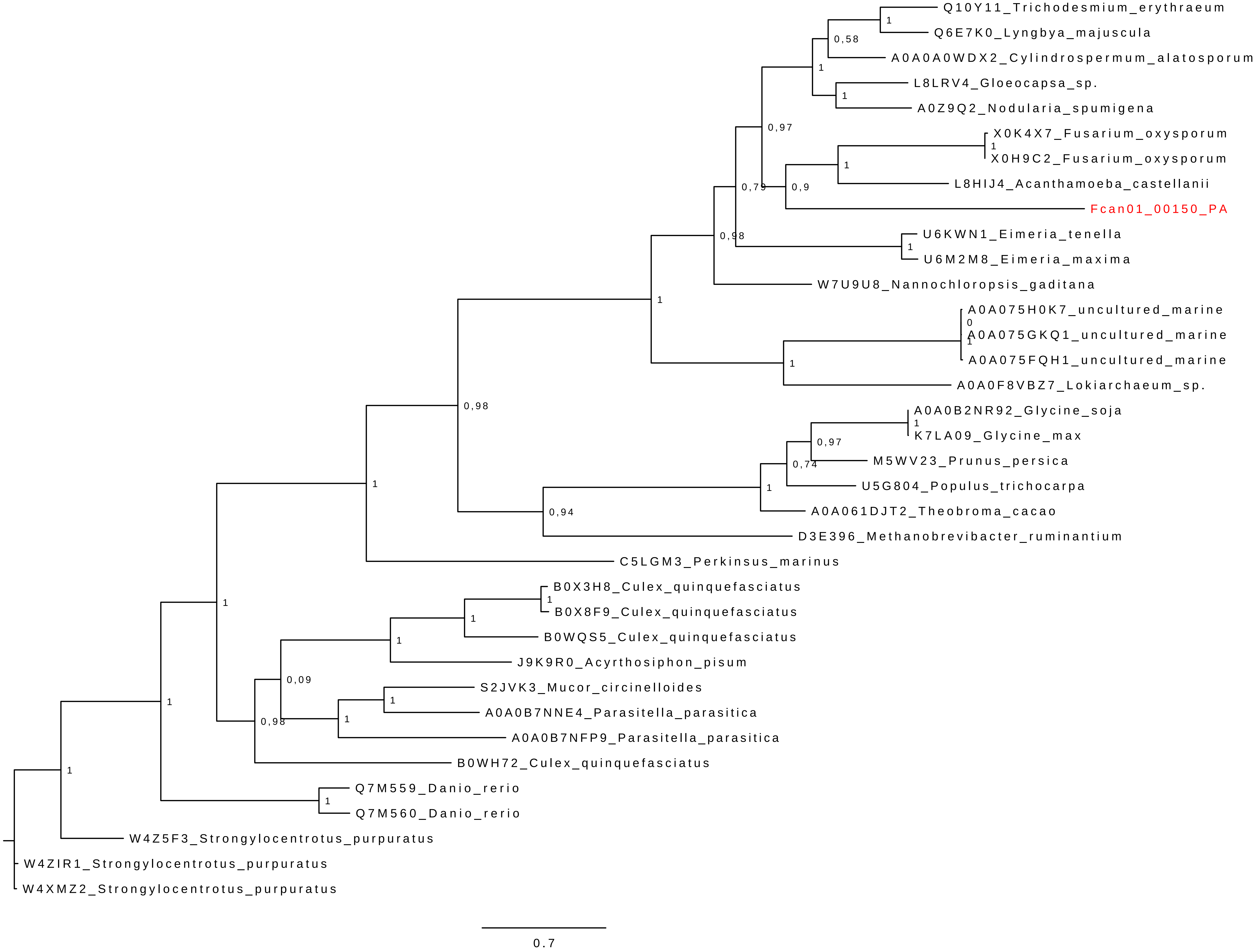
Q10Y11_Trichodesmium_erythraeum
Q6E7K0_Lyngbya_majuscula
A0A0A0WDX2_Cylindrospermum_alatosporum
L8LRV4_Gloeocapsa_sp.
A0Z9Q2_Nodularia_spumigena
X0K4X7_Fusarium_oxysporum
X0H9C2_Fusarium_oxysporum
L8HIJ4_Acanthamoeba_castellanii
Fcan01_00150_PA
U6KWN1_Eimeria_tenella
U6M2M8_Eimeria_maxima
W7U9U8_Nannochloropsis_gaditana
A0A075H0K7_uncultured_marine
A0A075GKQ1_uncultured_marine
A0A075FQH1_uncultured_marine
A0A0F8VBZ7_Lokiarchaeum_sp.
A0A0B2NR92_Glycine_soja
K7LA09_Glycine_max
M5WV23_Prunus_persica
U5G804_Populus_trichocarpa
A0A061DJT2_Theobroma_cacao
D3E396_Methanobrevibacter_ruminantium
C5LGM3_Perkinsus_marinus
B0X3H8_Culex_quinquefasciatus
B0X8F9_Culex_quinquefasciatus
B0WQS5_Culex_quinquefasciatus
J9K9R0_Acyrthosiphon_pisum
S2JVK3_Mucor_circinelloides
A0A0B7NNE4_Parasitella_parasitica
A0A0B7NFP9_Parasitella_parasitica
B0WH72_Culex_quinquefasciatus
Q7M559_Danio_rerio
Q7M560_Danio_rerio
W4Z5F3_Strongylocentrotus_purpuratus
W4ZIR1_Strongylocentrotus_purpuratus
W4XMZ2_Strongylocentrotus_purpuratus
1
0,58
1
1
0,97
1
1
0,79
0,9
0,98
1
1
0
1
1
1
0,97
0,74
1
0,98
0,94
1
1
1
1
0,09
1
1
0,98
1
1
1
0.7

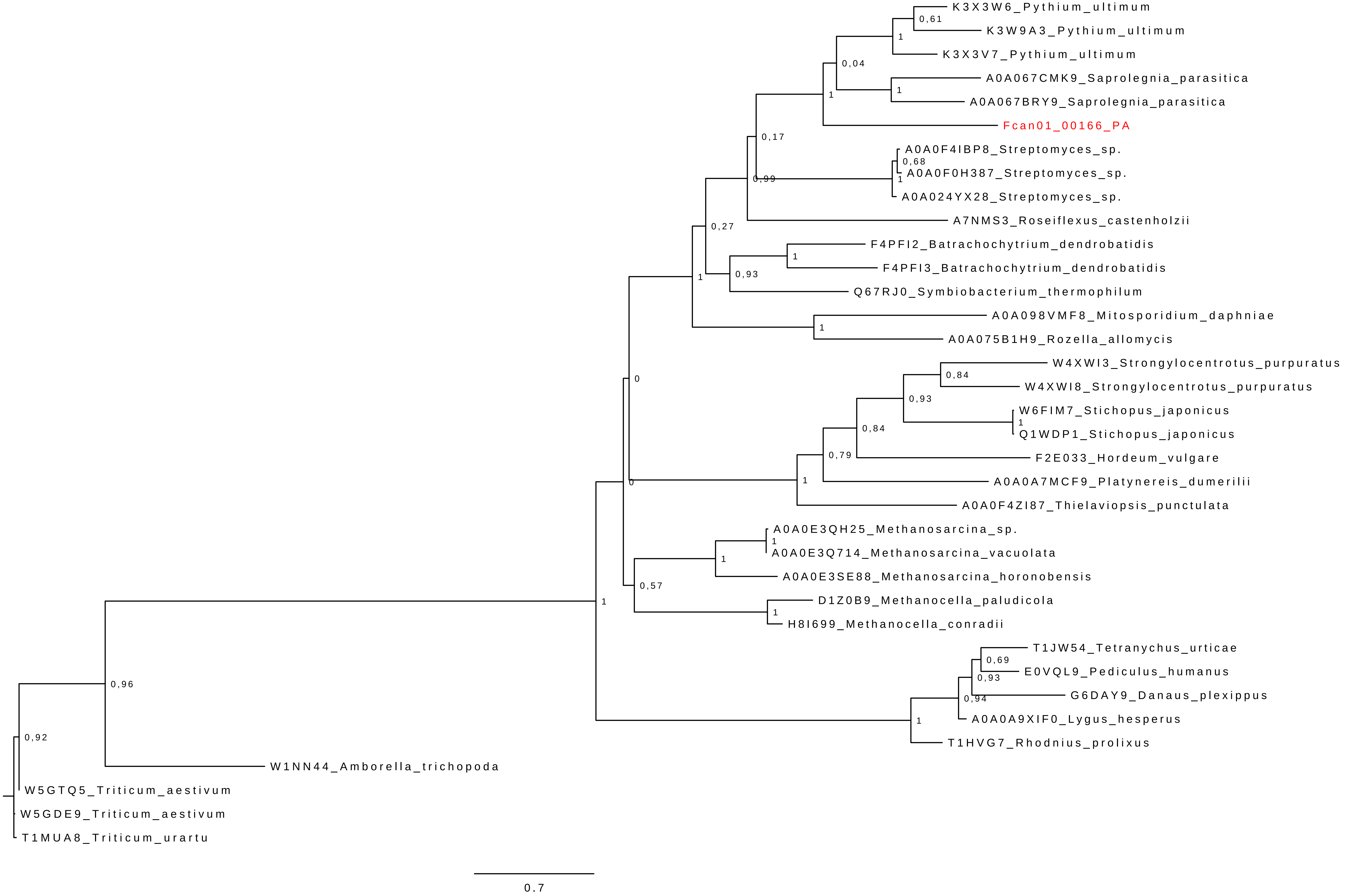

K3X3W6_Pythium_ultimum
K3W9A3_Pythium_ultimum
K3X3V7_Pythium_ultimum
A0A067CMK9_Saprolegnia_parasitica
A0A067BRY9_Saprolegnia_parasitica
Fcan01_00166_PA
A0A0F4IBP8_Streptomyces_sp.
A0A0F0H387_Streptomyces_sp.
A0A024YX28_Streptomyces_sp.
A7NMS3_Roseiflexus_castenholzii
F4PFI2_Batrachochytrium_dendrobatidis
F4PFI3_Batrachochytrium_dendrobatidis
Q67RJ0_Symbiobacterium_thermophilum
A0A098VMF8_Mitosporidium_daphniae
A0A075B1H9_Rozella_allomycis
W4XWI3_Strongylocentrotus_purpuratus
W4XWI8_Strongylocentrotus_purpuratus
W6FIM7_Stichopus_japonicus
Q1WDP1_Stichopus_japonicus
F2E033_Hordeum_vulgare
A0A0A7MCF9_Platynereis_dumerilii
A0A0F4ZI87_Thielaviopsis_punctulata
A0A0E3QH25_Methanosarcina_sp.
A0A0E3Q714_Methanosarcina_vacuolata
A0A0E3SE88_Methanosarcina_horonobensis
D1Z0B9_Methanocella_paludicola
H8I699_Methanocella_conradii
T1JW54_Tetranychus_urticae
E0VQL9_Pediculus_humanus
G6DAY9_Danaus_plexippus
A0A0A9XIF0_Lygus_hesperus
T1HVG7_Rhodnius_prolixus
W1NN44_Amborella_trichopoda
W5GTQ5_Triticum_aestivum
W5GDE9_Triticum_aestivum
T1MUA8_Triticum_urartu
0.7

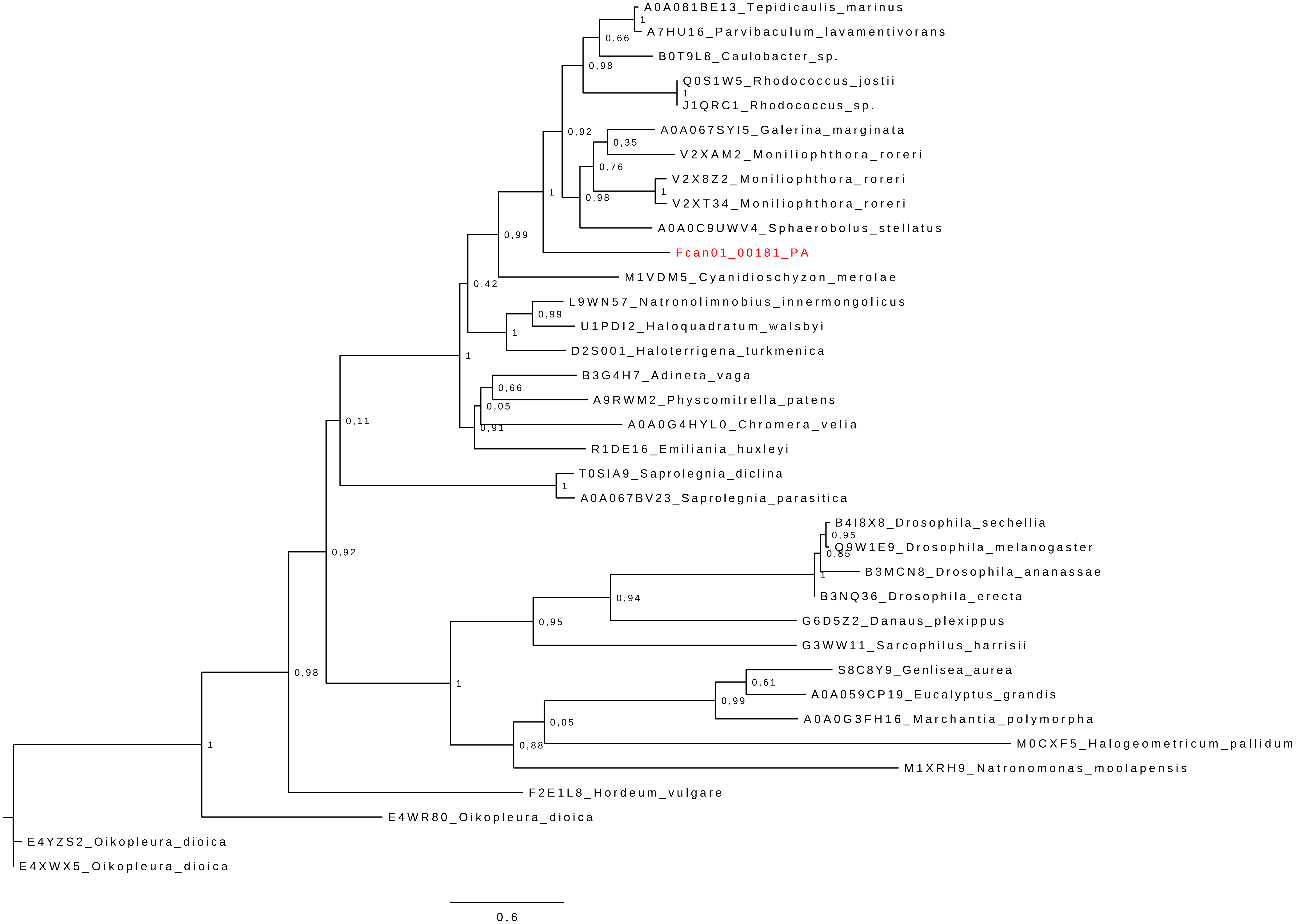

A0A081BE13_Tepidicaulis_marinus
1
A7HU16_Parvibaculum_lavamentivorans
0,66
B0T9L8_Caulobacter_sp.
0,98
Q0S1W5_Rhodococcus_jostii
1
J1QRC1_Rhodococcus_sp.
0,92
A0A067SYI5_Galerina_marginata
0,35
V2XAM2_Moniliophthora_roreri
0,76
V2X8Z2_Moniliophthora_roreri
1
1
V2XT34_Moniliophthora_roreri
0,98
A0A0C9UWV4_Sphaerobolus_stellatus
0,99
Fcan01_00181_PA
M1VDM5_Cyanidioschyzon_merolae
0,42
L9WN57_Natronolimnobius_innermongolicus
0,99
U1PDI2_Haloquadratum_walsbyi
1
D2S001_Haloterrigena_turkmenica
1
B3G4H7_Adineta_vaga
0,66
A9RWM2_Physcomitrella_patens
0,05
0,11
A0A0G4HYL0_Chromera_velia
0,91
R1DE16_Emiliania_huxleyi
T0SIA9_Saprolegnia_diclina
1
A0A067BV23_Saprolegnia_parasitica
B4I8X8_Drosophila_sechellia
0,95
0,92
Q9W1E9_Drosophila_melanogaster
0,85
B3MCN8_Drosophila_ananassae
1
B3NQ36_Drosophila_erecta
0,94
G6D5Z2_Danaus_plexippus
0,95
G3WW11_Sarcophilus_harrisii
S8C8Y9_Genlisea_aurea
0,61
0,98
1
A0A059CP19_Eucalyptus_grandis
0,99
A0A0G3FH16_Marchantia_polymorpha
0,05
1
M0CXF5_Halogeometricum_pallidum
0,88
M1XRH9_Natronomonas_moolapensis
F2E1L8_Hordeum_vulgare
E4WR80_Oikopleura_dioica
E4YZS2_Oikopleura_dioica
E4XWX5_Oikopleura_dioica
0.6

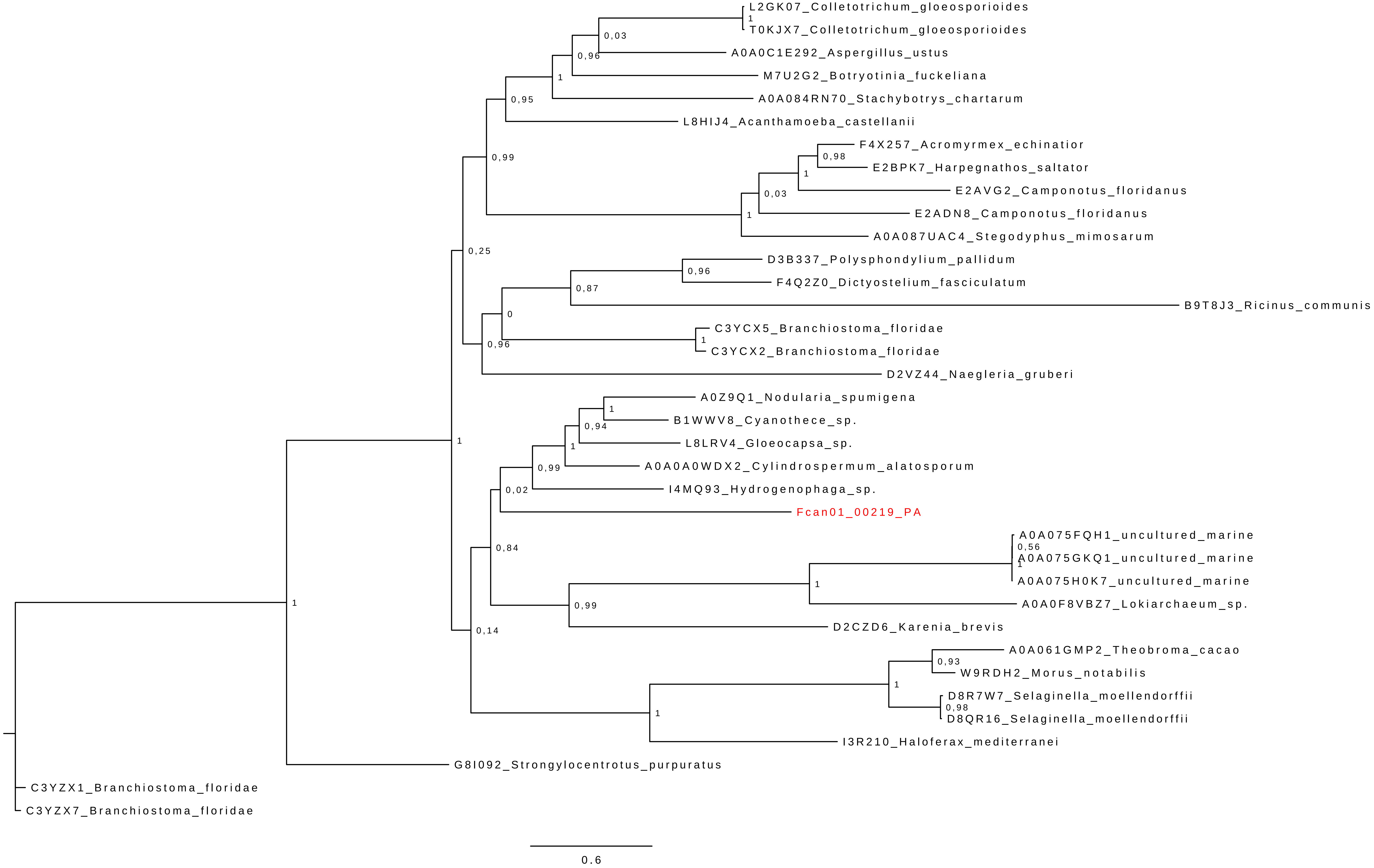
L2GK07_Colletotrichum_gloeosporioides
T0KJX7_Colletotrichum_gloeosporioides
A0A0C1E292_Aspergillus_ustus
M7U2G2_Botryotinia_fuckeliana
A0A084RN70_Stachybotrys_chartarum
L8HIJ4_Acanthamoeba_castellanii
F4X257_Acromyrmex_echinatior
E2BPK7_Harpegnathos_saltator
E2AVG2_Camponotus_floridanus
E2ADN8_Camponotus_floridanus
A0A087UAC4_Stegodyphus_mimosarum
D3B337_Polysphondylium_pallidum
F4Q2Z0_Dictyostelium_fasciculatum
B9T8J3_Ricinus_communis
C3YCX5_Branchiostoma_floridae
C3YCX2_Branchiostoma_floridae
D2VZ44_Naegleria_gruberi
A0Z9Q1_Nodularia_spumigena
B1WWV8_Cyanothece_sp.
L8LRV4_Gloeocapsa_sp.
A0A0A0WDX2_Cylindrospermum_alatosporum
I4MQ93_Hydrogenophaga_sp.
Fcan01_00219_PA
A0A075FQH1_uncultured_marine
A0A075GKQ1_uncultured_marine
A0A075H0K7_uncultured_marine
A0A0F8VBZ7_Lokiarchaeum_sp.
D2CZD6_Karenia_brevis
A0A061GMP2_Theobroma_cacao
W9RDH2_Morus_notabilis
D8R7W7_Selaginella_moellendorffii
D8QR16_Selaginella_moellendorffii
I3R210_Haloferax_mediterranei
G8I092_Strongylocentrotus_purpuratus
C3YZX1_Branchiostoma_floridae
C3YZX7_Branchiostoma_floridae
0,03
0,96
0,95
0,99
0,98
0,25
0,87
0,96
0,94
0,99
0,02
0,84
0,56
0,14
0,93
0,98
0.6

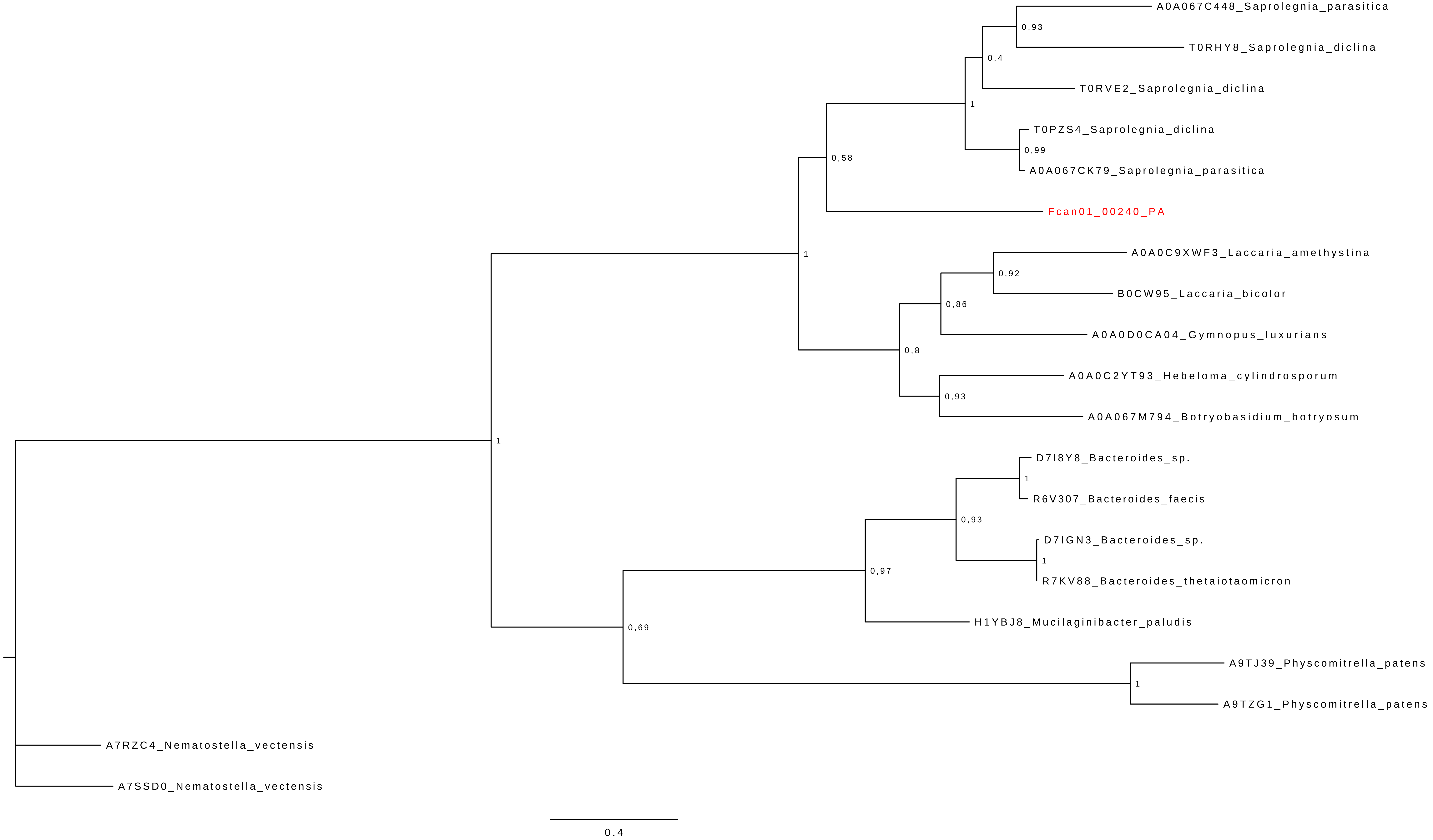
A0A067C448_Saprolegnia_parasitica
0,93
T0RHY8_Saprolegnia_diclina
0,4
T0RVE2_Saprolegnia_diclina
1
T0PZS4_Saprolegnia_diclina
0,99
A0A067CK79_Saprolegnia_parasitica
0,58
Fcan01_00240_PA
1
A0A0C9XWF3_Laccaria_amethystina
0,92
B0CW95_Laccaria_bicolor
0,86
A0A0D0CA04_Gymnopus_luxurians
0,8
A0A0C2YT93_Hebeloma_cylindrosporum
0,93
A0A067M794_Botryobasidium_botryosum
1
D7I8Y8_Bacteroides_sp.
1
R6V307_Bacteroides_faecis
0,93
D7IGN3_Bacteroides_sp.
1
R7KV88_Bacteroides_thetaiotaomicron
0,97
H1YBJ8_Mucilaginibacter_paludis
0,69
A9TJ39_Physcomitrella_patens
1
A9TZG1_Physcomitrella_patens
A7RZC4_Nematostella_vectensis
A7SSD0_Nematostella_vectensis
0.4

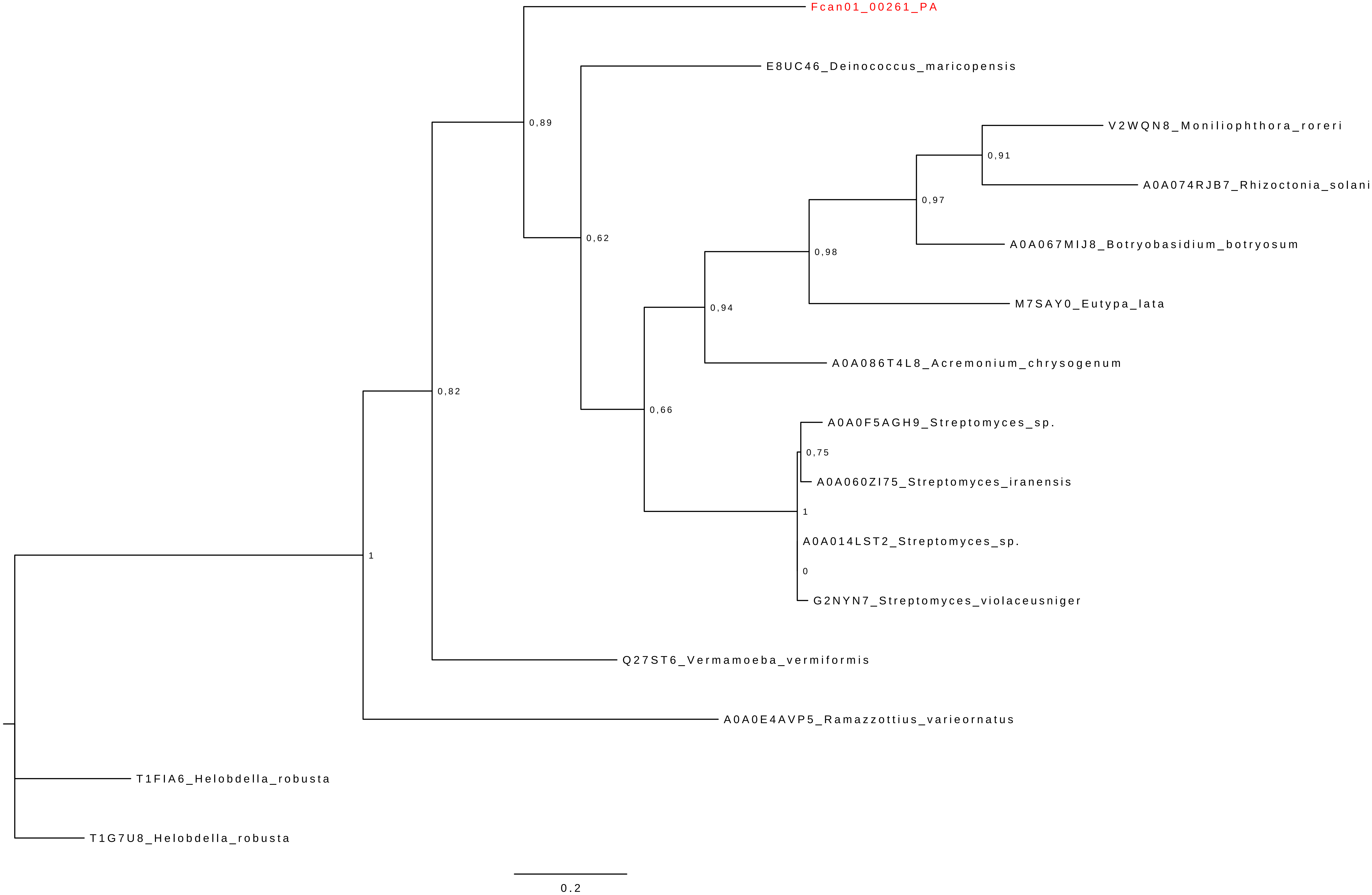

Fcan01_00261_PA
E8UC46_Deinococcus_maricopensis
V2WQN8_Moniliophthora_roreri
A0A074RJB7_Rhizoctonia_solani
A0A067MIJ8_Botryobasidium_botryosum
M7SAY0_Eutypa_lata
A0A086T4L8_Acremonium_chrysogenum
A0A0F5AGH9_Streptomyces_sp.
A0A060ZI75_Streptomyces_iranensis
A0A014LST2_Streptomyces_sp.
G2NYN7_Streptomyces_violaceusniger
Q27ST6_Vermamoeba_vermiformis
A0A0E4AVP5_Ramazzottius_varieornatus
T1FIA6_Helobdella_robusta
T1G7U8_Helobdella_robusta
0,89
0,62
0,91
0,97
0,98
0,94
0,82
0,66
0,75
1
0
1
0.2

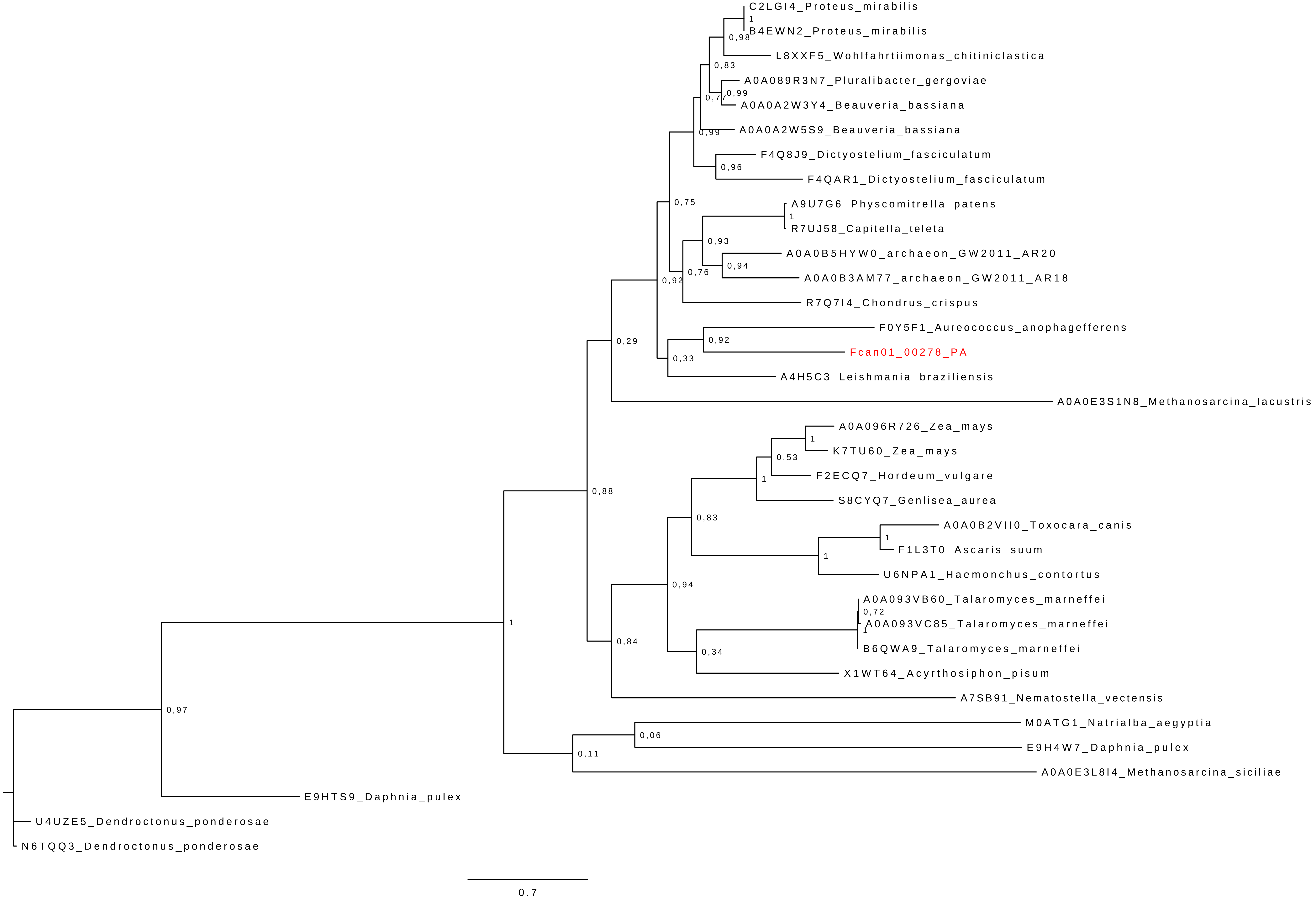
C2LGI4_Proteus_mirabilis
B4EWN2_Proteus_mirabilis
L8XXF5_Wohlfahrtiimonas_chitiniclastica
A0A089R3N7_Pluralibacter_gergoviae
A0A0A2W3Y4_Beauveria_bassiana
A0A0A2W5S9_Beauveria_bassiana
F4Q8J9_Dictyostelium_fasciculatum
F4QAR1_Dictyostelium_fasciculatum
A9U7G6_Physcomitrella_patens
R7UJ58_Capitella_teleta
A0A0B5HYW0_archaeon_GW2011_AR20
A0A0B3AM77_archaeon_GW2011_AR18
R7Q7I4_Chondrus_crispus
F0Y5F1_Aureococcus_anophagefferens
Fcan01_00278_PA
A4H5C3_Leishmania_braziliensis
A0A0E3S1N8_Methanosarcina_lacustris
A0A096R726_Zea_mays
K7TU60_Zea_mays
F2ECQ7_Hordeum_vulgare
S8CYQ7_Genlisea_aurea
A0A0B2VII0_Toxocara_canis
F1L3T0_Ascaris_suum
U6NPA1_Haemonchus_contortus
A0A093VB60_Talaromyces_marneffei
A0A093VC85_Talaromyces_marneffei
B6QWA9_Talaromyces_marneffei
X1WT64_Acyrthosiphon_pisum
A7SB91_Nematostella_vectensis
M0ATG1_Natrialba_aegyptia
E9H4W7_Daphnia_pulex
A0A0E3L8I4_Methanosarcina_siciliae
E9HTS9_Daphnia_pulex
U4UZE5_Dendroctonus_ponderosae
N6TQQ3_Dendroctonus_ponderosae
0.7

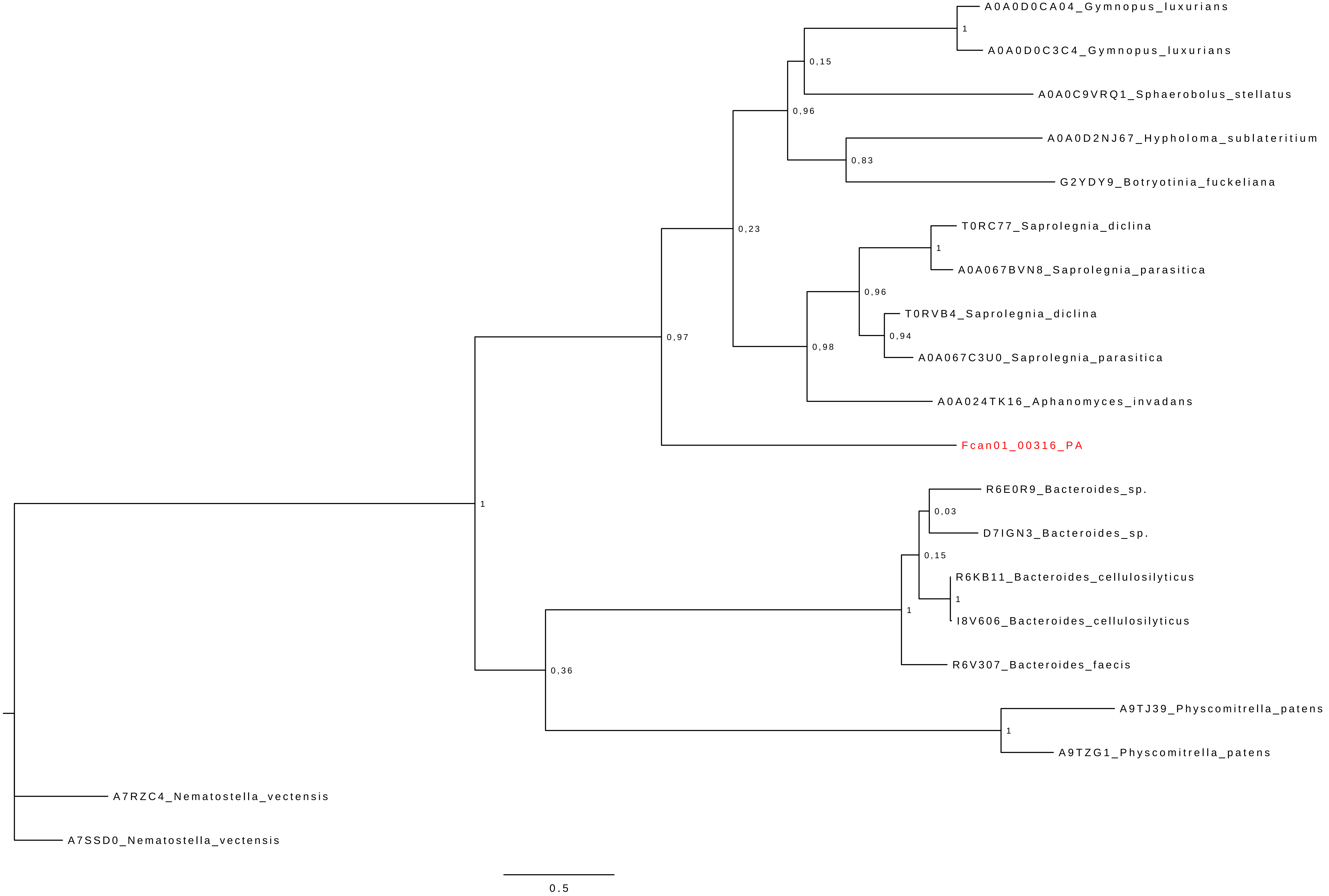
A0A0D0CA04_Gymnopus_luxurians
1
A0A0D0C3C4_Gymnopus_luxurians
0,15
A0A0C9VRQ1_Sphaerobolus_stellatus
0,96
A0A0D2NJ67_Hypholoma_sublateritium
0,83
G2YDY9_Botryotinia_fuckeliana
0,23
T0RC77_Saprolegnia_diclina
1
A0A067BVN8_Saprolegnia_parasitica
0,96
T0RVB4_Saprolegnia_diclina
0,94
A0A067C3U0_Saprolegnia_parasitica
0,98
0,97
A0A024TK16_Aphanomyces_invadans
Fcan01_00316_PA
1
R6E0R9_Bacteroides_sp.
0,03
D7IGN3_Bacteroides_sp.
0,15
R6KB11_Bacteroides_cellulosilyticus
1
I8V606_Bacteroides_cellulosilyticus
1
R6V307_Bacteroides_faecis
0,36
A9TJ39_Physcomitrella_patens
1
A9TZG1_Physcomitrella_patens
A7RZC4_Nematostella_vectensis
A7SSD0_Nematostella_vectensis
0.5

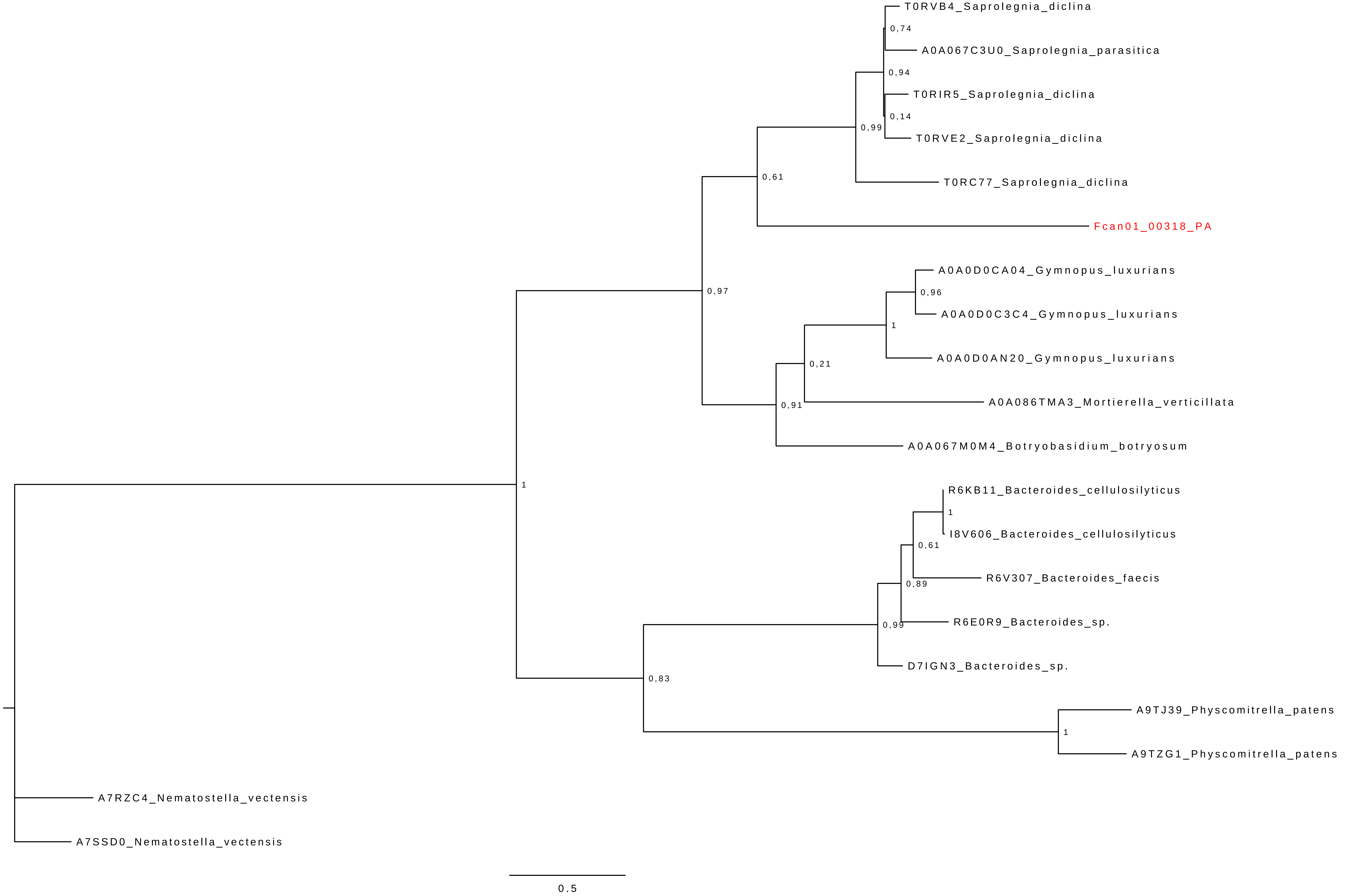

T0RVB4_Saprolegnia_diclina
0,74
A0A067C3U0_Saprolegnia_parasitica
0,94
T0RIR5_Saprolegnia_diclina
0,14
0,99
T0RVE2_Saprolegnia_diclina
0,61
T0RC77_Saprolegnia_diclina
Fcan01_00318_PA
A0A0D0CA04_Gymnopus_luxurians
0,97
0,96
A0A0D0C3C4_Gymnopus_luxurians
1
A0A0D0AN20_Gymnopus_luxurians
0,21
A0A086TMA3_Mortierella_verticillata
0,91
A0A067M0M4_Botryobasidium_botryosum
1
R6KB11_Bacteroides_cellulosilyticus
1
I8V606_Bacteroides_cellulosilyticus
0,61
R6V307_Bacteroides_faecis
0,89
R6E0R9_Bacteroides_sp.
0,99
0,83
D7IGN3_Bacteroides_sp.
A9TJ39_Physcomitrella_patens
1
A9TZG1_Physcomitrella_patens
A7RZC4_Nematostella_vectensis
A7SSD0_Nematostella_vectensis
0.5

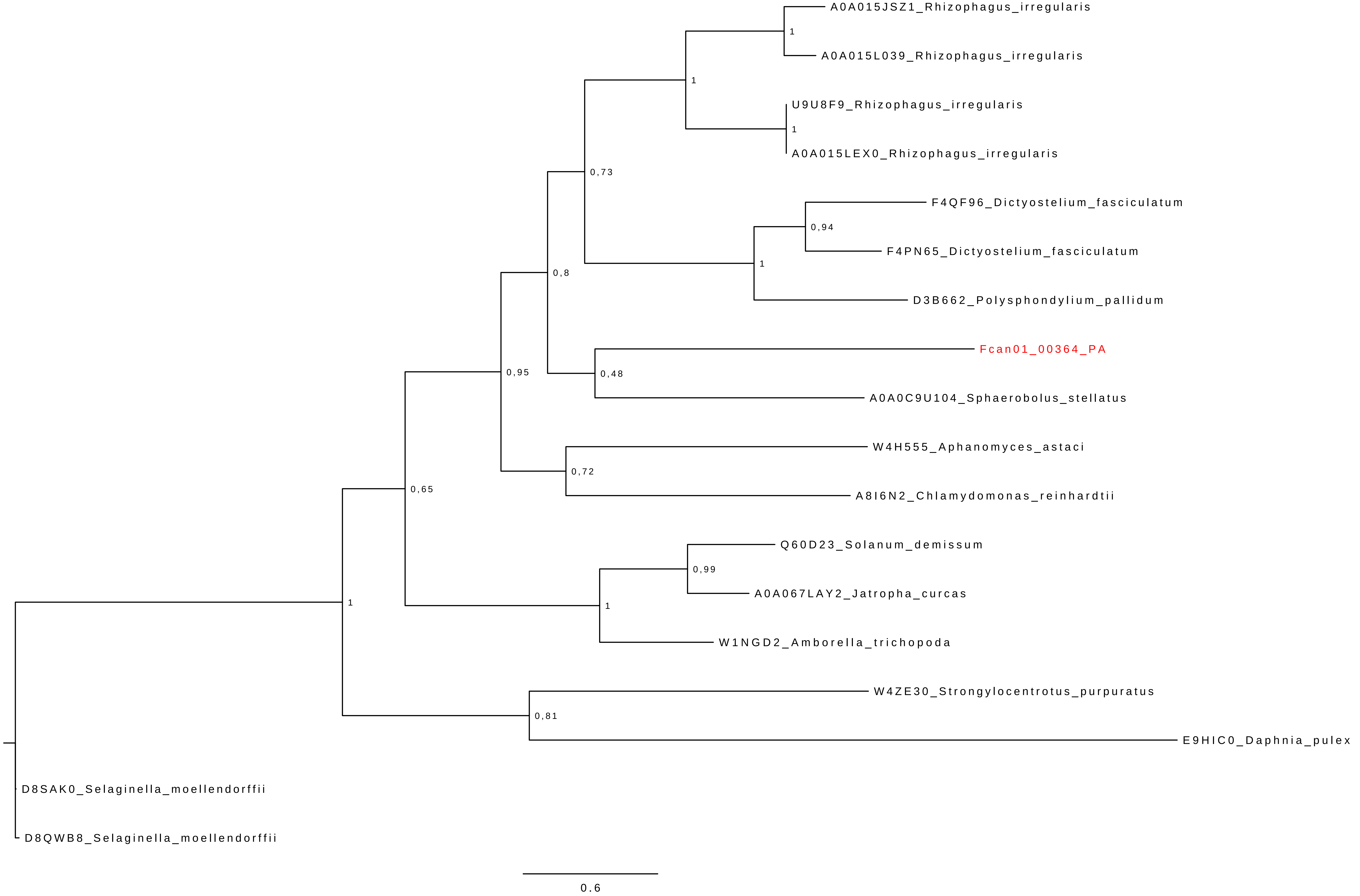
A0A015JSZ1_Rhizophagus_irregularis
A0A015L039_Rhizophagus_irregularis
U9U8F9_Rhizophagus_irregularis
A0A015LEX0_Rhizophagus_irregularis
F4QF96_Dictyostelium_fasciculatum
F4PN65_Dictyostelium_fasciculatum
D3B662_Polysphondylium_pallidum
Fcan01_00364_PA
A0A0C9U104_Sphaerobolus_stellatus
W4H555_Aphanomyces_astaci
A8I6N2_Chlamydomonas_reinhardtii
Q60D23_Solanum_demissum
A0A067LAY2_Jatropha_curcas
W1NGD2_Amborella_trichopoda
W4ZE30_Strongylocentrotus_purpuratus
E9HIC0_Daphnia_pulex
D8SAK0_Selaginella_moellendorffii
D8QWB8_Selaginella_moellendorffii
1
1
1
0,73
0,94
1
0,8
0,48
0,95
0,72
0,65
0,99
1
1
0,81
0.6

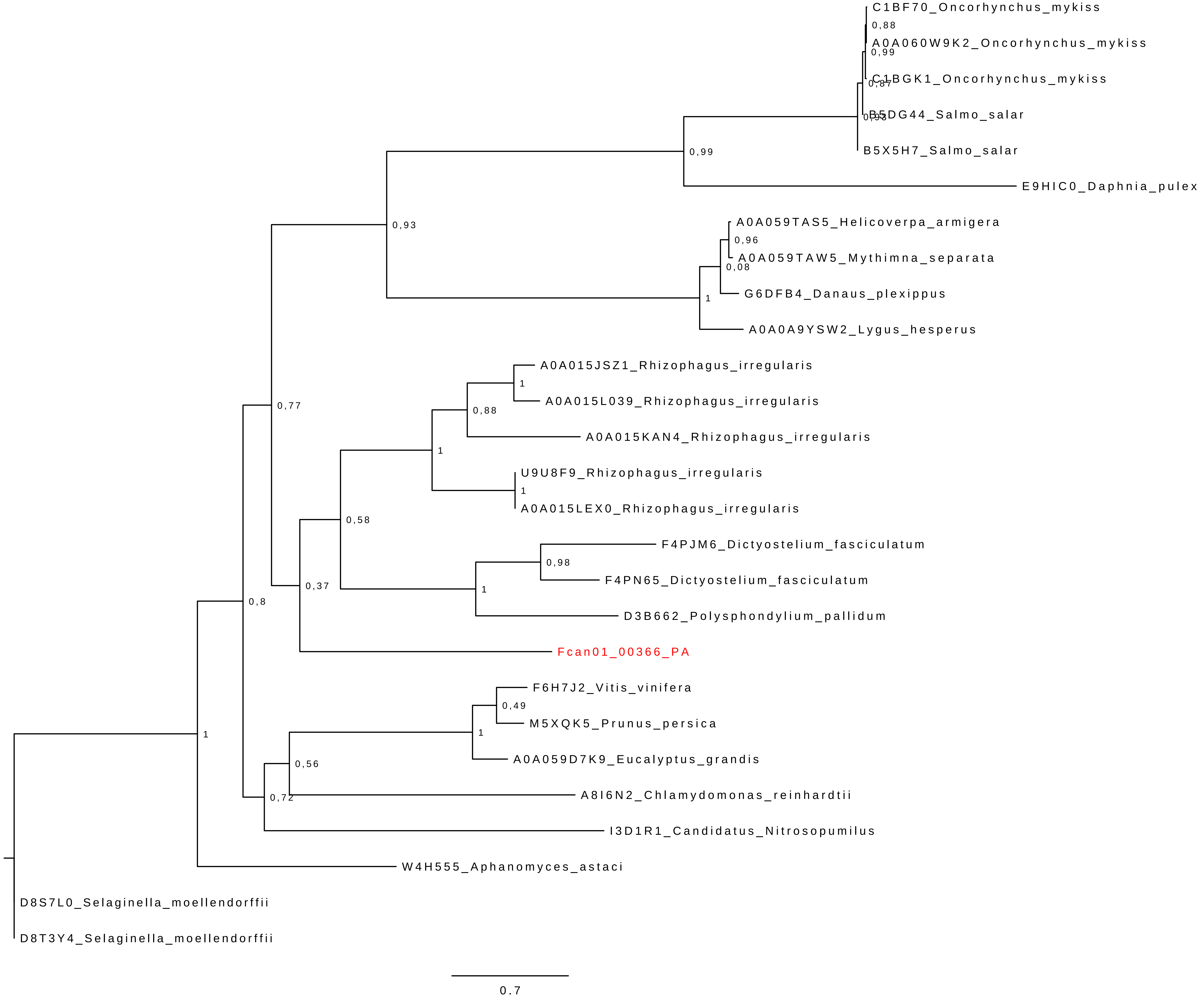

C1BF70_Oncorhynchus_mykiss
0,88
A0A060W9K2_Oncorhynchus_mykiss
0,99
C1BGK1_Oncorhynchus_mykiss
0,87
B5DG44_Salmo_salar
0,93
B5X5H7_Salmo_salar
0,99
E9HIC0_Daphnia_pulex
0,93
A0A059TAS5_Helicoverpa_armigera
0,96
A0A059TAW5_Mythimna_separata
0,08
G6DFB4_Danaus_plexippus
1
A0A0A9YSW2_Lygus_hesperus
A0A015JSZ1_Rhizophagus_irregularis
1
A0A015L039_Rhizophagus_irregularis
0,88
0,77
A0A015KAN4_Rhizophagus_irregularis
1
U9U8F9_Rhizophagus_irregularis
1
A0A015LEX0_Rhizophagus_irregularis
0,58
F4PJM6_Dictyostelium_fasciculatum
0,98
F4PN65_Dictyostelium_fasciculatum
0,37
1
0,8
D3B662_Polysphondylium_pallidum
Fcan01_00366_PA
F6H7J2_Vitis_vinifera
0,49
M5XQK5_Prunus_persica
1
1
A0A059D7K9_Eucalyptus_grandis
0,56
A8I6N2_Chlamydomonas_reinhardtii
0,72
I3D1R1_Candidatus_Nitrosopumilus
W4H555_Aphanomyces_astaci
D8S7L0_Selaginella_moellendorffii
D8T3Y4_Selaginella_moellendorffii
0.7

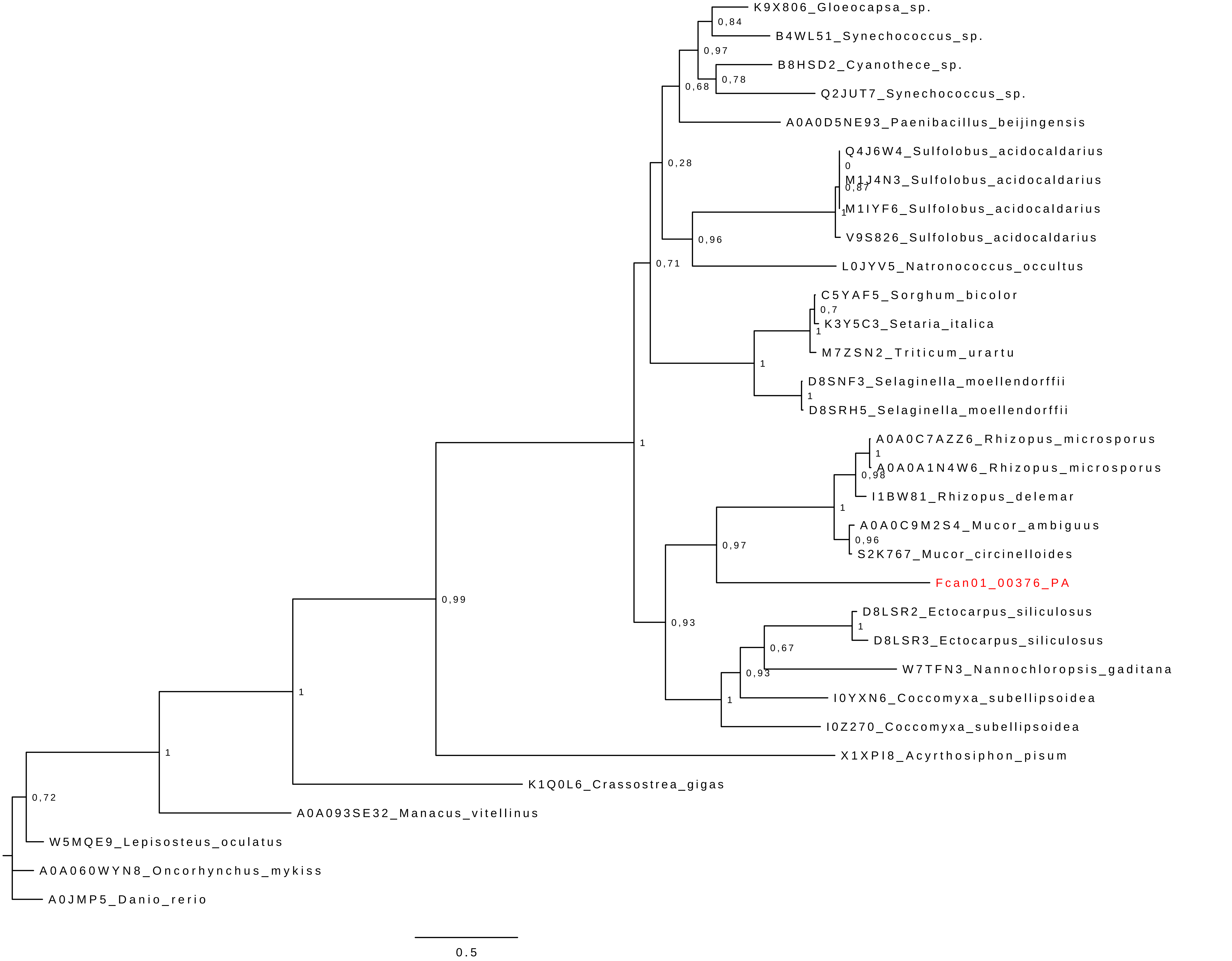
K9X806_Gloeocapsa_sp.
B4WL51_Synechococcus_sp.
B8HSD2_Cyanothece_sp.
Q2JUT7_Synechococcus_sp.
A0A0D5NE93_Paenibacillus_beijingensis
Q4J6W4_Sulfolobus_acidocaldarius
M1J4N3_Sulfolobus_acidocaldarius
M1IYF6_Sulfolobus_acidocaldarius
V9S826_Sulfolobus_acidocaldarius
L0JYV5_Natronococcus_occultus
C5YAF5_Sorghum_bicolor
K3Y5C3_Setaria_italica
M7ZSN2_Triticum_urartu
D8SNF3_Selaginella_moellendorffii
D8SRH5_Selaginella_moellendorffii
A0A0C7AZZ6_Rhizopus_microsporus
A0A0A1N4W6_Rhizopus_microsporus
I1BW81_Rhizopus_delemar
A0A0C9M2S4_Mucor_ambiguus
S2K767_Mucor_circinelloides
Fcan01_00376_PA
D8LSR2_Ectocarpus_siliculosus
D8LSR3_Ectocarpus_siliculosus
W7TFN3_Nannochloropsis_gaditana
I0YXN6_Coccomyxa_subellipsoidea
I0Z270_Coccomyxa_subellipsoidea
X1XPI8_Acyrthosiphon_pisum
K1Q0L6_Crassostrea_gigas
A0A093SE32_Manacus_vitellinus
W5MQE9_Lepisosteus_oculatus
A0A060WYN8_Oncorhynchus_mykiss
A0JMP5_Danio_rerio
0,84
0,97
0,78
0,68
0,28
0
0,87
1
0,96
0,71
0,7
1
1
1
1
1
0,98
1
0,96
0,97
1
0,67
0,93
1
0,93
0,99
1
1
0,72
0.5

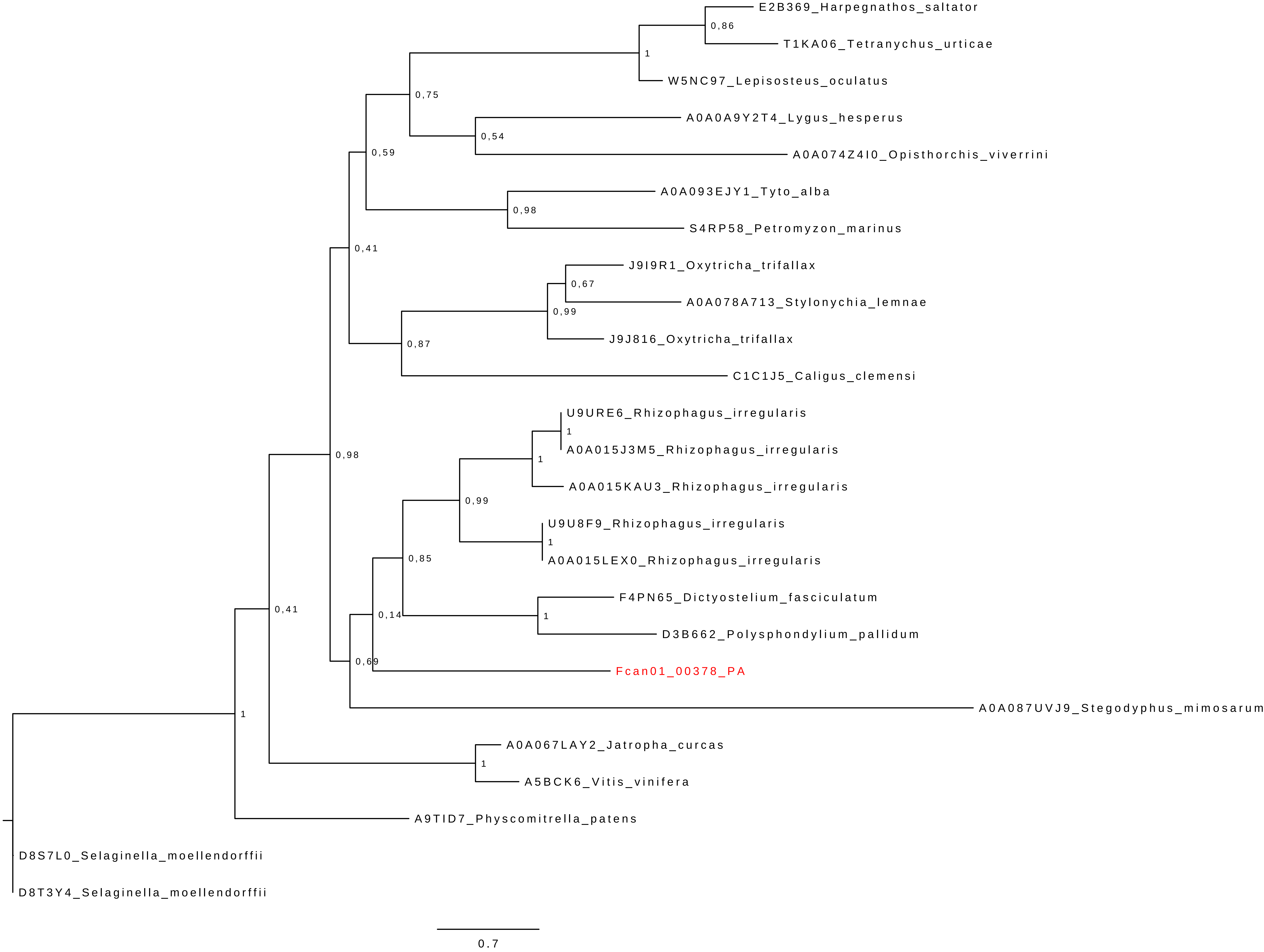

E2B369_Harpegnathos_saltator
0,86
T1KA06_Tetranychus_urticae
1
W5NC97_Lepisosteus_oculatus
0,75
A0A0A9Y2T4_Lygus_hesperus
0,54
A0A074Z4I0_Opisthorchis_viverrini
0,59
A0A093EJY1_Tyto_alba
0,98
S4RP58_Petromyzon_marinus
0,41
J9I9R1_Oxytricha_trifallax
0,67
A0A078A713_Stylonychia_lemnae
0,99
J9J816_Oxytricha_trifallax
0,87
C1C1J5_Caligus_clemensi
U9URE6_Rhizophagus_irregularis
1
A0A015J3M5_Rhizophagus_irregularis
0,98
1
A0A015KAU3_Rhizophagus_irregularis
0,99
U9U8F9_Rhizophagus_irregularis
1
A0A015LEX0_Rhizophagus_irregularis
0,85
F4PN65_Dictyostelium_fasciculatum
0,41
0,14
1
D3B662_Polysphondylium_pallidum
0,69
Fcan01_00378_PA
A0A087UVJ9_Stegodyphus_mimosarum
1
A0A067LAY2_Jatropha_curcas
1
A5BCK6_Vitis_vinifera
A9TID7_Physcomitrella_patens
D8S7L0_Selaginella_moellendorffii
D8T3Y4_Selaginella_moellendorffii
0.7

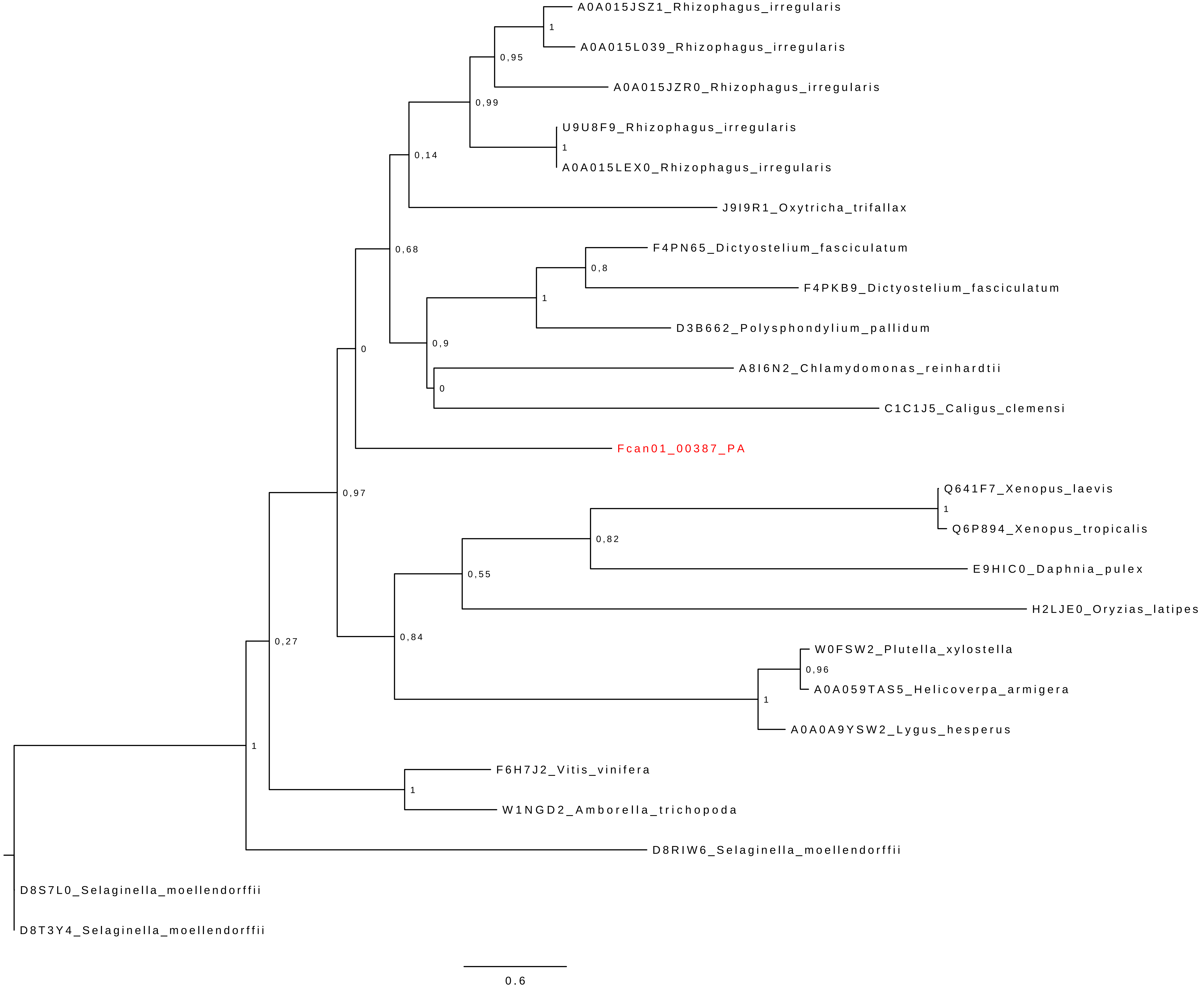
A0A015JSZ1_Rhizophagus_irregularis
1
A0A015L039_Rhizophagus_irregularis
0,95
A0A015JZR0_Rhizophagus_irregularis
0,99
U9U8F9_Rhizophagus_irregularis
1
A0A015LEX0_Rhizophagus_irregularis
0,14
J9I9R1_Oxytricha_trifallax
F4PN65_Dictyostelium_fasciculatum
0,68
0,8
F4PKB9_Dictyostelium_fasciculatum
1
D3B662_Polysphondylium_pallidum
0,9
0
A8I6N2_Chlamydomonas_reinhardtii
0
C1C1J5_Caligus_clemensi
Fcan01_00387_PA
Q641F7_Xenopus_laevis
0,97
1
Q6P894_Xenopus_tropicalis
0,82
E9HIC0_Daphnia_pulex
0,55
H2LJE0_Oryzias_latipes
0,27
0,84
W0FSW2_Plutella_xylostella
0,96
A0A059TAS5_Helicoverpa_armigera
1
A0A0A9YSW2_Lygus_hesperus
1
F6H7J2_Vitis_vinifera
1
W1NGD2_Amborella_trichopoda
D8RIW6_Selaginella_moellendorffii
D8S7L0_Selaginella_moellendorffii
D8T3Y4_Selaginella_moellendorffii
0.6

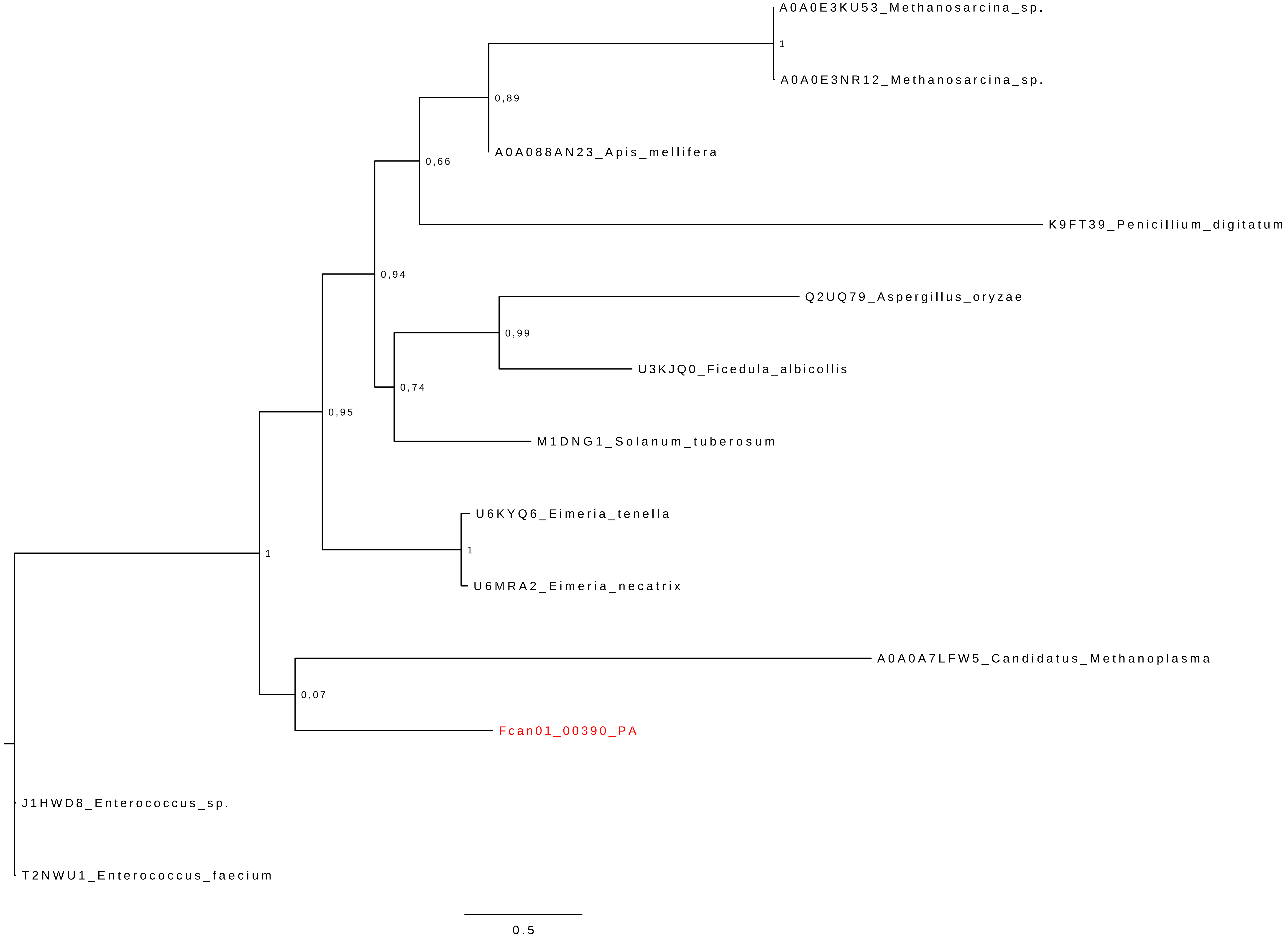
A0A0E3KU53_Methanosarcina_sp.
1
A0A0E3NR12_Methanosarcina_sp.
0,89
A0A088AN23_Apis_mellifera
0,66
K9FT39_Penicillium_digitatum
0,94
Q2UQ79_Aspergillus_oryzae
0,99
U3KJQ0_Ficedula_albicollis
0,74
M1DNG1_Solanum_tuberosum
0,95
U6KYQ6_Eimeria_tenella
1
1
U6MRA2_Eimeria_necatrix
A0A0A7LFW5_Candidatus_Methanoplasma
0,07
Fcan01_00390_PA
J1HWD8_Enterococcus_sp.
T2NWU1_Enterococcus_faecium
0.5

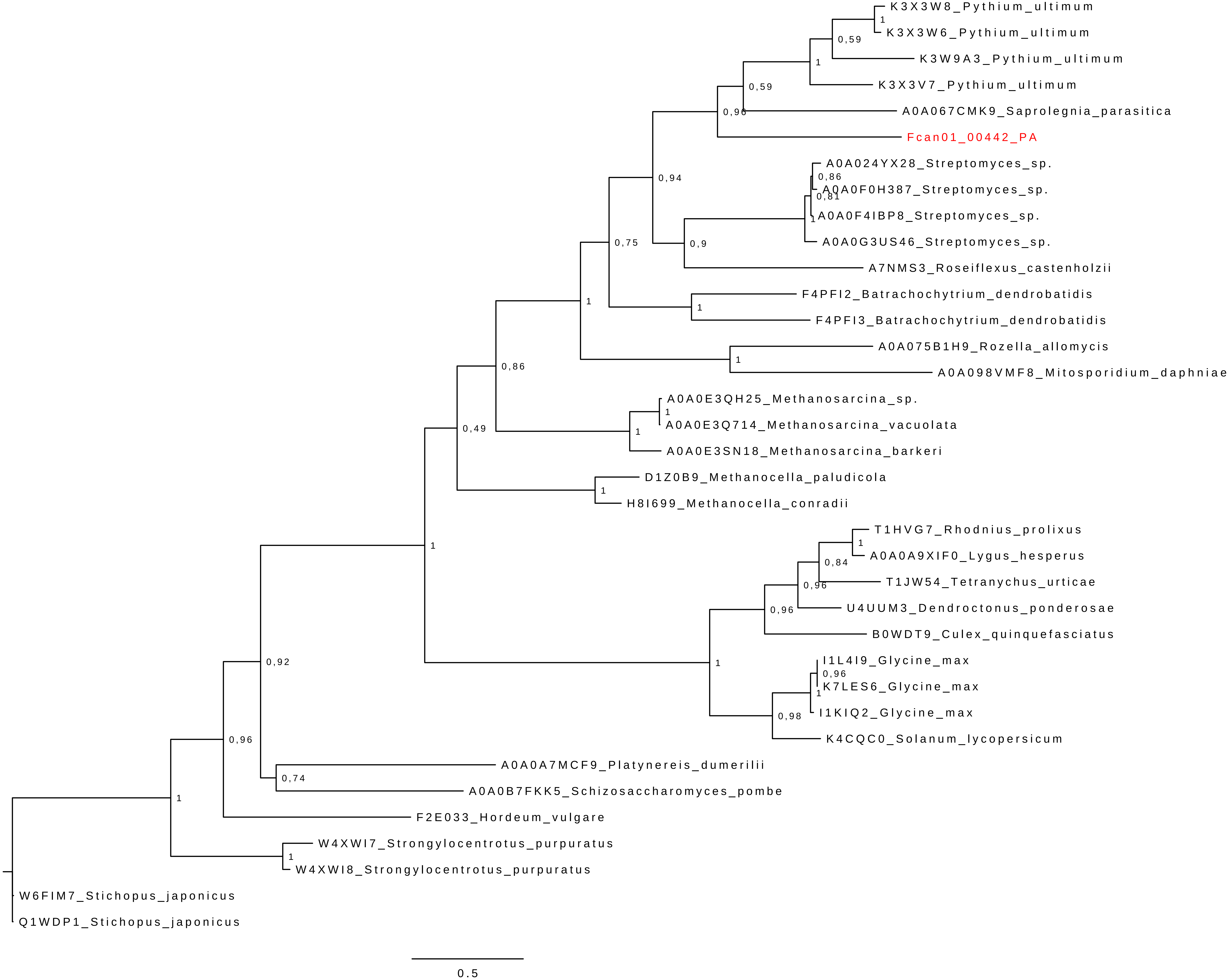
K3X3W8_Pythium_ultimum
K3X3W6_Pythium_ultimum
K3W9A3_Pythium_ultimum
K3X3V7_Pythium_ultimum
A0A067CMK9_Saprolegnia_parasitica
Fcan01_00442_PA
A0A024YX28_Streptomyces_sp.
A0A0F0H387_Streptomyces_sp.
A0A0F4IBP8_Streptomyces_sp.
A0A0G3US46_Streptomyces_sp.
A7NMS3_Roseiflexus_castenholzii
F4PFI2_Batrachochytrium_dendrobatidis
F4PFI3_Batrachochytrium_dendrobatidis
A0A075B1H9_Rozella_allomycis
A0A098VMF8_Mitosporidium_daphniae
A0A0E3QH25_Methanosarcina_sp.
A0A0E3Q714_Methanosarcina_vacuolata
A0A0E3SN18_Methanosarcina_barkeri
D1Z0B9_Methanocella_paludicola
H8I699_Methanocella_conradii
T1HVG7_Rhodnius_prolixus
A0A0A9XIF0_Lygus_hesperus
T1JW54_Tetranychus_urticae
U4UUM3_Dendroctonus_ponderosae
B0WDT9_Culex_quinquefasciatus
I1L4I9_Glycine_max
K7LES6_Glycine_max
I1KIQ2_Glycine_max
K4CQC0_Solanum_lycopersicum
A0A0A7MCF9_Platynereis_dumerilii
A0A0B7FKK5_Schizosaccharomyces_pombe
F2E033_Hordeum_vulgare
W4XWI7_Strongylocentrotus_purpuratus
W4XWI8_Strongylocentrotus_purpuratus
W6FIM7_Stichopus_japonicus
Q1WDP1_Stichopus_japonicus
0.5

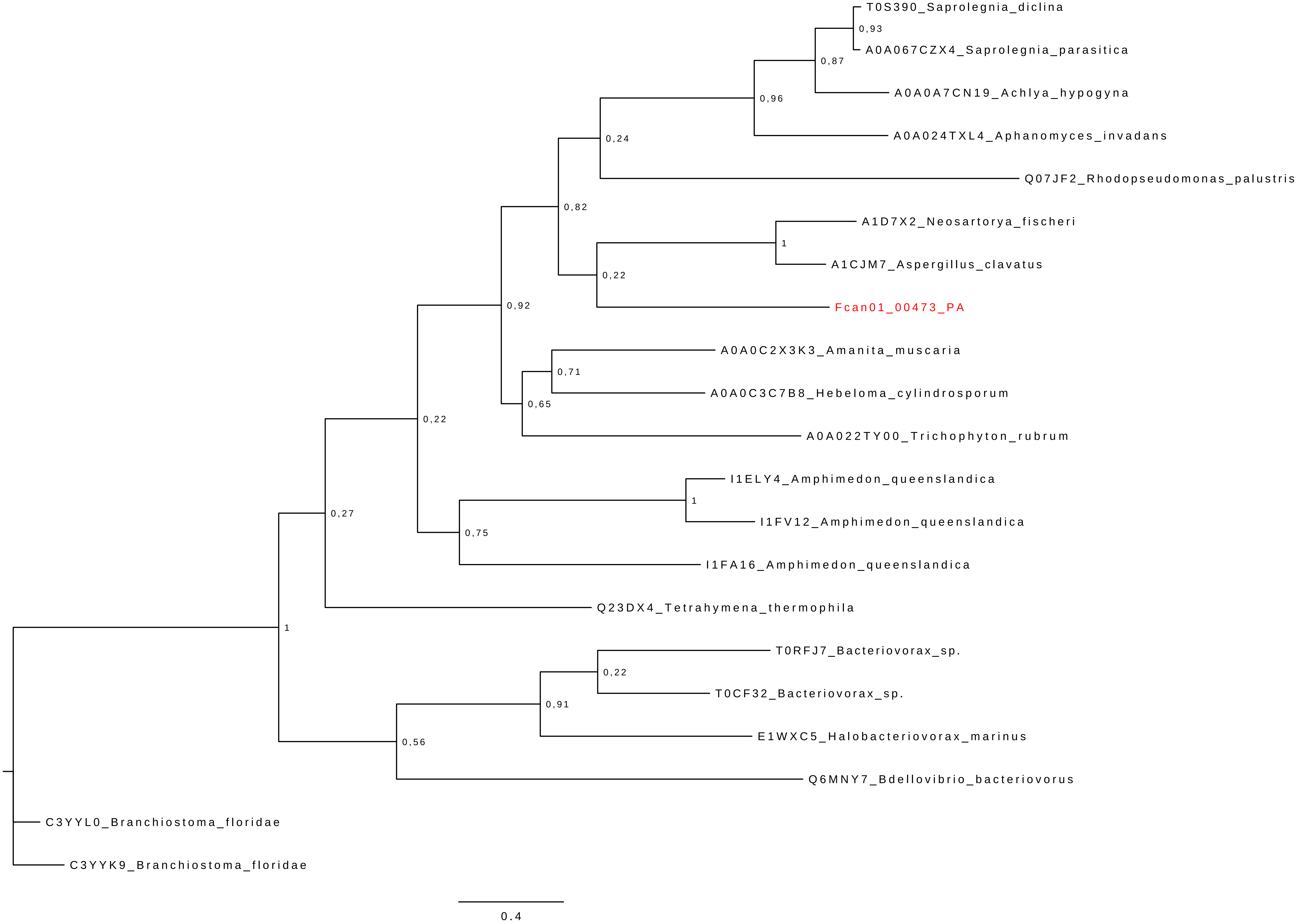

T0S390_Saprolegnia_diclina
0,93
A0A067CZX4_Saprolegnia_parasitica
0,87
A0A0A7CN19_Achlya_hypogyna
0,96
A0A024TXL4_Aphanomyces_invadans
0,24
Q07JF2_Rhodopseudomonas_palustris
0,82
A1D7X2_Neosartorya_fischeri
1
A1CJM7_Aspergillus_clavatus
0,22
Fcan01_00473_PA
0,92
A0A0C2X3K3_Amanita_muscaria
0,71
A0A0C3C7B8_Hebeloma_cylindrosporum
0,65
A0A022TY00_Trichophyton_rubrum
0,22
I1ELY4_Amphimedon_queenslandica
1
I1FV12_Amphimedon_queenslandica
0,75
I1FA16_Amphimedon_queenslandica
0,27
Q23DX4_Tetrahymena_thermophila
1
T0RFJ7_Bacteriovorax_sp.
0,22
T0CF32_Bacteriovorax_sp.
0,91
E1WXC5_Halobacteriovorax_marinus
0,56
Q6MNY7_Bdellovibrio_bacteriovorus
C3YYL0_Branchiostoma_floridae
C3YYK9_Branchiostoma_floridae
0.4

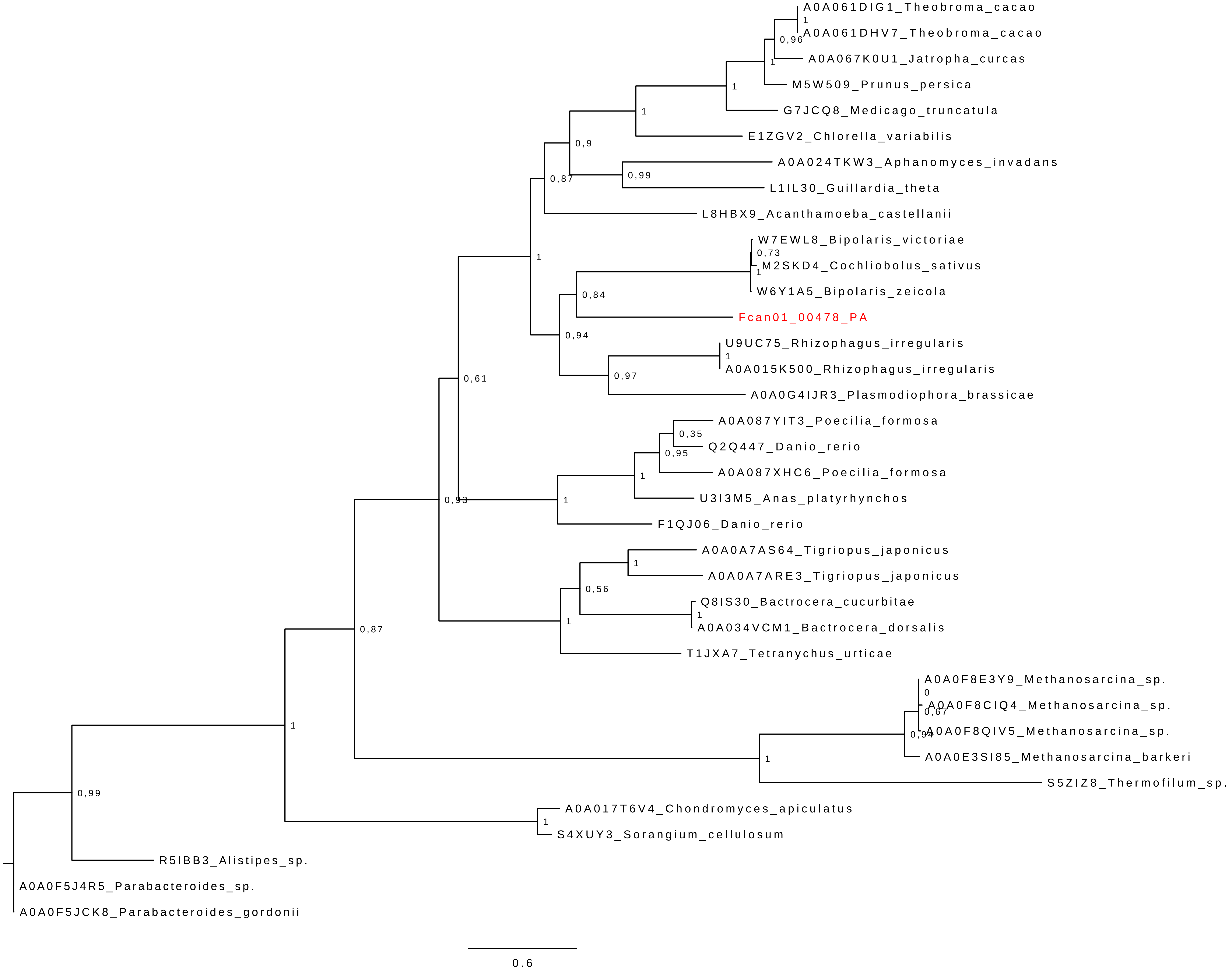
A0A061DIG1_Theobroma_cacao
1
A0A061DHV7_Theobroma_cacao
0,96
A0A067K0U1_Jatropha_curcas
1
M5W509_Prunus_persica
1
G7JCQ8_Medicago_truncatula
1
E1ZGV2_Chlorella_variabilis
0,9
A0A024TKW3_Aphanomyces_invadans
0,99
0,87
L1IL30_Guillardia_theta
L8HBX9_Acanthamoeba_castellanii
W7EWL8_Bipolaris_victoriae
0,73
M2SKD4_Cochliobolus_sativus
1
1
W6Y1A5_Bipolaris_zeicola
0,84
Fcan01_00478_PA
0,94
U9UC75_Rhizophagus_irregularis
1
A0A015K500_Rhizophagus_irregularis
0,97
0,61
A0A0G4IJR3_Plasmodiophora_brassicae
A0A087YIT3_Poecilia_formosa
0,35
Q2Q447_Danio_rerio
0,95
A0A087XHC6_Poecilia_formosa
1
U3I3M5_Anas_platyrhynchos
0,93
1
F1QJ06_Danio_rerio
A0A0A7AS64_Tigriopus_japonicus
1
A0A0A7ARE3_Tigriopus_japonicus
0,56
Q8IS30_Bactrocera_cucurbitae
1
1
0,87
A0A034VCM1_Bactrocera_dorsalis
T1JXA7_Tetranychus_urticae
A0A0F8E3Y9_Methanosarcina_sp.
0
A0A0F8CIQ4_Methanosarcina_sp.
0,67
0,94
A0A0F8QIV5_Methanosarcina_sp.
1
1
A0A0E3SI85_Methanosarcina_barkeri
S5ZIZ8_Thermofilum_sp.
0,99
A0A017T6V4_Chondromyces_apiculatus
1
S4XUY3_Sorangium_cellulosum
R5IBB3_Alistipes_sp.
A0A0F5J4R5_Parabacteroides_sp.
A0A0F5JCK8_Parabacteroides_gordonii
0.6

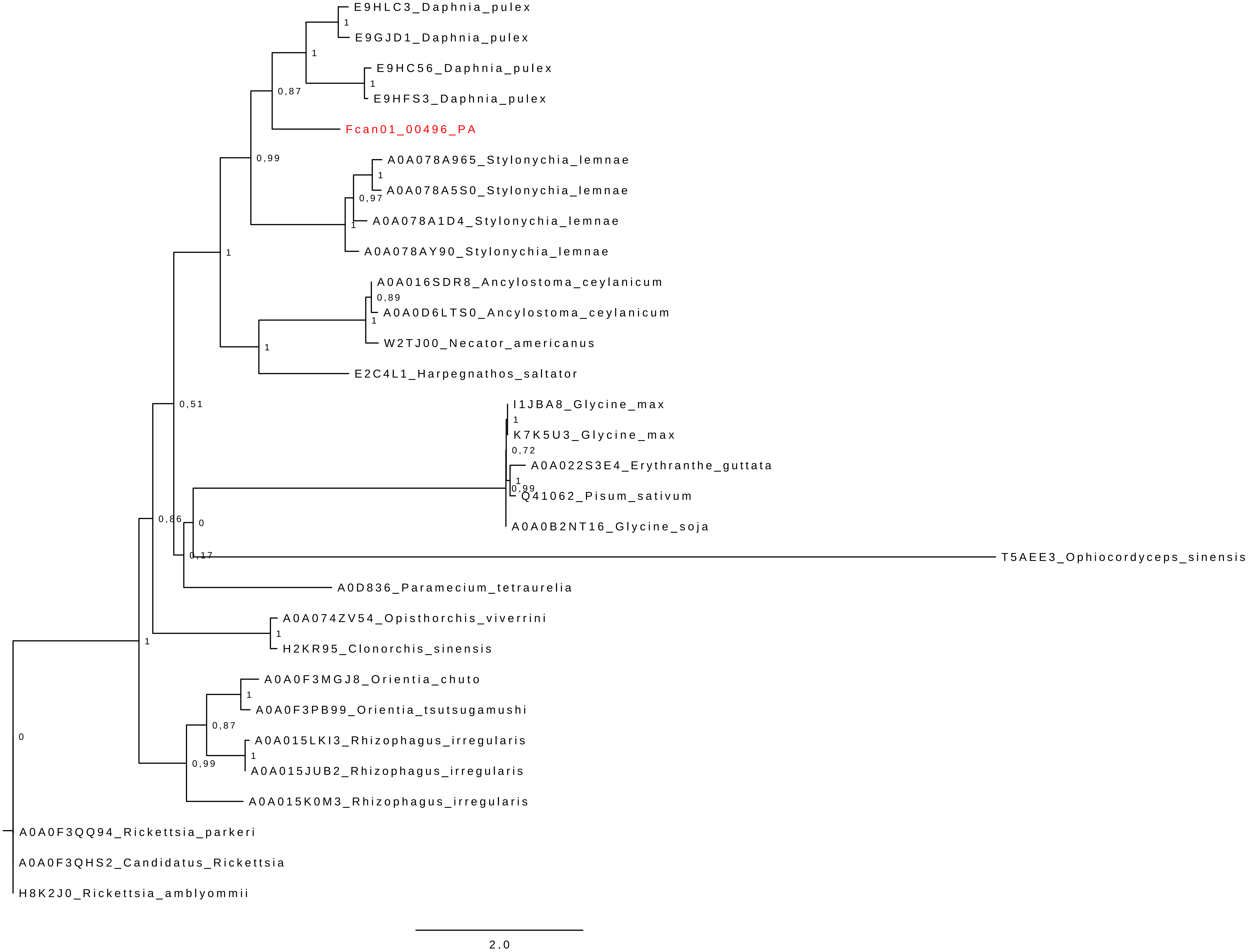

E9HLC3_Daphnia_pulex
1
E9GJD1_Daphnia_pulex
1
E9HC56_Daphnia_pulex
1
E9HFS3_Daphnia_pulex
0,87
Fcan01_00496_PA
0,99
A0A078A965_Stylonychia_lemnae
1
A0A078A5S0_Stylonychia_lemnae
0,97
A0A078A1D4_Stylonychia_lemnae
1
A0A078AY90_Stylonychia_lemnae
1
A0A016SDR8_Ancylostoma_ceylanicum
0,89
A0A0D6LTS0_Ancylostoma_ceylanicum
1
W2TJ00_Necator_americanus
1
E2C4L1_Harpegnathos_saltator
0,51
I1JBA8_Glycine_max
1
K7K5U3_Glycine_max
0,72
A0A022S3E4_Erythranthe_guttata
1
0,99
Q41062_Pisum_sativum
A0A0B2NT16_Glycine_soja
0,86
0
0,17
T5AEE3_Ophiocordyceps_sinensis
A0D836_Paramecium_tetraurelia
A0A074ZV54_Opisthorchis_viverrini
1
H2KR95_Clonorchis_sinensis
1
A0A0F3MGJ8_Orientia_chuto
1
A0A0F3PB99_Orientia_tsutsugamushi
0,87
A0A015LKI3_Rhizophagus_irregularis
1
A0A015JUB2_Rhizophagus_irregularis
0,99
A0A015K0M3_Rhizophagus_irregularis
0
A0A0F3QQ94_Rickettsia_parkeri
A0A0F3QHS2_Candidatus_Rickettsia
H8K2J0_Rickettsia_amblyommii
2.0

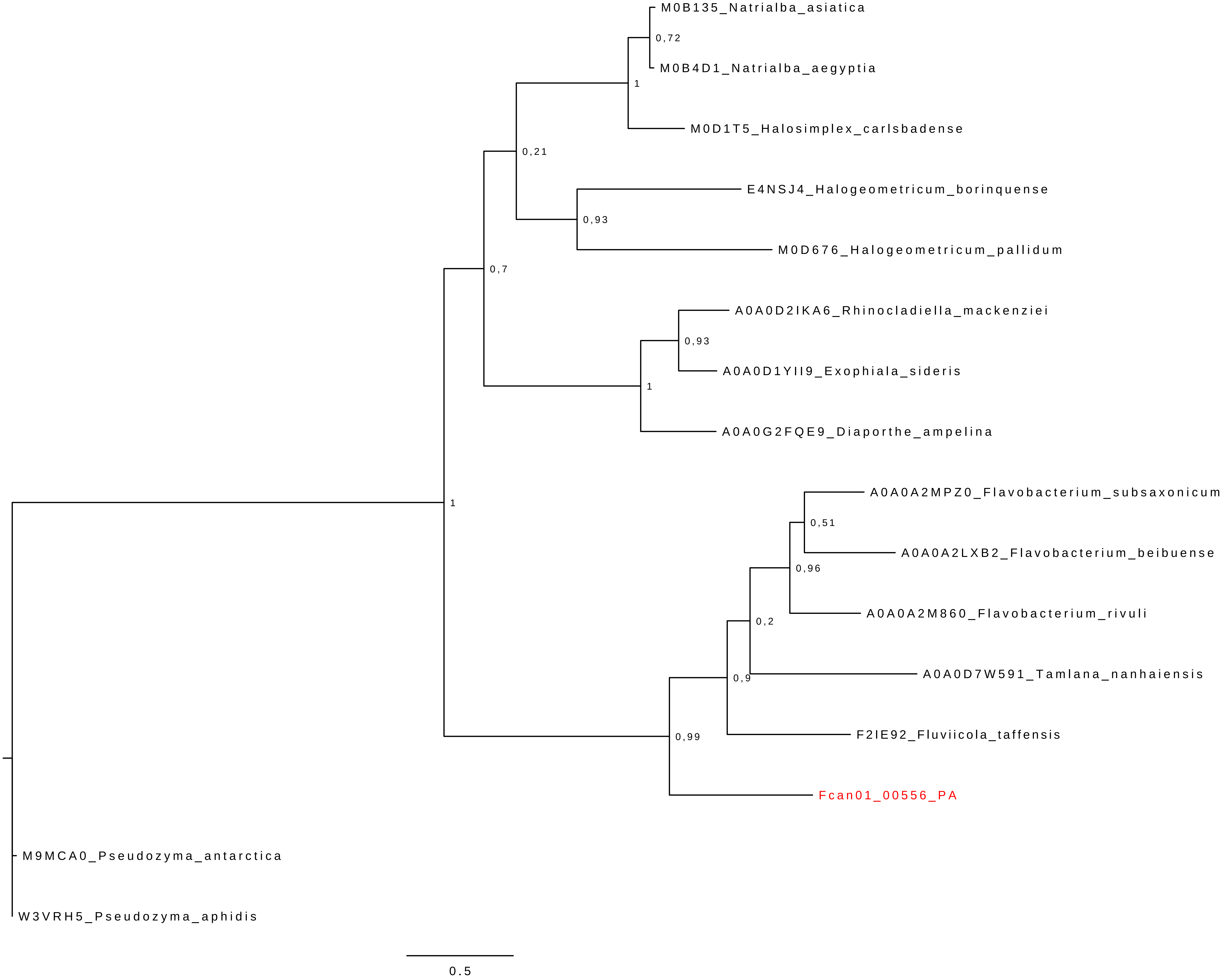
M0B135_Natrialba_asiatica
0,72
M0B4D1_Natrialba_aegyptia
1
M0D1T5_Halosimplex_carlsbadense
0,21
E4NSJ4_Halogeometricum_borinquense
0,93
M0D676_Halogeometricum_pallidum
0,7
A0A0D2IKA6_Rhinocladiella_mackenziei
0,93
A0A0D1YII9_Exophiala_sideris
1
A0A0G2FQE9_Diaporthe_ampelina
1
A0A0A2MPZ0_Flavobacterium_subsaxonicum
0,51
A0A0A2LXB2_Flavobacterium_beibuense
0,96
A0A0A2M860_Flavobacterium_rivuli
0,2
A0A0D7W591_Tamlana_nanhaiensis
0,9
F2IE92_Fluviicola_taffensis
0,99
Fcan01_00556_PA
M9MCA0_Pseudozyma_antarctica
W3VRH5_Pseudozyma_aphidis
0.5

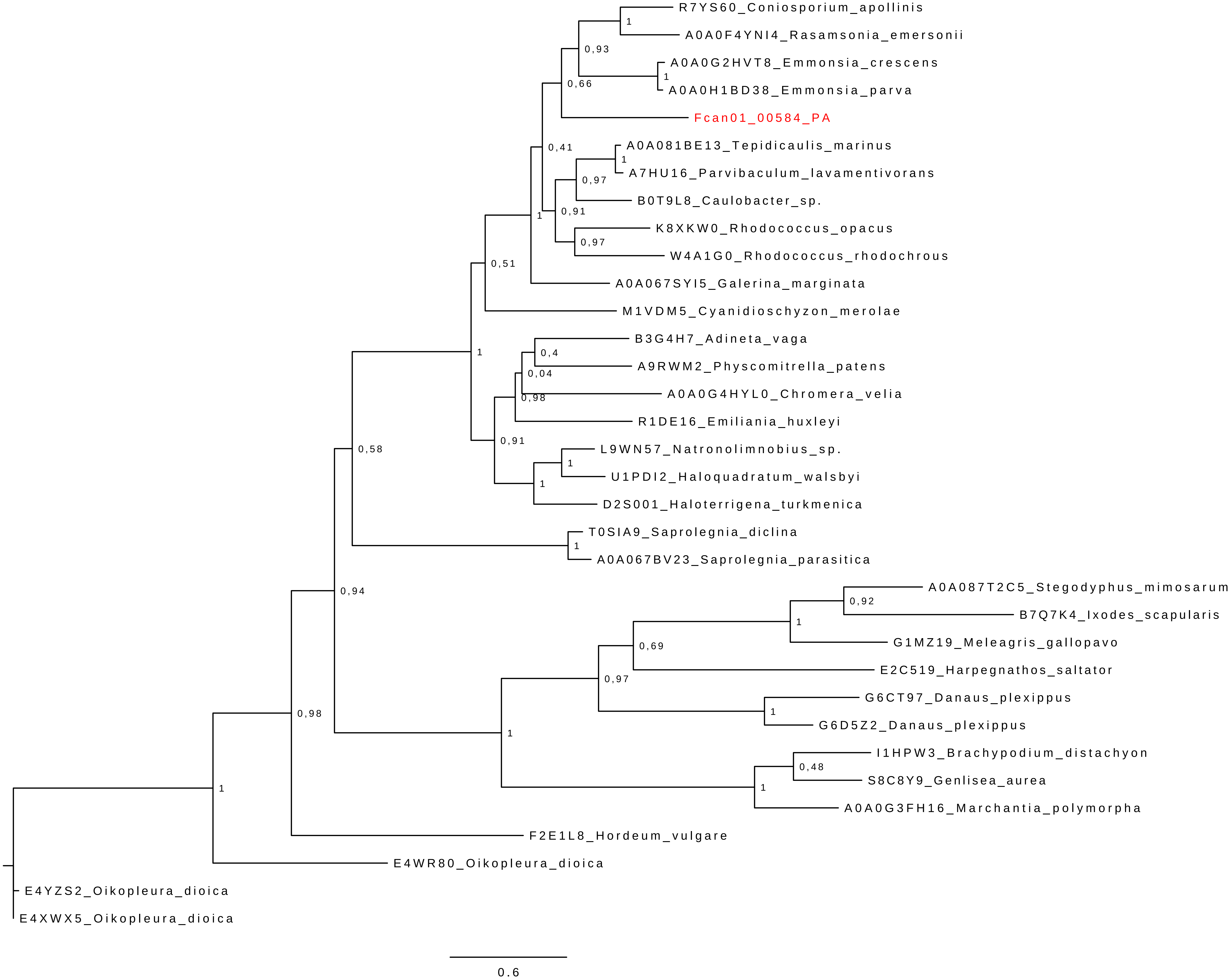
R7YS60_Coniosporium_apollinis
A0A0F4YNI4_Rasamsonia_emersonii
A0A0G2HVT8_Emmonsia_crescens
A0A0H1BD38_Emmonsia_parva
Fcan01_00584_PA
A0A081BE13_Tepidicaulis_marinus
A7HU16_Parvibaculum_lavamentivorans
B0T9L8_Caulobacter_sp.
K8XKW0_Rhodococcus_opacus
W4A1G0_Rhodococcus_rhodochrous
A0A067SYI5_Galerina_marginata
M1VDM5_Cyanidioschyzon_merolae
B3G4H7_Adineta_vaga
A9RWM2_Physcomitrella_patens
A0A0G4HYL0_Chromera_velia
R1DE16_Emiliania_huxleyi
L9WN57_Natronolimnobius_sp.
U1PDI2_Haloquadratum_walsbyi
D2S001_Haloterrigena_turkmenica
T0SIA9_Saprolegnia_diclina
A0A067BV23_Saprolegnia_parasitica
A0A087T2C5_Stegodyphus_mimosarum
B7Q7K4_Ixodes_scapularis
G1MZ19_Meleagris_gallopavo
E2C519_Harpegnathos_saltator
G6CT97_Danaus_plexippus
G6D5Z2_Danaus_plexippus
I1HPW3_Brachypodium_distachyon
S8C8Y9_Genlisea_aurea
A0A0G3FH16_Marchantia_polymorpha
F2E1L8_Hordeum_vulgare
E4WR80_Oikopleura_dioica
E4YZS2_Oikopleura_dioica
E4XWX5_Oikopleura_dioica
1
0,93
1
0,66
0,41
1
0,97
0,91
0,97
1
0,51
1
0,4
0,04
0,98
0,91
1
1
0,58
1
0,94
0,92
1
0,69
0,97
1
1
0,48
1
0,98
1
0.6

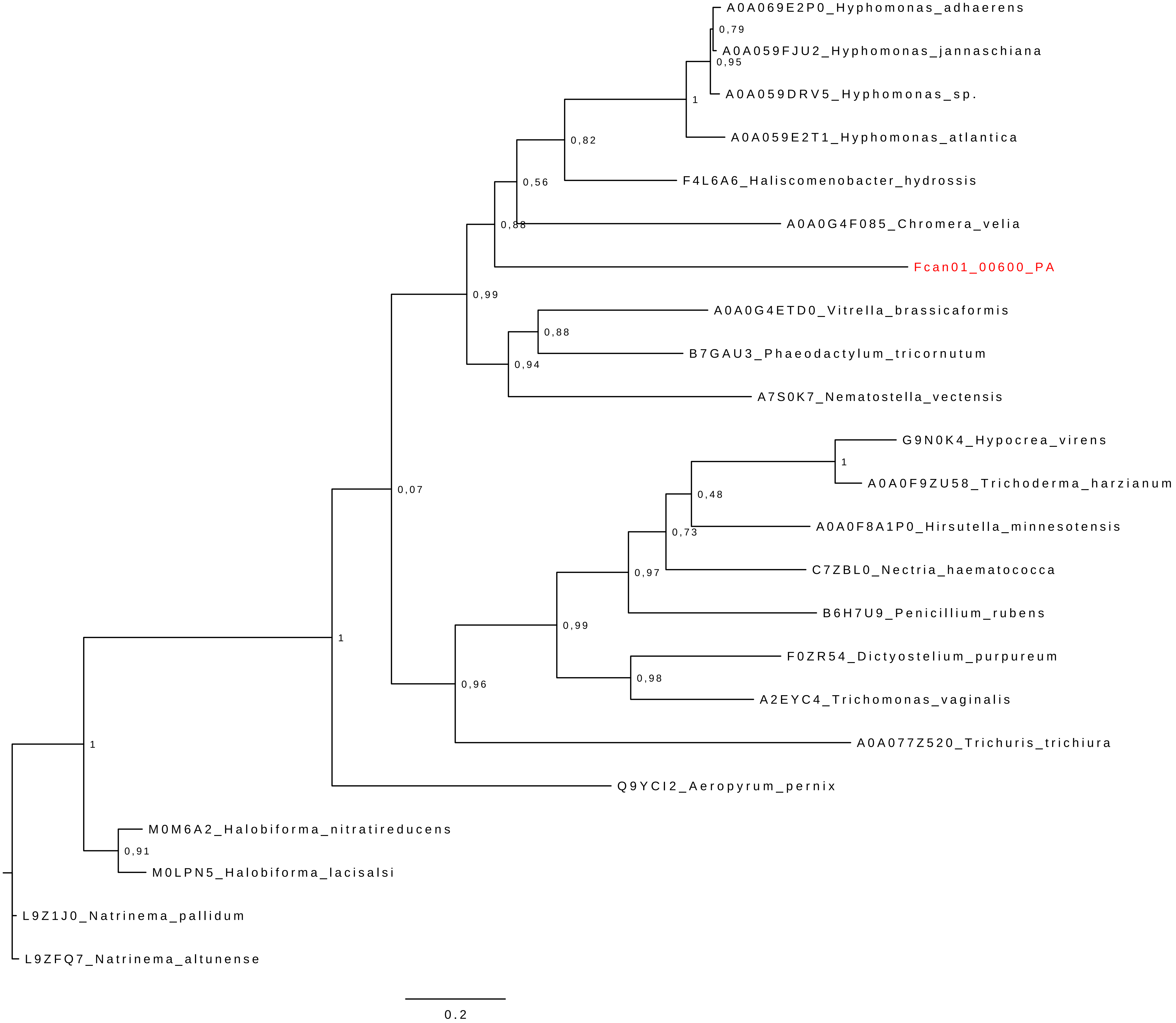
A0A069E2P0_Hyphomonas_adhaerens
0,79
A0A059FJU2_Hyphomonas_jannaschiana
0,95
A0A059DRV5_Hyphomonas_sp.
1
A0A059E2T1_Hyphomonas_atlantica
0,82
F4L6A6_Haliscomenobacter_hydrossis
0,56
A0A0G4F085_Chromera_velia
0,88
Fcan01_00600_PA
0,99
A0A0G4ETD0_Vitrella_brassicaformis
0,88
B7GAU3_Phaeodactylum_tricornutum
0,94
A7S0K7_Nematostella_vectensis
G9N0K4_Hypocrea_virens
1
A0A0F9ZU58_Trichoderma_harzianum
0,48
A0A0F8A1P0_Hirsutella_minnesotensis
0,73
C7ZBL0_Nectria_haematococca
0,97
B6H7U9_Penicillium_rubens
0,99
F0ZR54_Dictyostelium_purpureum
0,98
A2EYC4_Trichomonas_vaginalis
0,07
0,96
A0A077Z520_Trichuris_trichiura
1
Q9YCI2_Aeropyrum_pernix
1
M0M6A2_Halobiforma_nitratireducens
0,91
M0LPN5_Halobiforma_lacisalsi
L9Z1J0_Natrinema_pallidum
L9ZFQ7_Natrinema_altunense
0.2

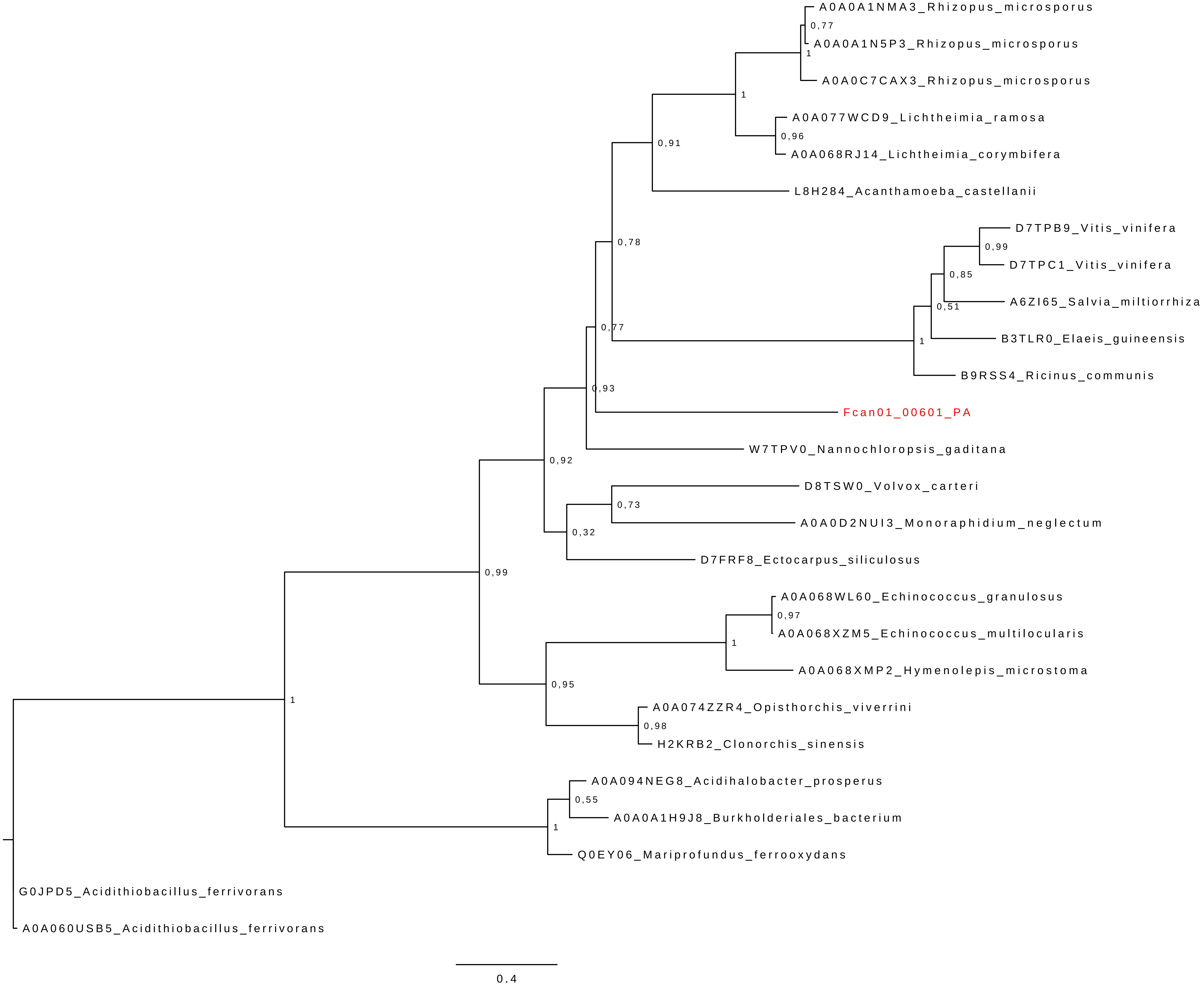

A0A0A1NMA3_Rhizopus_microsporus
0,77
A0A0A1N5P3_Rhizopus_microsporus
1
A0A0C7CAX3_Rhizopus_microsporus
1
A0A077WCD9_Lichtheimia_ramosa
0,96
A0A068RJ14_Lichtheimia_corymbifera
0,91
L8H284_Acanthamoeba_castellanii
D7TPB9_Vitis_vinifera
0,99
D7TPC1_Vitis_vinifera
0,85
A6ZI65_Salvia_miltiorrhiza
0,51
0,78
B3TLR0_Elaeis_guineensis
1
0,77
B9RSS4_Ricinus_communis
0,93
Fcan01_00601_PA
W7TPV0_Nannochloropsis_gaditana
0,92
D8TSW0_Volvox_carteri
0,73
A0A0D2NUI3_Monoraphidium_neglectum
0,32
D7FRF8_Ectocarpus_siliculosus
0,99
A0A068WL60_Echinococcus_granulosus
0,97
A0A068XZM5_Echinococcus_multilocularis
1
A0A068XMP2_Hymenolepis_microstoma
0,95
A0A074ZZR4_Opisthorchis_viverrini
0,98
H2KRB2_Clonorchis_sinensis
1
A0A094NEG8_Acidihalobacter_prosperus
0,55
A0A0A1H9J8_Burkholderiales_bacterium
1
Q0EY06_Mariprofundus_ferrooxydans
G0JPD5_Acidithiobacillus_ferrivorans
A0A060USB5_Acidithiobacillus_ferrivorans
0.4

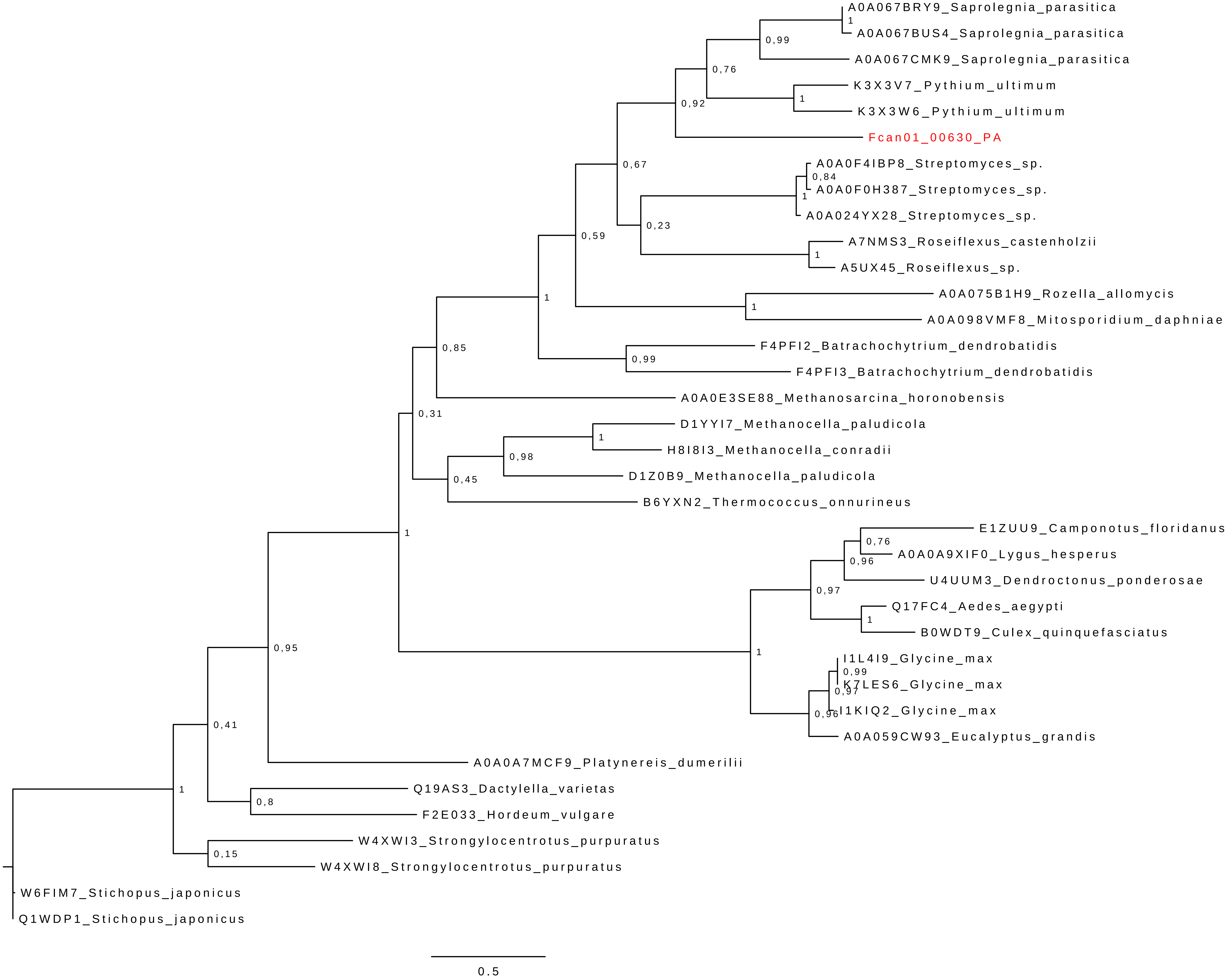
A0A067BRY9_Saprolegnia_parasitica
1
A0A067BUS4_Saprolegnia_parasitica
0,99
A0A067CMK9_Saprolegnia_parasitica
0,76
K3X3V7_Pythium_ultimum
1
K3X3W6_Pythium_ultimum
0,92
Fcan01_00630_PA
A0A0F4IBP8_Streptomyces_sp.
0,84
A0A0F0H387_Streptomyces_sp.
1
0,67
A0A024YX28_Streptomyces_sp.
0,23
A7NMS3_Roseiflexus_castenholzii
1
A5UX45_Roseiflexus_sp.
0,59
A0A075B1H9_Rozella_allomycis
1
1
A0A098VMF8_Mitosporidium_daphniae
F4PFI2_Batrachochytrium_dendrobatidis
0,99
F4PFI3_Batrachochytrium_dendrobatidis
0,85
A0A0E3SE88_Methanosarcina_horonobensis
0,31
D1YYI7_Methanocella_paludicola
1
H8I8I3_Methanocella_conradii
0,98
D1Z0B9_Methanocella_paludicola
0,45
B6YXN2_Thermococcus_onnurineus
1
E1ZUU9_Camponotus_floridanus
0,76
A0A0A9XIF0_Lygus_hesperus
0,96
U4UUM3_Dendroctonus_ponderosae
0,97
Q17FC4_Aedes_aegypti
1
B0WDT9_Culex_quinquefasciatus
0,95
1
I1L4I9_Glycine_max
0,99
K7LES6_Glycine_max
0,97
0,96
I1KIQ2_Glycine_max
A0A059CW93_Eucalyptus_grandis
0,41
A0A0A7MCF9_Platynereis_dumerilii
1
Q19AS3_Dactylella_varietas
0,8
F2E033_Hordeum_vulgare
W4XWI3_Strongylocentrotus_purpuratus
0,15
W4XWI8_Strongylocentrotus_purpuratus
W6FIM7_Stichopus_japonicus
Q1WDP1_Stichopus_japonicus
0.5

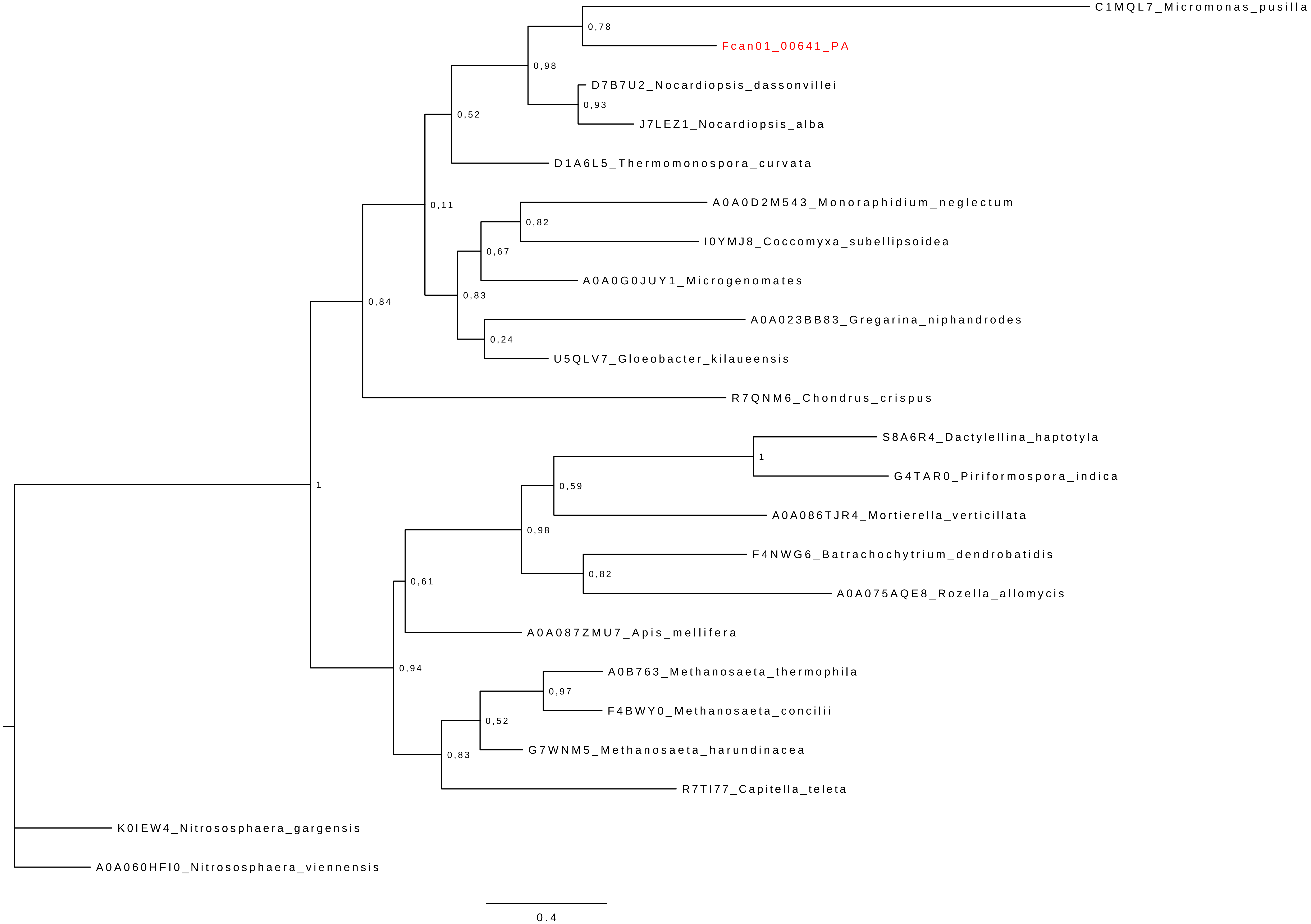
C1MQL7_Micromonas_pusilla
0,78
Fcan01_00641_PA
0,98
D7B7U2_Nocardiopsis_dassonvillei
0,93
J7LEZ1_Nocardiopsis_alba
0,52
D1A6L5_Thermomonospora_curvata
0,11
A0A0D2M543_Monoraphidium_neglectum
0,82
I0YMJ8_Coccomyxa_subellipsoidea
0,67
A0A0G0JUY1_Microgenomates
0,83
0,84
A0A023BB83_Gregarina_niphandrodes
0,24
U5QLV7_Gloeobacter_kilaueensis
R7QNM6_Chondrus_crispus
S8A6R4_Dactylellina_haptotyla
1
G4TAR0_Piriformospora_indica
0,59
1
A0A086TJR4_Mortierella_verticillata
0,98
F4NWG6_Batrachochytrium_dendrobatidis
0,82
0,61
A0A075AQE8_Rozella_allomycis
A0A087ZMU7_Apis_mellifera
0,94
A0B763_Methanosaeta_thermophila
0,97
F4BWY0_Methanosaeta_concilii
0,52
G7WNM5_Methanosaeta_harundinacea
0,83
R7TI77_Capitella_teleta
K0IEW4_Nitrososphaera_gargensis
A0A060HFI0_Nitrososphaera_viennensis
0.4

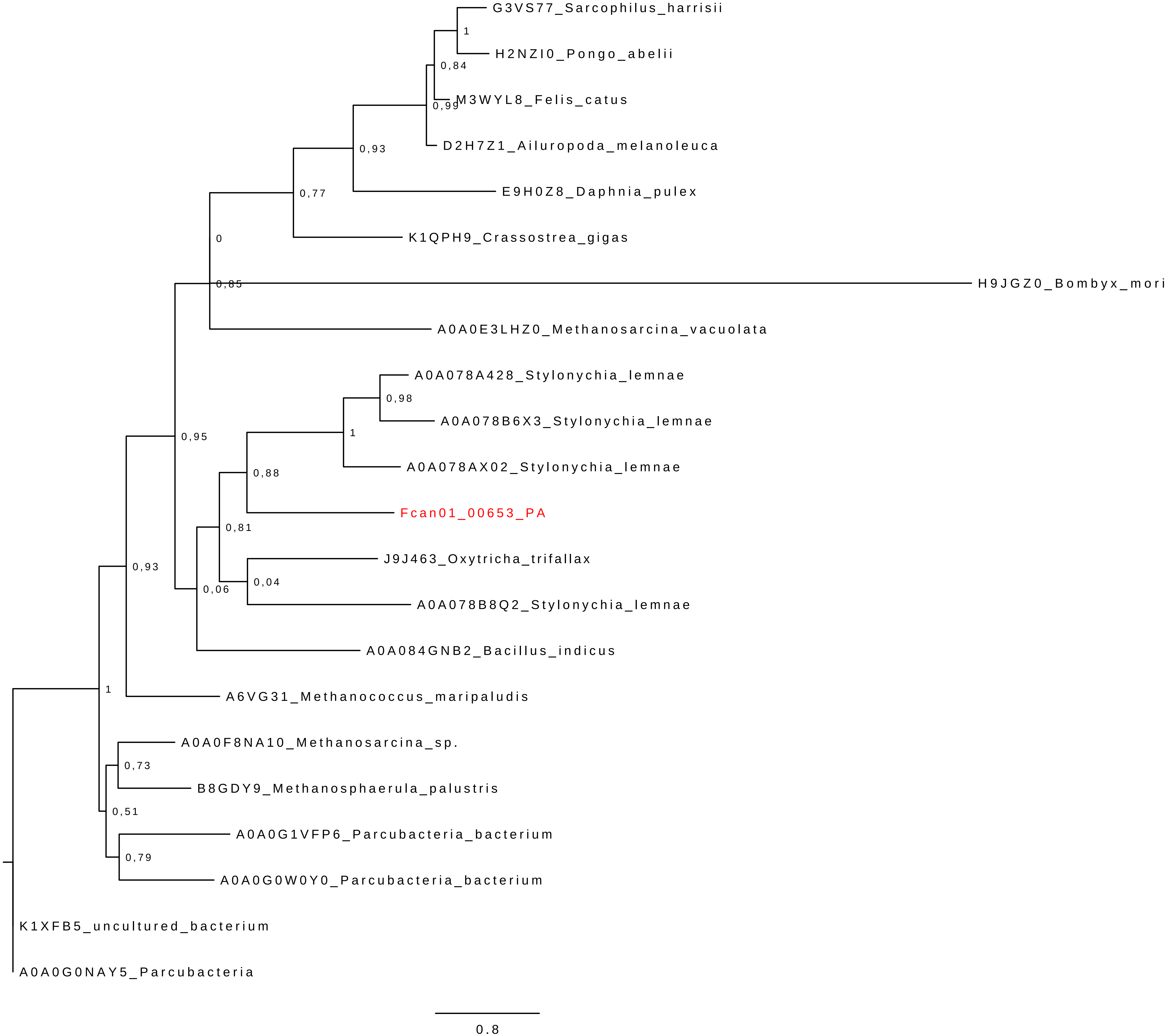
G3VS77_Sarcophilus_harrisii
1
H2NZI0_Pongo_abelii
0,84
M3WYL8_Felis_catus
0,99
D2H7Z1_Ailuropoda_melanoleuca
0,93
E9H0Z8_Daphnia_pulex
0,77
K1QPH9_Crassostrea_gigas
0
H9JGZ0_Bombyx_mori
0,85
A0A0E3LHZ0_Methanosarcina_vacuolata
A0A078A428_Stylonychia_lemnae
0,98
A0A078B6X3_Stylonychia_lemnae
1
0,95
A0A078AX02_Stylonychia_lemnae
0,88
Fcan01_00653_PA
0,81
J9J463_Oxytricha_trifallax
0,93
0,04
0,06
A0A078B8Q2_Stylonychia_lemnae
A0A084GNB2_Bacillus_indicus
1
A6VG31_Methanococcus_maripaludis
A0A0F8NA10_Methanosarcina_sp.
0,73
B8GDY9_Methanosphaerula_palustris
0,51
A0A0G1VFP6_Parcubacteria_bacterium
0,79
A0A0G0W0Y0_Parcubacteria_bacterium
K1XFB5_uncultured_bacterium
A0A0G0NAY5_Parcubacteria
0.8

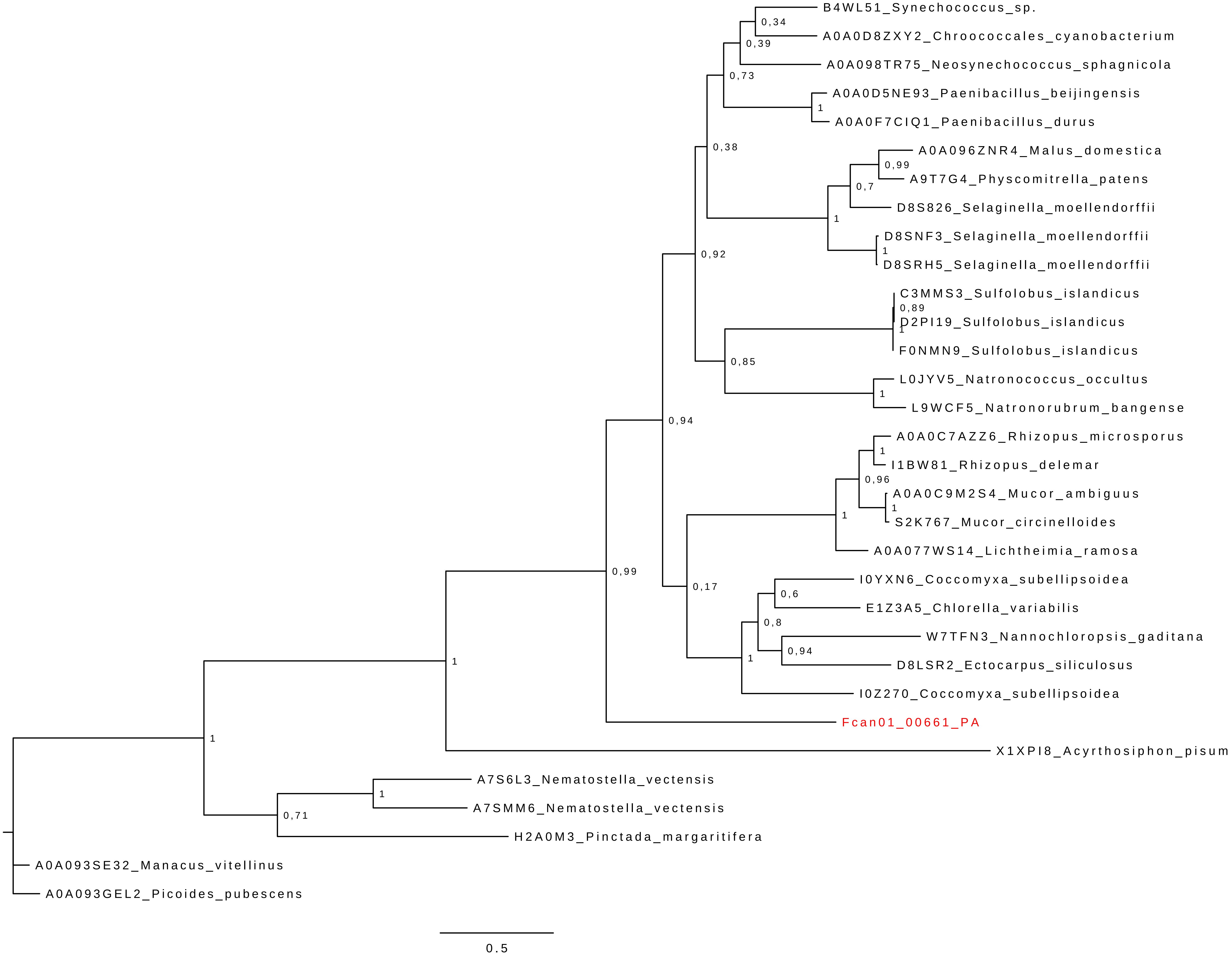
B4WL51_Synechococcus_sp.
0,34
A0A0D8ZXY2_Chroococcales_cyanobacterium
0,39
A0A098TR75_Neosynechococcus_sphagnicola
0,73
A0A0D5NE93_Paenibacillus_beijingensis
1
A0A0F7CIQ1_Paenibacillus_durus
0,38
A0A096ZNR4_Malus_domestica
0,99
A9T7G4_Physcomitrella_patens
0,7
D8S826_Selaginella_moellendorffii
1
D8SNF3_Selaginella_moellendorffii
1
D8SRH5_Selaginella_moellendorffii
0,92
C3MMS3_Sulfolobus_islandicus
0,89
D2PI19_Sulfolobus_islandicus
1
F0NMN9_Sulfolobus_islandicus
0,85
L0JYV5_Natronococcus_occultus
1
L9WCF5_Natronorubrum_bangense
0,94
A0A0C7AZZ6_Rhizopus_microsporus
1
I1BW81_Rhizopus_delemar
0,96
A0A0C9M2S4_Mucor_ambiguus
1
S2K767_Mucor_circinelloides
1
A0A077WS14_Lichtheimia_ramosa
0,99
I0YXN6_Coccomyxa_subellipsoidea
0,17
0,6
E1Z3A5_Chlorella_variabilis
0,8
W7TFN3_Nannochloropsis_gaditana
0,94
D8LSR2_Ectocarpus_siliculosus
1
1
I0Z270_Coccomyxa_subellipsoidea
Fcan01_00661_PA
1
X1XPI8_Acyrthosiphon_pisum
A7S6L3_Nematostella_vectensis
1
A7SMM6_Nematostella_vectensis
0,71
H2A0M3_Pinctada_margaritifera
A0A093SE32_Manacus_vitellinus
A0A093GEL2_Picoides_pubescens
0.5

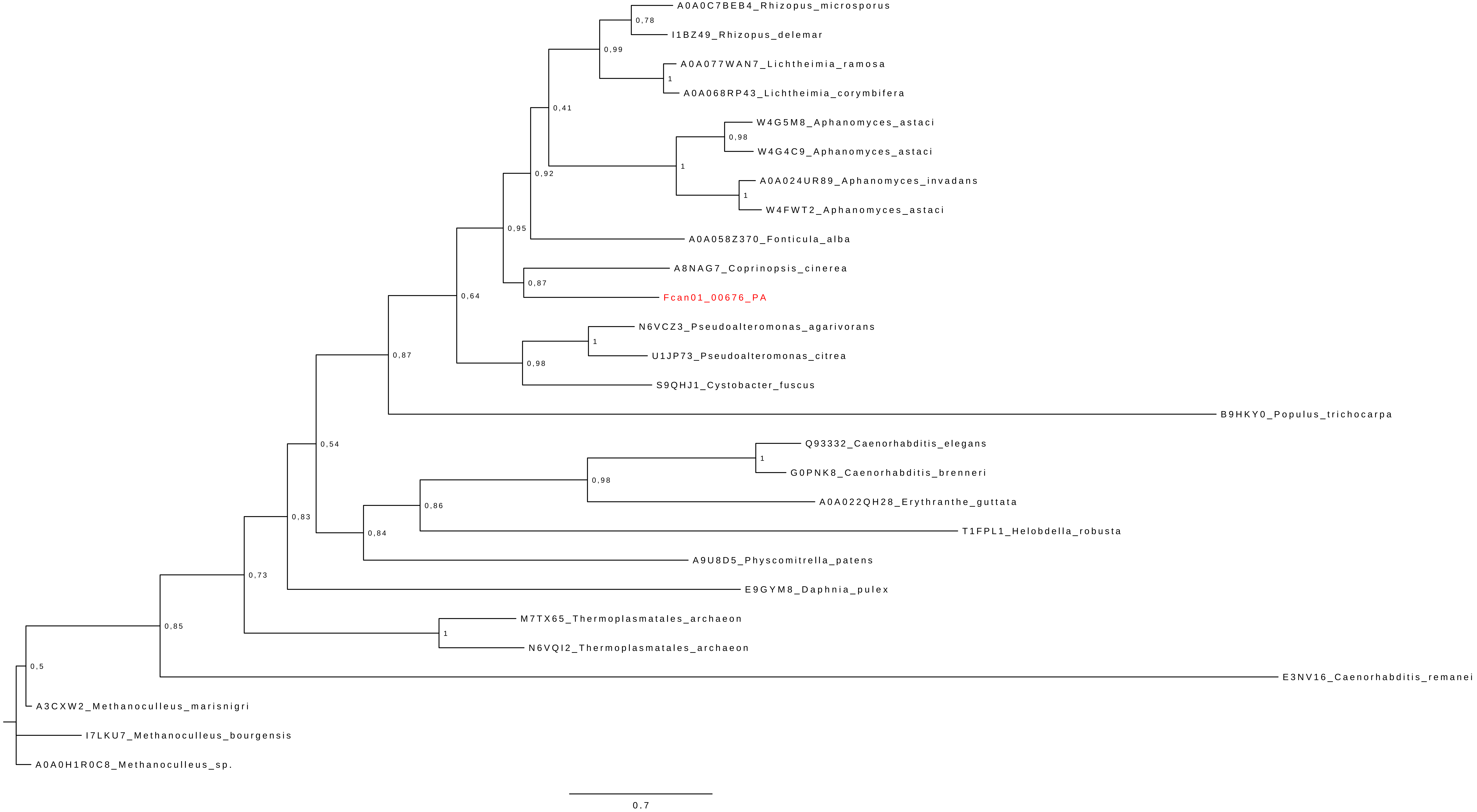

A0A0C7BEB4_Rhizopus_microsporus
0,78
I1BZ49_Rhizopus_delemar
0,99
A0A077WAN7_Lichtheimia_ramosa
1
A0A068RP43_Lichtheimia_corymbifera
0,41
W4G5M8_Aphanomyces_astaci
0,98
W4G4C9_Aphanomyces_astaci
1
A0A024UR89_Aphanomyces_invadans
1
W4FWT2_Aphanomyces_astaci
0,92
A0A058Z370_Fonticula_alba
0,95
A8NAG7_Coprinopsis_cinerea
0,87
Fcan01_00676_PA
0,64
N6VCZ3_Pseudoalteromonas_agarivorans
1
U1JP73_Pseudoalteromonas_citrea
0,98
S9QHJ1_Cystobacter_fuscus
0,87
B9HKY0_Populus_trichocarpa
0,54
Q93332_Caenorhabditis_elegans
1
G0PNK8_Caenorhabditis_brenneri
0,98
A0A022QH28_Erythranthe_guttata
0,86
T1FPL1_Helobdella_robusta
0,84
A9U8D5_Physcomitrella_patens
0,83
E9GYM8_Daphnia_pulex
0,73
M7TX65_Thermoplasmatales_archaeon
1
N6VQI2_Thermoplasmatales_archaeon
0,85
E3NV16_Caenorhabditis_remanei
0,5
A3CXW2_Methanoculleus_marisnigri
I7LKU7_Methanoculleus_bourgensis
A0A0H1R0C8_Methanoculleus_sp.
0.7

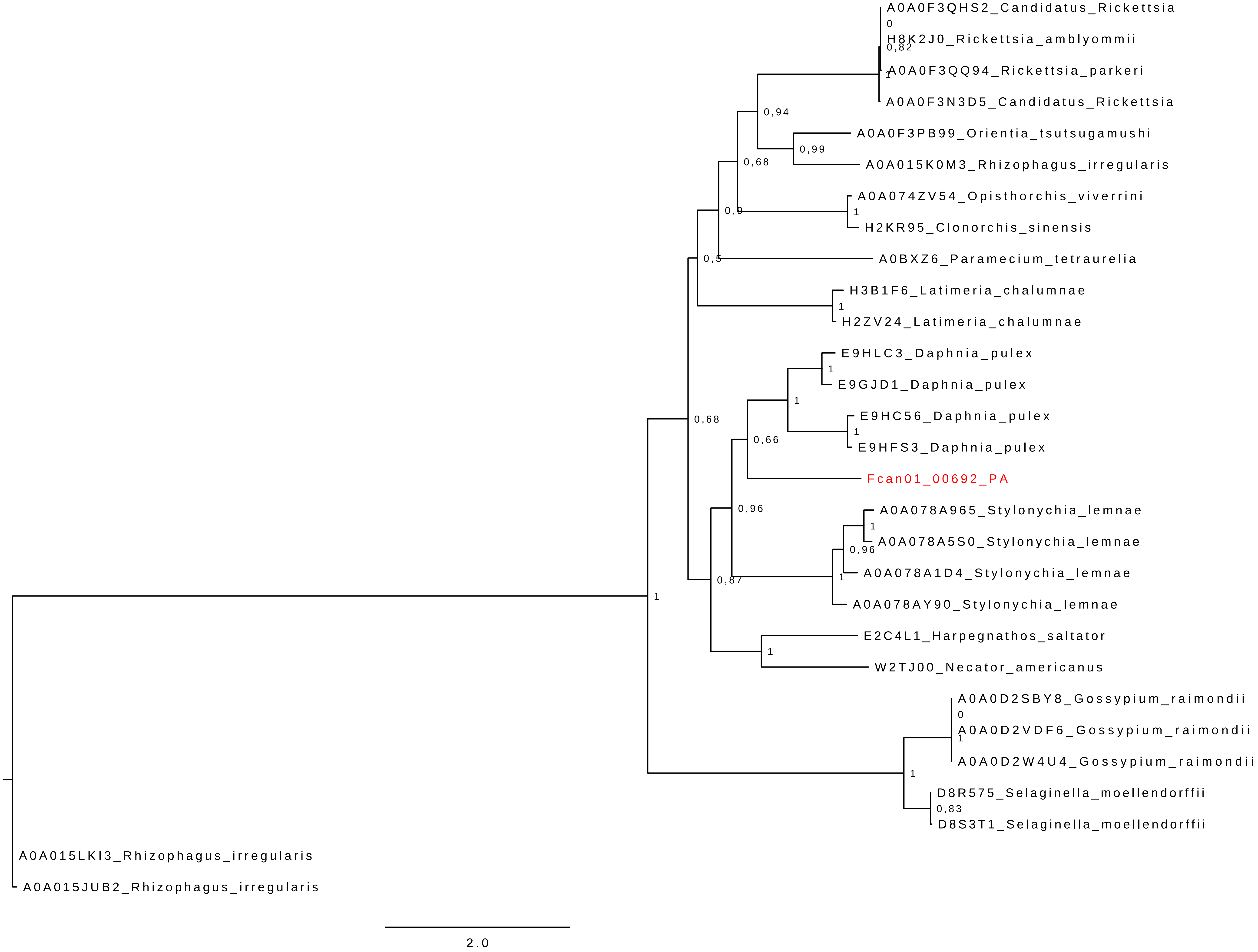
A0A0F3QHS2_Candidatus_Rickettsia
0
H8K2J0_Rickettsia_amblyommii
0,82
A0A0F3QQ94_Rickettsia_parkeri
1
A0A0F3N3D5_Candidatus_Rickettsia
0,94
A0A0F3PB99_Orientia_tsutsugamushi
0,99
A0A015K0M3_Rhizophagus_irregularis
0,68
A0A074ZV54_Opisthorchis_viverrini
0,9
1
H2KR95_Clonorchis_sinensis
0,5
A0BXZ6_Paramecium_tetraurelia
H3B1F6_Latimeria_chalumnae
1
H2ZV24_Latimeria_chalumnae
E9HLC3_Daphnia_pulex
1
E9GJD1_Daphnia_pulex
1
E9HC56_Daphnia_pulex
0,68
1
0,66
E9HFS3_Daphnia_pulex
Fcan01_00692_PA
0,96
A0A078A965_Stylonychia_lemnae
1
A0A078A5S0_Stylonychia_lemnae
0,96
A0A078A1D4_Stylonychia_lemnae
0,87
1
1
A0A078AY90_Stylonychia_lemnae
E2C4L1_Harpegnathos_saltator
1
W2TJ00_Necator_americanus
A0A0D2SBY8_Gossypium_raimondii
0
A0A0D2VDF6_Gossypium_raimondii
1
A0A0D2W4U4_Gossypium_raimondii
1
D8R575_Selaginella_moellendorffii
0,83
D8S3T1_Selaginella_moellendorffii
A0A015LKI3_Rhizophagus_irregularis
A0A015JUB2_Rhizophagus_irregularis
2.0

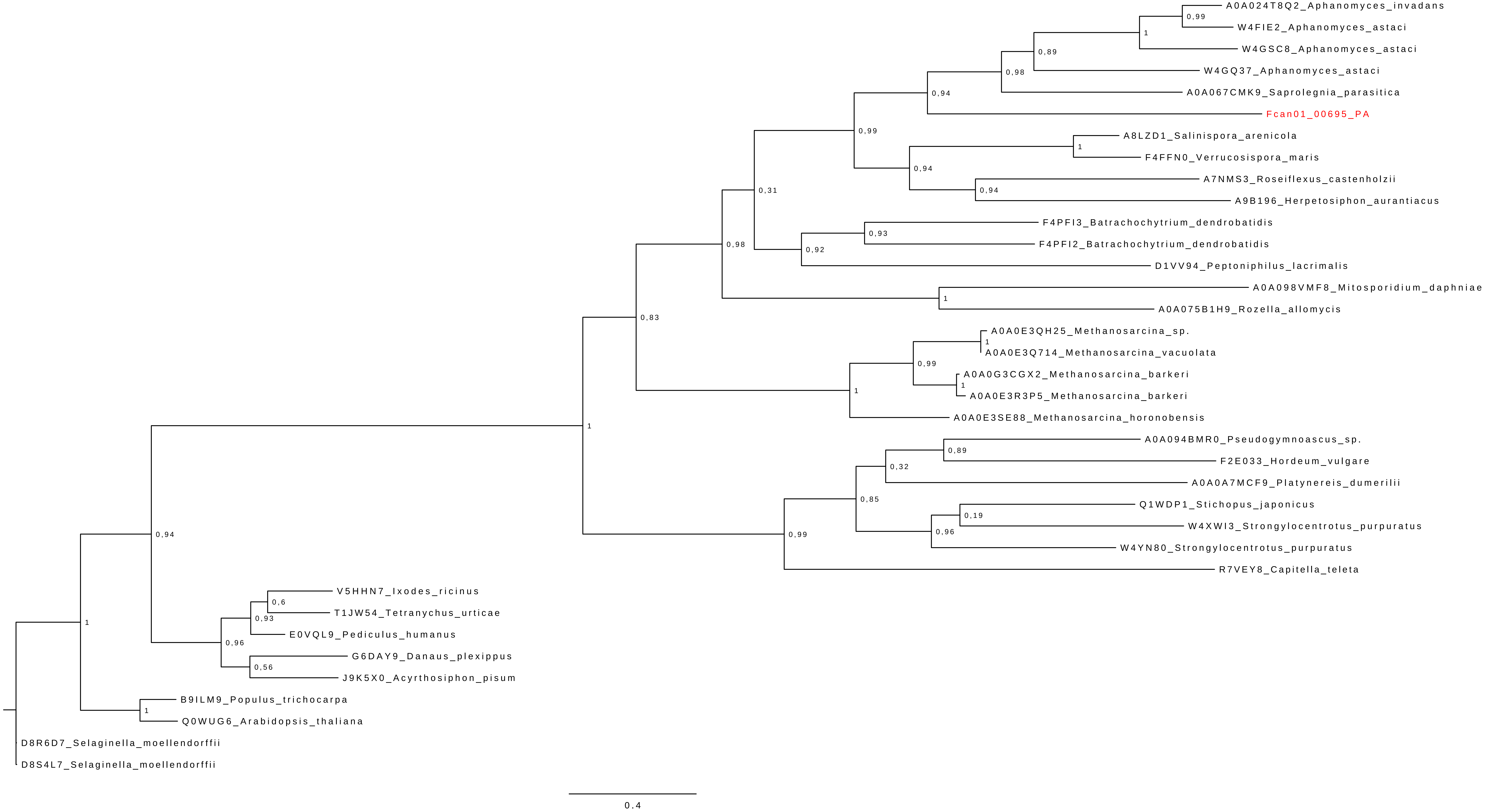
A0A024T8Q2_Aphanomyces_invadans
W4FIE2_Aphanomyces_astaci
W4GSC8_Aphanomyces_astaci
W4GQ37_Aphanomyces_astaci
A0A067CMK9_Saprolegnia_parasitica
Fcan01_00695_PA
A8LZD1_Salinispora_arenicola
F4FFN0_Verrucosispora_maris
A7NMS3_Roseiflexus_castenholzii
A9B196_Herpetosiphon_aurantiacus
F4PFI3_Batrachochytrium_dendrobatidis
F4PFI2_Batrachochytrium_dendrobatidis
D1VV94_Peptoniphilus_lacrimalis
A0A098VMF8_Mitosporidium_daphniae
A0A075B1H9_Rozella_allomycis
A0A0E3QH25_Methanosarcina_sp.
A0A0E3Q714_Methanosarcina_vacuolata
A0A0G3CGX2_Methanosarcina_barkeri
A0A0E3R3P5_Methanosarcina_barkeri
A0A0E3SE88_Methanosarcina_horonobensis
A0A094BMR0_Pseudogymnoascus_sp.
F2E033_Hordeum_vulgare
A0A0A7MCF9_Platynereis_dumerilii
Q1WDP1_Stichopus_japonicus
W4XWI3_Strongylocentrotus_purpuratus
W4YN80_Strongylocentrotus_purpuratus
R7VEY8_Capitella_teleta
V5HHN7_Ixodes_ricinus
T1JW54_Tetranychus_urticae
E0VQL9_Pediculus_humanus
G6DAY9_Danaus_plexippus
J9K5X0_Acyrthosiphon_pisum
B9ILM9_Populus_trichocarpa
Q0WUG6_Arabidopsis_thaliana
D8R6D7_Selaginella_moellendorffii
D8S4L7_Selaginella_moellendorffii
0,99
1
0,89
0,98
0,94
0,99
1
0,94
0,94
0,31
0,93
0,92
0,98
1
0,83
1
0,99
1
1
1
0,89
0,32
0,85
0,19
0,96
0,99
0,94
0,6
0,93
0,96
0,56
1
1
0.4

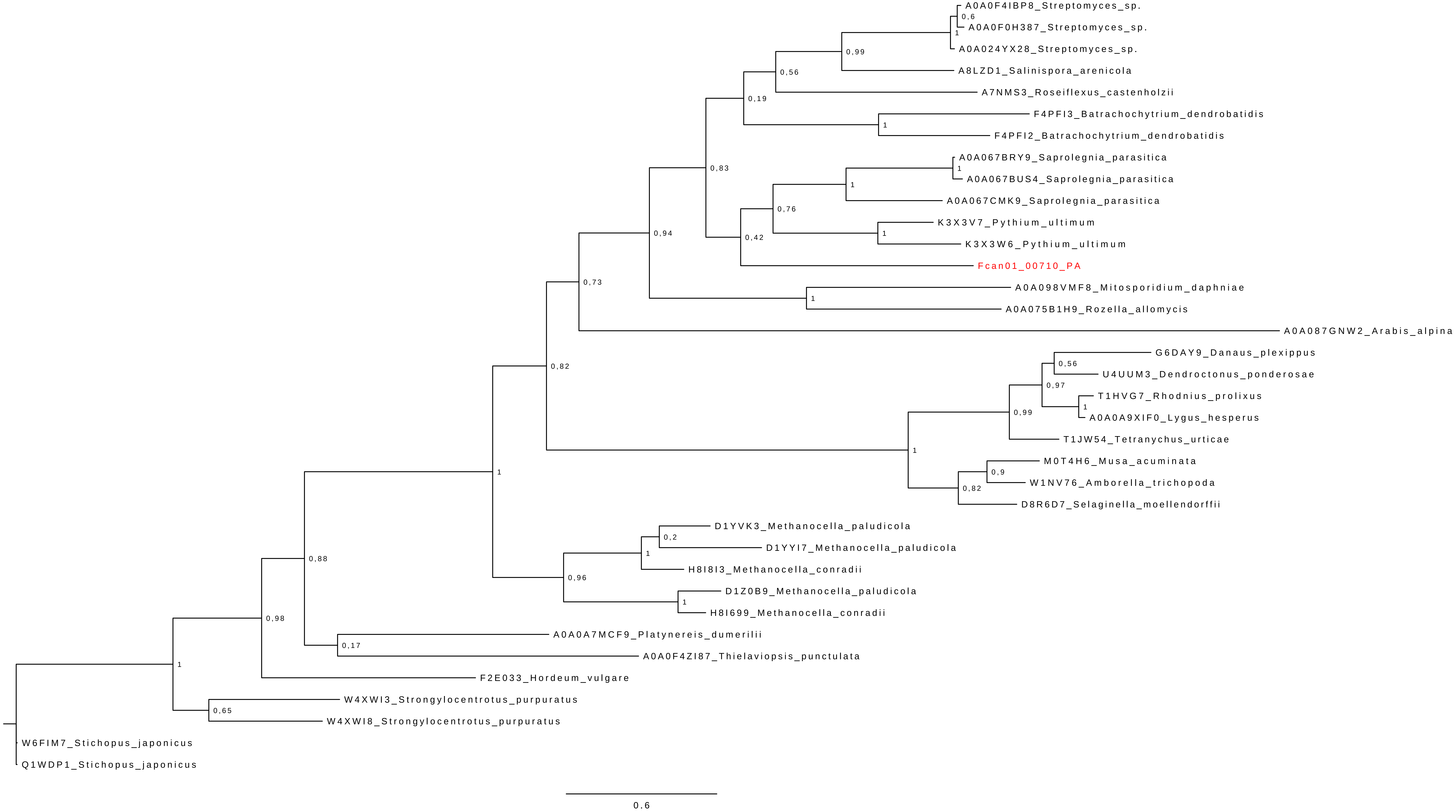

A0A0F4IBP8_Streptomyces_sp.
0,6
A0A0F0H387_Streptomyces_sp.
1
A0A024YX28_Streptomyces_sp.
0,99
A8LZD1_Salinispora_arenicola
0,56
A7NMS3_Roseiflexus_castenholzii
0,19
F4PFI3_Batrachochytrium_dendrobatidis
1
F4PFI2_Batrachochytrium_dendrobatidis
A0A067BRY9_Saprolegnia_parasitica
1
A0A067BUS4_Saprolegnia_parasitica
1
A0A067CMK9_Saprolegnia_parasitica
0,83
0,76
K3X3V7_Pythium_ultimum
1
K3X3W6_Pythium_ultimum
0,42
Fcan01_00710_PA
0,94
A0A098VMF8_Mitosporidium_daphniae
1
A0A075B1H9_Rozella_allomycis
0,73
A0A087GNW2_Arabis_alpina
G6DAY9_Danaus_plexippus
0,56
U4UUM3_Dendroctonus_ponderosae
0,97
T1HVG7_Rhodnius_prolixus
1
A0A0A9XIF0_Lygus_hesperus
0,82
0,99
T1JW54_Tetranychus_urticae
1
M0T4H6_Musa_acuminata
0,9
W1NV76_Amborella_trichopoda
0,82
D8R6D7_Selaginella_moellendorffii
1
D1YVK3_Methanocella_paludicola
0,2
D1YYI7_Methanocella_paludicola
1
H8I8I3_Methanocella_conradii
0,96
D1Z0B9_Methanocella_paludicola
1
H8I699_Methanocella_conradii
0,88
A0A0A7MCF9_Platynereis_dumerilii
0,17
A0A0F4ZI87_Thielaviopsis_punctulata
0,98
F2E033_Hordeum_vulgare
1
W4XWI3_Strongylocentrotus_purpuratus
0,65
W4XWI8_Strongylocentrotus_purpuratus
W6FIM7_Stichopus_japonicus
Q1WDP1_Stichopus_japonicus
0.6

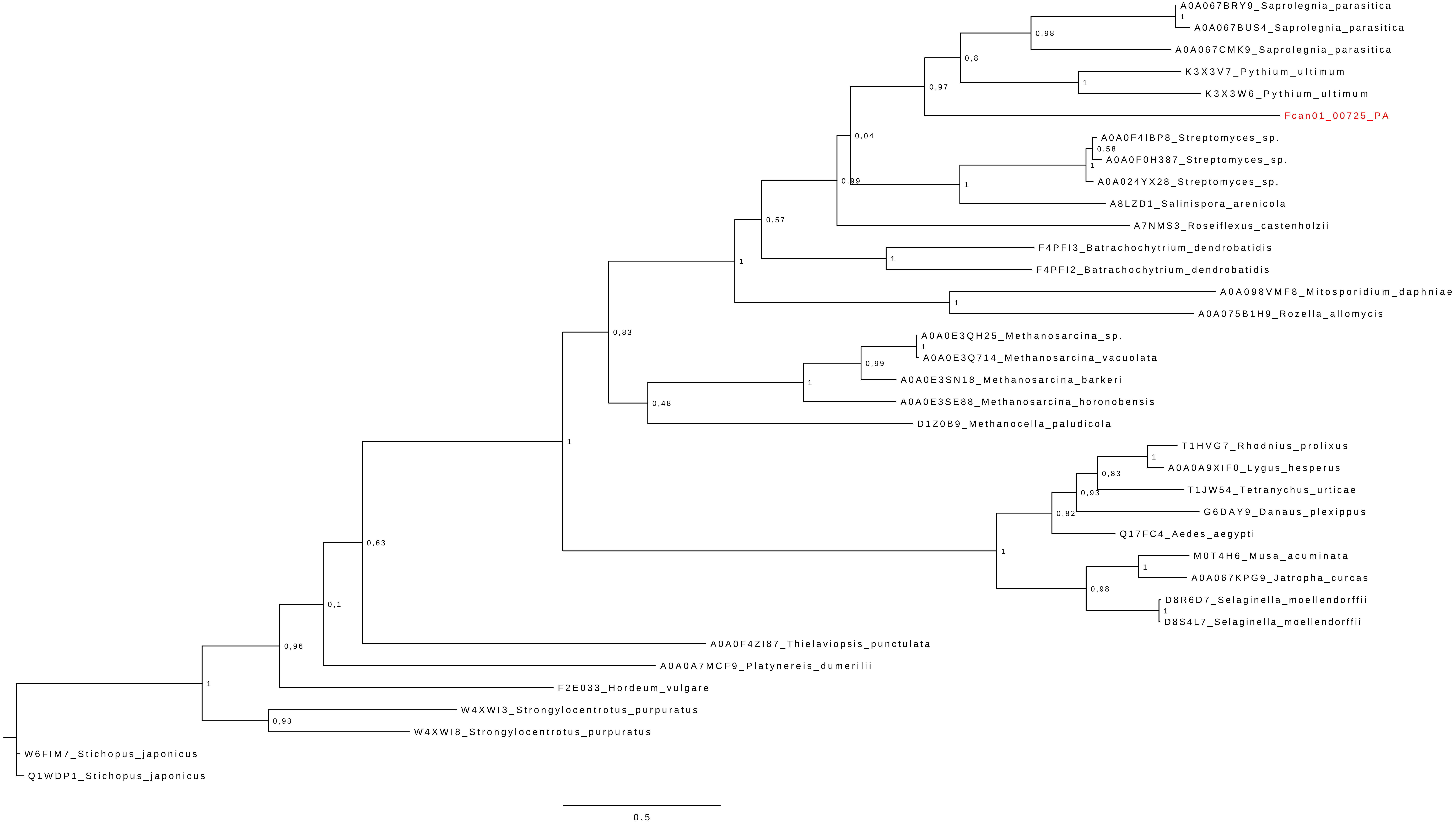
A0A067BRY9_Saprolegnia_parasitica
A0A067BUS4_Saprolegnia_parasitica
A0A067CMK9_Saprolegnia_parasitica
K3X3V7_Pythium_ultimum
K3X3W6_Pythium_ultimum
Fcan01_00725_PA
A0A0F4IBP8_Streptomyces_sp.
A0A0F0H387_Streptomyces_sp.
A0A024YX28_Streptomyces_sp.
A8LZD1_Salinispora_arenicola
A7NMS3_Roseiflexus_castenholzii
F4PFI3_Batrachochytrium_dendrobatidis
F4PFI2_Batrachochytrium_dendrobatidis
A0A098VMF8_Mitosporidium_daphniae
A0A075B1H9_Rozella_allomycis
A0A0E3QH25_Methanosarcina_sp.
A0A0E3Q714_Methanosarcina_vacuolata
A0A0E3SN18_Methanosarcina_barkeri
A0A0E3SE88_Methanosarcina_horonobensis
D1Z0B9_Methanocella_paludicola
T1HVG7_Rhodnius_prolixus
A0A0A9XIF0_Lygus_hesperus
T1JW54_Tetranychus_urticae
G6DAY9_Danaus_plexippus
Q17FC4_Aedes_aegypti
M0T4H6_Musa_acuminata
A0A067KPG9_Jatropha_curcas
D8R6D7_Selaginella_moellendorffii
D8S4L7_Selaginella_moellendorffii
A0A0F4ZI87_Thielaviopsis_punctulata
A0A0A7MCF9_Platynereis_dumerilii
F2E033_Hordeum_vulgare
W4XWI3_Strongylocentrotus_purpuratus
W4XWI8_Strongylocentrotus_purpuratus
W6FIM7_Stichopus_japonicus
Q1WDP1_Stichopus_japonicus
0.5

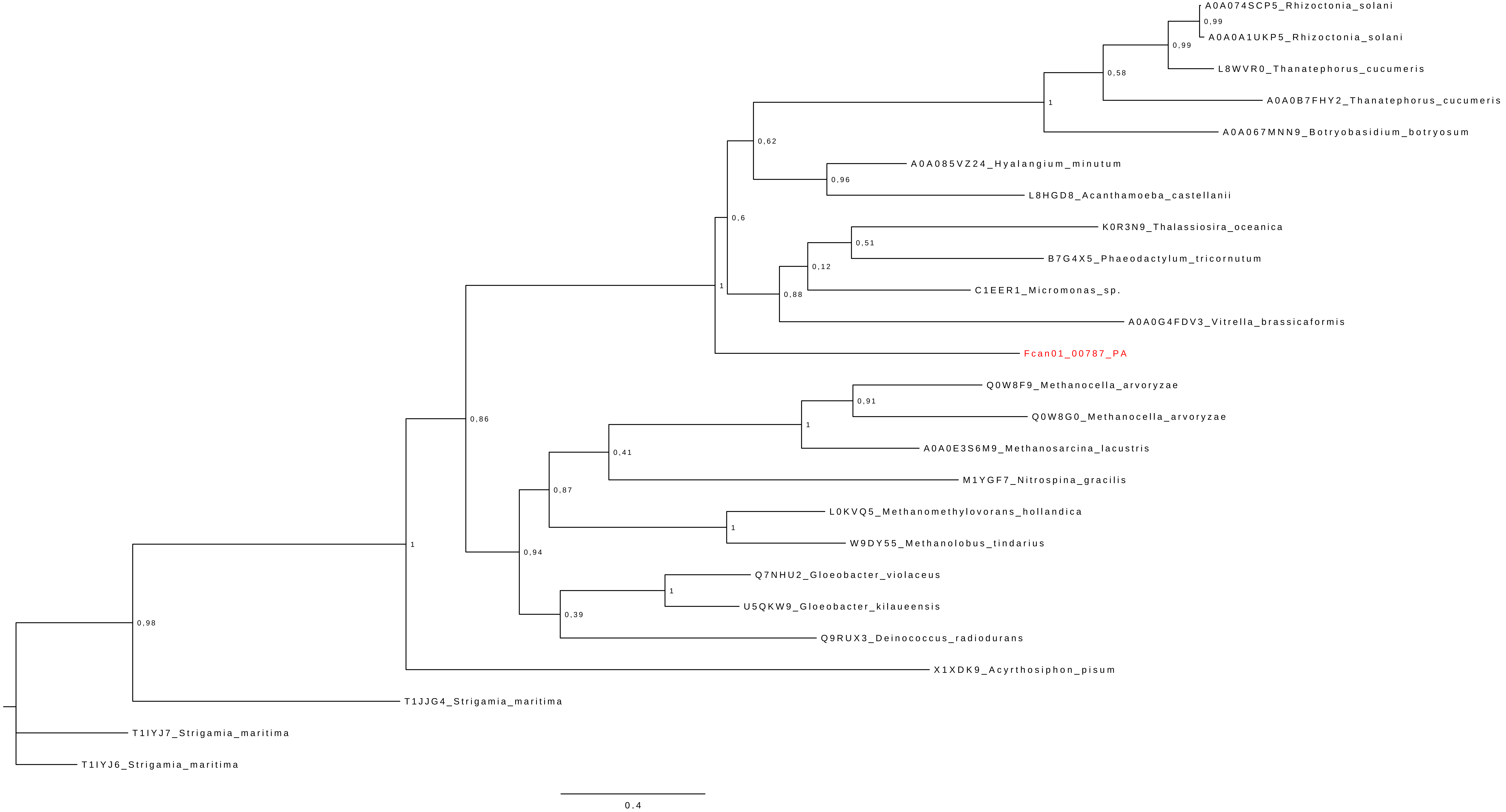
A0A074SCP5_Rhizoctonia_solani
0,99
A0A0A1UKP5_Rhizoctonia_solani
0,99
L8WVR0_Thanatephorus_cucumeris
0,58
A0A0B7FHY2_Thanatephorus_cucumeris
1
A0A067MNN9_Botryobasidium_botryosum
0,62
A0A085VZ24_Hyalangium_minutum
0,96
L8HGD8_Acanthamoeba_castellanii
0,6
K0R3N9_Thalassiosira_oceanica
0,51
B7G4X5_Phaeodactylum_tricornutum
0,12
C1EER1_Micromonas_sp.
1
0,88
A0A0G4FDV3_Vitrella_brassicaformis
Fcan01_00787_PA
Q0W8F9_Methanocella_arvoryzae
0,91
Q0W8G0_Methanocella_arvoryzae
0,86
1
A0A0E3S6M9_Methanosarcina_lacustris
0,41
M1YGF7_Nitrospina_gracilis
0,87
L0KVQ5_Methanomethylovorans_hollandica
1
1
W9DY55_Methanolobus_tindarius
0,94
Q7NHU2_Gloeobacter_violaceus
1
U5QKW9_Gloeobacter_kilaueensis
0,98
0,39
Q9RUX3_Deinococcus_radiodurans
X1XDK9_Acyrthosiphon_pisum
T1JJG4_Strigamia_maritima
T1IYJ7_Strigamia_maritima
T1IYJ6_Strigamia_maritima
0.4

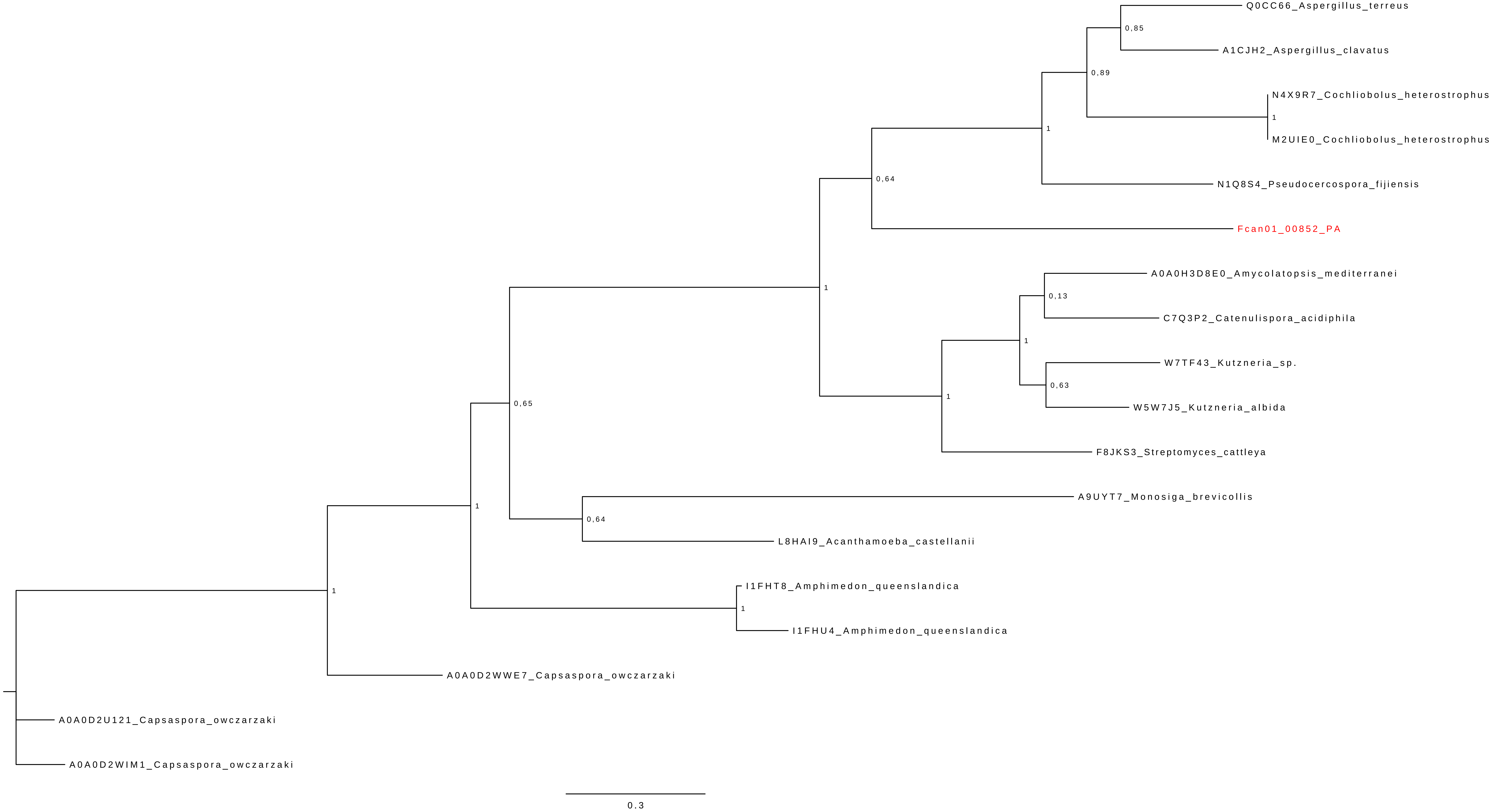
Q0CC66_Aspergillus_terreus
0,85
A1CJH2_Aspergillus_clavatus
0,89
N4X9R7_Cochliobolus_heterostrophus
1
M2UIE0_Cochliobolus_heterostrophus
1
N1Q8S4_Pseudocercospora_fijiensis
0,64
Fcan01_00852_PA
1
A0A0H3D8E0_Amycolatopsis_mediterranei
0,13
C7Q3P2_Catenulispora_acidiphila
1
W7TF43_Kutzneria_sp.
0,63
W5W7J5_Kutzneria_albida
1
F8JKS3_Streptomyces_cattleya
0,65
A9UYT7_Monosiga_brevicollis
1
0,64
L8HAI9_Acanthamoeba_castellanii
I1FHT8_Amphimedon_queenslandica
1
1
I1FHU4_Amphimedon_queenslandica
A0A0D2WWE7_Capsaspora_owczarzaki
A0A0D2U121_Capsaspora_owczarzaki
A0A0D2WIM1_Capsaspora_owczarzaki
0.3

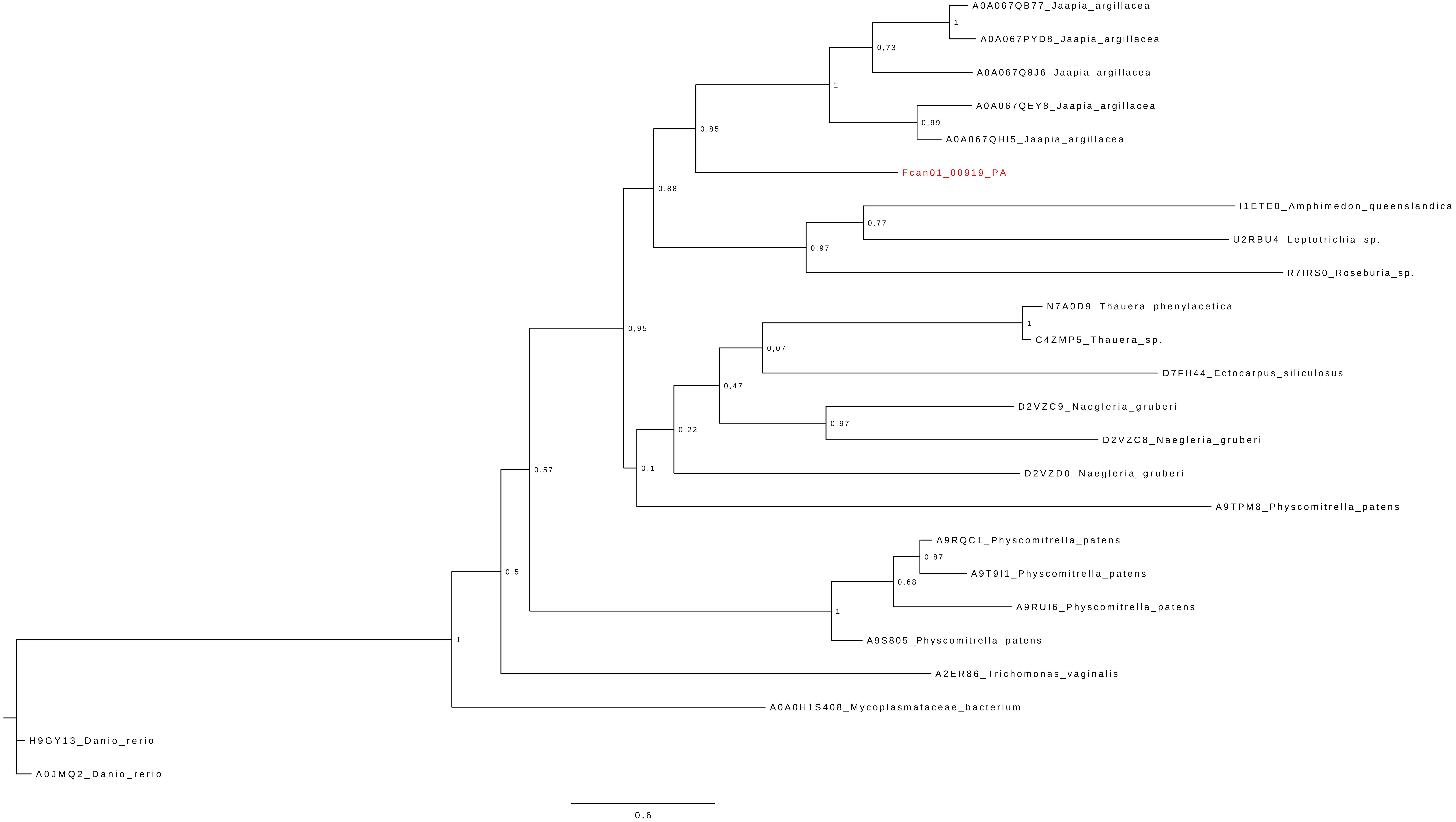
A0A067QB77_Jaapia_argillacea
A0A067PYD8_Jaapia_argillacea
A0A067Q8J6_Jaapia_argillacea
A0A067QEY8_Jaapia_argillacea
A0A067QHI5_Jaapia_argillacea
Fcan01_00919_PA
I1ETE0_Amphimedon_queenslandica
U2RBU4_Leptotrichia_sp.
R7IRS0_Roseburia_sp.
N7A0D9_Thauera_phenylacetica
C4ZMP5_Thauera_sp.
D7FH44_Ectocarpus_siliculosus
D2VZC9_Naegleria_gruberi
D2VZC8_Naegleria_gruberi
D2VZD0_Naegleria_gruberi
A9TPM8_Physcomitrella_patens
A9RQC1_Physcomitrella_patens
A9T9I1_Physcomitrella_patens
A9RUI6_Physcomitrella_patens
A9S805_Physcomitrella_patens
A2ER86_Trichomonas_vaginalis
A0A0H1S408_Mycoplasmataceae_bacterium
H9GY13_Danio_rerio
A0JMQ2_Danio_rerio
1
0,73
1
0,99
0,85
0,88
0,77
0,97
0,95
1
0,07
0,47
0,97
0,22
0,1
0,57
0,87
0,68
1
0,5
1
0.6

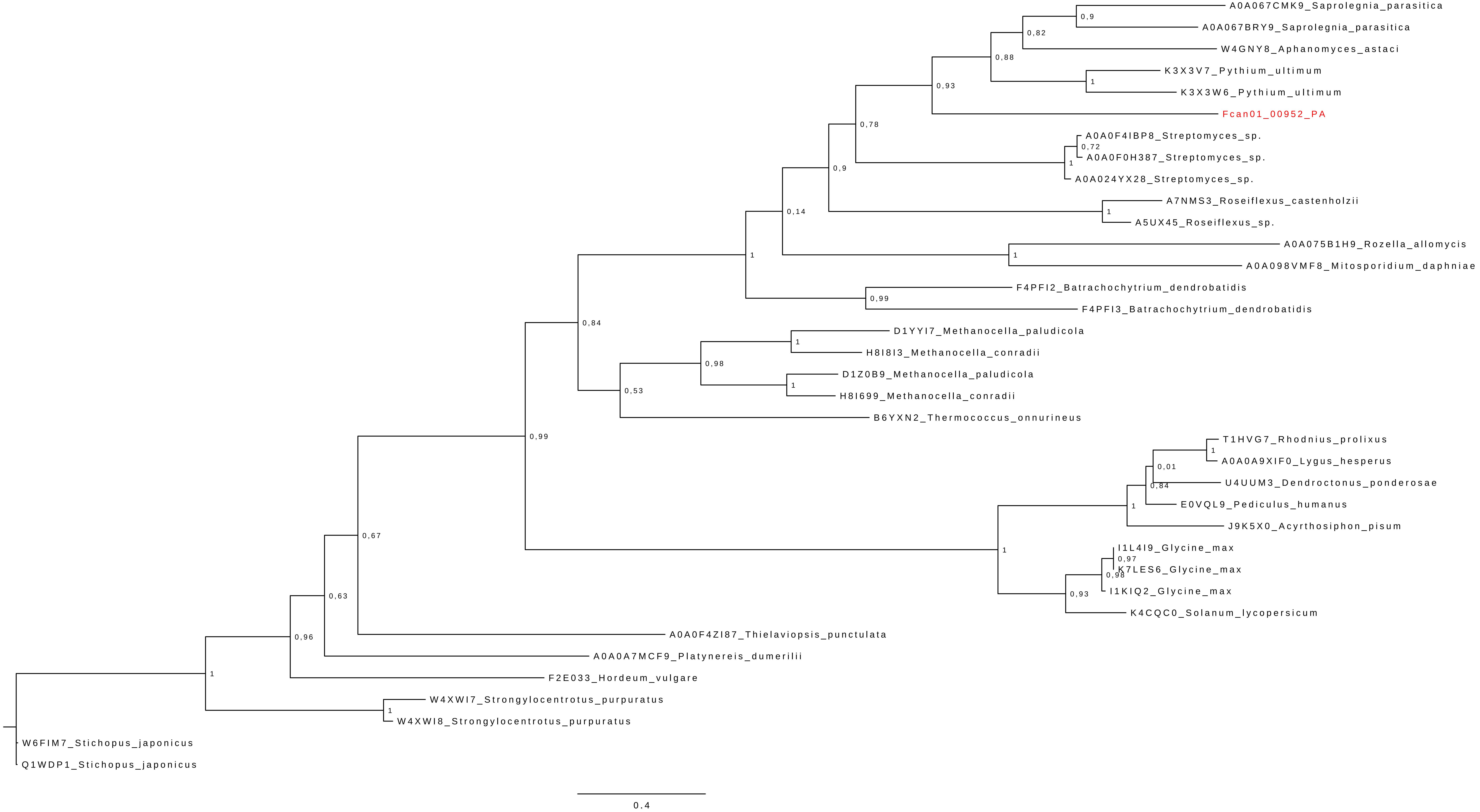
A0A067CMK9_Saprolegnia_parasitica
0,9
A0A067BRY9_Saprolegnia_parasitica
0,82
W4GNY8_Aphanomyces_astaci
0,88
K3X3V7_Pythium_ultimum
1
K3X3W6_Pythium_ultimum
0,93
Fcan01_00952_PA
A0A0F4IBP8_Streptomyces_sp.
0,72
A0A0F0H387_Streptomyces_sp.
0,78
1
A0A024YX28_Streptomyces_sp.
0,9
A7NMS3_Roseiflexus_castenholzii
1
A5UX45_Roseiflexus_sp.
0,14
A0A075B1H9_Rozella_allomycis
1
1
A0A098VMF8_Mitosporidium_daphniae
F4PFI2_Batrachochytrium_dendrobatidis
0,99
F4PFI3_Batrachochytrium_dendrobatidis
0,84
D1YYI7_Methanocella_paludicola
1
H8I8I3_Methanocella_conradii
0,98
D1Z0B9_Methanocella_paludicola
1
H8I699_Methanocella_conradii
0,53
B6YXN2_Thermococcus_onnurineus
0,99
T1HVG7_Rhodnius_prolixus
1
A0A0A9XIF0_Lygus_hesperus
0,01
U4UUM3_Dendroctonus_ponderosae
0,84
E0VQL9_Pediculus_humanus
1
J9K5X0_Acyrthosiphon_pisum
1
I1L4I9_Glycine_max
0,97
K7LES6_Glycine_max
0,98
I1KIQ2_Glycine_max
0,93
K4CQC0_Solanum_lycopersicum
0,67
A0A0F4ZI87_Thielaviopsis_punctulata
0,63
A0A0A7MCF9_Platynereis_dumerilii
0,96
F2E033_Hordeum_vulgare
1
W4XWI7_Strongylocentrotus_purpuratus
1
W4XWI8_Strongylocentrotus_purpuratus
W6FIM7_Stichopus_japonicus
Q1WDP1_Stichopus_japonicus
0.4

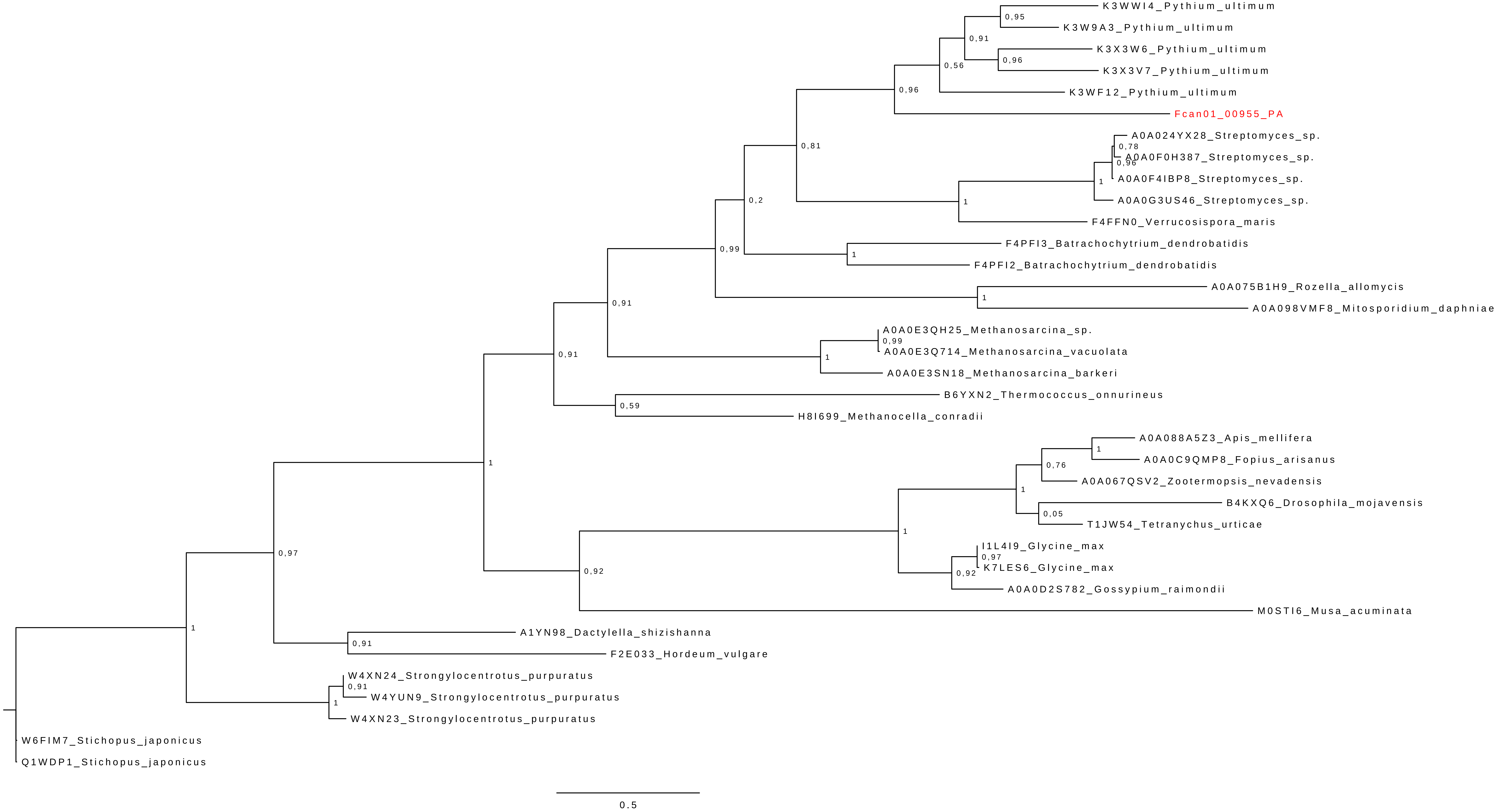

K3WWI4_Pythium_ultimum
K3W9A3_Pythium_ultimum
K3X3W6_Pythium_ultimum
K3X3V7_Pythium_ultimum
K3WF12_Pythium_ultimum
Fcan01_00955_PA
A0A024YX28_Streptomyces_sp.
A0A0F0H387_Streptomyces_sp.
A0A0F4IBP8_Streptomyces_sp.
A0A0G3US46_Streptomyces_sp.
F4FFN0_Verrucosispora_maris
F4PFI3_Batrachochytrium_dendrobatidis
F4PFI2_Batrachochytrium_dendrobatidis
A0A075B1H9_Rozella_allomycis
A0A098VMF8_Mitosporidium_daphniae
A0A0E3QH25_Methanosarcina_sp.
A0A0E3Q714_Methanosarcina_vacuolata
A0A0E3SN18_Methanosarcina_barkeri
B6YXN2_Thermococcus_onnurineus
H8I699_Methanocella_conradii
A0A088A5Z3_Apis_mellifera
A0A0C9QMP8_Fopius_arisanus
A0A067QSV2_Zootermopsis_nevadensis
B4KXQ6_Drosophila_mojavensis
T1JW54_Tetranychus_urticae
I1L4I9_Glycine_max
K7LES6_Glycine_max
A0A0D2S782_Gossypium_raimondii
M0STI6_Musa_acuminata
A1YN98_Dactylella_shizishanna
F2E033_Hordeum_vulgare
W4XN24_Strongylocentrotus_purpuratus
W4YUN9_Strongylocentrotus_purpuratus
W4XN23_Strongylocentrotus_purpuratus
W6FIM7_Stichopus_japonicus
Q1WDP1_Stichopus_japonicus
0,95
0,91
0,96
0,56
0,96
0,81
0,78
0,96
1
1
0,2
0,99
1
1
0,91
0,99
1
0,91
0,59
1
0,76
1
0,05
1
0,97
0,92
0,92
0,97
1
0,91
0,91
1
0.5

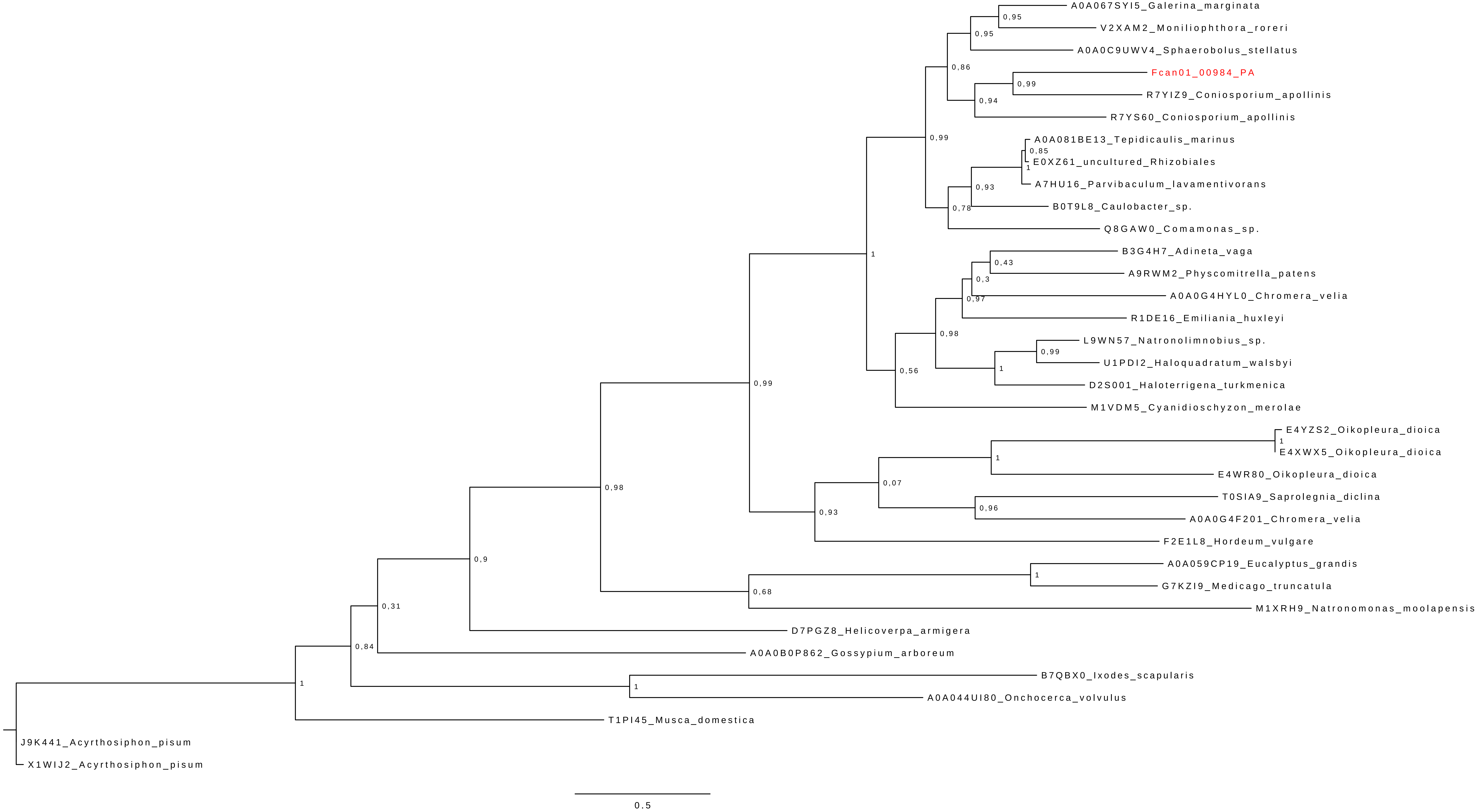
A0A067SYI5_Galerina_marginata
0,95
V2XAM2_Moniliophthora_roreri
0,95
A0A0C9UWV4_Sphaerobolus_stellatus
0,86
Fcan01_00984_PA
0,99
R7YIZ9_Coniosporium_apollinis
0,94
R7YS60_Coniosporium_apollinis
0,99
A0A081BE13_Tepidicaulis_marinus
0,85
E0XZ61_uncultured_Rhizobiales
1
A7HU16_Parvibaculum_lavamentivorans
0,93
B0T9L8_Caulobacter_sp.
0,78
Q8GAW0_Comamonas_sp.
1
B3G4H7_Adineta_vaga
0,43
A9RWM2_Physcomitrella_patens
0,3
A0A0G4HYL0_Chromera_velia
0,97
R1DE16_Emiliania_huxleyi
0,98
L9WN57_Natronolimnobius_sp.
0,99
U1PDI2_Haloquadratum_walsbyi
1
D2S001_Haloterrigena_turkmenica
0,56
M1VDM5_Cyanidioschyzon_merolae
0,99
E4YZS2_Oikopleura_dioica
1
E4XWX5_Oikopleura_dioica
1
E4WR80_Oikopleura_dioica
0,07
T0SIA9_Saprolegnia_diclina
0,96
A0A0G4F201_Chromera_velia
0,93
F2E1L8_Hordeum_vulgare
0,98
A0A059CP19_Eucalyptus_grandis
1
G7KZI9_Medicago_truncatula
0,68
M1XRH9_Natronomonas_moolapensis
0,9
D7PGZ8_Helicoverpa_armigera
0,31
A0A0B0P862_Gossypium_arboreum
0,84
B7QBX0_Ixodes_scapularis
1
A0A044UI80_Onchocerca_volvulus
1
T1PI45_Musca_domestica
J9K441_Acyrthosiphon_pisum
X1WIJ2_Acyrthosiphon_pisum
0.5

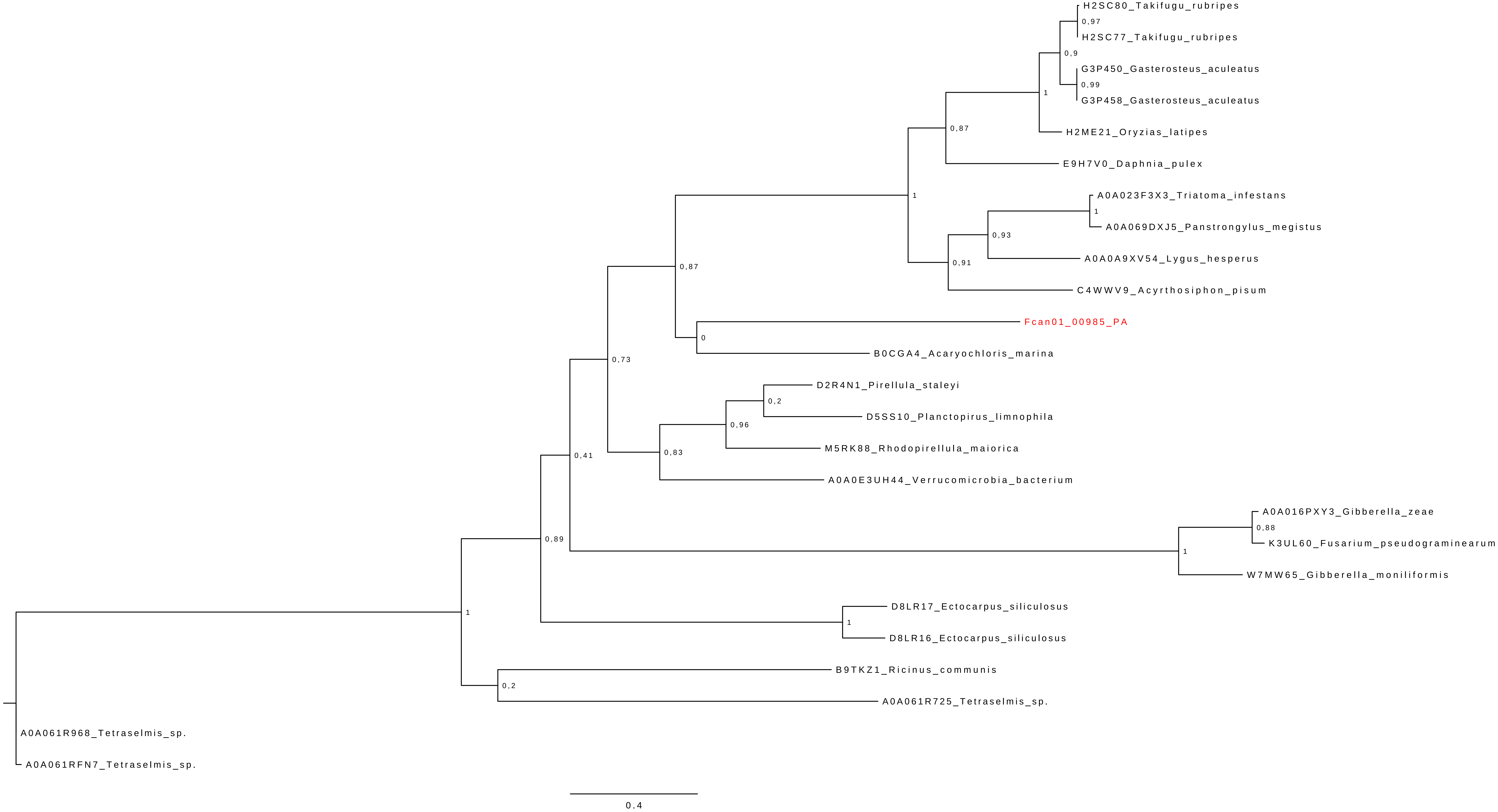
H2SC80_Takifugu_rubripes
0,97
H2SC77_Takifugu_rubripes
0,9
G3P450_Gasterosteus_aculeatus
0,99
G3P458_Gasterosteus_aculeatus
1
H2ME21_Oryzias_latipes
0,87
E9H7V0_Daphnia_pulex
1
A0A023F3X3_Triatoma_infestans
1
A0A069DXJ5_Panstrongylus_megistus
0,93
A0A0A9XV54_Lygus_hesperus
0,91
C4WWV9_Acyrthosiphon_pisum
0,87
Fcan01_00985_PA
0
B0CGA4_Acaryochloris_marina
0,73
D2R4N1_Pirellula_staleyi
0,2
D5SS10_Planctopirus_limnophila
0,96
M5RK88_Rhodopirellula_maiorica
0,83
A0A0E3UH44_Verrucomicrobia_bacterium
0,41
A0A016PXY3_Gibberella_zeae
0,88
K3UL60_Fusarium_pseudograminearum
1
W7MW65_Gibberella_moniliformis
0,89
D8LR17_Ectocarpus_siliculosus
1
D8LR16_Ectocarpus_siliculosus
1
B9TKZ1_Ricinus_communis
0,2
A0A061R725_Tetraselmis_sp.
A0A061R968_Tetraselmis_sp.
A0A061RFN7_Tetraselmis_sp.
0.4

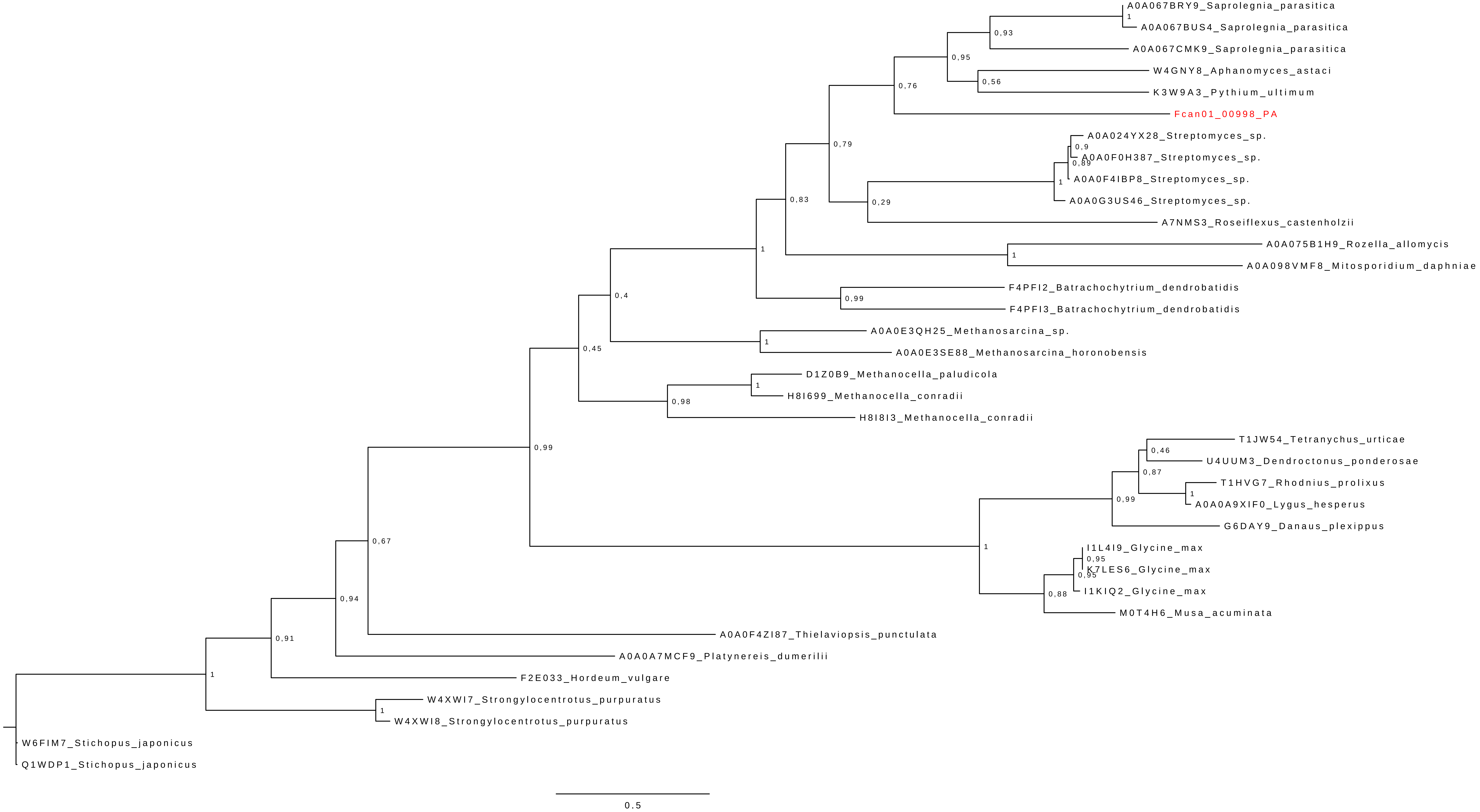

A0A067BRY9_Saprolegnia_parasitica
1
A0A067BUS4_Saprolegnia_parasitica
0,93
A0A067CMK9_Saprolegnia_parasitica
0,95
W4GNY8_Aphanomyces_astaci
0,56
K3W9A3_Pythium_ultimum
0,76
Fcan01_00998_PA
A0A024YX28_Streptomyces_sp.
0,9
0,79
A0A0F0H387_Streptomyces_sp.
0,89
A0A0F4IBP8_Streptomyces_sp.
1
0,83
A0A0G3US46_Streptomyces_sp.
0,29
A7NMS3_Roseiflexus_castenholzii
A0A075B1H9_Rozella_allomycis
1
1
A0A098VMF8_Mitosporidium_daphniae
F4PFI2_Batrachochytrium_dendrobatidis
0,4
0,99
F4PFI3_Batrachochytrium_dendrobatidis
A0A0E3QH25_Methanosarcina_sp.
1
0,45
A0A0E3SE88_Methanosarcina_horonobensis
D1Z0B9_Methanocella_paludicola
1
H8I699_Methanocella_conradii
0,98
H8I8I3_Methanocella_conradii
T1JW54_Tetranychus_urticae
0,99
0,46
U4UUM3_Dendroctonus_ponderosae
0,87
T1HVG7_Rhodnius_prolixus
1
0,99
A0A0A9XIF0_Lygus_hesperus
G6DAY9_Danaus_plexippus
0,67
1
I1L4I9_Glycine_max
0,95
K7LES6_Glycine_max
0,95
I1KIQ2_Glycine_max
0,94
0,88
M0T4H6_Musa_acuminata
A0A0F4ZI87_Thielaviopsis_punctulata
0,91
A0A0A7MCF9_Platynereis_dumerilii
1
F2E033_Hordeum_vulgare
W4XWI7_Strongylocentrotus_purpuratus
1
W4XWI8_Strongylocentrotus_purpuratus
W6FIM7_Stichopus_japonicus
Q1WDP1_Stichopus_japonicus
0.5

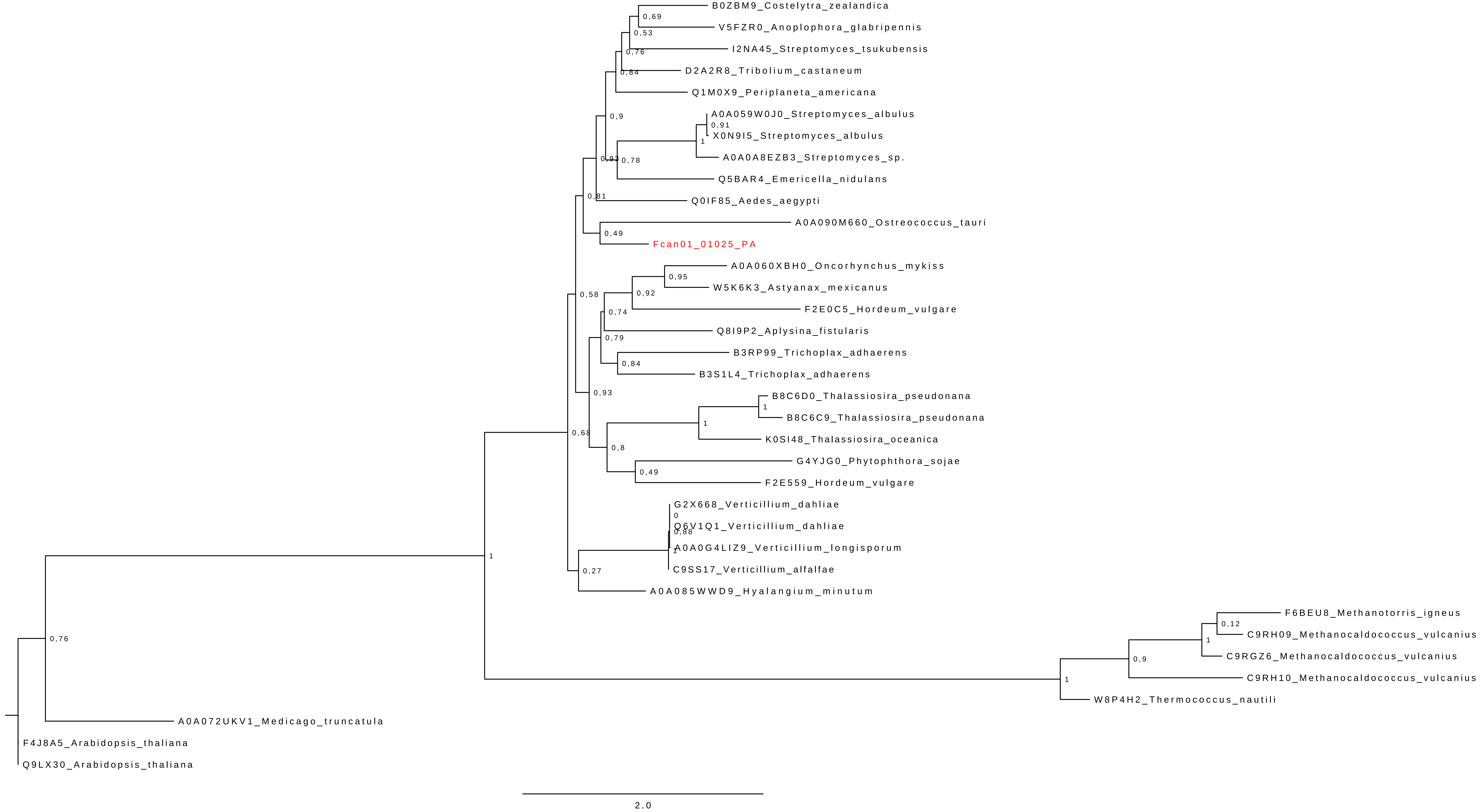
B0ZBM9_Costelytra_zealandica
V5FZR0_Anoplophora_glabripennis
I2NA45_Streptomyces_tsukubensis
D2A2R8_Tribolium_castaneum
Q1M0X9_Periplaneta_americana
A0A059W0J0_Streptomyces_albulus
X0N9I5_Streptomyces_albulus
A0A0A8EZB3_Streptomyces_sp.
Q5BAR4_Emericella_nidulans
Q0IF85_Aedes_aegypti
A0A090M660_Ostreococcus_tauri
Fcan01_01025_PA
A0A060XBH0_Oncorhynchus_mykiss
W5K6K3_Astyanax_mexicanus
F2E0C5_Hordeum_vulgare
Q8I9P2_Aplysina_fistularis
B3RP99_Trichoplax_adhaerens
B3S1L4_Trichoplax_adhaerens
B8C6D0_Thalassiosira_pseudonana
B8C6C9_Thalassiosira_pseudonana
K0SI48_Thalassiosira_oceanica
G4YJG0_Phytophthora_sojae
F2E559_Hordeum_vulgare
G2X668_Verticillium_dahliae
Q6V1Q1_Verticillium_dahliae
A0A0G4LIZ9_Verticillium_longisporum
C9SS17_Verticillium_alfalfae
A0A085WWD9_Hyalangium_minutum
F6BEU8_Methanotorris_igneus
C9RH09_Methanocaldococcus_vulcanius
C9RGZ6_Methanocaldococcus_vulcanius
C9RH10_Methanocaldococcus_vulcanius
W8P4H2_Thermococcus_nautili
A0A072UKV1_Medicago_truncatula
F4J8A5_Arabidopsis_thaliana
Q9LX30_Arabidopsis_thaliana
2.0

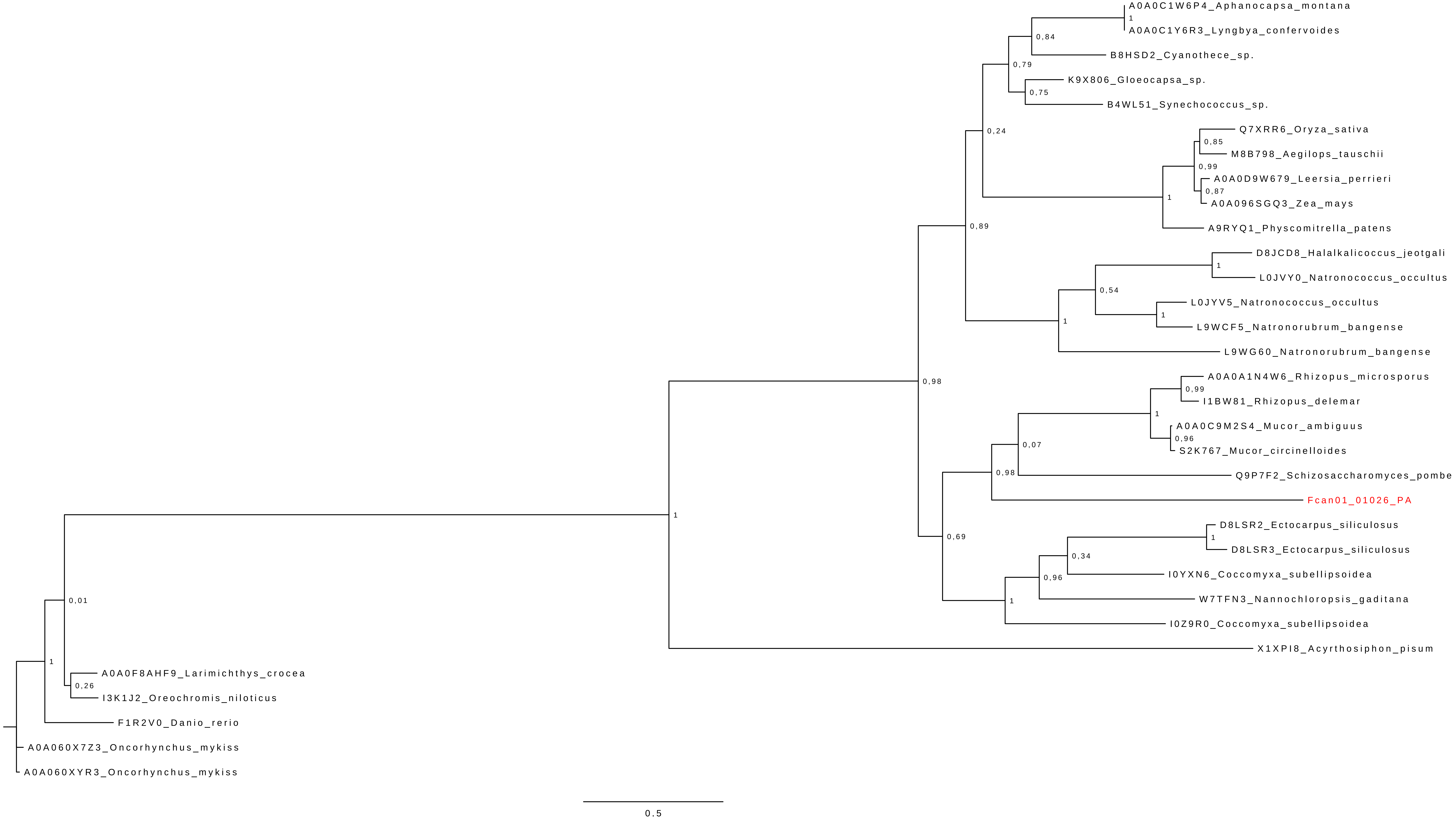
A0A0C1W6P4_Aphanocapsa_montana
A0A0C1Y6R3_Lyngbya_confervoides
B8HSD2_Cyanothece_sp.
K9X806_Gloeocapsa_sp.
B4WL51_Synechococcus_sp.
Q7XRR6_Oryza_sativa
M8B798_Aegilops_tauschii
A0A0D9W679_Leersia_perrieri
A0A096SGQ3_Zea_mays
A9RYQ1_Physcomitrella_patens
D8JCD8_Halalkalicoccus_jeotgali
L0JVY0_Natronococcus_occultus
L0JYV5_Natronococcus_occultus
L9WCF5_Natronorubrum_bangense
L9WG60_Natronorubrum_bangense
A0A0A1N4W6_Rhizopus_microsporus
I1BW81_Rhizopus_delemar
A0A0C9M2S4_Mucor_ambiguus
S2K767_Mucor_circinelloides
Q9P7F2_Schizosaccharomyces_pombe
Fcan01_01026_PA
D8LSR2_Ectocarpus_siliculosus
D8LSR3_Ectocarpus_siliculosus
I0YXN6_Coccomyxa_subellipsoidea
W7TFN3_Nannochloropsis_gaditana
I0Z9R0_Coccomyxa_subellipsoidea
X1XPI8_Acyrthosiphon_pisum
A0A0F8AHF9_Larimichthys_crocea
I3K1J2_Oreochromis_niloticus
F1R2V0_Danio_rerio
A0A060X7Z3_Oncorhynchus_mykiss
A0A060XYR3_Oncorhynchus_mykiss
1
0,84
0,79
0,75
0,24
0,85
0,99
0,87
1
0,89
1
0,54
1
1
0,98
0,99
1
0,96
0,07
0,98
0,69
1
0,34
0,96
1
1
0,01
1
0,26
0.5

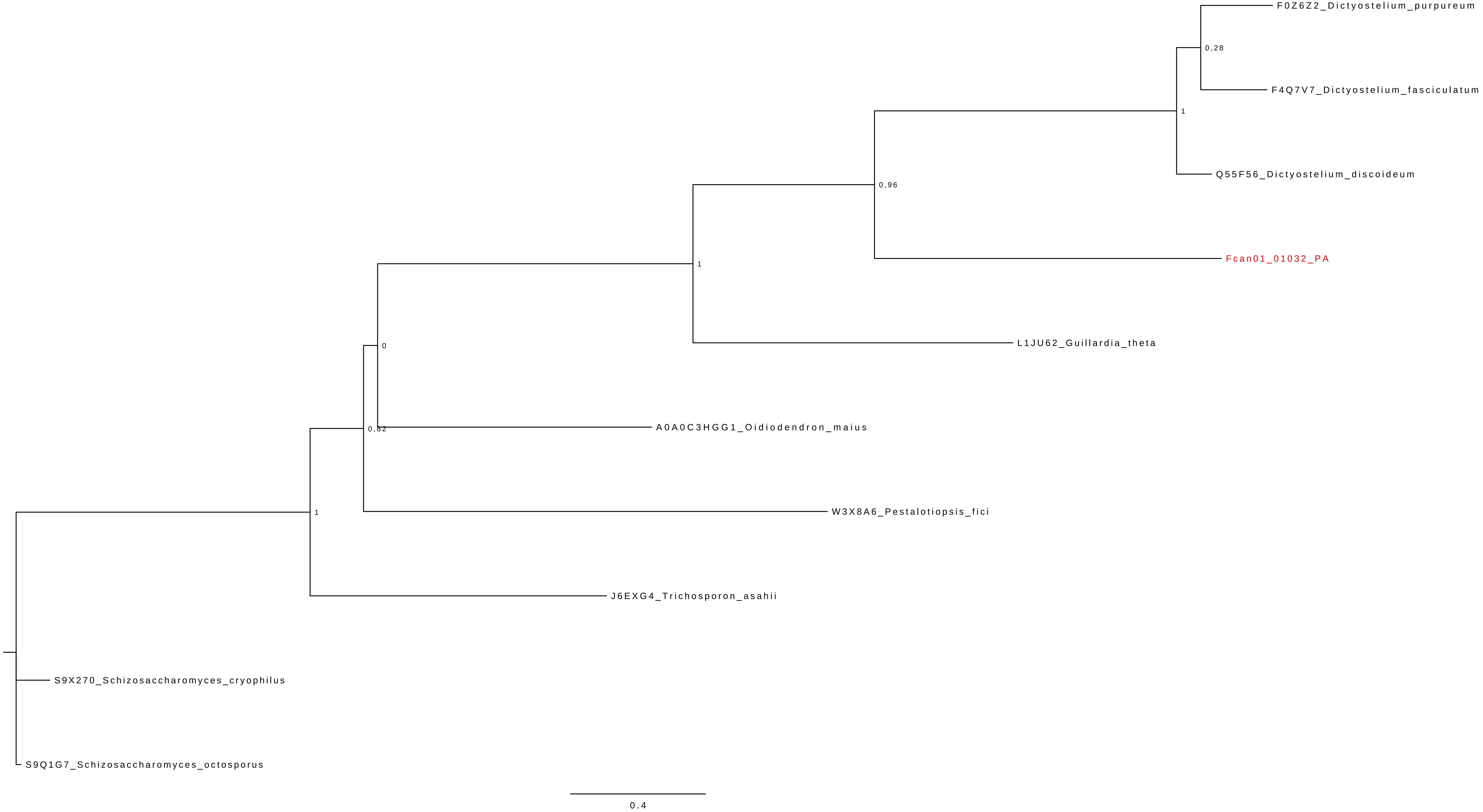
F0Z6Z2_Dictyostelium_purpureum
0,28
F4Q7V7_Dictyostelium_fasciculatum
1
Q55F56_Dictyostelium_discoideum
0,96
Fcan01_01032_PA
1
L1JU62_Guillardia_theta
0
A0A0C3HGG1_Oidiodendron_maius
0,82
W3X8A6_Pestalotiopsis_fici
1
J6EXG4_Trichosporon_asahii
S9X270_Schizosaccharomyces_cryophilus
S9Q1G7_Schizosaccharomyces_octosporus
0.4

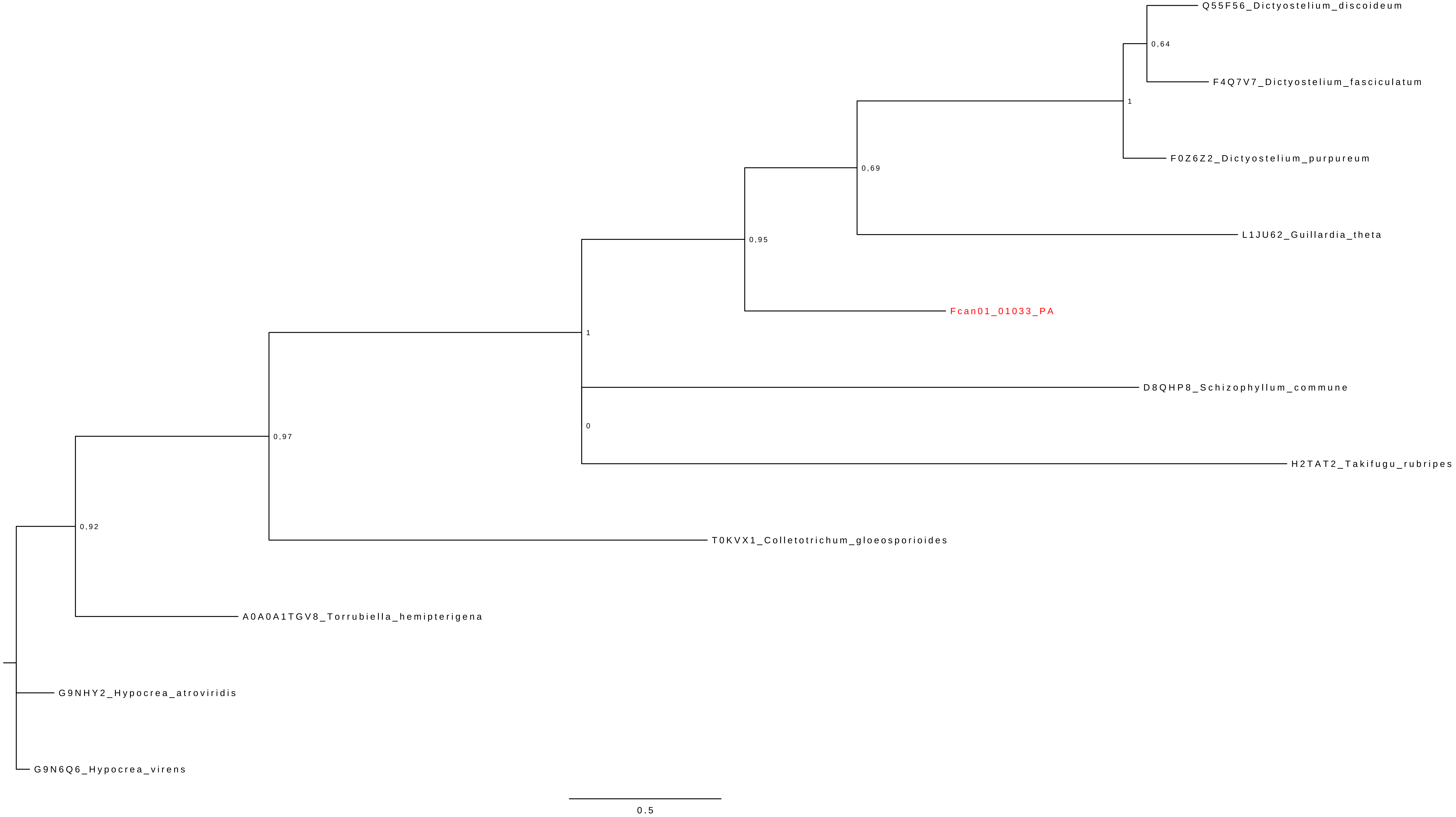
Q55F56_Dictyostelium_discoideum
0,64
F4Q7V7_Dictyostelium_fasciculatum
1
F0Z6Z2_Dictyostelium_purpureum
0,69
L1JU62_Guillardia_theta
0,95
Fcan01_01033_PA
1
D8QHP8_Schizophyllum_commune
0
H2TAT2_Takifugu_rubripes
0,97
0,92
T0KVX1_Colletotrichum_gloeosporioides
A0A0A1TGV8_Torrubiella_hemipterigena
G9NHY2_Hypocrea_atroviridis
G9N6Q6_Hypocrea_virens
0.5

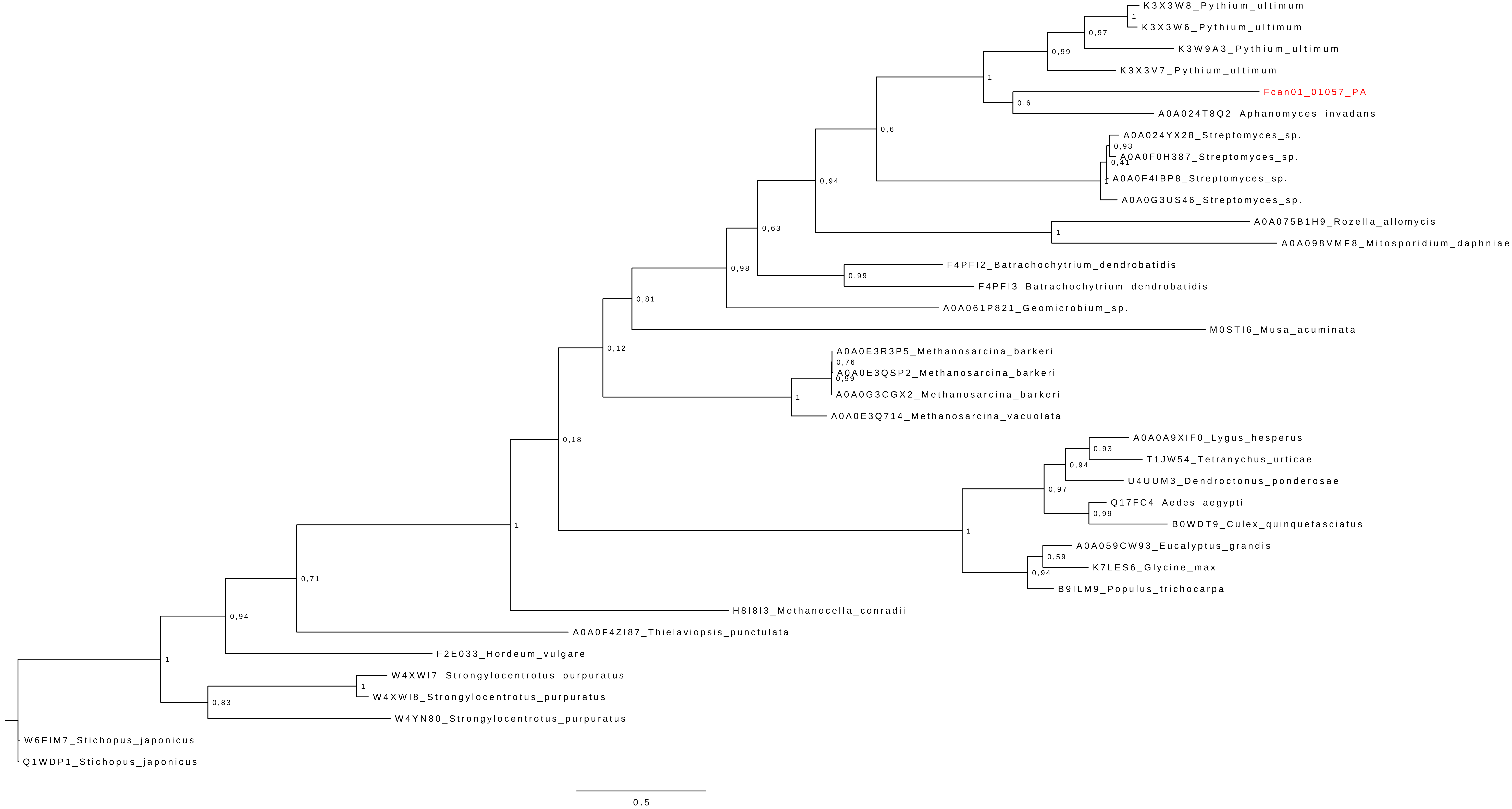
K3X3W8_Pythium_ultimum
K3X3W6_Pythium_ultimum
K3W9A3_Pythium_ultimum
K3X3V7_Pythium_ultimum
Fcan01_01057_PA
A0A024T8Q2_Aphanomyces_invadans
A0A024YX28_Streptomyces_sp.
A0A0F0H387_Streptomyces_sp.
A0A0F4IBP8_Streptomyces_sp.
A0A0G3US46_Streptomyces_sp.
A0A075B1H9_Rozella_allomycis
A0A098VMF8_Mitosporidium_daphniae
F4PFI2_Batrachochytrium_dendrobatidis
F4PFI3_Batrachochytrium_dendrobatidis
A0A061P821_Geomicrobium_sp.
M0STI6_Musa_acuminata
A0A0E3R3P5_Methanosarcina_barkeri
A0A0E3QSP2_Methanosarcina_barkeri
A0A0G3CGX2_Methanosarcina_barkeri
A0A0E3Q714_Methanosarcina_vacuolata
A0A0A9XIF0_Lygus_hesperus
T1JW54_Tetranychus_urticae
U4UUM3_Dendroctonus_ponderosae
Q17FC4_Aedes_aegypti
B0WDT9_Culex_quinquefasciatus
A0A059CW93_Eucalyptus_grandis
K7LES6_Glycine_max
B9ILM9_Populus_trichocarpa
H8I8I3_Methanocella_conradii
A0A0F4ZI87_Thielaviopsis_punctulata
F2E033_Hordeum_vulgare
W4XWI7_Strongylocentrotus_purpuratus
W4XWI8_Strongylocentrotus_purpuratus
W4YN80_Strongylocentrotus_purpuratus
W6FIM7_Stichopus_japonicus
Q1WDP1_Stichopus_japonicus
0.5

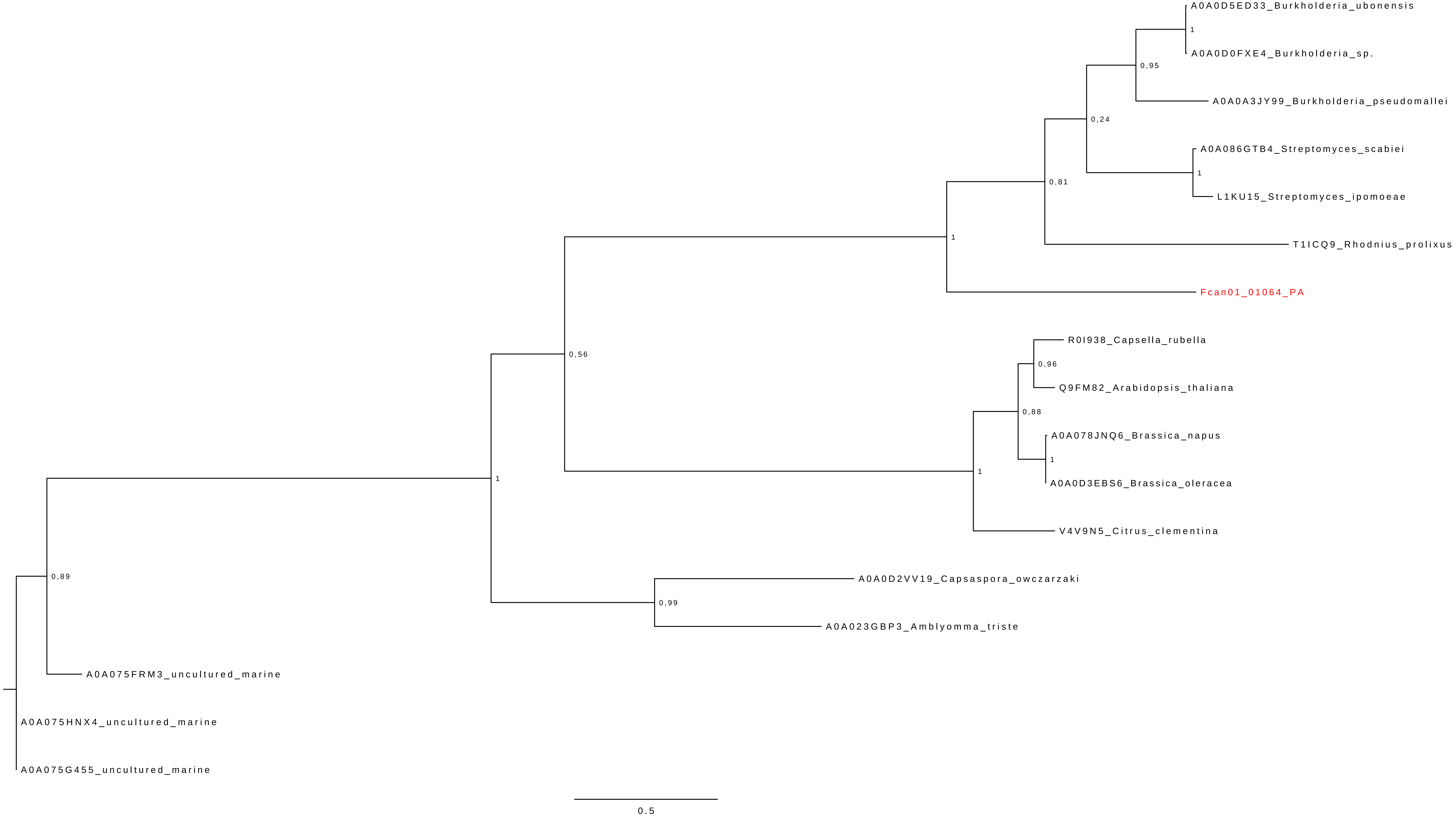
A0A0D5ED33_Burkholderia_ubonensis
1
A0A0D0FXE4_Burkholderia_sp.
0,95
A0A0A3JY99_Burkholderia_pseudomallei
0,24
A0A086GTB4_Streptomyces_scabiei
1
L1KU15_Streptomyces_ipomoeae
0,81
T1ICQ9_Rhodnius_prolixus
1
Fcan01_01064_PA
R0I938_Capsella_rubella
0,56
0,96
Q9FM82_Arabidopsis_thaliana
0,88
A0A078JNQ6_Brassica_napus
1
1
A0A0D3EBS6_Brassica_oleracea
1
V4V9N5_Citrus_clementina
A0A0D2VV19_Capsaspora_owczarzaki
0,89
0,99
A0A023GBP3_Amblyomma_triste
A0A075FRM3_uncultured_marine
A0A075HNX4_uncultured_marine
A0A075G455_uncultured_marine
0.5

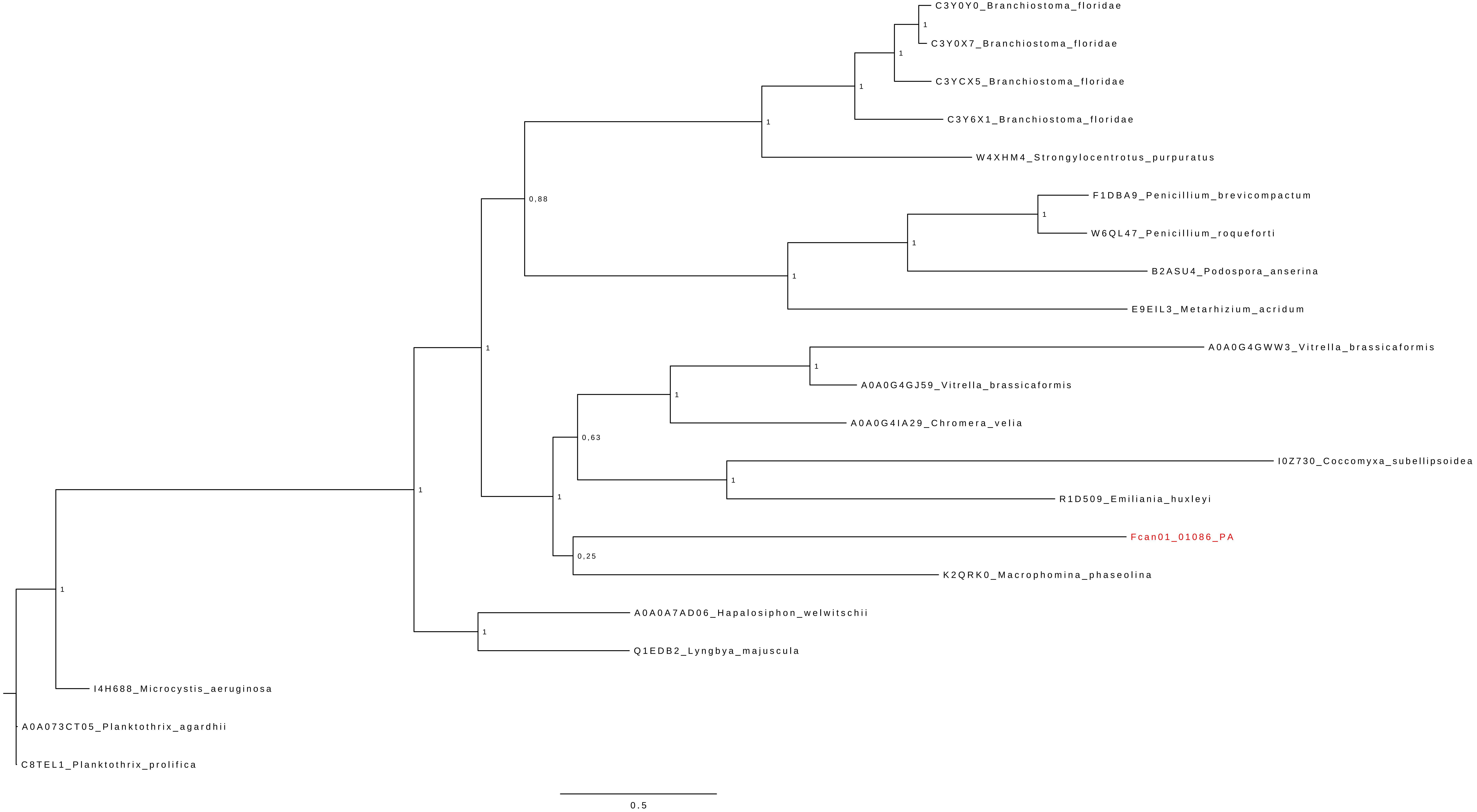
C3Y0Y0_Branchiostoma_floridae
C3Y0X7_Branchiostoma_floridae
C3YCX5_Branchiostoma_floridae
C3Y6X1_Branchiostoma_floridae
W4XHM4_Strongylocentrotus_purpuratus
F1DBA9_Penicillium_brevicompactum
W6QL47_Penicillium_roqueforti
B2ASU4_Podospora_anserina
E9EIL3_Metarhizium_acridum
A0A0G4GWW3_Vitrella_brassicaformis
A0A0G4GJ59_Vitrella_brassicaformis
A0A0G4IA29_Chromera_velia
I0Z730_Coccomyxa_subellipsoidea
R1D509_Emiliania_huxleyi
Fcan01_01086_PA
K2QRK0_Macrophomina_phaseolina
A0A0A7AD06_Hapalosiphon_welwitschii
Q1EDB2_Lyngbya_majuscula
I4H688_Microcystis_aeruginosa
A0A073CT05_Planktothrix_agardhii
C8TEL1_Planktothrix_prolifica
0,88
0,63
0,25
1
0.5

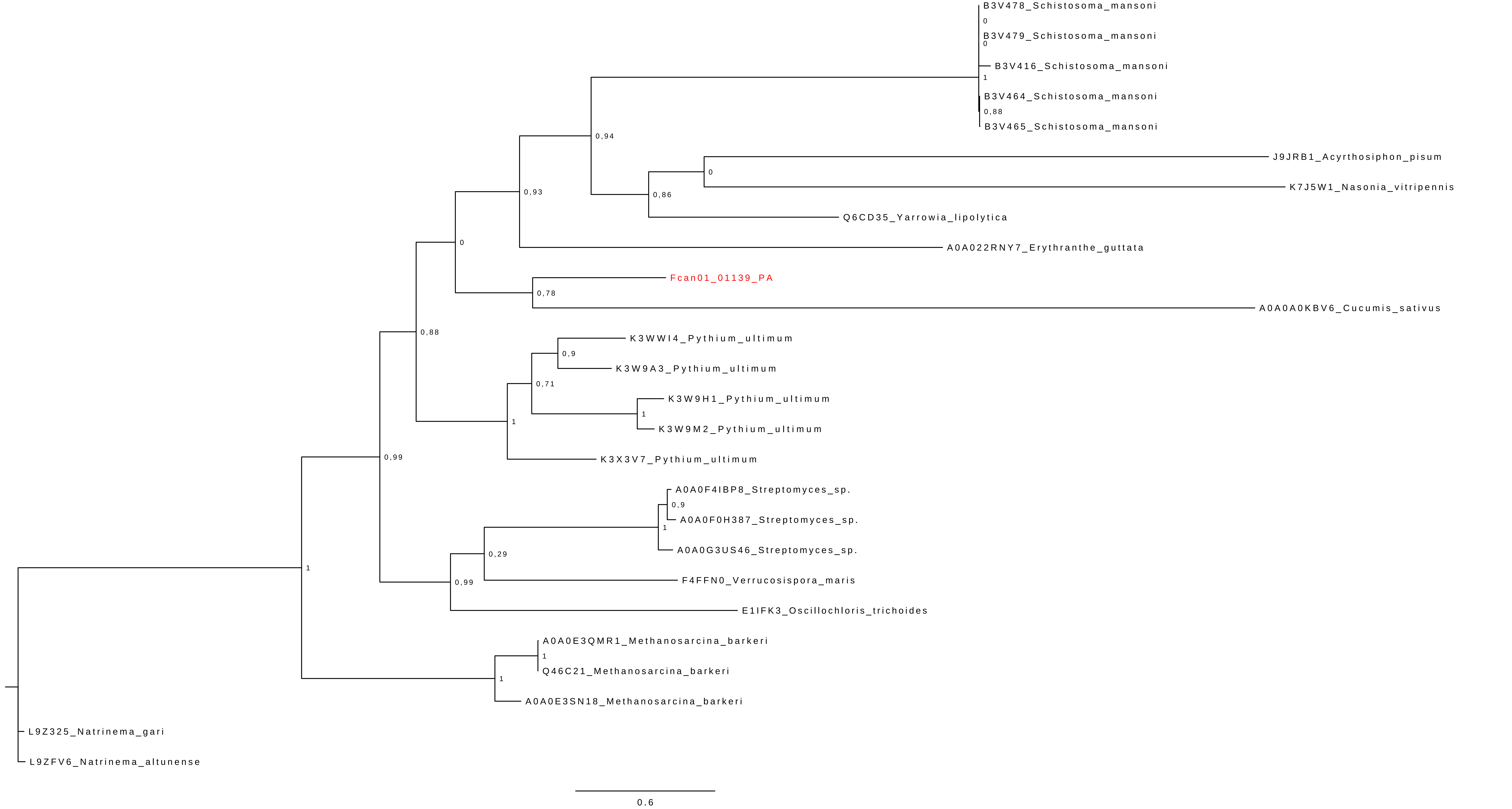
B3V478_Schistosoma_mansoni
0
B3V479_Schistosoma_mansoni
0
B3V416_Schistosoma_mansoni
1
B3V464_Schistosoma_mansoni
0,88
B3V465_Schistosoma_mansoni
0,94
J9JRB1_Acyrthosiphon_pisum
0
K7J5W1_Nasonia_vitripennis
0,86
Q6CD35_Yarrowia_lipolytica
0,93
A0A022RNY7_Erythranthe_guttata
0
Fcan01_01139_PA
0,78
A0A0A0KBV6_Cucumis_sativus
0,88
K3WWI4_Pythium_ultimum
0,9
K3W9A3_Pythium_ultimum
0,71
K3W9H1_Pythium_ultimum
1
K3W9M2_Pythium_ultimum
1
K3X3V7_Pythium_ultimum
0,99
A0A0F4IBP8_Streptomyces_sp.
0,9
A0A0F0H387_Streptomyces_sp.
1
A0A0G3US46_Streptomyces_sp.
0,29
F4FFN0_Verrucosispora_maris
0,99
E1IFK3_Oscillochloris_trichoides
1
A0A0E3QMR1_Methanosarcina_barkeri
1
Q46C21_Methanosarcina_barkeri
1
A0A0E3SN18_Methanosarcina_barkeri
L9Z325_Natrinema_gari
L9ZFV6_Natrinema_altunense
0.6

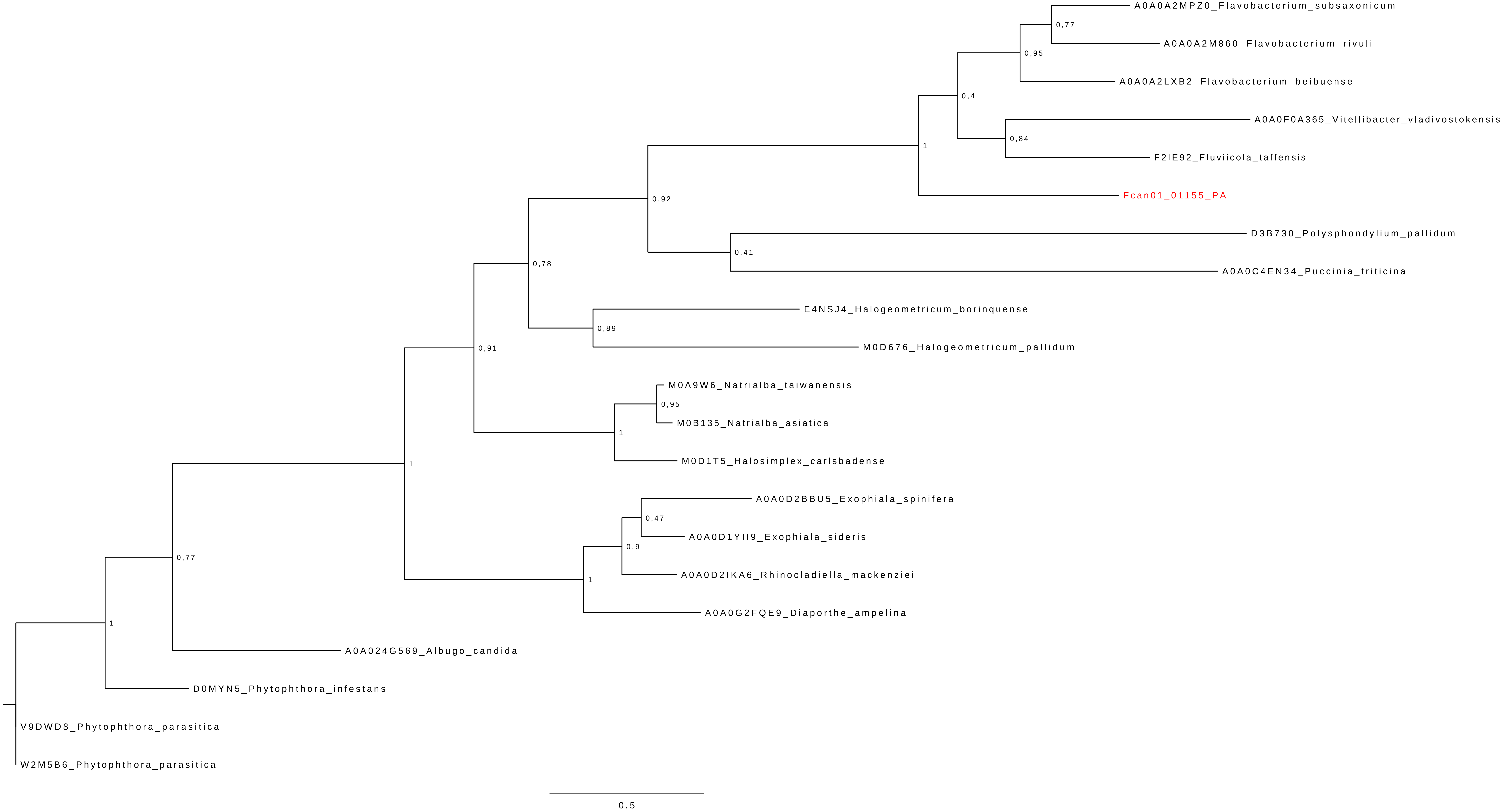
A0A0A2MPZ0_Flavobacterium_subsaxonicum
0,77
A0A0A2M860_Flavobacterium_rivuli
0,95
A0A0A2LXB2_Flavobacterium_beibuense
0,4
A0A0F0A365_Vitellibacter_vladivostokensis
0,84
F2IE92_Fluviicola_taffensis
1
Fcan01_01155_PA
0,92
D3B730_Polysphondylium_pallidum
0,41
A0A0C4EN34_Puccinia_triticina
0,78
E4NSJ4_Halogeometricum_borinquense
0,89
M0D676_Halogeometricum_pallidum
0,91
M0A9W6_Natrialba_taiwanensis
0,95
M0B135_Natrialba_asiatica
1
M0D1T5_Halosimplex_carlsbadense
1
A0A0D2BBU5_Exophiala_spinifera
0,47
A0A0D1YII9_Exophiala_sideris
0,9
A0A0D2IKA6_Rhinocladiella_mackenziei
1
A0A0G2FQE9_Diaporthe_ampelina
0,77
A0A024G569_Albugo_candida
1
D0MYN5_Phytophthora_infestans
V9DWD8_Phytophthora_parasitica
W2M5B6_Phytophthora_parasitica
0.5

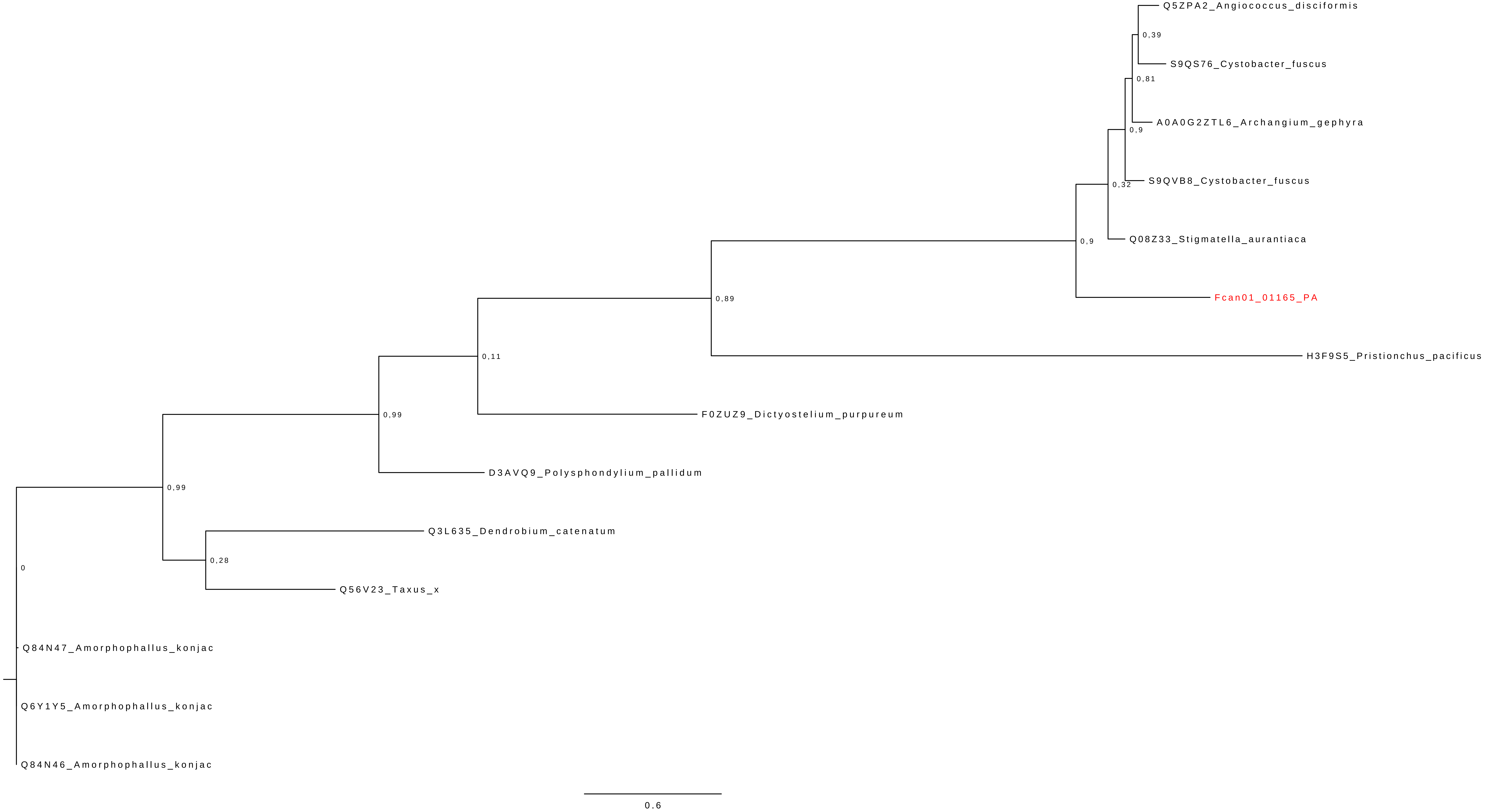
Q5ZPA2_Angiococcus_disciformis
0,39
S9QS76_Cystobacter_fuscus
0,81
A0A0G2ZTL6_Archangium_gephyra
0,9
S9QVB8_Cystobacter_fuscus
0,32
Q08Z33_Stigmatella_aurantiaca
0,9
Fcan01_01165_PA
0,89
H3F9S5_Pristionchus_pacificus
0,11
F0ZUZ9_Dictyostelium_purpureum
0,99
D3AVQ9_Polysphondylium_pallidum
0,99
Q3L635_Dendrobium_catenatum
0,28
Q56V23_Taxus_x
0
Q84N47_Amorphophallus_konjac
Q6Y1Y5_Amorphophallus_konjac
Q84N46_Amorphophallus_konjac
0.6

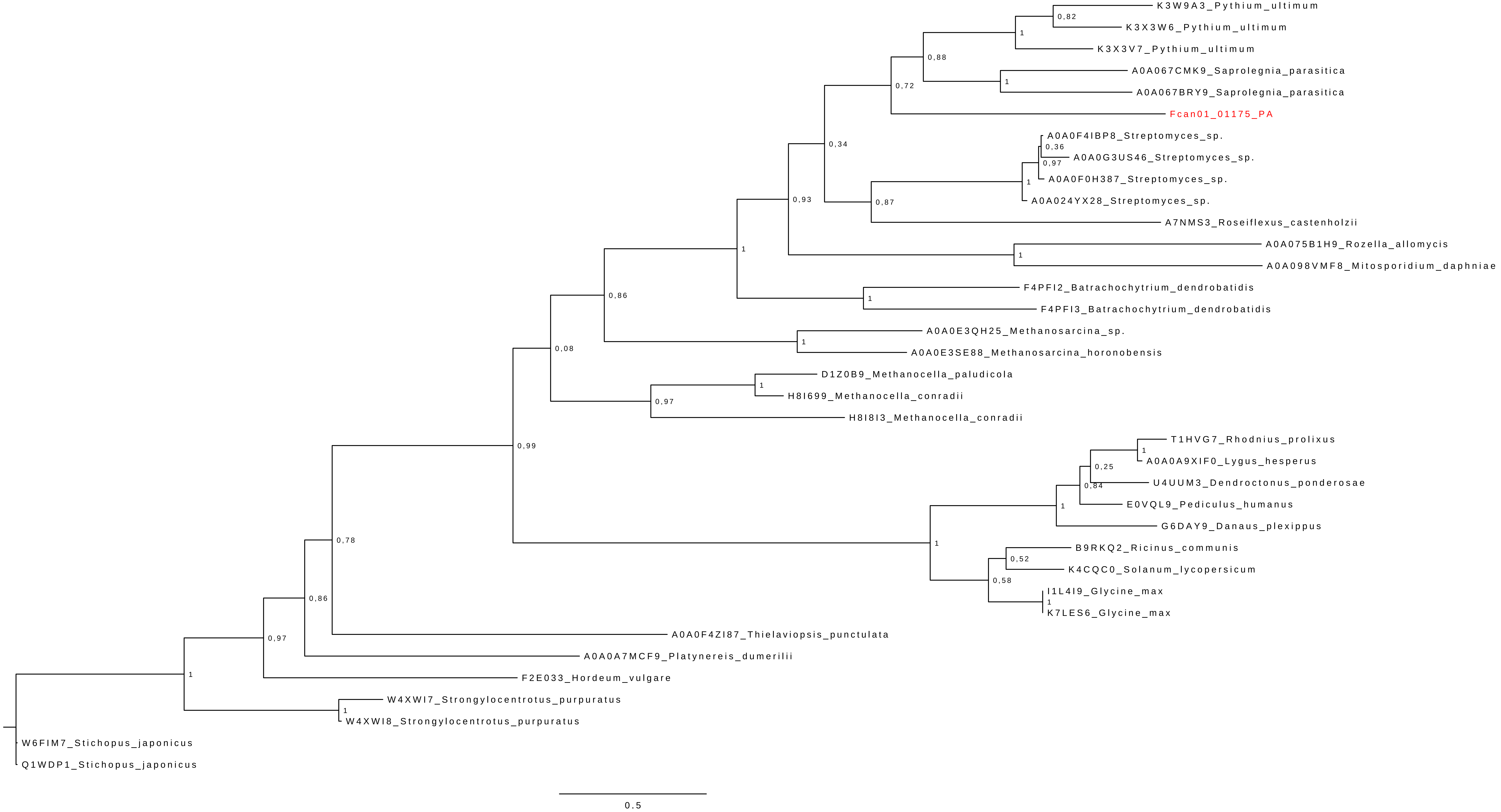
K3W9A3_Pythium_ultimum
K3X3W6_Pythium_ultimum
K3X3V7_Pythium_ultimum
A0A067CMK9_Saprolegnia_parasitica
A0A067BRY9_Saprolegnia_parasitica
Fcan01_01175_PA
A0A0F4IBP8_Streptomyces_sp.
A0A0G3US46_Streptomyces_sp.
A0A0F0H387_Streptomyces_sp.
A0A024YX28_Streptomyces_sp.
A7NMS3_Roseiflexus_castenholzii
A0A075B1H9_Rozella_allomycis
A0A098VMF8_Mitosporidium_daphniae
F4PFI2_Batrachochytrium_dendrobatidis
F4PFI3_Batrachochytrium_dendrobatidis
A0A0E3QH25_Methanosarcina_sp.
A0A0E3SE88_Methanosarcina_horonobensis
D1Z0B9_Methanocella_paludicola
H8I699_Methanocella_conradii
H8I8I3_Methanocella_conradii
T1HVG7_Rhodnius_prolixus
A0A0A9XIF0_Lygus_hesperus
U4UUM3_Dendroctonus_ponderosae
E0VQL9_Pediculus_humanus
G6DAY9_Danaus_plexippus
B9RKQ2_Ricinus_communis
K4CQC0_Solanum_lycopersicum
I1L4I9_Glycine_max
K7LES6_Glycine_max
A0A0F4ZI87_Thielaviopsis_punctulata
A0A0A7MCF9_Platynereis_dumerilii
F2E033_Hordeum_vulgare
W4XWI7_Strongylocentrotus_purpuratus
W4XWI8_Strongylocentrotus_purpuratus
W6FIM7_Stichopus_japonicus
Q1WDP1_Stichopus_japonicus
0,82
1
0,88
1
0,72
0,36
0,97
1
0,34
0,87
0,93
1
1
1
0,86
1
0,08
1
0,97
0,99
1
0,25
0,84
1
1
0,52
0,58
1
0,78
0,86
0,97
1
1
0.5

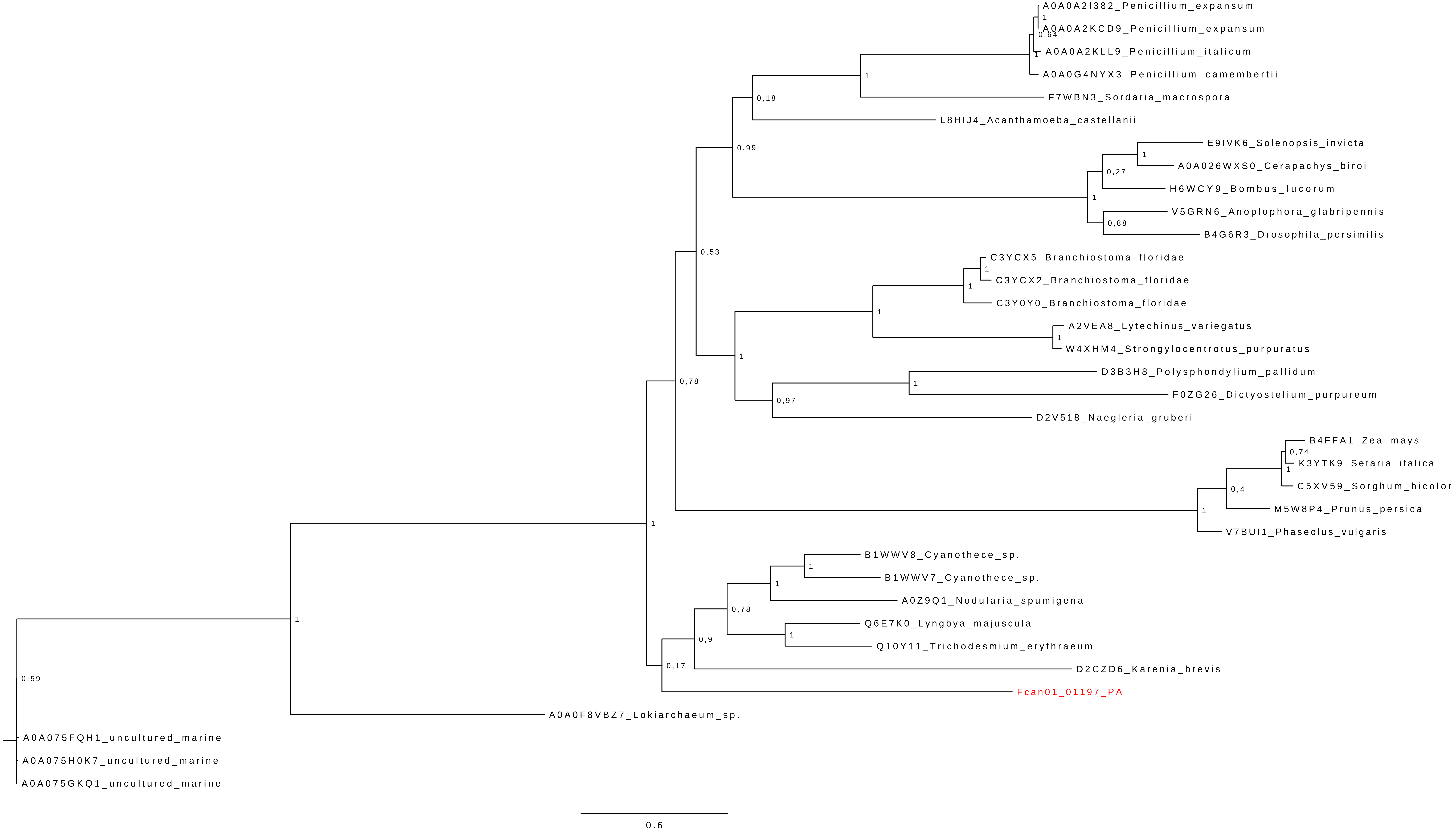
A0A0A2I382_Penicillium_expansum
1
A0A0A2KCD9_Penicillium_expansum
0,64
A0A0A2KLL9_Penicillium_italicum
1
A0A0G4NYX3_Penicillium_camembertii
1
F7WBN3_Sordaria_macrospora
0,18
L8HIJ4_Acanthamoeba_castellanii
E9IVK6_Solenopsis_invicta
1
A0A026WXS0_Cerapachys_biroi
0,27
H6WCY9_Bombus_lucorum
0,99
1
V5GRN6_Anoplophora_glabripennis
0,88
B4G6R3_Drosophila_persimilis
0,53
C3YCX5_Branchiostoma_floridae
1
C3YCX2_Branchiostoma_floridae
1
C3Y0Y0_Branchiostoma_floridae
1
A2VEA8_Lytechinus_variegatus
1
W4XHM4_Strongylocentrotus_purpuratus
1
D3B3H8_Polysphondylium_pallidum
1
0,78
F0ZG26_Dictyostelium_purpureum
0,97
D2V518_Naegleria_gruberi
B4FFA1_Zea_mays
0,74
K3YTK9_Setaria_italica
1
C5XV59_Sorghum_bicolor
0,4
M5W8P4_Prunus_persica
1
V7BUI1_Phaseolus_vulgaris
1
B1WWV8_Cyanothece_sp.
1
B1WWV7_Cyanothece_sp.
1
A0Z9Q1_Nodularia_spumigena
0,78
Q6E7K0_Lyngbya_majuscula
1
1
0,9
Q10Y11_Trichodesmium_erythraeum
0,17
D2CZD6_Karenia_brevis
Fcan01_01197_PA
A0A0F8VBZ7_Lokiarchaeum_sp.
0,59
A0A075FQH1_uncultured_marine
A0A075H0K7_uncultured_marine
A0A075GKQ1_uncultured_marine
0.6

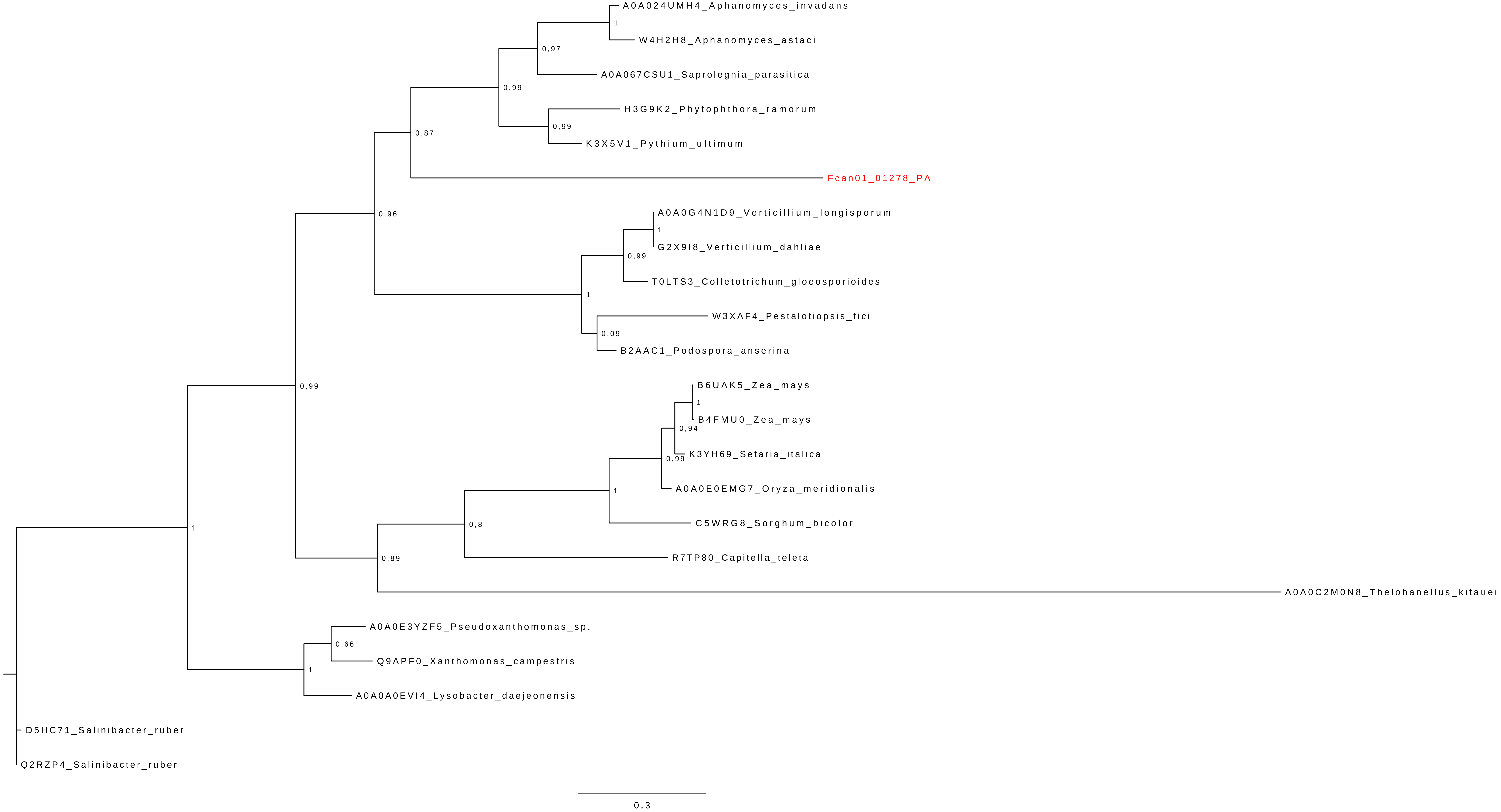
A0A024UMH4_Aphanomyces_invadans
1
W4H2H8_Aphanomyces_astaci
0,97
A0A067CSU1_Saprolegnia_parasitica
0,99
H3G9K2_Phytophthora_ramorum
0,99
K3X5V1_Pythium_ultimum
0,87
Fcan01_01278_PA
0,96
A0A0G4N1D9_Verticillium_longisporum
1
G2X9I8_Verticillium_dahliae
0,99
T0LTS3_Colletotrichum_gloeosporioides
1
W3XAF4_Pestalotiopsis_fici
0,09
B2AAC1_Podospora_anserina
0,99
B6UAK5_Zea_mays
1
B4FMU0_Zea_mays
0,94
K3YH69_Setaria_italica
0,99
A0A0E0EMG7_Oryza_meridionalis
1
C5WRG8_Sorghum_bicolor
0,8
1
R7TP80_Capitella_teleta
0,89
A0A0C2M0N8_Thelohanellus_kitauei
A0A0E3YZF5_Pseudoxanthomonas_sp.
0,66
Q9APF0_Xanthomonas_campestris
1
A0A0A0EVI4_Lysobacter_daejeonensis
D5HC71_Salinibacter_ruber
Q2RZP4_Salinibacter_ruber
0.3

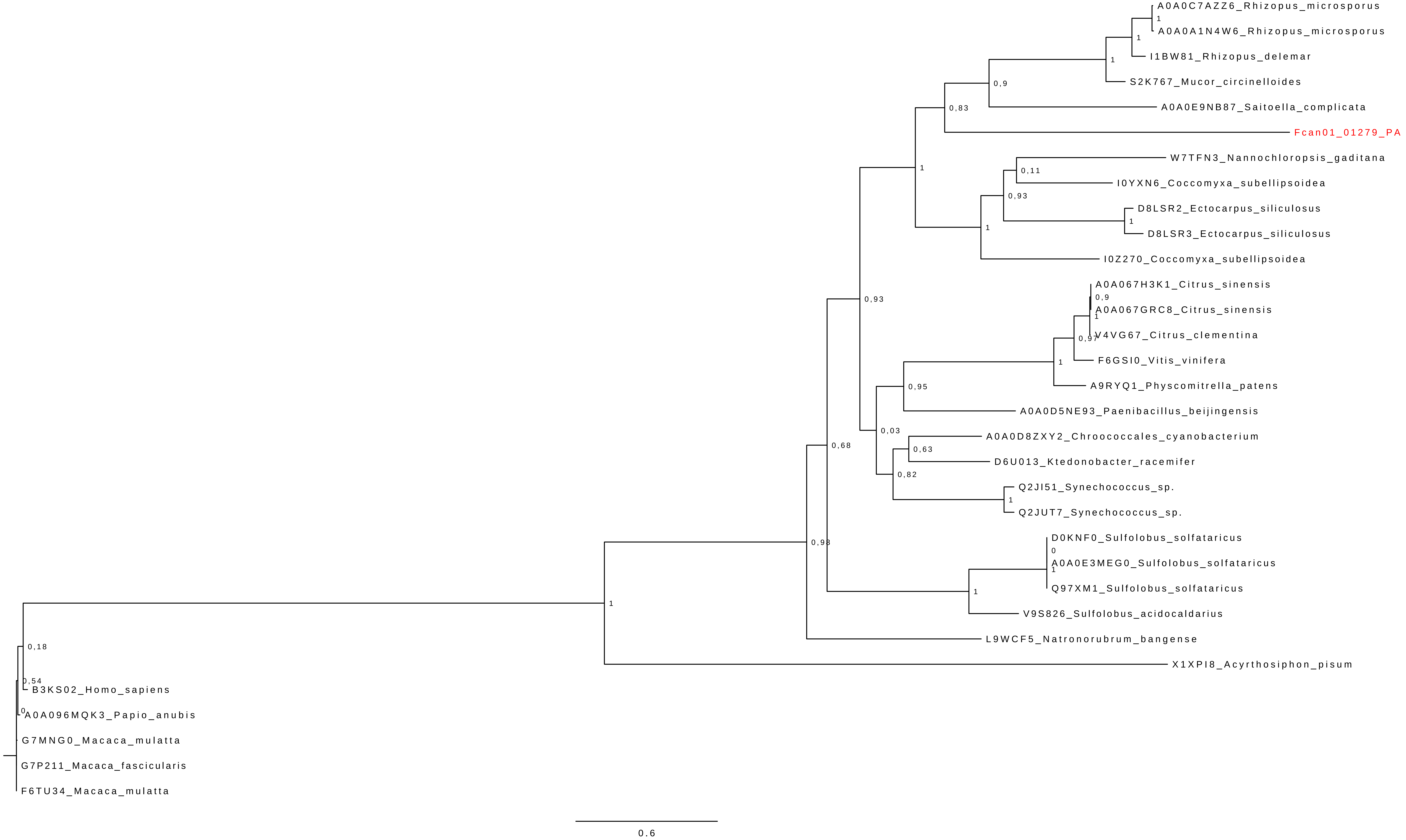
A0A0C7AZZ6_Rhizopus_microsporus
A0A0A1N4W6_Rhizopus_microsporus
I1BW81_Rhizopus_delemar
S2K767_Mucor_circinelloides
A0A0E9NB87_Saitoella_complicata
Fcan01_01279_PA
W7TFN3_Nannochloropsis_gaditana
I0YXN6_Coccomyxa_subellipsoidea
D8LSR2_Ectocarpus_siliculosus
D8LSR3_Ectocarpus_siliculosus
I0Z270_Coccomyxa_subellipsoidea
A0A067H3K1_Citrus_sinensis
A0A067GRC8_Citrus_sinensis
V4VG67_Citrus_clementina
F6GSI0_Vitis_vinifera
A9RYQ1_Physcomitrella_patens
A0A0D5NE93_Paenibacillus_beijingensis
A0A0D8ZXY2_Chroococcales_cyanobacterium
D6U013_Ktedonobacter_racemifer
Q2JI51_Synechococcus_sp.
Q2JUT7_Synechococcus_sp.
D0KNF0_Sulfolobus_solfataricus
A0A0E3MEG0_Sulfolobus_solfataricus
Q97XM1_Sulfolobus_solfataricus
V9S826_Sulfolobus_acidocaldarius
L9WCF5_Natronorubrum_bangense
X1XPI8_Acyrthosiphon_pisum
B3KS02_Homo_sapiens
A0A096MQK3_Papio_anubis
G7MNG0_Macaca_mulatta
G7P211_Macaca_fascicularis
F6TU34_Macaca_mulatta
0.6

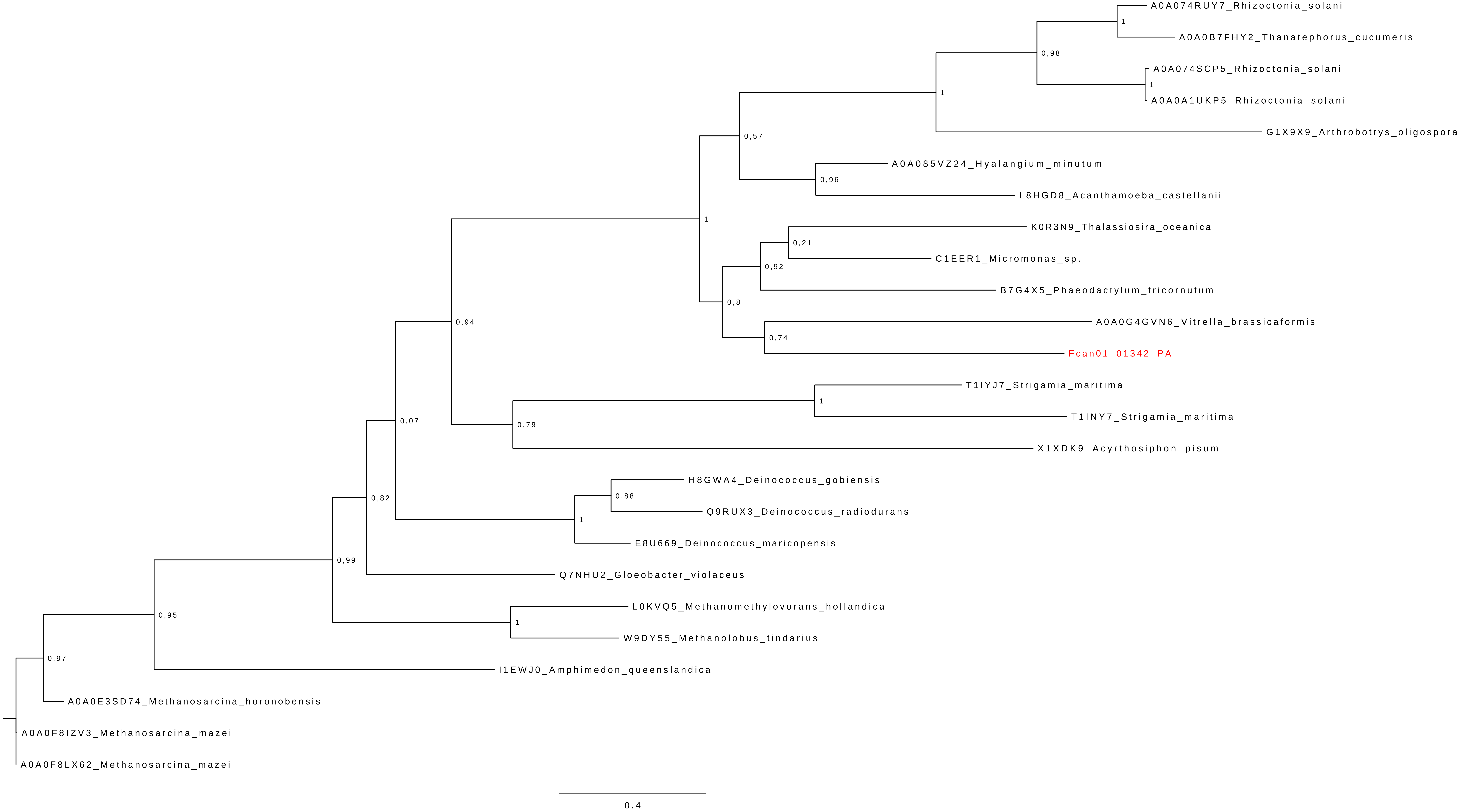
A0A074RUY7_Rhizoctonia_solani
1
A0A0B7FHY2_Thanatephorus_cucumeris
0,98
A0A074SCP5_Rhizoctonia_solani
1
1
A0A0A1UKP5_Rhizoctonia_solani
G1X9X9_Arthrobotrys_oligospora
0,57
A0A085VZ24_Hyalangium_minutum
0,96
L8HGD8_Acanthamoeba_castellanii
1
K0R3N9_Thalassiosira_oceanica
0,21
C1EER1_Micromonas_sp.
0,92
B7G4X5_Phaeodactylum_tricornutum
0,8
A0A0G4GVN6_Vitrella_brassicaformis
0,94
0,74
Fcan01_01342_PA
T1IYJ7_Strigamia_maritima
1
0,07
0,79
T1INY7_Strigamia_maritima
X1XDK9_Acyrthosiphon_pisum
H8GWA4_Deinococcus_gobiensis
0,88
0,82
Q9RUX3_Deinococcus_radiodurans
1
E8U669_Deinococcus_maricopensis
0,99
Q7NHU2_Gloeobacter_violaceus
L0KVQ5_Methanomethylovorans_hollandica
0,95
1
W9DY55_Methanolobus_tindarius
0,97
I1EWJ0_Amphimedon_queenslandica
A0A0E3SD74_Methanosarcina_horonobensis
A0A0F8IZV3_Methanosarcina_mazei
A0A0F8LX62_Methanosarcina_mazei
0.4

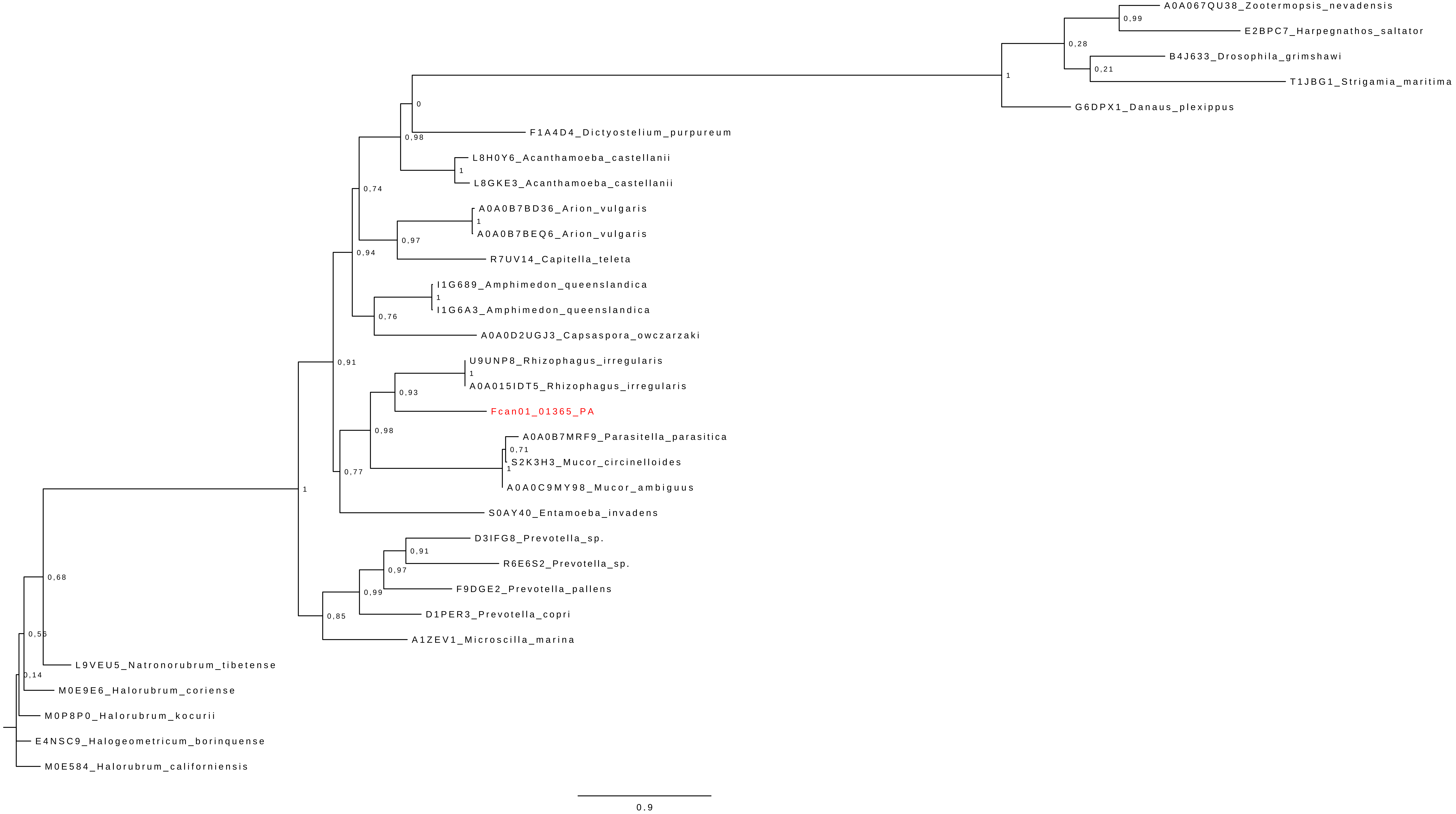
A0A067QU38_Zootermopsis_nevadensis
0,99
E2BPC7_Harpegnathos_saltator
0,28
B4J633_Drosophila_grimshawi
0,21
T1JBG1_Strigamia_maritima
1
G6DPX1_Danaus_plexippus
0
F1A4D4_Dictyostelium_purpureum
0,98
L8H0Y6_Acanthamoeba_castellanii
1
L8GKE3_Acanthamoeba_castellanii
0,74
A0A0B7BD36_Arion_vulgaris
1
A0A0B7BEQ6_Arion_vulgaris
0,97
R7UV14_Capitella_teleta
0,94
I1G689_Amphimedon_queenslandica
1
I1G6A3_Amphimedon_queenslandica
0,76
A0A0D2UGJ3_Capsaspora_owczarzaki
0,91
U9UNP8_Rhizophagus_irregularis
1
A0A015IDT5_Rhizophagus_irregularis
0,93
Fcan01_01365_PA
0,98
A0A0B7MRF9_Parasitella_parasitica
0,71
S2K3H3_Mucor_circinelloides
1
A0A0C9MY98_Mucor_ambiguus
0,77
S0AY40_Entamoeba_invadens
1
D3IFG8_Prevotella_sp.
0,91
R6E6S2_Prevotella_sp.
0,97
F9DGE2_Prevotella_pallens
0,99
D1PER3_Prevotella_copri
0,85
A1ZEV1_Microscilla_marina
0,68
0,56
L9VEU5_Natronorubrum_tibetense
0,14
M0E9E6_Halorubrum_coriense
M0P8P0_Halorubrum_kocurii
E4NSC9_Halogeometricum_borinquense
M0E584_Halorubrum_californiensis
0.9

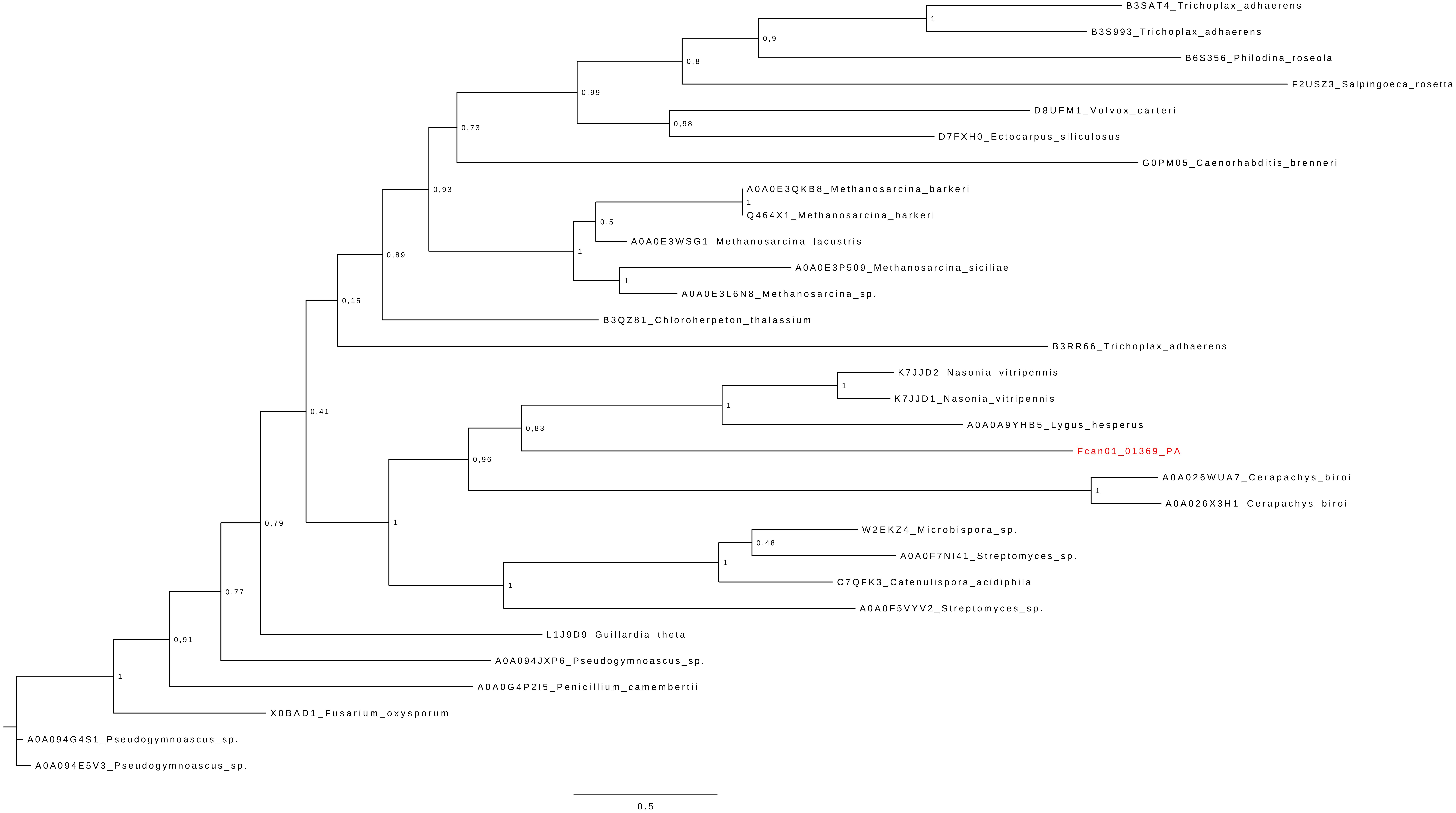
B3SAT4_Trichoplax_adhaerens
B3S993_Trichoplax_adhaerens
B6S356_Philodina_roseola
F2USZ3_Salpingoeca_rosetta
D8UFM1_Volvox_carteri
D7FXH0_Ectocarpus_siliculosus
G0PM05_Caenorhabditis_brenneri
A0A0E3QKB8_Methanosarcina_barkeri
Q464X1_Methanosarcina_barkeri
A0A0E3WSG1_Methanosarcina_lacustris
A0A0E3P509_Methanosarcina_siciliae
A0A0E3L6N8_Methanosarcina_sp.
B3QZ81_Chloroherpeton_thalassium
B3RR66_Trichoplax_adhaerens
K7JJD2_Nasonia_vitripennis
K7JJD1_Nasonia_vitripennis
A0A0A9YHB5_Lygus_hesperus
Fcan01_01369_PA
A0A026WUA7_Cerapachys_biroi
A0A026X3H1_Cerapachys_biroi
W2EKZ4_Microbispora_sp.
A0A0F7NI41_Streptomyces_sp.
C7QFK3_Catenulispora_acidiphila
A0A0F5VYV2_Streptomyces_sp.
L1J9D9_Guillardia_theta
A0A094JXP6_Pseudogymnoascus_sp.
A0A0G4P2I5_Penicillium_camembertii
X0BAD1_Fusarium_oxysporum
A0A094G4S1_Pseudogymnoascus_sp.
A0A094E5V3_Pseudogymnoascus_sp.
1
0,9
0,8
0,99
0,98
0,73
0,93
1
0,5
1
1
0,89
0,15
1
1
0,83
0,41
0,96
1
0,48
1
1
1
0,79
0,77
0,91
1
0.5

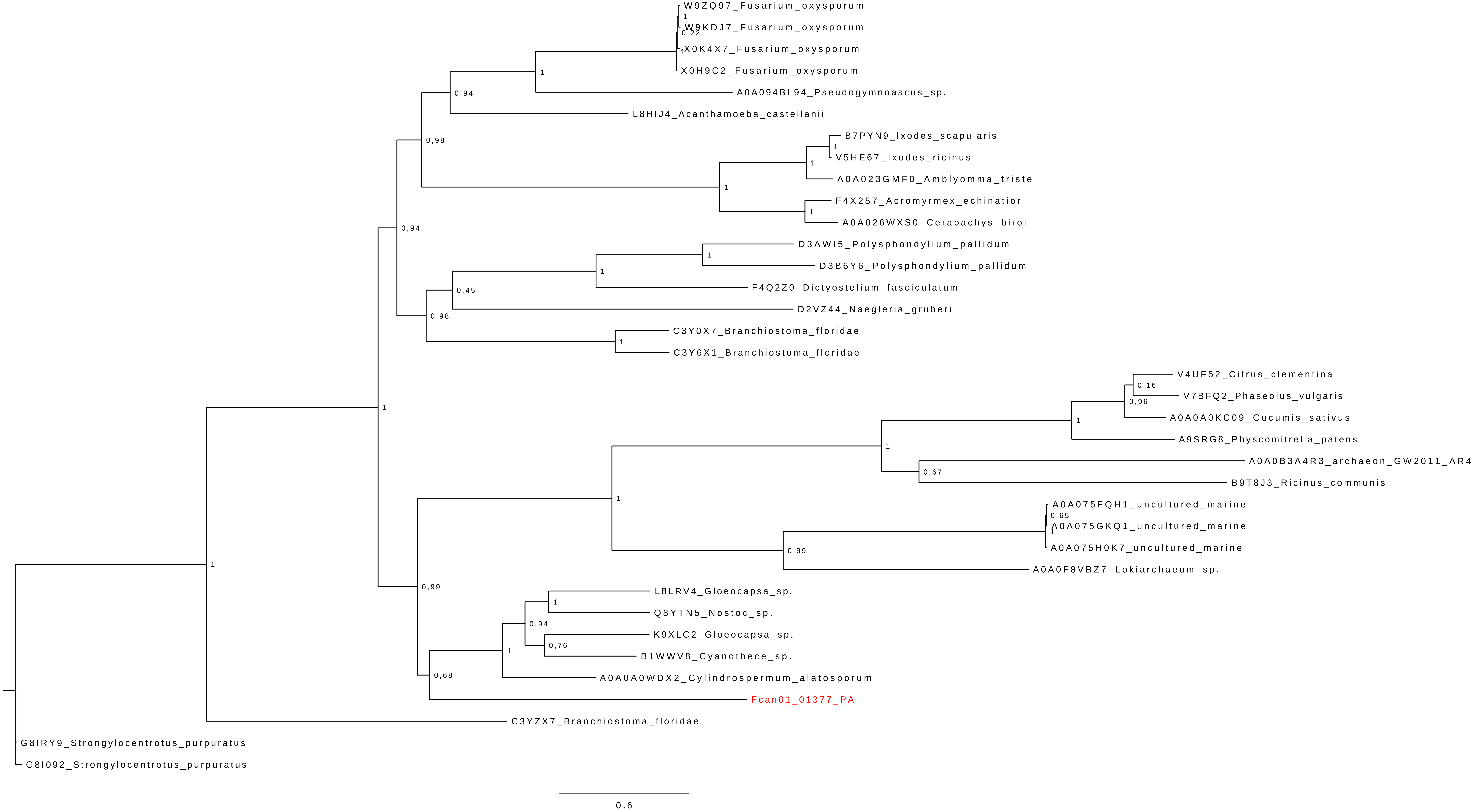
W9ZQ97_Fusarium_oxysporum
W9KDJ7_Fusarium_oxysporum
X0K4X7_Fusarium_oxysporum
X0H9C2_Fusarium_oxysporum
A0A094BL94_Pseudogymnoascus_sp.
L8HIJ4_Acanthamoeba_castellanii
B7PYN9_Ixodes_scapularis
V5HE67_Ixodes_ricinus
A0A023GMF0_Amblyomma_triste
F4X257_Acromyrmex_echinatior
A0A026WXS0_Cerapachys_biroi
D3AWI5_Polysphondylium_pallidum
D3B6Y6_Polysphondylium_pallidum
F4Q2Z0_Dictyostelium_fasciculatum
D2VZ44_Naegleria_gruberi
C3Y0X7_Branchiostoma_floridae
C3Y6X1_Branchiostoma_floridae
V4UF52_Citrus_clementina
V7BFQ2_Phaseolus_vulgaris
A0A0A0KC09_Cucumis_sativus
A9SRG8_Physcomitrella_patens
A0A0B3A4R3_archaeon_GW2011_AR4
B9T8J3_Ricinus_communis
A0A075FQH1_uncultured_marine
A0A075GKQ1_uncultured_marine
A0A075H0K7_uncultured_marine
A0A0F8VBZ7_Lokiarchaeum_sp.
L8LRV4_Gloeocapsa_sp.
Q8YTN5_Nostoc_sp.
K9XLC2_Gloeocapsa_sp.
B1WWV8_Cyanothece_sp.
A0A0A0WDX2_Cylindrospermum_alatosporum
Fcan01_01377_PA
C3YZX7_Branchiostoma_floridae
G8IRY9_Strongylocentrotus_purpuratus
G8I092_Strongylocentrotus_purpuratus
1
0,22
1
1
0,94
0,98
1
1
1
1
0,94
1
1
0,45
0,98
1
1
0,16
0,96
1
1
0,67
1
0,65
1
0,99
1
0,99
1
0,94
0,76
1
0,68
0.6

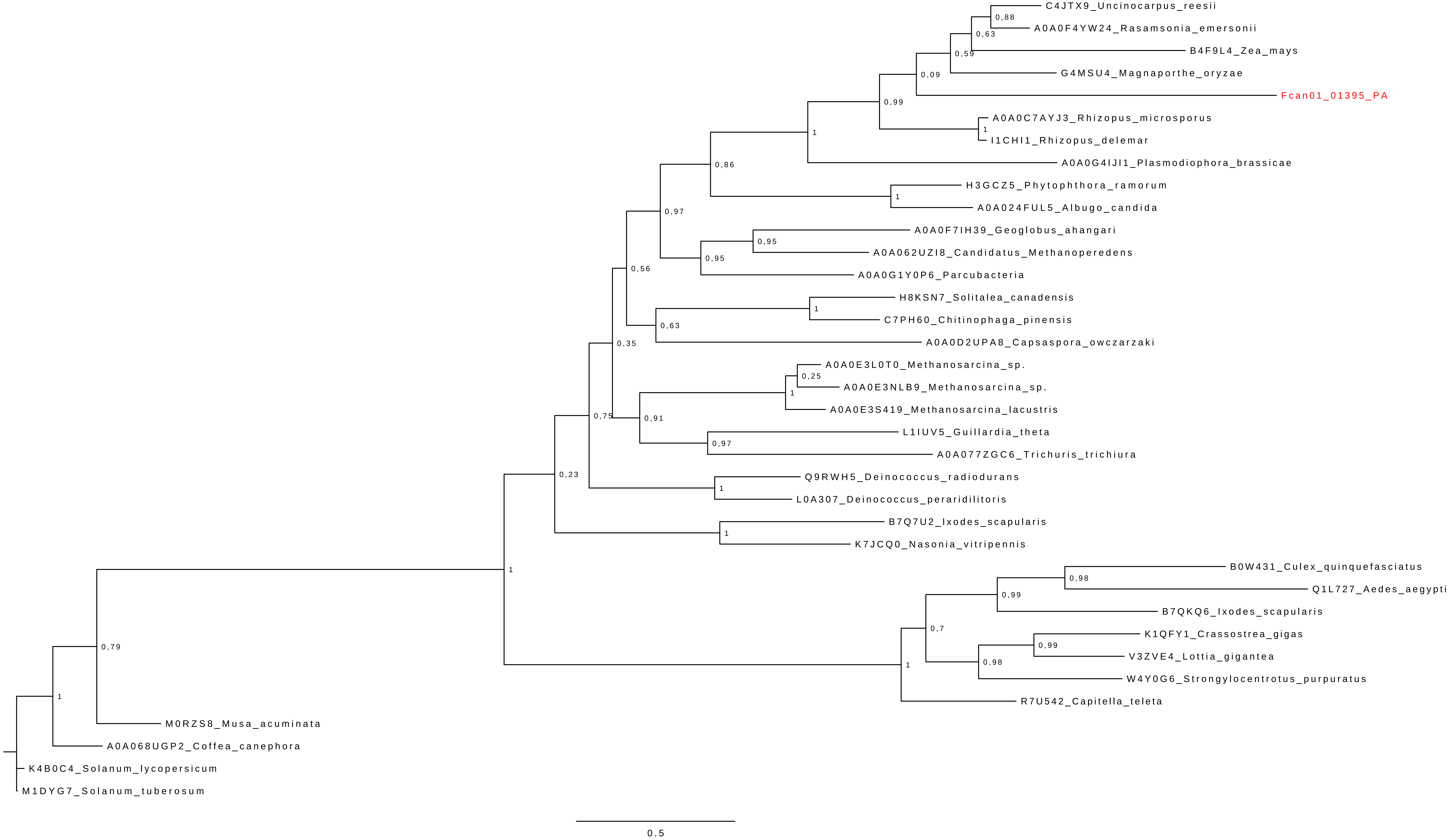
C4JTX9_Uncinocarpus_reesii
A0A0F4YW24_Rasamsonia_emersonii
B4F9L4_Zea_mays
G4MSU4_Magnaporthe_oryzae
Fcan01_01395_PA
A0A0C7AYJ3_Rhizopus_microsporus
I1CHI1_Rhizopus_delemar
A0A0G4IJI1_Plasmodiophora_brassicae
H3GCZ5_Phytophthora_ramorum
A0A024FUL5_Albugo_candida
A0A0F7IH39_Geoglobus_ahangari
A0A062UZI8_Candidatus_Methanoperedens
A0A0G1Y0P6_Parcubacteria
H8KSN7_Solitalea_canadensis
C7PH60_Chitinophaga_pinensis
A0A0D2UPA8_Capsaspora_owczarzaki
A0A0E3L0T0_Methanosarcina_sp.
A0A0E3NLB9_Methanosarcina_sp.
A0A0E3S419_Methanosarcina_lacustris
L1IUV5_Guillardia_theta
A0A077ZGC6_Trichuris_trichiura
Q9RWH5_Deinococcus_radiodurans
L0A307_Deinococcus_peraridilitoris
B7Q7U2_Ixodes_scapularis
K7JCQ0_Nasonia_vitripennis
B0W431_Culex_quinquefasciatus
Q1L727_Aedes_aegypti
B7QKQ6_Ixodes_scapularis
K1QFY1_Crassostrea_gigas
V3ZVE4_Lottia_gigantea
W4Y0G6_Strongylocentrotus_purpuratus
R7U542_Capitella_teleta
M0RZS8_Musa_acuminata
A0A068UGP2_Coffea_canephora
K4B0C4_Solanum_lycopersicum
M1DYG7_Solanum_tuberosum
0,88
0,63
0,59
0,09
0,99
1
1
0,86
1
0,97
0,95
0,95
0,56
1
0,63
0,35
0,25
1
0,75
0,91
0,97
0,23
1
1
1
0,98
0,99
0,7
0,99
0,98
1
0,79
1
0.5

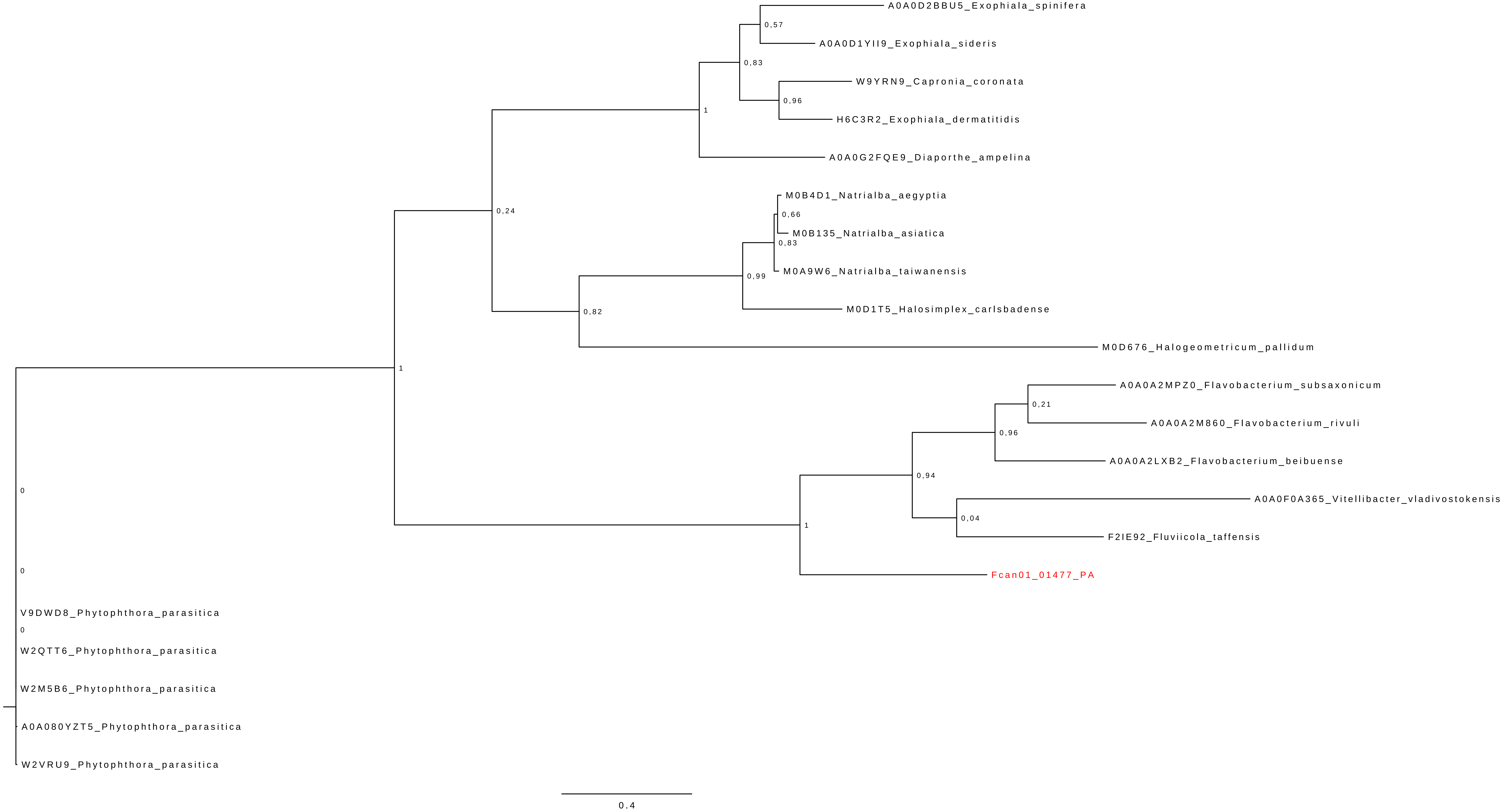
A0A0D2BBU5_Exophiala_spinifera
0,57
A0A0D1YII9_Exophiala_sideris
0,83
W9YRN9_Capronia_coronata
0,96
H6C3R2_Exophiala_dermatitidis
1
A0A0G2FQE9_Diaporthe_ampelina
M0B4D1_Natrialba_aegyptia
0,66
M0B135_Natrialba_asiatica
0,83
0,24
M0A9W6_Natrialba_taiwanensis
0,99
M0D1T5_Halosimplex_carlsbadense
0,82
M0D676_Halogeometricum_pallidum
1
A0A0A2MPZ0_Flavobacterium_subsaxonicum
0,21
A0A0A2M860_Flavobacterium_rivuli
0,96
A0A0A2LXB2_Flavobacterium_beibuense
0,94
A0A0F0A365_Vitellibacter_vladivostokensis
0,04
F2IE92_Fluviicola_taffensis
1
Fcan01_01477_PA
0
0
V9DWD8_Phytophthora_parasitica
0
W2QTT6_Phytophthora_parasitica
W2M5B6_Phytophthora_parasitica
A0A080YZT5_Phytophthora_parasitica
W2VRU9_Phytophthora_parasitica
0.4

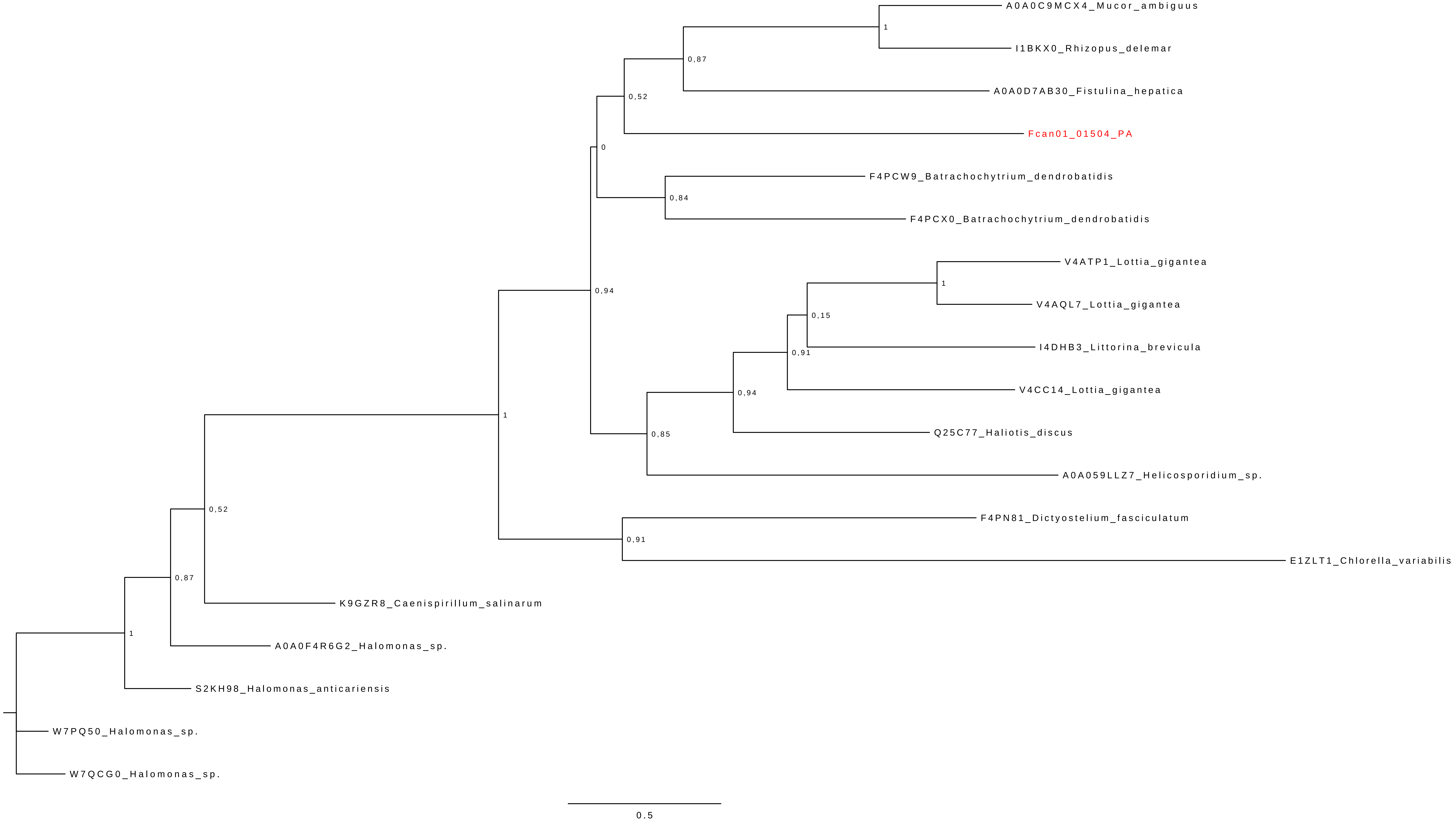
A0A0C9MCX4_Mucor_ambiguus
I1BKX0_Rhizopus_delemar
A0A0D7AB30_Fistulina_hepatica
Fcan01_01504_PA
F4PCW9_Batrachochytrium_dendrobatidis
F4PCX0_Batrachochytrium_dendrobatidis
V4ATP1_Lottia_gigantea
V4AQL7_Lottia_gigantea
I4DHB3_Littorina_brevicula
V4CC14_Lottia_gigantea
Q25C77_Haliotis_discus
A0A059LLZ7_Helicosporidium_sp.
F4PN81_Dictyostelium_fasciculatum
E1ZLT1_Chlorella_variabilis
K9GZR8_Caenispirillum_salinarum
A0A0F4R6G2_Halomonas_sp.
S2KH98_Halomonas_anticariensis
W7PQ50_Halomonas_sp.
W7QCG0_Halomonas_sp.
1
0,87
0,52
0
0,84
0,94
1
0,15
0,91
0,94
0,85
1
0,91
0,52
0,87
1
0.5

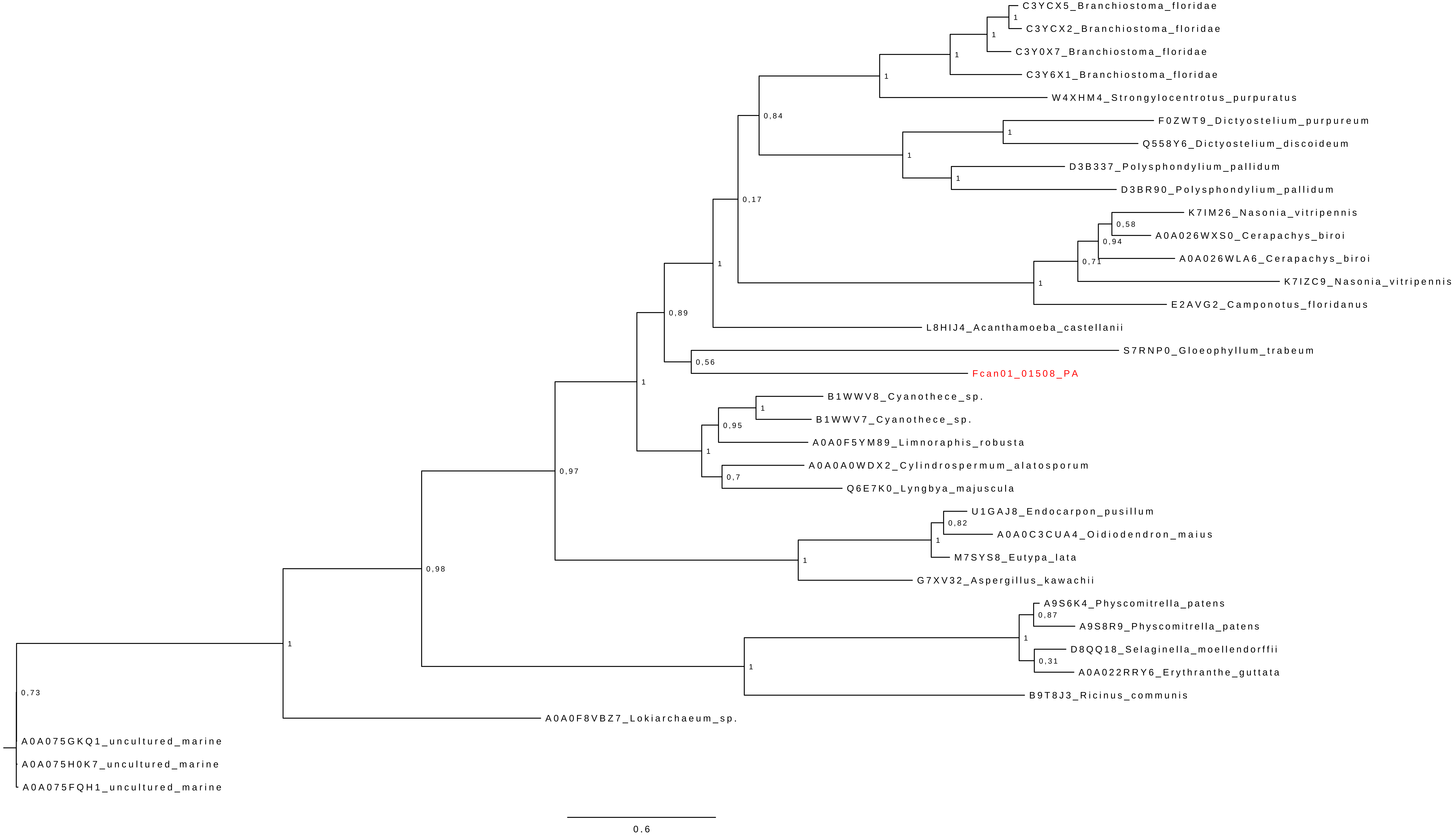
C3YCX5_Branchiostoma_floridae
C3YCX2_Branchiostoma_floridae
C3Y0X7_Branchiostoma_floridae
C3Y6X1_Branchiostoma_floridae
W4XHM4_Strongylocentrotus_purpuratus
F0ZWT9_Dictyostelium_purpureum
Q558Y6_Dictyostelium_discoideum
D3B337_Polysphondylium_pallidum
D3BR90_Polysphondylium_pallidum
K7IM26_Nasonia_vitripennis
A0A026WXS0_Cerapachys_biroi
A0A026WLA6_Cerapachys_biroi
K7IZC9_Nasonia_vitripennis
E2AVG2_Camponotus_floridanus
L8HIJ4_Acanthamoeba_castellanii
S7RNP0_Gloeophyllum_trabeum
Fcan01_01508_PA
B1WWV8_Cyanothece_sp.
B1WWV7_Cyanothece_sp.
A0A0F5YM89_Limnoraphis_robusta
A0A0A0WDX2_Cylindrospermum_alatosporum
Q6E7K0_Lyngbya_majuscula
U1GAJ8_Endocarpon_pusillum
A0A0C3CUA4_Oidiodendron_maius
M7SYS8_Eutypa_lata
G7XV32_Aspergillus_kawachii
A9S6K4_Physcomitrella_patens
A9S8R9_Physcomitrella_patens
D8QQ18_Selaginella_moellendorffii
A0A022RRY6_Erythranthe_guttata
B9T8J3_Ricinus_communis
A0A0F8VBZ7_Lokiarchaeum_sp.
A0A075GKQ1_uncultured_marine
A0A075H0K7_uncultured_marine
A0A075FQH1_uncultured_marine
0.6

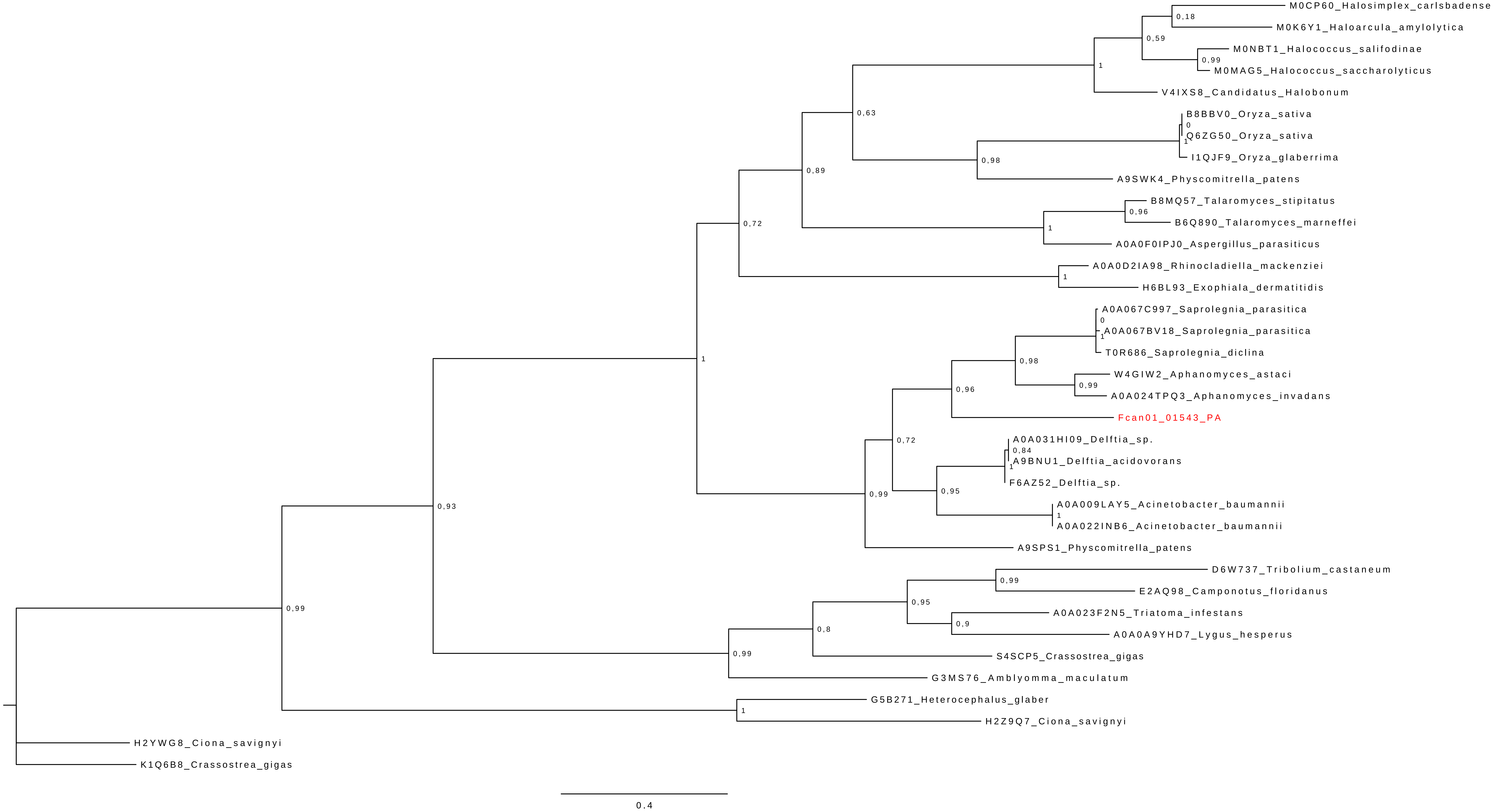
M0CP60_Halosimplex_carlsbadense
0,18
M0K6Y1_Haloarcula_amylolytica
0,59
M0NBT1_Halococcus_salifodinae
0,99
M0MAG5_Halococcus_saccharolyticus
1
V4IXS8_Candidatus_Halobonum
0,63
B8BBV0_Oryza_sativa
0
Q6ZG50_Oryza_sativa
1
I1QJF9_Oryza_glaberrima
0,98
A9SWK4_Physcomitrella_patens
0,89
B8MQ57_Talaromyces_stipitatus
0,96
B6Q890_Talaromyces_marneffei
1
A0A0F0IPJ0_Aspergillus_parasiticus
0,72
A0A0D2IA98_Rhinocladiella_mackenziei
1
H6BL93_Exophiala_dermatitidis
A0A067C997_Saprolegnia_parasitica
0
A0A067BV18_Saprolegnia_parasitica
1
T0R686_Saprolegnia_diclina
0,98
W4GIW2_Aphanomyces_astaci
0,99
A0A024TPQ3_Aphanomyces_invadans
0,96
Fcan01_01543_PA
1
A0A031HI09_Delftia_sp.
0,84
A9BNU1_Delftia_acidovorans
1
F6AZ52_Delftia_sp.
0,72
0,95
A0A009LAY5_Acinetobacter_baumannii
1
A0A022INB6_Acinetobacter_baumannii
0,99
A9SPS1_Physcomitrella_patens
0,93
D6W737_Tribolium_castaneum
0,99
E2AQ98_Camponotus_floridanus
0,95
A0A023F2N5_Triatoma_infestans
0,9
A0A0A9YHD7_Lygus_hesperus
0,8
S4SCP5_Crassostrea_gigas
0,99
0,99
G3MS76_Amblyomma_maculatum
G5B271_Heterocephalus_glaber
1
H2Z9Q7_Ciona_savignyi
H2YWG8_Ciona_savignyi
K1Q6B8_Crassostrea_gigas
0.4

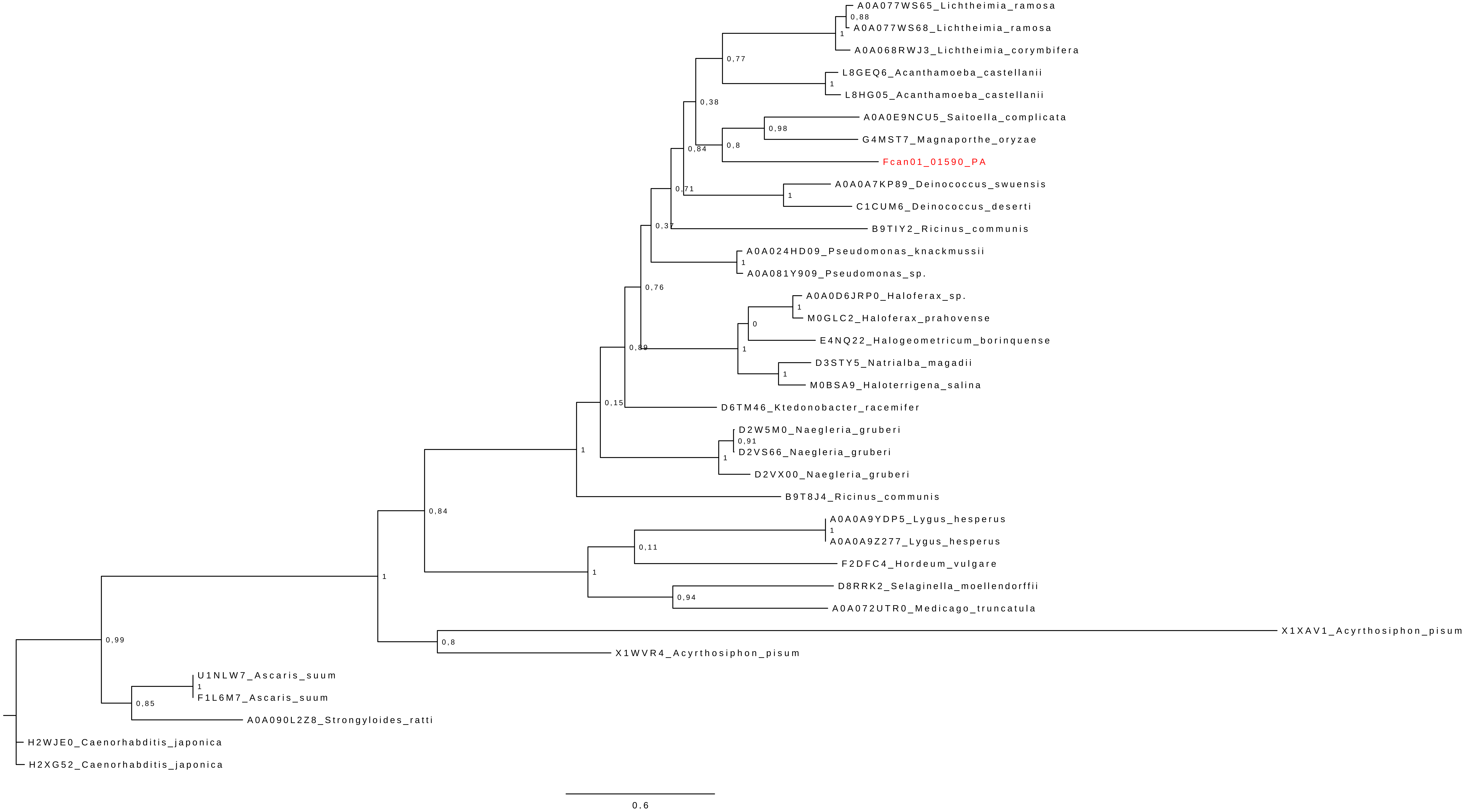
A0A077WS65_Lichtheimia_ramosa
0,88
A0A077WS68_Lichtheimia_ramosa
1
A0A068RWJ3_Lichtheimia_corymbifera
0,77
L8GEQ6_Acanthamoeba_castellanii
1
L8HG05_Acanthamoeba_castellanii
0,38
A0A0E9NCU5_Saitoella_complicata
0,98
G4MST7_Magnaporthe_oryzae
0,84
0,8
Fcan01_01590_PA
A0A0A7KP89_Deinococcus_swuensis
0,71
1
C1CUM6_Deinococcus_deserti
0,37
B9TIY2_Ricinus_communis
A0A024HD09_Pseudomonas_knackmussii
1
A0A081Y909_Pseudomonas_sp.
0,76
A0A0D6JRP0_Haloferax_sp.
1
M0GLC2_Haloferax_prahovense
0
E4NQ22_Halogeometricum_borinquense
0,89
1
D3STY5_Natrialba_magadii
1
M0BSA9_Haloterrigena_salina
0,15
D6TM46_Ktedonobacter_racemifer
D2W5M0_Naegleria_gruberi
0,91
D2VS66_Naegleria_gruberi
1
1
D2VX00_Naegleria_gruberi
B9T8J4_Ricinus_communis
0,84
A0A0A9YDP5_Lygus_hesperus
1
A0A0A9Z277_Lygus_hesperus
0,11
F2DFC4_Hordeum_vulgare
1
1
D8RRK2_Selaginella_moellendorffii
0,94
A0A072UTR0_Medicago_truncatula
X1XAV1_Acyrthosiphon_pisum
0,99
0,8
X1WVR4_Acyrthosiphon_pisum
U1NLW7_Ascaris_suum
1
F1L6M7_Ascaris_suum
0,85
A0A090L2Z8_Strongyloides_ratti
H2WJE0_Caenorhabditis_japonica
H2XG52_Caenorhabditis_japonica
0.6

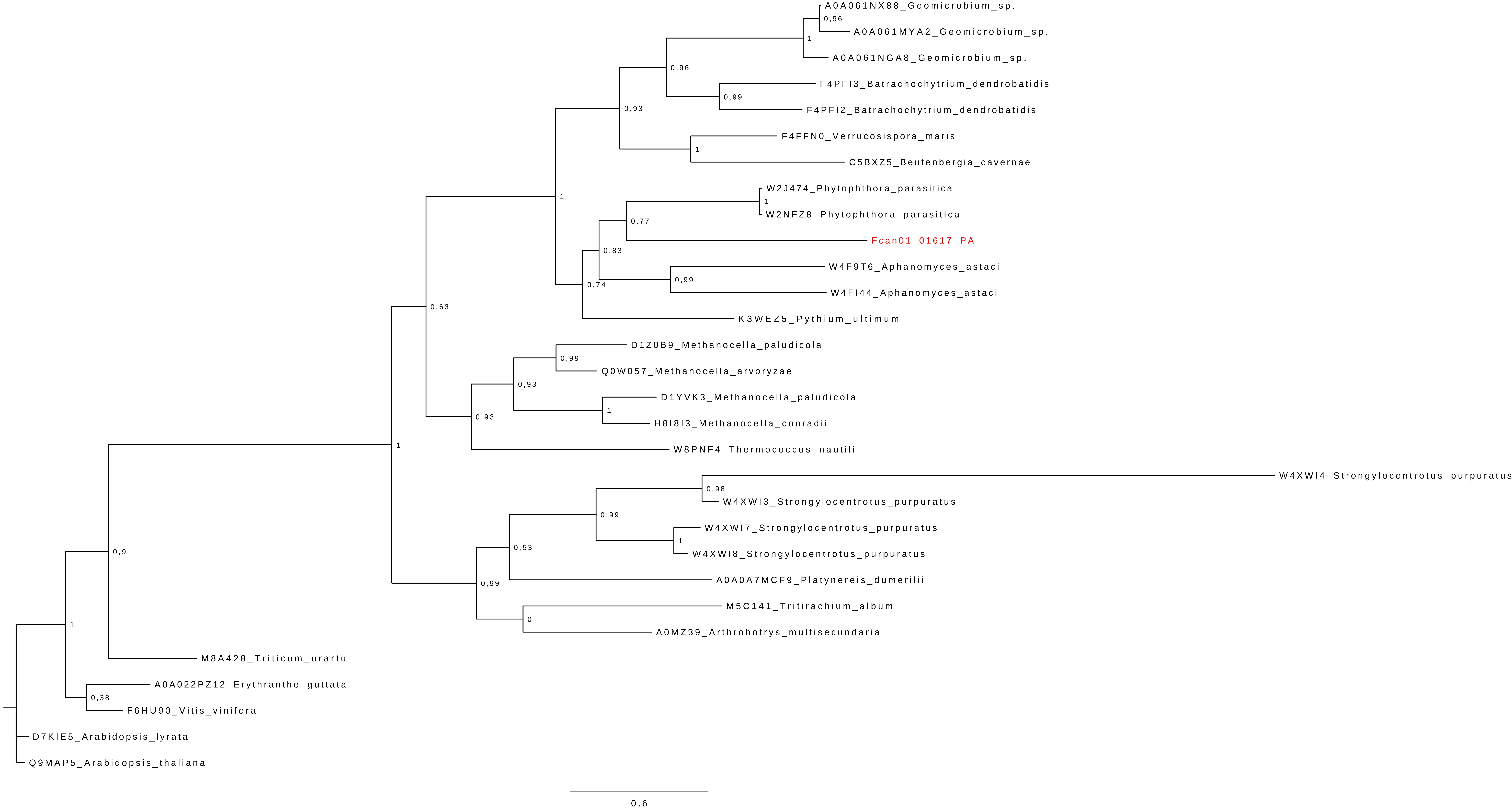
A0A061NX88_Geomicrobium_sp.
0,96
A0A061MYA2_Geomicrobium_sp.
1
A0A061NGA8_Geomicrobium_sp.
0,96
F4PFI3_Batrachochytrium_dendrobatidis
0,99
F4PFI2_Batrachochytrium_dendrobatidis
0,93
F4FFN0_Verrucosispora_maris
1
C5BXZ5_Beutenbergia_cavernae
W2J474_Phytophthora_parasitica
1
1
W2NFZ8_Phytophthora_parasitica
0,77
Fcan01_01617_PA
0,83
W4F9T6_Aphanomyces_astaci
0,99
0,74
W4FI44_Aphanomyces_astaci
0,63
K3WEZ5_Pythium_ultimum
D1Z0B9_Methanocella_paludicola
0,99
Q0W057_Methanocella_arvoryzae
0,93
D1YVK3_Methanocella_paludicola
1
0,93
H8I8I3_Methanocella_conradii
1
W8PNF4_Thermococcus_nautili
W4XWI4_Strongylocentrotus_purpuratus
0,98
W4XWI3_Strongylocentrotus_purpuratus
0,99
W4XWI7_Strongylocentrotus_purpuratus
1
0,9
0,53
W4XWI8_Strongylocentrotus_purpuratus
A0A0A7MCF9_Platynereis_dumerilii
0,99
M5C141_Tritirachium_album
1
0
A0MZ39_Arthrobotrys_multisecundaria
M8A428_Triticum_urartu
A0A022PZ12_Erythranthe_guttata
0,38
F6HU90_Vitis_vinifera
D7KIE5_Arabidopsis_lyrata
Q9MAP5_Arabidopsis_thaliana
0.6

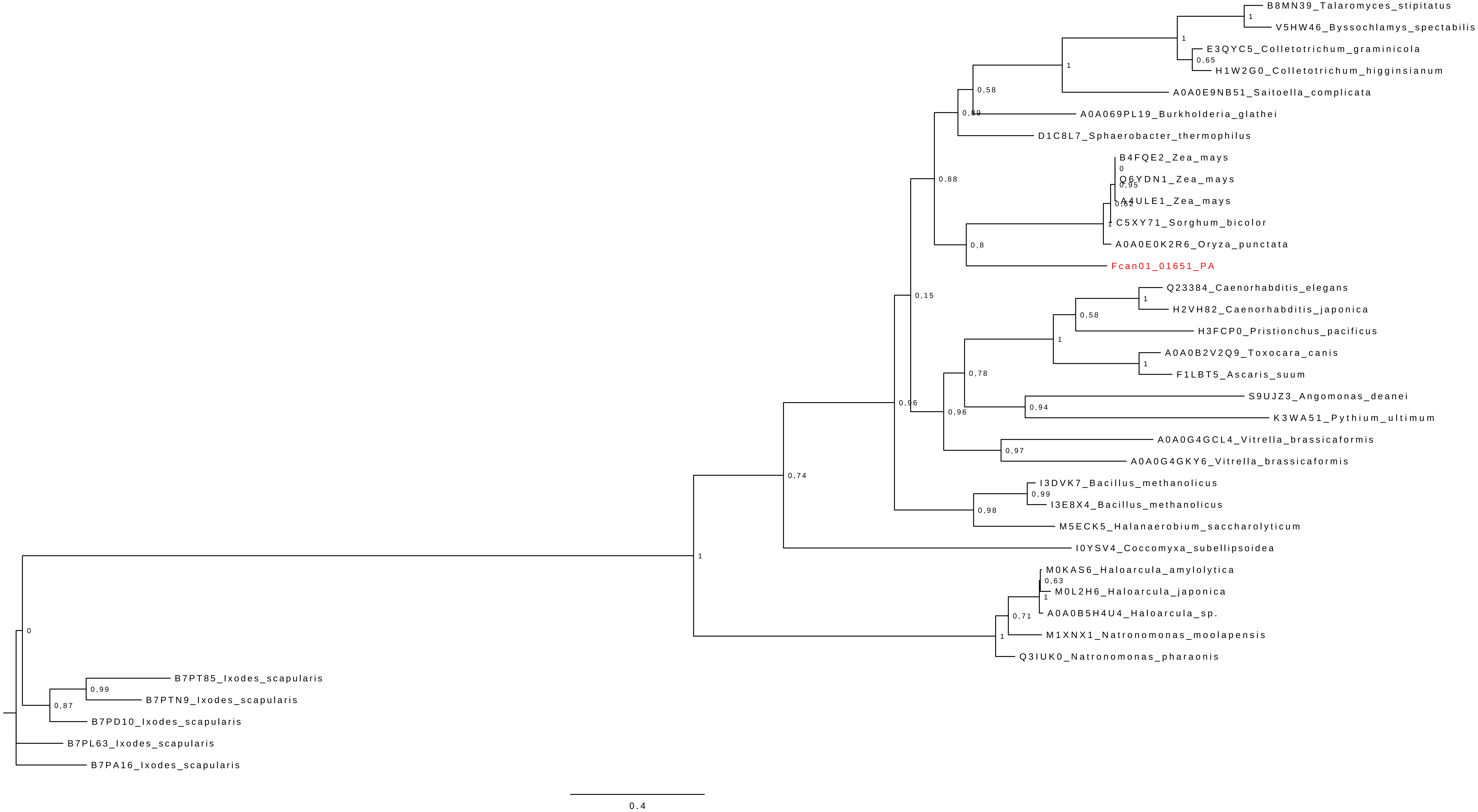
B8MN39_Talaromyces_stipitatus
V5HW46_Byssochlamys_spectabilis
E3QYC5_Colletotrichum_graminicola
H1W2G0_Colletotrichum_higginsianum
A0A0E9NB51_Saitoella_complicata
A0A069PL19_Burkholderia_glathei
D1C8L7_Sphaerobacter_thermophilus
B4FQE2_Zea_mays
Q6YDN1_Zea_mays
A4ULE1_Zea_mays
C5XY71_Sorghum_bicolor
A0A0E0K2R6_Oryza_punctata
Fcan01_01651_PA
Q23384_Caenorhabditis_elegans
H2VH82_Caenorhabditis_japonica
H3FCP0_Pristionchus_pacificus
A0A0B2V2Q9_Toxocara_canis
F1LBT5_Ascaris_suum
S9UJZ3_Angomonas_deanei
K3WA51_Pythium_ultimum
A0A0G4GCL4_Vitrella_brassicaformis
A0A0G4GKY6_Vitrella_brassicaformis
I3DVK7_Bacillus_methanolicus
I3E8X4_Bacillus_methanolicus
M5ECK5_Halanaerobium_saccharolyticum
I0YSV4_Coccomyxa_subellipsoidea
M0KAS6_Haloarcula_amylolytica
M0L2H6_Haloarcula_japonica
A0A0B5H4U4_Haloarcula_sp.
M1XNX1_Natronomonas_moolapensis
Q3IUK0_Natronomonas_pharaonis
B7PT85_Ixodes_scapularis
B7PTN9_Ixodes_scapularis
B7PD10_Ixodes_scapularis
B7PL63_Ixodes_scapularis
B7PA16_Ixodes_scapularis
0.4

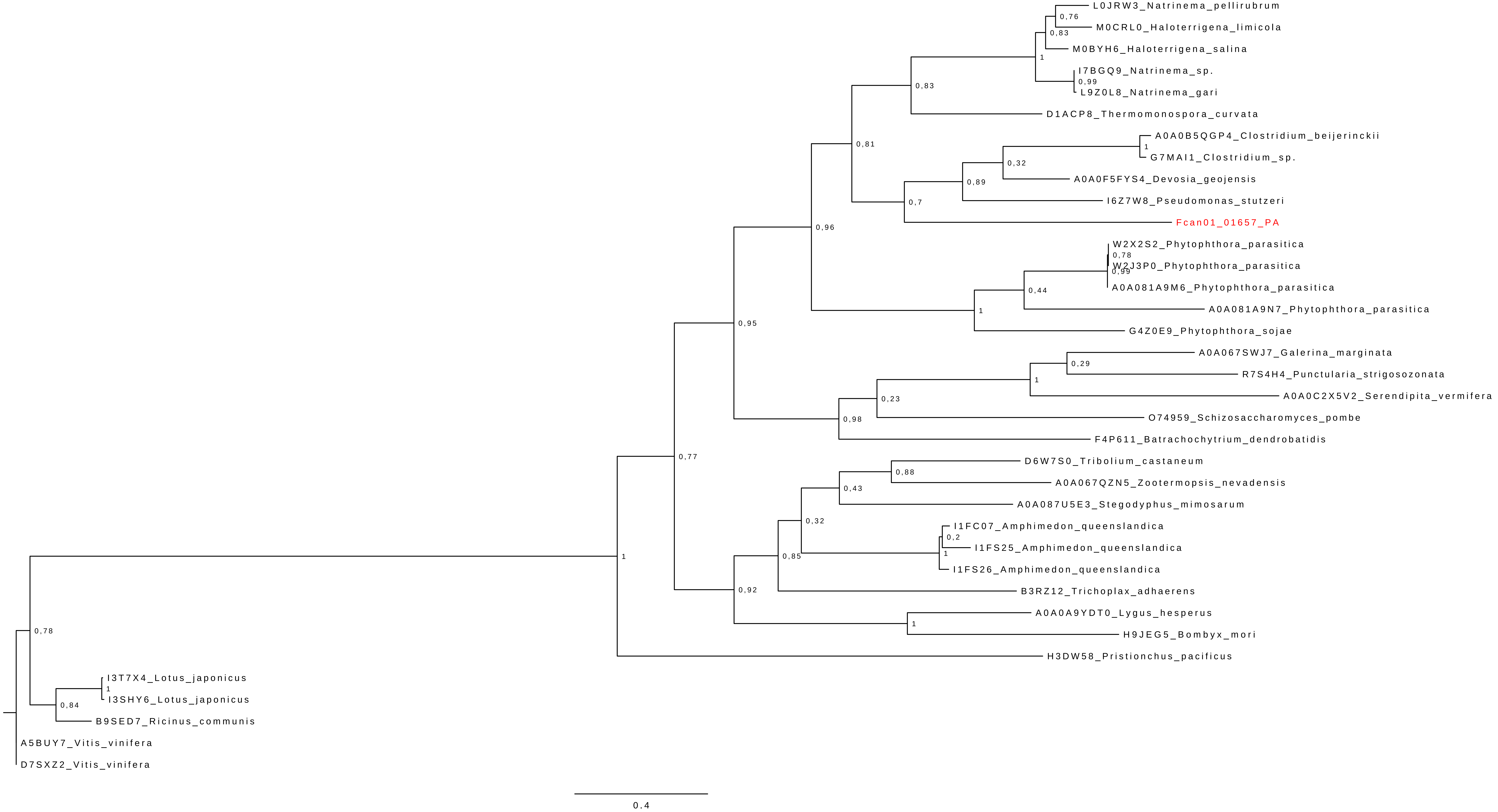
L0JRW3_Natrinema_pellirubrum
0,76
M0CRL0_Haloterrigena_limicola
0,83
M0BYH6_Haloterrigena_salina
1
I7BGQ9_Natrinema_sp.
0,99
L9Z0L8_Natrinema_gari
0,83
D1ACP8_Thermomonospora_curvata
A0A0B5QGP4_Clostridium_beijerinckii
0,81
1
G7MAI1_Clostridium_sp.
0,32
A0A0F5FYS4_Devosia_geojensis
0,89
I6Z7W8_Pseudomonas_stutzeri
0,7
Fcan01_01657_PA
0,96
W2X2S2_Phytophthora_parasitica
0,78
W2J3P0_Phytophthora_parasitica
0,99
A0A081A9M6_Phytophthora_parasitica
0,44
A0A081A9N7_Phytophthora_parasitica
1
G4Z0E9_Phytophthora_sojae
0,95
A0A067SWJ7_Galerina_marginata
0,29
R7S4H4_Punctularia_strigosozonata
1
A0A0C2X5V2_Serendipita_vermifera
0,23
O74959_Schizosaccharomyces_pombe
0,98
F4P611_Batrachochytrium_dendrobatidis
0,77
D6W7S0_Tribolium_castaneum
0,88
A0A067QZN5_Zootermopsis_nevadensis
0,43
A0A087U5E3_Stegodyphus_mimosarum
0,32
I1FC07_Amphimedon_queenslandica
0,2
I1FS25_Amphimedon_queenslandica
1
0,85
I1FS26_Amphimedon_queenslandica
B3RZ12_Trichoplax_adhaerens
0,92
A0A0A9YDT0_Lygus_hesperus
1
H9JEG5_Bombyx_mori
H3DW58_Pristionchus_pacificus
0,78
I3T7X4_Lotus_japonicus
1
I3SHY6_Lotus_japonicus
0,84
B9SED7_Ricinus_communis
A5BUY7_Vitis_vinifera
D7SXZ2_Vitis_vinifera
0.4

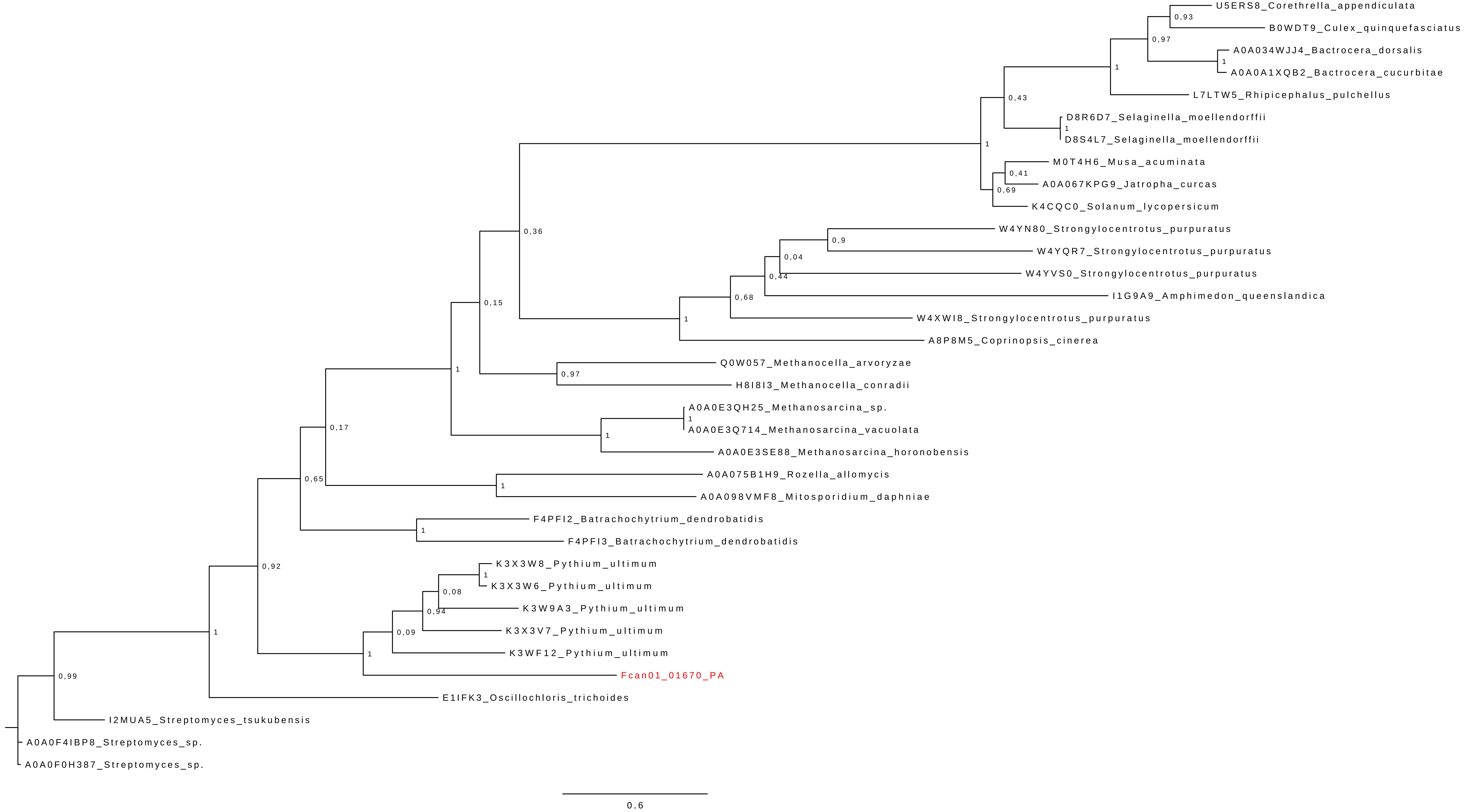
U5ERS8_Corethrella_appendiculata
0,93
B0WDT9_Culex_quinquefasciatus
0,97
A0A034WJJ4_Bactrocera_dorsalis
1
A0A0A1XQB2_Bactrocera_cucurbitae
1
L7LTW5_Rhipicephalus_pulchellus
0,43
D8R6D7_Selaginella_moellendorffii
1
D8S4L7_Selaginella_moellendorffii
1
M0T4H6_Musa_acuminata
0,41
A0A067KPG9_Jatropha_curcas
0,69
K4CQC0_Solanum_lycopersicum
W4YN80_Strongylocentrotus_purpuratus
0,36
0,9
W4YQR7_Strongylocentrotus_purpuratus
0,04
W4YVS0_Strongylocentrotus_purpuratus
0,44
I1G9A9_Amphimedon_queenslandica
0,68
0,15
W4XWI8_Strongylocentrotus_purpuratus
1
A8P8M5_Coprinopsis_cinerea
Q0W057_Methanocella_arvoryzae
1
0,97
H8I8I3_Methanocella_conradii
A0A0E3QH25_Methanosarcina_sp.
1
A0A0E3Q714_Methanosarcina_vacuolata
0,17
1
A0A0E3SE88_Methanosarcina_horonobensis
A0A075B1H9_Rozella_allomycis
0,65
1
A0A098VMF8_Mitosporidium_daphniae
F4PFI2_Batrachochytrium_dendrobatidis
1
F4PFI3_Batrachochytrium_dendrobatidis
K3X3W8_Pythium_ultimum
0,92
1
K3X3W6_Pythium_ultimum
0,08
K3W9A3_Pythium_ultimum
0,94
K3X3V7_Pythium_ultimum
0,09
1
K3WF12_Pythium_ultimum
1
Fcan01_01670_PA
0,99
E1IFK3_Oscillochloris_trichoides
I2MUA5_Streptomyces_tsukubensis
A0A0F4IBP8_Streptomyces_sp.
A0A0F0H387_Streptomyces_sp.
0.6

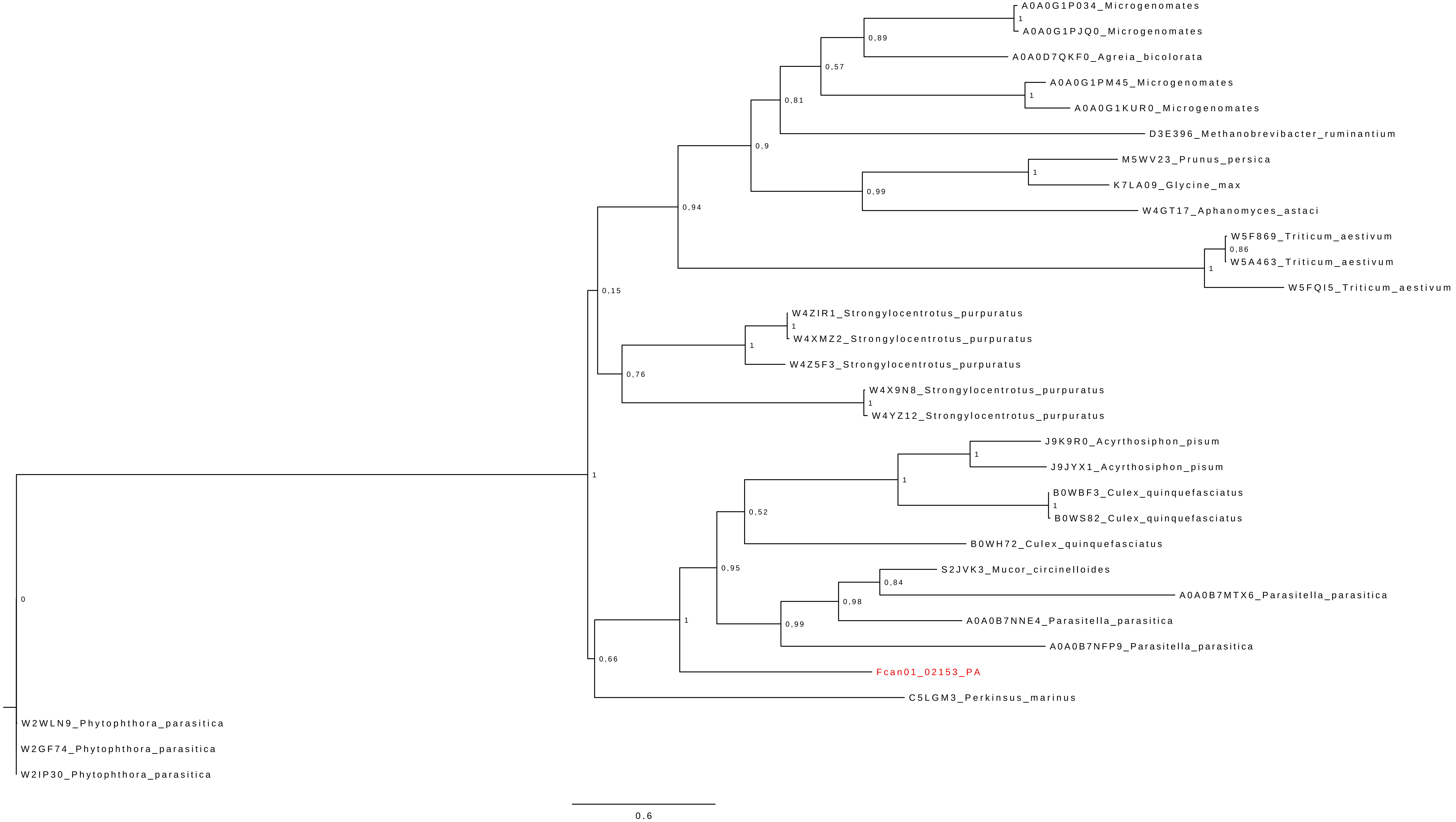
A0A0G1P034_Microgenomates
A0A0G1PJQ0_Microgenomates
A0A0D7QKF0_Agreia_bicolorata
A0A0G1PM45_Microgenomates
A0A0G1KUR0_Microgenomates
D3E396_Methanobrevibacter_ruminantium
M5WV23_Prunus_persica
K7LA09_Glycine_max
W4GT17_Aphanomyces_astaci
W5F869_Triticum_aestivum
W5A463_Triticum_aestivum
W5FQI5_Triticum_aestivum
W4ZIR1_Strongylocentrotus_purpuratus
W4XMZ2_Strongylocentrotus_purpuratus
W4Z5F3_Strongylocentrotus_purpuratus
W4X9N8_Strongylocentrotus_purpuratus
W4YZ12_Strongylocentrotus_purpuratus
J9K9R0_Acyrthosiphon_pisum
J9JYX1_Acyrthosiphon_pisum
B0WBF3_Culex_quinquefasciatus
B0WS82_Culex_quinquefasciatus
B0WH72_Culex_quinquefasciatus
S2JVK3_Mucor_circinelloides
A0A0B7MTX6_Parasitella_parasitica
A0A0B7NNE4_Parasitella_parasitica
A0A0B7NFP9_Parasitella_parasitica
Fcan01_02153_PA
C5LGM3_Perkinsus_marinus
W2WLN9_Phytophthora_parasitica
W2GF74_Phytophthora_parasitica
W2IP30_Phytophthora_parasitica
0.6

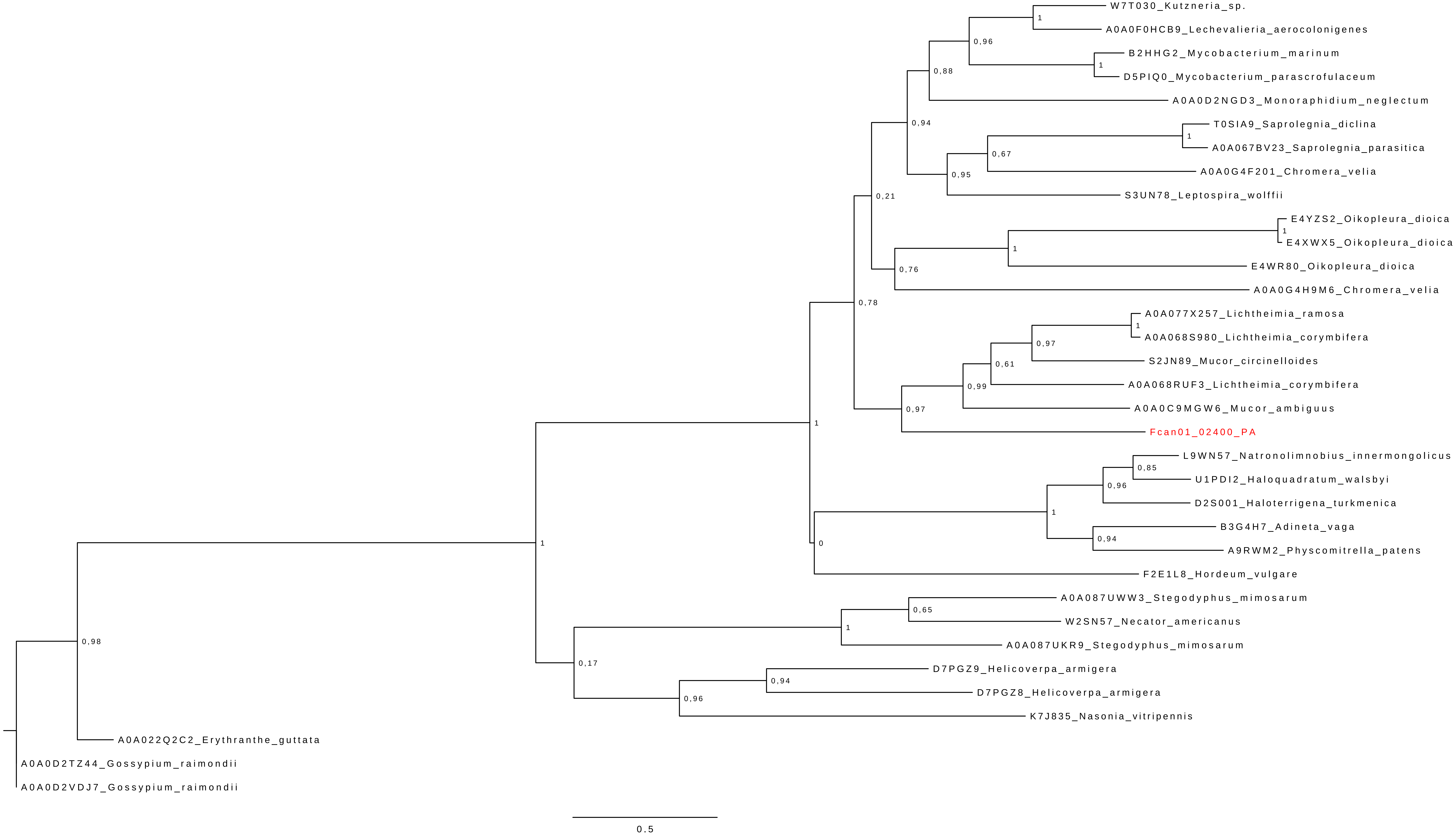
W7T030_Kutzneria_sp.
A0A0F0HCB9_Lechevalieria_aerocolonigenes
B2HHG2_Mycobacterium_marinum
D5PIQ0_Mycobacterium_parascrofulaceum
A0A0D2NGD3_Monoraphidium_neglectum
T0SIA9_Saprolegnia_diclina
A0A067BV23_Saprolegnia_parasitica
A0A0G4F201_Chromera_velia
S3UN78_Leptospira_wolffii
E4YZS2_Oikopleura_dioica
E4XWX5_Oikopleura_dioica
E4WR80_Oikopleura_dioica
A0A0G4H9M6_Chromera_velia
A0A077X257_Lichtheimia_ramosa
A0A068S980_Lichtheimia_corymbifera
S2JN89_Mucor_circinelloides
A0A068RUF3_Lichtheimia_corymbifera
A0A0C9MGW6_Mucor_ambiguus
Fcan01_02400_PA
L9WN57_Natronolimnobius_innermongolicus
U1PDI2_Haloquadratum_walsbyi
D2S001_Haloterrigena_turkmenica
B3G4H7_Adineta_vaga
A9RWM2_Physcomitrella_patens
F2E1L8_Hordeum_vulgare
A0A087UWW3_Stegodyphus_mimosarum
W2SN57_Necator_americanus
A0A087UKR9_Stegodyphus_mimosarum
D7PGZ9_Helicoverpa_armigera
D7PGZ8_Helicoverpa_armigera
K7J835_Nasonia_vitripennis
A0A022Q2C2_Erythranthe_guttata
A0A0D2TZ44_Gossypium_raimondii
A0A0D2VDJ7_Gossypium_raimondii
0.5

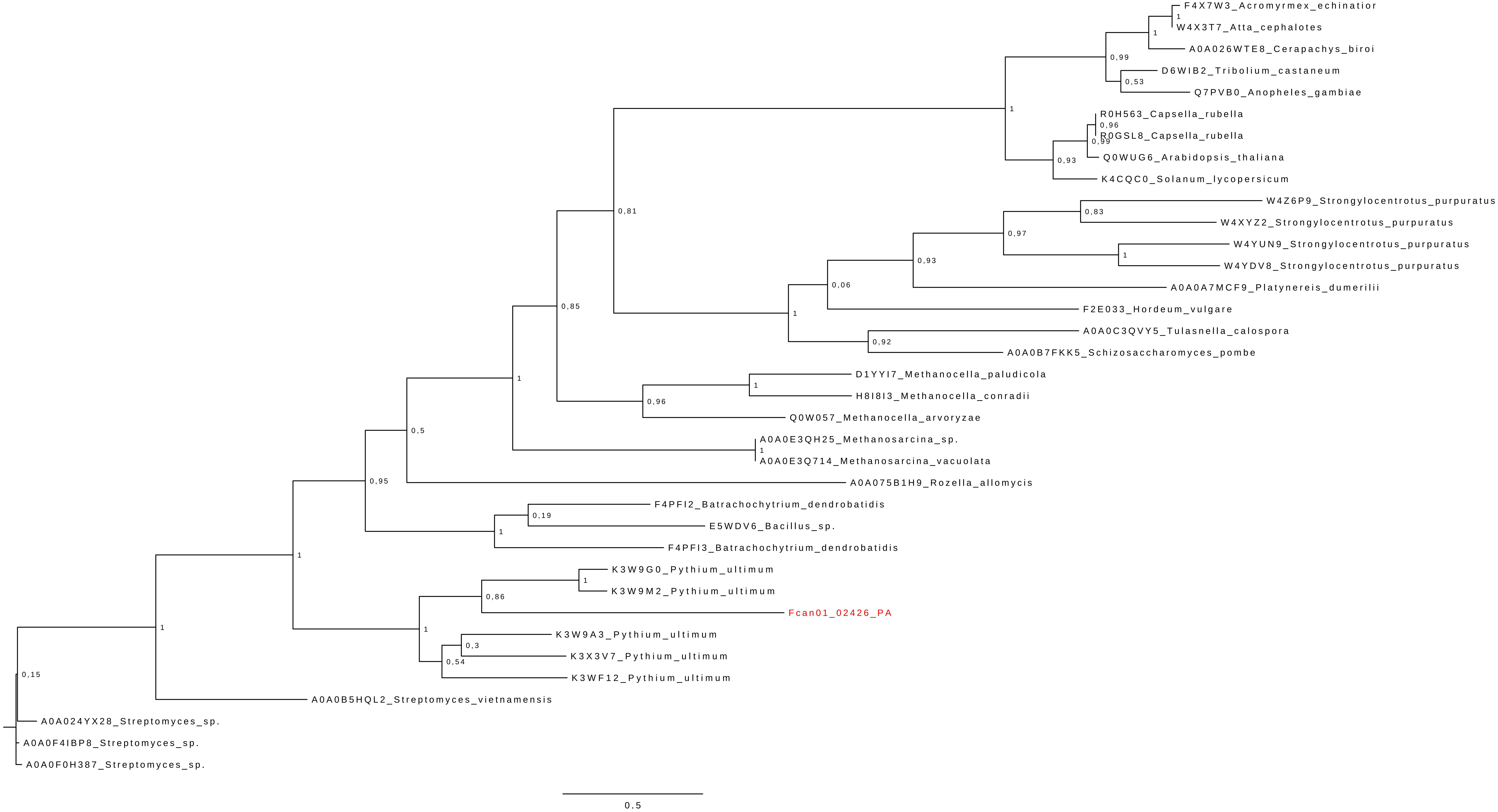
F4X7W3_Acromyrmex_echinatior
W4X3T7_Atta_cephalotes
A0A026WTE8_Cerapachys_biroi
D6WIB2_Tribolium_castaneum
Q7PVB0_Anopheles_gambiae
R0H563_Capsella_rubella
R0GSL8_Capsella_rubella
Q0WUG6_Arabidopsis_thaliana
K4CQC0_Solanum_lycopersicum
W4Z6P9_Strongylocentrotus_purpuratus
W4XYZ2_Strongylocentrotus_purpuratus
W4YUN9_Strongylocentrotus_purpuratus
W4YDV8_Strongylocentrotus_purpuratus
A0A0A7MCF9_Platynereis_dumerilii
F2E033_Hordeum_vulgare
A0A0C3QVY5_Tulasnella_calospora
A0A0B7FKK5_Schizosaccharomyces_pombe
D1YYI7_Methanocella_paludicola
H8I8I3_Methanocella_conradii
Q0W057_Methanocella_arvoryzae
A0A0E3QH25_Methanosarcina_sp.
A0A0E3Q714_Methanosarcina_vacuolata
A0A075B1H9_Rozella_allomycis
F4PFI2_Batrachochytrium_dendrobatidis
E5WDV6_Bacillus_sp.
F4PFI3_Batrachochytrium_dendrobatidis
K3W9G0_Pythium_ultimum
K3W9M2_Pythium_ultimum
Fcan01_02426_PA
K3W9A3_Pythium_ultimum
K3X3V7_Pythium_ultimum
K3WF12_Pythium_ultimum
A0A0B5HQL2_Streptomyces_vietnamensis
A0A024YX28_Streptomyces_sp.
A0A0F4IBP8_Streptomyces_sp.
A0A0F0H387_Streptomyces_sp.
0.5

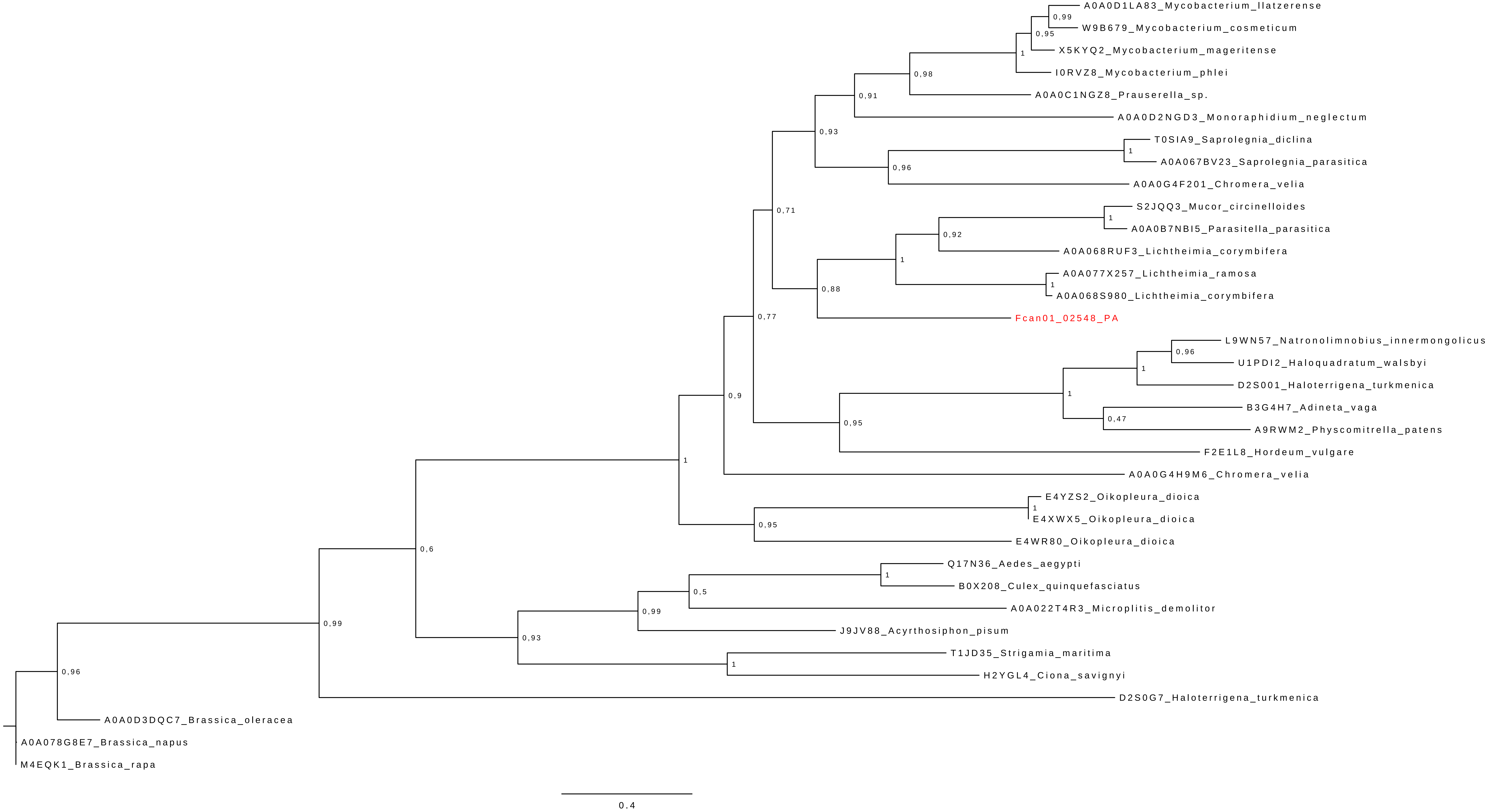
A0A0D1LA83_Mycobacterium_llatzerense
W9B679_Mycobacterium_cosmeticum
X5KYQ2_Mycobacterium_mageritense
I0RVZ8_Mycobacterium_phlei
A0A0C1NGZ8_Prauserella_sp.
A0A0D2NGD3_Monoraphidium_neglectum
T0SIA9_Saprolegnia_diclina
A0A067BV23_Saprolegnia_parasitica
A0A0G4F201_Chromera_velia
S2JQQ3_Mucor_circinelloides
A0A0B7NBI5_Parasitella_parasitica
A0A068RUF3_Lichtheimia_corymbifera
A0A077X257_Lichtheimia_ramosa
A0A068S980_Lichtheimia_corymbifera
Fcan01_02548_PA
L9WN57_Natronolimnobius_innermongolicus
U1PDI2_Haloquadratum_walsbyi
D2S001_Haloterrigena_turkmenica
B3G4H7_Adineta_vaga
A9RWM2_Physcomitrella_patens
F2E1L8_Hordeum_vulgare
A0A0G4H9M6_Chromera_velia
E4YZS2_Oikopleura_dioica
E4XWX5_Oikopleura_dioica
E4WR80_Oikopleura_dioica
Q17N36_Aedes_aegypti
B0X208_Culex_quinquefasciatus
A0A022T4R3_Microplitis_demolitor
J9JV88_Acyrthosiphon_pisum
T1JD35_Strigamia_maritima
H2YGL4_Ciona_savignyi
D2S0G7_Haloterrigena_turkmenica
A0A0D3DQC7_Brassica_oleracea
A0A078G8E7_Brassica_napus
M4EQK1_Brassica_rapa
0.4

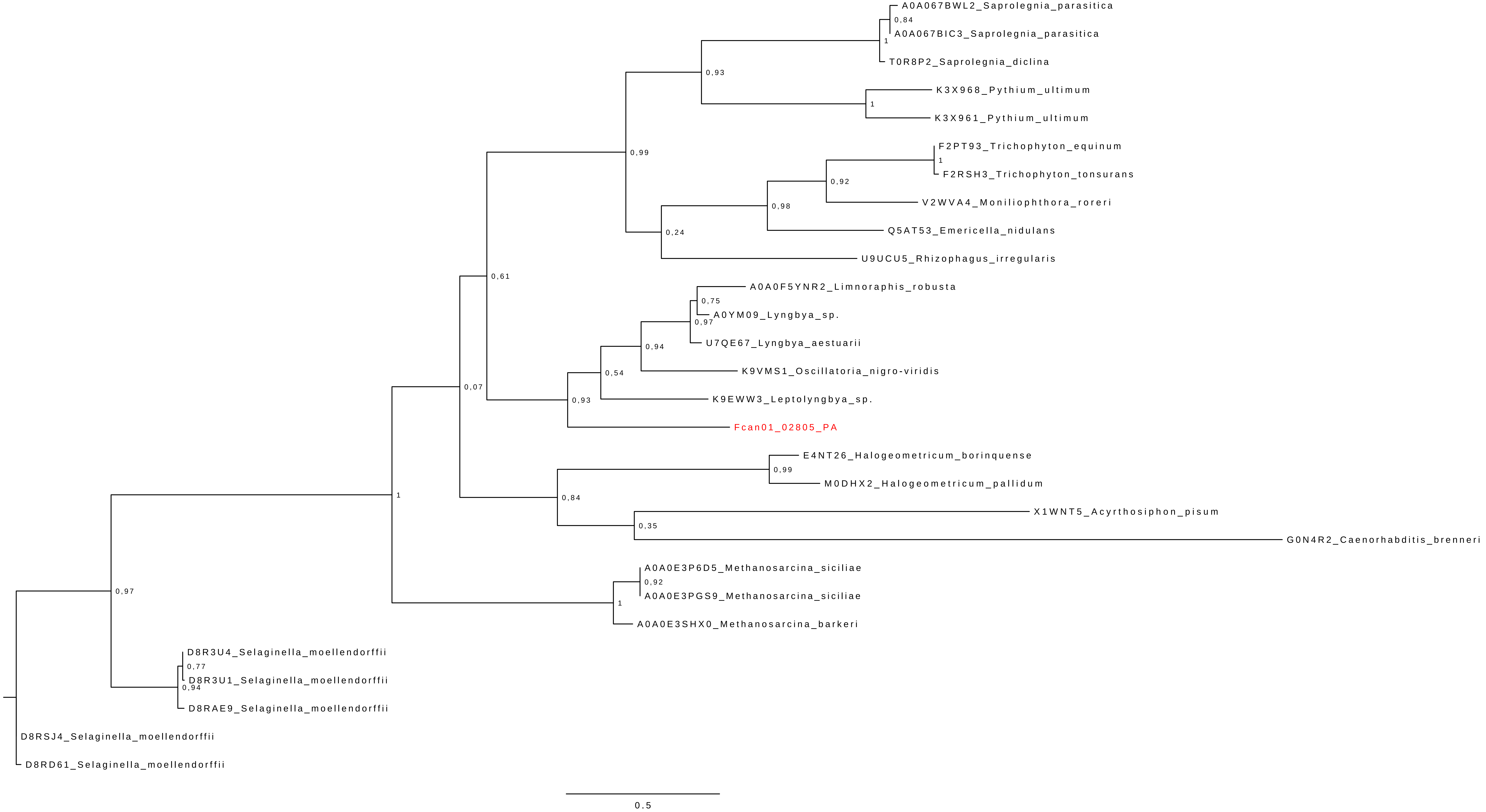
A0A067BWL2_Saprolegnia_parasitica
A0A067BIC3_Saprolegnia_parasitica
T0R8P2_Saprolegnia_diclina
K3X968_Pythium_ultimum
K3X961_Pythium_ultimum
F2PT93_Trichophyton_equinum
F2RSH3_Trichophyton_tonsurans
V2WVA4_Moniliophthora_roreri
Q5AT53_Emericella_nidulans
U9UCU5_Rhizophagus_irregularis
A0A0F5YNR2_Limnoraphis_robusta
A0YM09_Lyngbya_sp.
U7QE67_Lyngbya_aestuarii
K9VMS1_Oscillatoria_nigro-viridis
K9EWW3_Leptolyngbya_sp.
Fcan01_02805_PA
E4NT26_Halogeometricum_borinquense
M0DHX2_Halogeometricum_pallidum
X1WNT5_Acyrthosiphon_pisum
G0N4R2_Caenorhabditis_brenneri
A0A0E3P6D5_Methanosarcina_siciliae
A0A0E3PGS9_Methanosarcina_siciliae
A0A0E3SHX0_Methanosarcina_barkeri
D8R3U4_Selaginella_moellendorffii
D8R3U1_Selaginella_moellendorffii
D8RAE9_Selaginella_moellendorffii
D8RSJ4_Selaginella_moellendorffii
D8RD61_Selaginella_moellendorffii
0.5

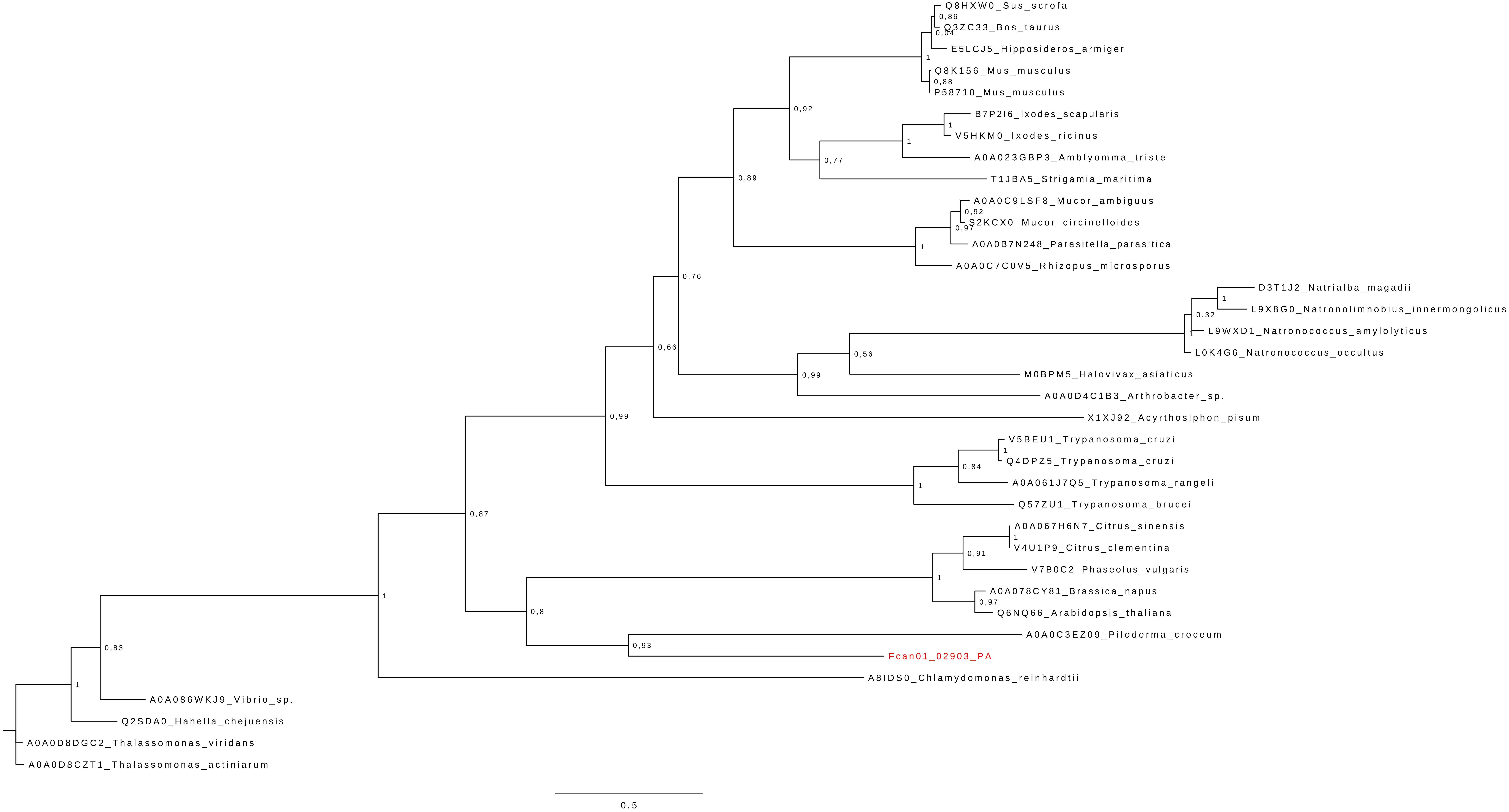
Q8HXW0_Sus_scrofa
0,86
Q3ZC33_Bos_taurus
0,04
E5LCJ5_Hipposideros_armiger
1
Q8K156_Mus_musculus
0,88
P58710_Mus_musculus
0,92
B7P2I6_Ixodes_scapularis
1
V5HKM0_Ixodes_ricinus
1
A0A023GBP3_Amblyomma_triste
0,77
T1JBA5_Strigamia_maritima
0,89
A0A0C9LSF8_Mucor_ambiguus
0,92
S2KCX0_Mucor_circinelloides
0,97
A0A0B7N248_Parasitella_parasitica
1
A0A0C7C0V5_Rhizopus_microsporus
0,76
D3T1J2_Natrialba_magadii
1
L9X8G0_Natronolimnobius_innermongolicus
0,32
L9WXD1_Natronococcus_amylolyticus
1
L0K4G6_Natronococcus_occultus
0,56
M0BPM5_Halovivax_asiaticus
0,99
A0A0D4C1B3_Arthrobacter_sp.
0,66
X1XJ92_Acyrthosiphon_pisum
0,99
V5BEU1_Trypanosoma_cruzi
1
Q4DPZ5_Trypanosoma_cruzi
0,84
A0A061J7Q5_Trypanosoma_rangeli
1
Q57ZU1_Trypanosoma_brucei
0,87
A0A067H6N7_Citrus_sinensis
1
V4U1P9_Citrus_clementina
0,91
V7B0C2_Phaseolus_vulgaris
1
A0A078CY81_Brassica_napus
0,97
Q6NQ66_Arabidopsis_thaliana
0,8
A0A0C3EZ09_Piloderma_croceum
0,93
Fcan01_02903_PA
1
A8IDS0_Chlamydomonas_reinhardtii
0,83
1
A0A086WKJ9_Vibrio_sp.
Q2SDA0_Hahella_chejuensis
A0A0D8DGC2_Thalassomonas_viridans
A0A0D8CZT1_Thalassomonas_actiniarum
0.5

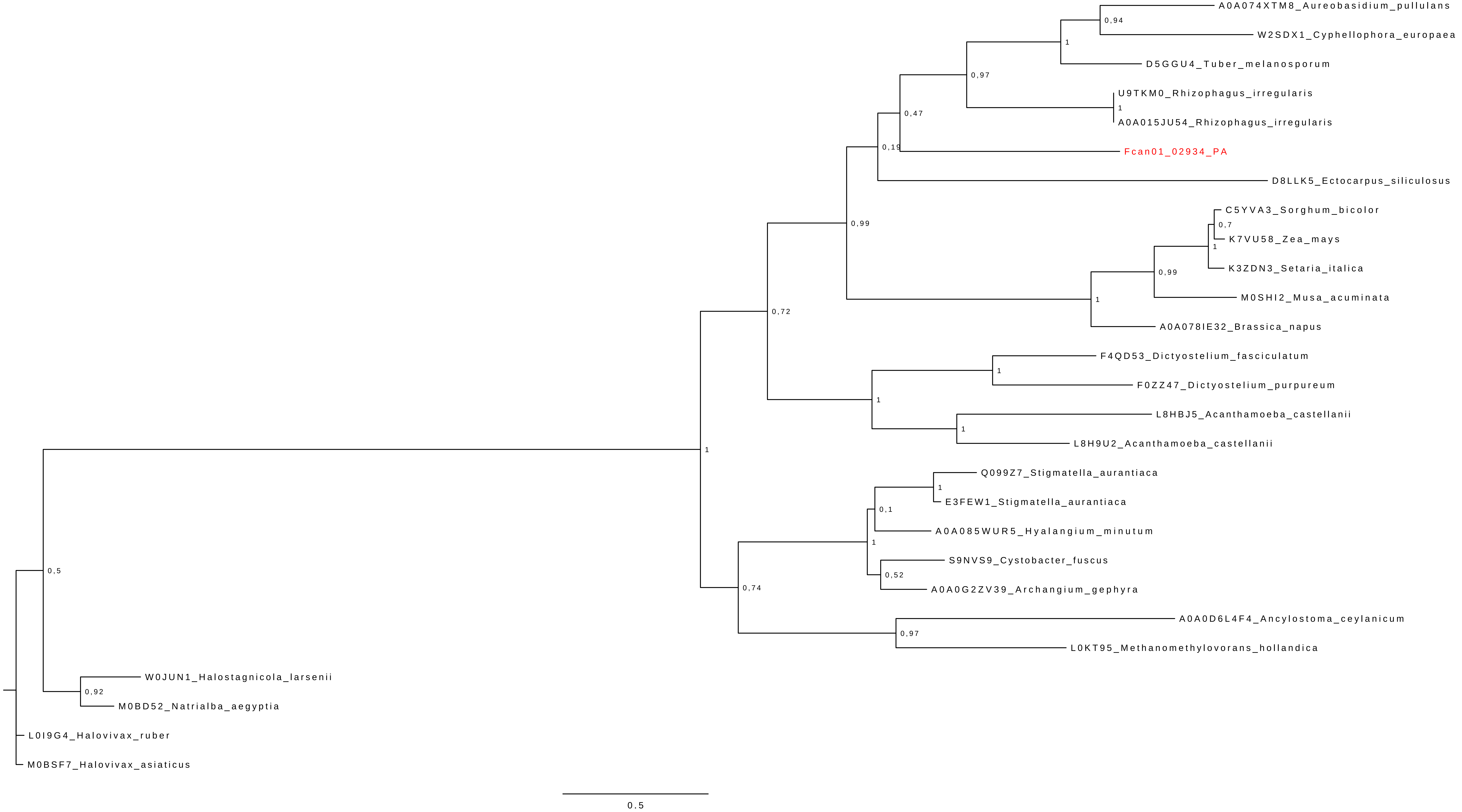
A0A074XTM8_Aureobasidium_pullulans
0,94
W2SDX1_Cyphellophora_europaea
1
D5GGU4_Tuber_melanosporum
0,97
U9TKM0_Rhizophagus_irregularis
1
A0A015JU54_Rhizophagus_irregularis
0,47
Fcan01_02934_PA
0,19
D8LLK5_Ectocarpus_siliculosus
C5YVA3_Sorghum_bicolor
0,7
K7VU58_Zea_mays
1
K3ZDN3_Setaria_italica
0,99
0,99
M0SHI2_Musa_acuminata
1
A0A078IE32_Brassica_napus
0,72
F4QD53_Dictyostelium_fasciculatum
1
F0ZZ47_Dictyostelium_purpureum
1
L8HBJ5_Acanthamoeba_castellanii
1
L8H9U2_Acanthamoeba_castellanii
1
Q099Z7_Stigmatella_aurantiaca
1
E3FEW1_Stigmatella_aurantiaca
0,1
A0A085WUR5_Hyalangium_minutum
1
S9NVS9_Cystobacter_fuscus
0,52
A0A0G2ZV39_Archangium_gephyra
0,74
A0A0D6L4F4_Ancylostoma_ceylanicum
0,97
L0KT95_Methanomethylovorans_hollandica
0,5
W0JUN1_Halostagnicola_larsenii
0,92
M0BD52_Natrialba_aegyptia
L0I9G4_Halovivax_ruber
M0BSF7_Halovivax_asiaticus
0.5

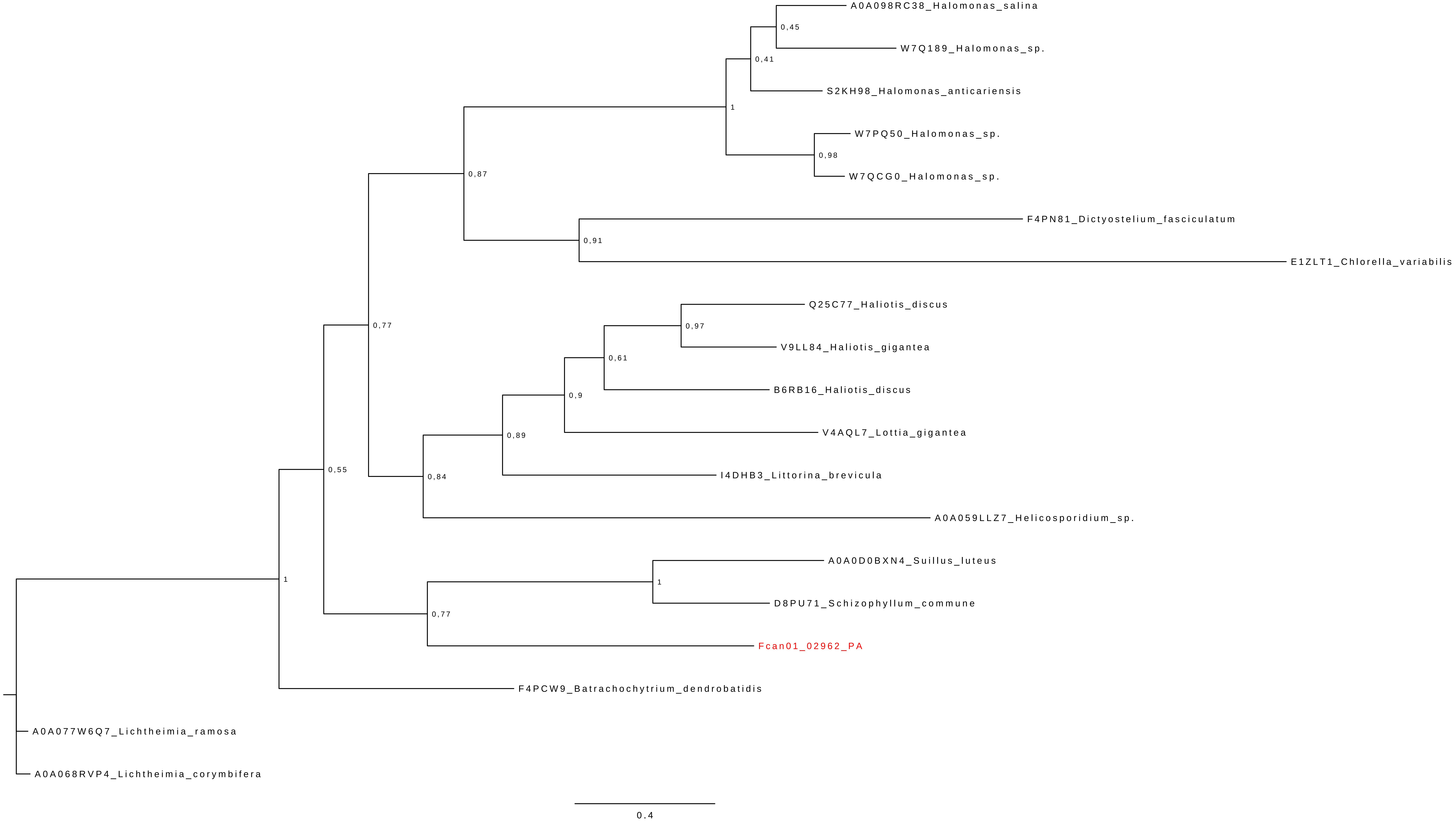

A0A098RC38_Halomonas_salina
0,45
W7Q189_Halomonas_sp.
0,41
S2KH98_Halomonas_anticariensis
1
W7PQ50_Halomonas_sp.
0,98
W7QCG0_Halomonas_sp.
0,87
F4PN81_Dictyostelium_fasciculatum
0,91
E1ZLT1_Chlorella_variabilis
Q25C77_Haliotis_discus
0,97
V9LL84_Haliotis_gigantea
0,61
B6RB16_Haliotis_discus
0,9
V4AQL7_Lottia_gigantea
0,89
I4DHB3_Littorina_brevicula
0,77
0,55
0,84
A0A059LLZ7_Helicosporidium_sp.
A0A0D0BXN4_Suillus_luteus
1
D8PU71_Schizophyllum_commune
0,77
Fcan01_02962_PA
1
F4PCW9_Batrachochytrium_dendrobatidis
A0A077W6Q7_Lichtheimia_ramosa
A0A068RVP4_Lichtheimia_corymbifera
0.4

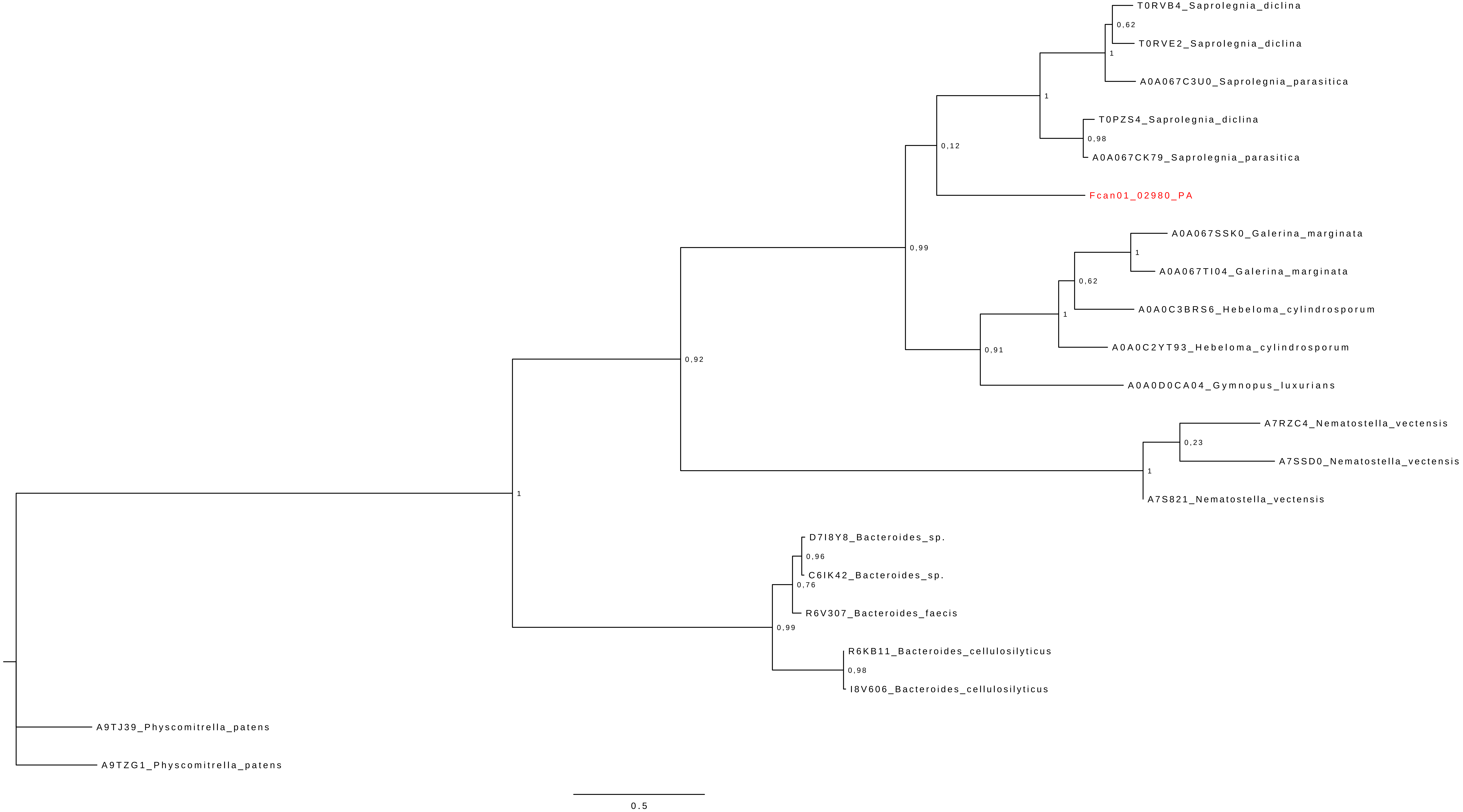
T0RVB4_Saprolegnia_diclina
0,62
T0RVE2_Saprolegnia_diclina
1
A0A067C3U0_Saprolegnia_parasitica
1
T0PZS4_Saprolegnia_diclina
0,98
A0A067CK79_Saprolegnia_parasitica
0,12
Fcan01_02980_PA
A0A067SSK0_Galerina_marginata
1
A0A067TI04_Galerina_marginata
0,99
0,62
A0A0C3BRS6_Hebeloma_cylindrosporum
1
A0A0C2YT93_Hebeloma_cylindrosporum
0,91
0,92
A0A0D0CA04_Gymnopus_luxurians
A7RZC4_Nematostella_vectensis
0,23
A7SSD0_Nematostella_vectensis
1
A7S821_Nematostella_vectensis
1
D7I8Y8_Bacteroides_sp.
0,96
C6IK42_Bacteroides_sp.
0,76
R6V307_Bacteroides_faecis
0,99
R6KB11_Bacteroides_cellulosilyticus
0,98
I8V606_Bacteroides_cellulosilyticus
A9TJ39_Physcomitrella_patens
A9TZG1_Physcomitrella_patens
0.5

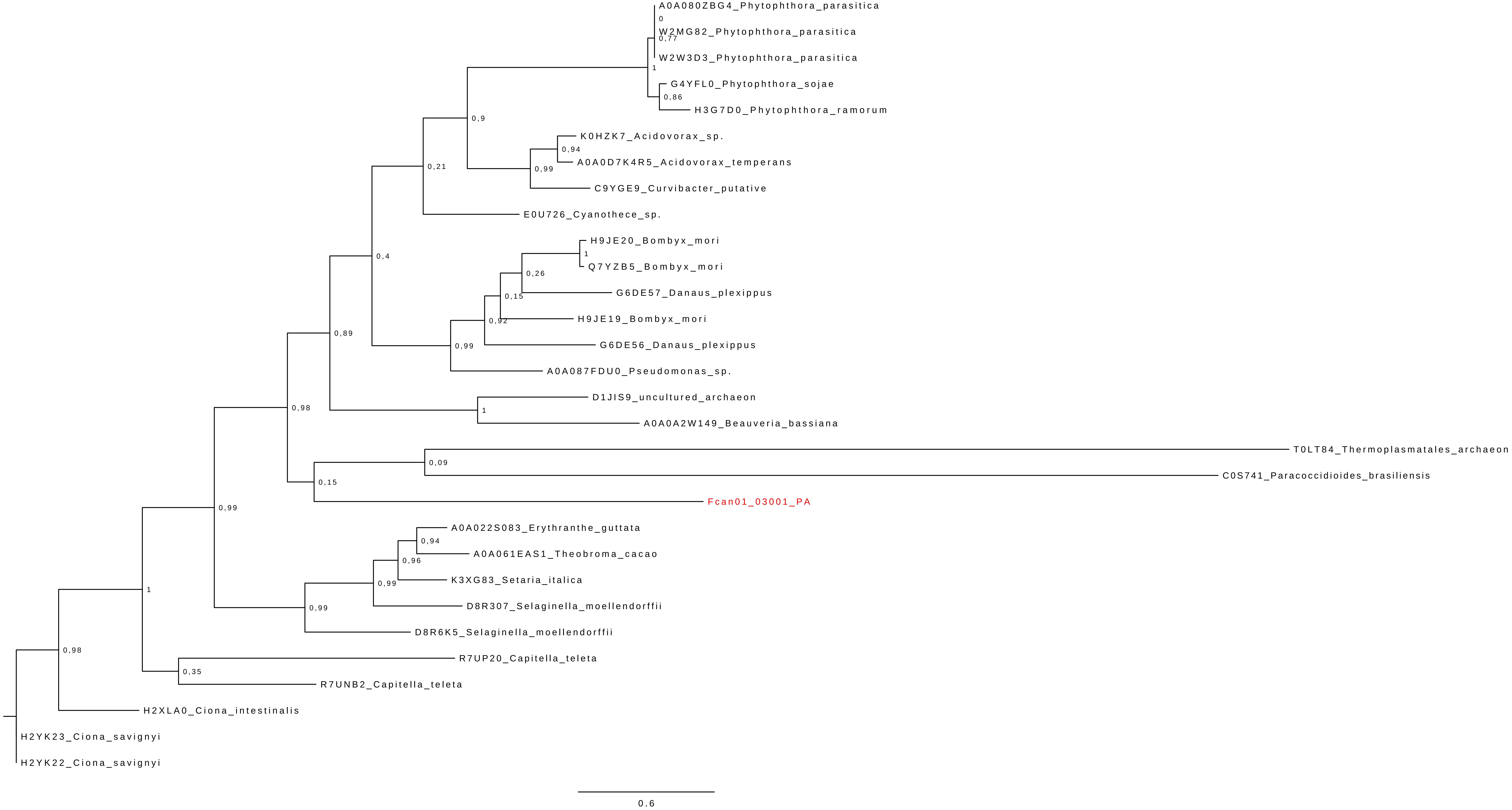
A0A080ZBG4_Phytophthora_parasitica
0
W2MG82_Phytophthora_parasitica
0,77
W2W3D3_Phytophthora_parasitica
1
G4YFL0_Phytophthora_sojae
0,86
H3G7D0_Phytophthora_ramorum
0,9
K0HZK7_Acidovorax_sp.
0,94
A0A0D7K4R5_Acidovorax_temperans
0,99
C9YGE9_Curvibacter_putative
0,21
E0U726_Cyanothece_sp.
H9JE20_Bombyx_mori
1
Q7YZB5_Bombyx_mori
0,26
G6DE57_Danaus_plexippus
0,15
0,4
H9JE19_Bombyx_mori
0,92
G6DE56_Danaus_plexippus
0,99
A0A087FDU0_Pseudomonas_sp.
0,89
D1JIS9_uncultured_archaeon
1
A0A0A2W149_Beauveria_bassiana
0,98
T0LT84_Thermoplasmatales_archaeon
0,09
C0S741_Paracoccidioides_brasiliensis
0,15
Fcan01_03001_PA
0,99
A0A022S083_Erythranthe_guttata
0,94
A0A061EAS1_Theobroma_cacao
0,96
K3XG83_Setaria_italica
0,99
D8R307_Selaginella_moellendorffii
0,99
1
D8R6K5_Selaginella_moellendorffii
R7UP20_Capitella_teleta
0,35
R7UNB2_Capitella_teleta
0,98
H2XLA0_Ciona_intestinalis
H2YK23_Ciona_savignyi
H2YK22_Ciona_savignyi
0.6

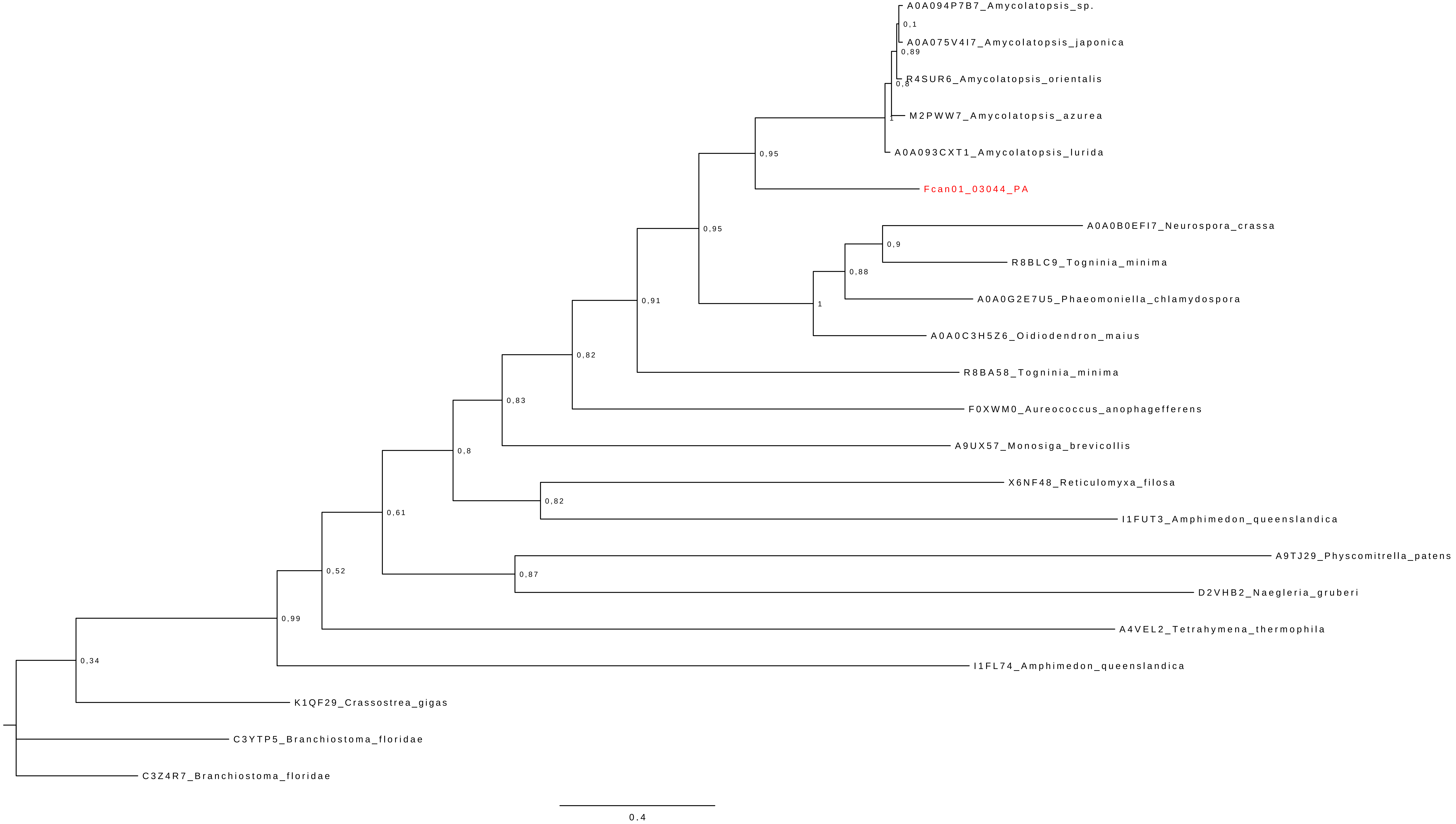
A0A094P7B7_Amycolatopsis_sp.
0,1
A0A075V4I7_Amycolatopsis_japonica
0,89
R4SUR6_Amycolatopsis_orientalis
0,8
M2PWW7_Amycolatopsis_azurea
1
A0A093CXT1_Amycolatopsis_lurida
0,95
Fcan01_03044_PA
A0A0B0EFI7_Neurospora_crassa
0,9
R8BLC9_Togninia_minima
0,88
A0A0G2E7U5_Phaeomoniella_chlamydospora
0,95
1
A0A0C3H5Z6_Oidiodendron_maius
0,91
R8BA58_Togninia_minima
0,82
F0XWM0_Aureococcus_anophagefferens
0,83
A9UX57_Monosiga_brevicollis
0,8
X6NF48_Reticulomyxa_filosa
0,82
I1FUT3_Amphimedon_queenslandica
0,61
A9TJ29_Physcomitrella_patens
0,87
D2VHB2_Naegleria_gruberi
0,52
A4VEL2_Tetrahymena_thermophila
0,99
I1FL74_Amphimedon_queenslandica
0,34
K1QF29_Crassostrea_gigas
C3YTP5_Branchiostoma_floridae
C3Z4R7_Branchiostoma_floridae
0.4

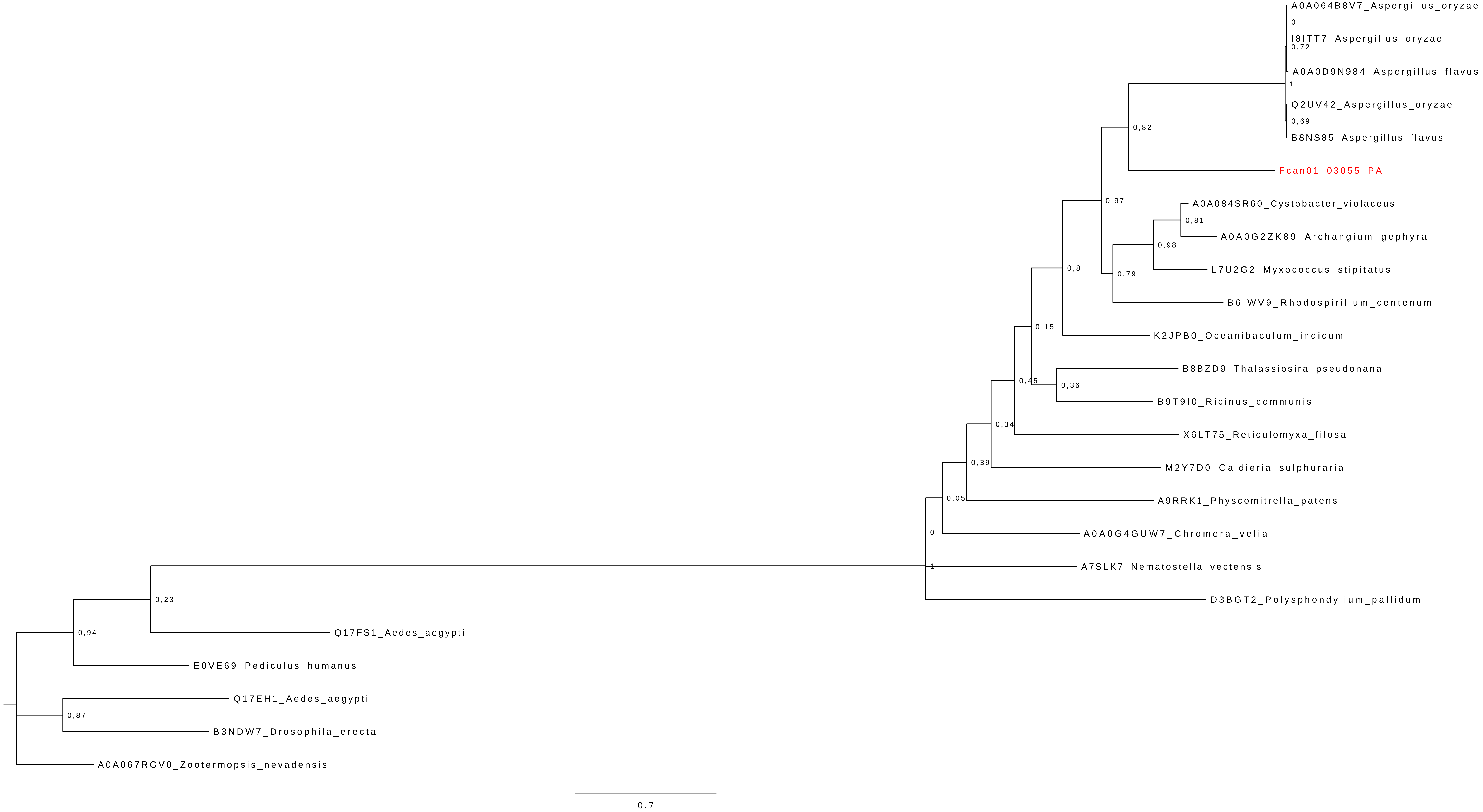
A0A064B8V7_Aspergillus_oryzae
0
I8ITT7_Aspergillus_oryzae
0,72
A0A0D9N984_Aspergillus_flavus
1
Q2UV42_Aspergillus_oryzae
0,69
B8NS85_Aspergillus_flavus
0,82
Fcan01_03055_PA
0,97
A0A084SR60_Cystobacter_violaceus
0,81
A0A0G2ZK89_Archangium_gephyra
0,98
L7U2G2_Myxococcus_stipitatus
0,8
0,79
B6IWV9_Rhodospirillum_centenum
0,15
K2JPB0_Oceanibaculum_indicum
B8BZD9_Thalassiosira_pseudonana
0,45
0,36
B9T9I0_Ricinus_communis
0,34
X6LT75_Reticulomyxa_filosa
0,39
M2Y7D0_Galdieria_sulphuraria
0,05
A9RRK1_Physcomitrella_patens
0
A0A0G4GUW7_Chromera_velia
1
A7SLK7_Nematostella_vectensis
D3BGT2_Polysphondylium_pallidum
0,23
Q17FS1_Aedes_aegypti
0,94
E0VE69_Pediculus_humanus
Q17EH1_Aedes_aegypti
0,87
B3NDW7_Drosophila_erecta
A0A067RGV0_Zootermopsis_nevadensis
0.7

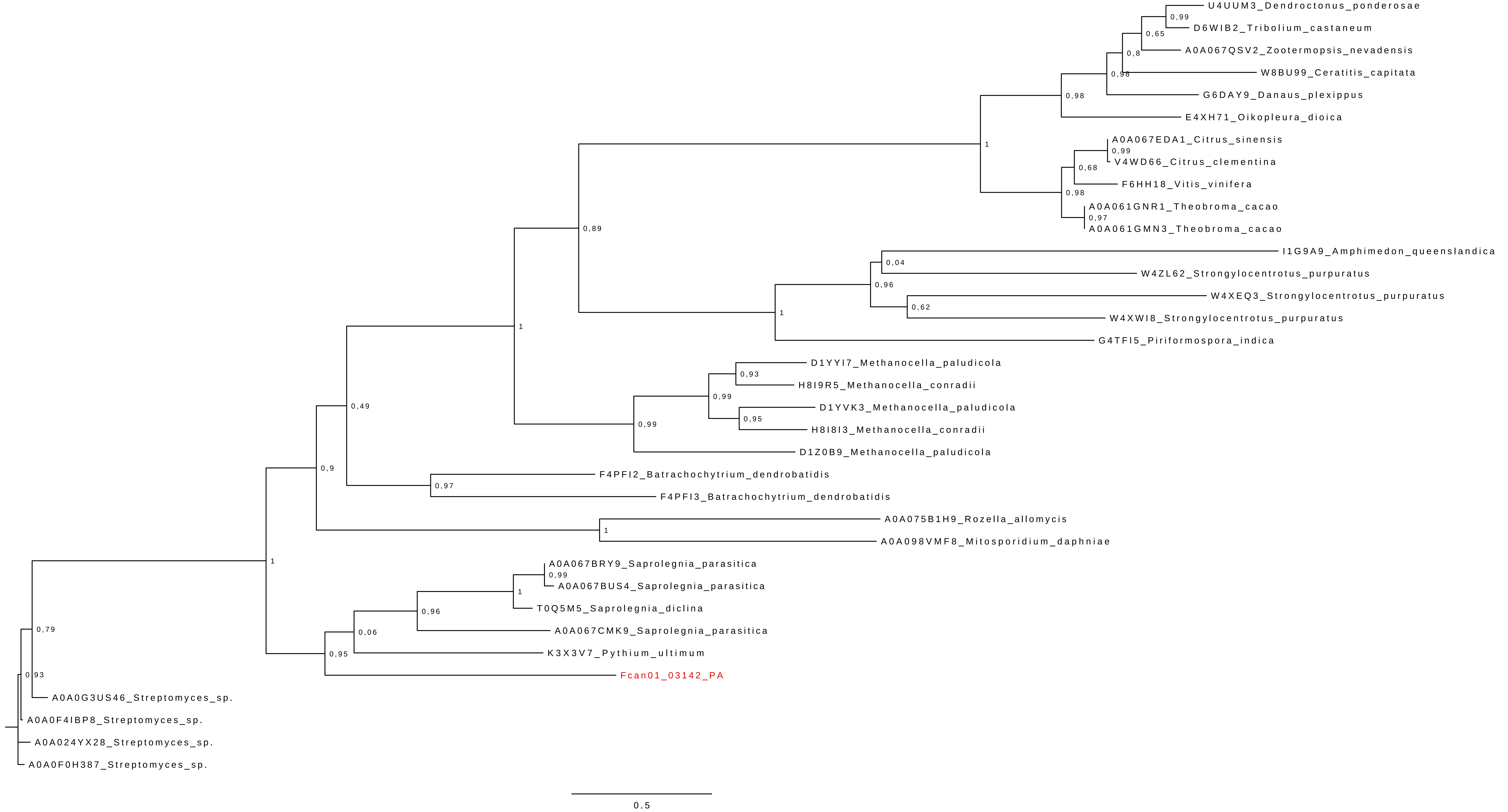
U4UUM3_Dendroctonus_ponderosae
D6WIB2_Tribolium_castaneum
A0A067QSV2_Zootermopsis_nevadensis
W8BU99_Ceratitis_capitata
G6DAY9_Danaus_plexippus
E4XH71_Oikopleura_dioica
A0A067EDA1_Citrus_sinensis
V4WD66_Citrus_clementina
F6HH18_Vitis_vinifera
A0A061GNR1_Theobroma_cacao
A0A061GMN3_Theobroma_cacao
I1G9A9_Amphimedon_queenslandica
W4ZL62_Strongylocentrotus_purpuratus
W4XEQ3_Strongylocentrotus_purpuratus
W4XWI8_Strongylocentrotus_purpuratus
G4TFI5_Piriformospora_indica
D1YYI7_Methanocella_paludicola
H8I9R5_Methanocella_conradii
D1YVK3_Methanocella_paludicola
H8I8I3_Methanocella_conradii
D1Z0B9_Methanocella_paludicola
F4PFI2_Batrachochytrium_dendrobatidis
F4PFI3_Batrachochytrium_dendrobatidis
A0A075B1H9_Rozella_allomycis
A0A098VMF8_Mitosporidium_daphniae
A0A067BRY9_Saprolegnia_parasitica
A0A067BUS4_Saprolegnia_parasitica
T0Q5M5_Saprolegnia_diclina
A0A067CMK9_Saprolegnia_parasitica
K3X3V7_Pythium_ultimum
Fcan01_03142_PA
A0A0G3US46_Streptomyces_sp.
A0A0F4IBP8_Streptomyces_sp.
A0A024YX28_Streptomyces_sp.
A0A0F0H387_Streptomyces_sp.
0.5

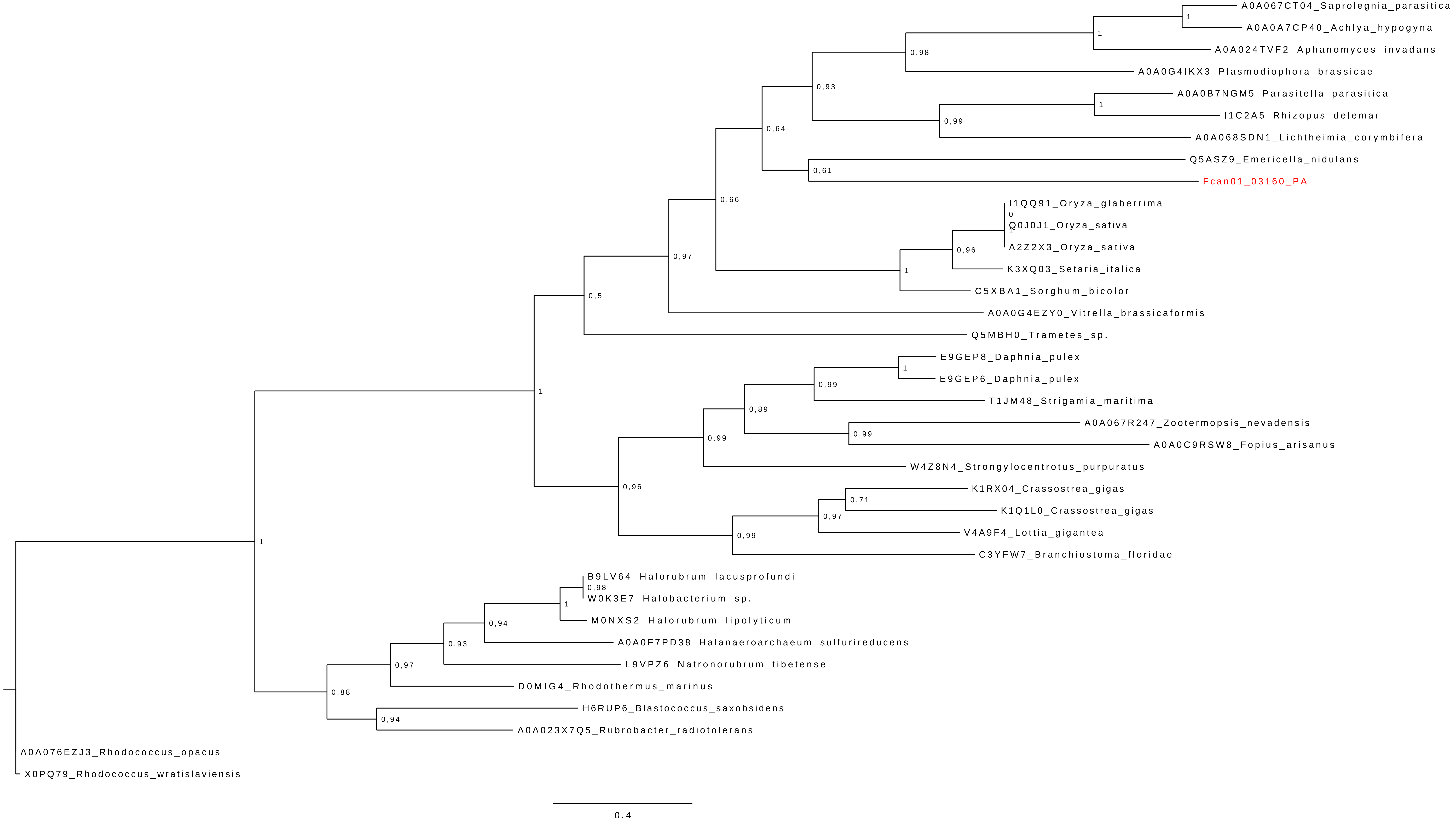
A0A067CT04_Saprolegnia_parasitica
A0A0A7CP40_Achlya_hypogyna
A0A024TVF2_Aphanomyces_invadans
A0A0G4IKX3_Plasmodiophora_brassicae
A0A0B7NGM5_Parasitella_parasitica
I1C2A5_Rhizopus_delemar
A0A068SDN1_Lichtheimia_corymbifera
Q5ASZ9_Emericella_nidulans
Fcan01_03160_PA
I1QQ91_Oryza_glaberrima
Q0J0J1_Oryza_sativa
A2Z2X3_Oryza_sativa
K3XQ03_Setaria_italica
C5XBA1_Sorghum_bicolor
A0A0G4EZY0_Vitrella_brassicaformis
Q5MBH0_Trametes_sp.
E9GEP8_Daphnia_pulex
E9GEP6_Daphnia_pulex
T1JM48_Strigamia_maritima
A0A067R247_Zootermopsis_nevadensis
A0A0C9RSW8_Fopius_arisanus
W4Z8N4_Strongylocentrotus_purpuratus
K1RX04_Crassostrea_gigas
K1Q1L0_Crassostrea_gigas
V4A9F4_Lottia_gigantea
C3YFW7_Branchiostoma_floridae
B9LV64_Halorubrum_lacusprofundi
W0K3E7_Halobacterium_sp.
M0NXS2_Halorubrum_lipolyticum
A0A0F7PD38_Halanaeroarchaeum_sulfurireducens
L9VPZ6_Natronorubrum_tibetense
D0MIG4_Rhodothermus_marinus
H6RUP6_Blastococcus_saxobsidens
A0A023X7Q5_Rubrobacter_radiotolerans
A0A076EZJ3_Rhodococcus_opacus
X0PQ79_Rhodococcus_wratislaviensis
0.4

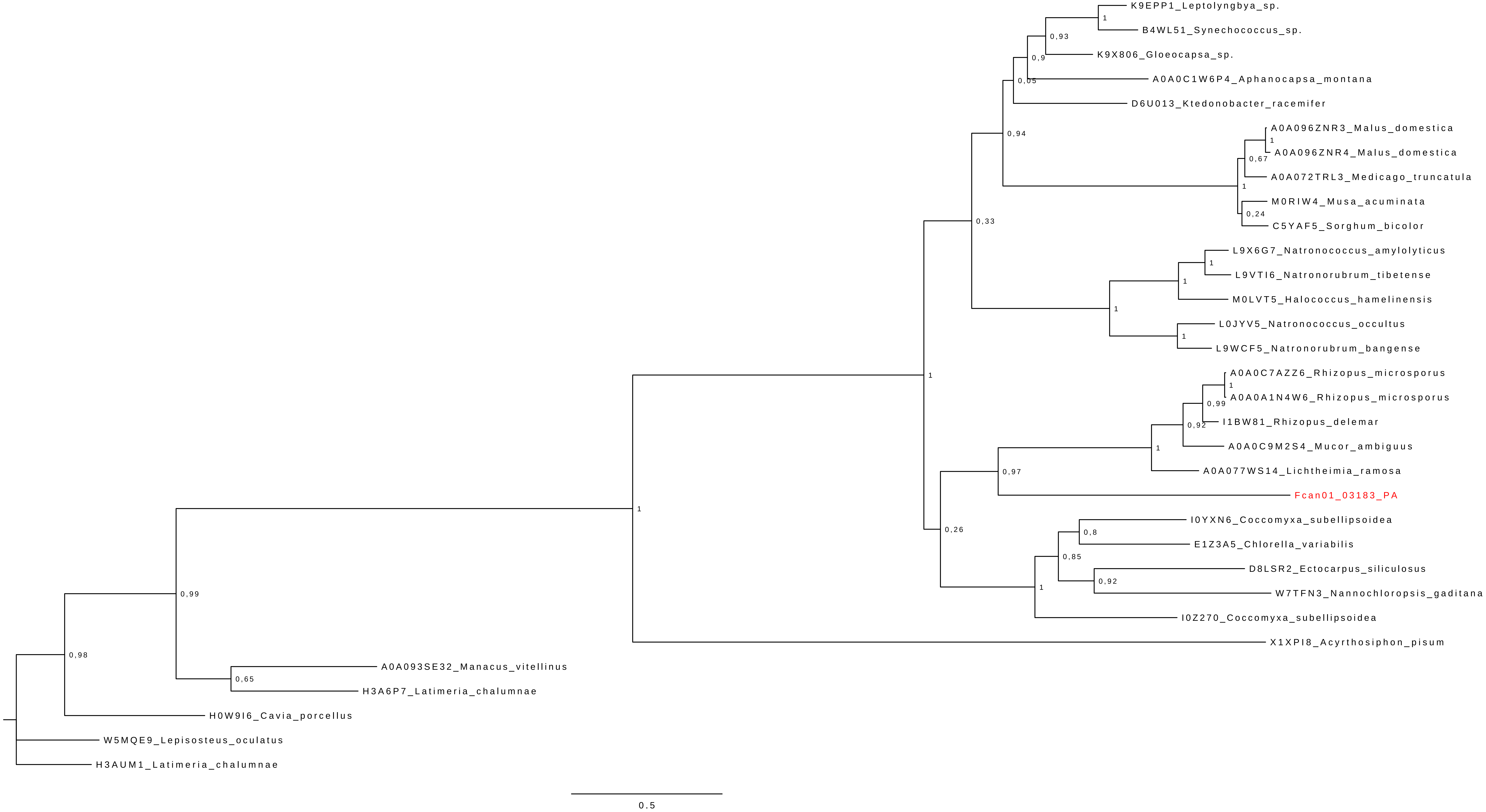
K9EPP1_Leptolyngbya_sp.
B4WL51_Synechococcus_sp.
K9X806_Gloeocapsa_sp.
A0A0C1W6P4_Aphanocapsa_montana
D6U013_Ktedonobacter_racemifer
A0A096ZNR3_Malus_domestica
A0A096ZNR4_Malus_domestica
A0A072TRL3_Medicago_truncatula
M0RIW4_Musa_acuminata
C5YAF5_Sorghum_bicolor
L9X6G7_Natronococcus_amylolyticus
L9VTI6_Natronorubrum_tibetense
M0LVT5_Halococcus_hamelinensis
L0JYV5_Natronococcus_occultus
L9WCF5_Natronorubrum_bangense
A0A0C7AZZ6_Rhizopus_microsporus
A0A0A1N4W6_Rhizopus_microsporus
I1BW81_Rhizopus_delemar
A0A0C9M2S4_Mucor_ambiguus
A0A077WS14_Lichtheimia_ramosa
Fcan01_03183_PA
I0YXN6_Coccomyxa_subellipsoidea
E1Z3A5_Chlorella_variabilis
D8LSR2_Ectocarpus_siliculosus
W7TFN3_Nannochloropsis_gaditana
I0Z270_Coccomyxa_subellipsoidea
X1XPI8_Acyrthosiphon_pisum
A0A093SE32_Manacus_vitellinus
H3A6P7_Latimeria_chalumnae
H0W9I6_Cavia_porcellus
W5MQE9_Lepisosteus_oculatus
H3AUM1_Latimeria_chalumnae
0.5

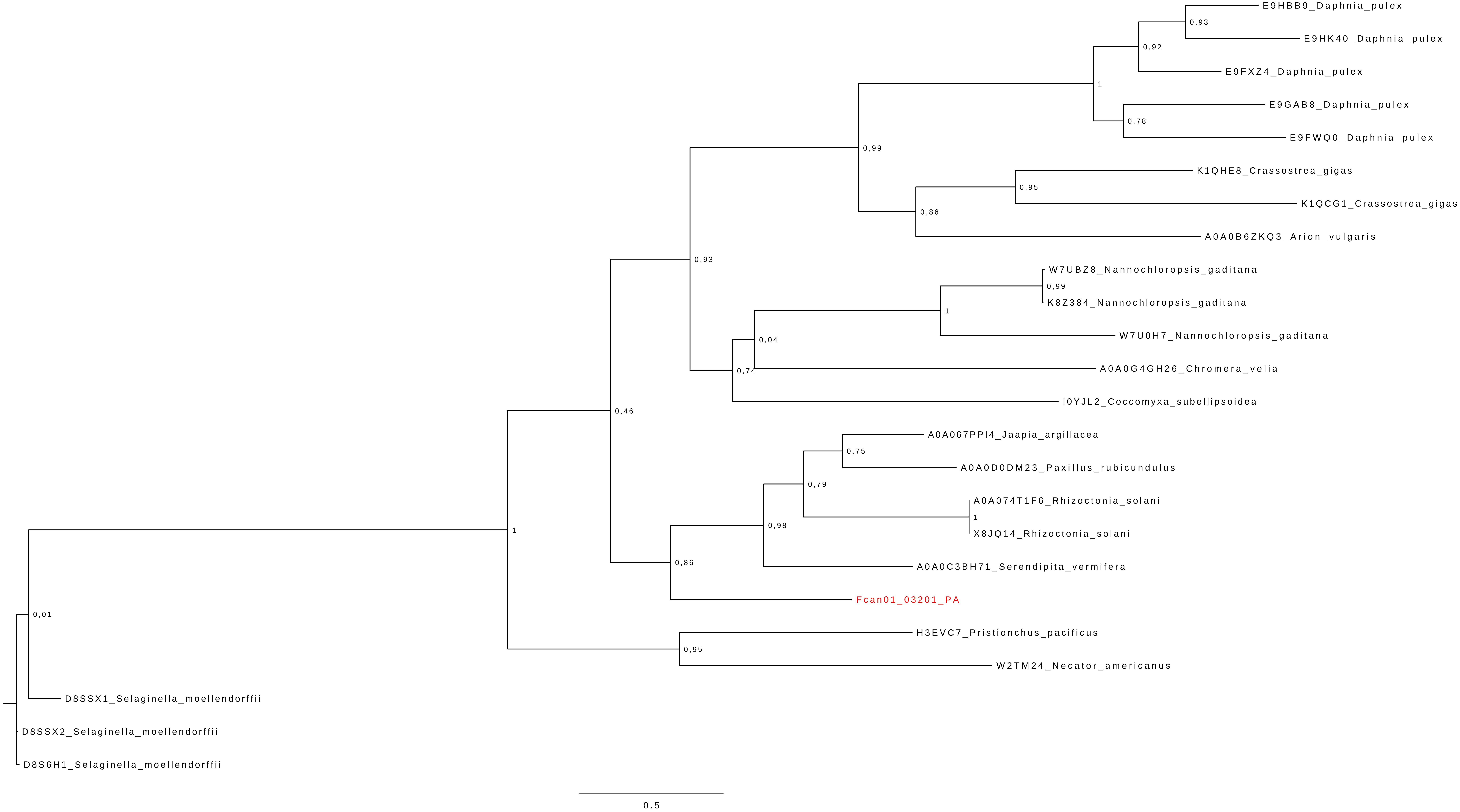
E9HBB9_Daphnia_pulex
0,93
E9HK40_Daphnia_pulex
0,92
E9FXZ4_Daphnia_pulex
1
E9GAB8_Daphnia_pulex
0,78
E9FWQ0_Daphnia_pulex
0,99
K1QHE8_Crassostrea_gigas
0,95
K1QCG1_Crassostrea_gigas
0,86
A0A0B6ZKQ3_Arion_vulgaris
0,93
W7UBZ8_Nannochloropsis_gaditana
0,99
K8Z384_Nannochloropsis_gaditana
1
W7U0H7_Nannochloropsis_gaditana
0,04
A0A0G4GH26_Chromera_velia
0,74
I0YJL2_Coccomyxa_subellipsoidea
0,46
A0A067PPI4_Jaapia_argillacea
0,75
A0A0D0DM23_Paxillus_rubicundulus
0,79
A0A074T1F6_Rhizoctonia_solani
1
X8JQ14_Rhizoctonia_solani
0,98
1
A0A0C3BH71_Serendipita_vermifera
0,86
Fcan01_03201_PA
0,01
H3EVC7_Pristionchus_pacificus
0,95
W2TM24_Necator_americanus
D8SSX1_Selaginella_moellendorffii
D8SSX2_Selaginella_moellendorffii
D8S6H1_Selaginella_moellendorffii
0.5

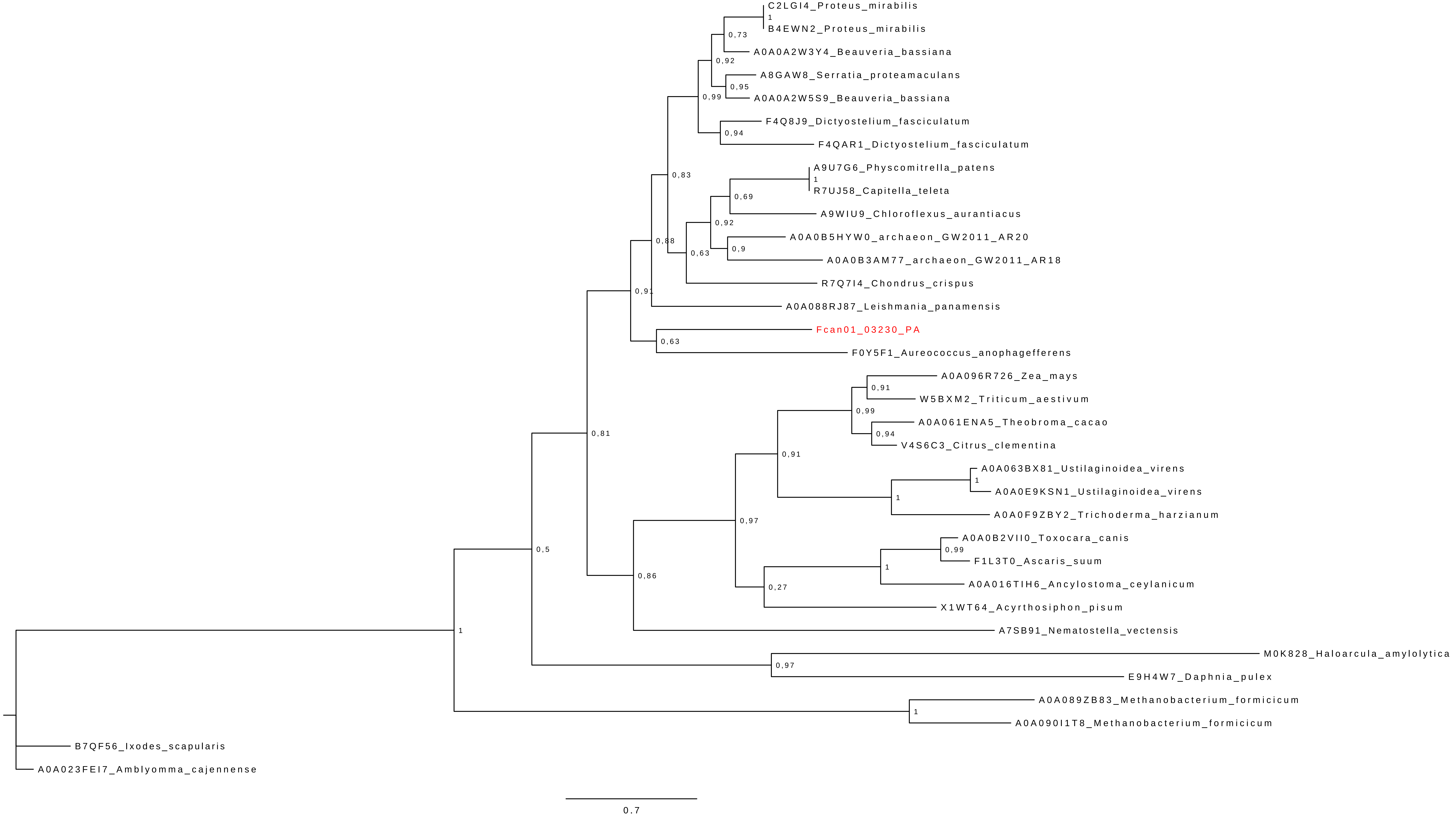

C2LGI4_Proteus_mirabilis
B4EWN2_Proteus_mirabilis
A0A0A2W3Y4_Beauveria_bassiana
A8GAW8_Serratia_proteamaculans
A0A0A2W5S9_Beauveria_bassiana
F4Q8J9_Dictyostelium_fasciculatum
F4QAR1_Dictyostelium_fasciculatum
A9U7G6_Physcomitrella_patens
R7UJ58_Capitella_teleta
A9WIU9_Chloroflexus_aurantiacus
A0A0B5HYW0_archaeon_GW2011_AR20
A0A0B3AM77_archaeon_GW2011_AR18
R7Q7I4_Chondrus_crispus
A0A088RJ87_Leishmania_panamensis
Fcan01_03230_PA
F0Y5F1_Aureococcus_anophagefferens
A0A096R726_Zea_mays
W5BXM2_Triticum_aestivum
A0A061ENA5_Theobroma_cacao
V4S6C3_Citrus_clementina
A0A063BX81_Ustilaginoidea_virens
A0A0E9KSN1_Ustilaginoidea_virens
A0A0F9ZBY2_Trichoderma_harzianum
A0A0B2VII0_Toxocara_canis
F1L3T0_Ascaris_suum
A0A016TIH6_Ancylostoma_ceylanicum
X1WT64_Acyrthosiphon_pisum
A7SB91_Nematostella_vectensis
M0K828_Haloarcula_amylolytica
E9H4W7_Daphnia_pulex
A0A089ZB83_Methanobacterium_formicicum
A0A090I1T8_Methanobacterium_formicicum
B7QF56_Ixodes_scapularis
A0A023FEI7_Amblyomma_cajennense
1
0,73
0,92
0,95
0,99
0,94
0,83
1
0,69
0,92
0,88
0,9
0,63
0,91
0,63
0,91
0,99
0,94
0,81
0,91
1
1
0,97
0,99
1
0,86
0,27
0,5
1
0,97
1
0.7

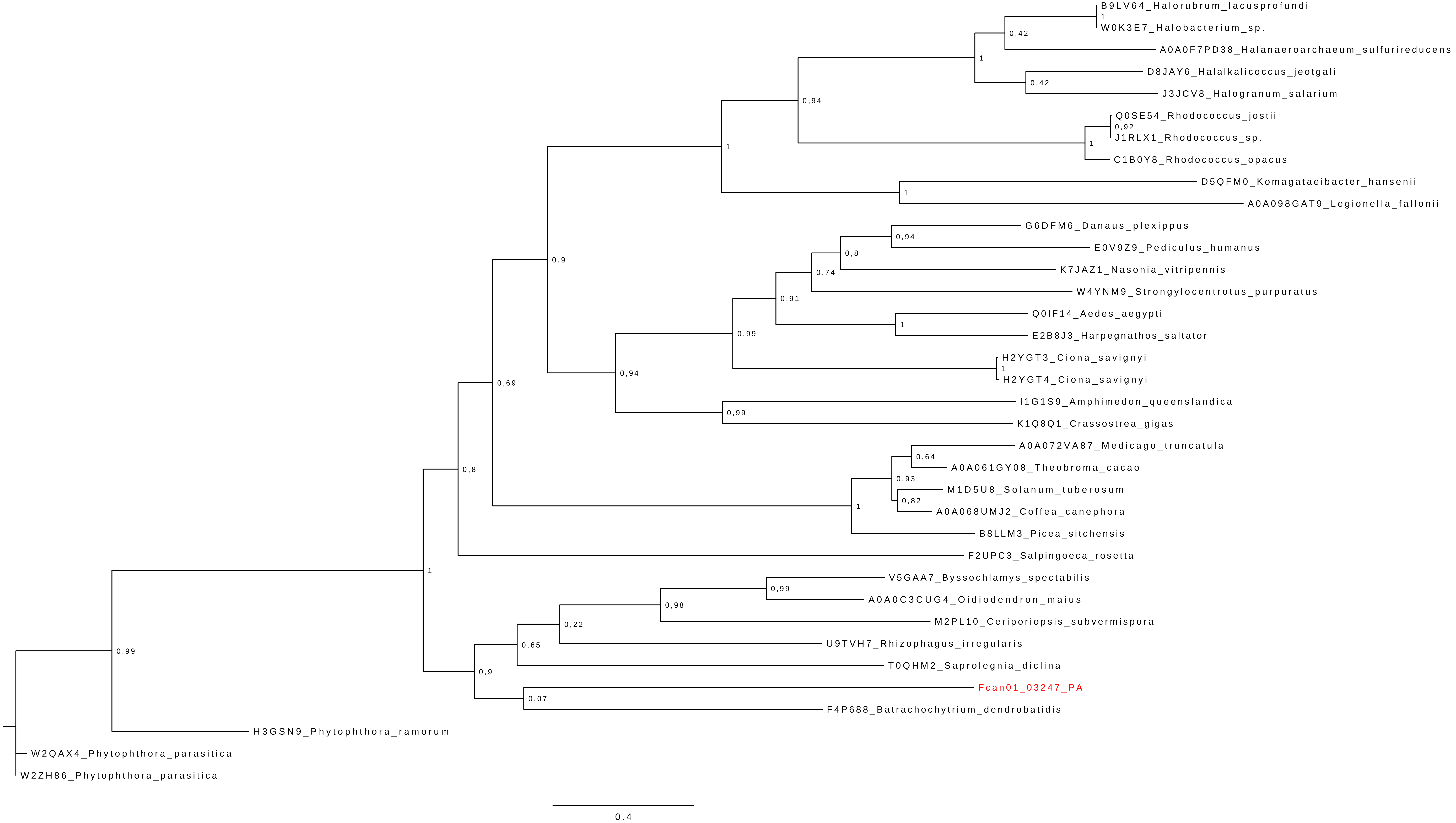
B9LV64_Halorubrum_lacusprofundi
W0K3E7_Halobacterium_sp.
A0A0F7PD38_Halanaeroarchaeum_sulfurireducens
D8JAY6_Halalkalicoccus_jeotgali
J3JCV8_Halogranum_salarium
Q0SE54_Rhodococcus_jostii
J1RLX1_Rhodococcus_sp.
C1B0Y8_Rhodococcus_opacus
D5QFM0_Komagataeibacter_hansenii
A0A098GAT9_Legionella_fallonii
G6DFM6_Danaus_plexippus
E0V9Z9_Pediculus_humanus
K7JAZ1_Nasonia_vitripennis
W4YNM9_Strongylocentrotus_purpuratus
Q0IF14_Aedes_aegypti
E2B8J3_Harpegnathos_saltator
H2YGT3_Ciona_savignyi
H2YGT4_Ciona_savignyi
I1G1S9_Amphimedon_queenslandica
K1Q8Q1_Crassostrea_gigas
A0A072VA87_Medicago_truncatula
A0A061GY08_Theobroma_cacao
M1D5U8_Solanum_tuberosum
A0A068UMJ2_Coffea_canephora
B8LLM3_Picea_sitchensis
F2UPC3_Salpingoeca_rosetta
V5GAA7_Byssochlamys_spectabilis
A0A0C3CUG4_Oidiodendron_maius
M2PL10_Ceriporiopsis_subvermispora
U9TVH7_Rhizophagus_irregularis
T0QHM2_Saprolegnia_diclina
Fcan01_03247_PA
F4P688_Batrachochytrium_dendrobatidis
H3GSN9_Phytophthora_ramorum
W2QAX4_Phytophthora_parasitica
W2ZH86_Phytophthora_parasitica
0.4

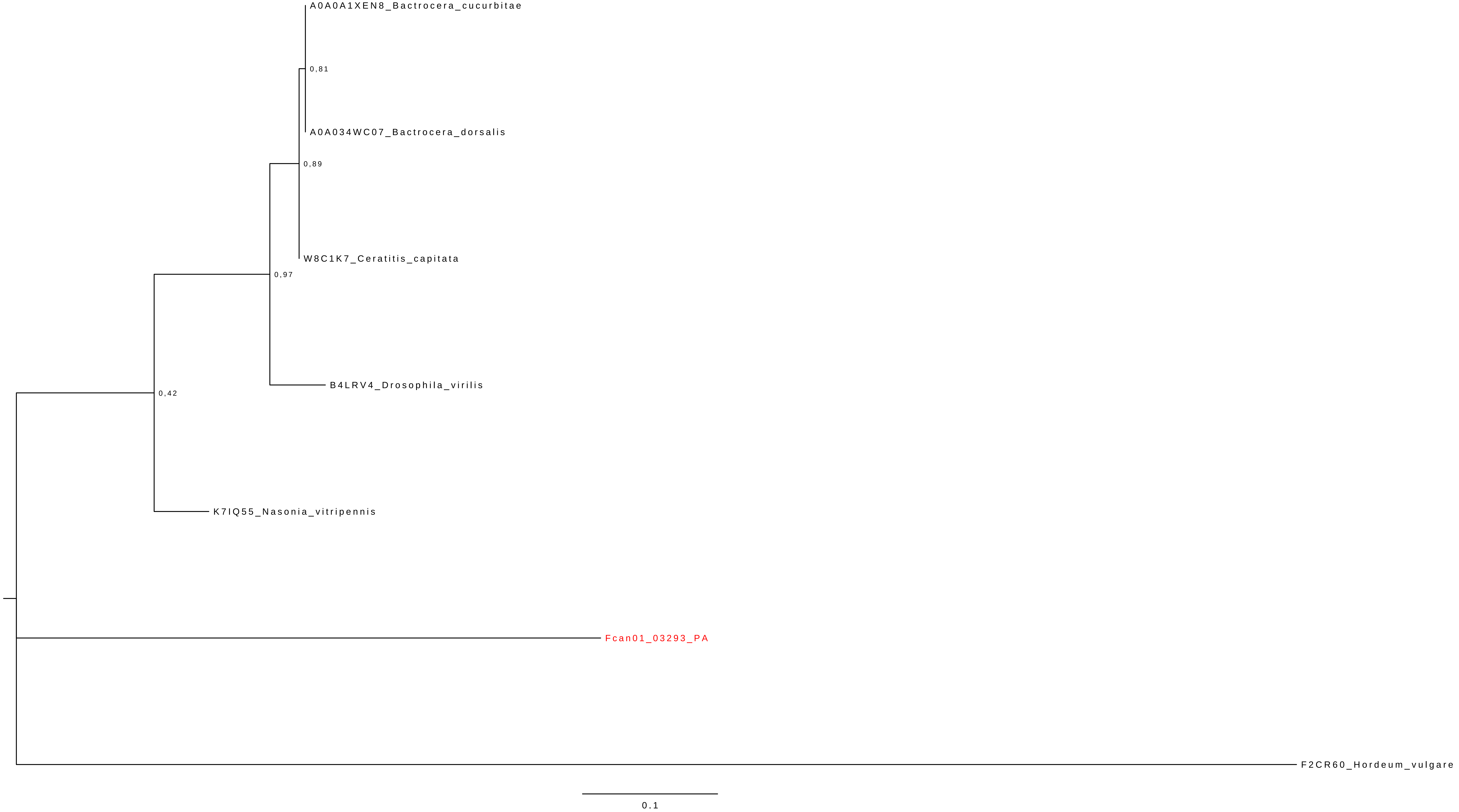

A0A0A1XEN8_Bactrocera_cucurbitae
0,81
A0A034WC07_Bactrocera_dorsalis
0,89
W8C1K7_Ceratitis_capitata
0,97
B4LRV4_Drosophila_virilis
0,42
K7IQ55_Nasonia_vitripennis
Fcan01_03293_PA
F2CR60_Hordeum_vulgare
0.1

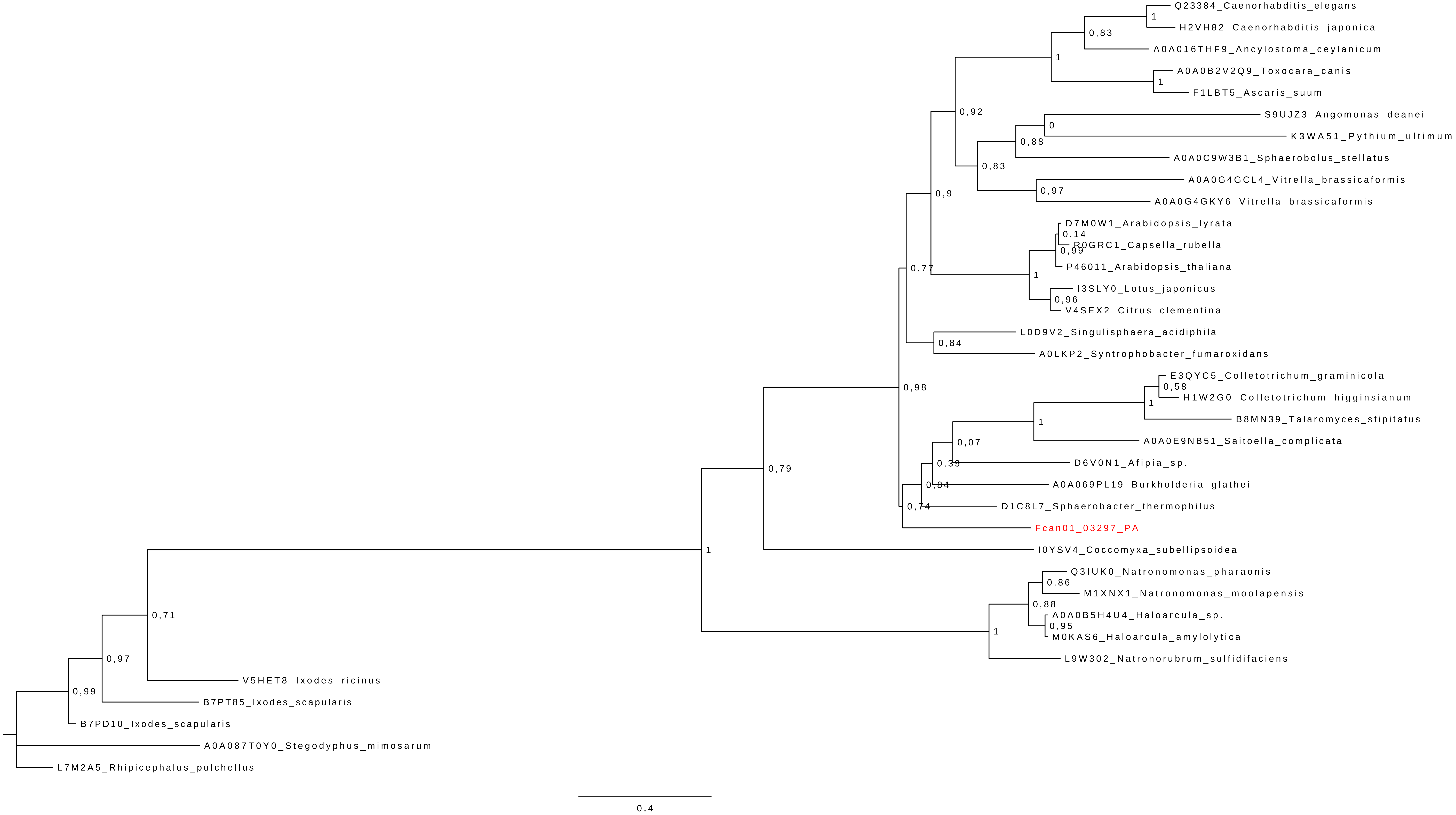
Q23384_Caenorhabditis_elegans
H2VH82_Caenorhabditis_japonica
A0A016THF9_Ancylostoma_ceylanicum
A0A0B2V2Q9_Toxocara_canis
F1LBT5_Ascaris_suum
S9UJZ3_Angomonas_deanei
K3WA51_Pythium_ultimum
A0A0C9W3B1_Sphaerobolus_stellatus
A0A0G4GCL4_Vitrella_brassicaformis
A0A0G4GKY6_Vitrella_brassicaformis
D7M0W1_Arabidopsis_lyrata
R0GRC1_Capsella_rubella
P46011_Arabidopsis_thaliana
I3SLY0_Lotus_japonicus
V4SEX2_Citrus_clementina
L0D9V2_Singulisphaera_acidiphila
A0LKP2_Syntrophobacter_fumaroxidans
E3QYC5_Colletotrichum_graminicola
H1W2G0_Colletotrichum_higginsianum
B8MN39_Talaromyces_stipitatus
A0A0E9NB51_Saitoella_complicata
D6V0N1_Afipia_sp.
A0A069PL19_Burkholderia_glathei
D1C8L7_Sphaerobacter_thermophilus
Fcan01_03297_PA
I0YSV4_Coccomyxa_subellipsoidea
Q3IUK0_Natronomonas_pharaonis
M1XNX1_Natronomonas_moolapensis
A0A0B5H4U4_Haloarcula_sp.
M0KAS6_Haloarcula_amylolytica
L9W302_Natronorubrum_sulfidifaciens
V5HET8_Ixodes_ricinus
B7PT85_Ixodes_scapularis
B7PD10_Ixodes_scapularis
A0A087T0Y0_Stegodyphus_mimosarum
L7M2A5_Rhipicephalus_pulchellus
0.4

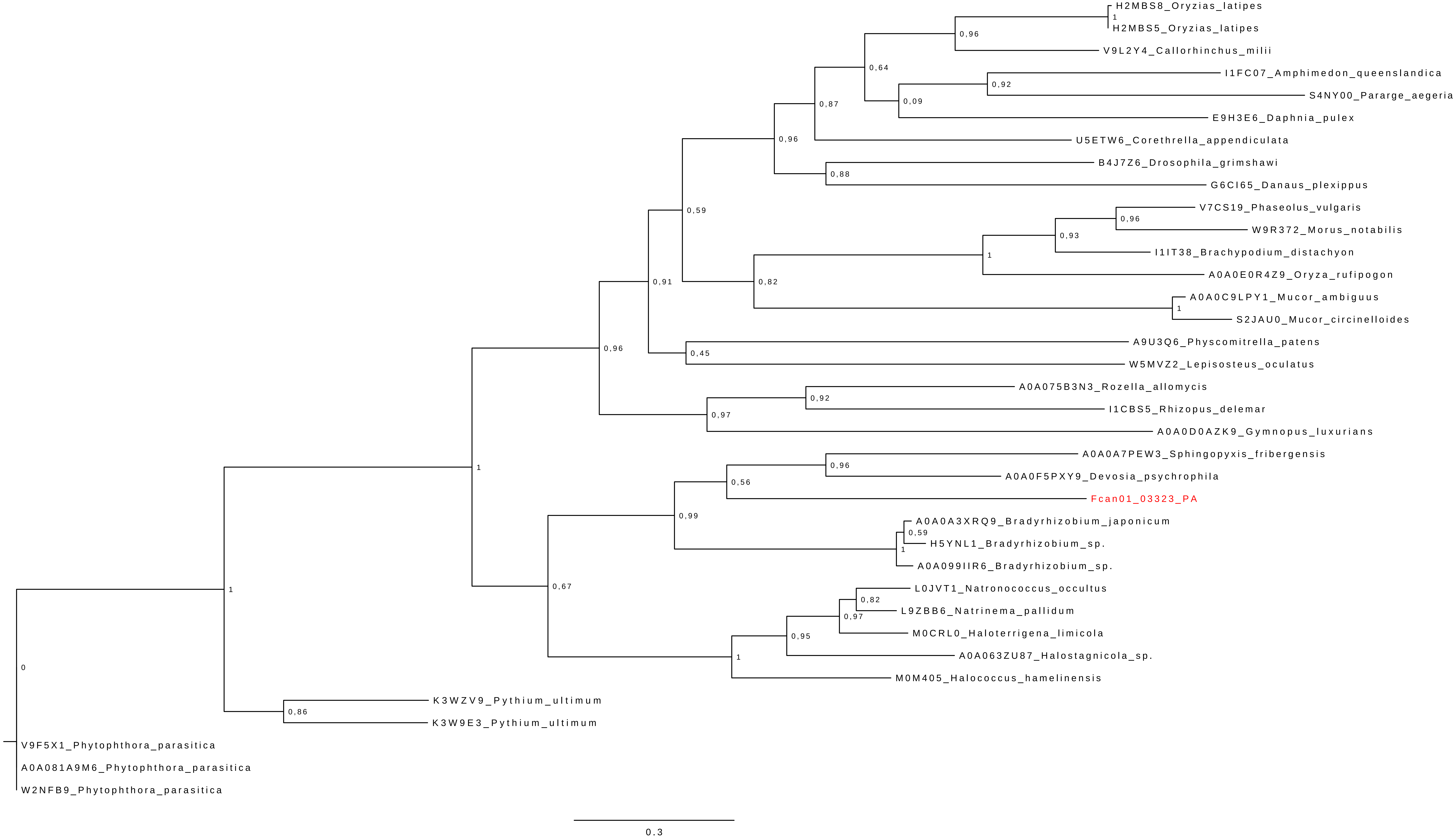
H2MBS8_Oryzias_latipes
H2MBS5_Oryzias_latipes
V9L2Y4_Callorhinchus_milii
I1FC07_Amphimedon_queenslandica
S4NY00_Pararge_aegeria
E9H3E6_Daphnia_pulex
U5ETW6_Corethrella_appendiculata
B4J7Z6_Drosophila_grimshawi
G6CI65_Danaus_plexippus
V7CS19_Phaseolus_vulgaris
W9R372_Morus_notabilis
I1IT38_Brachypodium_distachyon
A0A0E0R4Z9_Oryza_rufipogon
A0A0C9LPY1_Mucor_ambiguus
S2JAU0_Mucor_circinelloides
A9U3Q6_Physcomitrella_patens
W5MVZ2_Lepisosteus_oculatus
A0A075B3N3_Rozella_allomycis
I1CBS5_Rhizopus_delemar
A0A0D0AZK9_Gymnopus_luxurians
A0A0A7PEW3_Sphingopyxis_fribergensis
A0A0F5PXY9_Devosia_psychrophila
Fcan01_03323_PA
A0A0A3XRQ9_Bradyrhizobium_japonicum
H5YNL1_Bradyrhizobium_sp.
A0A099IIR6_Bradyrhizobium_sp.
L0JVT1_Natronococcus_occultus
L9ZBB6_Natrinema_pallidum
M0CRL0_Haloterrigena_limicola
A0A063ZU87_Halostagnicola_sp.
M0M405_Halococcus_hamelinensis
K3WZV9_Pythium_ultimum
K3W9E3_Pythium_ultimum
V9F5X1_Phytophthora_parasitica
A0A081A9M6_Phytophthora_parasitica
W2NFB9_Phytophthora_parasitica
0.3

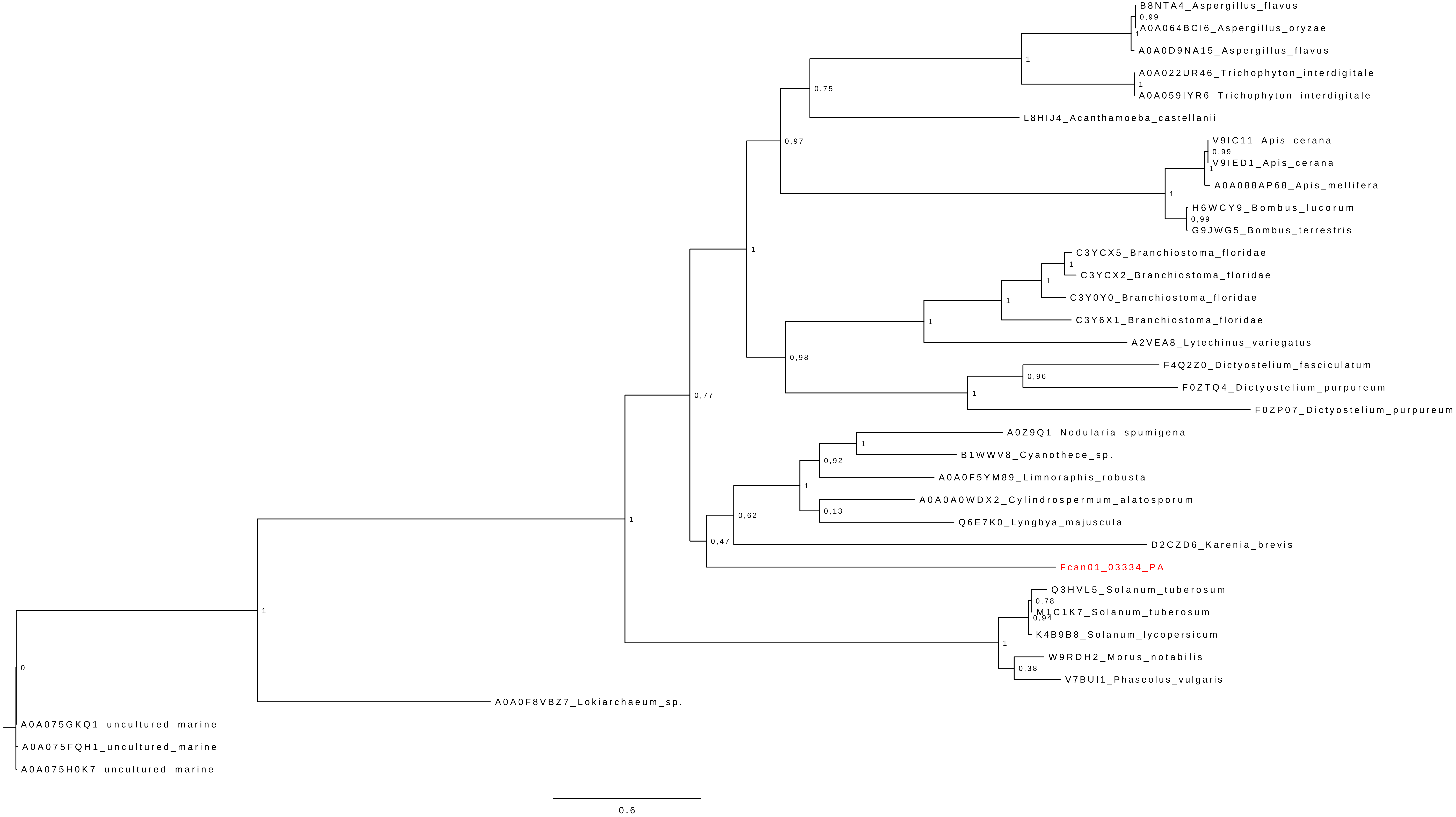
B8NTA4_Aspergillus_flavus
0,99
A0A064BCI6_Aspergillus_oryzae
1
A0A0D9NA15_Aspergillus_flavus
1
A0A022UR46_Trichophyton_interdigitale
1
A0A059IYR6_Trichophyton_interdigitale
0,75
L8HIJ4_Acanthamoeba_castellanii
0,97
V9IC11_Apis_cerana
0,99
V9IED1_Apis_cerana
1
A0A088AP68_Apis_mellifera
1
H6WCY9_Bombus_lucorum
0,99
G9JWG5_Bombus_terrestris
1
C3YCX5_Branchiostoma_floridae
1
C3YCX2_Branchiostoma_floridae
1
C3Y0Y0_Branchiostoma_floridae
1
C3Y6X1_Branchiostoma_floridae
1
A2VEA8_Lytechinus_variegatus
0,98
F4Q2Z0_Dictyostelium_fasciculatum
0,96
F0ZTQ4_Dictyostelium_purpureum
1
F0ZP07_Dictyostelium_purpureum
0,77
A0Z9Q1_Nodularia_spumigena
1
B1WWV8_Cyanothece_sp.
0,92
A0A0F5YM89_Limnoraphis_robusta
1
A0A0A0WDX2_Cylindrospermum_alatosporum
0,13
Q6E7K0_Lyngbya_majuscula
0,62
D2CZD6_Karenia_brevis
0,47
Fcan01_03334_PA
1
Q3HVL5_Solanum_tuberosum
0,78
M1C1K7_Solanum_tuberosum
0,94
K4B9B8_Solanum_lycopersicum
1
W9RDH2_Morus_notabilis
0,38
V7BUI1_Phaseolus_vulgaris
1
0
A0A0F8VBZ7_Lokiarchaeum_sp.
A0A075GKQ1_uncultured_marine
A0A075FQH1_uncultured_marine
A0A075H0K7_uncultured_marine
0.6

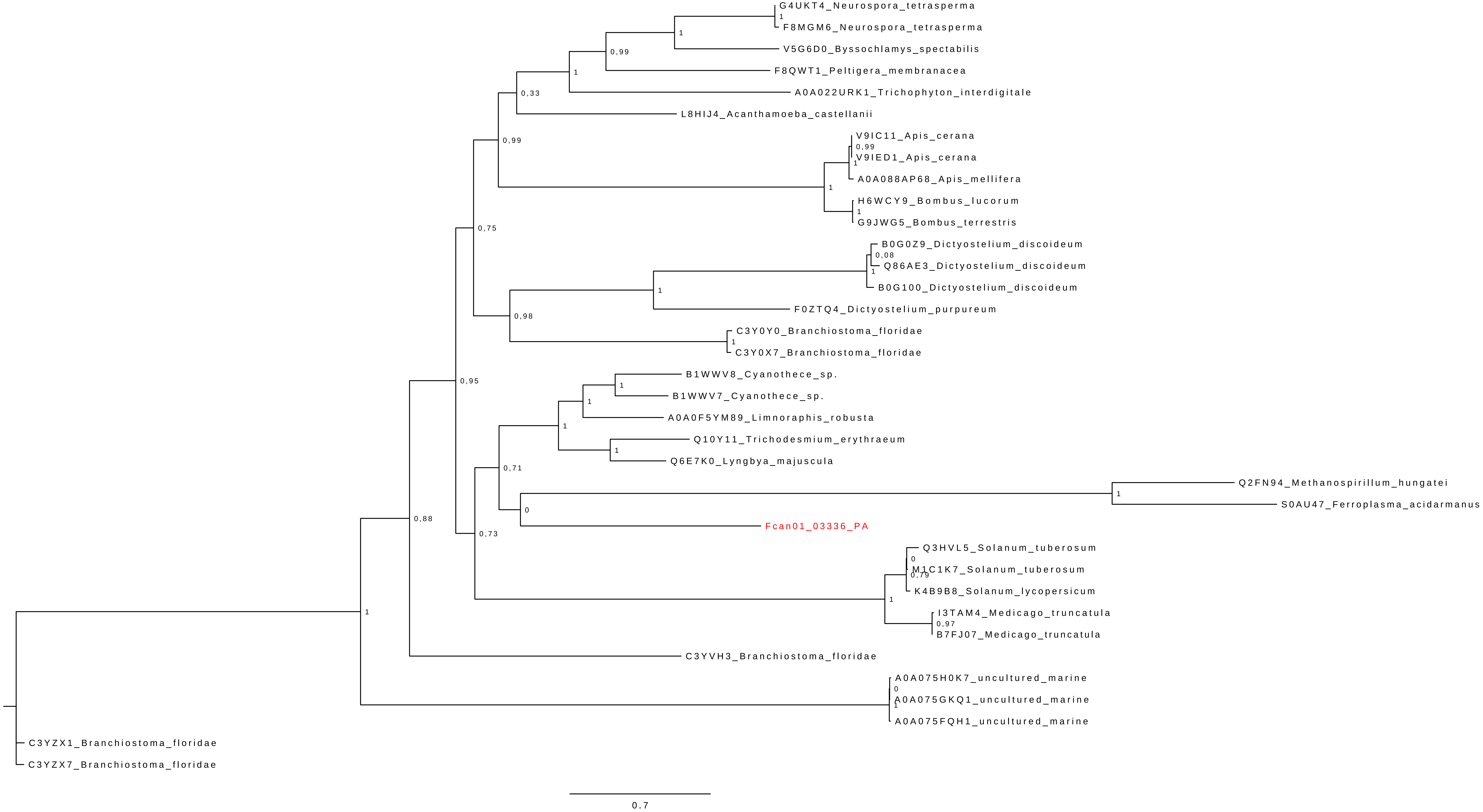

G4UKT4_Neurospora_tetrasperma
F8MGM6_Neurospora_tetrasperma
V5G6D0_Byssochlamys_spectabilis
F8QWT1_Peltigera_membranacea
A0A022URK1_Trichophyton_interdigitale
L8HIJ4_Acanthamoeba_castellanii
V9IC11_Apis_cerana
V9IED1_Apis_cerana
A0A088AP68_Apis_mellifera
H6WCY9_Bombus_lucorum
G9JWG5_Bombus_terrestris
B0G0Z9_Dictyostelium_discoideum
Q86AE3_Dictyostelium_discoideum
B0G100_Dictyostelium_discoideum
F0ZTQ4_Dictyostelium_purpureum
C3Y0Y0_Branchiostoma_floridae
C3Y0X7_Branchiostoma_floridae
B1WWV8_Cyanothece_sp.
B1WWV7_Cyanothece_sp.
A0A0F5YM89_Limnoraphis_robusta
Q10Y11_Trichodesmium_erythraeum
Q6E7K0_Lyngbya_majuscula
Q2FN94_Methanospirillum_hungatei
S0AU47_Ferroplasma_acidarmanus
Fcan01_03336_PA
Q3HVL5_Solanum_tuberosum
M1C1K7_Solanum_tuberosum
K4B9B8_Solanum_lycopersicum
I3TAM4_Medicago_truncatula
B7FJ07_Medicago_truncatula
C3YVH3_Branchiostoma_floridae
A0A075H0K7_uncultured_marine
A0A075GKQ1_uncultured_marine
A0A075FQH1_uncultured_marine
C3YZX1_Branchiostoma_floridae
C3YZX7_Branchiostoma_floridae
0.7

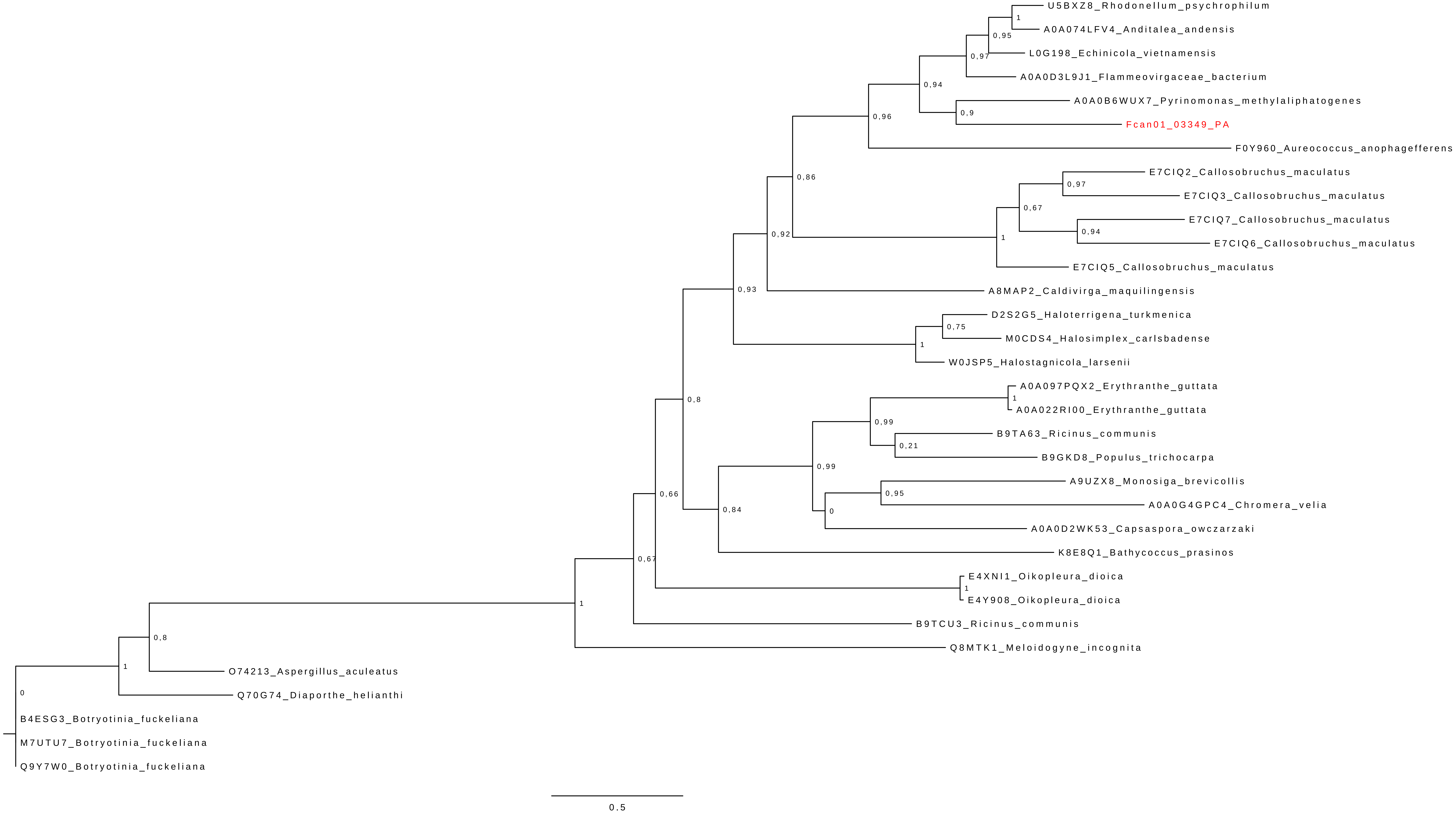
U5BXZ8_Rhodonellum_psychrophilum
1
A0A074LFV4_Anditalea_andensis
0,95
L0G198_Echinicola_vietnamensis
0,97
A0A0D3L9J1_Flammeovirgaceae_bacterium
0,94
A0A0B6WUX7_Pyrinomonas_methylaliphatogenes
0,9
Fcan01_03349_PA
0,96
F0Y960_Aureococcus_anophagefferens
E7CIQ2_Callosobruchus_maculatus
0,97
E7CIQ3_Callosobruchus_maculatus
0,67
E7CIQ7_Callosobruchus_maculatus
0,94
E7CIQ6_Callosobruchus_maculatus
0,86
1
0,92
E7CIQ5_Callosobruchus_maculatus
0,93
A8MAP2_Caldivirga_maquilingensis
D2S2G5_Haloterrigena_turkmenica
0,75
M0CDS4_Halosimplex_carlsbadense
1
W0JSP5_Halostagnicola_larsenii
A0A097PQX2_Erythranthe_guttata
0,8
1
A0A022RI00_Erythranthe_guttata
0,99
B9TA63_Ricinus_communis
0,21
B9GKD8_Populus_trichocarpa
0,99
A9UZX8_Monosiga_brevicollis
0,95
0,66
A0A0G4GPC4_Chromera_velia
0,84
0
A0A0D2WK53_Capsaspora_owczarzaki
K8E8Q1_Bathycoccus_prasinos
0,67
E4XNI1_Oikopleura_dioica
1
E4Y908_Oikopleura_dioica
1
B9TCU3_Ricinus_communis
Q8MTK1_Meloidogyne_incognita
0,8
O74213_Aspergillus_aculeatus
1
Q70G74_Diaporthe_helianthi
0
B4ESG3_Botryotinia_fuckeliana
M7UTU7_Botryotinia_fuckeliana
Q9Y7W0_Botryotinia_fuckeliana
0.5

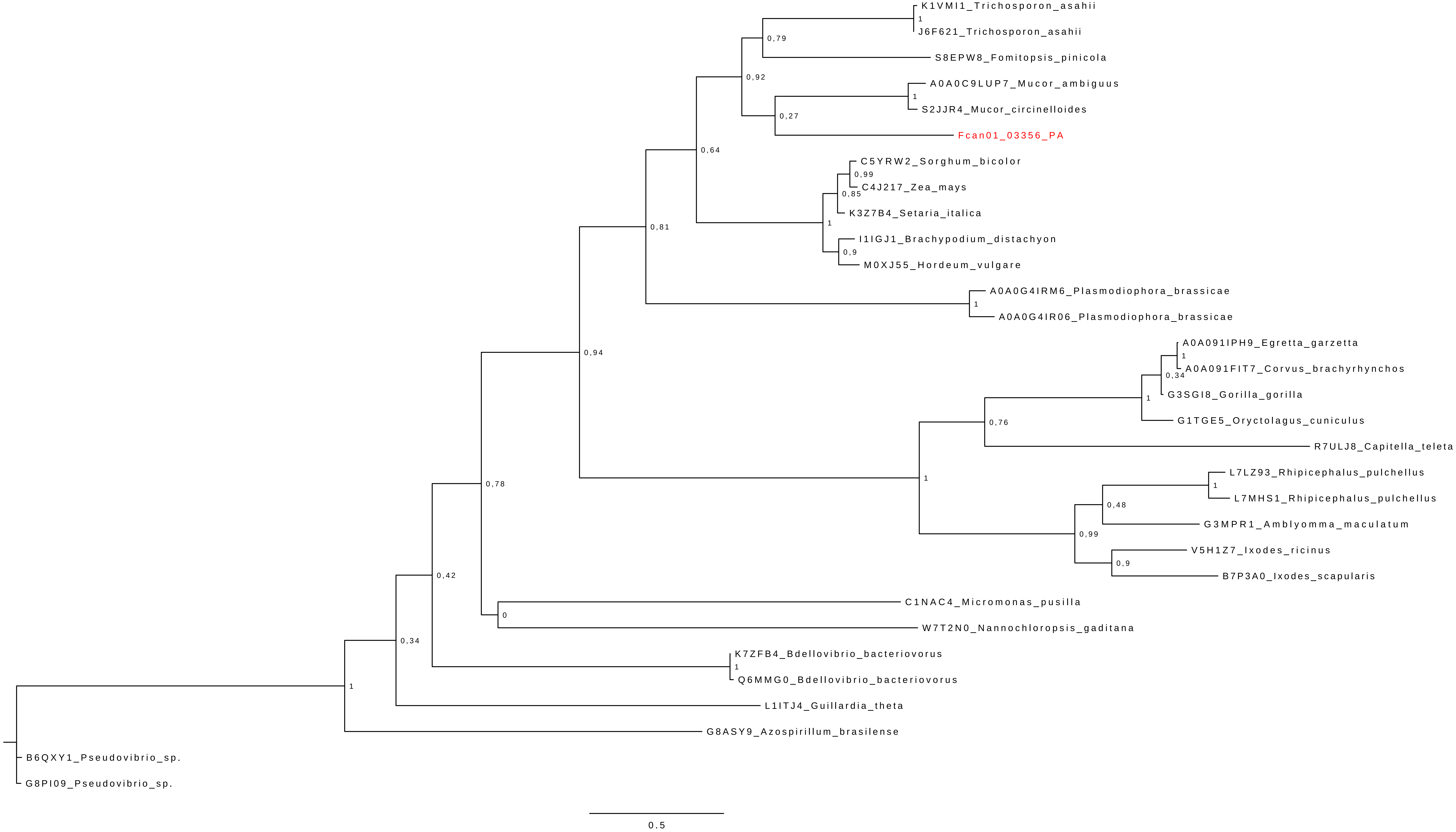
K1VMI1_Trichosporon_asahii
1
J6F621_Trichosporon_asahii
0,79
S8EPW8_Fomitopsis_pinicola
0,92
A0A0C9LUP7_Mucor_ambiguus
1
S2JJR4_Mucor_circinelloides
0,27
Fcan01_03356_PA
0,64
C5YRW2_Sorghum_bicolor
0,99
C4J217_Zea_mays
0,85
K3Z7B4_Setaria_italica
1
I1IGJ1_Brachypodium_distachyon
0,9
M0XJ55_Hordeum_vulgare
0,81
A0A0G4IRM6_Plasmodiophora_brassicae
1
A0A0G4IR06_Plasmodiophora_brassicae
0,94
A0A091IPH9_Egretta_garzetta
1
A0A091FIT7_Corvus_brachyrhynchos
0,34
G3SGI8_Gorilla_gorilla
1
G1TGE5_Oryctolagus_cuniculus
0,76
R7ULJ8_Capitella_teleta
L7LZ93_Rhipicephalus_pulchellus
1
1
L7MHS1_Rhipicephalus_pulchellus
0,48
G3MPR1_Amblyomma_maculatum
0,99
V5H1Z7_Ixodes_ricinus
0,9
B7P3A0_Ixodes_scapularis
0,78
C1NAC4_Micromonas_pusilla
0
W7T2N0_Nannochloropsis_gaditana
0,42
K7ZFB4_Bdellovibrio_bacteriovorus
1
Q6MMG0_Bdellovibrio_bacteriovorus
0,34
L1ITJ4_Guillardia_theta
1
G8ASY9_Azospirillum_brasilense
B6QXY1_Pseudovibrio_sp.
G8PI09_Pseudovibrio_sp.
0.5

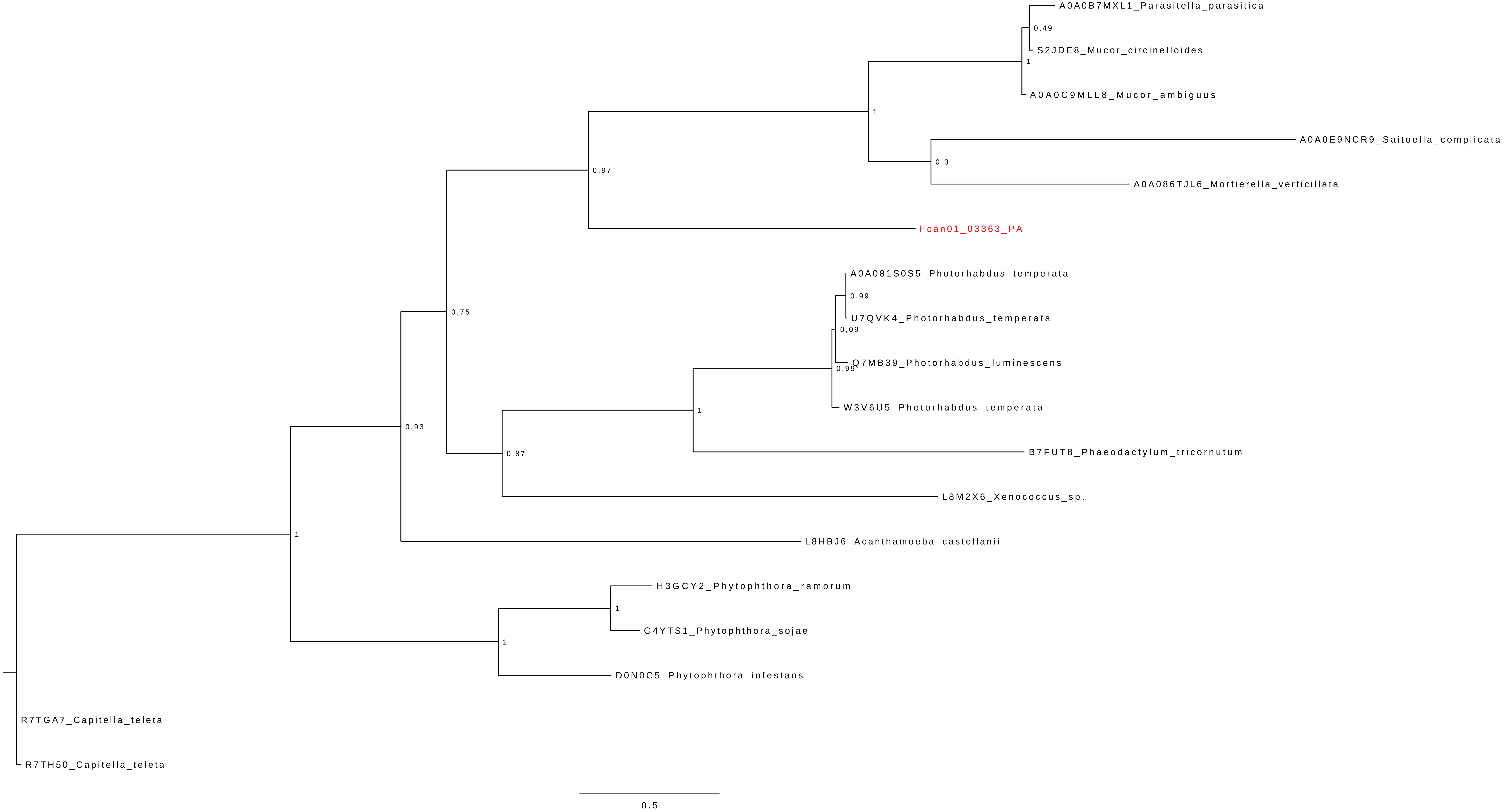
A0A0B7MXL1_Parasitella_parasitica
0,49
S2JDE8_Mucor_circinelloides
1
A0A0C9MLL8_Mucor_ambiguus
1
A0A0E9NCR9_Saitoella_complicata
0,3
A0A086TJL6_Mortierella_verticillata
0,97
Fcan01_03363_PA
A0A081S0S5_Photorhabdus_temperata
0,99
U7QVK4_Photorhabdus_temperata
0,09
Q7MB39_Photorhabdus_luminescens
0,99
W3V6U5_Photorhabdus_temperata
0,75
1
B7FUT8_Phaeodactylum_tricornutum
0,93
0,87
L8M2X6_Xenococcus_sp.
1
L8HBJ6_Acanthamoeba_castellanii
H3GCY2_Phytophthora_ramorum
1
G4YTS1_Phytophthora_sojae
1
D0N0C5_Phytophthora_infestans
R7TGA7_Capitella_teleta
R7TH50_Capitella_teleta
0.5

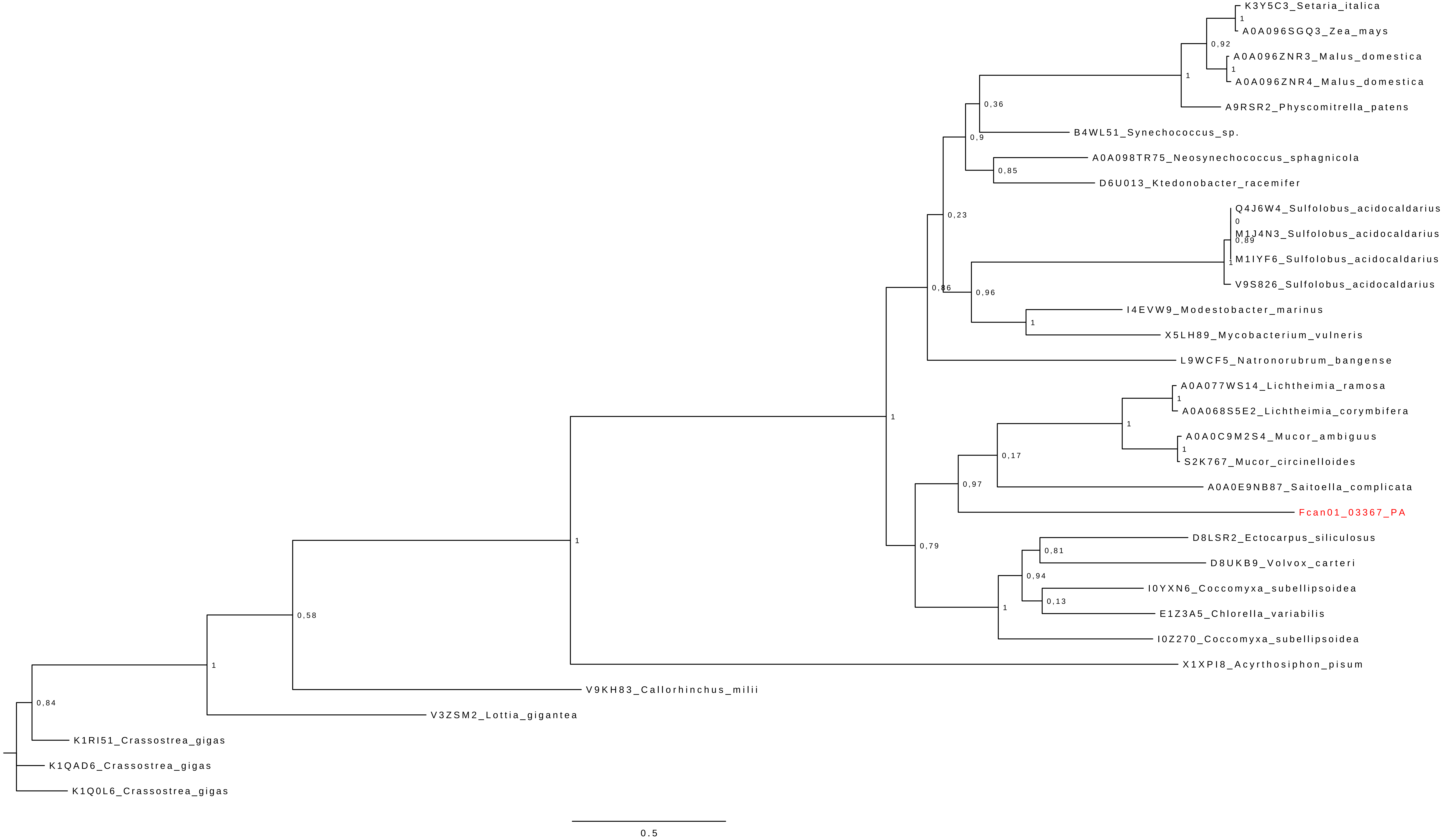
K3Y5C3_Setaria_italica
A0A096SGQ3_Zea_mays
A0A096ZNR3_Malus_domestica
A0A096ZNR4_Malus_domestica
A9RSR2_Physcomitrella_patens
B4WL51_Synechococcus_sp.
A0A098TR75_Neosynechococcus_sphagnicola
D6U013_Ktedonobacter_racemifer
Q4J6W4_Sulfolobus_acidocaldarius
M1J4N3_Sulfolobus_acidocaldarius
M1IYF6_Sulfolobus_acidocaldarius
V9S826_Sulfolobus_acidocaldarius
I4EVW9_Modestobacter_marinus
X5LH89_Mycobacterium_vulneris
L9WCF5_Natronorubrum_bangense
A0A077WS14_Lichtheimia_ramosa
A0A068S5E2_Lichtheimia_corymbifera
A0A0C9M2S4_Mucor_ambiguus
S2K767_Mucor_circinelloides
A0A0E9NB87_Saitoella_complicata
Fcan01_03367_PA
D8LSR2_Ectocarpus_siliculosus
D8UKB9_Volvox_carteri
I0YXN6_Coccomyxa_subellipsoidea
E1Z3A5_Chlorella_variabilis
I0Z270_Coccomyxa_subellipsoidea
X1XPI8_Acyrthosiphon_pisum
V9KH83_Callorhinchus_milii
V3ZSM2_Lottia_gigantea
K1RI51_Crassostrea_gigas
K1QAD6_Crassostrea_gigas
K1Q0L6_Crassostrea_gigas
0.5

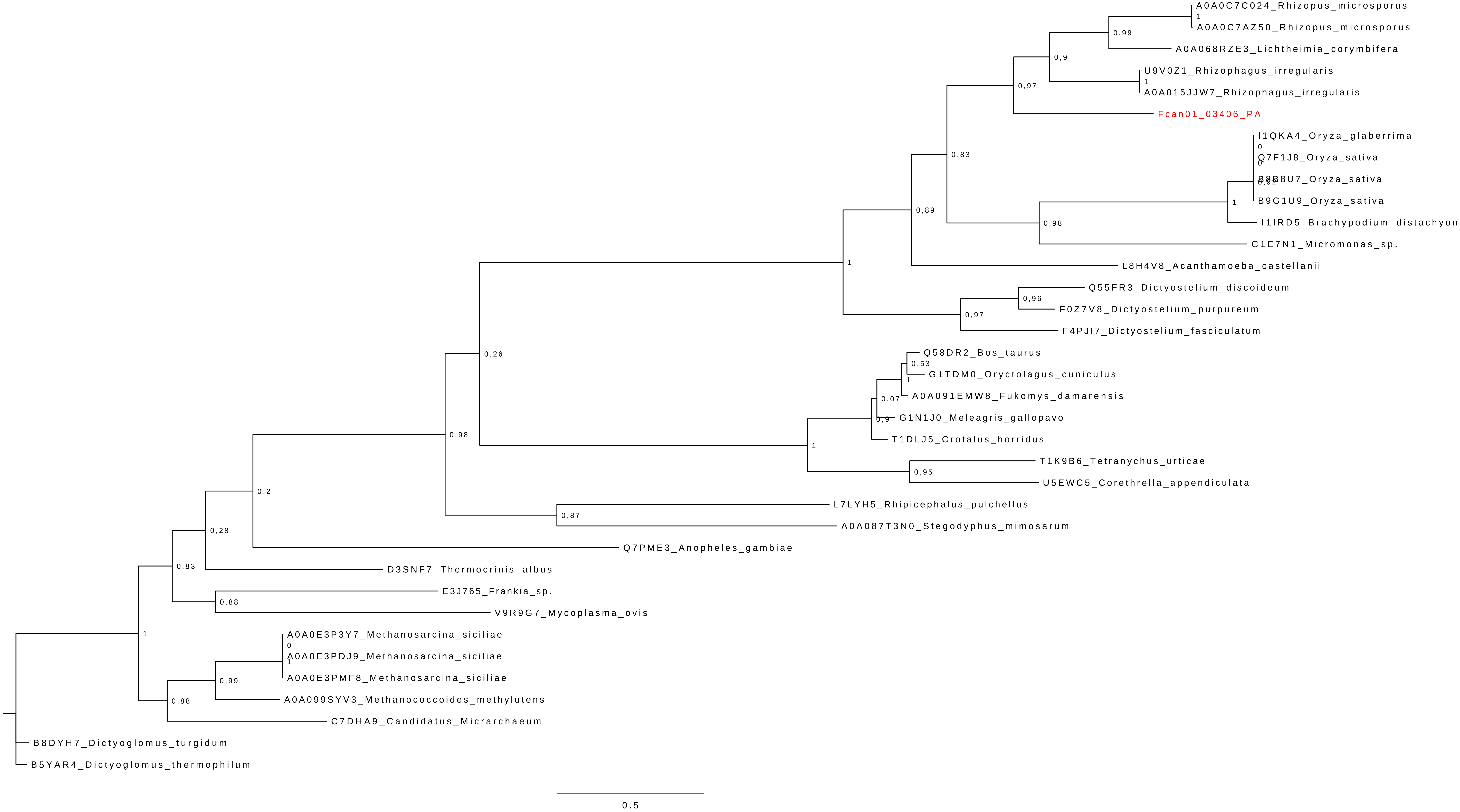
A0A0C7C024_Rhizopus_microsporus
A0A0C7AZ50_Rhizopus_microsporus
A0A068RZE3_Lichtheimia_corymbifera
U9V0Z1_Rhizophagus_irregularis
A0A015JJW7_Rhizophagus_irregularis
Fcan01_03406_PA
I1QKA4_Oryza_glaberrima
Q7F1J8_Oryza_sativa
B8B8U7_Oryza_sativa
B9G1U9_Oryza_sativa
I1IRD5_Brachypodium_distachyon
C1E7N1_Micromonas_sp.
L8H4V8_Acanthamoeba_castellanii
Q55FR3_Dictyostelium_discoideum
F0Z7V8_Dictyostelium_purpureum
F4PJI7_Dictyostelium_fasciculatum
Q58DR2_Bos_taurus
G1TDM0_Oryctolagus_cuniculus
A0A091EMW8_Fukomys_damarensis
G1N1J0_Meleagris_gallopavo
T1DLJ5_Crotalus_horridus
T1K9B6_Tetranychus_urticae
U5EWC5_Corethrella_appendiculata
L7LYH5_Rhipicephalus_pulchellus
A0A087T3N0_Stegodyphus_mimosarum
Q7PME3_Anopheles_gambiae
D3SNF7_Thermocrinis_albus
E3J765_Frankia_sp.
V9R9G7_Mycoplasma_ovis
A0A0E3P3Y7_Methanosarcina_siciliae
A0A0E3PDJ9_Methanosarcina_siciliae
A0A0E3PMF8_Methanosarcina_siciliae
A0A099SYV3_Methanococcoides_methylutens
C7DHA9_Candidatus_Micrarchaeum
B8DYH7_Dictyoglomus_turgidum
B5YAR4_Dictyoglomus_thermophilum
0.5

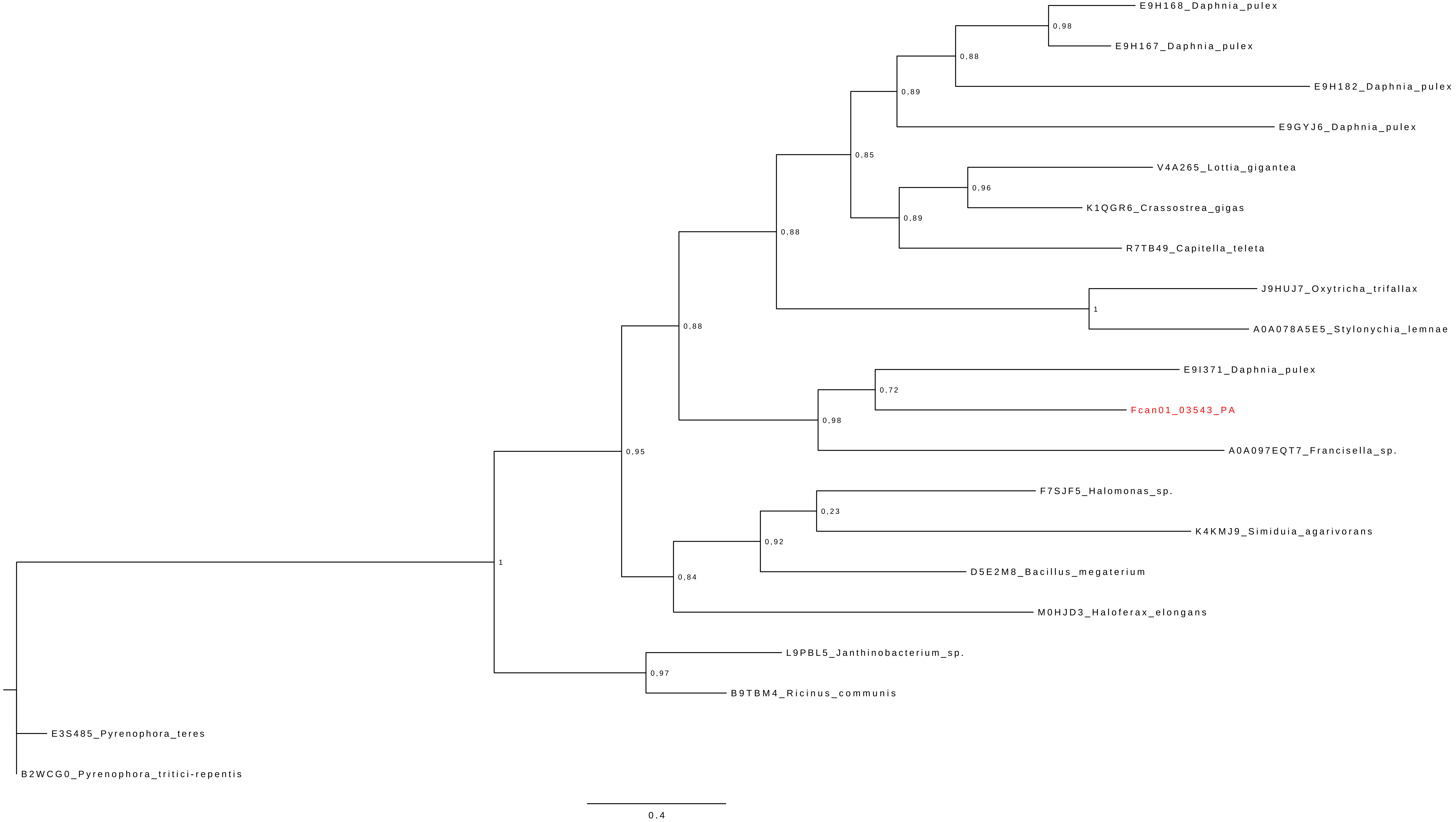

E9H168_Daphnia_pulex
0,98
E9H167_Daphnia_pulex
0,88
E9H182_Daphnia_pulex
0,89
E9GYJ6_Daphnia_pulex
0,85
V4A265_Lottia_gigantea
0,96
K1QGR6_Crassostrea_gigas
0,89
R7TB49_Capitella_teleta
0,88
J9HUJ7_Oxytricha_trifallax
1
A0A078A5E5_Stylonychia_lemnae
0,88
E9I371_Daphnia_pulex
0,72
Fcan01_03543_PA
0,98
A0A097EQT7_Francisella_sp.
0,95
F7SJF5_Halomonas_sp.
0,23
K4KMJ9_Simiduia_agarivorans
0,92
D5E2M8_Bacillus_megaterium
0,84
M0HJD3_Haloferax_elongans
1
L9PBL5_Janthinobacterium_sp.
0,97
B9TBM4_Ricinus_communis
E3S485_Pyrenophora_teres
B2WCG0_Pyrenophora_tritici-repentis
0.4

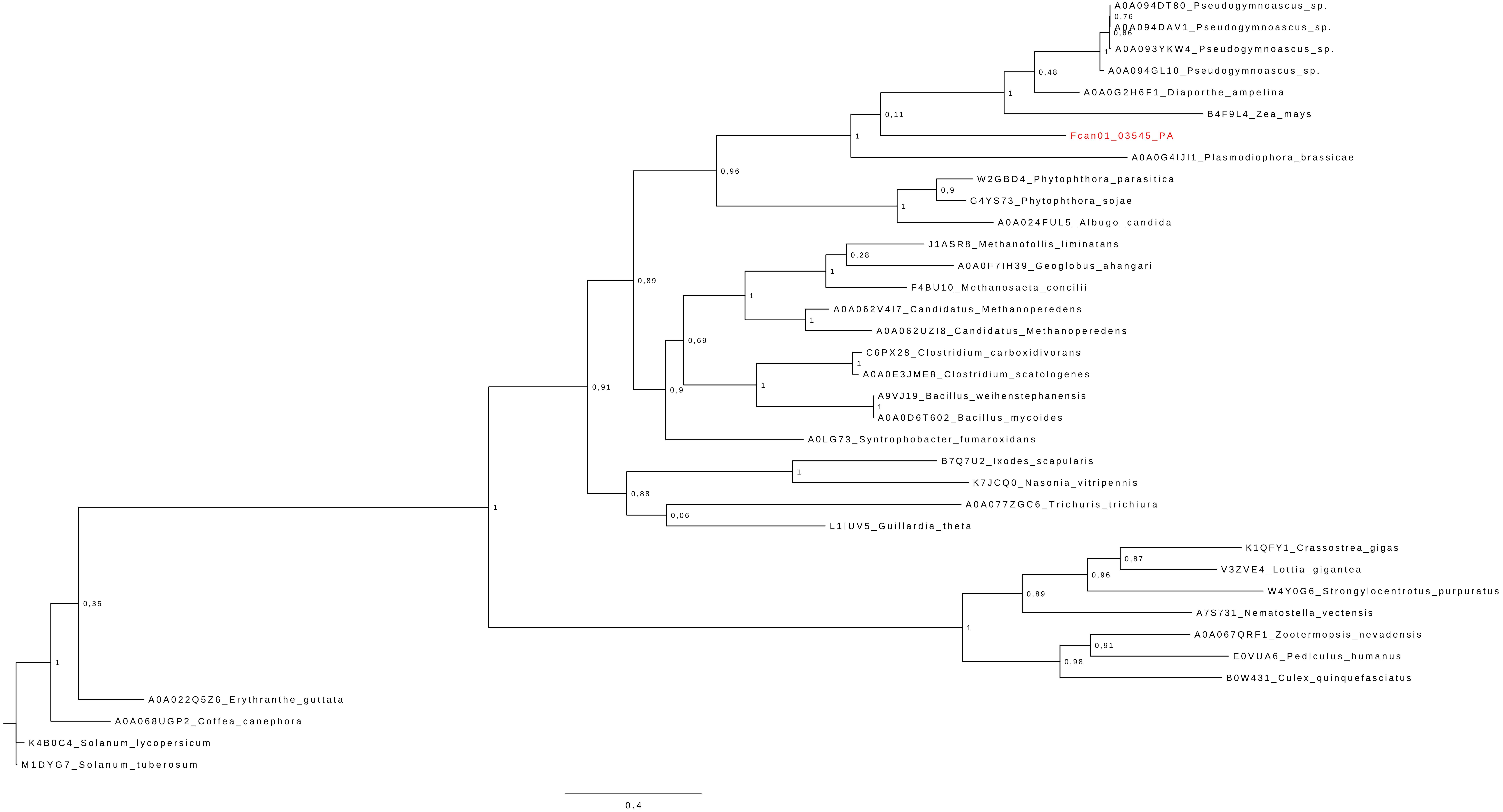
A0A094DT80_Pseudogymnoascus_sp.
0,76
A0A094DAV1_Pseudogymnoascus_sp.
0,86
A0A093YKW4_Pseudogymnoascus_sp.
1
A0A094GL10_Pseudogymnoascus_sp.
0,48
A0A0G2H6F1_Diaporthe_ampelina
1
B4F9L4_Zea_mays
0,11
Fcan01_03545_PA
1
A0A0G4IJI1_Plasmodiophora_brassicae
0,96
W2GBD4_Phytophthora_parasitica
0,9
G4YS73_Phytophthora_sojae
1
A0A024FUL5_Albugo_candida
J1ASR8_Methanofollis_liminatans
0,28
A0A0F7IH39_Geoglobus_ahangari
1
F4BU10_Methanosaeta_concilii
0,89
1
A0A062V4I7_Candidatus_Methanoperedens
1
A0A062UZI8_Candidatus_Methanoperedens
0,69
C6PX28_Clostridium_carboxidivorans
1
A0A0E3JME8_Clostridium_scatologenes
0,91
0,9
1
A9VJ19_Bacillus_weihenstephanensis
1
A0A0D6T602_Bacillus_mycoides
A0LG73_Syntrophobacter_fumaroxidans
B7Q7U2_Ixodes_scapularis
1
K7JCQ0_Nasonia_vitripennis
0,88
A0A077ZGC6_Trichuris_trichiura
1
0,06
L1IUV5_Guillardia_theta
K1QFY1_Crassostrea_gigas
0,87
V3ZVE4_Lottia_gigantea
0,96
W4Y0G6_Strongylocentrotus_purpuratus
0,89
A7S731_Nematostella_vectensis
0,35
1
A0A067QRF1_Zootermopsis_nevadensis
0,91
E0VUA6_Pediculus_humanus
0,98
B0W431_Culex_quinquefasciatus
1
A0A022Q5Z6_Erythranthe_guttata
A0A068UGP2_Coffea_canephora
K4B0C4_Solanum_lycopersicum
M1DYG7_Solanum_tuberosum
0.4

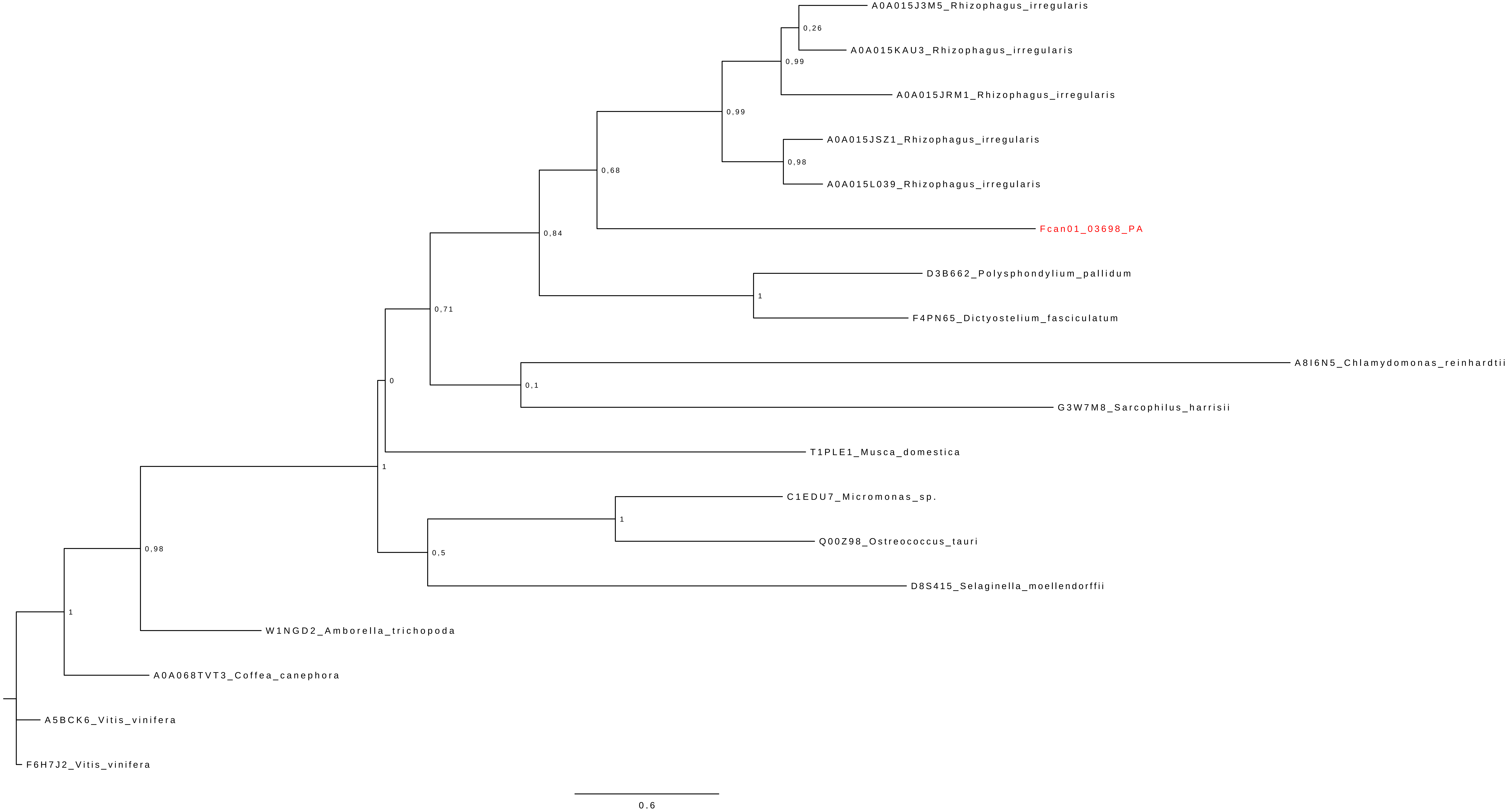
A0A015J3M5_Rhizophagus_irregularis
0,26
A0A015KAU3_Rhizophagus_irregularis
0,99
A0A015JRM1_Rhizophagus_irregularis
0,99
A0A015JSZ1_Rhizophagus_irregularis
0,98
A0A015L039_Rhizophagus_irregularis
0,68
Fcan01_03698_PA
0,84
D3B662_Polysphondylium_pallidum
1
F4PN65_Dictyostelium_fasciculatum
0,71
A8I6N5_Chlamydomonas_reinhardtii
0,1
G3W7M8_Sarcophilus_harrisii
0
T1PLE1_Musca_domestica
1
C1EDU7_Micromonas_sp.
1
Q00Z98_Ostreococcus_tauri
0,5
D8S415_Selaginella_moellendorffii
0,98
W1NGD2_Amborella_trichopoda
1
A0A068TVT3_Coffea_canephora
A5BCK6_Vitis_vinifera
F6H7J2_Vitis_vinifera
0.6

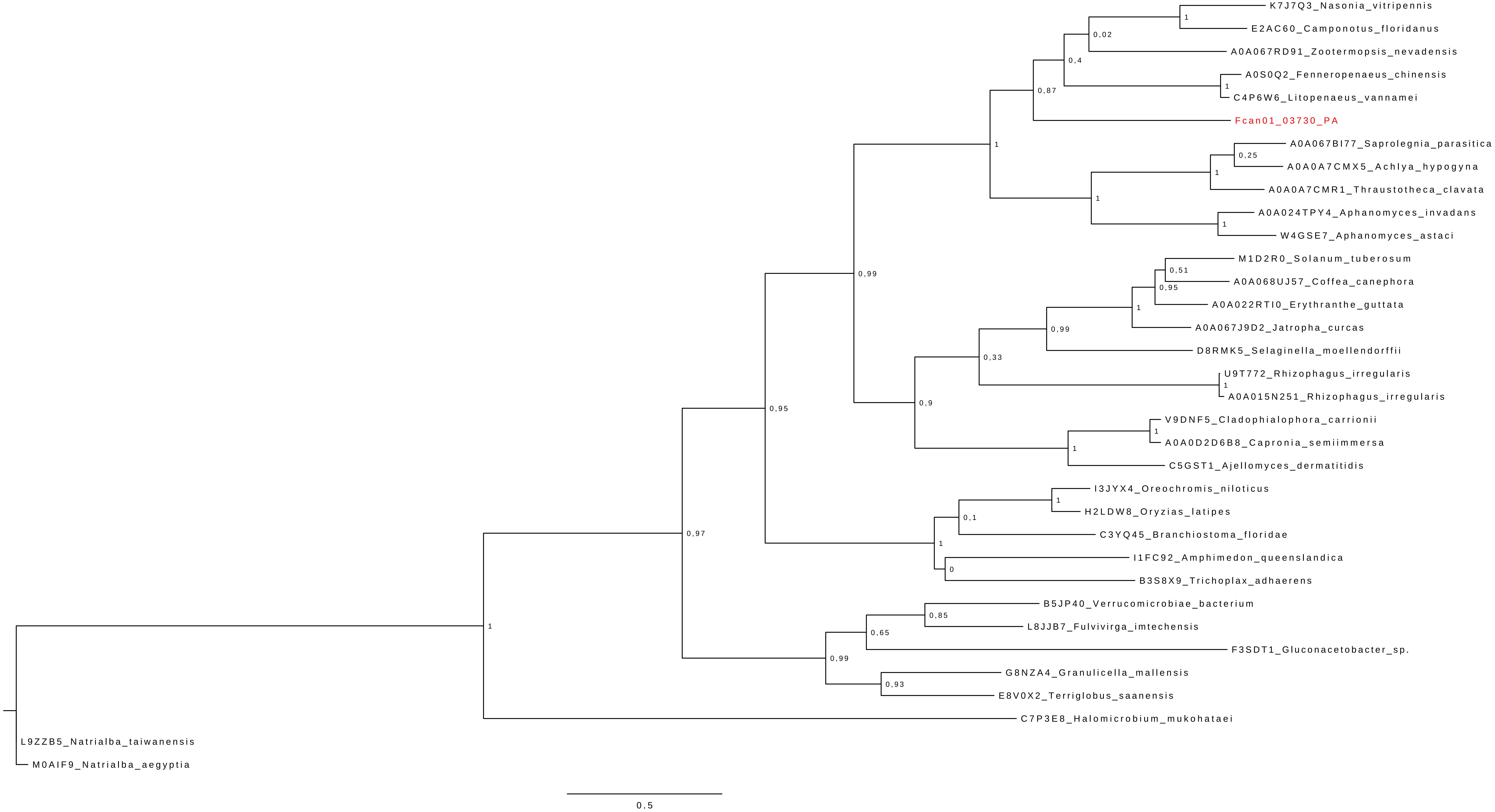

K7J7Q3_Nasonia_vitripennis
E2AC60_Camponotus_floridanus
A0A067RD91_Zootermopsis_nevadensis
A0S0Q2_Fenneropenaeus_chinensis
C4P6W6_Litopenaeus_vannamei
Fcan01_03730_PA
A0A067BI77_Saprolegnia_parasitica
A0A0A7CMX5_Achlya_hypogyna
A0A0A7CMR1_Thraustotheca_clavata
A0A024TPY4_Aphanomyces_invadans
W4GSE7_Aphanomyces_astaci
M1D2R0_Solanum_tuberosum
A0A068UJ57_Coffea_canephora
A0A022RTI0_Erythranthe_guttata
A0A067J9D2_Jatropha_curcas
D8RMK5_Selaginella_moellendorffii
U9T772_Rhizophagus_irregularis
A0A015N251_Rhizophagus_irregularis
V9DNF5_Cladophialophora_carrionii
A0A0D2D6B8_Capronia_semiimmersa
C5GST1_Ajellomyces_dermatitidis
I3JYX4_Oreochromis_niloticus
H2LDW8_Oryzias_latipes
C3YQ45_Branchiostoma_floridae
I1FC92_Amphimedon_queenslandica
B3S8X9_Trichoplax_adhaerens
B5JP40_Verrucomicrobiae_bacterium
L8JJB7_Fulvivirga_imtechensis
F3SDT1_Gluconacetobacter_sp.
G8NZA4_Granulicella_mallensis
E8V0X2_Terriglobus_saanensis
C7P3E8_Halomicrobium_mukohataei
L9ZZB5_Natrialba_taiwanensis
M0AIF9_Natrialba_aegyptia
1
0,02
0,4
1
0,87
1
0,25
1
1
1
0,99
0,51
0,95
1
0,99
0,33
1
0,9
1
1
0,95
1
0,1
1
0
0,97
0,85
0,65
0,99
0,93
1
0.5

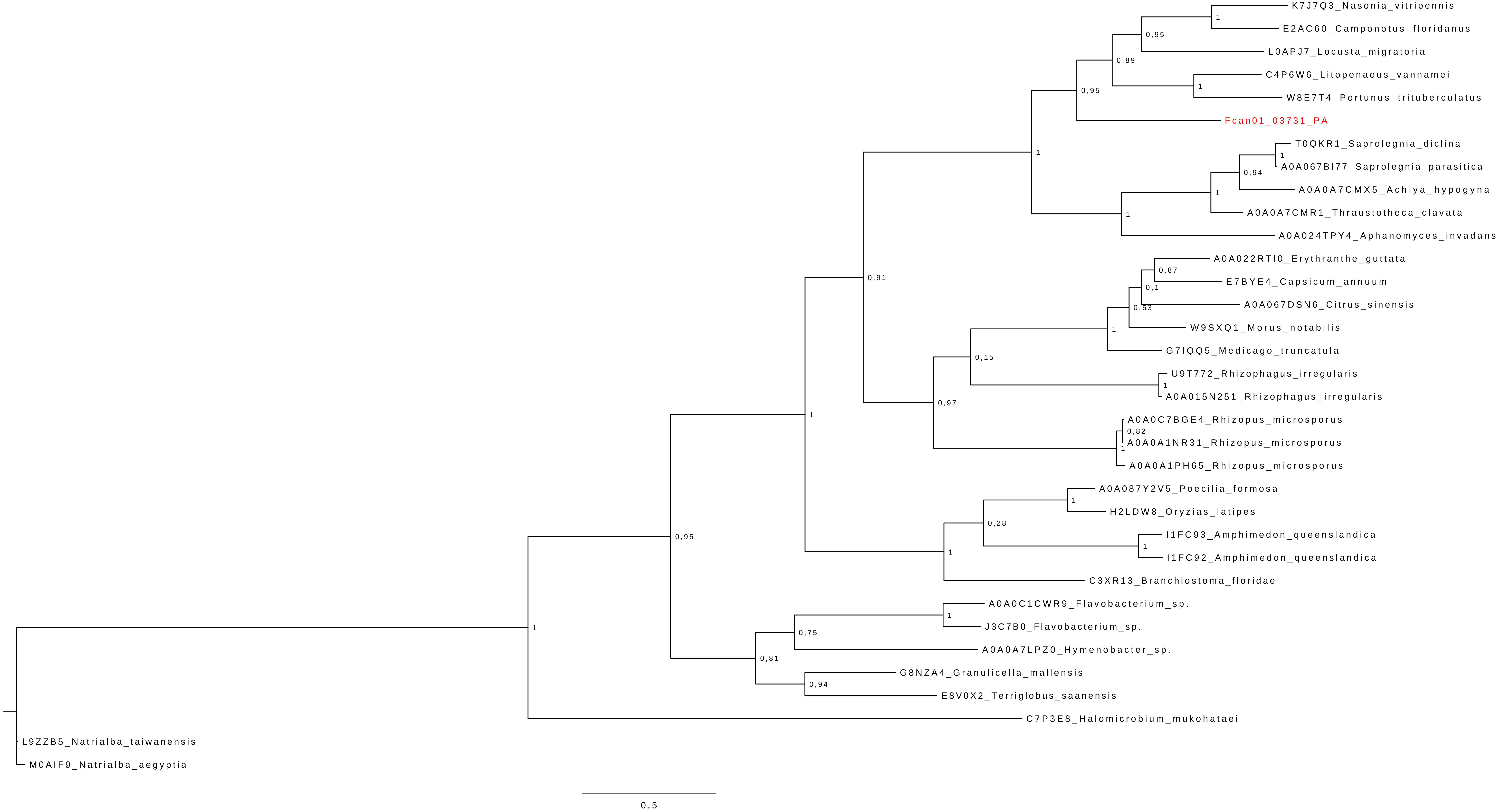
K7J7Q3_Nasonia_vitripennis
E2AC60_Camponotus_floridanus
L0APJ7_Locusta_migratoria
C4P6W6_Litopenaeus_vannamei
W8E7T4_Portunus_trituberculatus
Fcan01_03731_PA
T0QKR1_Saprolegnia_diclina
A0A067BI77_Saprolegnia_parasitica
A0A0A7CMX5_Achlya_hypogyna
A0A0A7CMR1_Thraustotheca_clavata
A0A024TPY4_Aphanomyces_invadans
A0A022RTI0_Erythranthe_guttata
E7BYE4_Capsicum_annuum
A0A067DSN6_Citrus_sinensis
W9SXQ1_Morus_notabilis
G7IQQ5_Medicago_truncatula
U9T772_Rhizophagus_irregularis
A0A015N251_Rhizophagus_irregularis
A0A0C7BGE4_Rhizopus_microsporus
A0A0A1NR31_Rhizopus_microsporus
A0A0A1PH65_Rhizopus_microsporus
A0A087Y2V5_Poecilia_formosa
H2LDW8_Oryzias_latipes
I1FC93_Amphimedon_queenslandica
I1FC92_Amphimedon_queenslandica
C3XR13_Branchiostoma_floridae
A0A0C1CWR9_Flavobacterium_sp.
J3C7B0_Flavobacterium_sp.
A0A0A7LPZ0_Hymenobacter_sp.
G8NZA4_Granulicella_mallensis
E8V0X2_Terriglobus_saanensis
C7P3E8_Halomicrobium_mukohataei
L9ZZB5_Natrialba_taiwanensis
M0AIF9_Natrialba_aegyptia
0,95
0,89
0,95
1
0,94
0,91
0,87
0,1
0,53
0,15
0,97
0,82
0,28
0,75
0,81
0,94
1
0.5

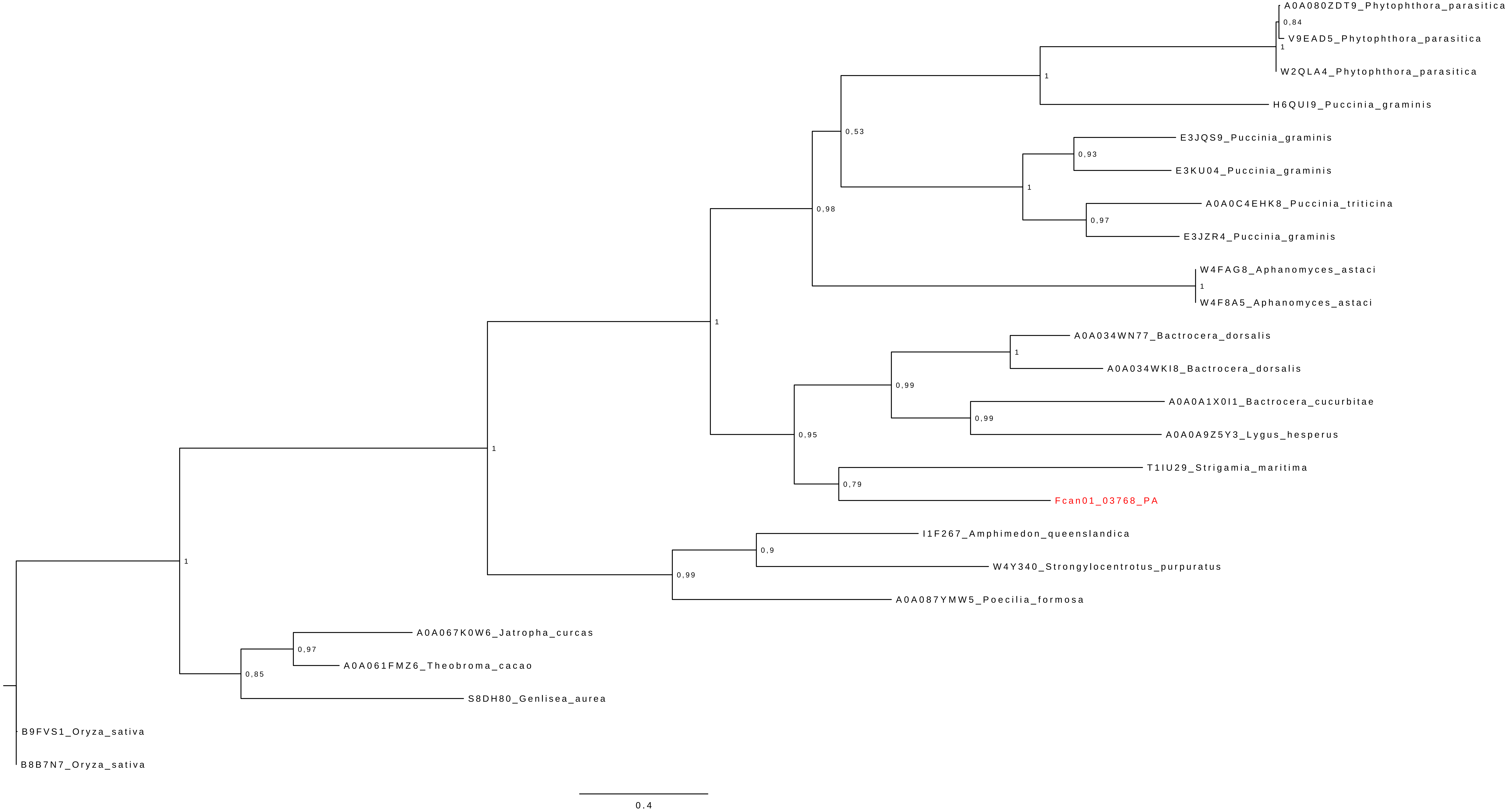
A0A080ZDT9_Phytophthora_parasitica
V9EAD5_Phytophthora_parasitica
W2QLA4_Phytophthora_parasitica
H6QUI9_Puccinia_graminis
E3JQS9_Puccinia_graminis
E3KU04_Puccinia_graminis
A0A0C4EHK8_Puccinia_triticina
E3JZR4_Puccinia_graminis
W4FAG8_Aphanomyces_astaci
W4F8A5_Aphanomyces_astaci
A0A034WN77_Bactrocera_dorsalis
A0A034WKI8_Bactrocera_dorsalis
A0A0A1X0I1_Bactrocera_cucurbitae
A0A0A9Z5Y3_Lygus_hesperus
T1IU29_Strigamia_maritima
Fcan01_03768_PA
I1F267_Amphimedon_queenslandica
W4Y340_Strongylocentrotus_purpuratus
A0A087YMW5_Poecilia_formosa
A0A067K0W6_Jatropha_curcas
A0A061FMZ6_Theobroma_cacao
S8DH80_Genlisea_aurea
B9FVS1_Oryza_sativa
B8B7N7_Oryza_sativa
0.4

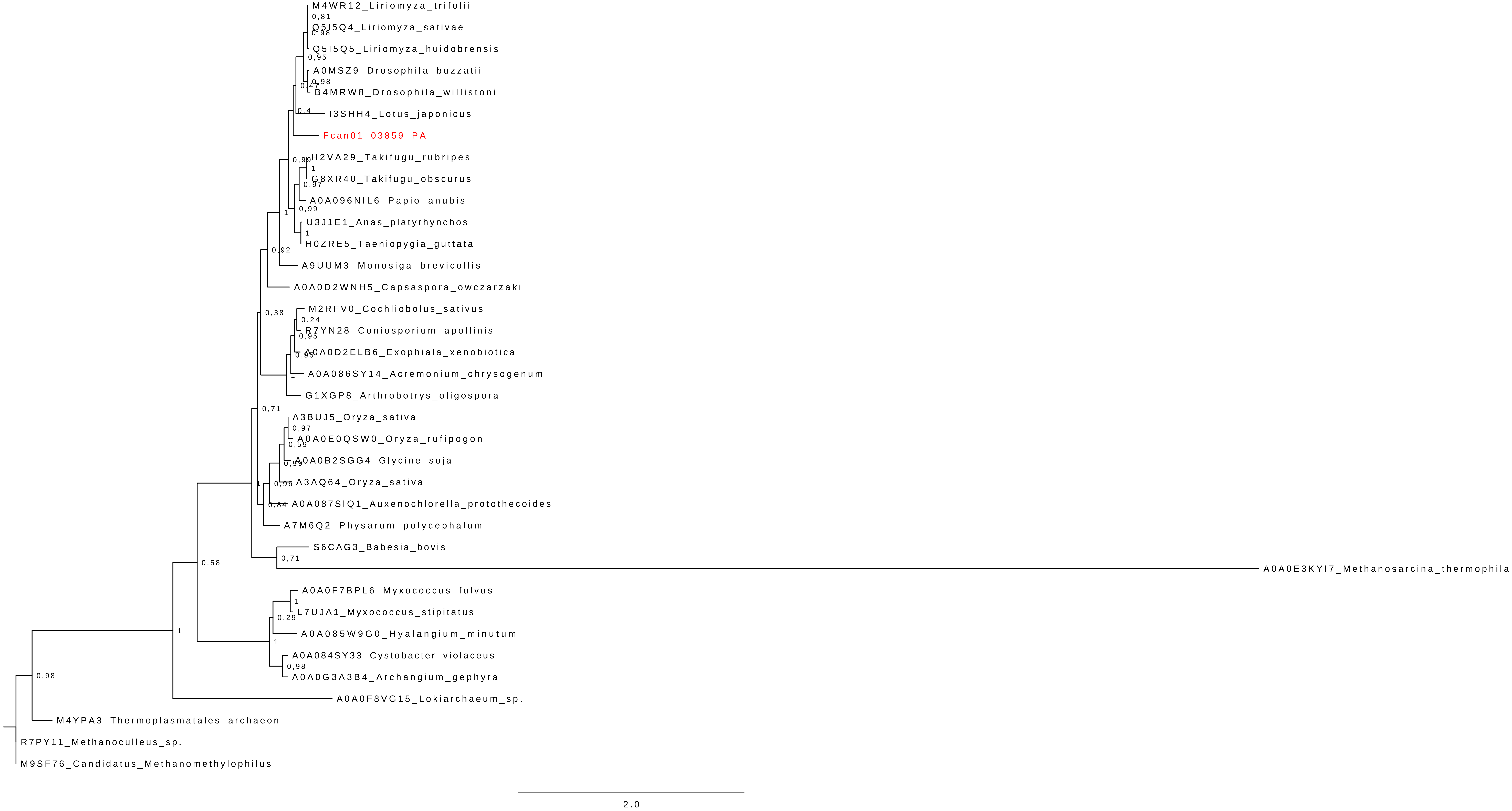
M4WR12_Liriomyza_trifolii
Q5I5Q4_Liriomyza_sativae
Q5I5Q5_Liriomyza_huidobrensis
A0MSZ9_Drosophila_buzzatii
B4MRW8_Drosophila_willistoni
I3SHH4_Lotus_japonicus
Fcan01_03859_PA
H2VA29_Takifugu_rubripes
G8XR40_Takifugu_obscurus
A0A096NIL6_Papio_anubis
U3J1E1_Anas_platyrhynchos
H0ZRE5_Taeniopygia_guttata
A9UUM3_Monosiga_brevicollis
A0A0D2WNH5_Capsaspora_owczarzaki
M2RFV0_Cochliobolus_sativus
R7YN28_Coniosporium_apollinis
A0A0D2ELB6_Exophiala_xenobiotica
A0A086SY14_Acremonium_chrysogenum
G1XGP8_Arthrobotrys_oligospora
A3BUJ5_Oryza_sativa
A0A0E0QSW0_Oryza_rufipogon
A0A0B2SGG4_Glycine_soja
A3AQ64_Oryza_sativa
A0A087SIQ1_Auxenochlorella_protothecoides
A7M6Q2_Physarum_polycephalum
S6CAG3_Babesia_bovis
A0A0E3KYI7_Methanosarcina_thermophila
A0A0F7BPL6_Myxococcus_fulvus
L7UJA1_Myxococcus_stipitatus
A0A085W9G0_Hyalangium_minutum
A0A084SY33_Cystobacter_violaceus
A0A0G3A3B4_Archangium_gephyra
A0A0F8VG15_Lokiarchaeum_sp.
M4YPA3_Thermoplasmatales_archaeon
R7PY11_Methanoculleus_sp.
M9SF76_Candidatus_Methanomethylophilus
2.0

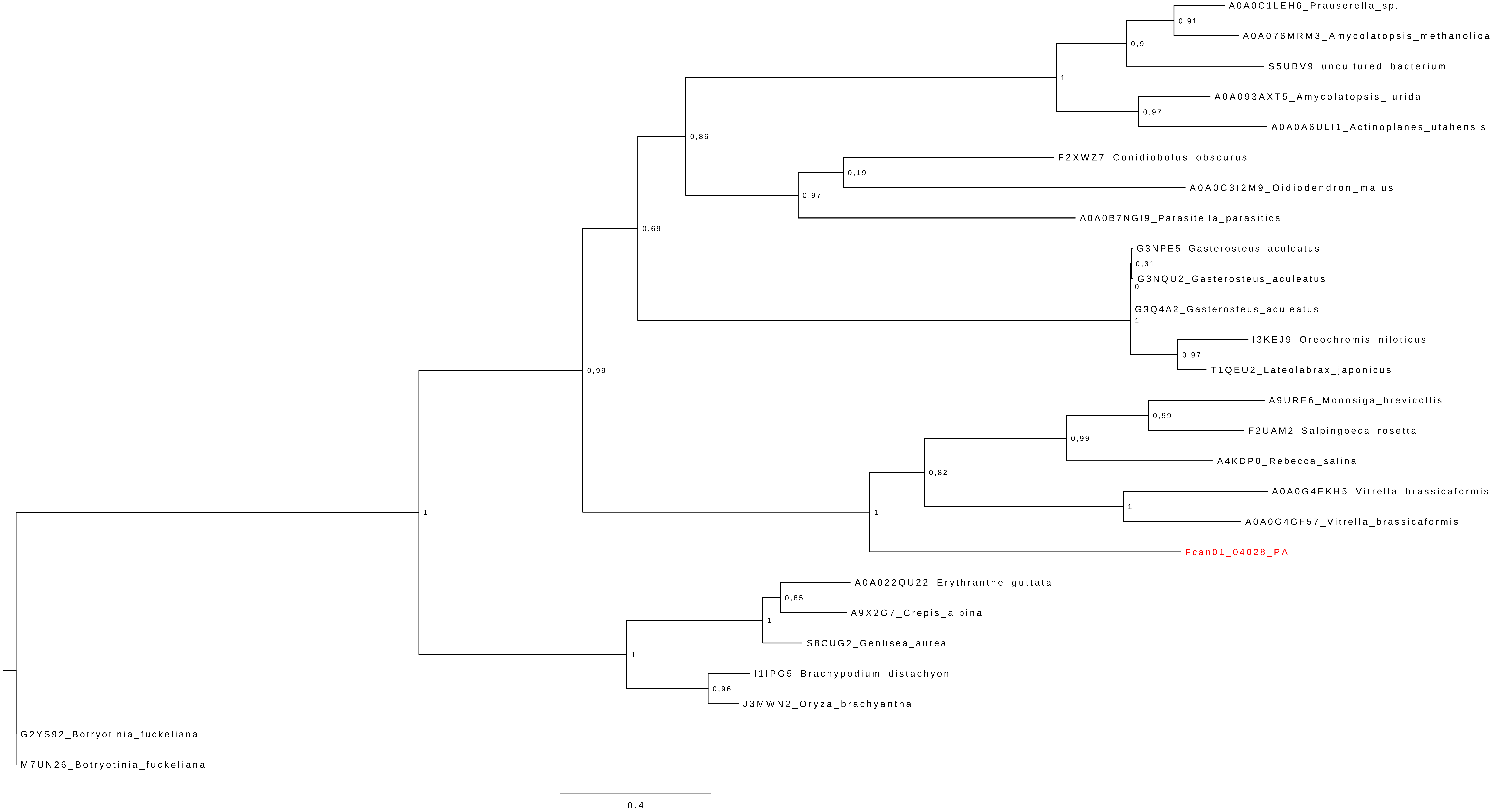
A0A0C1LEH6_Prauserella_sp.
A0A076MRM3_Amycolatopsis_methanolica
S5UBV9_uncultured_bacterium
A0A093AXT5_Amycolatopsis_lurida
A0A0A6ULI1_Actinoplanes_utahensis
F2XWZ7_Conidiobolus_obscurus
A0A0C3I2M9_Oidiodendron_maius
A0A0B7NGI9_Parasitella_parasitica
G3NPE5_Gasterosteus_aculeatus
G3NQU2_Gasterosteus_aculeatus
G3Q4A2_Gasterosteus_aculeatus
I3KEJ9_Oreochromis_niloticus
T1QEU2_Lateolabrax_japonicus
A9URE6_Monosiga_brevicollis
F2UAM2_Salpingoeca_rosetta
A4KDP0_Rebecca_salina
A0A0G4EKH5_Vitrella_brassicaformis
A0A0G4GF57_Vitrella_brassicaformis
Fcan01_04028_PA
A0A022QU22_Erythranthe_guttata
A9X2G7_Crepis_alpina
S8CUG2_Genlisea_aurea
I1IPG5_Brachypodium_distachyon
J3MWN2_Oryza_brachyantha
G2YS92_Botryotinia_fuckeliana
M7UN26_Botryotinia_fuckeliana
0,91
0,9
1
0,97
0,86
0,19
0,97
0,69
0,31
0
1
0,97
0,99
0,99
0,99
0,82
1
1
1
0,85
1
1
0,96
0.4

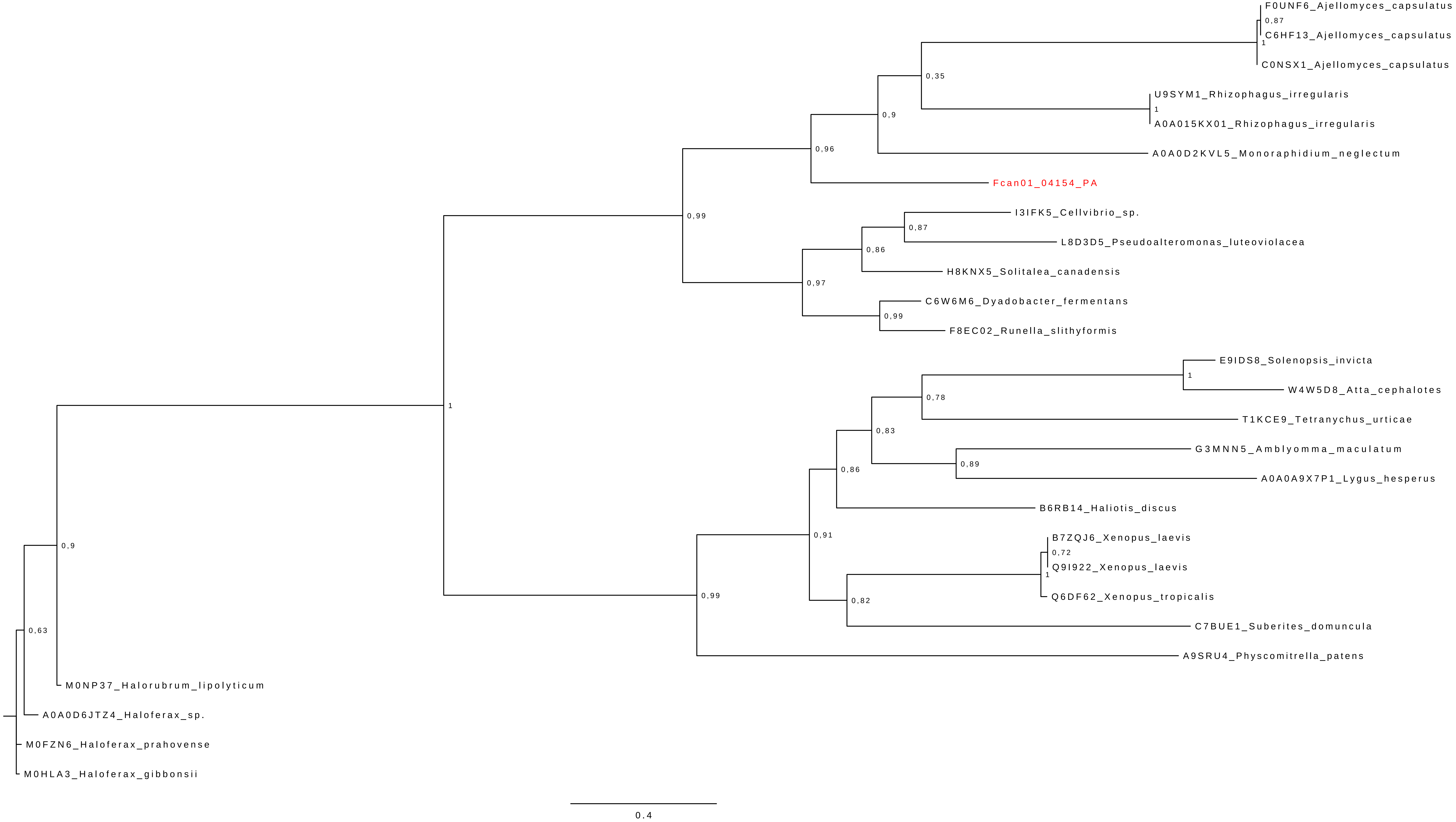
F0UNF6_Ajellomyces_capsulatus
0,87
C6HF13_Ajellomyces_capsulatus
1
C0NSX1_Ajellomyces_capsulatus
0,35
U9SYM1_Rhizophagus_irregularis
1
A0A015KX01_Rhizophagus_irregularis
0,9
A0A0D2KVL5_Monoraphidium_neglectum
0,96
Fcan01_04154_PA
0,99
I3IFK5_Cellvibrio_sp.
0,87
L8D3D5_Pseudoalteromonas_luteoviolacea
0,86
H8KNX5_Solitalea_canadensis
0,97
C6W6M6_Dyadobacter_fermentans
0,99
F8EC02_Runella_slithyformis
E9IDS8_Solenopsis_invicta
1
W4W5D8_Atta_cephalotes
0,78
T1KCE9_Tetranychus_urticae
0,83
G3MNN5_Amblyomma_maculatum
0,89
A0A0A9X7P1_Lygus_hesperus
0,86
B6RB14_Haliotis_discus
0,9
B7ZQJ6_Xenopus_laevis
0,72
Q9I922_Xenopus_laevis
1
0,91
Q6DF62_Xenopus_tropicalis
0,82
0,99
C7BUE1_Suberites_domuncula
0,63
A9SRU4_Physcomitrella_patens
M0NP37_Halorubrum_lipolyticum
A0A0D6JTZ4_Haloferax_sp.
M0FZN6_Haloferax_prahovense
M0HLA3_Haloferax_gibbonsii
0.4

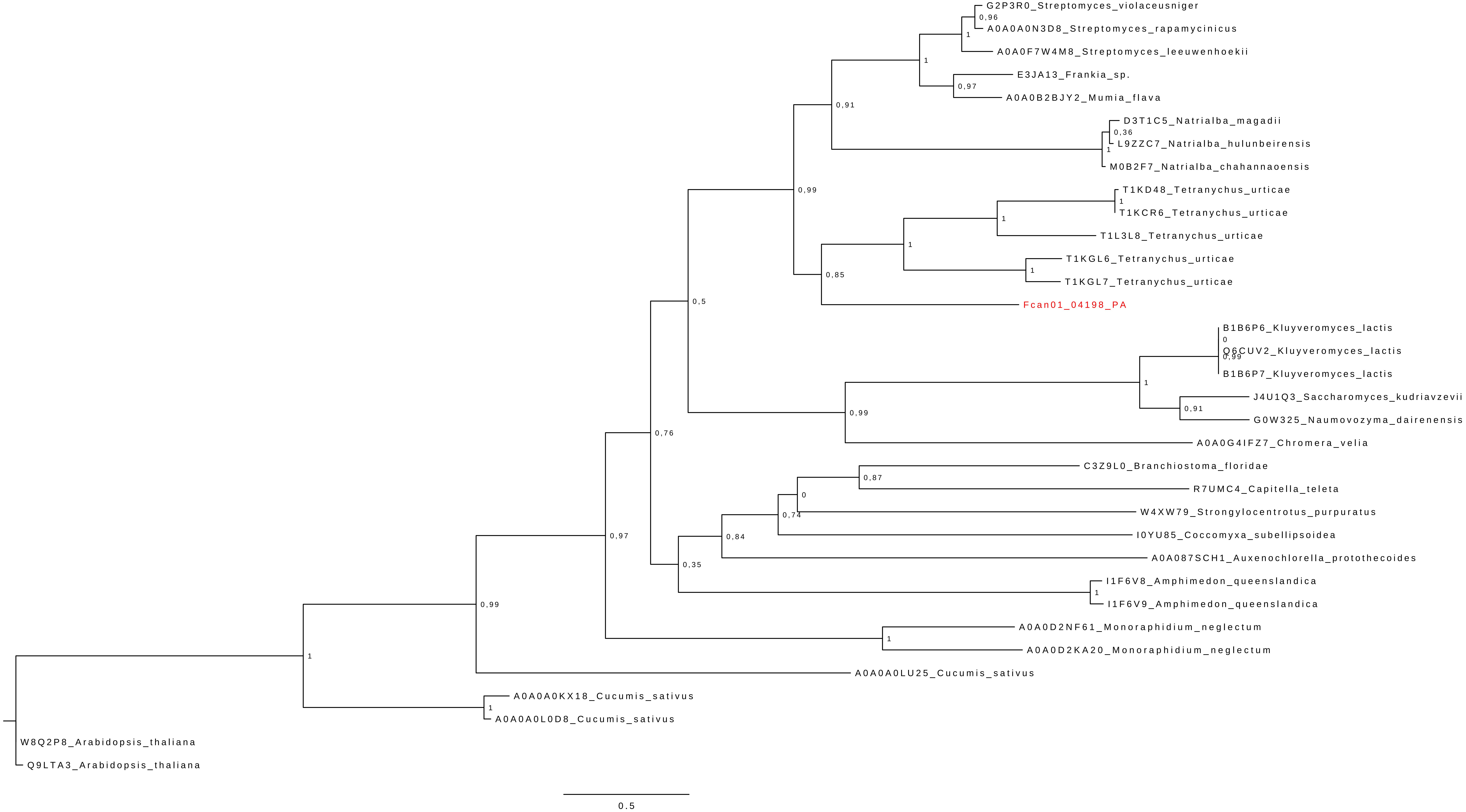
G2P3R0_Streptomyces_violaceusniger
0,96
A0A0A0N3D8_Streptomyces_rapamycinicus
1
A0A0F7W4M8_Streptomyces_leeuwenhoekii
1
E3JA13_Frankia_sp.
0,97
A0A0B2BJY2_Mumia_flava
0,91
D3T1C5_Natrialba_magadii
0,36
L9ZZC7_Natrialba_hulunbeirensis
1
M0B2F7_Natrialba_chahannaoensis
0,99
T1KD48_Tetranychus_urticae
1
T1KCR6_Tetranychus_urticae
1
T1L3L8_Tetranychus_urticae
1
T1KGL6_Tetranychus_urticae
1
T1KGL7_Tetranychus_urticae
0,85
Fcan01_04198_PA
0,5
B1B6P6_Kluyveromyces_lactis
0
Q6CUV2_Kluyveromyces_lactis
0,99
B1B6P7_Kluyveromyces_lactis
1
J4U1Q3_Saccharomyces_kudriavzevii
0,91
G0W325_Naumovozyma_dairenensis
0,99
A0A0G4IFZ7_Chromera_velia
C3Z9L0_Branchiostoma_floridae
0,87
R7UMC4_Capitella_teleta
0
W4XW79_Strongylocentrotus_purpuratus
0,74
I0YU85_Coccomyxa_subellipsoidea
0,84
A0A087SCH1_Auxenochlorella_protothecoides
0,35
I1F6V8_Amphimedon_queenslandica
1
I1F6V9_Amphimedon_queenslandica
0,76
0,97
A0A0D2NF61_Monoraphidium_neglectum
1
A0A0D2KA20_Monoraphidium_neglectum
0,99
A0A0A0LU25_Cucumis_sativus
1
A0A0A0KX18_Cucumis_sativus
1
A0A0A0L0D8_Cucumis_sativus
W8Q2P8_Arabidopsis_thaliana
Q9LTA3_Arabidopsis_thaliana
0.5

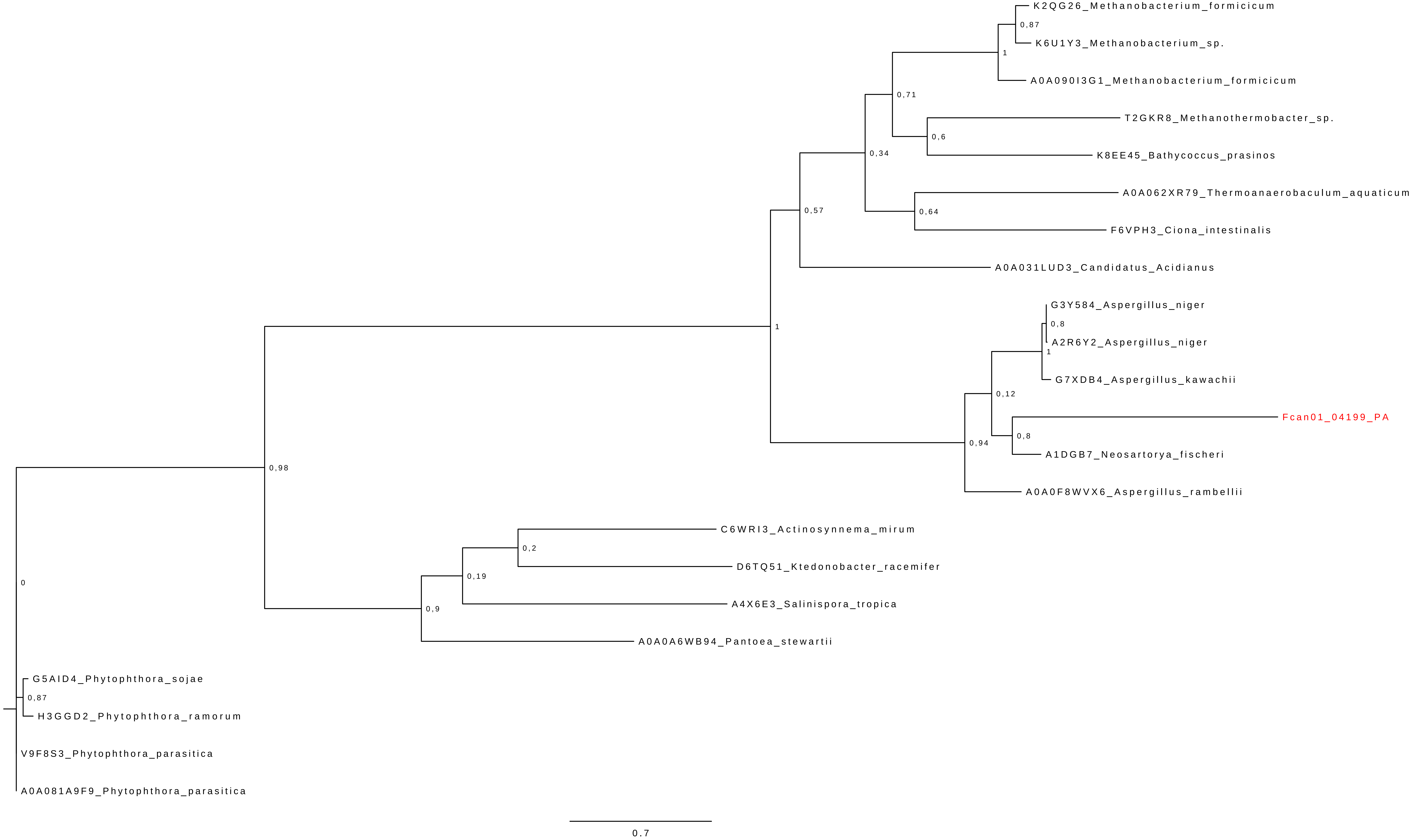
K2QG26_Methanobacterium_formicicum
0,87
K6U1Y3_Methanobacterium_sp.
1
A0A090I3G1_Methanobacterium_formicicum
0,71
T2GKR8_Methanothermobacter_sp.
0,6
K8EE45_Bathycoccus_prasinos
0,34
A0A062XR79_Thermoanaerobaculum_aquaticum
0,64
F6VPH3_Ciona_intestinalis
0,57
A0A031LUD3_Candidatus_Acidianus
1
G3Y584_Aspergillus_niger
0,8
A2R6Y2_Aspergillus_niger
1
G7XDB4_Aspergillus_kawachii
0,12
Fcan01_04199_PA
0,8
A1DGB7_Neosartorya_fischeri
0,94
A0A0F8WVX6_Aspergillus_rambellii
0,98
C6WRI3_Actinosynnema_mirum
0,2
D6TQ51_Ktedonobacter_racemifer
0,19
A4X6E3_Salinispora_tropica
0,9
A0A0A6WB94_Pantoea_stewartii
0
G5AID4_Phytophthora_sojae
0,87
H3GGD2_Phytophthora_ramorum
V9F8S3_Phytophthora_parasitica
A0A081A9F9_Phytophthora_parasitica
0.7

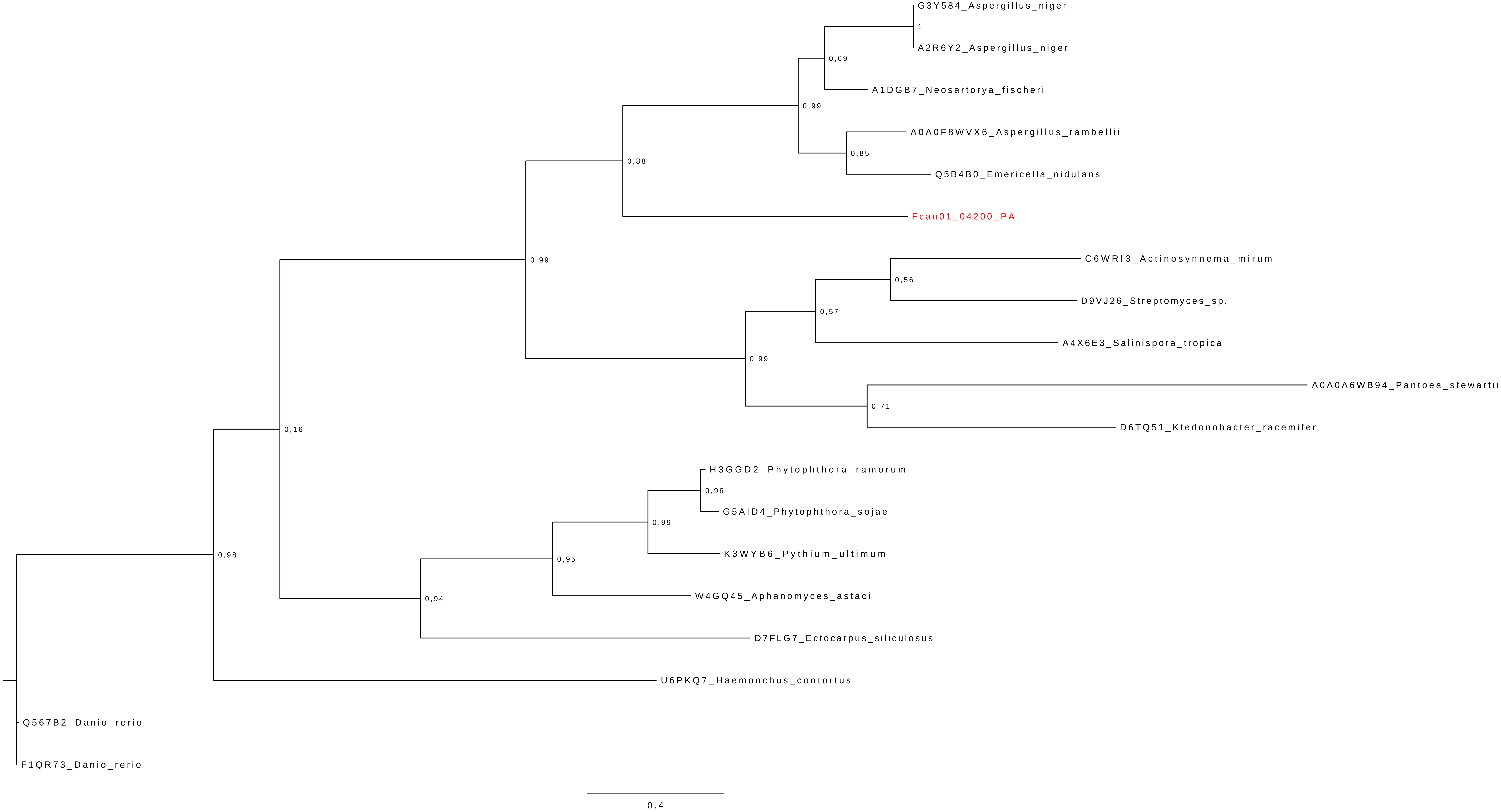

G3Y584_Aspergillus_niger
1
A2R6Y2_Aspergillus_niger
0,69
A1DGB7_Neosartorya_fischeri
0,99
A0A0F8WVX6_Aspergillus_rambellii
0,85
Q5B4B0_Emericella_nidulans
0,88
Fcan01_04200_PA
0,99
C6WRI3_Actinosynnema_mirum
0,56
D9VJ26_Streptomyces_sp.
0,57
A4X6E3_Salinispora_tropica
0,99
A0A0A6WB94_Pantoea_stewartii
0,71
D6TQ51_Ktedonobacter_racemifer
0,16
H3GGD2_Phytophthora_ramorum
0,96
G5AID4_Phytophthora_sojae
0,99
K3WYB6_Pythium_ultimum
0,95
W4GQ45_Aphanomyces_astaci
0,94
D7FLG7_Ectocarpus_siliculosus
0,98
U6PKQ7_Haemonchus_contortus
Q567B2_Danio_rerio
F1QR73_Danio_rerio
0.4

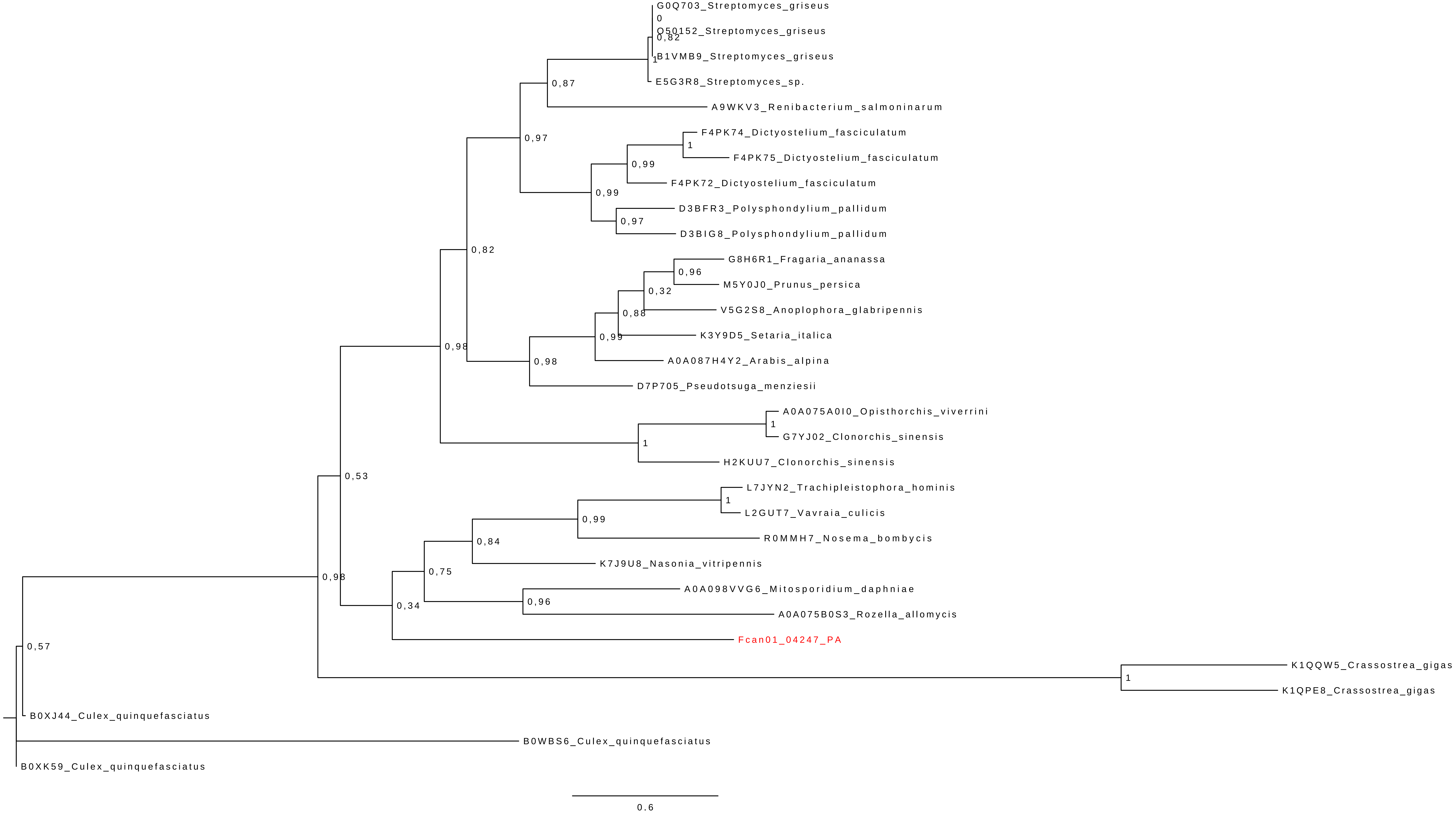
G0Q703_Streptomyces_griseus
0
O50152_Streptomyces_griseus
0,82
B1VMB9_Streptomyces_griseus
1
E5G3R8_Streptomyces_sp.
0,87
A9WKV3_Renibacterium_salmoninarum
F4PK74_Dictyostelium_fasciculatum
1
F4PK75_Dictyostelium_fasciculatum
0,97
0,99
F4PK72_Dictyostelium_fasciculatum
0,99
D3BFR3_Polysphondylium_pallidum
0,97
D3BIG8_Polysphondylium_pallidum
0,82
G8H6R1_Fragaria_ananassa
0,96
M5Y0J0_Prunus_persica
0,32
V5G2S8_Anoplophora_glabripennis
0,88
K3Y9D5_Setaria_italica
0,99
0,98
A0A087H4Y2_Arabis_alpina
0,98
D7P705_Pseudotsuga_menziesii
A0A075A0I0_Opisthorchis_viverrini
1
G7YJ02_Clonorchis_sinensis
1
H2KUU7_Clonorchis_sinensis
0,53
L7JYN2_Trachipleistophora_hominis
1
L2GUT7_Vavraia_culicis
0,99
R0MMH7_Nosema_bombycis
0,84
K7J9U8_Nasonia_vitripennis
0,98
0,75
A0A098VVG6_Mitosporidium_daphniae
0,34
0,96
A0A075B0S3_Rozella_allomycis
Fcan01_04247_PA
0,57
K1QQW5_Crassostrea_gigas
1
K1QPE8_Crassostrea_gigas
B0XJ44_Culex_quinquefasciatus
B0WBS6_Culex_quinquefasciatus
B0XK59_Culex_quinquefasciatus
0.6

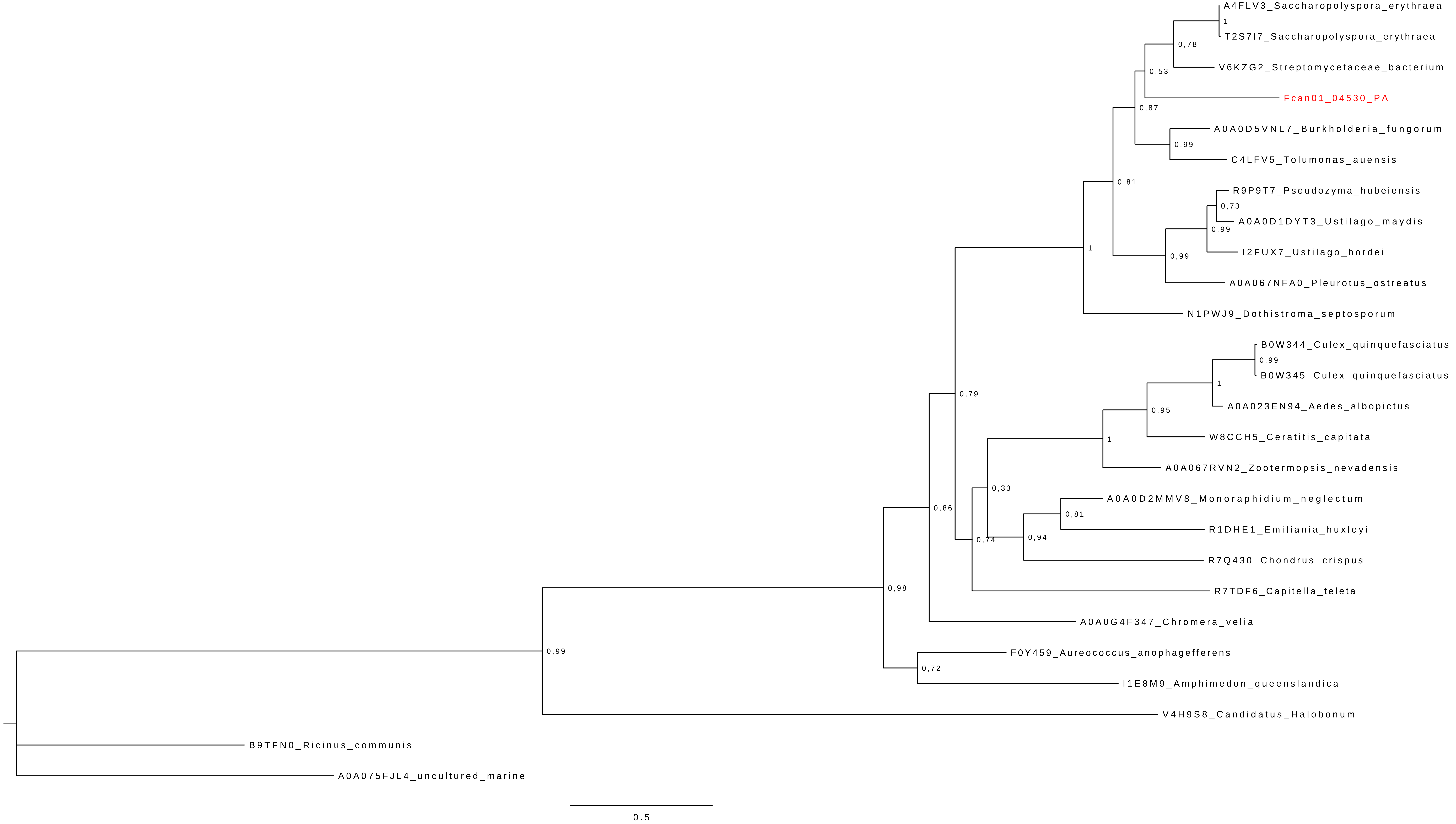

A4FLV3_Saccharopolyspora_erythraea
1
T2S7I7_Saccharopolyspora_erythraea
0,78
V6KZG2_Streptomycetaceae_bacterium
0,53
Fcan01_04530_PA
0,87
A0A0D5VNL7_Burkholderia_fungorum
0,99
C4LFV5_Tolumonas_auensis
0,81
R9P9T7_Pseudozyma_hubeiensis
0,73
A0A0D1DYT3_Ustilago_maydis
0,99
I2FUX7_Ustilago_hordei
1
0,99
A0A067NFA0_Pleurotus_ostreatus
N1PWJ9_Dothistroma_septosporum
B0W344_Culex_quinquefasciatus
0,99
B0W345_Culex_quinquefasciatus
1
0,79
A0A023EN94_Aedes_albopictus
0,95
W8CCH5_Ceratitis_capitata
1
A0A067RVN2_Zootermopsis_nevadensis
0,33
A0A0D2MMV8_Monoraphidium_neglectum
0,86
0,81
R1DHE1_Emiliania_huxleyi
0,74
0,94
R7Q430_Chondrus_crispus
0,98
R7TDF6_Capitella_teleta
A0A0G4F347_Chromera_velia
F0Y459_Aureococcus_anophagefferens
0,99
0,72
I1E8M9_Amphimedon_queenslandica
V4H9S8_Candidatus_Halobonum
B9TFN0_Ricinus_communis
A0A075FJL4_uncultured_marine
0.5

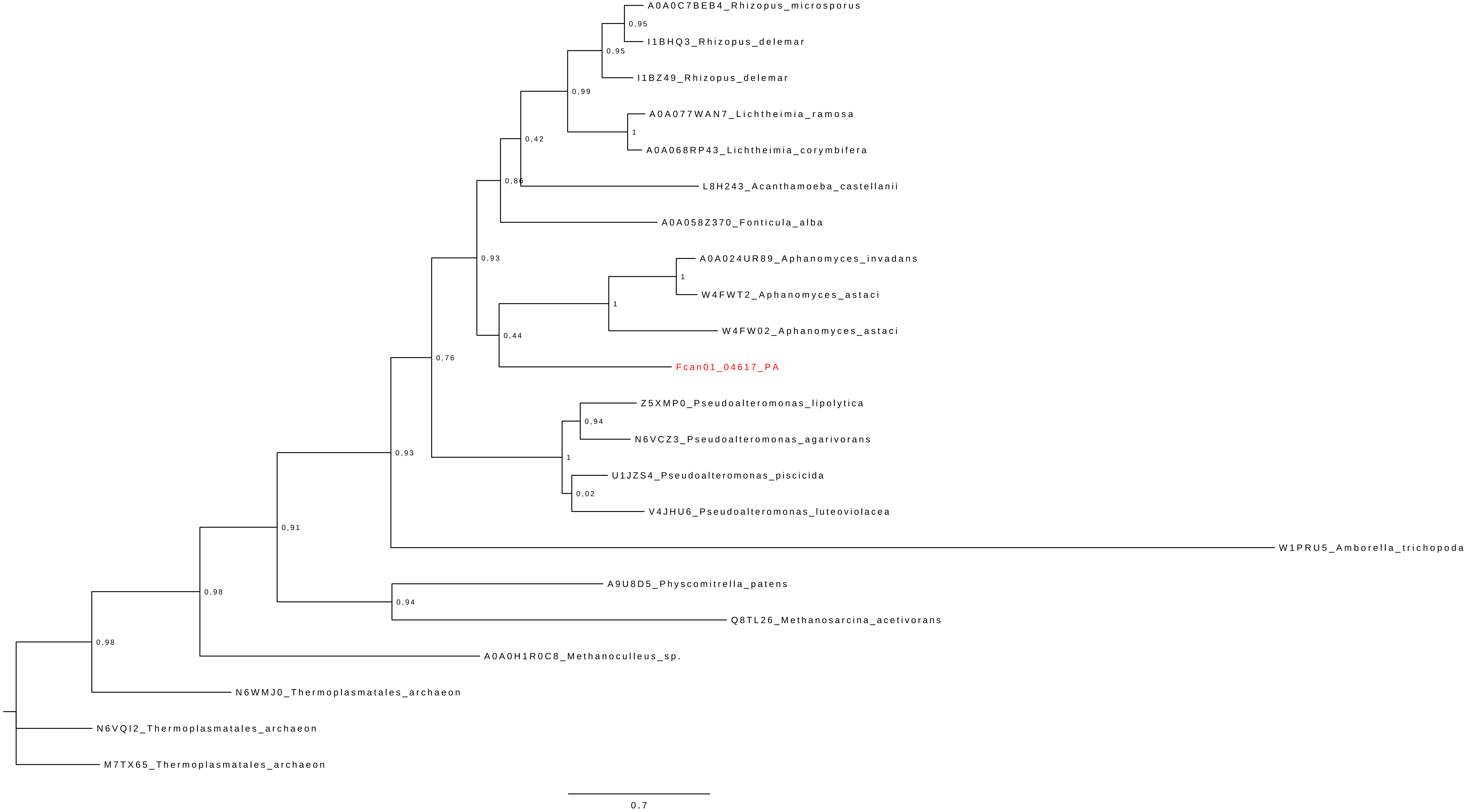

A0A0C7BEB4_Rhizopus_microsporus
0,95
I1BHQ3_Rhizopus_delemar
0,95
I1BZ49_Rhizopus_delemar
0,99
A0A077WAN7_Lichtheimia_ramosa
1
A0A068RP43_Lichtheimia_corymbifera
0,42
L8H243_Acanthamoeba_castellanii
0,86
A0A058Z370_Fonticula_alba
0,93
A0A024UR89_Aphanomyces_invadans
1
W4FWT2_Aphanomyces_astaci
1
W4FW02_Aphanomyces_astaci
0,44
Fcan01_04617_PA
0,76
Z5XMP0_Pseudoalteromonas_lipolytica
0,94
N6VCZ3_Pseudoalteromonas_agarivorans
1
U1JZS4_Pseudoalteromonas_piscicida
0,02
V4JHU6_Pseudoalteromonas_luteoviolacea
0,93
W1PRU5_Amborella_trichopoda
0,91
A9U8D5_Physcomitrella_patens
0,94
Q8TL26_Methanosarcina_acetivorans
0,98
A0A0H1R0C8_Methanoculleus_sp.
0,98
N6WMJ0_Thermoplasmatales_archaeon
N6VQI2_Thermoplasmatales_archaeon
M7TX65_Thermoplasmatales_archaeon
0.7

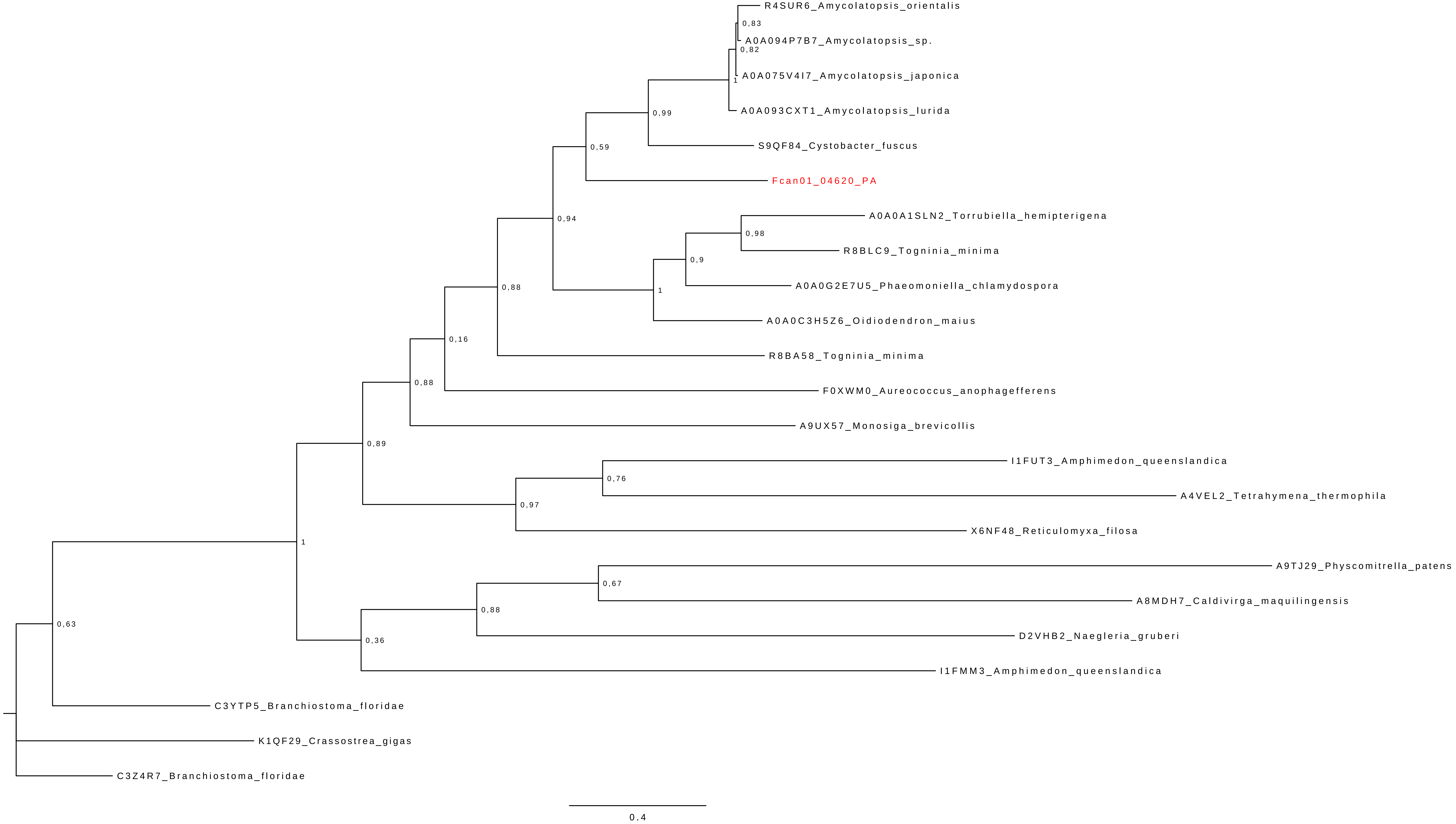
R4SUR6_Amycolatopsis_orientalis
0,83
A0A094P7B7_Amycolatopsis_sp.
0,82
A0A075V4I7_Amycolatopsis_japonica
1
A0A093CXT1_Amycolatopsis_lurida
0,99
S9QF84_Cystobacter_fuscus
0,59
Fcan01_04620_PA
A0A0A1SLN2_Torrubiella_hemipterigena
0,98
R8BLC9_Togninia_minima
0,9
0,94
A0A0G2E7U5_Phaeomoniella_chlamydospora
1
A0A0C3H5Z6_Oidiodendron_maius
0,88
R8BA58_Togninia_minima
0,16
F0XWM0_Aureococcus_anophagefferens
0,88
A9UX57_Monosiga_brevicollis
0,89
I1FUT3_Amphimedon_queenslandica
0,76
A4VEL2_Tetrahymena_thermophila
0,97
X6NF48_Reticulomyxa_filosa
1
A9TJ29_Physcomitrella_patens
0,67
A8MDH7_Caldivirga_maquilingensis
0,88
D2VHB2_Naegleria_gruberi
0,36
0,63
I1FMM3_Amphimedon_queenslandica
C3YTP5_Branchiostoma_floridae
K1QF29_Crassostrea_gigas
C3Z4R7_Branchiostoma_floridae
0.4

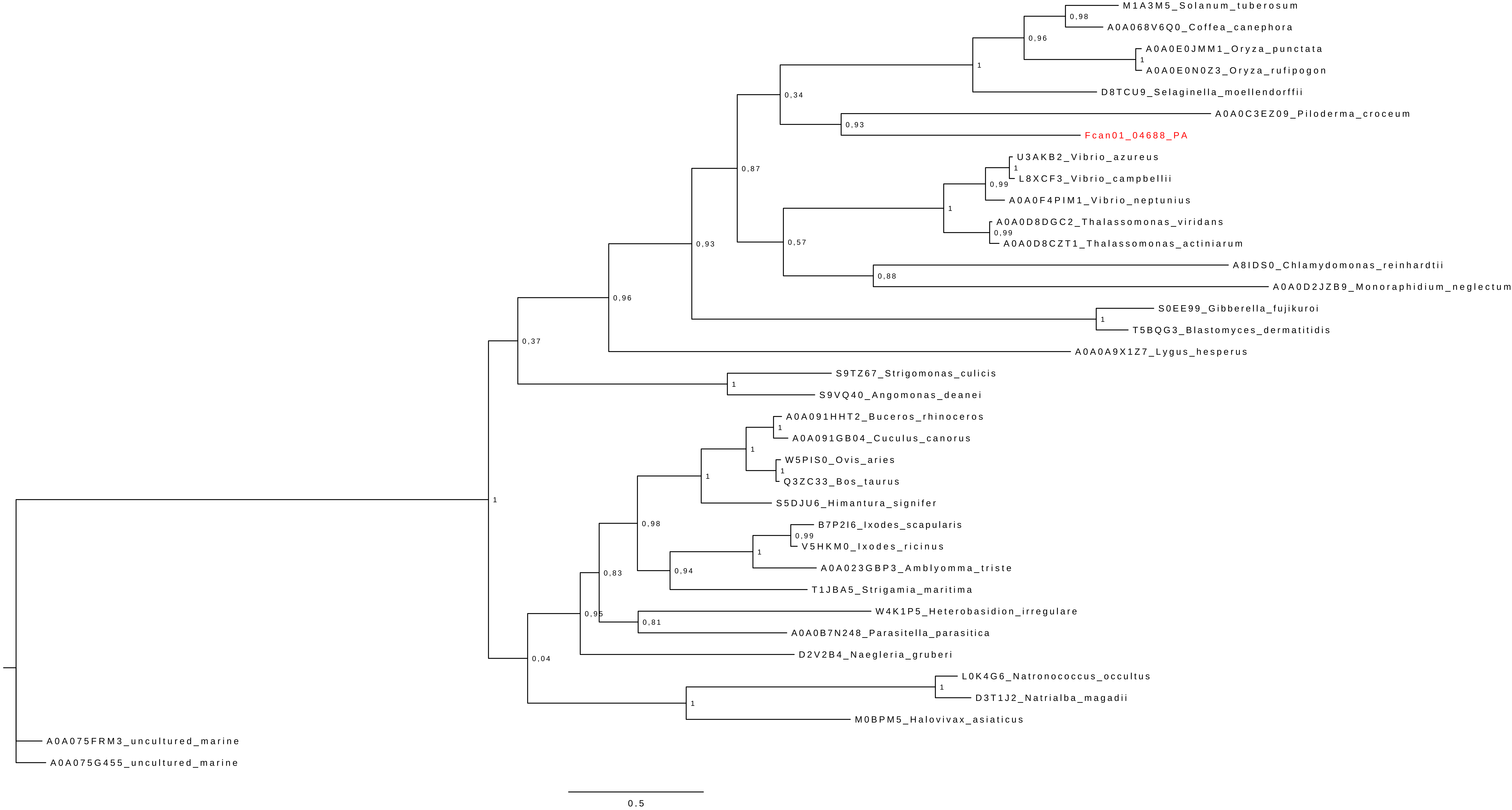
M1A3M5_Solanum_tuberosum
A0A068V6Q0_Coffea_canephora
A0A0E0JMM1_Oryza_punctata
A0A0E0N0Z3_Oryza_rufipogon
D8TCU9_Selaginella_moellendorffii
A0A0C3EZ09_Piloderma_croceum
Fcan01_04688_PA
U3AKB2_Vibrio_azureus
L8XCF3_Vibrio_campbellii
A0A0F4PIM1_Vibrio_neptunius
A0A0D8DGC2_Thalassomonas_viridans
A0A0D8CZT1_Thalassomonas_actiniarum
A8IDS0_Chlamydomonas_reinhardtii
A0A0D2JZB9_Monoraphidium_neglectum
S0EE99_Gibberella_fujikuroi
T5BQG3_Blastomyces_dermatitidis
A0A0A9X1Z7_Lygus_hesperus
S9TZ67_Strigomonas_culicis
S9VQ40_Angomonas_deanei
A0A091HHT2_Buceros_rhinoceros
A0A091GB04_Cuculus_canorus
W5PIS0_Ovis_aries
Q3ZC33_Bos_taurus
S5DJU6_Himantura_signifer
B7P2I6_Ixodes_scapularis
V5HKM0_Ixodes_ricinus
A0A023GBP3_Amblyomma_triste
T1JBA5_Strigamia_maritima
W4K1P5_Heterobasidion_irregulare
A0A0B7N248_Parasitella_parasitica
D2V2B4_Naegleria_gruberi
L0K4G6_Natronococcus_occultus
D3T1J2_Natrialba_magadii
M0BPM5_Halovivax_asiaticus
A0A075FRM3_uncultured_marine
A0A075G455_uncultured_marine
0,98
0,96
1
1
0,34
0,93
0,87
1
0,99
1
0,99
0,57
0,88
0,93
1
0,96
0,37
1
1
1
1
1
1
0,99
1
0,98
0,94
0,83
0,81
0,95
0,04
1
1
0.5

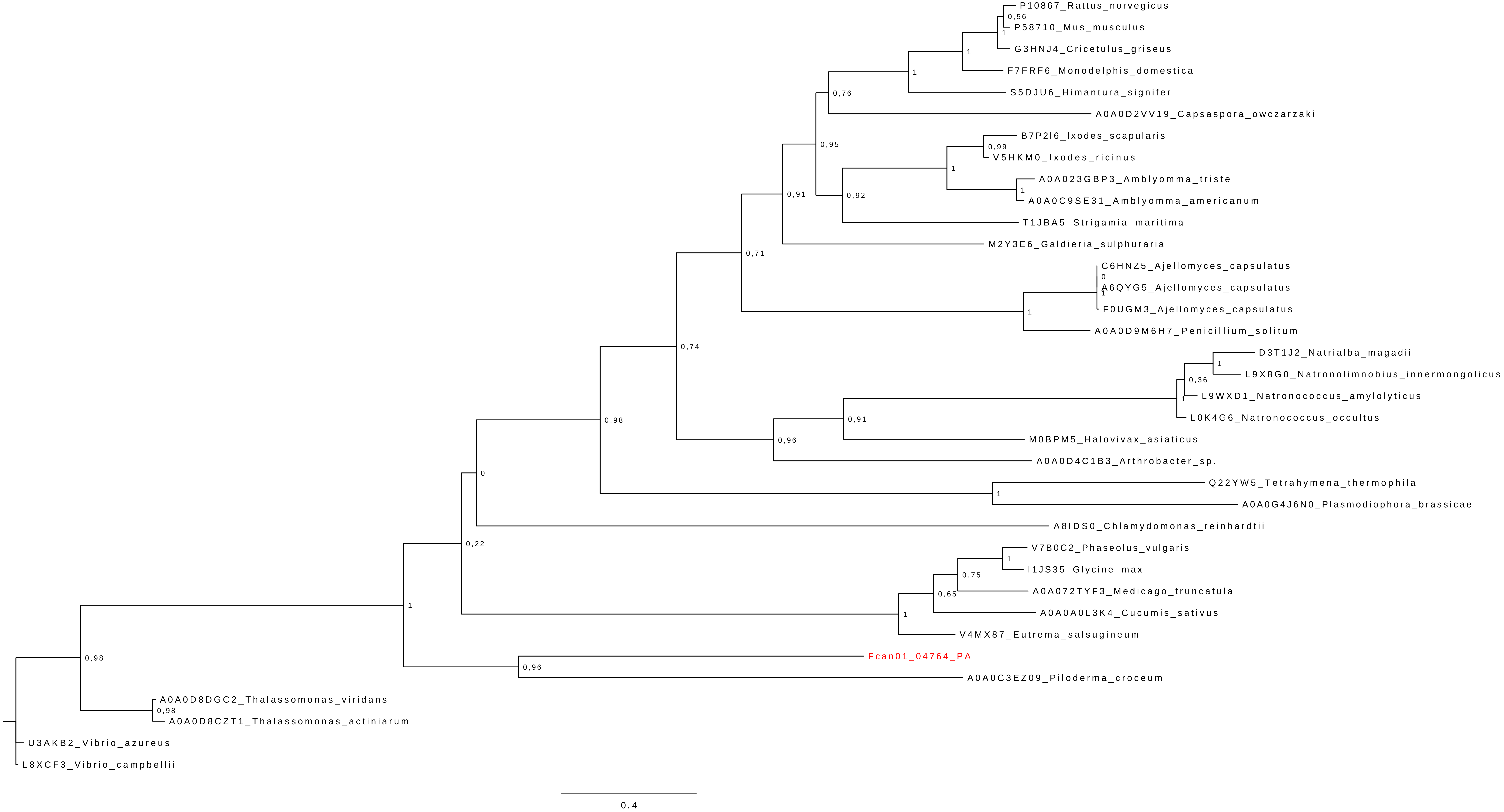
P10867_Rattus_norvegicus
P58710_Mus_musculus
G3HNJ4_Cricetulus_griseus
F7FRF6_Monodelphis_domestica
S5DJU6_Himantura_signifer
A0A0D2VV19_Capsaspora_owczarzaki
B7P2I6_Ixodes_scapularis
V5HKM0_Ixodes_ricinus
A0A023GBP3_Amblyomma_triste
A0A0C9SE31_Amblyomma_americanum
T1JBA5_Strigamia_maritima
M2Y3E6_Galdieria_sulphuraria
C6HNZ5_Ajellomyces_capsulatus
A6QYG5_Ajellomyces_capsulatus
F0UGM3_Ajellomyces_capsulatus
A0A0D9M6H7_Penicillium_solitum
D3T1J2_Natrialba_magadii
L9X8G0_Natronolimnobius_innermongolicus
L9WXD1_Natronococcus_amylolyticus
L0K4G6_Natronococcus_occultus
M0BPM5_Halovivax_asiaticus
A0A0D4C1B3_Arthrobacter_sp.
Q22YW5_Tetrahymena_thermophila
A0A0G4J6N0_Plasmodiophora_brassicae
A8IDS0_Chlamydomonas_reinhardtii
V7B0C2_Phaseolus_vulgaris
I1JS35_Glycine_max
A0A072TYF3_Medicago_truncatula
A0A0A0L3K4_Cucumis_sativus
V4MX87_Eutrema_salsugineum
Fcan01_04764_PA
A0A0C3EZ09_Piloderma_croceum
A0A0D8DGC2_Thalassomonas_viridans
A0A0D8CZT1_Thalassomonas_actiniarum
U3AKB2_Vibrio_azureus
L8XCF3_Vibrio_campbellii
0.4

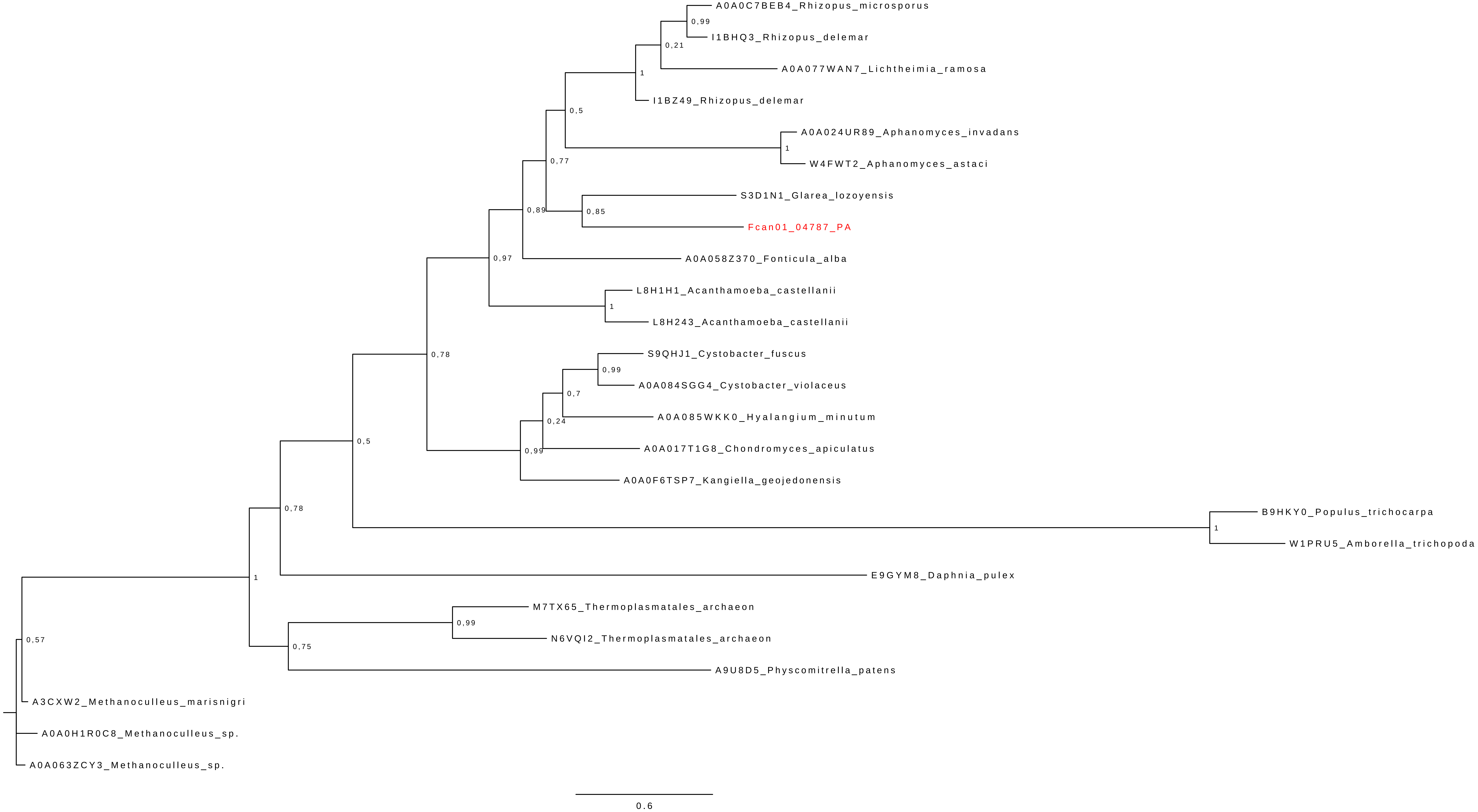

A0A0C7BEB4_Rhizopus_microsporus
0,99
I1BHQ3_Rhizopus_delemar
0,21
A0A077WAN7_Lichtheimia_ramosa
1
I1BZ49_Rhizopus_delemar
0,5
A0A024UR89_Aphanomyces_invadans
1
W4FWT2_Aphanomyces_astaci
0,77
S3D1N1_Glarea_lozoyensis
0,89
0,85
Fcan01_04787_PA
A0A058Z370_Fonticula_alba
0,97
L8H1H1_Acanthamoeba_castellanii
1
L8H243_Acanthamoeba_castellanii
S9QHJ1_Cystobacter_fuscus
0,99
A0A084SGG4_Cystobacter_violaceus
0,78
0,7
A0A085WKK0_Hyalangium_minutum
0,24
A0A017T1G8_Chondromyces_apiculatus
0,99
A0A0F6TSP7_Kangiella_geojedonensis
0,5
B9HKY0_Populus_trichocarpa
0,78
1
W1PRU5_Amborella_trichopoda
E9GYM8_Daphnia_pulex
1
M7TX65_Thermoplasmatales_archaeon
0,99
N6VQI2_Thermoplasmatales_archaeon
0,57
0,75
A9U8D5_Physcomitrella_patens
A3CXW2_Methanoculleus_marisnigri
A0A0H1R0C8_Methanoculleus_sp.
A0A063ZCY3_Methanoculleus_sp.
0.6

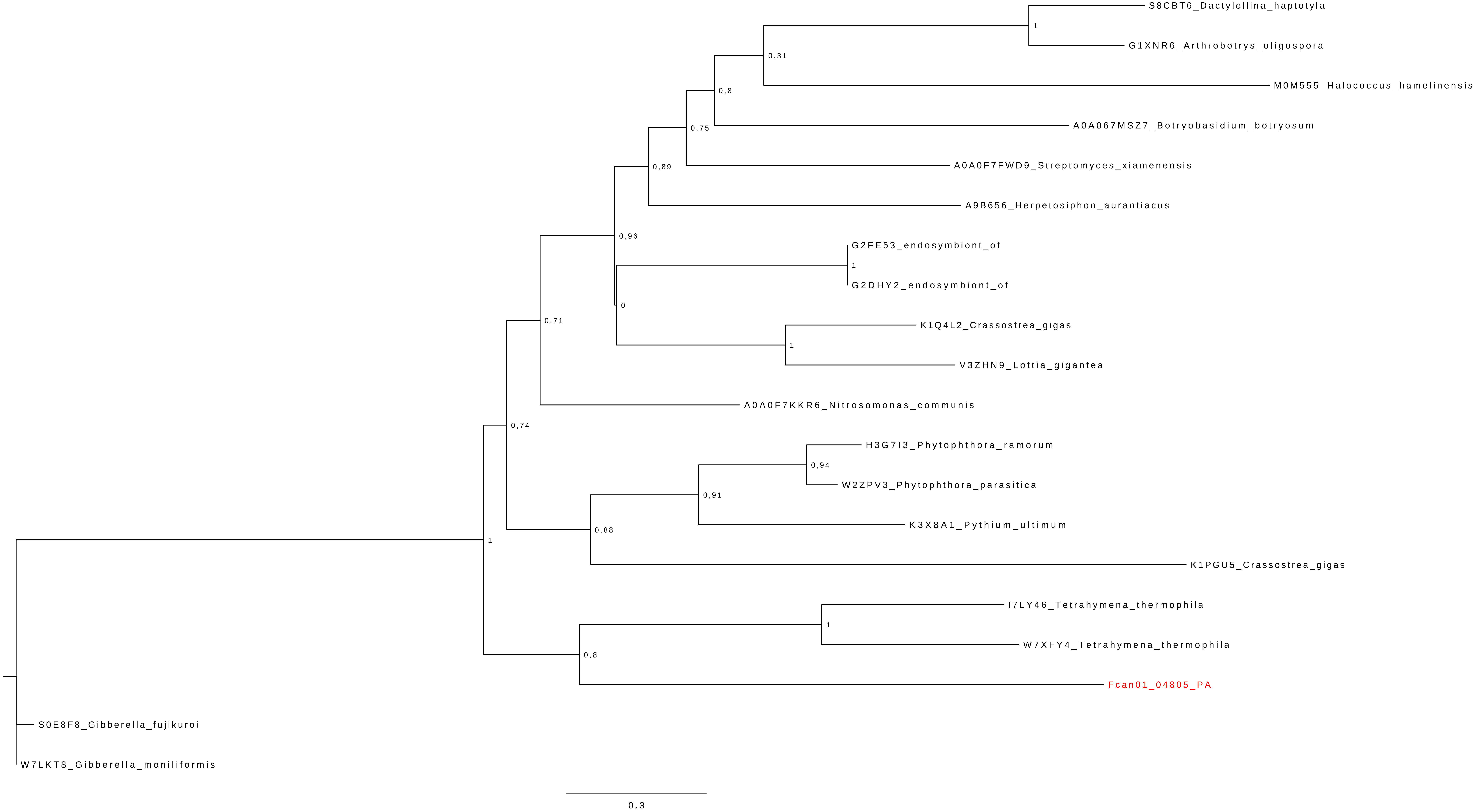
S8CBT6_Dactylellina_haptotyla
1
G1XNR6_Arthrobotrys_oligospora
0,31
M0M555_Halococcus_hamelinensis
0,8
A0A067MSZ7_Botryobasidium_botryosum
0,75
A0A0F7FWD9_Streptomyces_xiamenensis
0,89
A9B656_Herpetosiphon_aurantiacus
0,96
G2FE53_endosymbiont_of
1
G2DHY2_endosymbiont_of
0
K1Q4L2_Crassostrea_gigas
1
V3ZHN9_Lottia_gigantea
0,71
A0A0F7KKR6_Nitrosomonas_communis
0,74
H3G7I3_Phytophthora_ramorum
0,94
W2ZPV3_Phytophthora_parasitica
0,91
K3X8A1_Pythium_ultimum
0,88
1
K1PGU5_Crassostrea_gigas
I7LY46_Tetrahymena_thermophila
1
W7XFY4_Tetrahymena_thermophila
0,8
Fcan01_04805_PA
S0E8F8_Gibberella_fujikuroi
W7LKT8_Gibberella_moniliformis
0.3

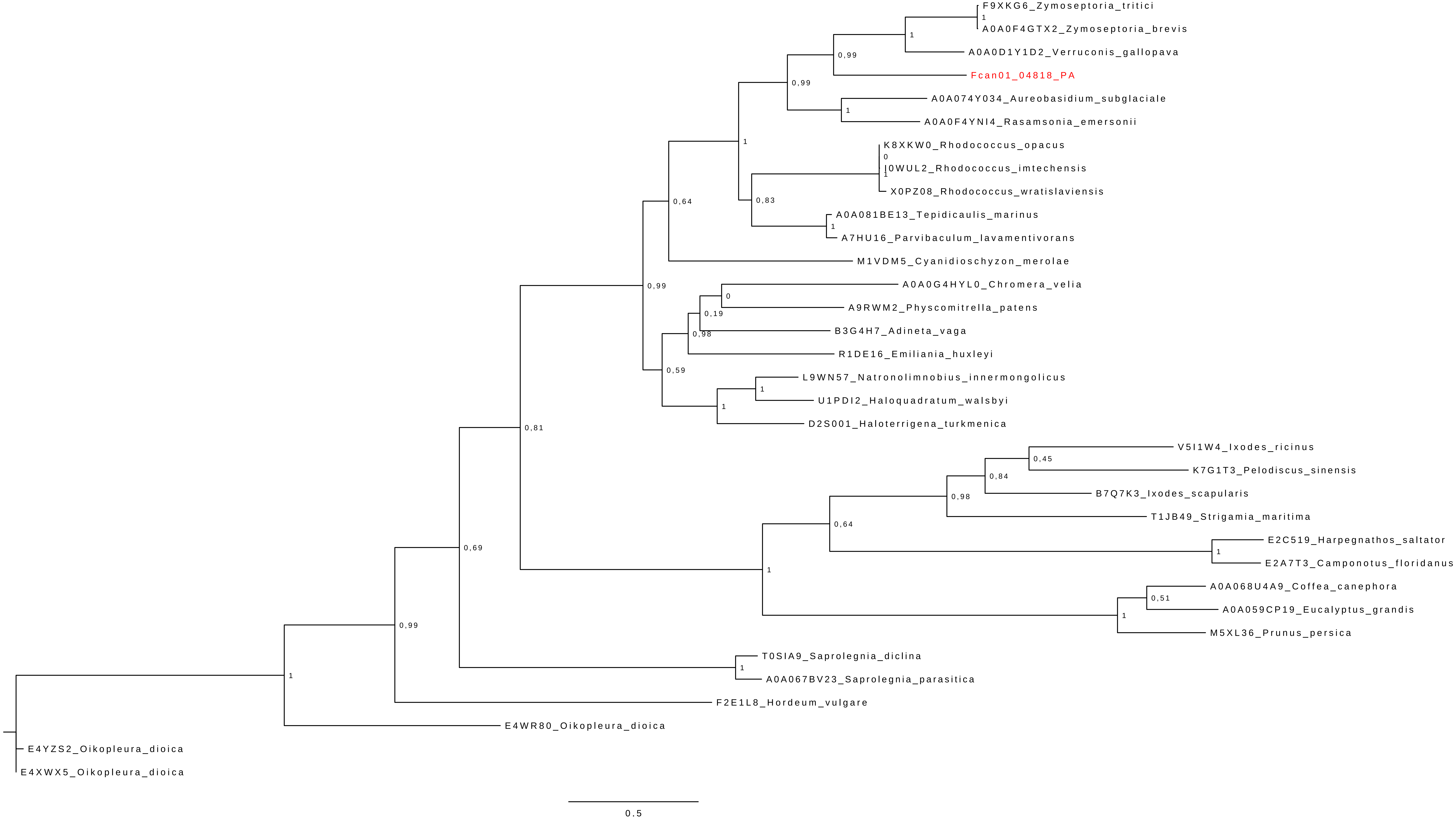
F9XKG6_Zymoseptoria_tritici
A0A0F4GTX2_Zymoseptoria_brevis
A0A0D1Y1D2_Verruconis_gallopava
Fcan01_04818_PA
A0A074Y034_Aureobasidium_subglaciale
A0A0F4YNI4_Rasamsonia_emersonii
K8XKW0_Rhodococcus_opacus
I0WUL2_Rhodococcus_imtechensis
X0PZ08_Rhodococcus_wratislaviensis
A0A081BE13_Tepidicaulis_marinus
A7HU16_Parvibaculum_lavamentivorans
M1VDM5_Cyanidioschyzon_merolae
A0A0G4HYL0_Chromera_velia
A9RWM2_Physcomitrella_patens
B3G4H7_Adineta_vaga
R1DE16_Emiliania_huxleyi
L9WN57_Natronolimnobius_innermongolicus
U1PDI2_Haloquadratum_walsbyi
D2S001_Haloterrigena_turkmenica
V5I1W4_Ixodes_ricinus
K7G1T3_Pelodiscus_sinensis
B7Q7K3_Ixodes_scapularis
T1JB49_Strigamia_maritima
E2C519_Harpegnathos_saltator
E2A7T3_Camponotus_floridanus
A0A068U4A9_Coffea_canephora
A0A059CP19_Eucalyptus_grandis
M5XL36_Prunus_persica
T0SIA9_Saprolegnia_diclina
A0A067BV23_Saprolegnia_parasitica
F2E1L8_Hordeum_vulgare
E4WR80_Oikopleura_dioica
E4YZS2_Oikopleura_dioica
E4XWX5_Oikopleura_dioica
1
1
0,99
0,99
1
1
0
1
0,83
1
0,64
0
0,19
0,98
0,59
1
1
0,99
0,81
0,45
0,84
0,98
0,64
1
1
0,51
1
0,69
1
0,99
1
0.5

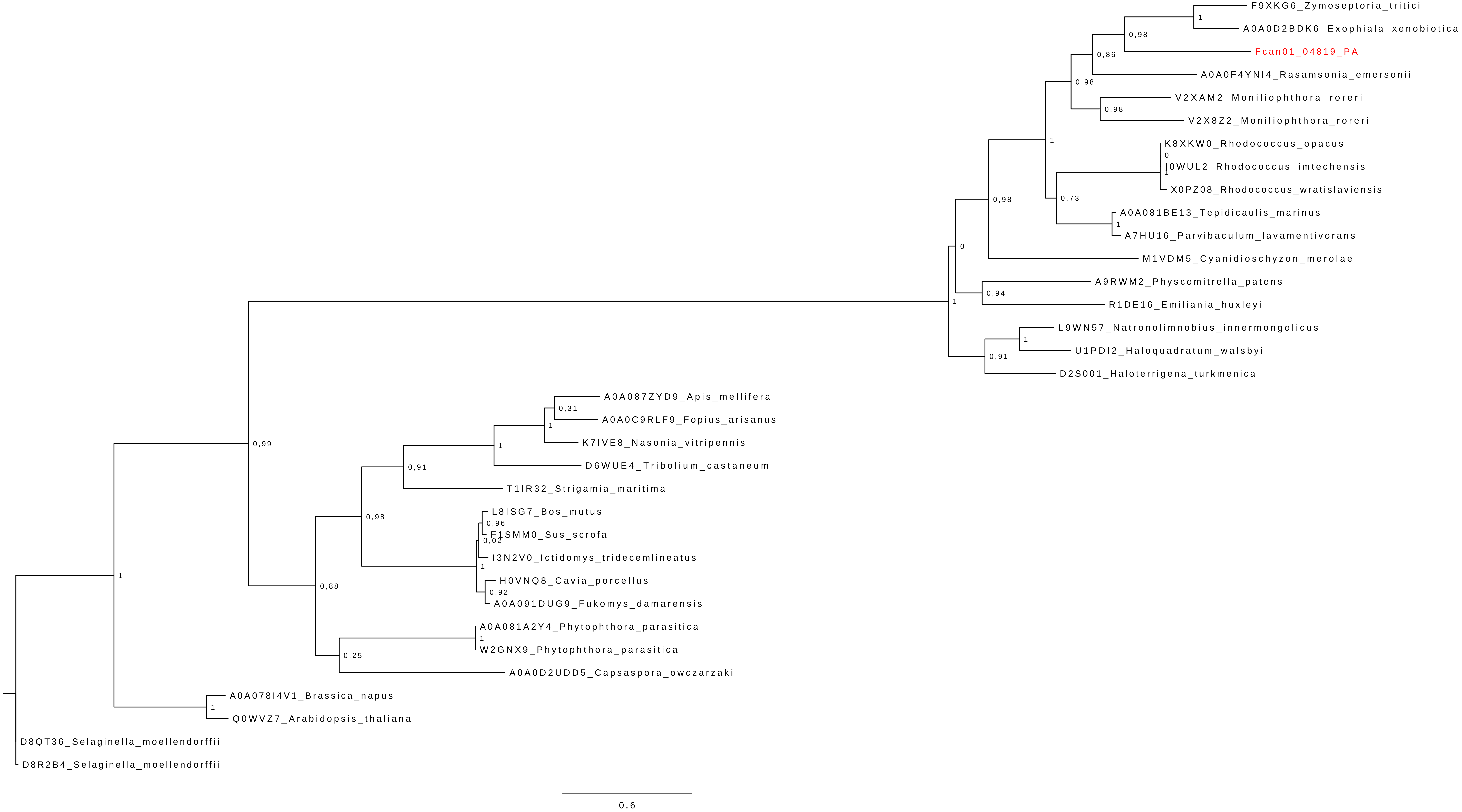
F9XKG6_Zymoseptoria_tritici
A0A0D2BDK6_Exophiala_xenobiotica
Fcan01_04819_PA
A0A0F4YNI4_Rasamsonia_emersonii
V2XAM2_Moniliophthora_roreri
V2X8Z2_Moniliophthora_roreri
K8XKW0_Rhodococcus_opacus
I0WUL2_Rhodococcus_imtechensis
X0PZ08_Rhodococcus_wratislaviensis
A0A081BE13_Tepidicaulis_marinus
A7HU16_Parvibaculum_lavamentivorans
M1VDM5_Cyanidioschyzon_merolae
A9RWM2_Physcomitrella_patens
R1DE16_Emiliania_huxleyi
L9WN57_Natronolimnobius_innermongolicus
U1PDI2_Haloquadratum_walsbyi
D2S001_Haloterrigena_turkmenica
A0A087ZYD9_Apis_mellifera
A0A0C9RLF9_Fopius_arisanus
K7IVE8_Nasonia_vitripennis
D6WUE4_Tribolium_castaneum
T1IR32_Strigamia_maritima
L8ISG7_Bos_mutus
F1SMM0_Sus_scrofa
I3N2V0_Ictidomys_tridecemlineatus
H0VNQ8_Cavia_porcellus
A0A091DUG9_Fukomys_damarensis
A0A081A2Y4_Phytophthora_parasitica
W2GNX9_Phytophthora_parasitica
A0A0D2UDD5_Capsaspora_owczarzaki
A0A078I4V1_Brassica_napus
Q0WVZ7_Arabidopsis_thaliana
D8QT36_Selaginella_moellendorffii
D8R2B4_Selaginella_moellendorffii
0.6

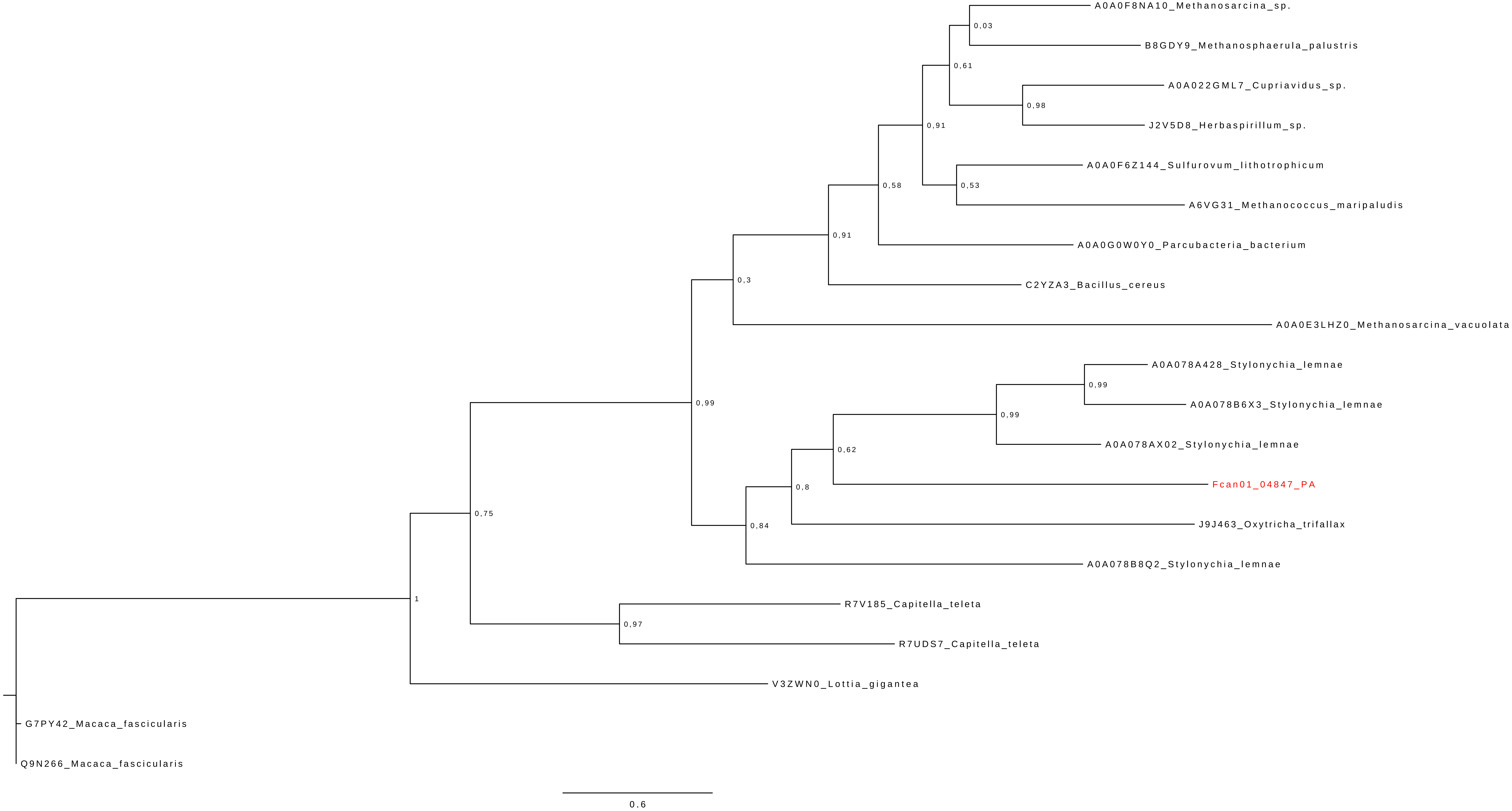
A0A0F8NA10_Methanosarcina_sp.
0,03
B8GDY9_Methanosphaerula_palustris
0,61
A0A022GML7_Cupriavidus_sp.
0,98
0,91
J2V5D8_Herbaspirillum_sp.
A0A0F6Z144_Sulfurovum_lithotrophicum
0,58
0,53
A6VG31_Methanococcus_maripaludis
0,91
A0A0G0W0Y0_Parcubacteria_bacterium
0,3
C2YZA3_Bacillus_cereus
A0A0E3LHZ0_Methanosarcina_vacuolata
A0A078A428_Stylonychia_lemnae
0,99
0,99
A0A078B6X3_Stylonychia_lemnae
0,99
A0A078AX02_Stylonychia_lemnae
0,62
0,8
Fcan01_04847_PA
0,75
0,84
J9J463_Oxytricha_trifallax
A0A078B8Q2_Stylonychia_lemnae
1
R7V185_Capitella_teleta
0,97
R7UDS7_Capitella_teleta
V3ZWN0_Lottia_gigantea
G7PY42_Macaca_fascicularis
Q9N266_Macaca_fascicularis
0.6

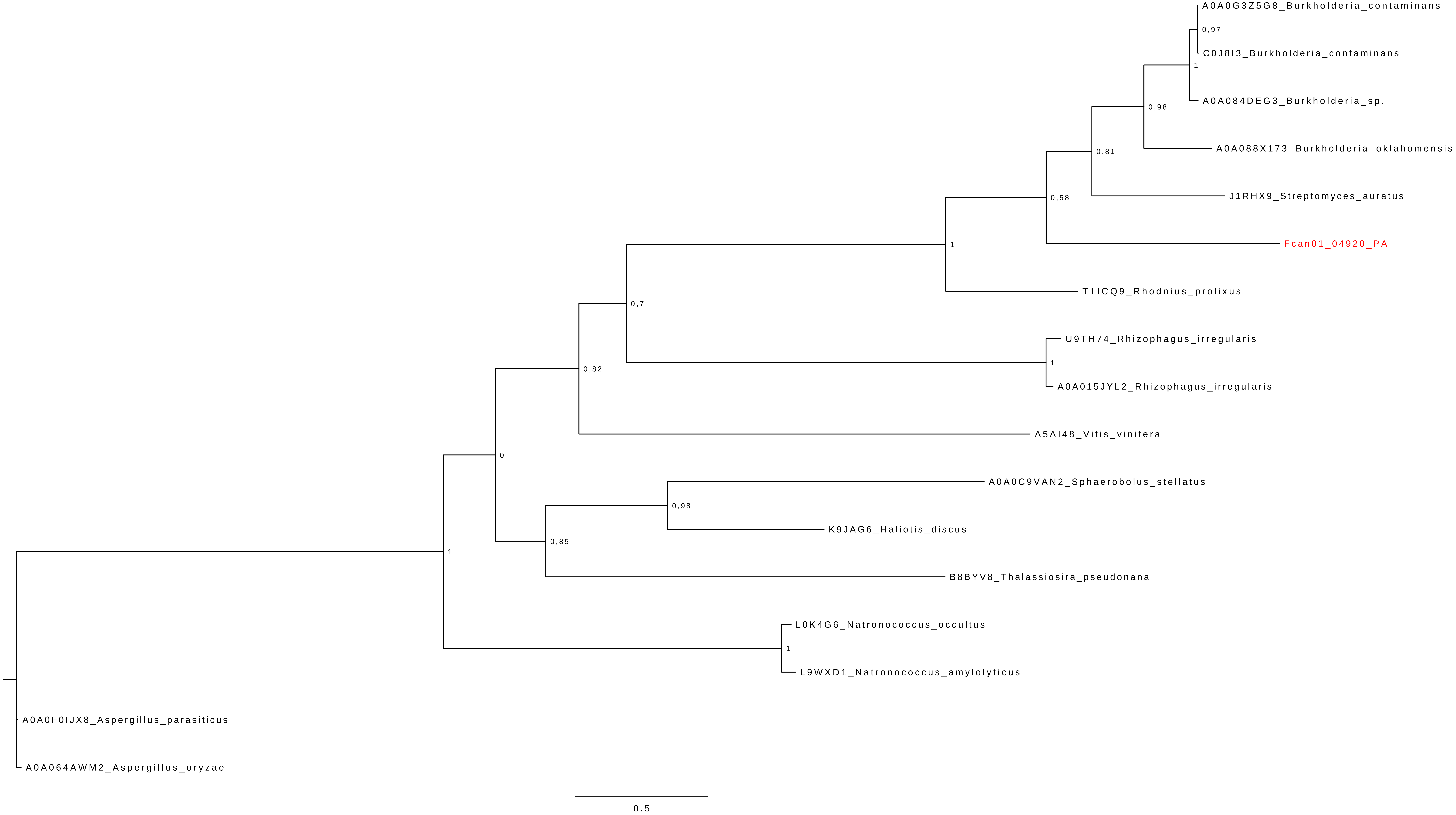
A0A0G3Z5G8_Burkholderia_contaminans
0,97
C0J8I3_Burkholderia_contaminans
1
A0A084DEG3_Burkholderia_sp.
0,98
A0A088X173_Burkholderia_oklahomensis
0,81
J1RHX9_Streptomyces_auratus
0,58
Fcan01_04920_PA
1
T1ICQ9_Rhodnius_prolixus
0,7
U9TH74_Rhizophagus_irregularis
1
A0A015JYL2_Rhizophagus_irregularis
0,82
A5AI48_Vitis_vinifera
0
A0A0C9VAN2_Sphaerobolus_stellatus
0,98
K9JAG6_Haliotis_discus
0,85
B8BYV8_Thalassiosira_pseudonana
1
L0K4G6_Natronococcus_occultus
1
L9WXD1_Natronococcus_amylolyticus
A0A0F0IJX8_Aspergillus_parasiticus
A0A064AWM2_Aspergillus_oryzae
0.5

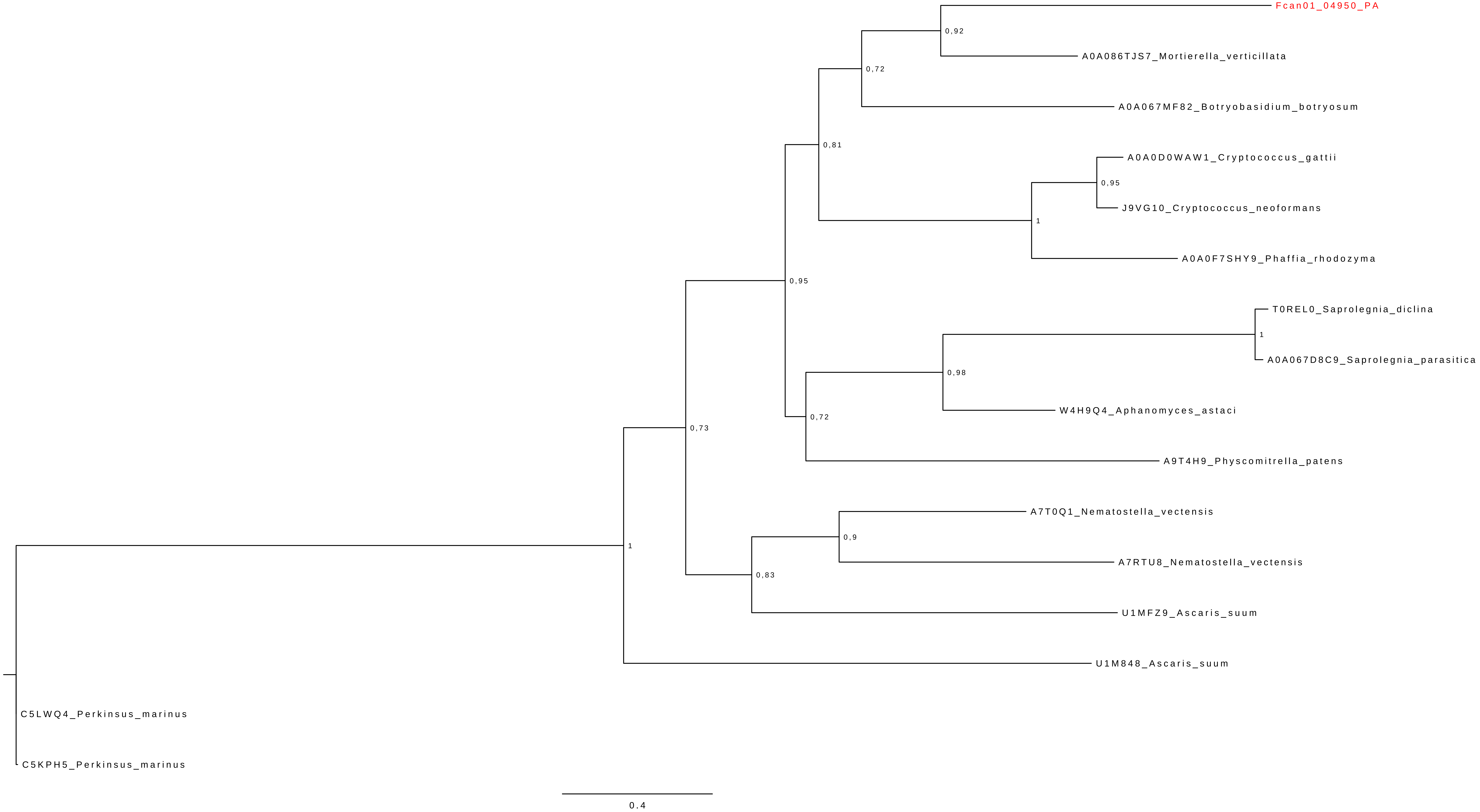

Fcan01_04950_PA
A0A086TJS7_Mortierella_verticillata
A0A067MF82_Botryobasidium_botryosum
A0A0D0WAW1_Cryptococcus_gattii
J9VG10_Cryptococcus_neoformans
A0A0F7SHY9_Phaffia_rhodozyma
T0REL0_Saprolegnia_diclina
A0A067D8C9_Saprolegnia_parasitica
W4H9Q4_Aphanomyces_astaci
A9T4H9_Physcomitrella_patens
A7T0Q1_Nematostella_vectensis
A7RTU8_Nematostella_vectensis
U1MFZ9_Ascaris_suum
U1M848_Ascaris_suum
C5LWQ4_Perkinsus_marinus
C5KPH5_Perkinsus_marinus
0,92
0,72
0,81
0,95
1
0,95
1
0,98
0,72
0,73
0,9
0,83
1
0.4

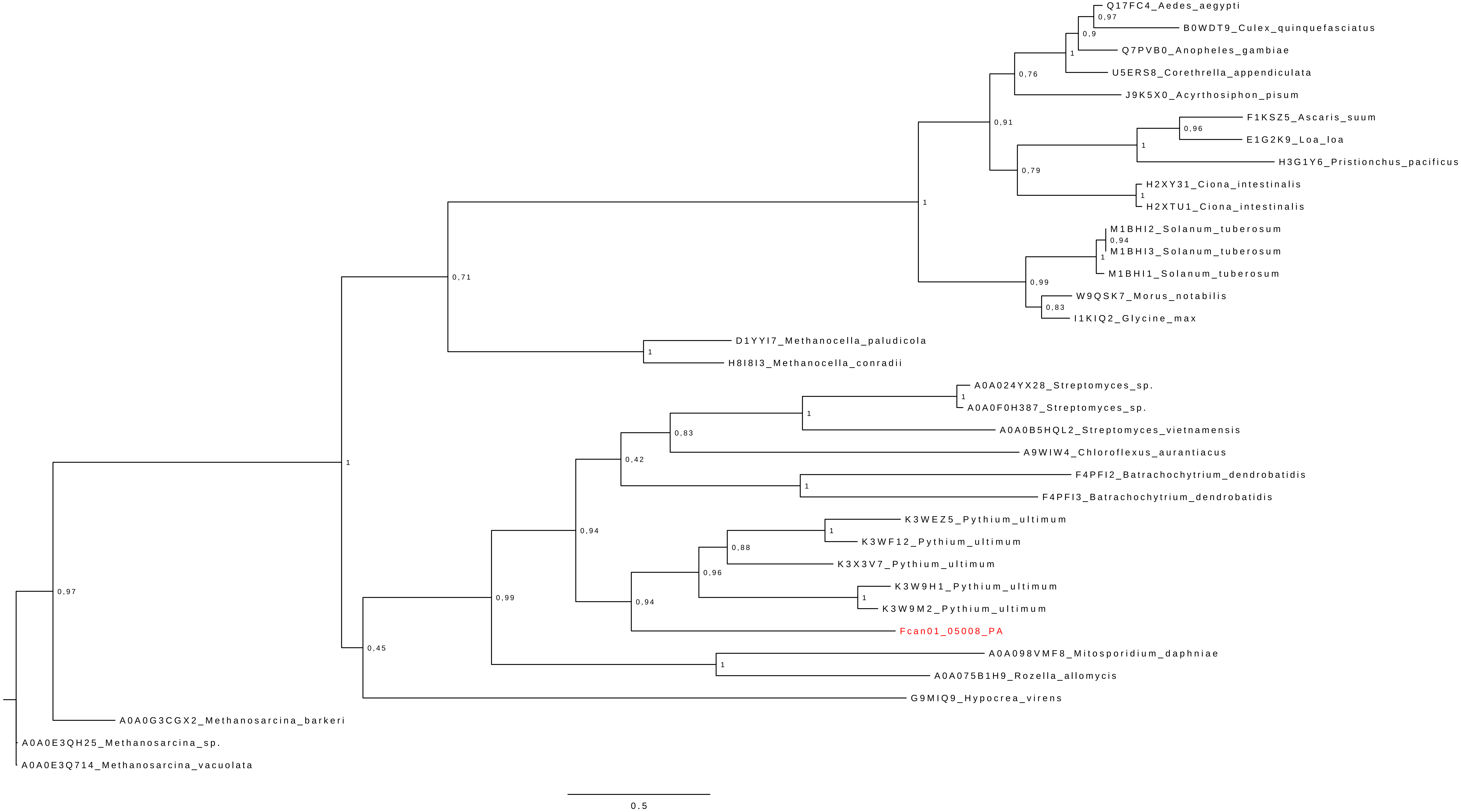
Q17FC4_Aedes_aegypti
0,97
B0WDT9_Culex_quinquefasciatus
0,9
Q7PVB0_Anopheles_gambiae
1
U5ERS8_Corethrella_appendiculata
0,76
J9K5X0_Acyrthosiphon_pisum
0,91
F1KSZ5_Ascaris_suum
0,96
E1G2K9_Loa_loa
1
H3G1Y6_Pristionchus_pacificus
0,79
H2XY31_Ciona_intestinalis
1
H2XTU1_Ciona_intestinalis
1
M1BHI2_Solanum_tuberosum
0,94
M1BHI3_Solanum_tuberosum
1
M1BHI1_Solanum_tuberosum
0,71
0,99
W9QSK7_Morus_notabilis
0,83
I1KIQ2_Glycine_max
D1YYI7_Methanocella_paludicola
1
H8I8I3_Methanocella_conradii
A0A024YX28_Streptomyces_sp.
1
A0A0F0H387_Streptomyces_sp.
1
A0A0B5HQL2_Streptomyces_vietnamensis
0,83
A9WIW4_Chloroflexus_aurantiacus
1
0,42
F4PFI2_Batrachochytrium_dendrobatidis
1
F4PFI3_Batrachochytrium_dendrobatidis
K3WEZ5_Pythium_ultimum
0,94
1
K3WF12_Pythium_ultimum
0,88
K3X3V7_Pythium_ultimum
0,96
K3W9H1_Pythium_ultimum
0,97
0,99
1
0,94
K3W9M2_Pythium_ultimum
Fcan01_05008_PA
A0A098VMF8_Mitosporidium_daphniae
0,45
1
A0A075B1H9_Rozella_allomycis
G9MIQ9_Hypocrea_virens
A0A0G3CGX2_Methanosarcina_barkeri
A0A0E3QH25_Methanosarcina_sp.
A0A0E3Q714_Methanosarcina_vacuolata
0.5

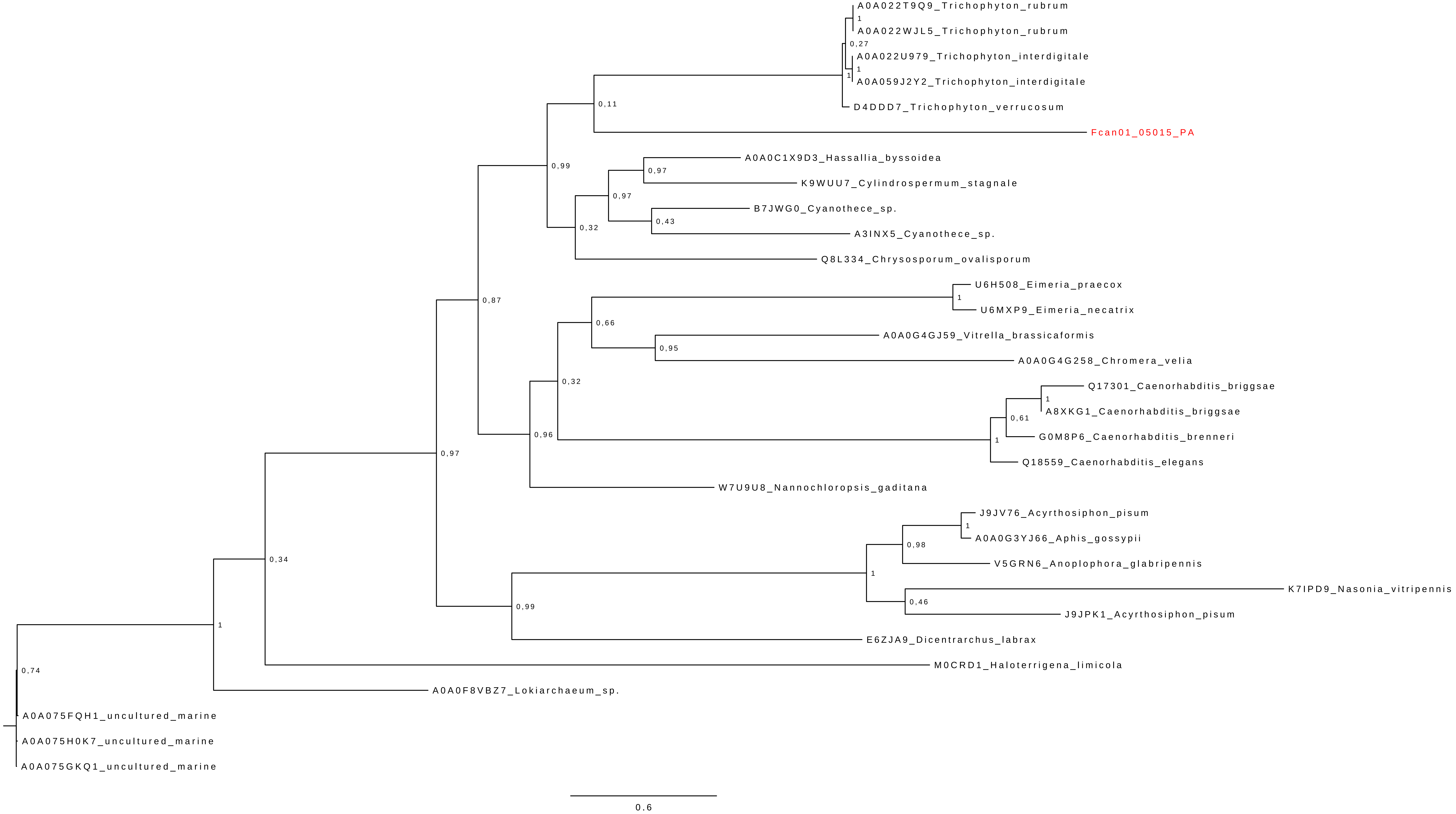
A0A022T9Q9_Trichophyton_rubrum
1
A0A022WJL5_Trichophyton_rubrum
0,27
A0A022U979_Trichophyton_interdigitale
1
1
A0A059J2Y2_Trichophyton_interdigitale
D4DDD7_Trichophyton_verrucosum
0,11
Fcan01_05015_PA
A0A0C1X9D3_Hassallia_byssoidea
0,99
0,97
K9WUU7_Cylindrospermum_stagnale
0,97
B7JWG0_Cyanothece_sp.
0,43
0,32
A3INX5_Cyanothece_sp.
Q8L334_Chrysosporum_ovalisporum
U6H508_Eimeria_praecox
1
0,87
U6MXP9_Eimeria_necatrix
0,66
A0A0G4GJ59_Vitrella_brassicaformis
0,95
A0A0G4G258_Chromera_velia
0,32
Q17301_Caenorhabditis_briggsae
1
A8XKG1_Caenorhabditis_briggsae
0,61
0,96
G0M8P6_Caenorhabditis_brenneri
1
0,97
Q18559_Caenorhabditis_elegans
W7U9U8_Nannochloropsis_gaditana
J9JV76_Acyrthosiphon_pisum
1
A0A0G3YJ66_Aphis_gossypii
0,98
V5GRN6_Anoplophora_glabripennis
0,34
1
K7IPD9_Nasonia_vitripennis
0,46
0,99
J9JPK1_Acyrthosiphon_pisum
1
E6ZJA9_Dicentrarchus_labrax
M0CRD1_Haloterrigena_limicola
0,74
A0A0F8VBZ7_Lokiarchaeum_sp.
A0A075FQH1_uncultured_marine
A0A075H0K7_uncultured_marine
A0A075GKQ1_uncultured_marine
0.6

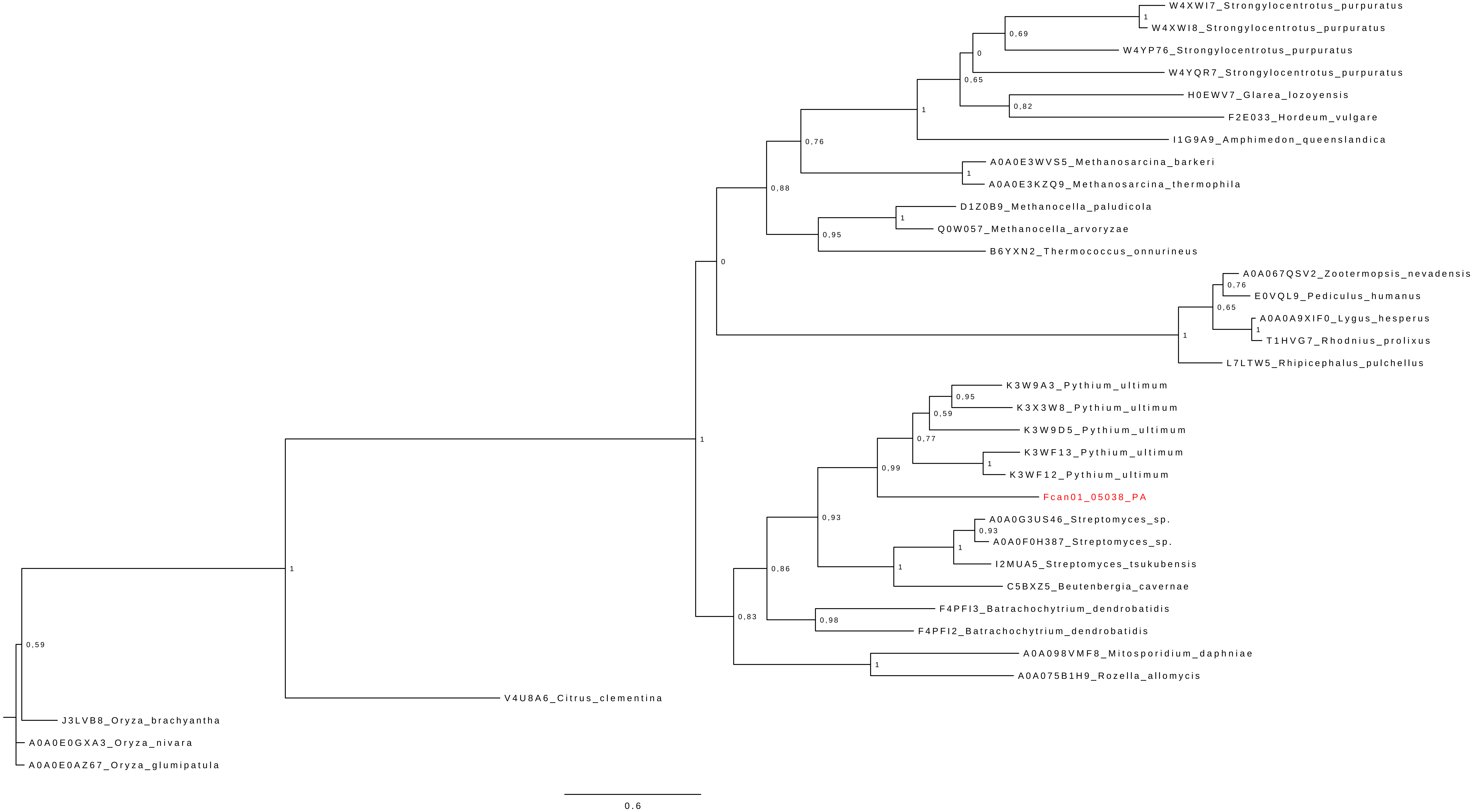
W4XWI7_Strongylocentrotus_purpuratus
W4XWI8_Strongylocentrotus_purpuratus
W4YP76_Strongylocentrotus_purpuratus
W4YQR7_Strongylocentrotus_purpuratus
H0EWV7_Glarea_lozoyensis
F2E033_Hordeum_vulgare
I1G9A9_Amphimedon_queenslandica
A0A0E3WVS5_Methanosarcina_barkeri
A0A0E3KZQ9_Methanosarcina_thermophila
D1Z0B9_Methanocella_paludicola
Q0W057_Methanocella_arvoryzae
B6YXN2_Thermococcus_onnurineus
A0A067QSV2_Zootermopsis_nevadensis
E0VQL9_Pediculus_humanus
A0A0A9XIF0_Lygus_hesperus
T1HVG7_Rhodnius_prolixus
L7LTW5_Rhipicephalus_pulchellus
K3W9A3_Pythium_ultimum
K3X3W8_Pythium_ultimum
K3W9D5_Pythium_ultimum
K3WF13_Pythium_ultimum
K3WF12_Pythium_ultimum
Fcan01_05038_PA
A0A0G3US46_Streptomyces_sp.
A0A0F0H387_Streptomyces_sp.
I2MUA5_Streptomyces_tsukubensis
C5BXZ5_Beutenbergia_cavernae
F4PFI3_Batrachochytrium_dendrobatidis
F4PFI2_Batrachochytrium_dendrobatidis
A0A098VMF8_Mitosporidium_daphniae
A0A075B1H9_Rozella_allomycis
V4U8A6_Citrus_clementina
J3LVB8_Oryza_brachyantha
A0A0E0GXA3_Oryza_nivara
A0A0E0AZ67_Oryza_glumipatula
1
0,69
0
0,65
0,82
1
0,76
1
0,88
1
0,95
0
0,76
0,65
1
1
0,95
0,59
0,77
1
0,99
0,93
0,93
1
1
0,86
0,98
0,83
1
1
1
0,59
0.6

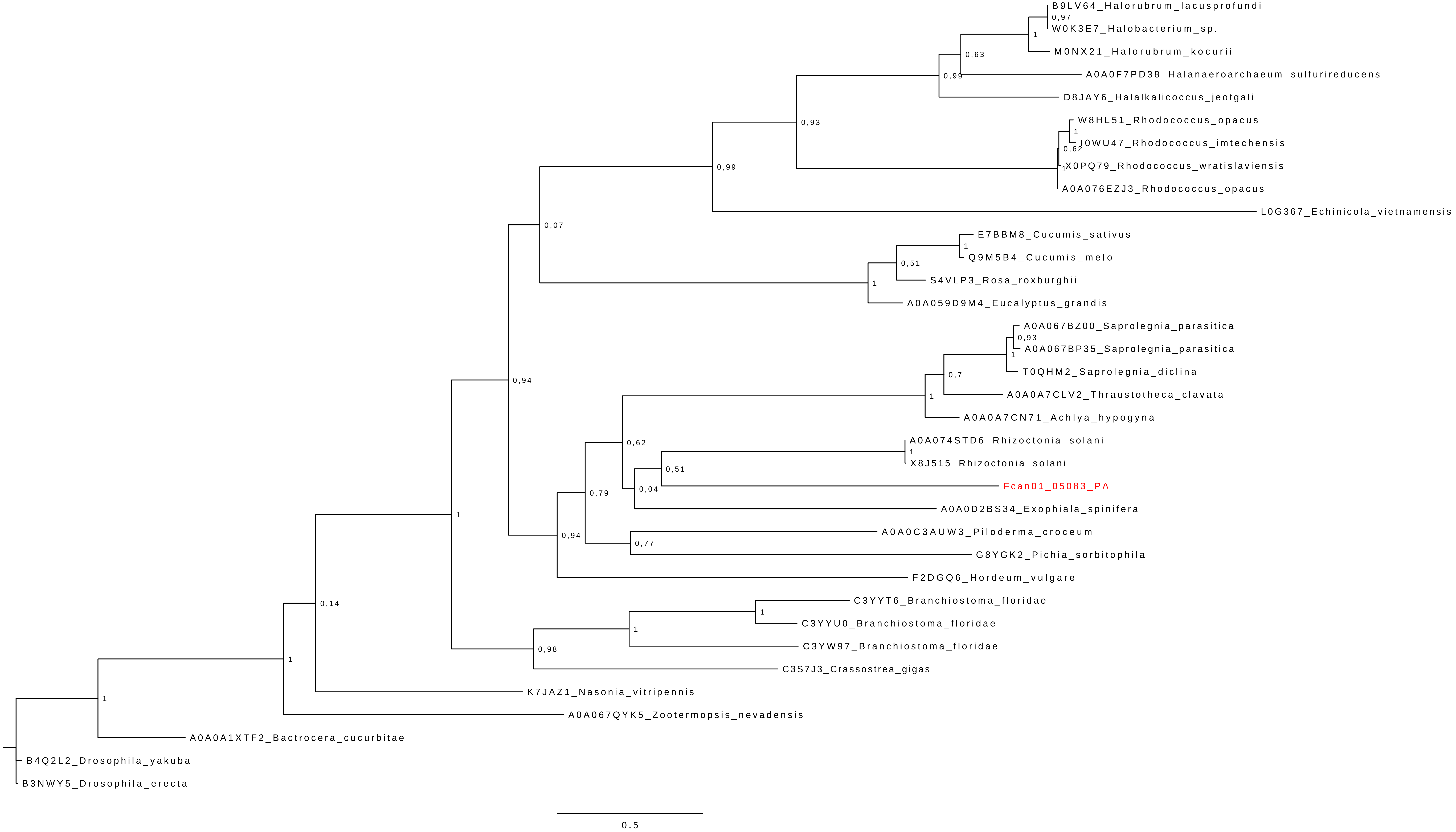
B9LV64_Halorubrum_lacusprofundi
W0K3E7_Halobacterium_sp.
M0NX21_Halorubrum_kocurii
A0A0F7PD38_Halanaeroarchaeum_sulfurireducens
D8JAY6_Halalkalicoccus_jeotgali
W8HL51_Rhodococcus_opacus
J0WU47_Rhodococcus_imtechensis
X0PQ79_Rhodococcus_wratislaviensis
A0A076EZJ3_Rhodococcus_opacus
L0G367_Echinicola_vietnamensis
E7BBM8_Cucumis_sativus
Q9M5B4_Cucumis_melo
S4VLP3_Rosa_roxburghii
A0A059D9M4_Eucalyptus_grandis
A0A067BZ00_Saprolegnia_parasitica
A0A067BP35_Saprolegnia_parasitica
T0QHM2_Saprolegnia_diclina
A0A0A7CLV2_Thraustotheca_clavata
A0A0A7CN71_Achlya_hypogyna
A0A074STD6_Rhizoctonia_solani
X8J515_Rhizoctonia_solani
Fcan01_05083_PA
A0A0D2BS34_Exophiala_spinifera
A0A0C3AUW3_Piloderma_croceum
G8YGK2_Pichia_sorbitophila
F2DGQ6_Hordeum_vulgare
C3YYT6_Branchiostoma_floridae
C3YYU0_Branchiostoma_floridae
C3YW97_Branchiostoma_floridae
C3S7J3_Crassostrea_gigas
K7JAZ1_Nasonia_vitripennis
A0A067QYK5_Zootermopsis_nevadensis
A0A0A1XTF2_Bactrocera_cucurbitae
B4Q2L2_Drosophila_yakuba
B3NWY5_Drosophila_erecta
0.5

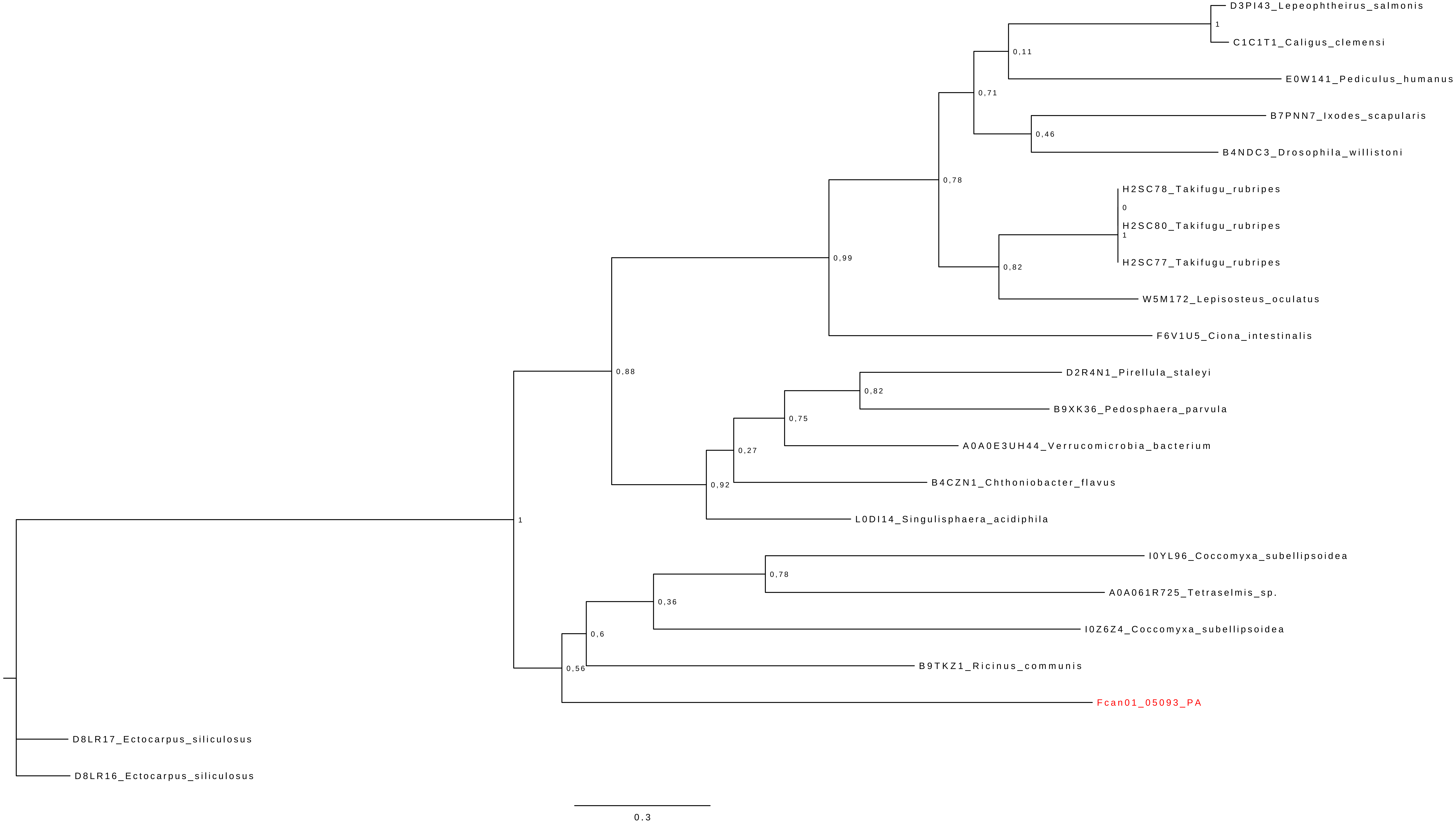
D3PI43_Lepeophtheirus_salmonis
1
C1C1T1_Caligus_clemensi
0,11
E0W141_Pediculus_humanus
0,71
B7PNN7_Ixodes_scapularis
0,46
B4NDC3_Drosophila_willistoni
0,78
H2SC78_Takifugu_rubripes
0
H2SC80_Takifugu_rubripes
1
H2SC77_Takifugu_rubripes
0,82
W5M172_Lepisosteus_oculatus
0,99
F6V1U5_Ciona_intestinalis
D2R4N1_Pirellula_staleyi
0,88
0,82
B9XK36_Pedosphaera_parvula
0,75
A0A0E3UH44_Verrucomicrobia_bacterium
0,27
B4CZN1_Chthoniobacter_flavus
0,92
L0DI14_Singulisphaera_acidiphila
1
I0YL96_Coccomyxa_subellipsoidea
0,78
A0A061R725_Tetraselmis_sp.
0,36
I0Z6Z4_Coccomyxa_subellipsoidea
0,6
B9TKZ1_Ricinus_communis
0,56
Fcan01_05093_PA
D8LR17_Ectocarpus_siliculosus
D8LR16_Ectocarpus_siliculosus
0.3

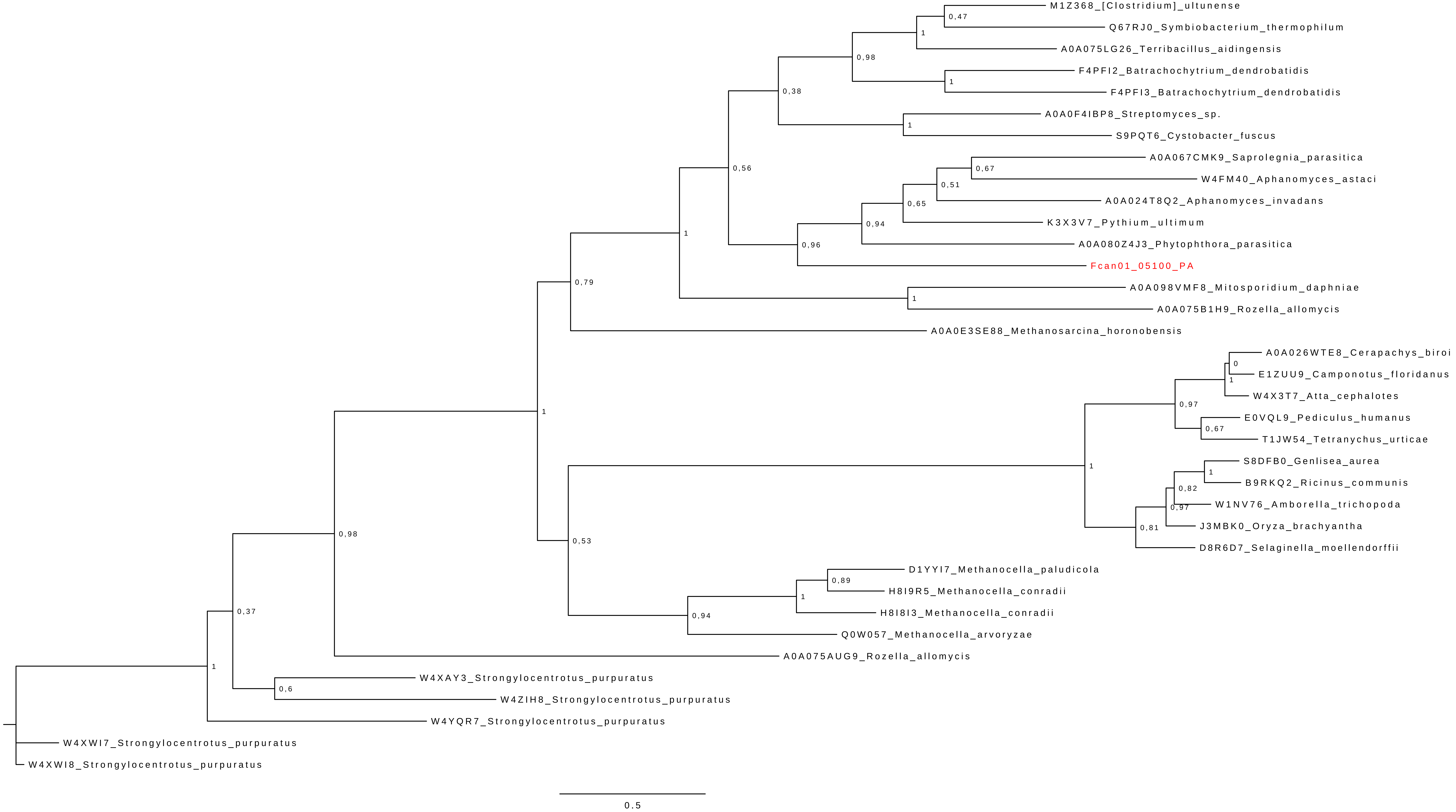
M1Z368_[Clostridium]_ultunense
Q67RJ0_Symbiobacterium_thermophilum
A0A075LG26_Terribacillus_aidingensis
F4PFI2_Batrachochytrium_dendrobatidis
F4PFI3_Batrachochytrium_dendrobatidis
A0A0F4IBP8_Streptomyces_sp.
S9PQT6_Cystobacter_fuscus
A0A067CMK9_Saprolegnia_parasitica
W4FM40_Aphanomyces_astaci
A0A024T8Q2_Aphanomyces_invadans
K3X3V7_Pythium_ultimum
A0A080Z4J3_Phytophthora_parasitica
Fcan01_05100_PA
A0A098VMF8_Mitosporidium_daphniae
A0A075B1H9_Rozella_allomycis
A0A0E3SE88_Methanosarcina_horonobensis
A0A026WTE8_Cerapachys_biroi
E1ZUU9_Camponotus_floridanus
W4X3T7_Atta_cephalotes
E0VQL9_Pediculus_humanus
T1JW54_Tetranychus_urticae
S8DFB0_Genlisea_aurea
B9RKQ2_Ricinus_communis
W1NV76_Amborella_trichopoda
J3MBK0_Oryza_brachyantha
D8R6D7_Selaginella_moellendorffii
D1YYI7_Methanocella_paludicola
H8I9R5_Methanocella_conradii
H8I8I3_Methanocella_conradii
Q0W057_Methanocella_arvoryzae
A0A075AUG9_Rozella_allomycis
W4XAY3_Strongylocentrotus_purpuratus
W4ZIH8_Strongylocentrotus_purpuratus
W4YQR7_Strongylocentrotus_purpuratus
W4XWI7_Strongylocentrotus_purpuratus
W4XWI8_Strongylocentrotus_purpuratus
0.5

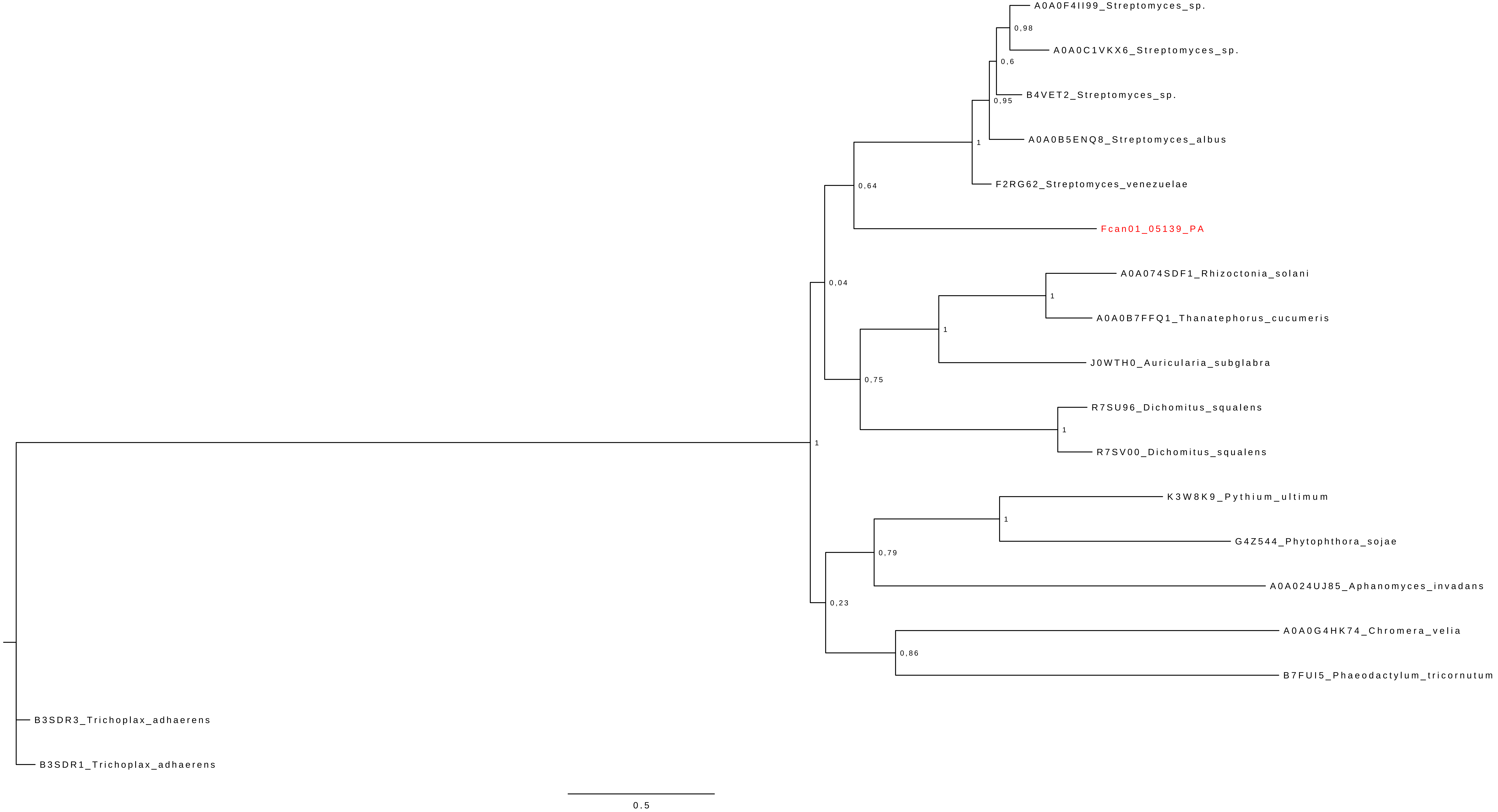
A0A0F4II99_Streptomyces_sp.
0,98
A0A0C1VKX6_Streptomyces_sp.
0,6
B4VET2_Streptomyces_sp.
0,95
A0A0B5ENQ8_Streptomyces_albus
1
F2RG62_Streptomyces_venezuelae
0,64
Fcan01_05139_PA
0,04
A0A074SDF1_Rhizoctonia_solani
1
A0A0B7FFQ1_Thanatephorus_cucumeris
1
J0WTH0_Auricularia_subglabra
0,75
R7SU96_Dichomitus_squalens
1
R7SV00_Dichomitus_squalens
1
K3W8K9_Pythium_ultimum
1
G4Z544_Phytophthora_sojae
0,79
A0A024UJ85_Aphanomyces_invadans
0,23
A0A0G4HK74_Chromera_velia
0,86
B7FUI5_Phaeodactylum_tricornutum
B3SDR3_Trichoplax_adhaerens
B3SDR1_Trichoplax_adhaerens
0.5

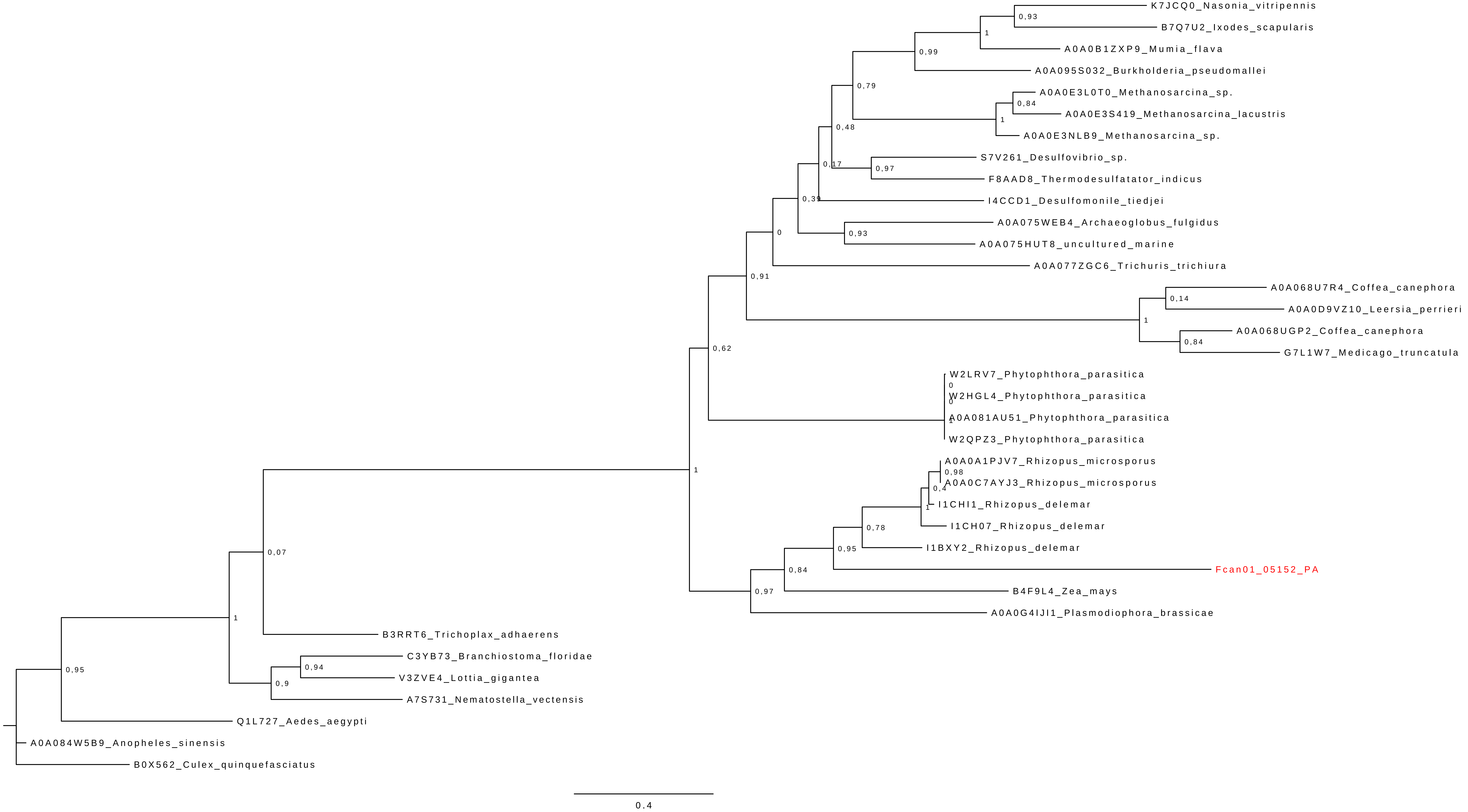
K7JCQ0_Nasonia_vitripennis
B7Q7U2_Ixodes_scapularis
A0A0B1ZXP9_Mumia_flava
A0A095S032_Burkholderia_pseudomallei
A0A0E3L0T0_Methanosarcina_sp.
A0A0E3S419_Methanosarcina_lacustris
A0A0E3NLB9_Methanosarcina_sp.
S7V261_Desulfovibrio_sp.
F8AAD8_Thermodesulfatator_indicus
I4CCD1_Desulfomonile_tiedjei
A0A075WEB4_Archaeoglobus_fulgidus
A0A075HUT8_uncultured_marine
A0A077ZGC6_Trichuris_trichiura
A0A068U7R4_Coffea_canephora
A0A0D9VZ10_Leersia_perrieri
A0A068UGP2_Coffea_canephora
G7L1W7_Medicago_truncatula
W2LRV7_Phytophthora_parasitica
W2HGL4_Phytophthora_parasitica
A0A081AU51_Phytophthora_parasitica
W2QPZ3_Phytophthora_parasitica
A0A0A1PJV7_Rhizopus_microsporus
A0A0C7AYJ3_Rhizopus_microsporus
I1CHI1_Rhizopus_delemar
I1CH07_Rhizopus_delemar
I1BXY2_Rhizopus_delemar
Fcan01_05152_PA
B4F9L4_Zea_mays
A0A0G4IJI1_Plasmodiophora_brassicae
B3RRT6_Trichoplax_adhaerens
C3YB73_Branchiostoma_floridae
V3ZVE4_Lottia_gigantea
A7S731_Nematostella_vectensis
Q1L727_Aedes_aegypti
A0A084W5B9_Anopheles_sinensis
B0X562_Culex_quinquefasciatus
0.4

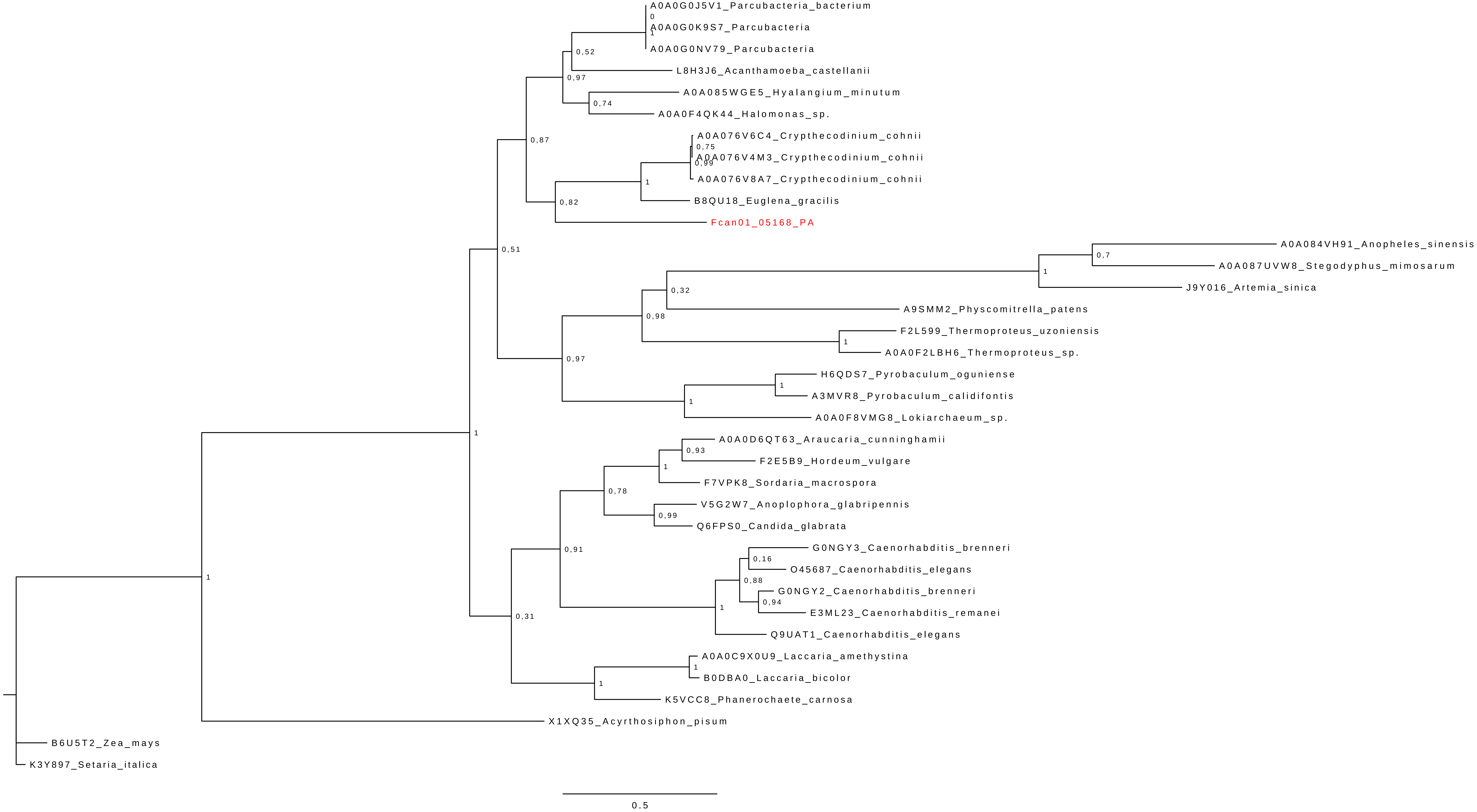
A0A0G0J5V1_Parcubacteria_bacterium
0
A0A0G0K9S7_Parcubacteria
1
A0A0G0NV79_Parcubacteria
0,52
L8H3J6_Acanthamoeba_castellanii
0,97
A0A085WGE5_Hyalangium_minutum
0,74
A0A0F4QK44_Halomonas_sp.
0,87
A0A076V6C4_Crypthecodinium_cohnii
0,75
A0A076V4M3_Crypthecodinium_cohnii
0,99
A0A076V8A7_Crypthecodinium_cohnii
1
B8QU18_Euglena_gracilis
0,82
Fcan01_05168_PA
0,51
A0A084VH91_Anopheles_sinensis
0,7
A0A087UVW8_Stegodyphus_mimosarum
1
J9Y016_Artemia_sinica
0,32
A9SMM2_Physcomitrella_patens
0,98
F2L599_Thermoproteus_uzoniensis
1
A0A0F2LBH6_Thermoproteus_sp.
0,97
H6QDS7_Pyrobaculum_oguniense
1
A3MVR8_Pyrobaculum_calidifontis
1
A0A0F8VMG8_Lokiarchaeum_sp.
1
A0A0D6QT63_Araucaria_cunninghamii
0,93
F2E5B9_Hordeum_vulgare
1
F7VPK8_Sordaria_macrospora
0,78
V5G2W7_Anoplophora_glabripennis
0,99
Q6FPS0_Candida_glabrata
0,91
G0NGY3_Caenorhabditis_brenneri
0,16
O45687_Caenorhabditis_elegans
0,88
G0NGY2_Caenorhabditis_brenneri
0,94
E3ML23_Caenorhabditis_remanei
1
Q9UAT1_Caenorhabditis_elegans
0,31
A0A0C9X0U9_Laccaria_amethystina
1
B0DBA0_Laccaria_bicolor
1
K5VCC8_Phanerochaete_carnosa
1
X1XQ35_Acyrthosiphon_pisum
B6U5T2_Zea_mays
K3Y897_Setaria_italica
0.5

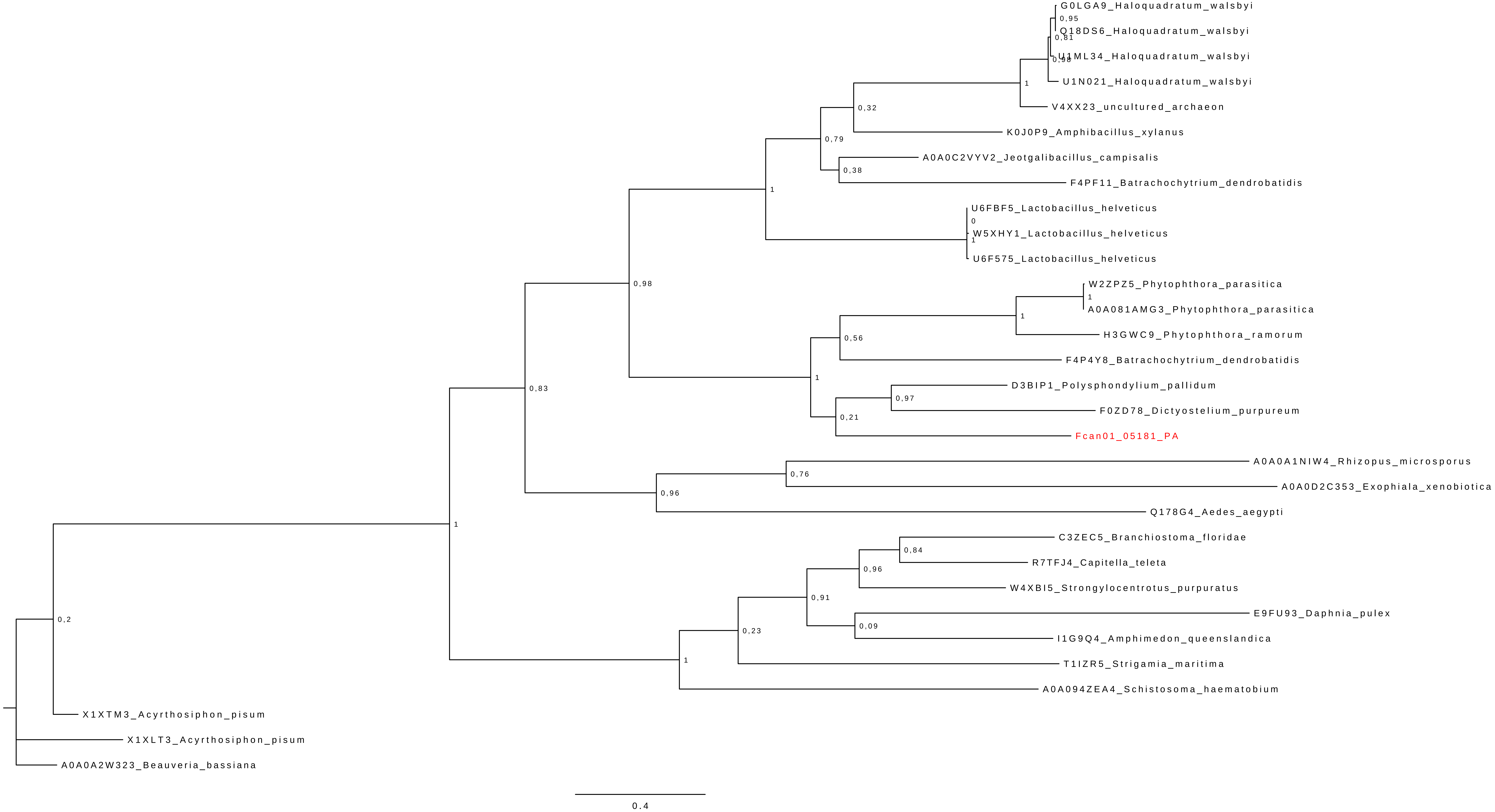
G0LGA9_Haloquadratum_walsbyi
0,95
Q18DS6_Haloquadratum_walsbyi
0,81
U1ML34_Haloquadratum_walsbyi
0,98
U1N021_Haloquadratum_walsbyi
1
V4XX23_uncultured_archaeon
0,32
K0J0P9_Amphibacillus_xylanus
0,79
A0A0C2VYV2_Jeotgalibacillus_campisalis
0,38
F4PF11_Batrachochytrium_dendrobatidis
1
U6FBF5_Lactobacillus_helveticus
0
W5XHY1_Lactobacillus_helveticus
1
U6F575_Lactobacillus_helveticus
0,98
W2ZPZ5_Phytophthora_parasitica
1
A0A081AMG3_Phytophthora_parasitica
1
H3GWC9_Phytophthora_ramorum
0,56
F4P4Y8_Batrachochytrium_dendrobatidis
1
D3BIP1_Polysphondylium_pallidum
0,97
F0ZD78_Dictyostelium_purpureum
0,21
Fcan01_05181_PA
0,83
A0A0A1NIW4_Rhizopus_microsporus
0,76
A0A0D2C353_Exophiala_xenobiotica
0,96
Q178G4_Aedes_aegypti
1
C3ZEC5_Branchiostoma_floridae
0,84
R7TFJ4_Capitella_teleta
0,96
W4XBI5_Strongylocentrotus_purpuratus
0,91
E9FU93_Daphnia_pulex
0,09
I1G9Q4_Amphimedon_queenslandica
0,23
T1IZR5_Strigamia_maritima
1
A0A094ZEA4_Schistosoma_haematobium
0,2
X1XTM3_Acyrthosiphon_pisum
X1XLT3_Acyrthosiphon_pisum
A0A0A2W323_Beauveria_bassiana
0.4

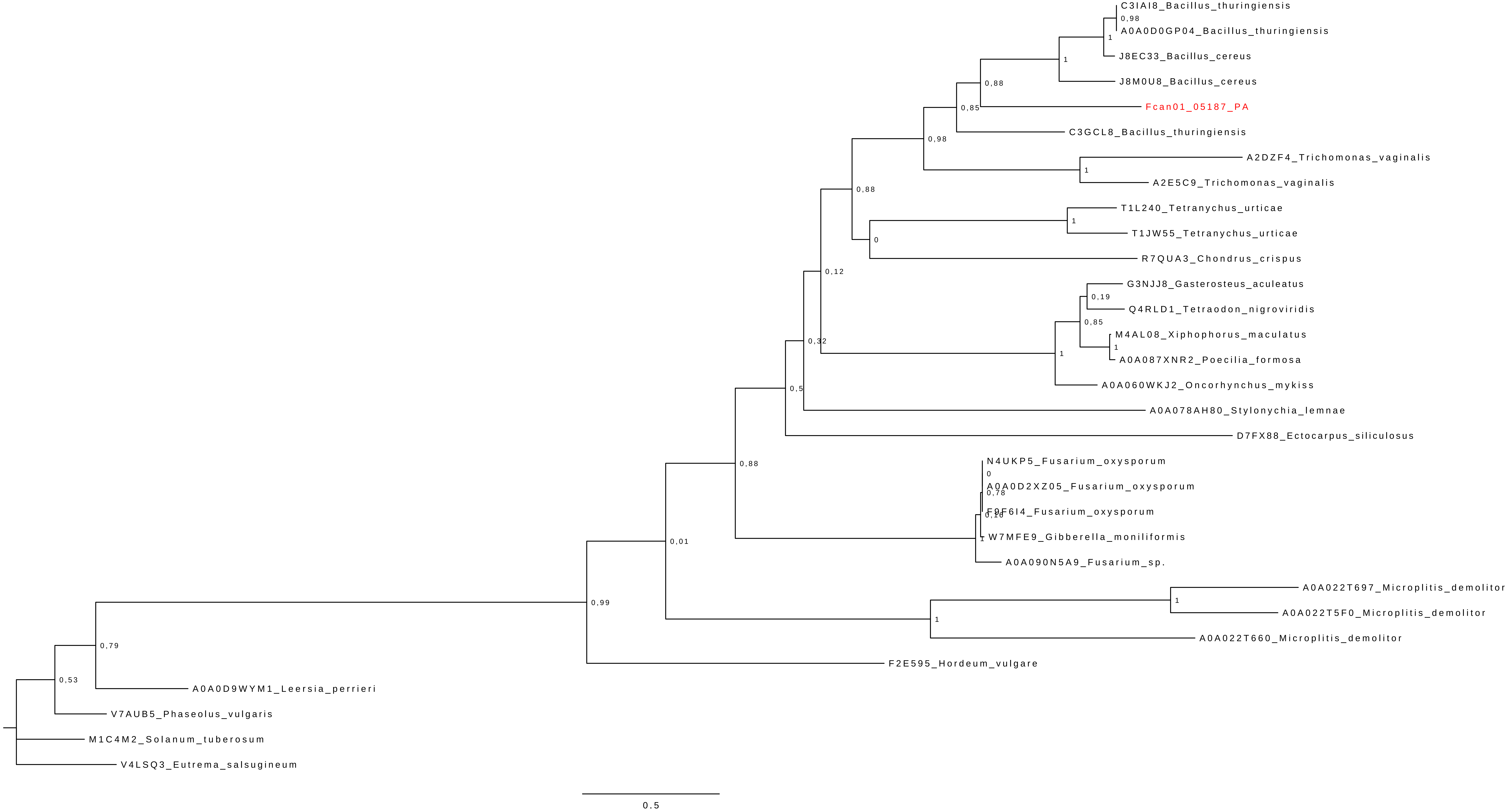
C3IAI8_Bacillus_thuringiensis
0,98
A0A0D0GP04_Bacillus_thuringiensis
1
J8EC33_Bacillus_cereus
1
J8M0U8_Bacillus_cereus
0,88
Fcan01_05187_PA
0,85
C3GCL8_Bacillus_thuringiensis
0,98
A2DZF4_Trichomonas_vaginalis
1
A2E5C9_Trichomonas_vaginalis
0,88
T1L240_Tetranychus_urticae
1
T1JW55_Tetranychus_urticae
0
R7QUA3_Chondrus_crispus
0,12
G3NJJ8_Gasterosteus_aculeatus
0,19
Q4RLD1_Tetraodon_nigroviridis
0,85
M4AL08_Xiphophorus_maculatus
1
A0A087XNR2_Poecilia_formosa
0,32
1
A0A060WKJ2_Oncorhynchus_mykiss
0,5
A0A078AH80_Stylonychia_lemnae
D7FX88_Ectocarpus_siliculosus
0,88
N4UKP5_Fusarium_oxysporum
0
A0A0D2XZ05_Fusarium_oxysporum
0,78
F9F6I4_Fusarium_oxysporum
0,26
W7MFE9_Gibberella_moniliformis
1
A0A090N5A9_Fusarium_sp.
0,01
A0A022T697_Microplitis_demolitor
1
A0A022T5F0_Microplitis_demolitor
0,99
1
A0A022T660_Microplitis_demolitor
F2E595_Hordeum_vulgare
0,79
A0A0D9WYM1_Leersia_perrieri
0,53
V7AUB5_Phaseolus_vulgaris
M1C4M2_Solanum_tuberosum
V4LSQ3_Eutrema_salsugineum
0.5

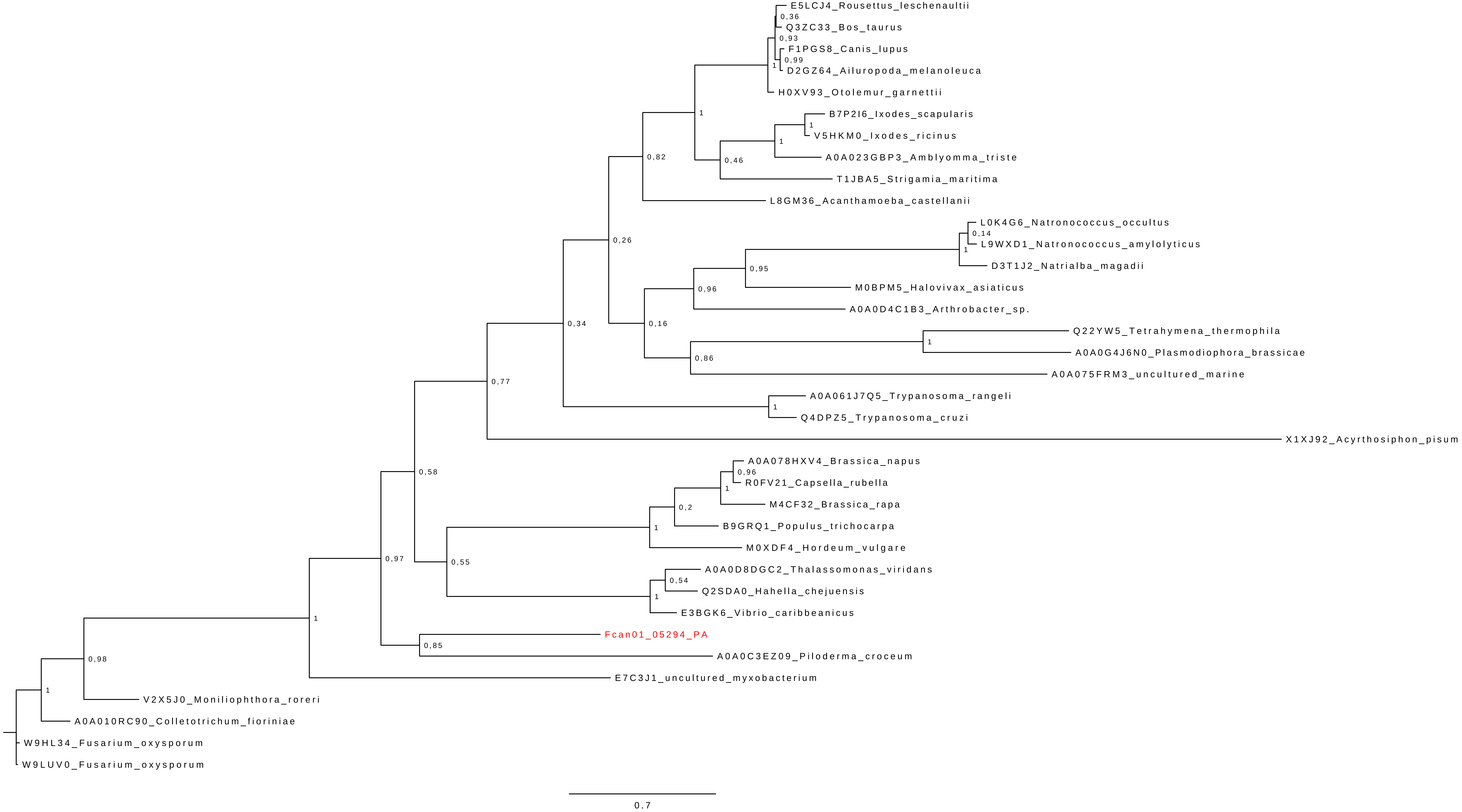
E5LCJ4_Rousettus_leschenaultii
0,36
Q3ZC33_Bos_taurus
0,93
F1PGS8_Canis_lupus
0,99
1
D2GZ64_Ailuropoda_melanoleuca
H0XV93_Otolemur_garnettii
1
B7P2I6_Ixodes_scapularis
1
V5HKM0_Ixodes_ricinus
1
A0A023GBP3_Amblyomma_triste
0,82
0,46
T1JBA5_Strigamia_maritima
L8GM36_Acanthamoeba_castellanii
L0K4G6_Natronococcus_occultus
0,14
L9WXD1_Natronococcus_amylolyticus
1
0,26
D3T1J2_Natrialba_magadii
0,95
M0BPM5_Halovivax_asiaticus
0,96
A0A0D4C1B3_Arthrobacter_sp.
0,34
0,16
Q22YW5_Tetrahymena_thermophila
1
A0A0G4J6N0_Plasmodiophora_brassicae
0,86
A0A075FRM3_uncultured_marine
0,77
A0A061J7Q5_Trypanosoma_rangeli
1
Q4DPZ5_Trypanosoma_cruzi
X1XJ92_Acyrthosiphon_pisum
A0A078HXV4_Brassica_napus
0,58
0,96
R0FV21_Capsella_rubella
1
M4CF32_Brassica_rapa
0,2
B9GRQ1_Populus_trichocarpa
1
M0XDF4_Hordeum_vulgare
0,97
0,55
A0A0D8DGC2_Thalassomonas_viridans
0,54
Q2SDA0_Hahella_chejuensis
1
E3BGK6_Vibrio_caribbeanicus
1
Fcan01_05294_PA
0,85
A0A0C3EZ09_Piloderma_croceum
E7C3J1_uncultured_myxobacterium
0,98
1
V2X5J0_Moniliophthora_roreri
A0A010RC90_Colletotrichum_fioriniae
W9HL34_Fusarium_oxysporum
W9LUV0_Fusarium_oxysporum
0.7

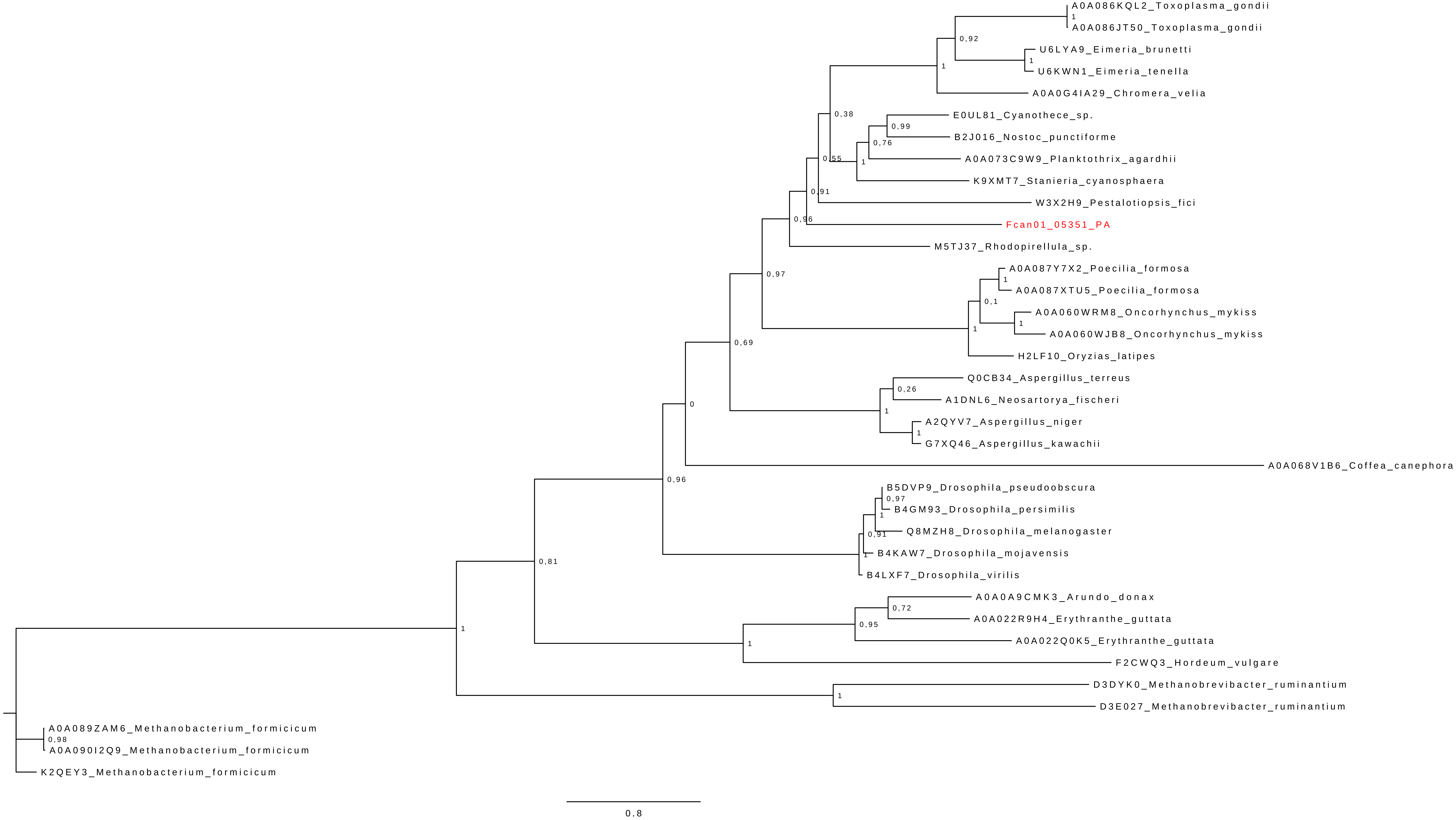
A0A086KQL2_Toxoplasma_gondii
A0A086JT50_Toxoplasma_gondii
U6LYA9_Eimeria_brunetti
U6KWN1_Eimeria_tenella
A0A0G4IA29_Chromera_velia
E0UL81_Cyanothece_sp.
B2J016_Nostoc_punctiforme
A0A073C9W9_Planktothrix_agardhii
K9XMT7_Stanieria_cyanosphaera
W3X2H9_Pestalotiopsis_fici
Fcan01_05351_PA
M5TJ37_Rhodopirellula_sp.
A0A087Y7X2_Poecilia_formosa
A0A087XTU5_Poecilia_formosa
A0A060WRM8_Oncorhynchus_mykiss
A0A060WJB8_Oncorhynchus_mykiss
H2LF10_Oryzias_latipes
Q0CB34_Aspergillus_terreus
A1DNL6_Neosartorya_fischeri
A2QYV7_Aspergillus_niger
G7XQ46_Aspergillus_kawachii
A0A068V1B6_Coffea_canephora
B5DVP9_Drosophila_pseudoobscura
B4GM93_Drosophila_persimilis
Q8MZH8_Drosophila_melanogaster
B4KAW7_Drosophila_mojavensis
B4LXF7_Drosophila_virilis
A0A0A9CMK3_Arundo_donax
A0A022R9H4_Erythranthe_guttata
A0A022Q0K5_Erythranthe_guttata
F2CWQ3_Hordeum_vulgare
D3DYK0_Methanobrevibacter_ruminantium
D3E027_Methanobrevibacter_ruminantium
A0A089ZAM6_Methanobacterium_formicicum
A0A090I2Q9_Methanobacterium_formicicum
K2QEY3_Methanobacterium_formicicum
0.8

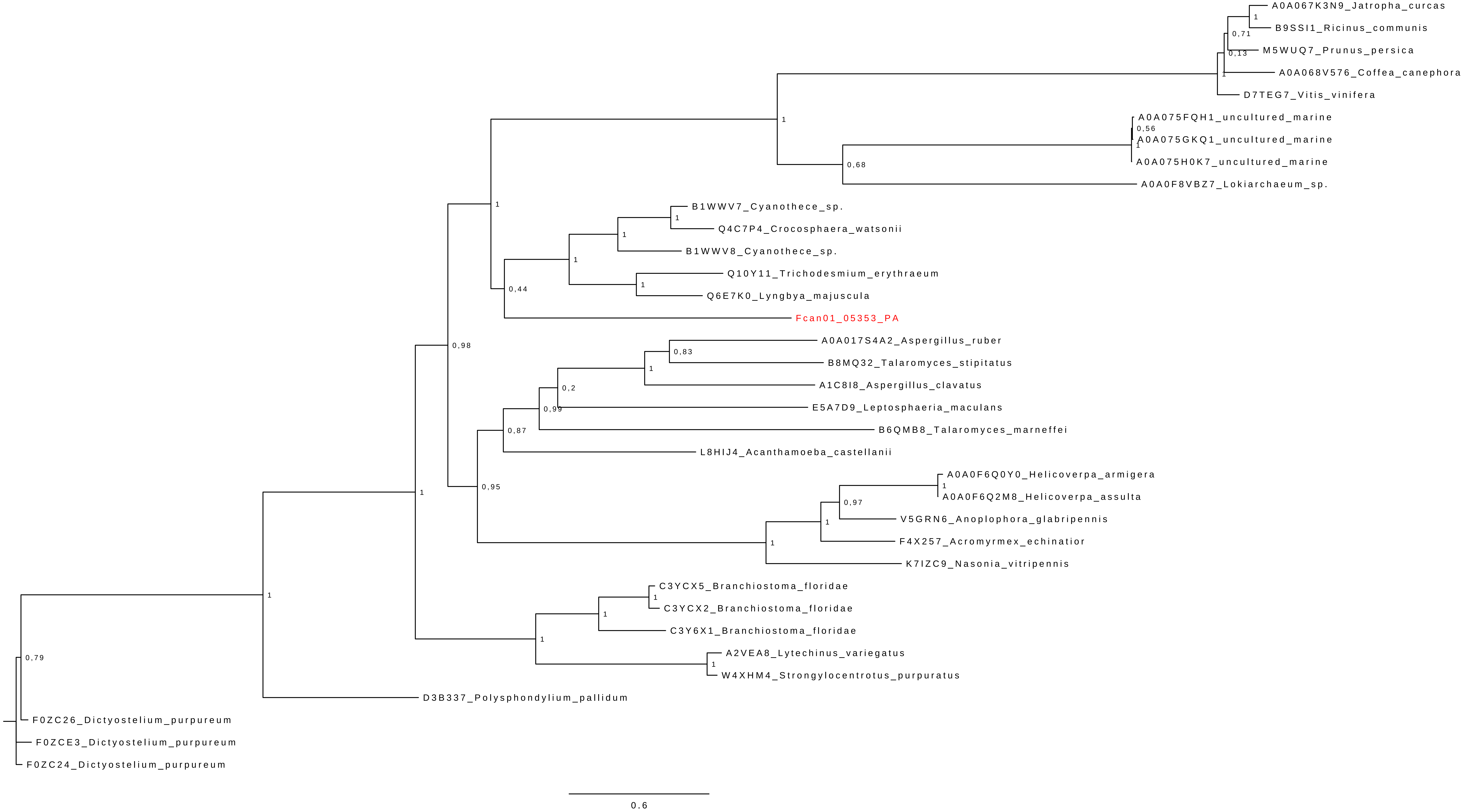
A0A067K3N9_Jatropha_curcas
B9SSI1_Ricinus_communis
M5WUQ7_Prunus_persica
A0A068V576_Coffea_canephora
D7TEG7_Vitis_vinifera
A0A075FQH1_uncultured_marine
A0A075GKQ1_uncultured_marine
A0A075H0K7_uncultured_marine
A0A0F8VBZ7_Lokiarchaeum_sp.
B1WWV7_Cyanothece_sp.
Q4C7P4_Crocosphaera_watsonii
B1WWV8_Cyanothece_sp.
Q10Y11_Trichodesmium_erythraeum
Q6E7K0_Lyngbya_majuscula
Fcan01_05353_PA
A0A017S4A2_Aspergillus_ruber
B8MQ32_Talaromyces_stipitatus
A1C8I8_Aspergillus_clavatus
E5A7D9_Leptosphaeria_maculans
B6QMB8_Talaromyces_marneffei
L8HIJ4_Acanthamoeba_castellanii
A0A0F6Q0Y0_Helicoverpa_armigera
A0A0F6Q2M8_Helicoverpa_assulta
V5GRN6_Anoplophora_glabripennis
F4X257_Acromyrmex_echinatior
K7IZC9_Nasonia_vitripennis
C3YCX5_Branchiostoma_floridae
C3YCX2_Branchiostoma_floridae
C3Y6X1_Branchiostoma_floridae
A2VEA8_Lytechinus_variegatus
W4XHM4_Strongylocentrotus_purpuratus
D3B337_Polysphondylium_pallidum
F0ZC26_Dictyostelium_purpureum
F0ZCE3_Dictyostelium_purpureum
F0ZC24_Dictyostelium_purpureum
0.6

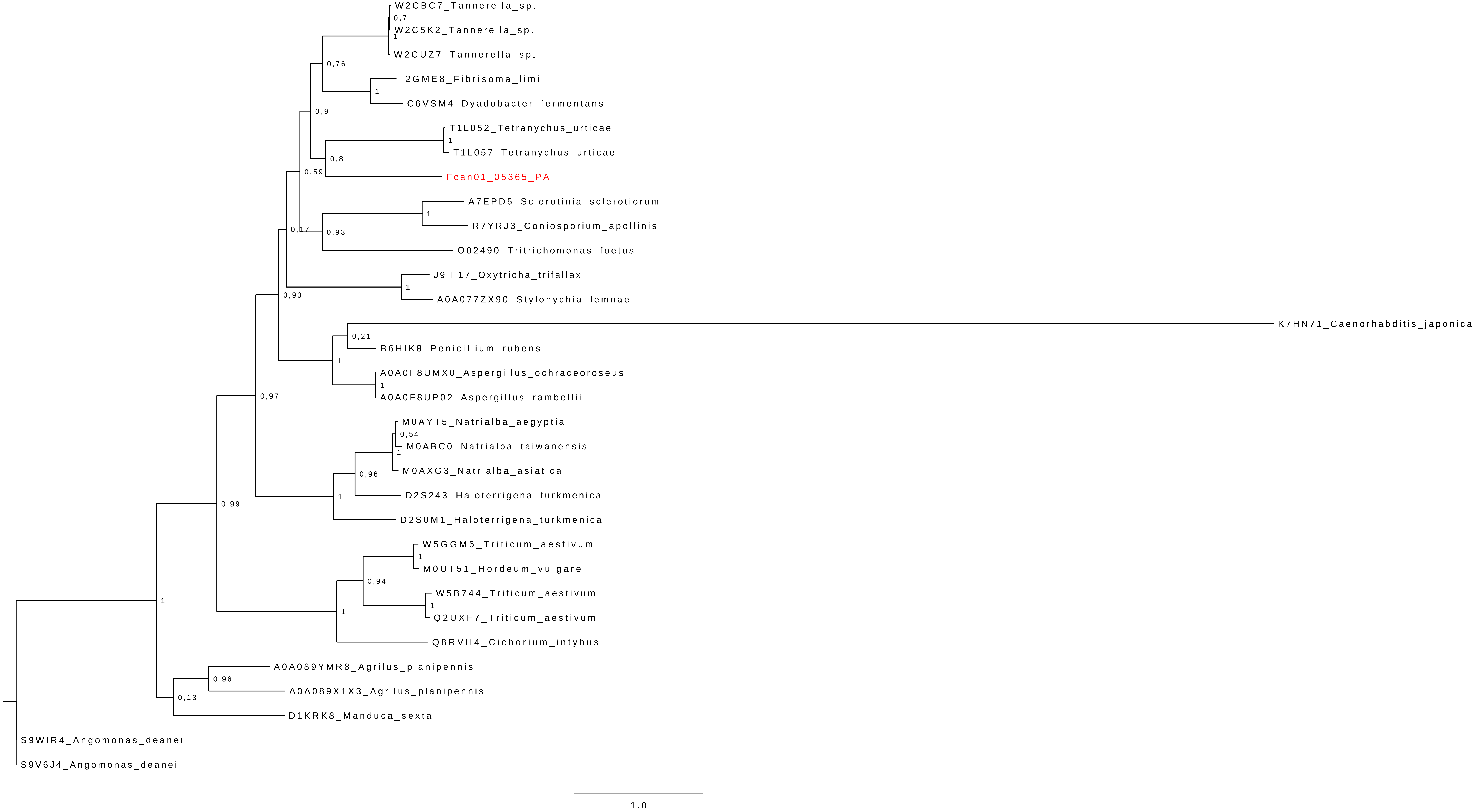
W2CBC7_Tannerella_sp.
0,7
W2C5K2_Tannerella_sp.
1
W2CUZ7_Tannerella_sp.
0,76
I2GME8_Fibrisoma_limi
1
C6VSM4_Dyadobacter_fermentans
0,9
T1L052_Tetranychus_urticae
1
T1L057_Tetranychus_urticae
0,8
0,59
Fcan01_05365_PA
A7EPD5_Sclerotinia_sclerotiorum
1
R7YRJ3_Coniosporium_apollinis
0,17
0,93
O02490_Tritrichomonas_foetus
J9IF17_Oxytricha_trifallax
1
0,93
A0A077ZX90_Stylonychia_lemnae
K7HN71_Caenorhabditis_japonica
0,21
B6HIK8_Penicillium_rubens
1
A0A0F8UMX0_Aspergillus_ochraceoroseus
1
A0A0F8UP02_Aspergillus_rambellii
0,97
M0AYT5_Natrialba_aegyptia
0,54
M0ABC0_Natrialba_taiwanensis
1
M0AXG3_Natrialba_asiatica
0,96
D2S243_Haloterrigena_turkmenica
1
D2S0M1_Haloterrigena_turkmenica
0,99
W5GGM5_Triticum_aestivum
1
M0UT51_Hordeum_vulgare
0,94
W5B744_Triticum_aestivum
1
Q2UXF7_Triticum_aestivum
1
Q8RVH4_Cichorium_intybus
1
A0A089YMR8_Agrilus_planipennis
0,96
A0A089X1X3_Agrilus_planipennis
0,13
D1KRK8_Manduca_sexta
S9WIR4_Angomonas_deanei
S9V6J4_Angomonas_deanei
1.0

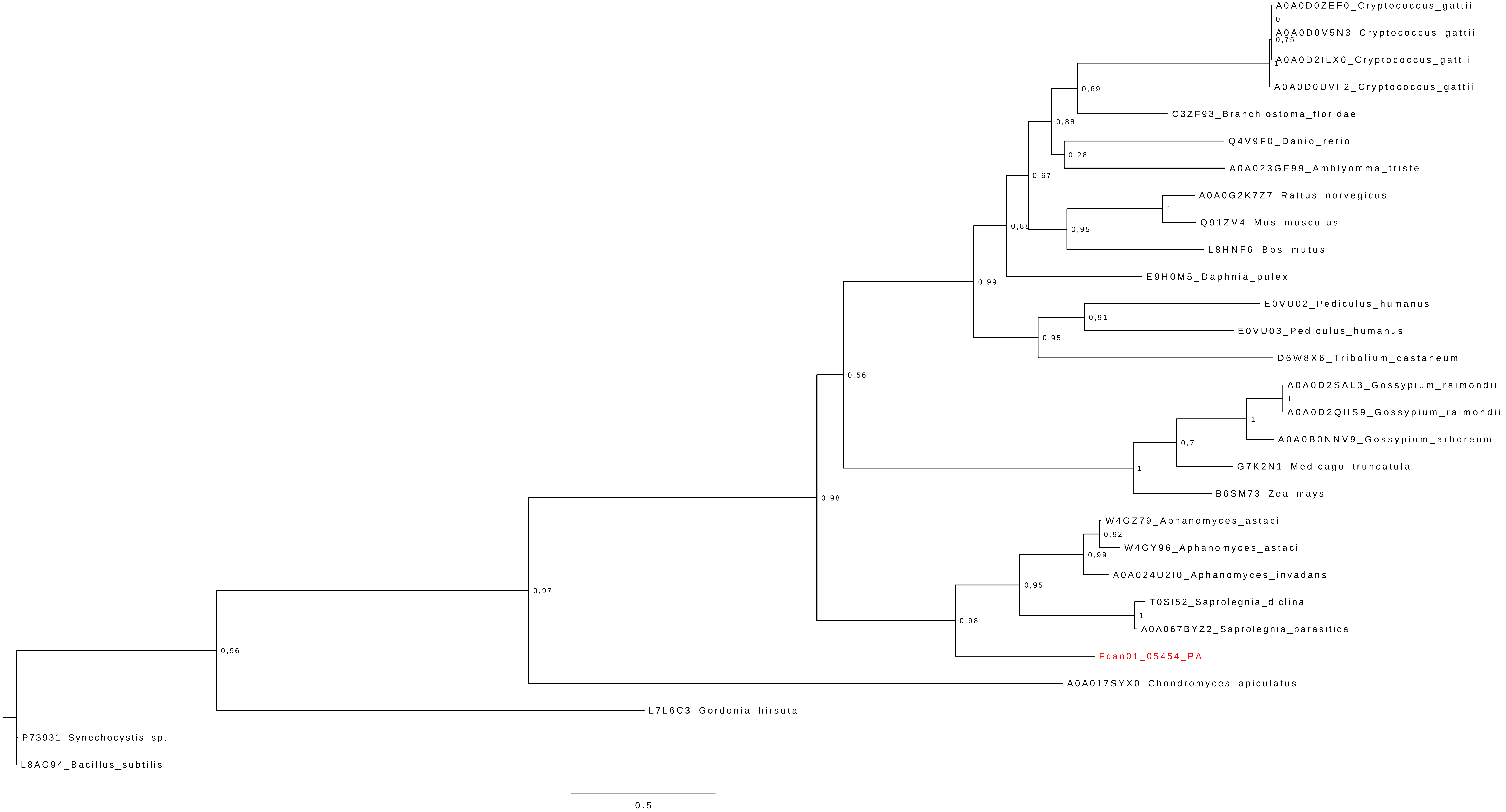
A0A0D0ZEF0_Cryptococcus_gattii
0
A0A0D0V5N3_Cryptococcus_gattii
0,75
A0A0D2ILX0_Cryptococcus_gattii
1
A0A0D0UVF2_Cryptococcus_gattii
0,69
C3ZF93_Branchiostoma_floridae
0,88
Q4V9F0_Danio_rerio
0,28
A0A023GE99_Amblyomma_triste
0,67
A0A0G2K7Z7_Rattus_norvegicus
1
Q91ZV4_Mus_musculus
0,88
0,95
L8HNF6_Bos_mutus
E9H0M5_Daphnia_pulex
0,99
E0VU02_Pediculus_humanus
0,91
E0VU03_Pediculus_humanus
0,95
D6W8X6_Tribolium_castaneum
0,56
A0A0D2SAL3_Gossypium_raimondii
1
A0A0D2QHS9_Gossypium_raimondii
1
A0A0B0NNV9_Gossypium_arboreum
0,7
G7K2N1_Medicago_truncatula
1
B6SM73_Zea_mays
0,98
W4GZ79_Aphanomyces_astaci
0,92
W4GY96_Aphanomyces_astaci
0,99
A0A024U2I0_Aphanomyces_invadans
0,97
0,95
T0SI52_Saprolegnia_diclina
1
0,98
A0A067BYZ2_Saprolegnia_parasitica
Fcan01_05454_PA
0,96
A0A017SYX0_Chondromyces_apiculatus
L7L6C3_Gordonia_hirsuta
P73931_Synechocystis_sp.
L8AG94_Bacillus_subtilis
0.5

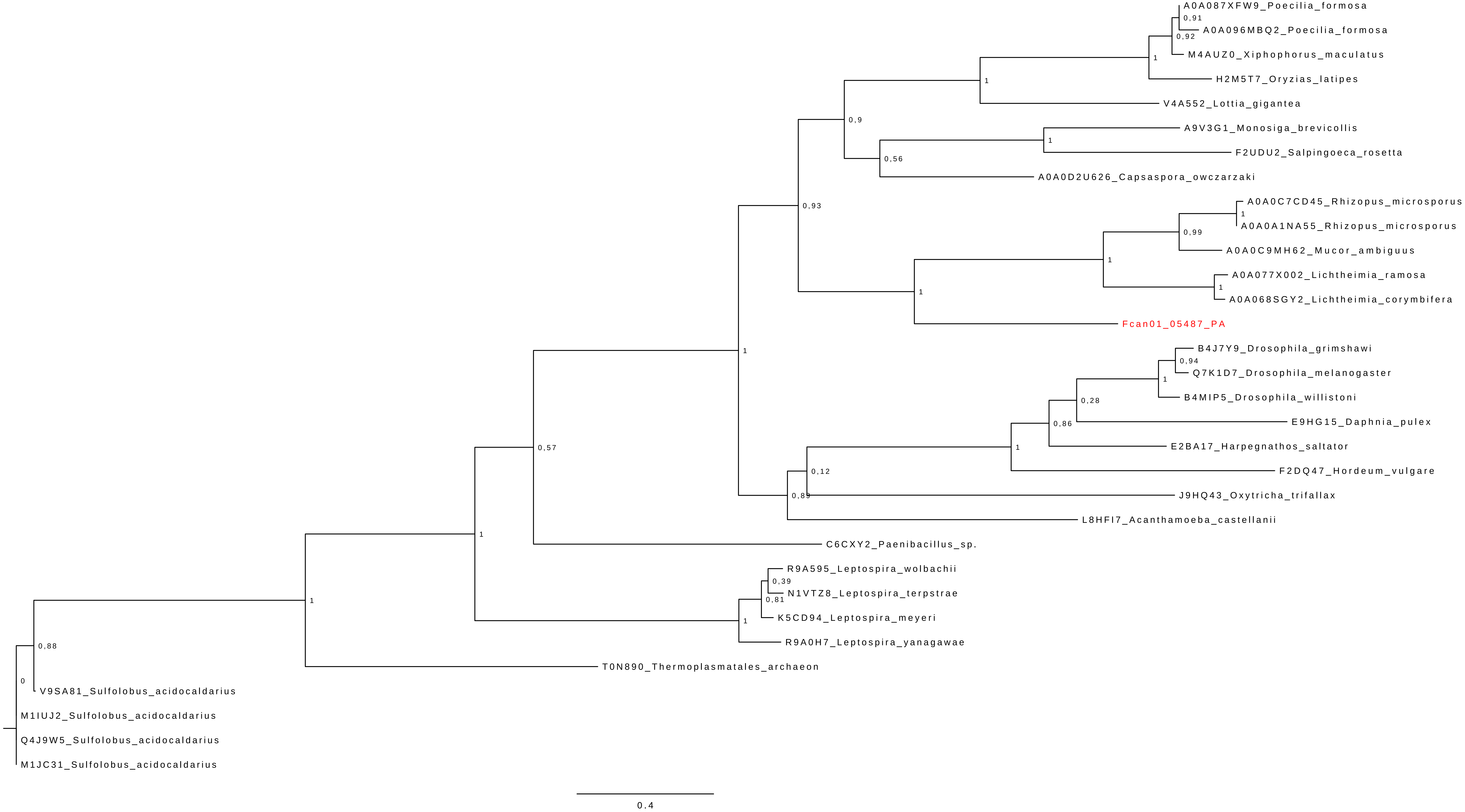
A0A087XFW9_Poecilia_formosa
0,91
A0A096MBQ2_Poecilia_formosa
0,92
M4AUZ0_Xiphophorus_maculatus
1
H2M5T7_Oryzias_latipes
1
V4A552_Lottia_gigantea
0,9
A9V3G1_Monosiga_brevicollis
1
F2UDU2_Salpingoeca_rosetta
0,56
A0A0D2U626_Capsaspora_owczarzaki
0,93
A0A0C7CD45_Rhizopus_microsporus
1
A0A0A1NA55_Rhizopus_microsporus
0,99
A0A0C9MH62_Mucor_ambiguus
1
A0A077X002_Lichtheimia_ramosa
1
A0A068SGY2_Lichtheimia_corymbifera
1
Fcan01_05487_PA
1
B4J7Y9_Drosophila_grimshawi
0,94
Q7K1D7_Drosophila_melanogaster
1
B4MIP5_Drosophila_willistoni
0,28
E9HG15_Daphnia_pulex
0,86
E2BA17_Harpegnathos_saltator
1
F2DQ47_Hordeum_vulgare
0,12
J9HQ43_Oxytricha_trifallax
0,89
L8HFI7_Acanthamoeba_castellanii
0,57
C6CXY2_Paenibacillus_sp.
1
R9A595_Leptospira_wolbachii
0,39
N1VTZ8_Leptospira_terpstrae
0,81
K5CD94_Leptospira_meyeri
1
R9A0H7_Leptospira_yanagawae
1
T0N890_Thermoplasmatales_archaeon
0,88
0
V9SA81_Sulfolobus_acidocaldarius
M1IUJ2_Sulfolobus_acidocaldarius
Q4J9W5_Sulfolobus_acidocaldarius
M1JC31_Sulfolobus_acidocaldarius
0.4

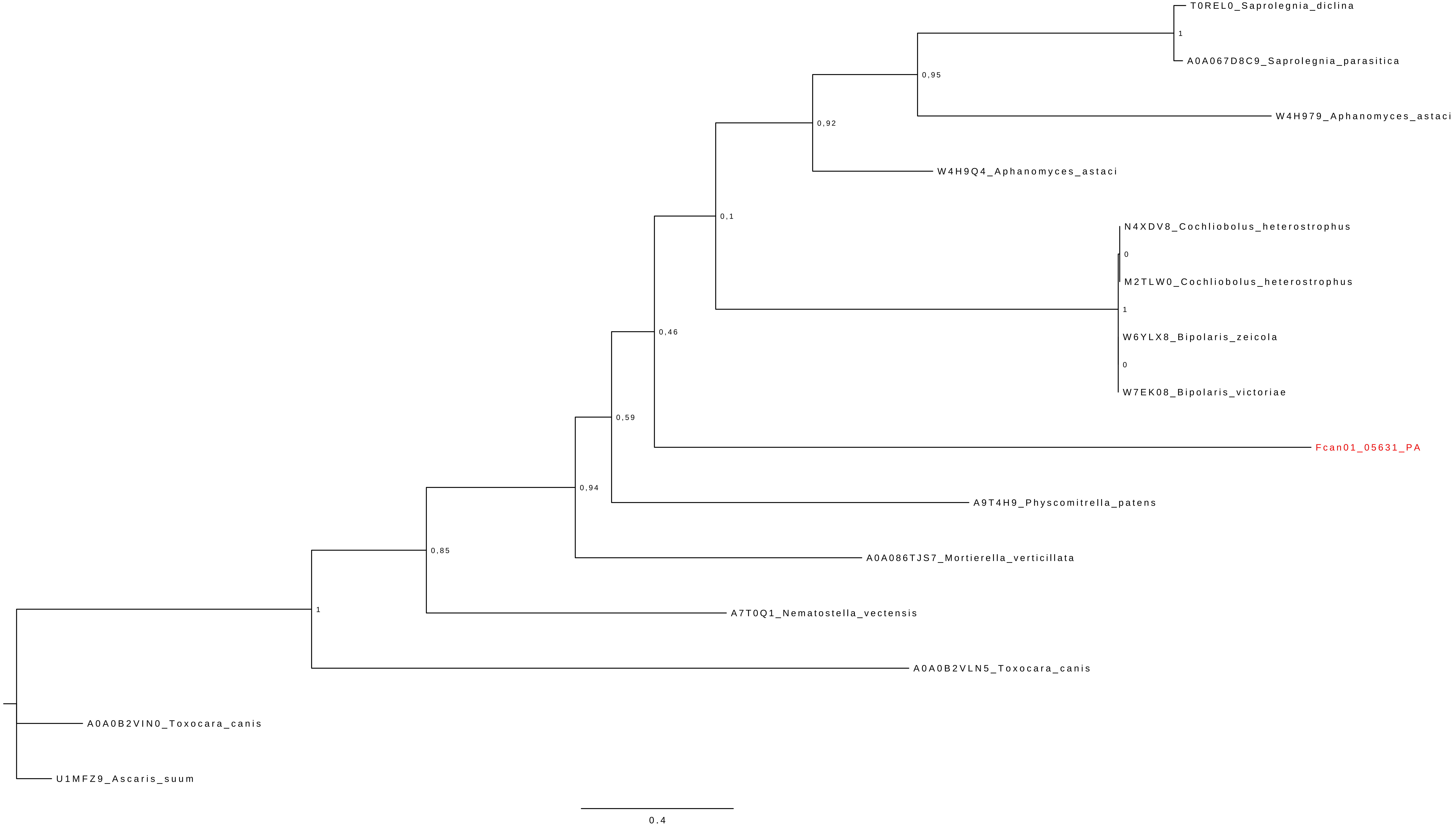
T0REL0_Saprolegnia_diclina
1
A0A067D8C9_Saprolegnia_parasitica
0,95
W4H979_Aphanomyces_astaci
0,92
W4H9Q4_Aphanomyces_astaci
0,1
N4XDV8_Cochliobolus_heterostrophus
0
M2TLW0_Cochliobolus_heterostrophus
1
W6YLX8_Bipolaris_zeicola
0
W7EK08_Bipolaris_victoriae
0,46
Fcan01_05631_PA
0,59
A9T4H9_Physcomitrella_patens
0,94
A0A086TJS7_Mortierella_verticillata
0,85
A7T0Q1_Nematostella_vectensis
1
A0A0B2VLN5_Toxocara_canis
A0A0B2VIN0_Toxocara_canis
U1MFZ9_Ascaris_suum
0.4

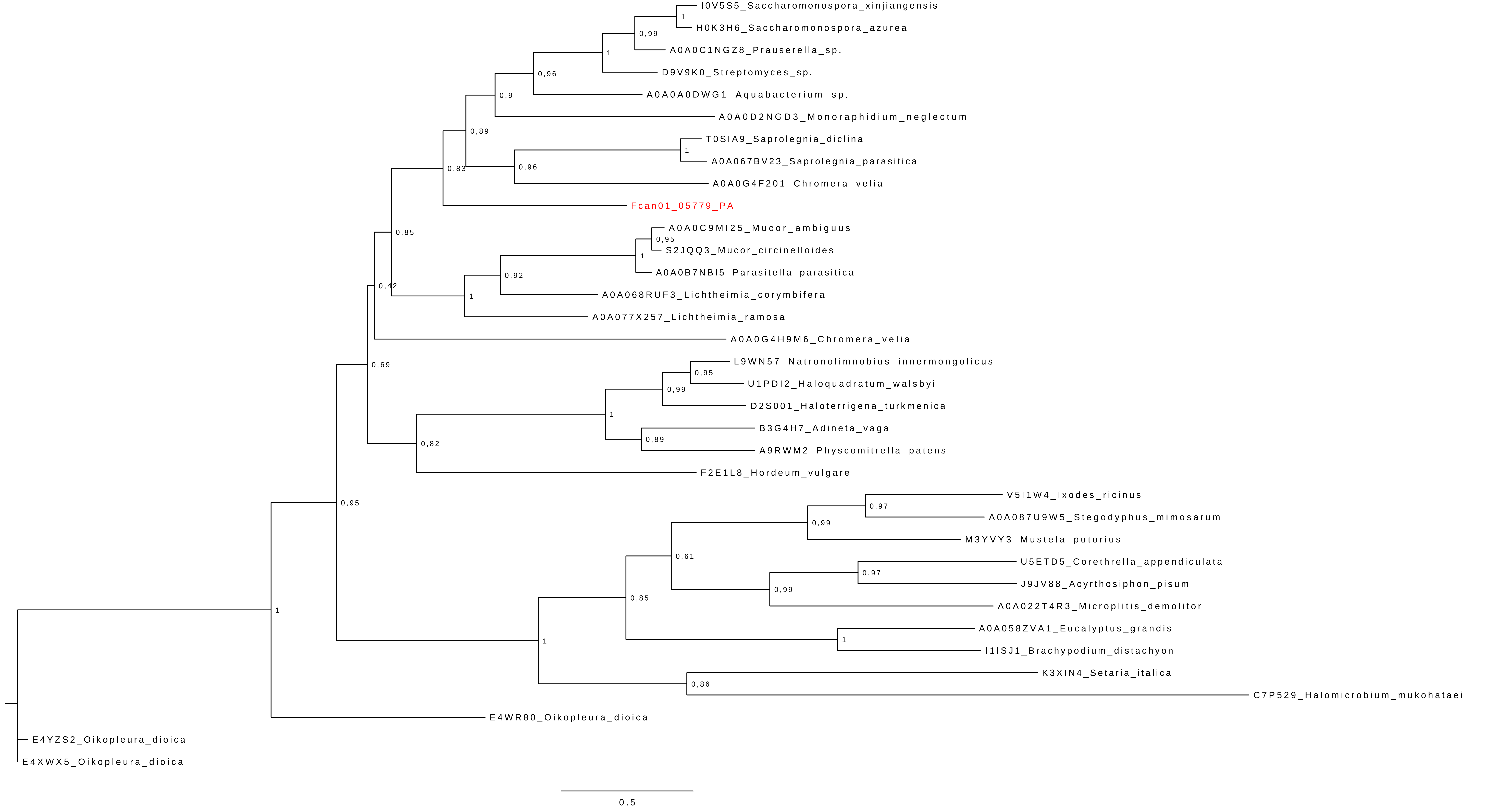
I0V5S5_Saccharomonospora_xinjiangensis
H0K3H6_Saccharomonospora_azurea
A0A0C1NGZ8_Prauserella_sp.
D9V9K0_Streptomyces_sp.
A0A0A0DWG1_Aquabacterium_sp.
A0A0D2NGD3_Monoraphidium_neglectum
T0SIA9_Saprolegnia_diclina
A0A067BV23_Saprolegnia_parasitica
A0A0G4F201_Chromera_velia
Fcan01_05779_PA
A0A0C9MI25_Mucor_ambiguus
S2JQQ3_Mucor_circinelloides
A0A0B7NBI5_Parasitella_parasitica
A0A068RUF3_Lichtheimia_corymbifera
A0A077X257_Lichtheimia_ramosa
A0A0G4H9M6_Chromera_velia
L9WN57_Natronolimnobius_innermongolicus
U1PDI2_Haloquadratum_walsbyi
D2S001_Haloterrigena_turkmenica
B3G4H7_Adineta_vaga
A9RWM2_Physcomitrella_patens
F2E1L8_Hordeum_vulgare
V5I1W4_Ixodes_ricinus
A0A087U9W5_Stegodyphus_mimosarum
M3YVY3_Mustela_putorius
U5ETD5_Corethrella_appendiculata
J9JV88_Acyrthosiphon_pisum
A0A022T4R3_Microplitis_demolitor
A0A058ZVA1_Eucalyptus_grandis
I1ISJ1_Brachypodium_distachyon
K3XIN4_Setaria_italica
C7P529_Halomicrobium_mukohataei
E4WR80_Oikopleura_dioica
E4YZS2_Oikopleura_dioica
E4XWX5_Oikopleura_dioica
1
0,99
1
0,96
0,9
0,89
1
0,96
0,83
0,85
0,95
1
0,92
1
0,42
0,69
0,95
0,99
1
0,89
0,82
0,95
0,97
0,99
0,61
0,97
0,99
0,85
1
1
0,86
1
0.5

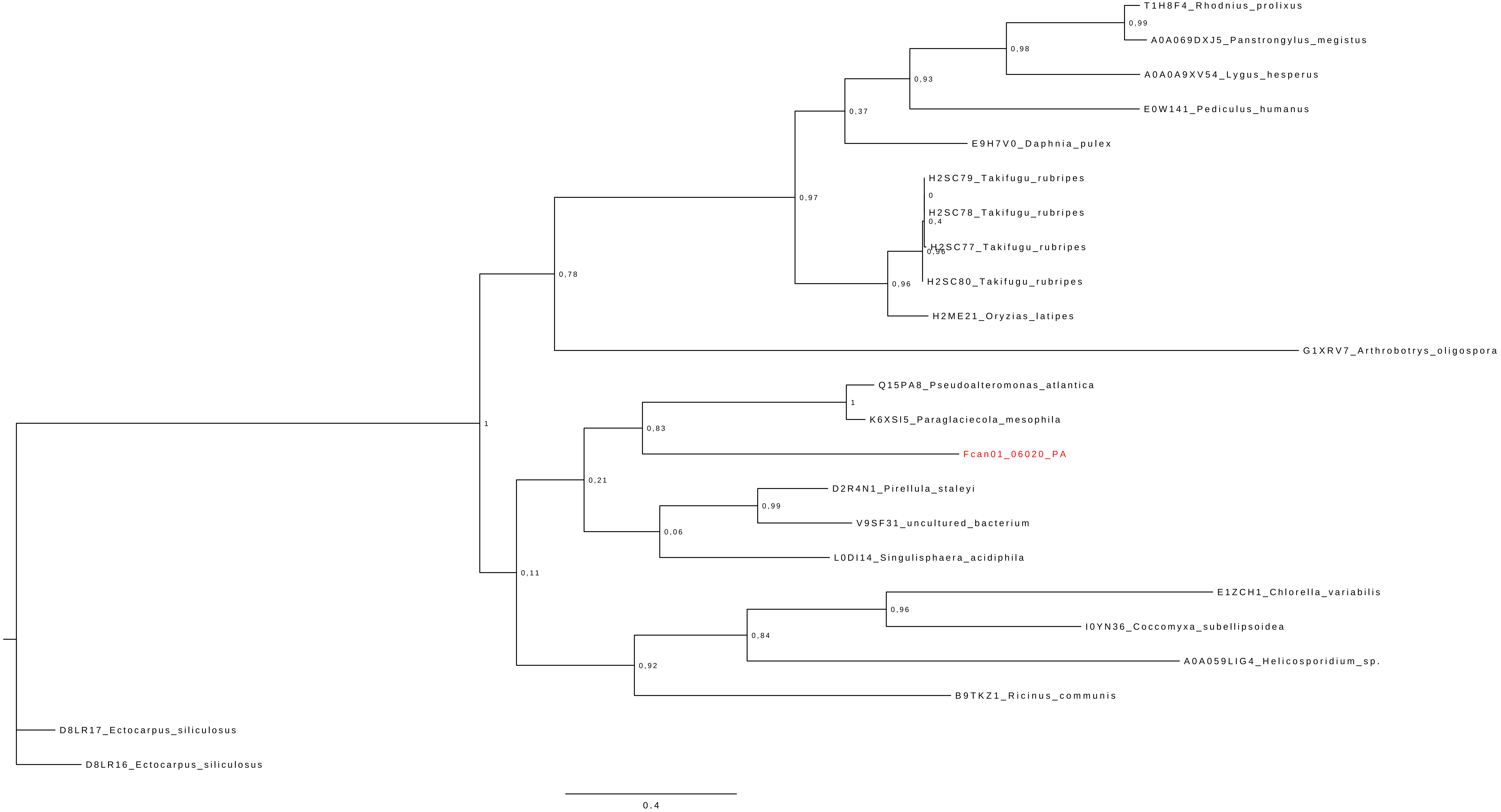
T1H8F4_Rhodnius_prolixus
0,99
A0A069DXJ5_Panstrongylus_megistus
0,98
A0A0A9XV54_Lygus_hesperus
0,93
E0W141_Pediculus_humanus
0,37
E9H7V0_Daphnia_pulex
H2SC79_Takifugu_rubripes
0
H2SC78_Takifugu_rubripes
0,4
H2SC77_Takifugu_rubripes
0,96
0,97
H2SC80_Takifugu_rubripes
0,96
H2ME21_Oryzias_latipes
0,78
G1XRV7_Arthrobotrys_oligospora
Q15PA8_Pseudoalteromonas_atlantica
1
K6XSI5_Paraglaciecola_mesophila
1
0,83
Fcan01_06020_PA
0,21
D2R4N1_Pirellula_staleyi
0,99
V9SF31_uncultured_bacterium
0,06
L0DI14_Singulisphaera_acidiphila
0,11
E1ZCH1_Chlorella_variabilis
0,96
I0YN36_Coccomyxa_subellipsoidea
0,84
A0A059LIG4_Helicosporidium_sp.
0,92
B9TKZ1_Ricinus_communis
D8LR17_Ectocarpus_siliculosus
D8LR16_Ectocarpus_siliculosus
0.4

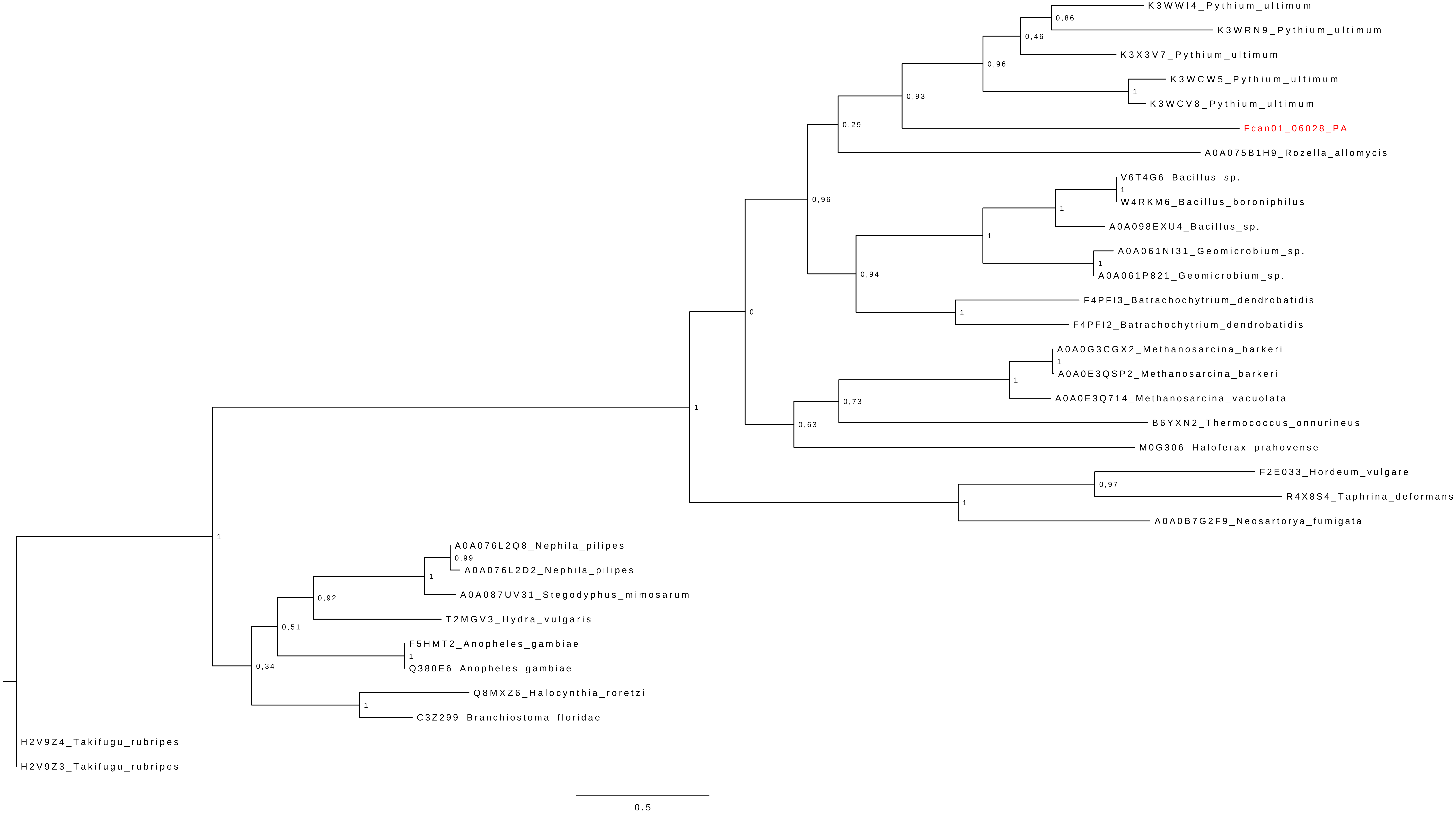
K3WWI4_Pythium_ultimum
0,86
K3WRN9_Pythium_ultimum
0,46
K3X3V7_Pythium_ultimum
0,96
K3WCW5_Pythium_ultimum
1
K3WCV8_Pythium_ultimum
0,93
Fcan01_06028_PA
0,29
A0A075B1H9_Rozella_allomycis
V6T4G6_Bacillus_sp.
1
W4RKM6_Bacillus_boroniphilus
0,96
1
A0A098EXU4_Bacillus_sp.
1
A0A061NI31_Geomicrobium_sp.
1
A0A061P821_Geomicrobium_sp.
0,94
F4PFI3_Batrachochytrium_dendrobatidis
0
1
F4PFI2_Batrachochytrium_dendrobatidis
A0A0G3CGX2_Methanosarcina_barkeri
1
A0A0E3QSP2_Methanosarcina_barkeri
1
A0A0E3Q714_Methanosarcina_vacuolata
0,73
1
B6YXN2_Thermococcus_onnurineus
0,63
M0G306_Haloferax_prahovense
F2E033_Hordeum_vulgare
0,97
R4X8S4_Taphrina_deformans
1
A0A0B7G2F9_Neosartorya_fumigata
1
A0A076L2Q8_Nephila_pilipes
0,99
A0A076L2D2_Nephila_pilipes
1
A0A087UV31_Stegodyphus_mimosarum
0,92
T2MGV3_Hydra_vulgaris
0,51
F5HMT2_Anopheles_gambiae
1
Q380E6_Anopheles_gambiae
0,34
Q8MXZ6_Halocynthia_roretzi
1
C3Z299_Branchiostoma_floridae
H2V9Z4_Takifugu_rubripes
H2V9Z3_Takifugu_rubripes
0.5

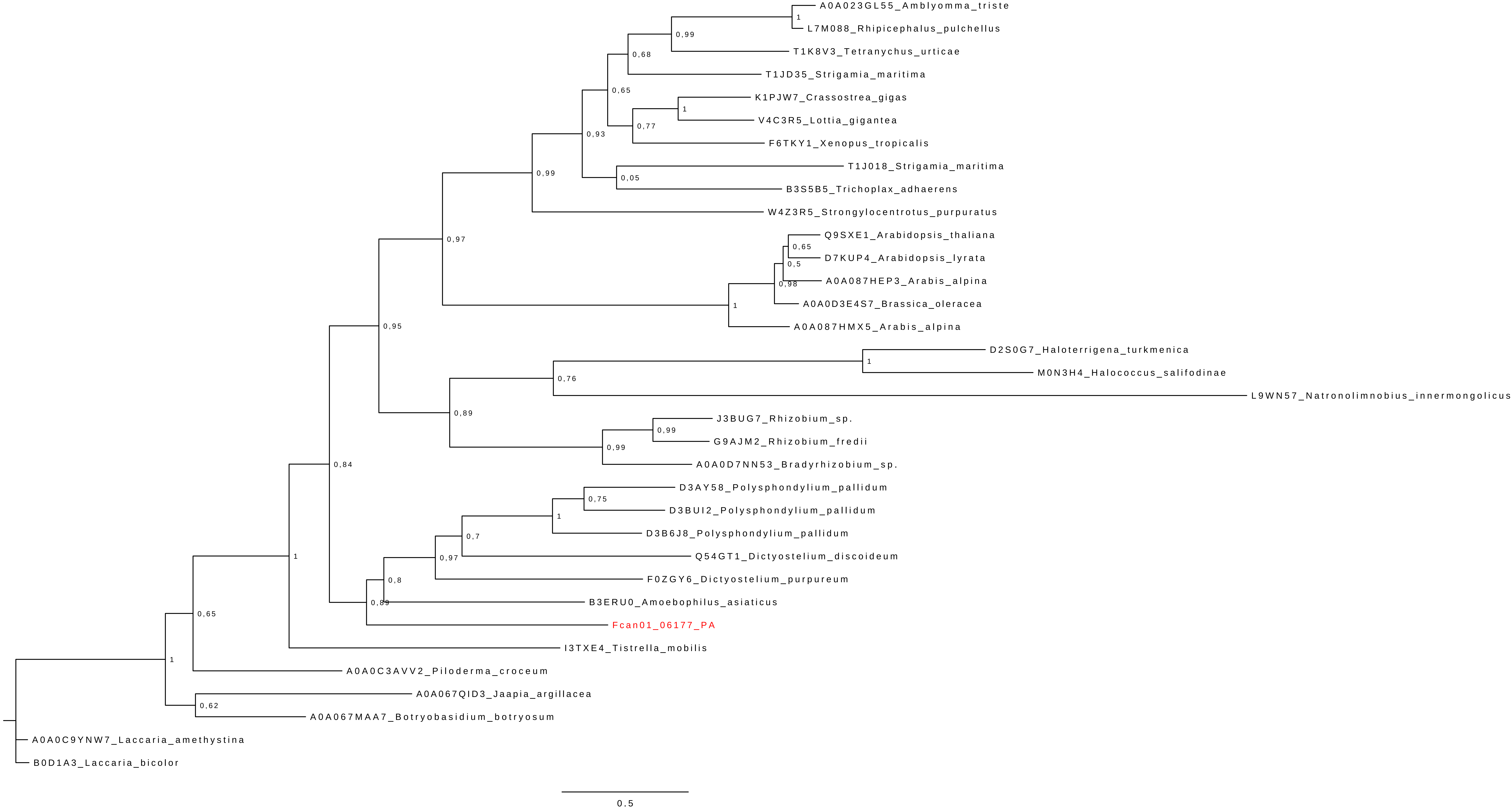
A0A023GL55_Amblyomma_triste
L7M088_Rhipicephalus_pulchellus
T1K8V3_Tetranychus_urticae
T1JD35_Strigamia_maritima
K1PJW7_Crassostrea_gigas
V4C3R5_Lottia_gigantea
F6TKY1_Xenopus_tropicalis
T1J018_Strigamia_maritima
B3S5B5_Trichoplax_adhaerens
W4Z3R5_Strongylocentrotus_purpuratus
Q9SXE1_Arabidopsis_thaliana
D7KUP4_Arabidopsis_lyrata
A0A087HEP3_Arabis_alpina
A0A0D3E4S7_Brassica_oleracea
A0A087HMX5_Arabis_alpina
D2S0G7_Haloterrigena_turkmenica
M0N3H4_Halococcus_salifodinae
L9WN57_Natronolimnobius_innermongolicus
J3BUG7_Rhizobium_sp.
G9AJM2_Rhizobium_fredii
A0A0D7NN53_Bradyrhizobium_sp.
D3AY58_Polysphondylium_pallidum
D3BUI2_Polysphondylium_pallidum
D3B6J8_Polysphondylium_pallidum
Q54GT1_Dictyostelium_discoideum
F0ZGY6_Dictyostelium_purpureum
B3ERU0_Amoebophilus_asiaticus
Fcan01_06177_PA
I3TXE4_Tistrella_mobilis
A0A0C3AVV2_Piloderma_croceum
A0A067QID3_Jaapia_argillacea
A0A067MAA7_Botryobasidium_botryosum
A0A0C9YNW7_Laccaria_amethystina
B0D1A3_Laccaria_bicolor
1
0,99
0,68
0,65
1
0,77
0,93
0,05
0,99
0,97
0,65
0,5
0,98
1
0,95
1
0,76
0,89
0,99
0,99
0,84
0,75
1
0,7
0,97
0,8
0,89
1
0,65
1
0,62
0.5

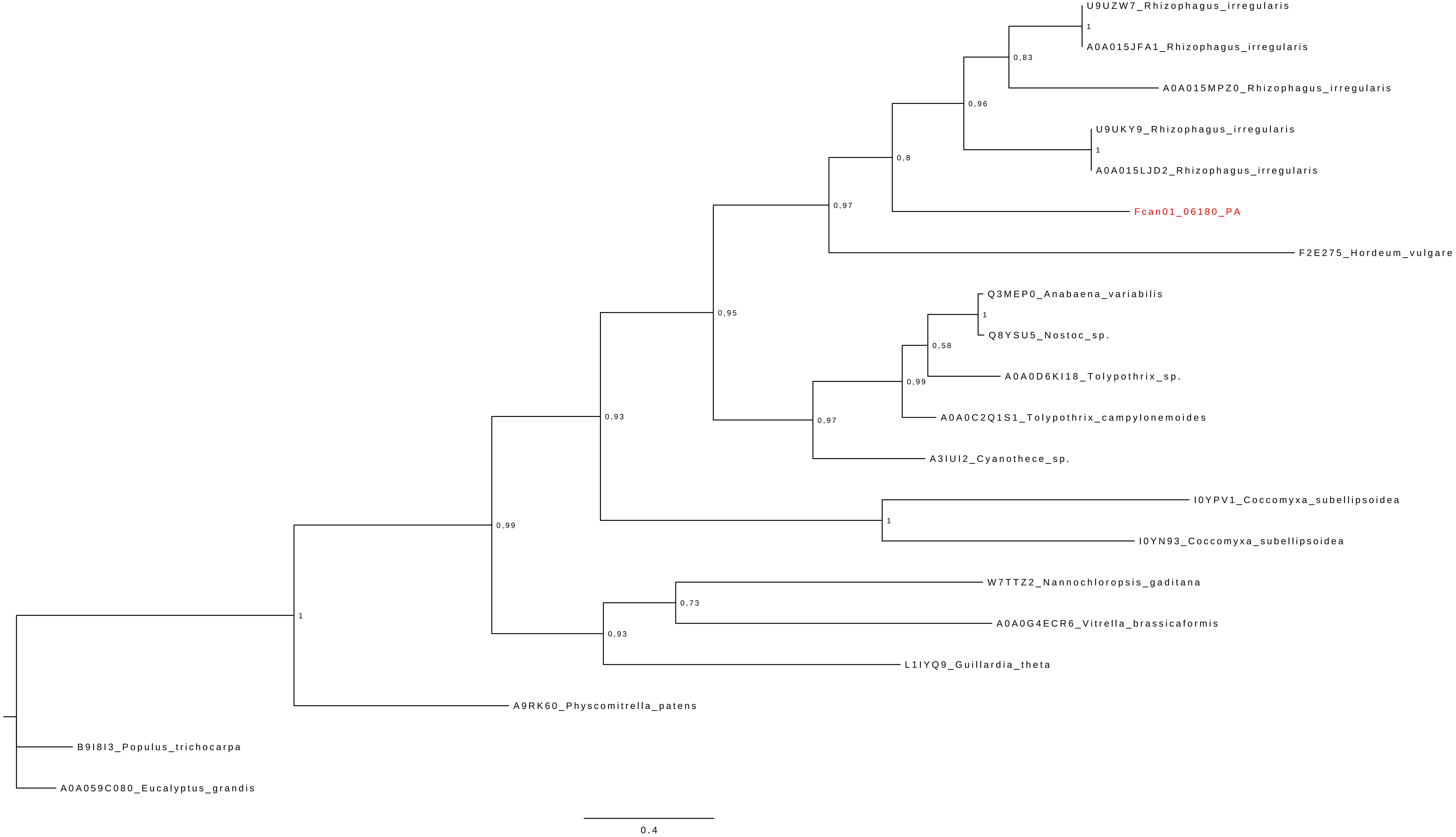

U9UZW7_Rhizophagus_irregularis
1
A0A015JFA1_Rhizophagus_irregularis
0,83
A0A015MPZ0_Rhizophagus_irregularis
0,96
U9UKY9_Rhizophagus_irregularis
1
A0A015LJD2_Rhizophagus_irregularis
0,8
Fcan01_06180_PA
0,97
F2E275_Hordeum_vulgare
Q3MEP0_Anabaena_variabilis
0,95
1
Q8YSU5_Nostoc_sp.
0,58
A0A0D6KI18_Tolypothrix_sp.
0,99
0,93
A0A0C2Q1S1_Tolypothrix_campylonemoides
0,97
A3IUI2_Cyanothece_sp.
I0YPV1_Coccomyxa_subellipsoidea
1
0,99
I0YN93_Coccomyxa_subellipsoidea
W7TTZ2_Nannochloropsis_gaditana
0,73
1
A0A0G4ECR6_Vitrella_brassicaformis
0,93
L1IYQ9_Guillardia_theta
A9RK60_Physcomitrella_patens
B9I8I3_Populus_trichocarpa
A0A059C080_Eucalyptus_grandis
0.4

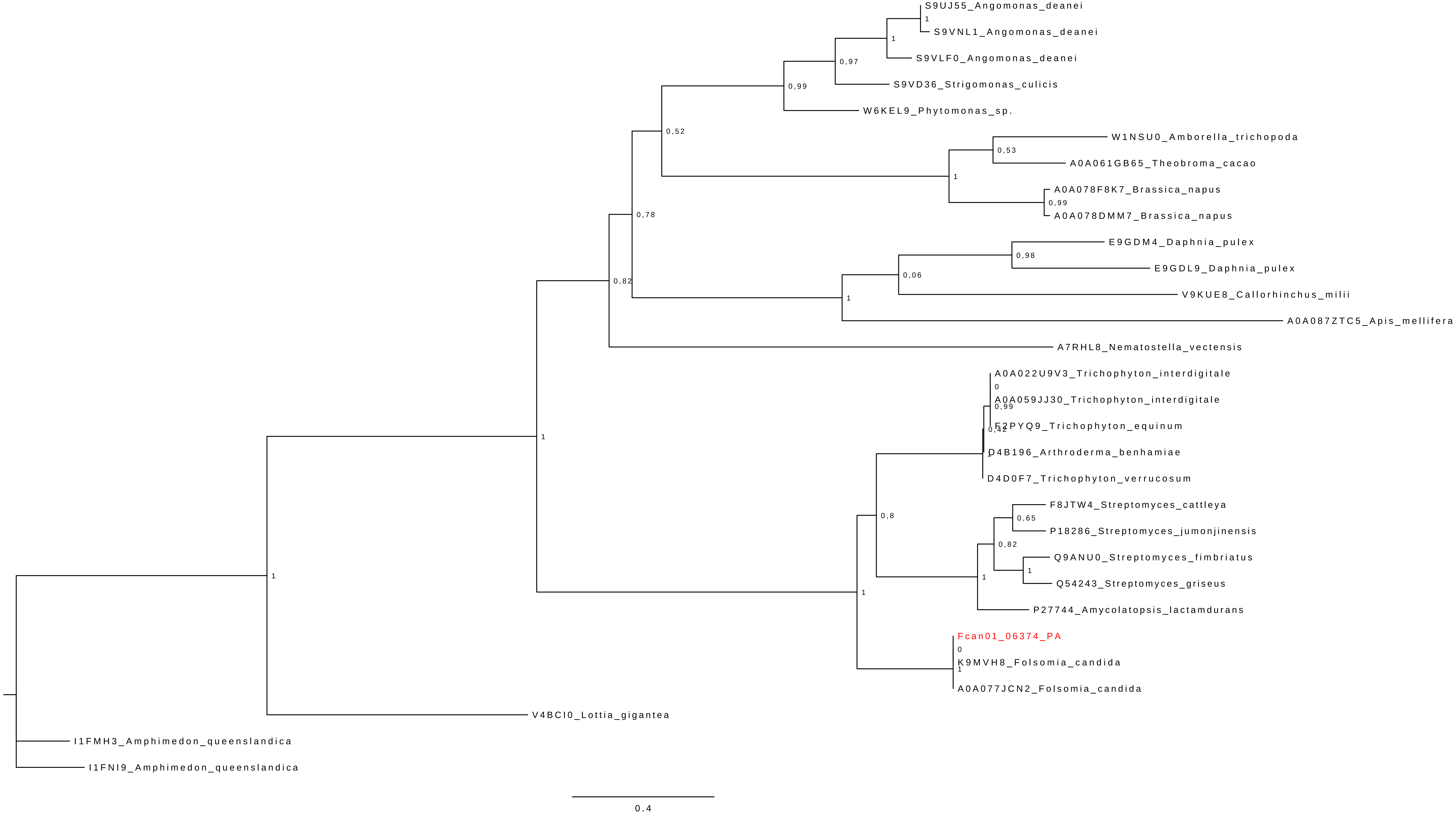
S9UJ55_Angomonas_deanei
S9VNL1_Angomonas_deanei
S9VLF0_Angomonas_deanei
S9VD36_Strigomonas_culicis
W6KEL9_Phytomonas_sp.
W1NSU0_Amborella_trichopoda
A0A061GB65_Theobroma_cacao
A0A078F8K7_Brassica_napus
A0A078DMM7_Brassica_napus
E9GDM4_Daphnia_pulex
E9GDL9_Daphnia_pulex
V9KUE8_Callorhinchus_milii
A0A087ZTC5_Apis_mellifera
A7RHL8_Nematostella_vectensis
A0A022U9V3_Trichophyton_interdigitale
A0A059JJ30_Trichophyton_interdigitale
F2PYQ9_Trichophyton_equinum
D4B196_Arthroderma_benhamiae
D4D0F7_Trichophyton_verrucosum
F8JTW4_Streptomyces_cattleya
P18286_Streptomyces_jumonjinensis
Q9ANU0_Streptomyces_fimbriatus
Q54243_Streptomyces_griseus
P27744_Amycolatopsis_lactamdurans
Fcan01_06374_PA
K9MVH8_Folsomia_candida
A0A077JCN2_Folsomia_candida
V4BCI0_Lottia_gigantea
I1FMH3_Amphimedon_queenslandica
I1FNI9_Amphimedon_queenslandica
0.4

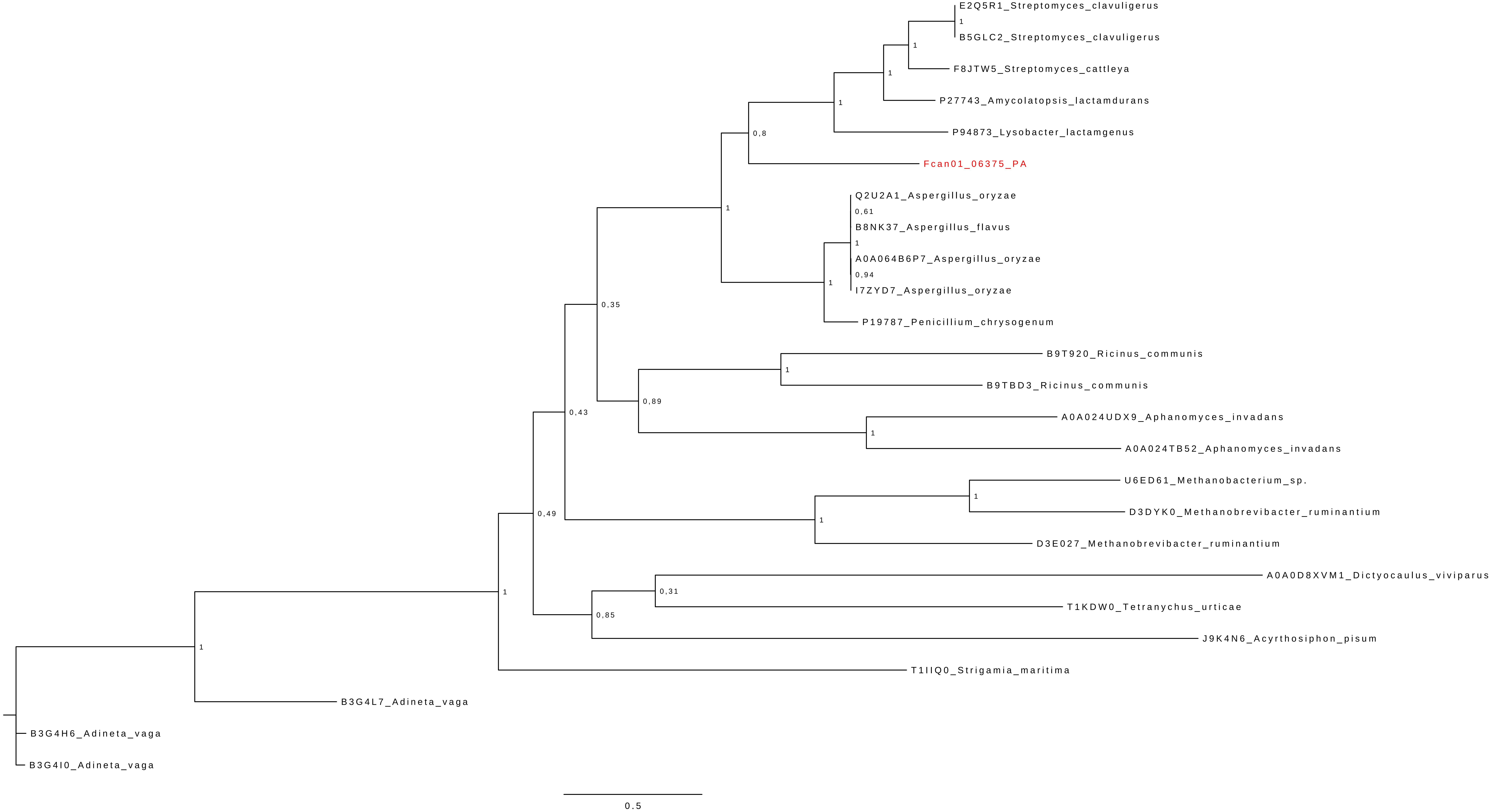

E2Q5R1_Streptomyces_clavuligerus
1
B5GLC2_Streptomyces_clavuligerus
1
F8JTW5_Streptomyces_cattleya
1
P27743_Amycolatopsis_lactamdurans
1
P94873_Lysobacter_lactamgenus
0,8
Fcan01_06375_PA
Q2U2A1_Aspergillus_oryzae
0,61
1
B8NK37_Aspergillus_flavus
1
A0A064B6P7_Aspergillus_oryzae
0,94
I7ZYD7_Aspergillus_oryzae
1
P19787_Penicillium_chrysogenum
0,35
B9T920_Ricinus_communis
1
B9TBD3_Ricinus_communis
0,89
A0A024UDX9_Aphanomyces_invadans
1
A0A024TB52_Aphanomyces_invadans
0,43
U6ED61_Methanobacterium_sp.
1
D3DYK0_Methanobrevibacter_ruminantium
0,49
1
D3E027_Methanobrevibacter_ruminantium
A0A0D8XVM1_Dictyocaulus_viviparus
0,31
1
T1KDW0_Tetranychus_urticae
0,85
J9K4N6_Acyrthosiphon_pisum
T1IIQ0_Strigamia_maritima
1
B3G4L7_Adineta_vaga
B3G4H6_Adineta_vaga
B3G4I0_Adineta_vaga
0.5

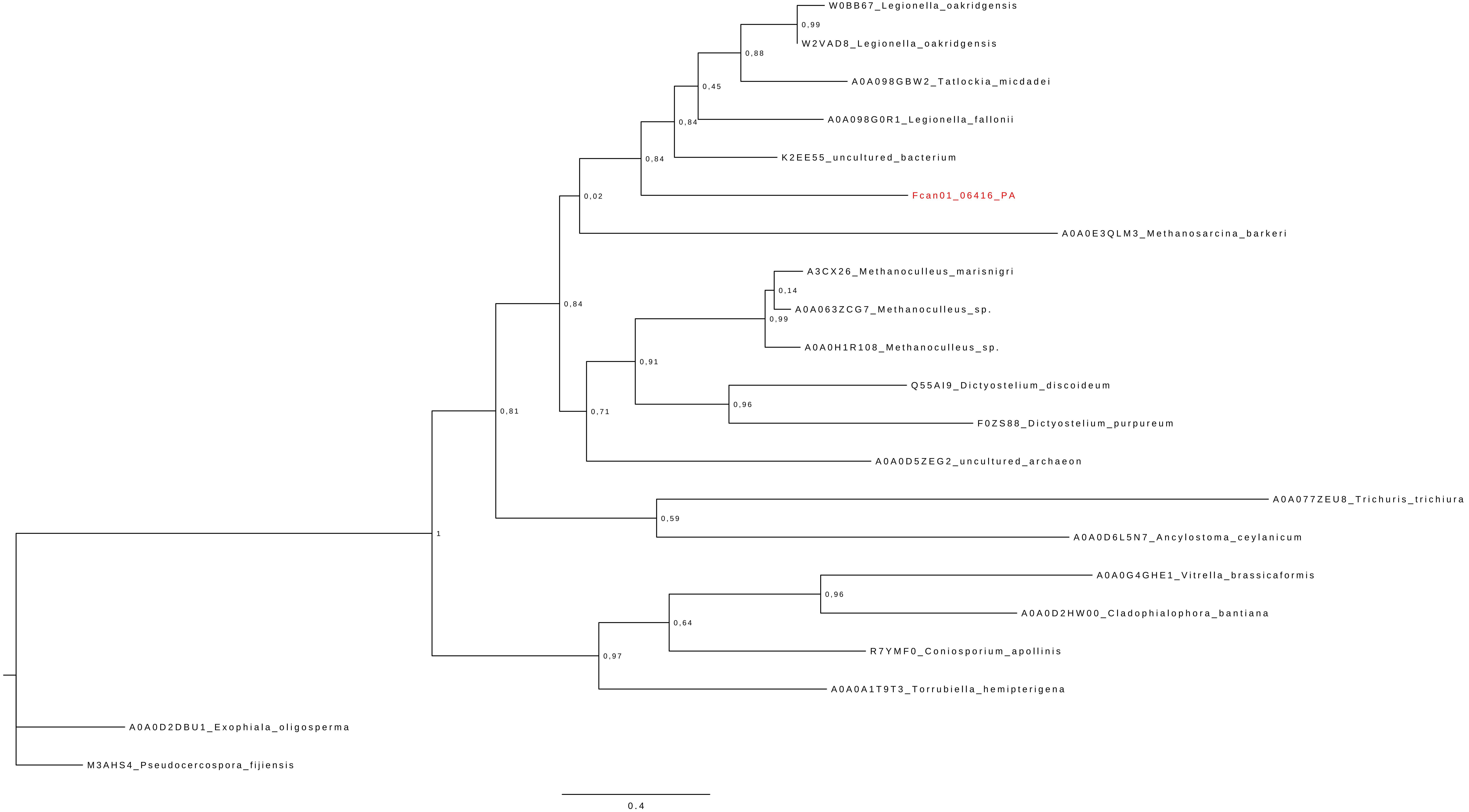
W0BB67_Legionella_oakridgensis
0,99
W2VAD8_Legionella_oakridgensis
0,88
A0A098GBW2_Tatlockia_micdadei
0,45
A0A098G0R1_Legionella_fallonii
0,84
K2EE55_uncultured_bacterium
0,84
Fcan01_06416_PA
0,02
A0A0E3QLM3_Methanosarcina_barkeri
A3CX26_Methanoculleus_marisnigri
0,14
A0A063ZCG7_Methanoculleus_sp.
0,99
A0A0H1R108_Methanoculleus_sp.
0,84
0,91
Q55AI9_Dictyostelium_discoideum
0,96
F0ZS88_Dictyostelium_purpureum
0,81
0,71
A0A0D5ZEG2_uncultured_archaeon
A0A077ZEU8_Trichuris_trichiura
0,59
A0A0D6L5N7_Ancylostoma_ceylanicum
1
A0A0G4GHE1_Vitrella_brassicaformis
0,96
A0A0D2HW00_Cladophialophora_bantiana
0,64
R7YMF0_Coniosporium_apollinis
0,97
A0A0A1T9T3_Torrubiella_hemipterigena
A0A0D2DBU1_Exophiala_oligosperma
M3AHS4_Pseudocercospora_fijiensis
0.4

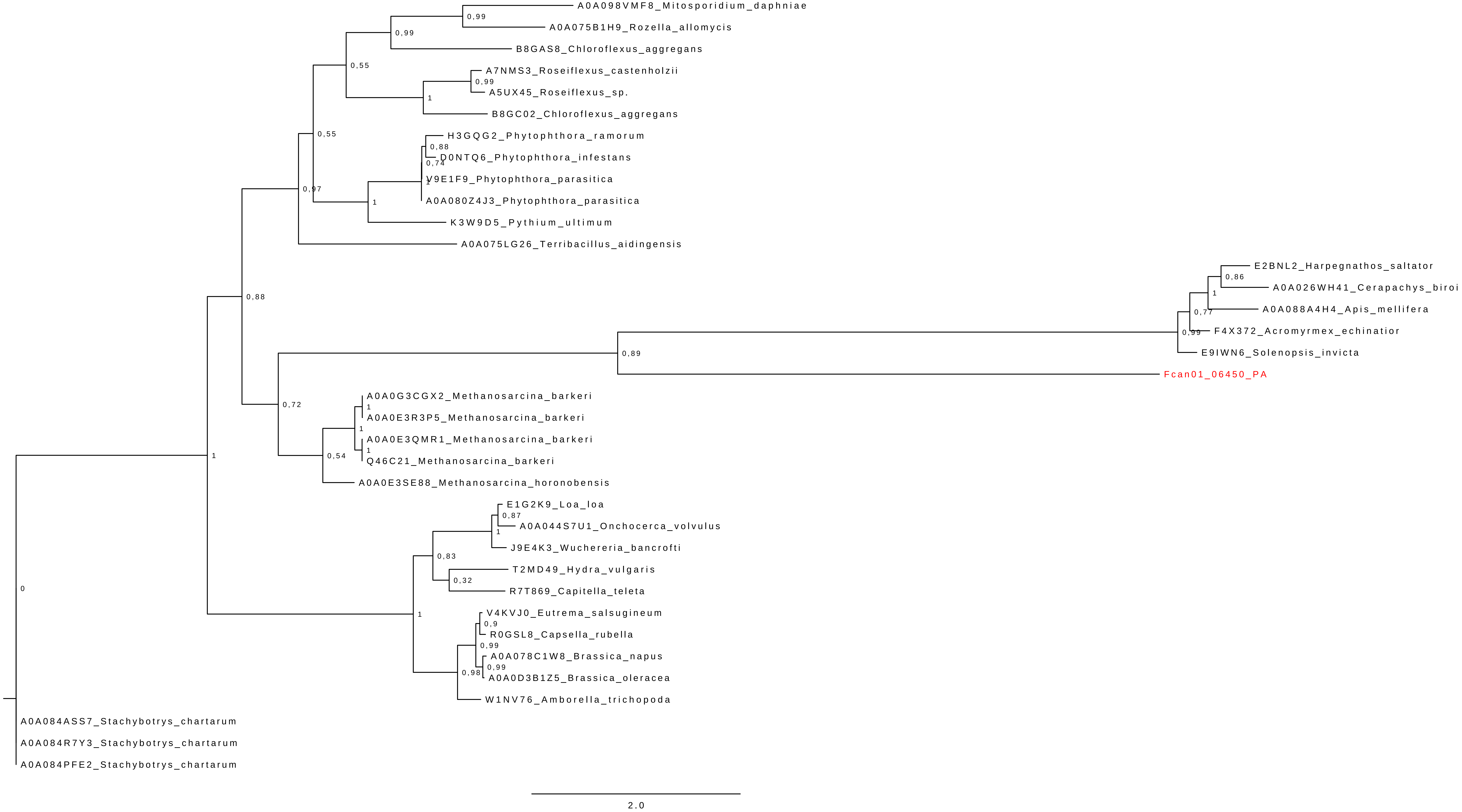
A0A098VMF8_Mitosporidium_daphniae
0,99
A0A075B1H9_Rozella_allomycis
0,99
B8GAS8_Chloroflexus_aggregans
0,55
A7NMS3_Roseiflexus_castenholzii
0,99
A5UX45_Roseiflexus_sp.
1
B8GC02_Chloroflexus_aggregans
0,55
H3GQG2_Phytophthora_ramorum
0,88
D0NTQ6_Phytophthora_infestans
0,74
V9E1F9_Phytophthora_parasitica
1
A0A080Z4J3_Phytophthora_parasitica
0,97
1
K3W9D5_Pythium_ultimum
A0A075LG26_Terribacillus_aidingensis
E2BNL2_Harpegnathos_saltator
0,86
A0A026WH41_Cerapachys_biroi
1
A0A088A4H4_Apis_mellifera
0,77
F4X372_Acromyrmex_echinatior
0,99
0,88
E9IWN6_Solenopsis_invicta
0,89
Fcan01_06450_PA
A0A0G3CGX2_Methanosarcina_barkeri
1
0,72
A0A0E3R3P5_Methanosarcina_barkeri
1
A0A0E3QMR1_Methanosarcina_barkeri
1
1
0,54
Q46C21_Methanosarcina_barkeri
A0A0E3SE88_Methanosarcina_horonobensis
E1G2K9_Loa_loa
0,87
A0A044S7U1_Onchocerca_volvulus
1
J9E4K3_Wuchereria_bancrofti
0,83
T2MD49_Hydra_vulgaris
0,32
0
R7T869_Capitella_teleta
V4KVJ0_Eutrema_salsugineum
1
0,9
R0GSL8_Capsella_rubella
0,99
A0A078C1W8_Brassica_napus
0,99
0,98
A0A0D3B1Z5_Brassica_oleracea
W1NV76_Amborella_trichopoda
A0A084ASS7_Stachybotrys_chartarum
A0A084R7Y3_Stachybotrys_chartarum
A0A084PFE2_Stachybotrys_chartarum
2.0

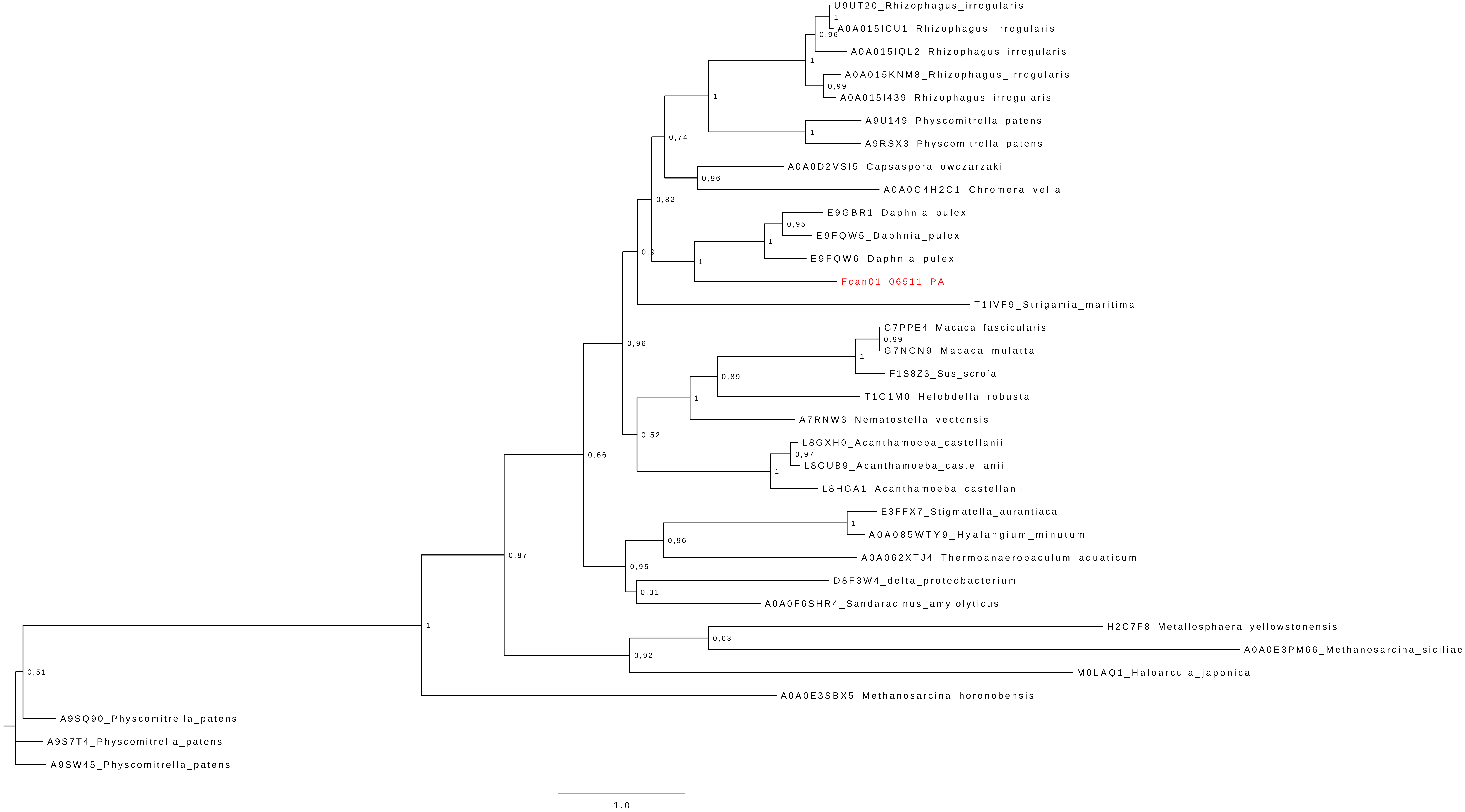
U9UT20_Rhizophagus_irregularis
A0A015ICU1_Rhizophagus_irregularis
A0A015IQL2_Rhizophagus_irregularis
A0A015KNM8_Rhizophagus_irregularis
A0A015I439_Rhizophagus_irregularis
A9U149_Physcomitrella_patens
A9RSX3_Physcomitrella_patens
A0A0D2VSI5_Capsaspora_owczarzaki
A0A0G4H2C1_Chromera_velia
E9GBR1_Daphnia_pulex
E9FQW5_Daphnia_pulex
E9FQW6_Daphnia_pulex
Fcan01_06511_PA
T1IVF9_Strigamia_maritima
G7PPE4_Macaca_fascicularis
G7NCN9_Macaca_mulatta
F1S8Z3_Sus_scrofa
T1G1M0_Helobdella_robusta
A7RNW3_Nematostella_vectensis
L8GXH0_Acanthamoeba_castellanii
L8GUB9_Acanthamoeba_castellanii
L8HGA1_Acanthamoeba_castellanii
E3FFX7_Stigmatella_aurantiaca
A0A085WTY9_Hyalangium_minutum
A0A062XTJ4_Thermoanaerobaculum_aquaticum
D8F3W4_delta_proteobacterium
A0A0F6SHR4_Sandaracinus_amylolyticus
H2C7F8_Metallosphaera_yellowstonensis
A0A0E3PM66_Methanosarcina_siciliae
M0LAQ1_Haloarcula_japonica
A0A0E3SBX5_Methanosarcina_horonobensis
A9SQ90_Physcomitrella_patens
A9S7T4_Physcomitrella_patens
A9SW45_Physcomitrella_patens
1.0

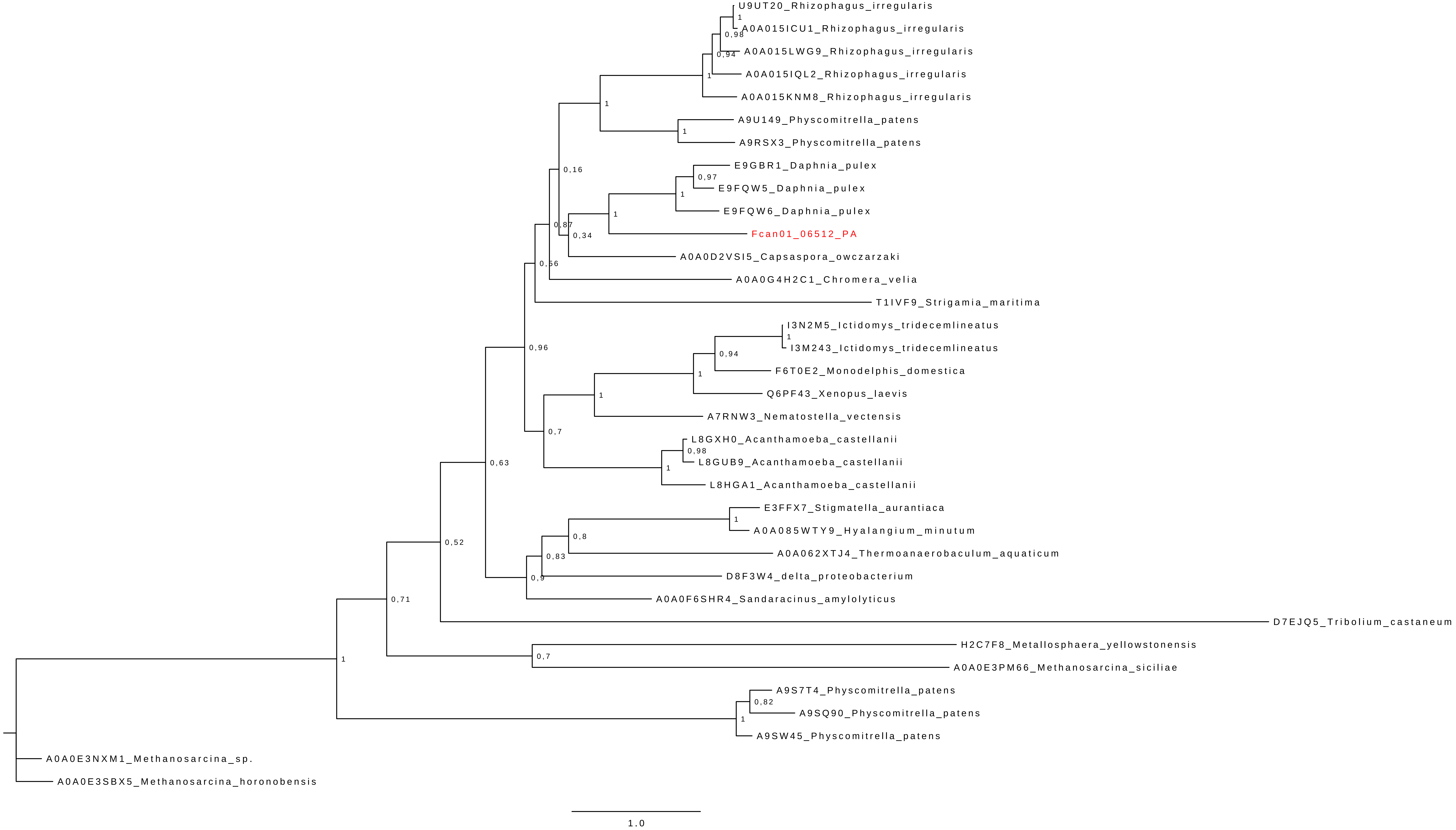
U9UT20_Rhizophagus_irregularis
A0A015ICU1_Rhizophagus_irregularis
A0A015LWG9_Rhizophagus_irregularis
A0A015IQL2_Rhizophagus_irregularis
A0A015KNM8_Rhizophagus_irregularis
A9U149_Physcomitrella_patens
A9RSX3_Physcomitrella_patens
E9GBR1_Daphnia_pulex
E9FQW5_Daphnia_pulex
E9FQW6_Daphnia_pulex
Fcan01_06512_PA
A0A0D2VSI5_Capsaspora_owczarzaki
A0A0G4H2C1_Chromera_velia
T1IVF9_Strigamia_maritima
I3N2M5_Ictidomys_tridecemlineatus
I3M243_Ictidomys_tridecemlineatus
F6T0E2_Monodelphis_domestica
Q6PF43_Xenopus_laevis
A7RNW3_Nematostella_vectensis
L8GXH0_Acanthamoeba_castellanii
L8GUB9_Acanthamoeba_castellanii
L8HGA1_Acanthamoeba_castellanii
E3FFX7_Stigmatella_aurantiaca
A0A085WTY9_Hyalangium_minutum
A0A062XTJ4_Thermoanaerobaculum_aquaticum
D8F3W4_delta_proteobacterium
A0A0F6SHR4_Sandaracinus_amylolyticus
D7EJQ5_Tribolium_castaneum
H2C7F8_Metallosphaera_yellowstonensis
A0A0E3PM66_Methanosarcina_siciliae
A9S7T4_Physcomitrella_patens
A9SQ90_Physcomitrella_patens
A9SW45_Physcomitrella_patens
A0A0E3NXM1_Methanosarcina_sp.
A0A0E3SBX5_Methanosarcina_horonobensis
1
0,98
0,94
1
1
1
0,16
0,97
1
1
0,87
0,34
0,56
0,96
1
0,94
1
1
0,7
0,98
1
0,63
1
0,8
0,83
0,9
0,52
0,71
0,7
1
0,82
1
1.0

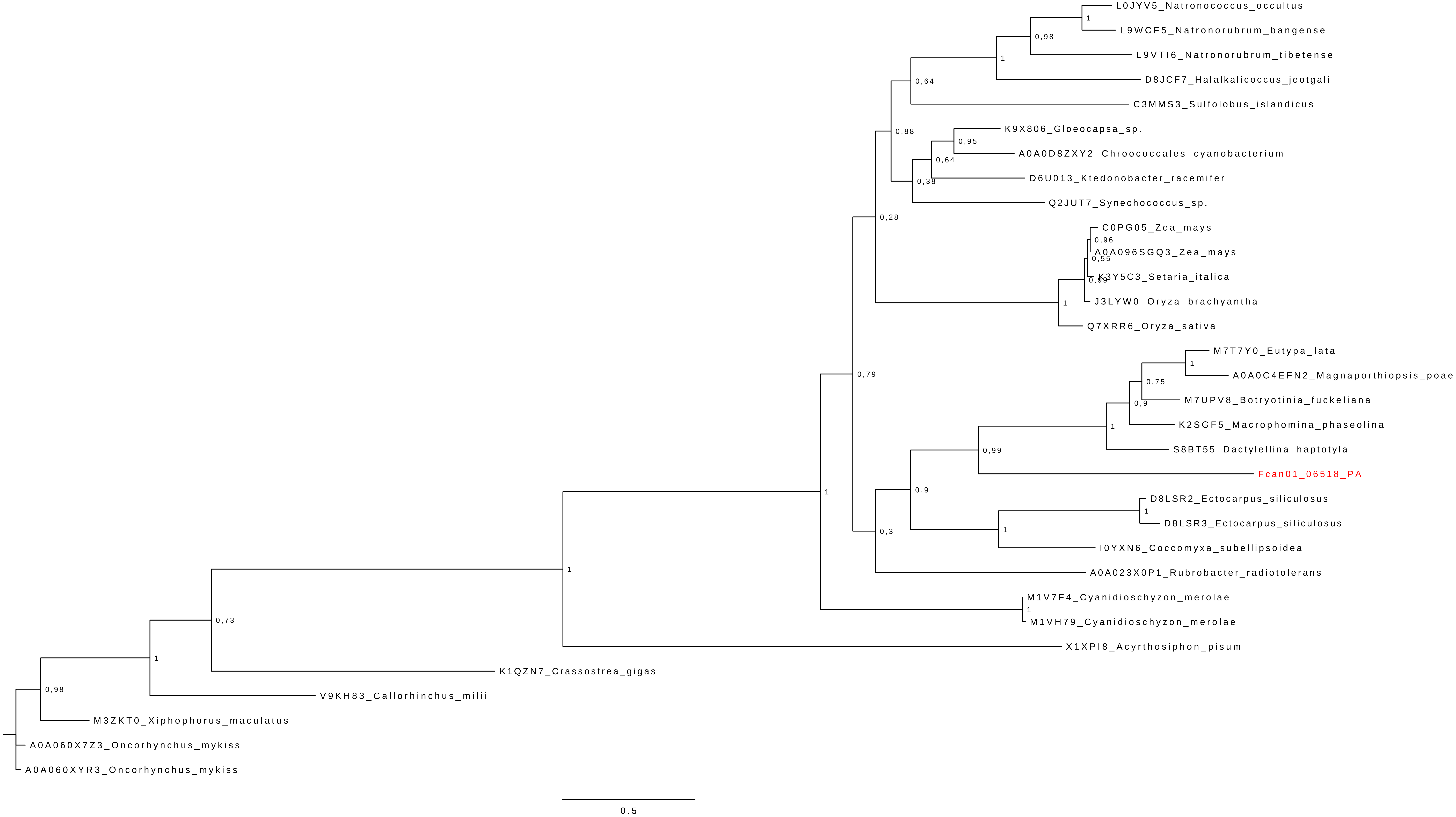
L0JYV5_Natronococcus_occultus
L9WCF5_Natronorubrum_bangense
L9VTI6_Natronorubrum_tibetense
D8JCF7_Halalkalicoccus_jeotgali
C3MMS3_Sulfolobus_islandicus
K9X806_Gloeocapsa_sp.
A0A0D8ZXY2_Chroococcales_cyanobacterium
D6U013_Ktedonobacter_racemifer
Q2JUT7_Synechococcus_sp.
C0PG05_Zea_mays
A0A096SGQ3_Zea_mays
K3Y5C3_Setaria_italica
J3LYW0_Oryza_brachyantha
Q7XRR6_Oryza_sativa
M7T7Y0_Eutypa_lata
A0A0C4EFN2_Magnaporthiopsis_poae
M7UPV8_Botryotinia_fuckeliana
K2SGF5_Macrophomina_phaseolina
S8BT55_Dactylellina_haptotyla
Fcan01_06518_PA
D8LSR2_Ectocarpus_siliculosus
D8LSR3_Ectocarpus_siliculosus
I0YXN6_Coccomyxa_subellipsoidea
A0A023X0P1_Rubrobacter_radiotolerans
M1V7F4_Cyanidioschyzon_merolae
M1VH79_Cyanidioschyzon_merolae
X1XPI8_Acyrthosiphon_pisum
K1QZN7_Crassostrea_gigas
V9KH83_Callorhinchus_milii
M3ZKT0_Xiphophorus_maculatus
A0A060X7Z3_Oncorhynchus_mykiss
A0A060XYR3_Oncorhynchus_mykiss
0.5

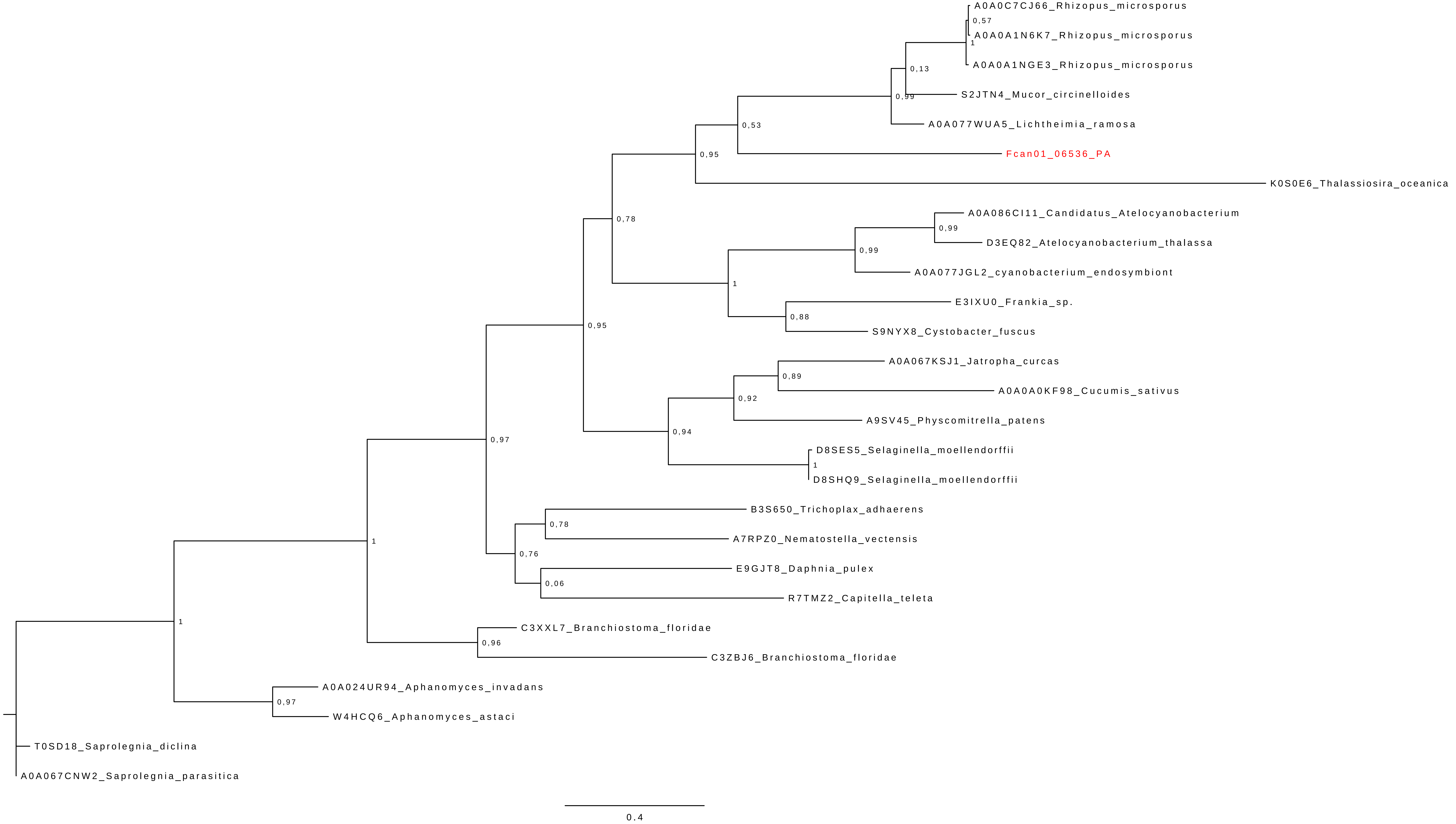
A0A0C7CJ66_Rhizopus_microsporus
0,57
A0A0A1N6K7_Rhizopus_microsporus
1
A0A0A1NGE3_Rhizopus_microsporus
0,13
S2JTN4_Mucor_circinelloides
0,99
A0A077WUA5_Lichtheimia_ramosa
0,53
Fcan01_06536_PA
0,95
K0S0E6_Thalassiosira_oceanica
0,78
A0A086CI11_Candidatus_Atelocyanobacterium
0,99
D3EQ82_Atelocyanobacterium_thalassa
0,99
A0A077JGL2_cyanobacterium_endosymbiont
1
E3IXU0_Frankia_sp.
0,88
S9NYX8_Cystobacter_fuscus
0,95
A0A067KSJ1_Jatropha_curcas
0,89
A0A0A0KF98_Cucumis_sativus
0,92
A9SV45_Physcomitrella_patens
0,94
D8SES5_Selaginella_moellendorffii
1
D8SHQ9_Selaginella_moellendorffii
0,97
B3S650_Trichoplax_adhaerens
0,78
A7RPZ0_Nematostella_vectensis
0,76
E9GJT8_Daphnia_pulex
0,06
R7TMZ2_Capitella_teleta
1
C3XXL7_Branchiostoma_floridae
0,96
C3ZBJ6_Branchiostoma_floridae
1
A0A024UR94_Aphanomyces_invadans
0,97
W4HCQ6_Aphanomyces_astaci
T0SD18_Saprolegnia_diclina
A0A067CNW2_Saprolegnia_parasitica
0.4

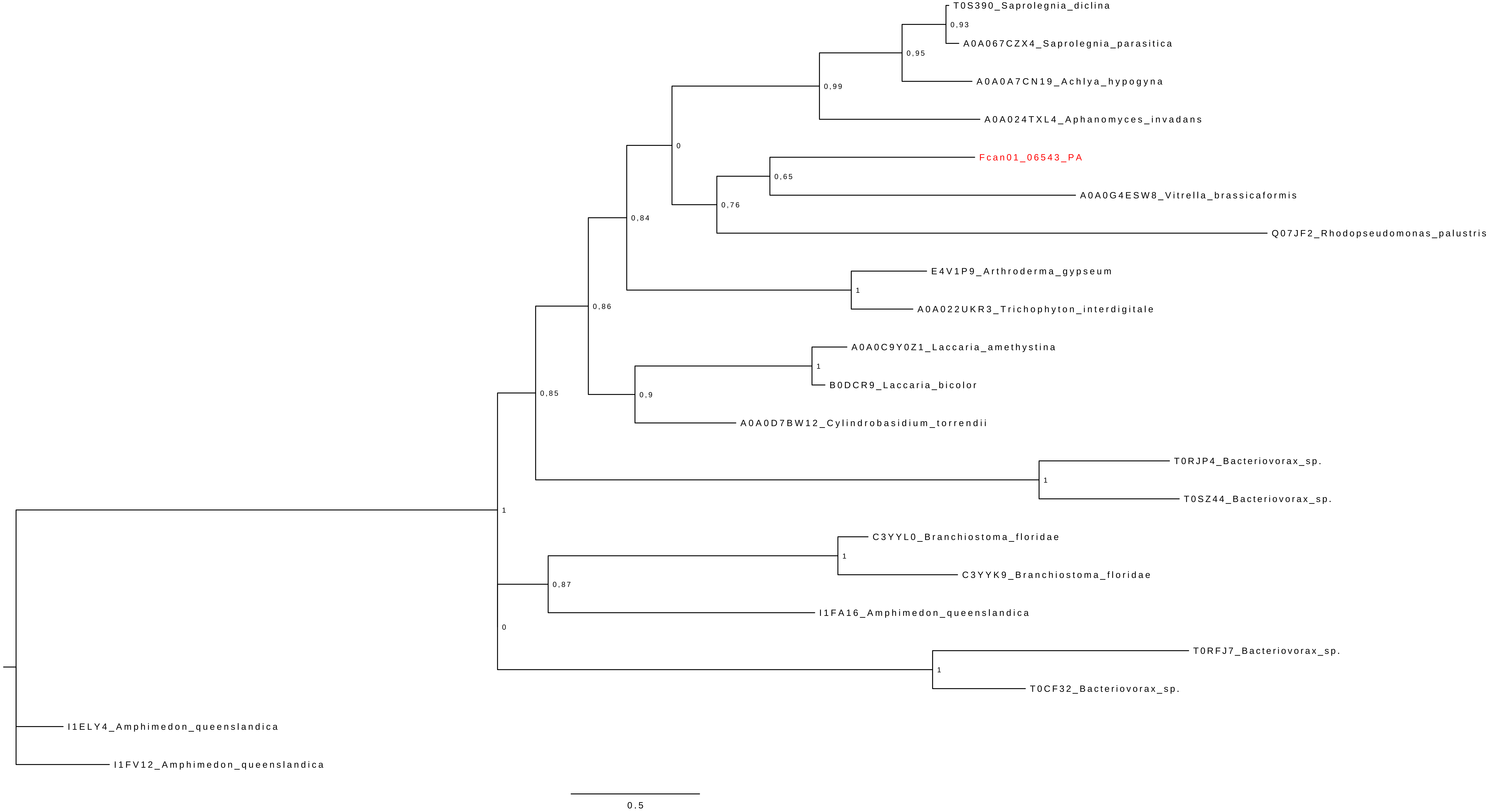

T0S390_Saprolegnia_diclina
0,93
A0A067CZX4_Saprolegnia_parasitica
0,95
A0A0A7CN19_Achlya_hypogyna
0,99
A0A024TXL4_Aphanomyces_invadans
0
Fcan01_06543_PA
0,65
A0A0G4ESW8_Vitrella_brassicaformis
0,76
Q07JF2_Rhodopseudomonas_palustris
0,84
E4V1P9_Arthroderma_gypseum
1
A0A022UKR3_Trichophyton_interdigitale
0,86
A0A0C9Y0Z1_Laccaria_amethystina
1
B0DCR9_Laccaria_bicolor
0,85
0,9
A0A0D7BW12_Cylindrobasidium_torrendii
T0RJP4_Bacteriovorax_sp.
1
T0SZ44_Bacteriovorax_sp.
1
C3YYL0_Branchiostoma_floridae
1
C3YYK9_Branchiostoma_floridae
0,87
I1FA16_Amphimedon_queenslandica
0
T0RFJ7_Bacteriovorax_sp.
1
T0CF32_Bacteriovorax_sp.
I1ELY4_Amphimedon_queenslandica
I1FV12_Amphimedon_queenslandica
0.5

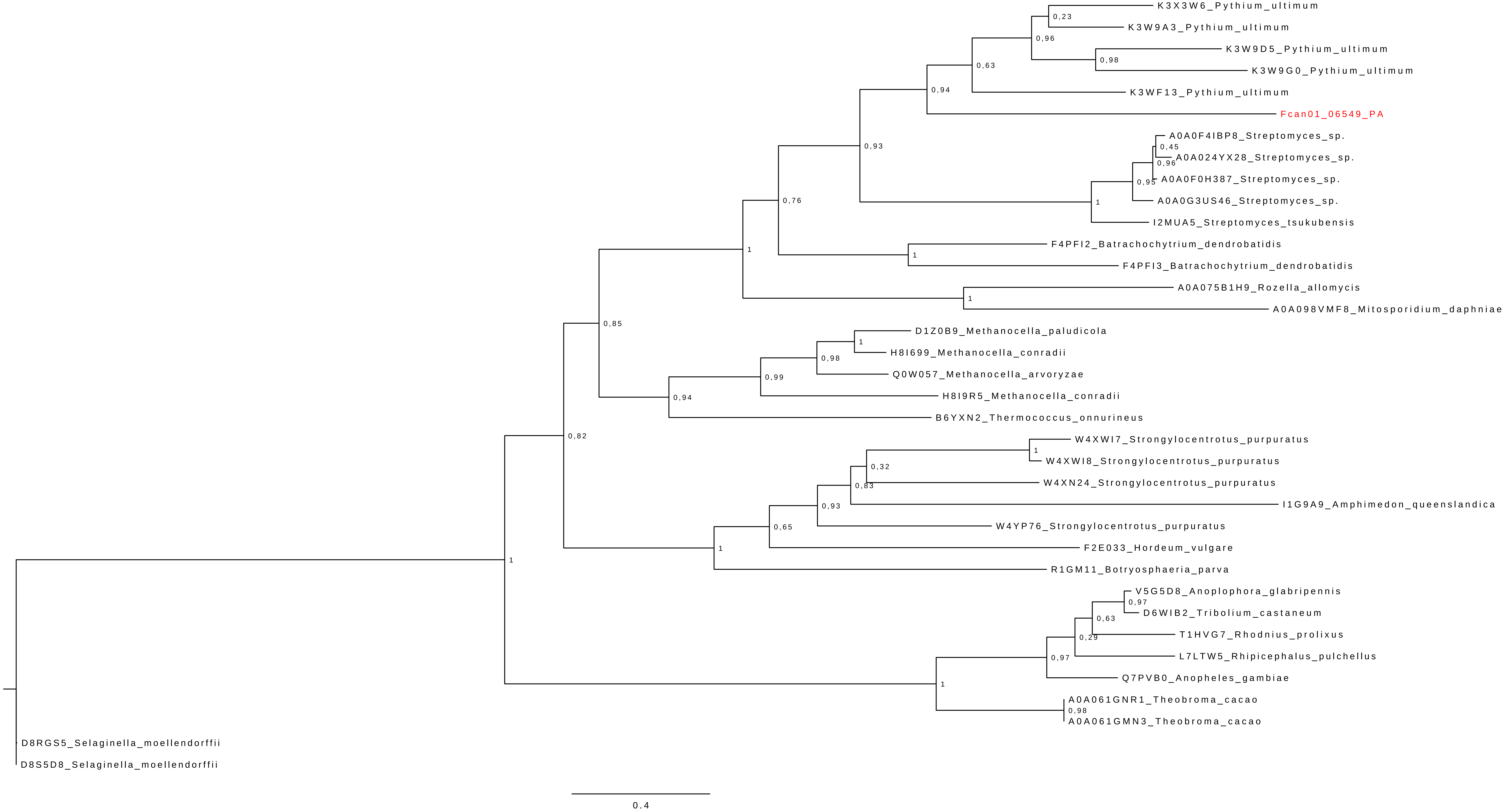
K3X3W6_Pythium_ultimum
K3W9A3_Pythium_ultimum
K3W9D5_Pythium_ultimum
K3W9G0_Pythium_ultimum
K3WF13_Pythium_ultimum
Fcan01_06549_PA
A0A0F4IBP8_Streptomyces_sp.
A0A024YX28_Streptomyces_sp.
A0A0F0H387_Streptomyces_sp.
A0A0G3US46_Streptomyces_sp.
I2MUA5_Streptomyces_tsukubensis
F4PFI2_Batrachochytrium_dendrobatidis
F4PFI3_Batrachochytrium_dendrobatidis
A0A075B1H9_Rozella_allomycis
A0A098VMF8_Mitosporidium_daphniae
D1Z0B9_Methanocella_paludicola
H8I699_Methanocella_conradii
Q0W057_Methanocella_arvoryzae
H8I9R5_Methanocella_conradii
B6YXN2_Thermococcus_onnurineus
W4XWI7_Strongylocentrotus_purpuratus
W4XWI8_Strongylocentrotus_purpuratus
W4XN24_Strongylocentrotus_purpuratus
I1G9A9_Amphimedon_queenslandica
W4YP76_Strongylocentrotus_purpuratus
F2E033_Hordeum_vulgare
R1GM11_Botryosphaeria_parva
V5G5D8_Anoplophora_glabripennis
D6WIB2_Tribolium_castaneum
T1HVG7_Rhodnius_prolixus
L7LTW5_Rhipicephalus_pulchellus
Q7PVB0_Anopheles_gambiae
A0A061GNR1_Theobroma_cacao
A0A061GMN3_Theobroma_cacao
D8RGS5_Selaginella_moellendorffii
D8S5D8_Selaginella_moellendorffii
0.4

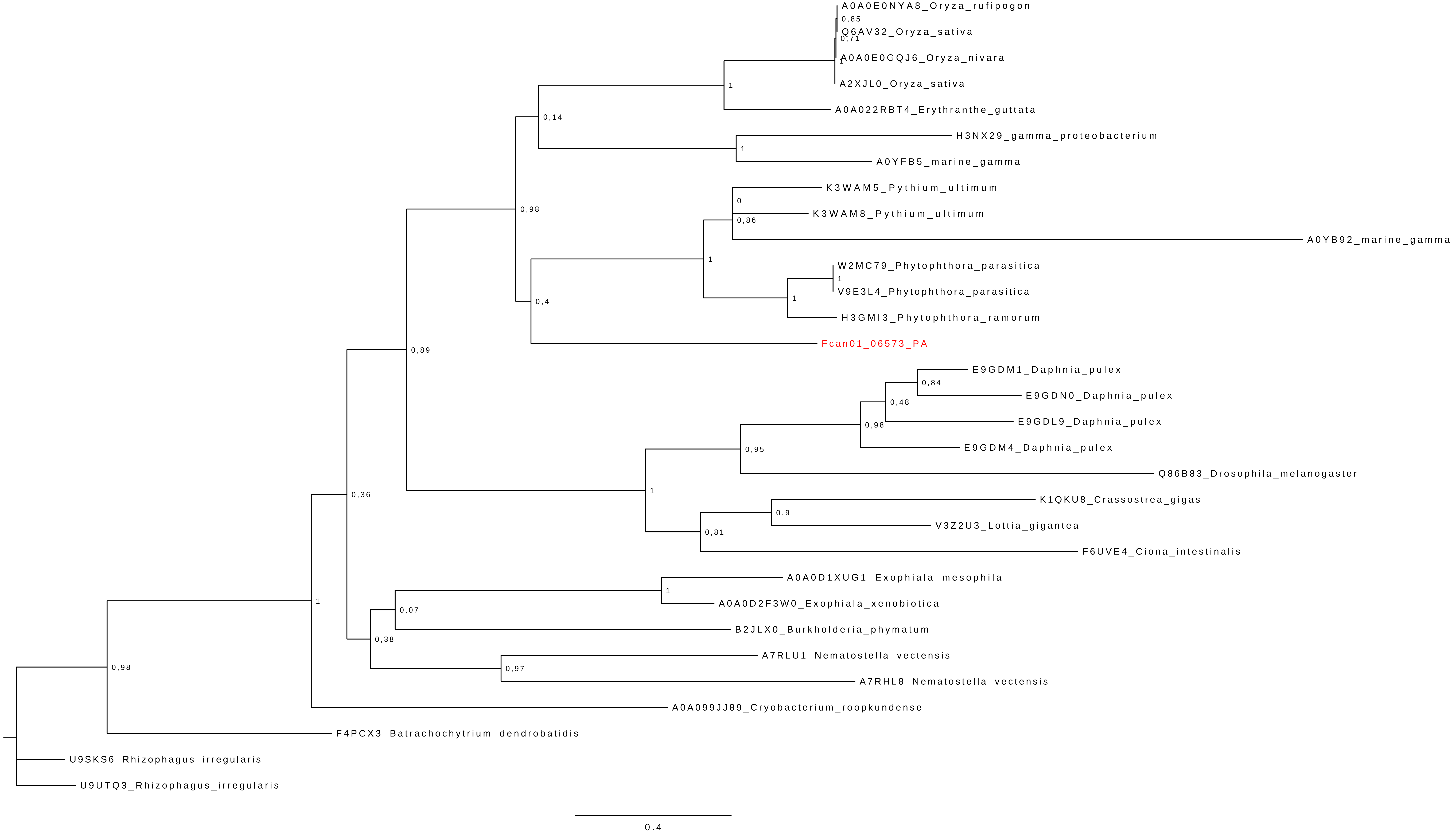
A0A0E0NYA8_Oryza_rufipogon
0,85
Q6AV32_Oryza_sativa
0,71
A0A0E0GQJ6_Oryza_nivara
1
A2XJL0_Oryza_sativa
1
A0A022RBT4_Erythranthe_guttata
0,14
H3NX29_gamma_proteobacterium
1
A0YFB5_marine_gamma
K3WAM5_Pythium_ultimum
0
K3WAM8_Pythium_ultimum
0,98
0,86
A0YB92_marine_gamma
1
W2MC79_Phytophthora_parasitica
1
V9E3L4_Phytophthora_parasitica
0,4
1
H3GMI3_Phytophthora_ramorum
Fcan01_06573_PA
0,89
E9GDM1_Daphnia_pulex
0,84
E9GDN0_Daphnia_pulex
0,48
E9GDL9_Daphnia_pulex
0,98
E9GDM4_Daphnia_pulex
0,95
Q86B83_Drosophila_melanogaster
1
0,36
K1QKU8_Crassostrea_gigas
0,9
V3Z2U3_Lottia_gigantea
0,81
F6UVE4_Ciona_intestinalis
A0A0D1XUG1_Exophiala_mesophila
1
1
A0A0D2F3W0_Exophiala_xenobiotica
0,07
B2JLX0_Burkholderia_phymatum
0,38
A7RLU1_Nematostella_vectensis
0,98
0,97
A7RHL8_Nematostella_vectensis
A0A099JJ89_Cryobacterium_roopkundense
F4PCX3_Batrachochytrium_dendrobatidis
U9SKS6_Rhizophagus_irregularis
U9UTQ3_Rhizophagus_irregularis
0.4

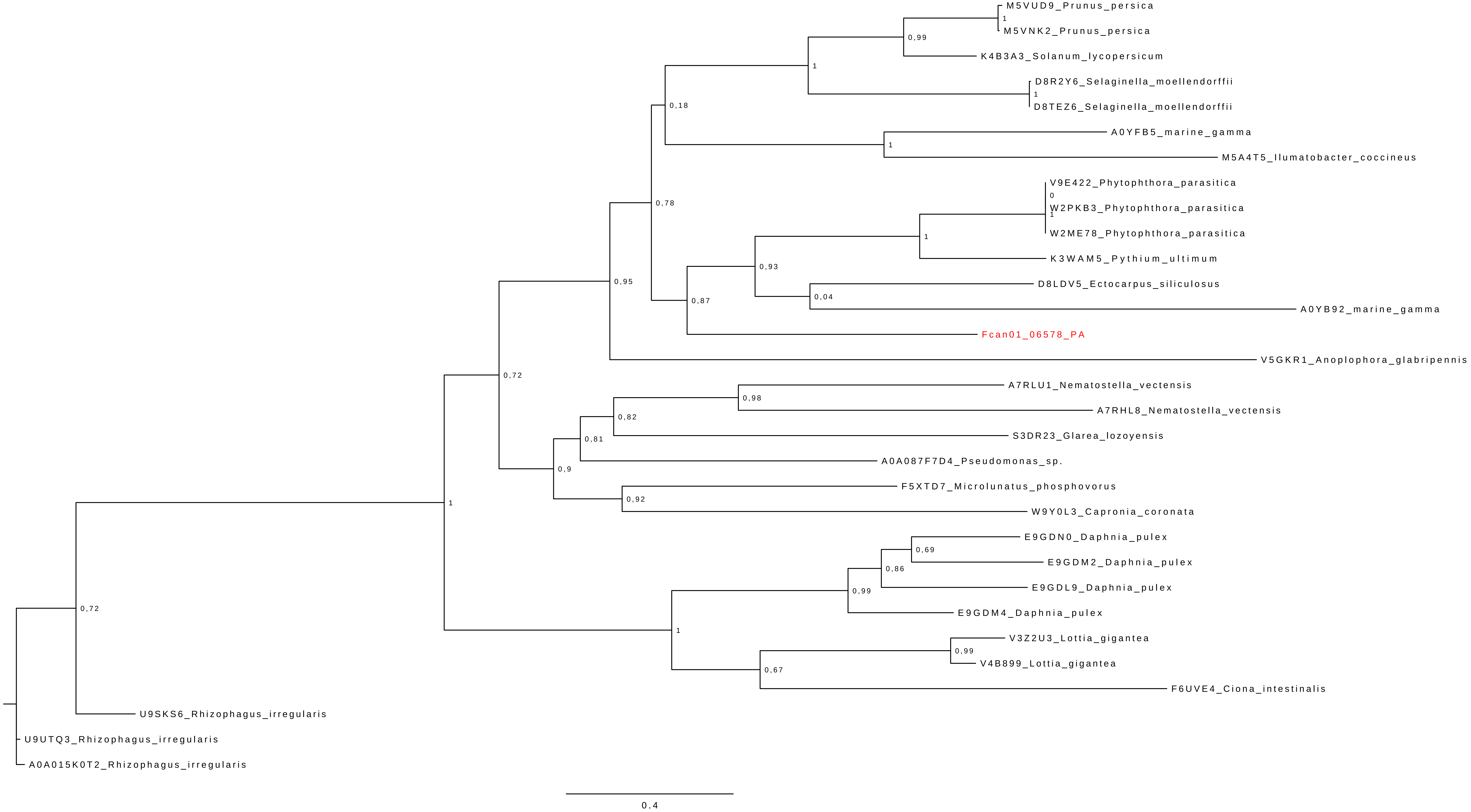
M5VUD9_Prunus_persica
1
M5VNK2_Prunus_persica
0,99
K4B3A3_Solanum_lycopersicum
1
D8R2Y6_Selaginella_moellendorffii
1
D8TEZ6_Selaginella_moellendorffii
0,18
A0YFB5_marine_gamma
1
M5A4T5_Ilumatobacter_coccineus
V9E422_Phytophthora_parasitica
0
W2PKB3_Phytophthora_parasitica
1
0,78
W2ME78_Phytophthora_parasitica
1
K3WAM5_Pythium_ultimum
0,93
D8LDV5_Ectocarpus_siliculosus
0,95
0,04
0,87
A0YB92_marine_gamma
Fcan01_06578_PA
V5GKR1_Anoplophora_glabripennis
0,72
A7RLU1_Nematostella_vectensis
0,98
A7RHL8_Nematostella_vectensis
0,82
S3DR23_Glarea_lozoyensis
0,81
A0A087F7D4_Pseudomonas_sp.
0,9
F5XTD7_Microlunatus_phosphovorus
1
0,92
W9Y0L3_Capronia_coronata
E9GDN0_Daphnia_pulex
0,69
E9GDM2_Daphnia_pulex
0,86
E9GDL9_Daphnia_pulex
0,99
E9GDM4_Daphnia_pulex
0,72
1
V3Z2U3_Lottia_gigantea
0,99
V4B899_Lottia_gigantea
0,67
F6UVE4_Ciona_intestinalis
U9SKS6_Rhizophagus_irregularis
U9UTQ3_Rhizophagus_irregularis
A0A015K0T2_Rhizophagus_irregularis
0.4

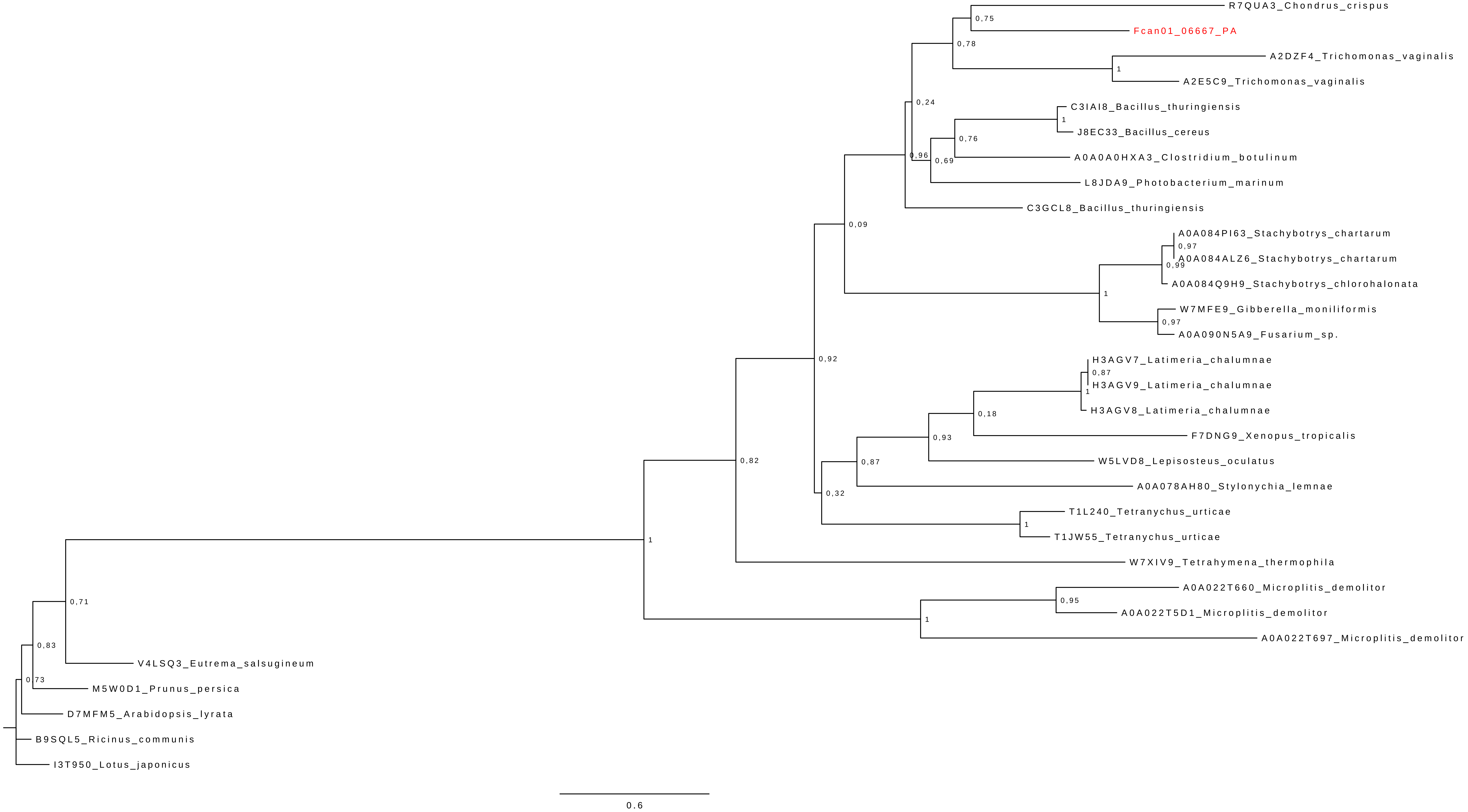
R7QUA3_Chondrus_crispus
0,75
Fcan01_06667_PA
0,78
A2DZF4_Trichomonas_vaginalis
1
A2E5C9_Trichomonas_vaginalis
0,24
C3IAI8_Bacillus_thuringiensis
1
J8EC33_Bacillus_cereus
0,76
0,96
A0A0A0HXA3_Clostridium_botulinum
0,69
L8JDA9_Photobacterium_marinum
C3GCL8_Bacillus_thuringiensis
0,09
A0A084PI63_Stachybotrys_chartarum
0,97
A0A084ALZ6_Stachybotrys_chartarum
0,99
A0A084Q9H9_Stachybotrys_chlorohalonata
1
W7MFE9_Gibberella_moniliformis
0,97
A0A090N5A9_Fusarium_sp.
0,92
H3AGV7_Latimeria_chalumnae
0,87
H3AGV9_Latimeria_chalumnae
1
H3AGV8_Latimeria_chalumnae
0,18
F7DNG9_Xenopus_tropicalis
0,93
W5LVD8_Lepisosteus_oculatus
0,87
A0A078AH80_Stylonychia_lemnae
0,32
T1L240_Tetranychus_urticae
1
T1JW55_Tetranychus_urticae
0,82
W7XIV9_Tetrahymena_thermophila
1
A0A022T660_Microplitis_demolitor
0,95
A0A022T5D1_Microplitis_demolitor
1
A0A022T697_Microplitis_demolitor
0,71
0,83
V4LSQ3_Eutrema_salsugineum
0,73
M5W0D1_Prunus_persica
D7MFM5_Arabidopsis_lyrata
B9SQL5_Ricinus_communis
I3T950_Lotus_japonicus
0.6

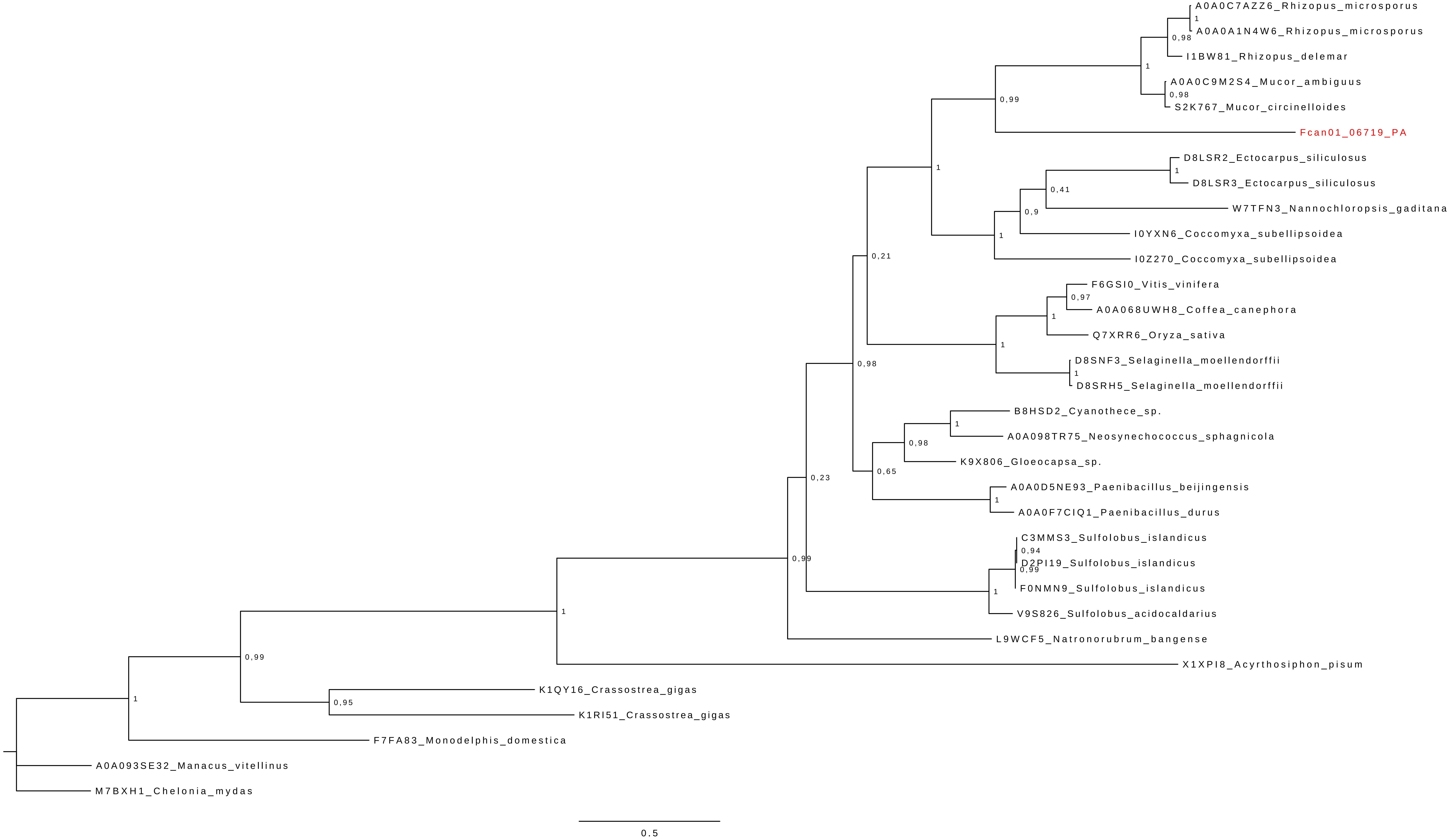
A0A0C7AZZ6_Rhizopus_microsporus
A0A0A1N4W6_Rhizopus_microsporus
I1BW81_Rhizopus_delemar
A0A0C9M2S4_Mucor_ambiguus
S2K767_Mucor_circinelloides
Fcan01_06719_PA
D8LSR2_Ectocarpus_siliculosus
D8LSR3_Ectocarpus_siliculosus
W7TFN3_Nannochloropsis_gaditana
I0YXN6_Coccomyxa_subellipsoidea
I0Z270_Coccomyxa_subellipsoidea
F6GSI0_Vitis_vinifera
A0A068UWH8_Coffea_canephora
Q7XRR6_Oryza_sativa
D8SNF3_Selaginella_moellendorffii
D8SRH5_Selaginella_moellendorffii
B8HSD2_Cyanothece_sp.
A0A098TR75_Neosynechococcus_sphagnicola
K9X806_Gloeocapsa_sp.
A0A0D5NE93_Paenibacillus_beijingensis
A0A0F7CIQ1_Paenibacillus_durus
C3MMS3_Sulfolobus_islandicus
D2PI19_Sulfolobus_islandicus
F0NMN9_Sulfolobus_islandicus
V9S826_Sulfolobus_acidocaldarius
L9WCF5_Natronorubrum_bangense
X1XPI8_Acyrthosiphon_pisum
K1QY16_Crassostrea_gigas
K1RI51_Crassostrea_gigas
F7FA83_Monodelphis_domestica
A0A093SE32_Manacus_vitellinus
M7BXH1_Chelonia_mydas
0.5

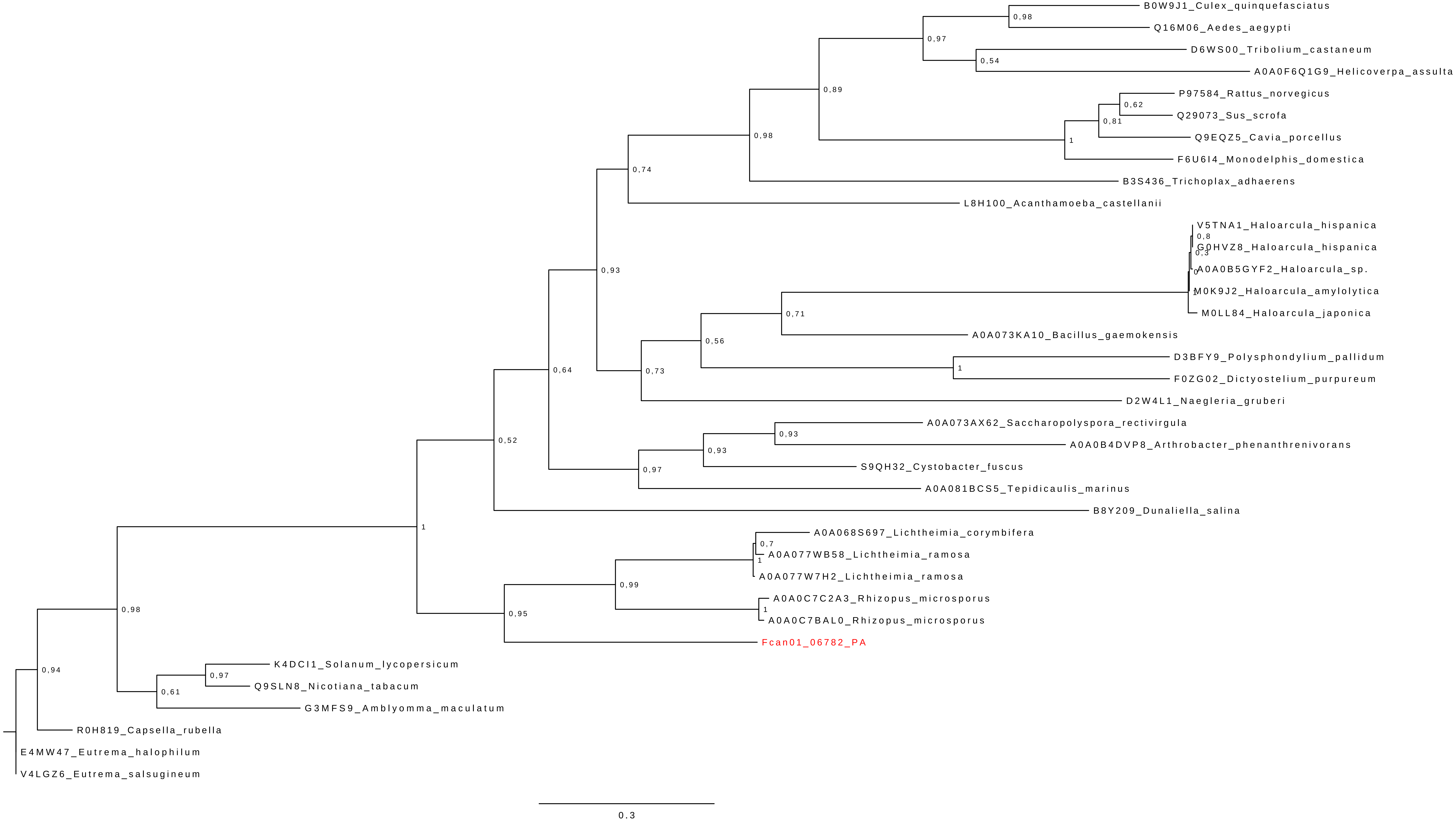
B0W9J1_Culex_quinquefasciatus
0,98
Q16M06_Aedes_aegypti
0,97
D6WS00_Tribolium_castaneum
0,54
A0A0F6Q1G9_Helicoverpa_assulta
0,89
P97584_Rattus_norvegicus
0,62
Q29073_Sus_scrofa
0,81
0,98
1
Q9EQZ5_Cavia_porcellus
F6U6I4_Monodelphis_domestica
0,74
B3S436_Trichoplax_adhaerens
L8H100_Acanthamoeba_castellanii
V5TNA1_Haloarcula_hispanica
0,8
G0HVZ8_Haloarcula_hispanica
0,3
A0A0B5GYF2_Haloarcula_sp.
0,93
M0K9J2_Haloarcula_amylolytica
M0LL84_Haloarcula_japonica
0,71
A0A073KA10_Bacillus_gaemokensis
0,56
D3BFY9_Polysphondylium_pallidum
1
0,64
0,73
F0ZG02_Dictyostelium_purpureum
D2W4L1_Naegleria_gruberi
A0A073AX62_Saccharopolyspora_rectivirgula
0,93
A0A0B4DVP8_Arthrobacter_phenanthrenivorans
0,52
0,93
S9QH32_Cystobacter_fuscus
0,97
A0A081BCS5_Tepidicaulis_marinus
B8Y209_Dunaliella_salina
1
A0A068S697_Lichtheimia_corymbifera
0,7
A0A077WB58_Lichtheimia_ramosa
1
A0A077W7H2_Lichtheimia_ramosa
0,99
A0A0C7C2A3_Rhizopus_microsporus
0,98
0,95
1
A0A0C7BAL0_Rhizopus_microsporus
Fcan01_06782_PA
K4DCI1_Solanum_lycopersicum
0,97
0,94
Q9SLN8_Nicotiana_tabacum
0,61
G3MFS9_Amblyomma_maculatum
R0H819_Capsella_rubella
E4MW47_Eutrema_halophilum
V4LGZ6_Eutrema_salsugineum
0.3

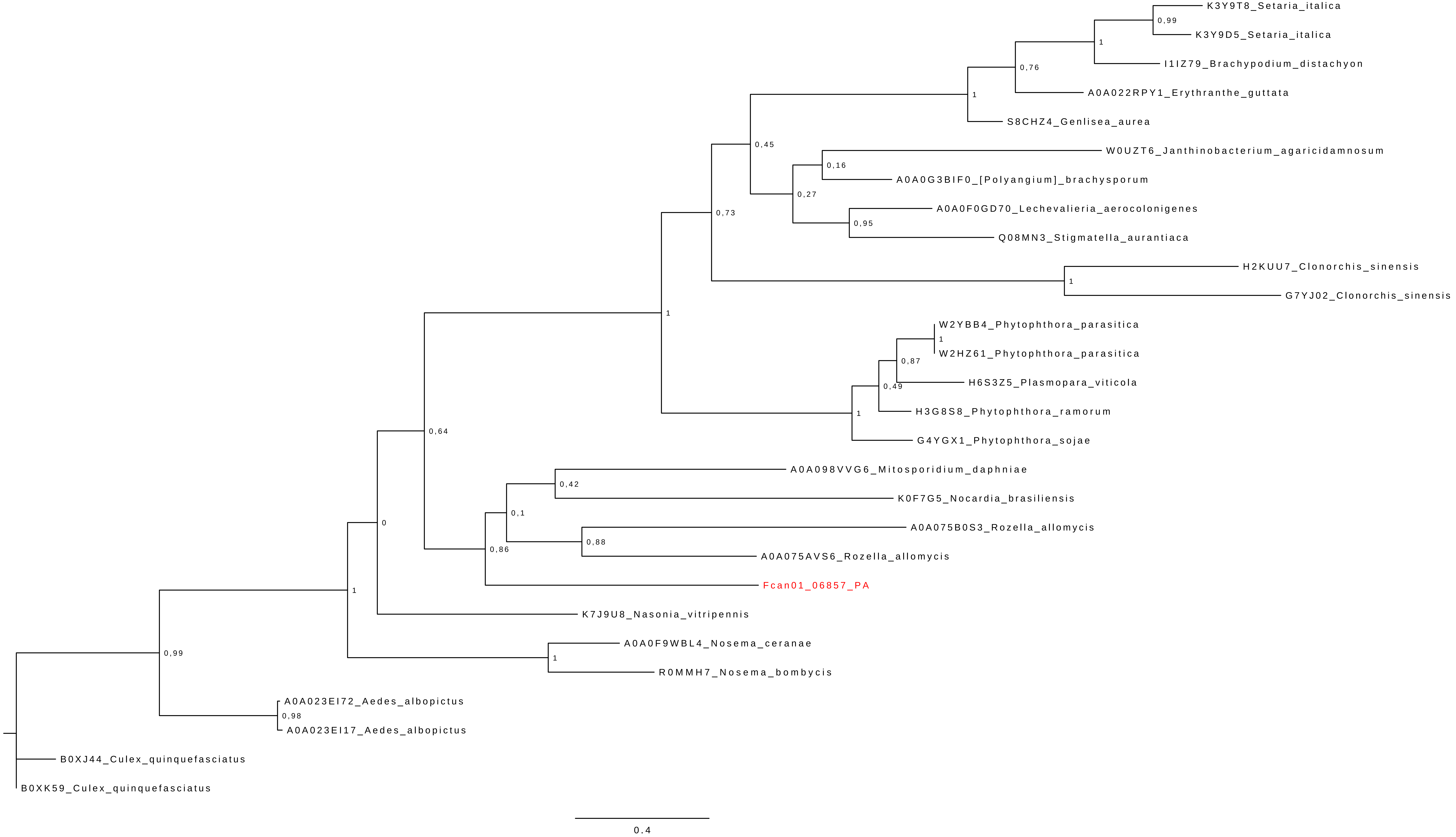
K3Y9T8_Setaria_italica
0,99
K3Y9D5_Setaria_italica
1
I1IZ79_Brachypodium_distachyon
0,76
A0A022RPY1_Erythranthe_guttata
1
S8CHZ4_Genlisea_aurea
0,45
W0UZT6_Janthinobacterium_agaricidamnosum
0,16
A0A0G3BIF0_[Polyangium]_brachysporum
0,27
A0A0F0GD70_Lechevalieria_aerocolonigenes
0,73
0,95
Q08MN3_Stigmatella_aurantiaca
H2KUU7_Clonorchis_sinensis
1
G7YJ02_Clonorchis_sinensis
1
W2YBB4_Phytophthora_parasitica
1
W2HZ61_Phytophthora_parasitica
0,87
H6S3Z5_Plasmopara_viticola
0,49
H3G8S8_Phytophthora_ramorum
1
G4YGX1_Phytophthora_sojae
0,64
A0A098VVG6_Mitosporidium_daphniae
0,42
K0F7G5_Nocardia_brasiliensis
0,1
A0A075B0S3_Rozella_allomycis
0,88
A0A075AVS6_Rozella_allomycis
0,86
0
Fcan01_06857_PA
1
K7J9U8_Nasonia_vitripennis
A0A0F9WBL4_Nosema_ceranae
1
R0MMH7_Nosema_bombycis
0,99
A0A023EI72_Aedes_albopictus
0,98
A0A023EI17_Aedes_albopictus
B0XJ44_Culex_quinquefasciatus
B0XK59_Culex_quinquefasciatus
0.4

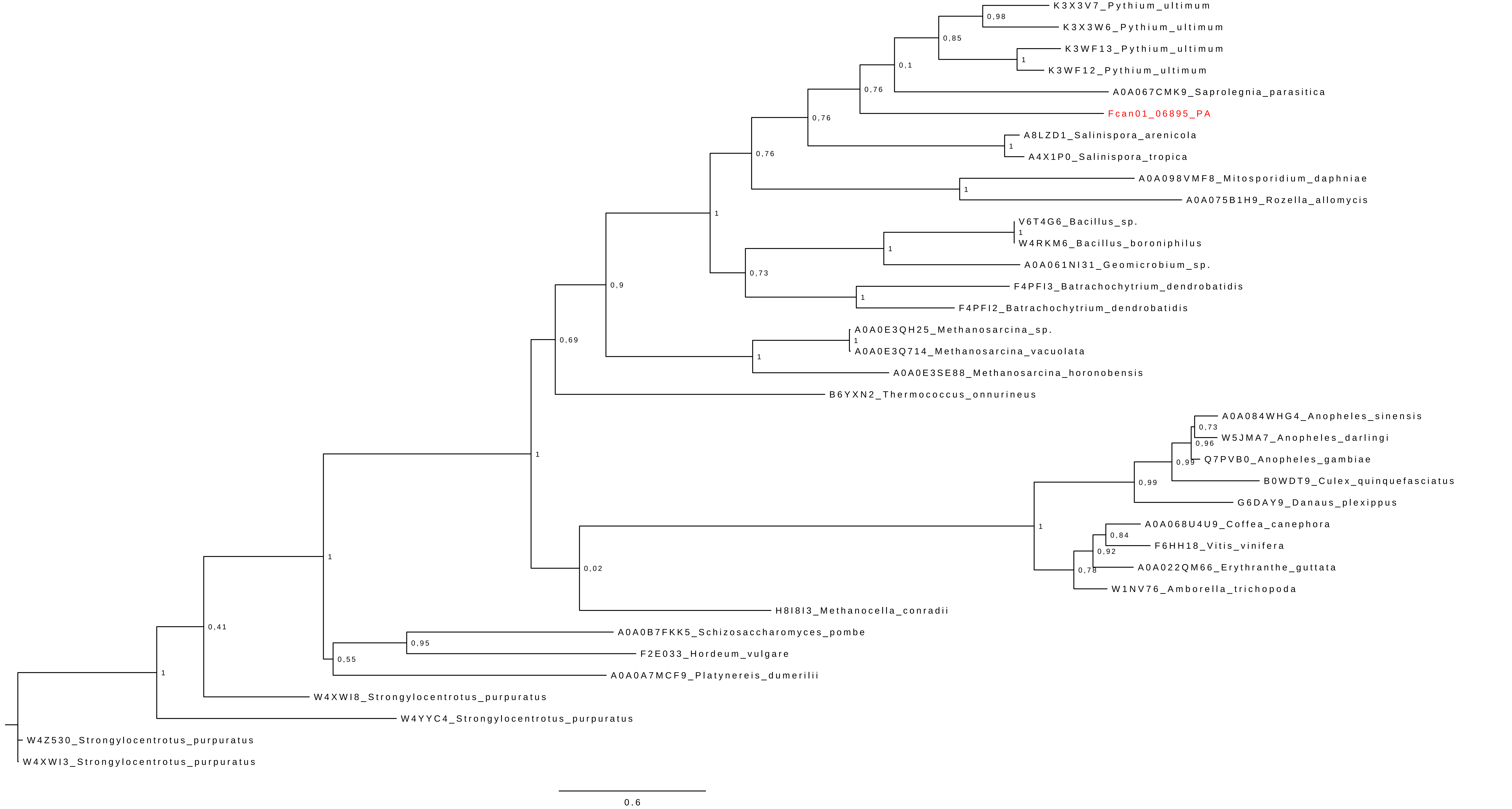
K3X3V7_Pythium_ultimum
K3X3W6_Pythium_ultimum
K3WF13_Pythium_ultimum
K3WF12_Pythium_ultimum
A0A067CMK9_Saprolegnia_parasitica
Fcan01_06895_PA
A8LZD1_Salinispora_arenicola
A4X1P0_Salinispora_tropica
A0A098VMF8_Mitosporidium_daphniae
A0A075B1H9_Rozella_allomycis
V6T4G6_Bacillus_sp.
W4RKM6_Bacillus_boroniphilus
A0A061NI31_Geomicrobium_sp.
F4PFI3_Batrachochytrium_dendrobatidis
F4PFI2_Batrachochytrium_dendrobatidis
A0A0E3QH25_Methanosarcina_sp.
A0A0E3Q714_Methanosarcina_vacuolata
A0A0E3SE88_Methanosarcina_horonobensis
B6YXN2_Thermococcus_onnurineus
A0A084WHG4_Anopheles_sinensis
W5JMA7_Anopheles_darlingi
Q7PVB0_Anopheles_gambiae
B0WDT9_Culex_quinquefasciatus
G6DAY9_Danaus_plexippus
A0A068U4U9_Coffea_canephora
F6HH18_Vitis_vinifera
A0A022QM66_Erythranthe_guttata
W1NV76_Amborella_trichopoda
H8I8I3_Methanocella_conradii
A0A0B7FKK5_Schizosaccharomyces_pombe
F2E033_Hordeum_vulgare
A0A0A7MCF9_Platynereis_dumerilii
W4XWI8_Strongylocentrotus_purpuratus
W4YYC4_Strongylocentrotus_purpuratus
W4Z530_Strongylocentrotus_purpuratus
W4XWI3_Strongylocentrotus_purpuratus
0.6

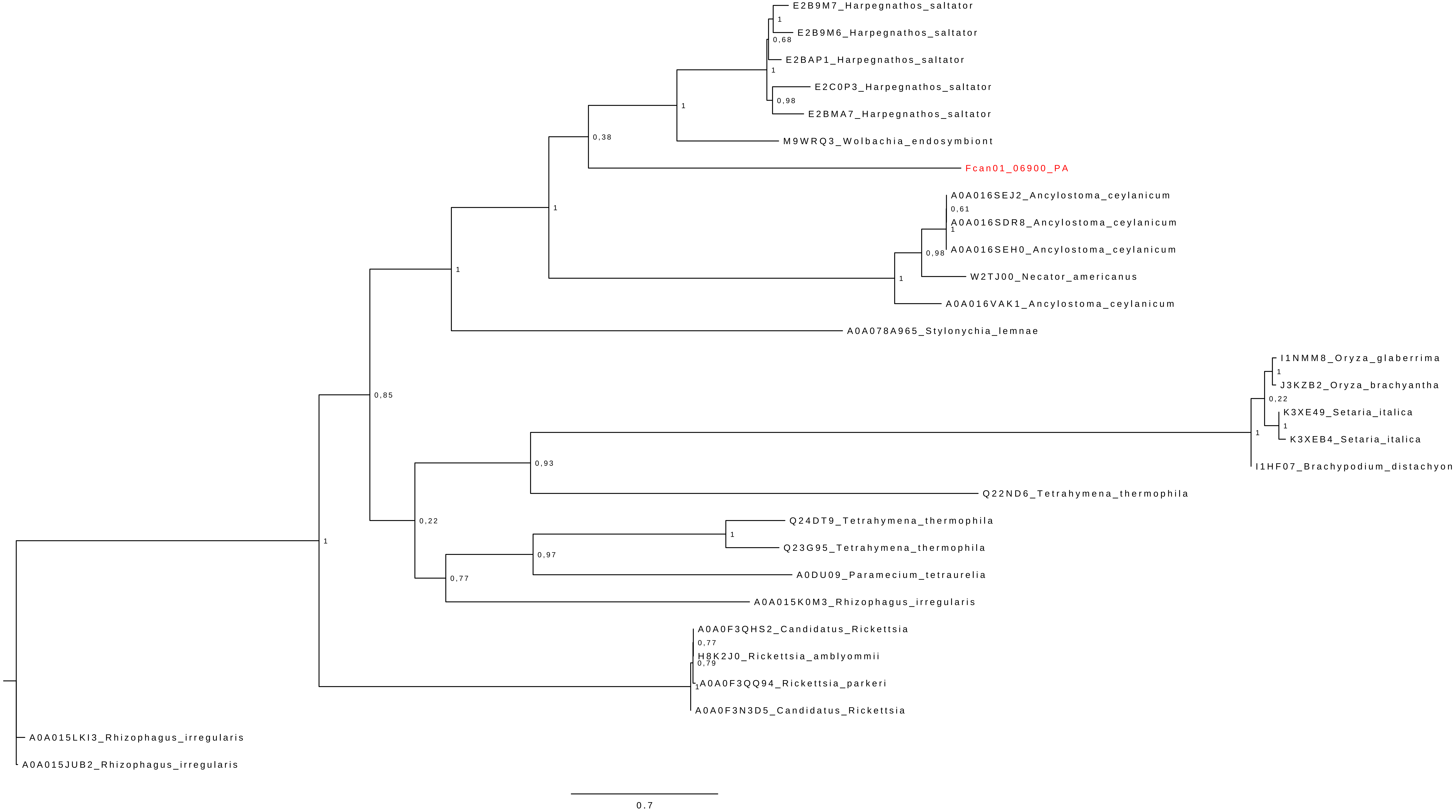
E2B9M7_Harpegnathos_saltator
E2B9M6_Harpegnathos_saltator
E2BAP1_Harpegnathos_saltator
E2C0P3_Harpegnathos_saltator
E2BMA7_Harpegnathos_saltator
M9WRQ3_Wolbachia_endosymbiont
Fcan01_06900_PA
A0A016SEJ2_Ancylostoma_ceylanicum
A0A016SDR8_Ancylostoma_ceylanicum
A0A016SEH0_Ancylostoma_ceylanicum
W2TJ00_Necator_americanus
A0A016VAK1_Ancylostoma_ceylanicum
A0A078A965_Stylonychia_lemnae
I1NMM8_Oryza_glaberrima
J3KZB2_Oryza_brachyantha
K3XE49_Setaria_italica
K3XEB4_Setaria_italica
I1HF07_Brachypodium_distachyon
Q22ND6_Tetrahymena_thermophila
Q24DT9_Tetrahymena_thermophila
Q23G95_Tetrahymena_thermophila
A0DU09_Paramecium_tetraurelia
A0A015K0M3_Rhizophagus_irregularis
A0A0F3QHS2_Candidatus_Rickettsia
H8K2J0_Rickettsia_amblyommii
A0A0F3QQ94_Rickettsia_parkeri
A0A0F3N3D5_Candidatus_Rickettsia
A0A015LKI3_Rhizophagus_irregularis
A0A015JUB2_Rhizophagus_irregularis
0.7

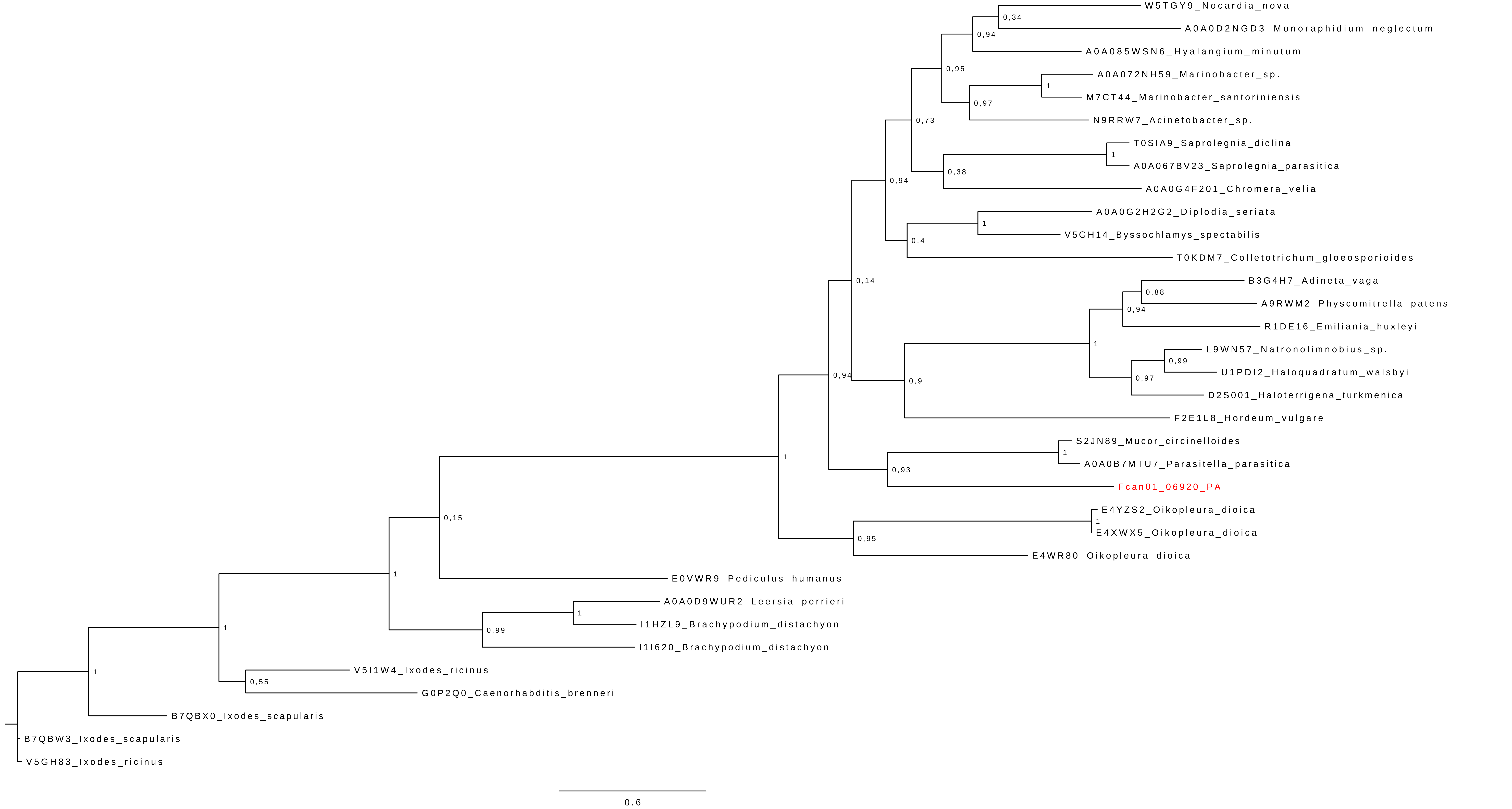
W5TGY9_Nocardia_nova
A0A0D2NGD3_Monoraphidium_neglectum
A0A085WSN6_Hyalangium_minutum
A0A072NH59_Marinobacter_sp.
M7CT44_Marinobacter_santoriniensis
N9RRW7_Acinetobacter_sp.
T0SIA9_Saprolegnia_diclina
A0A067BV23_Saprolegnia_parasitica
A0A0G4F201_Chromera_velia
A0A0G2H2G2_Diplodia_seriata
V5GH14_Byssochlamys_spectabilis
T0KDM7_Colletotrichum_gloeosporioides
B3G4H7_Adineta_vaga
A9RWM2_Physcomitrella_patens
R1DE16_Emiliania_huxleyi
L9WN57_Natronolimnobius_sp.
U1PDI2_Haloquadratum_walsbyi
D2S001_Haloterrigena_turkmenica
F2E1L8_Hordeum_vulgare
S2JN89_Mucor_circinelloides
A0A0B7MTU7_Parasitella_parasitica
Fcan01_06920_PA
E4YZS2_Oikopleura_dioica
E4XWX5_Oikopleura_dioica
E4WR80_Oikopleura_dioica
E0VWR9_Pediculus_humanus
A0A0D9WUR2_Leersia_perrieri
I1HZL9_Brachypodium_distachyon
I1I620_Brachypodium_distachyon
V5I1W4_Ixodes_ricinus
G0P2Q0_Caenorhabditis_brenneri
B7QBX0_Ixodes_scapularis
B7QBW3_Ixodes_scapularis
V5GH83_Ixodes_ricinus
0,34
0,94
0,95
1
0,97
0,73
1
0,38
0,94
1
0,4
0,14
0,88
0,94
1
0,99
0,97
0,94
0,9
1
1
0,93
1
0,95
0,15
1
1
0,99
1
0,55
1
0.6

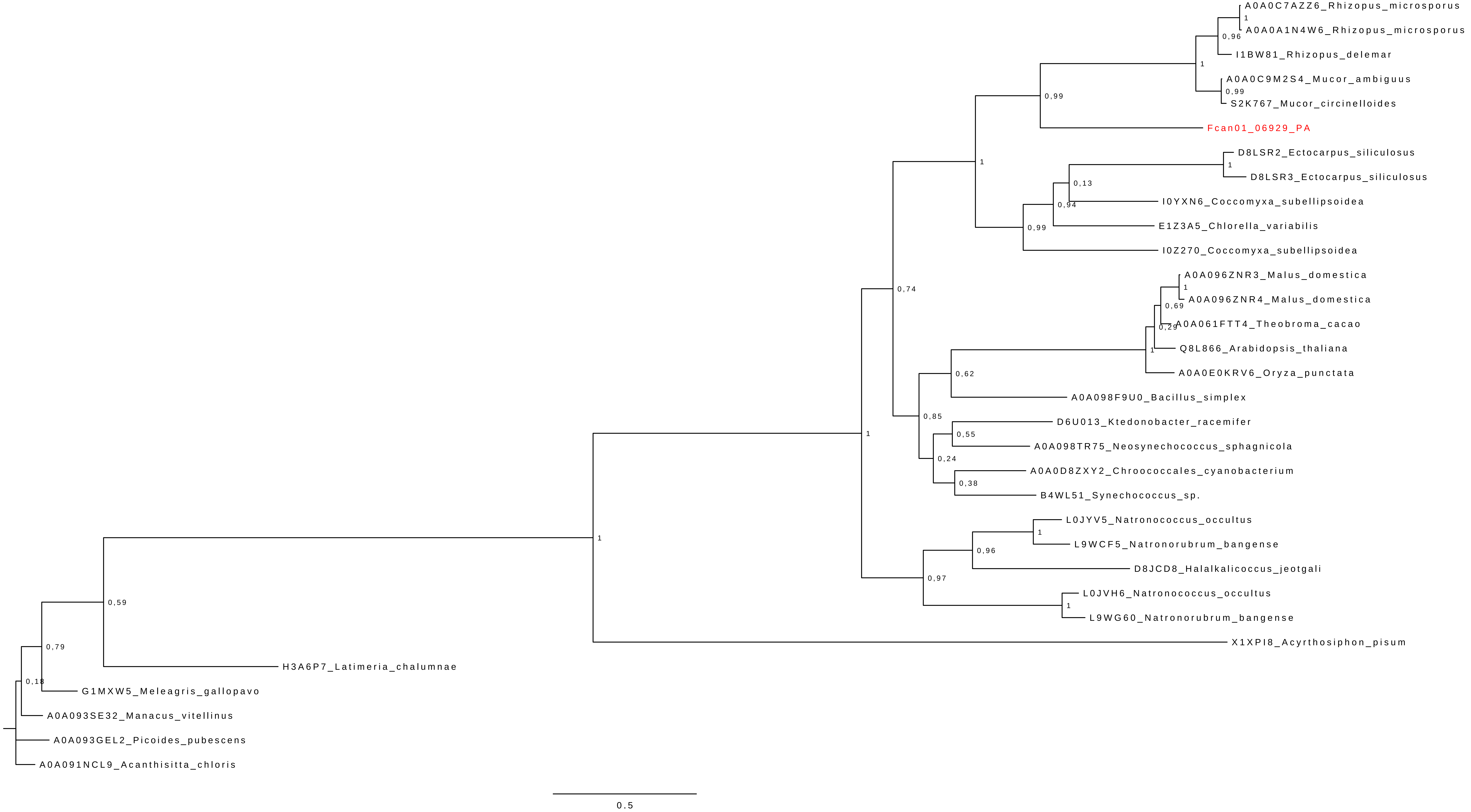
A0A0C7AZZ6_Rhizopus_microsporus
A0A0A1N4W6_Rhizopus_microsporus
I1BW81_Rhizopus_delemar
A0A0C9M2S4_Mucor_ambiguus
S2K767_Mucor_circinelloides
Fcan01_06929_PA
D8LSR2_Ectocarpus_siliculosus
D8LSR3_Ectocarpus_siliculosus
I0YXN6_Coccomyxa_subellipsoidea
E1Z3A5_Chlorella_variabilis
I0Z270_Coccomyxa_subellipsoidea
A0A096ZNR3_Malus_domestica
A0A096ZNR4_Malus_domestica
A0A061FTT4_Theobroma_cacao
Q8L866_Arabidopsis_thaliana
A0A0E0KRV6_Oryza_punctata
A0A098F9U0_Bacillus_simplex
D6U013_Ktedonobacter_racemifer
A0A098TR75_Neosynechococcus_sphagnicola
A0A0D8ZXY2_Chroococcales_cyanobacterium
B4WL51_Synechococcus_sp.
L0JYV5_Natronococcus_occultus
L9WCF5_Natronorubrum_bangense
D8JCD8_Halalkalicoccus_jeotgali
L0JVH6_Natronococcus_occultus
L9WG60_Natronorubrum_bangense
X1XPI8_Acyrthosiphon_pisum
H3A6P7_Latimeria_chalumnae
G1MXW5_Meleagris_gallopavo
A0A093SE32_Manacus_vitellinus
A0A093GEL2_Picoides_pubescens
A0A091NCL9_Acanthisitta_chloris
1
0,96
1
0,99
0,99
1
1
0,13
0,94
0,99
1
0,69
0,29
1
0,74
0,62
0,85
0,55
0,24
0,38
1
1
0,96
0,97
1
1
0,59
0,79
0,18
0.5

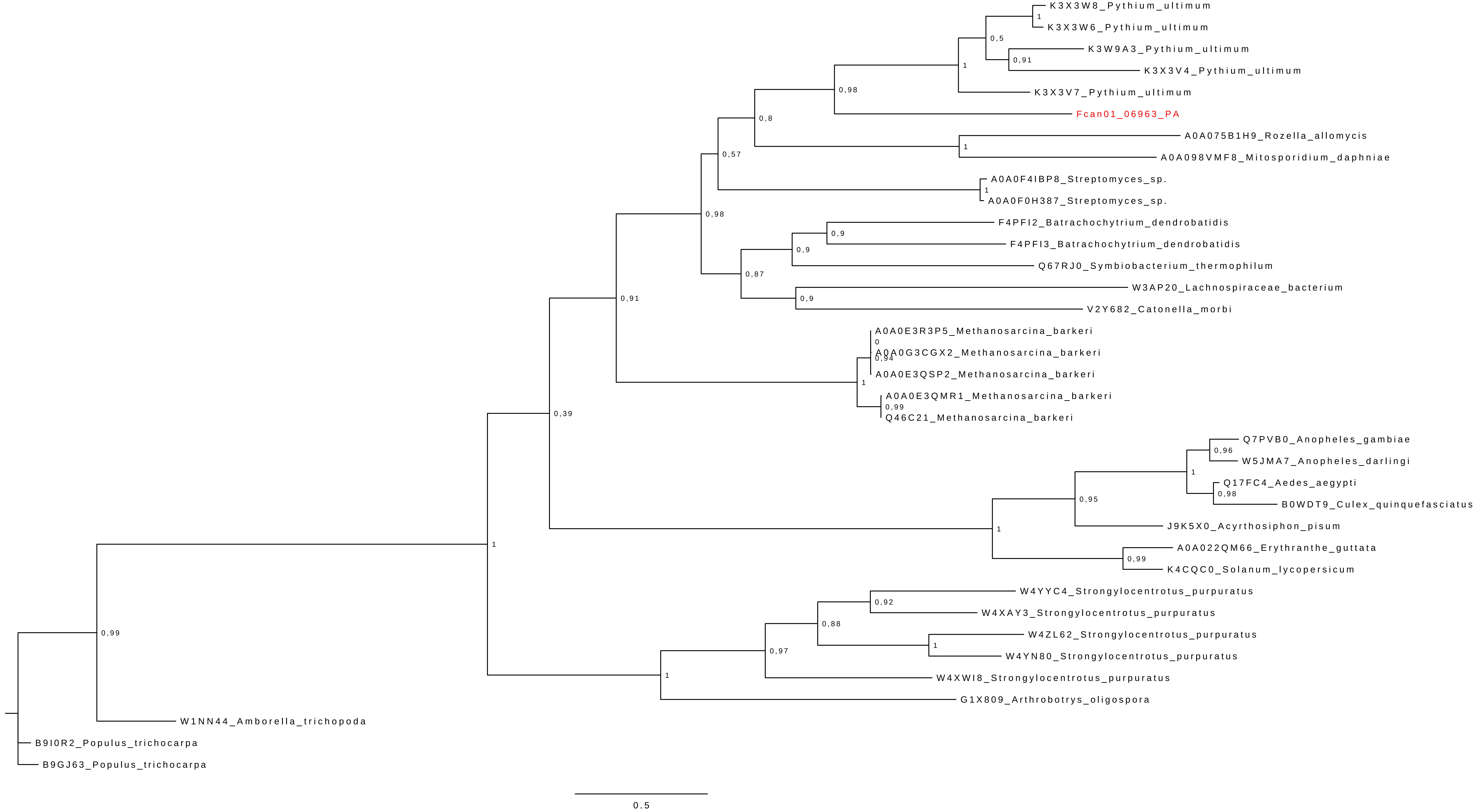
K3X3W8_Pythium_ultimum
K3X3W6_Pythium_ultimum
K3W9A3_Pythium_ultimum
K3X3V4_Pythium_ultimum
K3X3V7_Pythium_ultimum
Fcan01_06963_PA
A0A075B1H9_Rozella_allomycis
A0A098VMF8_Mitosporidium_daphniae
A0A0F4IBP8_Streptomyces_sp.
A0A0F0H387_Streptomyces_sp.
F4PFI2_Batrachochytrium_dendrobatidis
F4PFI3_Batrachochytrium_dendrobatidis
Q67RJ0_Symbiobacterium_thermophilum
W3AP20_Lachnospiraceae_bacterium
V2Y682_Catonella_morbi
A0A0E3R3P5_Methanosarcina_barkeri
A0A0G3CGX2_Methanosarcina_barkeri
A0A0E3QSP2_Methanosarcina_barkeri
A0A0E3QMR1_Methanosarcina_barkeri
Q46C21_Methanosarcina_barkeri
Q7PVB0_Anopheles_gambiae
W5JMA7_Anopheles_darlingi
Q17FC4_Aedes_aegypti
B0WDT9_Culex_quinquefasciatus
J9K5X0_Acyrthosiphon_pisum
A0A022QM66_Erythranthe_guttata
K4CQC0_Solanum_lycopersicum
W4YYC4_Strongylocentrotus_purpuratus
W4XAY3_Strongylocentrotus_purpuratus
W4ZL62_Strongylocentrotus_purpuratus
W4YN80_Strongylocentrotus_purpuratus
W4XWI8_Strongylocentrotus_purpuratus
G1X809_Arthrobotrys_oligospora
W1NN44_Amborella_trichopoda
B9I0R2_Populus_trichocarpa
B9GJ63_Populus_trichocarpa
0.5

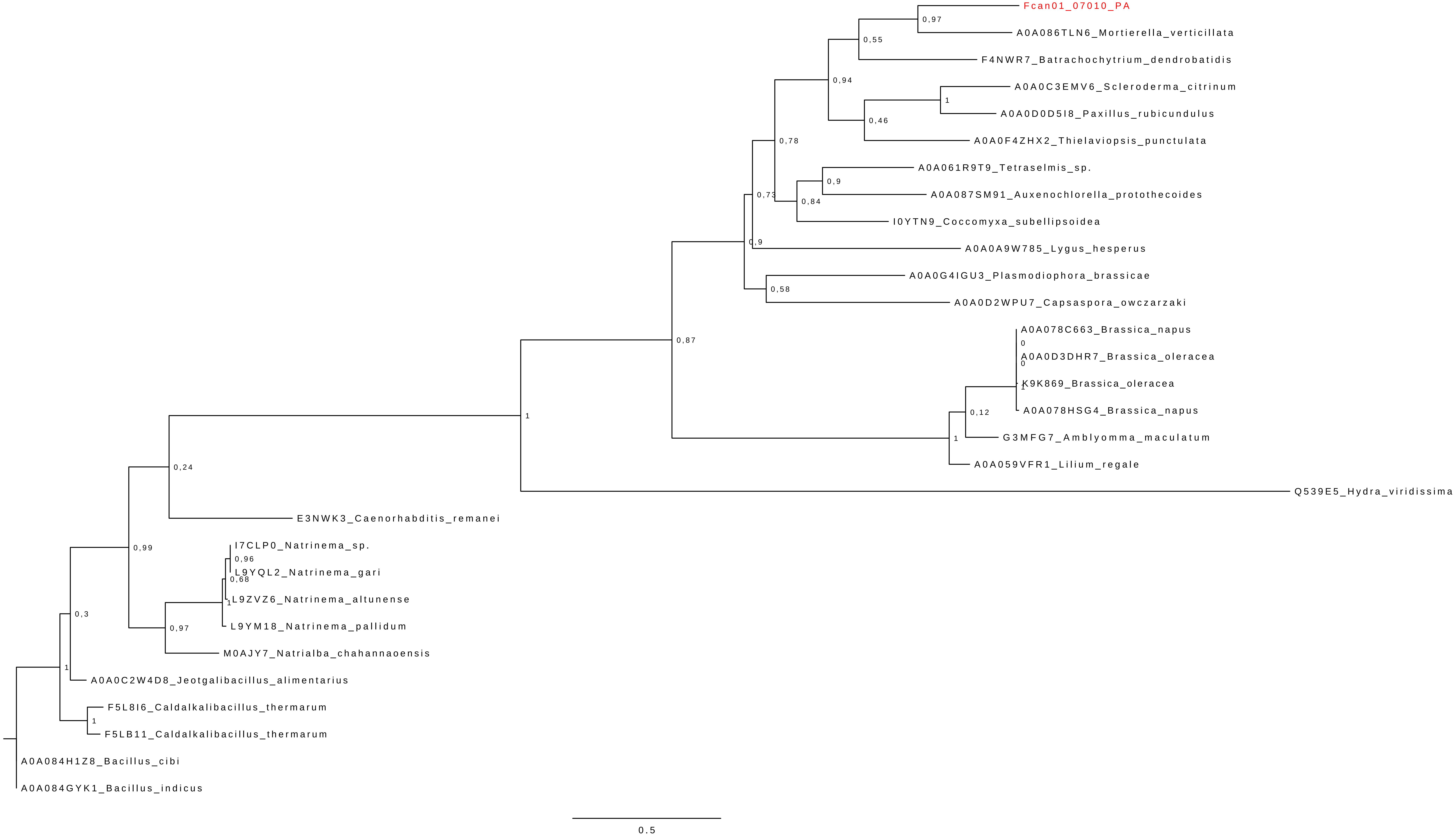

Fcan01_07010_PA
A0A086TLN6_Mortierella_verticillata
F4NWR7_Batrachochytrium_dendrobatidis
A0A0C3EMV6_Scleroderma_citrinum
A0A0D0D5I8_Paxillus_rubicundulus
A0A0F4ZHX2_Thielaviopsis_punctulata
A0A061R9T9_Tetraselmis_sp.
A0A087SM91_Auxenochlorella_protothecoides
I0YTN9_Coccomyxa_subellipsoidea
A0A0A9W785_Lygus_hesperus
A0A0G4IGU3_Plasmodiophora_brassicae
A0A0D2WPU7_Capsaspora_owczarzaki
A0A078C663_Brassica_napus
A0A0D3DHR7_Brassica_oleracea
K9K869_Brassica_oleracea
A0A078HSG4_Brassica_napus
G3MFG7_Amblyomma_maculatum
A0A059VFR1_Lilium_regale
Q539E5_Hydra_viridissima
E3NWK3_Caenorhabditis_remanei
I7CLP0_Natrinema_sp.
L9YQL2_Natrinema_gari
L9ZVZ6_Natrinema_altunense
L9YM18_Natrinema_pallidum
M0AJY7_Natrialba_chahannaoensis
A0A0C2W4D8_Jeotgalibacillus_alimentarius
F5L8I6_Caldalkalibacillus_thermarum
F5LB11_Caldalkalibacillus_thermarum
A0A084H1Z8_Bacillus_cibi
A0A084GYK1_Bacillus_indicus
0.5

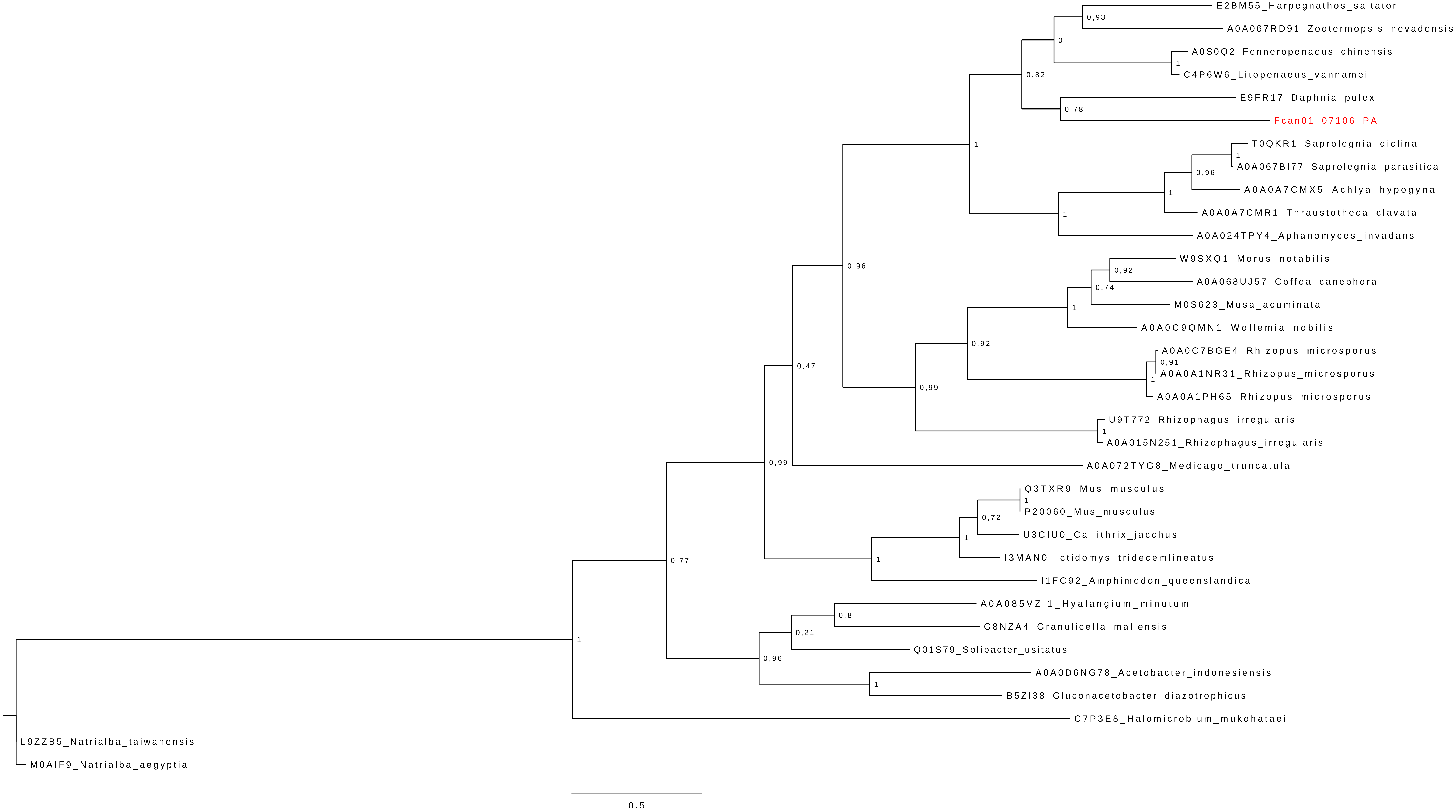
E2BM55_Harpegnathos_saltator
A0A067RD91_Zootermopsis_nevadensis
A0S0Q2_Fenneropenaeus_chinensis
C4P6W6_Litopenaeus_vannamei
E9FR17_Daphnia_pulex
Fcan01_07106_PA
T0QKR1_Saprolegnia_diclina
A0A067BI77_Saprolegnia_parasitica
A0A0A7CMX5_Achlya_hypogyna
A0A0A7CMR1_Thraustotheca_clavata
A0A024TPY4_Aphanomyces_invadans
W9SXQ1_Morus_notabilis
A0A068UJ57_Coffea_canephora
M0S623_Musa_acuminata
A0A0C9QMN1_Wollemia_nobilis
A0A0C7BGE4_Rhizopus_microsporus
A0A0A1NR31_Rhizopus_microsporus
A0A0A1PH65_Rhizopus_microsporus
U9T772_Rhizophagus_irregularis
A0A015N251_Rhizophagus_irregularis
A0A072TYG8_Medicago_truncatula
Q3TXR9_Mus_musculus
P20060_Mus_musculus
U3CIU0_Callithrix_jacchus
I3MAN0_Ictidomys_tridecemlineatus
I1FC92_Amphimedon_queenslandica
A0A085VZI1_Hyalangium_minutum
G8NZA4_Granulicella_mallensis
Q01S79_Solibacter_usitatus
A0A0D6NG78_Acetobacter_indonesiensis
B5ZI38_Gluconacetobacter_diazotrophicus
C7P3E8_Halomicrobium_mukohataei
L9ZZB5_Natrialba_taiwanensis
M0AIF9_Natrialba_aegyptia
0,93
0
1
0,82
0,78
1
1
0,96
1
1
0,96
0,92
0,74
1
0,92
0,91
1
0,99
1
0,47
0,99
1
0,72
1
1
0,77
0,8
0,21
0,96
1
1
0.5

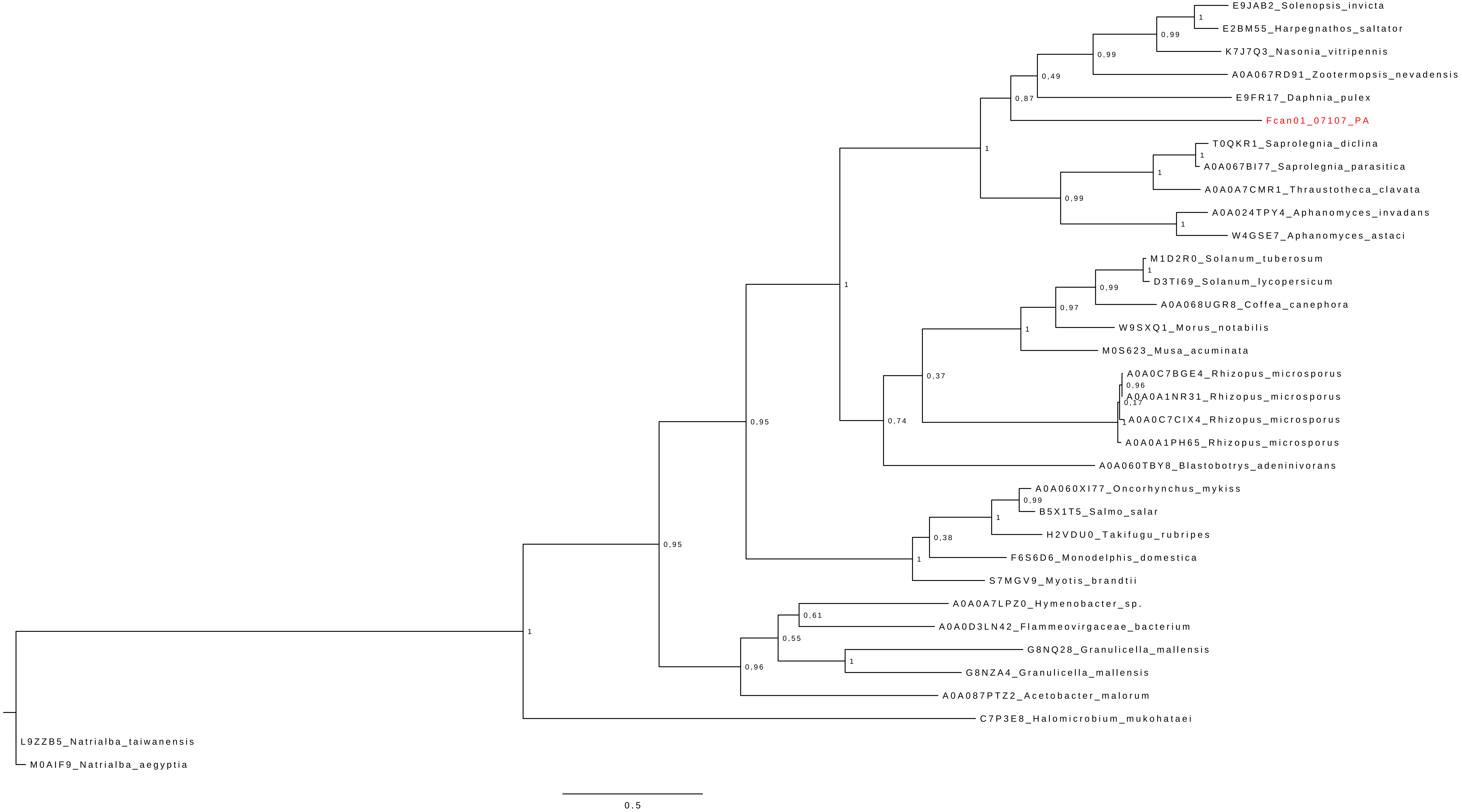
E9JAB2_Solenopsis_invicta
E2BM55_Harpegnathos_saltator
K7J7Q3_Nasonia_vitripennis
A0A067RD91_Zootermopsis_nevadensis
E9FR17_Daphnia_pulex
Fcan01_07107_PA
T0QKR1_Saprolegnia_diclina
A0A067BI77_Saprolegnia_parasitica
A0A0A7CMR1_Thraustotheca_clavata
A0A024TPY4_Aphanomyces_invadans
W4GSE7_Aphanomyces_astaci
M1D2R0_Solanum_tuberosum
D3TI69_Solanum_lycopersicum
A0A068UGR8_Coffea_canephora
W9SXQ1_Morus_notabilis
M0S623_Musa_acuminata
A0A0C7BGE4_Rhizopus_microsporus
A0A0A1NR31_Rhizopus_microsporus
A0A0C7CIX4_Rhizopus_microsporus
A0A0A1PH65_Rhizopus_microsporus
A0A060TBY8_Blastobotrys_adeninivorans
A0A060XI77_Oncorhynchus_mykiss
B5X1T5_Salmo_salar
H2VDU0_Takifugu_rubripes
F6S6D6_Monodelphis_domestica
S7MGV9_Myotis_brandtii
A0A0A7LPZ0_Hymenobacter_sp.
A0A0D3LN42_Flammeovirgaceae_bacterium
G8NQ28_Granulicella_mallensis
G8NZA4_Granulicella_mallensis
A0A087PTZ2_Acetobacter_malorum
C7P3E8_Halomicrobium_mukohataei
L9ZZB5_Natrialba_taiwanensis
M0AIF9_Natrialba_aegyptia
1
0,99
0,99
0,49
0,87
1
1
1
0,99
1
1
0,99
0,97
1
0,96
0,17
1
0,37
0,74
0,99
1
0,38
1
0,95
0,61
0,55
0,96
0,95
1
0.5

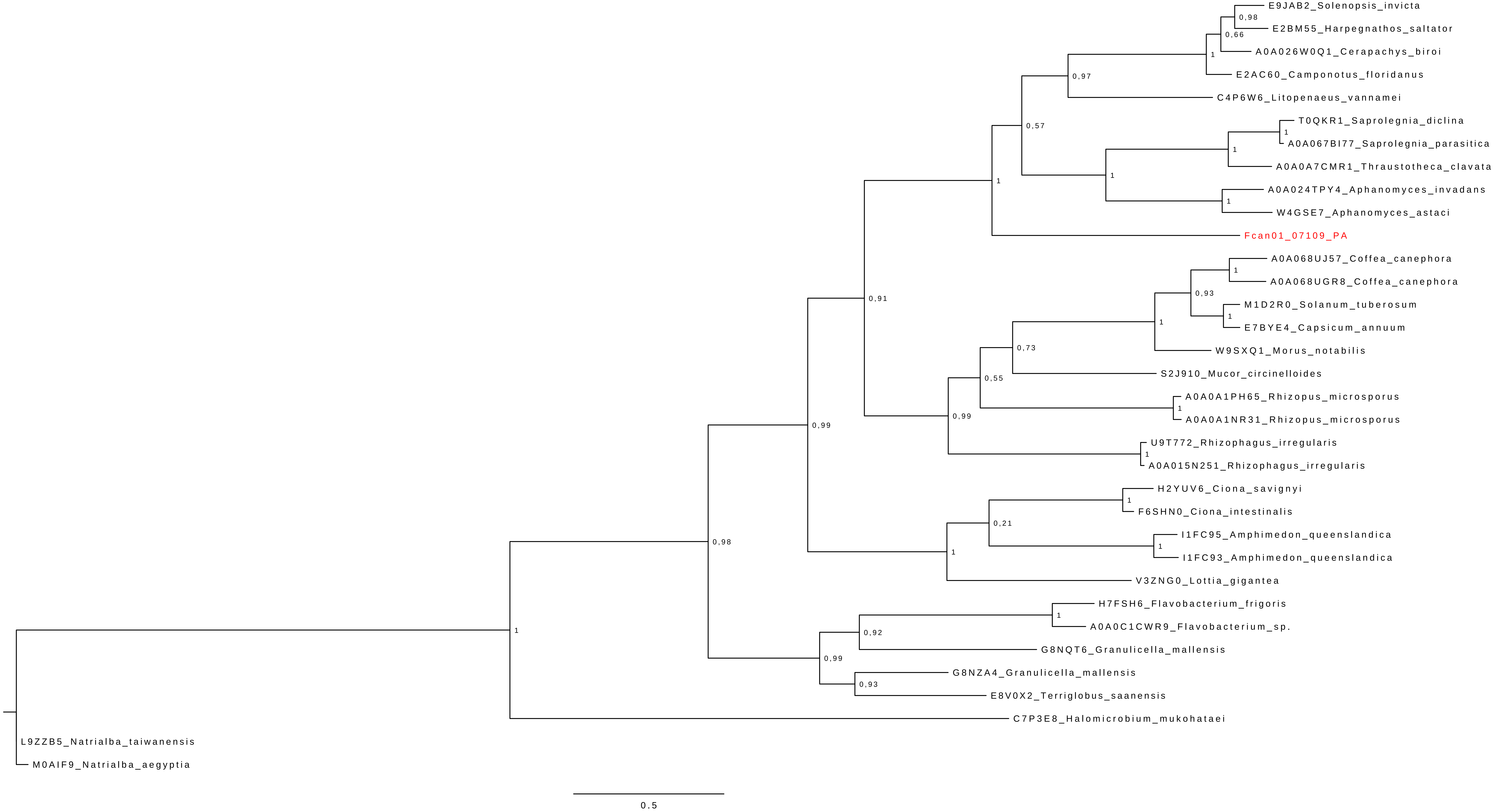
E9JAB2_Solenopsis_invicta
E2BM55_Harpegnathos_saltator
A0A026W0Q1_Cerapachys_biroi
E2AC60_Camponotus_floridanus
C4P6W6_Litopenaeus_vannamei
T0QKR1_Saprolegnia_diclina
A0A067BI77_Saprolegnia_parasitica
A0A0A7CMR1_Thraustotheca_clavata
A0A024TPY4_Aphanomyces_invadans
W4GSE7_Aphanomyces_astaci
Fcan01_07109_PA
A0A068UJ57_Coffea_canephora
A0A068UGR8_Coffea_canephora
M1D2R0_Solanum_tuberosum
E7BYE4_Capsicum_annuum
W9SXQ1_Morus_notabilis
S2J910_Mucor_circinelloides
A0A0A1PH65_Rhizopus_microsporus
A0A0A1NR31_Rhizopus_microsporus
U9T772_Rhizophagus_irregularis
A0A015N251_Rhizophagus_irregularis
H2YUV6_Ciona_savignyi
F6SHN0_Ciona_intestinalis
I1FC95_Amphimedon_queenslandica
I1FC93_Amphimedon_queenslandica
V3ZNG0_Lottia_gigantea
H7FSH6_Flavobacterium_frigoris
A0A0C1CWR9_Flavobacterium_sp.
G8NQT6_Granulicella_mallensis
G8NZA4_Granulicella_mallensis
E8V0X2_Terriglobus_saanensis
C7P3E8_Halomicrobium_mukohataei
L9ZZB5_Natrialba_taiwanensis
M0AIF9_Natrialba_aegyptia
0.5

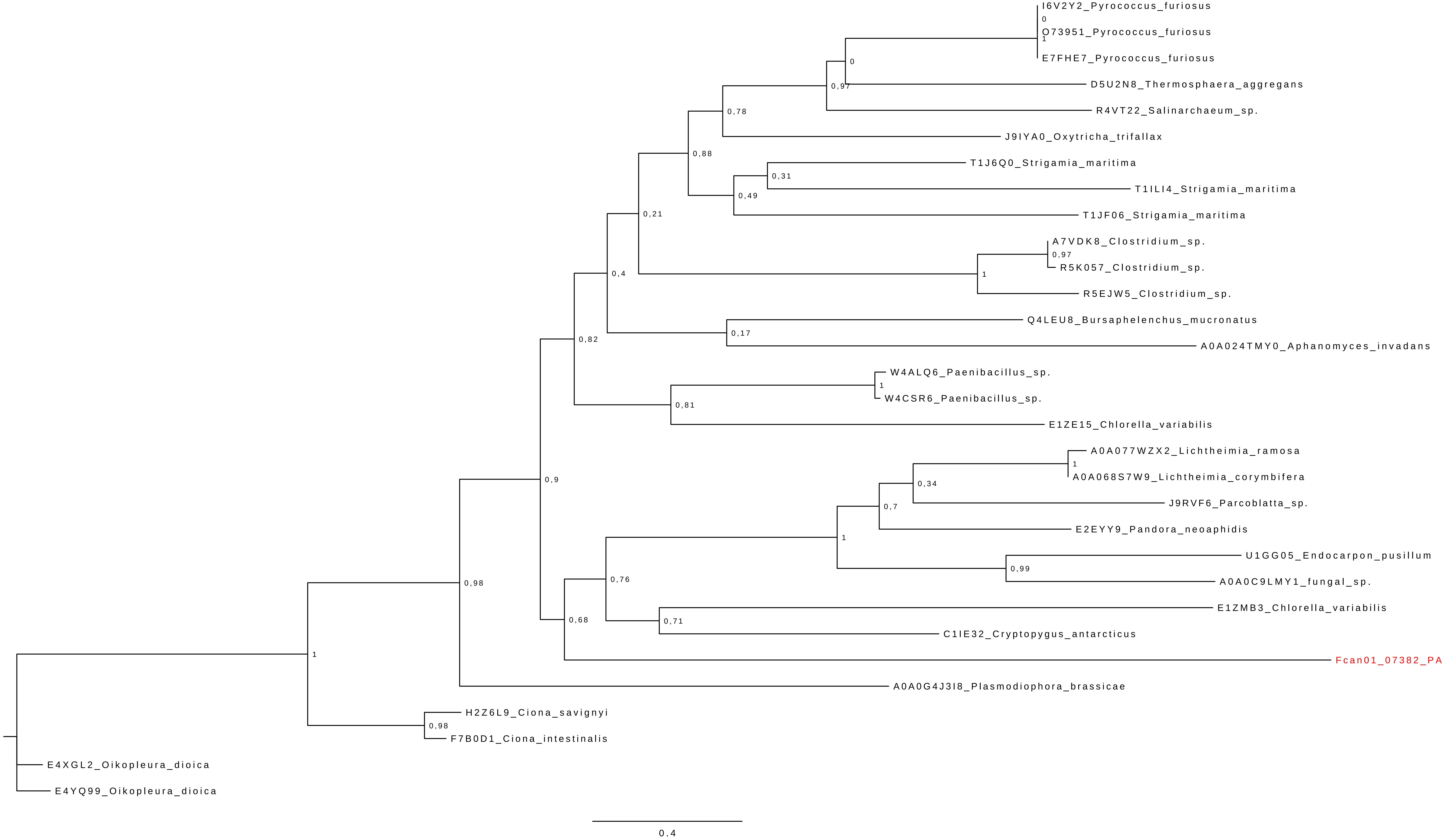
I6V2Y2_Pyrococcus_furiosus
0
O73951_Pyrococcus_furiosus
1
E7FHE7_Pyrococcus_furiosus
0
0,97
D5U2N8_Thermosphaera_aggregans
R4VT22_Salinarchaeum_sp.
0,78
J9IYA0_Oxytricha_trifallax
0,88
T1J6Q0_Strigamia_maritima
0,31
T1ILI4_Strigamia_maritima
0,49
T1JF06_Strigamia_maritima
0,21
A7VDK8_Clostridium_sp.
0,97
R5K057_Clostridium_sp.
1
0,4
R5EJW5_Clostridium_sp.
Q4LEU8_Bursaphelenchus_mucronatus
0,17
A0A024TMY0_Aphanomyces_invadans
0,82
W4ALQ6_Paenibacillus_sp.
1
W4CSR6_Paenibacillus_sp.
0,81
E1ZE15_Chlorella_variabilis
A0A077WZX2_Lichtheimia_ramosa
1
A0A068S7W9_Lichtheimia_corymbifera
0,34
0,9
J9RVF6_Parcoblatta_sp.
0,7
E2EYY9_Pandora_neoaphidis
1
U1GG05_Endocarpon_pusillum
0,99
A0A0C9LMY1_fungal_sp.
0,76
0,98
E1ZMB3_Chlorella_variabilis
0,68
0,71
C1IE32_Cryptopygus_antarcticus
1
Fcan01_07382_PA
A0A0G4J3I8_Plasmodiophora_brassicae
H2Z6L9_Ciona_savignyi
0,98
F7B0D1_Ciona_intestinalis
E4XGL2_Oikopleura_dioica
E4YQ99_Oikopleura_dioica
0.4

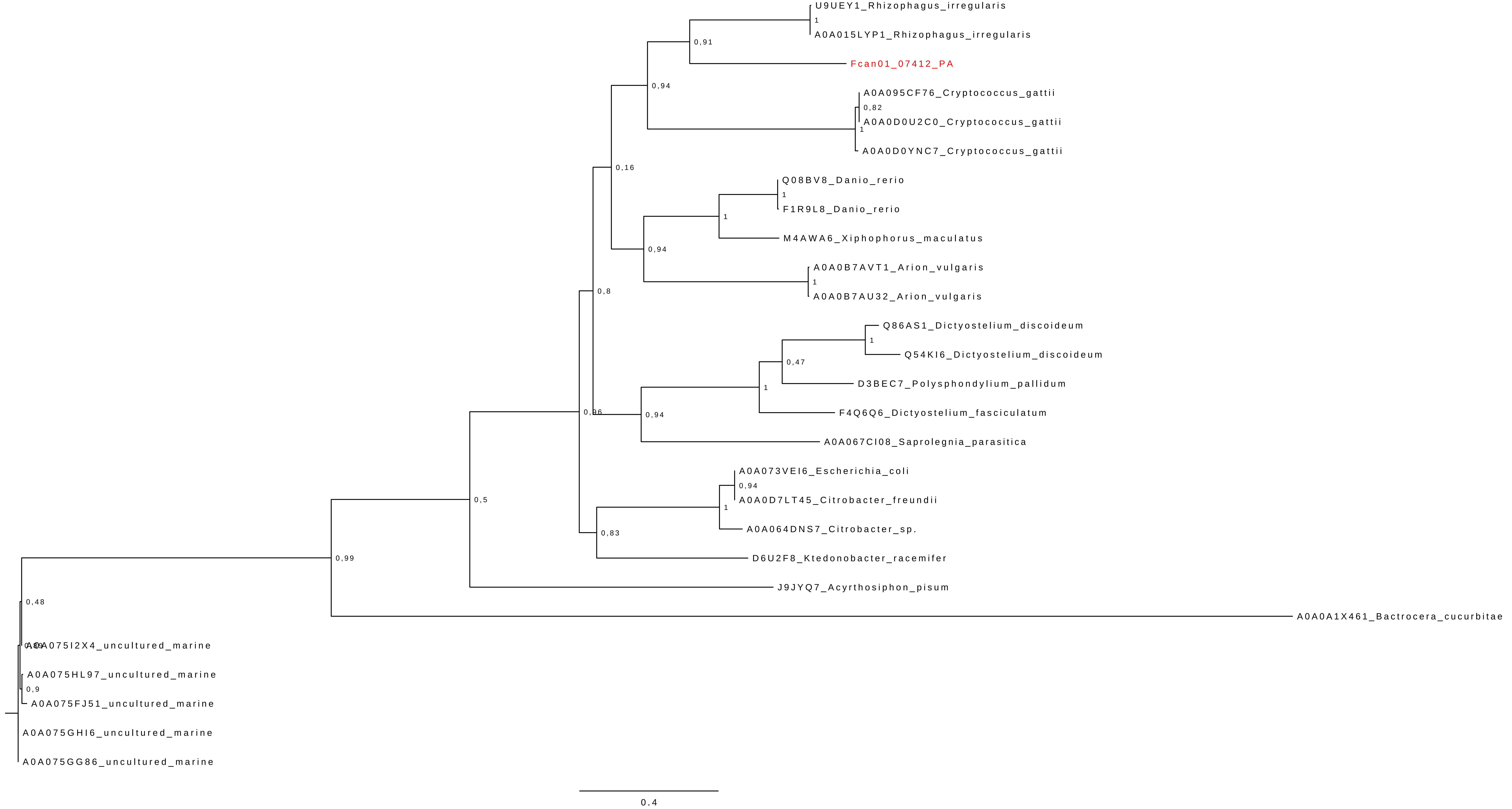
U9UEY1_Rhizophagus_irregularis
1
A0A015LYP1_Rhizophagus_irregularis
0,91
Fcan01_07412_PA
0,94
A0A095CF76_Cryptococcus_gattii
0,82
A0A0D0U2C0_Cryptococcus_gattii
1
A0A0D0YNC7_Cryptococcus_gattii
0,16
Q08BV8_Danio_rerio
1
F1R9L8_Danio_rerio
1
M4AWA6_Xiphophorus_maculatus
0,94
A0A0B7AVT1_Arion_vulgaris
1
A0A0B7AU32_Arion_vulgaris
0,8
Q86AS1_Dictyostelium_discoideum
1
Q54KI6_Dictyostelium_discoideum
0,47
D3BEC7_Polysphondylium_pallidum
1
F4Q6Q6_Dictyostelium_fasciculatum
0,94
A0A067CI08_Saprolegnia_parasitica
A0A073VEI6_Escherichia_coli
0,94
A0A0D7LT45_Citrobacter_freundii
1
A0A064DNS7_Citrobacter_sp.
0,83
D6U2F8_Ktedonobacter_racemifer
0,5
J9JYQ7_Acyrthosiphon_pisum
0,99
A0A0A1X461_Bactrocera_cucurbitae
0,48
A0A075I2X4_uncultured_marine
A0A075HL97_uncultured_marine
0,9
A0A075FJ51_uncultured_marine
A0A075GHI6_uncultured_marine
A0A075GG86_uncultured_marine
0.4

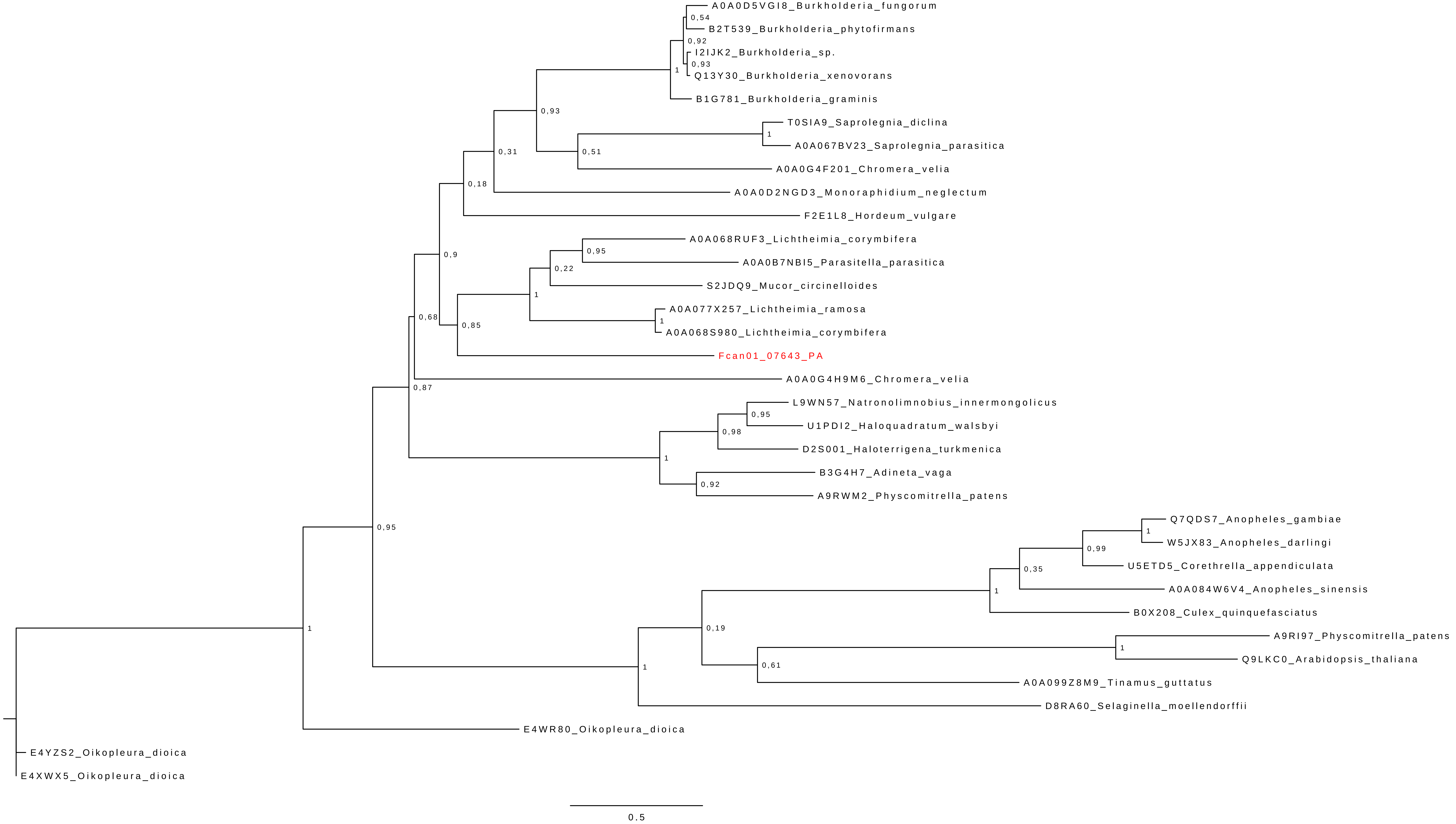
A0A0D5VGI8_Burkholderia_fungorum
0,54
B2T539_Burkholderia_phytofirmans
0,92
I2IJK2_Burkholderia_sp.
0,93
Q13Y30_Burkholderia_xenovorans
1
B1G781_Burkholderia_graminis
0,93
T0SIA9_Saprolegnia_diclina
1
A0A067BV23_Saprolegnia_parasitica
0,51
A0A0G4F201_Chromera_velia
0,31
A0A0D2NGD3_Monoraphidium_neglectum
0,18
F2E1L8_Hordeum_vulgare
A0A068RUF3_Lichtheimia_corymbifera
0,95
A0A0B7NBI5_Parasitella_parasitica
0,22
S2JDQ9_Mucor_circinelloides
0,9
1
A0A077X257_Lichtheimia_ramosa
1
A0A068S980_Lichtheimia_corymbifera
0,68
0,85
Fcan01_07643_PA
A0A0G4H9M6_Chromera_velia
0,87
L9WN57_Natronolimnobius_innermongolicus
0,95
U1PDI2_Haloquadratum_walsbyi
0,98
D2S001_Haloterrigena_turkmenica
1
B3G4H7_Adineta_vaga
0,92
A9RWM2_Physcomitrella_patens
Q7QDS7_Anopheles_gambiae
0,95
1
W5JX83_Anopheles_darlingi
0,99
U5ETD5_Corethrella_appendiculata
0,35
A0A084W6V4_Anopheles_sinensis
1
B0X208_Culex_quinquefasciatus
1
0,19
A9RI97_Physcomitrella_patens
1
Q9LKC0_Arabidopsis_thaliana
0,61
A0A099Z8M9_Tinamus_guttatus
1
D8RA60_Selaginella_moellendorffii
E4WR80_Oikopleura_dioica
E4YZS2_Oikopleura_dioica
E4XWX5_Oikopleura_dioica
0.5

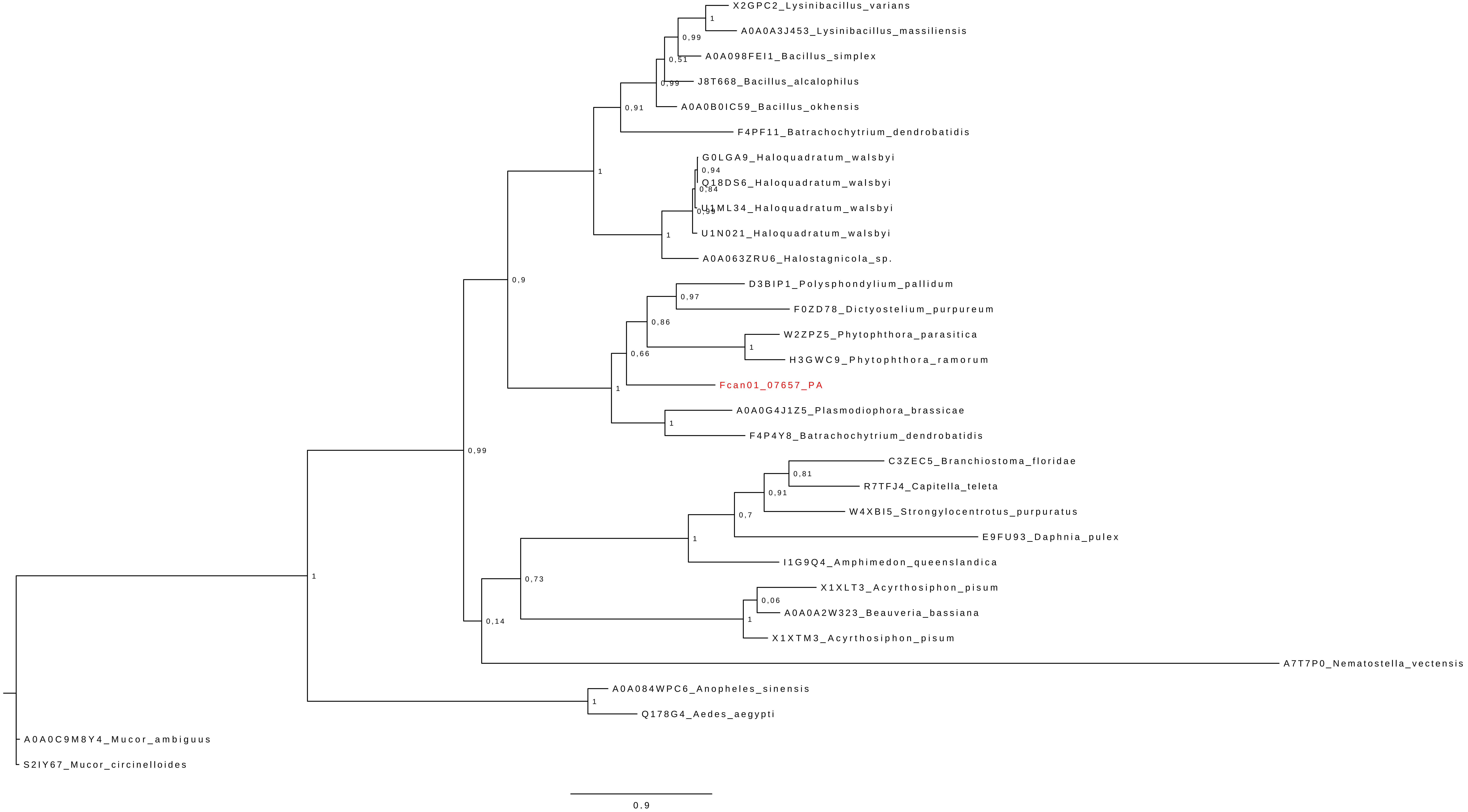

X2GPC2_Lysinibacillus_varians
A0A0A3J453_Lysinibacillus_massiliensis
A0A098FEI1_Bacillus_simplex
J8T668_Bacillus_alcalophilus
A0A0B0IC59_Bacillus_okhensis
F4PF11_Batrachochytrium_dendrobatidis
G0LGA9_Haloquadratum_walsbyi
Q18DS6_Haloquadratum_walsbyi
U1ML34_Haloquadratum_walsbyi
U1N021_Haloquadratum_walsbyi
A0A063ZRU6_Halostagnicola_sp.
D3BIP1_Polysphondylium_pallidum
F0ZD78_Dictyostelium_purpureum
W2ZPZ5_Phytophthora_parasitica
H3GWC9_Phytophthora_ramorum
Fcan01_07657_PA
A0A0G4J1Z5_Plasmodiophora_brassicae
F4P4Y8_Batrachochytrium_dendrobatidis
C3ZEC5_Branchiostoma_floridae
R7TFJ4_Capitella_teleta
W4XBI5_Strongylocentrotus_purpuratus
E9FU93_Daphnia_pulex
I1G9Q4_Amphimedon_queenslandica
X1XLT3_Acyrthosiphon_pisum
A0A0A2W323_Beauveria_bassiana
X1XTM3_Acyrthosiphon_pisum
A7T7P0_Nematostella_vectensis
A0A084WPC6_Anopheles_sinensis
Q178G4_Aedes_aegypti
A0A0C9M8Y4_Mucor_ambiguus
S2IY67_Mucor_circinelloides
1
0,99
0,51
0,99
0,91
1
0,94
0,84
0,99
1
0,9
0,97
0,86
1
0,66
1
1
0,99
0,81
0,91
0,7
1
0,73
0,06
1
0,14
1
1
0.9

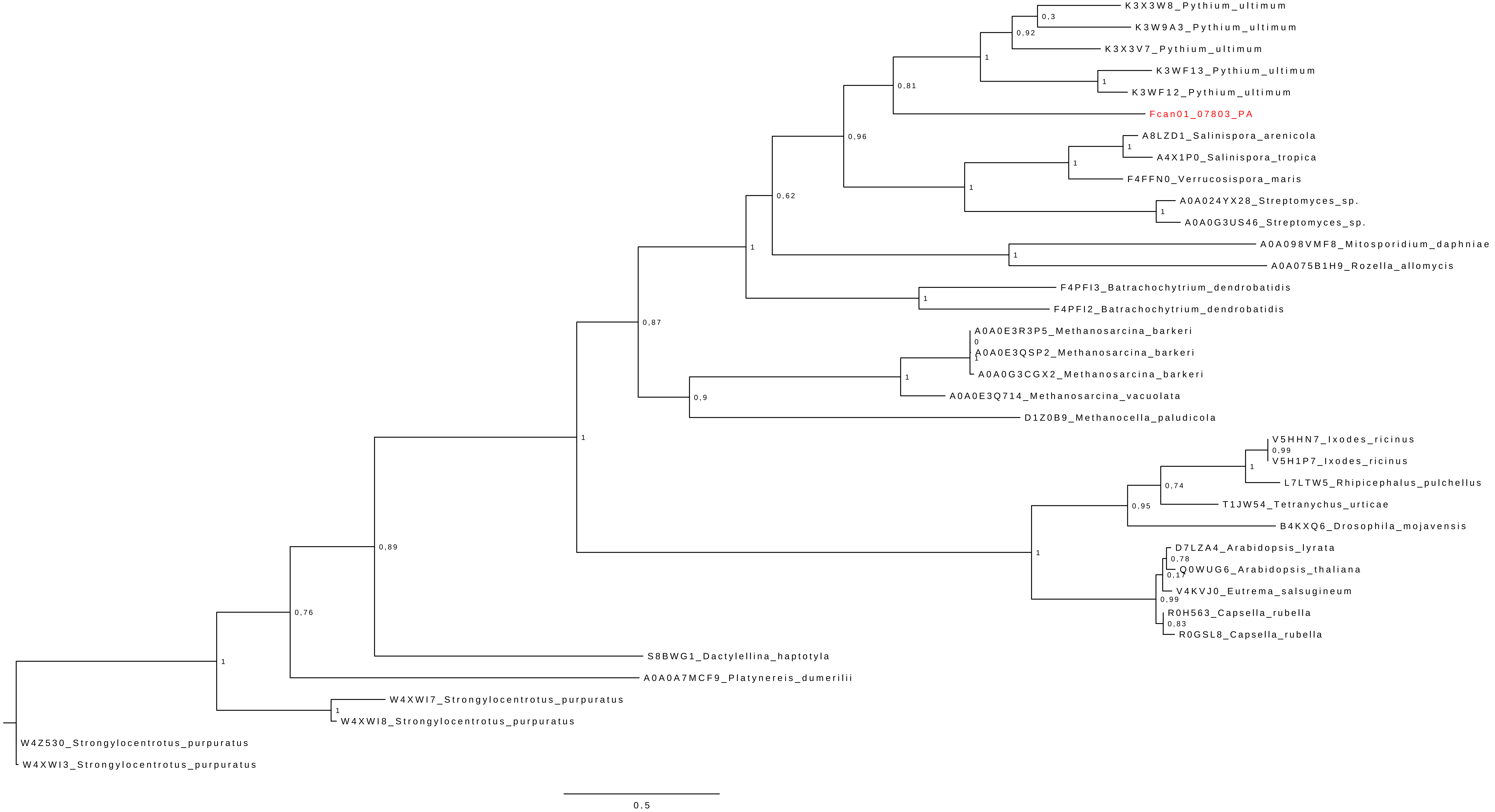

K3X3W8_Pythium_ultimum
K3W9A3_Pythium_ultimum
K3X3V7_Pythium_ultimum
K3WF13_Pythium_ultimum
K3WF12_Pythium_ultimum
Fcan01_07803_PA
A8LZD1_Salinispora_arenicola
A4X1P0_Salinispora_tropica
F4FFN0_Verrucosispora_maris
A0A024YX28_Streptomyces_sp.
A0A0G3US46_Streptomyces_sp.
A0A098VMF8_Mitosporidium_daphniae
A0A075B1H9_Rozella_allomycis
F4PFI3_Batrachochytrium_dendrobatidis
F4PFI2_Batrachochytrium_dendrobatidis
A0A0E3R3P5_Methanosarcina_barkeri
A0A0E3QSP2_Methanosarcina_barkeri
A0A0G3CGX2_Methanosarcina_barkeri
A0A0E3Q714_Methanosarcina_vacuolata
D1Z0B9_Methanocella_paludicola
V5HHN7_Ixodes_ricinus
V5H1P7_Ixodes_ricinus
L7LTW5_Rhipicephalus_pulchellus
T1JW54_Tetranychus_urticae
B4KXQ6_Drosophila_mojavensis
D7LZA4_Arabidopsis_lyrata
Q0WUG6_Arabidopsis_thaliana
V4KVJ0_Eutrema_salsugineum
R0H563_Capsella_rubella
R0GSL8_Capsella_rubella
S8BWG1_Dactylellina_haptotyla
A0A0A7MCF9_Platynereis_dumerilii
W4XWI7_Strongylocentrotus_purpuratus
W4XWI8_Strongylocentrotus_purpuratus
W4Z530_Strongylocentrotus_purpuratus
W4XWI3_Strongylocentrotus_purpuratus
0.5

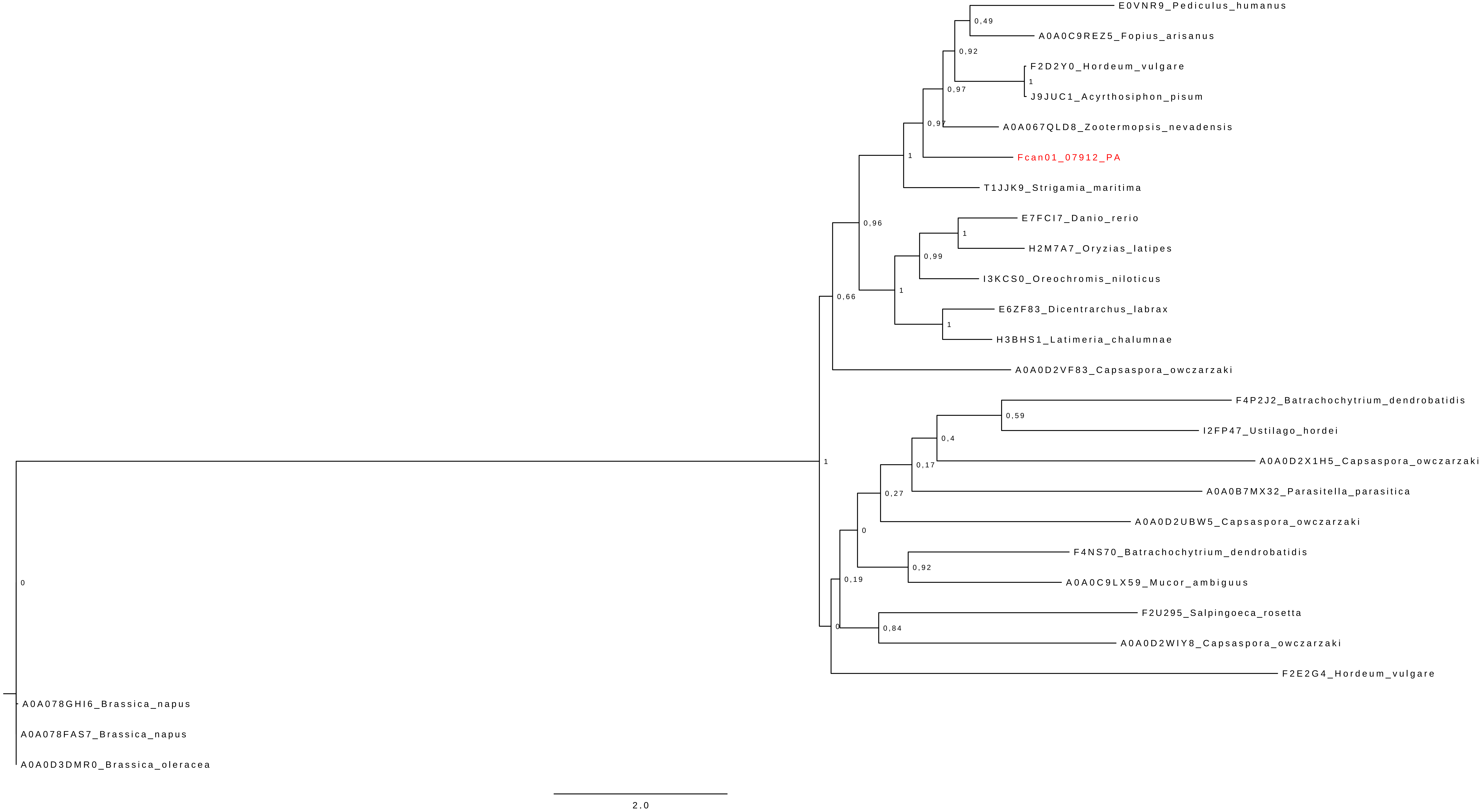
E0VNR9_Pediculus_humanus
0,49
A0A0C9REZ5_Fopius_arisanus
0,92
F2D2Y0_Hordeum_vulgare
1
J9JUC1_Acyrthosiphon_pisum
0,97
A0A067QLD8_Zootermopsis_nevadensis
0,97
Fcan01_07912_PA
1
T1JJK9_Strigamia_maritima
0,96
E7FCI7_Danio_rerio
1
H2M7A7_Oryzias_latipes
0,99
I3KCS0_Oreochromis_niloticus
1
E6ZF83_Dicentrarchus_labrax
1
H3BHS1_Latimeria_chalumnae
0,66
A0A0D2VF83_Capsaspora_owczarzaki
F4P2J2_Batrachochytrium_dendrobatidis
0,59
I2FP47_Ustilago_hordei
0,4
A0A0D2X1H5_Capsaspora_owczarzaki
1
0,17
A0A0B7MX32_Parasitella_parasitica
0,27
A0A0D2UBW5_Capsaspora_owczarzaki
0
F4NS70_Batrachochytrium_dendrobatidis
0,92
0
A0A0C9LX59_Mucor_ambiguus
0,19
F2U295_Salpingoeca_rosetta
0
0,84
A0A0D2WIY8_Capsaspora_owczarzaki
F2E2G4_Hordeum_vulgare
A0A078GHI6_Brassica_napus
A0A078FAS7_Brassica_napus
A0A0D3DMR0_Brassica_oleracea
2.0

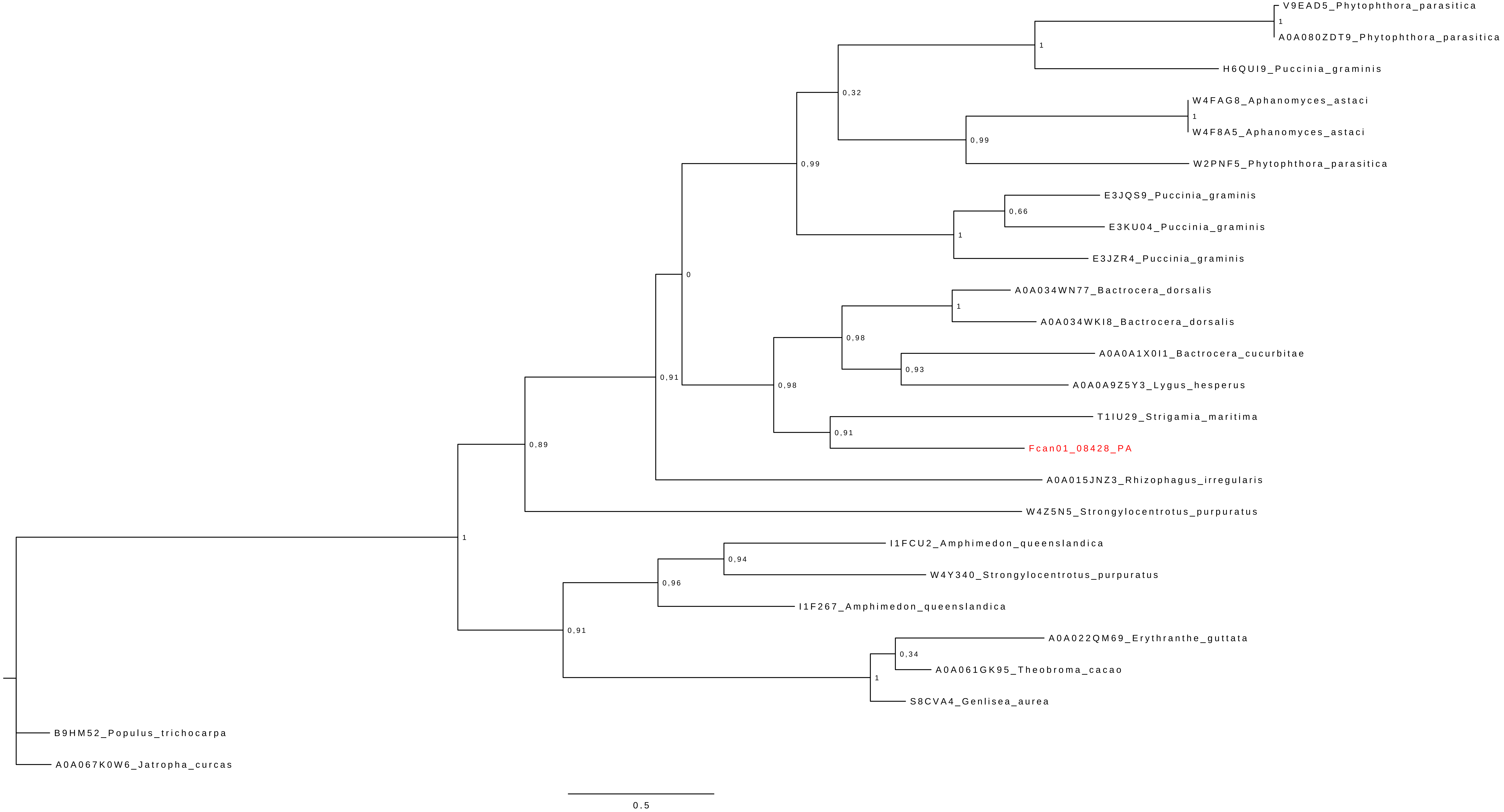
V9EAD5_Phytophthora_parasitica
A0A080ZDT9_Phytophthora_parasitica
H6QUI9_Puccinia_graminis
W4FAG8_Aphanomyces_astaci
W4F8A5_Aphanomyces_astaci
W2PNF5_Phytophthora_parasitica
E3JQS9_Puccinia_graminis
E3KU04_Puccinia_graminis
E3JZR4_Puccinia_graminis
A0A034WN77_Bactrocera_dorsalis
A0A034WKI8_Bactrocera_dorsalis
A0A0A1X0I1_Bactrocera_cucurbitae
A0A0A9Z5Y3_Lygus_hesperus
T1IU29_Strigamia_maritima
Fcan01_08428_PA
A0A015JNZ3_Rhizophagus_irregularis
W4Z5N5_Strongylocentrotus_purpuratus
I1FCU2_Amphimedon_queenslandica
W4Y340_Strongylocentrotus_purpuratus
I1F267_Amphimedon_queenslandica
A0A022QM69_Erythranthe_guttata
A0A061GK95_Theobroma_cacao
S8CVA4_Genlisea_aurea
B9HM52_Populus_trichocarpa
A0A067K0W6_Jatropha_curcas
1
1
0,32
1
0,99
0,99
0,66
1
0
1
0,98
0,93
0,91
0,98
0,91
0,89
1
0,94
0,96
0,91
0,34
1
0.5

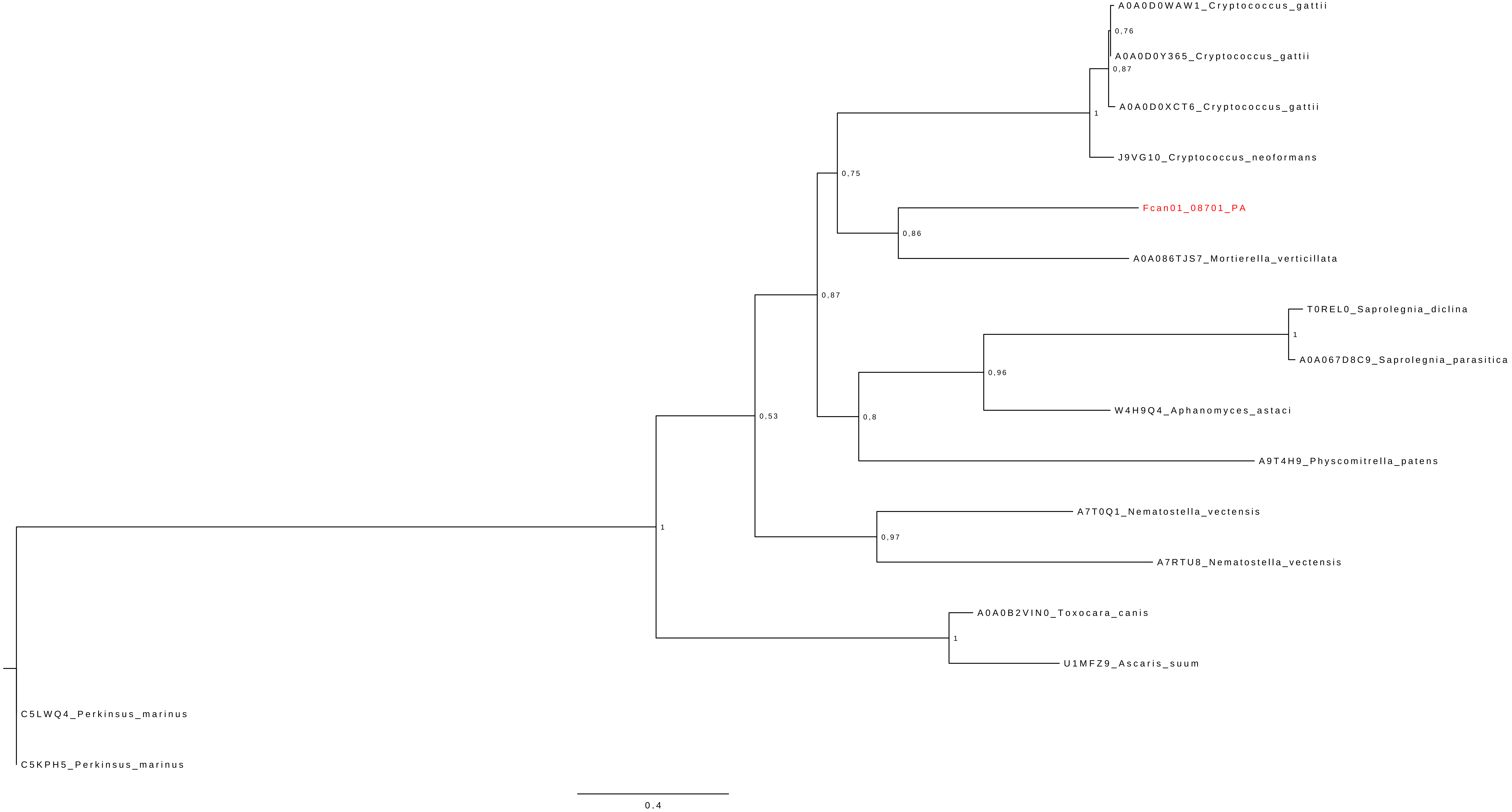
A0A0D0WAW1_Cryptococcus_gattii
0,76
A0A0D0Y365_Cryptococcus_gattii
0,87
A0A0D0XCT6_Cryptococcus_gattii
1
J9VG10_Cryptococcus_neoformans
0,75
Fcan01_08701_PA
0,86
A0A086TJS7_Mortierella_verticillata
0,87
T0REL0_Saprolegnia_diclina
1
A0A067D8C9_Saprolegnia_parasitica
0,96
W4H9Q4_Aphanomyces_astaci
0,53
0,8
A9T4H9_Physcomitrella_patens
A7T0Q1_Nematostella_vectensis
1
0,97
A7RTU8_Nematostella_vectensis
A0A0B2VIN0_Toxocara_canis
1
U1MFZ9_Ascaris_suum
C5LWQ4_Perkinsus_marinus
C5KPH5_Perkinsus_marinus
0.4

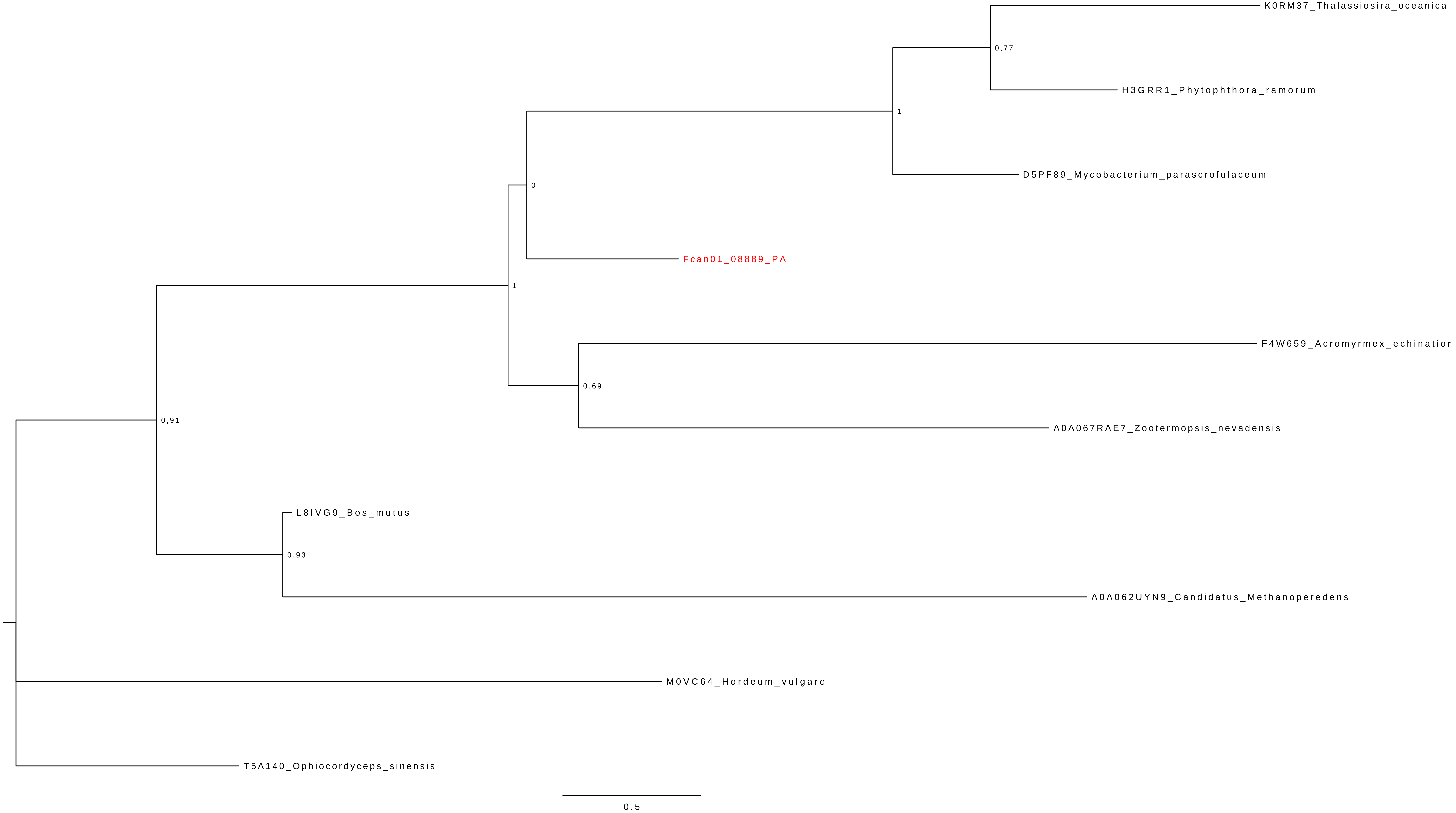
K0RM37_Thalassiosira_oceanica
0,77
H3GRR1_Phytophthora_ramorum
1
D5PF89_Mycobacterium_parascrofulaceum
0
Fcan01_08889_PA
1
F4W659_Acromyrmex_echinatior
0,69
A0A067RAE7_Zootermopsis_nevadensis
0,91
L8IVG9_Bos_mutus
0,93
A0A062UYN9_Candidatus_Methanoperedens
M0VC64_Hordeum_vulgare
T5A140_Ophiocordyceps_sinensis
0.5

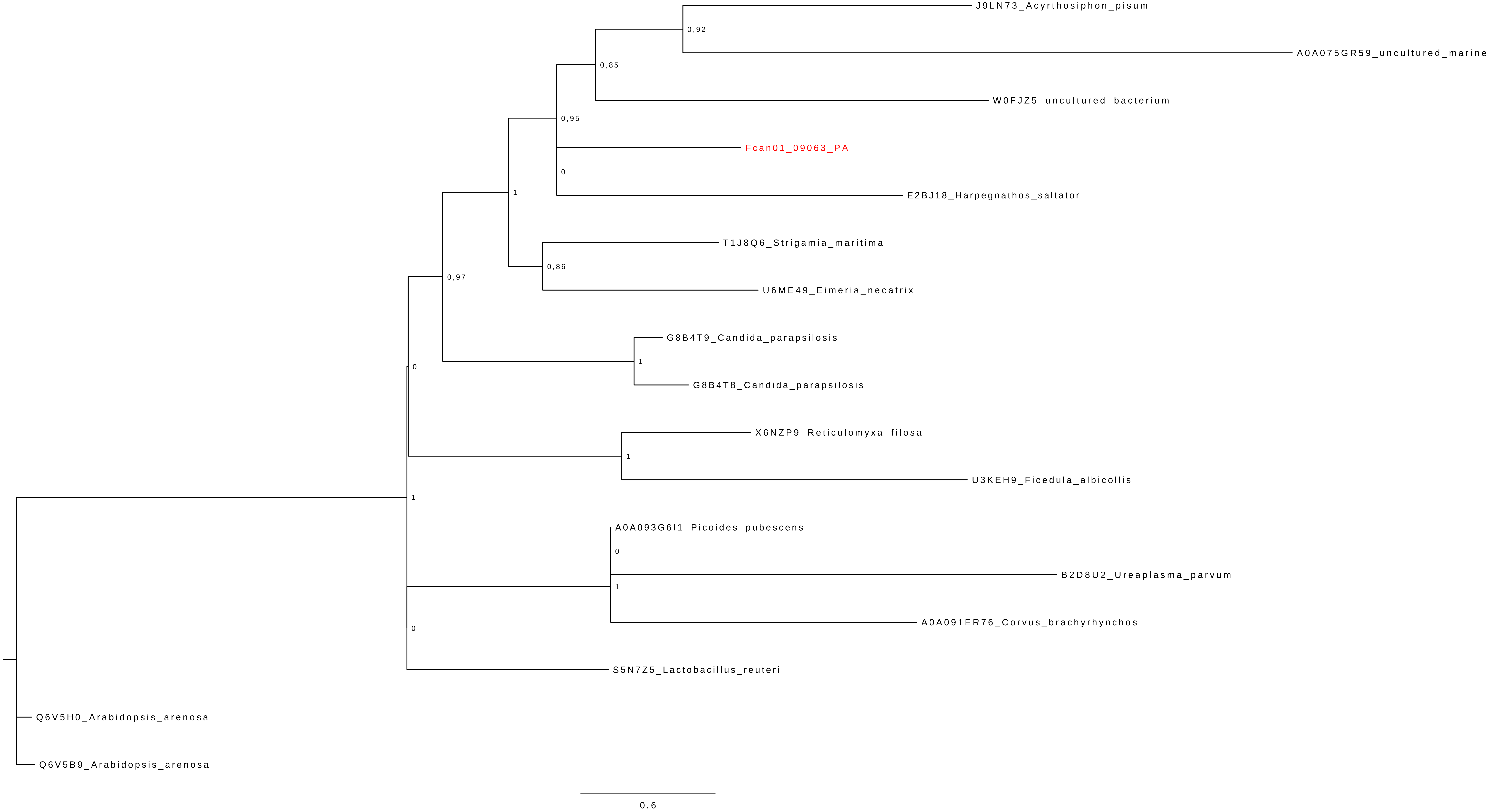
J9LN73_Acyrthosiphon_pisum
0,92
A0A075GR59_uncultured_marine
0,85
W0FJZ5_uncultured_bacterium
0,95
Fcan01_09063_PA
0
E2BJ18_Harpegnathos_saltator
1
T1J8Q6_Strigamia_maritima
0,86
U6ME49_Eimeria_necatrix
0,97
G8B4T9_Candida_parapsilosis
1
G8B4T8_Candida_parapsilosis
0
X6NZP9_Reticulomyxa_filosa
1
U3KEH9_Ficedula_albicollis
1
A0A093G6I1_Picoides_pubescens
0
B2D8U2_Ureaplasma_parvum
1
A0A091ER76_Corvus_brachyrhynchos
0
S5N7Z5_Lactobacillus_reuteri
Q6V5H0_Arabidopsis_arenosa
Q6V5B9_Arabidopsis_arenosa
0.6

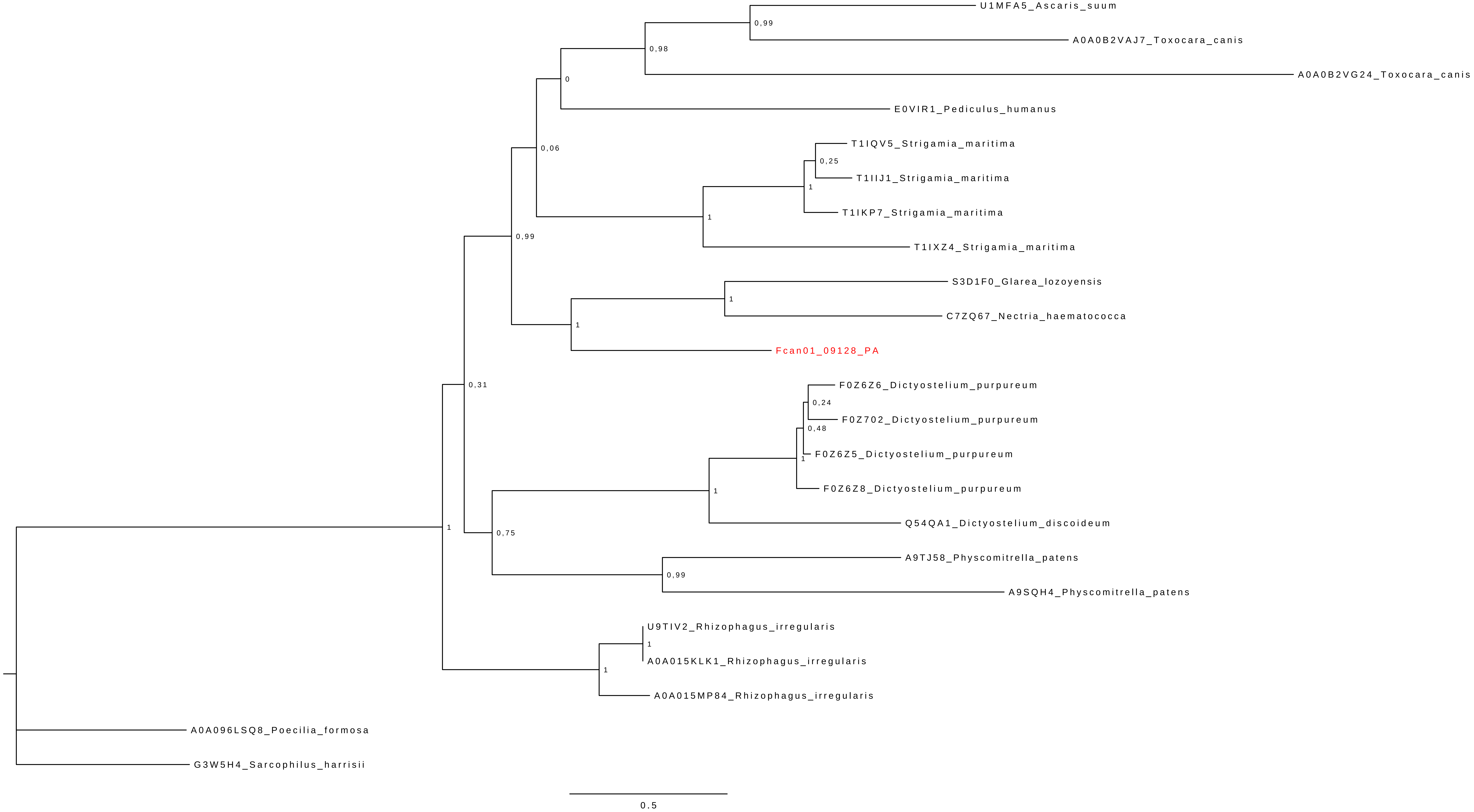
U1MFA5_Ascaris_suum
0,99
A0A0B2VAJ7_Toxocara_canis
0,98
A0A0B2VG24_Toxocara_canis
0
E0VIR1_Pediculus_humanus
T1IQV5_Strigamia_maritima
0,06
0,25
T1IIJ1_Strigamia_maritima
1
T1IKP7_Strigamia_maritima
1
0,99
T1IXZ4_Strigamia_maritima
S3D1F0_Glarea_lozoyensis
1
C7ZQ67_Nectria_haematococca
1
Fcan01_09128_PA
F0Z6Z6_Dictyostelium_purpureum
0,31
0,24
F0Z702_Dictyostelium_purpureum
0,48
F0Z6Z5_Dictyostelium_purpureum
1
F0Z6Z8_Dictyostelium_purpureum
1
Q54QA1_Dictyostelium_discoideum
1
0,75
A9TJ58_Physcomitrella_patens
0,99
A9SQH4_Physcomitrella_patens
U9TIV2_Rhizophagus_irregularis
1
A0A015KLK1_Rhizophagus_irregularis
1
A0A015MP84_Rhizophagus_irregularis
A0A096LSQ8_Poecilia_formosa
G3W5H4_Sarcophilus_harrisii
0.5

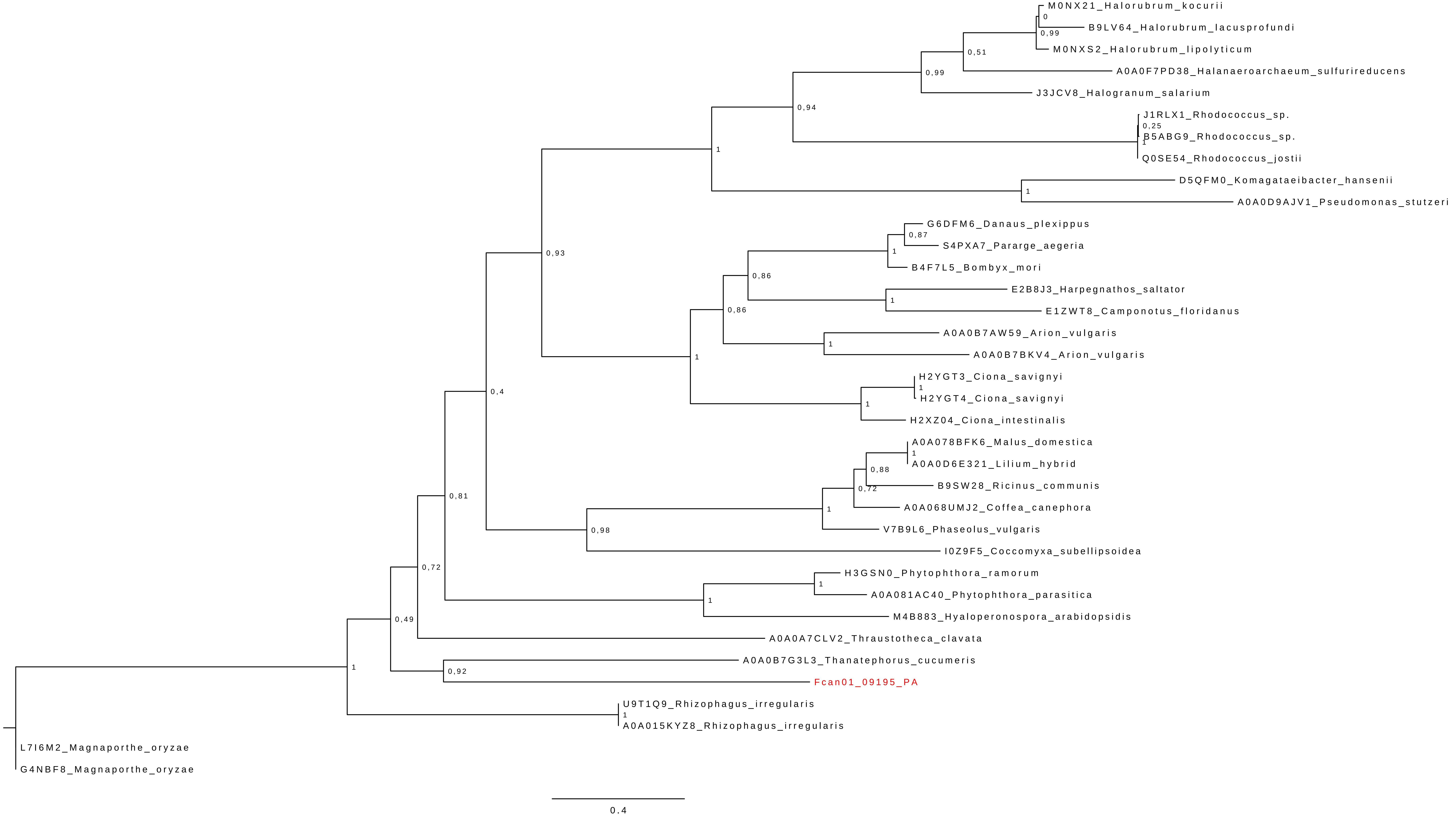
M0NX21_Halorubrum_kocurii
0
B9LV64_Halorubrum_lacusprofundi
0,99
M0NXS2_Halorubrum_lipolyticum
0,51
A0A0F7PD38_Halanaeroarchaeum_sulfurireducens
0,99
J3JCV8_Halogranum_salarium
0,94
J1RLX1_Rhodococcus_sp.
0,25
B5ABG9_Rhodococcus_sp.
1
Q0SE54_Rhodococcus_jostii
1
D5QFM0_Komagataeibacter_hansenii
1
A0A0D9AJV1_Pseudomonas_stutzeri
G6DFM6_Danaus_plexippus
0,87
S4PXA7_Pararge_aegeria
1
B4F7L5_Bombyx_mori
0,93
0,86
E2B8J3_Harpegnathos_saltator
1
E1ZWT8_Camponotus_floridanus
0,86
A0A0B7AW59_Arion_vulgaris
1
A0A0B7BKV4_Arion_vulgaris
1
H2YGT3_Ciona_savignyi
1
H2YGT4_Ciona_savignyi
0,4
1
H2XZ04_Ciona_intestinalis
A0A078BFK6_Malus_domestica
1
A0A0D6E321_Lilium_hybrid
0,88
B9SW28_Ricinus_communis
0,72
0,81
A0A068UMJ2_Coffea_canephora
1
V7B9L6_Phaseolus_vulgaris
0,98
I0Z9F5_Coccomyxa_subellipsoidea
0,72
H3GSN0_Phytophthora_ramorum
1
A0A081AC40_Phytophthora_parasitica
1
M4B883_Hyaloperonospora_arabidopsidis
0,49
A0A0A7CLV2_Thraustotheca_clavata
A0A0B7G3L3_Thanatephorus_cucumeris
1
0,92
Fcan01_09195_PA
U9T1Q9_Rhizophagus_irregularis
1
A0A015KYZ8_Rhizophagus_irregularis
L7I6M2_Magnaporthe_oryzae
G4NBF8_Magnaporthe_oryzae
0.4

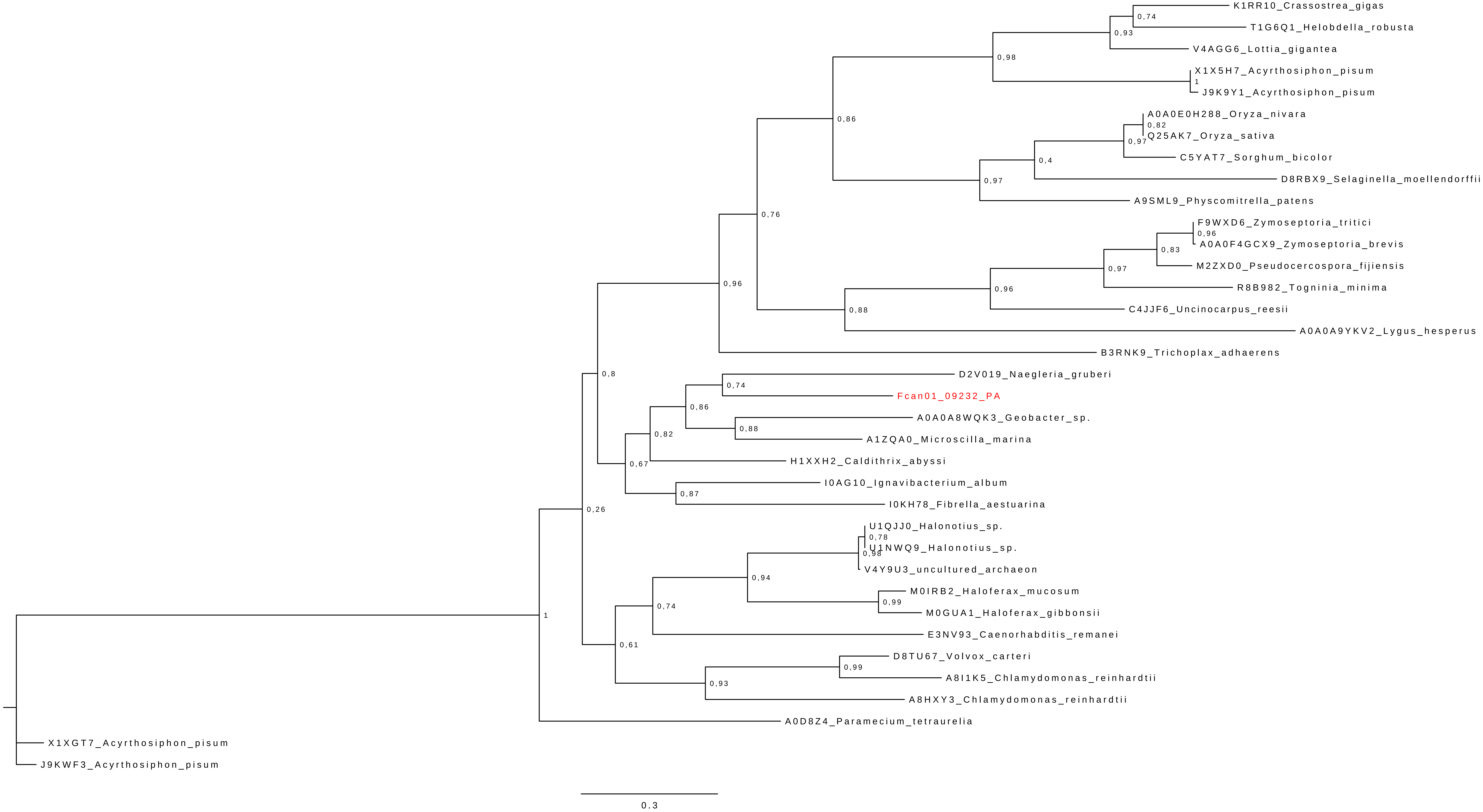
K1RR10_Crassostrea_gigas
0,74
T1G6Q1_Helobdella_robusta
0,93
V4AGG6_Lottia_gigantea
0,98
X1X5H7_Acyrthosiphon_pisum
1
J9K9Y1_Acyrthosiphon_pisum
A0A0E0H288_Oryza_nivara
0,82
Q25AK7_Oryza_sativa
0,97
C5YAT7_Sorghum_bicolor
0,4
D8RBX9_Selaginella_moellendorffii
0,86
0,97
A9SML9_Physcomitrella_patens
0,76
F9WXD6_Zymoseptoria_tritici
0,96
A0A0F4GCX9_Zymoseptoria_brevis
0,83
M2ZXD0_Pseudocercospora_fijiensis
0,97
R8B982_Togninia_minima
0,96
0,96
C4JJF6_Uncinocarpus_reesii
0,88
A0A0A9YKV2_Lygus_hesperus
B3RNK9_Trichoplax_adhaerens
D2V019_Naegleria_gruberi
0,8
0,74
Fcan01_09232_PA
0,86
A0A0A8WQK3_Geobacter_sp.
0,88
A1ZQA0_Microscilla_marina
0,82
H1XXH2_Caldithrix_abyssi
0,67
I0AG10_Ignavibacterium_album
0,87
I0KH78_Fibrella_aestuarina
0,26
U1QJJ0_Halonotius_sp.
0,78
U1NWQ9_Halonotius_sp.
0,98
V4Y9U3_uncultured_archaeon
0,94
M0IRB2_Haloferax_mucosum
0,99
M0GUA1_Haloferax_gibbonsii
0,74
1
E3NV93_Caenorhabditis_remanei
0,61
D8TU67_Volvox_carteri
0,99
A8I1K5_Chlamydomonas_reinhardtii
0,93
A8HXY3_Chlamydomonas_reinhardtii
A0D8Z4_Paramecium_tetraurelia
X1XGT7_Acyrthosiphon_pisum
J9KWF3_Acyrthosiphon_pisum
0.3

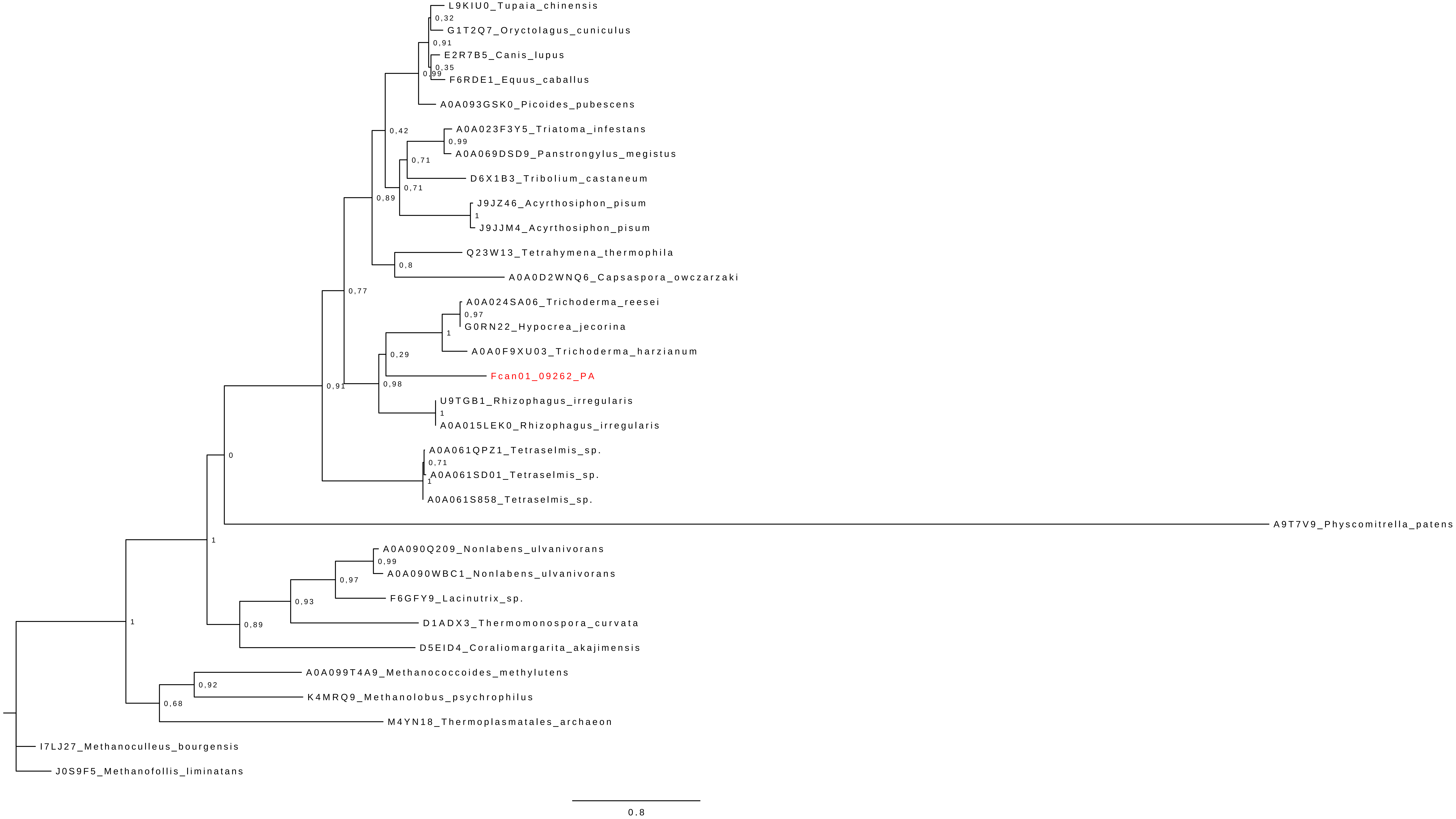

L9KIU0_Tupaia_chinensis
G1T2Q7_Oryctolagus_cuniculus
E2R7B5_Canis_lupus
F6RDE1_Equus_caballus
A0A093GSK0_Picoides_pubescens
A0A023F3Y5_Triatoma_infestans
A0A069DSD9_Panstrongylus_megistus
D6X1B3_Tribolium_castaneum
J9JZ46_Acyrthosiphon_pisum
J9JJM4_Acyrthosiphon_pisum
Q23W13_Tetrahymena_thermophila
A0A0D2WNQ6_Capsaspora_owczarzaki
A0A024SA06_Trichoderma_reesei
G0RN22_Hypocrea_jecorina
A0A0F9XU03_Trichoderma_harzianum
Fcan01_09262_PA
U9TGB1_Rhizophagus_irregularis
A0A015LEK0_Rhizophagus_irregularis
A0A061QPZ1_Tetraselmis_sp.
A0A061SD01_Tetraselmis_sp.
A0A061S858_Tetraselmis_sp.
A9T7V9_Physcomitrella_patens
A0A090Q209_Nonlabens_ulvanivorans
A0A090WBC1_Nonlabens_ulvanivorans
F6GFY9_Lacinutrix_sp.
D1ADX3_Thermomonospora_curvata
D5EID4_Coraliomargarita_akajimensis
A0A099T4A9_Methanococcoides_methylutens
K4MRQ9_Methanolobus_psychrophilus
M4YN18_Thermoplasmatales_archaeon
I7LJ27_Methanoculleus_bourgensis
J0S9F5_Methanofollis_liminatans
0.8

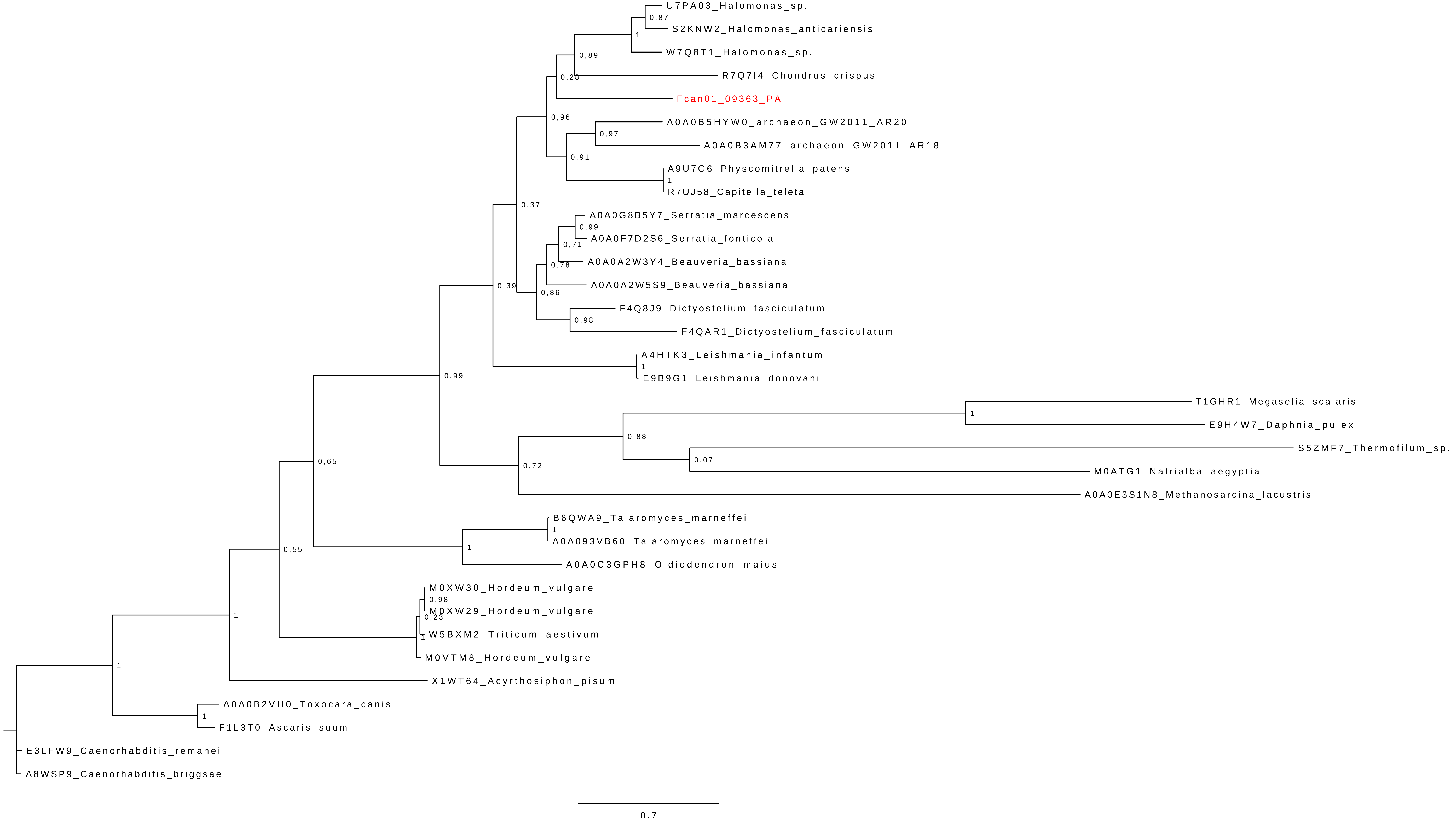
U7PA03_Halomonas_sp.
S2KNW2_Halomonas_anticariensis
W7Q8T1_Halomonas_sp.
R7Q7I4_Chondrus_crispus
Fcan01_09363_PA
A0A0B5HYW0_archaeon_GW2011_AR20
A0A0B3AM77_archaeon_GW2011_AR18
A9U7G6_Physcomitrella_patens
R7UJ58_Capitella_teleta
A0A0G8B5Y7_Serratia_marcescens
A0A0F7D2S6_Serratia_fonticola
A0A0A2W3Y4_Beauveria_bassiana
A0A0A2W5S9_Beauveria_bassiana
F4Q8J9_Dictyostelium_fasciculatum
F4QAR1_Dictyostelium_fasciculatum
A4HTK3_Leishmania_infantum
E9B9G1_Leishmania_donovani
T1GHR1_Megaselia_scalaris
E9H4W7_Daphnia_pulex
S5ZMF7_Thermofilum_sp.
M0ATG1_Natrialba_aegyptia
A0A0E3S1N8_Methanosarcina_lacustris
B6QWA9_Talaromyces_marneffei
A0A093VB60_Talaromyces_marneffei
A0A0C3GPH8_Oidiodendron_maius
M0XW30_Hordeum_vulgare
M0XW29_Hordeum_vulgare
W5BXM2_Triticum_aestivum
M0VTM8_Hordeum_vulgare
X1WT64_Acyrthosiphon_pisum
A0A0B2VII0_Toxocara_canis
F1L3T0_Ascaris_suum
E3LFW9_Caenorhabditis_remanei
A8WSP9_Caenorhabditis_briggsae
0.7

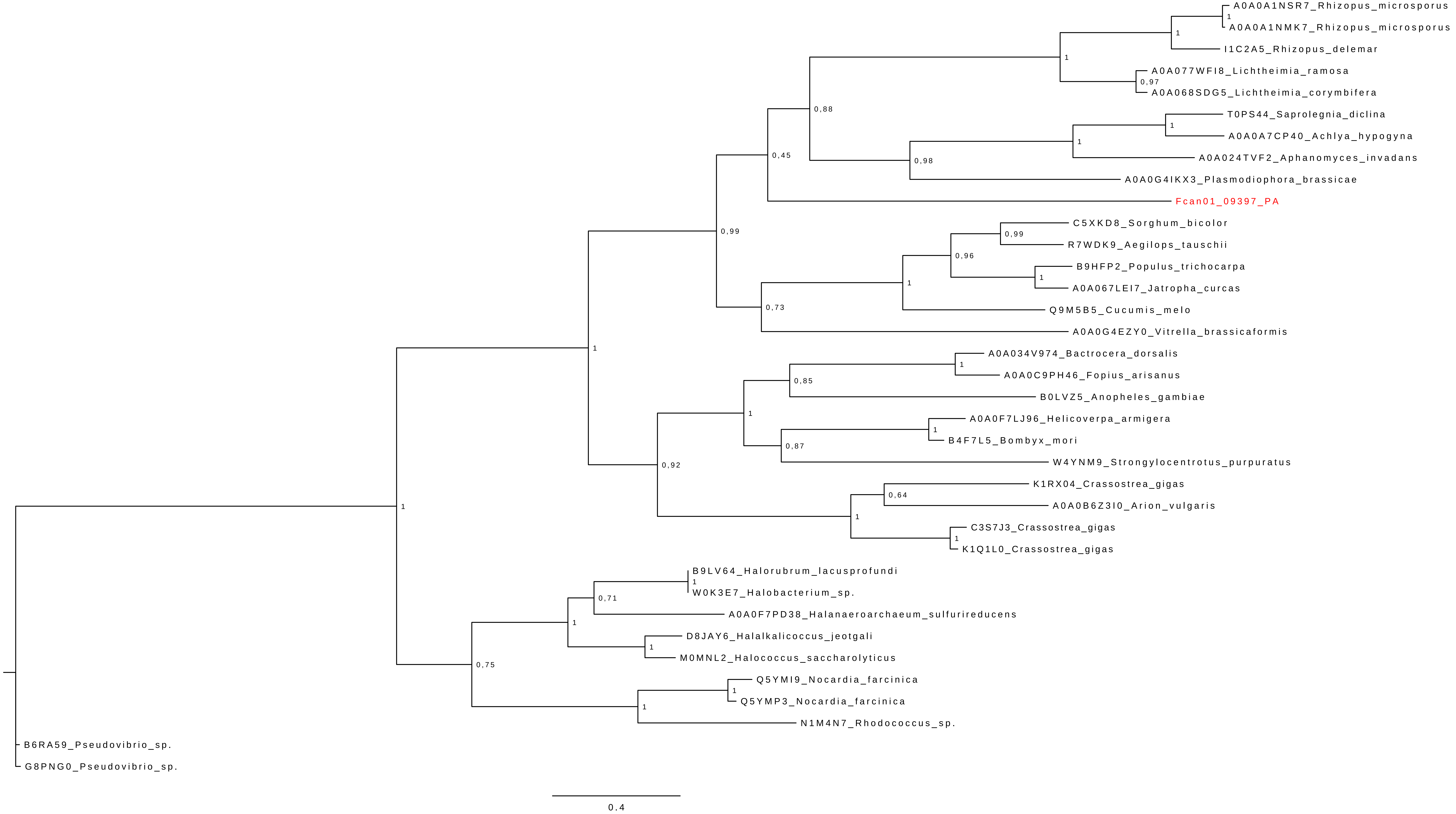
A0A0A1NSR7_Rhizopus_microsporus
A0A0A1NMK7_Rhizopus_microsporus
I1C2A5_Rhizopus_delemar
A0A077WFI8_Lichtheimia_ramosa
A0A068SDG5_Lichtheimia_corymbifera
T0PS44_Saprolegnia_diclina
A0A0A7CP40_Achlya_hypogyna
A0A024TVF2_Aphanomyces_invadans
A0A0G4IKX3_Plasmodiophora_brassicae
Fcan01_09397_PA
C5XKD8_Sorghum_bicolor
R7WDK9_Aegilops_tauschii
B9HFP2_Populus_trichocarpa
A0A067LEI7_Jatropha_curcas
Q9M5B5_Cucumis_melo
A0A0G4EZY0_Vitrella_brassicaformis
A0A034V974_Bactrocera_dorsalis
A0A0C9PH46_Fopius_arisanus
B0LVZ5_Anopheles_gambiae
A0A0F7LJ96_Helicoverpa_armigera
B4F7L5_Bombyx_mori
W4YNM9_Strongylocentrotus_purpuratus
K1RX04_Crassostrea_gigas
A0A0B6Z3I0_Arion_vulgaris
C3S7J3_Crassostrea_gigas
K1Q1L0_Crassostrea_gigas
B9LV64_Halorubrum_lacusprofundi
W0K3E7_Halobacterium_sp.
A0A0F7PD38_Halanaeroarchaeum_sulfurireducens
D8JAY6_Halalkalicoccus_jeotgali
M0MNL2_Halococcus_saccharolyticus
Q5YMI9_Nocardia_farcinica
Q5YMP3_Nocardia_farcinica
N1M4N7_Rhodococcus_sp.
B6RA59_Pseudovibrio_sp.
G8PNG0_Pseudovibrio_sp.
0,88
0,45
0,97
0,98
0,99
0,96
0,73
0,85
0,87
0,92
0,64
0,71
0,75
0.4

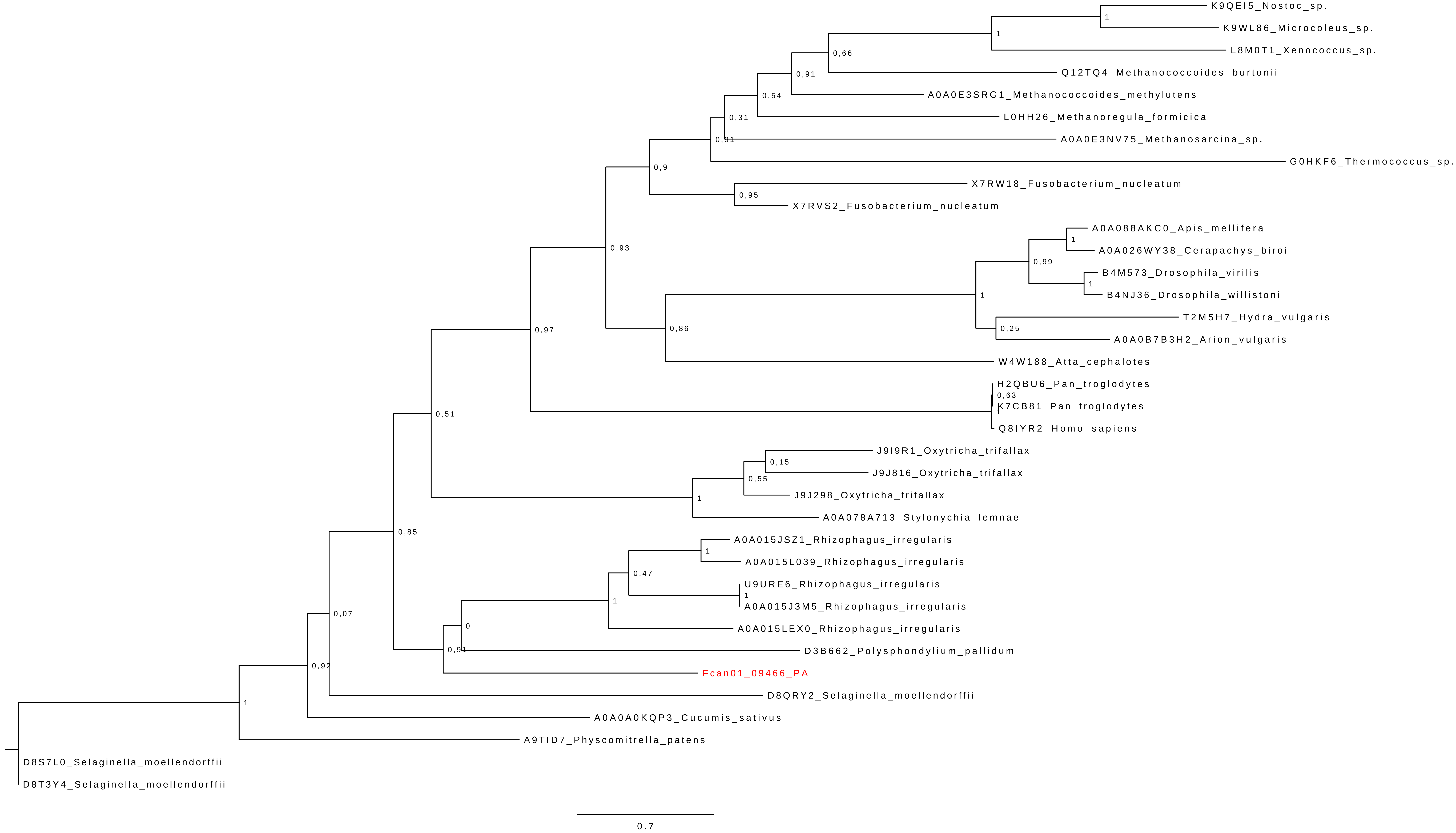
K9QEI5_Nostoc_sp.
K9WL86_Microcoleus_sp.
L8M0T1_Xenococcus_sp.
Q12TQ4_Methanococcoides_burtonii
A0A0E3SRG1_Methanococcoides_methylutens
L0HH26_Methanoregula_formicica
A0A0E3NV75_Methanosarcina_sp.
G0HKF6_Thermococcus_sp.
X7RW18_Fusobacterium_nucleatum
X7RVS2_Fusobacterium_nucleatum
A0A088AKC0_Apis_mellifera
A0A026WY38_Cerapachys_biroi
B4M573_Drosophila_virilis
B4NJ36_Drosophila_willistoni
T2M5H7_Hydra_vulgaris
A0A0B7B3H2_Arion_vulgaris
W4W188_Atta_cephalotes
H2QBU6_Pan_troglodytes
K7CB81_Pan_troglodytes
Q8IYR2_Homo_sapiens
J9I9R1_Oxytricha_trifallax
J9J816_Oxytricha_trifallax
J9J298_Oxytricha_trifallax
A0A078A713_Stylonychia_lemnae
A0A015JSZ1_Rhizophagus_irregularis
A0A015L039_Rhizophagus_irregularis
U9URE6_Rhizophagus_irregularis
A0A015J3M5_Rhizophagus_irregularis
A0A015LEX0_Rhizophagus_irregularis
D3B662_Polysphondylium_pallidum
Fcan01_09466_PA
D8QRY2_Selaginella_moellendorffii
A0A0A0KQP3_Cucumis_sativus
A9TID7_Physcomitrella_patens
D8S7L0_Selaginella_moellendorffii
D8T3Y4_Selaginella_moellendorffii
0.7

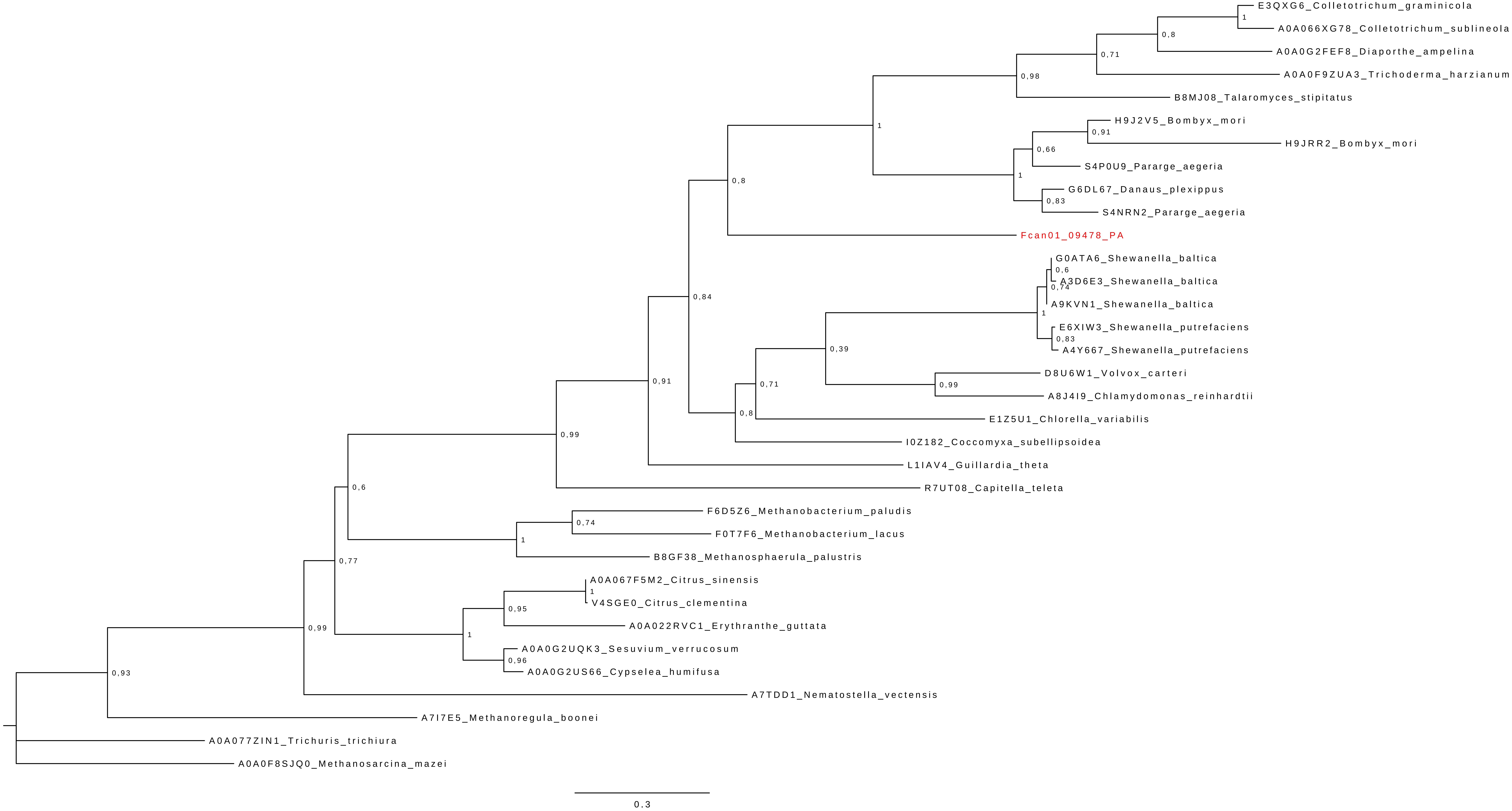
E3QXG6_Colletotrichum_graminicola
A0A066XG78_Colletotrichum_sublineola
A0A0G2FEF8_Diaporthe_ampelina
A0A0F9ZUA3_Trichoderma_harzianum
B8MJ08_Talaromyces_stipitatus
H9J2V5_Bombyx_mori
H9JRR2_Bombyx_mori
S4P0U9_Pararge_aegeria
G6DL67_Danaus_plexippus
S4NRN2_Pararge_aegeria
Fcan01_09478_PA
G0ATA6_Shewanella_baltica
A3D6E3_Shewanella_baltica
A9KVN1_Shewanella_baltica
E6XIW3_Shewanella_putrefaciens
A4Y667_Shewanella_putrefaciens
D8U6W1_Volvox_carteri
A8J4I9_Chlamydomonas_reinhardtii
E1Z5U1_Chlorella_variabilis
I0Z182_Coccomyxa_subellipsoidea
L1IAV4_Guillardia_theta
R7UT08_Capitella_teleta
F6D5Z6_Methanobacterium_paludis
F0T7F6_Methanobacterium_lacus
B8GF38_Methanosphaerula_palustris
A0A067F5M2_Citrus_sinensis
V4SGE0_Citrus_clementina
A0A022RVC1_Erythranthe_guttata
A0A0G2UQK3_Sesuvium_verrucosum
A0A0G2US66_Cypselea_humifusa
A7TDD1_Nematostella_vectensis
A7I7E5_Methanoregula_boonei
A0A077ZIN1_Trichuris_trichiura
A0A0F8SJQ0_Methanosarcina_mazei
1
0,8
0,71
0,98
1
0,91
0,66
1
0,83
0,8
0,6
0,74
1
0,83
0,84
0,39
0,99
0,71
0,91
0,8
0,99
0,6
0,74
1
0,77
1
0,95
1
0,96
0,99
0,93
0.3

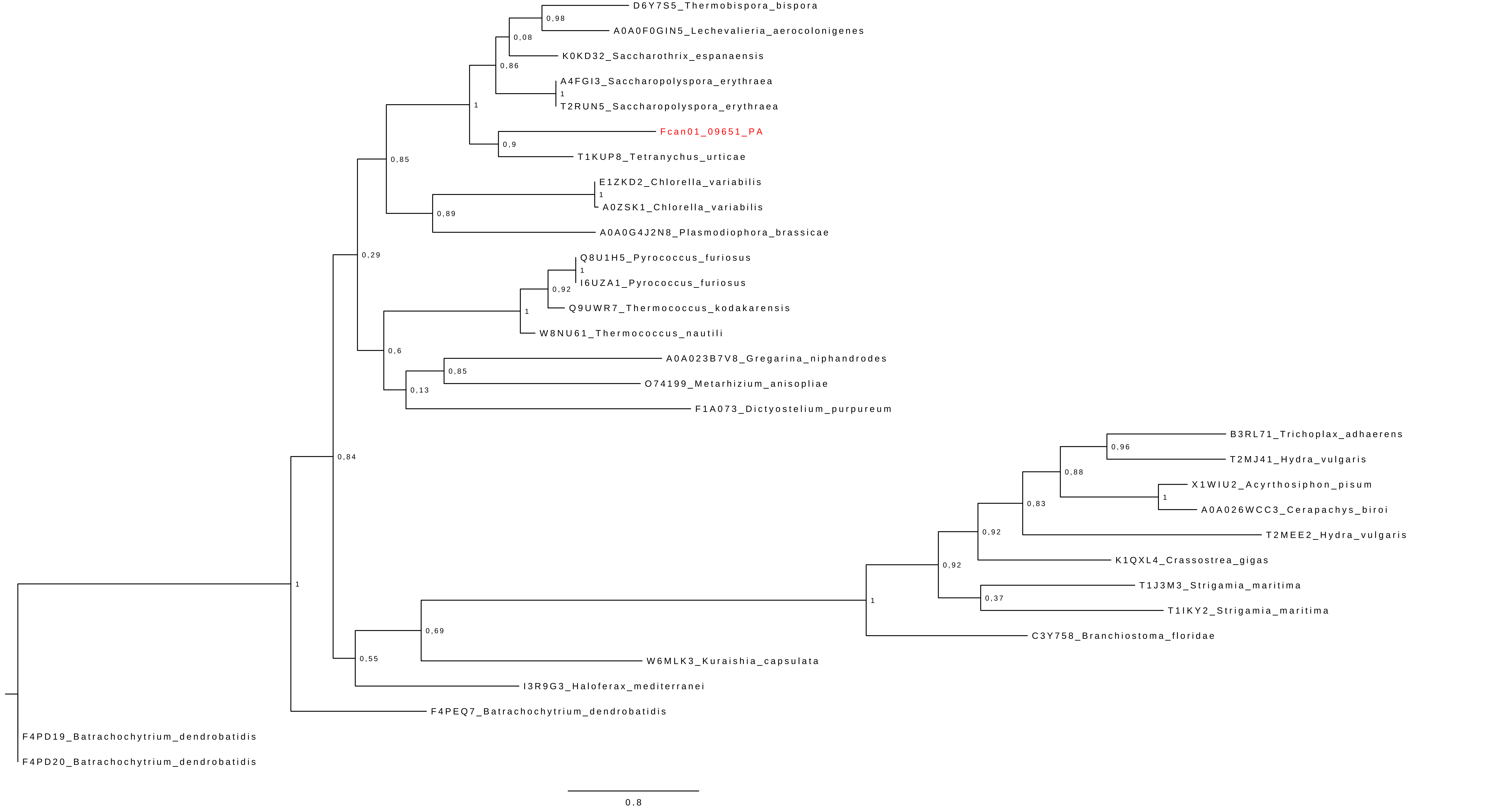
D6Y7S5_Thermobispora_bispora
A0A0F0GIN5_Lechevalieria_aerocolonigenes
K0KD32_Saccharothrix_espanaensis
A4FGI3_Saccharopolyspora_erythraea
T2RUN5_Saccharopolyspora_erythraea
Fcan01_09651_PA
T1KUP8_Tetranychus_urticae
E1ZKD2_Chlorella_variabilis
A0ZSK1_Chlorella_variabilis
A0A0G4J2N8_Plasmodiophora_brassicae
Q8U1H5_Pyrococcus_furiosus
I6UZA1_Pyrococcus_furiosus
Q9UWR7_Thermococcus_kodakarensis
W8NU61_Thermococcus_nautili
A0A023B7V8_Gregarina_niphandrodes
O74199_Metarhizium_anisopliae
F1A073_Dictyostelium_purpureum
B3RL71_Trichoplax_adhaerens
T2MJ41_Hydra_vulgaris
X1WIU2_Acyrthosiphon_pisum
A0A026WCC3_Cerapachys_biroi
T2MEE2_Hydra_vulgaris
K1QXL4_Crassostrea_gigas
T1J3M3_Strigamia_maritima
T1IKY2_Strigamia_maritima
C3Y758_Branchiostoma_floridae
W6MLK3_Kuraishia_capsulata
I3R9G3_Haloferax_mediterranei
F4PEQ7_Batrachochytrium_dendrobatidis
F4PD19_Batrachochytrium_dendrobatidis
F4PD20_Batrachochytrium_dendrobatidis
0.8

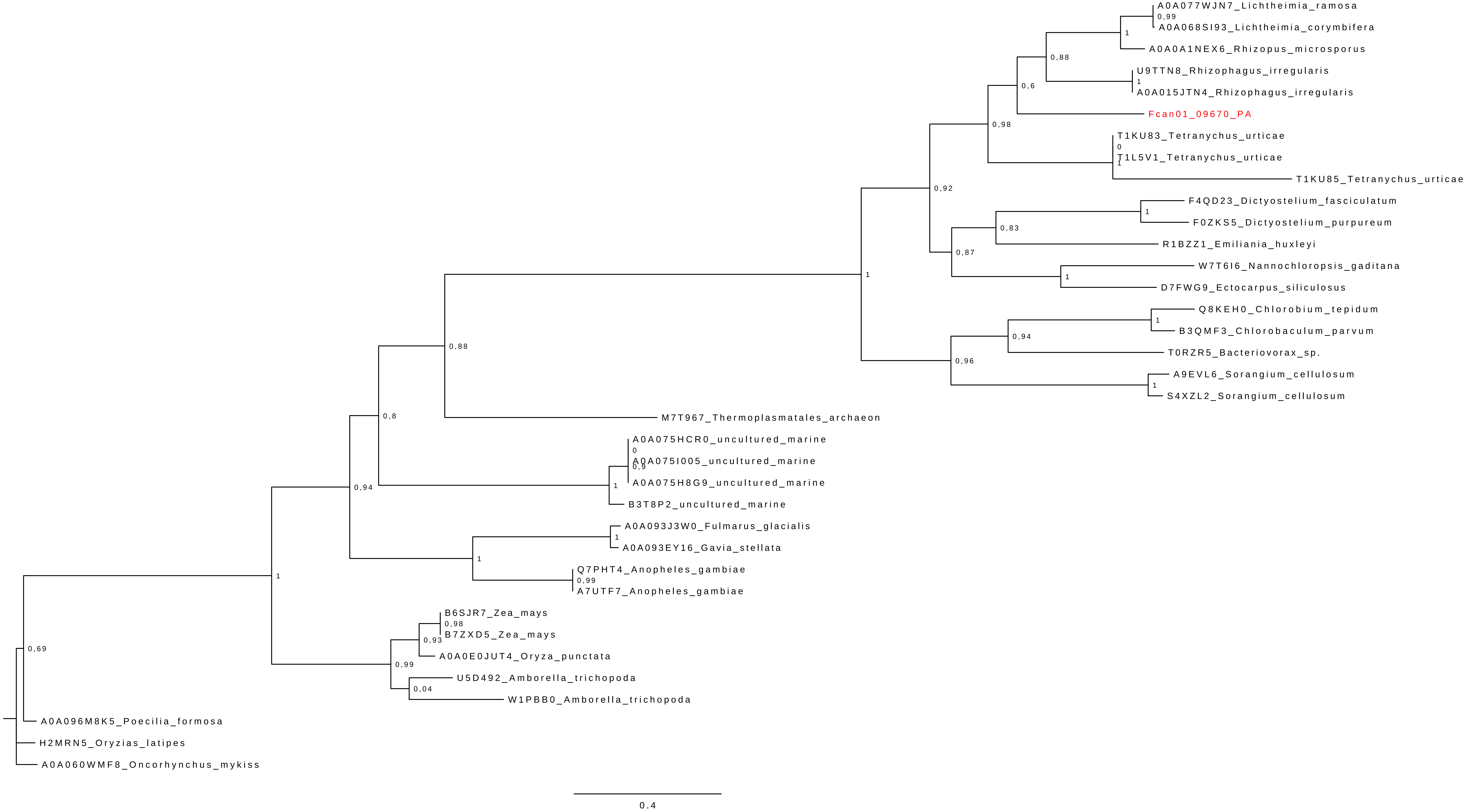
A0A077WJN7_Lichtheimia_ramosa
0,99
A0A068SI93_Lichtheimia_corymbifera
1
A0A0A1NEX6_Rhizopus_microsporus
0,88
U9TTN8_Rhizophagus_irregularis
1
A0A015JTN4_Rhizophagus_irregularis
0,6
Fcan01_09670_PA
0,98
T1KU83_Tetranychus_urticae
0
T1L5V1_Tetranychus_urticae
1
T1KU85_Tetranychus_urticae
0,92
F4QD23_Dictyostelium_fasciculatum
1
F0ZKS5_Dictyostelium_purpureum
0,83
R1BZZ1_Emiliania_huxleyi
0,87
W7T6I6_Nannochloropsis_gaditana
1
D7FWG9_Ectocarpus_siliculosus
1
Q8KEH0_Chlorobium_tepidum
1
B3QMF3_Chlorobaculum_parvum
0,94
T0RZR5_Bacteriovorax_sp.
0,96
A9EVL6_Sorangium_cellulosum
1
S4XZL2_Sorangium_cellulosum
0,88
M7T967_Thermoplasmatales_archaeon
0,8
A0A075HCR0_uncultured_marine
0
A0A075I005_uncultured_marine
0,9
A0A075H8G9_uncultured_marine
1
B3T8P2_uncultured_marine
0,94
A0A093J3W0_Fulmarus_glacialis
1
A0A093EY16_Gavia_stellata
1
Q7PHT4_Anopheles_gambiae
0,99
A7UTF7_Anopheles_gambiae
1
B6SJR7_Zea_mays
0,98
B7ZXD5_Zea_mays
0,93
A0A0E0JUT4_Oryza_punctata
0,99
U5D492_Amborella_trichopoda
0,04
W1PBB0_Amborella_trichopoda
0,69
A0A096M8K5_Poecilia_formosa
H2MRN5_Oryzias_latipes
A0A060WMF8_Oncorhynchus_mykiss
0.4

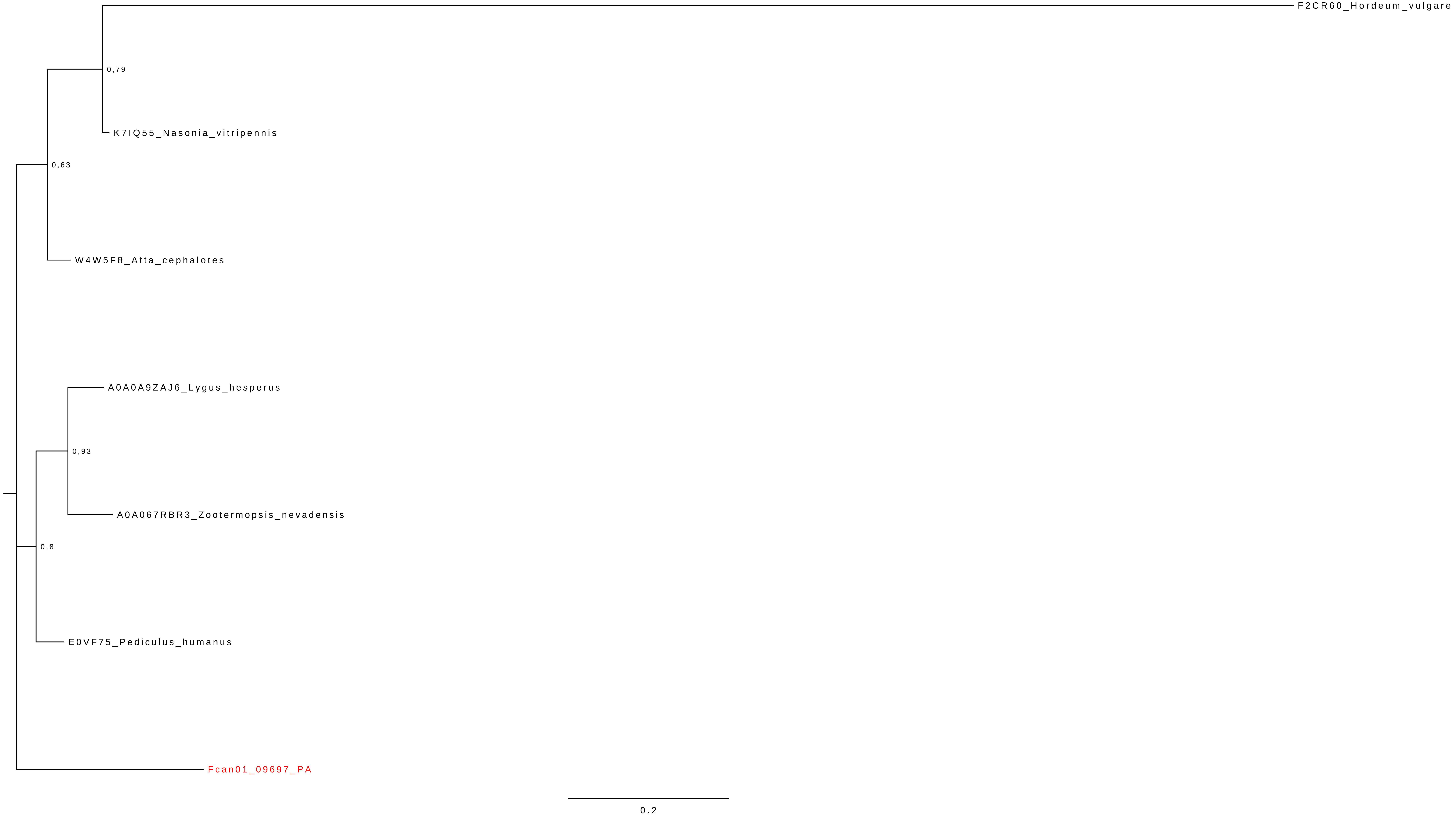
F2CR60_Hordeum_vulgare
0,79
K7IQ55_Nasonia_vitripennis
0,63
W4W5F8_Atta_cephalotes
A0A0A9ZAJ6_Lygus_hesperus
0,93
A0A067RBR3_Zootermopsis_nevadensis
0,8
E0VF75_Pediculus_humanus
Fcan01_09697_PA
0.2

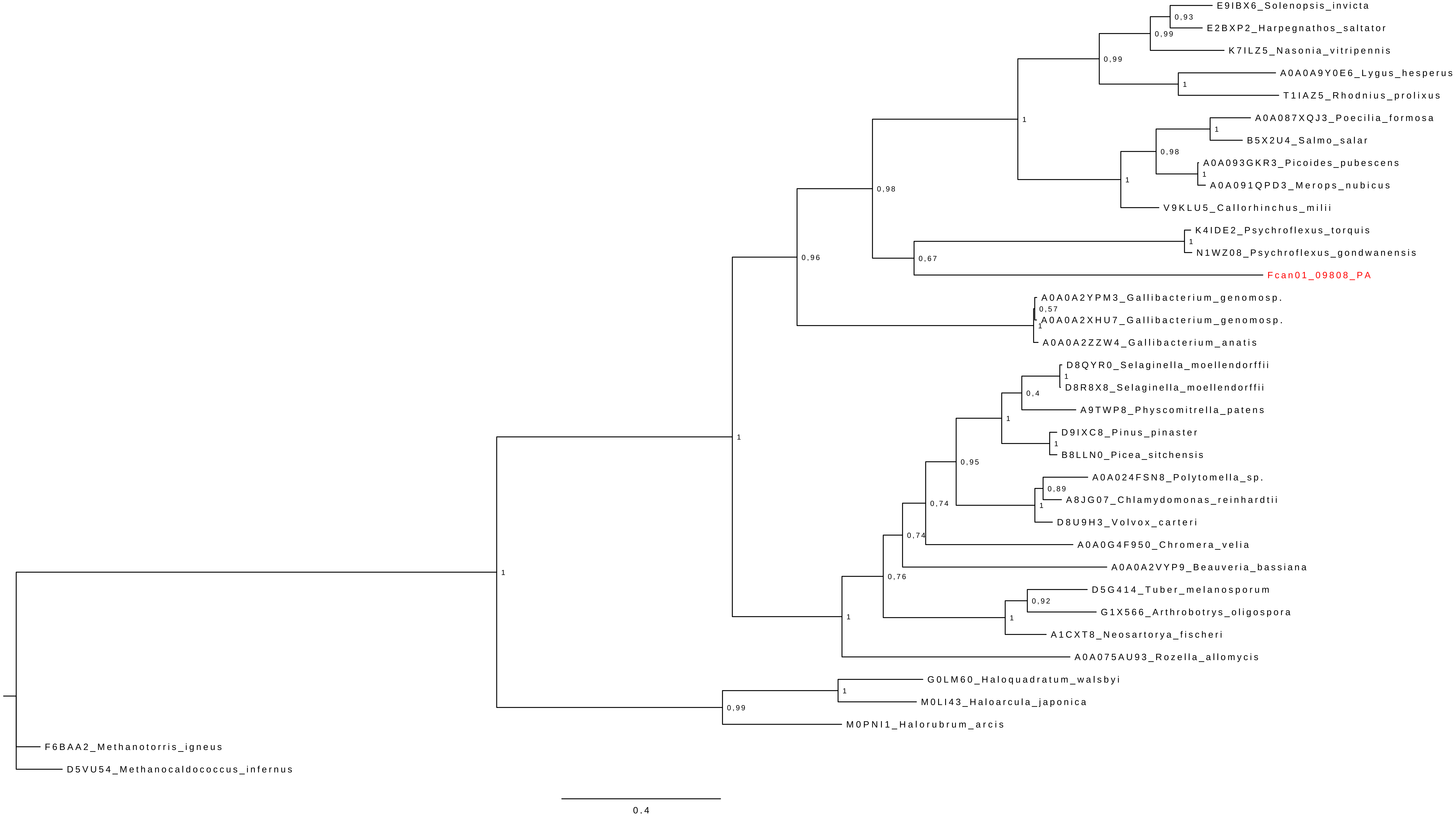
E9IBX6_Solenopsis_invicta
E2BXP2_Harpegnathos_saltator
K7ILZ5_Nasonia_vitripennis
A0A0A9Y0E6_Lygus_hesperus
T1IAZ5_Rhodnius_prolixus
A0A087XQJ3_Poecilia_formosa
B5X2U4_Salmo_salar
A0A093GKR3_Picoides_pubescens
A0A091QPD3_Merops_nubicus
V9KLU5_Callorhinchus_milii
K4IDE2_Psychroflexus_torquis
N1WZ08_Psychroflexus_gondwanensis
Fcan01_09808_PA
A0A0A2YPM3_Gallibacterium_genomosp.
A0A0A2XHU7_Gallibacterium_genomosp.
A0A0A2ZZW4_Gallibacterium_anatis
D8QYR0_Selaginella_moellendorffii
D8R8X8_Selaginella_moellendorffii
A9TWP8_Physcomitrella_patens
D9IXC8_Pinus_pinaster
B8LLN0_Picea_sitchensis
A0A024FSN8_Polytomella_sp.
A8JG07_Chlamydomonas_reinhardtii
D8U9H3_Volvox_carteri
A0A0G4F950_Chromera_velia
A0A0A2VYP9_Beauveria_bassiana
D5G414_Tuber_melanosporum
G1X566_Arthrobotrys_oligospora
A1CXT8_Neosartorya_fischeri
A0A075AU93_Rozella_allomycis
G0LM60_Haloquadratum_walsbyi
M0LI43_Haloarcula_japonica
M0PNI1_Halorubrum_arcis
F6BAA2_Methanotorris_igneus
D5VU54_Methanocaldococcus_infernus
0.4

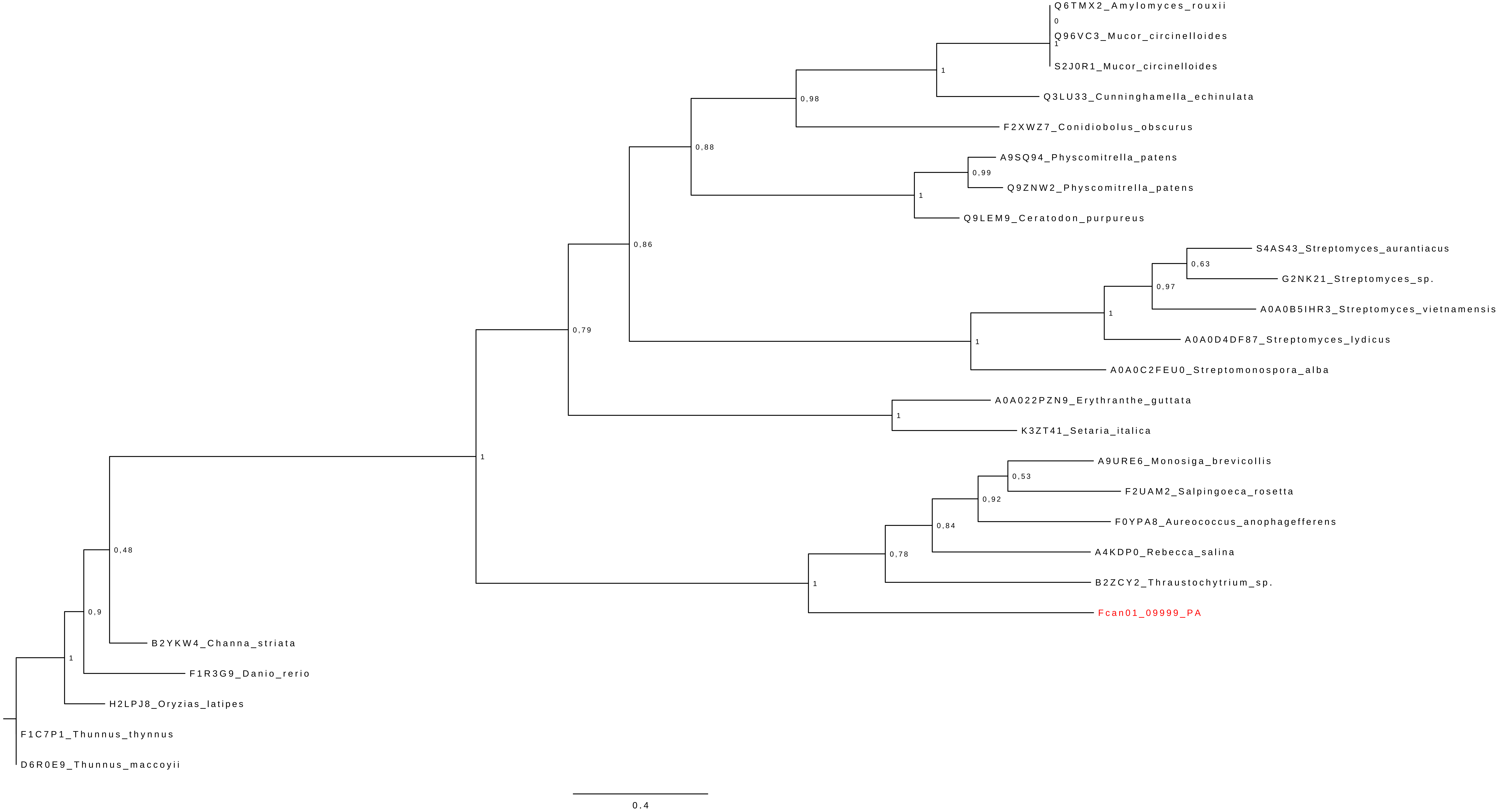
Q6TMX2_Amylomyces_rouxii
0
Q96VC3_Mucor_circinelloides
1
S2J0R1_Mucor_circinelloides
1
Q3LU33_Cunninghamella_echinulata
0,98
F2XWZ7_Conidiobolus_obscurus
0,88
A9SQ94_Physcomitrella_patens
0,99
Q9ZNW2_Physcomitrella_patens
1
Q9LEM9_Ceratodon_purpureus
0,86
S4AS43_Streptomyces_aurantiacus
0,63
G2NK21_Streptomyces_sp.
0,97
A0A0B5IHR3_Streptomyces_vietnamensis
1
A0A0D4DF87_Streptomyces_lydicus
1
A0A0C2FEU0_Streptomonospora_alba
0,79
A0A022PZN9_Erythranthe_guttata
1
K3ZT41_Setaria_italica
1
A9URE6_Monosiga_brevicollis
0,53
F2UAM2_Salpingoeca_rosetta
0,92
F0YPA8_Aureococcus_anophagefferens
0,84
A4KDP0_Rebecca_salina
0,78
B2ZCY2_Thraustochytrium_sp.
1
Fcan01_09999_PA
0,48
0,9
B2YKW4_Channa_striata
1
F1R3G9_Danio_rerio
H2LPJ8_Oryzias_latipes
F1C7P1_Thunnus_thynnus
D6R0E9_Thunnus_maccoyii
0.4

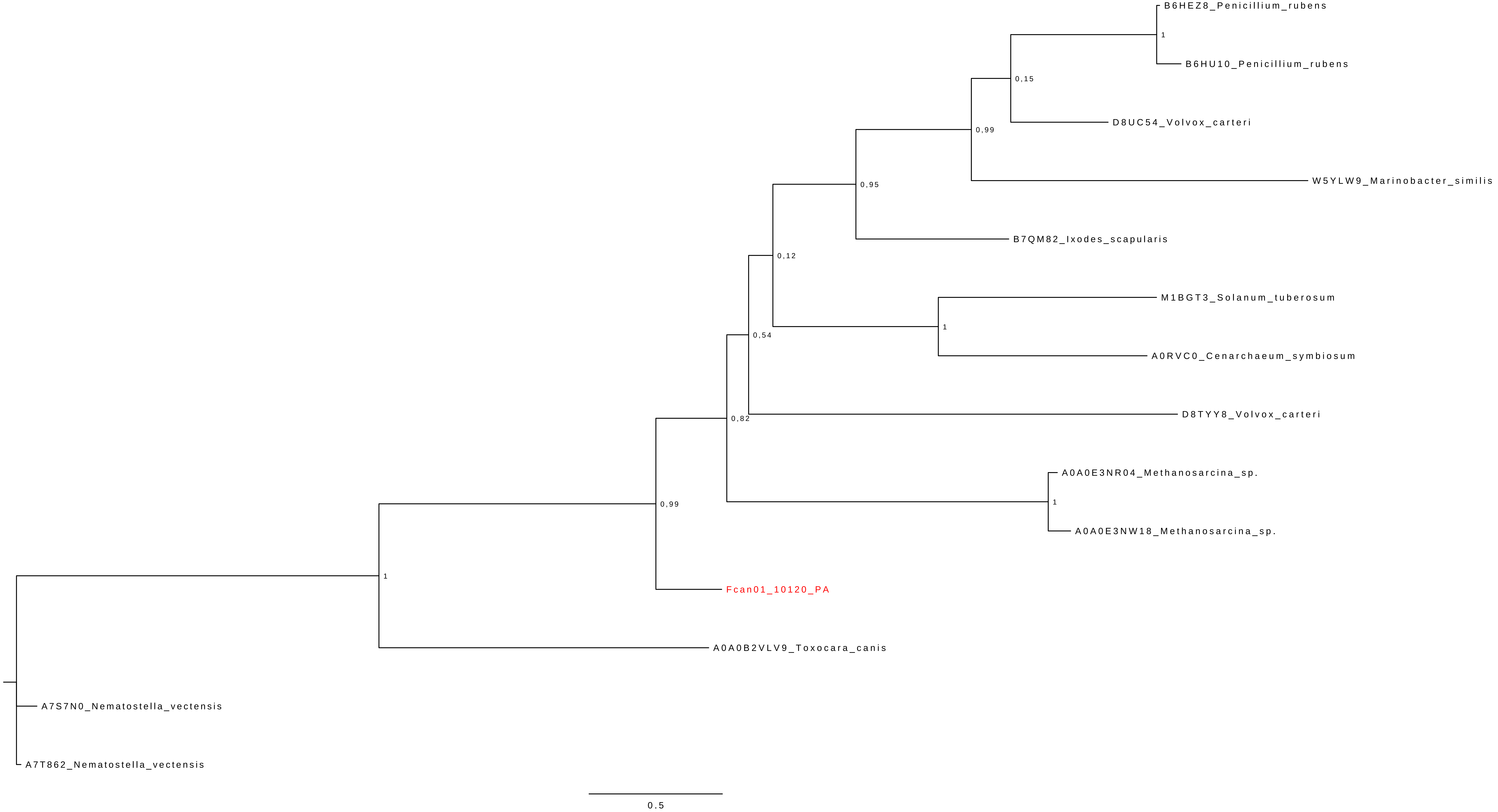
B6HEZ8_Penicillium_rubens
1
B6HU10_Penicillium_rubens
0,15
D8UC54_Volvox_carteri
0,99
W5YLW9_Marinobacter_similis
0,95
B7QM82_Ixodes_scapularis
0,12
M1BGT3_Solanum_tuberosum
1
A0RVC0_Cenarchaeum_symbiosum
0,54
D8TYY8_Volvox_carteri
0,82
A0A0E3NR04_Methanosarcina_sp.
1
A0A0E3NW18_Methanosarcina_sp.
0,99
Fcan01_10120_PA
1
A0A0B2VLV9_Toxocara_canis
A7S7N0_Nematostella_vectensis
A7T862_Nematostella_vectensis
0.5

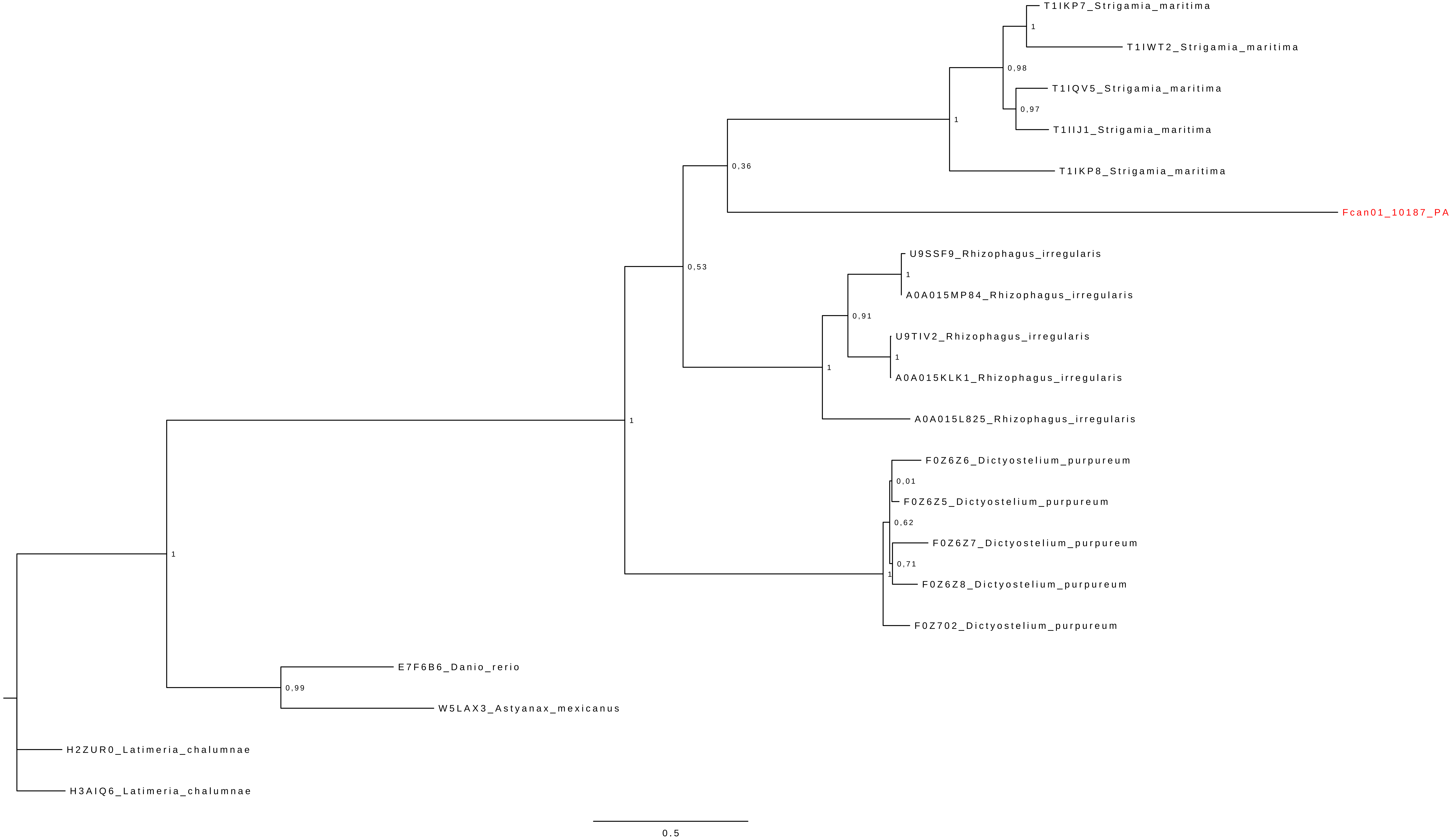
T1IKP7_Strigamia_maritima
1
T1IWT2_Strigamia_maritima
0,98
T1IQV5_Strigamia_maritima
0,97
T1IIJ1_Strigamia_maritima
1
T1IKP8_Strigamia_maritima
0,36
Fcan01_10187_PA
U9SSF9_Rhizophagus_irregularis
1
A0A015MP84_Rhizophagus_irregularis
0,53
0,91
U9TIV2_Rhizophagus_irregularis
1
A0A015KLK1_Rhizophagus_irregularis
1
A0A015L825_Rhizophagus_irregularis
1
F0Z6Z6_Dictyostelium_purpureum
0,01
F0Z6Z5_Dictyostelium_purpureum
0,62
F0Z6Z7_Dictyostelium_purpureum
0,71
1
F0Z6Z8_Dictyostelium_purpureum
F0Z702_Dictyostelium_purpureum
1
E7F6B6_Danio_rerio
0,99
W5LAX3_Astyanax_mexicanus
H2ZUR0_Latimeria_chalumnae
H3AIQ6_Latimeria_chalumnae
0.5

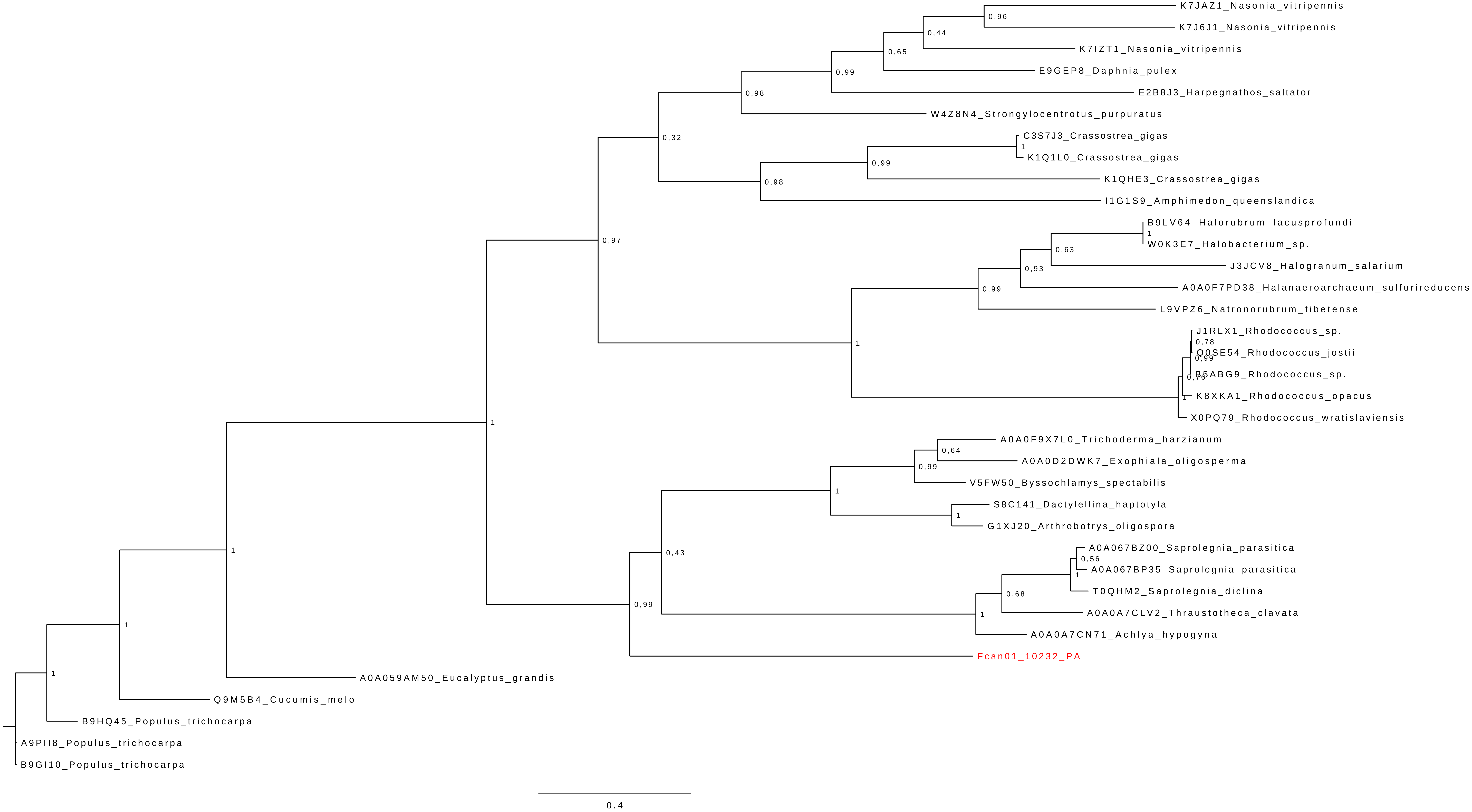

K7JAZ1_Nasonia_vitripennis
K7J6J1_Nasonia_vitripennis
K7IZT1_Nasonia_vitripennis
E9GEP8_Daphnia_pulex
E2B8J3_Harpegnathos_saltator
W4Z8N4_Strongylocentrotus_purpuratus
C3S7J3_Crassostrea_gigas
K1Q1L0_Crassostrea_gigas
K1QHE3_Crassostrea_gigas
I1G1S9_Amphimedon_queenslandica
B9LV64_Halorubrum_lacusprofundi
W0K3E7_Halobacterium_sp.
J3JCV8_Halogranum_salarium
A0A0F7PD38_Halanaeroarchaeum_sulfurireducens
L9VPZ6_Natronorubrum_tibetense
J1RLX1_Rhodococcus_sp.
Q0SE54_Rhodococcus_jostii
B5ABG9_Rhodococcus_sp.
K8XKA1_Rhodococcus_opacus
X0PQ79_Rhodococcus_wratislaviensis
A0A0F9X7L0_Trichoderma_harzianum
A0A0D2DWK7_Exophiala_oligosperma
V5FW50_Byssochlamys_spectabilis
S8C141_Dactylellina_haptotyla
G1XJ20_Arthrobotrys_oligospora
A0A067BZ00_Saprolegnia_parasitica
A0A067BP35_Saprolegnia_parasitica
T0QHM2_Saprolegnia_diclina
A0A0A7CLV2_Thraustotheca_clavata
A0A0A7CN71_Achlya_hypogyna
Fcan01_10232_PA
A0A059AM50_Eucalyptus_grandis
Q9M5B4_Cucumis_melo
B9HQ45_Populus_trichocarpa
A9PII8_Populus_trichocarpa
B9GI10_Populus_trichocarpa
0.4

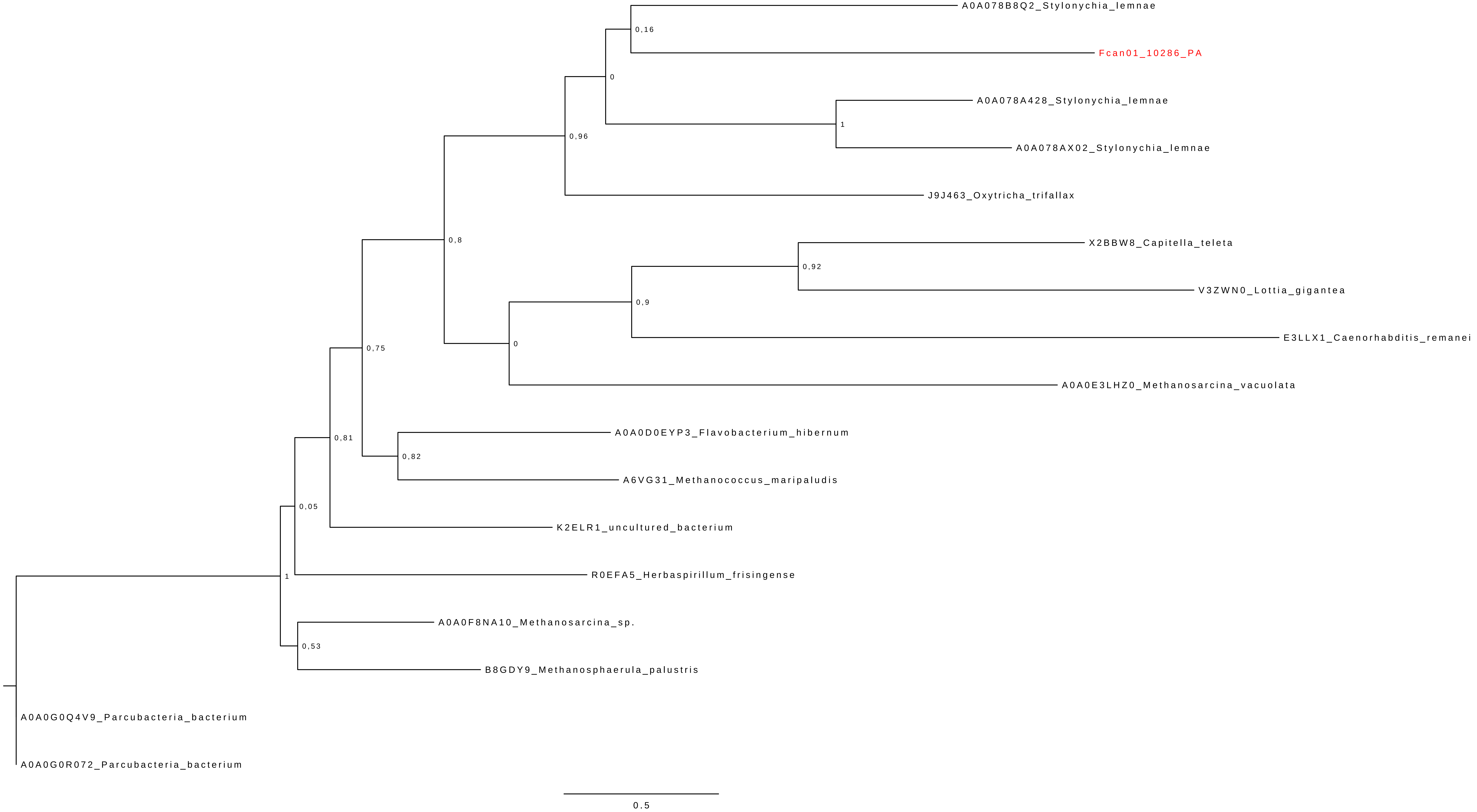
A0A078B8Q2_Stylonychia_lemnae
0,16
Fcan01_10286_PA
0
A0A078A428_Stylonychia_lemnae
1
A0A078AX02_Stylonychia_lemnae
0,96
J9J463_Oxytricha_trifallax
0,8
X2BBW8_Capitella_teleta
0,92
V3ZWN0_Lottia_gigantea
0,9
E3LLX1_Caenorhabditis_remanei
0
A0A0E3LHZ0_Methanosarcina_vacuolata
0,75
A0A0D0EYP3_Flavobacterium_hibernum
0,81
0,82
A6VG31_Methanococcus_maripaludis
0,05
K2ELR1_uncultured_bacterium
1
R0EFA5_Herbaspirillum_frisingense
A0A0F8NA10_Methanosarcina_sp.
0,53
B8GDY9_Methanosphaerula_palustris
A0A0G0Q4V9_Parcubacteria_bacterium
A0A0G0R072_Parcubacteria_bacterium
0.5

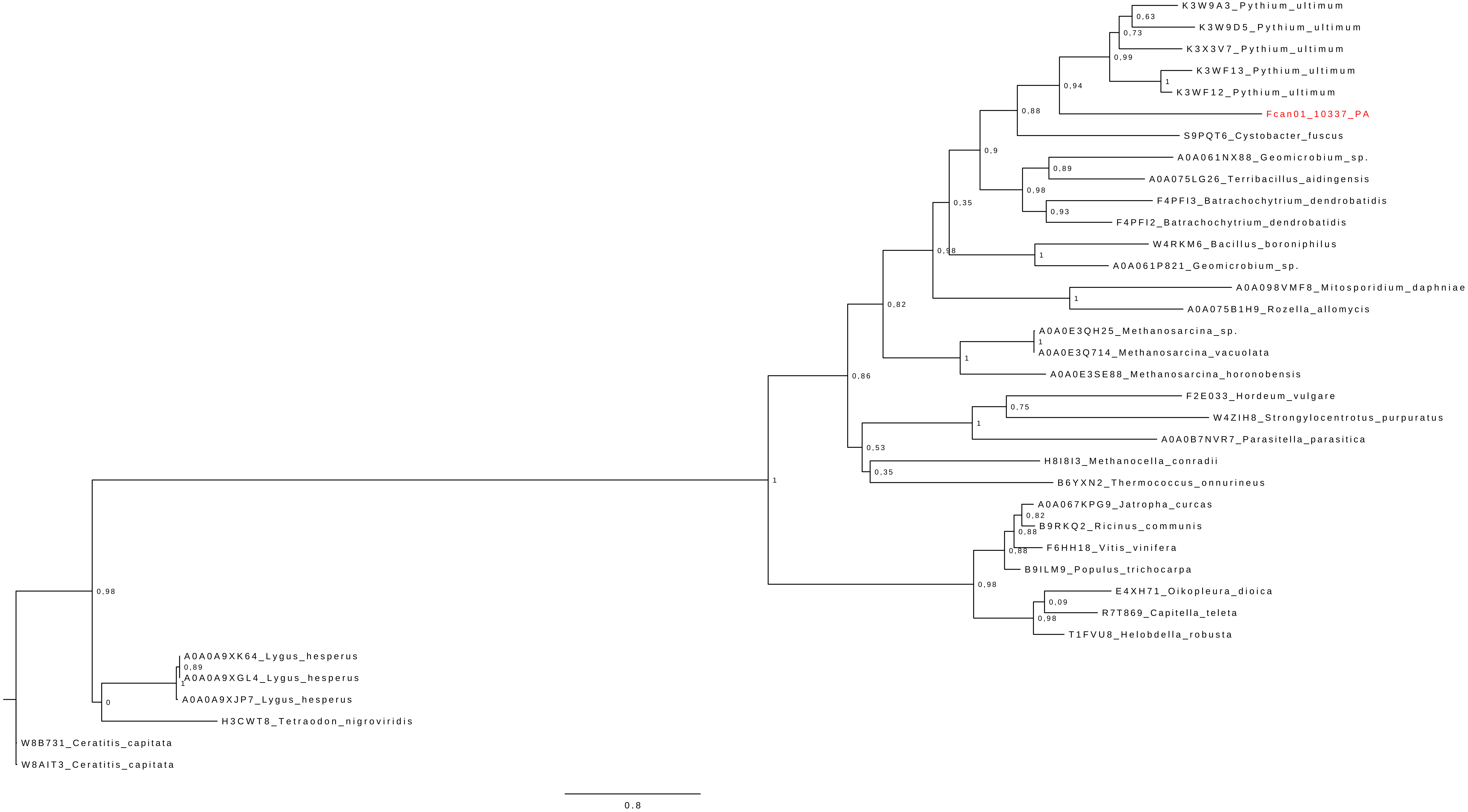
K3W9A3_Pythium_ultimum
0,63
K3W9D5_Pythium_ultimum
0,73
K3X3V7_Pythium_ultimum
0,99
K3WF13_Pythium_ultimum
1
K3WF12_Pythium_ultimum
0,94
Fcan01_10337_PA
0,88
S9PQT6_Cystobacter_fuscus
0,9
A0A061NX88_Geomicrobium_sp.
0,89
A0A075LG26_Terribacillus_aidingensis
0,98
F4PFI3_Batrachochytrium_dendrobatidis
0,35
0,93
F4PFI2_Batrachochytrium_dendrobatidis
W4RKM6_Bacillus_boroniphilus
0,98
1
A0A061P821_Geomicrobium_sp.
A0A098VMF8_Mitosporidium_daphniae
1
A0A075B1H9_Rozella_allomycis
0,82
A0A0E3QH25_Methanosarcina_sp.
1
A0A0E3Q714_Methanosarcina_vacuolata
1
A0A0E3SE88_Methanosarcina_horonobensis
0,86
F2E033_Hordeum_vulgare
0,75
W4ZIH8_Strongylocentrotus_purpuratus
1
A0A0B7NVR7_Parasitella_parasitica
0,53
H8I8I3_Methanocella_conradii
0,35
B6YXN2_Thermococcus_onnurineus
1
A0A067KPG9_Jatropha_curcas
0,82
B9RKQ2_Ricinus_communis
0,88
F6HH18_Vitis_vinifera
0,88
B9ILM9_Populus_trichocarpa
0,98
E4XH71_Oikopleura_dioica
0,09
R7T869_Capitella_teleta
0,98
T1FVU8_Helobdella_robusta
0,98
A0A0A9XK64_Lygus_hesperus
0,89
A0A0A9XGL4_Lygus_hesperus
1
A0A0A9XJP7_Lygus_hesperus
0
H3CWT8_Tetraodon_nigroviridis
W8B731_Ceratitis_capitata
W8AIT3_Ceratitis_capitata
0.8

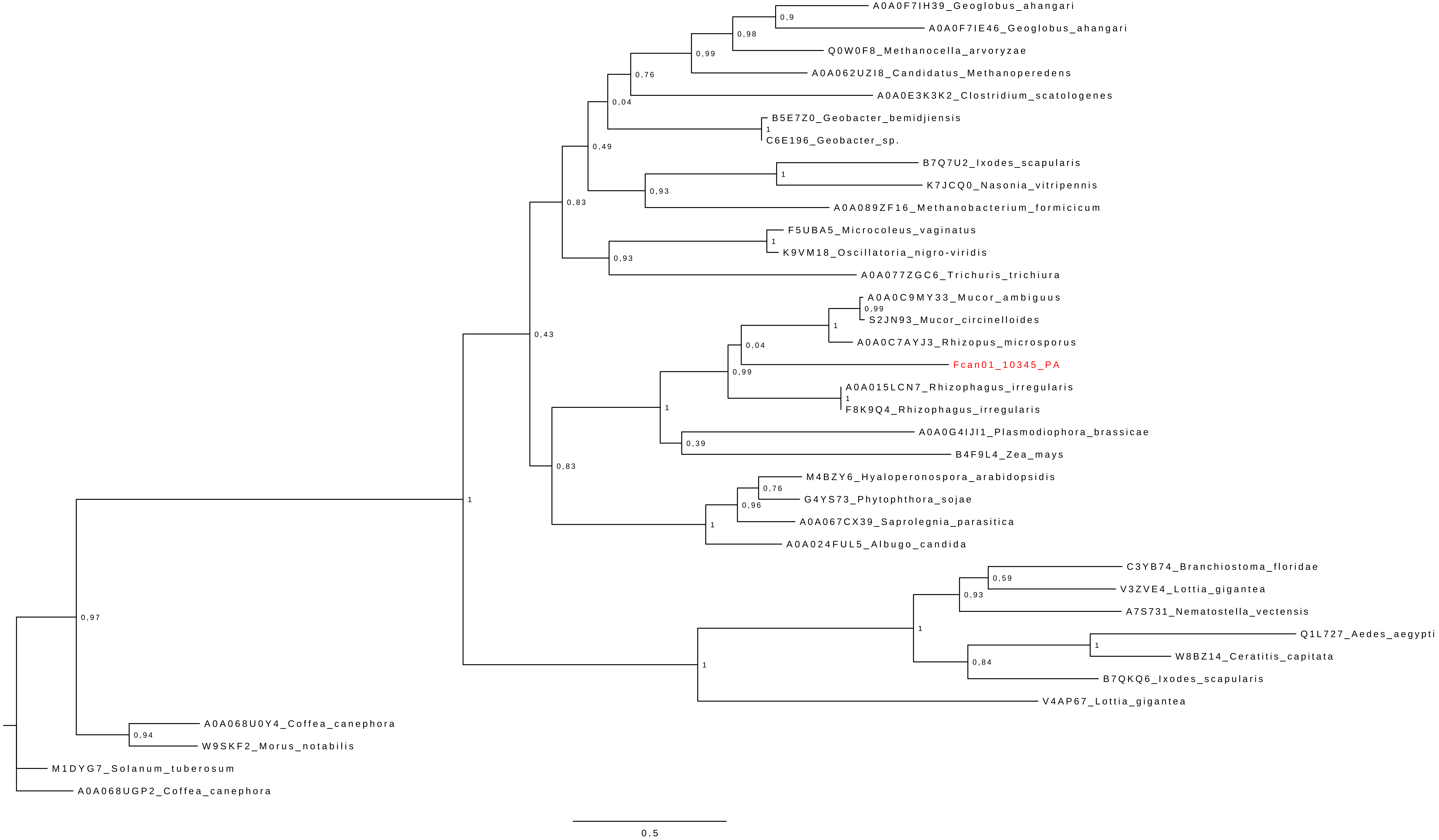
A0A0F7IH39_Geoglobus_ahangari
A0A0F7IE46_Geoglobus_ahangari
Q0W0F8_Methanocella_arvoryzae
A0A062UZI8_Candidatus_Methanoperedens
A0A0E3K3K2_Clostridium_scatologenes
B5E7Z0_Geobacter_bemidjiensis
C6E196_Geobacter_sp.
B7Q7U2_Ixodes_scapularis
K7JCQ0_Nasonia_vitripennis
A0A089ZF16_Methanobacterium_formicicum
F5UBA5_Microcoleus_vaginatus
K9VM18_Oscillatoria_nigro-viridis
A0A077ZGC6_Trichuris_trichiura
A0A0C9MY33_Mucor_ambiguus
S2JN93_Mucor_circinelloides
A0A0C7AYJ3_Rhizopus_microsporus
Fcan01_10345_PA
A0A015LCN7_Rhizophagus_irregularis
F8K9Q4_Rhizophagus_irregularis
A0A0G4IJI1_Plasmodiophora_brassicae
B4F9L4_Zea_mays
M4BZY6_Hyaloperonospora_arabidopsidis
G4YS73_Phytophthora_sojae
A0A067CX39_Saprolegnia_parasitica
A0A024FUL5_Albugo_candida
C3YB74_Branchiostoma_floridae
V3ZVE4_Lottia_gigantea
A7S731_Nematostella_vectensis
Q1L727_Aedes_aegypti
W8BZ14_Ceratitis_capitata
B7QKQ6_Ixodes_scapularis
V4AP67_Lottia_gigantea
A0A068U0Y4_Coffea_canephora
W9SKF2_Morus_notabilis
M1DYG7_Solanum_tuberosum
A0A068UGP2_Coffea_canephora
0,9
0,98
0,99
0,76
0,04
1
0,49
1
0,93
0,83
1
0,93
0,99
1
0,04
0,99
1
1
0,39
0,43
0,83
0,76
0,96
1
1
0,59
0,93
1
0,84
1
0,97
0,94
0.5

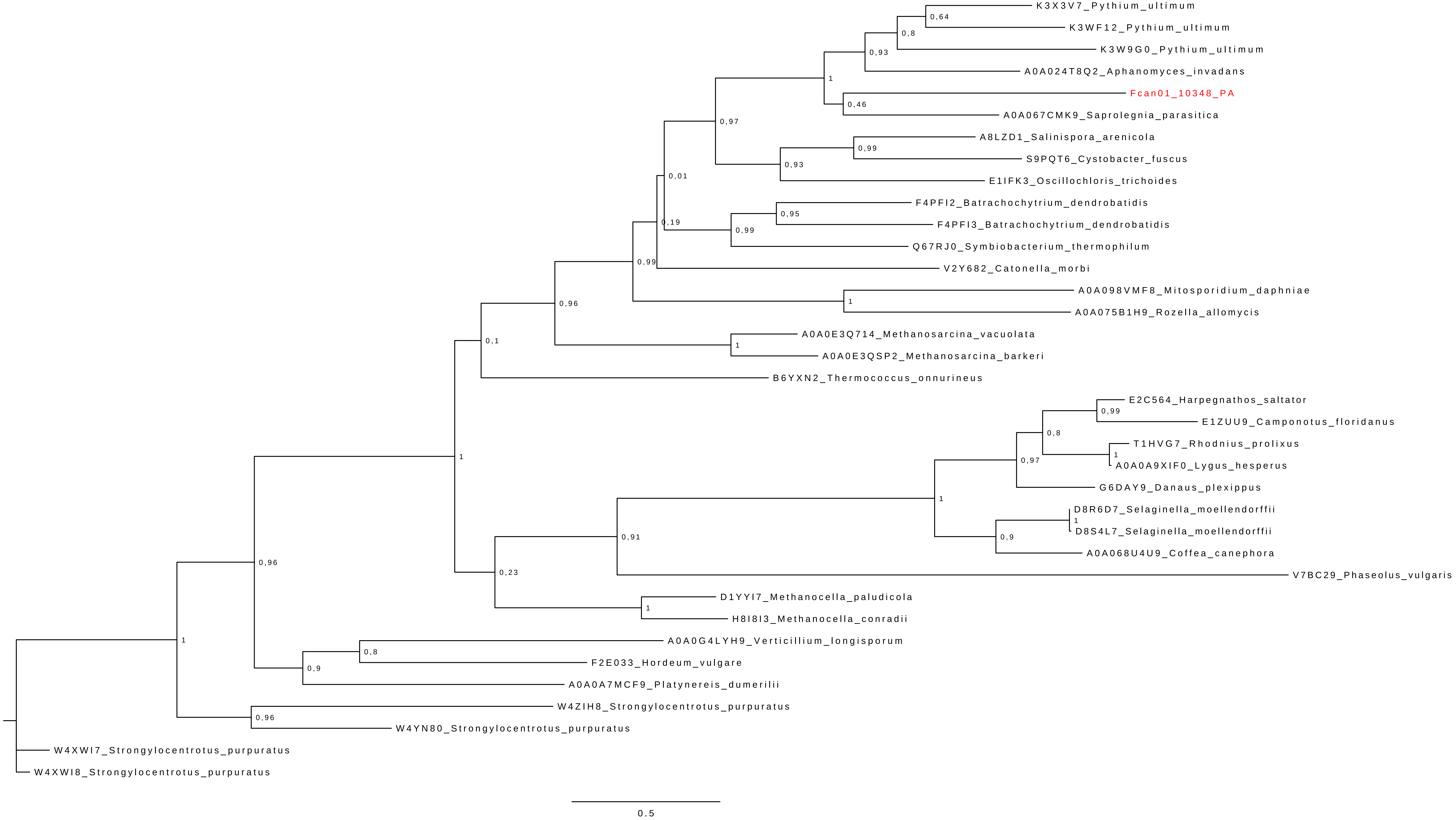
K3X3V7_Pythium_ultimum
K3WF12_Pythium_ultimum
K3W9G0_Pythium_ultimum
A0A024T8Q2_Aphanomyces_invadans
Fcan01_10348_PA
A0A067CMK9_Saprolegnia_parasitica
A8LZD1_Salinispora_arenicola
S9PQT6_Cystobacter_fuscus
E1IFK3_Oscillochloris_trichoides
F4PFI2_Batrachochytrium_dendrobatidis
F4PFI3_Batrachochytrium_dendrobatidis
Q67RJ0_Symbiobacterium_thermophilum
V2Y682_Catonella_morbi
A0A098VMF8_Mitosporidium_daphniae
A0A075B1H9_Rozella_allomycis
A0A0E3Q714_Methanosarcina_vacuolata
A0A0E3QSP2_Methanosarcina_barkeri
B6YXN2_Thermococcus_onnurineus
E2C564_Harpegnathos_saltator
E1ZUU9_Camponotus_floridanus
T1HVG7_Rhodnius_prolixus
A0A0A9XIF0_Lygus_hesperus
G6DAY9_Danaus_plexippus
D8R6D7_Selaginella_moellendorffii
D8S4L7_Selaginella_moellendorffii
A0A068U4U9_Coffea_canephora
V7BC29_Phaseolus_vulgaris
D1YYI7_Methanocella_paludicola
H8I8I3_Methanocella_conradii
A0A0G4LYH9_Verticillium_longisporum
F2E033_Hordeum_vulgare
A0A0A7MCF9_Platynereis_dumerilii
W4ZIH8_Strongylocentrotus_purpuratus
W4YN80_Strongylocentrotus_purpuratus
W4XWI7_Strongylocentrotus_purpuratus
W4XWI8_Strongylocentrotus_purpuratus
0,64
0,8
0,93
1
0,46
0,97
0,99
0,93
0,01
0,95
0,19
0,99
0,99
1
0,96
1
0,1
0,99
0,8
1
0,97
1
1
0,9
0,91
0,23
1
1
0,96
0,8
0,9
1
0,96
0.5

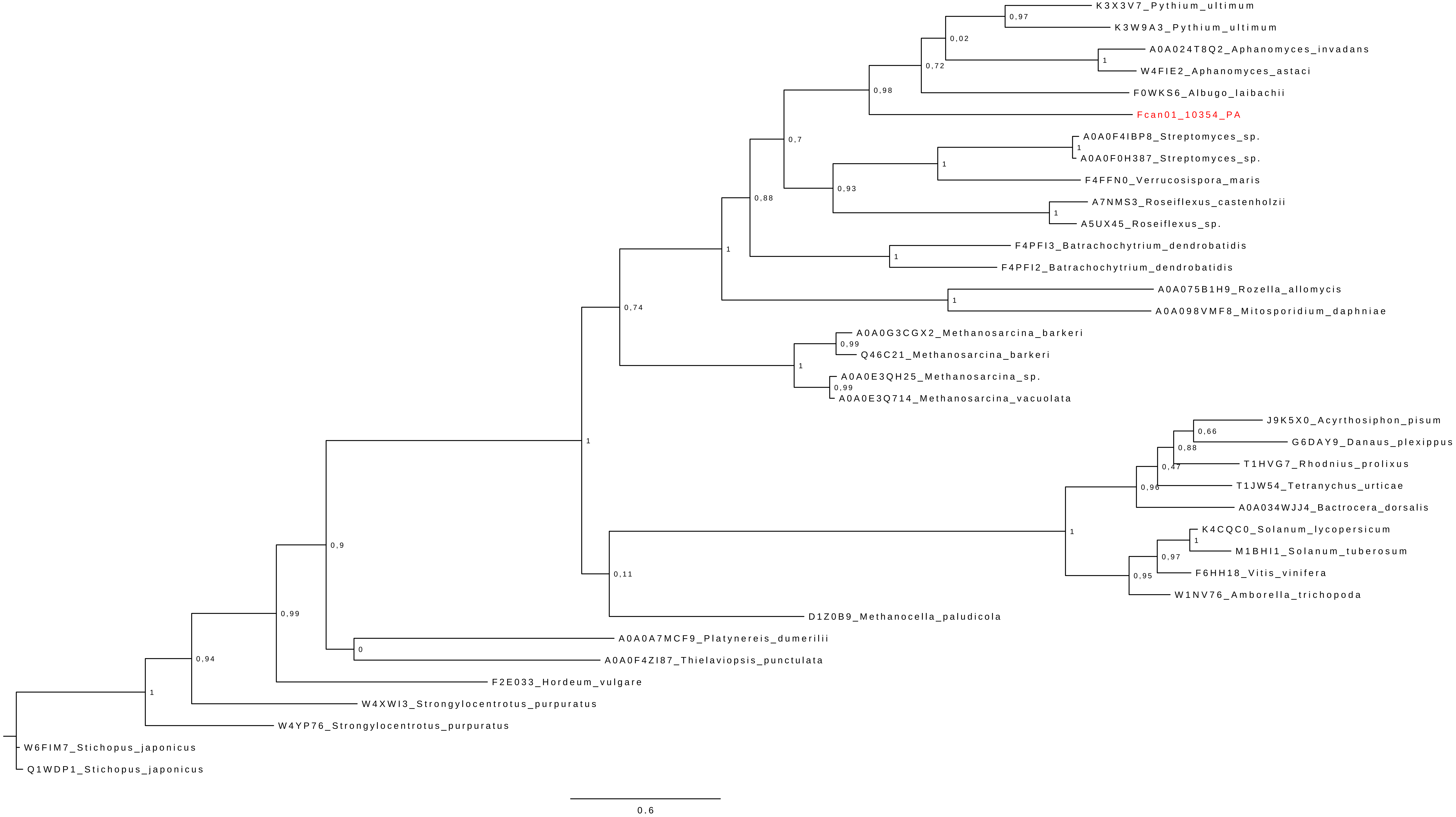
K3X3V7_Pythium_ultimum
K3W9A3_Pythium_ultimum
A0A024T8Q2_Aphanomyces_invadans
W4FIE2_Aphanomyces_astaci
F0WKS6_Albugo_laibachii
Fcan01_10354_PA
A0A0F4IBP8_Streptomyces_sp.
A0A0F0H387_Streptomyces_sp.
F4FFN0_Verrucosispora_maris
A7NMS3_Roseiflexus_castenholzii
A5UX45_Roseiflexus_sp.
F4PFI3_Batrachochytrium_dendrobatidis
F4PFI2_Batrachochytrium_dendrobatidis
A0A075B1H9_Rozella_allomycis
A0A098VMF8_Mitosporidium_daphniae
A0A0G3CGX2_Methanosarcina_barkeri
Q46C21_Methanosarcina_barkeri
A0A0E3QH25_Methanosarcina_sp.
A0A0E3Q714_Methanosarcina_vacuolata
J9K5X0_Acyrthosiphon_pisum
G6DAY9_Danaus_plexippus
T1HVG7_Rhodnius_prolixus
T1JW54_Tetranychus_urticae
A0A034WJJ4_Bactrocera_dorsalis
K4CQC0_Solanum_lycopersicum
M1BHI1_Solanum_tuberosum
F6HH18_Vitis_vinifera
W1NV76_Amborella_trichopoda
D1Z0B9_Methanocella_paludicola
A0A0A7MCF9_Platynereis_dumerilii
A0A0F4ZI87_Thielaviopsis_punctulata
F2E033_Hordeum_vulgare
W4XWI3_Strongylocentrotus_purpuratus
W4YP76_Strongylocentrotus_purpuratus
W6FIM7_Stichopus_japonicus
Q1WDP1_Stichopus_japonicus
0.6

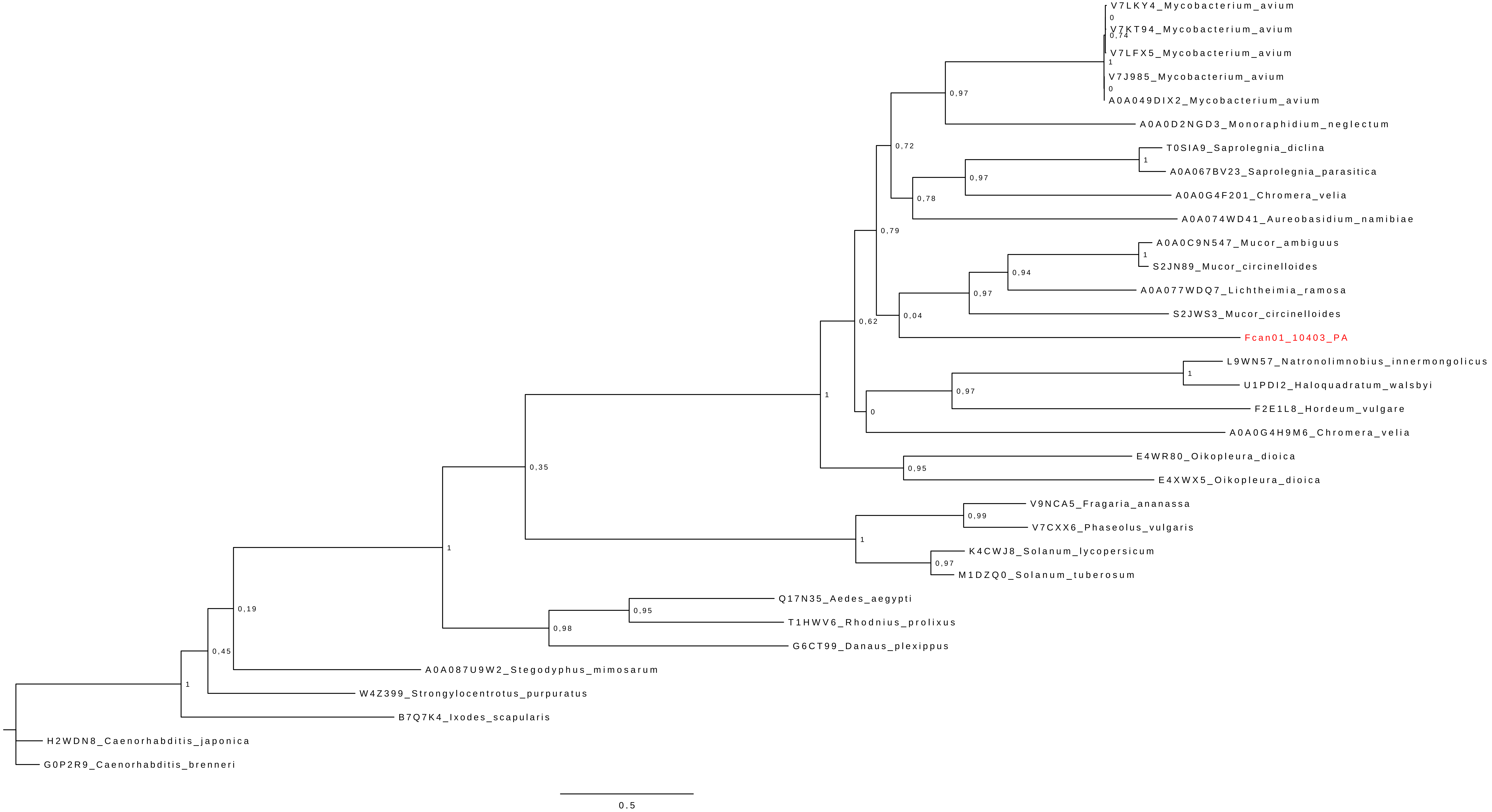

V7LKY4_Mycobacterium_avium
0
V7KT94_Mycobacterium_avium
0,74
V7LFX5_Mycobacterium_avium
1
V7J985_Mycobacterium_avium
0
A0A049DIX2_Mycobacterium_avium
0,97
A0A0D2NGD3_Monoraphidium_neglectum
T0SIA9_Saprolegnia_diclina
0,72
1
A0A067BV23_Saprolegnia_parasitica
0,97
A0A0G4F201_Chromera_velia
0,78
A0A074WD41_Aureobasidium_namibiae
0,79
A0A0C9N547_Mucor_ambiguus
1
S2JN89_Mucor_circinelloides
0,94
A0A077WDQ7_Lichtheimia_ramosa
0,97
S2JWS3_Mucor_circinelloides
0,04
0,62
Fcan01_10403_PA
L9WN57_Natronolimnobius_innermongolicus
1
U1PDI2_Haloquadratum_walsbyi
1
0,97
F2E1L8_Hordeum_vulgare
0
A0A0G4H9M6_Chromera_velia
E4WR80_Oikopleura_dioica
0,35
0,95
E4XWX5_Oikopleura_dioica
V9NCA5_Fragaria_ananassa
0,99
V7CXX6_Phaseolus_vulgaris
1
1
K4CWJ8_Solanum_lycopersicum
0,97
M1DZQ0_Solanum_tuberosum
Q17N35_Aedes_aegypti
0,19
0,95
T1HWV6_Rhodnius_prolixus
0,98
G6CT99_Danaus_plexippus
0,45
A0A087U9W2_Stegodyphus_mimosarum
1
W4Z399_Strongylocentrotus_purpuratus
B7Q7K4_Ixodes_scapularis
H2WDN8_Caenorhabditis_japonica
G0P2R9_Caenorhabditis_brenneri
0.5

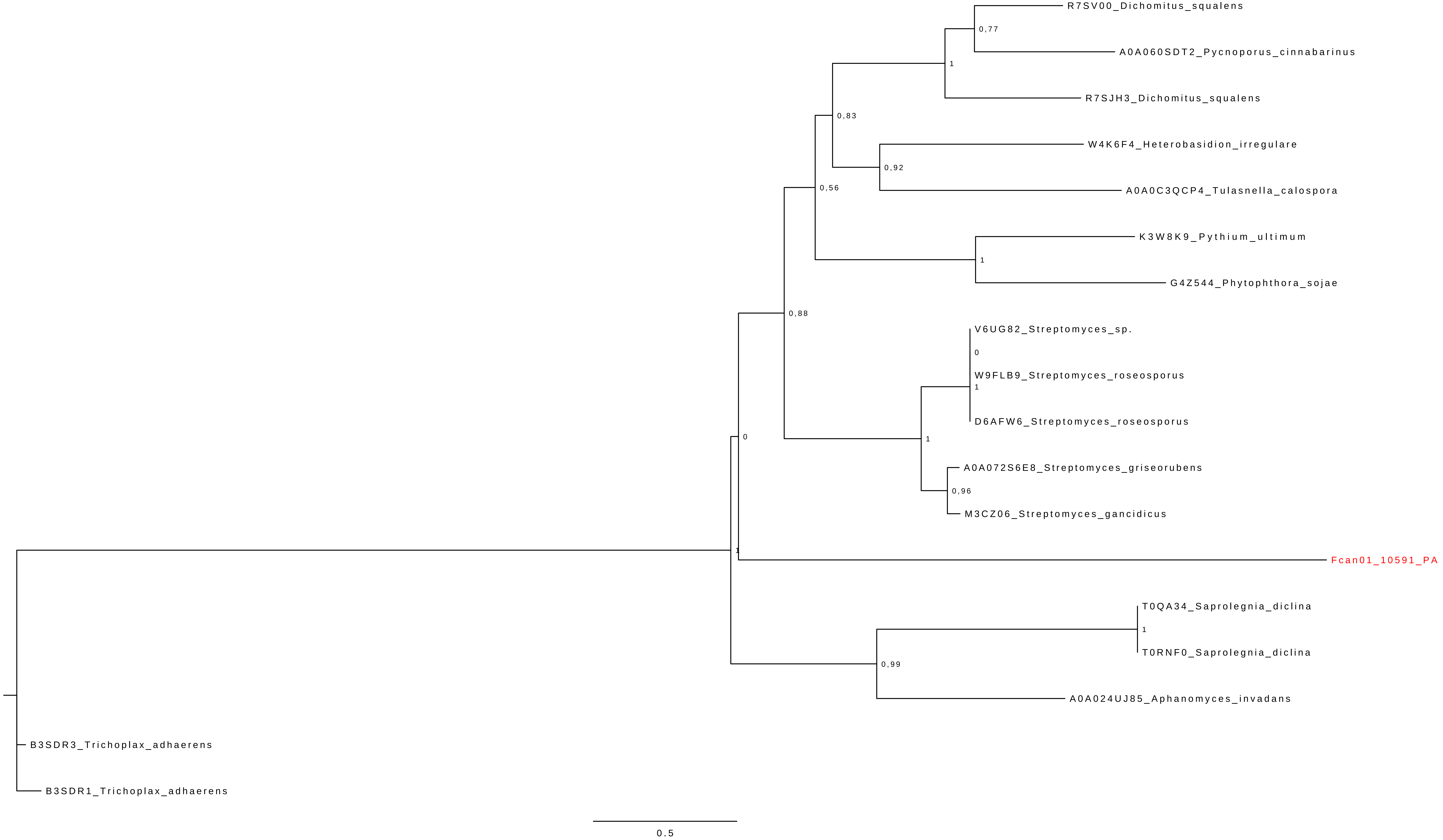
R7SV00_Dichomitus_squalens
0,77
A0A060SDT2_Pycnoporus_cinnabarinus
1
R7SJH3_Dichomitus_squalens
0,83
W4K6F4_Heterobasidion_irregulare
0,92
0,56
A0A0C3QCP4_Tulasnella_calospora
K3W8K9_Pythium_ultimum
1
G4Z544_Phytophthora_sojae
0,88
V6UG82_Streptomyces_sp.
0
W9FLB9_Streptomyces_roseosporus
1
D6AFW6_Streptomyces_roseosporus
0
1
A0A072S6E8_Streptomyces_griseorubens
0,96
M3CZ06_Streptomyces_gancidicus
1
Fcan01_10591_PA
T0QA34_Saprolegnia_diclina
1
T0RNF0_Saprolegnia_diclina
0,99
A0A024UJ85_Aphanomyces_invadans
B3SDR3_Trichoplax_adhaerens
B3SDR1_Trichoplax_adhaerens
0.5

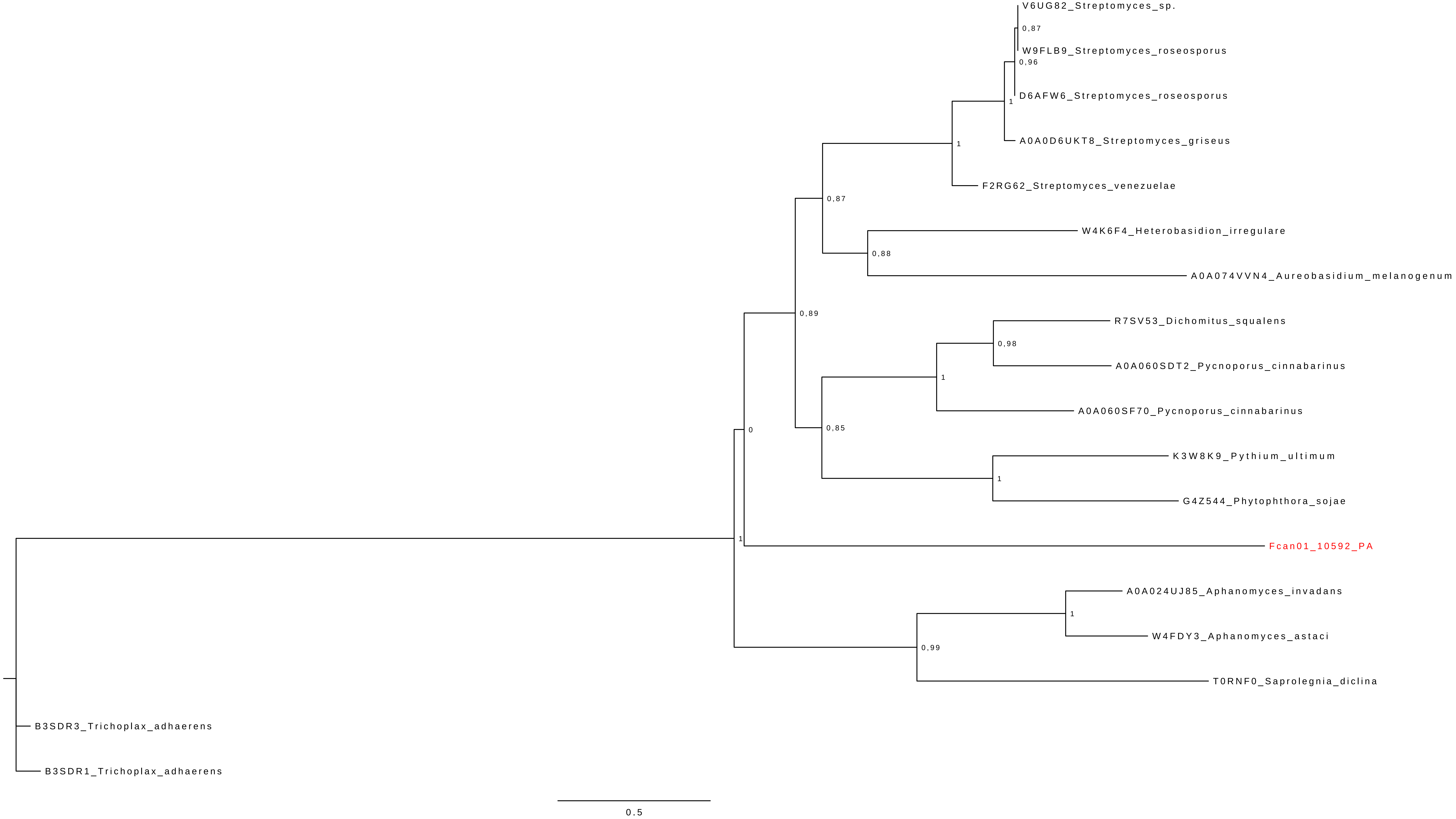
V6UG82_Streptomyces_sp.
0,87
W9FLB9_Streptomyces_roseosporus
0,96
D6AFW6_Streptomyces_roseosporus
1
A0A0D6UKT8_Streptomyces_griseus
1
F2RG62_Streptomyces_venezuelae
0,87
W4K6F4_Heterobasidion_irregulare
0,88
A0A074VVN4_Aureobasidium_melanogenum
0,89
R7SV53_Dichomitus_squalens
0,98
A0A060SDT2_Pycnoporus_cinnabarinus
1
A0A060SF70_Pycnoporus_cinnabarinus
0
0,85
K3W8K9_Pythium_ultimum
1
G4Z544_Phytophthora_sojae
1
Fcan01_10592_PA
A0A024UJ85_Aphanomyces_invadans
1
W4FDY3_Aphanomyces_astaci
0,99
T0RNF0_Saprolegnia_diclina
B3SDR3_Trichoplax_adhaerens
B3SDR1_Trichoplax_adhaerens
0.5

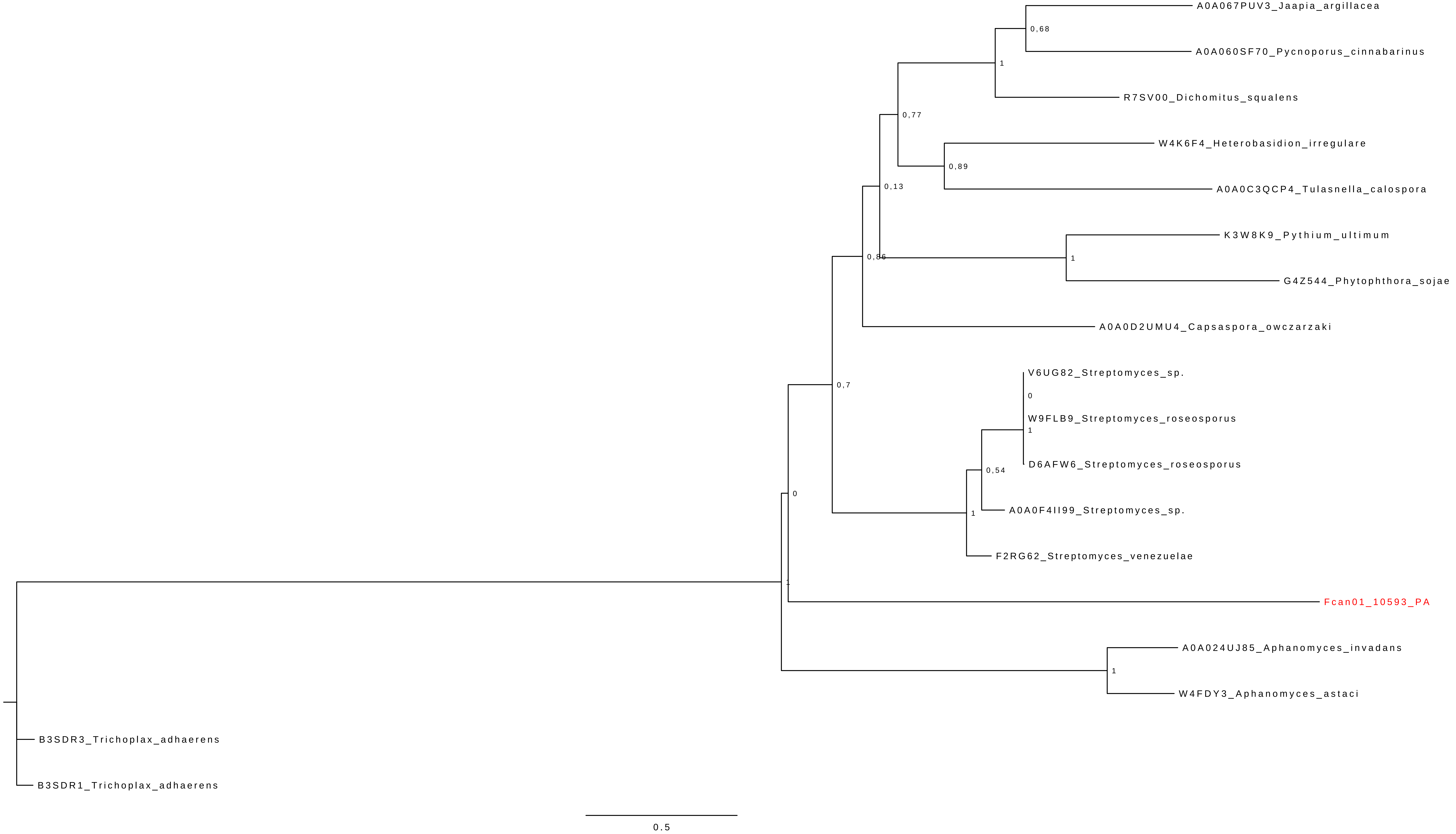
A0A067PUV3_Jaapia_argillacea
0,68
A0A060SF70_Pycnoporus_cinnabarinus
1
R7SV00_Dichomitus_squalens
0,77
W4K6F4_Heterobasidion_irregulare
0,89
A0A0C3QCP4_Tulasnella_calospora
0,13
K3W8K9_Pythium_ultimum
0,86
1
G4Z544_Phytophthora_sojae
A0A0D2UMU4_Capsaspora_owczarzaki
V6UG82_Streptomyces_sp.
0,7
0
W9FLB9_Streptomyces_roseosporus
1
D6AFW6_Streptomyces_roseosporus
0,54
0
A0A0F4II99_Streptomyces_sp.
1
F2RG62_Streptomyces_venezuelae
1
Fcan01_10593_PA
A0A024UJ85_Aphanomyces_invadans
1
W4FDY3_Aphanomyces_astaci
B3SDR3_Trichoplax_adhaerens
B3SDR1_Trichoplax_adhaerens
0.5

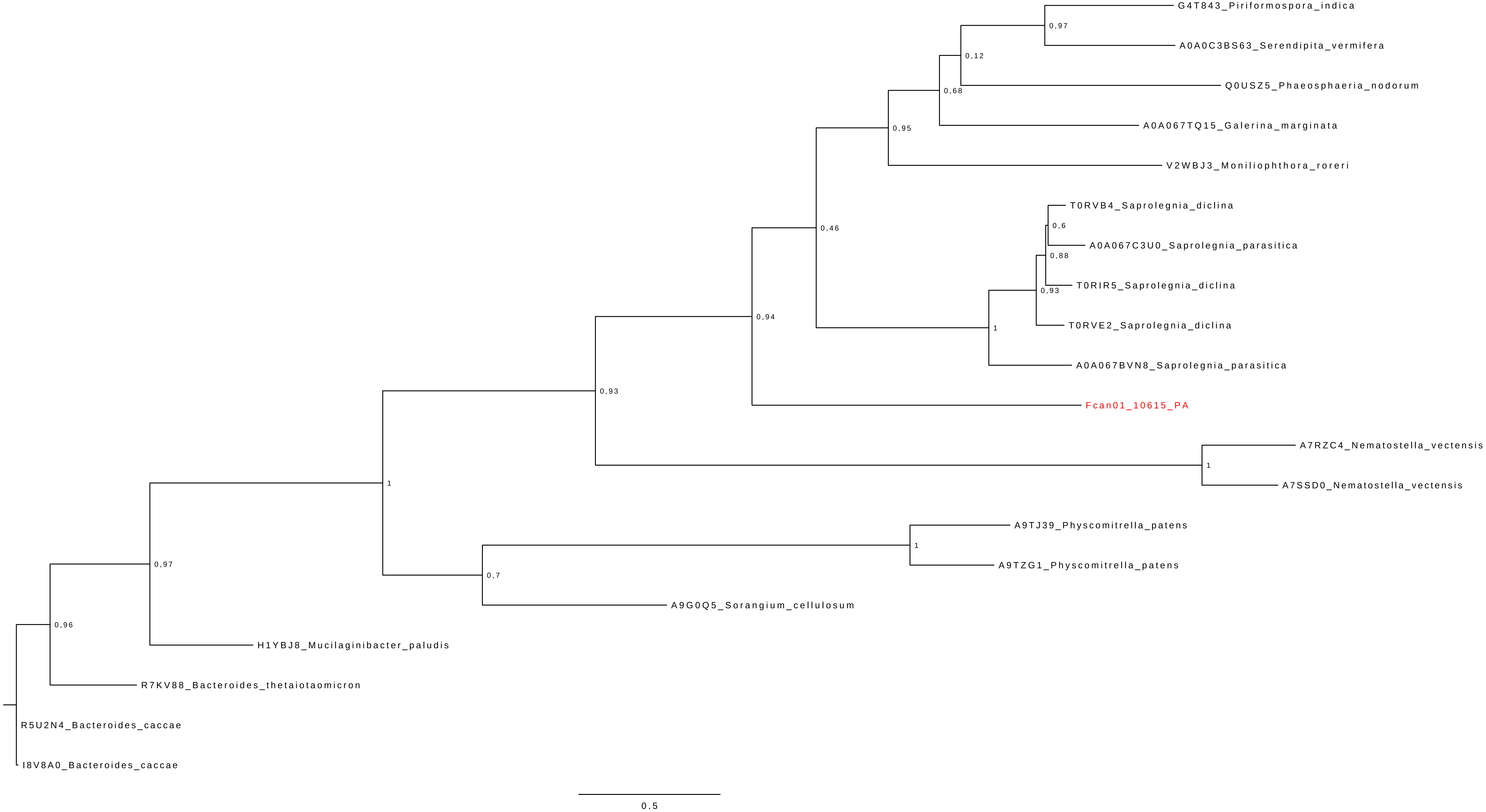
G4T843_Piriformospora_indica
0,97
A0A0C3BS63_Serendipita_vermifera
0,12
Q0USZ5_Phaeosphaeria_nodorum
0,68
A0A067TQ15_Galerina_marginata
0,95
V2WBJ3_Moniliophthora_roreri
T0RVB4_Saprolegnia_diclina
0,6
0,46
A0A067C3U0_Saprolegnia_parasitica
0,88
T0RIR5_Saprolegnia_diclina
0,93
0,94
1
T0RVE2_Saprolegnia_diclina
A0A067BVN8_Saprolegnia_parasitica
0,93
Fcan01_10615_PA
A7RZC4_Nematostella_vectensis
1
1
A7SSD0_Nematostella_vectensis
A9TJ39_Physcomitrella_patens
1
0,97
A9TZG1_Physcomitrella_patens
0,7
A9G0Q5_Sorangium_cellulosum
0,96
H1YBJ8_Mucilaginibacter_paludis
R7KV88_Bacteroides_thetaiotaomicron
R5U2N4_Bacteroides_caccae
I8V8A0_Bacteroides_caccae
0.5

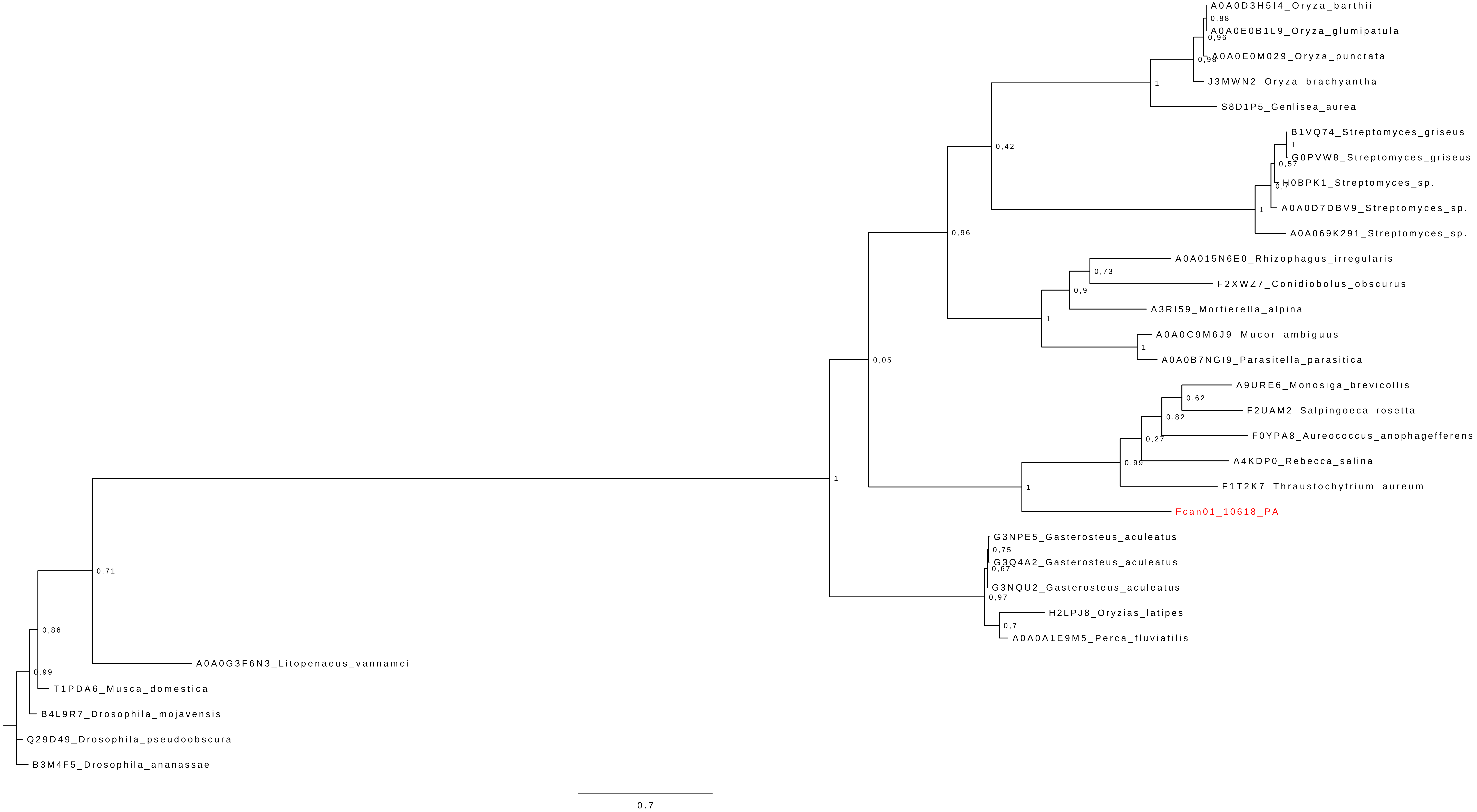
A0A0D3H5I4_Oryza_barthii
0,88
A0A0E0B1L9_Oryza_glumipatula
0,96
A0A0E0M029_Oryza_punctata
0,98
J3MWN2_Oryza_brachyantha
1
S8D1P5_Genlisea_aurea
0,42
B1VQ74_Streptomyces_griseus
1
G0PVW8_Streptomyces_griseus
0,57
H0BPK1_Streptomyces_sp.
0,7
A0A0D7DBV9_Streptomyces_sp.
1
A0A069K291_Streptomyces_sp.
0,96
A0A015N6E0_Rhizophagus_irregularis
0,73
F2XWZ7_Conidiobolus_obscurus
0,9
A3RI59_Mortierella_alpina
1
A0A0C9M6J9_Mucor_ambiguus
1
A0A0B7NGI9_Parasitella_parasitica
0,05
A9URE6_Monosiga_brevicollis
0,62
F2UAM2_Salpingoeca_rosetta
0,82
F0YPA8_Aureococcus_anophagefferens
0,27
A4KDP0_Rebecca_salina
0,99
F1T2K7_Thraustochytrium_aureum
1
Fcan01_10618_PA
1
G3NPE5_Gasterosteus_aculeatus
0,75
G3Q4A2_Gasterosteus_aculeatus
0,67
G3NQU2_Gasterosteus_aculeatus
0,97
H2LPJ8_Oryzias_latipes
0,7
A0A0A1E9M5_Perca_fluviatilis
0,71
0,86
A0A0G3F6N3_Litopenaeus_vannamei
0,99
T1PDA6_Musca_domestica
B4L9R7_Drosophila_mojavensis
Q29D49_Drosophila_pseudoobscura
B3M4F5_Drosophila_ananassae
0.7

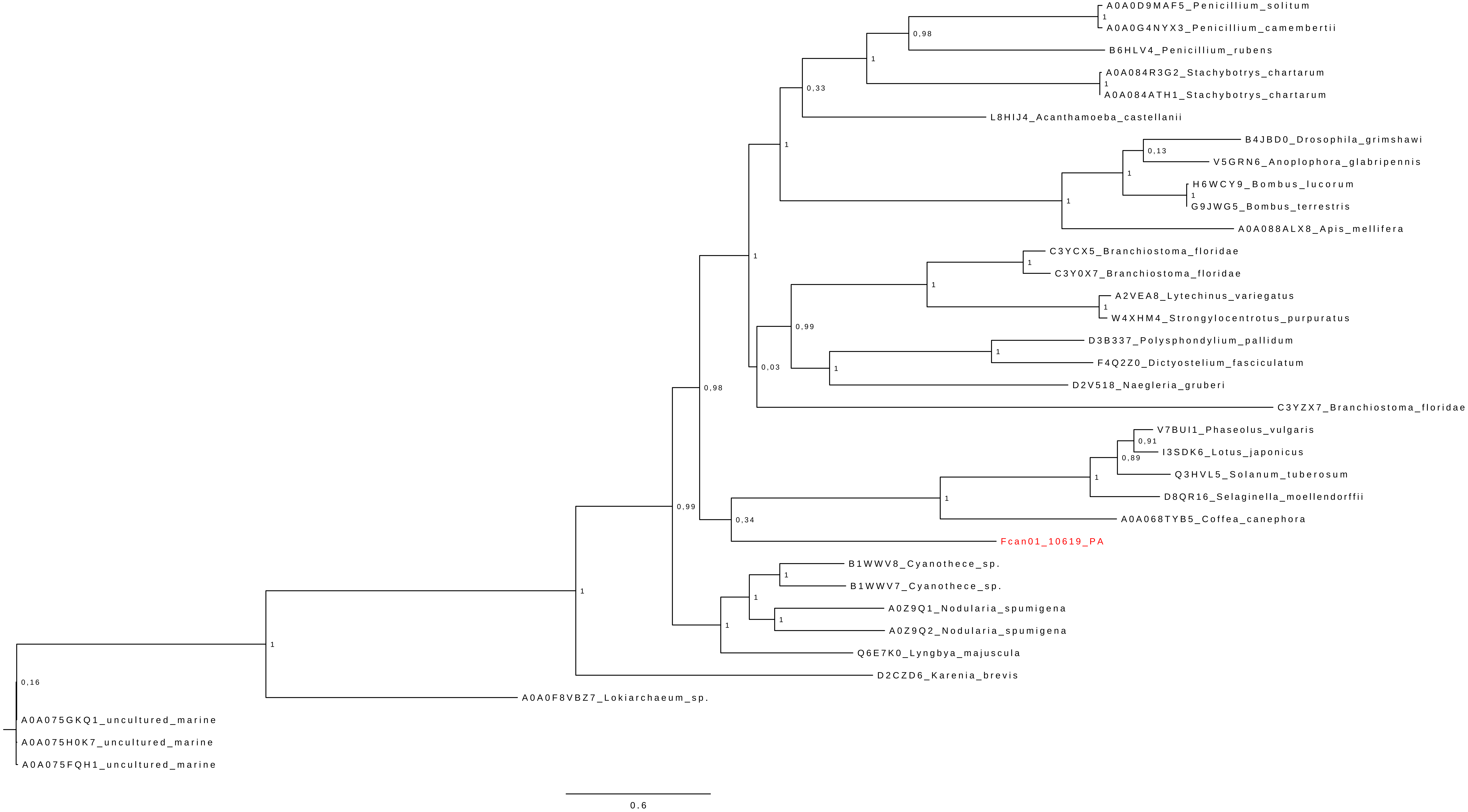
A0A0D9MAF5_Penicillium_solitum
A0A0G4NYX3_Penicillium_camembertii
B6HLV4_Penicillium_rubens
A0A084R3G2_Stachybotrys_chartarum
A0A084ATH1_Stachybotrys_chartarum
L8HIJ4_Acanthamoeba_castellanii
B4JBD0_Drosophila_grimshawi
V5GRN6_Anoplophora_glabripennis
H6WCY9_Bombus_lucorum
G9JWG5_Bombus_terrestris
A0A088ALX8_Apis_mellifera
C3YCX5_Branchiostoma_floridae
C3Y0X7_Branchiostoma_floridae
A2VEA8_Lytechinus_variegatus
W4XHM4_Strongylocentrotus_purpuratus
D3B337_Polysphondylium_pallidum
F4Q2Z0_Dictyostelium_fasciculatum
D2V518_Naegleria_gruberi
C3YZX7_Branchiostoma_floridae
V7BUI1_Phaseolus_vulgaris
I3SDK6_Lotus_japonicus
Q3HVL5_Solanum_tuberosum
D8QR16_Selaginella_moellendorffii
A0A068TYB5_Coffea_canephora
Fcan01_10619_PA
B1WWV8_Cyanothece_sp.
B1WWV7_Cyanothece_sp.
A0Z9Q1_Nodularia_spumigena
A0Z9Q2_Nodularia_spumigena
Q6E7K0_Lyngbya_majuscula
D2CZD6_Karenia_brevis
A0A0F8VBZ7_Lokiarchaeum_sp.
A0A075GKQ1_uncultured_marine
A0A075H0K7_uncultured_marine
A0A075FQH1_uncultured_marine
0,98
1
0,33
1
1
0,13
1
1
1
1
1
1
1
0,99
0,03
1
1
0,98
0,91
0,89
1
1
0,99
0,34
1
1
1
1
1
1
0,16
0.6

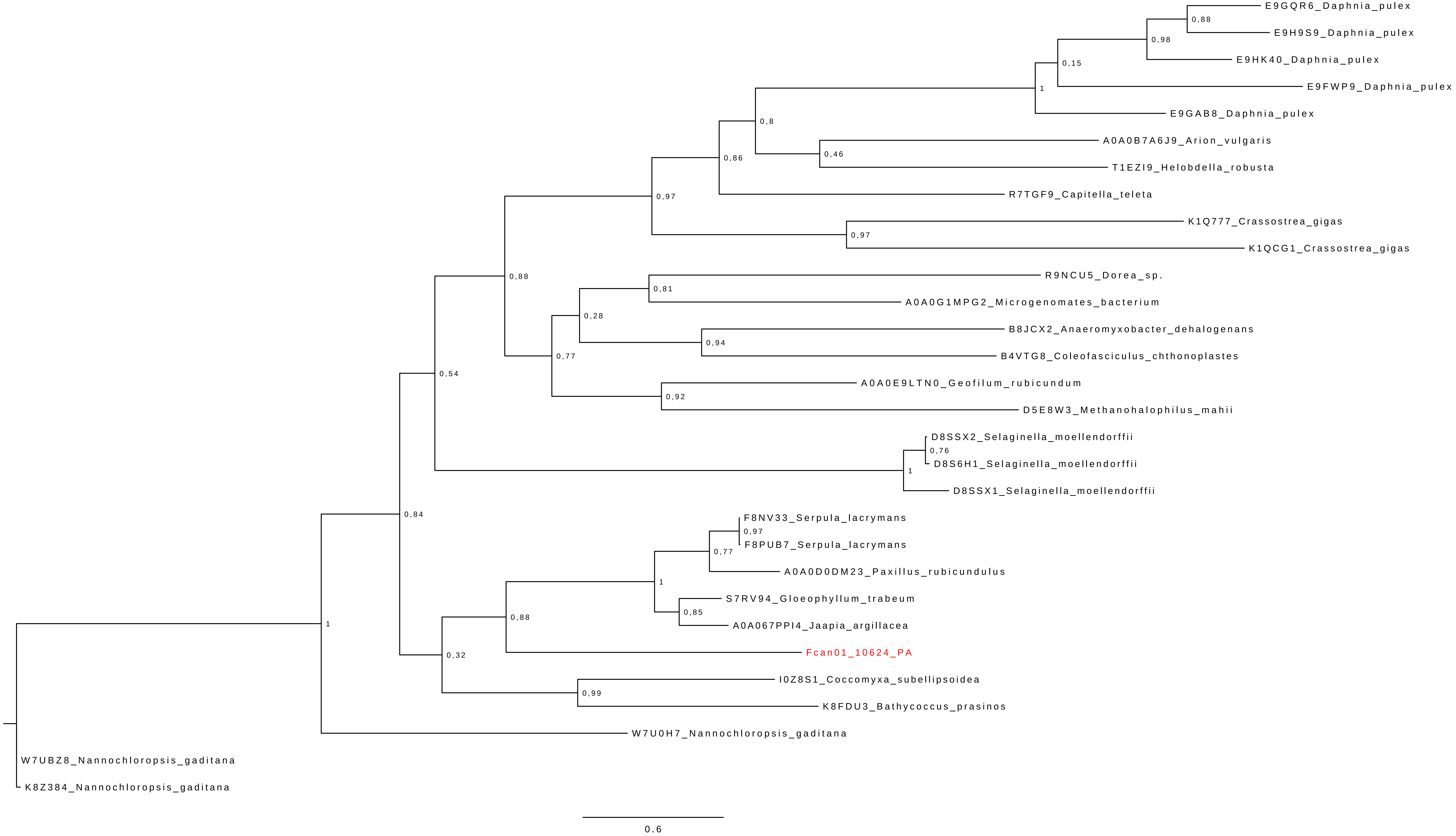
E9GQR6_Daphnia_pulex
0,88
E9H9S9_Daphnia_pulex
0,98
E9HK40_Daphnia_pulex
0,15
E9FWP9_Daphnia_pulex
1
E9GAB8_Daphnia_pulex
0,8
A0A0B7A6J9_Arion_vulgaris
0,46
T1EZI9_Helobdella_robusta
0,86
R7TGF9_Capitella_teleta
0,97
K1Q777_Crassostrea_gigas
0,97
K1QCG1_Crassostrea_gigas
0,88
R9NCU5_Dorea_sp.
0,81
A0A0G1MPG2_Microgenomates_bacterium
0,28
B8JCX2_Anaeromyxobacter_dehalogenans
0,94
B4VTG8_Coleofasciculus_chthonoplastes
0,77
A0A0E9LTN0_Geofilum_rubicundum
0,92
D5E8W3_Methanohalophilus_mahii
0,54
D8SSX2_Selaginella_moellendorffii
0,76
D8S6H1_Selaginella_moellendorffii
1
D8SSX1_Selaginella_moellendorffii
0,84
F8NV33_Serpula_lacrymans
0,97
F8PUB7_Serpula_lacrymans
0,77
A0A0D0DM23_Paxillus_rubicundulus
1
S7RV94_Gloeophyllum_trabeum
0,85
A0A067PPI4_Jaapia_argillacea
0,88
1
Fcan01_10624_PA
0,32
I0Z8S1_Coccomyxa_subellipsoidea
0,99
K8FDU3_Bathycoccus_prasinos
W7U0H7_Nannochloropsis_gaditana
W7UBZ8_Nannochloropsis_gaditana
K8Z384_Nannochloropsis_gaditana
0.6

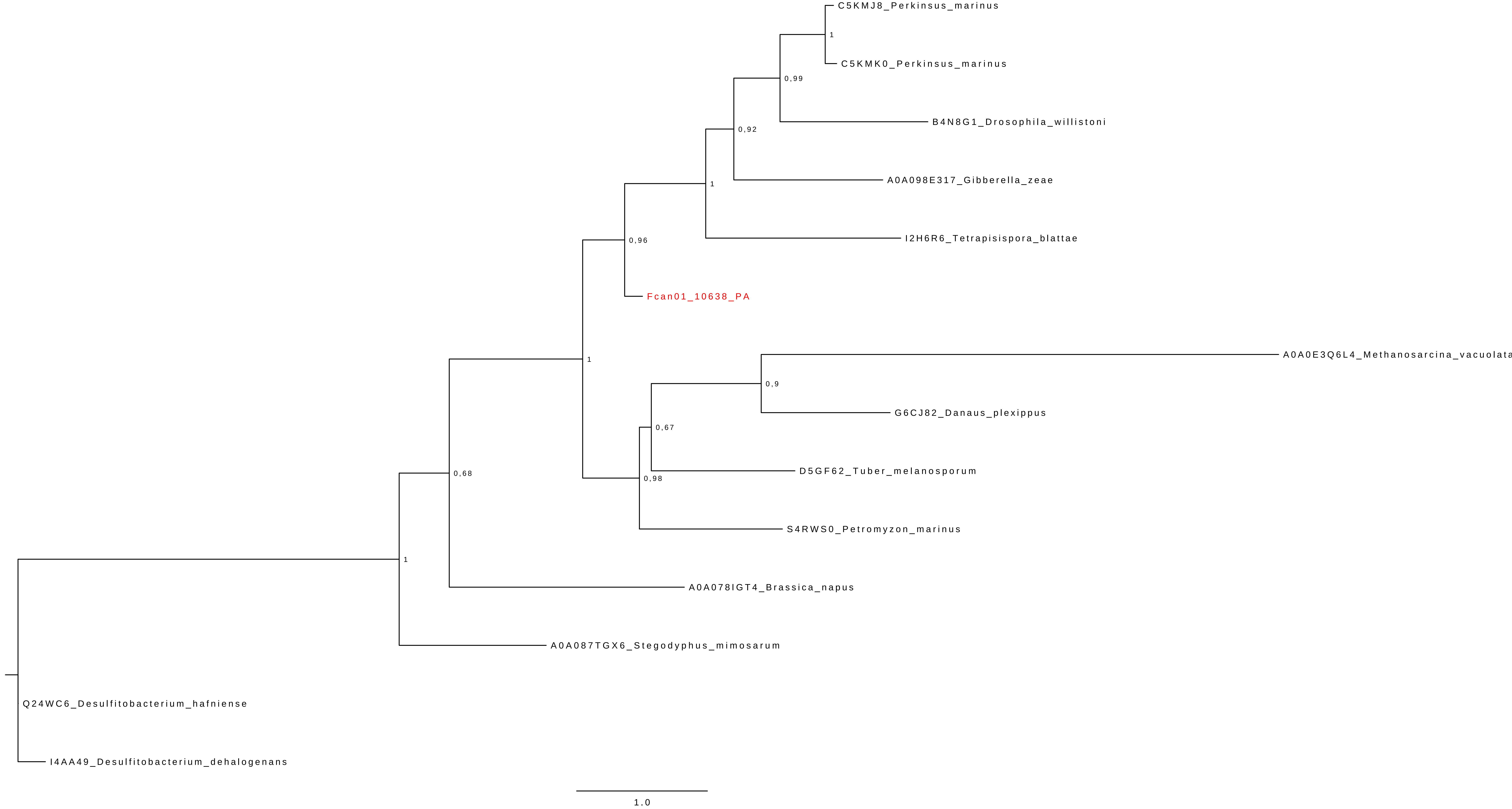
C5KMJ8_Perkinsus_marinus
1
C5KMK0_Perkinsus_marinus
0,99
B4N8G1_Drosophila_willistoni
0,92
A0A098E317_Gibberella_zeae
1
I2H6R6_Tetrapisispora_blattae
0,96
Fcan01_10638_PA
1
A0A0E3Q6L4_Methanosarcina_vacuolata
0,9
G6CJ82_Danaus_plexippus
0,67
D5GF62_Tuber_melanosporum
0,68
0,98
S4RWS0_Petromyzon_marinus
1
A0A078IGT4_Brassica_napus
A0A087TGX6_Stegodyphus_mimosarum
Q24WC6_Desulfitobacterium_hafniense
I4AA49_Desulfitobacterium_dehalogenans
1.0

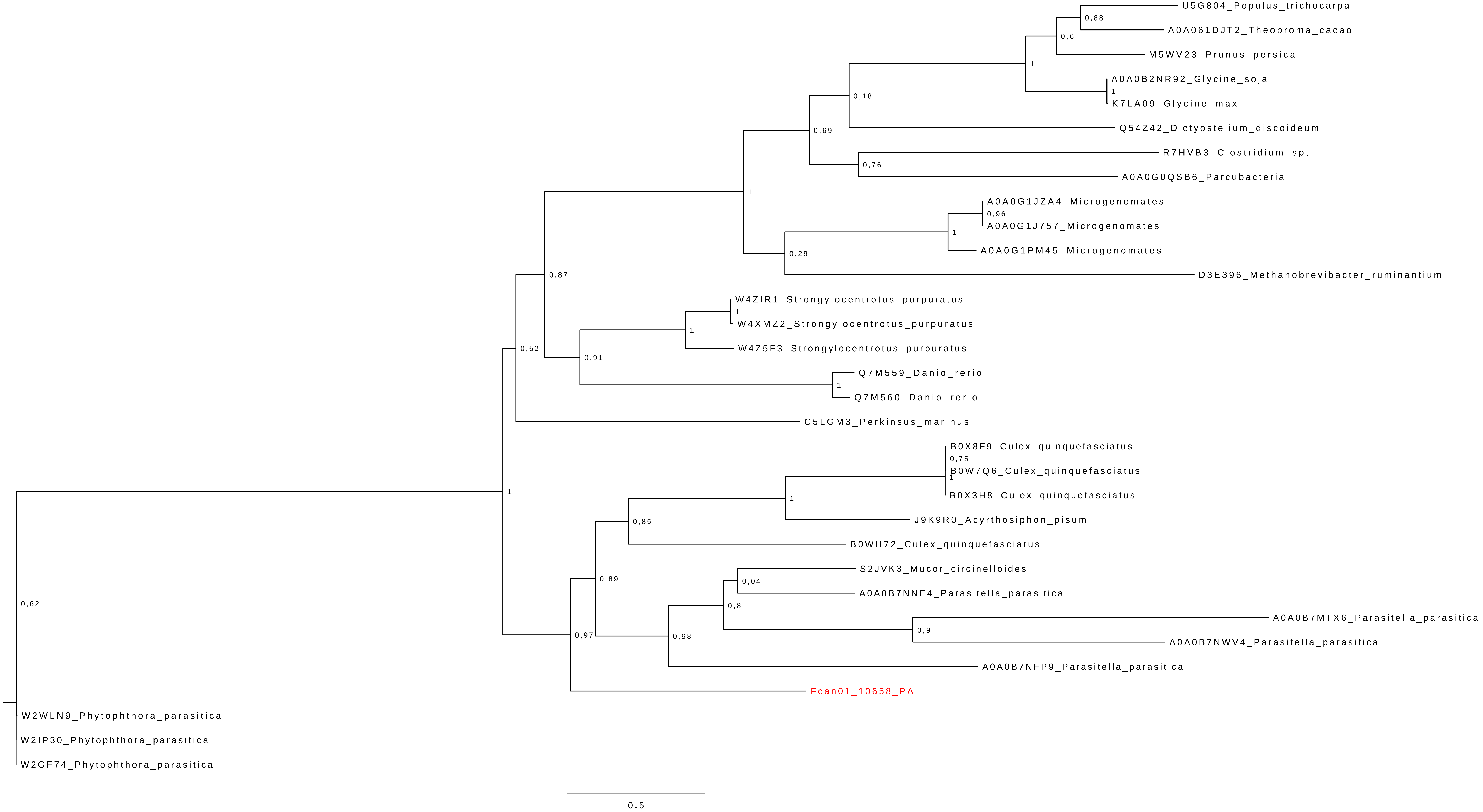

U5G804_Populus_trichocarpa
0,88
A0A061DJT2_Theobroma_cacao
0,6
M5WV23_Prunus_persica
1
A0A0B2NR92_Glycine_soja
1
K7LA09_Glycine_max
0,18
Q54Z42_Dictyostelium_discoideum
0,69
R7HVB3_Clostridium_sp.
0,76
A0A0G0QSB6_Parcubacteria
1
A0A0G1JZA4_Microgenomates
0,96
A0A0G1J757_Microgenomates
1
A0A0G1PM45_Microgenomates
0,29
D3E396_Methanobrevibacter_ruminantium
0,87
W4ZIR1_Strongylocentrotus_purpuratus
1
W4XMZ2_Strongylocentrotus_purpuratus
1
W4Z5F3_Strongylocentrotus_purpuratus
0,52
0,91
Q7M559_Danio_rerio
1
Q7M560_Danio_rerio
C5LGM3_Perkinsus_marinus
B0X8F9_Culex_quinquefasciatus
0,75
B0W7Q6_Culex_quinquefasciatus
1
B0X3H8_Culex_quinquefasciatus
1
1
J9K9R0_Acyrthosiphon_pisum
0,85
B0WH72_Culex_quinquefasciatus
S2JVK3_Mucor_circinelloides
0,04
A0A0B7NNE4_Parasitella_parasitica
0,62
0,8
0,89
A0A0B7MTX6_Parasitella_parasitica
0,9
0,97
0,98
A0A0B7NWV4_Parasitella_parasitica
A0A0B7NFP9_Parasitella_parasitica
Fcan01_10658_PA
W2WLN9_Phytophthora_parasitica
W2IP30_Phytophthora_parasitica
W2GF74_Phytophthora_parasitica
0.5

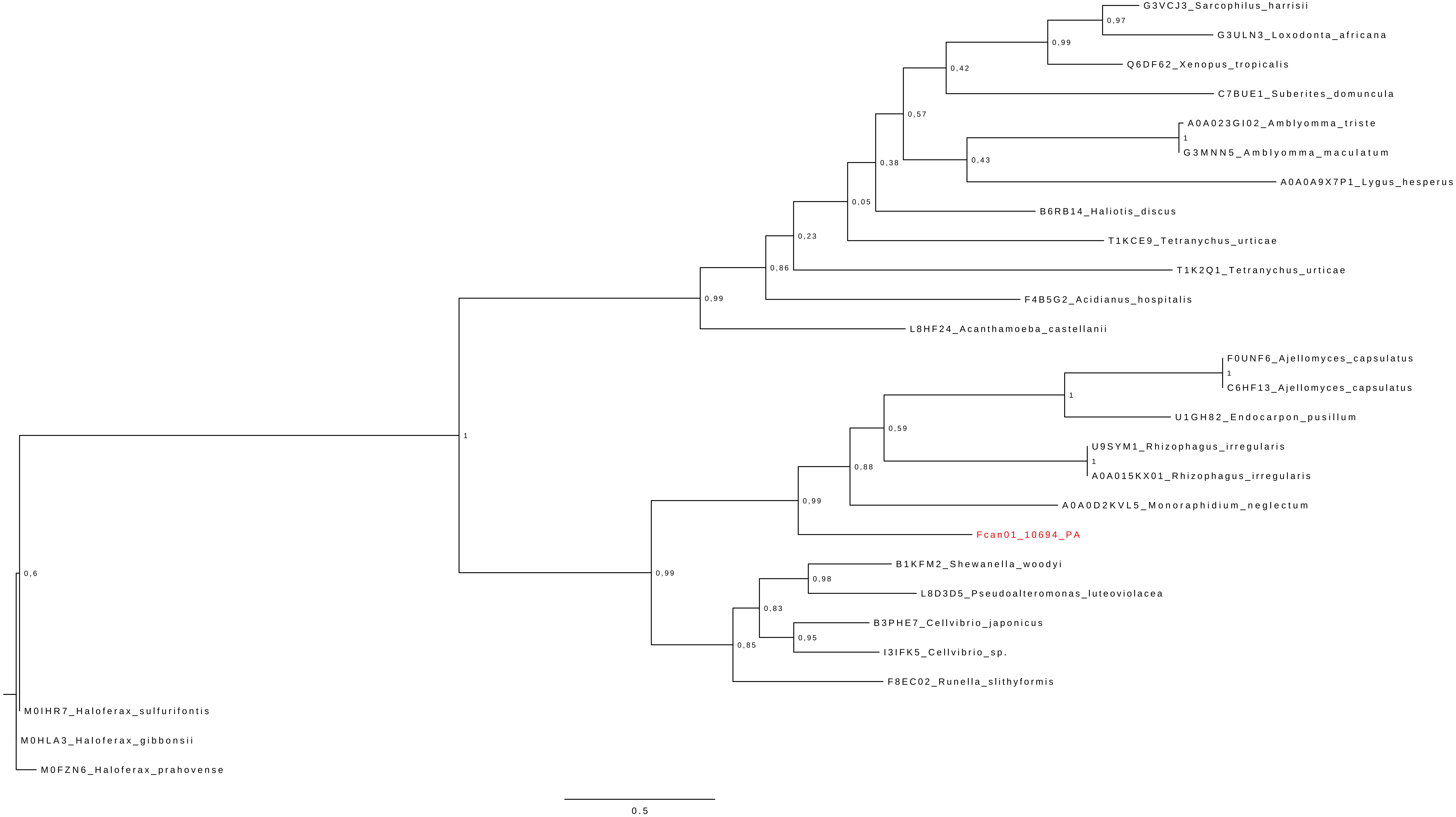
G3VCJ3_Sarcophilus_harrisii
0,97
G3ULN3_Loxodonta_africana
0,99
Q6DF62_Xenopus_tropicalis
0,42
C7BUE1_Suberites_domuncula
0,57
A0A023GI02_Amblyomma_triste
1
G3MNN5_Amblyomma_maculatum
0,43
A0A0A9X7P1_Lygus_hesperus
0,38
B6RB14_Haliotis_discus
0,05
T1KCE9_Tetranychus_urticae
0,23
T1K2Q1_Tetranychus_urticae
0,86
F4B5G2_Acidianus_hospitalis
0,99
L8HF24_Acanthamoeba_castellanii
F0UNF6_Ajellomyces_capsulatus
1
C6HF13_Ajellomyces_capsulatus
1
U1GH82_Endocarpon_pusillum
0,59
U9SYM1_Rhizophagus_irregularis
1
A0A015KX01_Rhizophagus_irregularis
0,88
A0A0D2KVL5_Monoraphidium_neglectum
0,99
Fcan01_10694_PA
1
B1KFM2_Shewanella_woodyi
0,98
L8D3D5_Pseudoalteromonas_luteoviolacea
0,83
B3PHE7_Cellvibrio_japonicus
0,95
I3IFK5_Cellvibrio_sp.
0,99
0,85
F8EC02_Runella_slithyformis
0,6
M0IHR7_Haloferax_sulfurifontis
M0HLA3_Haloferax_gibbonsii
M0FZN6_Haloferax_prahovense
0.5

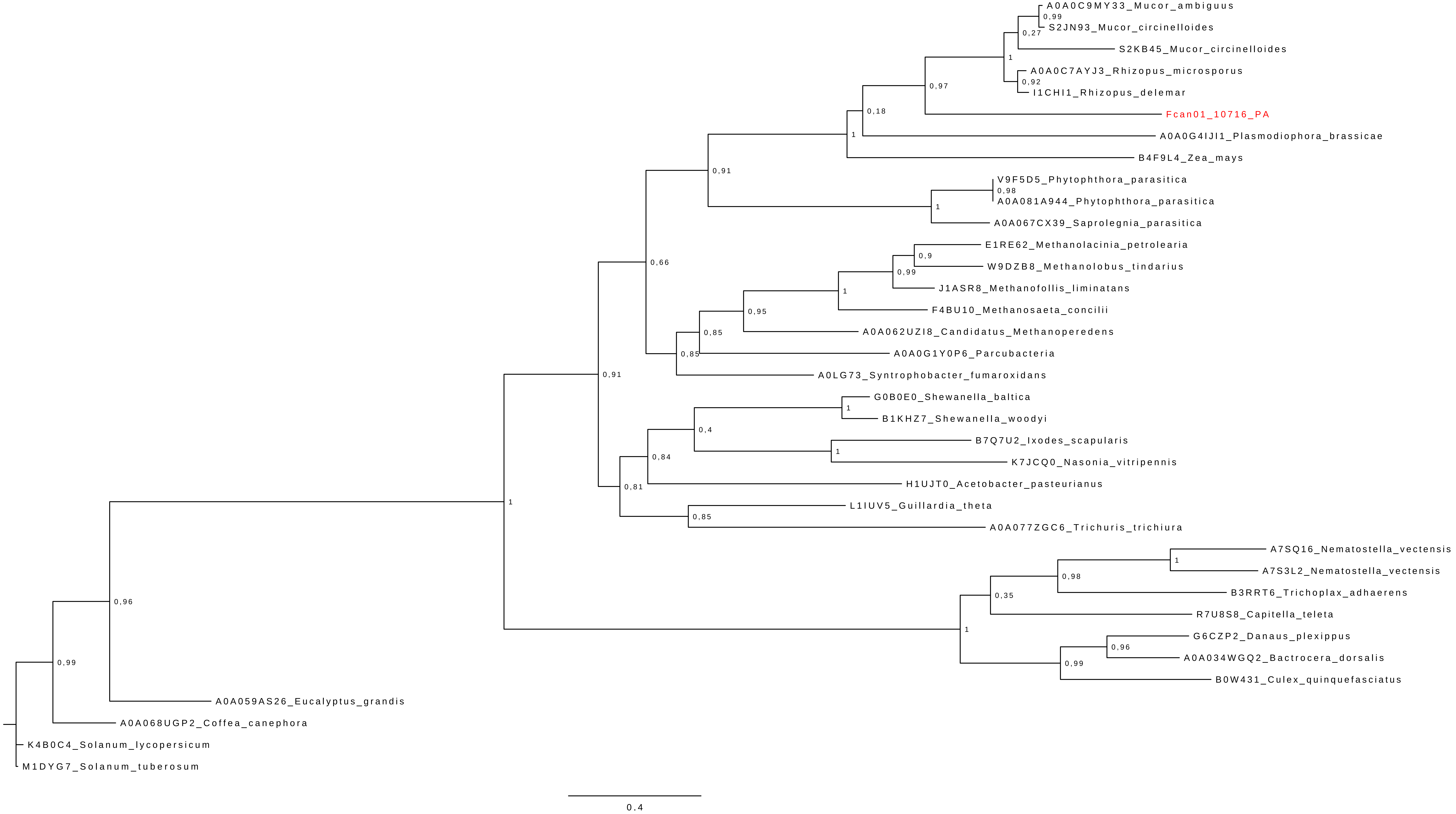
A0A0C9MY33_Mucor_ambiguus
0,99
S2JN93_Mucor_circinelloides
0,27
S2KB45_Mucor_circinelloides
1
A0A0C7AYJ3_Rhizopus_microsporus
0,92
I1CHI1_Rhizopus_delemar
0,97
Fcan01_10716_PA
0,18
1
A0A0G4IJI1_Plasmodiophora_brassicae
B4F9L4_Zea_mays
0,91
V9F5D5_Phytophthora_parasitica
0,98
A0A081A944_Phytophthora_parasitica
1
A0A067CX39_Saprolegnia_parasitica
E1RE62_Methanolacinia_petrolearia
0,9
W9DZB8_Methanolobus_tindarius
0,99
0,66
J1ASR8_Methanofollis_liminatans
1
F4BU10_Methanosaeta_concilii
0,95
A0A062UZI8_Candidatus_Methanoperedens
0,85
A0A0G1Y0P6_Parcubacteria
0,85
A0LG73_Syntrophobacter_fumaroxidans
0,91
G0B0E0_Shewanella_baltica
1
B1KHZ7_Shewanella_woodyi
0,4
B7Q7U2_Ixodes_scapularis
1
K7JCQ0_Nasonia_vitripennis
0,84
H1UJT0_Acetobacter_pasteurianus
0,81
1
L1IUV5_Guillardia_theta
0,85
A0A077ZGC6_Trichuris_trichiura
A7SQ16_Nematostella_vectensis
1
A7S3L2_Nematostella_vectensis
0,98
B3RRT6_Trichoplax_adhaerens
0,35
R7U8S8_Capitella_teleta
1
0,96
G6CZP2_Danaus_plexippus
0,96
A0A034WGQ2_Bactrocera_dorsalis
0,99
0,99
B0W431_Culex_quinquefasciatus
A0A059AS26_Eucalyptus_grandis
A0A068UGP2_Coffea_canephora
K4B0C4_Solanum_lycopersicum
M1DYG7_Solanum_tuberosum
0.4

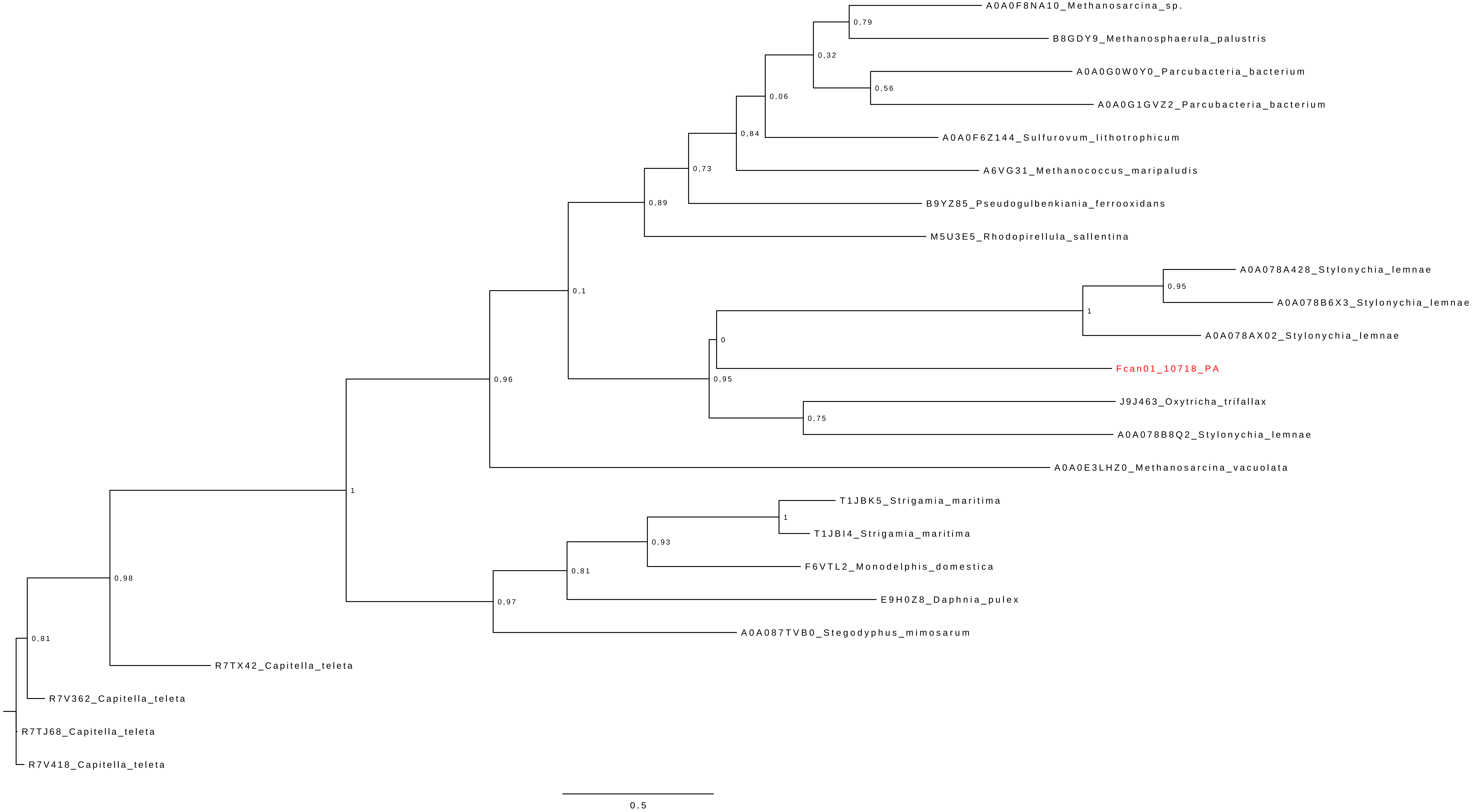
A0A0F8NA10_Methanosarcina_sp.
0,79
B8GDY9_Methanosphaerula_palustris
0,32
A0A0G0W0Y0_Parcubacteria_bacterium
0,56
A0A0G1GVZ2_Parcubacteria_bacterium
0,06
A0A0F6Z144_Sulfurovum_lithotrophicum
0,84
A6VG31_Methanococcus_maripaludis
0,73
B9YZ85_Pseudogulbenkiania_ferrooxidans
0,89
M5U3E5_Rhodopirellula_sallentina
A0A078A428_Stylonychia_lemnae
0,95
A0A078B6X3_Stylonychia_lemnae
0,1
1
A0A078AX02_Stylonychia_lemnae
0
Fcan01_10718_PA
0,95
J9J463_Oxytricha_trifallax
0,75
A0A078B8Q2_Stylonychia_lemnae
0,96
A0A0E3LHZ0_Methanosarcina_vacuolata
1
T1JBK5_Strigamia_maritima
1
T1JBI4_Strigamia_maritima
0,93
F6VTL2_Monodelphis_domestica
0,81
E9H0Z8_Daphnia_pulex
0,98
0,97
A0A087TVB0_Stegodyphus_mimosarum
0,81
R7TX42_Capitella_teleta
R7V362_Capitella_teleta
R7TJ68_Capitella_teleta
R7V418_Capitella_teleta
0.5

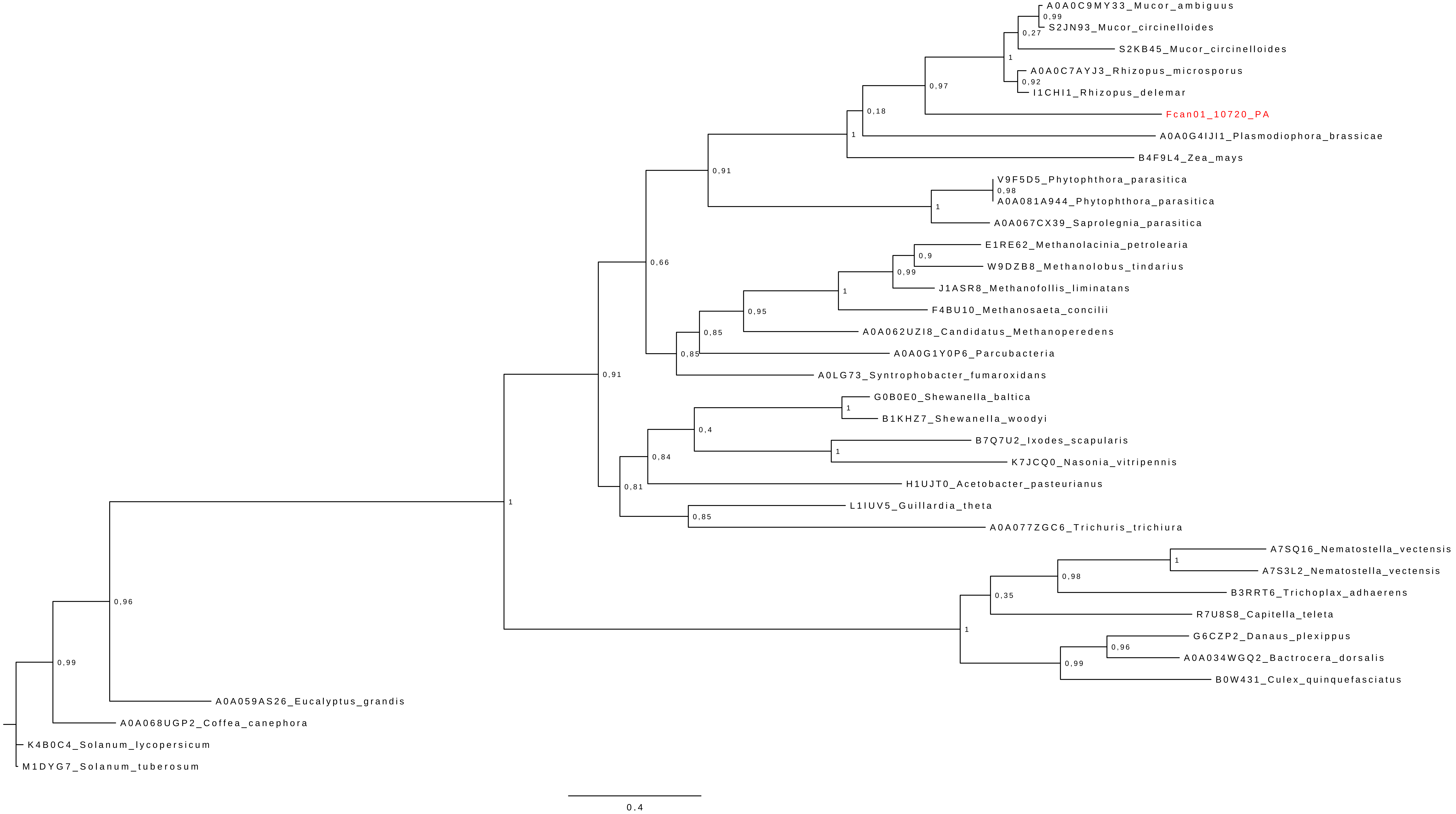
A0A0C9MY33_Mucor_ambiguus
S2JN93_Mucor_circinelloides
S2KB45_Mucor_circinelloides
A0A0C7AYJ3_Rhizopus_microsporus
I1CHI1_Rhizopus_delemar
Fcan01_10720_PA
A0A0G4IJI1_Plasmodiophora_brassicae
B4F9L4_Zea_mays
V9F5D5_Phytophthora_parasitica
A0A081A944_Phytophthora_parasitica
A0A067CX39_Saprolegnia_parasitica
E1RE62_Methanolacinia_petrolearia
W9DZB8_Methanolobus_tindarius
J1ASR8_Methanofollis_liminatans
F4BU10_Methanosaeta_concilii
A0A062UZI8_Candidatus_Methanoperedens
A0A0G1Y0P6_Parcubacteria
A0LG73_Syntrophobacter_fumaroxidans
G0B0E0_Shewanella_baltica
B1KHZ7_Shewanella_woodyi
B7Q7U2_Ixodes_scapularis
K7JCQ0_Nasonia_vitripennis
H1UJT0_Acetobacter_pasteurianus
L1IUV5_Guillardia_theta
A0A077ZGC6_Trichuris_trichiura
A7SQ16_Nematostella_vectensis
A7S3L2_Nematostella_vectensis
B3RRT6_Trichoplax_adhaerens
R7U8S8_Capitella_teleta
G6CZP2_Danaus_plexippus
A0A034WGQ2_Bactrocera_dorsalis
B0W431_Culex_quinquefasciatus
A0A059AS26_Eucalyptus_grandis
A0A068UGP2_Coffea_canephora
K4B0C4_Solanum_lycopersicum
M1DYG7_Solanum_tuberosum
0.4

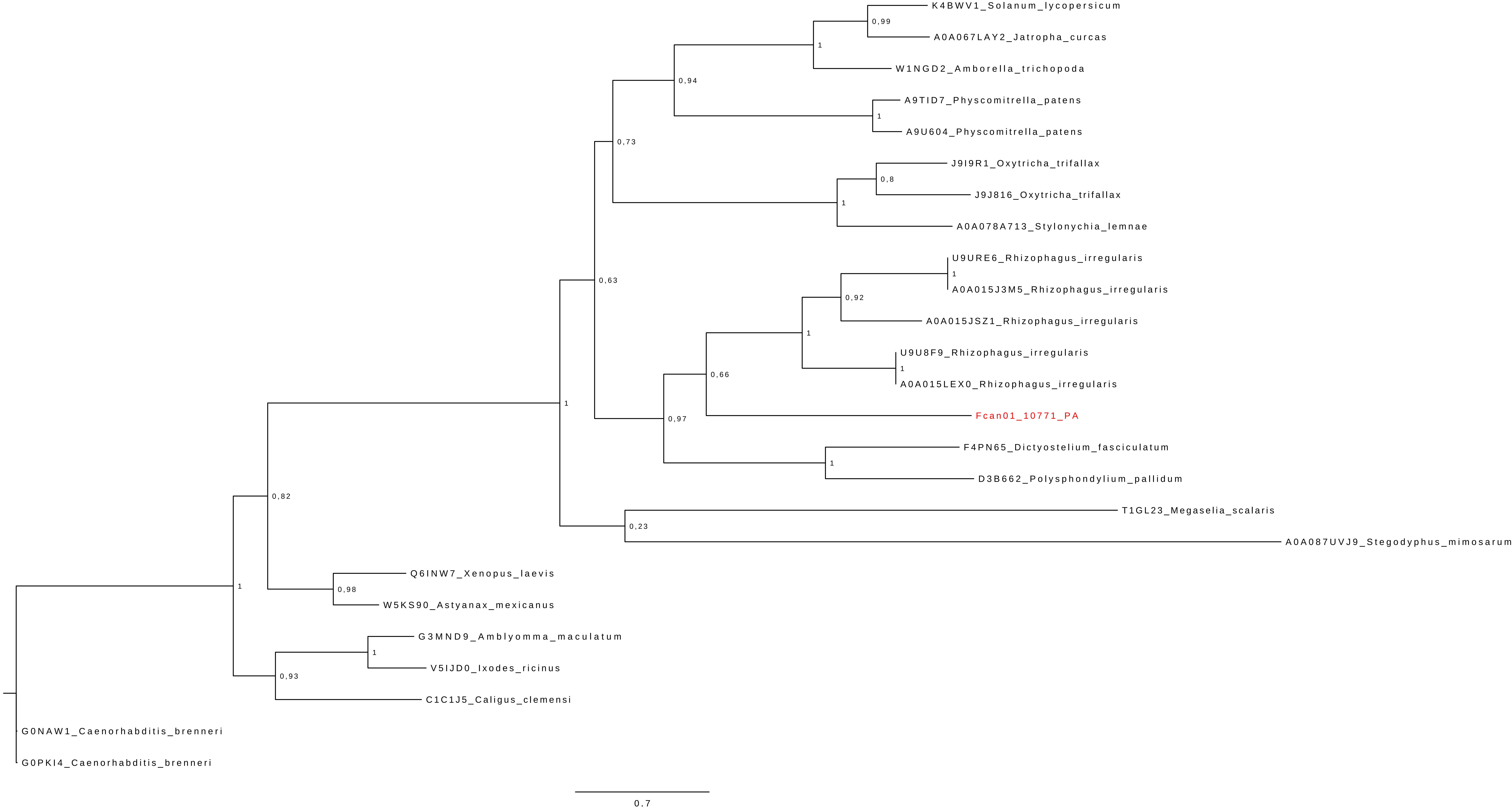

K4BWV1_Solanum_lycopersicum
0,99
A0A067LAY2_Jatropha_curcas
1
W1NGD2_Amborella_trichopoda
0,94
A9TID7_Physcomitrella_patens
1
A9U604_Physcomitrella_patens
0,73
J9I9R1_Oxytricha_trifallax
0,8
J9J816_Oxytricha_trifallax
1
A0A078A713_Stylonychia_lemnae
U9URE6_Rhizophagus_irregularis
1
A0A015J3M5_Rhizophagus_irregularis
0,63
0,92
A0A015JSZ1_Rhizophagus_irregularis
1
U9U8F9_Rhizophagus_irregularis
1
A0A015LEX0_Rhizophagus_irregularis
0,66
1
0,97
Fcan01_10771_PA
F4PN65_Dictyostelium_fasciculatum
1
D3B662_Polysphondylium_pallidum
0,82
T1GL23_Megaselia_scalaris
0,23
A0A087UVJ9_Stegodyphus_mimosarum
Q6INW7_Xenopus_laevis
0,98
1
W5KS90_Astyanax_mexicanus
G3MND9_Amblyomma_maculatum
1
V5IJD0_Ixodes_ricinus
0,93
C1C1J5_Caligus_clemensi
G0NAW1_Caenorhabditis_brenneri
G0PKI4_Caenorhabditis_brenneri
0.7

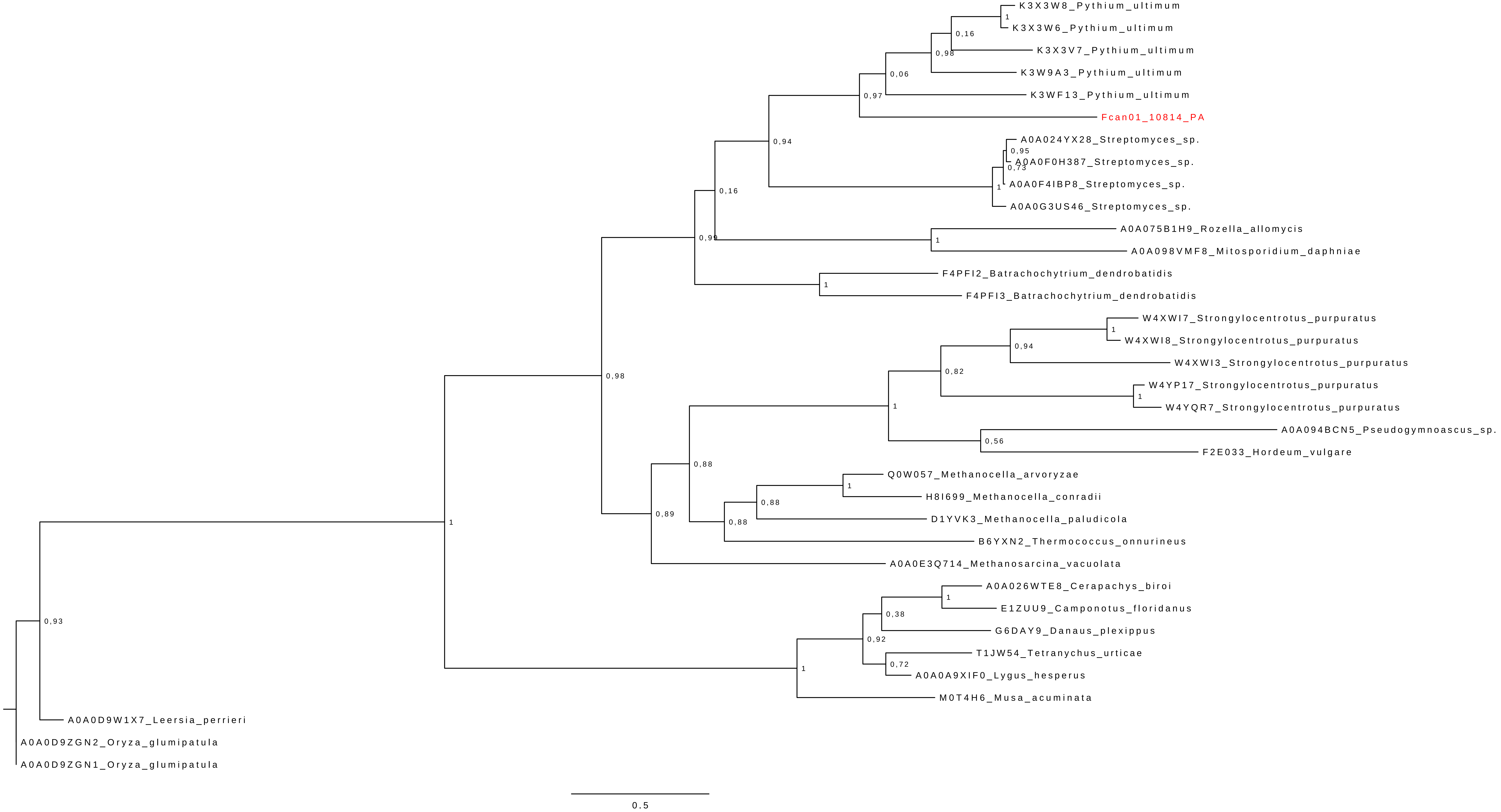
K3X3W8_Pythium_ultimum
K3X3W6_Pythium_ultimum
K3X3V7_Pythium_ultimum
K3W9A3_Pythium_ultimum
K3WF13_Pythium_ultimum
Fcan01_10814_PA
A0A024YX28_Streptomyces_sp.
A0A0F0H387_Streptomyces_sp.
A0A0F4IBP8_Streptomyces_sp.
A0A0G3US46_Streptomyces_sp.
A0A075B1H9_Rozella_allomycis
A0A098VMF8_Mitosporidium_daphniae
F4PFI2_Batrachochytrium_dendrobatidis
F4PFI3_Batrachochytrium_dendrobatidis
W4XWI7_Strongylocentrotus_purpuratus
W4XWI8_Strongylocentrotus_purpuratus
W4XWI3_Strongylocentrotus_purpuratus
W4YP17_Strongylocentrotus_purpuratus
W4YQR7_Strongylocentrotus_purpuratus
A0A094BCN5_Pseudogymnoascus_sp.
F2E033_Hordeum_vulgare
Q0W057_Methanocella_arvoryzae
H8I699_Methanocella_conradii
D1YVK3_Methanocella_paludicola
B6YXN2_Thermococcus_onnurineus
A0A0E3Q714_Methanosarcina_vacuolata
A0A026WTE8_Cerapachys_biroi
E1ZUU9_Camponotus_floridanus
G6DAY9_Danaus_plexippus
T1JW54_Tetranychus_urticae
A0A0A9XIF0_Lygus_hesperus
M0T4H6_Musa_acuminata
A0A0D9W1X7_Leersia_perrieri
A0A0D9ZGN2_Oryza_glumipatula
A0A0D9ZGN1_Oryza_glumipatula
1
0,16
0,98
0,06
0,97
0,94
0,95
0,73
1
0,16
0,99
1
1
0,98
1
0,94
0,82
1
1
0,56
0,88
1
0,88
0,88
0,89
1
1
0,38
0,92
0,72
1
0,93
0.5

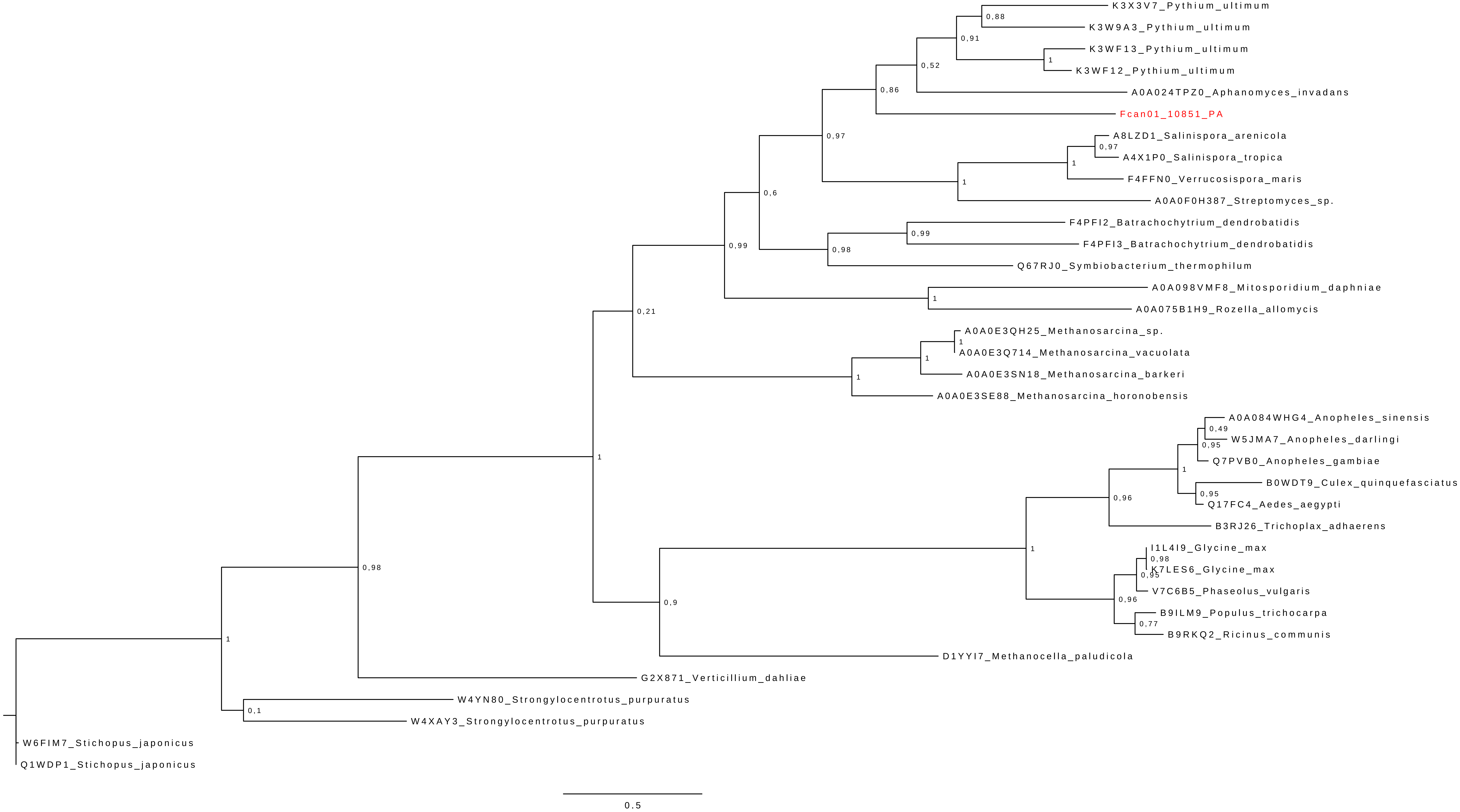
K3X3V7_Pythium_ultimum
K3W9A3_Pythium_ultimum
K3WF13_Pythium_ultimum
K3WF12_Pythium_ultimum
A0A024TPZ0_Aphanomyces_invadans
Fcan01_10851_PA
A8LZD1_Salinispora_arenicola
A4X1P0_Salinispora_tropica
F4FFN0_Verrucosispora_maris
A0A0F0H387_Streptomyces_sp.
F4PFI2_Batrachochytrium_dendrobatidis
F4PFI3_Batrachochytrium_dendrobatidis
Q67RJ0_Symbiobacterium_thermophilum
A0A098VMF8_Mitosporidium_daphniae
A0A075B1H9_Rozella_allomycis
A0A0E3QH25_Methanosarcina_sp.
A0A0E3Q714_Methanosarcina_vacuolata
A0A0E3SN18_Methanosarcina_barkeri
A0A0E3SE88_Methanosarcina_horonobensis
A0A084WHG4_Anopheles_sinensis
W5JMA7_Anopheles_darlingi
Q7PVB0_Anopheles_gambiae
B0WDT9_Culex_quinquefasciatus
Q17FC4_Aedes_aegypti
B3RJ26_Trichoplax_adhaerens
I1L4I9_Glycine_max
K7LES6_Glycine_max
V7C6B5_Phaseolus_vulgaris
B9ILM9_Populus_trichocarpa
B9RKQ2_Ricinus_communis
D1YYI7_Methanocella_paludicola
G2X871_Verticillium_dahliae
W4YN80_Strongylocentrotus_purpuratus
W4XAY3_Strongylocentrotus_purpuratus
W6FIM7_Stichopus_japonicus
Q1WDP1_Stichopus_japonicus
0.5

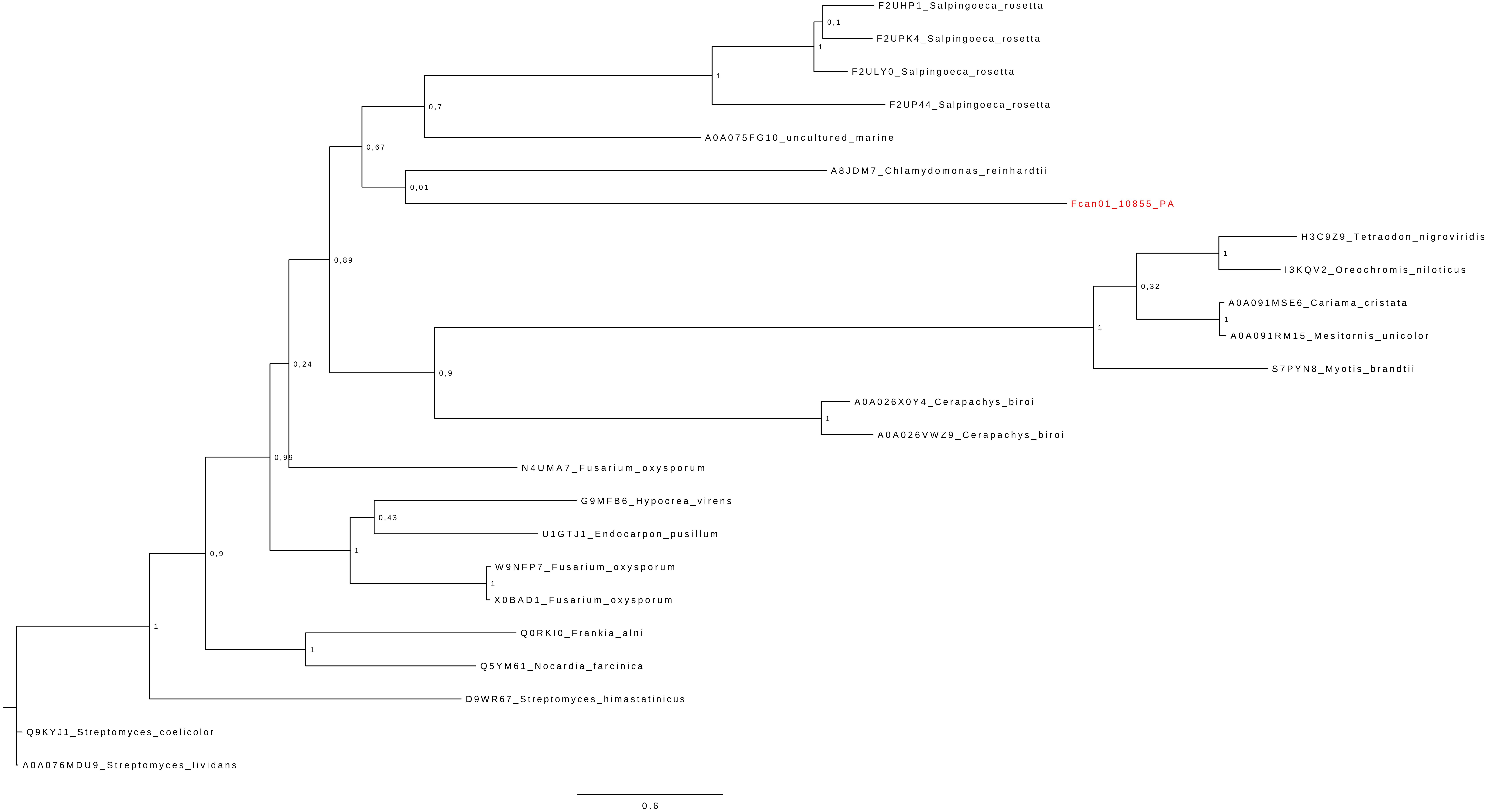
F2UHP1_Salpingoeca_rosetta
0,1
F2UPK4_Salpingoeca_rosetta
1
F2ULY0_Salpingoeca_rosetta
1
F2UP44_Salpingoeca_rosetta
0,7
A0A075FG10_uncultured_marine
0,67
A8JDM7_Chlamydomonas_reinhardtii
0,01
Fcan01_10855_PA
H3C9Z9_Tetraodon_nigroviridis
1
I3KQV2_Oreochromis_niloticus
0,32
A0A091MSE6_Cariama_cristata
1
A0A091RM15_Mesitornis_unicolor
1
0,89
S7PYN8_Myotis_brandtii
0,24
0,9
A0A026X0Y4_Cerapachys_biroi
1
A0A026VWZ9_Cerapachys_biroi
0,99
N4UMA7_Fusarium_oxysporum
G9MFB6_Hypocrea_virens
0,43
U1GTJ1_Endocarpon_pusillum
0,9
1
W9NFP7_Fusarium_oxysporum
1
X0BAD1_Fusarium_oxysporum
1
Q0RKI0_Frankia_alni
1
Q5YM61_Nocardia_farcinica
D9WR67_Streptomyces_himastatinicus
Q9KYJ1_Streptomyces_coelicolor
A0A076MDU9_Streptomyces_lividans
0.6

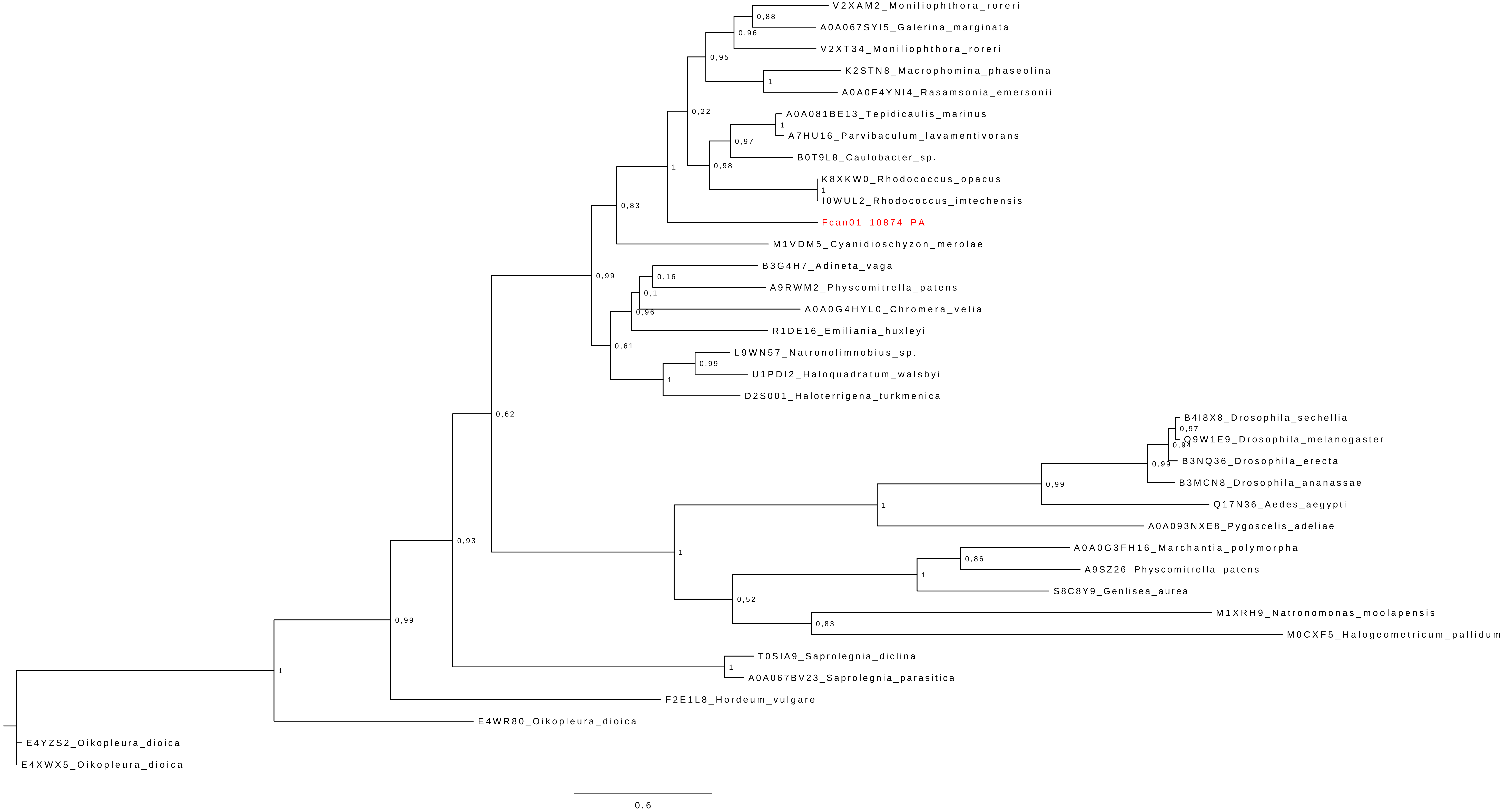

V2XAM2_Moniliophthora_roreri
0,88
A0A067SYI5_Galerina_marginata
0,96
V2XT34_Moniliophthora_roreri
0,95
K2STN8_Macrophomina_phaseolina
1
A0A0F4YNI4_Rasamsonia_emersonii
0,22
A0A081BE13_Tepidicaulis_marinus
1
A7HU16_Parvibaculum_lavamentivorans
0,97
B0T9L8_Caulobacter_sp.
1
0,98
K8XKW0_Rhodococcus_opacus
1
I0WUL2_Rhodococcus_imtechensis
0,83
Fcan01_10874_PA
M1VDM5_Cyanidioschyzon_merolae
B3G4H7_Adineta_vaga
0,16
0,99
A9RWM2_Physcomitrella_patens
0,1
A0A0G4HYL0_Chromera_velia
0,96
R1DE16_Emiliania_huxleyi
0,61
L9WN57_Natronolimnobius_sp.
0,99
U1PDI2_Haloquadratum_walsbyi
1
D2S001_Haloterrigena_turkmenica
0,62
B4I8X8_Drosophila_sechellia
0,97
Q9W1E9_Drosophila_melanogaster
0,94
B3NQ36_Drosophila_erecta
0,99
B3MCN8_Drosophila_ananassae
0,99
Q17N36_Aedes_aegypti
1
A0A093NXE8_Pygoscelis_adeliae
0,93
A0A0G3FH16_Marchantia_polymorpha
1
0,86
A9SZ26_Physcomitrella_patens
1
S8C8Y9_Genlisea_aurea
0,52
M1XRH9_Natronomonas_moolapensis
0,99
0,83
M0CXF5_Halogeometricum_pallidum
T0SIA9_Saprolegnia_diclina
1
1
A0A067BV23_Saprolegnia_parasitica
F2E1L8_Hordeum_vulgare
E4WR80_Oikopleura_dioica
E4YZS2_Oikopleura_dioica
E4XWX5_Oikopleura_dioica
0.6

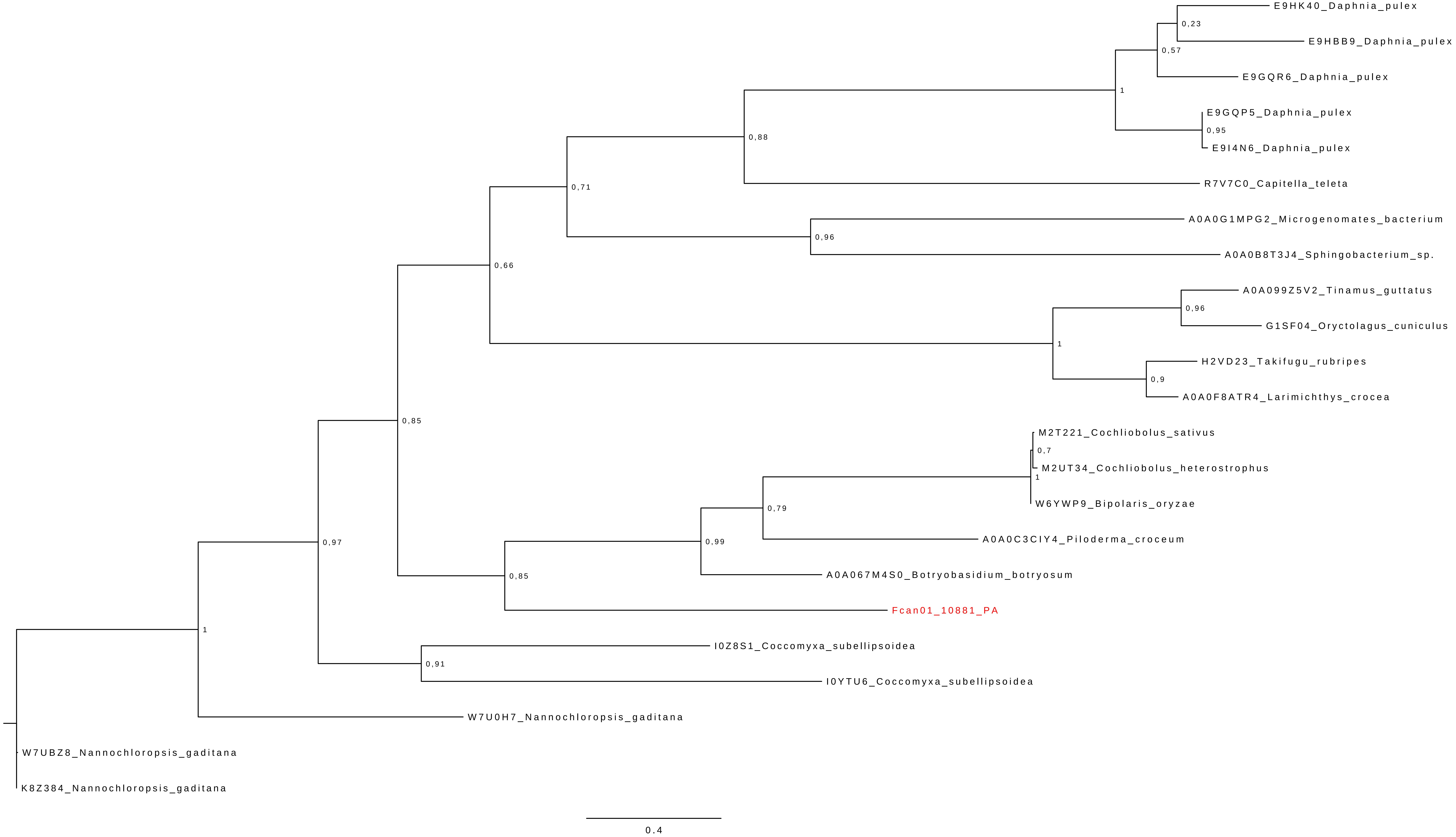
E9HK40_Daphnia_pulex
0,23
E9HBB9_Daphnia_pulex
0,57
E9GQR6_Daphnia_pulex
1
E9GQP5_Daphnia_pulex
0,95
E9I4N6_Daphnia_pulex
0,88
R7V7C0_Capitella_teleta
0,71
A0A0G1MPG2_Microgenomates_bacterium
0,96
A0A0B8T3J4_Sphingobacterium_sp.
0,66
A0A099Z5V2_Tinamus_guttatus
0,96
G1SF04_Oryctolagus_cuniculus
1
H2VD23_Takifugu_rubripes
0,9
A0A0F8ATR4_Larimichthys_crocea
0,85
M2T221_Cochliobolus_sativus
0,7
M2UT34_Cochliobolus_heterostrophus
1
W6YWP9_Bipolaris_oryzae
0,79
A0A0C3CIY4_Piloderma_croceum
0,97
0,99
0,85
A0A067M4S0_Botryobasidium_botryosum
Fcan01_10881_PA
1
I0Z8S1_Coccomyxa_subellipsoidea
0,91
I0YTU6_Coccomyxa_subellipsoidea
W7U0H7_Nannochloropsis_gaditana
W7UBZ8_Nannochloropsis_gaditana
K8Z384_Nannochloropsis_gaditana
0.4

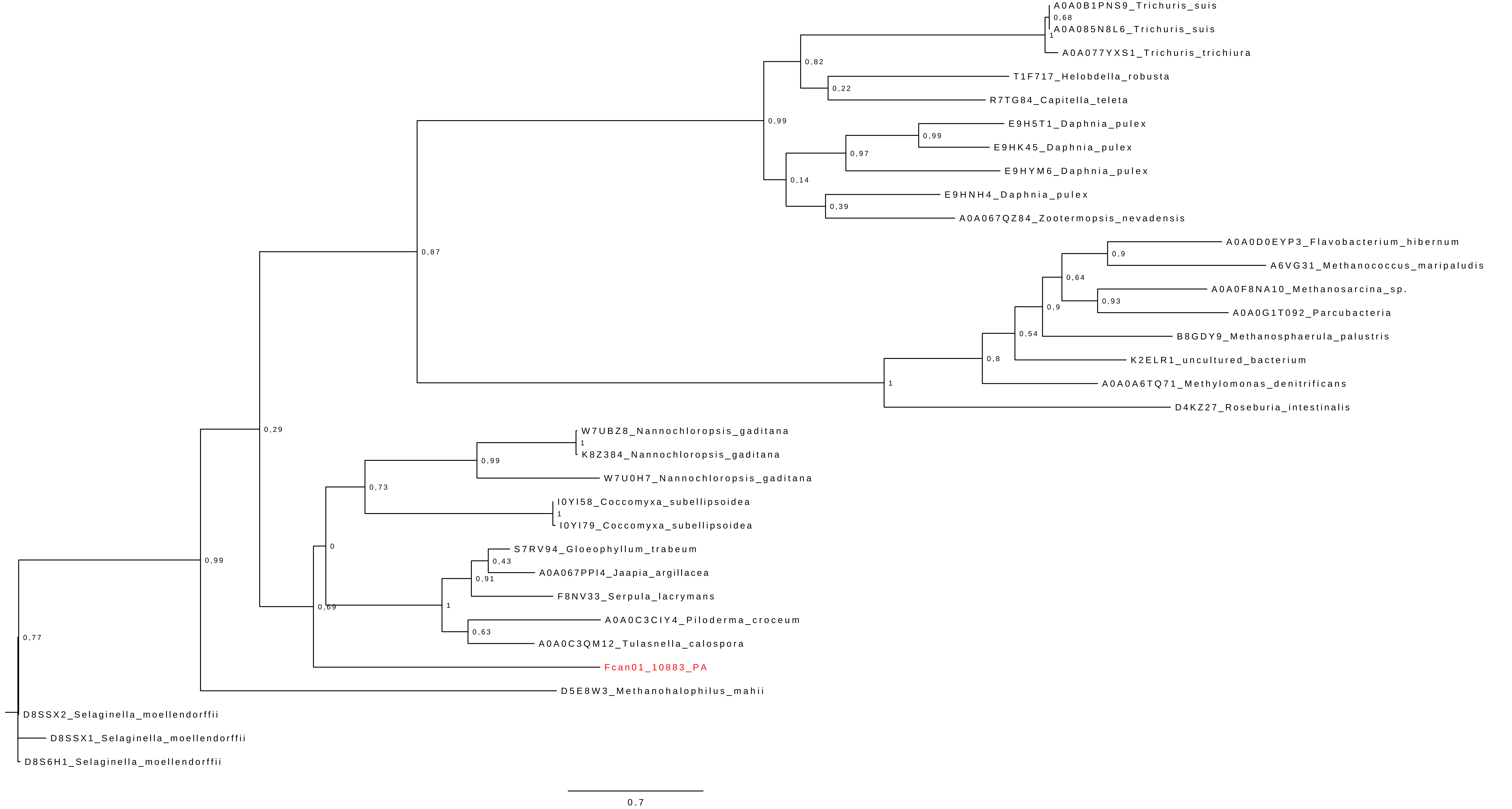
A0A0B1PNS9_Trichuris_suis
0,68
A0A085N8L6_Trichuris_suis
1
A0A077YXS1_Trichuris_trichiura
0,82
T1F717_Helobdella_robusta
0,22
R7TG84_Capitella_teleta
0,99
E9H5T1_Daphnia_pulex
0,99
E9HK45_Daphnia_pulex
0,97
E9HYM6_Daphnia_pulex
0,14
E9HNH4_Daphnia_pulex
0,39
A0A067QZ84_Zootermopsis_nevadensis
0,87
A0A0D0EYP3_Flavobacterium_hibernum
0,9
A6VG31_Methanococcus_maripaludis
0,64
A0A0F8NA10_Methanosarcina_sp.
0,93
0,9
A0A0G1T092_Parcubacteria
0,54
B8GDY9_Methanosphaerula_palustris
0,8
K2ELR1_uncultured_bacterium
1
A0A0A6TQ71_Methylomonas_denitrificans
D4KZ27_Roseburia_intestinalis
W7UBZ8_Nannochloropsis_gaditana
0,29
1
K8Z384_Nannochloropsis_gaditana
0,99
W7U0H7_Nannochloropsis_gaditana
0,73
I0YI58_Coccomyxa_subellipsoidea
1
I0YI79_Coccomyxa_subellipsoidea
0
S7RV94_Gloeophyllum_trabeum
0,43
0,99
A0A067PPI4_Jaapia_argillacea
0,91
F8NV33_Serpula_lacrymans
0,69
1
A0A0C3CIY4_Piloderma_croceum
0,77
0,63
A0A0C3QM12_Tulasnella_calospora
Fcan01_10883_PA
D5E8W3_Methanohalophilus_mahii
D8SSX2_Selaginella_moellendorffii
D8SSX1_Selaginella_moellendorffii
D8S6H1_Selaginella_moellendorffii
0.7

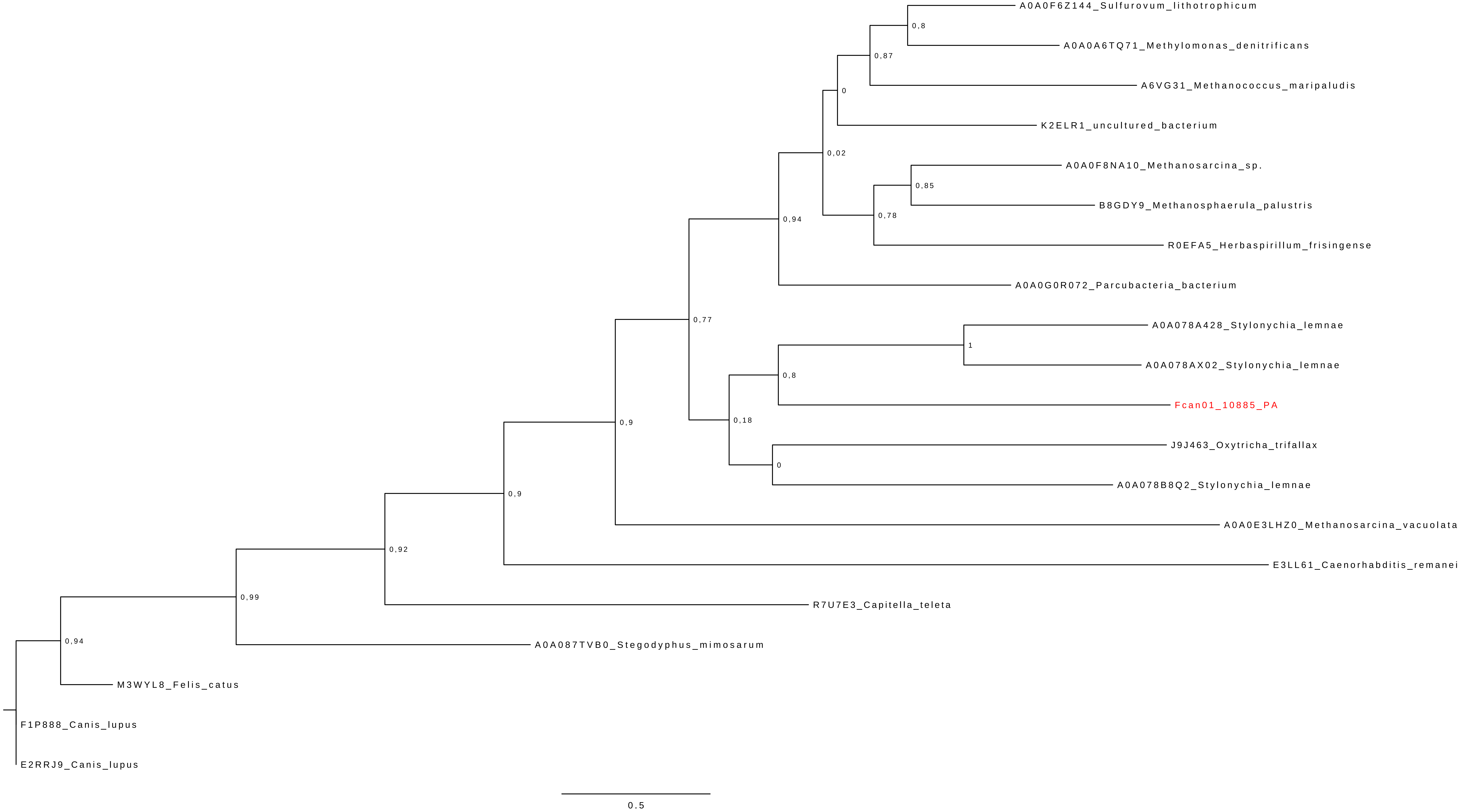
A0A0F6Z144_Sulfurovum_lithotrophicum
0,8
A0A0A6TQ71_Methylomonas_denitrificans
0,87
A6VG31_Methanococcus_maripaludis
0
K2ELR1_uncultured_bacterium
0,02
A0A0F8NA10_Methanosarcina_sp.
0,85
B8GDY9_Methanosphaerula_palustris
0,78
R0EFA5_Herbaspirillum_frisingense
0,94
A0A0G0R072_Parcubacteria_bacterium
0,77
A0A078A428_Stylonychia_lemnae
1
A0A078AX02_Stylonychia_lemnae
0,8
Fcan01_10885_PA
0,18
0,9
J9J463_Oxytricha_trifallax
0
A0A078B8Q2_Stylonychia_lemnae
0,9
A0A0E3LHZ0_Methanosarcina_vacuolata
0,92
E3LL61_Caenorhabditis_remanei
0,99
R7U7E3_Capitella_teleta
0,94
A0A087TVB0_Stegodyphus_mimosarum
M3WYL8_Felis_catus
F1P888_Canis_lupus
E2RRJ9_Canis_lupus
0.5

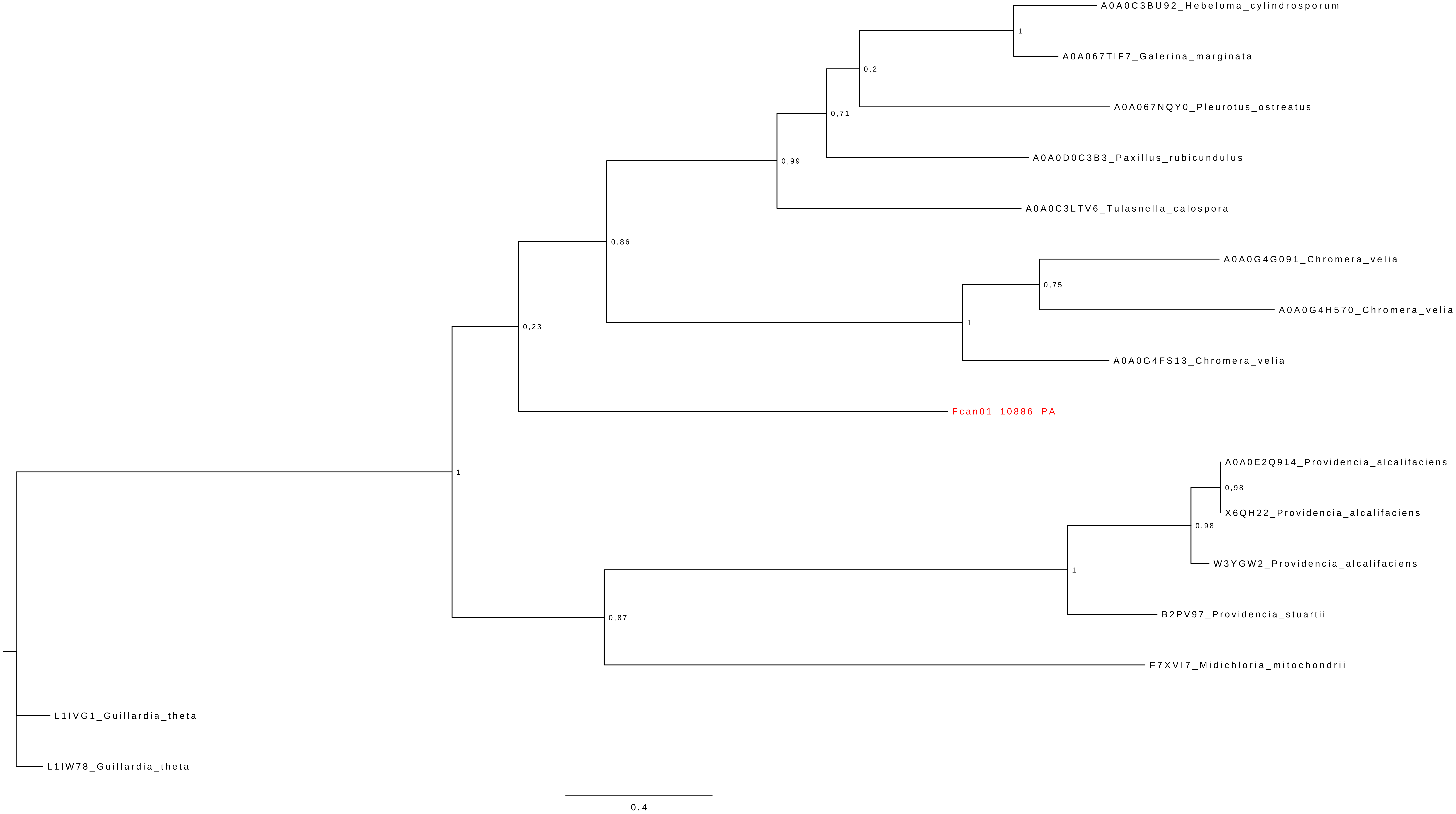
A0A0C3BU92_Hebeloma_cylindrosporum
1
A0A067TIF7_Galerina_marginata
0,2
A0A067NQY0_Pleurotus_ostreatus
0,71
A0A0D0C3B3_Paxillus_rubicundulus
0,99
A0A0C3LTV6_Tulasnella_calospora
0,86
A0A0G4G091_Chromera_velia
0,75
A0A0G4H570_Chromera_velia
1
0,23
A0A0G4FS13_Chromera_velia
Fcan01_10886_PA
A0A0E2Q914_Providencia_alcalifaciens
1
0,98
X6QH22_Providencia_alcalifaciens
0,98
W3YGW2_Providencia_alcalifaciens
1
B2PV97_Providencia_stuartii
0,87
F7XVI7_Midichloria_mitochondrii
L1IVG1_Guillardia_theta
L1IW78_Guillardia_theta
0.4

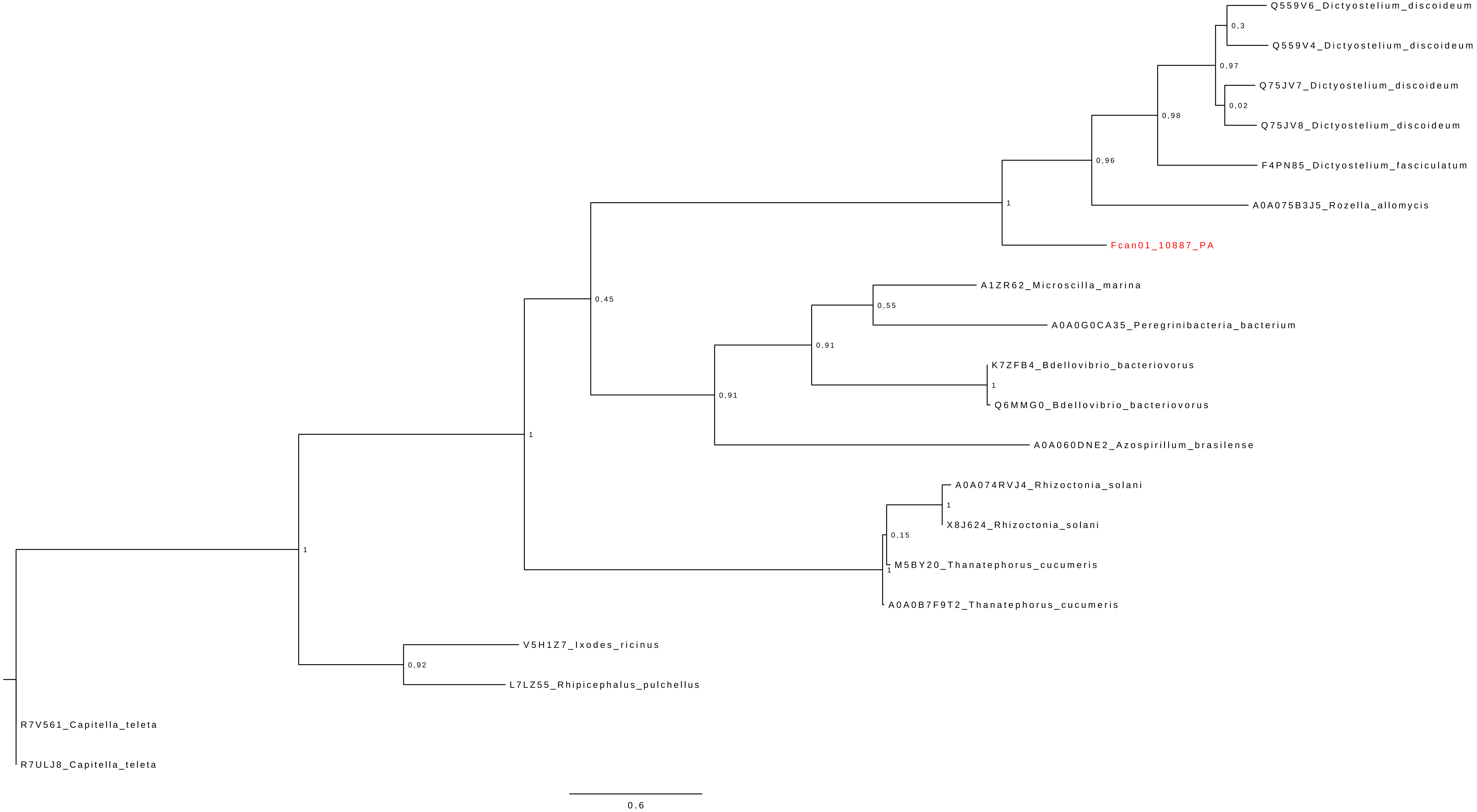

Q559V6_Dictyostelium_discoideum
0,3
Q559V4_Dictyostelium_discoideum
0,97
Q75JV7_Dictyostelium_discoideum
0,02
Q75JV8_Dictyostelium_discoideum
0,98
F4PN85_Dictyostelium_fasciculatum
0,96
A0A075B3J5_Rozella_allomycis
1
Fcan01_10887_PA
A1ZR62_Microscilla_marina
0,45
0,55
A0A0G0CA35_Peregrinibacteria_bacterium
0,91
K7ZFB4_Bdellovibrio_bacteriovorus
1
Q6MMG0_Bdellovibrio_bacteriovorus
0,91
A0A060DNE2_Azospirillum_brasilense
1
A0A074RVJ4_Rhizoctonia_solani
1
X8J624_Rhizoctonia_solani
0,15
M5BY20_Thanatephorus_cucumeris
1
A0A0B7F9T2_Thanatephorus_cucumeris
1
V5H1Z7_Ixodes_ricinus
0,92
L7LZ55_Rhipicephalus_pulchellus
R7V561_Capitella_teleta
R7ULJ8_Capitella_teleta
0.6

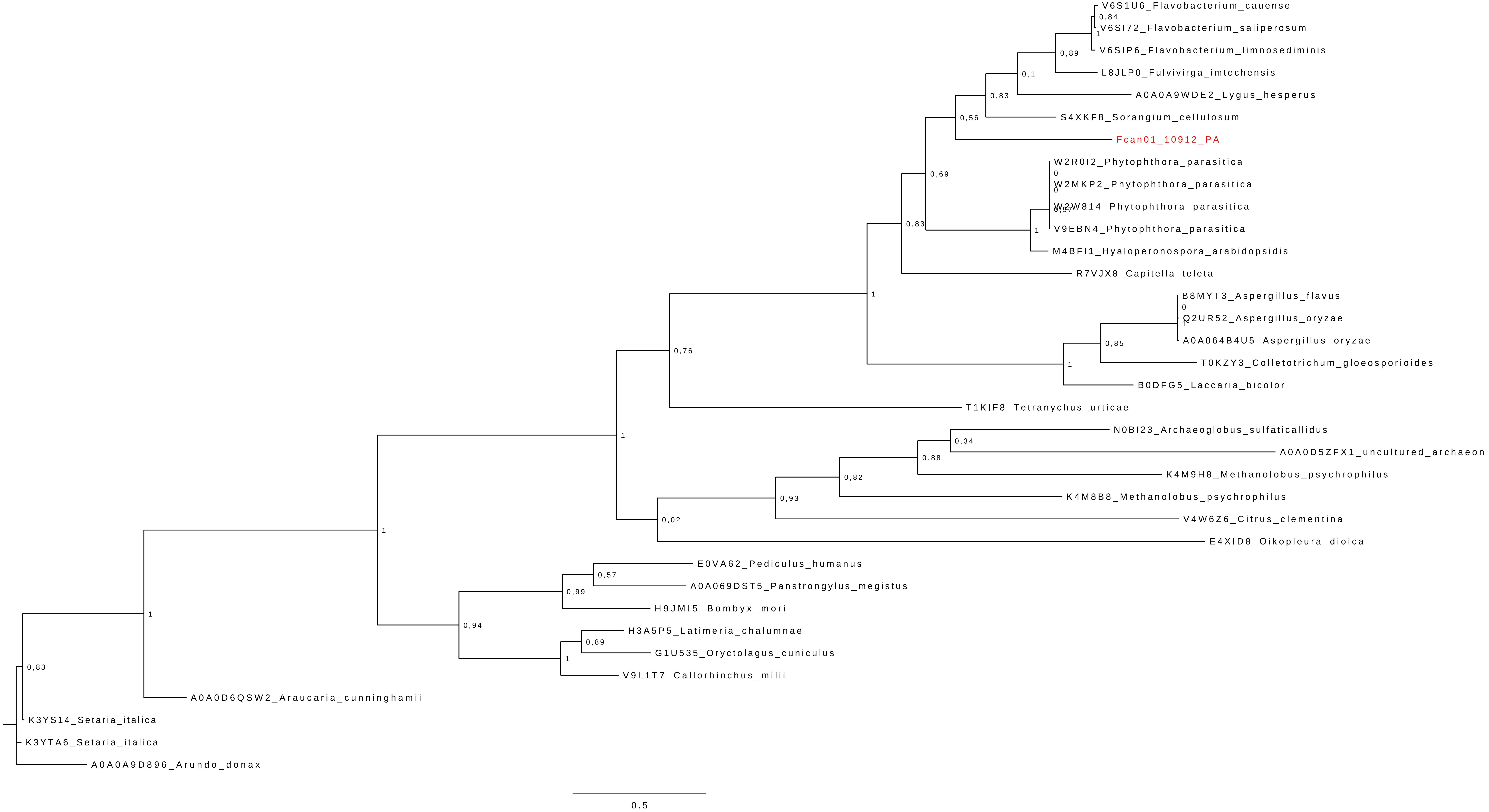
V6S1U6_Flavobacterium_cauense
V6SI72_Flavobacterium_saliperosum
V6SIP6_Flavobacterium_limnosediminis
L8JLP0_Fulvivirga_imtechensis
A0A0A9WDE2_Lygus_hesperus
S4XKF8_Sorangium_cellulosum
Fcan01_10912_PA
W2R0I2_Phytophthora_parasitica
W2MKP2_Phytophthora_parasitica
W2W814_Phytophthora_parasitica
V9EBN4_Phytophthora_parasitica
M4BFI1_Hyaloperonospora_arabidopsidis
R7VJX8_Capitella_teleta
B8MYT3_Aspergillus_flavus
Q2UR52_Aspergillus_oryzae
A0A064B4U5_Aspergillus_oryzae
T0KZY3_Colletotrichum_gloeosporioides
B0DFG5_Laccaria_bicolor
T1KIF8_Tetranychus_urticae
N0BI23_Archaeoglobus_sulfaticallidus
A0A0D5ZFX1_uncultured_archaeon
K4M9H8_Methanolobus_psychrophilus
K4M8B8_Methanolobus_psychrophilus
V4W6Z6_Citrus_clementina
E4XID8_Oikopleura_dioica
E0VA62_Pediculus_humanus
A0A069DST5_Panstrongylus_megistus
H9JMI5_Bombyx_mori
H3A5P5_Latimeria_chalumnae
G1U535_Oryctolagus_cuniculus
V9L1T7_Callorhinchus_milii
A0A0D6QSW2_Araucaria_cunninghamii
K3YS14_Setaria_italica
K3YTA6_Setaria_italica
A0A0A9D896_Arundo_donax
0.5

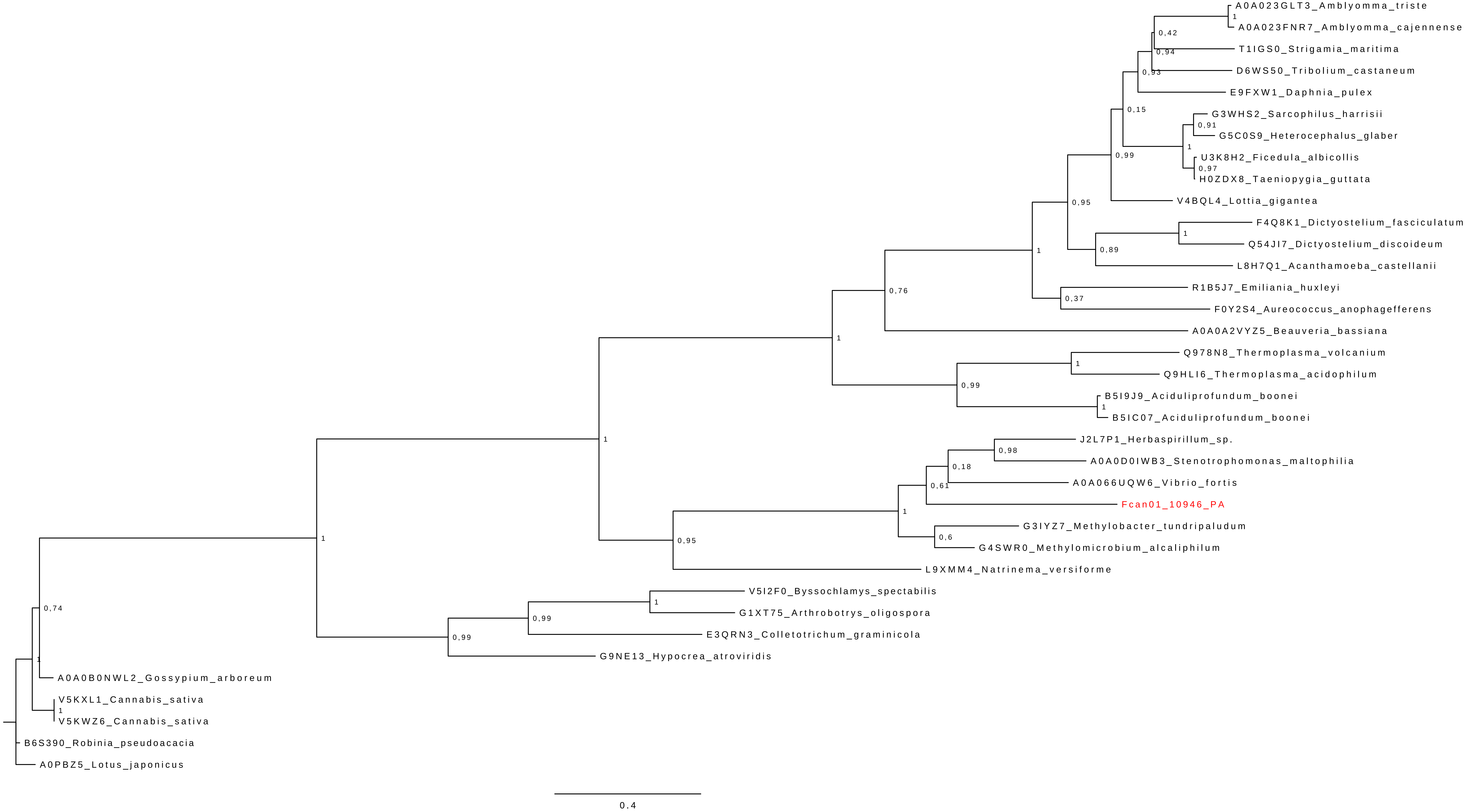
A0A023GLT3_Amblyomma_triste
1
A0A023FNR7_Amblyomma_cajennense
0,42
T1IGS0_Strigamia_maritima
0,94
D6WS50_Tribolium_castaneum
0,93
E9FXW1_Daphnia_pulex
0,15
G3WHS2_Sarcophilus_harrisii
0,91
G5C0S9_Heterocephalus_glaber
1
0,99
U3K8H2_Ficedula_albicollis
0,97
H0ZDX8_Taeniopygia_guttata
V4BQL4_Lottia_gigantea
0,95
F4Q8K1_Dictyostelium_fasciculatum
1
Q54JI7_Dictyostelium_discoideum
0,89
1
L8H7Q1_Acanthamoeba_castellanii
R1B5J7_Emiliania_huxleyi
0,76
0,37
F0Y2S4_Aureococcus_anophagefferens
A0A0A2VYZ5_Beauveria_bassiana
1
Q978N8_Thermoplasma_volcanium
1
Q9HLI6_Thermoplasma_acidophilum
0,99
B5I9J9_Aciduliprofundum_boonei
1
B5IC07_Aciduliprofundum_boonei
J2L7P1_Herbaspirillum_sp.
1
0,98
A0A0D0IWB3_Stenotrophomonas_maltophilia
0,18
A0A066UQW6_Vibrio_fortis
0,61
Fcan01_10946_PA
1
G3IYZ7_Methylobacter_tundripaludum
0,6
1
0,95
G4SWR0_Methylomicrobium_alcaliphilum
L9XMM4_Natrinema_versiforme
V5I2F0_Byssochlamys_spectabilis
1
0,74
G1XT75_Arthrobotrys_oligospora
0,99
E3QRN3_Colletotrichum_graminicola
0,99
G9NE13_Hypocrea_atroviridis
1
A0A0B0NWL2_Gossypium_arboreum
V5KXL1_Cannabis_sativa
1
V5KWZ6_Cannabis_sativa
B6S390_Robinia_pseudoacacia
A0PBZ5_Lotus_japonicus
0.4

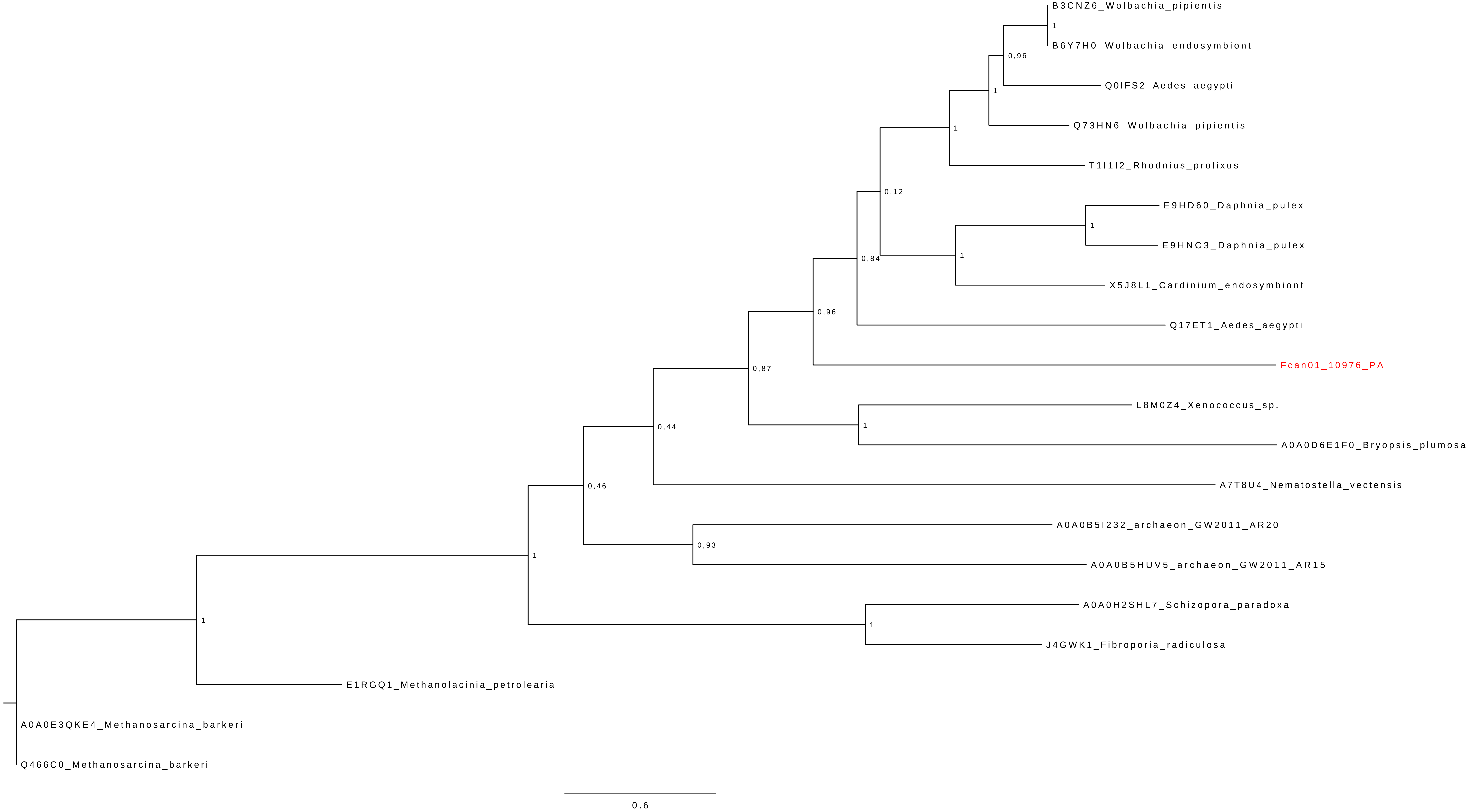
B3CNZ6_Wolbachia_pipientis
1
B6Y7H0_Wolbachia_endosymbiont
0,96
Q0IFS2_Aedes_aegypti
1
Q73HN6_Wolbachia_pipientis
1
T1I1I2_Rhodnius_prolixus
0,12
E9HD60_Daphnia_pulex
1
E9HNC3_Daphnia_pulex
0,84
1
X5J8L1_Cardinium_endosymbiont
0,96
Q17ET1_Aedes_aegypti
Fcan01_10976_PA
0,87
L8M0Z4_Xenococcus_sp.
0,44
1
A0A0D6E1F0_Bryopsis_plumosa
0,46
A7T8U4_Nematostella_vectensis
A0A0B5I232_archaeon_GW2011_AR20
0,93
1
A0A0B5HUV5_archaeon_GW2011_AR15
A0A0H2SHL7_Schizopora_paradoxa
1
1
J4GWK1_Fibroporia_radiculosa
E1RGQ1_Methanolacinia_petrolearia
A0A0E3QKE4_Methanosarcina_barkeri
Q466C0_Methanosarcina_barkeri
0.6

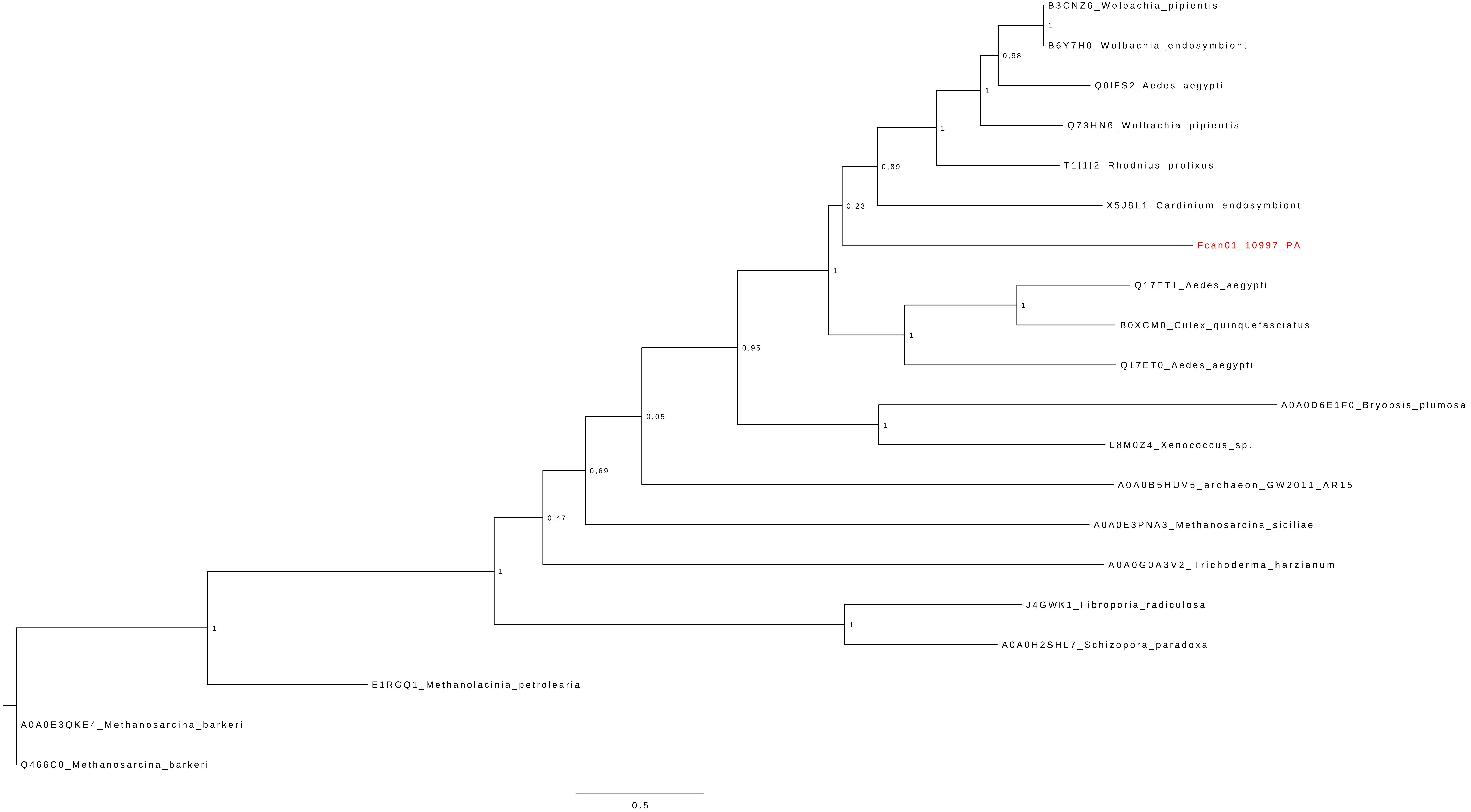
B3CNZ6_Wolbachia_pipientis
1
B6Y7H0_Wolbachia_endosymbiont
0,98
Q0IFS2_Aedes_aegypti
1
Q73HN6_Wolbachia_pipientis
1
T1I1I2_Rhodnius_prolixus
0,89
X5J8L1_Cardinium_endosymbiont
0,23
Fcan01_10997_PA
1
Q17ET1_Aedes_aegypti
1
B0XCM0_Culex_quinquefasciatus
1
Q17ET0_Aedes_aegypti
0,95
A0A0D6E1F0_Bryopsis_plumosa
1
L8M0Z4_Xenococcus_sp.
0,05
A0A0B5HUV5_archaeon_GW2011_AR15
0,69
A0A0E3PNA3_Methanosarcina_siciliae
0,47
A0A0G0A3V2_Trichoderma_harzianum
1
J4GWK1_Fibroporia_radiculosa
1
A0A0H2SHL7_Schizopora_paradoxa
1
E1RGQ1_Methanolacinia_petrolearia
A0A0E3QKE4_Methanosarcina_barkeri
Q466C0_Methanosarcina_barkeri
0.5

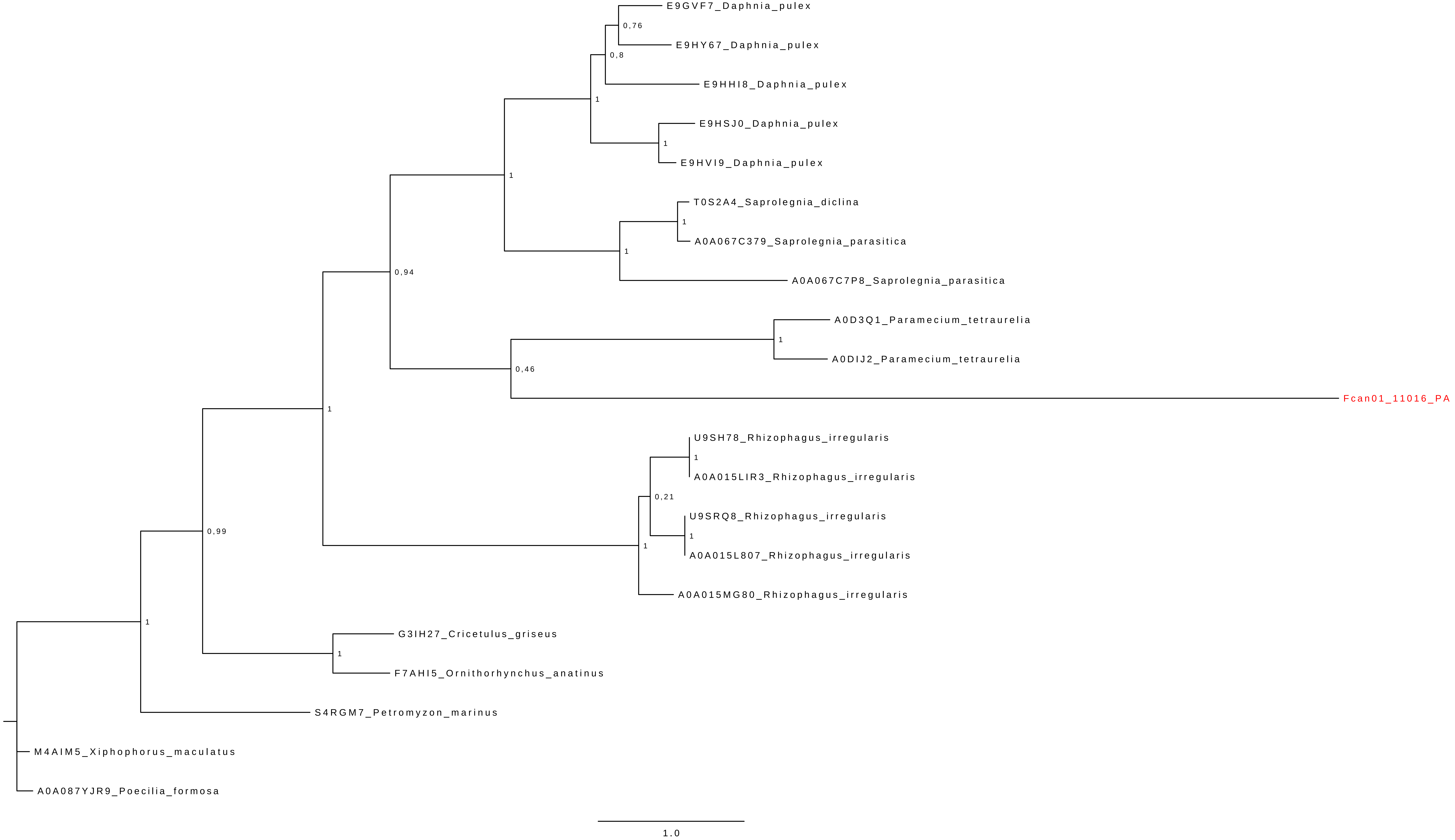
E9GVF7_Daphnia_pulex
0,76
E9HY67_Daphnia_pulex
0,8
E9HHI8_Daphnia_pulex
1
E9HSJ0_Daphnia_pulex
1
E9HVI9_Daphnia_pulex
1
T0S2A4_Saprolegnia_diclina
1
A0A067C379_Saprolegnia_parasitica
1
A0A067C7P8_Saprolegnia_parasitica
0,94
A0D3Q1_Paramecium_tetraurelia
1
A0DIJ2_Paramecium_tetraurelia
0,46
Fcan01_11016_PA
1
U9SH78_Rhizophagus_irregularis
1
A0A015LIR3_Rhizophagus_irregularis
0,21
U9SRQ8_Rhizophagus_irregularis
1
A0A015L807_Rhizophagus_irregularis
0,99
1
A0A015MG80_Rhizophagus_irregularis
G3IH27_Cricetulus_griseus
1
1
F7AHI5_Ornithorhynchus_anatinus
S4RGM7_Petromyzon_marinus
M4AIM5_Xiphophorus_maculatus
A0A087YJR9_Poecilia_formosa
1.0

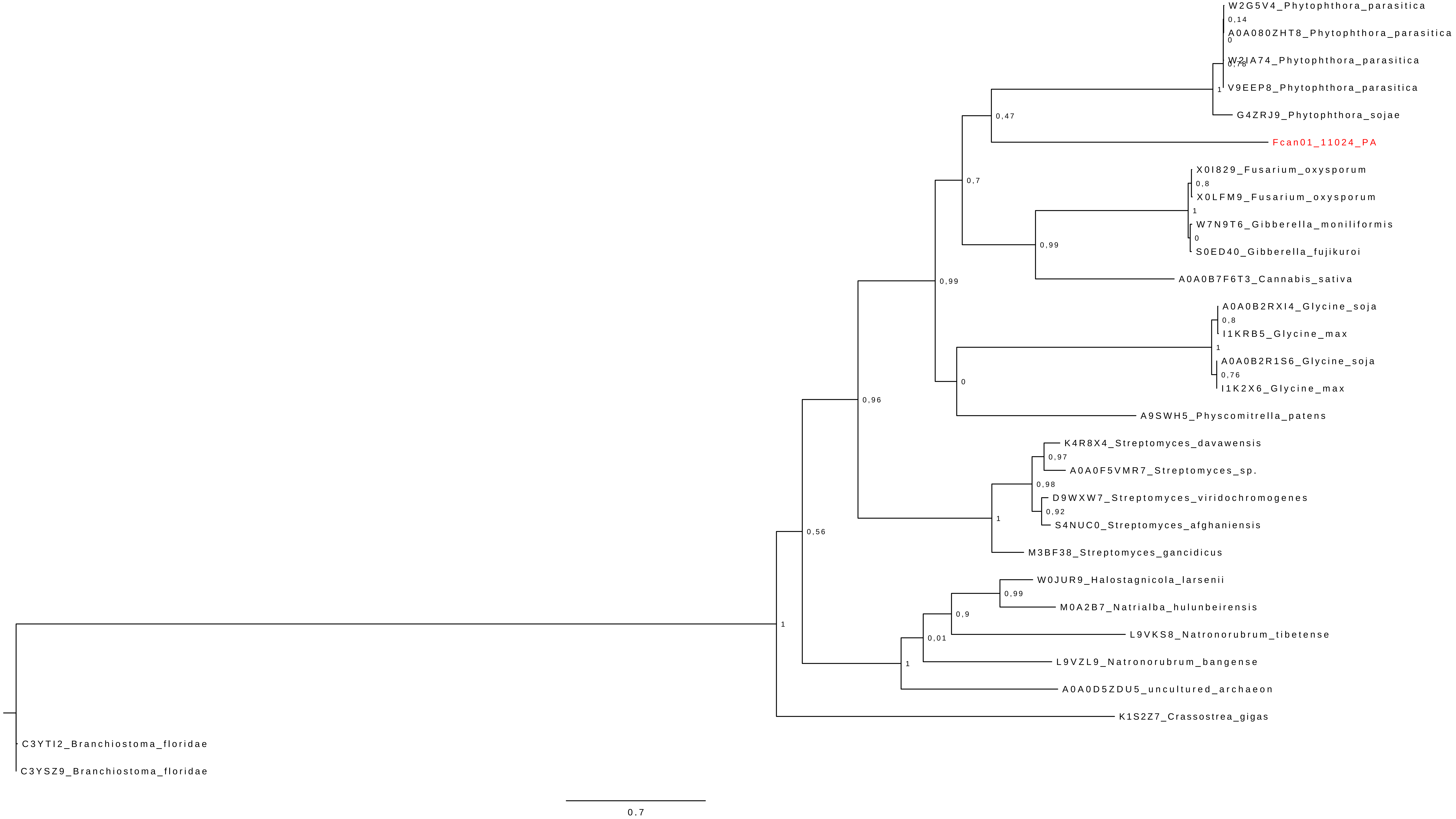
W2G5V4_Phytophthora_parasitica
A0A080ZHT8_Phytophthora_parasitica
W2IA74_Phytophthora_parasitica
V9EEP8_Phytophthora_parasitica
G4ZRJ9_Phytophthora_sojae
Fcan01_11024_PA
X0I829_Fusarium_oxysporum
X0LFM9_Fusarium_oxysporum
W7N9T6_Gibberella_moniliformis
S0ED40_Gibberella_fujikuroi
A0A0B7F6T3_Cannabis_sativa
A0A0B2RXI4_Glycine_soja
I1KRB5_Glycine_max
A0A0B2R1S6_Glycine_soja
I1K2X6_Glycine_max
A9SWH5_Physcomitrella_patens
K4R8X4_Streptomyces_davawensis
A0A0F5VMR7_Streptomyces_sp.
D9WXW7_Streptomyces_viridochromogenes
S4NUC0_Streptomyces_afghaniensis
M3BF38_Streptomyces_gancidicus
W0JUR9_Halostagnicola_larsenii
M0A2B7_Natrialba_hulunbeirensis
L9VKS8_Natronorubrum_tibetense
L9VZL9_Natronorubrum_bangense
A0A0D5ZDU5_uncultured_archaeon
K1S2Z7_Crassostrea_gigas
C3YTI2_Branchiostoma_floridae
C3YSZ9_Branchiostoma_floridae
0.7

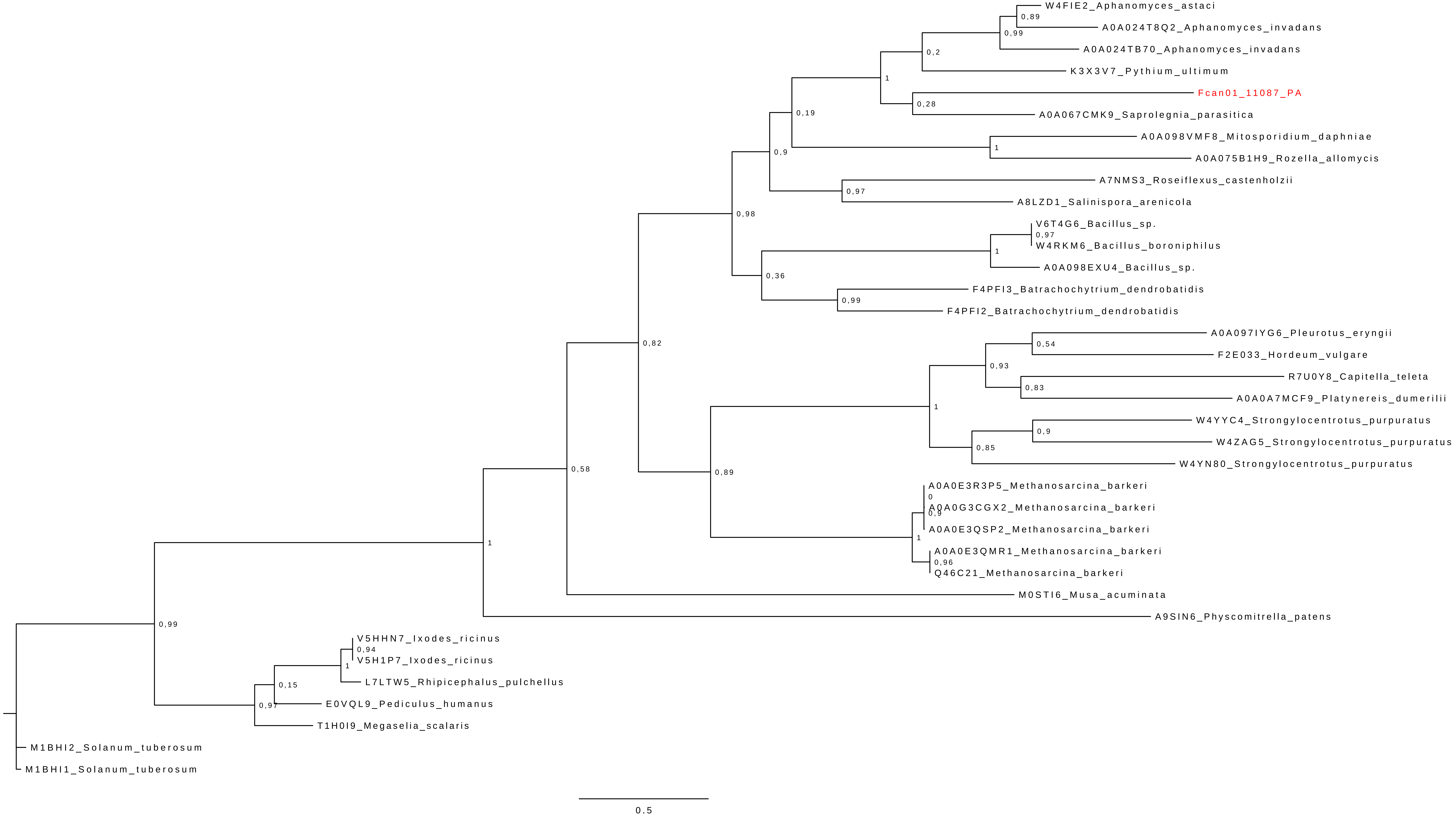

W4FIE2_Aphanomyces_astaci
0,89
A0A024T8Q2_Aphanomyces_invadans
0,99
A0A024TB70_Aphanomyces_invadans
0,2
K3X3V7_Pythium_ultimum
1
Fcan01_11087_PA
0,28
A0A067CMK9_Saprolegnia_parasitica
0,19
A0A098VMF8_Mitosporidium_daphniae
1
A0A075B1H9_Rozella_allomycis
0,9
A7NMS3_Roseiflexus_castenholzii
0,97
A8LZD1_Salinispora_arenicola
0,98
V6T4G6_Bacillus_sp.
0,97
W4RKM6_Bacillus_boroniphilus
1
A0A098EXU4_Bacillus_sp.
0,36
F4PFI3_Batrachochytrium_dendrobatidis
0,99
F4PFI2_Batrachochytrium_dendrobatidis
0,82
A0A097IYG6_Pleurotus_eryngii
0,54
F2E033_Hordeum_vulgare
0,93
R7U0Y8_Capitella_teleta
0,83
A0A0A7MCF9_Platynereis_dumerilii
1
W4YYC4_Strongylocentrotus_purpuratus
0,9
W4ZAG5_Strongylocentrotus_purpuratus
0,85
W4YN80_Strongylocentrotus_purpuratus
0,89
A0A0E3R3P5_Methanosarcina_barkeri
0
A0A0G3CGX2_Methanosarcina_barkeri
0,9
A0A0E3QSP2_Methanosarcina_barkeri
1
A0A0E3QMR1_Methanosarcina_barkeri
0,96
Q46C21_Methanosarcina_barkeri
0,58
M0STI6_Musa_acuminata
1
A9SIN6_Physcomitrella_patens
0,99
V5HHN7_Ixodes_ricinus
0,94
V5H1P7_Ixodes_ricinus
1
L7LTW5_Rhipicephalus_pulchellus
0,15
E0VQL9_Pediculus_humanus
0,97
T1H0I9_Megaselia_scalaris
M1BHI2_Solanum_tuberosum
M1BHI1_Solanum_tuberosum
0.5

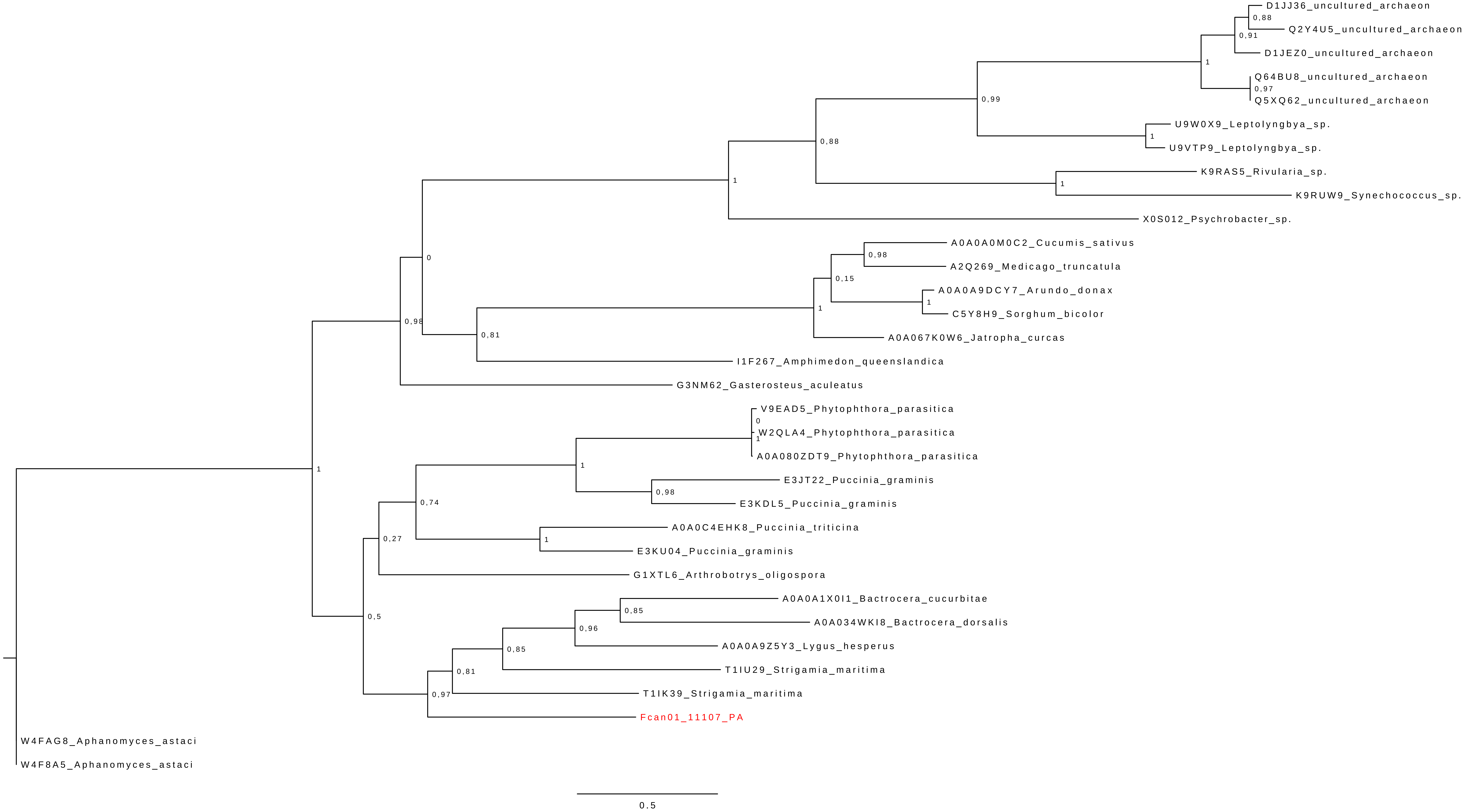

D1JJ36_uncultured_archaeon
Q2Y4U5_uncultured_archaeon
D1JEZ0_uncultured_archaeon
Q64BU8_uncultured_archaeon
Q5XQ62_uncultured_archaeon
U9W0X9_Leptolyngbya_sp.
U9VTP9_Leptolyngbya_sp.
K9RAS5_Rivularia_sp.
K9RUW9_Synechococcus_sp.
X0S012_Psychrobacter_sp.
A0A0A0M0C2_Cucumis_sativus
A2Q269_Medicago_truncatula
A0A0A9DCY7_Arundo_donax
C5Y8H9_Sorghum_bicolor
A0A067K0W6_Jatropha_curcas
I1F267_Amphimedon_queenslandica
G3NM62_Gasterosteus_aculeatus
V9EAD5_Phytophthora_parasitica
W2QLA4_Phytophthora_parasitica
A0A080ZDT9_Phytophthora_parasitica
E3JT22_Puccinia_graminis
E3KDL5_Puccinia_graminis
A0A0C4EHK8_Puccinia_triticina
E3KU04_Puccinia_graminis
G1XTL6_Arthrobotrys_oligospora
A0A0A1X0I1_Bactrocera_cucurbitae
A0A034WKI8_Bactrocera_dorsalis
A0A0A9Z5Y3_Lygus_hesperus
T1IU29_Strigamia_maritima
T1IK39_Strigamia_maritima
Fcan01_11107_PA
W4FAG8_Aphanomyces_astaci
W4F8A5_Aphanomyces_astaci
0.5

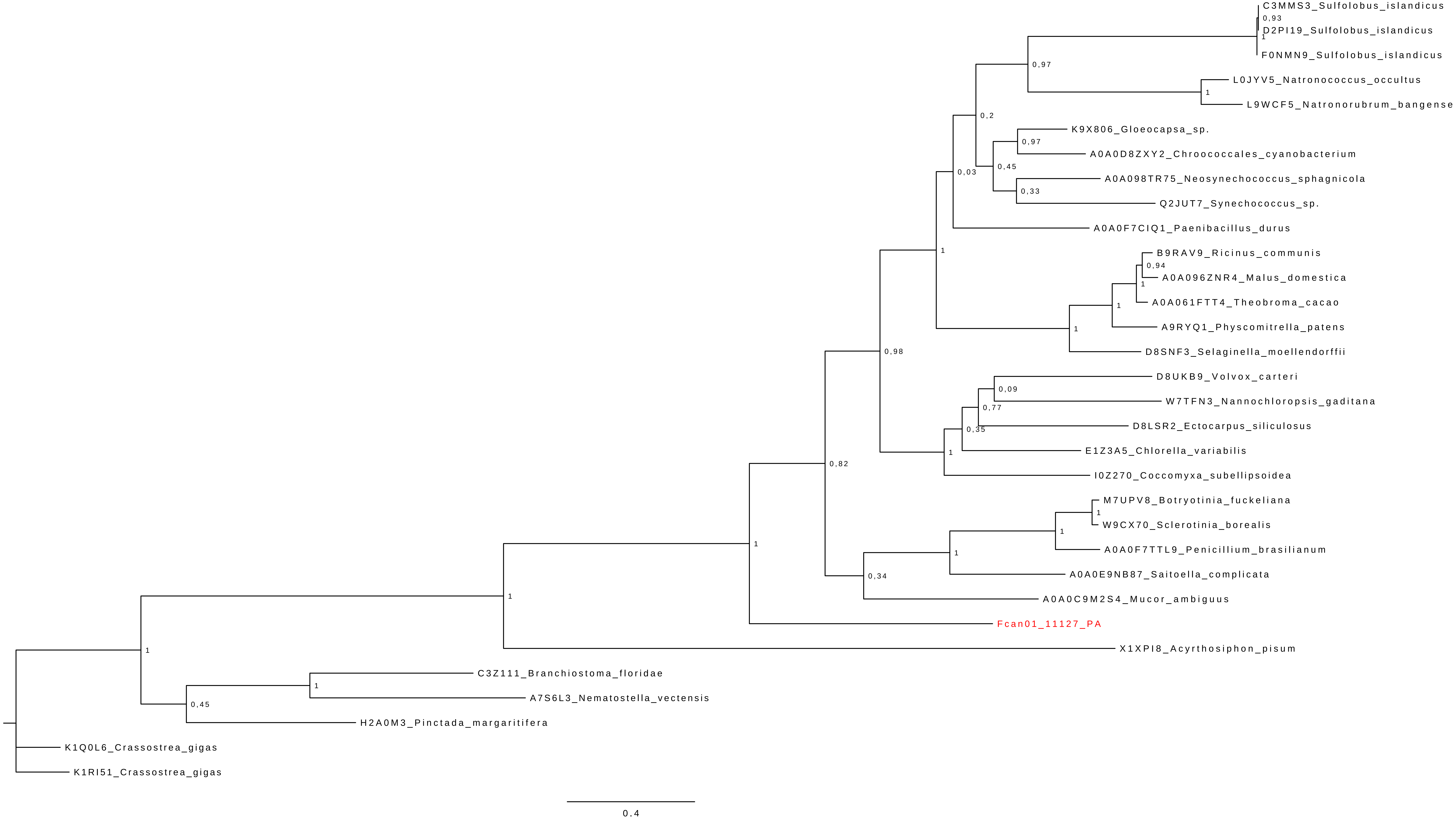
C3MMS3_Sulfolobus_islandicus
D2PI19_Sulfolobus_islandicus
F0NMN9_Sulfolobus_islandicus
L0JYV5_Natronococcus_occultus
L9WCF5_Natronorubrum_bangense
K9X806_Gloeocapsa_sp.
A0A0D8ZXY2_Chroococcales_cyanobacterium
A0A098TR75_Neosynechococcus_sphagnicola
Q2JUT7_Synechococcus_sp.
A0A0F7CIQ1_Paenibacillus_durus
B9RAV9_Ricinus_communis
A0A096ZNR4_Malus_domestica
A0A061FTT4_Theobroma_cacao
A9RYQ1_Physcomitrella_patens
D8SNF3_Selaginella_moellendorffii
D8UKB9_Volvox_carteri
W7TFN3_Nannochloropsis_gaditana
D8LSR2_Ectocarpus_siliculosus
E1Z3A5_Chlorella_variabilis
I0Z270_Coccomyxa_subellipsoidea
M7UPV8_Botryotinia_fuckeliana
W9CX70_Sclerotinia_borealis
A0A0F7TTL9_Penicillium_brasilianum
A0A0E9NB87_Saitoella_complicata
A0A0C9M2S4_Mucor_ambiguus
Fcan01_11127_PA
X1XPI8_Acyrthosiphon_pisum
C3Z111_Branchiostoma_floridae
A7S6L3_Nematostella_vectensis
H2A0M3_Pinctada_margaritifera
K1Q0L6_Crassostrea_gigas
K1RI51_Crassostrea_gigas
0,93
1
0,97
1
0,2
0,97
0,45
0,33
0,03
1
0,94
1
1
1
0,98
0,09
0,77
0,35
1
0,82
1
1
1
0,34
1
1
1
1
0,45
0.4

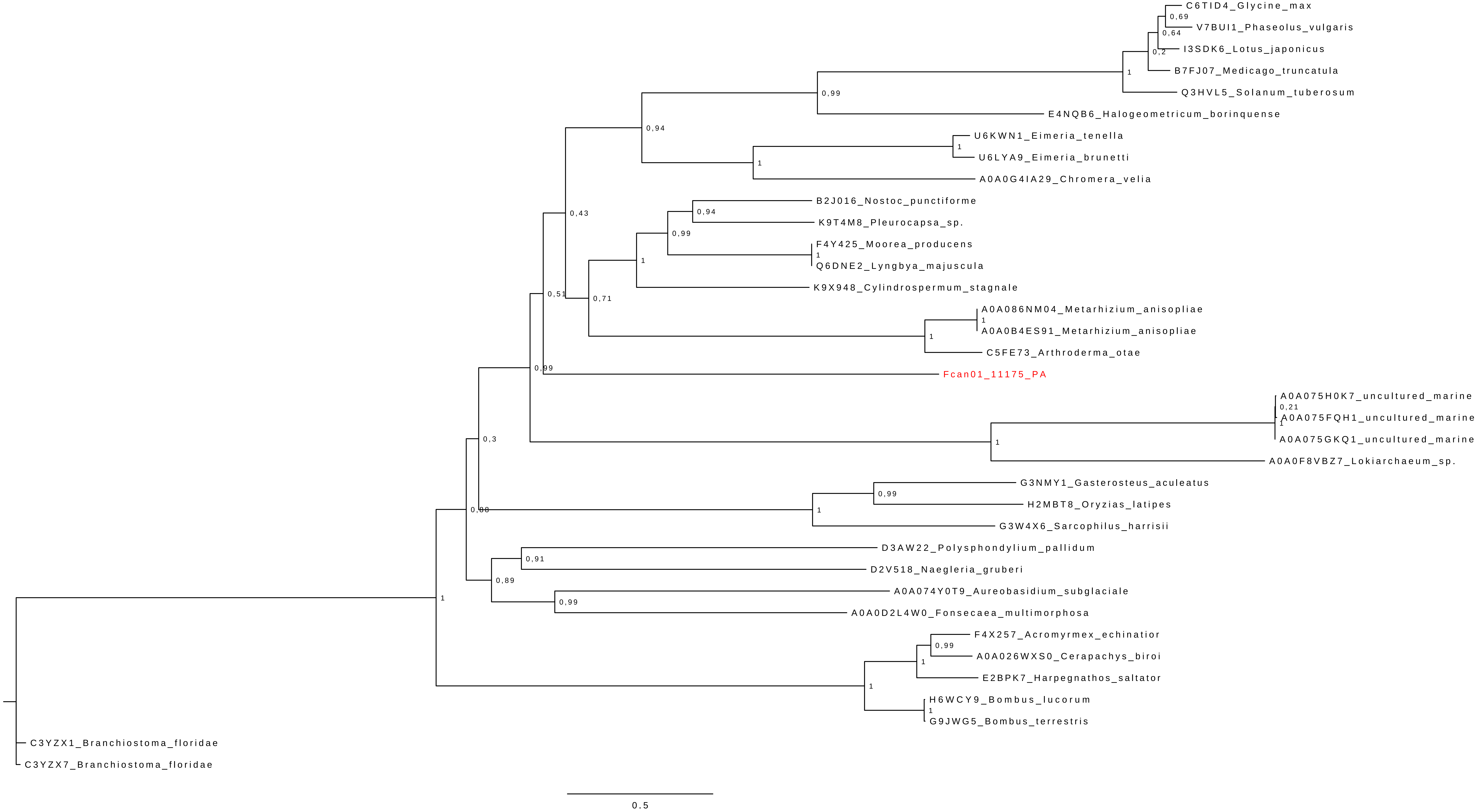
C6TID4_Glycine_max
V7BUI1_Phaseolus_vulgaris
I3SDK6_Lotus_japonicus
B7FJ07_Medicago_truncatula
Q3HVL5_Solanum_tuberosum
E4NQB6_Halogeometricum_borinquense
U6KWN1_Eimeria_tenella
U6LYA9_Eimeria_brunetti
A0A0G4IA29_Chromera_velia
B2J016_Nostoc_punctiforme
K9T4M8_Pleurocapsa_sp.
F4Y425_Moorea_producens
Q6DNE2_Lyngbya_majuscula
K9X948_Cylindrospermum_stagnale
A0A086NM04_Metarhizium_anisopliae
A0A0B4ES91_Metarhizium_anisopliae
C5FE73_Arthroderma_otae
Fcan01_11175_PA
A0A075H0K7_uncultured_marine
A0A075FQH1_uncultured_marine
A0A075GKQ1_uncultured_marine
A0A0F8VBZ7_Lokiarchaeum_sp.
G3NMY1_Gasterosteus_aculeatus
H2MBT8_Oryzias_latipes
G3W4X6_Sarcophilus_harrisii
D3AW22_Polysphondylium_pallidum
D2V518_Naegleria_gruberi
A0A074Y0T9_Aureobasidium_subglaciale
A0A0D2L4W0_Fonsecaea_multimorphosa
F4X257_Acromyrmex_echinatior
A0A026WXS0_Cerapachys_biroi
E2BPK7_Harpegnathos_saltator
H6WCY9_Bombus_lucorum
G9JWG5_Bombus_terrestris
C3YZX1_Branchiostoma_floridae
C3YZX7_Branchiostoma_floridae
0.5

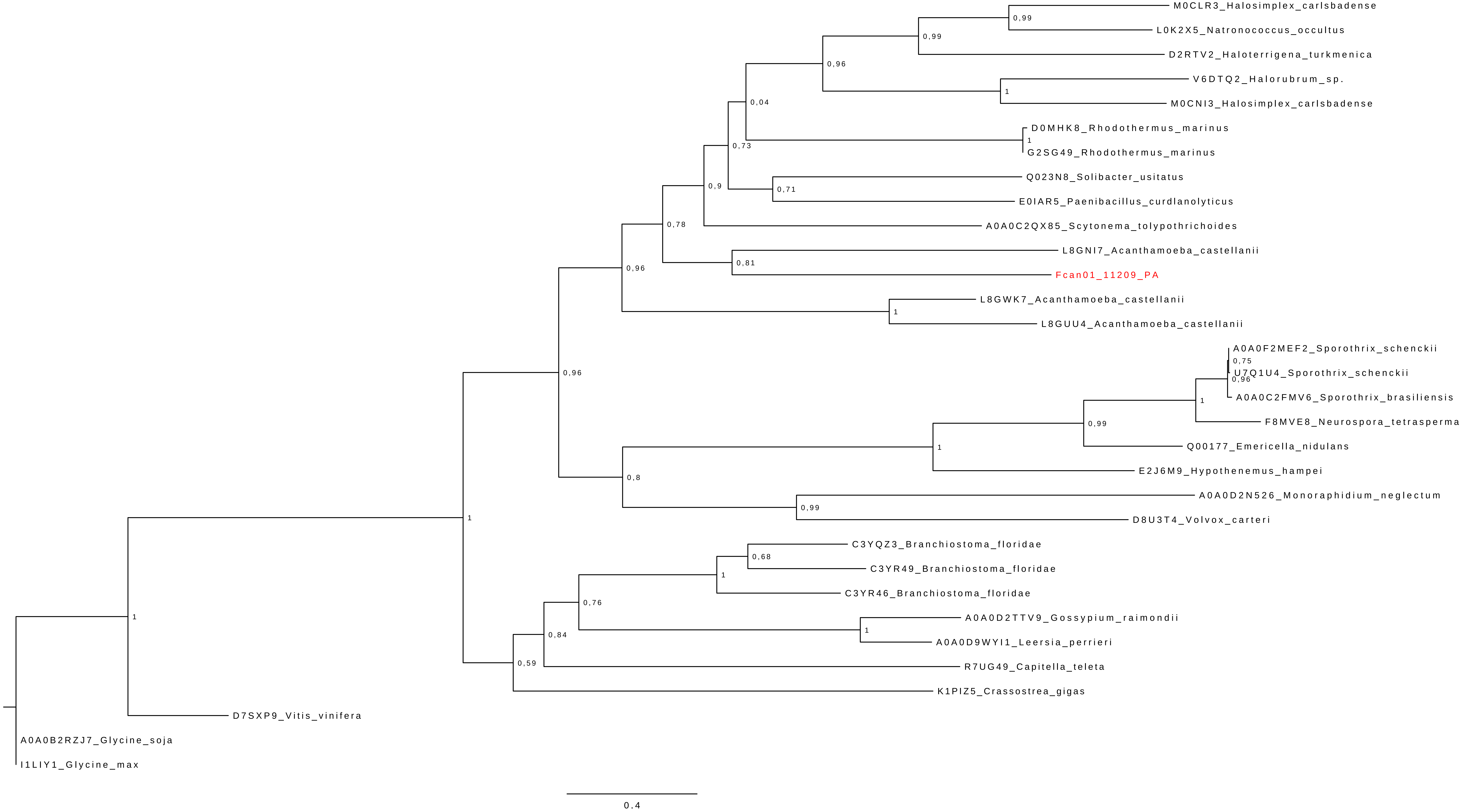
M0CLR3_Halosimplex_carlsbadense
L0K2X5_Natronococcus_occultus
D2RTV2_Haloterrigena_turkmenica
V6DTQ2_Halorubrum_sp.
M0CNI3_Halosimplex_carlsbadense
D0MHK8_Rhodothermus_marinus
G2SG49_Rhodothermus_marinus
Q023N8_Solibacter_usitatus
E0IAR5_Paenibacillus_curdlanolyticus
A0A0C2QX85_Scytonema_tolypothrichoides
L8GNI7_Acanthamoeba_castellanii
Fcan01_11209_PA
L8GWK7_Acanthamoeba_castellanii
L8GUU4_Acanthamoeba_castellanii
A0A0F2MEF2_Sporothrix_schenckii
U7Q1U4_Sporothrix_schenckii
A0A0C2FMV6_Sporothrix_brasiliensis
F8MVE8_Neurospora_tetrasperma
Q00177_Emericella_nidulans
E2J6M9_Hypothenemus_hampei
A0A0D2N526_Monoraphidium_neglectum
D8U3T4_Volvox_carteri
C3YQZ3_Branchiostoma_floridae
C3YR49_Branchiostoma_floridae
C3YR46_Branchiostoma_floridae
A0A0D2TTV9_Gossypium_raimondii
A0A0D9WYI1_Leersia_perrieri
R7UG49_Capitella_teleta
K1PIZ5_Crassostrea_gigas
D7SXP9_Vitis_vinifera
A0A0B2RZJ7_Glycine_soja
I1LIY1_Glycine_max
0.4

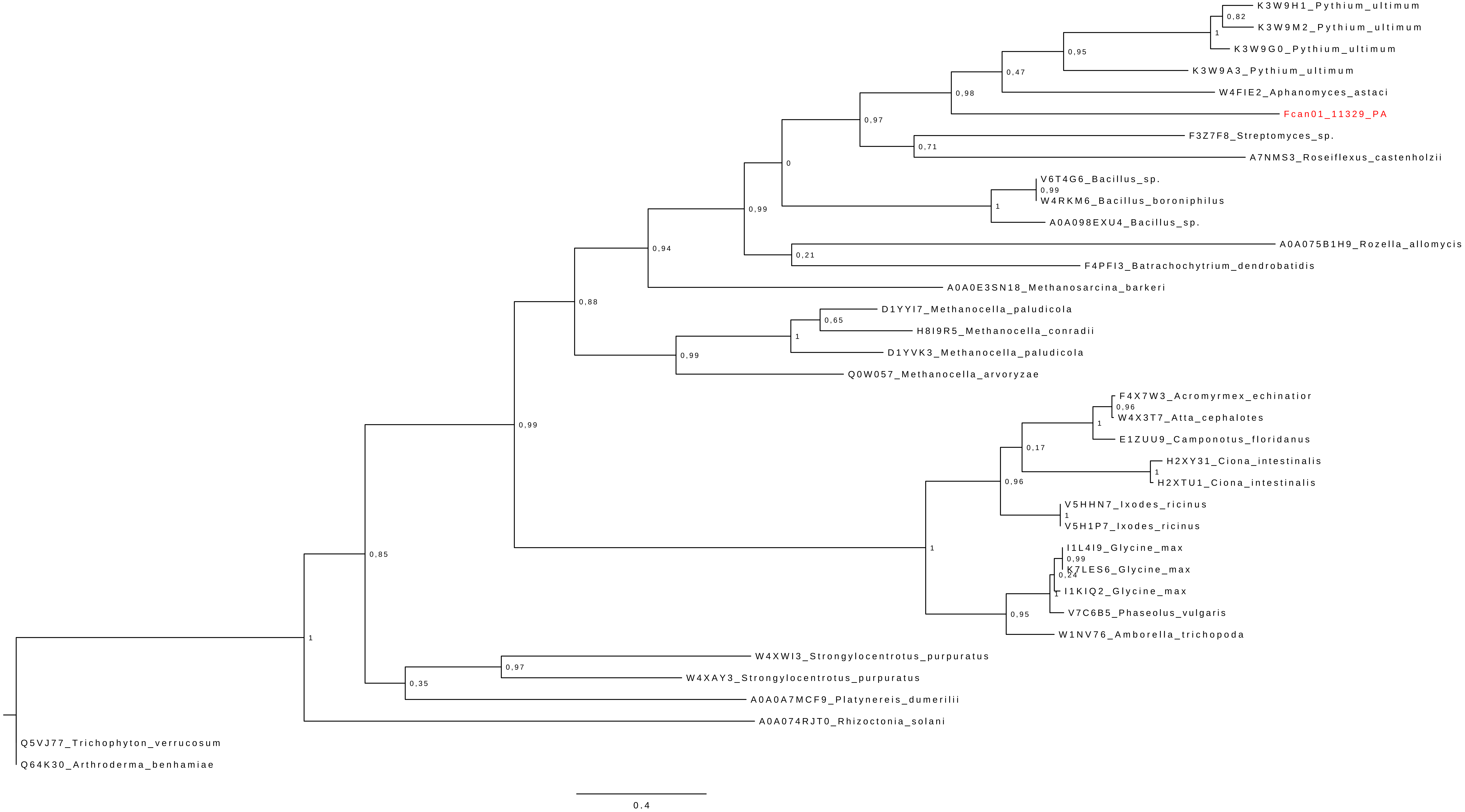

K3W9H1_Pythium_ultimum
K3W9M2_Pythium_ultimum
K3W9G0_Pythium_ultimum
K3W9A3_Pythium_ultimum
W4FIE2_Aphanomyces_astaci
Fcan01_11329_PA
F3Z7F8_Streptomyces_sp.
A7NMS3_Roseiflexus_castenholzii
V6T4G6_Bacillus_sp.
W4RKM6_Bacillus_boroniphilus
A0A098EXU4_Bacillus_sp.
A0A075B1H9_Rozella_allomycis
F4PFI3_Batrachochytrium_dendrobatidis
A0A0E3SN18_Methanosarcina_barkeri
D1YYI7_Methanocella_paludicola
H8I9R5_Methanocella_conradii
D1YVK3_Methanocella_paludicola
Q0W057_Methanocella_arvoryzae
F4X7W3_Acromyrmex_echinatior
W4X3T7_Atta_cephalotes
E1ZUU9_Camponotus_floridanus
H2XY31_Ciona_intestinalis
H2XTU1_Ciona_intestinalis
V5HHN7_Ixodes_ricinus
V5H1P7_Ixodes_ricinus
I1L4I9_Glycine_max
K7LES6_Glycine_max
I1KIQ2_Glycine_max
V7C6B5_Phaseolus_vulgaris
W1NV76_Amborella_trichopoda
W4XWI3_Strongylocentrotus_purpuratus
W4XAY3_Strongylocentrotus_purpuratus
A0A0A7MCF9_Platynereis_dumerilii
A0A074RJT0_Rhizoctonia_solani
Q5VJ77_Trichophyton_verrucosum
Q64K30_Arthroderma_benhamiae
0.4

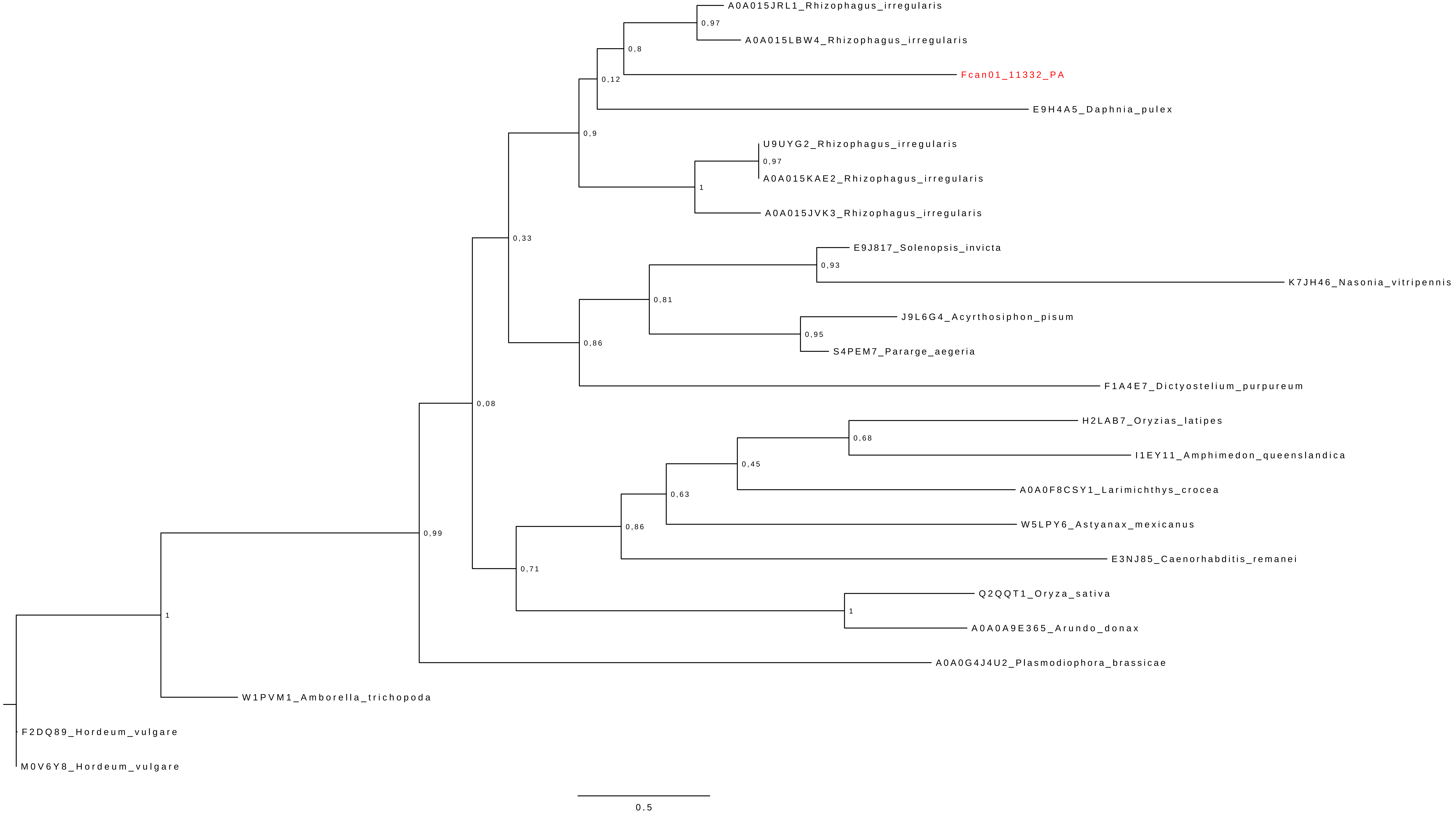
A0A015JRL1_Rhizophagus_irregularis
0,97
A0A015LBW4_Rhizophagus_irregularis
0,8
Fcan01_11332_PA
0,12
E9H4A5_Daphnia_pulex
0,9
U9UYG2_Rhizophagus_irregularis
0,97
A0A015KAE2_Rhizophagus_irregularis
1
A0A015JVK3_Rhizophagus_irregularis
0,33
E9J817_Solenopsis_invicta
0,93
K7JH46_Nasonia_vitripennis
0,81
J9L6G4_Acyrthosiphon_pisum
0,95
S4PEM7_Pararge_aegeria
0,86
F1A4E7_Dictyostelium_purpureum
0,08
H2LAB7_Oryzias_latipes
0,68
I1EY11_Amphimedon_queenslandica
0,45
A0A0F8CSY1_Larimichthys_crocea
0,63
W5LPY6_Astyanax_mexicanus
0,86
E3NJ85_Caenorhabditis_remanei
0,71
Q2QQT1_Oryza_sativa
1
A0A0A9E365_Arundo_donax
0,99
A0A0G4J4U2_Plasmodiophora_brassicae
1
W1PVM1_Amborella_trichopoda
F2DQ89_Hordeum_vulgare
M0V6Y8_Hordeum_vulgare
0.5

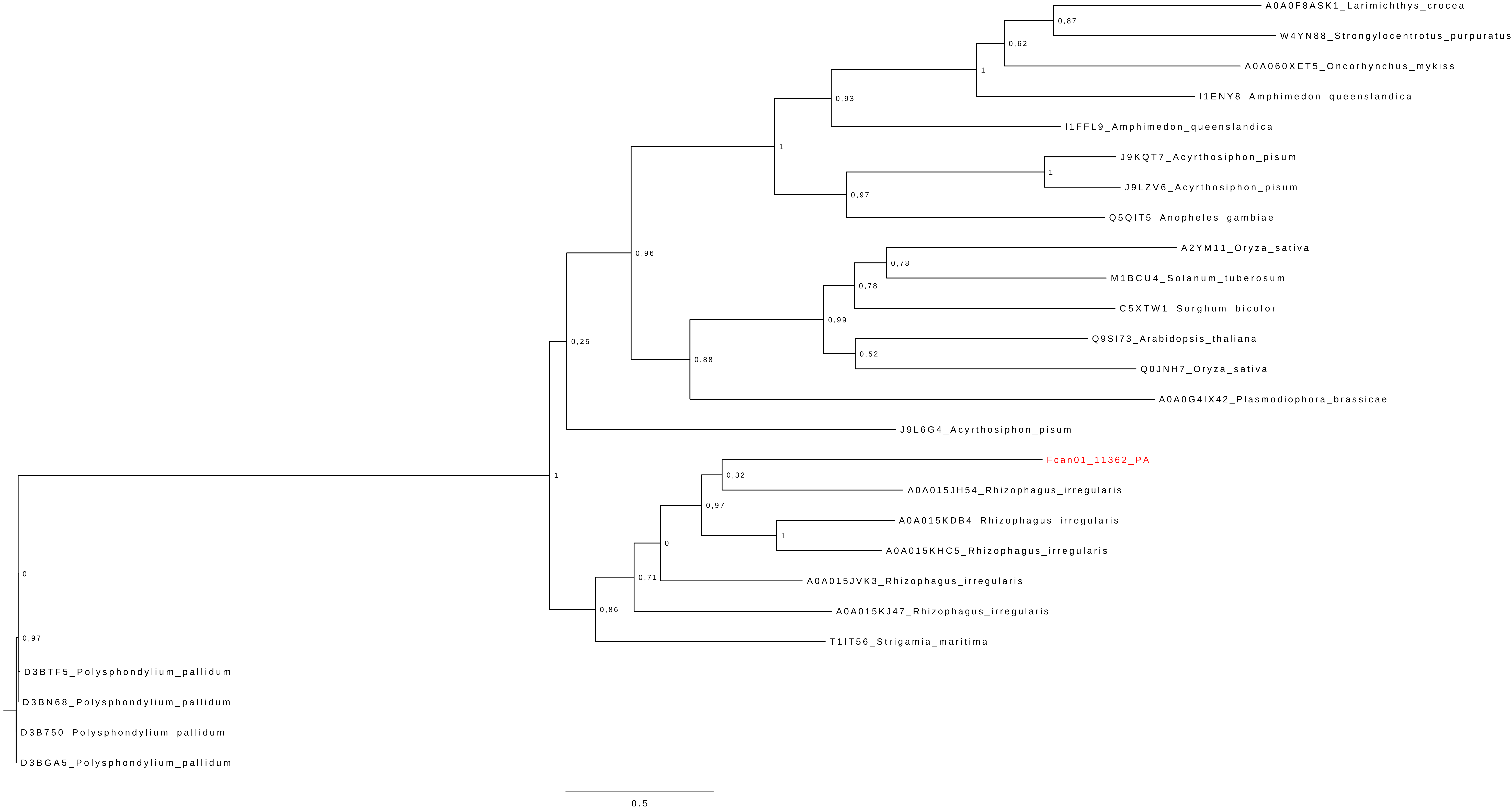
A0A0F8ASK1_Larimichthys_crocea
W4YN88_Strongylocentrotus_purpuratus
A0A060XET5_Oncorhynchus_mykiss
I1ENY8_Amphimedon_queenslandica
I1FFL9_Amphimedon_queenslandica
J9KQT7_Acyrthosiphon_pisum
J9LZV6_Acyrthosiphon_pisum
Q5QIT5_Anopheles_gambiae
A2YM11_Oryza_sativa
M1BCU4_Solanum_tuberosum
C5XTW1_Sorghum_bicolor
Q9SI73_Arabidopsis_thaliana
Q0JNH7_Oryza_sativa
A0A0G4IX42_Plasmodiophora_brassicae
J9L6G4_Acyrthosiphon_pisum
Fcan01_11362_PA
A0A015JH54_Rhizophagus_irregularis
A0A015KDB4_Rhizophagus_irregularis
A0A015KHC5_Rhizophagus_irregularis
A0A015JVK3_Rhizophagus_irregularis
A0A015KJ47_Rhizophagus_irregularis
T1IT56_Strigamia_maritima
D3BTF5_Polysphondylium_pallidum
D3BN68_Polysphondylium_pallidum
D3B750_Polysphondylium_pallidum
D3BGA5_Polysphondylium_pallidum
0,87
0,62
1
0,93
1
1
0,97
0,96
0,78
0,78
0,99
0,52
0,88
0,25
1
0,32
0,97
1
0
0,71
0,86
0
0,97
0.5

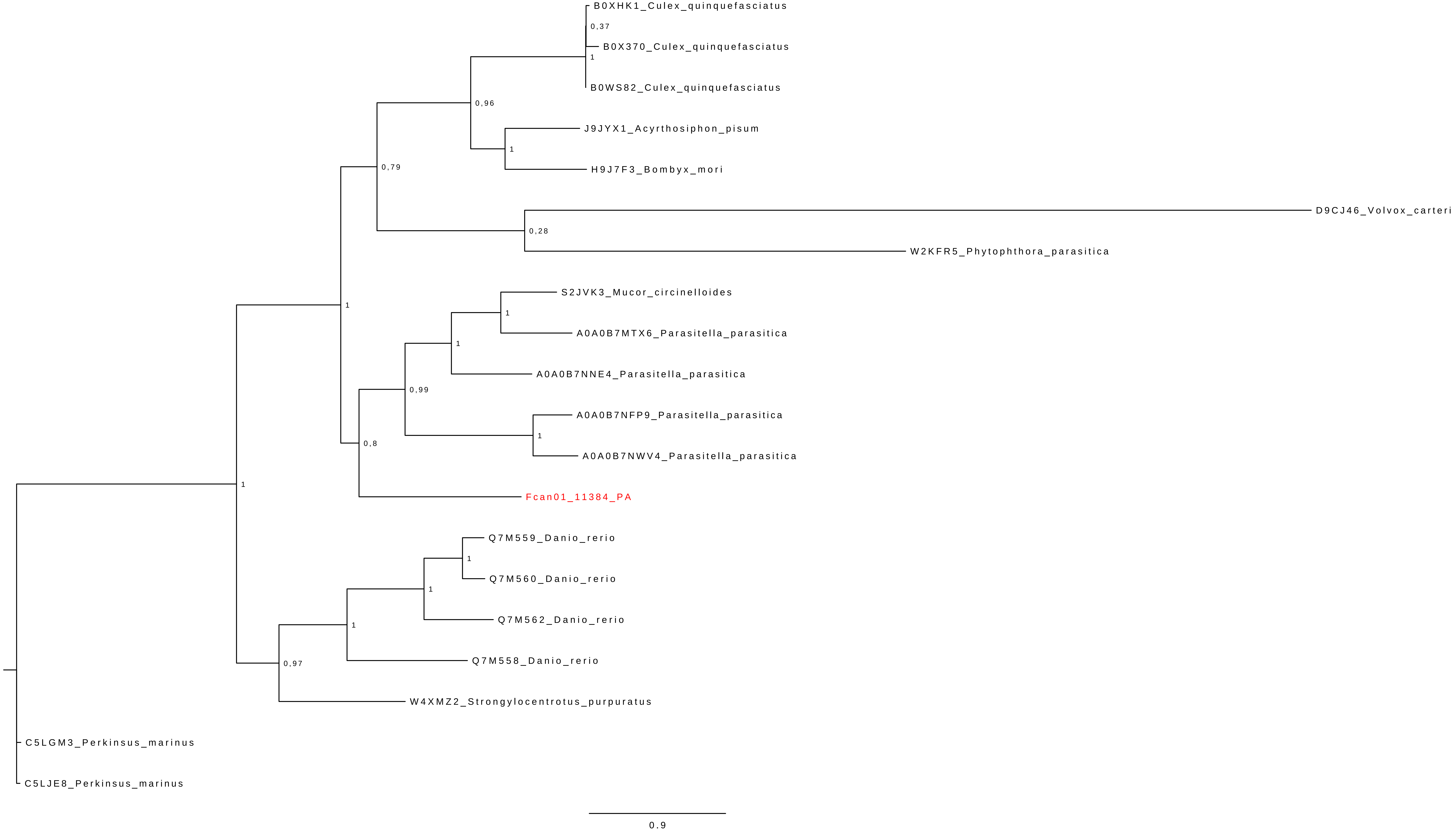
B0XHK1_Culex_quinquefasciatus
0,37
B0X370_Culex_quinquefasciatus
1
B0WS82_Culex_quinquefasciatus
0,96
J9JYX1_Acyrthosiphon_pisum
1
H9J7F3_Bombyx_mori
0,79
D9CJ46_Volvox_carteri
0,28
W2KFR5_Phytophthora_parasitica
S2JVK3_Mucor_circinelloides
1
A0A0B7MTX6_Parasitella_parasitica
1
A0A0B7NNE4_Parasitella_parasitica
0,99
A0A0B7NFP9_Parasitella_parasitica
1
A0A0B7NWV4_Parasitella_parasitica
0,8
Fcan01_11384_PA
1
Q7M559_Danio_rerio
1
Q7M560_Danio_rerio
1
Q7M562_Danio_rerio
1
Q7M558_Danio_rerio
0,97
W4XMZ2_Strongylocentrotus_purpuratus
C5LGM3_Perkinsus_marinus
C5LJE8_Perkinsus_marinus
0.9

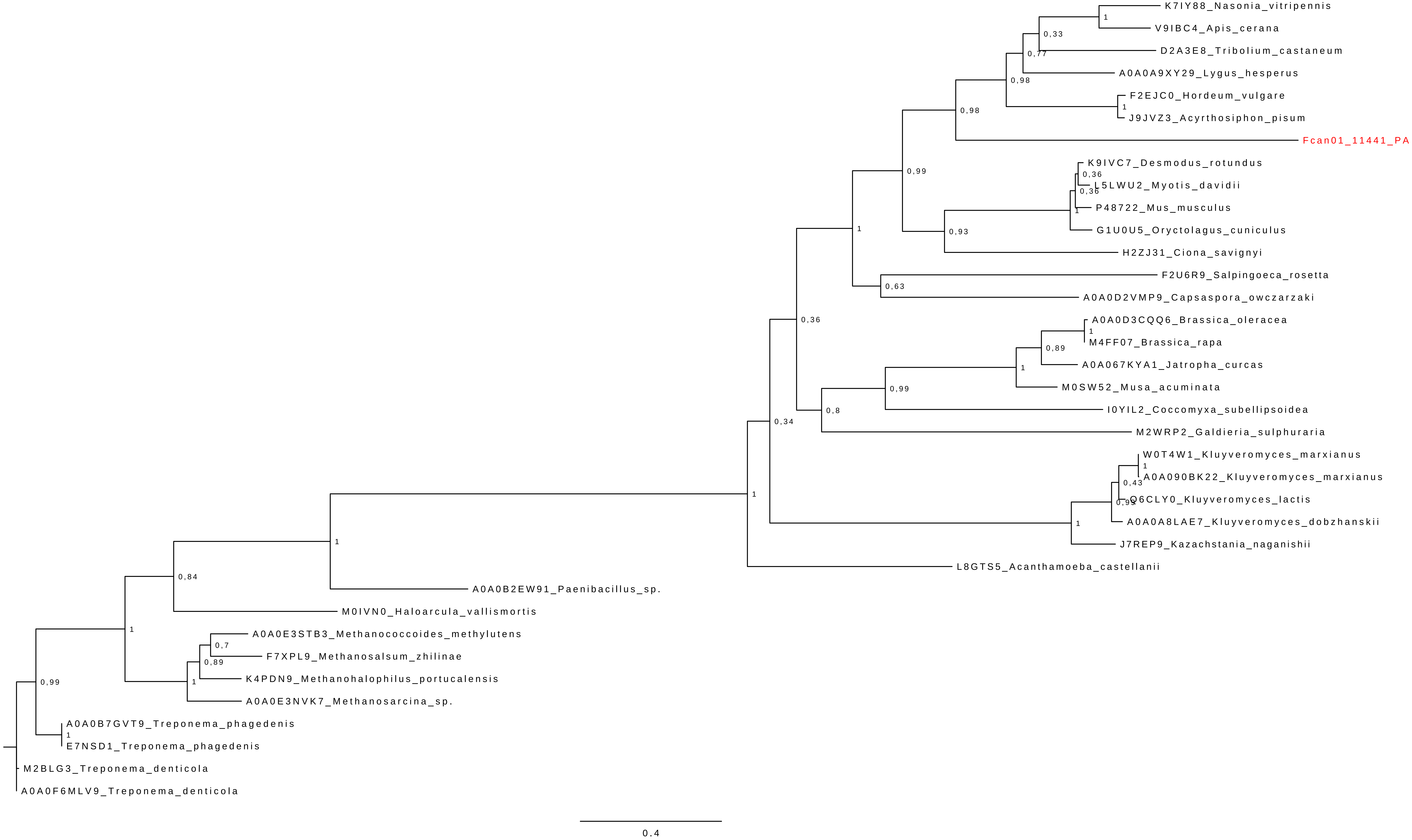
K7IY88_Nasonia_vitripennis
V9IBC4_Apis_cerana
D2A3E8_Tribolium_castaneum
A0A0A9XY29_Lygus_hesperus
F2EJC0_Hordeum_vulgare
J9JVZ3_Acyrthosiphon_pisum
Fcan01_11441_PA
K9IVC7_Desmodus_rotundus
L5LWU2_Myotis_davidii
P48722_Mus_musculus
G1U0U5_Oryctolagus_cuniculus
H2ZJ31_Ciona_savignyi
F2U6R9_Salpingoeca_rosetta
A0A0D2VMP9_Capsaspora_owczarzaki
A0A0D3CQQ6_Brassica_oleracea
M4FF07_Brassica_rapa
A0A067KYA1_Jatropha_curcas
M0SW52_Musa_acuminata
I0YIL2_Coccomyxa_subellipsoidea
M2WRP2_Galdieria_sulphuraria
W0T4W1_Kluyveromyces_marxianus
A0A090BK22_Kluyveromyces_marxianus
Q6CLY0_Kluyveromyces_lactis
A0A0A8LAE7_Kluyveromyces_dobzhanskii
J7REP9_Kazachstania_naganishii
L8GTS5_Acanthamoeba_castellanii
A0A0B2EW91_Paenibacillus_sp.
M0IVN0_Haloarcula_vallismortis
A0A0E3STB3_Methanococcoides_methylutens
F7XPL9_Methanosalsum_zhilinae
K4PDN9_Methanohalophilus_portucalensis
A0A0E3NVK7_Methanosarcina_sp.
A0A0B7GVT9_Treponema_phagedenis
E7NSD1_Treponema_phagedenis
M2BLG3_Treponema_denticola
A0A0F6MLV9_Treponema_denticola
0.4

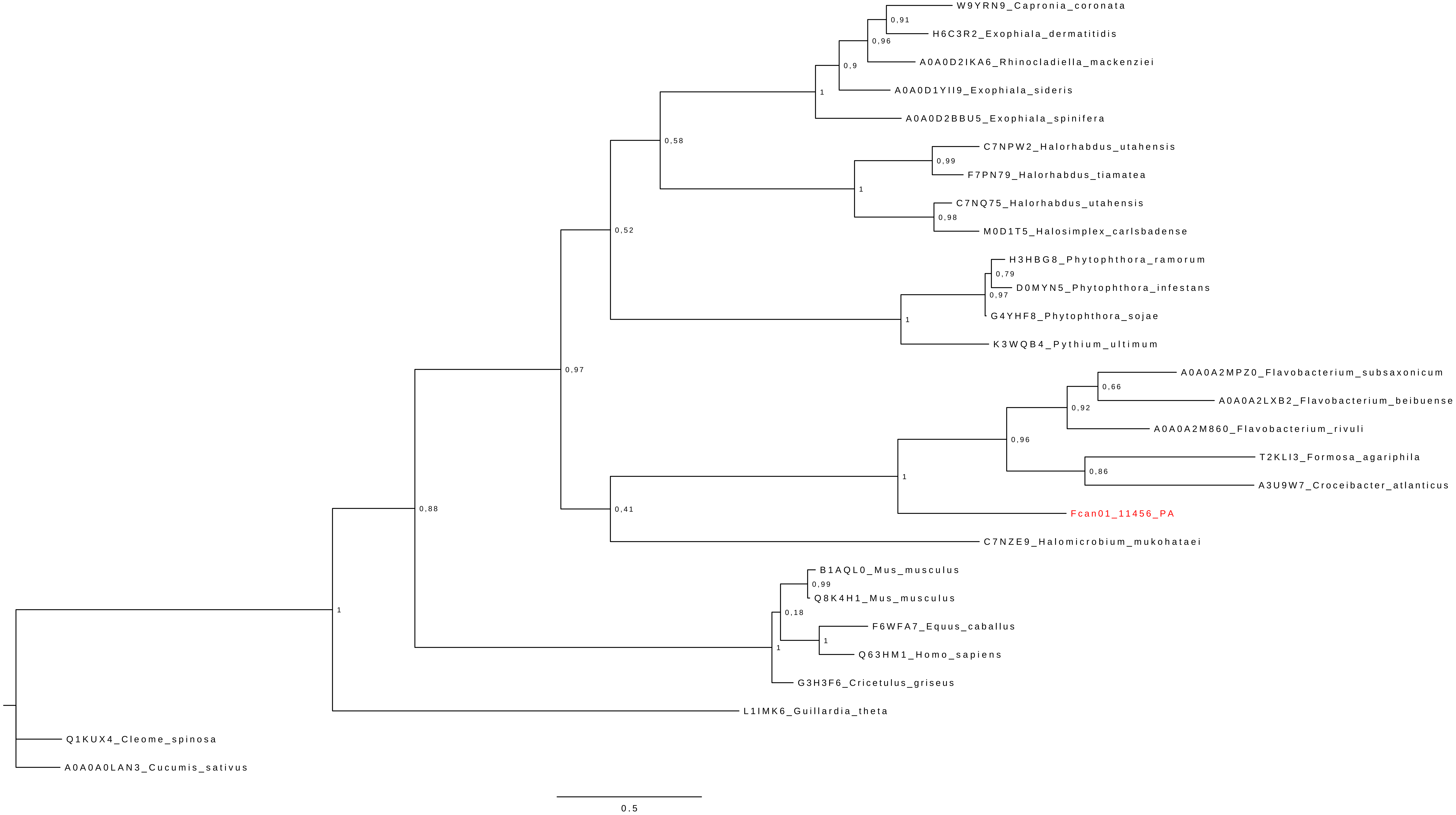
W9YRN9_Capronia_coronata
0,91
H6C3R2_Exophiala_dermatitidis
0,96
A0A0D2IKA6_Rhinocladiella_mackenziei
0,9
A0A0D1YII9_Exophiala_sideris
1
A0A0D2BBU5_Exophiala_spinifera
0,58
C7NPW2_Halorhabdus_utahensis
0,99
F7PN79_Halorhabdus_tiamatea
1
C7NQ75_Halorhabdus_utahensis
0,98
M0D1T5_Halosimplex_carlsbadense
0,52
H3HBG8_Phytophthora_ramorum
0,79
D0MYN5_Phytophthora_infestans
0,97
G4YHF8_Phytophthora_sojae
1
K3WQB4_Pythium_ultimum
0,97
A0A0A2MPZ0_Flavobacterium_subsaxonicum
0,66
A0A0A2LXB2_Flavobacterium_beibuense
0,92
A0A0A2M860_Flavobacterium_rivuli
0,96
T2KLI3_Formosa_agariphila
0,86
A3U9W7_Croceibacter_atlanticus
1
Fcan01_11456_PA
0,88
0,41
C7NZE9_Halomicrobium_mukohataei
B1AQL0_Mus_musculus
0,99
Q8K4H1_Mus_musculus
0,18
F6WFA7_Equus_caballus
1
Q63HM1_Homo_sapiens
1
1
G3H3F6_Cricetulus_griseus
L1IMK6_Guillardia_theta
Q1KUX4_Cleome_spinosa
A0A0A0LAN3_Cucumis_sativus
0.5

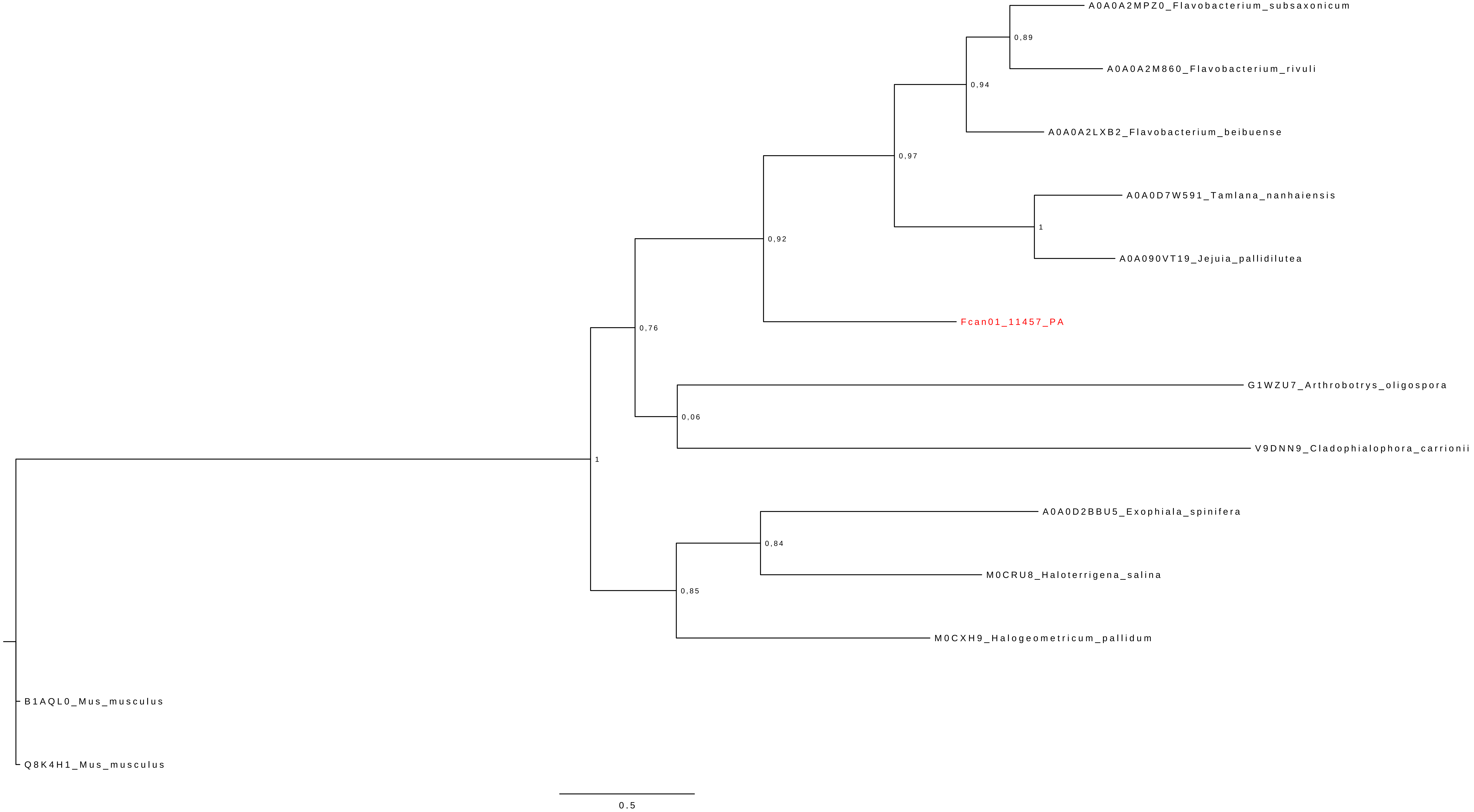
A0A0A2MPZ0_Flavobacterium_subsaxonicum
0,89
A0A0A2M860_Flavobacterium_rivuli
0,94
A0A0A2LXB2_Flavobacterium_beibuense
0,97
A0A0D7W591_Tamlana_nanhaiensis
1
A0A090VT19_Jejuia_pallidilutea
0,92
Fcan01_11457_PA
0,76
G1WZU7_Arthrobotrys_oligospora
0,06
V9DNN9_Cladophialophora_carrionii
1
A0A0D2BBU5_Exophiala_spinifera
0,84
M0CRU8_Haloterrigena_salina
0,85
M0CXH9_Halogeometricum_pallidum
B1AQL0_Mus_musculus
Q8K4H1_Mus_musculus
0.5

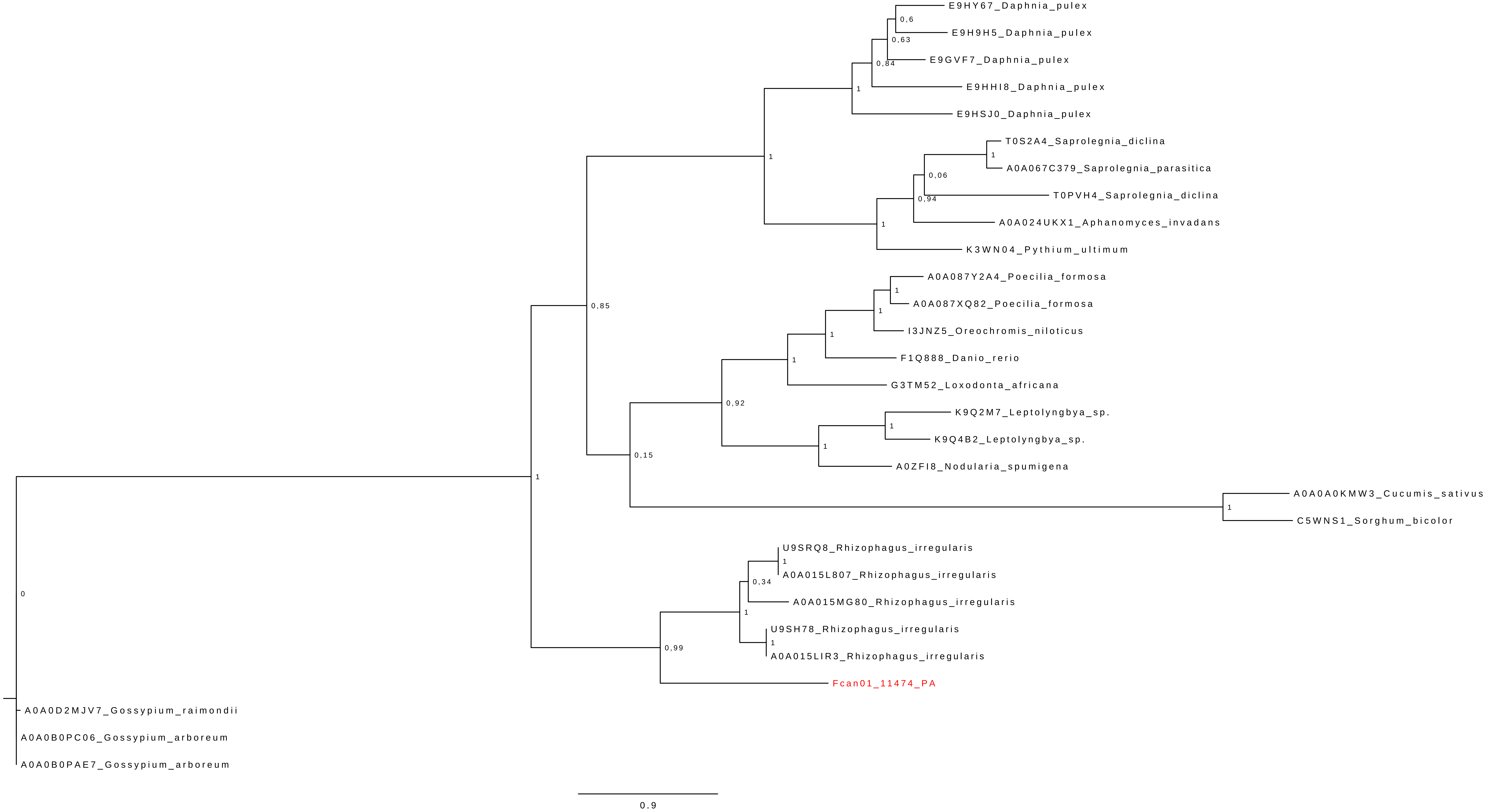
E9HY67_Daphnia_pulex
0,6
E9H9H5_Daphnia_pulex
0,63
E9GVF7_Daphnia_pulex
0,84
E9HHI8_Daphnia_pulex
1
E9HSJ0_Daphnia_pulex
1
T0S2A4_Saprolegnia_diclina
1
A0A067C379_Saprolegnia_parasitica
0,06
T0PVH4_Saprolegnia_diclina
0,94
A0A024UKX1_Aphanomyces_invadans
1
K3WN04_Pythium_ultimum
A0A087Y2A4_Poecilia_formosa
1
A0A087XQ82_Poecilia_formosa
1
I3JNZ5_Oreochromis_niloticus
1
F1Q888_Danio_rerio
1
G3TM52_Loxodonta_africana
0,85
0,92
K9Q2M7_Leptolyngbya_sp.
1
K9Q4B2_Leptolyngbya_sp.
1
A0ZFI8_Nodularia_spumigena
0,15
1
A0A0A0KMW3_Cucumis_sativus
1
C5WNS1_Sorghum_bicolor
U9SRQ8_Rhizophagus_irregularis
1
A0A015L807_Rhizophagus_irregularis
0,34
A0A015MG80_Rhizophagus_irregularis
1
U9SH78_Rhizophagus_irregularis
1
A0A015LIR3_Rhizophagus_irregularis
0,99
Fcan01_11474_PA
0
A0A0D2MJV7_Gossypium_raimondii
A0A0B0PC06_Gossypium_arboreum
A0A0B0PAE7_Gossypium_arboreum
0.9

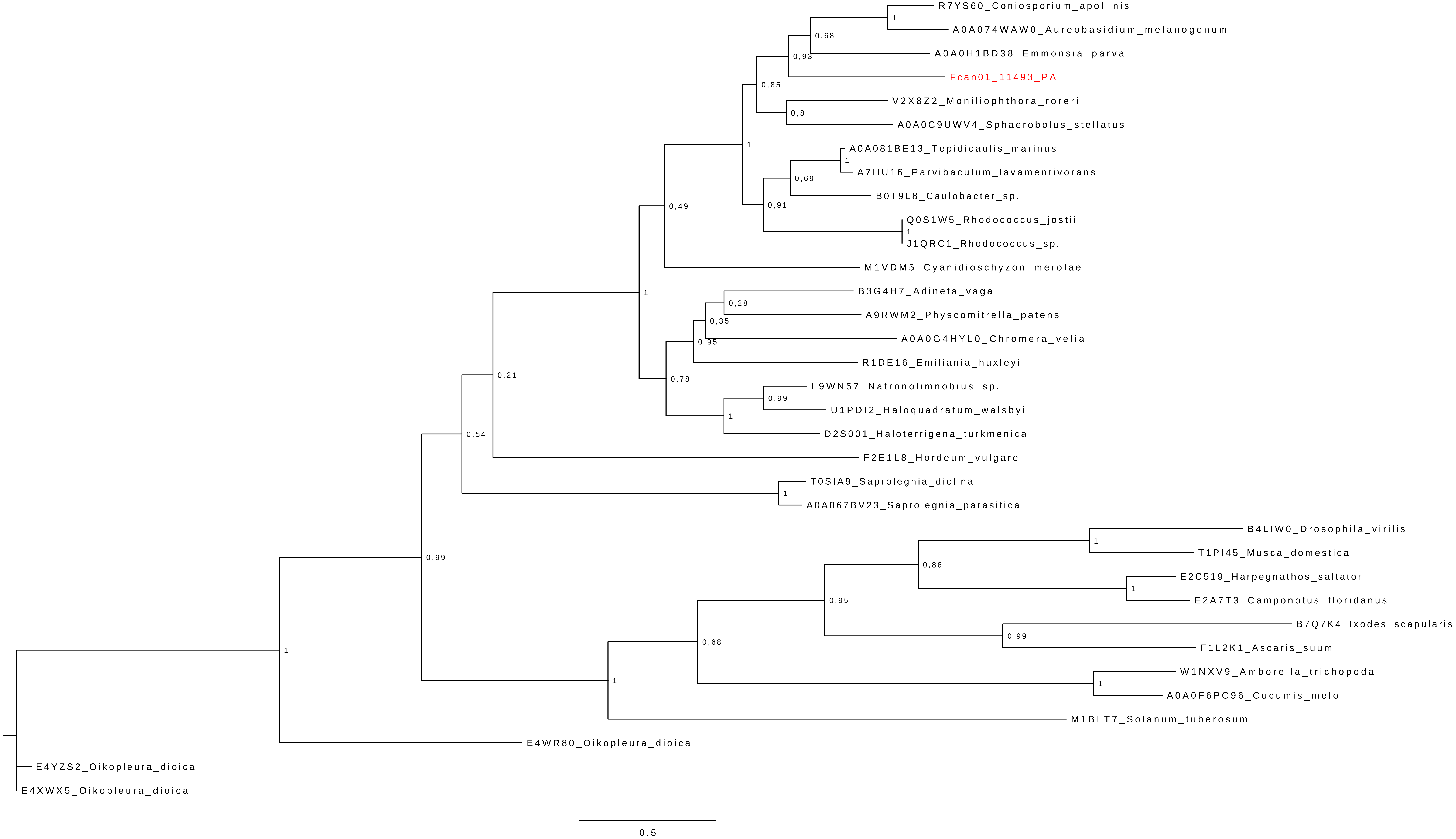
R7YS60_Coniosporium_apollinis
1
A0A074WAW0_Aureobasidium_melanogenum
0,68
A0A0H1BD38_Emmonsia_parva
0,93
Fcan01_11493_PA
0,85
V2X8Z2_Moniliophthora_roreri
0,8
A0A0C9UWV4_Sphaerobolus_stellatus
1
A0A081BE13_Tepidicaulis_marinus
1
A7HU16_Parvibaculum_lavamentivorans
0,69
B0T9L8_Caulobacter_sp.
0,91
Q0S1W5_Rhodococcus_jostii
1
J1QRC1_Rhodococcus_sp.
0,49
M1VDM5_Cyanidioschyzon_merolae
1
B3G4H7_Adineta_vaga
0,28
A9RWM2_Physcomitrella_patens
0,35
A0A0G4HYL0_Chromera_velia
0,95
R1DE16_Emiliania_huxleyi
0,78
L9WN57_Natronolimnobius_sp.
0,99
U1PDI2_Haloquadratum_walsbyi
1
D2S001_Haloterrigena_turkmenica
0,21
0,54
F2E1L8_Hordeum_vulgare
T0SIA9_Saprolegnia_diclina
1
A0A067BV23_Saprolegnia_parasitica
B4LIW0_Drosophila_virilis
1
T1PI45_Musca_domestica
0,86
E2C519_Harpegnathos_saltator
1
E2A7T3_Camponotus_floridanus
0,95
B7Q7K4_Ixodes_scapularis
0,99
F1L2K1_Ascaris_suum
0,68
W1NXV9_Amborella_trichopoda
1
A0A0F6PC96_Cucumis_melo
1
M1BLT7_Solanum_tuberosum
0,99
1
E4WR80_Oikopleura_dioica
E4YZS2_Oikopleura_dioica
E4XWX5_Oikopleura_dioica
0.5

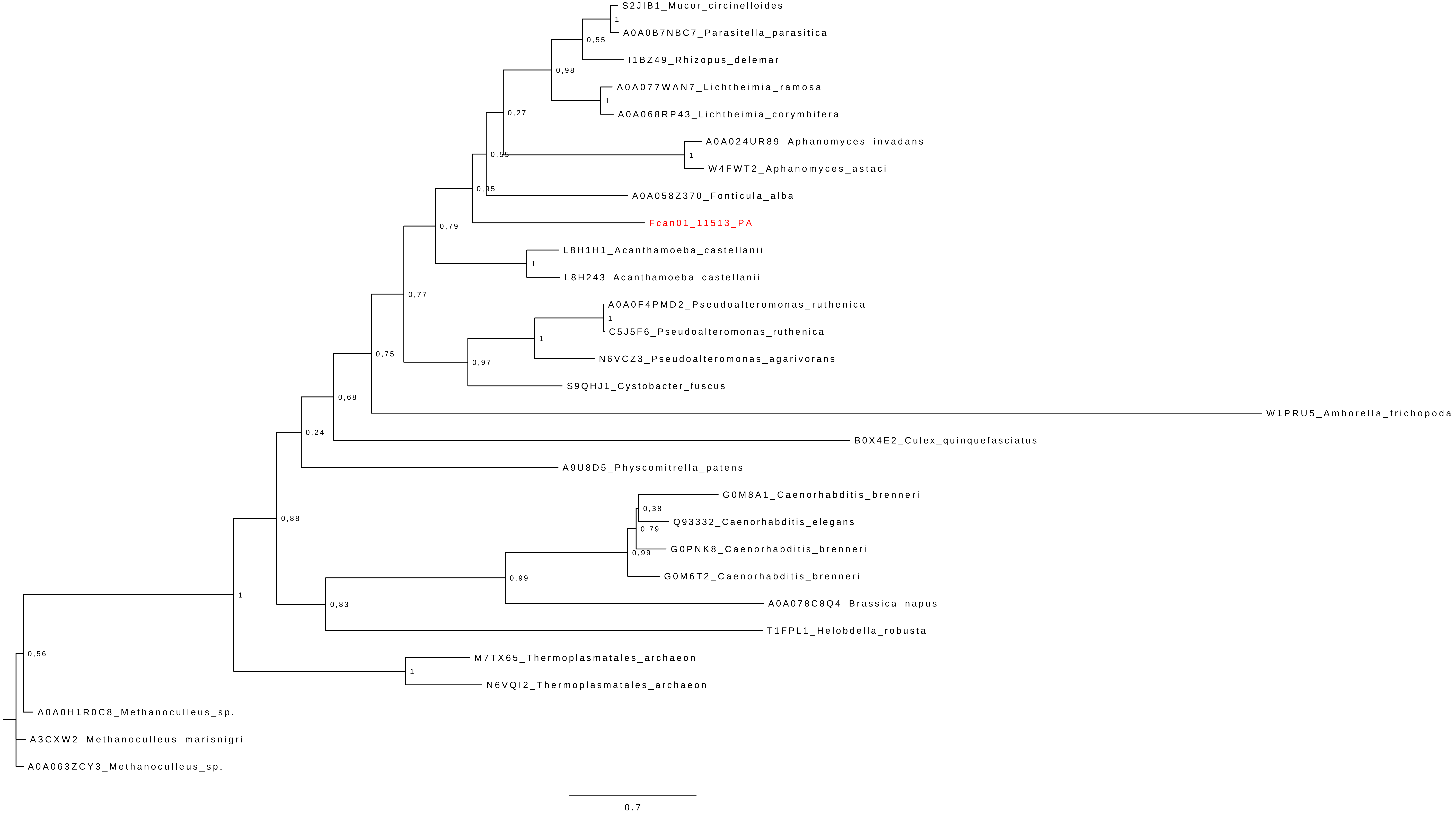
S2JIB1_Mucor_circinelloides
1
A0A0B7NBC7_Parasitella_parasitica
0,55
I1BZ49_Rhizopus_delemar
0,98
A0A077WAN7_Lichtheimia_ramosa
1
A0A068RP43_Lichtheimia_corymbifera
0,27
A0A024UR89_Aphanomyces_invadans
0,55
1
W4FWT2_Aphanomyces_astaci
0,95
A0A058Z370_Fonticula_alba
Fcan01_11513_PA
0,79
L8H1H1_Acanthamoeba_castellanii
1
L8H243_Acanthamoeba_castellanii
0,77
A0A0F4PMD2_Pseudoalteromonas_ruthenica
1
1
C5J5F6_Pseudoalteromonas_ruthenica
0,75
N6VCZ3_Pseudoalteromonas_agarivorans
0,97
S9QHJ1_Cystobacter_fuscus
0,68
W1PRU5_Amborella_trichopoda
0,24
B0X4E2_Culex_quinquefasciatus
A9U8D5_Physcomitrella_patens
G0M8A1_Caenorhabditis_brenneri
0,38
0,88
Q93332_Caenorhabditis_elegans
0,79
G0PNK8_Caenorhabditis_brenneri
0,99
G0M6T2_Caenorhabditis_brenneri
0,99
1
A0A078C8Q4_Brassica_napus
0,83
T1FPL1_Helobdella_robusta
M7TX65_Thermoplasmatales_archaeon
1
0,56
N6VQI2_Thermoplasmatales_archaeon
A0A0H1R0C8_Methanoculleus_sp.
A3CXW2_Methanoculleus_marisnigri
A0A063ZCY3_Methanoculleus_sp.
0.7

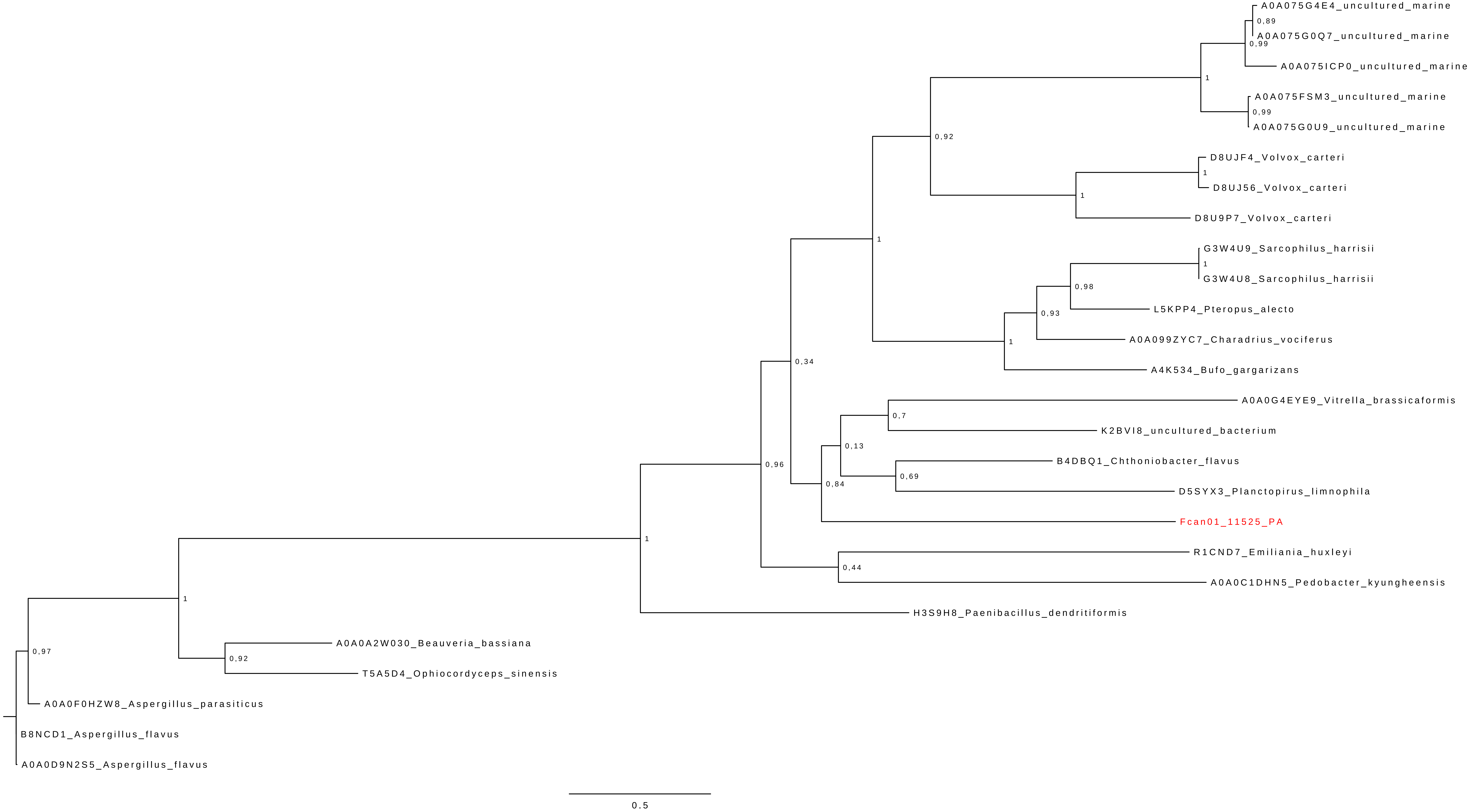
A0A075G4E4_uncultured_marine
0,89
A0A075G0Q7_uncultured_marine
0,99
A0A075ICP0_uncultured_marine
1
A0A075FSM3_uncultured_marine
0,99
A0A075G0U9_uncultured_marine
0,92
D8UJF4_Volvox_carteri
1
D8UJ56_Volvox_carteri
1
D8U9P7_Volvox_carteri
1
G3W4U9_Sarcophilus_harrisii
1
G3W4U8_Sarcophilus_harrisii
0,98
L5KPP4_Pteropus_alecto
0,93
A0A099ZYC7_Charadrius_vociferus
1
A4K534_Bufo_gargarizans
0,34
A0A0G4EYE9_Vitrella_brassicaformis
0,7
K2BVI8_uncultured_bacterium
0,13
B4DBQ1_Chthoniobacter_flavus
0,69
D5SYX3_Planctopirus_limnophila
0,84
0,96
Fcan01_11525_PA
R1CND7_Emiliania_huxleyi
0,44
A0A0C1DHN5_Pedobacter_kyungheensis
1
H3S9H8_Paenibacillus_dendritiformis
1
A0A0A2W030_Beauveria_bassiana
0,92
T5A5D4_Ophiocordyceps_sinensis
0,97
A0A0F0HZW8_Aspergillus_parasiticus
B8NCD1_Aspergillus_flavus
A0A0D9N2S5_Aspergillus_flavus
0.5

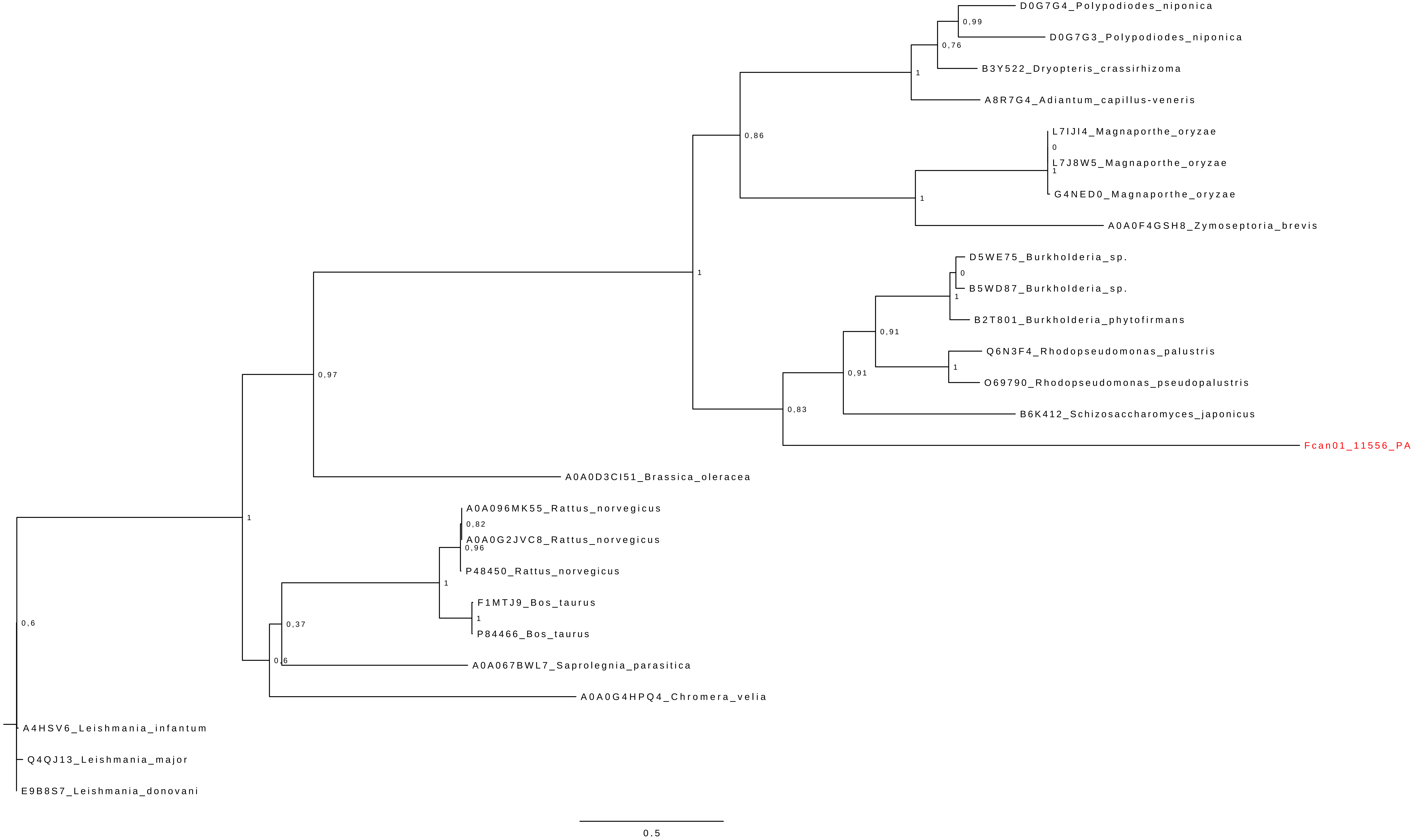
D0G7G4_Polypodiodes_niponica
0,99
D0G7G3_Polypodiodes_niponica
0,76
B3Y522_Dryopteris_crassirhizoma
1
A8R7G4_Adiantum_capillus-veneris
0,86
L7IJI4_Magnaporthe_oryzae
0
L7J8W5_Magnaporthe_oryzae
1
G4NED0_Magnaporthe_oryzae
1
A0A0F4GSH8_Zymoseptoria_brevis
1
D5WE75_Burkholderia_sp.
0
B5WD87_Burkholderia_sp.
1
B2T801_Burkholderia_phytofirmans
0,91
Q6N3F4_Rhodopseudomonas_palustris
1
O69790_Rhodopseudomonas_pseudopalustris
0,91
B6K412_Schizosaccharomyces_japonicus
0,83
Fcan01_11556_PA
0,97
A0A0D3CI51_Brassica_oleracea
1
A0A096MK55_Rattus_norvegicus
0,82
A0A0G2JVC8_Rattus_norvegicus
0,96
P48450_Rattus_norvegicus
1
F1MTJ9_Bos_taurus
1
P84466_Bos_taurus
0,37
A0A067BWL7_Saprolegnia_parasitica
0,6
A0A0G4HPQ4_Chromera_velia
0,6
A4HSV6_Leishmania_infantum
Q4QJ13_Leishmania_major
E9B8S7_Leishmania_donovani
0.5

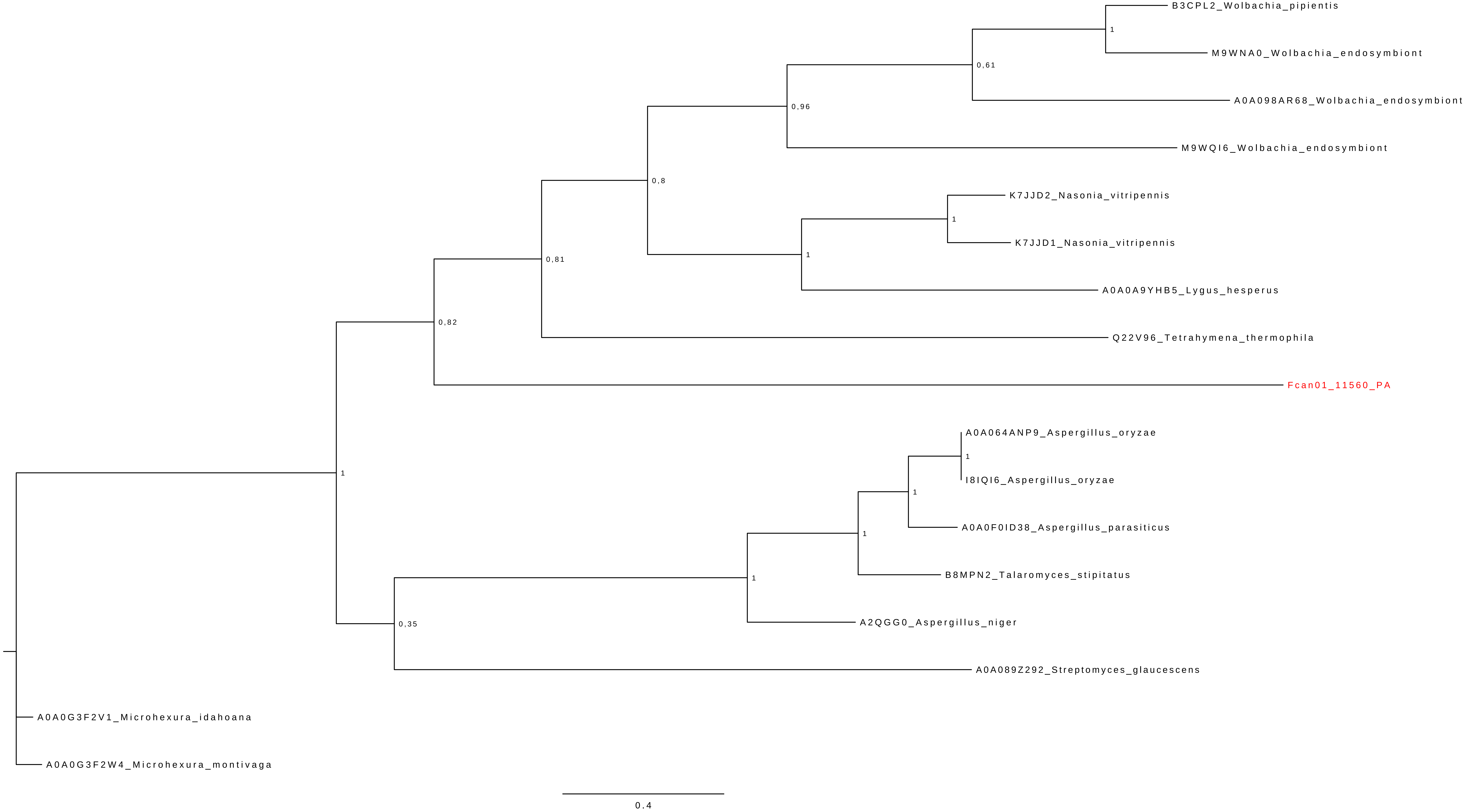
B3CPL2_Wolbachia_pipientis
1
M9WNA0_Wolbachia_endosymbiont
0,61
A0A098AR68_Wolbachia_endosymbiont
0,96
M9WQI6_Wolbachia_endosymbiont
0,8
K7JJD2_Nasonia_vitripennis
1
K7JJD1_Nasonia_vitripennis
1
A0A0A9YHB5_Lygus_hesperus
0,81
Q22V96_Tetrahymena_thermophila
0,82
Fcan01_11560_PA
A0A064ANP9_Aspergillus_oryzae
1
I8IQI6_Aspergillus_oryzae
1
A0A0F0ID38_Aspergillus_parasiticus
1
B8MPN2_Talaromyces_stipitatus
1
A2QGG0_Aspergillus_niger
0,35
A0A089Z292_Streptomyces_glaucescens
1
A0A0G3F2V1_Microhexura_idahoana
A0A0G3F2W4_Microhexura_montivaga
0.4

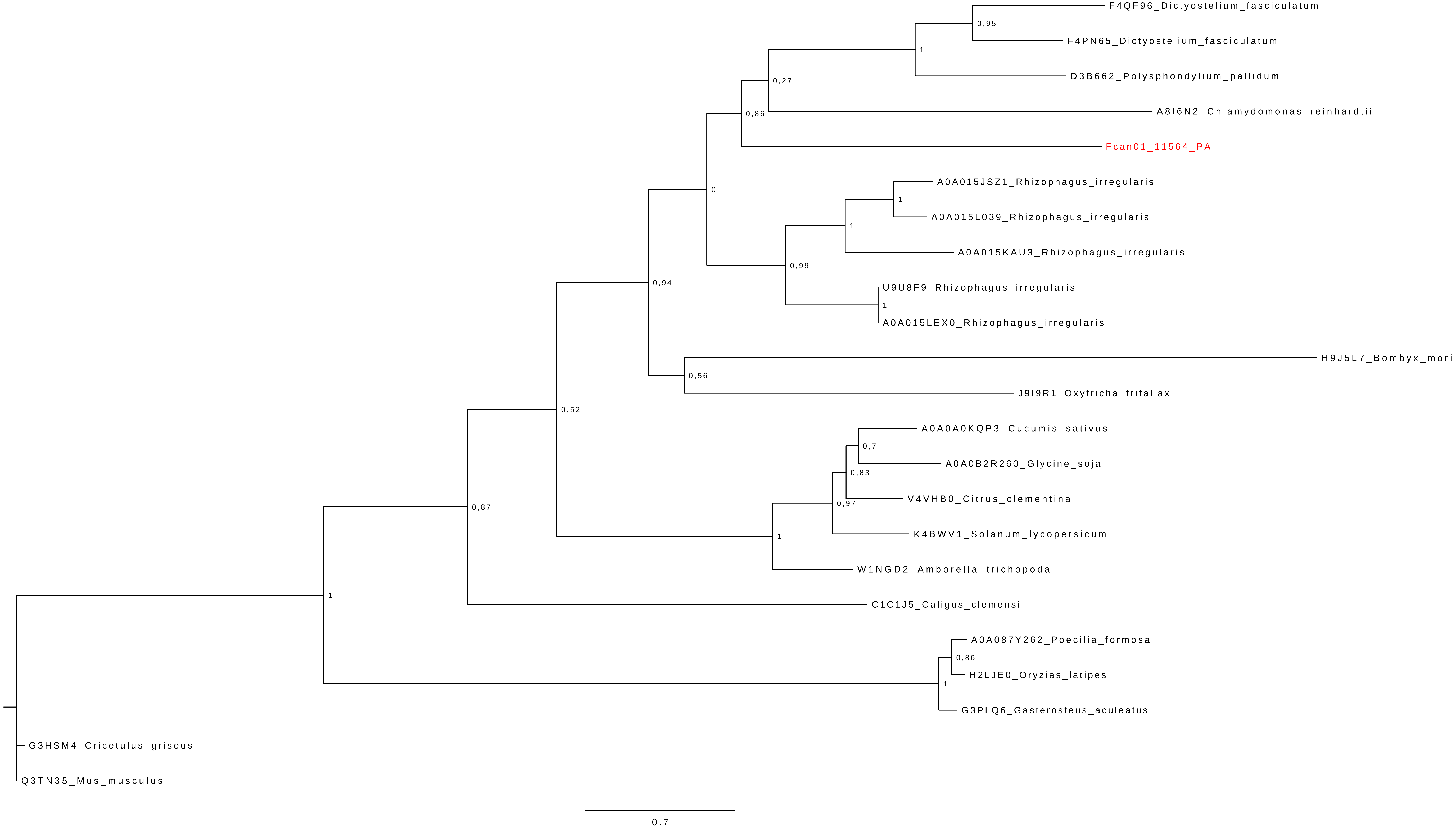
F4QF96_Dictyostelium_fasciculatum
0,95
F4PN65_Dictyostelium_fasciculatum
1
D3B662_Polysphondylium_pallidum
0,27
A8I6N2_Chlamydomonas_reinhardtii
0,86
Fcan01_11564_PA
A0A015JSZ1_Rhizophagus_irregularis
1
0
A0A015L039_Rhizophagus_irregularis
1
A0A015KAU3_Rhizophagus_irregularis
0,99
U9U8F9_Rhizophagus_irregularis
1
0,94
A0A015LEX0_Rhizophagus_irregularis
H9J5L7_Bombyx_mori
0,56
J9I9R1_Oxytricha_trifallax
0,52
A0A0A0KQP3_Cucumis_sativus
0,7
A0A0B2R260_Glycine_soja
0,83
V4VHB0_Citrus_clementina
0,97
0,87
K4BWV1_Solanum_lycopersicum
1
W1NGD2_Amborella_trichopoda
1
C1C1J5_Caligus_clemensi
A0A087Y262_Poecilia_formosa
0,86
H2LJE0_Oryzias_latipes
1
G3PLQ6_Gasterosteus_aculeatus
G3HSM4_Cricetulus_griseus
Q3TN35_Mus_musculus
0.7

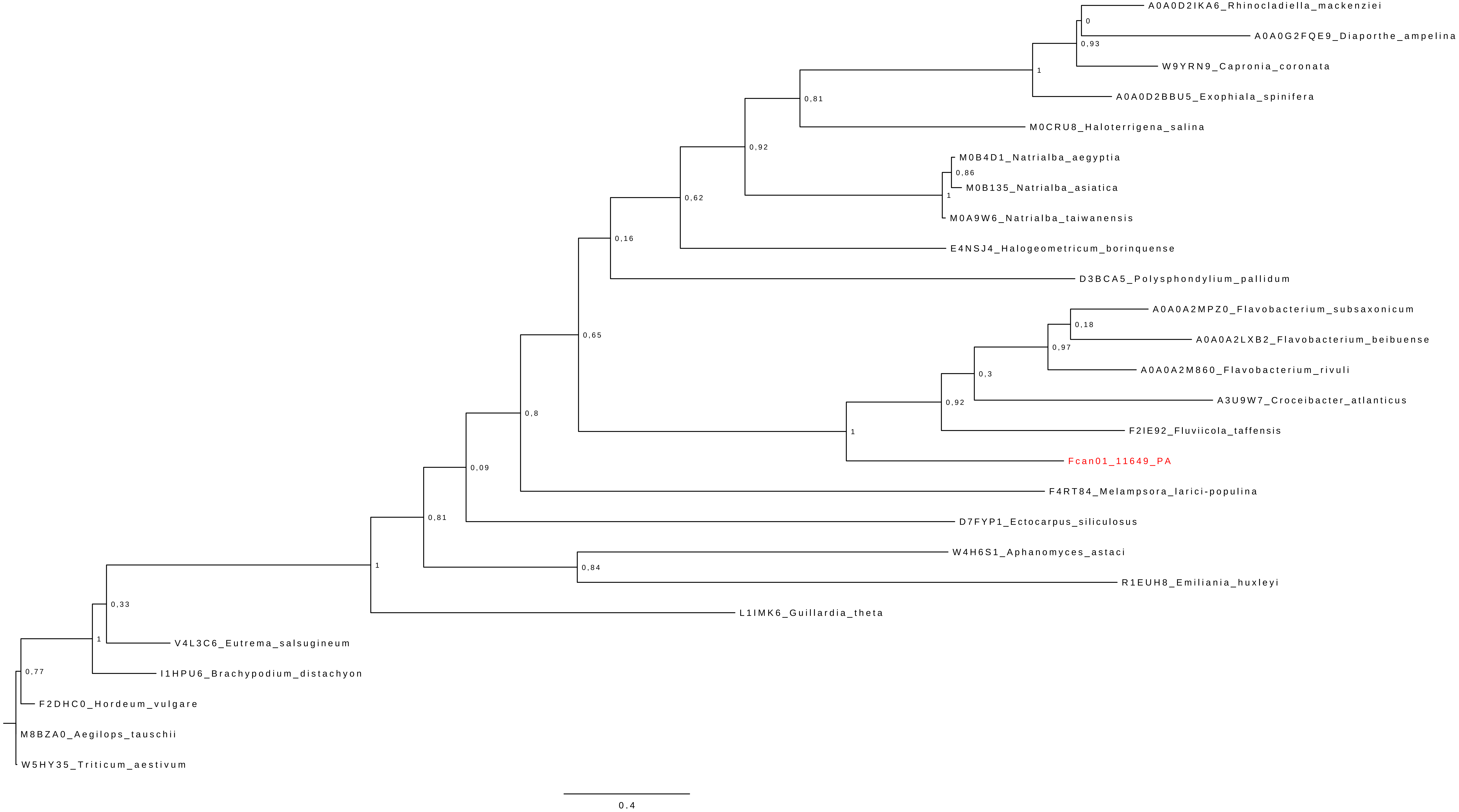

A0A0D2IKA6_Rhinocladiella_mackenziei
0
A0A0G2FQE9_Diaporthe_ampelina
0,93
W9YRN9_Capronia_coronata
1
A0A0D2BBU5_Exophiala_spinifera
0,81
M0CRU8_Haloterrigena_salina
0,92
M0B4D1_Natrialba_aegyptia
0,86
M0B135_Natrialba_asiatica
1
M0A9W6_Natrialba_taiwanensis
0,62
E4NSJ4_Halogeometricum_borinquense
0,16
D3BCA5_Polysphondylium_pallidum
A0A0A2MPZ0_Flavobacterium_subsaxonicum
0,18
A0A0A2LXB2_Flavobacterium_beibuense
0,97
A0A0A2M860_Flavobacterium_rivuli
0,65
0,3
A3U9W7_Croceibacter_atlanticus
0,92
0,8
F2IE92_Fluviicola_taffensis
1
Fcan01_11649_PA
F4RT84_Melampsora_larici-populina
0,09
D7FYP1_Ectocarpus_siliculosus
0,81
W4H6S1_Aphanomyces_astaci
0,84
R1EUH8_Emiliania_huxleyi
1
L1IMK6_Guillardia_theta
0,33
V4L3C6_Eutrema_salsugineum
1
I1HPU6_Brachypodium_distachyon
0,77
F2DHC0_Hordeum_vulgare
M8BZA0_Aegilops_tauschii
W5HY35_Triticum_aestivum
0.4

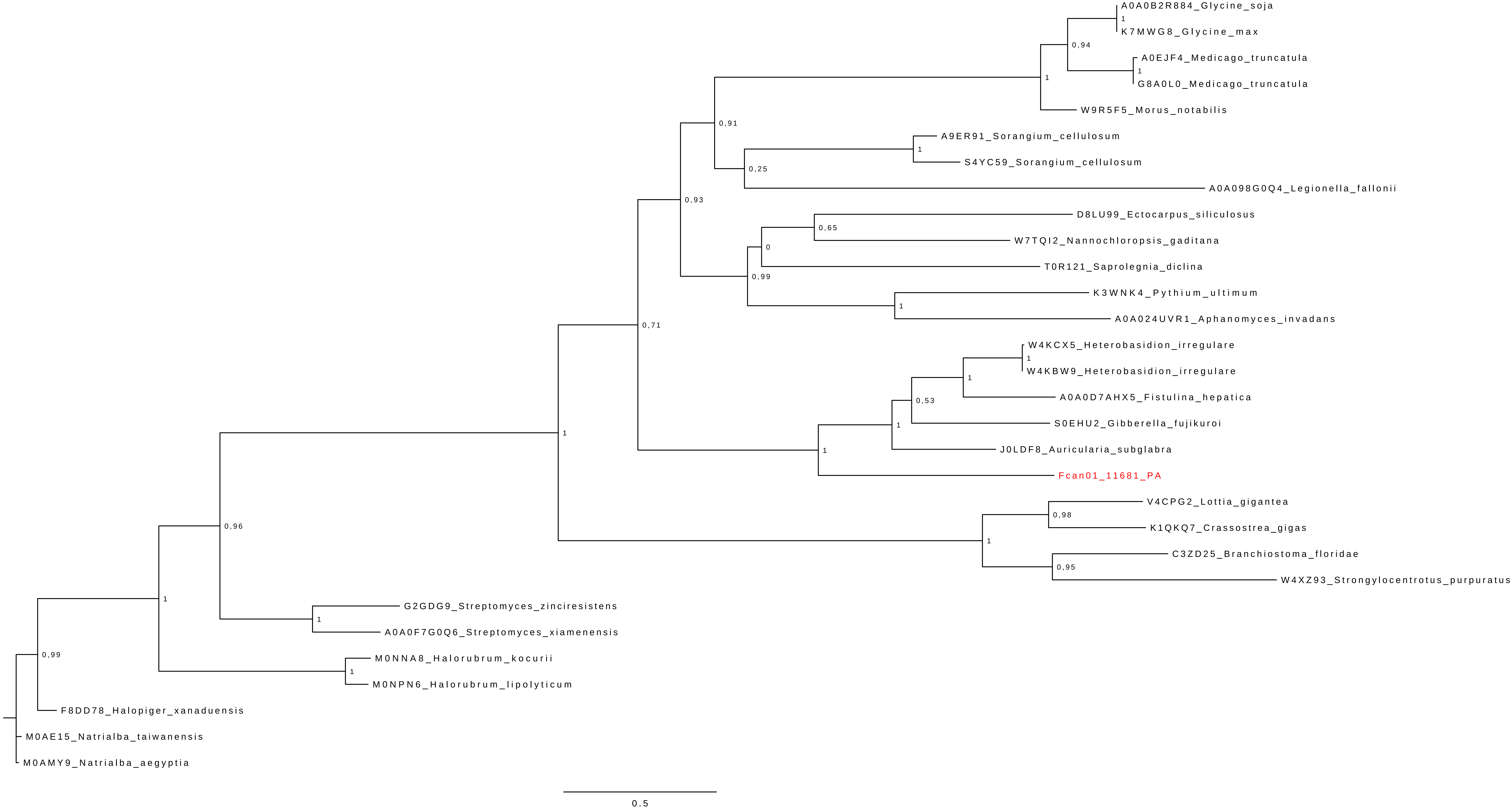
A0A0B2R884_Glycine_soja
1
K7MWG8_Glycine_max
0,94
A0EJF4_Medicago_truncatula
1
G8A0L0_Medicago_truncatula
1
W9R5F5_Morus_notabilis
0,91
A9ER91_Sorangium_cellulosum
1
S4YC59_Sorangium_cellulosum
0,25
A0A098G0Q4_Legionella_fallonii
0,93
D8LU99_Ectocarpus_siliculosus
0,65
W7TQI2_Nannochloropsis_gaditana
0
T0R121_Saprolegnia_diclina
0,99
K3WNK4_Pythium_ultimum
1
A0A024UVR1_Aphanomyces_invadans
0,71
W4KCX5_Heterobasidion_irregulare
1
W4KBW9_Heterobasidion_irregulare
1
A0A0D7AHX5_Fistulina_hepatica
0,53
S0EHU2_Gibberella_fujikuroi
1
J0LDF8_Auricularia_subglabra
1
Fcan01_11681_PA
1
V4CPG2_Lottia_gigantea
0,98
K1QKQ7_Crassostrea_gigas
1
C3ZD25_Branchiostoma_floridae
0,95
W4XZ93_Strongylocentrotus_purpuratus
0,96
G2GDG9_Streptomyces_zinciresistens
1
A0A0F7G0Q6_Streptomyces_xiamenensis
1
M0NNA8_Halorubrum_kocurii
1
M0NPN6_Halorubrum_lipolyticum
0,99
F8DD78_Halopiger_xanaduensis
M0AE15_Natrialba_taiwanensis
M0AMY9_Natrialba_aegyptia
0.5

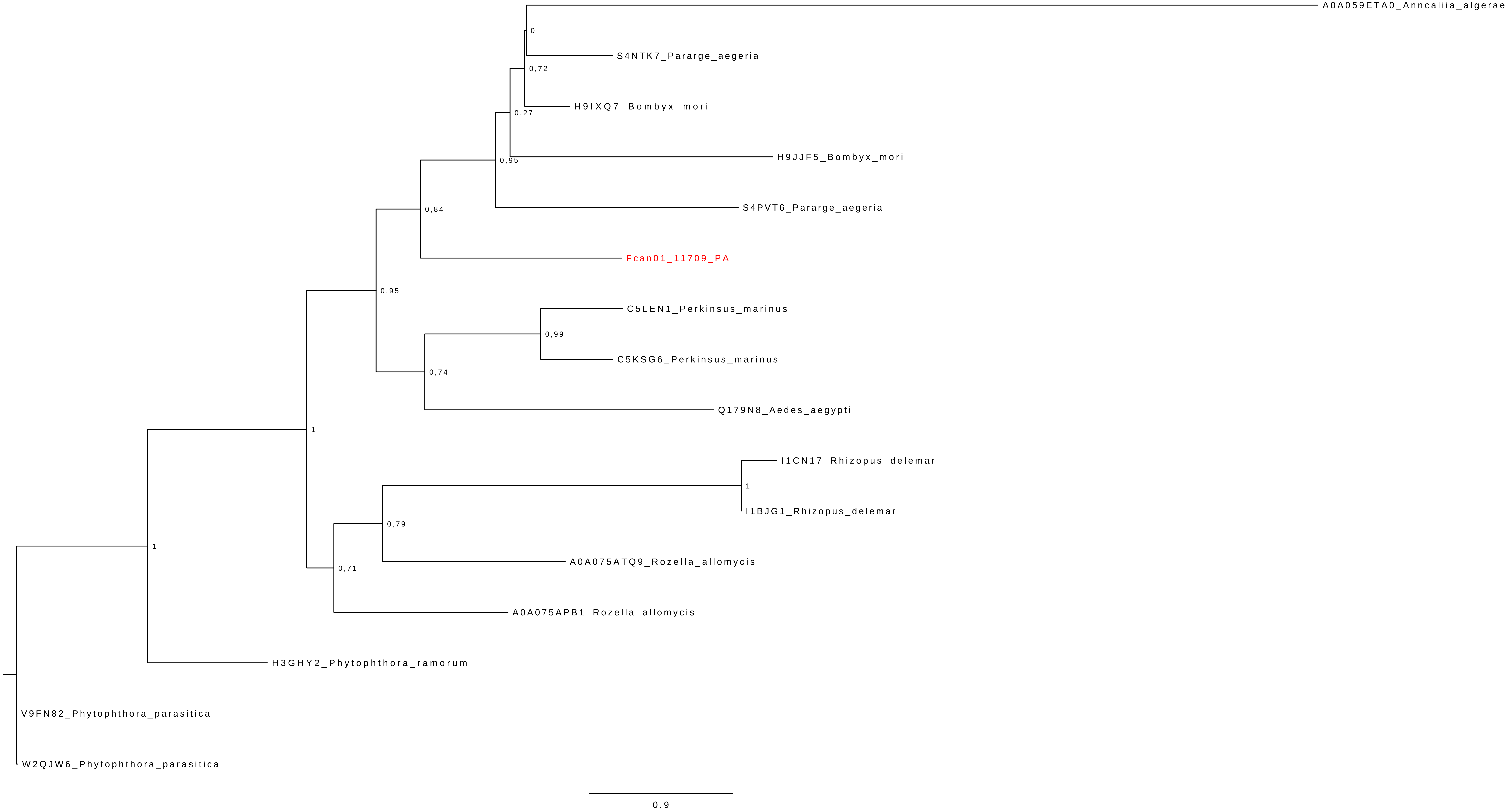
A0A059ETA0_Anncaliia_algerae
0
S4NTK7_Pararge_aegeria
0,72
H9IXQ7_Bombyx_mori
0,27
H9JJF5_Bombyx_mori
0,95
S4PVT6_Pararge_aegeria
0,84
Fcan01_11709_PA
0,95
C5LEN1_Perkinsus_marinus
0,99
C5KSG6_Perkinsus_marinus
0,74
Q179N8_Aedes_aegypti
1
I1CN17_Rhizopus_delemar
1
I1BJG1_Rhizopus_delemar
0,79
A0A075ATQ9_Rozella_allomycis
1
0,71
A0A075APB1_Rozella_allomycis
H3GHY2_Phytophthora_ramorum
V9FN82_Phytophthora_parasitica
W2QJW6_Phytophthora_parasitica
0.9

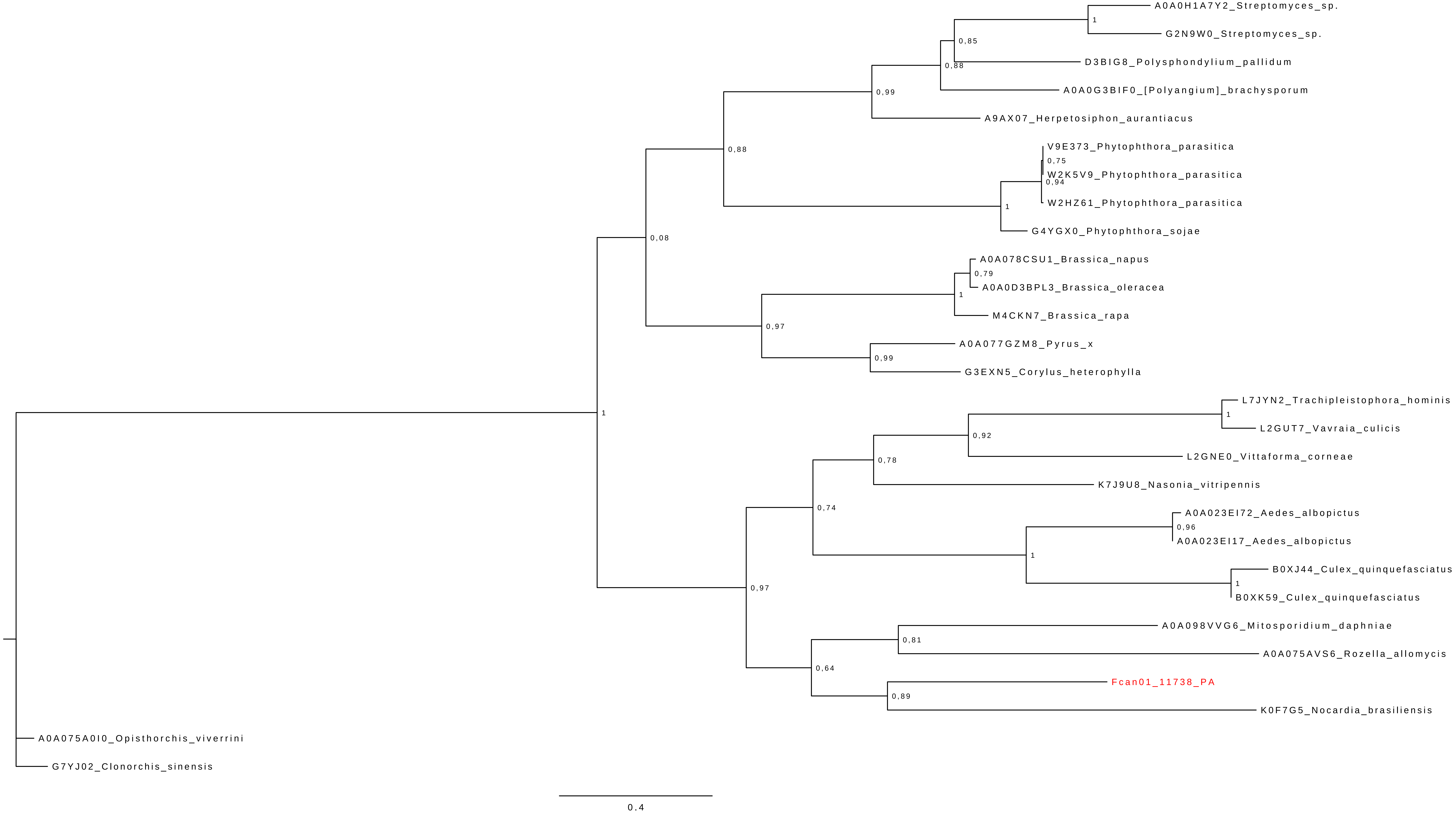
A0A0H1A7Y2_Streptomyces_sp.
G2N9W0_Streptomyces_sp.
D3BIG8_Polysphondylium_pallidum
A0A0G3BIF0_[Polyangium]_brachysporum
A9AX07_Herpetosiphon_aurantiacus
V9E373_Phytophthora_parasitica
W2K5V9_Phytophthora_parasitica
W2HZ61_Phytophthora_parasitica
G4YGX0_Phytophthora_sojae
A0A078CSU1_Brassica_napus
A0A0D3BPL3_Brassica_oleracea
M4CKN7_Brassica_rapa
A0A077GZM8_Pyrus_x
G3EXN5_Corylus_heterophylla
L7JYN2_Trachipleistophora_hominis
L2GUT7_Vavraia_culicis
L2GNE0_Vittaforma_corneae
K7J9U8_Nasonia_vitripennis
A0A023EI72_Aedes_albopictus
A0A023EI17_Aedes_albopictus
B0XJ44_Culex_quinquefasciatus
B0XK59_Culex_quinquefasciatus
A0A098VVG6_Mitosporidium_daphniae
A0A075AVS6_Rozella_allomycis
Fcan01_11738_PA
K0F7G5_Nocardia_brasiliensis
A0A075A0I0_Opisthorchis_viverrini
G7YJ02_Clonorchis_sinensis
1
0,85
0,88
0,99
0,88
0,75
0,94
1
0,08
0,79
1
0,97
0,99
1
1
0,92
0,78
0,74
0,96
1
1
0,97
0,81
0,64
0,89
0.4

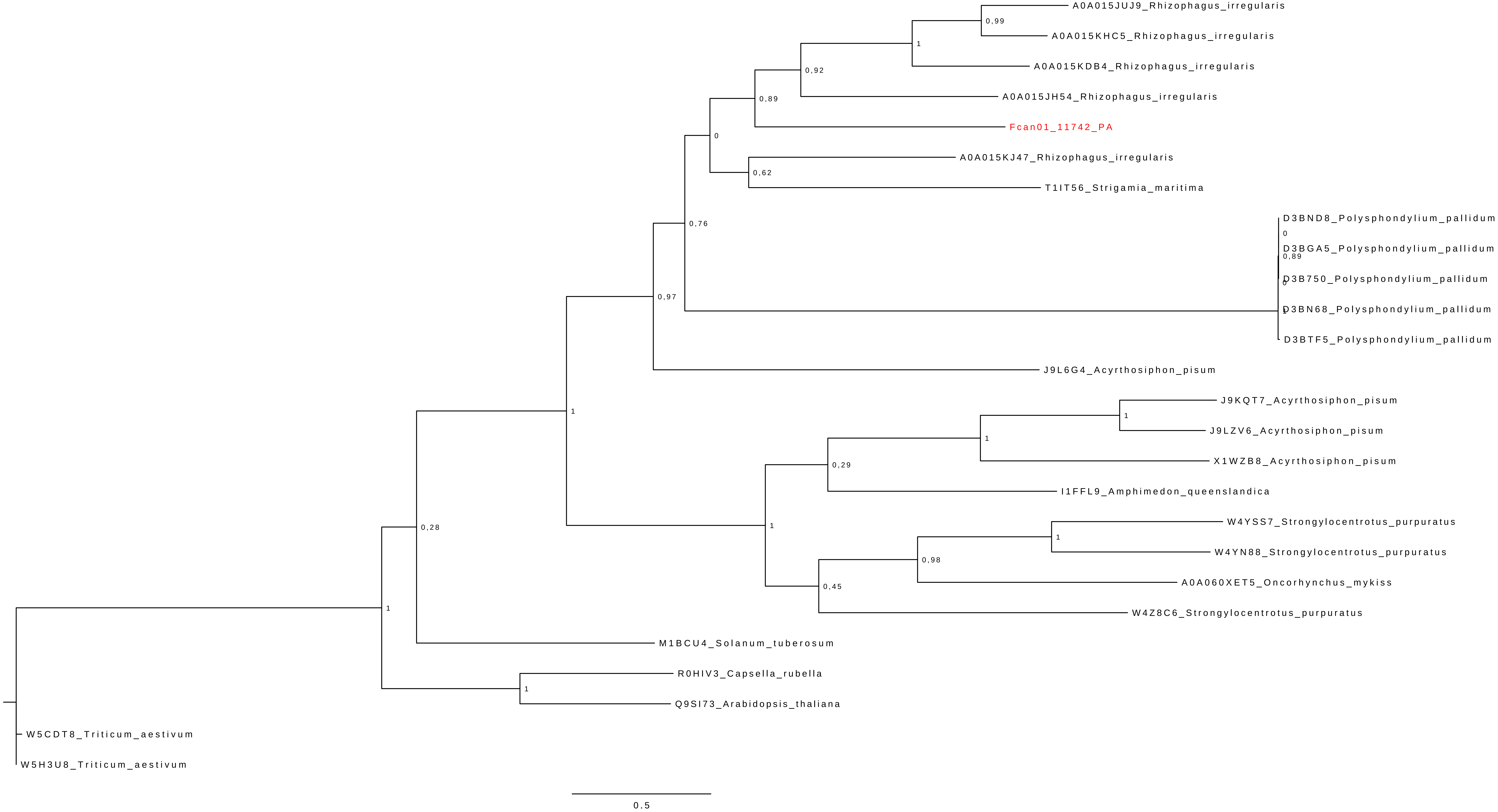
A0A015JUJ9_Rhizophagus_irregularis
A0A015KHC5_Rhizophagus_irregularis
A0A015KDB4_Rhizophagus_irregularis
A0A015JH54_Rhizophagus_irregularis
Fcan01_11742_PA
A0A015KJ47_Rhizophagus_irregularis
T1IT56_Strigamia_maritima
D3BND8_Polysphondylium_pallidum
D3BGA5_Polysphondylium_pallidum
D3B750_Polysphondylium_pallidum
D3BN68_Polysphondylium_pallidum
D3BTF5_Polysphondylium_pallidum
J9L6G4_Acyrthosiphon_pisum
J9KQT7_Acyrthosiphon_pisum
J9LZV6_Acyrthosiphon_pisum
X1WZB8_Acyrthosiphon_pisum
I1FFL9_Amphimedon_queenslandica
W4YSS7_Strongylocentrotus_purpuratus
W4YN88_Strongylocentrotus_purpuratus
A0A060XET5_Oncorhynchus_mykiss
W4Z8C6_Strongylocentrotus_purpuratus
M1BCU4_Solanum_tuberosum
R0HIV3_Capsella_rubella
Q9SI73_Arabidopsis_thaliana
W5CDT8_Triticum_aestivum
W5H3U8_Triticum_aestivum
0.5

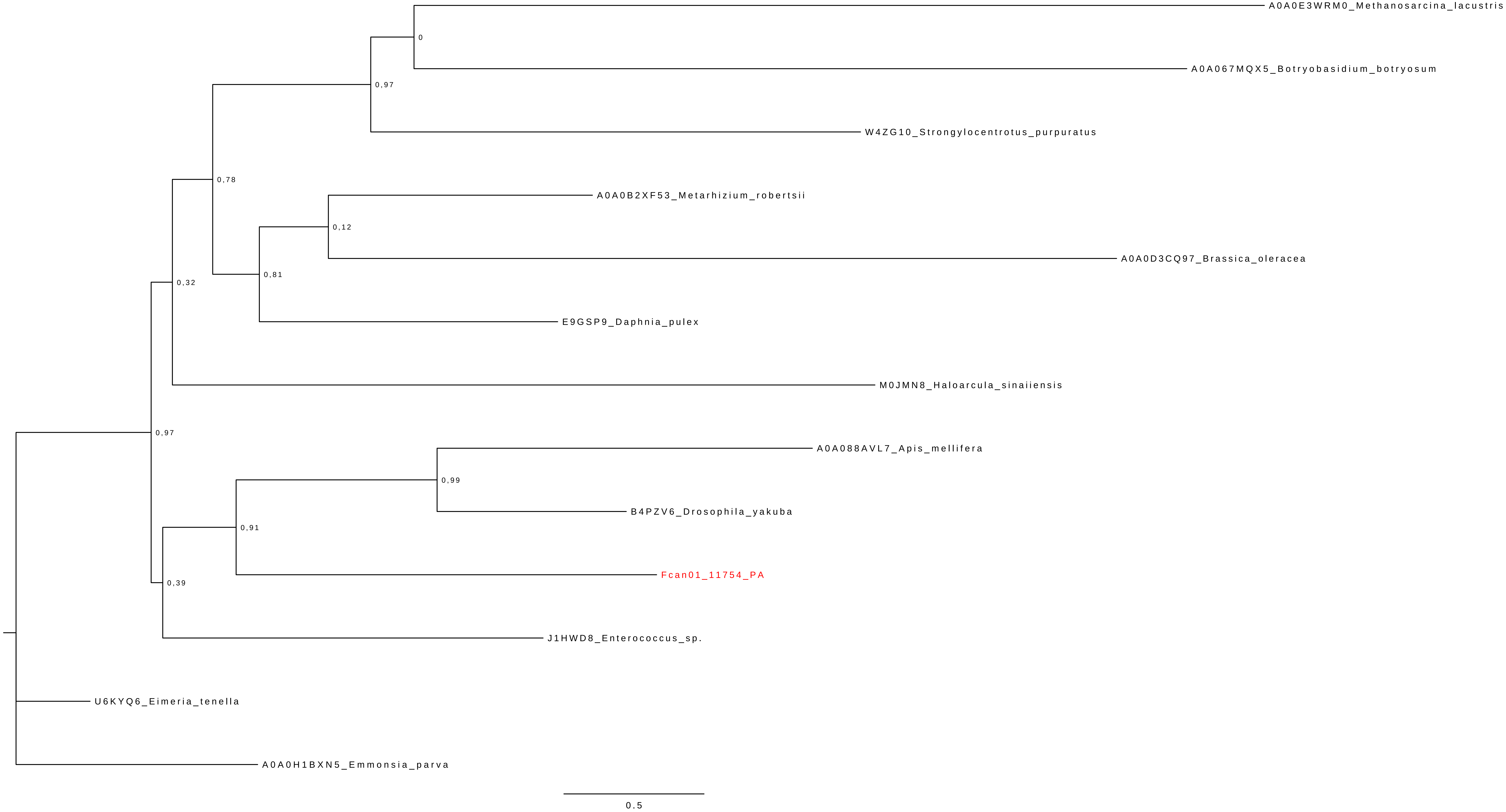
A0A0E3WRM0_Methanosarcina_lacustris
0
A0A067MQX5_Botryobasidium_botryosum
0,97
W4ZG10_Strongylocentrotus_purpuratus
0,78
A0A0B2XF53_Metarhizium_robertsii
0,12
A0A0D3CQ97_Brassica_oleracea
0,81
0,32
E9GSP9_Daphnia_pulex
M0JMN8_Haloarcula_sinaiiensis
0,97
A0A088AVL7_Apis_mellifera
0,99
B4PZV6_Drosophila_yakuba
0,91
Fcan01_11754_PA
0,39
J1HWD8_Enterococcus_sp.
U6KYQ6_Eimeria_tenella
A0A0H1BXN5_Emmonsia_parva
0.5

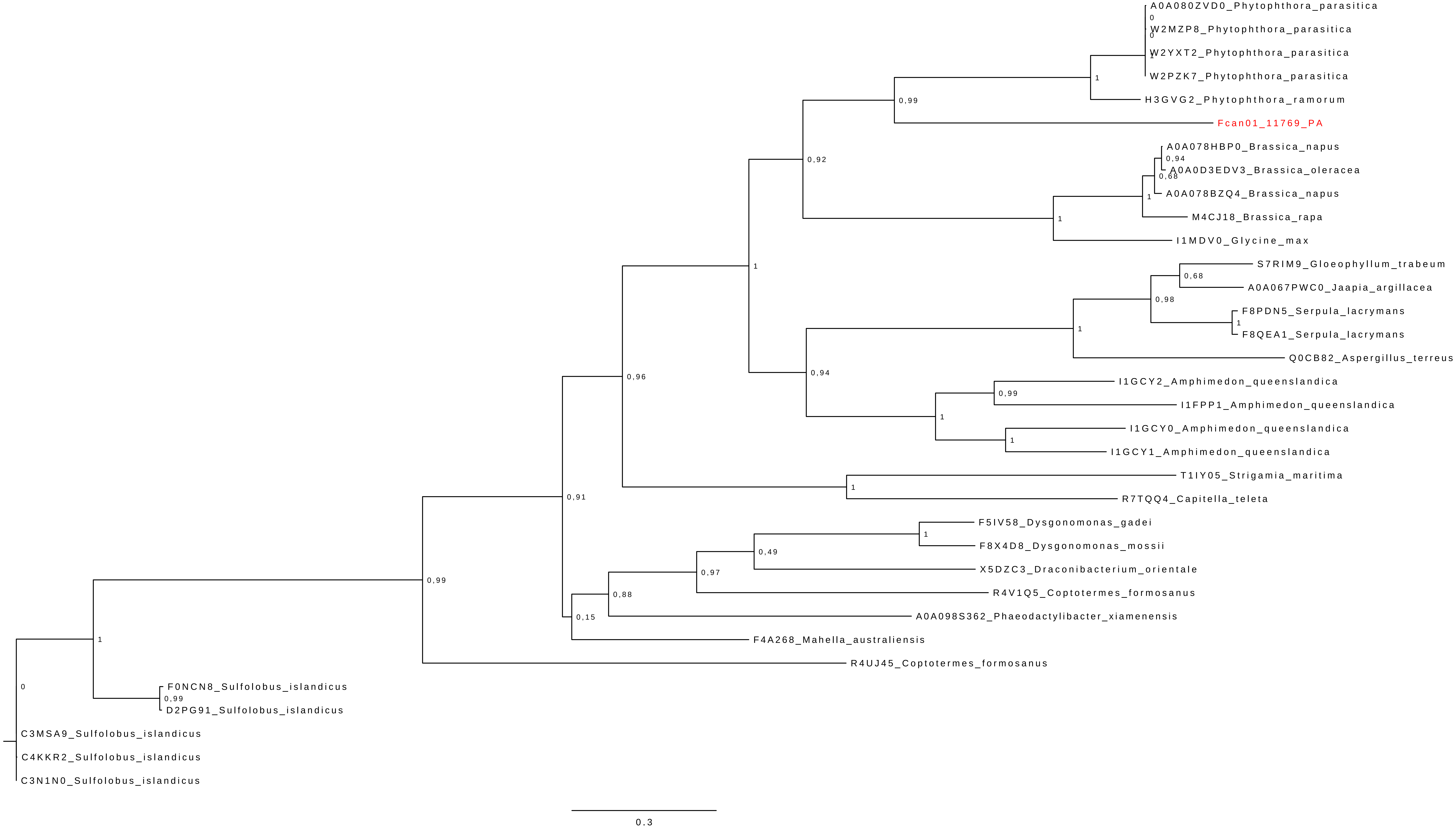
A0A080ZVD0_Phytophthora_parasitica
W2MZP8_Phytophthora_parasitica
W2YXT2_Phytophthora_parasitica
W2PZK7_Phytophthora_parasitica
H3GVG2_Phytophthora_ramorum
Fcan01_11769_PA
A0A078HBP0_Brassica_napus
A0A0D3EDV3_Brassica_oleracea
A0A078BZQ4_Brassica_napus
M4CJ18_Brassica_rapa
I1MDV0_Glycine_max
S7RIM9_Gloeophyllum_trabeum
A0A067PWC0_Jaapia_argillacea
F8PDN5_Serpula_lacrymans
F8QEA1_Serpula_lacrymans
Q0CB82_Aspergillus_terreus
I1GCY2_Amphimedon_queenslandica
I1FPP1_Amphimedon_queenslandica
I1GCY0_Amphimedon_queenslandica
I1GCY1_Amphimedon_queenslandica
T1IY05_Strigamia_maritima
R7TQQ4_Capitella_teleta
F5IV58_Dysgonomonas_gadei
F8X4D8_Dysgonomonas_mossii
X5DZC3_Draconibacterium_orientale
R4V1Q5_Coptotermes_formosanus
A0A098S362_Phaeodactylibacter_xiamenensis
F4A268_Mahella_australiensis
R4UJ45_Coptotermes_formosanus
F0NCN8_Sulfolobus_islandicus
D2PG91_Sulfolobus_islandicus
C3MSA9_Sulfolobus_islandicus
C4KKR2_Sulfolobus_islandicus
C3N1N0_Sulfolobus_islandicus
0,99
0,92
0,94
0,68
0,96
0,98
0,91
0,49
0,97
0,88
0,15
0
1
0.3

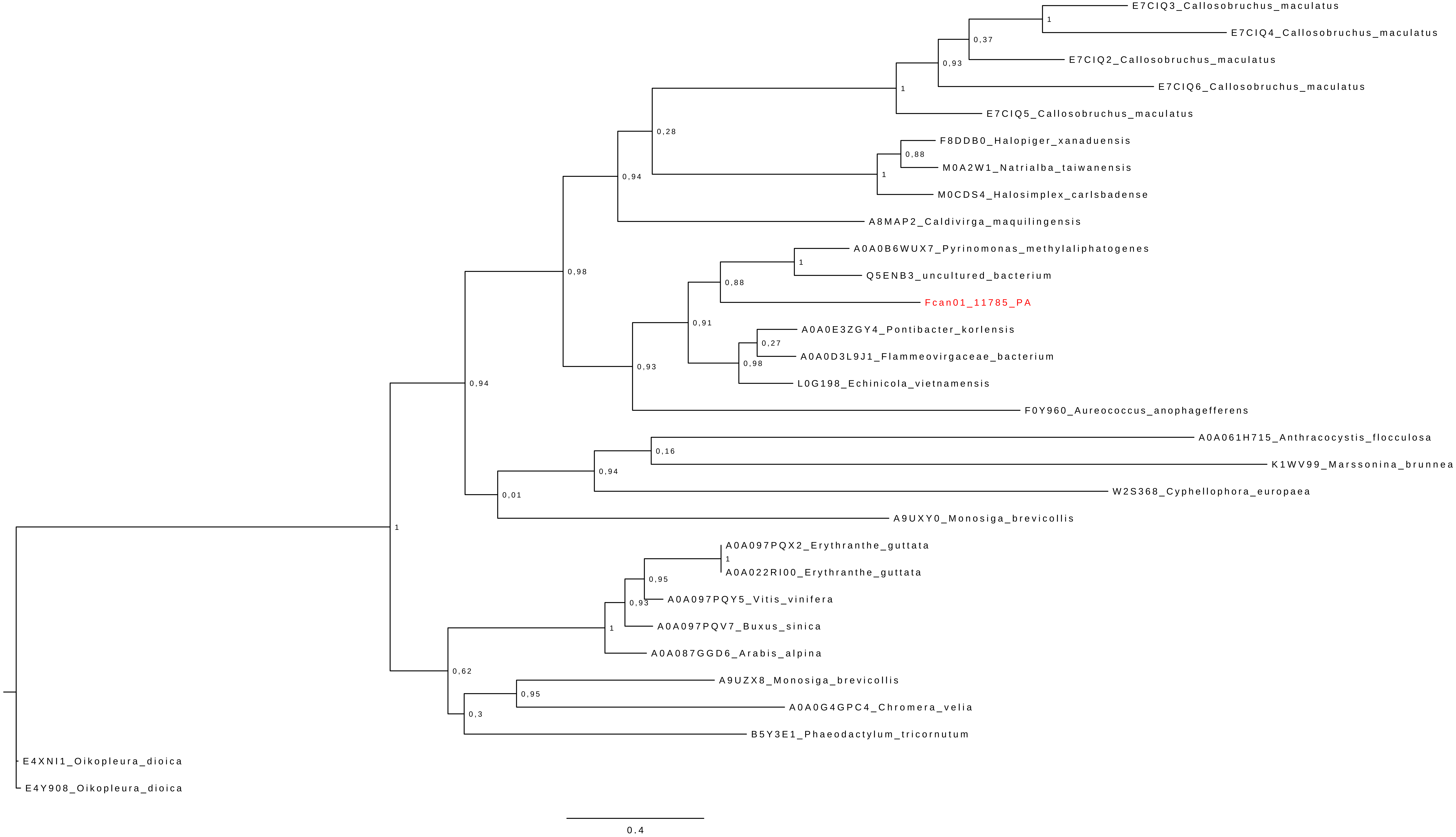
E7CIQ3_Callosobruchus_maculatus
1
E7CIQ4_Callosobruchus_maculatus
0,37
E7CIQ2_Callosobruchus_maculatus
0,93
E7CIQ6_Callosobruchus_maculatus
1
E7CIQ5_Callosobruchus_maculatus
0,28
F8DDB0_Halopiger_xanaduensis
0,88
M0A2W1_Natrialba_taiwanensis
1
M0CDS4_Halosimplex_carlsbadense
0,94
A8MAP2_Caldivirga_maquilingensis
A0A0B6WUX7_Pyrinomonas_methylaliphatogenes
1
Q5ENB3_uncultured_bacterium
0,98
0,88
Fcan01_11785_PA
0,91
A0A0E3ZGY4_Pontibacter_korlensis
0,27
A0A0D3L9J1_Flammeovirgaceae_bacterium
0,98
0,93
L0G198_Echinicola_vietnamensis
0,94
F0Y960_Aureococcus_anophagefferens
A0A061H715_Anthracocystis_flocculosa
0,16
K1WV99_Marssonina_brunnea
0,94
0,01
W2S368_Cyphellophora_europaea
A9UXY0_Monosiga_brevicollis
1
A0A097PQX2_Erythranthe_guttata
1
A0A022RI00_Erythranthe_guttata
0,95
A0A097PQY5_Vitis_vinifera
0,93
A0A097PQV7_Buxus_sinica
1
A0A087GGD6_Arabis_alpina
0,62
A9UZX8_Monosiga_brevicollis
0,95
A0A0G4GPC4_Chromera_velia
0,3
B5Y3E1_Phaeodactylum_tricornutum
E4XNI1_Oikopleura_dioica
E4Y908_Oikopleura_dioica
0.4

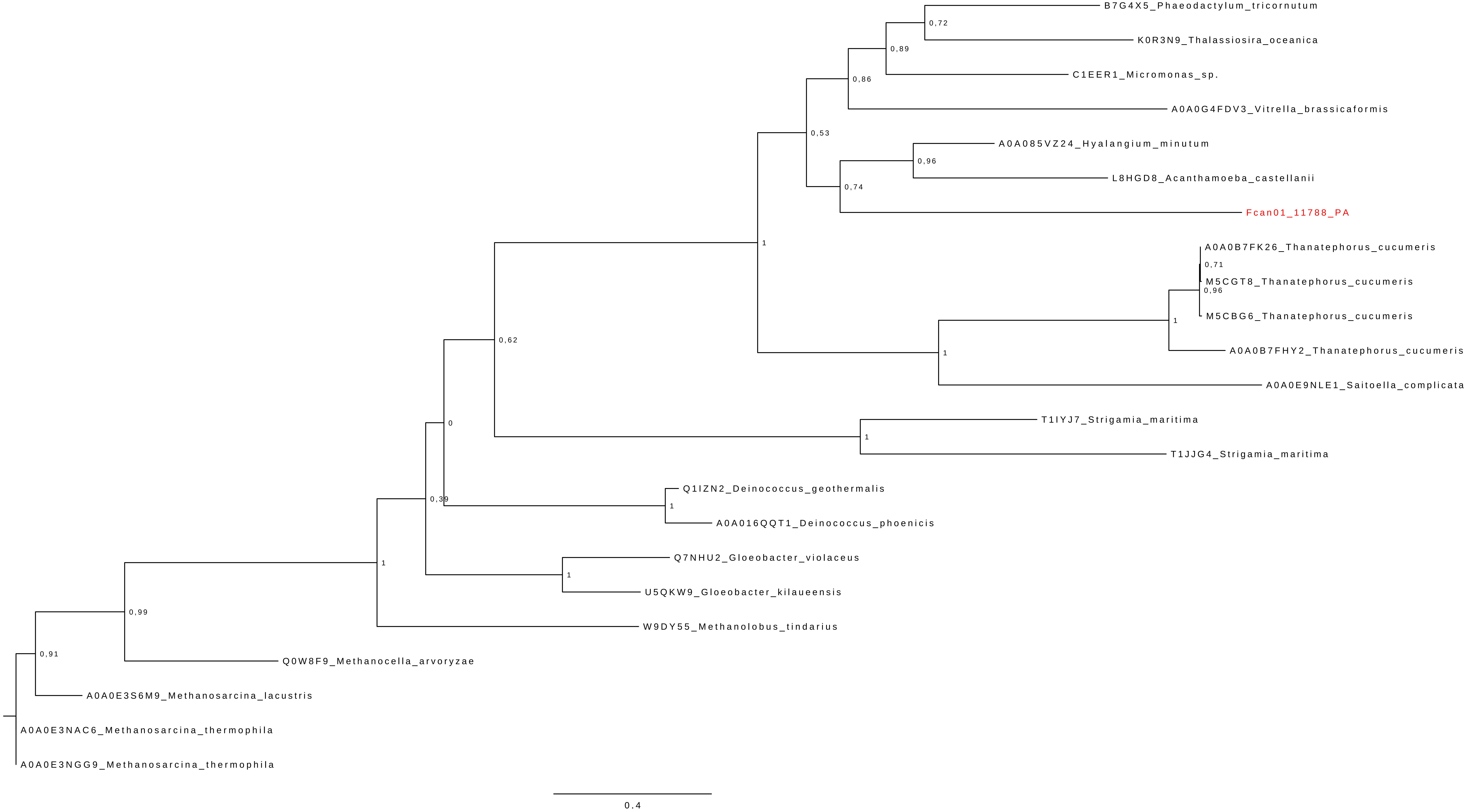

B7G4X5_Phaeodactylum_tricornutum
K0R3N9_Thalassiosira_oceanica
C1EER1_Micromonas_sp.
A0A0G4FDV3_Vitrella_brassicaformis
A0A085VZ24_Hyalangium_minutum
L8HGD8_Acanthamoeba_castellanii
Fcan01_11788_PA
A0A0B7FK26_Thanatephorus_cucumeris
M5CGT8_Thanatephorus_cucumeris
M5CBG6_Thanatephorus_cucumeris
A0A0B7FHY2_Thanatephorus_cucumeris
A0A0E9NLE1_Saitoella_complicata
T1IYJ7_Strigamia_maritima
T1JJG4_Strigamia_maritima
Q1IZN2_Deinococcus_geothermalis
A0A016QQT1_Deinococcus_phoenicis
Q7NHU2_Gloeobacter_violaceus
U5QKW9_Gloeobacter_kilaueensis
W9DY55_Methanolobus_tindarius
Q0W8F9_Methanocella_arvoryzae
A0A0E3S6M9_Methanosarcina_lacustris
A0A0E3NAC6_Methanosarcina_thermophila
A0A0E3NGG9_Methanosarcina_thermophila
0,72
0,89
0,86
0,53
0,96
0,74
1
0,71
0,96
1
1
0,62
0
1
0,39
1
1
1
0,99
0,91
0.4

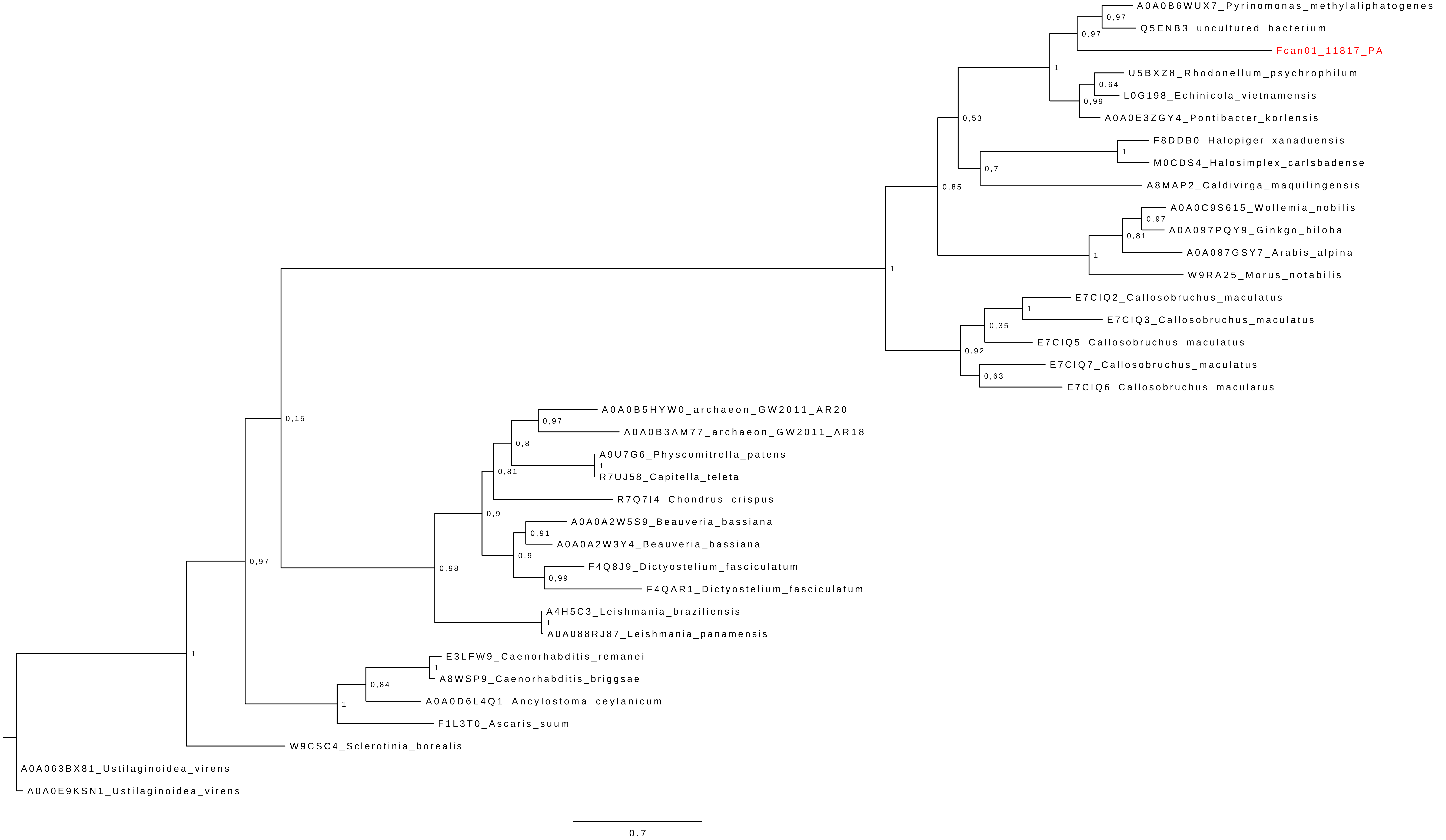
A0A0B6WUX7_Pyrinomonas_methylaliphatogenes
0,97
Q5ENB3_uncultured_bacterium
0,97
Fcan01_11817_PA
1
U5BXZ8_Rhodonellum_psychrophilum
0,64
L0G198_Echinicola_vietnamensis
0,99
A0A0E3ZGY4_Pontibacter_korlensis
0,53
F8DDB0_Halopiger_xanaduensis
1
M0CDS4_Halosimplex_carlsbadense
0,7
A8MAP2_Caldivirga_maquilingensis
0,85
A0A0C9S615_Wollemia_nobilis
0,97
A0A097PQY9_Ginkgo_biloba
0,81
A0A087GSY7_Arabis_alpina
1
W9RA25_Morus_notabilis
1
E7CIQ2_Callosobruchus_maculatus
1
E7CIQ3_Callosobruchus_maculatus
0,35
E7CIQ5_Callosobruchus_maculatus
0,92
E7CIQ7_Callosobruchus_maculatus
0,63
E7CIQ6_Callosobruchus_maculatus
A0A0B5HYW0_archaeon_GW2011_AR20
0,15
0,97
A0A0B3AM77_archaeon_GW2011_AR18
0,8
A9U7G6_Physcomitrella_patens
1
0,81
R7UJ58_Capitella_teleta
R7Q7I4_Chondrus_crispus
0,9
A0A0A2W5S9_Beauveria_bassiana
0,91
A0A0A2W3Y4_Beauveria_bassiana
0,9
0,97
F4Q8J9_Dictyostelium_fasciculatum
0,98
0,99
F4QAR1_Dictyostelium_fasciculatum
A4H5C3_Leishmania_braziliensis
1
A0A088RJ87_Leishmania_panamensis
1
E3LFW9_Caenorhabditis_remanei
1
A8WSP9_Caenorhabditis_briggsae
0,84
A0A0D6L4Q1_Ancylostoma_ceylanicum
1
F1L3T0_Ascaris_suum
W9CSC4_Sclerotinia_borealis
A0A063BX81_Ustilaginoidea_virens
A0A0E9KSN1_Ustilaginoidea_virens
0.7

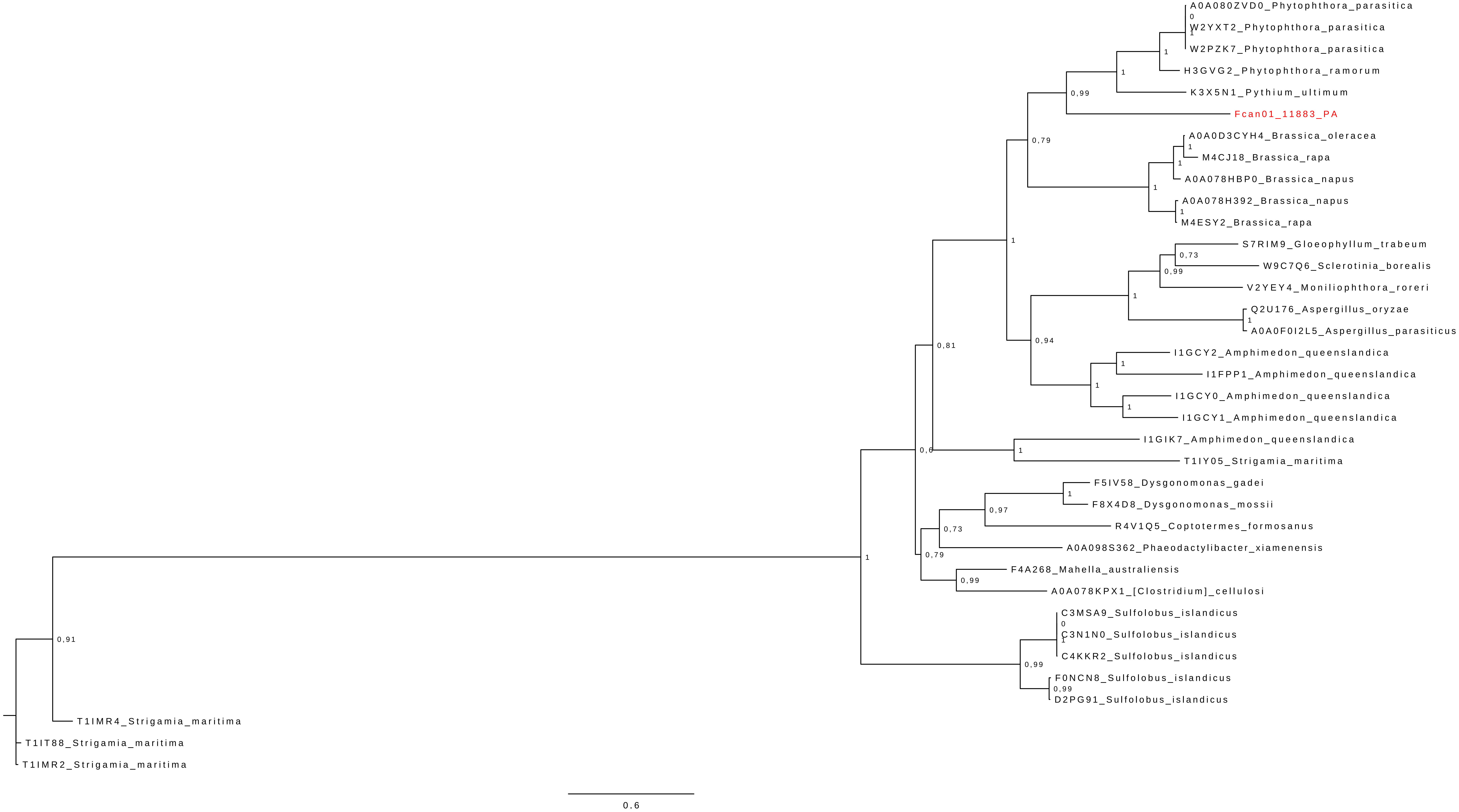
A0A080ZVD0_Phytophthora_parasitica
W2YXT2_Phytophthora_parasitica
W2PZK7_Phytophthora_parasitica
H3GVG2_Phytophthora_ramorum
K3X5N1_Pythium_ultimum
Fcan01_11883_PA
A0A0D3CYH4_Brassica_oleracea
M4CJ18_Brassica_rapa
A0A078HBP0_Brassica_napus
A0A078H392_Brassica_napus
M4ESY2_Brassica_rapa
S7RIM9_Gloeophyllum_trabeum
W9C7Q6_Sclerotinia_borealis
V2YEY4_Moniliophthora_roreri
Q2U176_Aspergillus_oryzae
A0A0F0I2L5_Aspergillus_parasiticus
I1GCY2_Amphimedon_queenslandica
I1FPP1_Amphimedon_queenslandica
I1GCY0_Amphimedon_queenslandica
I1GCY1_Amphimedon_queenslandica
I1GIK7_Amphimedon_queenslandica
T1IY05_Strigamia_maritima
F5IV58_Dysgonomonas_gadei
F8X4D8_Dysgonomonas_mossii
R4V1Q5_Coptotermes_formosanus
A0A098S362_Phaeodactylibacter_xiamenensis
F4A268_Mahella_australiensis
A0A078KPX1_[Clostridium]_cellulosi
C3MSA9_Sulfolobus_islandicus
C3N1N0_Sulfolobus_islandicus
C4KKR2_Sulfolobus_islandicus
F0NCN8_Sulfolobus_islandicus
D2PG91_Sulfolobus_islandicus
T1IMR4_Strigamia_maritima
T1IT88_Strigamia_maritima
T1IMR2_Strigamia_maritima
0.6

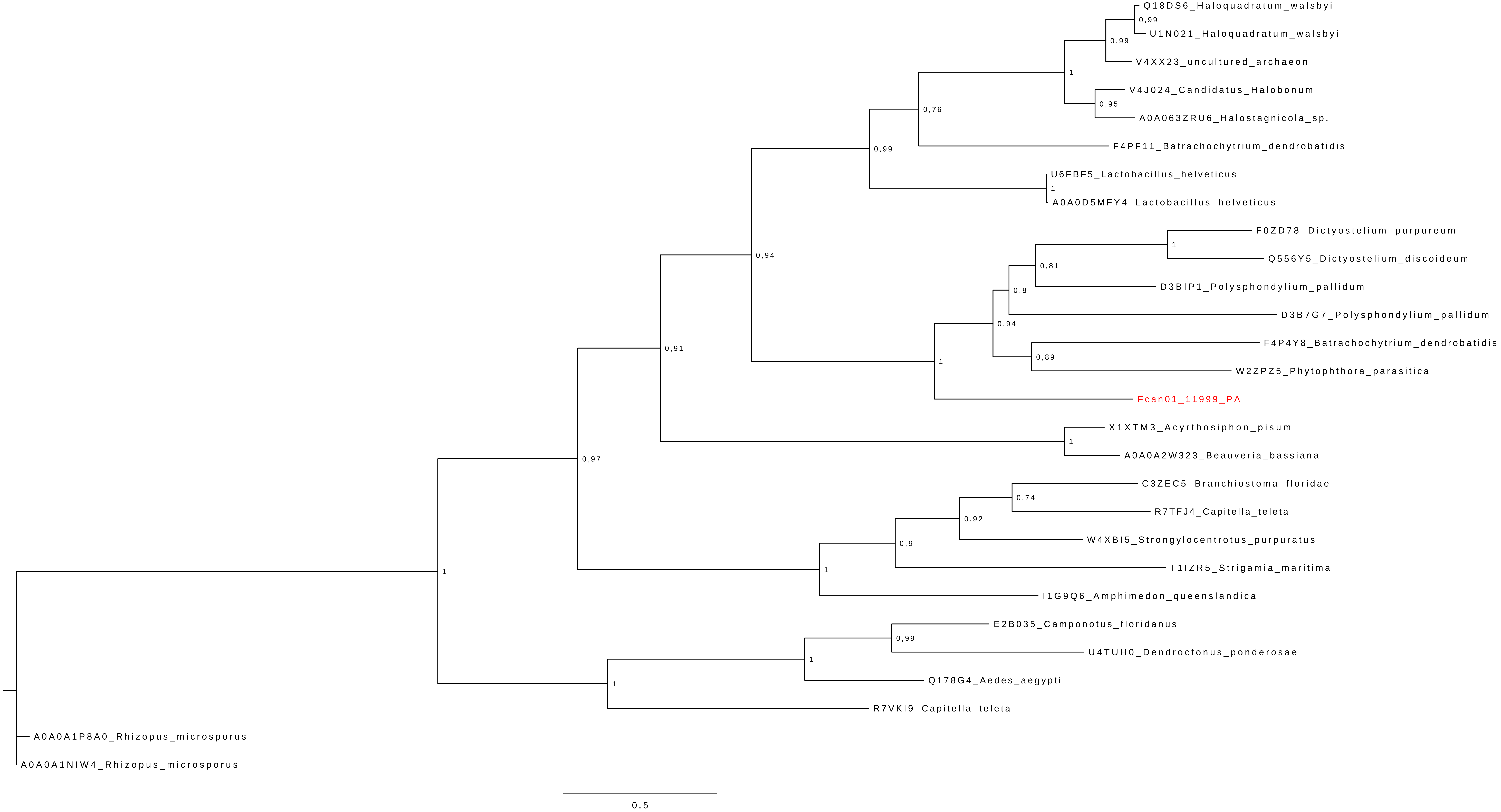
Q18DS6_Haloquadratum_walsbyi
0,99
U1N021_Haloquadratum_walsbyi
0,99
V4XX23_uncultured_archaeon
1
V4J024_Candidatus_Halobonum
0,95
A0A063ZRU6_Halostagnicola_sp.
0,76
F4PF11_Batrachochytrium_dendrobatidis
0,99
U6FBF5_Lactobacillus_helveticus
1
A0A0D5MFY4_Lactobacillus_helveticus
F0ZD78_Dictyostelium_purpureum
1
Q556Y5_Dictyostelium_discoideum
0,81
D3BIP1_Polysphondylium_pallidum
0,8
D3B7G7_Polysphondylium_pallidum
0,94
F4P4Y8_Batrachochytrium_dendrobatidis
0,89
W2ZPZ5_Phytophthora_parasitica
1
Fcan01_11999_PA
0,94
0,91
X1XTM3_Acyrthosiphon_pisum
1
A0A0A2W323_Beauveria_bassiana
0,97
C3ZEC5_Branchiostoma_floridae
0,74
R7TFJ4_Capitella_teleta
0,92
W4XBI5_Strongylocentrotus_purpuratus
0,9
T1IZR5_Strigamia_maritima
1
I1G9Q6_Amphimedon_queenslandica
1
E2B035_Camponotus_floridanus
0,99
U4TUH0_Dendroctonus_ponderosae
1
Q178G4_Aedes_aegypti
1
R7VKI9_Capitella_teleta
A0A0A1P8A0_Rhizopus_microsporus
A0A0A1NIW4_Rhizopus_microsporus
0.5

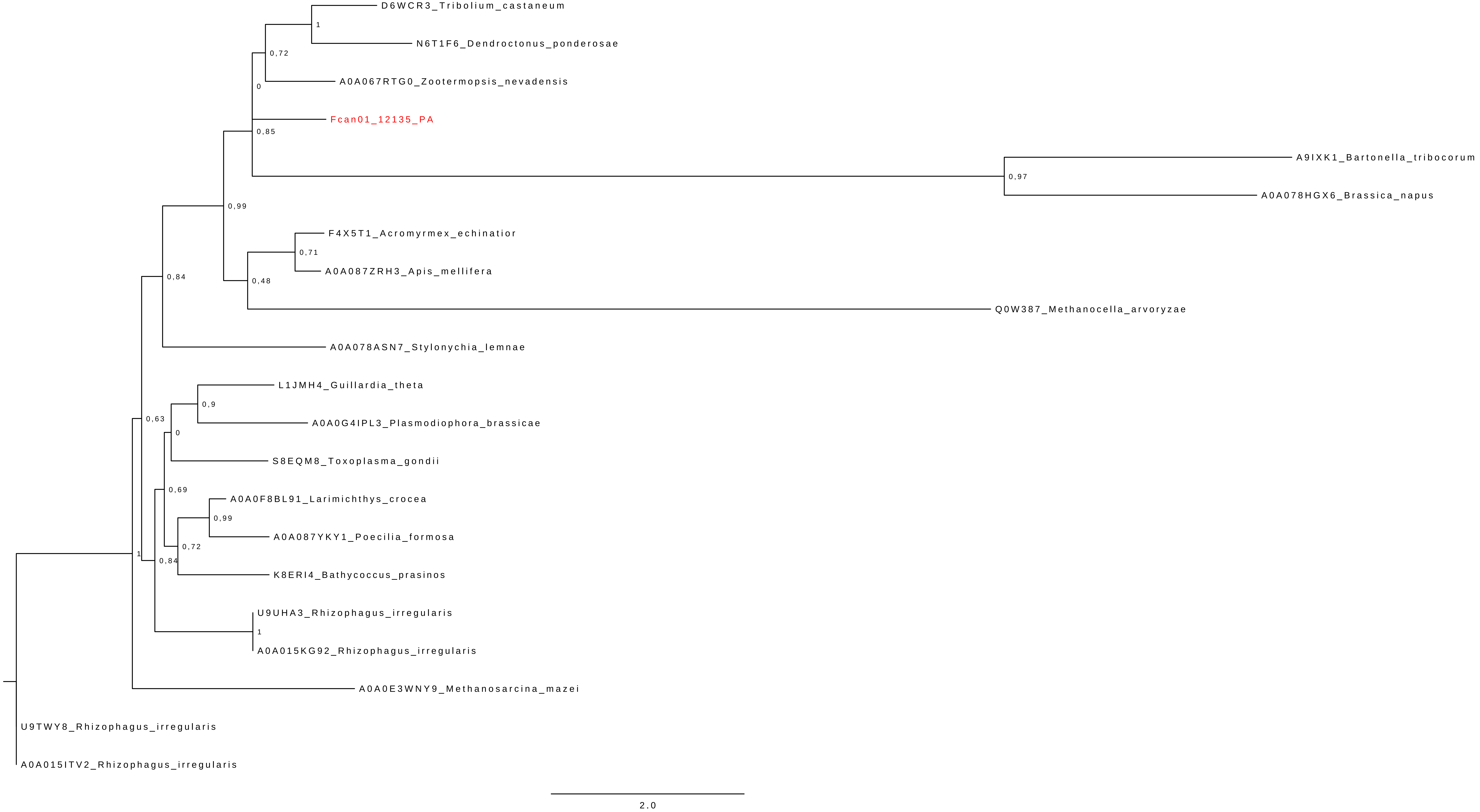
D6WCR3_Tribolium_castaneum
1
N6T1F6_Dendroctonus_ponderosae
0,72
A0A067RTG0_Zootermopsis_nevadensis
0
Fcan01_12135_PA
0,85
A9IXK1_Bartonella_tribocorum
0,97
A0A078HGX6_Brassica_napus
0,99
F4X5T1_Acromyrmex_echinatior
0,71
A0A087ZRH3_Apis_mellifera
0,84
0,48
Q0W387_Methanocella_arvoryzae
A0A078ASN7_Stylonychia_lemnae
L1JMH4_Guillardia_theta
0,9
0,63
A0A0G4IPL3_Plasmodiophora_brassicae
0
S8EQM8_Toxoplasma_gondii
0,69
A0A0F8BL91_Larimichthys_crocea
0,99
A0A087YKY1_Poecilia_formosa
0,72
1
0,84
K8ERI4_Bathycoccus_prasinos
U9UHA3_Rhizophagus_irregularis
1
A0A015KG92_Rhizophagus_irregularis
A0A0E3WNY9_Methanosarcina_mazei
U9TWY8_Rhizophagus_irregularis
A0A015ITV2_Rhizophagus_irregularis
2.0

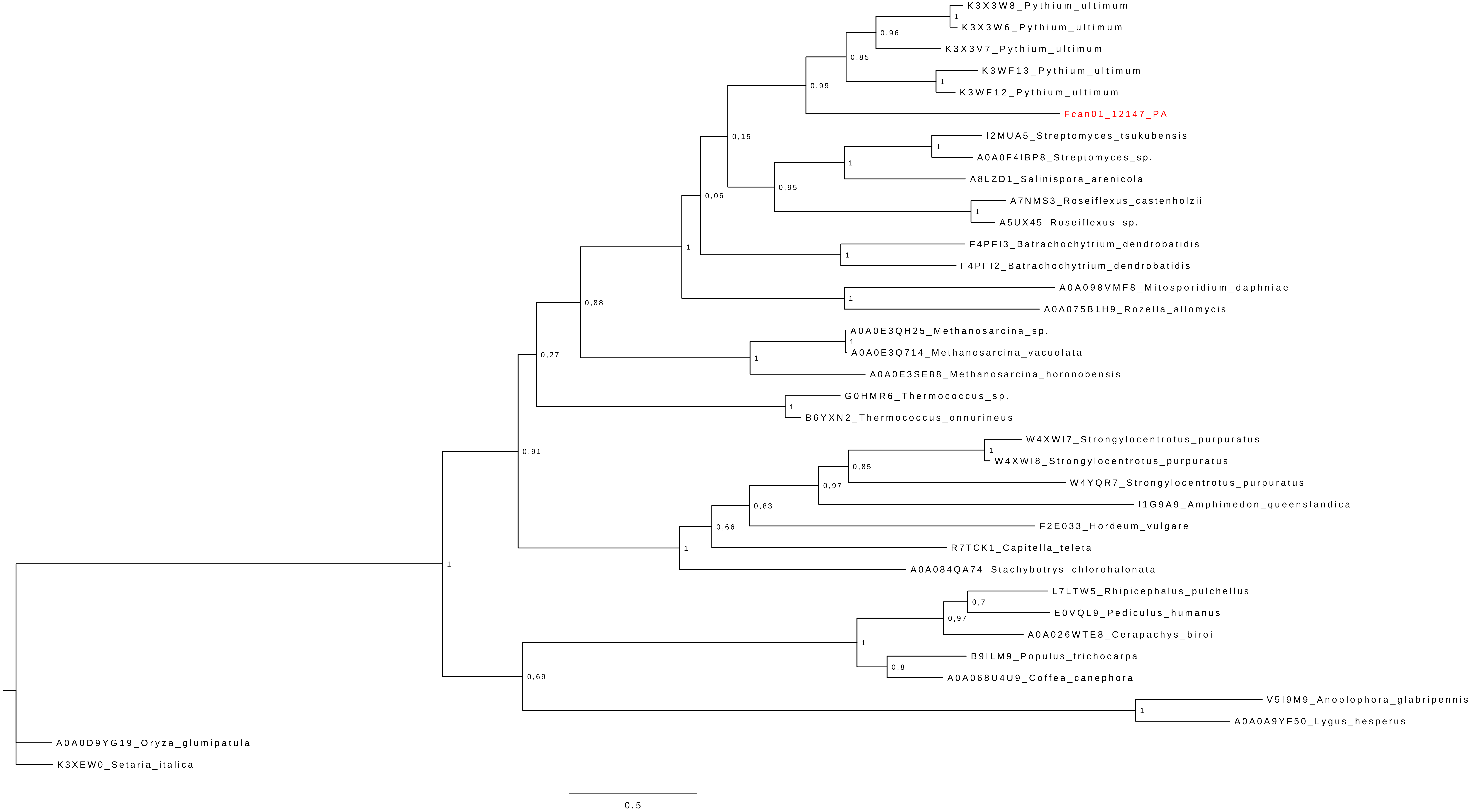
K3X3W8_Pythium_ultimum
K3X3W6_Pythium_ultimum
K3X3V7_Pythium_ultimum
K3WF13_Pythium_ultimum
K3WF12_Pythium_ultimum
Fcan01_12147_PA
I2MUA5_Streptomyces_tsukubensis
A0A0F4IBP8_Streptomyces_sp.
A8LZD1_Salinispora_arenicola
A7NMS3_Roseiflexus_castenholzii
A5UX45_Roseiflexus_sp.
F4PFI3_Batrachochytrium_dendrobatidis
F4PFI2_Batrachochytrium_dendrobatidis
A0A098VMF8_Mitosporidium_daphniae
A0A075B1H9_Rozella_allomycis
A0A0E3QH25_Methanosarcina_sp.
A0A0E3Q714_Methanosarcina_vacuolata
A0A0E3SE88_Methanosarcina_horonobensis
G0HMR6_Thermococcus_sp.
B6YXN2_Thermococcus_onnurineus
W4XWI7_Strongylocentrotus_purpuratus
W4XWI8_Strongylocentrotus_purpuratus
W4YQR7_Strongylocentrotus_purpuratus
I1G9A9_Amphimedon_queenslandica
F2E033_Hordeum_vulgare
R7TCK1_Capitella_teleta
A0A084QA74_Stachybotrys_chlorohalonata
L7LTW5_Rhipicephalus_pulchellus
E0VQL9_Pediculus_humanus
A0A026WTE8_Cerapachys_biroi
B9ILM9_Populus_trichocarpa
A0A068U4U9_Coffea_canephora
V5I9M9_Anoplophora_glabripennis
A0A0A9YF50_Lygus_hesperus
A0A0D9YG19_Oryza_glumipatula
K3XEW0_Setaria_italica
1
0,96
0,85
1
0,99
0,15
1
1
0,95
1
0,06
1
1
1
0,88
1
1
0,27
1
1
0,85
0,97
0,83
0,66
1
0,91
1
0,7
0,97
1
0,8
0,69
1
0.5

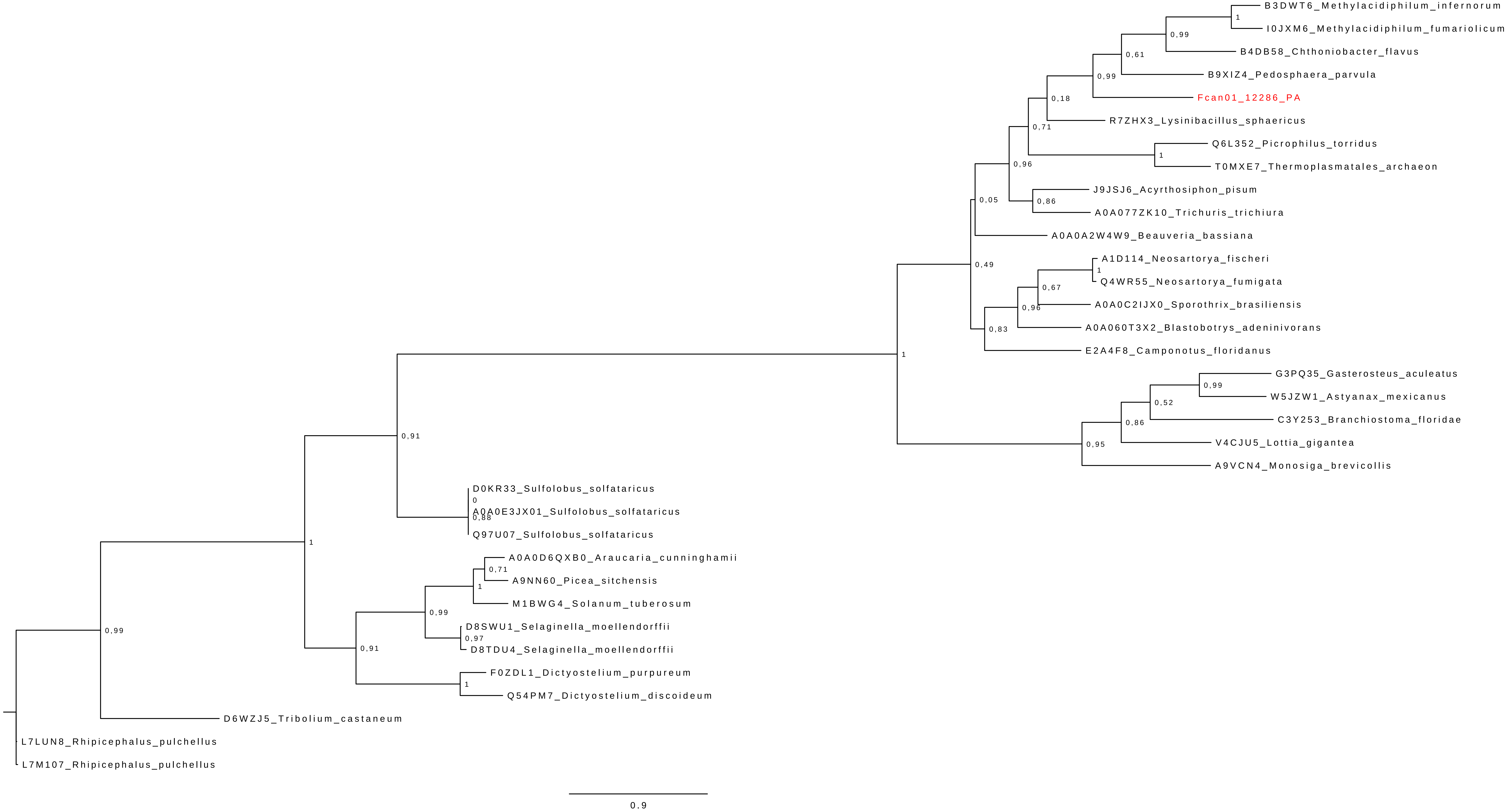
B3DWT6_Methylacidiphilum_infernorum
I0JXM6_Methylacidiphilum_fumariolicum
B4DB58_Chthoniobacter_flavus
B9XIZ4_Pedosphaera_parvula
Fcan01_12286_PA
R7ZHX3_Lysinibacillus_sphaericus
Q6L352_Picrophilus_torridus
T0MXE7_Thermoplasmatales_archaeon
J9JSJ6_Acyrthosiphon_pisum
A0A077ZK10_Trichuris_trichiura
A0A0A2W4W9_Beauveria_bassiana
A1D114_Neosartorya_fischeri
Q4WR55_Neosartorya_fumigata
A0A0C2IJX0_Sporothrix_brasiliensis
A0A060T3X2_Blastobotrys_adeninivorans
E2A4F8_Camponotus_floridanus
G3PQ35_Gasterosteus_aculeatus
W5JZW1_Astyanax_mexicanus
C3Y253_Branchiostoma_floridae
V4CJU5_Lottia_gigantea
A9VCN4_Monosiga_brevicollis
D0KR33_Sulfolobus_solfataricus
A0A0E3JX01_Sulfolobus_solfataricus
Q97U07_Sulfolobus_solfataricus
A0A0D6QXB0_Araucaria_cunninghamii
A9NN60_Picea_sitchensis
M1BWG4_Solanum_tuberosum
D8SWU1_Selaginella_moellendorffii
D8TDU4_Selaginella_moellendorffii
F0ZDL1_Dictyostelium_purpureum
Q54PM7_Dictyostelium_discoideum
D6WZJ5_Tribolium_castaneum
L7LUN8_Rhipicephalus_pulchellus
L7M107_Rhipicephalus_pulchellus
1
0,99
0,61
0,99
0,18
0,71
1
0,96
0,86
0,05
0,49
1
0,67
0,96
0,83
1
0,99
0,52
0,86
0,95
0,91
0
0,88
1
0,71
1
0,99
0,97
0,91
1
0,99
0.9

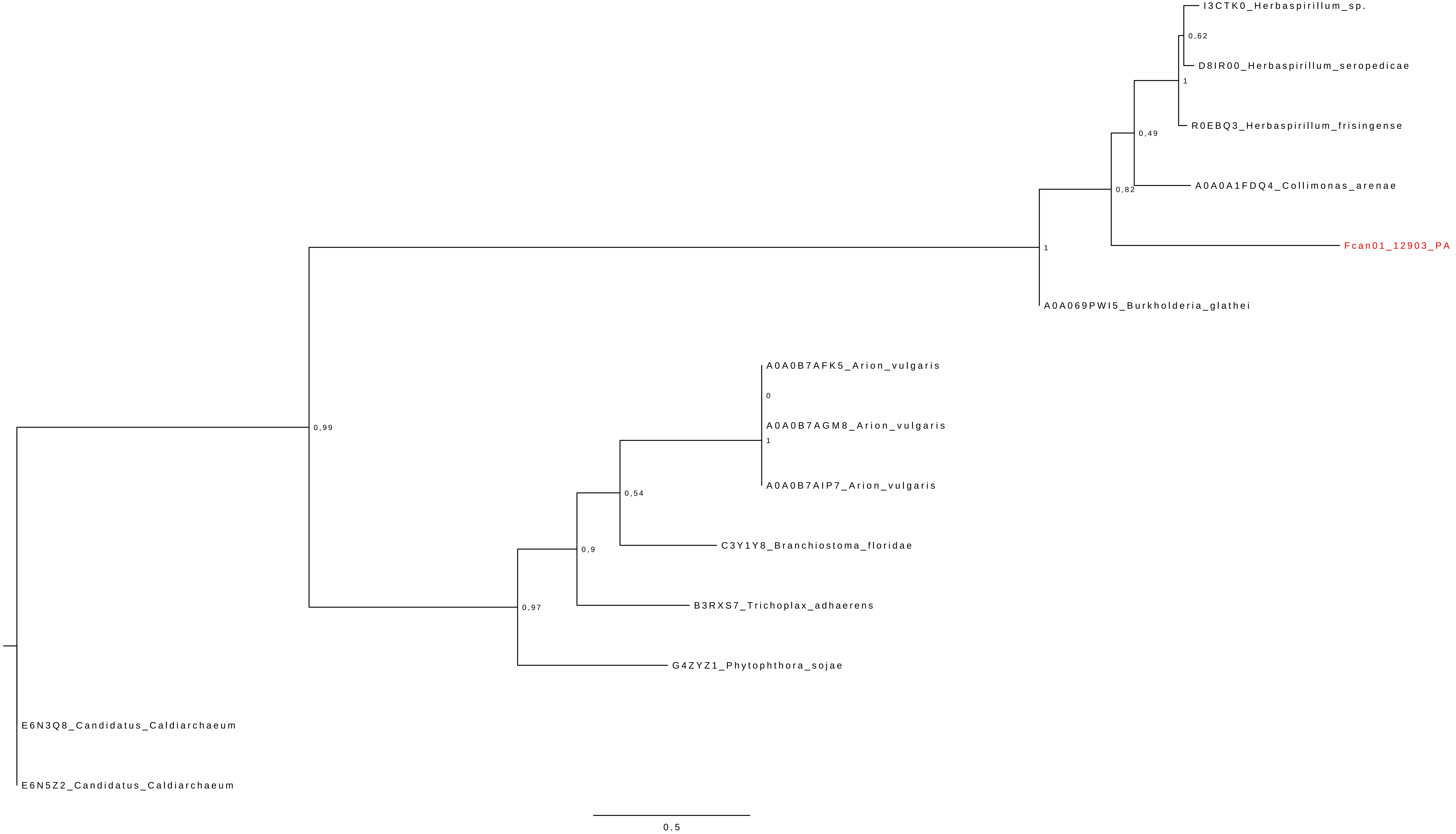
I3CTK0_Herbaspirillum_sp.
0,62
D8IR00_Herbaspirillum_seropedicae
1
R0EBQ3_Herbaspirillum_frisingense
0,49
A0A0A1FDQ4_Collimonas_arenae
0,82
Fcan01_12903_PA
1
A0A069PWI5_Burkholderia_glathei
A0A0B7AFK5_Arion_vulgaris
0
A0A0B7AGM8_Arion_vulgaris
1
A0A0B7AIP7_Arion_vulgaris
0,99
0,54
C3Y1Y8_Branchiostoma_floridae
0,9
B3RXS7_Trichoplax_adhaerens
0,97
G4ZYZ1_Phytophthora_sojae
E6N3Q8_Candidatus_Caldiarchaeum
E6N5Z2_Candidatus_Caldiarchaeum
0.5

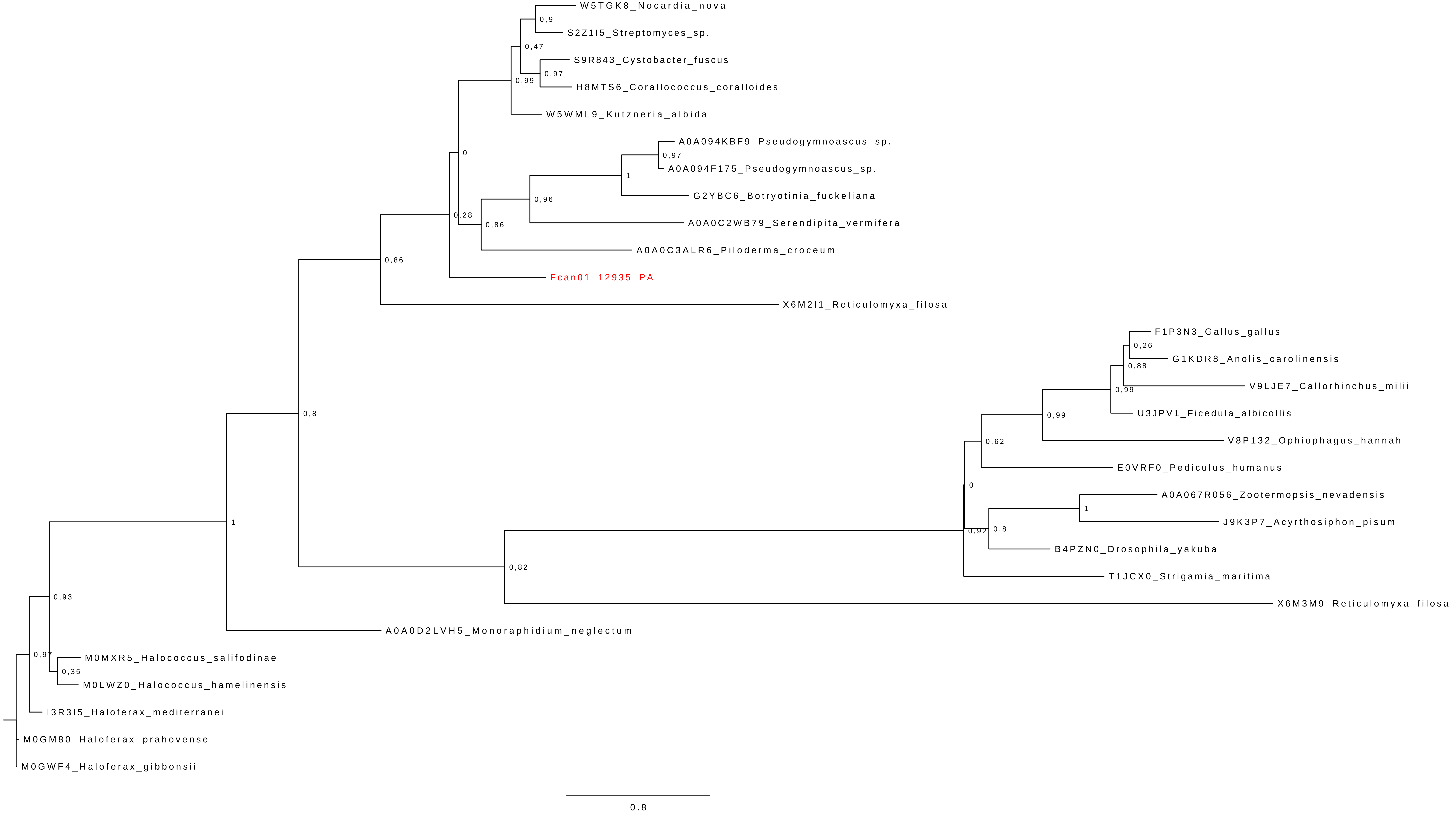
W5TGK8_Nocardia_nova
0,9
S2Z1I5_Streptomyces_sp.
0,47
S9R843_Cystobacter_fuscus
0,97
0,99
H8MTS6_Corallococcus_coralloides
W5WML9_Kutzneria_albida
A0A094KBF9_Pseudogymnoascus_sp.
0
0,97
A0A094F175_Pseudogymnoascus_sp.
1
G2YBC6_Botryotinia_fuckeliana
0,96
0,28
A0A0C2WB79_Serendipita_vermifera
0,86
A0A0C3ALR6_Piloderma_croceum
0,86
Fcan01_12935_PA
X6M2I1_Reticulomyxa_filosa
F1P3N3_Gallus_gallus
0,26
G1KDR8_Anolis_carolinensis
0,88
V9LJE7_Callorhinchus_milii
0,99
0,8
U3JPV1_Ficedula_albicollis
0,99
V8P132_Ophiophagus_hannah
0,62
E0VRF0_Pediculus_humanus
0
A0A067R056_Zootermopsis_nevadensis
1
1
J9K3P7_Acyrthosiphon_pisum
0,92
0,8
B4PZN0_Drosophila_yakuba
0,82
T1JCX0_Strigamia_maritima
X6M3M9_Reticulomyxa_filosa
0,93
A0A0D2LVH5_Monoraphidium_neglectum
0,97
M0MXR5_Halococcus_salifodinae
0,35
M0LWZ0_Halococcus_hamelinensis
I3R3I5_Haloferax_mediterranei
M0GM80_Haloferax_prahovense
M0GWF4_Haloferax_gibbonsii
0.8

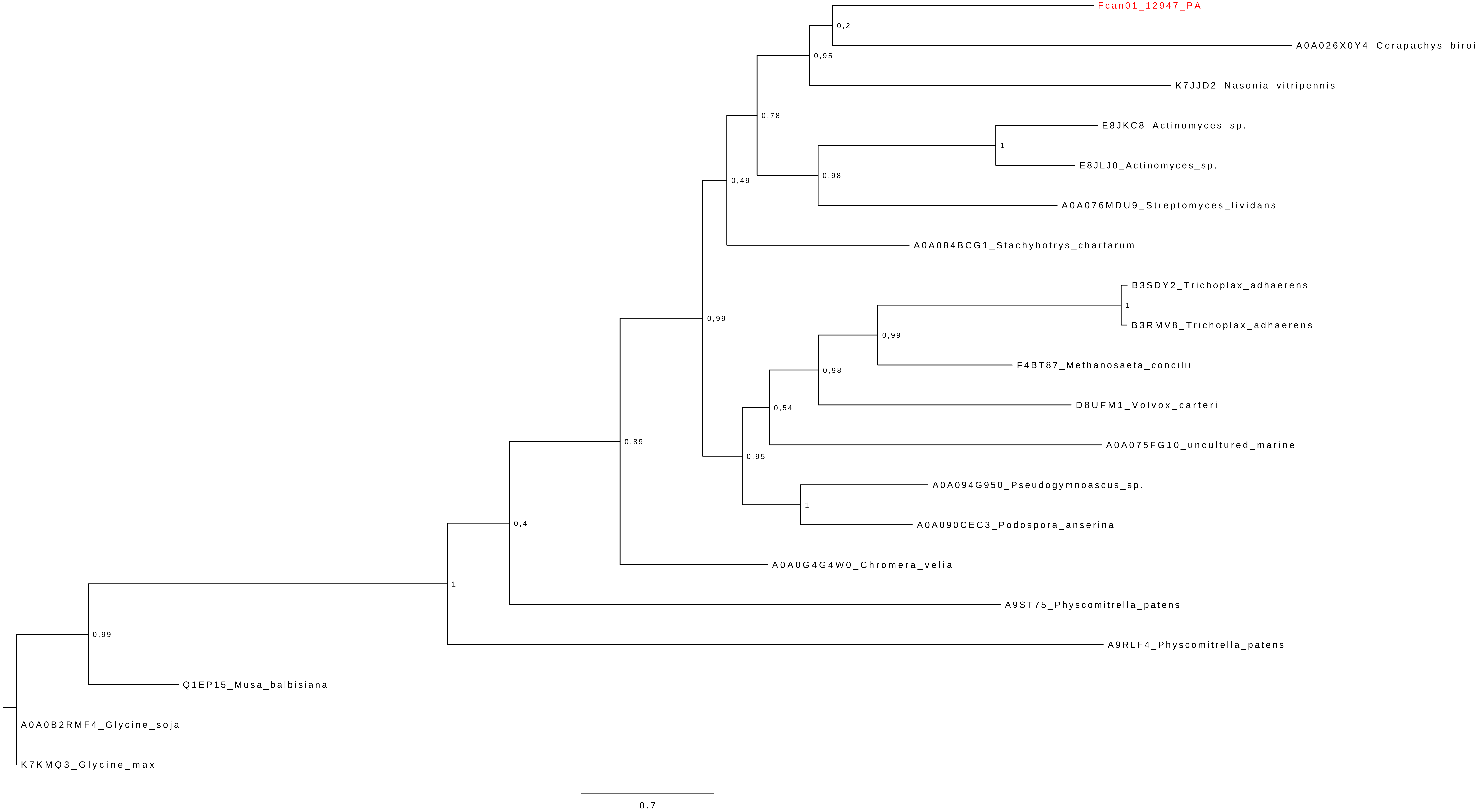
Fcan01_12947_PA
A0A026X0Y4_Cerapachys_biroi
K7JJD2_Nasonia_vitripennis
E8JKC8_Actinomyces_sp.
E8JLJ0_Actinomyces_sp.
A0A076MDU9_Streptomyces_lividans
A0A084BCG1_Stachybotrys_chartarum
B3SDY2_Trichoplax_adhaerens
B3RMV8_Trichoplax_adhaerens
F4BT87_Methanosaeta_concilii
D8UFM1_Volvox_carteri
A0A075FG10_uncultured_marine
A0A094G950_Pseudogymnoascus_sp.
A0A090CEC3_Podospora_anserina
A0A0G4G4W0_Chromera_velia
A9ST75_Physcomitrella_patens
A9RLF4_Physcomitrella_patens
Q1EP15_Musa_balbisiana
A0A0B2RMF4_Glycine_soja
K7KMQ3_Glycine_max
0,2
0,95
0,78
1
0,98
0,49
1
0,99
0,99
0,98
0,54
0,95
1
0,89
0,4
1
0,99
0.7

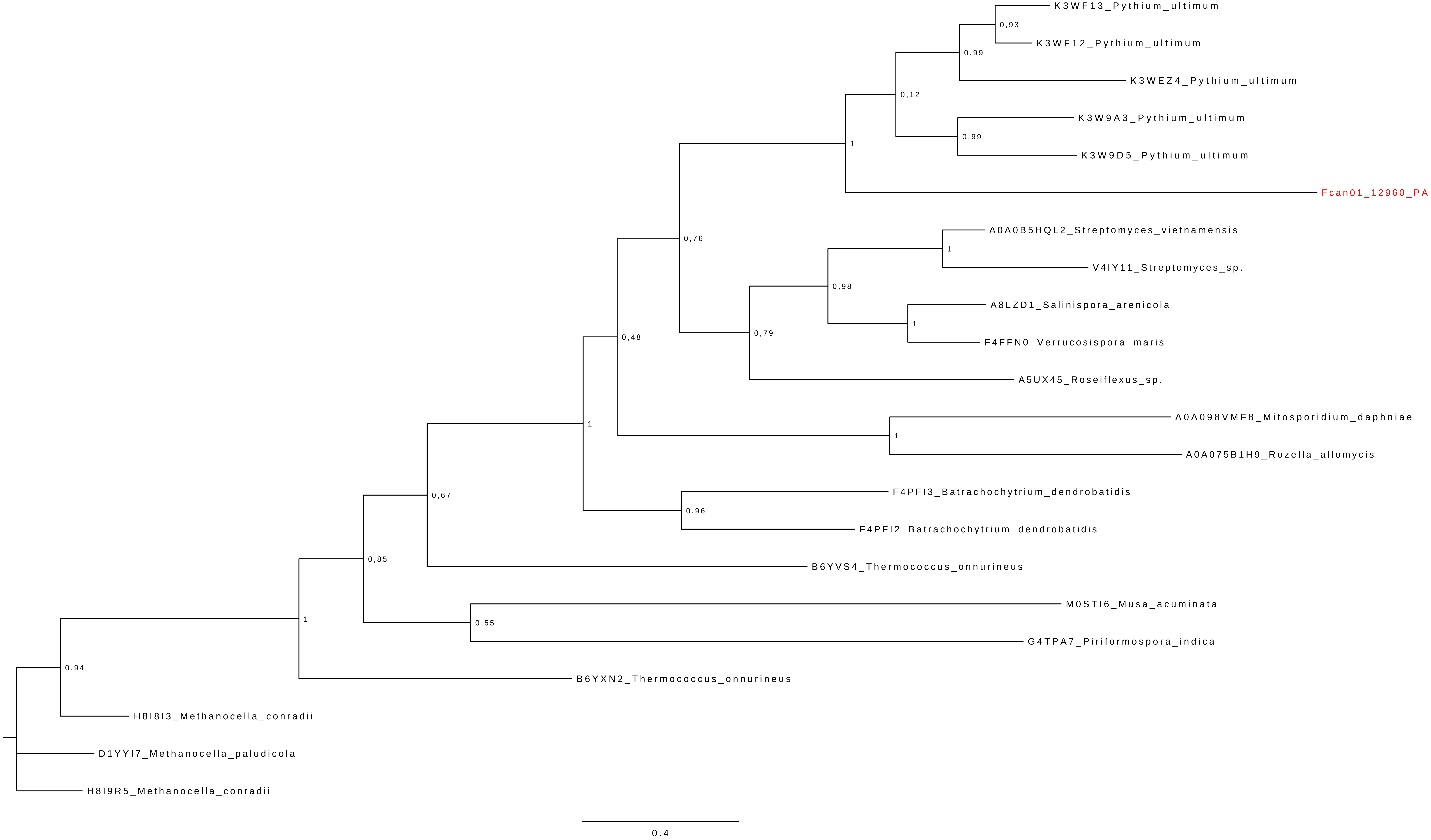
K3WF13_Pythium_ultimum
0,93
K3WF12_Pythium_ultimum
0,99
K3WEZ4_Pythium_ultimum
0,12
K3W9A3_Pythium_ultimum
0,99
K3W9D5_Pythium_ultimum
1
Fcan01_12960_PA
A0A0B5HQL2_Streptomyces_vietnamensis
0,76
1
V4IY11_Streptomyces_sp.
0,98
A8LZD1_Salinispora_arenicola
1
0,48
0,79
F4FFN0_Verrucosispora_maris
A5UX45_Roseiflexus_sp.
A0A098VMF8_Mitosporidium_daphniae
1
1
A0A075B1H9_Rozella_allomycis
F4PFI3_Batrachochytrium_dendrobatidis
0,67
0,96
F4PFI2_Batrachochytrium_dendrobatidis
0,85
B6YVS4_Thermococcus_onnurineus
M0STI6_Musa_acuminata
0,55
1
G4TPA7_Piriformospora_indica
B6YXN2_Thermococcus_onnurineus
0,94
H8I8I3_Methanocella_conradii
D1YYI7_Methanocella_paludicola
H8I9R5_Methanocella_conradii
0.4

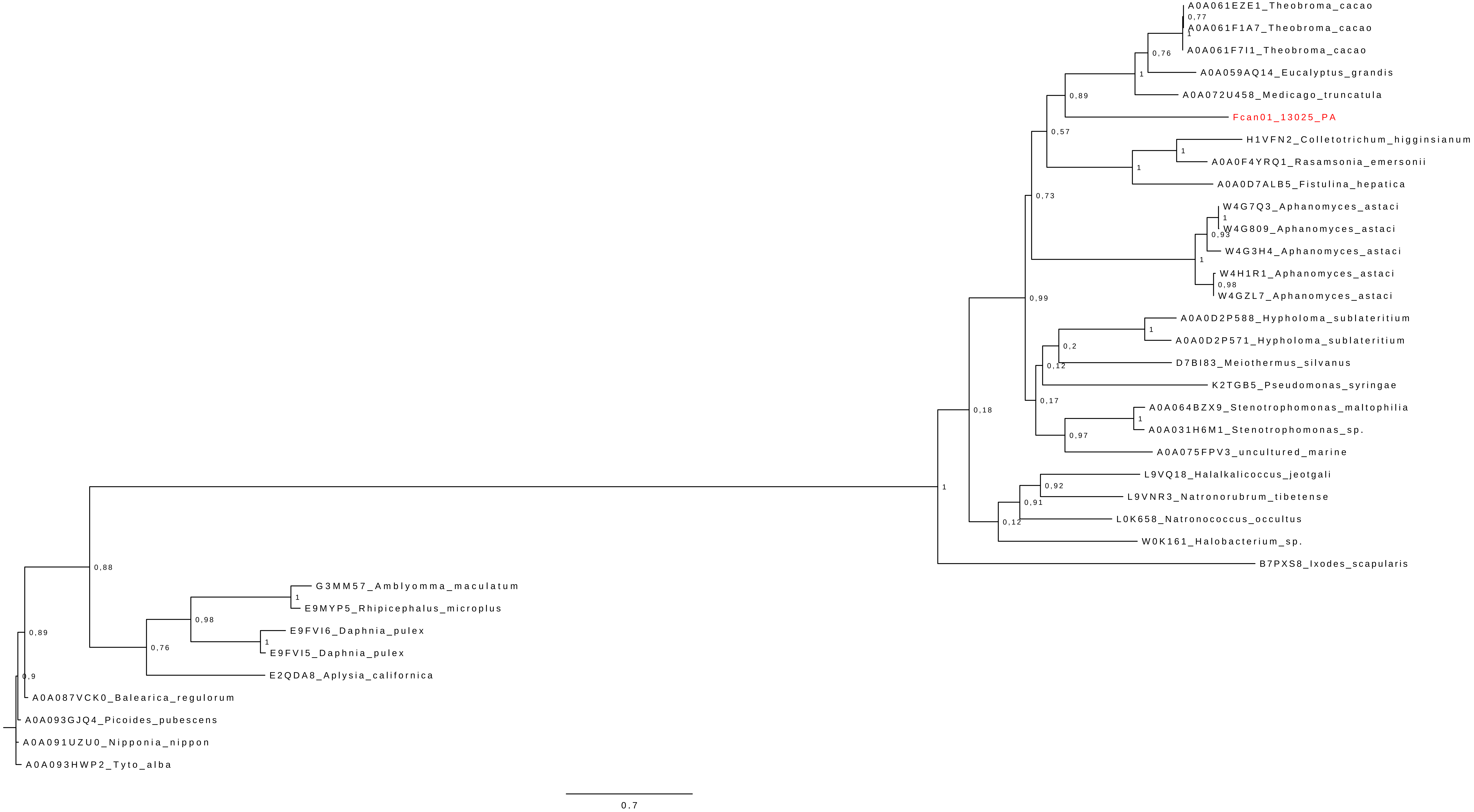

A0A061EZE1_Theobroma_cacao
0,77
A0A061F1A7_Theobroma_cacao
1
A0A061F7I1_Theobroma_cacao
0,76
1
A0A059AQ14_Eucalyptus_grandis
0,89
A0A072U458_Medicago_truncatula
Fcan01_13025_PA
0,57
H1VFN2_Colletotrichum_higginsianum
1
A0A0F4YRQ1_Rasamsonia_emersonii
1
A0A0D7ALB5_Fistulina_hepatica
0,73
W4G7Q3_Aphanomyces_astaci
1
W4G809_Aphanomyces_astaci
0,93
W4G3H4_Aphanomyces_astaci
1
W4H1R1_Aphanomyces_astaci
0,98
W4GZL7_Aphanomyces_astaci
0,99
A0A0D2P588_Hypholoma_sublateritium
1
A0A0D2P571_Hypholoma_sublateritium
0,2
D7BI83_Meiothermus_silvanus
0,12
K2TGB5_Pseudomonas_syringae
0,17
A0A064BZX9_Stenotrophomonas_maltophilia
1
A0A031H6M1_Stenotrophomonas_sp.
0,18
0,97
A0A075FPV3_uncultured_marine
L9VQ18_Halalkalicoccus_jeotgali
1
0,92
L9VNR3_Natronorubrum_tibetense
0,91
L0K658_Natronococcus_occultus
0,12
W0K161_Halobacterium_sp.
B7PXS8_Ixodes_scapularis
0,88
G3MM57_Amblyomma_maculatum
1
E9MYP5_Rhipicephalus_microplus
0,98
E9FVI6_Daphnia_pulex
1
E9FVI5_Daphnia_pulex
0,89
0,76
E2QDA8_Aplysia_californica
0,9
A0A087VCK0_Balearica_regulorum
A0A093GJQ4_Picoides_pubescens
A0A091UZU0_Nipponia_nippon
A0A093HWP2_Tyto_alba
0.7

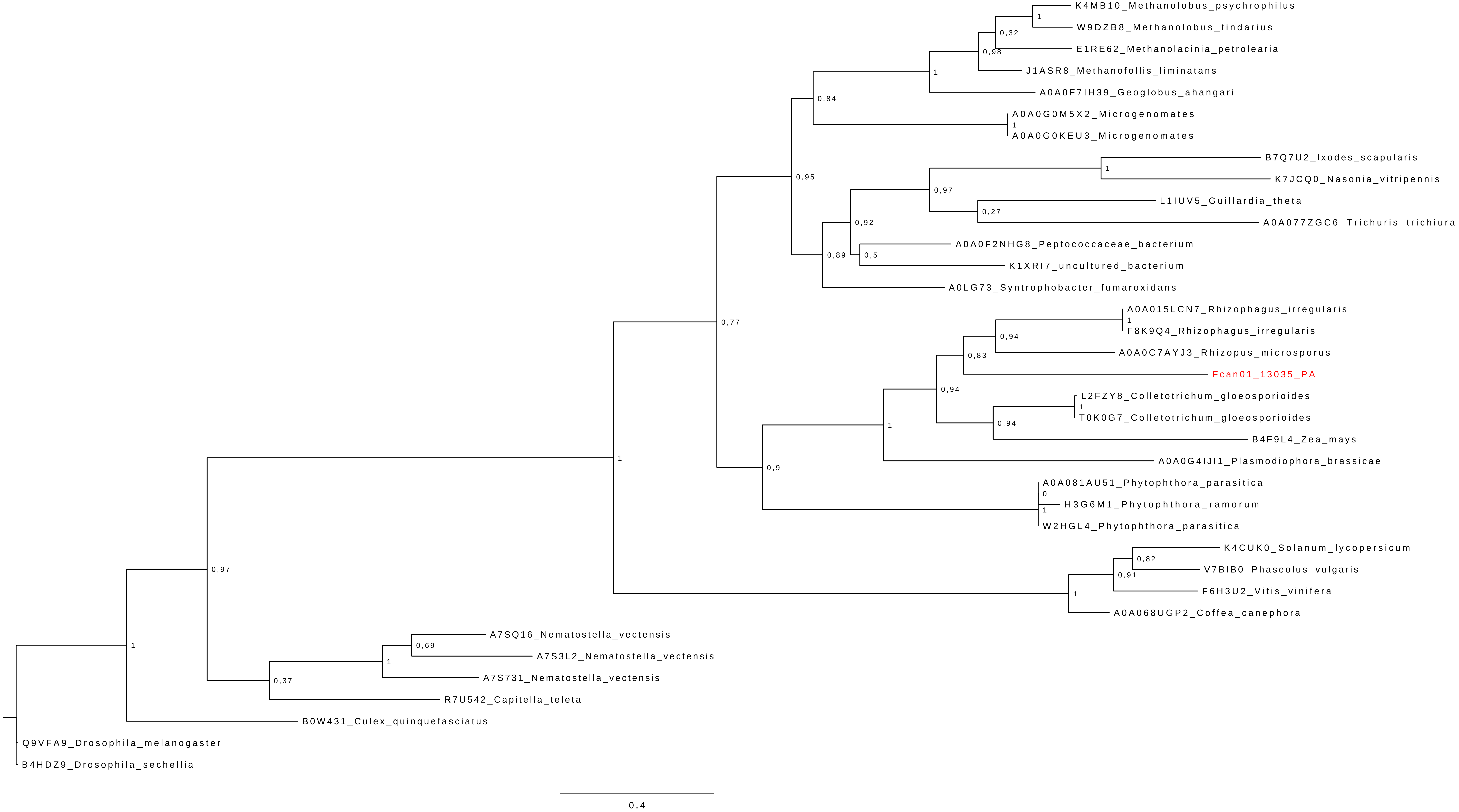
K4MB10_Methanolobus_psychrophilus
W9DZB8_Methanolobus_tindarius
E1RE62_Methanolacinia_petrolearia
J1ASR8_Methanofollis_liminatans
A0A0F7IH39_Geoglobus_ahangari
A0A0G0M5X2_Microgenomates
A0A0G0KEU3_Microgenomates
B7Q7U2_Ixodes_scapularis
K7JCQ0_Nasonia_vitripennis
L1IUV5_Guillardia_theta
A0A077ZGC6_Trichuris_trichiura
A0A0F2NHG8_Peptococcaceae_bacterium
K1XRI7_uncultured_bacterium
A0LG73_Syntrophobacter_fumaroxidans
A0A015LCN7_Rhizophagus_irregularis
F8K9Q4_Rhizophagus_irregularis
A0A0C7AYJ3_Rhizopus_microsporus
Fcan01_13035_PA
L2FZY8_Colletotrichum_gloeosporioides
T0K0G7_Colletotrichum_gloeosporioides
B4F9L4_Zea_mays
A0A0G4IJI1_Plasmodiophora_brassicae
A0A081AU51_Phytophthora_parasitica
H3G6M1_Phytophthora_ramorum
W2HGL4_Phytophthora_parasitica
K4CUK0_Solanum_lycopersicum
V7BIB0_Phaseolus_vulgaris
F6H3U2_Vitis_vinifera
A0A068UGP2_Coffea_canephora
A7SQ16_Nematostella_vectensis
A7S3L2_Nematostella_vectensis
A7S731_Nematostella_vectensis
R7U542_Capitella_teleta
B0W431_Culex_quinquefasciatus
Q9VFA9_Drosophila_melanogaster
B4HDZ9_Drosophila_sechellia
0.4

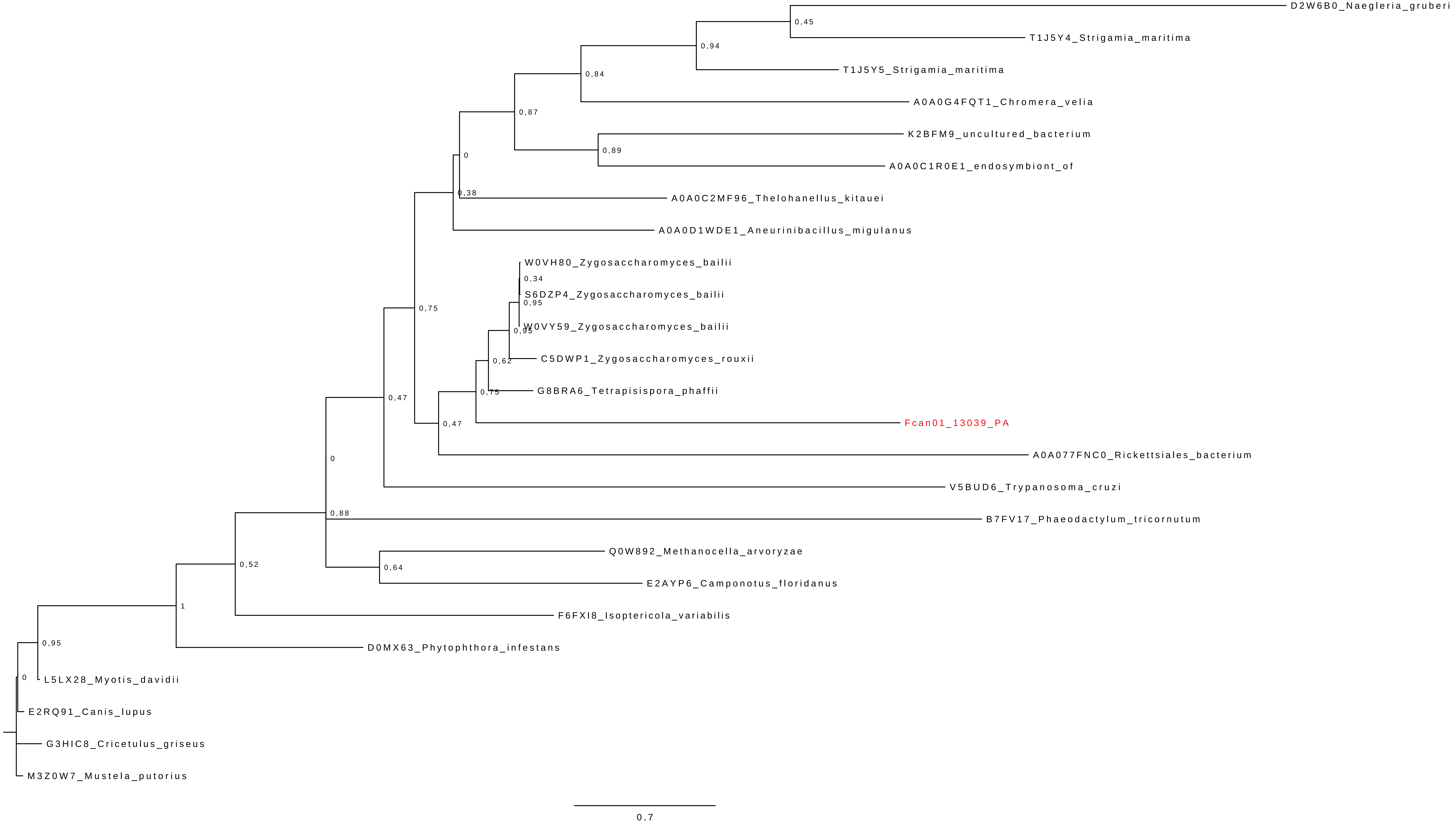
D2W6B0_Naegleria_gruberi
T1J5Y4_Strigamia_maritima
T1J5Y5_Strigamia_maritima
A0A0G4FQT1_Chromera_velia
K2BFM9_uncultured_bacterium
A0A0C1R0E1_endosymbiont_of
A0A0C2MF96_Thelohanellus_kitauei
A0A0D1WDE1_Aneurinibacillus_migulanus
W0VH80_Zygosaccharomyces_bailii
S6DZP4_Zygosaccharomyces_bailii
W0VY59_Zygosaccharomyces_bailii
C5DWP1_Zygosaccharomyces_rouxii
G8BRA6_Tetrapisispora_phaffii
Fcan01_13039_PA
A0A077FNC0_Rickettsiales_bacterium
V5BUD6_Trypanosoma_cruzi
B7FV17_Phaeodactylum_tricornutum
Q0W892_Methanocella_arvoryzae
E2AYP6_Camponotus_floridanus
F6FXI8_Isoptericola_variabilis
D0MX63_Phytophthora_infestans
L5LX28_Myotis_davidii
E2RQ91_Canis_lupus
G3HIC8_Cricetulus_griseus
M3Z0W7_Mustela_putorius
0,45
0,94
0,84
0,87
0,89
0
0,38
0,34
0,95
0,95
0,62
0,75
0,75
0,47
0,47
0
0,88
0,64
0,52
1
0,95
0
0.7

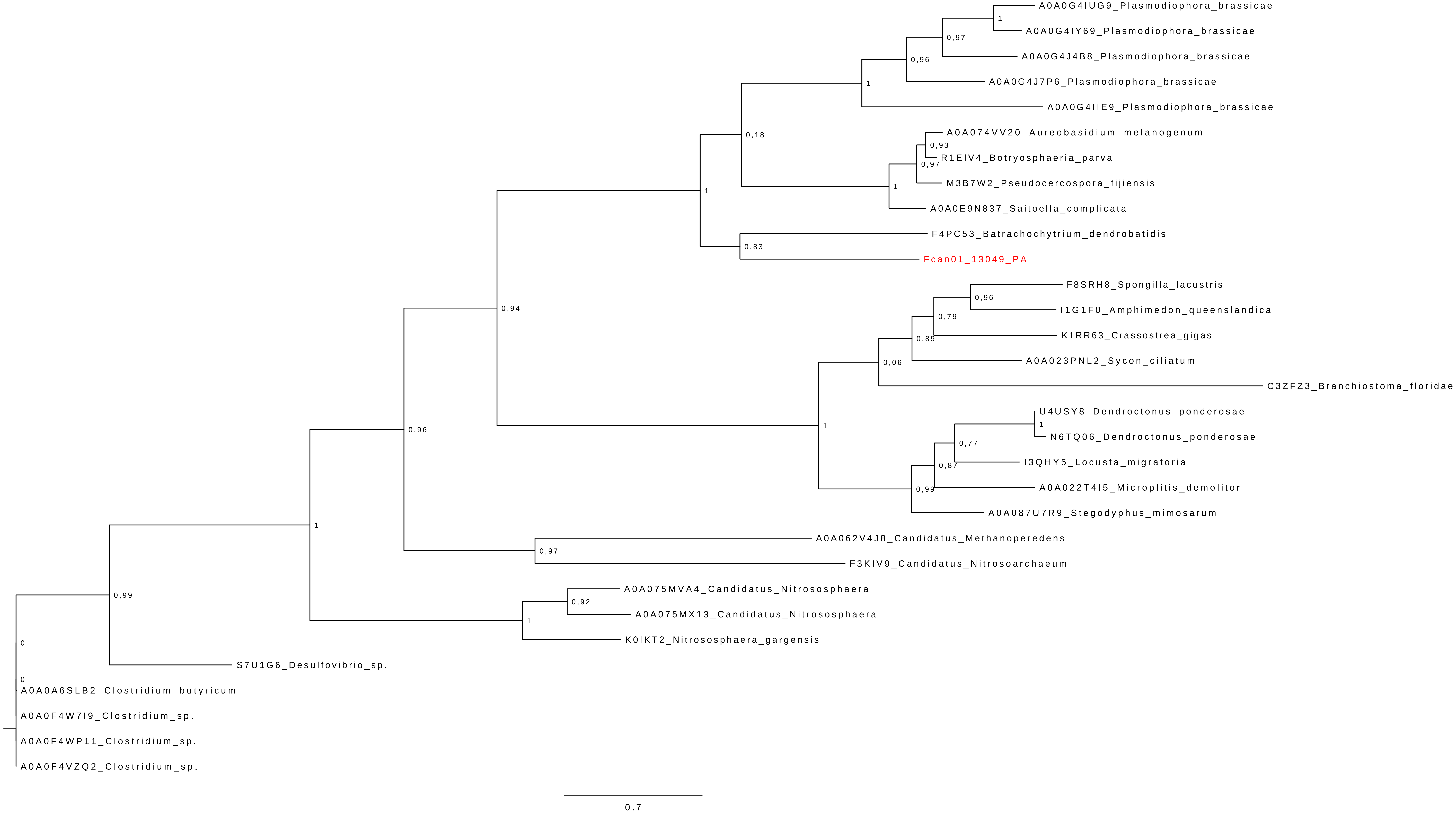
A0A0G4IUG9_Plasmodiophora_brassicae
1
A0A0G4IY69_Plasmodiophora_brassicae
0,97
A0A0G4J4B8_Plasmodiophora_brassicae
0,96
A0A0G4J7P6_Plasmodiophora_brassicae
1
A0A0G4IIE9_Plasmodiophora_brassicae
0,18
A0A074VV20_Aureobasidium_melanogenum
0,93
R1EIV4_Botryosphaeria_parva
0,97
M3B7W2_Pseudocercospora_fijiensis
1
1
A0A0E9N837_Saitoella_complicata
F4PC53_Batrachochytrium_dendrobatidis
0,83
Fcan01_13049_PA
F8SRH8_Spongilla_lacustris
0,96
I1G1F0_Amphimedon_queenslandica
0,79
K1RR63_Crassostrea_gigas
0,89
0,94
A0A023PNL2_Sycon_ciliatum
0,06
C3ZFZ3_Branchiostoma_floridae
U4USY8_Dendroctonus_ponderosae
1
1
N6TQ06_Dendroctonus_ponderosae
0,77
0,96
I3QHY5_Locusta_migratoria
0,87
A0A022T4I5_Microplitis_demolitor
0,99
A0A087U7R9_Stegodyphus_mimosarum
1
A0A062V4J8_Candidatus_Methanoperedens
0,97
F3KIV9_Candidatus_Nitrosoarchaeum
A0A075MVA4_Candidatus_Nitrososphaera
0,92
0,99
A0A075MX13_Candidatus_Nitrososphaera
1
K0IKT2_Nitrososphaera_gargensis
0
S7U1G6_Desulfovibrio_sp.
0
A0A0A6SLB2_Clostridium_butyricum
A0A0F4W7I9_Clostridium_sp.
A0A0F4WP11_Clostridium_sp.
A0A0F4VZQ2_Clostridium_sp.
0.7

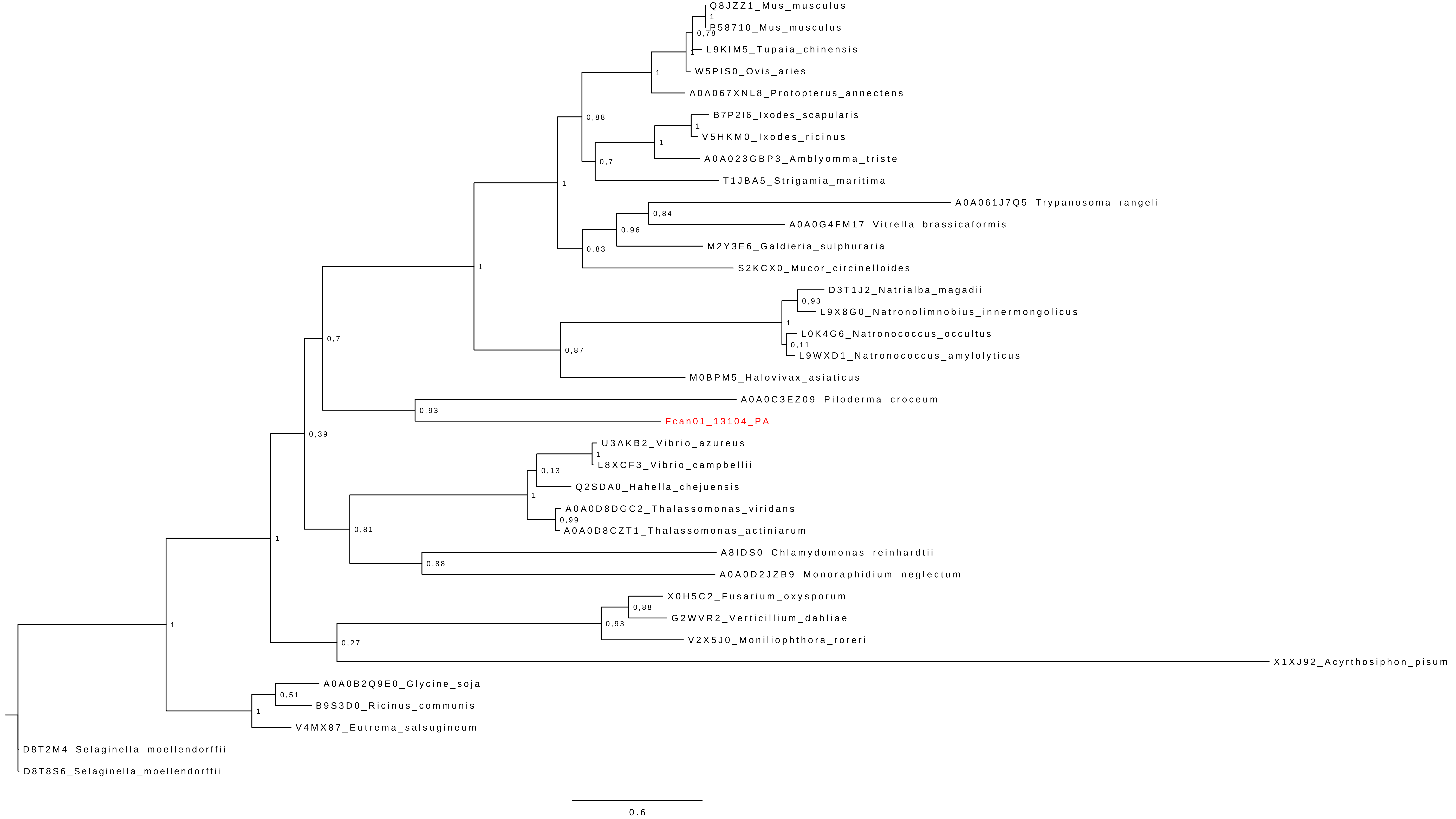
Q8JZZ1_Mus_musculus
P58710_Mus_musculus
L9KIM5_Tupaia_chinensis
W5PIS0_Ovis_aries
A0A067XNL8_Protopterus_annectens
B7P2I6_Ixodes_scapularis
V5HKM0_Ixodes_ricinus
A0A023GBP3_Amblyomma_triste
T1JBA5_Strigamia_maritima
A0A061J7Q5_Trypanosoma_rangeli
A0A0G4FM17_Vitrella_brassicaformis
M2Y3E6_Galdieria_sulphuraria
S2KCX0_Mucor_circinelloides
D3T1J2_Natrialba_magadii
L9X8G0_Natronolimnobius_innermongolicus
L0K4G6_Natronococcus_occultus
L9WXD1_Natronococcus_amylolyticus
M0BPM5_Halovivax_asiaticus
A0A0C3EZ09_Piloderma_croceum
Fcan01_13104_PA
U3AKB2_Vibrio_azureus
L8XCF3_Vibrio_campbellii
Q2SDA0_Hahella_chejuensis
A0A0D8DGC2_Thalassomonas_viridans
A0A0D8CZT1_Thalassomonas_actiniarum
A8IDS0_Chlamydomonas_reinhardtii
A0A0D2JZB9_Monoraphidium_neglectum
X0H5C2_Fusarium_oxysporum
G2WVR2_Verticillium_dahliae
V2X5J0_Moniliophthora_roreri
X1XJ92_Acyrthosiphon_pisum
A0A0B2Q9E0_Glycine_soja
B9S3D0_Ricinus_communis
V4MX87_Eutrema_salsugineum
D8T2M4_Selaginella_moellendorffii
D8T8S6_Selaginella_moellendorffii
0.6

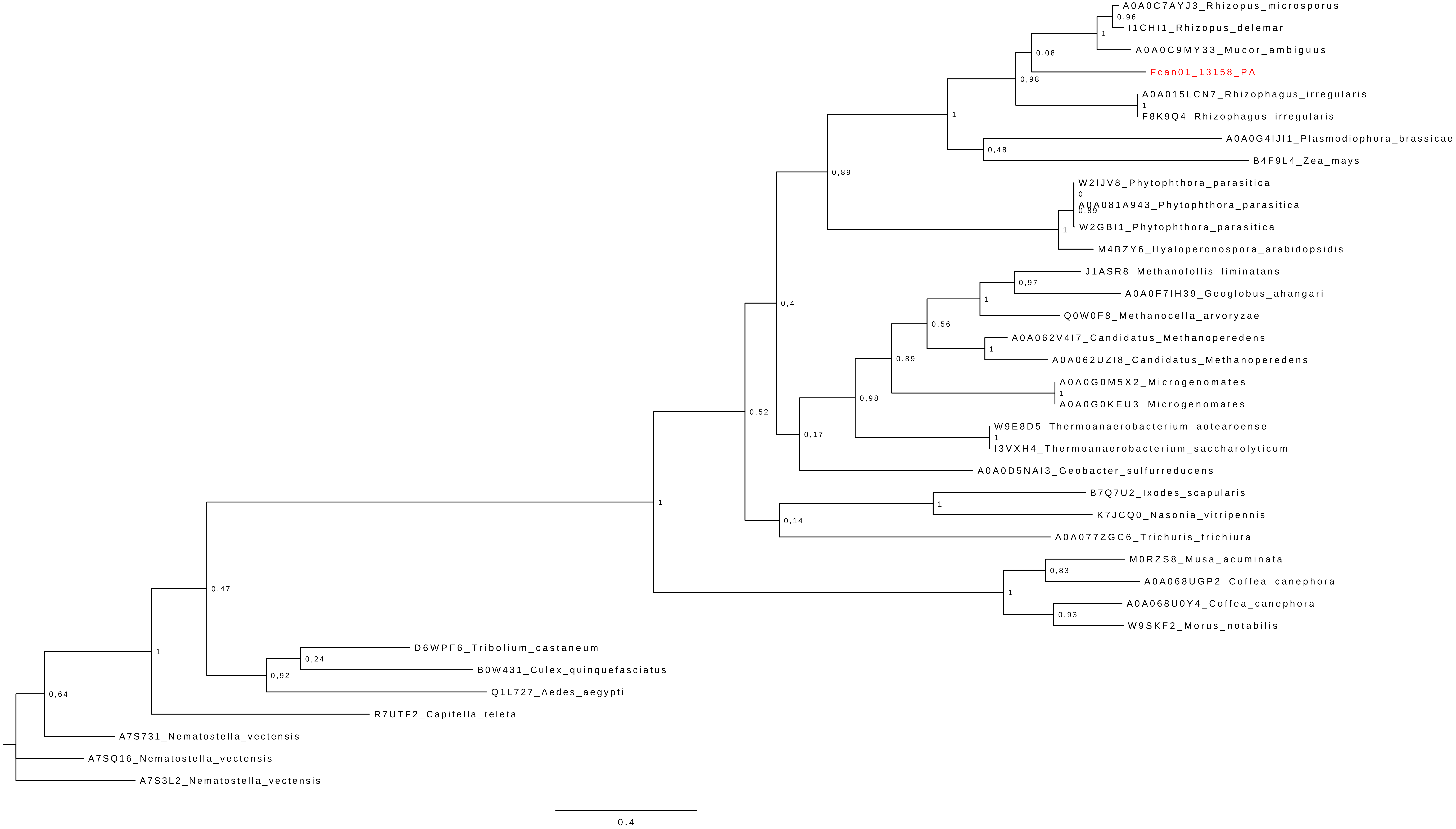
A0A0C7AYJ3_Rhizopus_microsporus
0,96
I1CHI1_Rhizopus_delemar
1
A0A0C9MY33_Mucor_ambiguus
0,08
Fcan01_13158_PA
0,98
A0A015LCN7_Rhizophagus_irregularis
1
F8K9Q4_Rhizophagus_irregularis
1
A0A0G4IJI1_Plasmodiophora_brassicae
0,48
B4F9L4_Zea_mays
0,89
W2IJV8_Phytophthora_parasitica
0
A0A081A943_Phytophthora_parasitica
0,89
W2GBI1_Phytophthora_parasitica
1
M4BZY6_Hyaloperonospora_arabidopsidis
J1ASR8_Methanofollis_liminatans
0,97
A0A0F7IH39_Geoglobus_ahangari
1
Q0W0F8_Methanocella_arvoryzae
0,4
0,56
A0A062V4I7_Candidatus_Methanoperedens
1
A0A062UZI8_Candidatus_Methanoperedens
0,89
A0A0G0M5X2_Microgenomates
1
A0A0G0KEU3_Microgenomates
0,98
0,52
W9E8D5_Thermoanaerobacterium_aotearoense
1
I3VXH4_Thermoanaerobacterium_saccharolyticum
0,17
A0A0D5NAI3_Geobacter_sulfurreducens
B7Q7U2_Ixodes_scapularis
1
1
K7JCQ0_Nasonia_vitripennis
0,14
A0A077ZGC6_Trichuris_trichiura
M0RZS8_Musa_acuminata
0,83
A0A068UGP2_Coffea_canephora
0,47
1
A0A068U0Y4_Coffea_canephora
0,93
W9SKF2_Morus_notabilis
D6WPF6_Tribolium_castaneum
0,24
1
B0W431_Culex_quinquefasciatus
0,92
Q1L727_Aedes_aegypti
0,64
R7UTF2_Capitella_teleta
A7S731_Nematostella_vectensis
A7SQ16_Nematostella_vectensis
A7S3L2_Nematostella_vectensis
0.4

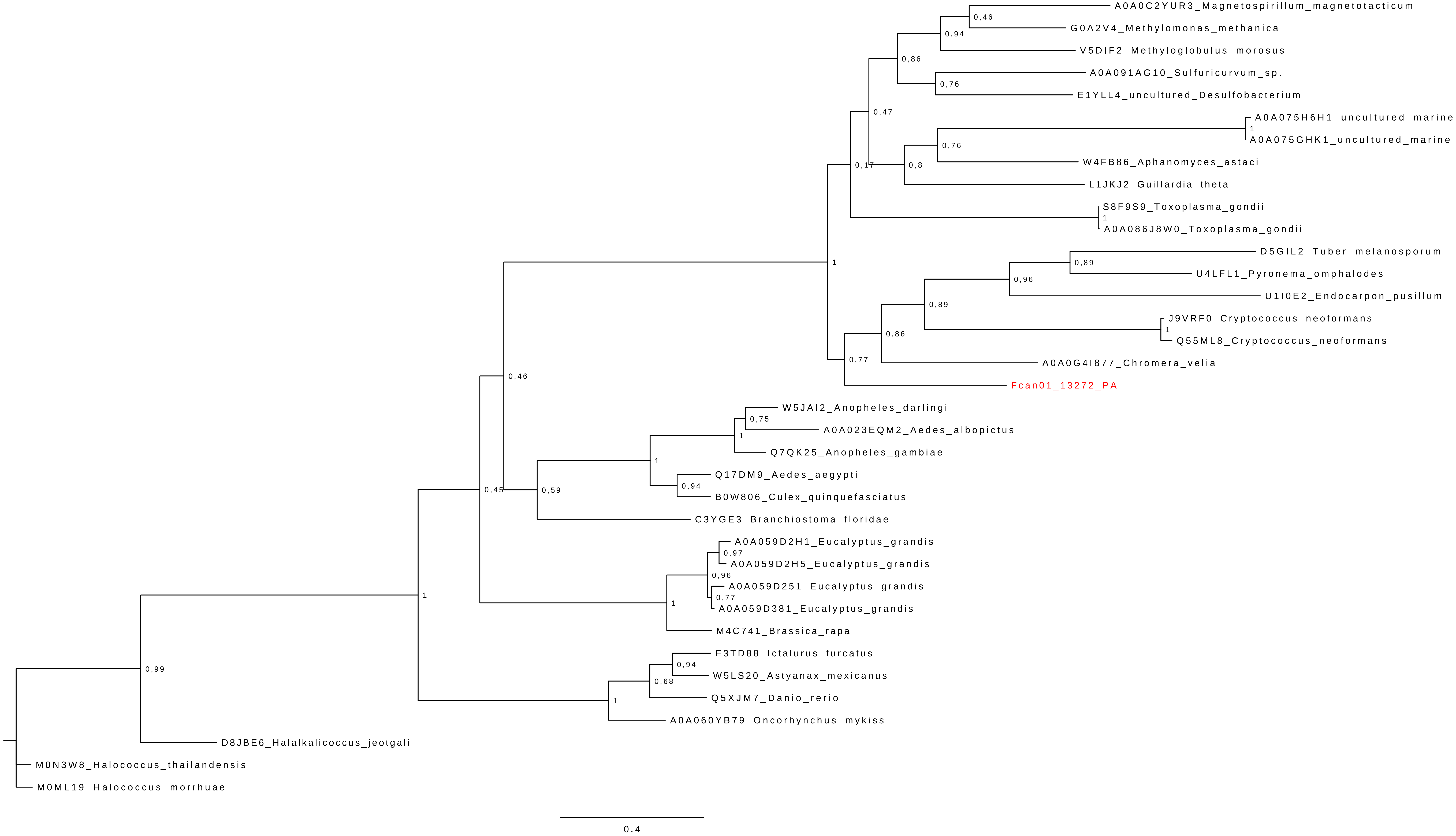
A0A0C2YUR3_Magnetospirillum_magnetotacticum
0,46
G0A2V4_Methylomonas_methanica
0,94
V5DIF2_Methyloglobulus_morosus
0,86
A0A091AG10_Sulfuricurvum_sp.
0,76
E1YLL4_uncultured_Desulfobacterium
0,47
A0A075H6H1_uncultured_marine
1
A0A075GHK1_uncultured_marine
0,76
W4FB86_Aphanomyces_astaci
0,17
0,8
L1JKJ2_Guillardia_theta
S8F9S9_Toxoplasma_gondii
1
A0A086J8W0_Toxoplasma_gondii
1
D5GIL2_Tuber_melanosporum
0,89
U4LFL1_Pyronema_omphalodes
0,96
U1I0E2_Endocarpon_pusillum
0,89
J9VRF0_Cryptococcus_neoformans
1
Q55ML8_Cryptococcus_neoformans
0,86
A0A0G4I877_Chromera_velia
0,77
Fcan01_13272_PA
0,46
W5JAI2_Anopheles_darlingi
0,75
A0A023EQM2_Aedes_albopictus
1
Q7QK25_Anopheles_gambiae
1
Q17DM9_Aedes_aegypti
0,94
B0W806_Culex_quinquefasciatus
0,45
0,59
C3YGE3_Branchiostoma_floridae
A0A059D2H1_Eucalyptus_grandis
0,97
A0A059D2H5_Eucalyptus_grandis
0,96
A0A059D251_Eucalyptus_grandis
0,77
A0A059D381_Eucalyptus_grandis
1
M4C741_Brassica_rapa
1
E3TD88_Ictalurus_furcatus
0,94
W5LS20_Astyanax_mexicanus
0,68
Q5XJM7_Danio_rerio
1
A0A060YB79_Oncorhynchus_mykiss
0,99
D8JBE6_Halalkalicoccus_jeotgali
M0N3W8_Halococcus_thailandensis
M0ML19_Halococcus_morrhuae
0.4

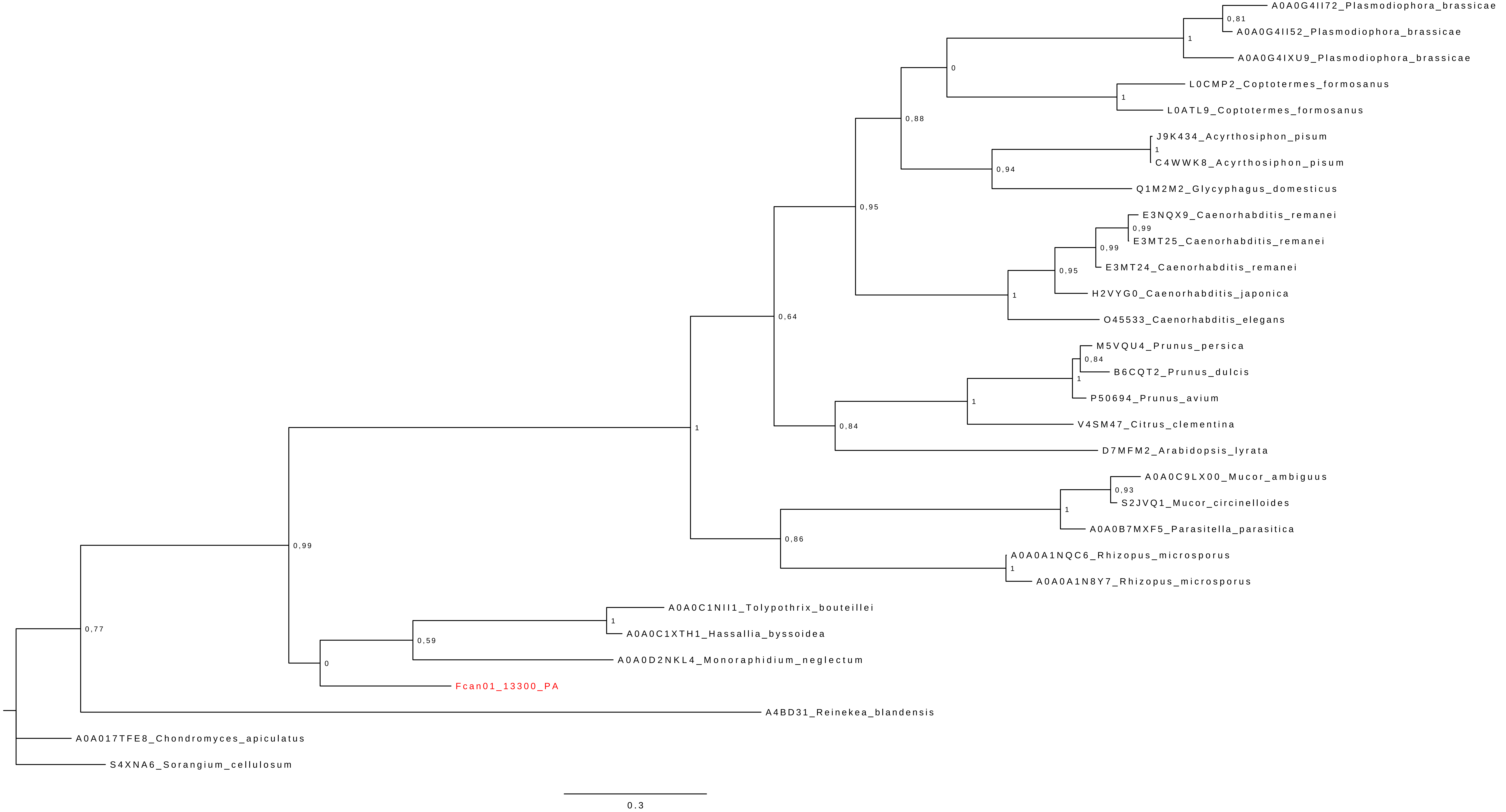
A0A0G4II72_Plasmodiophora_brassicae
0,81
A0A0G4II52_Plasmodiophora_brassicae
1
A0A0G4IXU9_Plasmodiophora_brassicae
0
L0CMP2_Coptotermes_formosanus
1
L0ATL9_Coptotermes_formosanus
0,88
J9K434_Acyrthosiphon_pisum
1
C4WWK8_Acyrthosiphon_pisum
0,94
Q1M2M2_Glycyphagus_domesticus
0,95
E3NQX9_Caenorhabditis_remanei
0,99
E3MT25_Caenorhabditis_remanei
0,99
E3MT24_Caenorhabditis_remanei
0,95
H2VYG0_Caenorhabditis_japonica
1
O45533_Caenorhabditis_elegans
0,64
M5VQU4_Prunus_persica
0,84
B6CQT2_Prunus_dulcis
1
P50694_Prunus_avium
1
V4SM47_Citrus_clementina
0,84
D7MFM2_Arabidopsis_lyrata
1
A0A0C9LX00_Mucor_ambiguus
0,93
S2JVQ1_Mucor_circinelloides
1
A0A0B7MXF5_Parasitella_parasitica
0,86
A0A0A1NQC6_Rhizopus_microsporus
1
A0A0A1N8Y7_Rhizopus_microsporus
0,99
A0A0C1NII1_Tolypothrix_bouteillei
1
A0A0C1XTH1_Hassallia_byssoidea
0,59
A0A0D2NKL4_Monoraphidium_neglectum
0
Fcan01_13300_PA
0,77
A4BD31_Reinekea_blandensis
A0A017TFE8_Chondromyces_apiculatus
S4XNA6_Sorangium_cellulosum
0.3

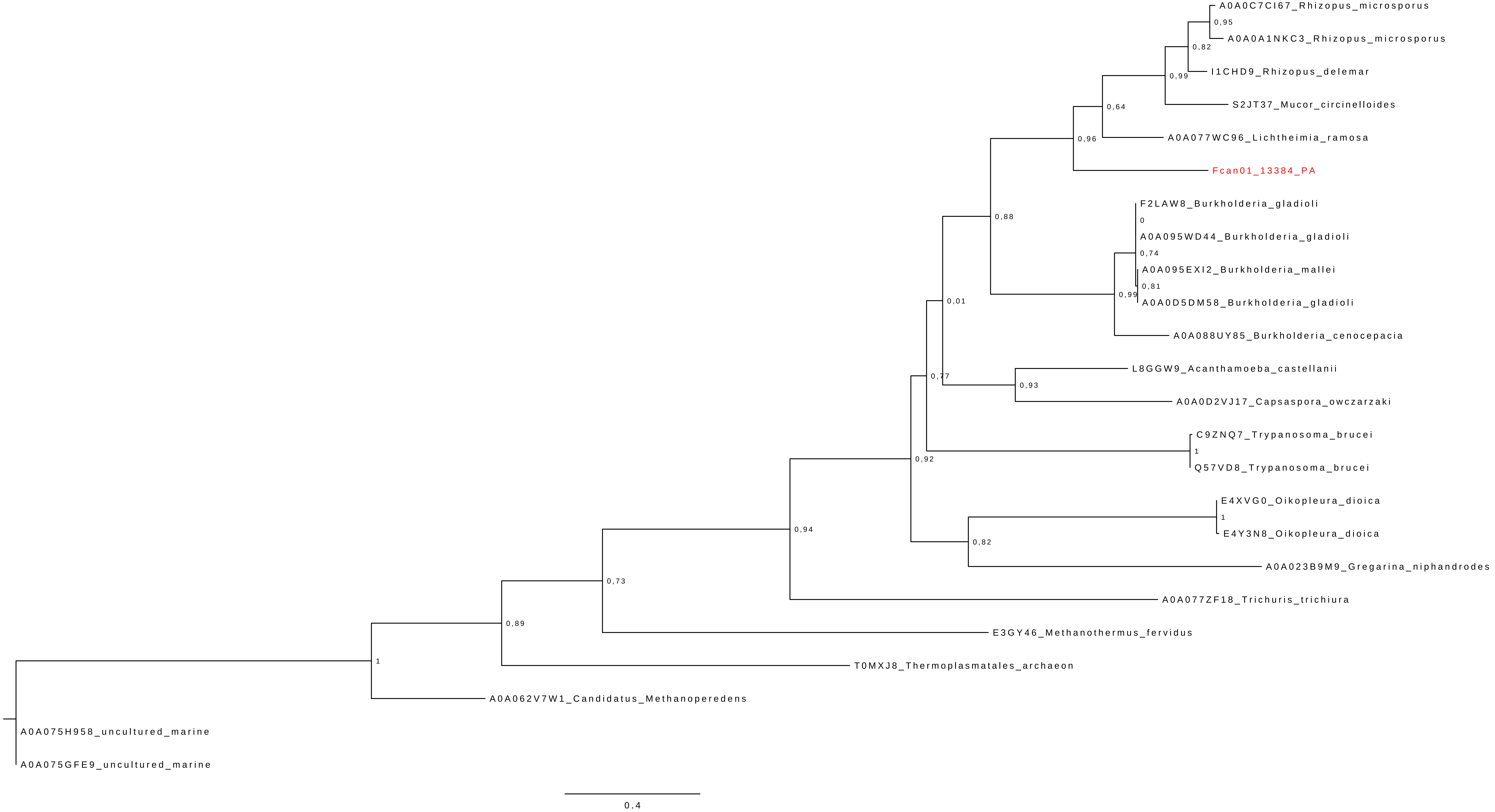

A0A0C7CI67_Rhizopus_microsporus
0,95
A0A0A1NKC3_Rhizopus_microsporus
0,82
I1CHD9_Rhizopus_delemar
0,99
S2JT37_Mucor_circinelloides
0,64
A0A077WC96_Lichtheimia_ramosa
0,96
Fcan01_13384_PA
F2LAW8_Burkholderia_gladioli
0
A0A095WD44_Burkholderia_gladioli
0,74
A0A095EXI2_Burkholderia_mallei
0,81
A0A0D5DM58_Burkholderia_gladioli
0,88
0,99
A0A088UY85_Burkholderia_cenocepacia
0,01
L8GGW9_Acanthamoeba_castellanii
0,77
0,93
A0A0D2VJ17_Capsaspora_owczarzaki
C9ZNQ7_Trypanosoma_brucei
1
Q57VD8_Trypanosoma_brucei
0,92
E4XVG0_Oikopleura_dioica
1
E4Y3N8_Oikopleura_dioica
0,94
0,82
A0A023B9M9_Gregarina_niphandrodes
A0A077ZF18_Trichuris_trichiura
0,73
E3GY46_Methanothermus_fervidus
0,89
T0MXJ8_Thermoplasmatales_archaeon
1
A0A062V7W1_Candidatus_Methanoperedens
A0A075H958_uncultured_marine
A0A075GFE9_uncultured_marine
0.4

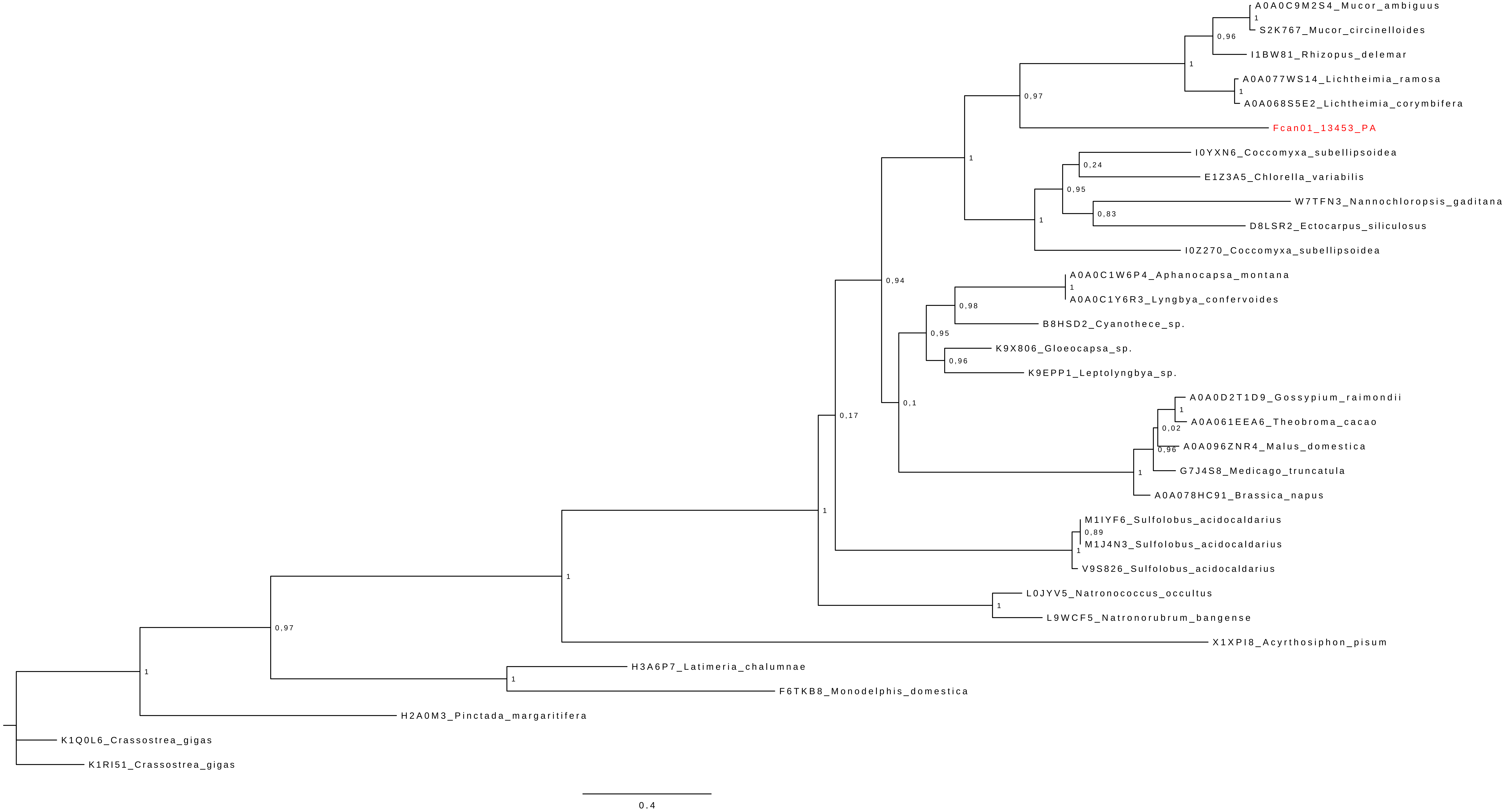

A0A0C9M2S4_Mucor_ambiguus
S2K767_Mucor_circinelloides
I1BW81_Rhizopus_delemar
A0A077WS14_Lichtheimia_ramosa
A0A068S5E2_Lichtheimia_corymbifera
Fcan01_13453_PA
I0YXN6_Coccomyxa_subellipsoidea
E1Z3A5_Chlorella_variabilis
W7TFN3_Nannochloropsis_gaditana
D8LSR2_Ectocarpus_siliculosus
I0Z270_Coccomyxa_subellipsoidea
A0A0C1W6P4_Aphanocapsa_montana
A0A0C1Y6R3_Lyngbya_confervoides
B8HSD2_Cyanothece_sp.
K9X806_Gloeocapsa_sp.
K9EPP1_Leptolyngbya_sp.
A0A0D2T1D9_Gossypium_raimondii
A0A061EEA6_Theobroma_cacao
A0A096ZNR4_Malus_domestica
G7J4S8_Medicago_truncatula
A0A078HC91_Brassica_napus
M1IYF6_Sulfolobus_acidocaldarius
M1J4N3_Sulfolobus_acidocaldarius
V9S826_Sulfolobus_acidocaldarius
L0JYV5_Natronococcus_occultus
L9WCF5_Natronorubrum_bangense
X1XPI8_Acyrthosiphon_pisum
H3A6P7_Latimeria_chalumnae
F6TKB8_Monodelphis_domestica
H2A0M3_Pinctada_margaritifera
K1Q0L6_Crassostrea_gigas
K1RI51_Crassostrea_gigas
1
0,96
1
1
0,97
1
0,24
0,95
0,83
1
0,94
1
0,98
0,95
0,96
0,1
1
0,02
0,96
1
0,17
0,89
1
1
1
1
0,97
1
1
0.4

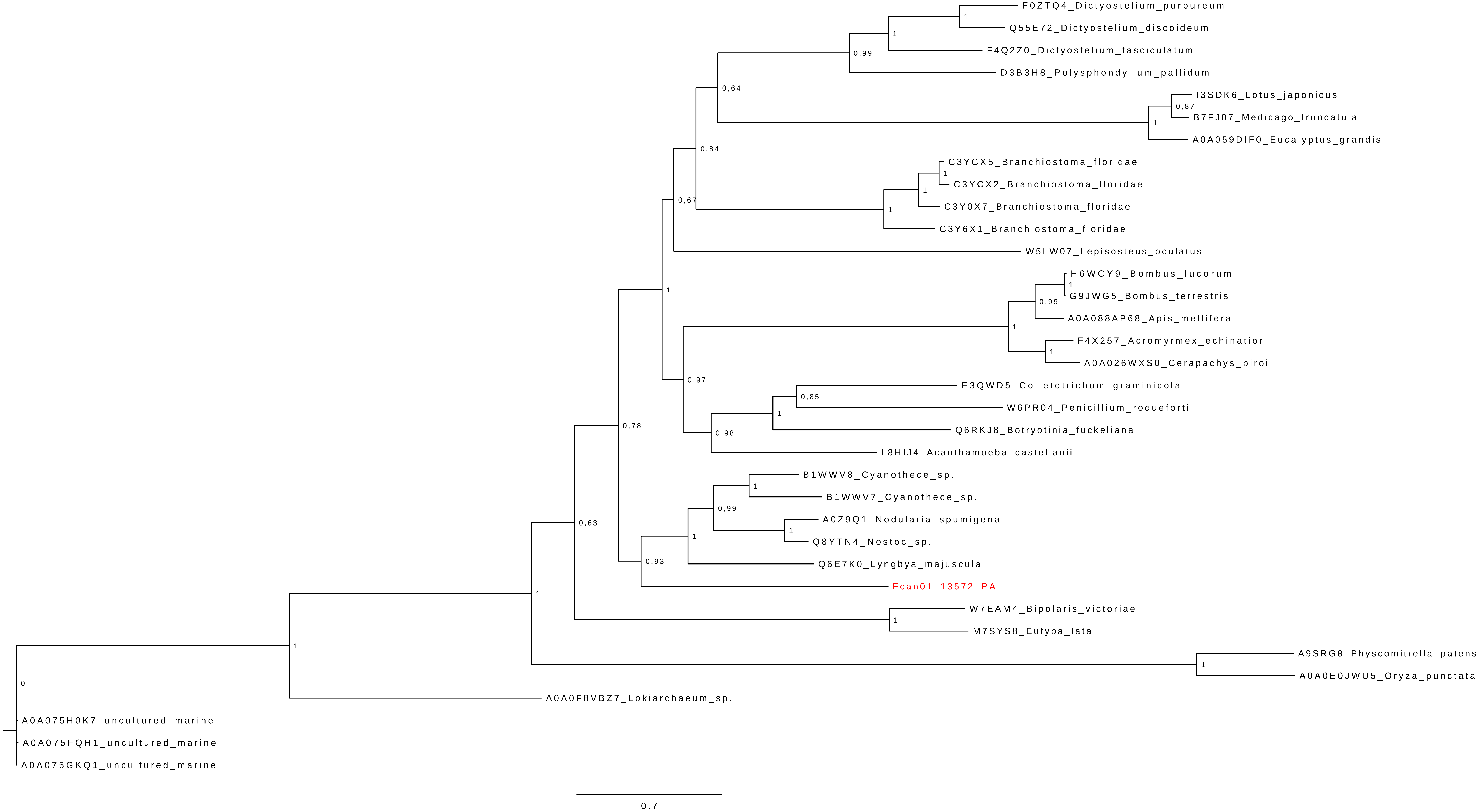
F0ZTQ4_Dictyostelium_purpureum
Q55E72_Dictyostelium_discoideum
F4Q2Z0_Dictyostelium_fasciculatum
D3B3H8_Polysphondylium_pallidum
I3SDK6_Lotus_japonicus
B7FJ07_Medicago_truncatula
A0A059DIF0_Eucalyptus_grandis
C3YCX5_Branchiostoma_floridae
C3YCX2_Branchiostoma_floridae
C3Y0X7_Branchiostoma_floridae
C3Y6X1_Branchiostoma_floridae
W5LW07_Lepisosteus_oculatus
H6WCY9_Bombus_lucorum
G9JWG5_Bombus_terrestris
A0A088AP68_Apis_mellifera
F4X257_Acromyrmex_echinatior
A0A026WXS0_Cerapachys_biroi
E3QWD5_Colletotrichum_graminicola
W6PR04_Penicillium_roqueforti
Q6RKJ8_Botryotinia_fuckeliana
L8HIJ4_Acanthamoeba_castellanii
B1WWV8_Cyanothece_sp.
B1WWV7_Cyanothece_sp.
A0Z9Q1_Nodularia_spumigena
Q8YTN4_Nostoc_sp.
Q6E7K0_Lyngbya_majuscula
Fcan01_13572_PA
W7EAM4_Bipolaris_victoriae
M7SYS8_Eutypa_lata
A9SRG8_Physcomitrella_patens
A0A0E0JWU5_Oryza_punctata
A0A0F8VBZ7_Lokiarchaeum_sp.
A0A075H0K7_uncultured_marine
A0A075FQH1_uncultured_marine
A0A075GKQ1_uncultured_marine
0,99
0,64
0,87
0,84
0,67
0,97
0,85
0,98
0,78
0,99
0,63
0,93
0
0.7

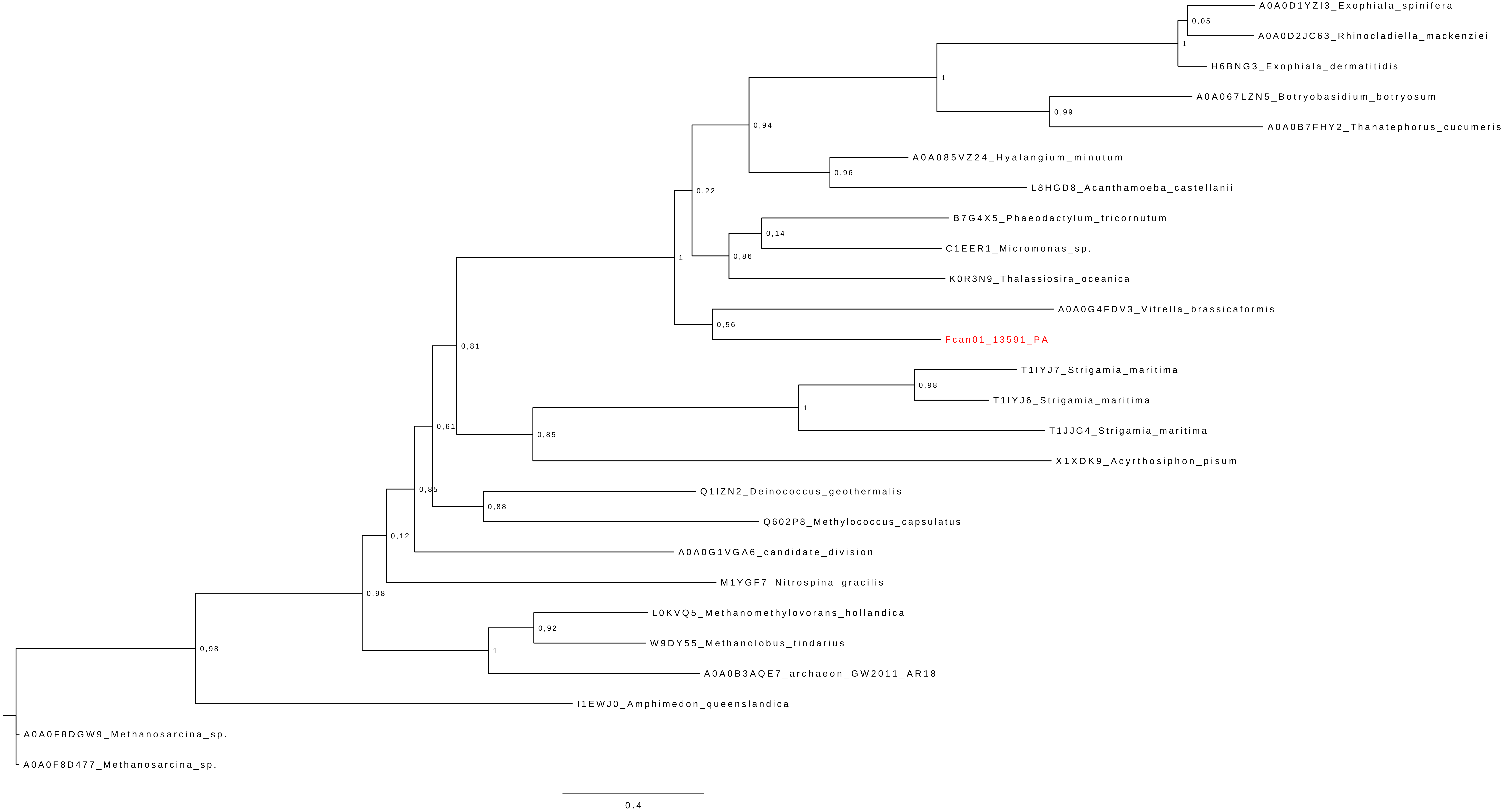
A0A0D1YZI3_Exophiala_spinifera
0,05
A0A0D2JC63_Rhinocladiella_mackenziei
1
H6BNG3_Exophiala_dermatitidis
1
A0A067LZN5_Botryobasidium_botryosum
0,99
A0A0B7FHY2_Thanatephorus_cucumeris
0,94
A0A085VZ24_Hyalangium_minutum
0,96
L8HGD8_Acanthamoeba_castellanii
0,22
B7G4X5_Phaeodactylum_tricornutum
0,14
C1EER1_Micromonas_sp.
1
0,86
K0R3N9_Thalassiosira_oceanica
A0A0G4FDV3_Vitrella_brassicaformis
0,56
Fcan01_13591_PA
0,81
T1IYJ7_Strigamia_maritima
0,98
T1IYJ6_Strigamia_maritima
1
0,61
0,85
T1JJG4_Strigamia_maritima
X1XDK9_Acyrthosiphon_pisum
Q1IZN2_Deinococcus_geothermalis
0,85
0,88
Q602P8_Methylococcus_capsulatus
0,12
A0A0G1VGA6_candidate_division
M1YGF7_Nitrospina_gracilis
0,98
L0KVQ5_Methanomethylovorans_hollandica
0,92
W9DY55_Methanolobus_tindarius
0,98
1
A0A0B3AQE7_archaeon_GW2011_AR18
I1EWJ0_Amphimedon_queenslandica
A0A0F8DGW9_Methanosarcina_sp.
A0A0F8D477_Methanosarcina_sp.
0.4

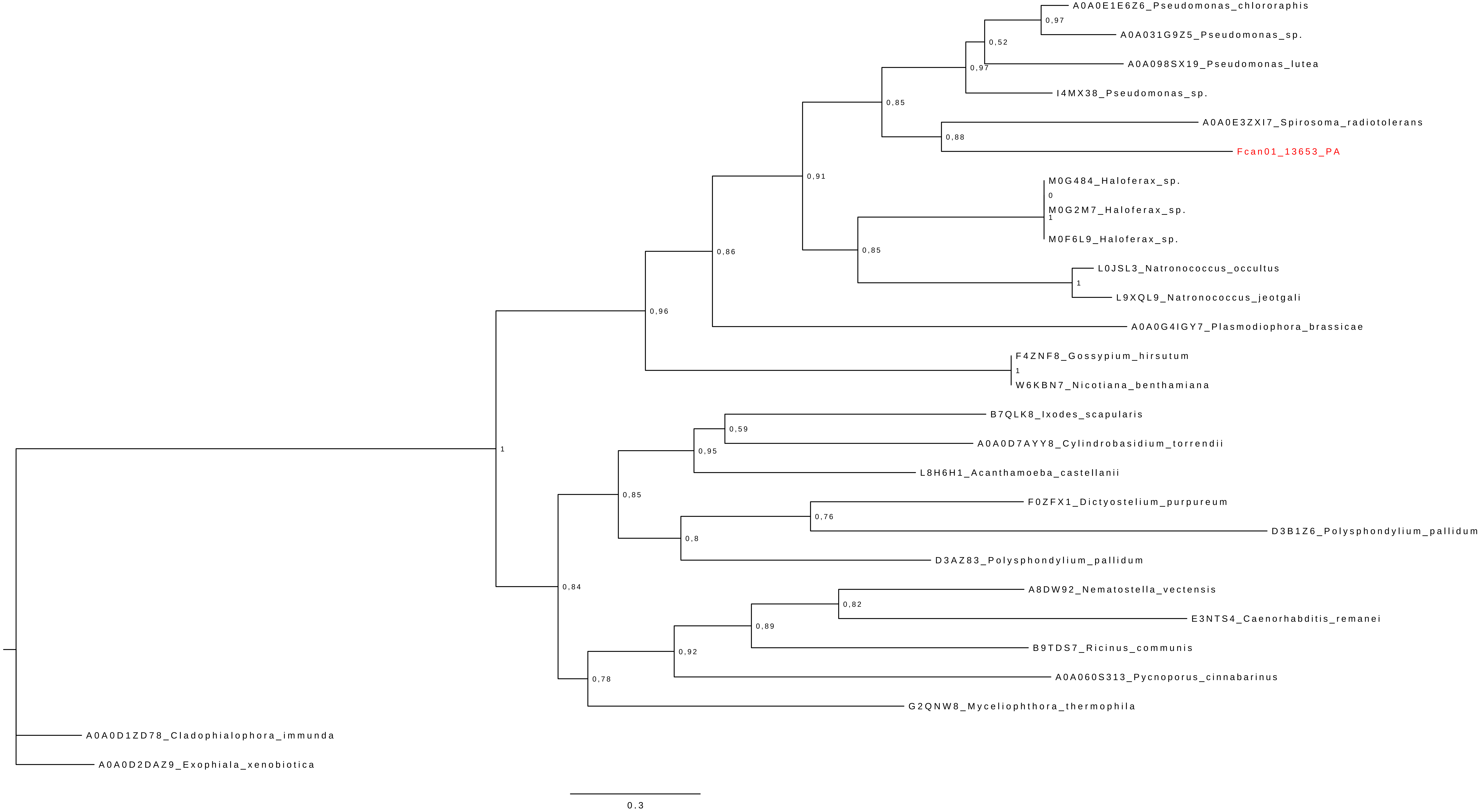
A0A0E1E6Z6_Pseudomonas_chlororaphis
0,97
A0A031G9Z5_Pseudomonas_sp.
0,52
A0A098SX19_Pseudomonas_lutea
0,97
I4MX38_Pseudomonas_sp.
0,85
A0A0E3ZXI7_Spirosoma_radiotolerans
0,88
Fcan01_13653_PA
0,91
M0G484_Haloferax_sp.
0
M0G2M7_Haloferax_sp.
1
M0F6L9_Haloferax_sp.
0,86
0,85
L0JSL3_Natronococcus_occultus
1
L9XQL9_Natronococcus_jeotgali
0,96
A0A0G4IGY7_Plasmodiophora_brassicae
F4ZNF8_Gossypium_hirsutum
1
W6KBN7_Nicotiana_benthamiana
B7QLK8_Ixodes_scapularis
0,59
A0A0D7AYY8_Cylindrobasidium_torrendii
1
0,95
L8H6H1_Acanthamoeba_castellanii
0,85
F0ZFX1_Dictyostelium_purpureum
0,76
D3B1Z6_Polysphondylium_pallidum
0,8
D3AZ83_Polysphondylium_pallidum
0,84
A8DW92_Nematostella_vectensis
0,82
E3NTS4_Caenorhabditis_remanei
0,89
B9TDS7_Ricinus_communis
0,92
A0A060S313_Pycnoporus_cinnabarinus
0,78
G2QNW8_Myceliophthora_thermophila
A0A0D1ZD78_Cladophialophora_immunda
A0A0D2DAZ9_Exophiala_xenobiotica
0.3

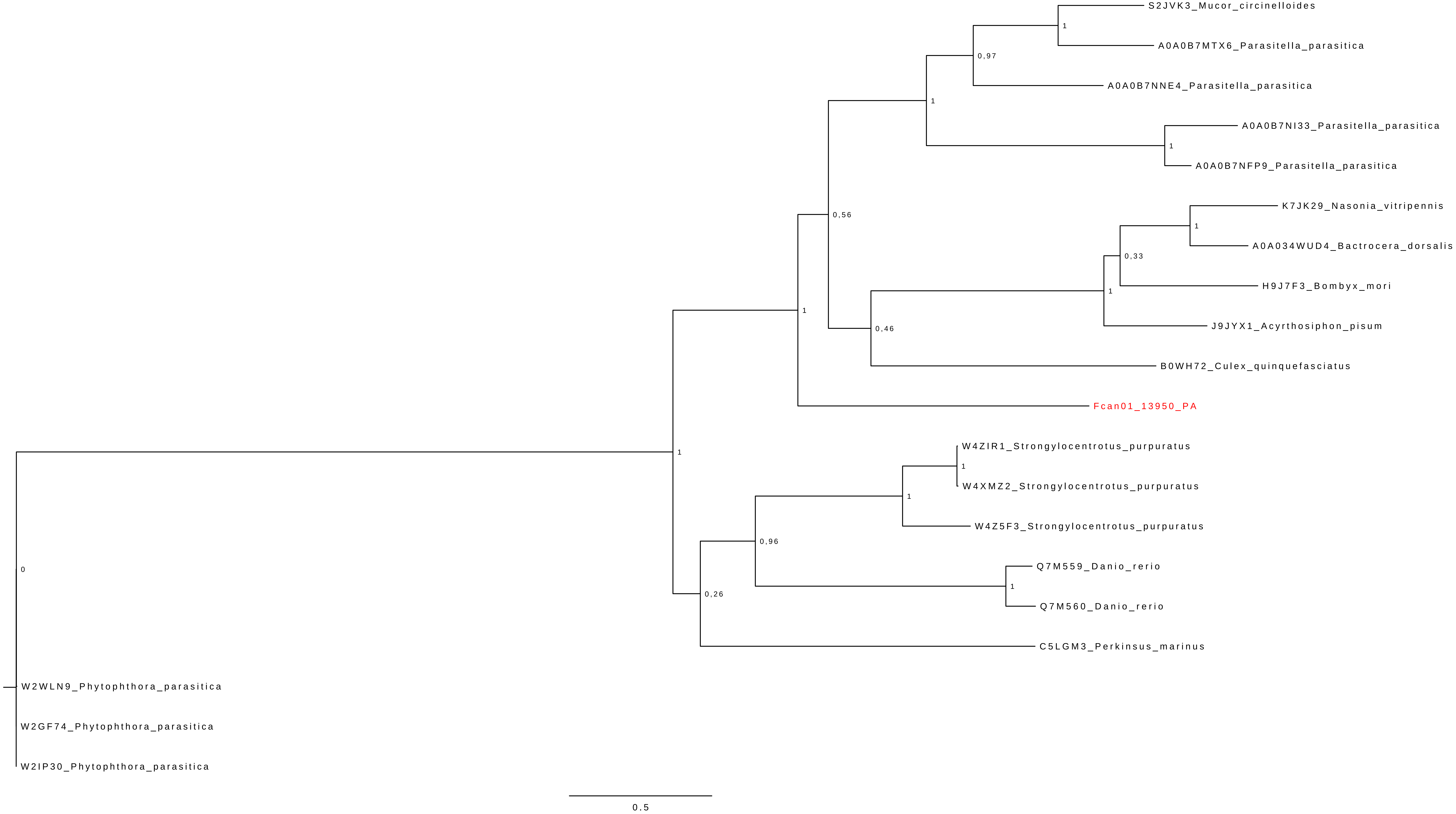
S2JVK3_Mucor_circinelloides
A0A0B7MTX6_Parasitella_parasitica
A0A0B7NNE4_Parasitella_parasitica
A0A0B7NI33_Parasitella_parasitica
A0A0B7NFP9_Parasitella_parasitica
K7JK29_Nasonia_vitripennis
A0A034WUD4_Bactrocera_dorsalis
H9J7F3_Bombyx_mori
J9JYX1_Acyrthosiphon_pisum
B0WH72_Culex_quinquefasciatus
Fcan01_13950_PA
W4ZIR1_Strongylocentrotus_purpuratus
W4XMZ2_Strongylocentrotus_purpuratus
W4Z5F3_Strongylocentrotus_purpuratus
Q7M559_Danio_rerio
Q7M560_Danio_rerio
C5LGM3_Perkinsus_marinus
W2WLN9_Phytophthora_parasitica
W2GF74_Phytophthora_parasitica
W2IP30_Phytophthora_parasitica
1
0,97
1
1
0,56
1
0,33
1
0,46
1
1
1
1
0,96
1
0,26
0
0.5

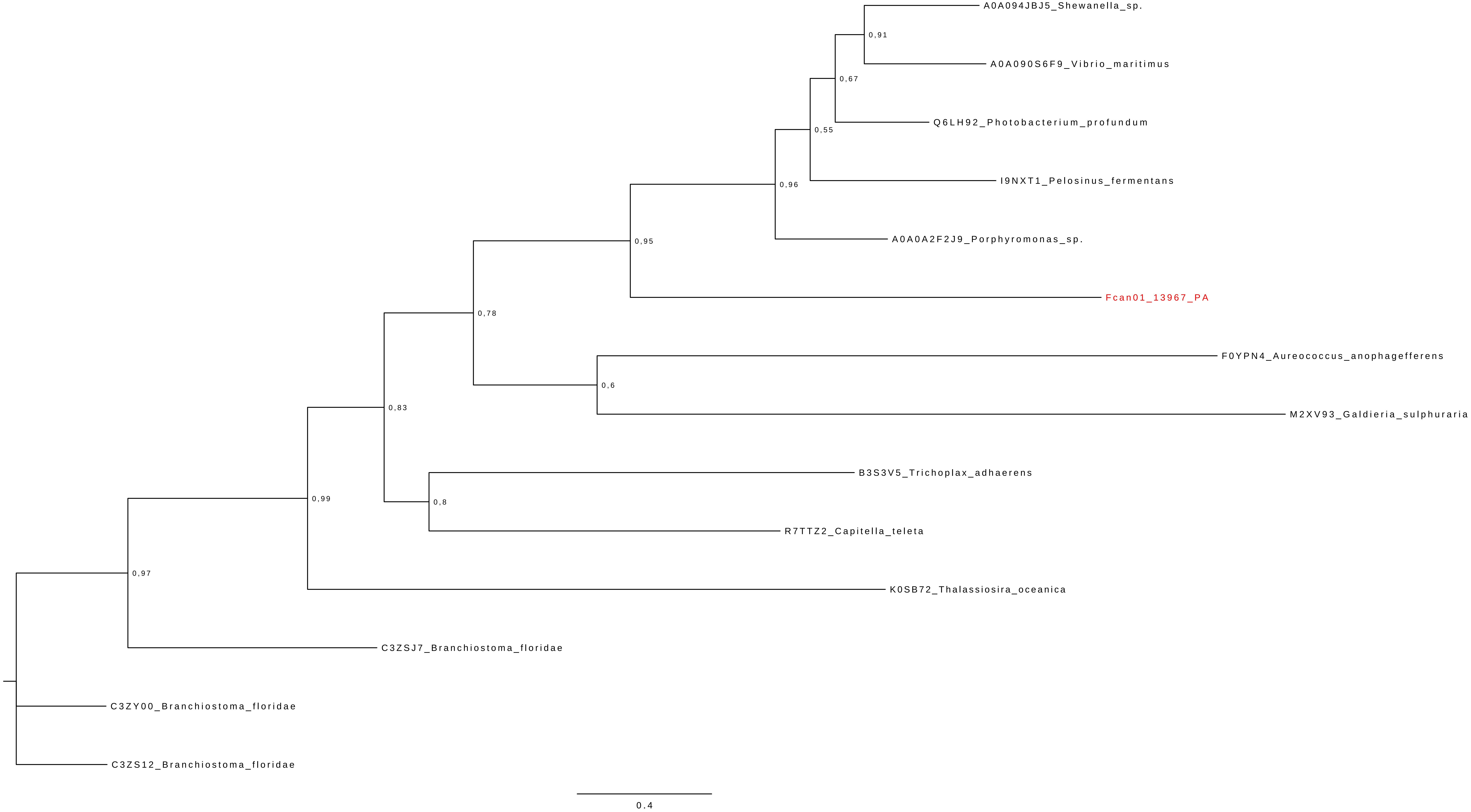
A0A094JBJ5_Shewanella_sp.
0,91
A0A090S6F9_Vibrio_maritimus
0,67
Q6LH92_Photobacterium_profundum
0,55
I9NXT1_Pelosinus_fermentans
0,96
A0A0A2F2J9_Porphyromonas_sp.
0,95
Fcan01_13967_PA
0,78
F0YPN4_Aureococcus_anophagefferens
0,6
M2XV93_Galdieria_sulphuraria
0,83
B3S3V5_Trichoplax_adhaerens
0,99
0,8
R7TTZ2_Capitella_teleta
0,97
K0SB72_Thalassiosira_oceanica
C3ZSJ7_Branchiostoma_floridae
C3ZY00_Branchiostoma_floridae
C3ZS12_Branchiostoma_floridae
0.4

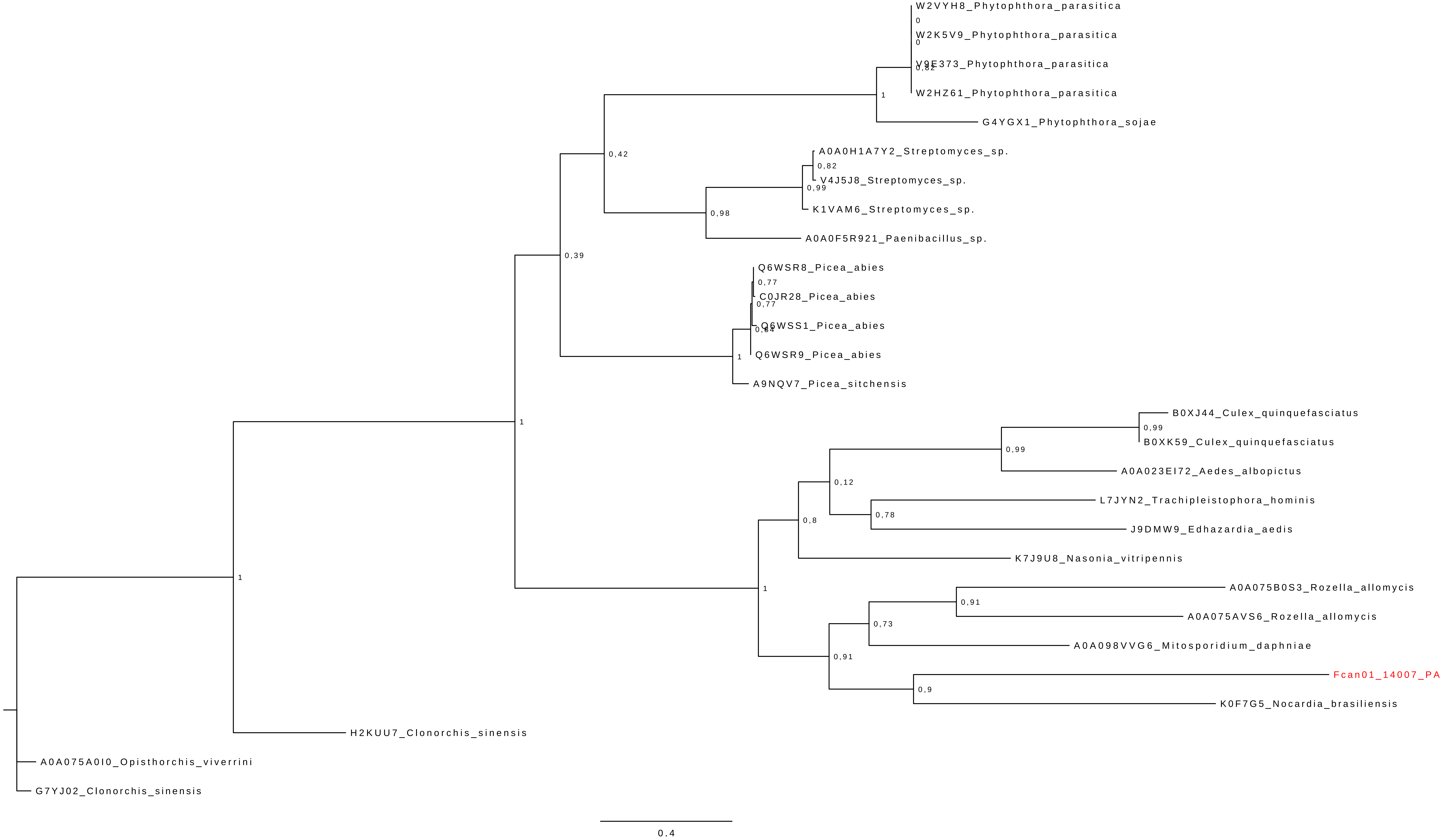
W2VYH8_Phytophthora_parasitica
0
W2K5V9_Phytophthora_parasitica
0
V9E373_Phytophthora_parasitica
0,82
W2HZ61_Phytophthora_parasitica
1
G4YGX1_Phytophthora_sojae
0,42
A0A0H1A7Y2_Streptomyces_sp.
0,82
V4J5J8_Streptomyces_sp.
0,99
K1VAM6_Streptomyces_sp.
0,98
A0A0F5R921_Paenibacillus_sp.
0,39
Q6WSR8_Picea_abies
0,77
C0JR28_Picea_abies
0,77
Q6WSS1_Picea_abies
0,84
Q6WSR9_Picea_abies
1
A9NQV7_Picea_sitchensis
1
B0XJ44_Culex_quinquefasciatus
0,99
B0XK59_Culex_quinquefasciatus
0,99
A0A023EI72_Aedes_albopictus
0,12
L7JYN2_Trachipleistophora_hominis
0,78
J9DMW9_Edhazardia_aedis
0,8
K7J9U8_Nasonia_vitripennis
1
A0A075B0S3_Rozella_allomycis
0,91
A0A075AVS6_Rozella_allomycis
0,73
A0A098VVG6_Mitosporidium_daphniae
0,91
Fcan01_14007_PA
0,9
K0F7G5_Nocardia_brasiliensis
1
H2KUU7_Clonorchis_sinensis
A0A075A0I0_Opisthorchis_viverrini
G7YJ02_Clonorchis_sinensis
0.4

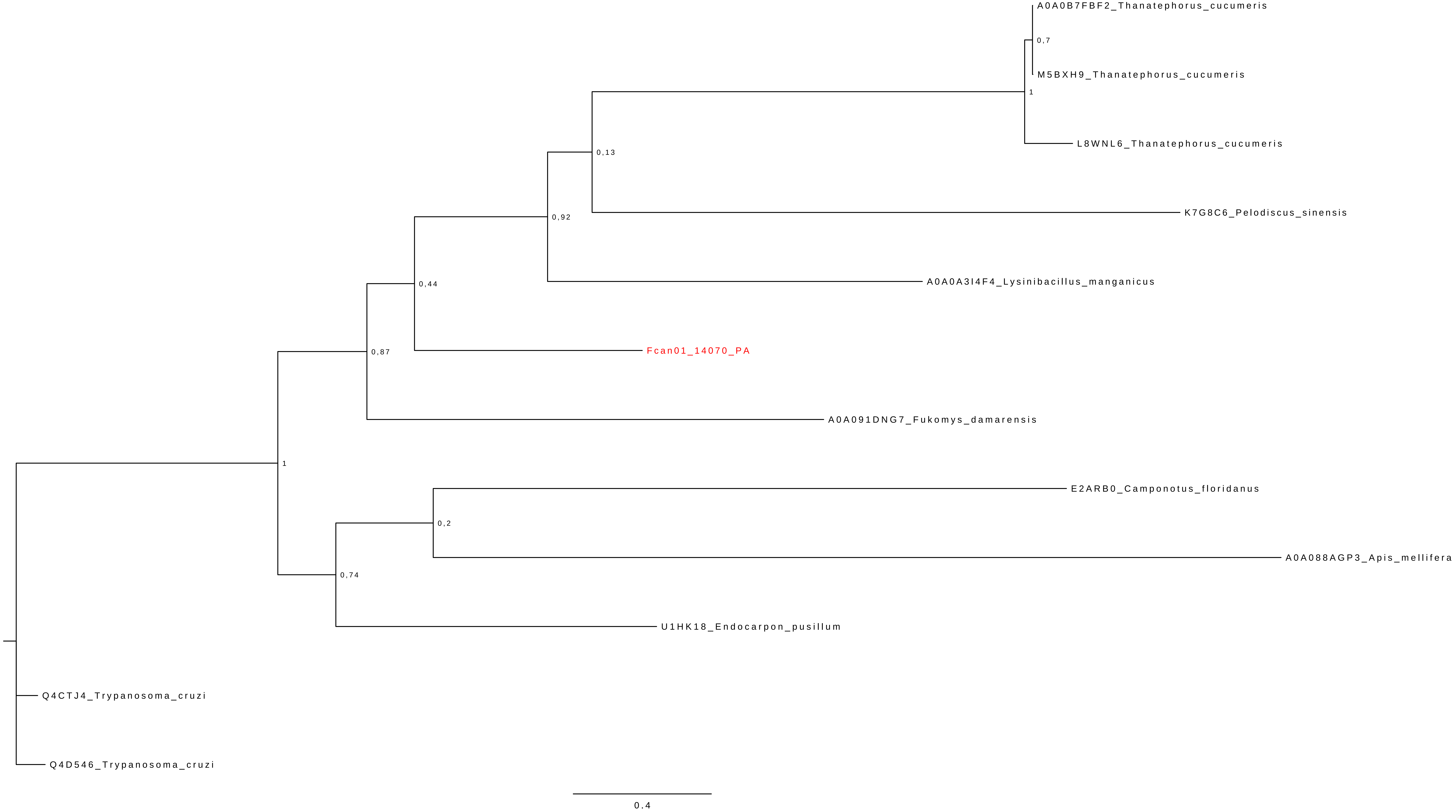
A0A0B7FBF2_Thanatephorus_cucumeris
0,7
M5BXH9_Thanatephorus_cucumeris
1
L8WNL6_Thanatephorus_cucumeris
0,13
K7G8C6_Pelodiscus_sinensis
0,92
A0A0A3I4F4_Lysinibacillus_manganicus
0,44
Fcan01_14070_PA
0,87
A0A091DNG7_Fukomys_damarensis
1
E2ARB0_Camponotus_floridanus
0,2
A0A088AGP3_Apis_mellifera
0,74
U1HK18_Endocarpon_pusillum
Q4CTJ4_Trypanosoma_cruzi
Q4D546_Trypanosoma_cruzi
0.4

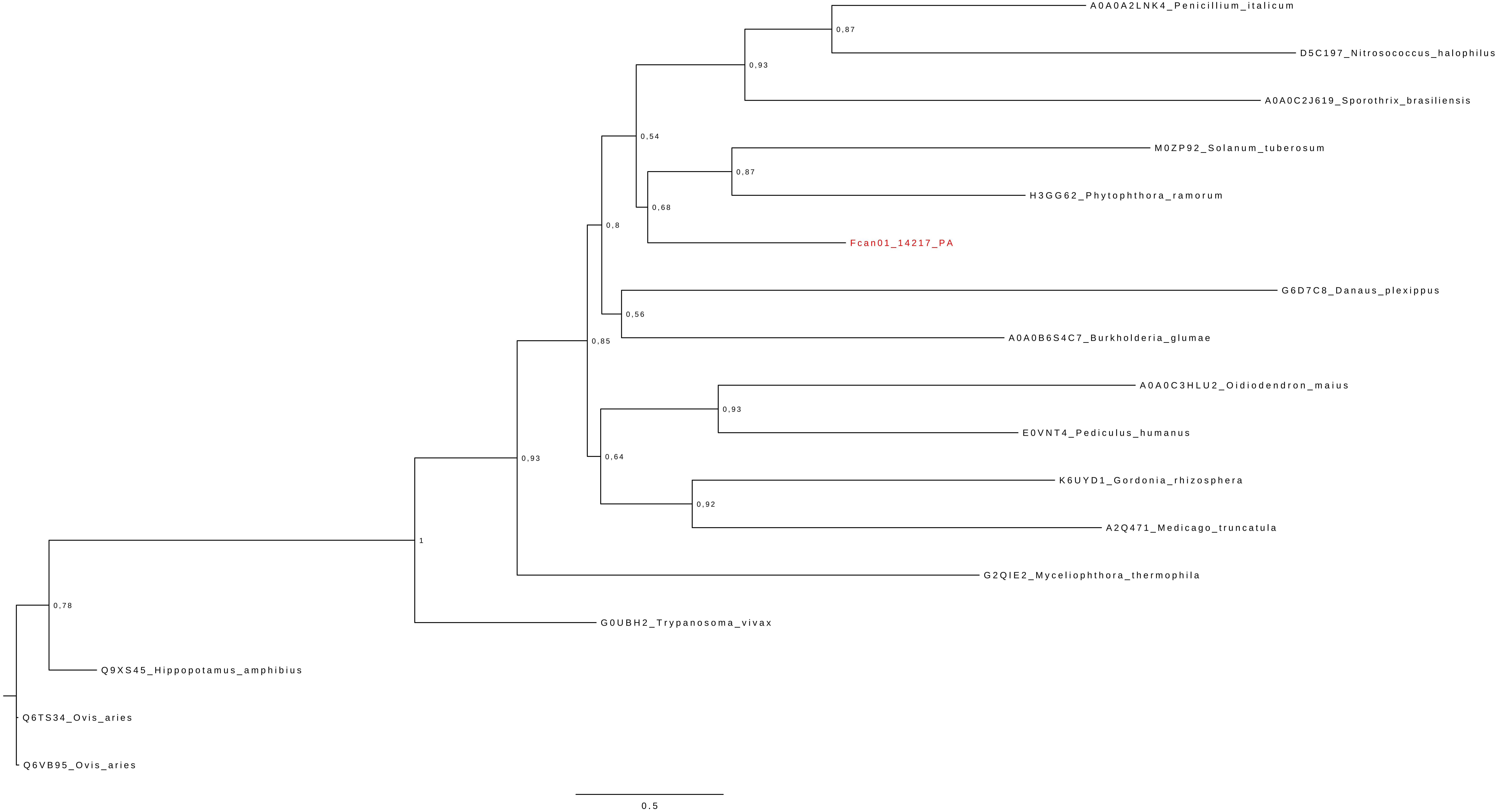
A0A0A2LNK4_Penicillium_italicum
0,87
D5C197_Nitrosococcus_halophilus
0,93
A0A0C2J619_Sporothrix_brasiliensis
0,54
M0ZP92_Solanum_tuberosum
0,87
H3GG62_Phytophthora_ramorum
0,68
0,8
Fcan01_14217_PA
G6D7C8_Danaus_plexippus
0,56
A0A0B6S4C7_Burkholderia_glumae
0,85
A0A0C3HLU2_Oidiodendron_maius
0,93
E0VNT4_Pediculus_humanus
0,64
K6UYD1_Gordonia_rhizosphera
0,92
A2Q471_Medicago_truncatula
0,93
G2QIE2_Myceliophthora_thermophila
1
G0UBH2_Trypanosoma_vivax
0,78
Q9XS45_Hippopotamus_amphibius
Q6TS34_Ovis_aries
Q6VB95_Ovis_aries
0.5

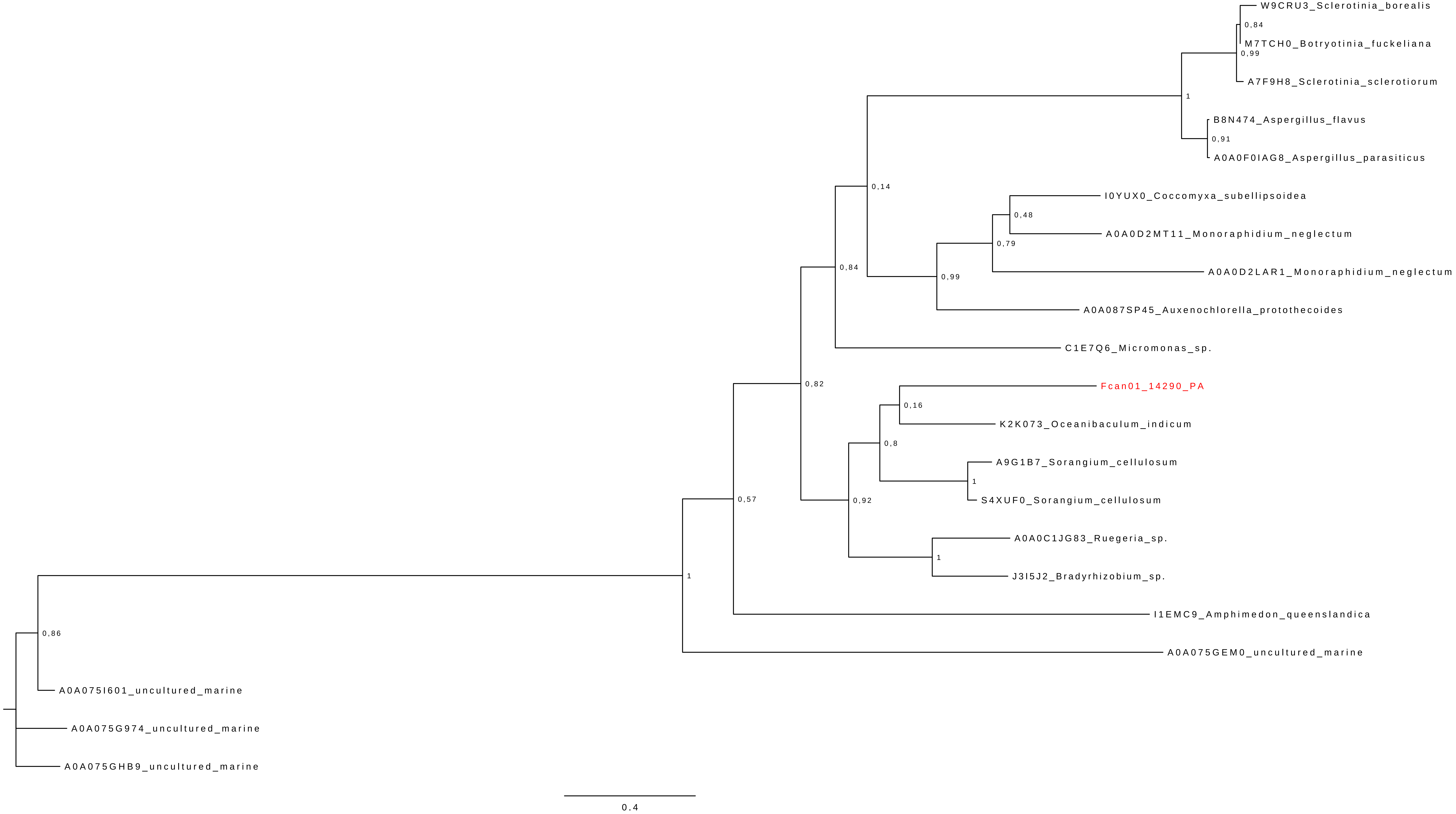
W9CRU3_Sclerotinia_borealis
0,84
M7TCH0_Botryotinia_fuckeliana
0,99
A7F9H8_Sclerotinia_sclerotiorum
1
B8N474_Aspergillus_flavus
0,91
A0A0F0IAG8_Aspergillus_parasiticus
0,14
I0YUX0_Coccomyxa_subellipsoidea
0,48
A0A0D2MT11_Monoraphidium_neglectum
0,79
A0A0D2LAR1_Monoraphidium_neglectum
0,84
0,99
A0A087SP45_Auxenochlorella_protothecoides
C1E7Q6_Micromonas_sp.
0,82
Fcan01_14290_PA
0,16
K2K073_Oceanibaculum_indicum
0,8
A9G1B7_Sorangium_cellulosum
1
0,57
0,92
S4XUF0_Sorangium_cellulosum
A0A0C1JG83_Ruegeria_sp.
1
1
J3I5J2_Bradyrhizobium_sp.
I1EMC9_Amphimedon_queenslandica
A0A075GEM0_uncultured_marine
0,86
A0A075I601_uncultured_marine
A0A075G974_uncultured_marine
A0A075GHB9_uncultured_marine
0.4

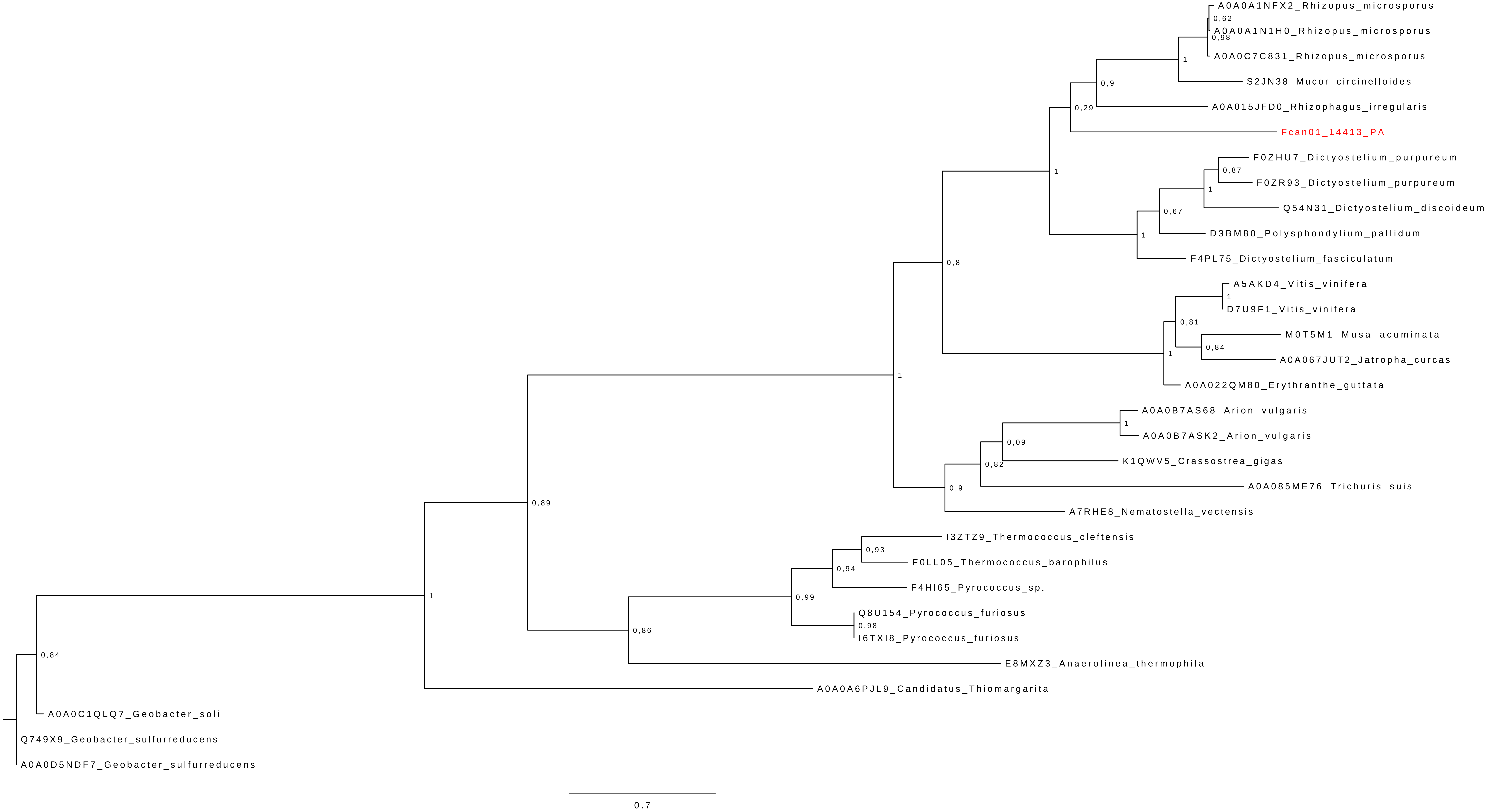

A0A0A1NFX2_Rhizopus_microsporus
0,62
A0A0A1N1H0_Rhizopus_microsporus
0,98
1
A0A0C7C831_Rhizopus_microsporus
S2JN38_Mucor_circinelloides
0,9
0,29
A0A015JFD0_Rhizophagus_irregularis
Fcan01_14413_PA
F0ZHU7_Dictyostelium_purpureum
0,87
F0ZR93_Dictyostelium_purpureum
1
1
Q54N31_Dictyostelium_discoideum
0,67
D3BM80_Polysphondylium_pallidum
1
F4PL75_Dictyostelium_fasciculatum
0,8
A5AKD4_Vitis_vinifera
1
D7U9F1_Vitis_vinifera
0,81
M0T5M1_Musa_acuminata
0,84
A0A067JUT2_Jatropha_curcas
1
A0A022QM80_Erythranthe_guttata
1
A0A0B7AS68_Arion_vulgaris
1
A0A0B7ASK2_Arion_vulgaris
0,09
K1QWV5_Crassostrea_gigas
0,82
A0A085ME76_Trichuris_suis
0,9
A7RHE8_Nematostella_vectensis
0,89
I3ZTZ9_Thermococcus_cleftensis
0,93
F0LL05_Thermococcus_barophilus
0,94
F4HI65_Pyrococcus_sp.
0,99
Q8U154_Pyrococcus_furiosus
0,98
I6TXI8_Pyrococcus_furiosus
0,86
E8MXZ3_Anaerolinea_thermophila
1
A0A0A6PJL9_Candidatus_Thiomargarita
0,84
A0A0C1QLQ7_Geobacter_soli
Q749X9_Geobacter_sulfurreducens
A0A0D5NDF7_Geobacter_sulfurreducens
0.7

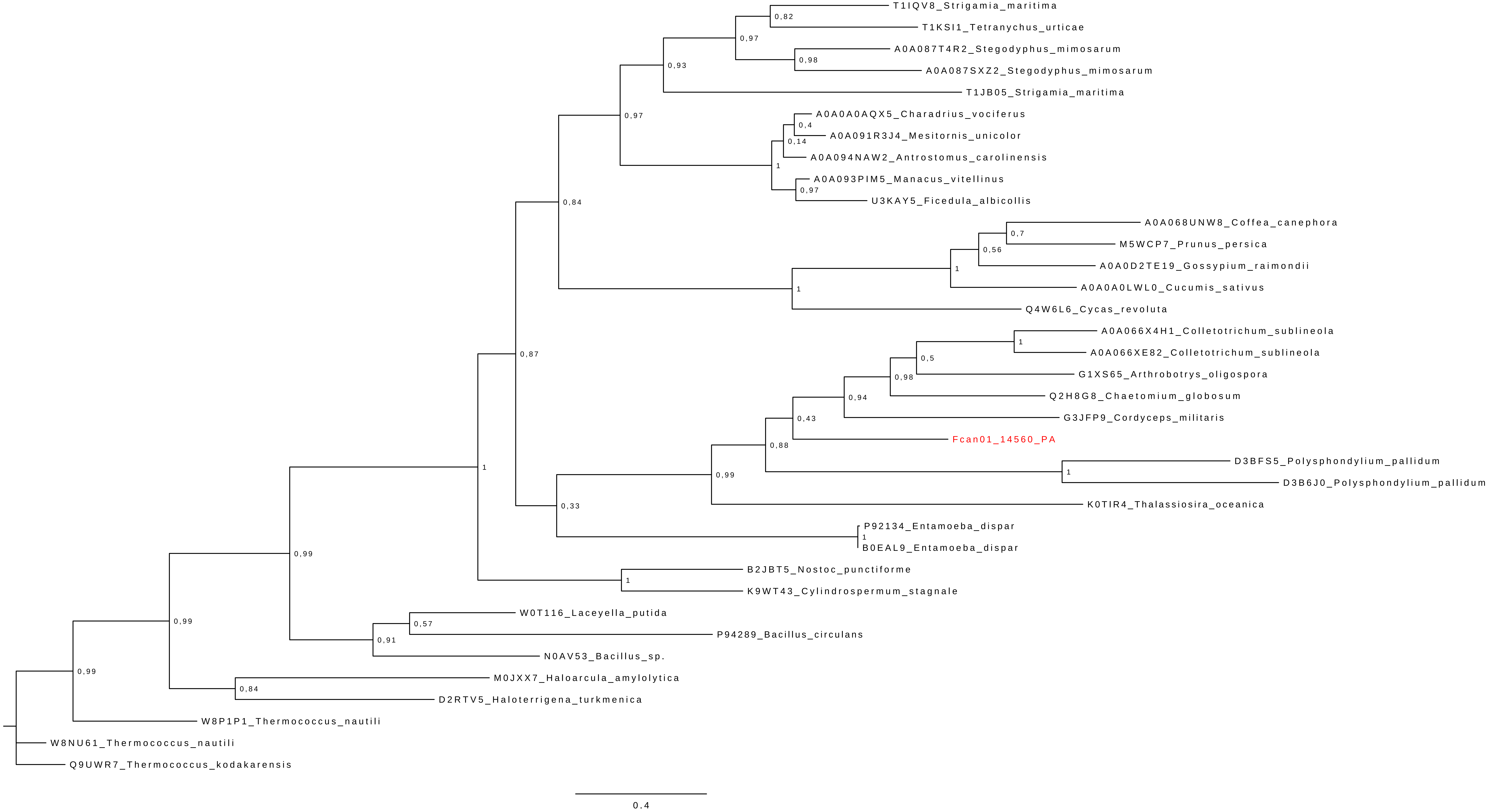
T1IQV8_Strigamia_maritima
T1KSI1_Tetranychus_urticae
A0A087T4R2_Stegodyphus_mimosarum
A0A087SXZ2_Stegodyphus_mimosarum
T1JB05_Strigamia_maritima
A0A0A0AQX5_Charadrius_vociferus
A0A091R3J4_Mesitornis_unicolor
A0A094NAW2_Antrostomus_carolinensis
A0A093PIM5_Manacus_vitellinus
U3KAY5_Ficedula_albicollis
A0A068UNW8_Coffea_canephora
M5WCP7_Prunus_persica
A0A0D2TE19_Gossypium_raimondii
A0A0A0LWL0_Cucumis_sativus
Q4W6L6_Cycas_revoluta
A0A066X4H1_Colletotrichum_sublineola
A0A066XE82_Colletotrichum_sublineola
G1XS65_Arthrobotrys_oligospora
Q2H8G8_Chaetomium_globosum
G3JFP9_Cordyceps_militaris
Fcan01_14560_PA
D3BFS5_Polysphondylium_pallidum
D3B6J0_Polysphondylium_pallidum
K0TIR4_Thalassiosira_oceanica
P92134_Entamoeba_dispar
B0EAL9_Entamoeba_dispar
B2JBT5_Nostoc_punctiforme
K9WT43_Cylindrospermum_stagnale
W0T116_Laceyella_putida
P94289_Bacillus_circulans
N0AV53_Bacillus_sp.
M0JXX7_Haloarcula_amylolytica
D2RTV5_Haloterrigena_turkmenica
W8P1P1_Thermococcus_nautili
W8NU61_Thermococcus_nautili
Q9UWR7_Thermococcus_kodakarensis
0.4

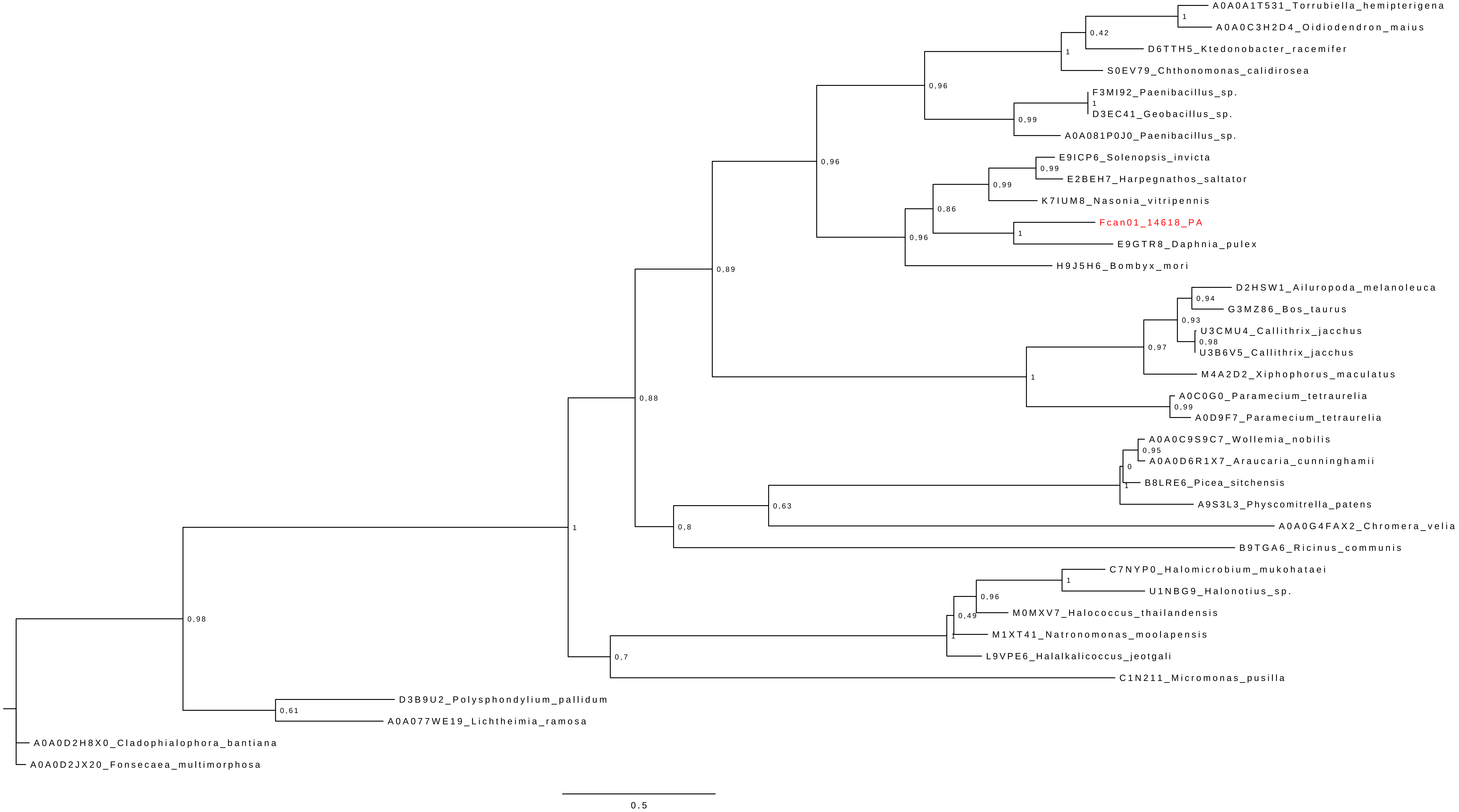
A0A0A1T531_Torrubiella_hemipterigena
A0A0C3H2D4_Oidiodendron_maius
D6TTH5_Ktedonobacter_racemifer
S0EV79_Chthonomonas_calidirosea
F3MI92_Paenibacillus_sp.
D3EC41_Geobacillus_sp.
A0A081P0J0_Paenibacillus_sp.
E9ICP6_Solenopsis_invicta
E2BEH7_Harpegnathos_saltator
K7IUM8_Nasonia_vitripennis
Fcan01_14618_PA
E9GTR8_Daphnia_pulex
H9J5H6_Bombyx_mori
D2HSW1_Ailuropoda_melanoleuca
G3MZ86_Bos_taurus
U3CMU4_Callithrix_jacchus
U3B6V5_Callithrix_jacchus
M4A2D2_Xiphophorus_maculatus
A0C0G0_Paramecium_tetraurelia
A0D9F7_Paramecium_tetraurelia
A0A0C9S9C7_Wollemia_nobilis
A0A0D6R1X7_Araucaria_cunninghamii
B8LRE6_Picea_sitchensis
A9S3L3_Physcomitrella_patens
A0A0G4FAX2_Chromera_velia
B9TGA6_Ricinus_communis
C7NYP0_Halomicrobium_mukohataei
U1NBG9_Halonotius_sp.
M0MXV7_Halococcus_thailandensis
M1XT41_Natronomonas_moolapensis
L9VPE6_Halalkalicoccus_jeotgali
C1N211_Micromonas_pusilla
D3B9U2_Polysphondylium_pallidum
A0A077WE19_Lichtheimia_ramosa
A0A0D2H8X0_Cladophialophora_bantiana
A0A0D2JX20_Fonsecaea_multimorphosa
0.5

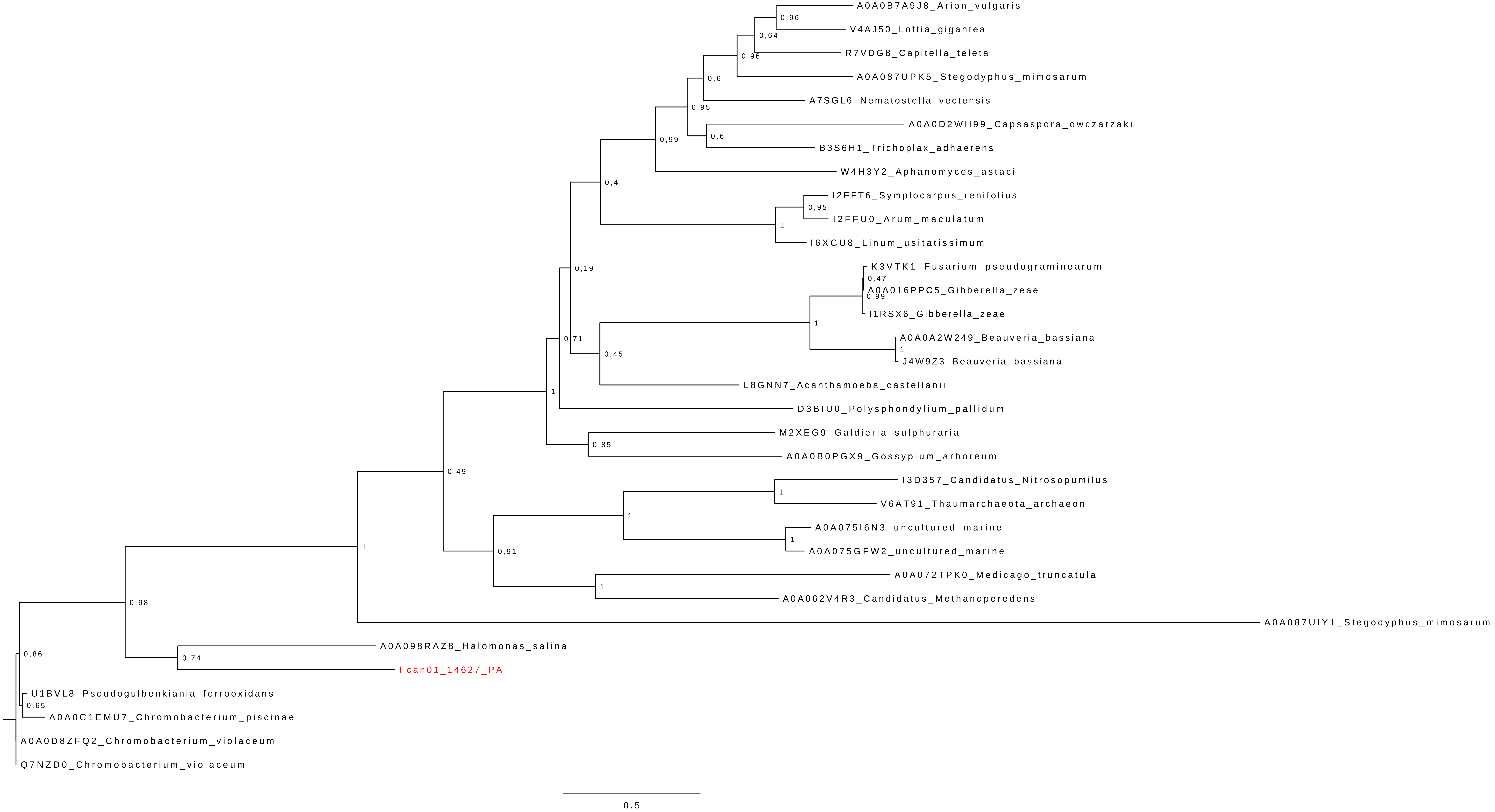
A0A0B7A9J8_Arion_vulgaris
V4AJ50_Lottia_gigantea
R7VDG8_Capitella_teleta
A0A087UPK5_Stegodyphus_mimosarum
A7SGL6_Nematostella_vectensis
A0A0D2WH99_Capsaspora_owczarzaki
B3S6H1_Trichoplax_adhaerens
W4H3Y2_Aphanomyces_astaci
I2FFT6_Symplocarpus_renifolius
I2FFU0_Arum_maculatum
I6XCU8_Linum_usitatissimum
K3VTK1_Fusarium_pseudograminearum
A0A016PPC5_Gibberella_zeae
I1RSX6_Gibberella_zeae
A0A0A2W249_Beauveria_bassiana
J4W9Z3_Beauveria_bassiana
L8GNN7_Acanthamoeba_castellanii
D3BIU0_Polysphondylium_pallidum
M2XEG9_Galdieria_sulphuraria
A0A0B0PGX9_Gossypium_arboreum
I3D357_Candidatus_Nitrosopumilus
V6AT91_Thaumarchaeota_archaeon
A0A075I6N3_uncultured_marine
A0A075GFW2_uncultured_marine
A0A072TPK0_Medicago_truncatula
A0A062V4R3_Candidatus_Methanoperedens
A0A087UIY1_Stegodyphus_mimosarum
A0A098RAZ8_Halomonas_salina
Fcan01_14627_PA
U1BVL8_Pseudogulbenkiania_ferrooxidans
A0A0C1EMU7_Chromobacterium_piscinae
A0A0D8ZFQ2_Chromobacterium_violaceum
Q7NZD0_Chromobacterium_violaceum
0,96
0,64
0,96
0,6
0,95
0,6
0,99
0,4
0,95
1
0,19
0,47
0,99
1
1
0,71
0,45
1
0,85
0,49
1
1
1
0,91
1
1
0,98
0,86
0,74
0,65
0.5

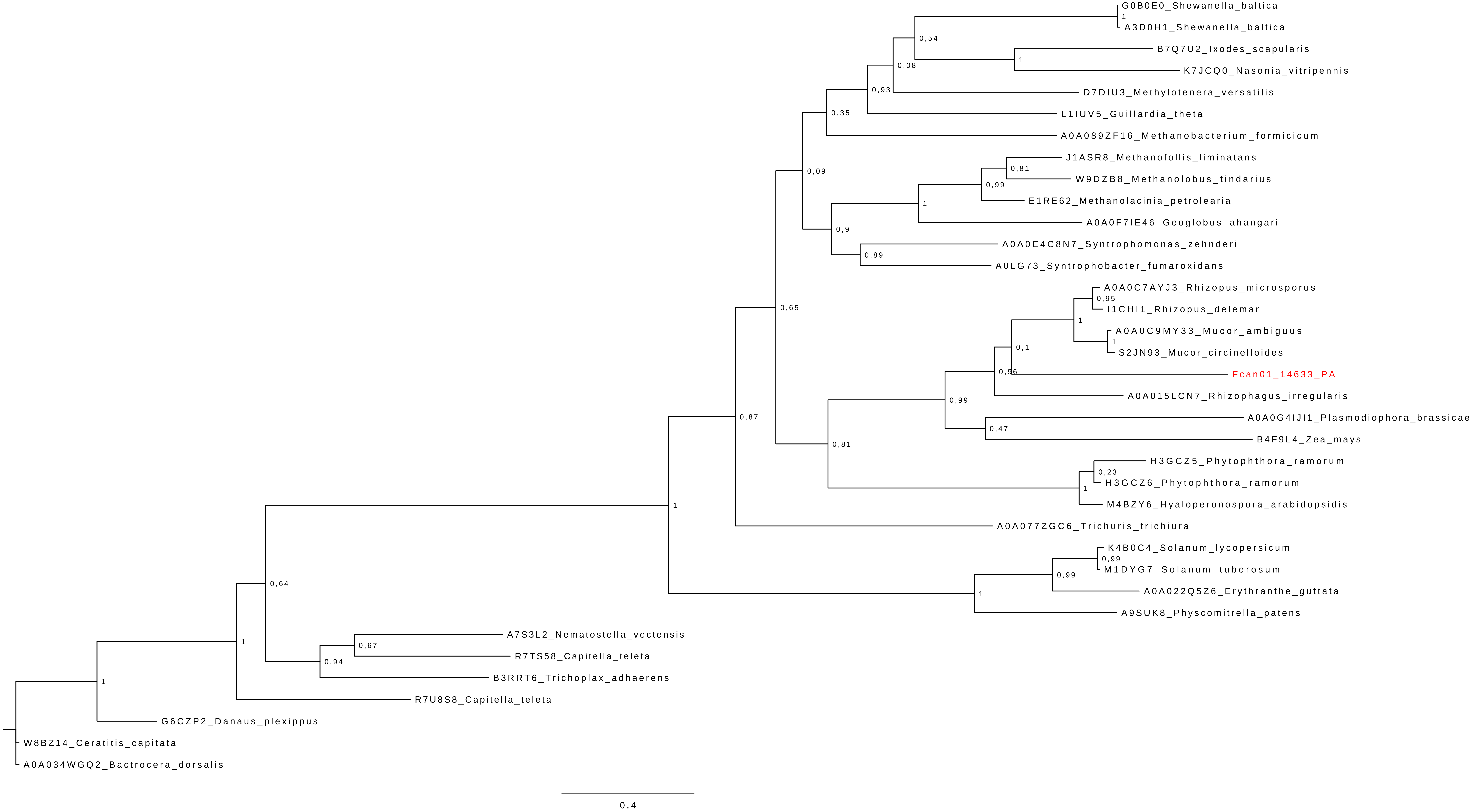
G0B0E0_Shewanella_baltica
A3D0H1_Shewanella_baltica
B7Q7U2_Ixodes_scapularis
K7JCQ0_Nasonia_vitripennis
D7DIU3_Methylotenera_versatilis
L1IUV5_Guillardia_theta
A0A089ZF16_Methanobacterium_formicicum
J1ASR8_Methanofollis_liminatans
W9DZB8_Methanolobus_tindarius
E1RE62_Methanolacinia_petrolearia
A0A0F7IE46_Geoglobus_ahangari
A0A0E4C8N7_Syntrophomonas_zehnderi
A0LG73_Syntrophobacter_fumaroxidans
A0A0C7AYJ3_Rhizopus_microsporus
I1CHI1_Rhizopus_delemar
A0A0C9MY33_Mucor_ambiguus
S2JN93_Mucor_circinelloides
Fcan01_14633_PA
A0A015LCN7_Rhizophagus_irregularis
A0A0G4IJI1_Plasmodiophora_brassicae
B4F9L4_Zea_mays
H3GCZ5_Phytophthora_ramorum
H3GCZ6_Phytophthora_ramorum
M4BZY6_Hyaloperonospora_arabidopsidis
A0A077ZGC6_Trichuris_trichiura
K4B0C4_Solanum_lycopersicum
M1DYG7_Solanum_tuberosum
A0A022Q5Z6_Erythranthe_guttata
A9SUK8_Physcomitrella_patens
A7S3L2_Nematostella_vectensis
R7TS58_Capitella_teleta
B3RRT6_Trichoplax_adhaerens
R7U8S8_Capitella_teleta
G6CZP2_Danaus_plexippus
W8BZ14_Ceratitis_capitata
A0A034WGQ2_Bactrocera_dorsalis
1
0,54
0,08
0,93
0,35
0,09
0,81
0,99
0,9
0,89
0,65
0,95
0,1
0,96
0,99
0,47
0,87
0,81
0,23
1
0,99
0,64
0,67
0,94
0.4

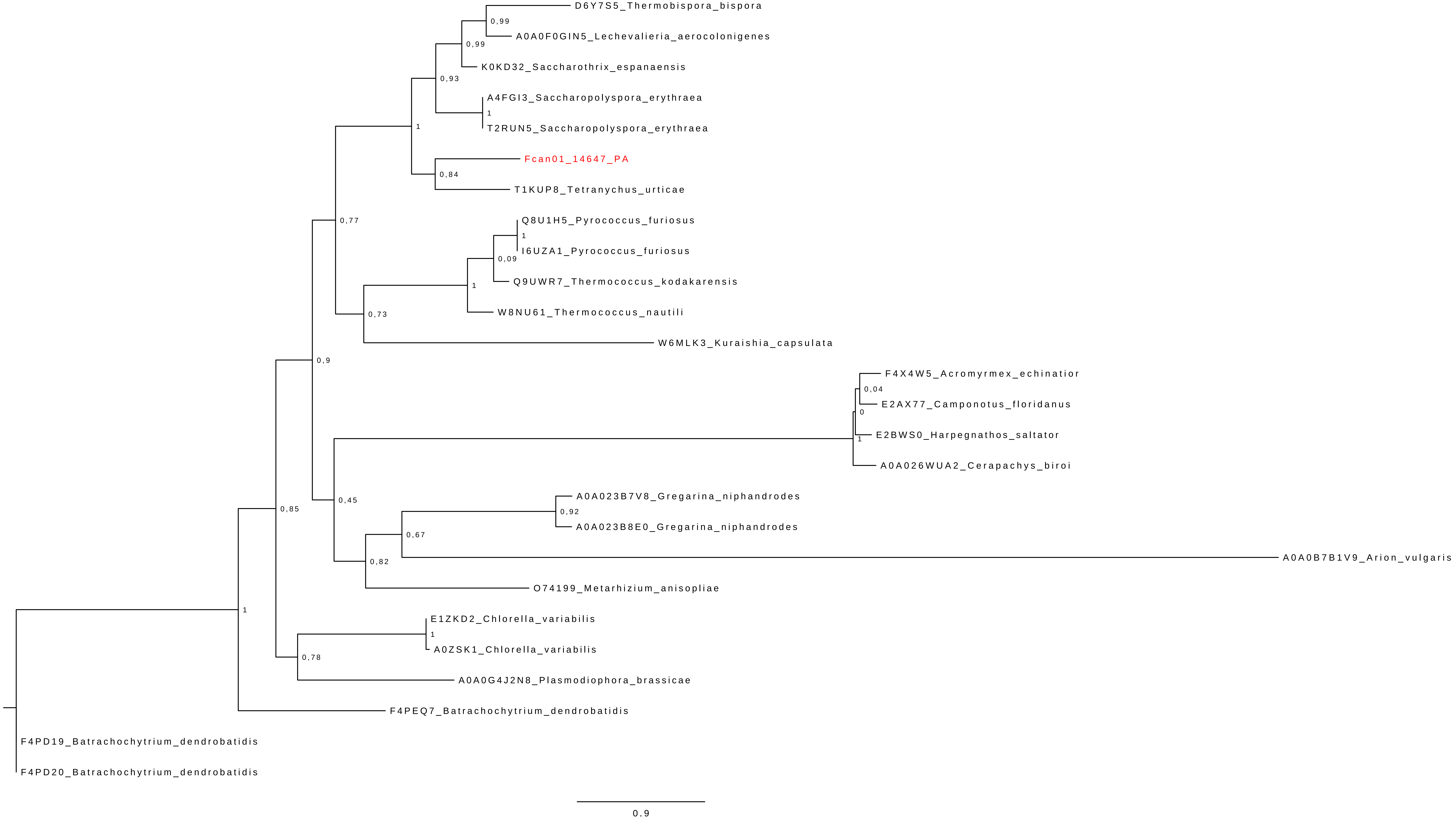
D6Y7S5_Thermobispora_bispora
0,99
A0A0F0GIN5_Lechevalieria_aerocolonigenes
0,99
K0KD32_Saccharothrix_espanaensis
0,93
A4FGI3_Saccharopolyspora_erythraea
1
T2RUN5_Saccharopolyspora_erythraea
1
Fcan01_14647_PA
0,84
T1KUP8_Tetranychus_urticae
0,77
Q8U1H5_Pyrococcus_furiosus
1
I6UZA1_Pyrococcus_furiosus
0,09
Q9UWR7_Thermococcus_kodakarensis
1
W8NU61_Thermococcus_nautili
0,73
W6MLK3_Kuraishia_capsulata
0,9
F4X4W5_Acromyrmex_echinatior
0,04
E2AX77_Camponotus_floridanus
0
E2BWS0_Harpegnathos_saltator
1
A0A026WUA2_Cerapachys_biroi
A0A023B7V8_Gregarina_niphandrodes
0,92
A0A023B8E0_Gregarina_niphandrodes
0,85
0,45
0,67
A0A0B7B1V9_Arion_vulgaris
0,82
O74199_Metarhizium_anisopliae
1
E1ZKD2_Chlorella_variabilis
1
A0ZSK1_Chlorella_variabilis
0,78
A0A0G4J2N8_Plasmodiophora_brassicae
F4PEQ7_Batrachochytrium_dendrobatidis
F4PD19_Batrachochytrium_dendrobatidis
F4PD20_Batrachochytrium_dendrobatidis
0.9

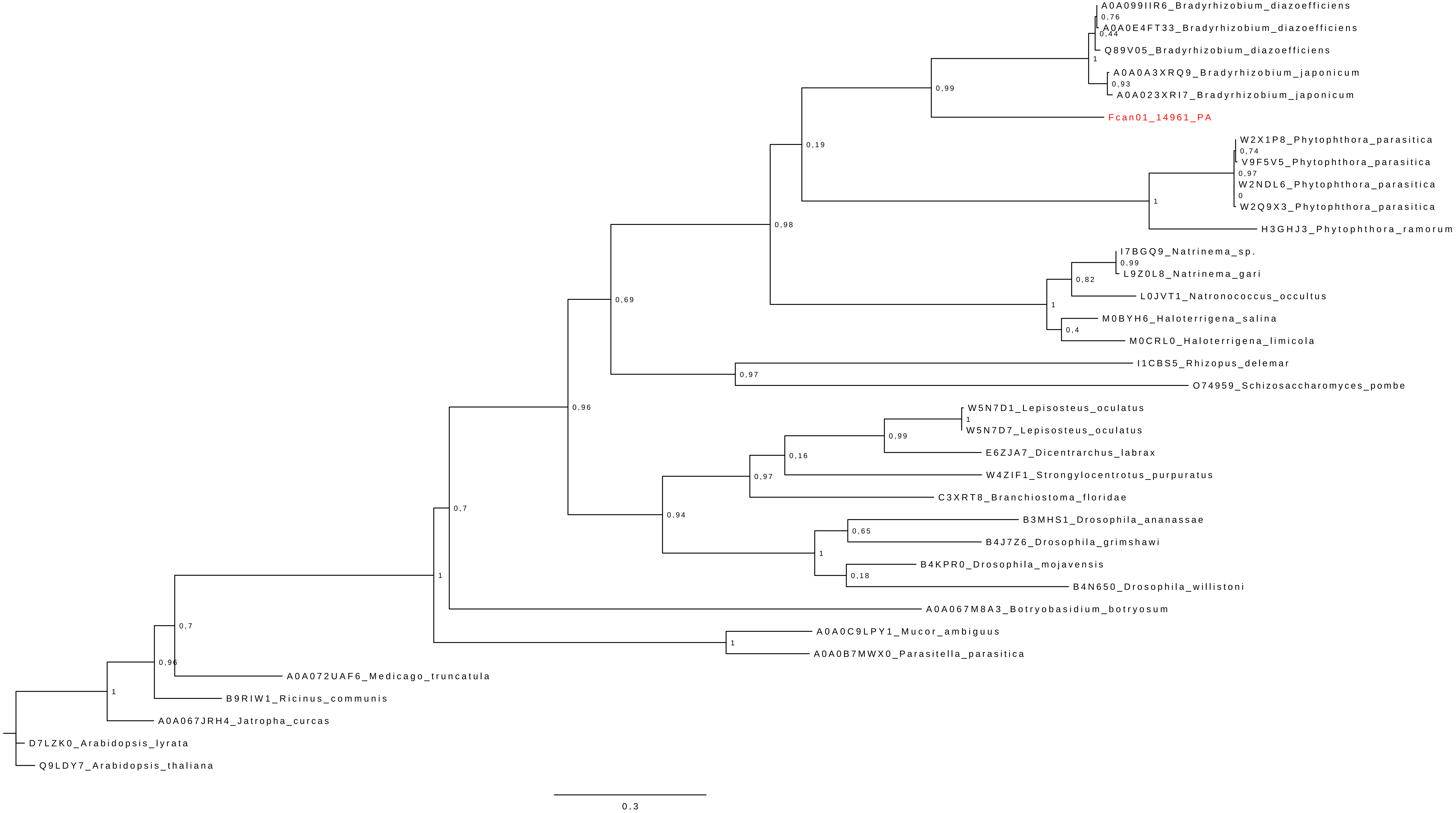
A0A099IIR6_Bradyrhizobium_diazoefficiens
0,76
A0A0E4FT33_Bradyrhizobium_diazoefficiens
0,44
Q89V05_Bradyrhizobium_diazoefficiens
1
A0A0A3XRQ9_Bradyrhizobium_japonicum
0,93
A0A023XRI7_Bradyrhizobium_japonicum
0,99
Fcan01_14961_PA
0,19
W2X1P8_Phytophthora_parasitica
0,74
V9F5V5_Phytophthora_parasitica
0,97
W2NDL6_Phytophthora_parasitica
0
W2Q9X3_Phytophthora_parasitica
1
H3GHJ3_Phytophthora_ramorum
0,98
I7BGQ9_Natrinema_sp.
0,99
L9Z0L8_Natrinema_gari
0,82
L0JVT1_Natronococcus_occultus
1
M0BYH6_Haloterrigena_salina
0,4
M0CRL0_Haloterrigena_limicola
0,69
I1CBS5_Rhizopus_delemar
0,97
O74959_Schizosaccharomyces_pombe
0,96
W5N7D1_Lepisosteus_oculatus
1
W5N7D7_Lepisosteus_oculatus
0,99
E6ZJA7_Dicentrarchus_labrax
0,16
W4ZIF1_Strongylocentrotus_purpuratus
0,97
C3XRT8_Branchiostoma_floridae
0,7
0,94
B3MHS1_Drosophila_ananassae
0,65
B4J7Z6_Drosophila_grimshawi
1
B4KPR0_Drosophila_mojavensis
0,18
B4N650_Drosophila_willistoni
1
A0A067M8A3_Botryobasidium_botryosum
A0A0C9LPY1_Mucor_ambiguus
1
A0A0B7MWX0_Parasitella_parasitica
0,7
0,96
A0A072UAF6_Medicago_truncatula
1
B9RIW1_Ricinus_communis
A0A067JRH4_Jatropha_curcas
D7LZK0_Arabidopsis_lyrata
Q9LDY7_Arabidopsis_thaliana
0.3

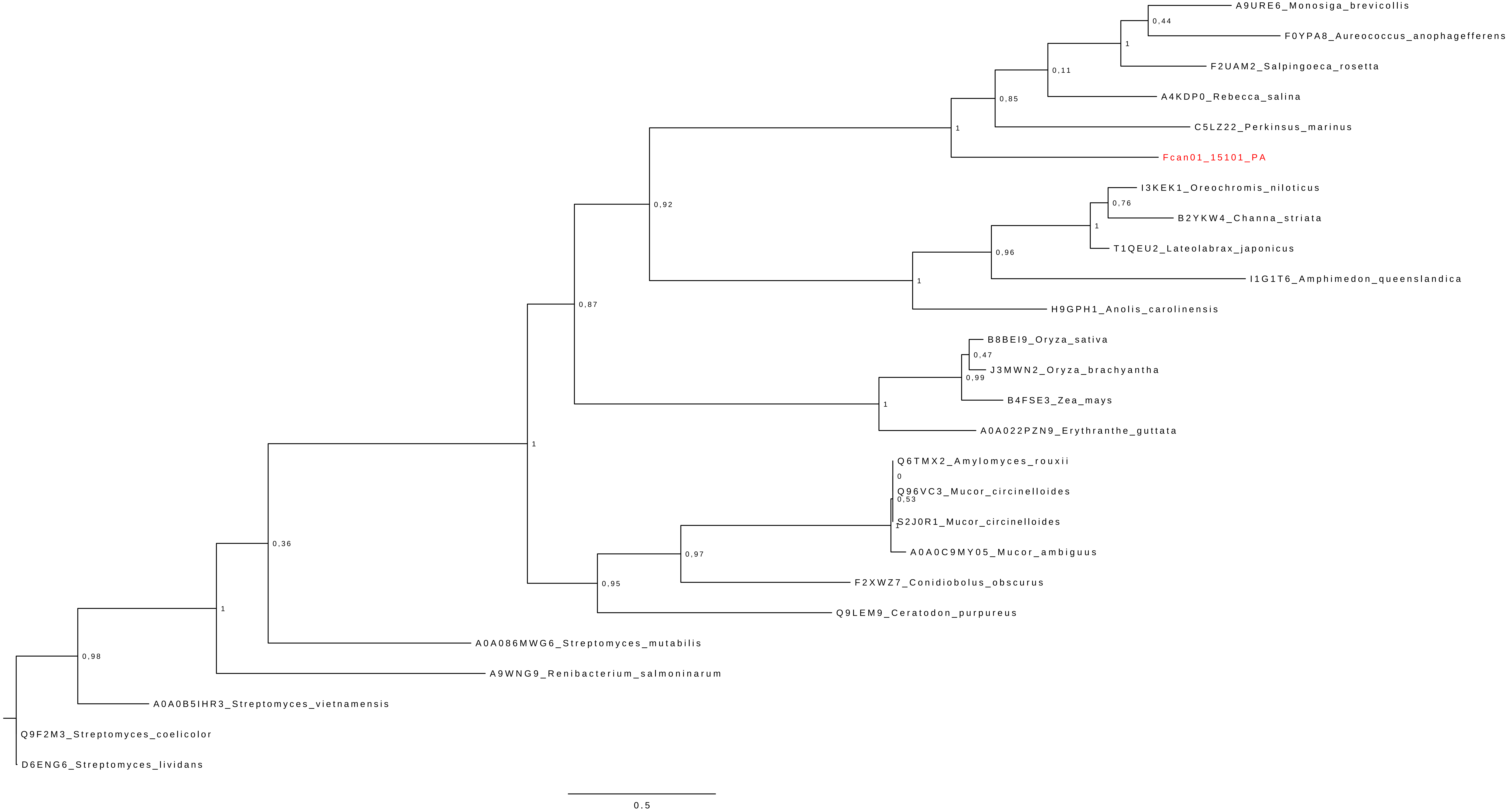
A9URE6_Monosiga_brevicollis
0,44
F0YPA8_Aureococcus_anophagefferens
1
F2UAM2_Salpingoeca_rosetta
0,11
A4KDP0_Rebecca_salina
0,85
C5LZ22_Perkinsus_marinus
1
Fcan01_15101_PA
0,92
I3KEK1_Oreochromis_niloticus
0,76
B2YKW4_Channa_striata
1
T1QEU2_Lateolabrax_japonicus
0,96
I1G1T6_Amphimedon_queenslandica
1
H9GPH1_Anolis_carolinensis
0,87
B8BEI9_Oryza_sativa
0,47
J3MWN2_Oryza_brachyantha
0,99
B4FSE3_Zea_mays
1
A0A022PZN9_Erythranthe_guttata
1
Q6TMX2_Amylomyces_rouxii
0
Q96VC3_Mucor_circinelloides
0,53
S2J0R1_Mucor_circinelloides
1
A0A0C9MY05_Mucor_ambiguus
0,97
F2XWZ7_Conidiobolus_obscurus
0,95
Q9LEM9_Ceratodon_purpureus
0,36
A0A086MWG6_Streptomyces_mutabilis
1
A9WNG9_Renibacterium_salmoninarum
0,98
A0A0B5IHR3_Streptomyces_vietnamensis
Q9F2M3_Streptomyces_coelicolor
D6ENG6_Streptomyces_lividans
0.5

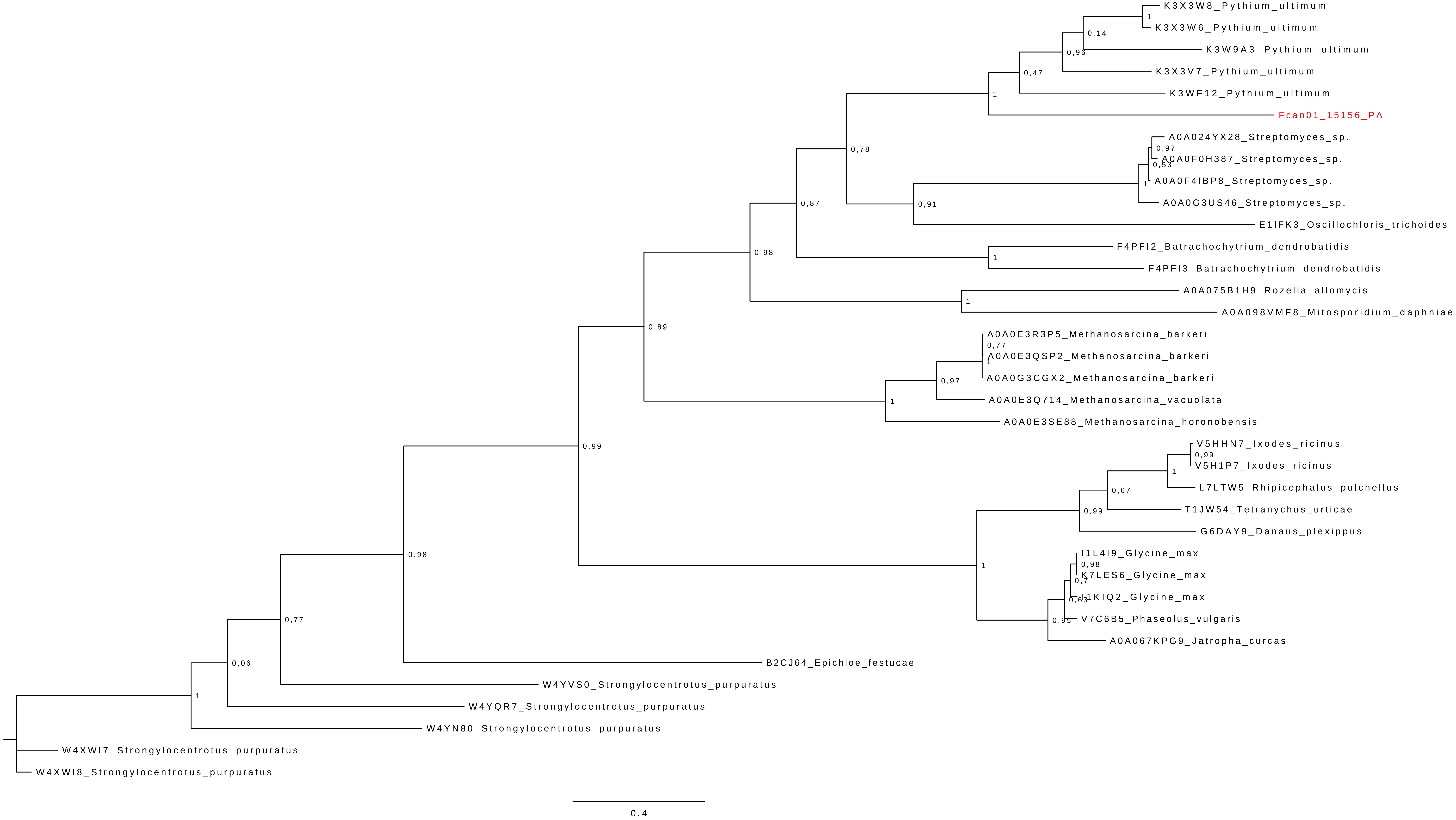
K3X3W8_Pythium_ultimum
K3X3W6_Pythium_ultimum
K3W9A3_Pythium_ultimum
K3X3V7_Pythium_ultimum
K3WF12_Pythium_ultimum
Fcan01_15156_PA
A0A024YX28_Streptomyces_sp.
A0A0F0H387_Streptomyces_sp.
A0A0F4IBP8_Streptomyces_sp.
A0A0G3US46_Streptomyces_sp.
E1IFK3_Oscillochloris_trichoides
F4PFI2_Batrachochytrium_dendrobatidis
F4PFI3_Batrachochytrium_dendrobatidis
A0A075B1H9_Rozella_allomycis
A0A098VMF8_Mitosporidium_daphniae
A0A0E3R3P5_Methanosarcina_barkeri
A0A0E3QSP2_Methanosarcina_barkeri
A0A0G3CGX2_Methanosarcina_barkeri
A0A0E3Q714_Methanosarcina_vacuolata
A0A0E3SE88_Methanosarcina_horonobensis
V5HHN7_Ixodes_ricinus
V5H1P7_Ixodes_ricinus
L7LTW5_Rhipicephalus_pulchellus
T1JW54_Tetranychus_urticae
G6DAY9_Danaus_plexippus
I1L4I9_Glycine_max
K7LES6_Glycine_max
I1KIQ2_Glycine_max
V7C6B5_Phaseolus_vulgaris
A0A067KPG9_Jatropha_curcas
B2CJ64_Epichloe_festucae
W4YVS0_Strongylocentrotus_purpuratus
W4YQR7_Strongylocentrotus_purpuratus
W4YN80_Strongylocentrotus_purpuratus
W4XWI7_Strongylocentrotus_purpuratus
W4XWI8_Strongylocentrotus_purpuratus
0.4

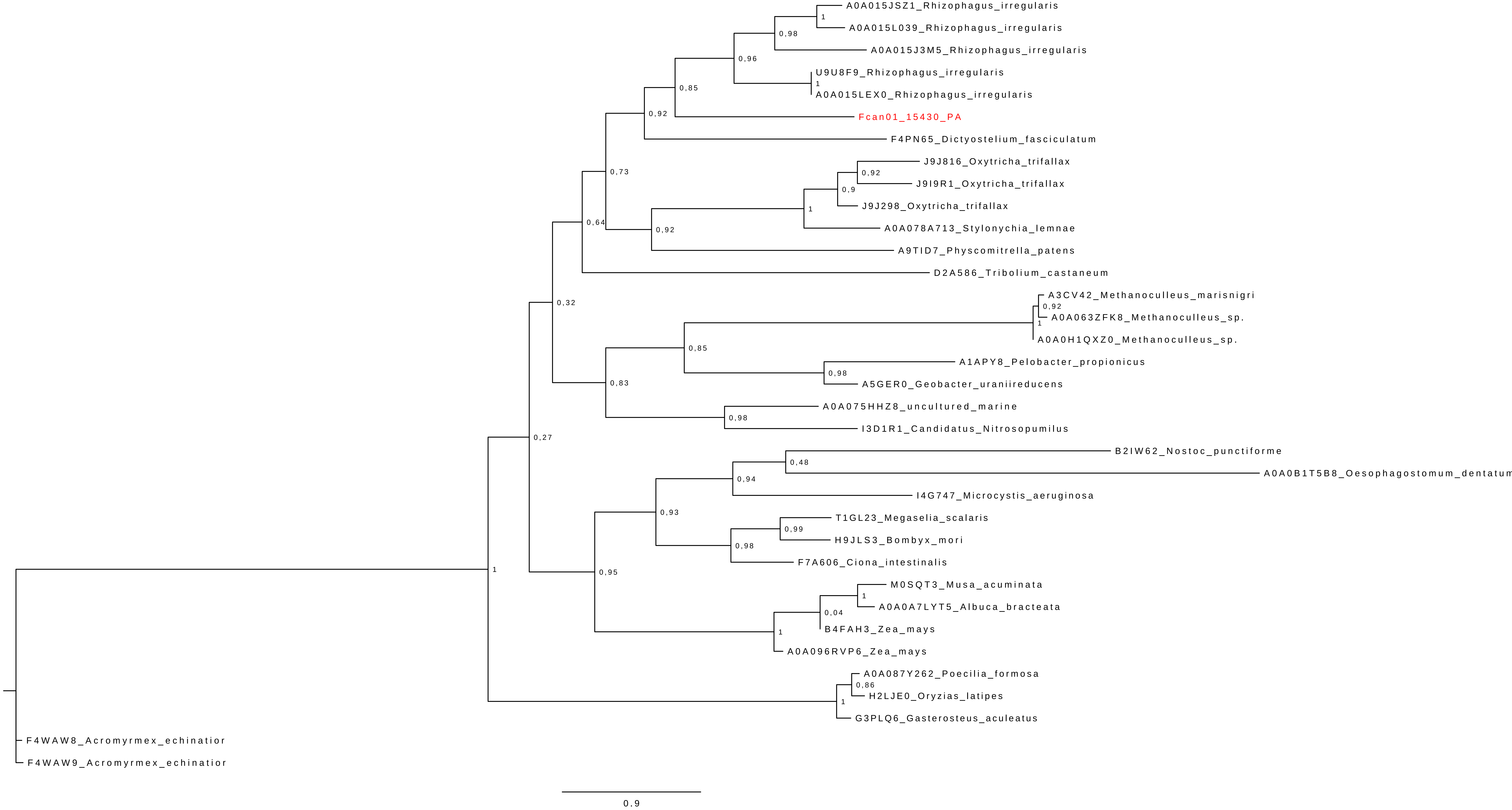
A0A015JSZ1_Rhizophagus_irregularis
1
A0A015L039_Rhizophagus_irregularis
0,98
A0A015J3M5_Rhizophagus_irregularis
0,96
U9U8F9_Rhizophagus_irregularis
1
A0A015LEX0_Rhizophagus_irregularis
0,85
Fcan01_15430_PA
0,92
F4PN65_Dictyostelium_fasciculatum
J9J816_Oxytricha_trifallax
0,92
J9I9R1_Oxytricha_trifallax
0,9
0,73
J9J298_Oxytricha_trifallax
1
0,64
0,92
A0A078A713_Stylonychia_lemnae
A9TID7_Physcomitrella_patens
D2A586_Tribolium_castaneum
A3CV42_Methanoculleus_marisnigri
0,92
A0A063ZFK8_Methanoculleus_sp.
1
A0A0H1QXZ0_Methanoculleus_sp.
0,32
0,85
A1APY8_Pelobacter_propionicus
0,98
A5GER0_Geobacter_uraniireducens
0,83
A0A075HHZ8_uncultured_marine
0,98
I3D1R1_Candidatus_Nitrosopumilus
0,27
B2IW62_Nostoc_punctiforme
0,48
A0A0B1T5B8_Oesophagostomum_dentatum
0,94
I4G747_Microcystis_aeruginosa
0,93
T1GL23_Megaselia_scalaris
0,99
H9JLS3_Bombyx_mori
0,98
F7A606_Ciona_intestinalis
1
0,95
M0SQT3_Musa_acuminata
1
A0A0A7LYT5_Albuca_bracteata
0,04
B4FAH3_Zea_mays
1
A0A096RVP6_Zea_mays
A0A087Y262_Poecilia_formosa
0,86
H2LJE0_Oryzias_latipes
1
G3PLQ6_Gasterosteus_aculeatus
F4WAW8_Acromyrmex_echinatior
F4WAW9_Acromyrmex_echinatior
0.9

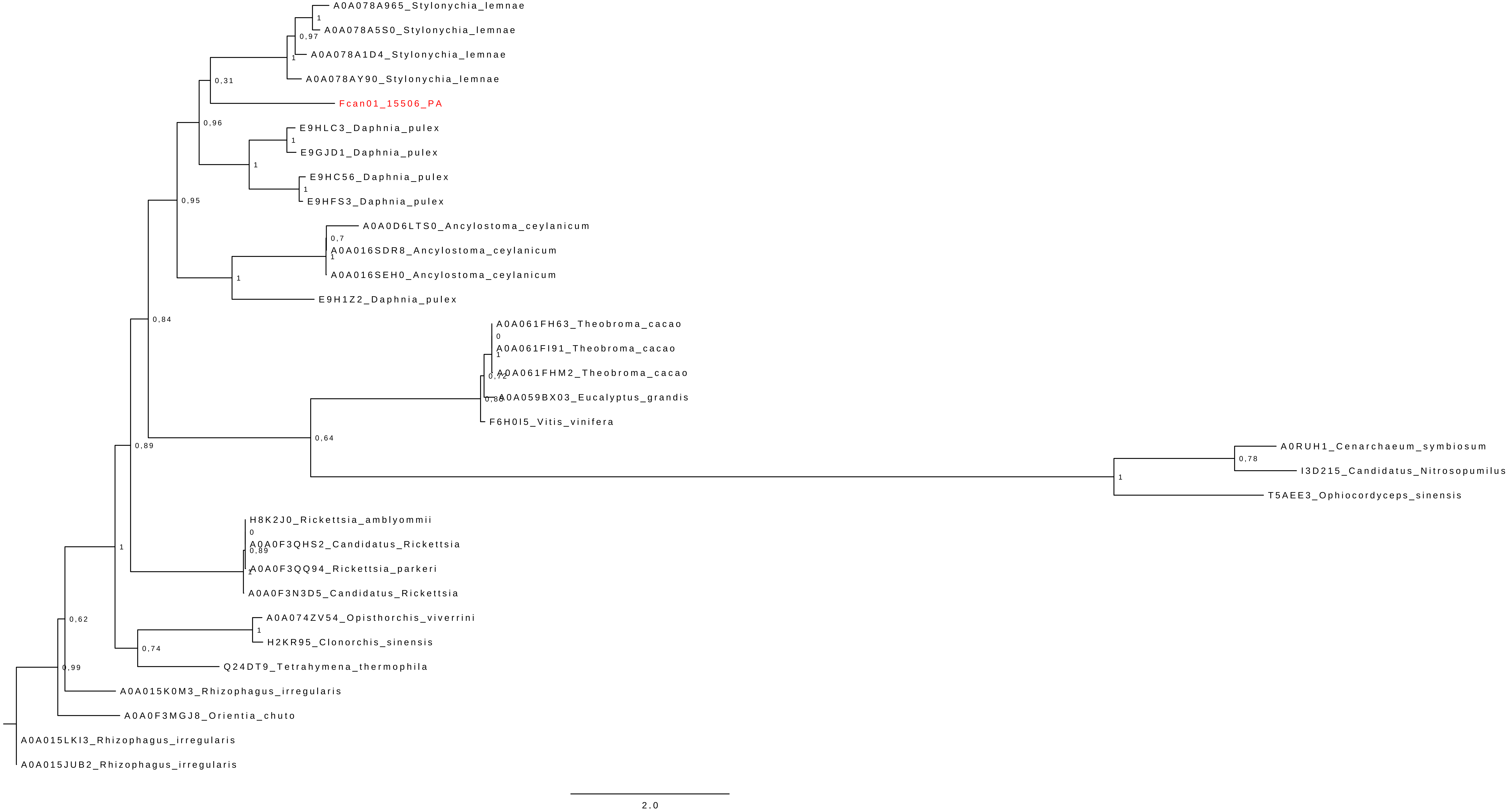
A0A078A965_Stylonychia_lemnae
A0A078A5S0_Stylonychia_lemnae
A0A078A1D4_Stylonychia_lemnae
A0A078AY90_Stylonychia_lemnae
Fcan01_15506_PA
E9HLC3_Daphnia_pulex
E9GJD1_Daphnia_pulex
E9HC56_Daphnia_pulex
E9HFS3_Daphnia_pulex
A0A0D6LTS0_Ancylostoma_ceylanicum
A0A016SDR8_Ancylostoma_ceylanicum
A0A016SEH0_Ancylostoma_ceylanicum
E9H1Z2_Daphnia_pulex
A0A061FH63_Theobroma_cacao
A0A061FI91_Theobroma_cacao
A0A061FHM2_Theobroma_cacao
A0A059BX03_Eucalyptus_grandis
F6H0I5_Vitis_vinifera
A0RUH1_Cenarchaeum_symbiosum
I3D215_Candidatus_Nitrosopumilus
T5AEE3_Ophiocordyceps_sinensis
H8K2J0_Rickettsia_amblyommii
A0A0F3QHS2_Candidatus_Rickettsia
A0A0F3QQ94_Rickettsia_parkeri
A0A0F3N3D5_Candidatus_Rickettsia
A0A074ZV54_Opisthorchis_viverrini
H2KR95_Clonorchis_sinensis
Q24DT9_Tetrahymena_thermophila
A0A015K0M3_Rhizophagus_irregularis
A0A0F3MGJ8_Orientia_chuto
A0A015LKI3_Rhizophagus_irregularis
A0A015JUB2_Rhizophagus_irregularis
2.0

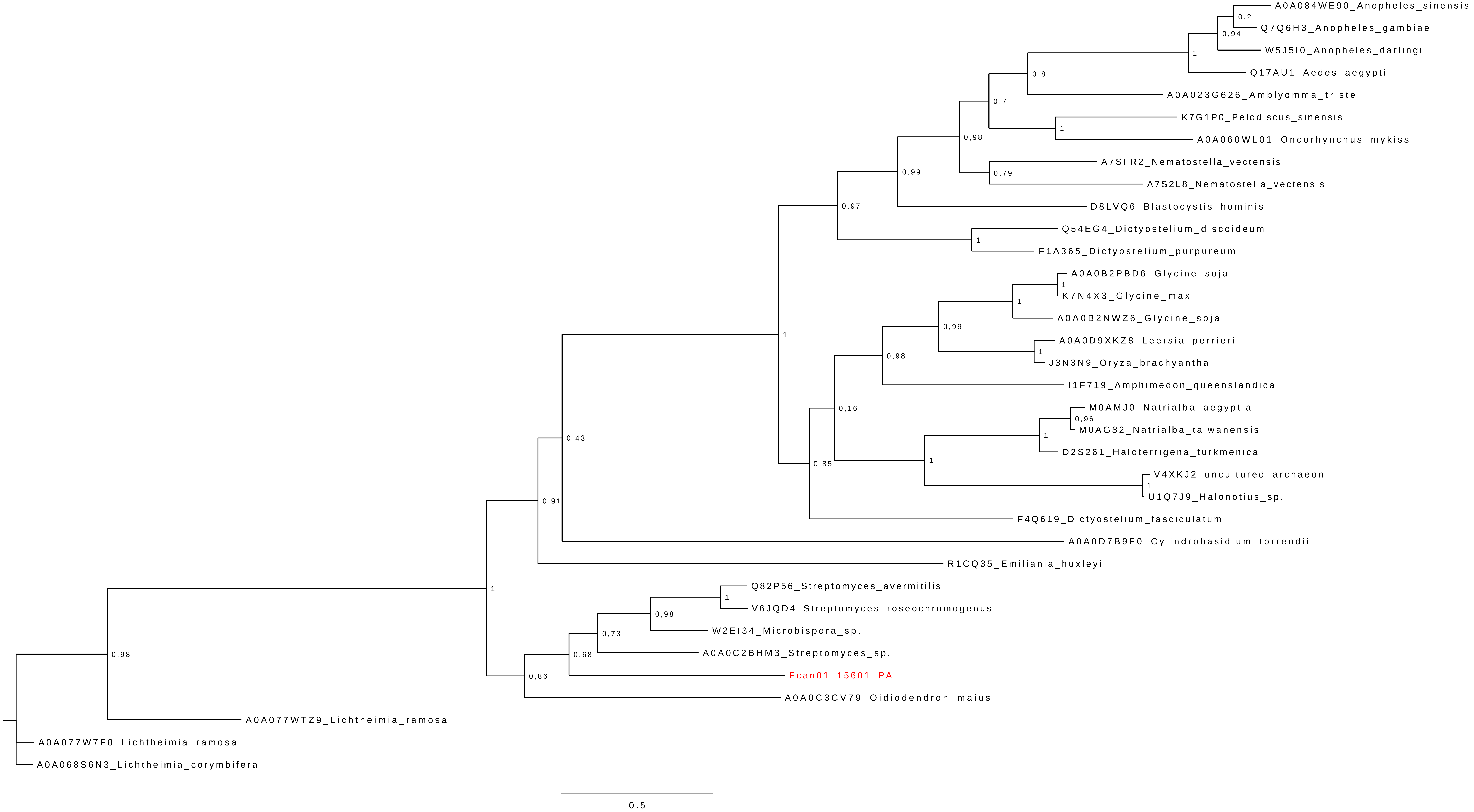

A0A084WE90_Anopheles_sinensis
Q7Q6H3_Anopheles_gambiae
W5J5I0_Anopheles_darlingi
Q17AU1_Aedes_aegypti
A0A023G626_Amblyomma_triste
K7G1P0_Pelodiscus_sinensis
A0A060WL01_Oncorhynchus_mykiss
A7SFR2_Nematostella_vectensis
A7S2L8_Nematostella_vectensis
D8LVQ6_Blastocystis_hominis
Q54EG4_Dictyostelium_discoideum
F1A365_Dictyostelium_purpureum
A0A0B2PBD6_Glycine_soja
K7N4X3_Glycine_max
A0A0B2NWZ6_Glycine_soja
A0A0D9XKZ8_Leersia_perrieri
J3N3N9_Oryza_brachyantha
I1F719_Amphimedon_queenslandica
M0AMJ0_Natrialba_aegyptia
M0AG82_Natrialba_taiwanensis
D2S261_Haloterrigena_turkmenica
V4XKJ2_uncultured_archaeon
U1Q7J9_Halonotius_sp.
F4Q619_Dictyostelium_fasciculatum
A0A0D7B9F0_Cylindrobasidium_torrendii
R1CQ35_Emiliania_huxleyi
Q82P56_Streptomyces_avermitilis
V6JQD4_Streptomyces_roseochromogenus
W2EI34_Microbispora_sp.
A0A0C2BHM3_Streptomyces_sp.
Fcan01_15601_PA
A0A0C3CV79_Oidiodendron_maius
A0A077WTZ9_Lichtheimia_ramosa
A0A077W7F8_Lichtheimia_ramosa
A0A068S6N3_Lichtheimia_corymbifera
0.5

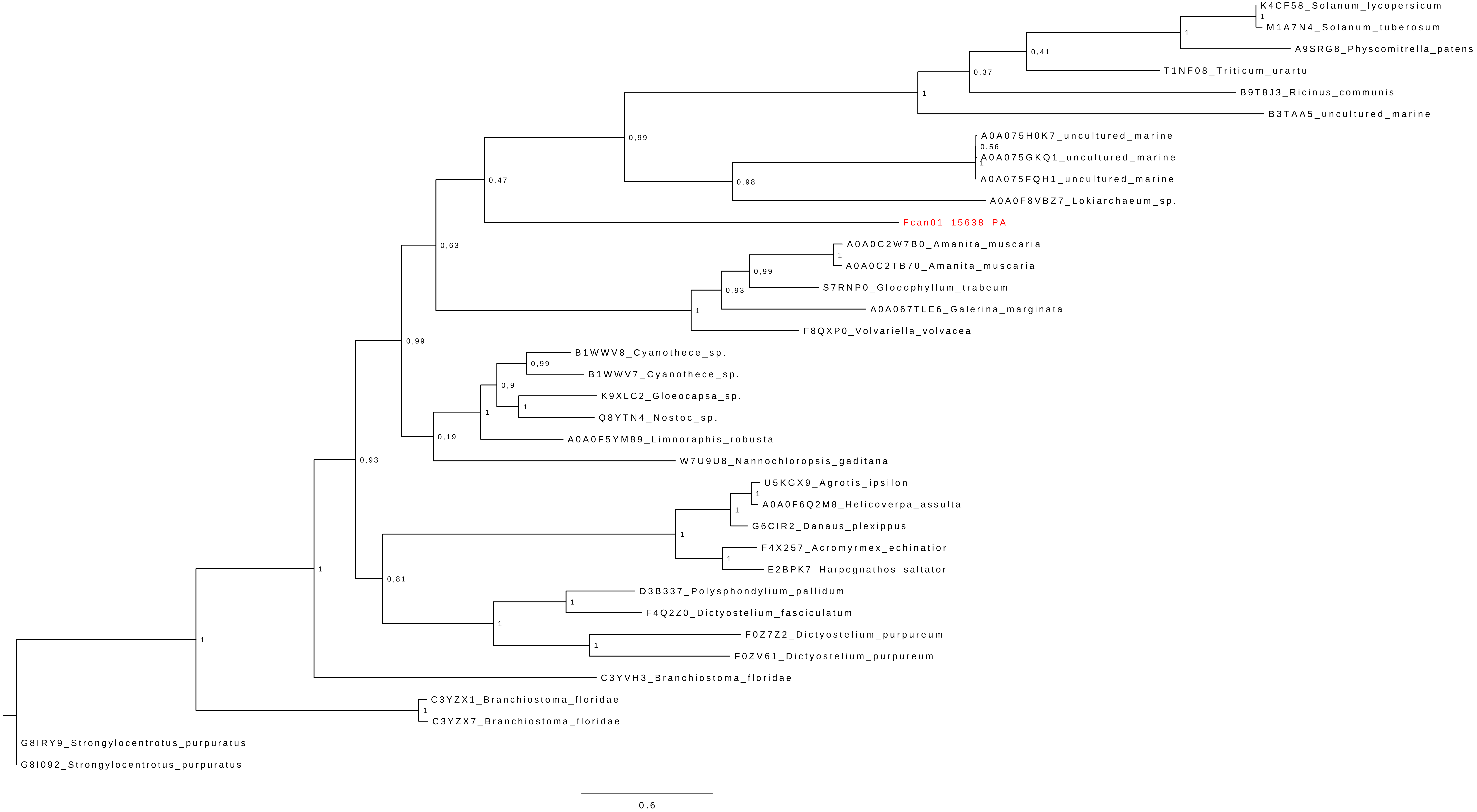

K4CF58_Solanum_lycopersicum
M1A7N4_Solanum_tuberosum
A9SRG8_Physcomitrella_patens
T1NF08_Triticum_urartu
B9T8J3_Ricinus_communis
B3TAA5_uncultured_marine
A0A075H0K7_uncultured_marine
A0A075GKQ1_uncultured_marine
A0A075FQH1_uncultured_marine
A0A0F8VBZ7_Lokiarchaeum_sp.
Fcan01_15638_PA
A0A0C2W7B0_Amanita_muscaria
A0A0C2TB70_Amanita_muscaria
S7RNP0_Gloeophyllum_trabeum
A0A067TLE6_Galerina_marginata
F8QXP0_Volvariella_volvacea
B1WWV8_Cyanothece_sp.
B1WWV7_Cyanothece_sp.
K9XLC2_Gloeocapsa_sp.
Q8YTN4_Nostoc_sp.
A0A0F5YM89_Limnoraphis_robusta
W7U9U8_Nannochloropsis_gaditana
U5KGX9_Agrotis_ipsilon
A0A0F6Q2M8_Helicoverpa_assulta
G6CIR2_Danaus_plexippus
F4X257_Acromyrmex_echinatior
E2BPK7_Harpegnathos_saltator
D3B337_Polysphondylium_pallidum
F4Q2Z0_Dictyostelium_fasciculatum
F0Z7Z2_Dictyostelium_purpureum
F0ZV61_Dictyostelium_purpureum
C3YVH3_Branchiostoma_floridae
C3YZX1_Branchiostoma_floridae
C3YZX7_Branchiostoma_floridae
G8IRY9_Strongylocentrotus_purpuratus
G8I092_Strongylocentrotus_purpuratus
0.6

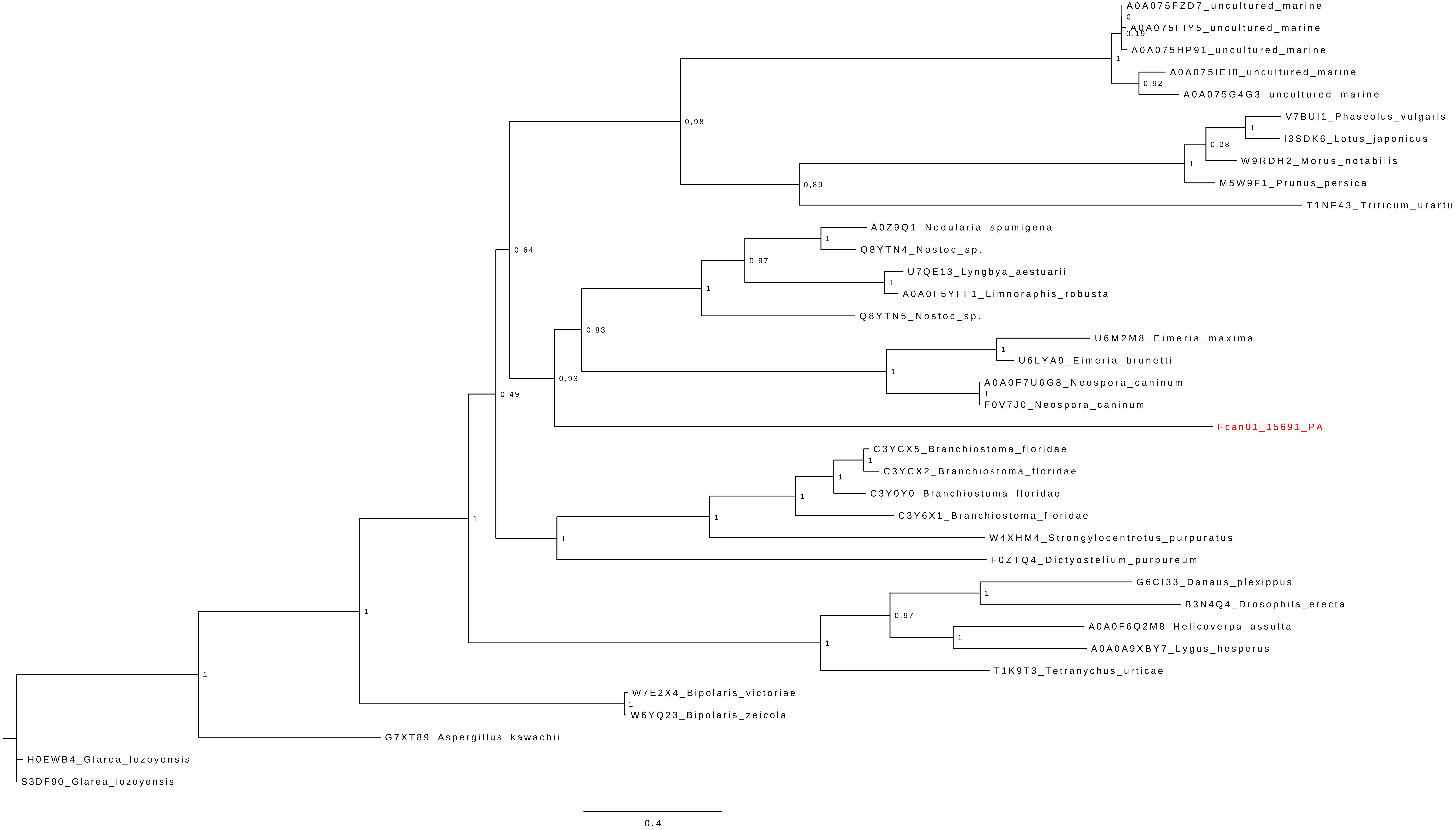
A0A075FZD7_uncultured_marine
A0A075FIY5_uncultured_marine
A0A075HP91_uncultured_marine
A0A075IEI8_uncultured_marine
A0A075G4G3_uncultured_marine
V7BUI1_Phaseolus_vulgaris
I3SDK6_Lotus_japonicus
W9RDH2_Morus_notabilis
M5W9F1_Prunus_persica
T1NF43_Triticum_urartu
A0Z9Q1_Nodularia_spumigena
Q8YTN4_Nostoc_sp.
U7QE13_Lyngbya_aestuarii
A0A0F5YFF1_Limnoraphis_robusta
Q8YTN5_Nostoc_sp.
U6M2M8_Eimeria_maxima
U6LYA9_Eimeria_brunetti
A0A0F7U6G8_Neospora_caninum
F0V7J0_Neospora_caninum
Fcan01_15691_PA
C3YCX5_Branchiostoma_floridae
C3YCX2_Branchiostoma_floridae
C3Y0Y0_Branchiostoma_floridae
C3Y6X1_Branchiostoma_floridae
W4XHM4_Strongylocentrotus_purpuratus
F0ZTQ4_Dictyostelium_purpureum
G6CI33_Danaus_plexippus
B3N4Q4_Drosophila_erecta
A0A0F6Q2M8_Helicoverpa_assulta
A0A0A9XBY7_Lygus_hesperus
T1K9T3_Tetranychus_urticae
W7E2X4_Bipolaris_victoriae
W6YQ23_Bipolaris_zeicola
G7XT89_Aspergillus_kawachii
H0EWB4_Glarea_lozoyensis
S3DF90_Glarea_lozoyensis
0.4

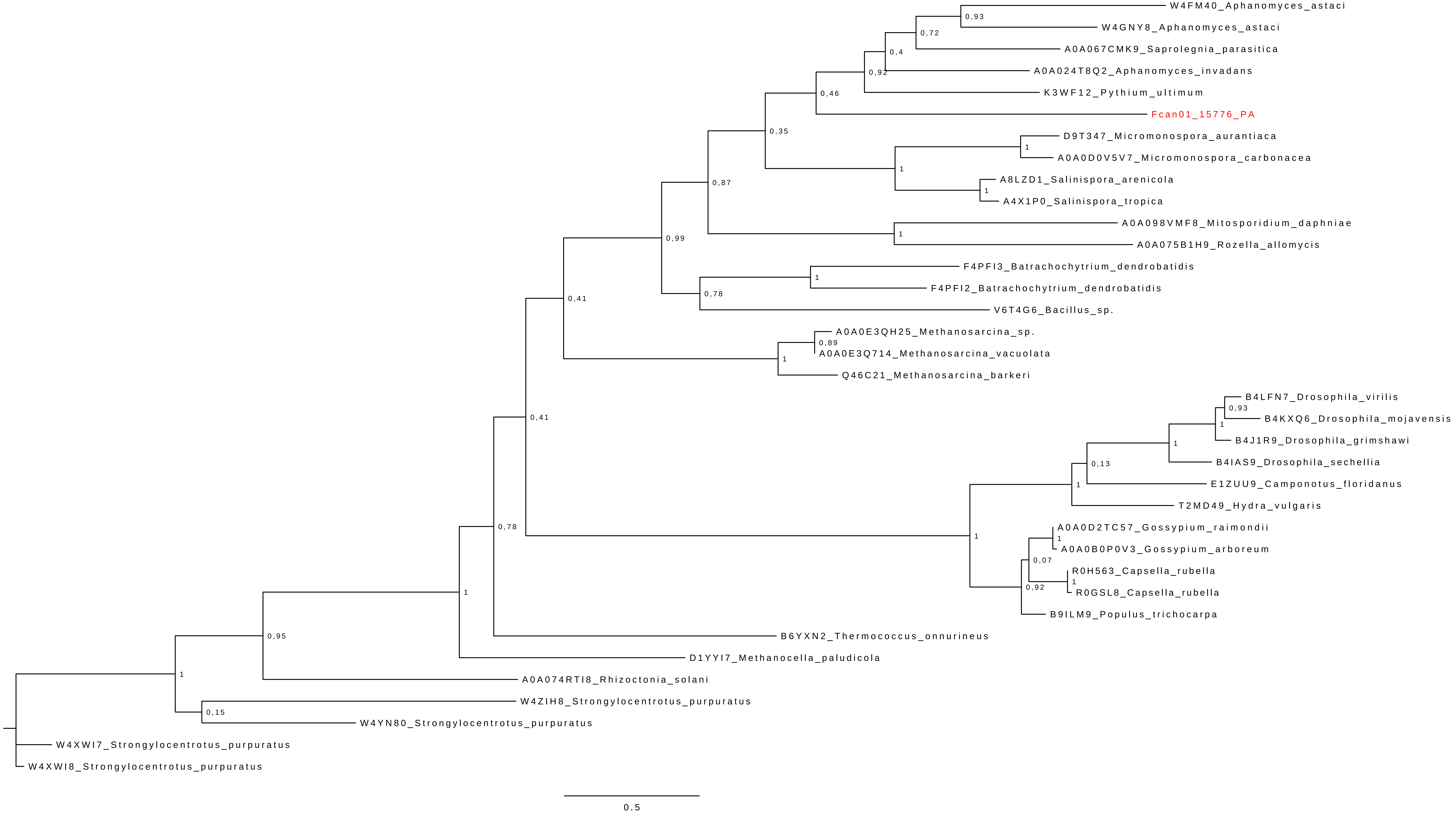
W4FM40_Aphanomyces_astaci
W4GNY8_Aphanomyces_astaci
A0A067CMK9_Saprolegnia_parasitica
A0A024T8Q2_Aphanomyces_invadans
K3WF12_Pythium_ultimum
Fcan01_15776_PA
D9T347_Micromonospora_aurantiaca
A0A0D0V5V7_Micromonospora_carbonacea
A8LZD1_Salinispora_arenicola
A4X1P0_Salinispora_tropica
A0A098VMF8_Mitosporidium_daphniae
A0A075B1H9_Rozella_allomycis
F4PFI3_Batrachochytrium_dendrobatidis
F4PFI2_Batrachochytrium_dendrobatidis
V6T4G6_Bacillus_sp.
A0A0E3QH25_Methanosarcina_sp.
A0A0E3Q714_Methanosarcina_vacuolata
Q46C21_Methanosarcina_barkeri
B4LFN7_Drosophila_virilis
B4KXQ6_Drosophila_mojavensis
B4J1R9_Drosophila_grimshawi
B4IAS9_Drosophila_sechellia
E1ZUU9_Camponotus_floridanus
T2MD49_Hydra_vulgaris
A0A0D2TC57_Gossypium_raimondii
A0A0B0P0V3_Gossypium_arboreum
R0H563_Capsella_rubella
R0GSL8_Capsella_rubella
B9ILM9_Populus_trichocarpa
B6YXN2_Thermococcus_onnurineus
D1YYI7_Methanocella_paludicola
A0A074RTI8_Rhizoctonia_solani
W4ZIH8_Strongylocentrotus_purpuratus
W4YN80_Strongylocentrotus_purpuratus
W4XWI7_Strongylocentrotus_purpuratus
W4XWI8_Strongylocentrotus_purpuratus
0.5

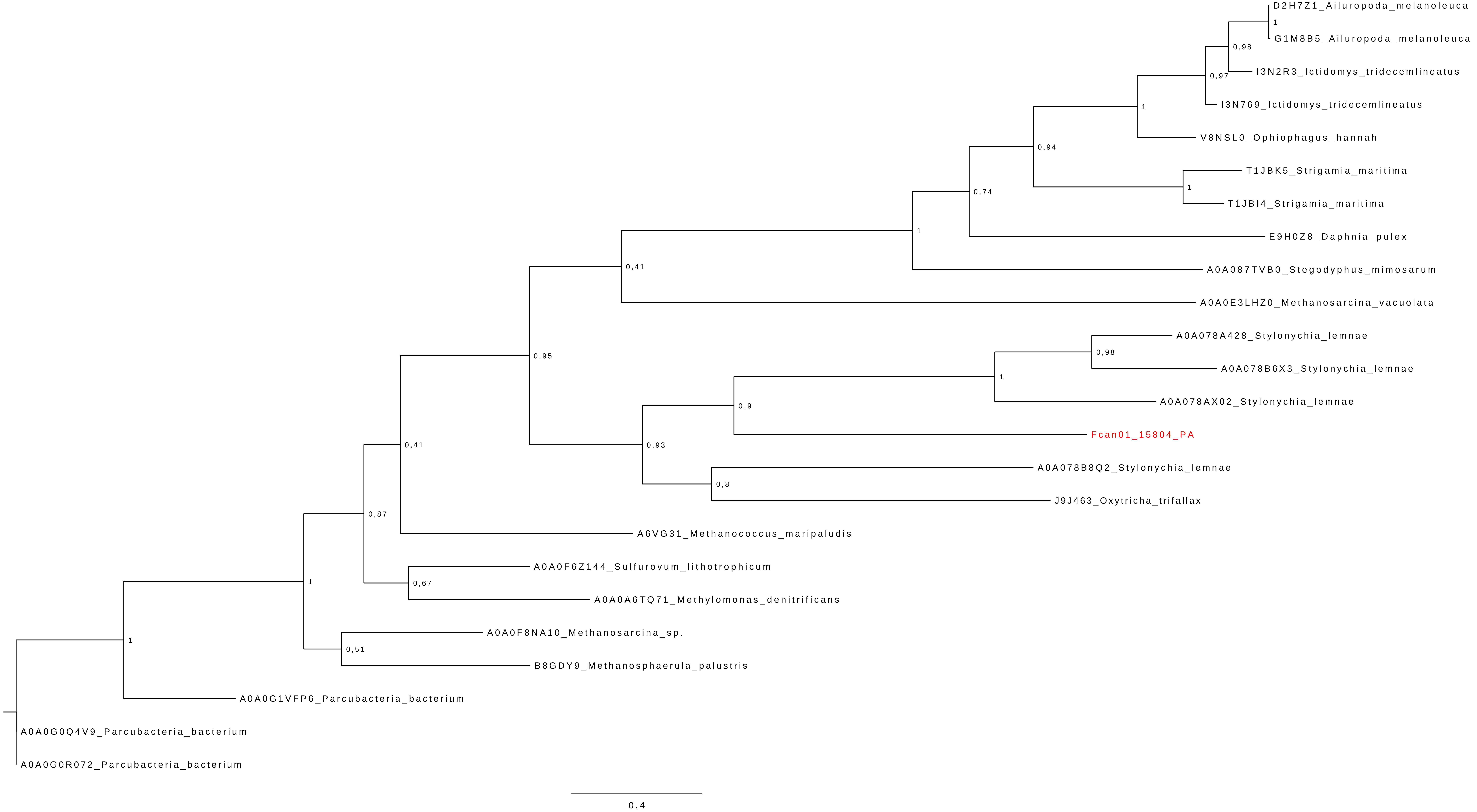
D2H7Z1_Ailuropoda_melanoleuca
G1M8B5_Ailuropoda_melanoleuca
I3N2R3_Ictidomys_tridecemlineatus
I3N769_Ictidomys_tridecemlineatus
V8NSL0_Ophiophagus_hannah
T1JBK5_Strigamia_maritima
T1JBI4_Strigamia_maritima
E9H0Z8_Daphnia_pulex
A0A087TVB0_Stegodyphus_mimosarum
A0A0E3LHZ0_Methanosarcina_vacuolata
A0A078A428_Stylonychia_lemnae
A0A078B6X3_Stylonychia_lemnae
A0A078AX02_Stylonychia_lemnae
Fcan01_15804_PA
A0A078B8Q2_Stylonychia_lemnae
J9J463_Oxytricha_trifallax
A6VG31_Methanococcus_maripaludis
A0A0F6Z144_Sulfurovum_lithotrophicum
A0A0A6TQ71_Methylomonas_denitrificans
A0A0F8NA10_Methanosarcina_sp.
B8GDY9_Methanosphaerula_palustris
A0A0G1VFP6_Parcubacteria_bacterium
A0A0G0Q4V9_Parcubacteria_bacterium
A0A0G0R072_Parcubacteria_bacterium
1
0,98
0,97
1
0,94
1
0,74
1
0,41
0,98
1
0,9
0,95
0,93
0,8
0,41
0,87
0,67
1
0,51
1
0.4

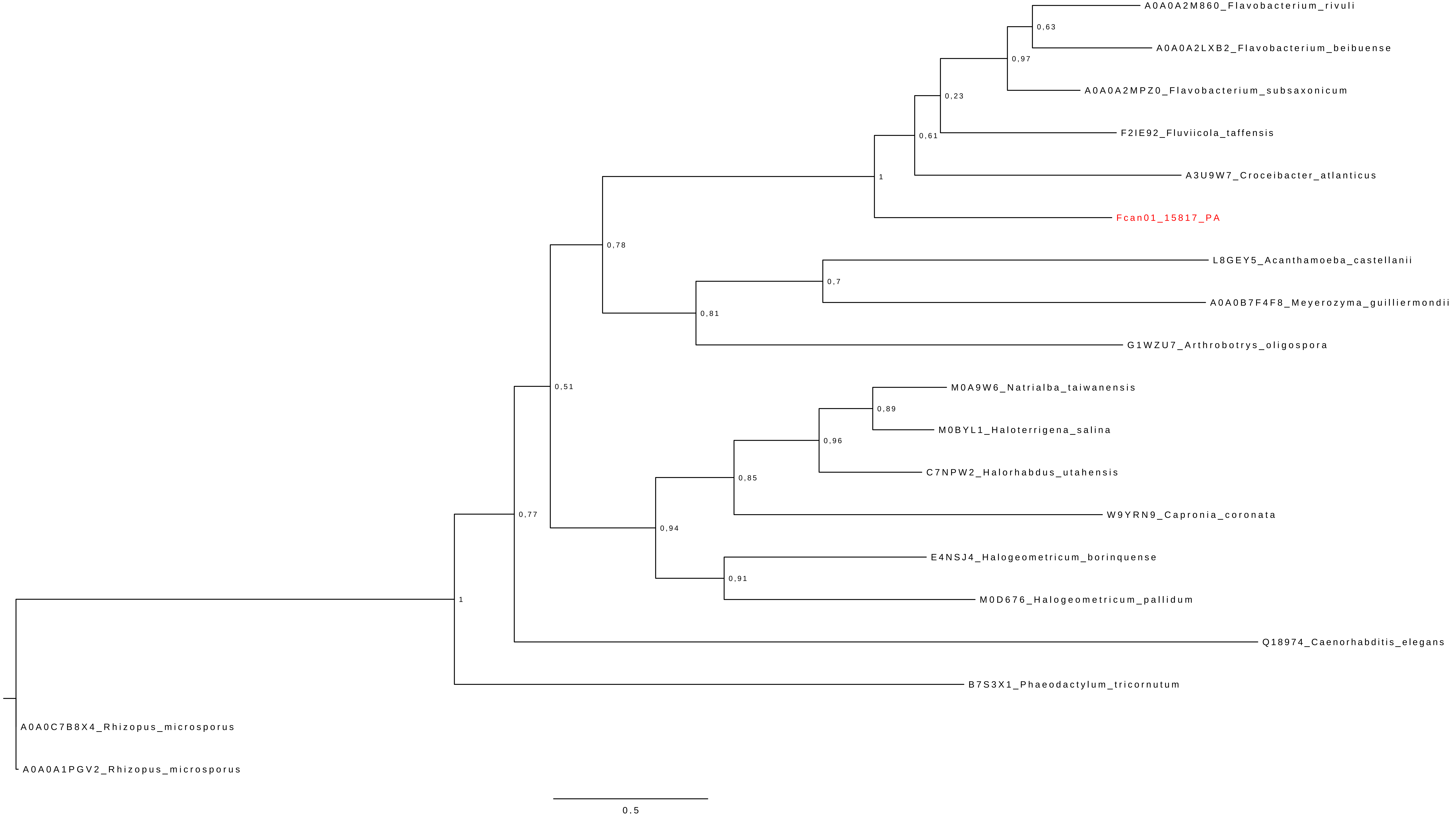
A0A0A2M860_Flavobacterium_rivuli
0,63
A0A0A2LXB2_Flavobacterium_beibuense
0,97
A0A0A2MPZ0_Flavobacterium_subsaxonicum
0,23
F2IE92_Fluviicola_taffensis
0,61
A3U9W7_Croceibacter_atlanticus
1
Fcan01_15817_PA
0,78
L8GEY5_Acanthamoeba_castellanii
0,7
A0A0B7F4F8_Meyerozyma_guilliermondii
0,81
G1WZU7_Arthrobotrys_oligospora
0,51
M0A9W6_Natrialba_taiwanensis
0,89
M0BYL1_Haloterrigena_salina
0,96
C7NPW2_Halorhabdus_utahensis
0,85
W9YRN9_Capronia_coronata
0,77
0,94
E4NSJ4_Halogeometricum_borinquense
0,91
M0D676_Halogeometricum_pallidum
1
Q18974_Caenorhabditis_elegans
B7S3X1_Phaeodactylum_tricornutum
A0A0C7B8X4_Rhizopus_microsporus
A0A0A1PGV2_Rhizopus_microsporus
0.5

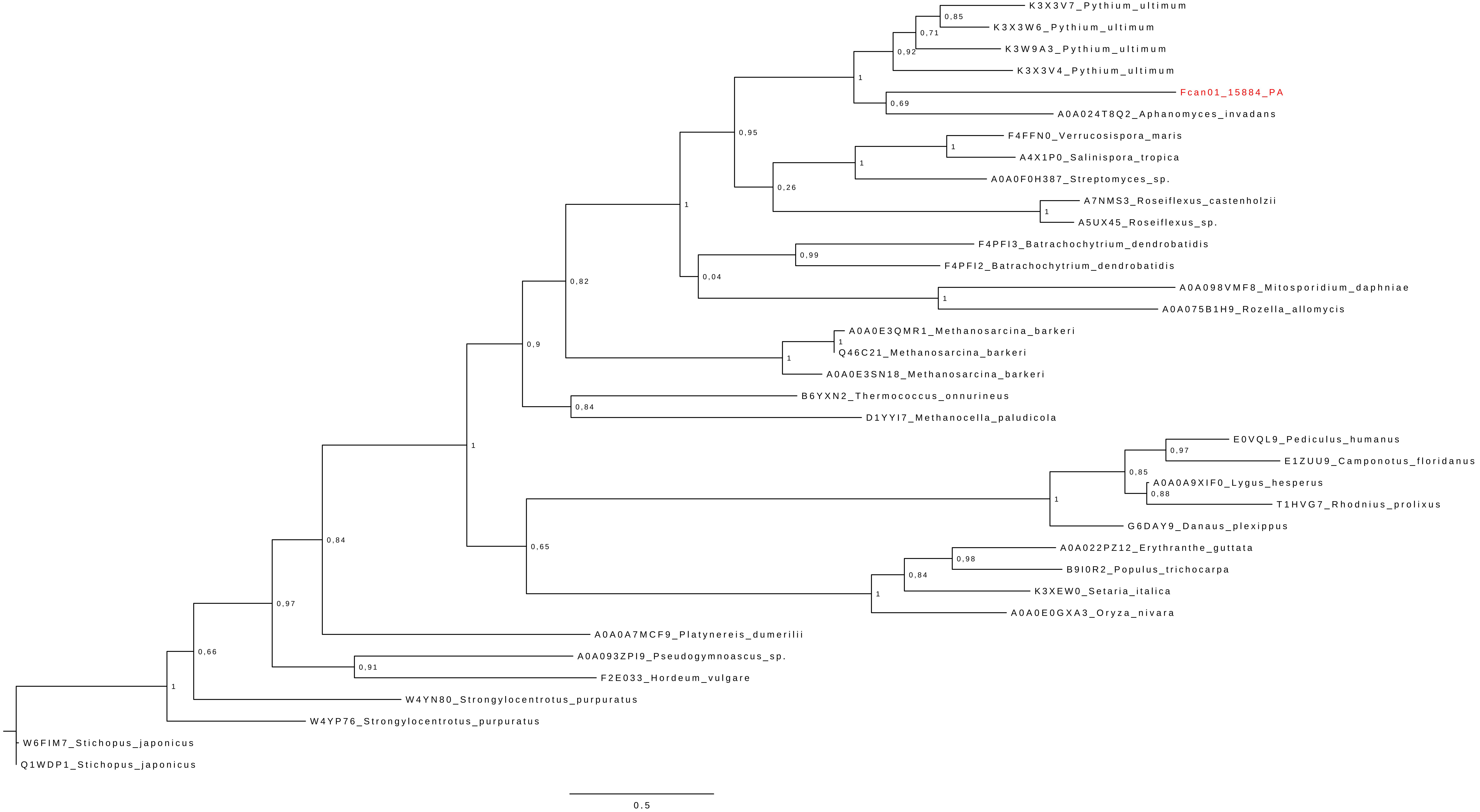
K3X3V7_Pythium_ultimum
K3X3W6_Pythium_ultimum
K3W9A3_Pythium_ultimum
K3X3V4_Pythium_ultimum
Fcan01_15884_PA
A0A024T8Q2_Aphanomyces_invadans
F4FFN0_Verrucosispora_maris
A4X1P0_Salinispora_tropica
A0A0F0H387_Streptomyces_sp.
A7NMS3_Roseiflexus_castenholzii
A5UX45_Roseiflexus_sp.
F4PFI3_Batrachochytrium_dendrobatidis
F4PFI2_Batrachochytrium_dendrobatidis
A0A098VMF8_Mitosporidium_daphniae
A0A075B1H9_Rozella_allomycis
A0A0E3QMR1_Methanosarcina_barkeri
Q46C21_Methanosarcina_barkeri
A0A0E3SN18_Methanosarcina_barkeri
B6YXN2_Thermococcus_onnurineus
D1YYI7_Methanocella_paludicola
E0VQL9_Pediculus_humanus
E1ZUU9_Camponotus_floridanus
A0A0A9XIF0_Lygus_hesperus
T1HVG7_Rhodnius_prolixus
G6DAY9_Danaus_plexippus
A0A022PZ12_Erythranthe_guttata
B9I0R2_Populus_trichocarpa
K3XEW0_Setaria_italica
A0A0E0GXA3_Oryza_nivara
A0A0A7MCF9_Platynereis_dumerilii
A0A093ZPI9_Pseudogymnoascus_sp.
F2E033_Hordeum_vulgare
W4YN80_Strongylocentrotus_purpuratus
W4YP76_Strongylocentrotus_purpuratus
W6FIM7_Stichopus_japonicus
Q1WDP1_Stichopus_japonicus
0.5

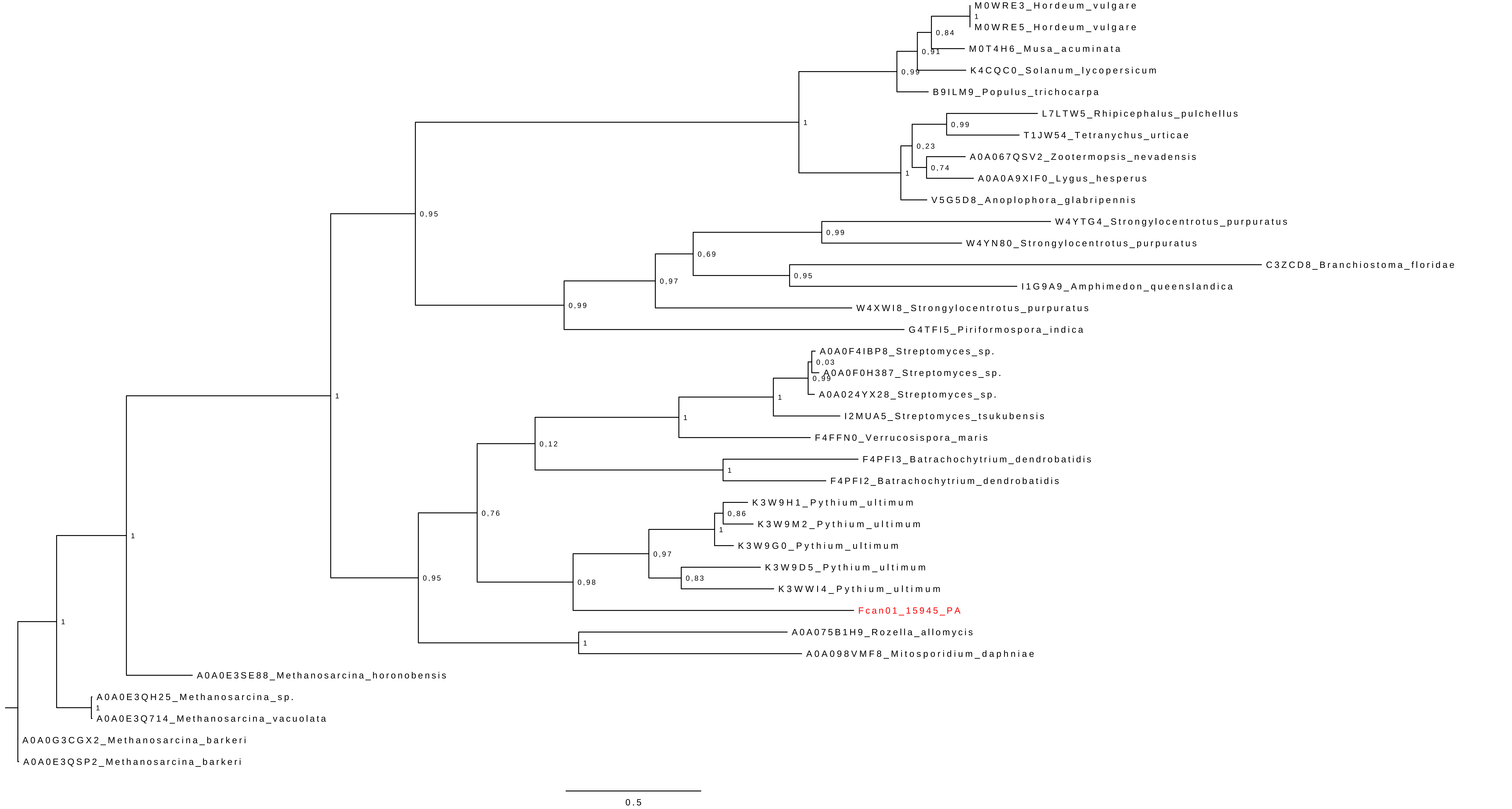
M0WRE3_Hordeum_vulgare
M0WRE5_Hordeum_vulgare
M0T4H6_Musa_acuminata
K4CQC0_Solanum_lycopersicum
B9ILM9_Populus_trichocarpa
L7LTW5_Rhipicephalus_pulchellus
T1JW54_Tetranychus_urticae
A0A067QSV2_Zootermopsis_nevadensis
A0A0A9XIF0_Lygus_hesperus
V5G5D8_Anoplophora_glabripennis
W4YTG4_Strongylocentrotus_purpuratus
W4YN80_Strongylocentrotus_purpuratus
C3ZCD8_Branchiostoma_floridae
I1G9A9_Amphimedon_queenslandica
W4XWI8_Strongylocentrotus_purpuratus
G4TFI5_Piriformospora_indica
A0A0F4IBP8_Streptomyces_sp.
A0A0F0H387_Streptomyces_sp.
A0A024YX28_Streptomyces_sp.
I2MUA5_Streptomyces_tsukubensis
F4FFN0_Verrucosispora_maris
F4PFI3_Batrachochytrium_dendrobatidis
F4PFI2_Batrachochytrium_dendrobatidis
K3W9H1_Pythium_ultimum
K3W9M2_Pythium_ultimum
K3W9G0_Pythium_ultimum
K3W9D5_Pythium_ultimum
K3WWI4_Pythium_ultimum
Fcan01_15945_PA
A0A075B1H9_Rozella_allomycis
A0A098VMF8_Mitosporidium_daphniae
A0A0E3SE88_Methanosarcina_horonobensis
A0A0E3QH25_Methanosarcina_sp.
A0A0E3Q714_Methanosarcina_vacuolata
A0A0G3CGX2_Methanosarcina_barkeri
A0A0E3QSP2_Methanosarcina_barkeri
0.5

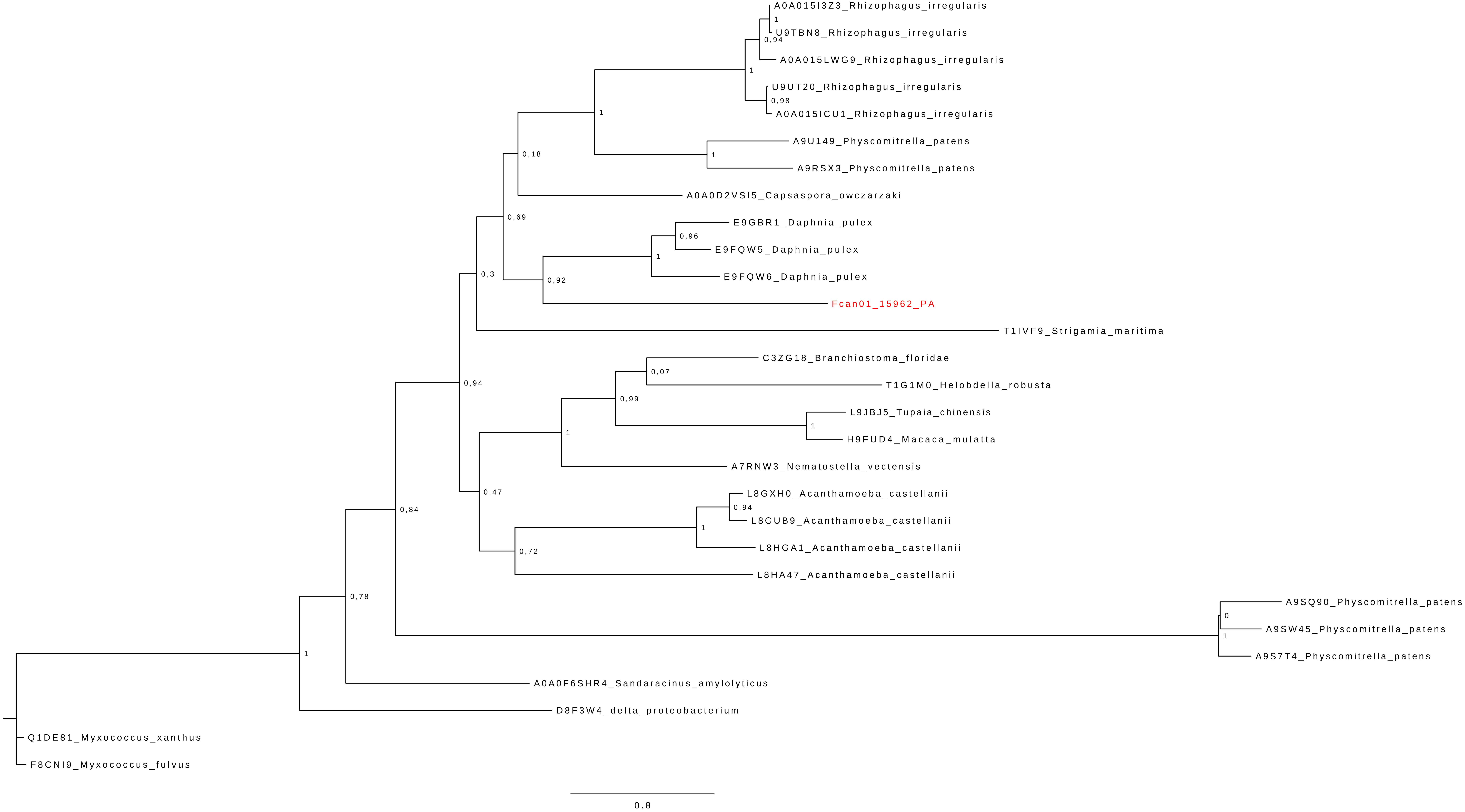
A0A015I3Z3_Rhizophagus_irregularis
U9TBN8_Rhizophagus_irregularis
A0A015LWG9_Rhizophagus_irregularis
U9UT20_Rhizophagus_irregularis
A0A015ICU1_Rhizophagus_irregularis
A9U149_Physcomitrella_patens
A9RSX3_Physcomitrella_patens
A0A0D2VSI5_Capsaspora_owczarzaki
E9GBR1_Daphnia_pulex
E9FQW5_Daphnia_pulex
E9FQW6_Daphnia_pulex
Fcan01_15962_PA
T1IVF9_Strigamia_maritima
C3ZG18_Branchiostoma_floridae
T1G1M0_Helobdella_robusta
L9JBJ5_Tupaia_chinensis
H9FUD4_Macaca_mulatta
A7RNW3_Nematostella_vectensis
L8GXH0_Acanthamoeba_castellanii
L8GUB9_Acanthamoeba_castellanii
L8HGA1_Acanthamoeba_castellanii
L8HA47_Acanthamoeba_castellanii
A9SQ90_Physcomitrella_patens
A9SW45_Physcomitrella_patens
A9S7T4_Physcomitrella_patens
A0A0F6SHR4_Sandaracinus_amylolyticus
D8F3W4_delta_proteobacterium
Q1DE81_Myxococcus_xanthus
F8CNI9_Myxococcus_fulvus
0.8

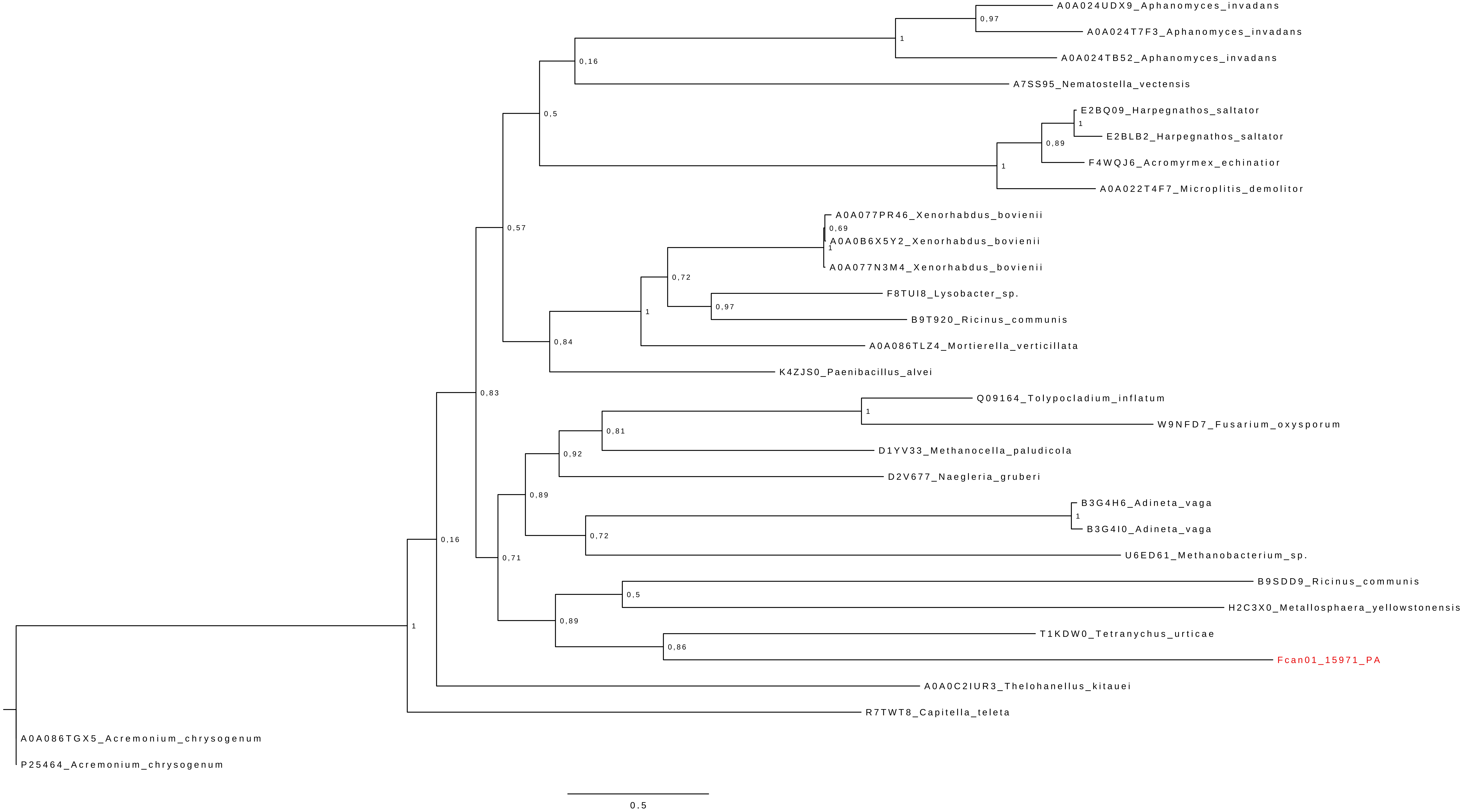

A0A024UDX9_Aphanomyces_invadans
0,97
A0A024T7F3_Aphanomyces_invadans
1
0,16
A0A024TB52_Aphanomyces_invadans
A7SS95_Nematostella_vectensis
0,5
E2BQ09_Harpegnathos_saltator
1
E2BLB2_Harpegnathos_saltator
0,89
F4WQJ6_Acromyrmex_echinatior
1
A0A022T4F7_Microplitis_demolitor
0,57
A0A077PR46_Xenorhabdus_bovienii
0,69
A0A0B6X5Y2_Xenorhabdus_bovienii
1
A0A077N3M4_Xenorhabdus_bovienii
0,72
F8TUI8_Lysobacter_sp.
0,97
B9T920_Ricinus_communis
1
A0A086TLZ4_Mortierella_verticillata
0,84
K4ZJS0_Paenibacillus_alvei
0,83
Q09164_Tolypocladium_inflatum
1
W9NFD7_Fusarium_oxysporum
0,81
D1YV33_Methanocella_paludicola
0,92
D2V677_Naegleria_gruberi
0,89
B3G4H6_Adineta_vaga
1
B3G4I0_Adineta_vaga
0,72
U6ED61_Methanobacterium_sp.
0,16
0,71
B9SDD9_Ricinus_communis
0,5
H2C3X0_Metallosphaera_yellowstonensis
0,89
T1KDW0_Tetranychus_urticae
0,86
Fcan01_15971_PA
A0A0C2IUR3_Thelohanellus_kitauei
1
R7TWT8_Capitella_teleta
A0A086TGX5_Acremonium_chrysogenum
P25464_Acremonium_chrysogenum
0.5

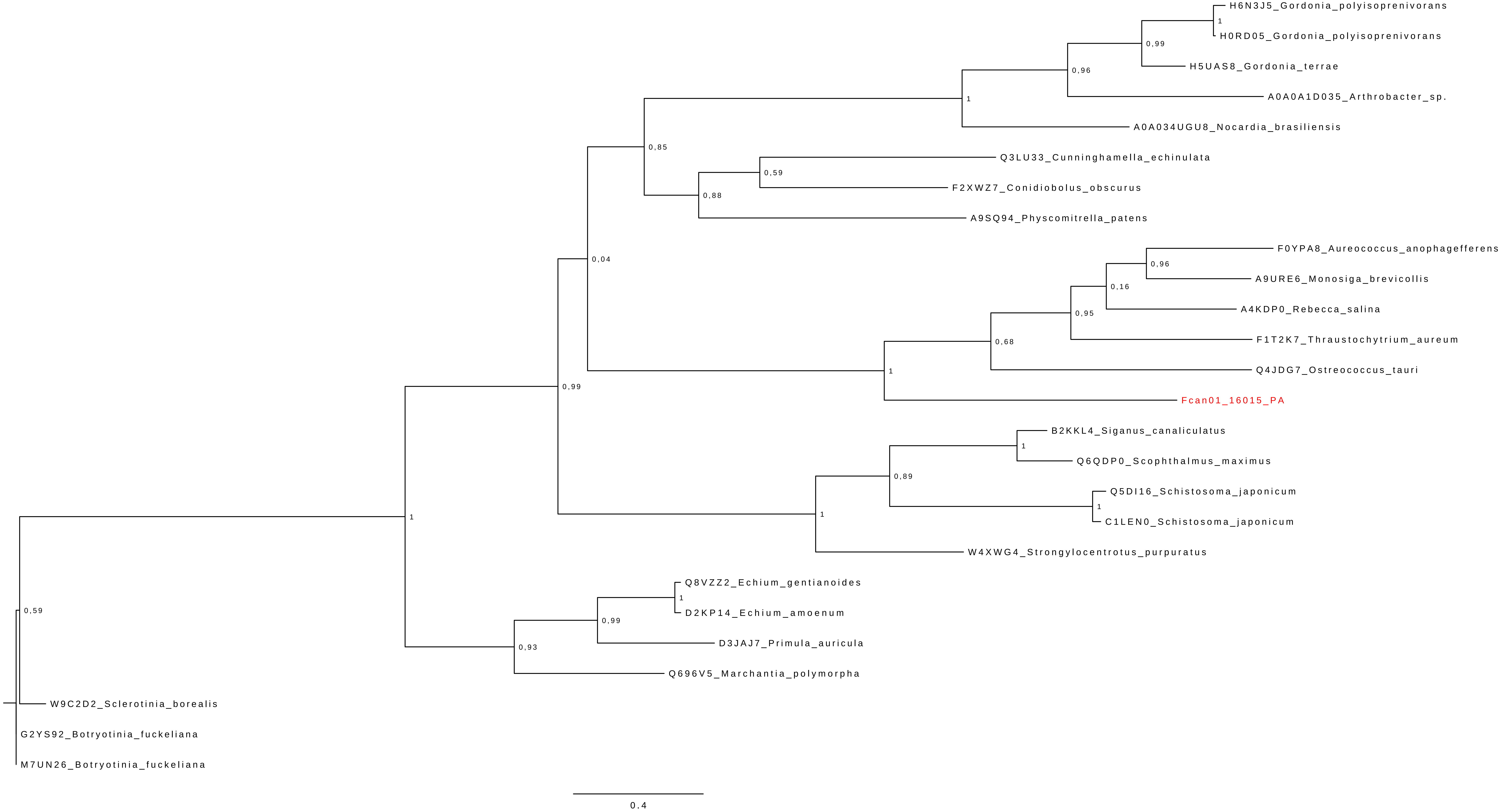

H6N3J5_Gordonia_polyisoprenivorans
H0RD05_Gordonia_polyisoprenivorans
H5UAS8_Gordonia_terrae
A0A0A1D035_Arthrobacter_sp.
A0A034UGU8_Nocardia_brasiliensis
Q3LU33_Cunninghamella_echinulata
F2XWZ7_Conidiobolus_obscurus
A9SQ94_Physcomitrella_patens
F0YPA8_Aureococcus_anophagefferens
A9URE6_Monosiga_brevicollis
A4KDP0_Rebecca_salina
F1T2K7_Thraustochytrium_aureum
Q4JDG7_Ostreococcus_tauri
Fcan01_16015_PA
B2KKL4_Siganus_canaliculatus
Q6QDP0_Scophthalmus_maximus
Q5DI16_Schistosoma_japonicum
C1LEN0_Schistosoma_japonicum
W4XWG4_Strongylocentrotus_purpuratus
Q8VZZ2_Echium_gentianoides
D2KP14_Echium_amoenum
D3JAJ7_Primula_auricula
Q696V5_Marchantia_polymorpha
W9C2D2_Sclerotinia_borealis
G2YS92_Botryotinia_fuckeliana
M7UN26_Botryotinia_fuckeliana
1
0,99
0,96
1
0,85
0,59
0,88
0,04
0,96
0,16
0,95
0,68
1
0,99
1
0,89
1
1
1
1
0,99
0,93
0,59
0.4

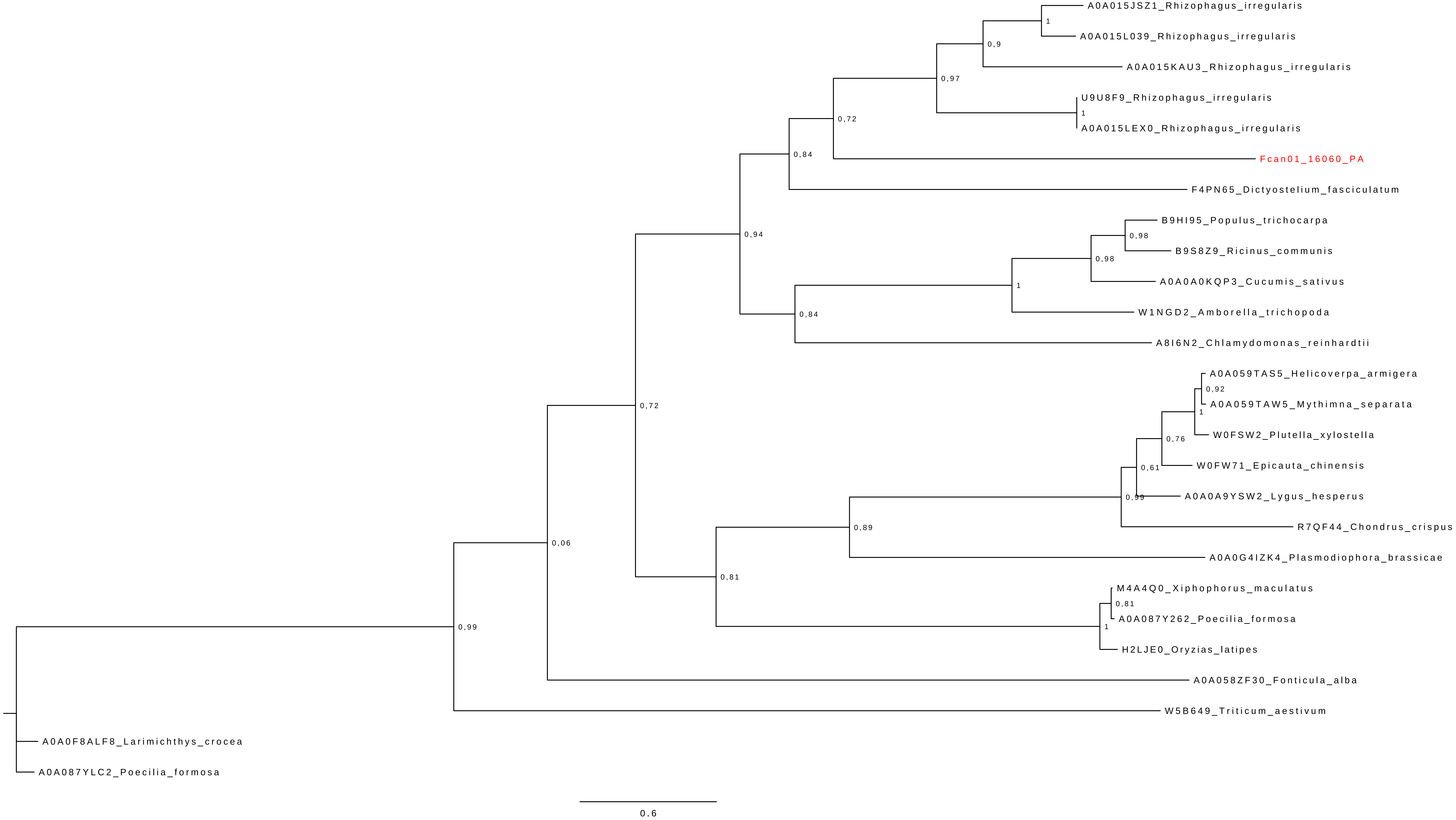
A0A015JSZ1_Rhizophagus_irregularis
1
A0A015L039_Rhizophagus_irregularis
0,9
A0A015KAU3_Rhizophagus_irregularis
0,97
U9U8F9_Rhizophagus_irregularis
1
A0A015LEX0_Rhizophagus_irregularis
0,72
Fcan01_16060_PA
0,84
F4PN65_Dictyostelium_fasciculatum
B9HI95_Populus_trichocarpa
0,98
B9S8Z9_Ricinus_communis
0,98
A0A0A0KQP3_Cucumis_sativus
0,94
1
W1NGD2_Amborella_trichopoda
0,84
A8I6N2_Chlamydomonas_reinhardtii
A0A059TAS5_Helicoverpa_armigera
0,92
A0A059TAW5_Mythimna_separata
1
0,72
W0FSW2_Plutella_xylostella
0,76
W0FW71_Epicauta_chinensis
0,61
A0A0A9YSW2_Lygus_hesperus
0,99
R7QF44_Chondrus_crispus
0,89
0,06
A0A0G4IZK4_Plasmodiophora_brassicae
0,81
M4A4Q0_Xiphophorus_maculatus
0,81
A0A087Y262_Poecilia_formosa
0,99
1
H2LJE0_Oryzias_latipes
A0A058ZF30_Fonticula_alba
W5B649_Triticum_aestivum
A0A0F8ALF8_Larimichthys_crocea
A0A087YLC2_Poecilia_formosa
0.6

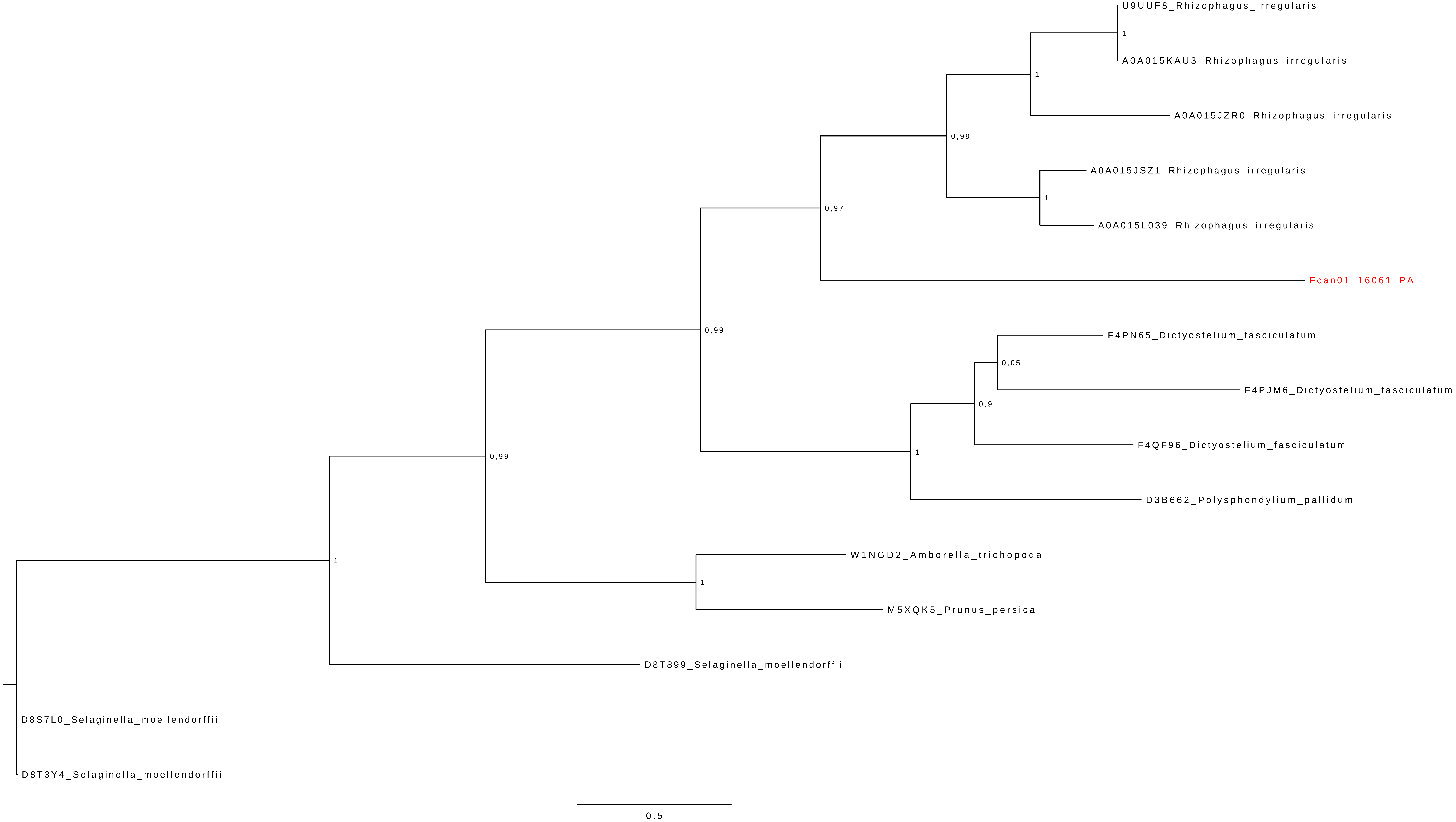
U9UUF8_Rhizophagus_irregularis
1
A0A015KAU3_Rhizophagus_irregularis
1
A0A015JZR0_Rhizophagus_irregularis
0,99
A0A015JSZ1_Rhizophagus_irregularis
1
A0A015L039_Rhizophagus_irregularis
0,97
Fcan01_16061_PA
0,99
F4PN65_Dictyostelium_fasciculatum
0,05
F4PJM6_Dictyostelium_fasciculatum
0,9
F4QF96_Dictyostelium_fasciculatum
1
D3B662_Polysphondylium_pallidum
0,99
W1NGD2_Amborella_trichopoda
1
M5XQK5_Prunus_persica
1
D8T899_Selaginella_moellendorffii
D8S7L0_Selaginella_moellendorffii
D8T3Y4_Selaginella_moellendorffii
0.5

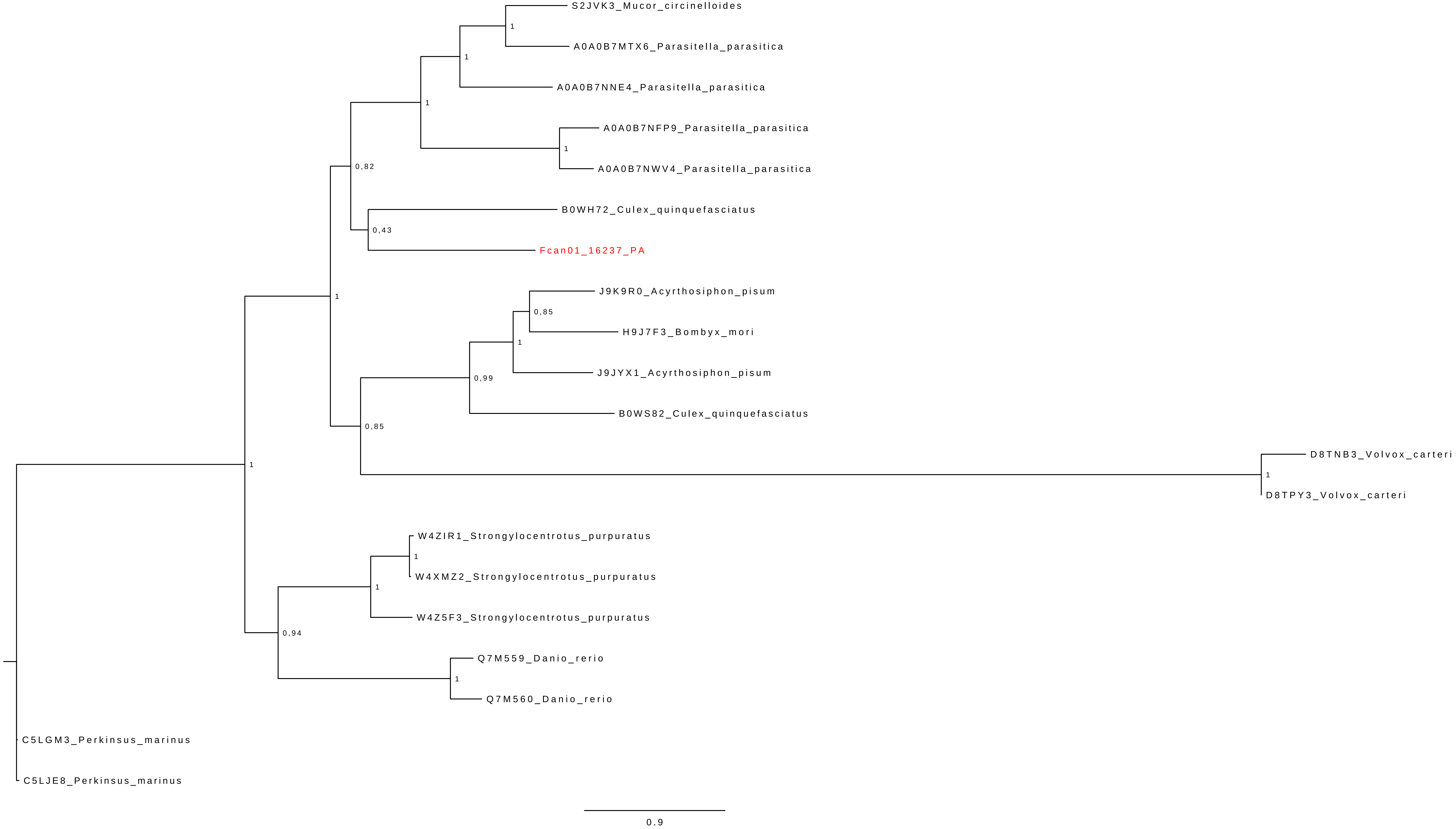
S2JVK3_Mucor_circinelloides
A0A0B7MTX6_Parasitella_parasitica
A0A0B7NNE4_Parasitella_parasitica
A0A0B7NFP9_Parasitella_parasitica
A0A0B7NWV4_Parasitella_parasitica
B0WH72_Culex_quinquefasciatus
Fcan01_16237_PA
J9K9R0_Acyrthosiphon_pisum
H9J7F3_Bombyx_mori
J9JYX1_Acyrthosiphon_pisum
B0WS82_Culex_quinquefasciatus
D8TNB3_Volvox_carteri
D8TPY3_Volvox_carteri
W4ZIR1_Strongylocentrotus_purpuratus
W4XMZ2_Strongylocentrotus_purpuratus
W4Z5F3_Strongylocentrotus_purpuratus
Q7M559_Danio_rerio
Q7M560_Danio_rerio
C5LGM3_Perkinsus_marinus
C5LJE8_Perkinsus_marinus
1
1
1
1
0,82
0,43
1
0,85
1
0,99
0,85
1
1
1
1
0,94
1
1
0.9

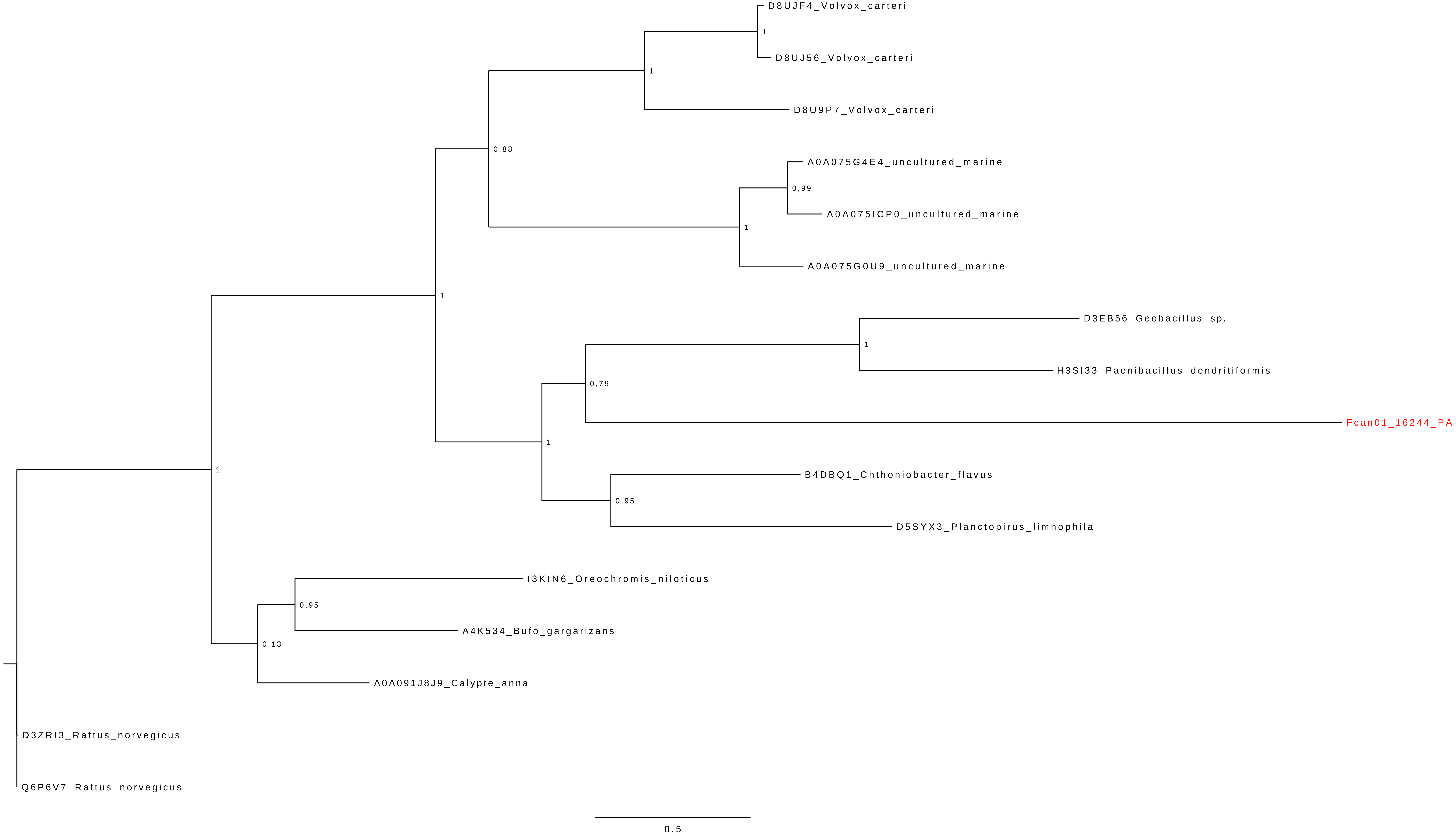
D8UJF4_Volvox_carteri
1
D8UJ56_Volvox_carteri
1
D8U9P7_Volvox_carteri
0,88
A0A075G4E4_uncultured_marine
0,99
A0A075ICP0_uncultured_marine
1
A0A075G0U9_uncultured_marine
1
D3EB56_Geobacillus_sp.
1
H3SI33_Paenibacillus_dendritiformis
0,79
Fcan01_16244_PA
1
B4DBQ1_Chthoniobacter_flavus
1
0,95
D5SYX3_Planctopirus_limnophila
I3KIN6_Oreochromis_niloticus
0,95
A4K534_Bufo_gargarizans
0,13
A0A091J8J9_Calypte_anna
D3ZRI3_Rattus_norvegicus
Q6P6V7_Rattus_norvegicus
0.5

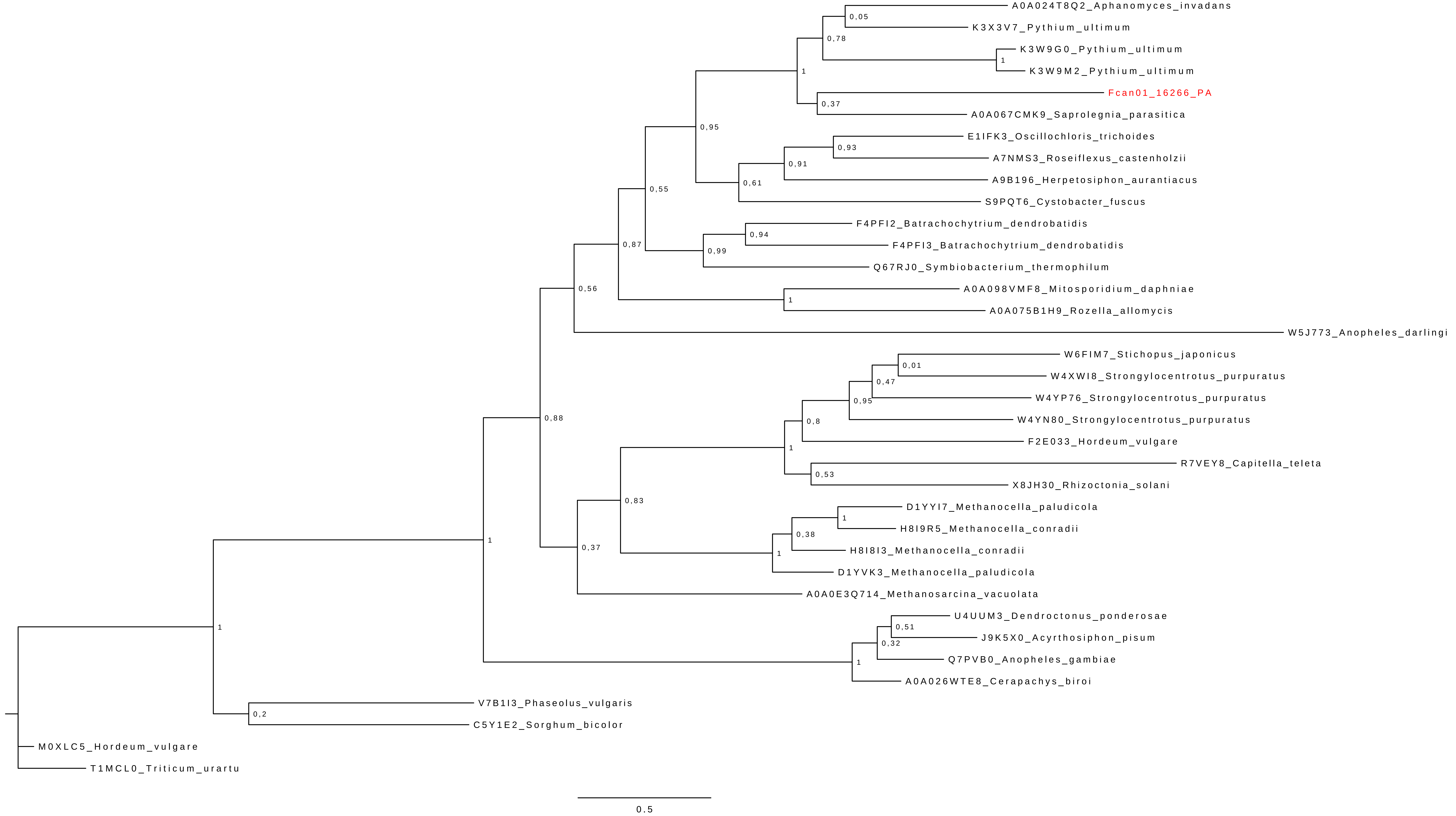
A0A024T8Q2_Aphanomyces_invadans
K3X3V7_Pythium_ultimum
K3W9G0_Pythium_ultimum
K3W9M2_Pythium_ultimum
Fcan01_16266_PA
A0A067CMK9_Saprolegnia_parasitica
E1IFK3_Oscillochloris_trichoides
A7NMS3_Roseiflexus_castenholzii
A9B196_Herpetosiphon_aurantiacus
S9PQT6_Cystobacter_fuscus
F4PFI2_Batrachochytrium_dendrobatidis
F4PFI3_Batrachochytrium_dendrobatidis
Q67RJ0_Symbiobacterium_thermophilum
A0A098VMF8_Mitosporidium_daphniae
A0A075B1H9_Rozella_allomycis
W5J773_Anopheles_darlingi
W6FIM7_Stichopus_japonicus
W4XWI8_Strongylocentrotus_purpuratus
W4YP76_Strongylocentrotus_purpuratus
W4YN80_Strongylocentrotus_purpuratus
F2E033_Hordeum_vulgare
R7VEY8_Capitella_teleta
X8JH30_Rhizoctonia_solani
D1YYI7_Methanocella_paludicola
H8I9R5_Methanocella_conradii
H8I8I3_Methanocella_conradii
D1YVK3_Methanocella_paludicola
A0A0E3Q714_Methanosarcina_vacuolata
U4UUM3_Dendroctonus_ponderosae
J9K5X0_Acyrthosiphon_pisum
Q7PVB0_Anopheles_gambiae
A0A026WTE8_Cerapachys_biroi
V7B1I3_Phaseolus_vulgaris
C5Y1E2_Sorghum_bicolor
M0XLC5_Hordeum_vulgare
T1MCL0_Triticum_urartu
0.5

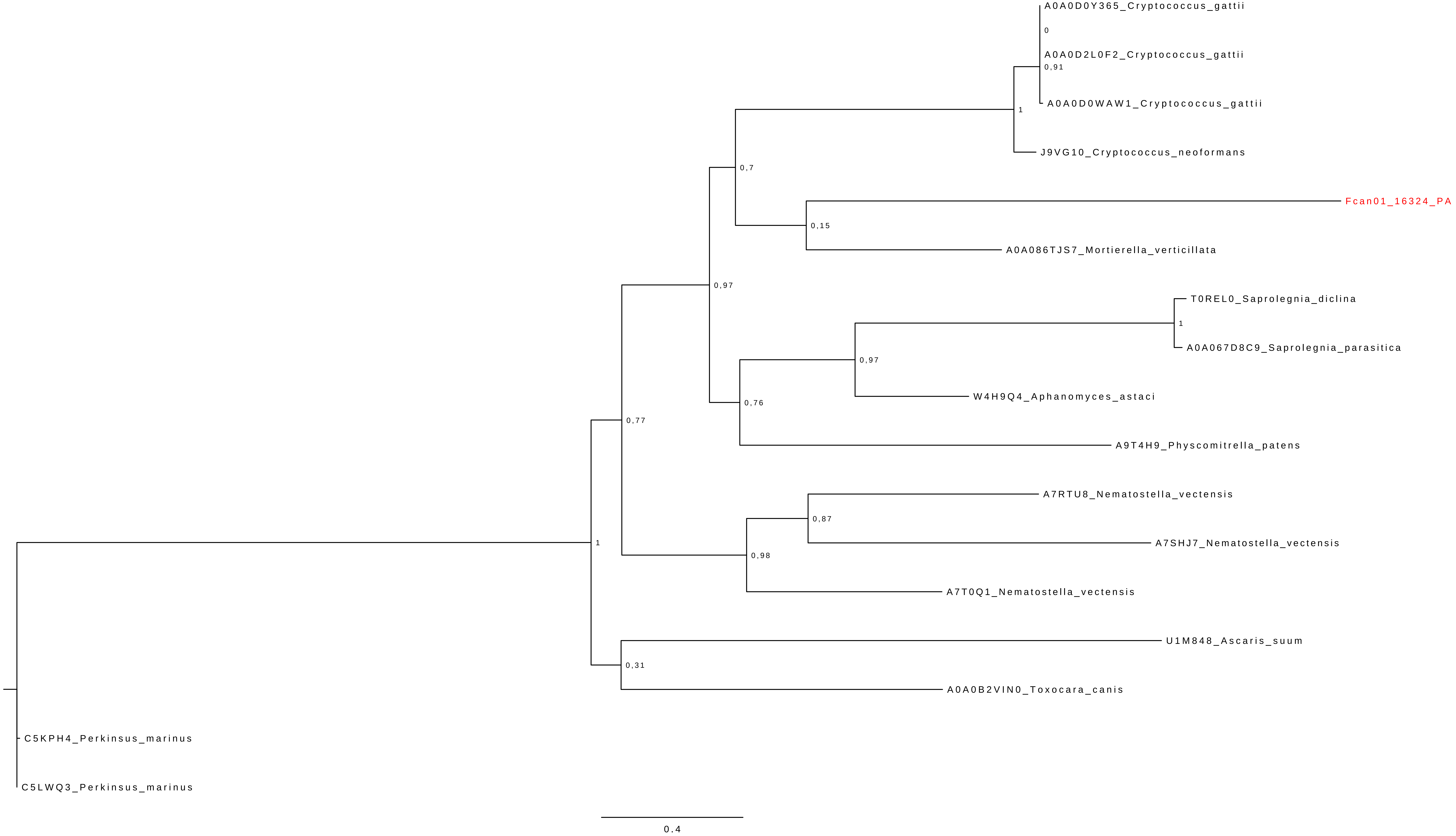
A0A0D0Y365_Cryptococcus_gattii
0
A0A0D2L0F2_Cryptococcus_gattii
0,91
A0A0D0WAW1_Cryptococcus_gattii
1
J9VG10_Cryptococcus_neoformans
0,7
Fcan01_16324_PA
0,15
A0A086TJS7_Mortierella_verticillata
0,97
T0REL0_Saprolegnia_diclina
1
A0A067D8C9_Saprolegnia_parasitica
0,97
W4H9Q4_Aphanomyces_astaci
0,76
A9T4H9_Physcomitrella_patens
0,77
A7RTU8_Nematostella_vectensis
0,87
A7SHJ7_Nematostella_vectensis
1
0,98
A7T0Q1_Nematostella_vectensis
U1M848_Ascaris_suum
0,31
A0A0B2VIN0_Toxocara_canis
C5KPH4_Perkinsus_marinus
C5LWQ3_Perkinsus_marinus
0.4

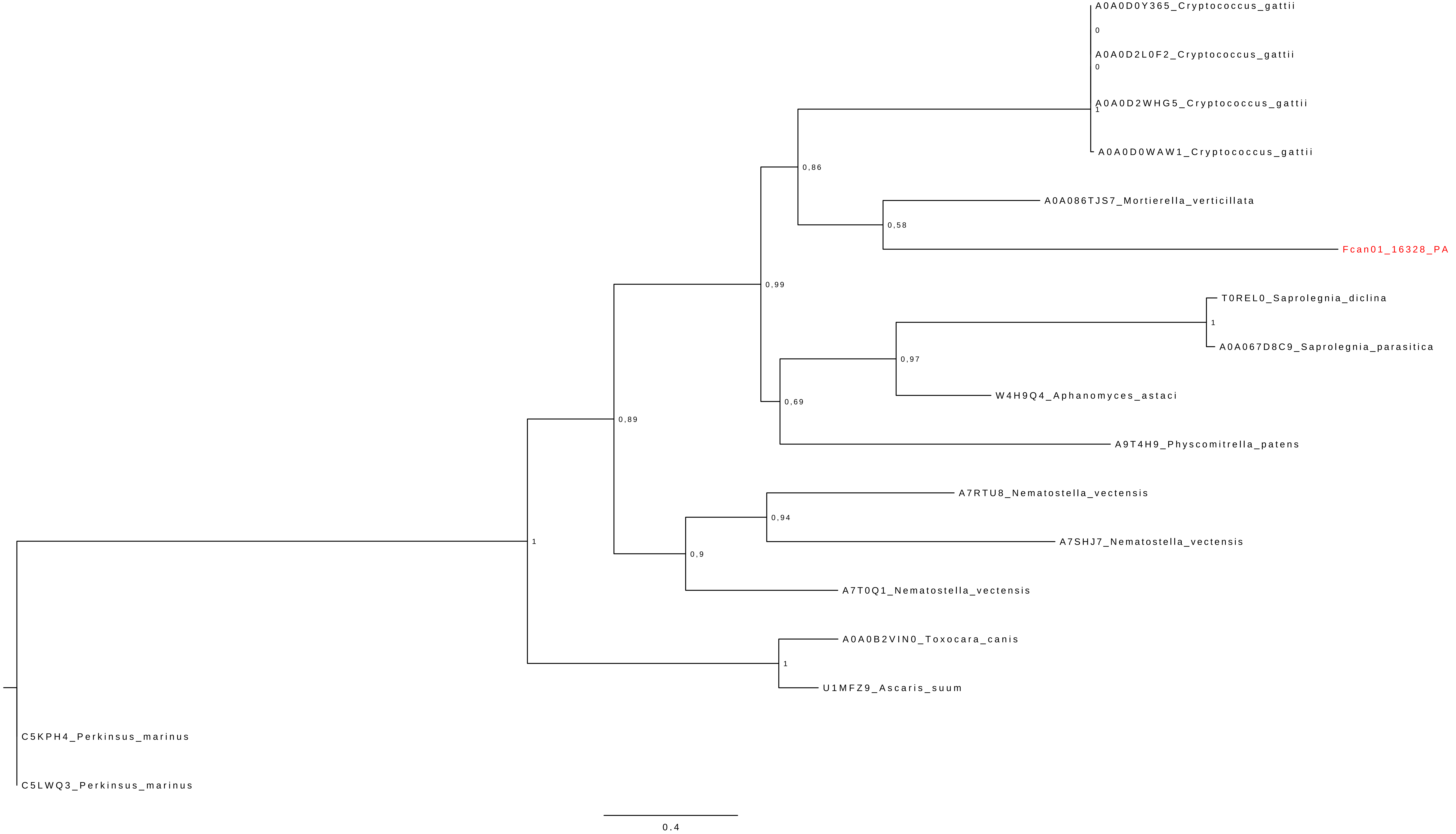
A0A0D0Y365_Cryptococcus_gattii
0
A0A0D2L0F2_Cryptococcus_gattii
0
A0A0D2WHG5_Cryptococcus_gattii
1
A0A0D0WAW1_Cryptococcus_gattii
0,86
A0A086TJS7_Mortierella_verticillata
0,58
Fcan01_16328_PA
0,99
T0REL0_Saprolegnia_diclina
1
A0A067D8C9_Saprolegnia_parasitica
0,97
W4H9Q4_Aphanomyces_astaci
0,69
A9T4H9_Physcomitrella_patens
0,89
A7RTU8_Nematostella_vectensis
0,94
A7SHJ7_Nematostella_vectensis
1
0,9
A7T0Q1_Nematostella_vectensis
A0A0B2VIN0_Toxocara_canis
1
U1MFZ9_Ascaris_suum
C5KPH4_Perkinsus_marinus
C5LWQ3_Perkinsus_marinus
0.4

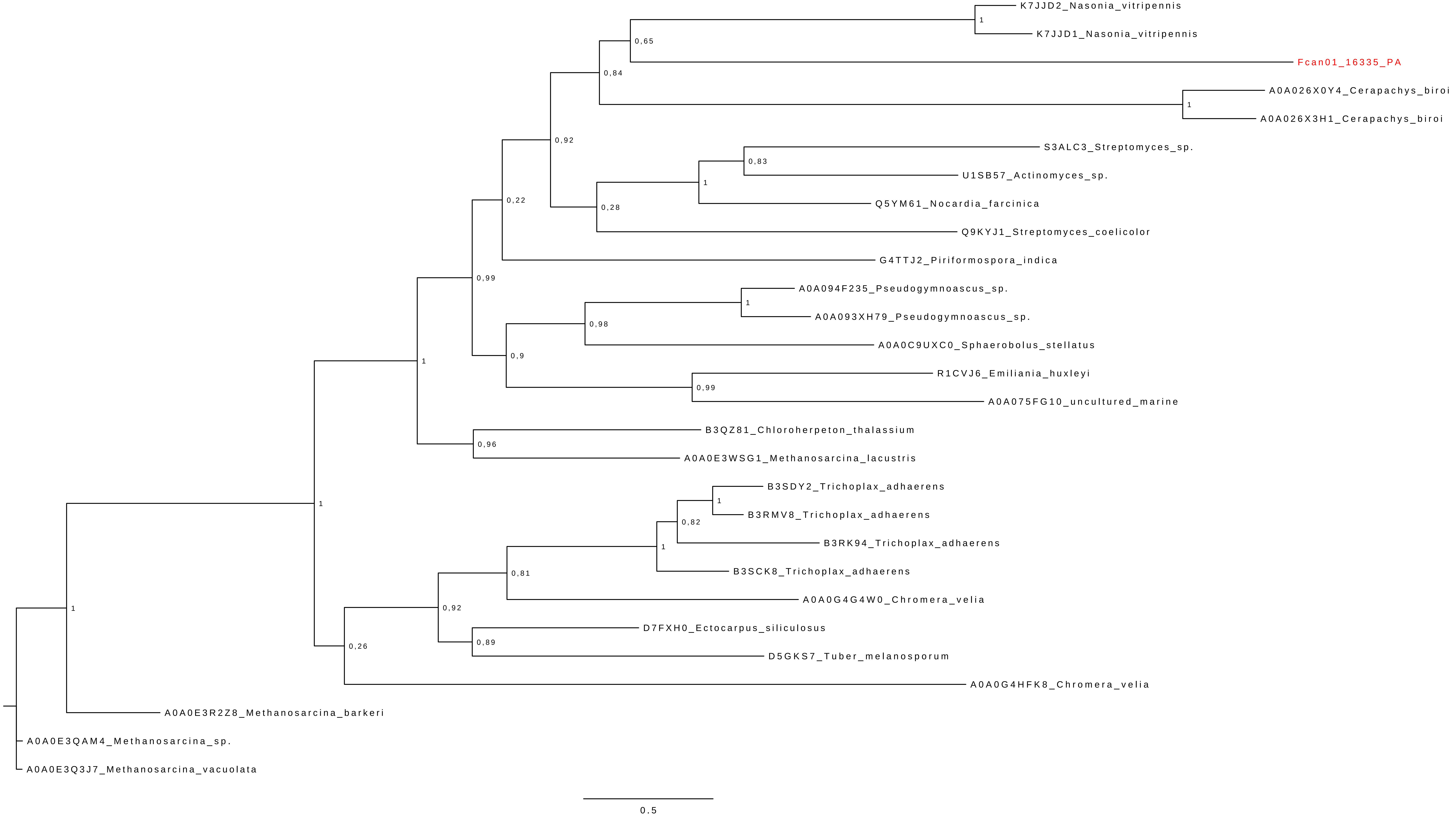
K7JJD2_Nasonia_vitripennis
K7JJD1_Nasonia_vitripennis
Fcan01_16335_PA
A0A026X0Y4_Cerapachys_biroi
A0A026X3H1_Cerapachys_biroi
S3ALC3_Streptomyces_sp.
U1SB57_Actinomyces_sp.
Q5YM61_Nocardia_farcinica
Q9KYJ1_Streptomyces_coelicolor
G4TTJ2_Piriformospora_indica
A0A094F235_Pseudogymnoascus_sp.
A0A093XH79_Pseudogymnoascus_sp.
A0A0C9UXC0_Sphaerobolus_stellatus
R1CVJ6_Emiliania_huxleyi
A0A075FG10_uncultured_marine
B3QZ81_Chloroherpeton_thalassium
A0A0E3WSG1_Methanosarcina_lacustris
B3SDY2_Trichoplax_adhaerens
B3RMV8_Trichoplax_adhaerens
B3RK94_Trichoplax_adhaerens
B3SCK8_Trichoplax_adhaerens
A0A0G4G4W0_Chromera_velia
D7FXH0_Ectocarpus_siliculosus
D5GKS7_Tuber_melanosporum
A0A0G4HFK8_Chromera_velia
A0A0E3R2Z8_Methanosarcina_barkeri
A0A0E3QAM4_Methanosarcina_sp.
A0A0E3Q3J7_Methanosarcina_vacuolata
1
0,65
0,84
1
0,83
0,92
1
0,28
0,22
1
0,99
0,98
0,9
0,99
1
0,96
1
1
0,82
1
0,81
0,92
0,89
0,26
1
0.5

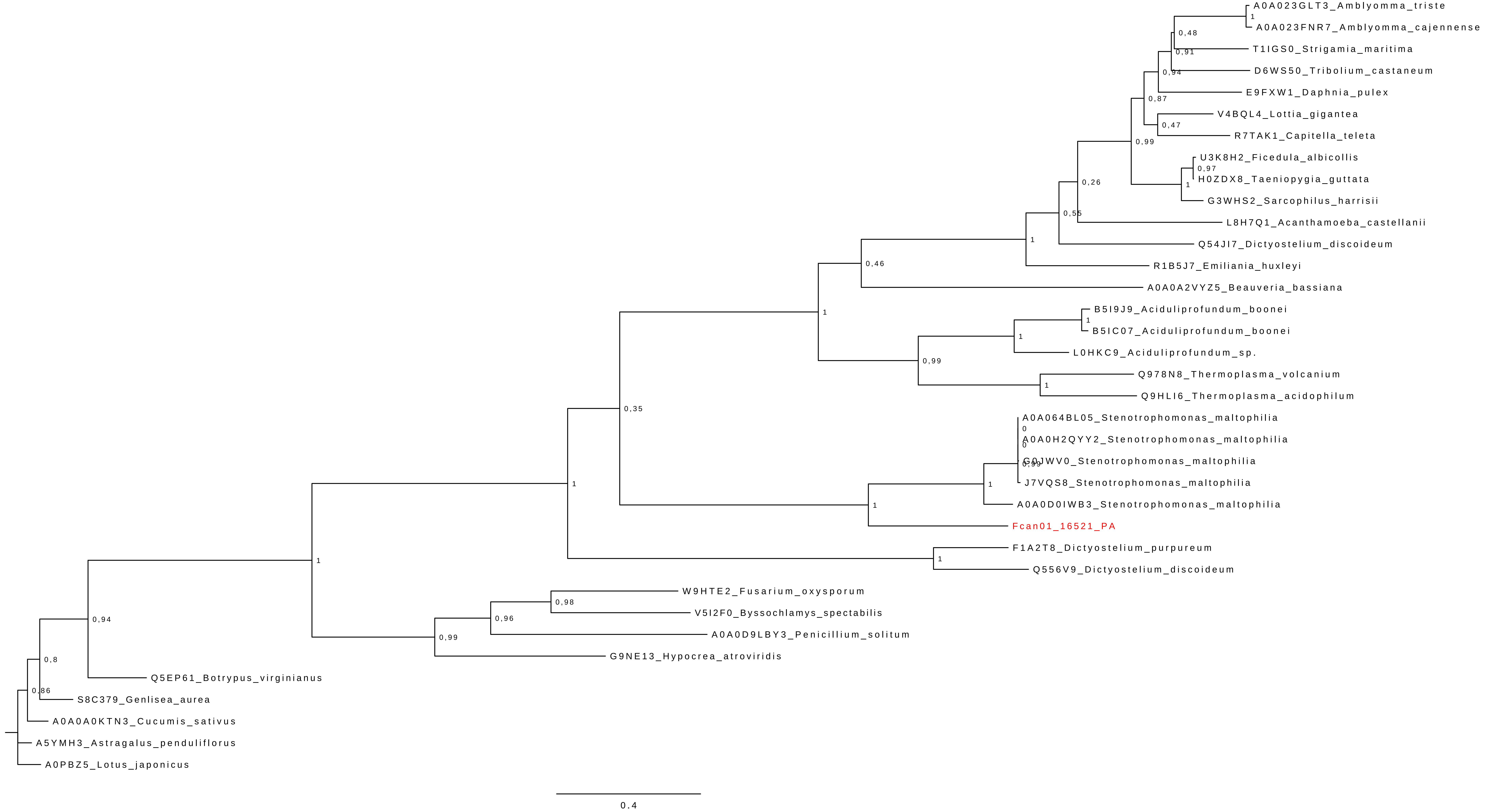
A0A023GLT3_Amblyomma_triste
A0A023FNR7_Amblyomma_cajennense
T1IGS0_Strigamia_maritima
D6WS50_Tribolium_castaneum
E9FXW1_Daphnia_pulex
V4BQL4_Lottia_gigantea
R7TAK1_Capitella_teleta
U3K8H2_Ficedula_albicollis
H0ZDX8_Taeniopygia_guttata
G3WHS2_Sarcophilus_harrisii
L8H7Q1_Acanthamoeba_castellanii
Q54JI7_Dictyostelium_discoideum
R1B5J7_Emiliania_huxleyi
A0A0A2VYZ5_Beauveria_bassiana
B5I9J9_Aciduliprofundum_boonei
B5IC07_Aciduliprofundum_boonei
L0HKC9_Aciduliprofundum_sp.
Q978N8_Thermoplasma_volcanium
Q9HLI6_Thermoplasma_acidophilum
A0A064BL05_Stenotrophomonas_maltophilia
A0A0H2QYY2_Stenotrophomonas_maltophilia
C0JWV0_Stenotrophomonas_maltophilia
J7VQS8_Stenotrophomonas_maltophilia
A0A0D0IWB3_Stenotrophomonas_maltophilia
Fcan01_16521_PA
F1A2T8_Dictyostelium_purpureum
Q556V9_Dictyostelium_discoideum
W9HTE2_Fusarium_oxysporum
V5I2F0_Byssochlamys_spectabilis
A0A0D9LBY3_Penicillium_solitum
G9NE13_Hypocrea_atroviridis
Q5EP61_Botrypus_virginianus
S8C379_Genlisea_aurea
A0A0A0KTN3_Cucumis_sativus
A5YMH3_Astragalus_penduliflorus
A0PBZ5_Lotus_japonicus
0.4

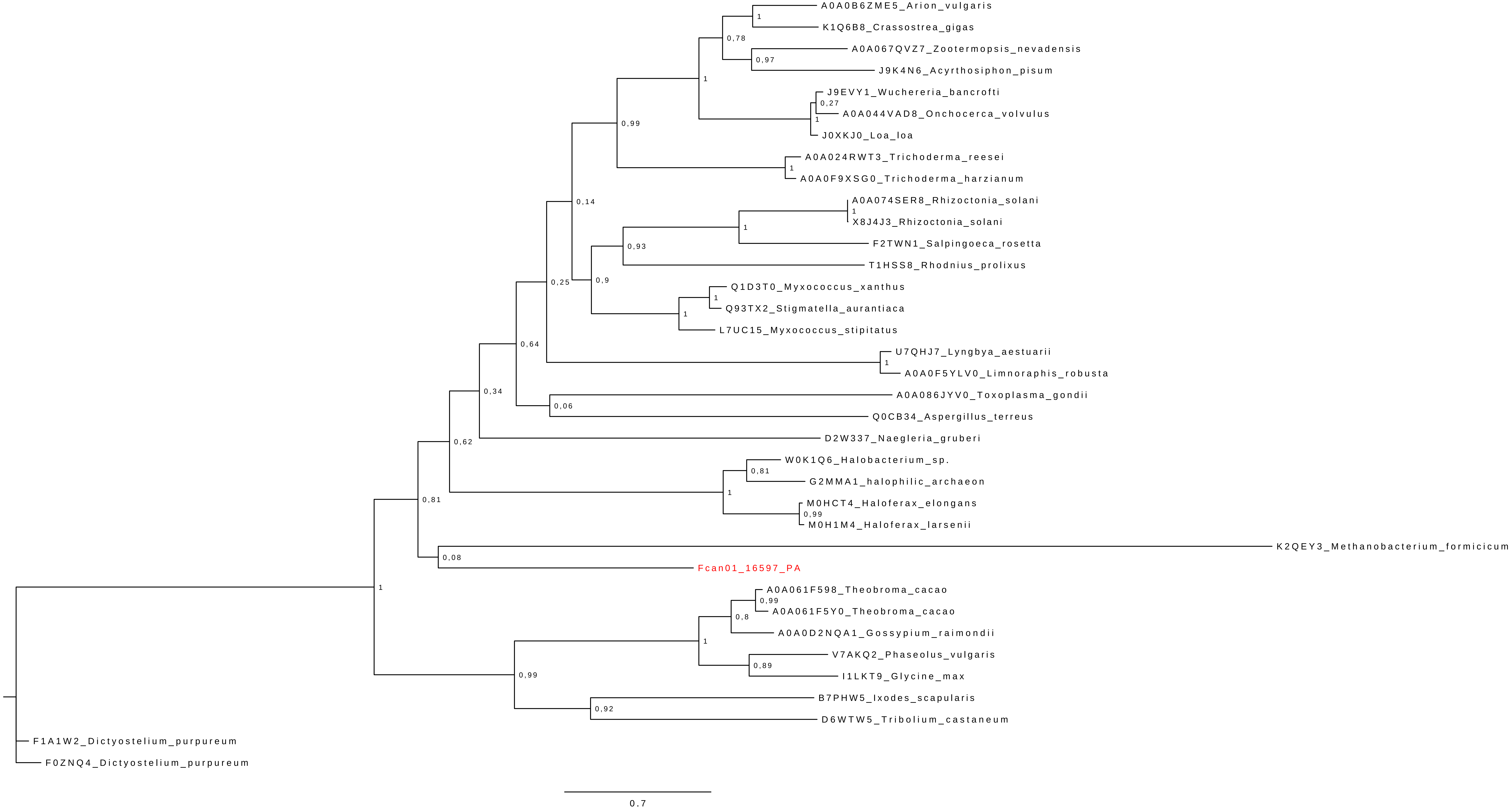
A0A0B6ZME5_Arion_vulgaris
K1Q6B8_Crassostrea_gigas
A0A067QVZ7_Zootermopsis_nevadensis
J9K4N6_Acyrthosiphon_pisum
J9EVY1_Wuchereria_bancrofti
A0A044VAD8_Onchocerca_volvulus
J0XKJ0_Loa_loa
A0A024RWT3_Trichoderma_reesei
A0A0F9XSG0_Trichoderma_harzianum
A0A074SER8_Rhizoctonia_solani
X8J4J3_Rhizoctonia_solani
F2TWN1_Salpingoeca_rosetta
T1HSS8_Rhodnius_prolixus
Q1D3T0_Myxococcus_xanthus
Q93TX2_Stigmatella_aurantiaca
L7UC15_Myxococcus_stipitatus
U7QHJ7_Lyngbya_aestuarii
A0A0F5YLV0_Limnoraphis_robusta
A0A086JYV0_Toxoplasma_gondii
Q0CB34_Aspergillus_terreus
D2W337_Naegleria_gruberi
W0K1Q6_Halobacterium_sp.
G2MMA1_halophilic_archaeon
M0HCT4_Haloferax_elongans
M0H1M4_Haloferax_larsenii
K2QEY3_Methanobacterium_formicicum
Fcan01_16597_PA
A0A061F598_Theobroma_cacao
A0A061F5Y0_Theobroma_cacao
A0A0D2NQA1_Gossypium_raimondii
V7AKQ2_Phaseolus_vulgaris
I1LKT9_Glycine_max
B7PHW5_Ixodes_scapularis
D6WTW5_Tribolium_castaneum
F1A1W2_Dictyostelium_purpureum
F0ZNQ4_Dictyostelium_purpureum
1
0,78
0,97
1
0,27
1
0,99
1
0,14
1
1
0,93
0,25
0,9
1
1
0,64
1
0,34
0,06
0,62
0,81
1
0,81
0,99
0,08
1
0,99
0,8
1
0,89
0,99
0,92
0.7

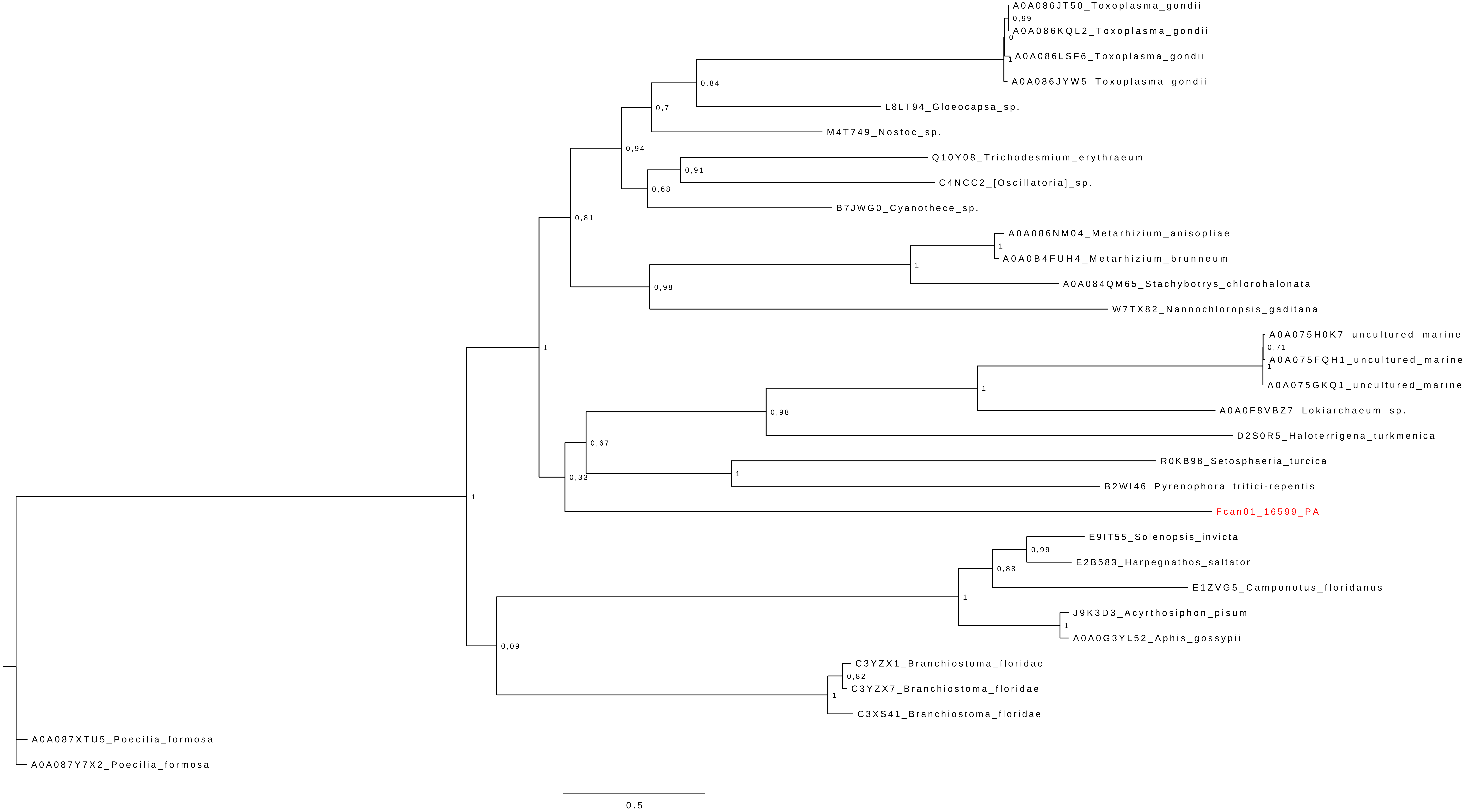

A0A086JT50_Toxoplasma_gondii
0,99
A0A086KQL2_Toxoplasma_gondii
0
A0A086LSF6_Toxoplasma_gondii
1
A0A086JYW5_Toxoplasma_gondii
0,84
L8LT94_Gloeocapsa_sp.
0,7
M4T749_Nostoc_sp.
0,94
Q10Y08_Trichodesmium_erythraeum
0,91
C4NCC2_[Oscillatoria]_sp.
0,68
B7JWG0_Cyanothece_sp.
0,81
A0A086NM04_Metarhizium_anisopliae
1
A0A0B4FUH4_Metarhizium_brunneum
1
A0A084QM65_Stachybotrys_chlorohalonata
0,98
W7TX82_Nannochloropsis_gaditana
1
A0A075H0K7_uncultured_marine
0,71
A0A075FQH1_uncultured_marine
1
A0A075GKQ1_uncultured_marine
1
A0A0F8VBZ7_Lokiarchaeum_sp.
0,98
D2S0R5_Haloterrigena_turkmenica
0,67
R0KB98_Setosphaeria_turcica
1
0,33
B2WI46_Pyrenophora_tritici-repentis
Fcan01_16599_PA
1
E9IT55_Solenopsis_invicta
0,99
E2B583_Harpegnathos_saltator
0,88
E1ZVG5_Camponotus_floridanus
1
J9K3D3_Acyrthosiphon_pisum
1
A0A0G3YL52_Aphis_gossypii
0,09
C3YZX1_Branchiostoma_floridae
0,82
C3YZX7_Branchiostoma_floridae
1
C3XS41_Branchiostoma_floridae
A0A087XTU5_Poecilia_formosa
A0A087Y7X2_Poecilia_formosa
0.5

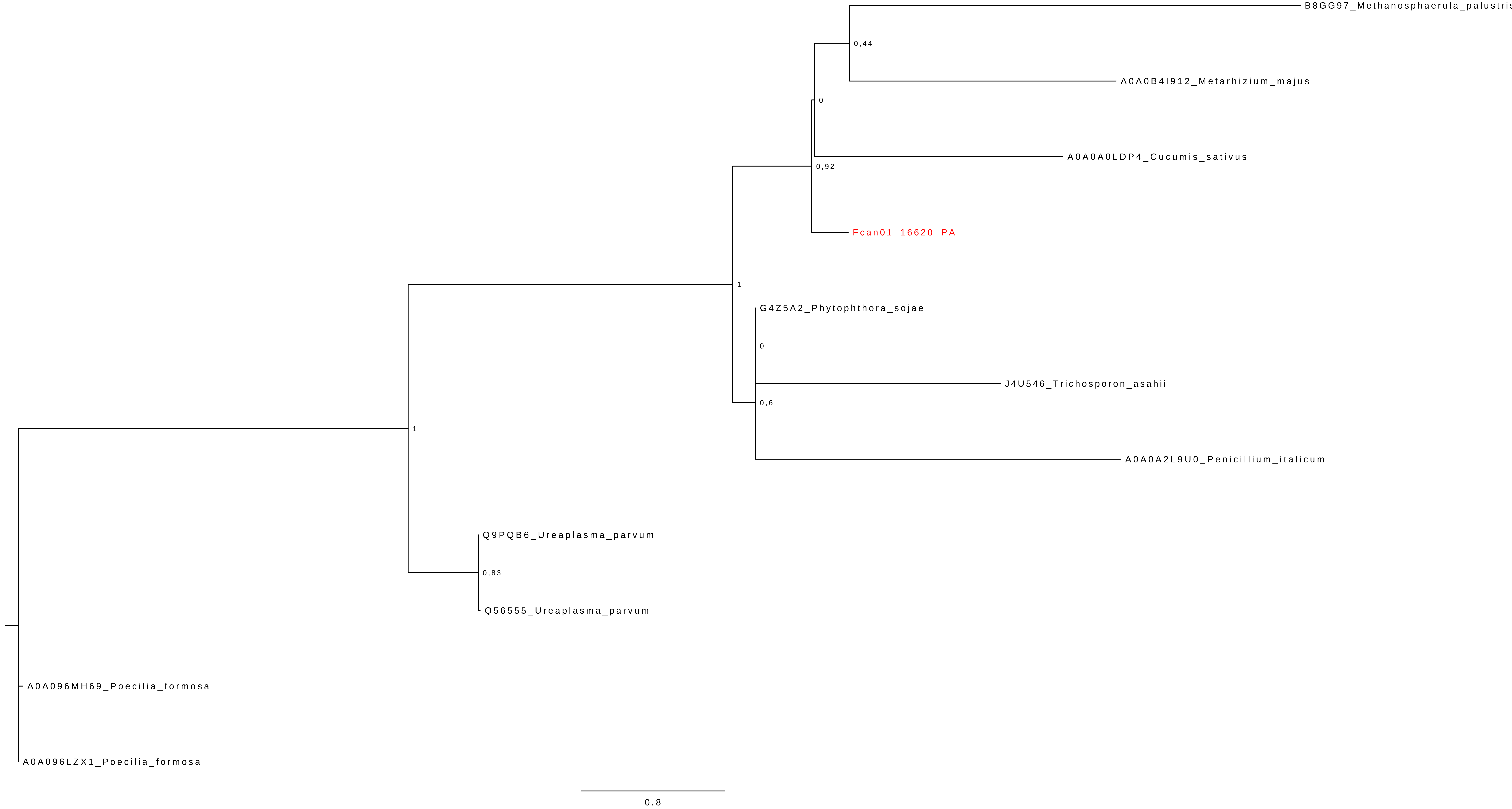
B8GG97_Methanosphaerula_palustris
0,44
A0A0B4I912_Metarhizium_majus
0
A0A0A0LDP4_Cucumis_sativus
0,92
Fcan01_16620_PA
1
G4Z5A2_Phytophthora_sojae
0
J4U546_Trichosporon_asahii
0,6
A0A0A2L9U0_Penicillium_italicum
1
Q9PQB6_Ureaplasma_parvum
0,83
Q56555_Ureaplasma_parvum
A0A096MH69_Poecilia_formosa
A0A096LZX1_Poecilia_formosa
0.8

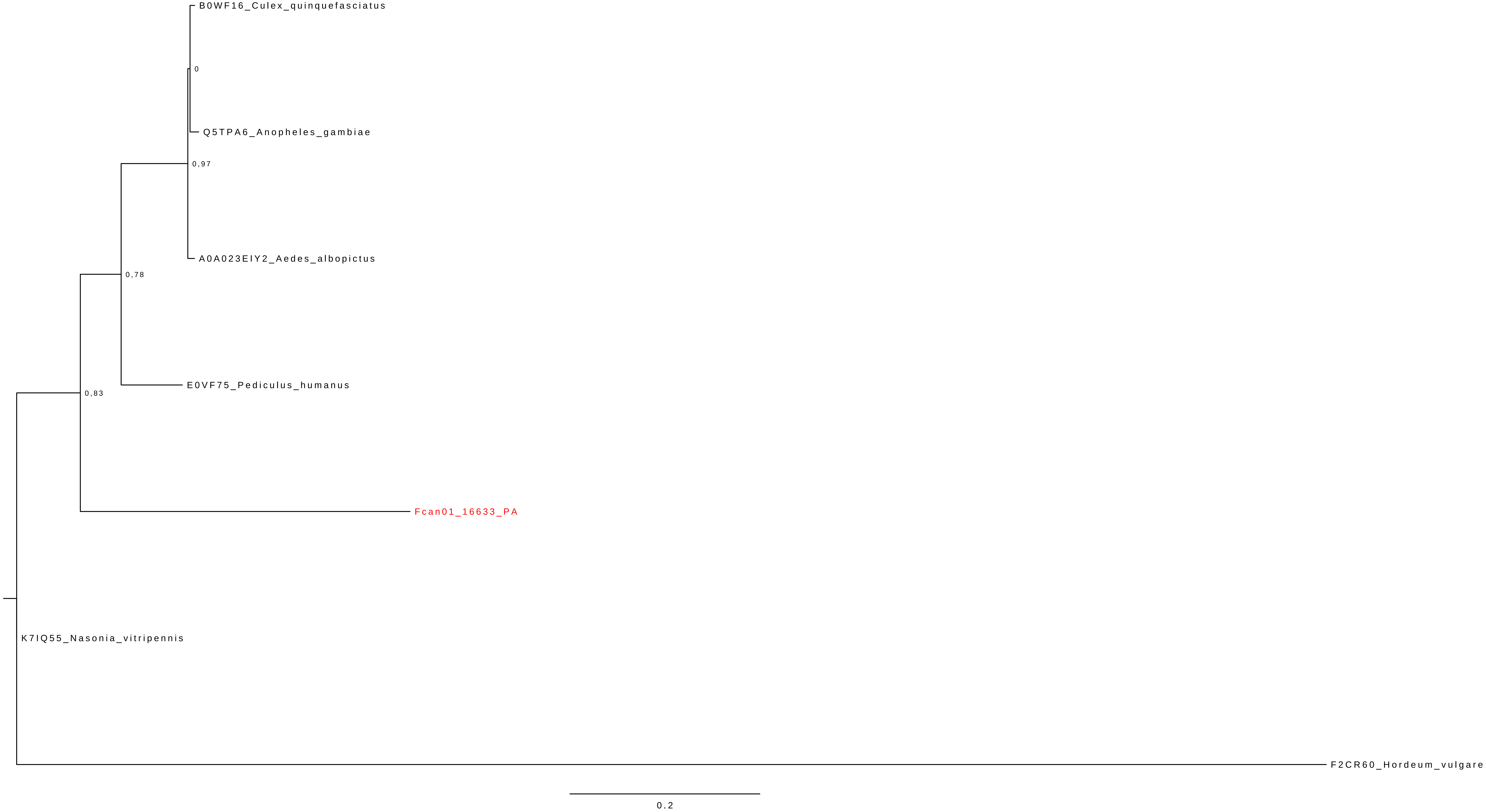
B0WF16_Culex_quinquefasciatus
0
Q5TPA6_Anopheles_gambiae
0,97
A0A023EIY2_Aedes_albopictus
0,78
E0VF75_Pediculus_humanus
0,83
Fcan01_16633_PA
K7IQ55_Nasonia_vitripennis
F2CR60_Hordeum_vulgare
0.2

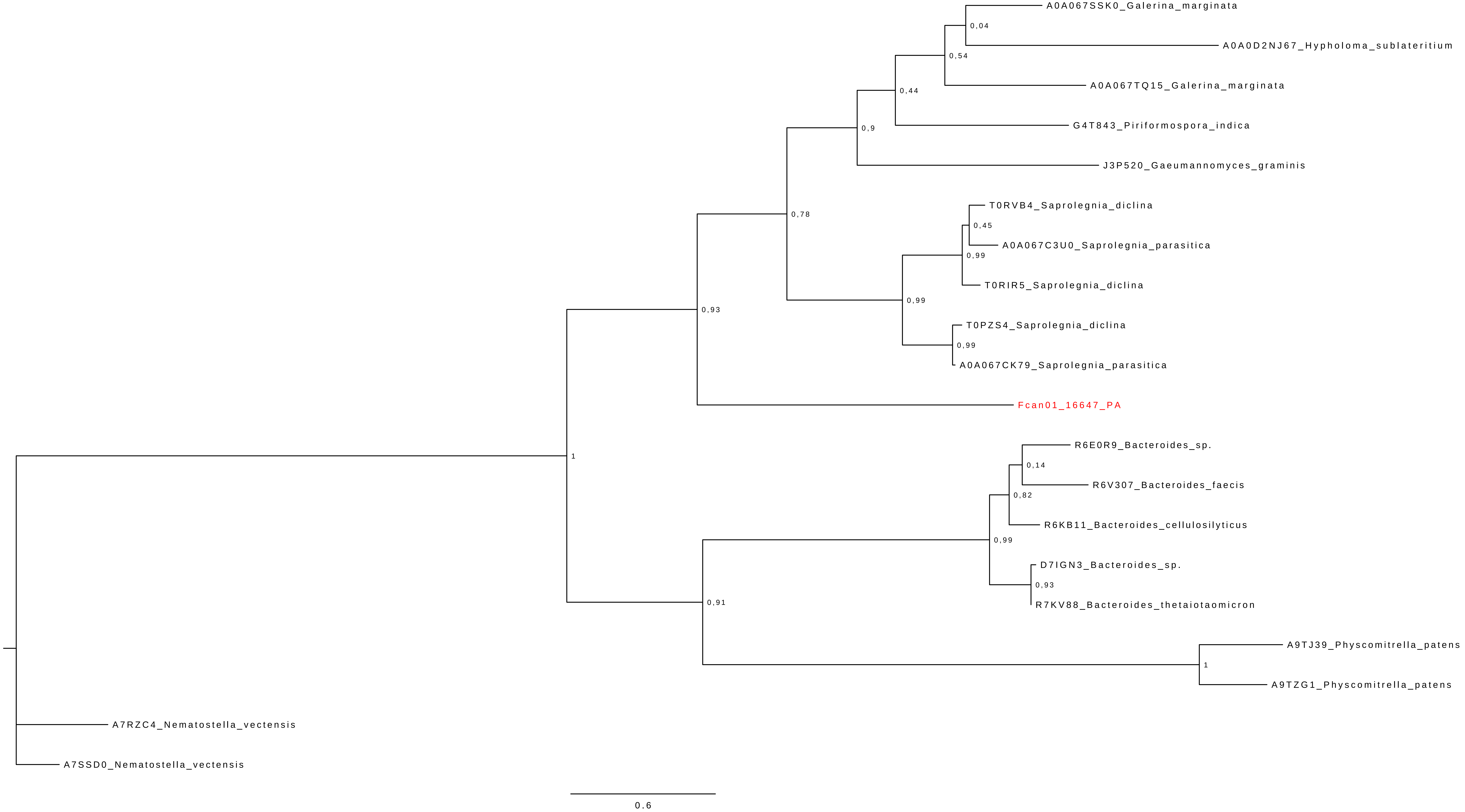
A0A067SSK0_Galerina_marginata
0,04
A0A0D2NJ67_Hypholoma_sublateritium
0,54
A0A067TQ15_Galerina_marginata
0,44
G4T843_Piriformospora_indica
0,9
J3P520_Gaeumannomyces_graminis
T0RVB4_Saprolegnia_diclina
0,45
A0A067C3U0_Saprolegnia_parasitica
0,99
T0RIR5_Saprolegnia_diclina
0,78
0,99
T0PZS4_Saprolegnia_diclina
0,99
A0A067CK79_Saprolegnia_parasitica
0,93
Fcan01_16647_PA
R6E0R9_Bacteroides_sp.
0,14
R6V307_Bacteroides_faecis
0,82
R6KB11_Bacteroides_cellulosilyticus
1
0,99
D7IGN3_Bacteroides_sp.
0,93
R7KV88_Bacteroides_thetaiotaomicron
0,91
A9TJ39_Physcomitrella_patens
1
A9TZG1_Physcomitrella_patens
A7RZC4_Nematostella_vectensis
A7SSD0_Nematostella_vectensis
0.6

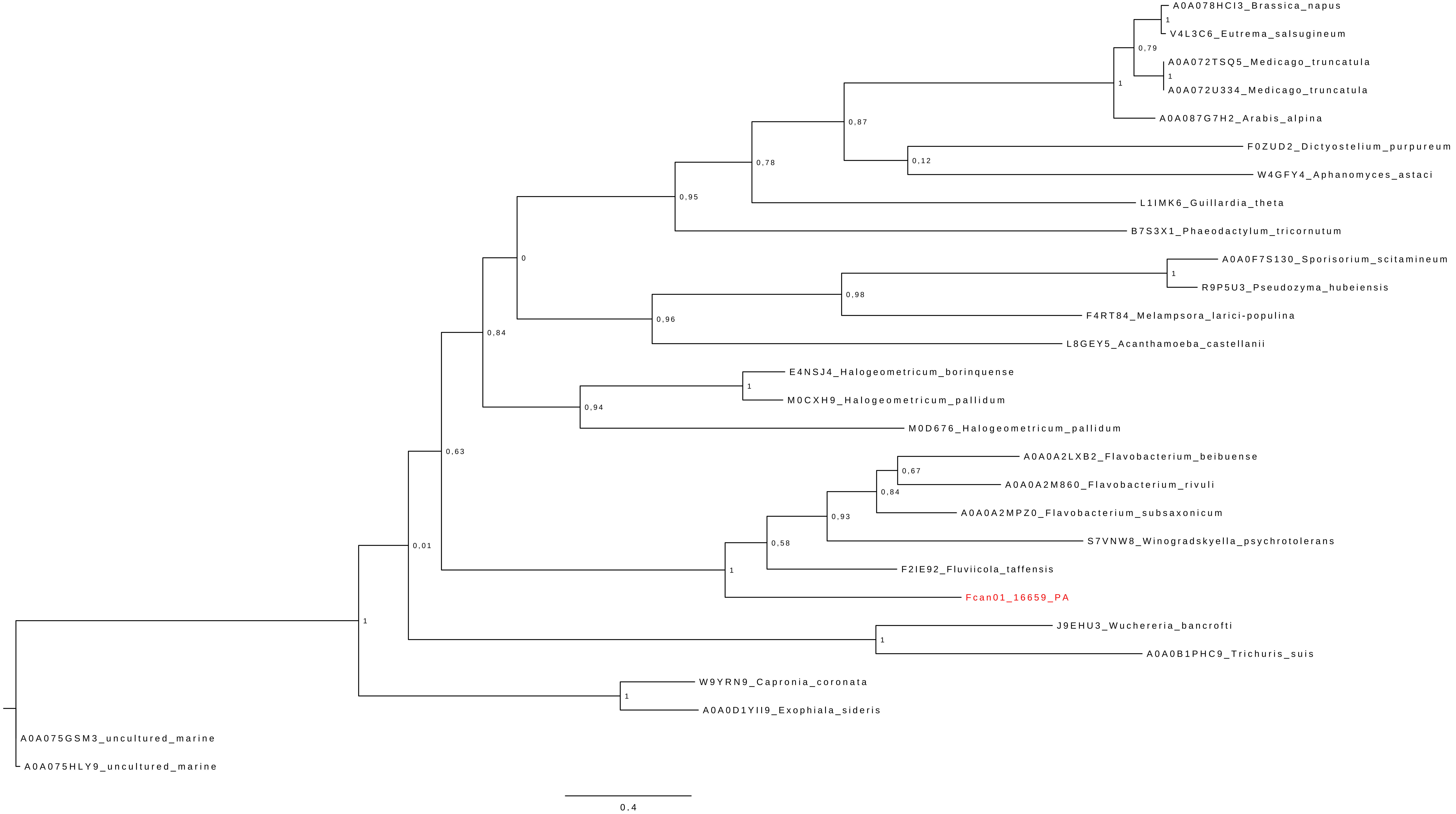

A0A078HCI3_Brassica_napus
1
V4L3C6_Eutrema_salsugineum
0,79
A0A072TSQ5_Medicago_truncatula
1
1
A0A072U334_Medicago_truncatula
A0A087G7H2_Arabis_alpina
0,87
F0ZUD2_Dictyostelium_purpureum
0,12
W4GFY4_Aphanomyces_astaci
0,78
L1IMK6_Guillardia_theta
0,95
B7S3X1_Phaeodactylum_tricornutum
0
A0A0F7S130_Sporisorium_scitamineum
1
R9P5U3_Pseudozyma_hubeiensis
0,98
F4RT84_Melampsora_larici-populina
0,96
L8GEY5_Acanthamoeba_castellanii
0,84
E4NSJ4_Halogeometricum_borinquense
1
M0CXH9_Halogeometricum_pallidum
0,94
M0D676_Halogeometricum_pallidum
0,63
A0A0A2LXB2_Flavobacterium_beibuense
0,67
A0A0A2M860_Flavobacterium_rivuli
0,84
A0A0A2MPZ0_Flavobacterium_subsaxonicum
0,93
S7VNW8_Winogradskyella_psychrotolerans
0,58
F2IE92_Fluviicola_taffensis
0,01
1
Fcan01_16659_PA
1
J9EHU3_Wuchereria_bancrofti
1
A0A0B1PHC9_Trichuris_suis
W9YRN9_Capronia_coronata
1
A0A0D1YII9_Exophiala_sideris
A0A075GSM3_uncultured_marine
A0A075HLY9_uncultured_marine
0.4

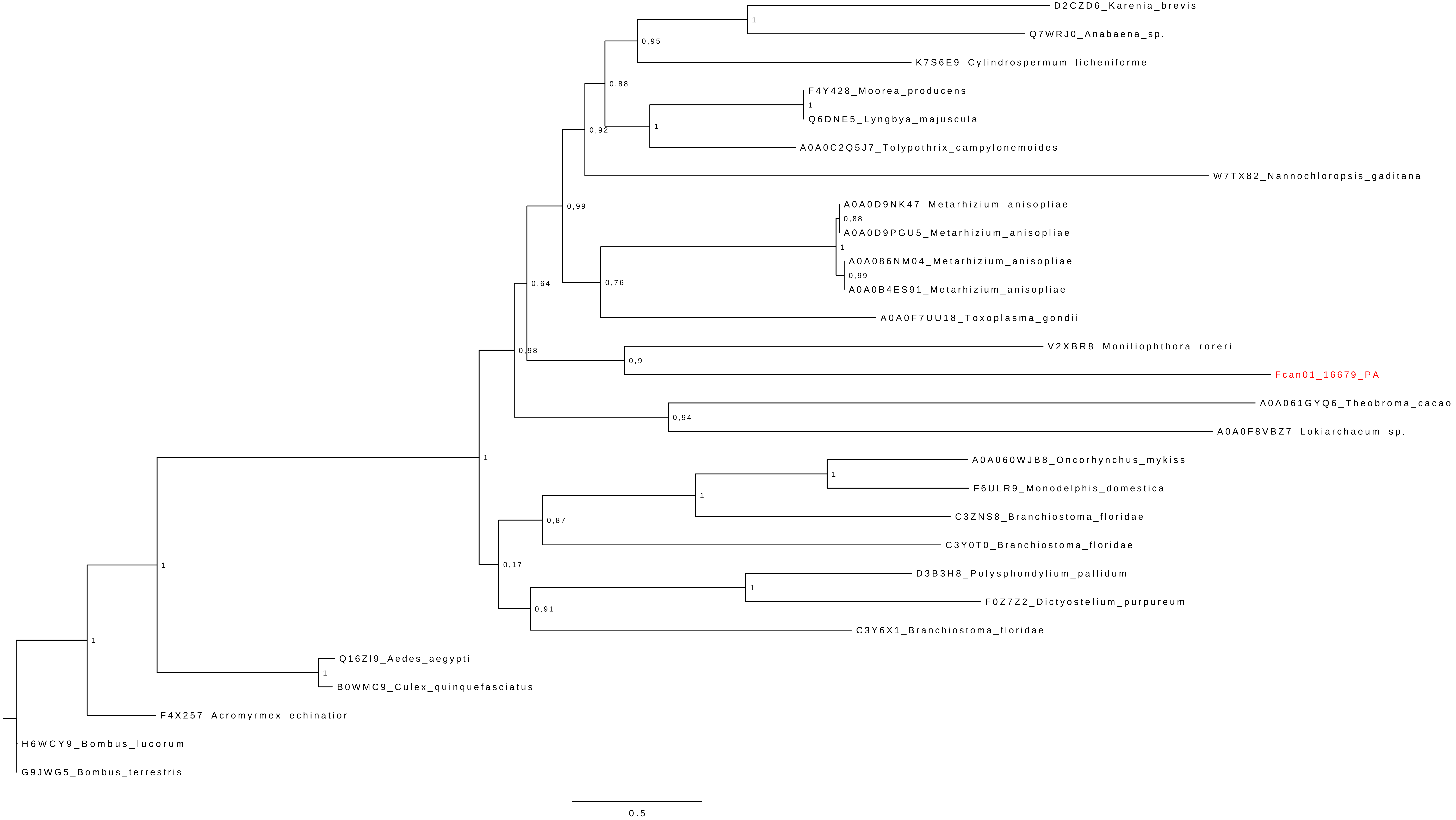
D2CZD6_Karenia_brevis
Q7WRJ0_Anabaena_sp.
K7S6E9_Cylindrospermum_licheniforme
F4Y428_Moorea_producens
Q6DNE5_Lyngbya_majuscula
A0A0C2Q5J7_Tolypothrix_campylonemoides
W7TX82_Nannochloropsis_gaditana
A0A0D9NK47_Metarhizium_anisopliae
A0A0D9PGU5_Metarhizium_anisopliae
A0A086NM04_Metarhizium_anisopliae
A0A0B4ES91_Metarhizium_anisopliae
A0A0F7UU18_Toxoplasma_gondii
V2XBR8_Moniliophthora_roreri
Fcan01_16679_PA
A0A061GYQ6_Theobroma_cacao
A0A0F8VBZ7_Lokiarchaeum_sp.
A0A060WJB8_Oncorhynchus_mykiss
F6ULR9_Monodelphis_domestica
C3ZNS8_Branchiostoma_floridae
C3Y0T0_Branchiostoma_floridae
D3B3H8_Polysphondylium_pallidum
F0Z7Z2_Dictyostelium_purpureum
C3Y6X1_Branchiostoma_floridae
Q16ZI9_Aedes_aegypti
B0WMC9_Culex_quinquefasciatus
F4X257_Acromyrmex_echinatior
H6WCY9_Bombus_lucorum
G9JWG5_Bombus_terrestris
0.5

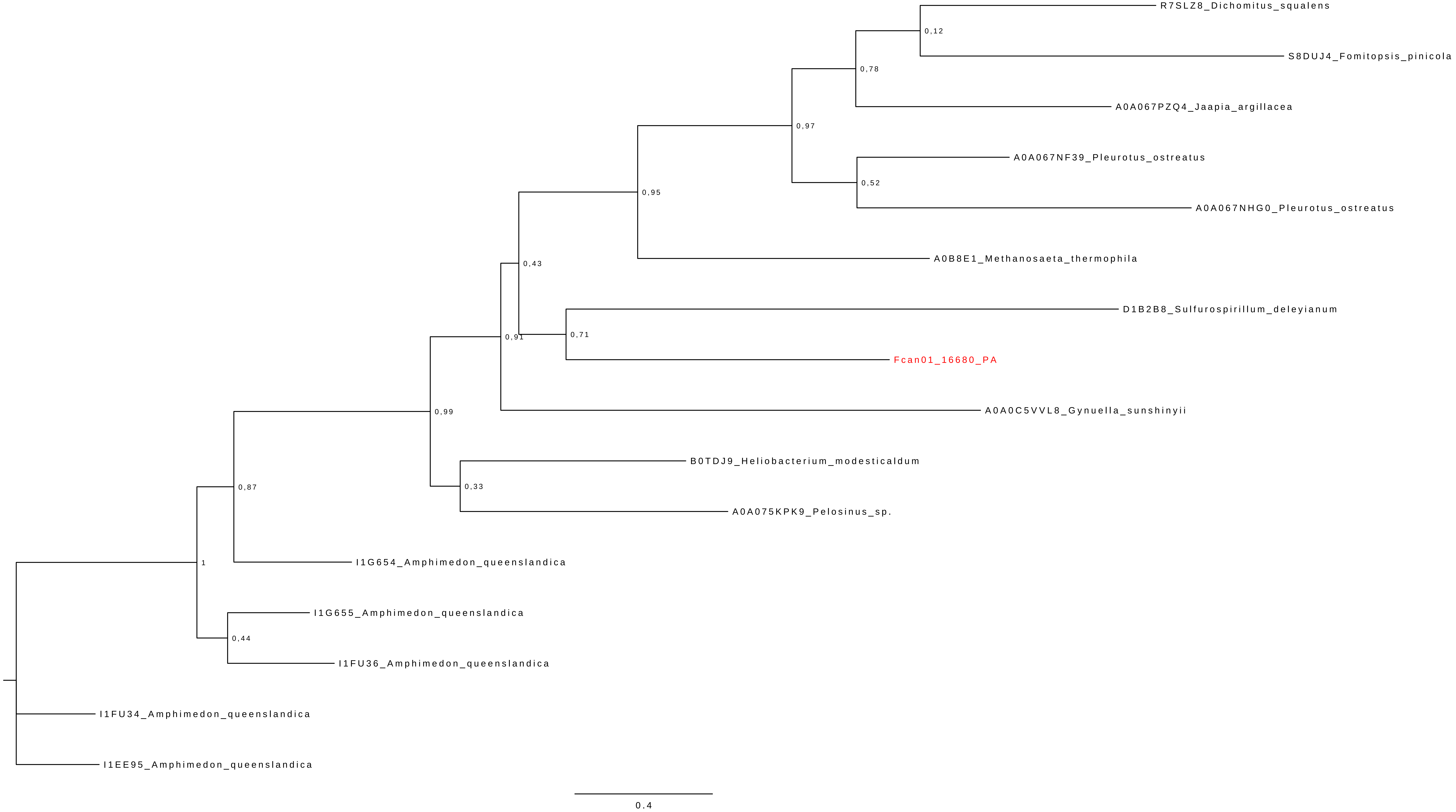
R7SLZ8_Dichomitus_squalens
0,12
S8DUJ4_Fomitopsis_pinicola
0,78
A0A067PZQ4_Jaapia_argillacea
0,97
A0A067NF39_Pleurotus_ostreatus
0,52
A0A067NHG0_Pleurotus_ostreatus
0,95
A0B8E1_Methanosaeta_thermophila
0,43
D1B2B8_Sulfurospirillum_deleyianum
0,71
0,91
Fcan01_16680_PA
A0A0C5VVL8_Gynuella_sunshinyii
0,99
B0TDJ9_Heliobacterium_modesticaldum
0,33
A0A075KPK9_Pelosinus_sp.
0,87
I1G654_Amphimedon_queenslandica
1
I1G655_Amphimedon_queenslandica
0,44
I1FU36_Amphimedon_queenslandica
I1FU34_Amphimedon_queenslandica
I1EE95_Amphimedon_queenslandica
0.4

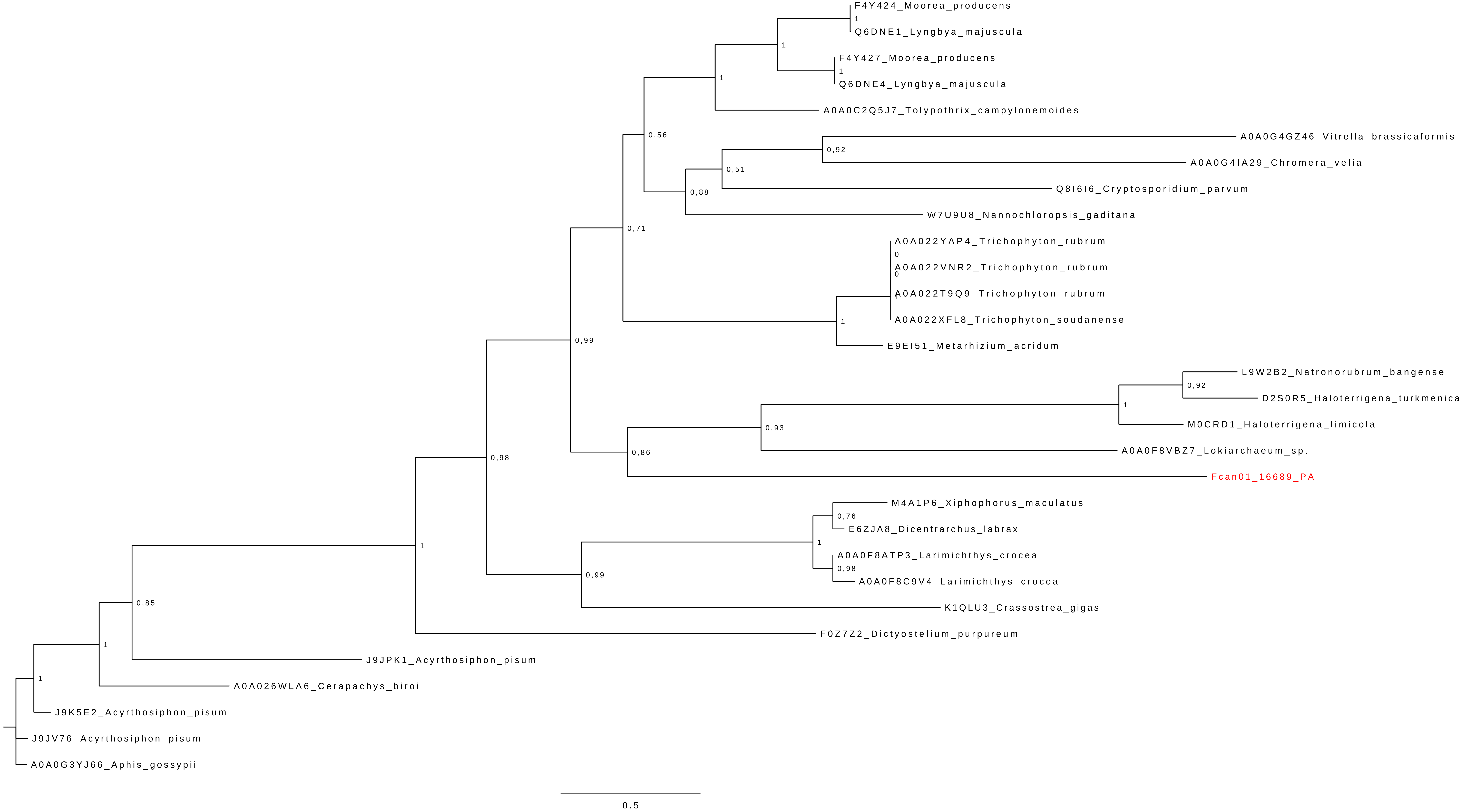
F4Y424_Moorea_producens
1
Q6DNE1_Lyngbya_majuscula
1
F4Y427_Moorea_producens
1
Q6DNE4_Lyngbya_majuscula
1
A0A0C2Q5J7_Tolypothrix_campylonemoides
0,56
A0A0G4GZ46_Vitrella_brassicaformis
0,92
A0A0G4IA29_Chromera_velia
0,51
Q8I6I6_Cryptosporidium_parvum
0,88
W7U9U8_Nannochloropsis_gaditana
0,71
A0A022YAP4_Trichophyton_rubrum
0
A0A022VNR2_Trichophyton_rubrum
0
A0A022T9Q9_Trichophyton_rubrum
1
A0A022XFL8_Trichophyton_soudanense
1
E9EI51_Metarhizium_acridum
0,99
L9W2B2_Natronorubrum_bangense
0,92
D2S0R5_Haloterrigena_turkmenica
1
M0CRD1_Haloterrigena_limicola
0,93
A0A0F8VBZ7_Lokiarchaeum_sp.
0,86
Fcan01_16689_PA
0,98
M4A1P6_Xiphophorus_maculatus
0,76
E6ZJA8_Dicentrarchus_labrax
1
A0A0F8ATP3_Larimichthys_crocea
0,98
A0A0F8C9V4_Larimichthys_crocea
0,99
K1QLU3_Crassostrea_gigas
1
F0Z7Z2_Dictyostelium_purpureum
0,85
J9JPK1_Acyrthosiphon_pisum
1
A0A026WLA6_Cerapachys_biroi
1
J9K5E2_Acyrthosiphon_pisum
J9JV76_Acyrthosiphon_pisum
A0A0G3YJ66_Aphis_gossypii
0.5

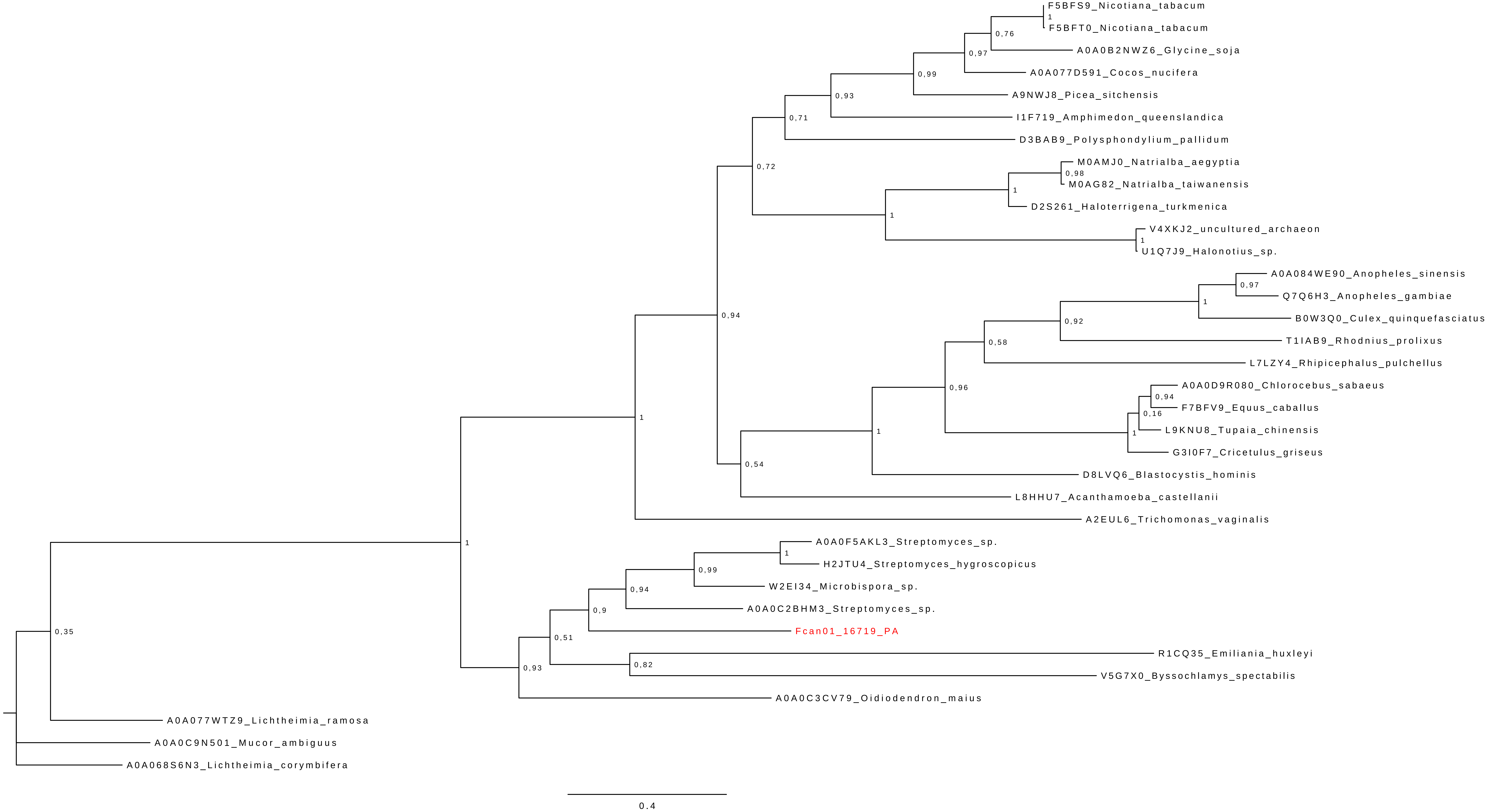
F5BFS9_Nicotiana_tabacum
1
F5BFT0_Nicotiana_tabacum
0,76
A0A0B2NWZ6_Glycine_soja
0,97
A0A077D591_Cocos_nucifera
0,99
A9NWJ8_Picea_sitchensis
0,93
I1F719_Amphimedon_queenslandica
0,71
D3BAB9_Polysphondylium_pallidum
M0AMJ0_Natrialba_aegyptia
0,98
M0AG82_Natrialba_taiwanensis
1
D2S261_Haloterrigena_turkmenica
0,72
1
V4XKJ2_uncultured_archaeon
1
U1Q7J9_Halonotius_sp.
A0A084WE90_Anopheles_sinensis
0,97
Q7Q6H3_Anopheles_gambiae
1
B0W3Q0_Culex_quinquefasciatus
0,94
0,92
T1IAB9_Rhodnius_prolixus
0,58
L7LZY4_Rhipicephalus_pulchellus
A0A0D9R080_Chlorocebus_sabaeus
0,96
0,94
F7BFV9_Equus_caballus
0,16
1
L9KNU8_Tupaia_chinensis
1
1
G3I0F7_Cricetulus_griseus
0,54
D8LVQ6_Blastocystis_hominis
L8HHU7_Acanthamoeba_castellanii
A2EUL6_Trichomonas_vaginalis
1
A0A0F5AKL3_Streptomyces_sp.
1
H2JTU4_Streptomyces_hygroscopicus
0,99
W2EI34_Microbispora_sp.
0,94
0,9
A0A0C2BHM3_Streptomyces_sp.
0,35
0,51
Fcan01_16719_PA
R1CQ35_Emiliania_huxleyi
0,93
0,82
V5G7X0_Byssochlamys_spectabilis
A0A0C3CV79_Oidiodendron_maius
A0A077WTZ9_Lichtheimia_ramosa
A0A0C9N501_Mucor_ambiguus
A0A068S6N3_Lichtheimia_corymbifera
0.4

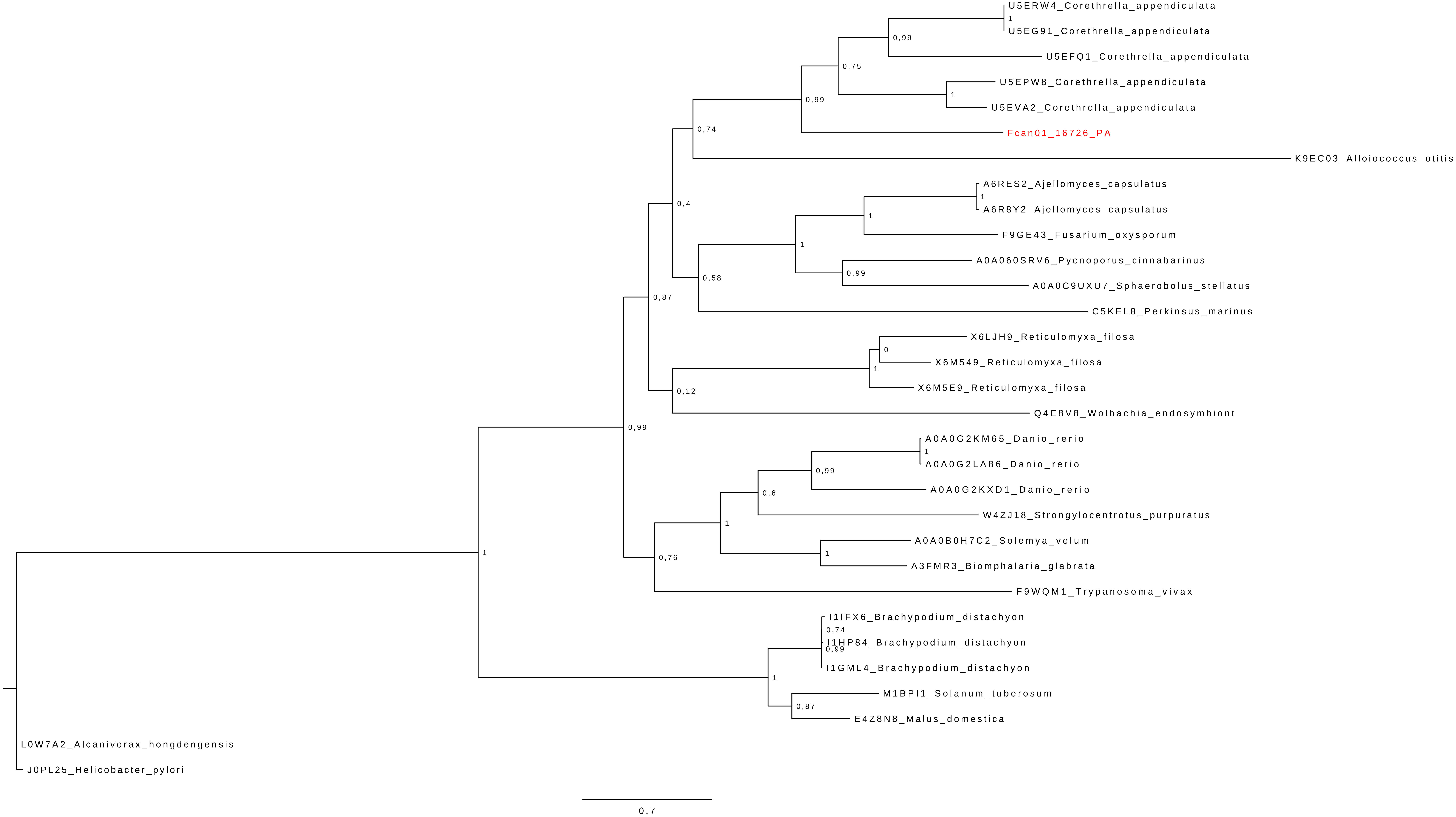
U5ERW4_Corethrella_appendiculata
1
U5EG91_Corethrella_appendiculata
0,99
U5EFQ1_Corethrella_appendiculata
0,75
U5EPW8_Corethrella_appendiculata
1
U5EVA2_Corethrella_appendiculata
0,99
Fcan01_16726_PA
0,74
K9EC03_Alloiococcus_otitis
A6RES2_Ajellomyces_capsulatus
1
A6R8Y2_Ajellomyces_capsulatus
1
F9GE43_Fusarium_oxysporum
0,4
1
A0A060SRV6_Pycnoporus_cinnabarinus
0,99
A0A0C9UXU7_Sphaerobolus_stellatus
0,58
C5KEL8_Perkinsus_marinus
0,87
X6LJH9_Reticulomyxa_filosa
0
X6M549_Reticulomyxa_filosa
1
X6M5E9_Reticulomyxa_filosa
0,12
Q4E8V8_Wolbachia_endosymbiont
0,99
A0A0G2KM65_Danio_rerio
1
A0A0G2LA86_Danio_rerio
0,99
A0A0G2KXD1_Danio_rerio
0,6
W4ZJ18_Strongylocentrotus_purpuratus
1
A0A0B0H7C2_Solemya_velum
1
A3FMR3_Biomphalaria_glabrata
0,76
F9WQM1_Trypanosoma_vivax
1
I1IFX6_Brachypodium_distachyon
0,74
I1HP84_Brachypodium_distachyon
0,99
I1GML4_Brachypodium_distachyon
1
M1BPI1_Solanum_tuberosum
0,87
E4Z8N8_Malus_domestica
L0W7A2_Alcanivorax_hongdengensis
J0PL25_Helicobacter_pylori
0.7

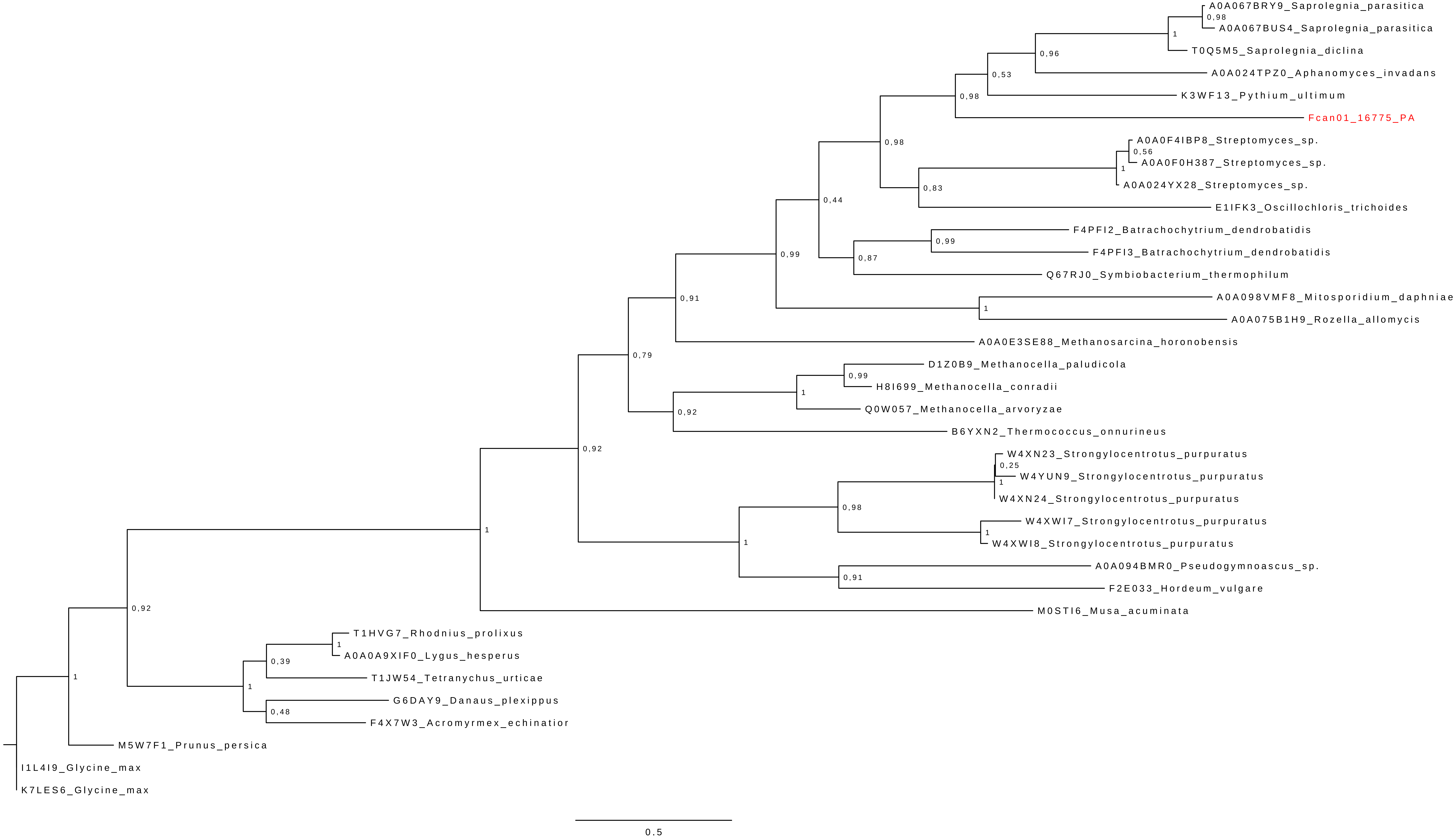

A0A067BRY9_Saprolegnia_parasitica
0,98
A0A067BUS4_Saprolegnia_parasitica
1
T0Q5M5_Saprolegnia_diclina
0,96
A0A024TPZ0_Aphanomyces_invadans
0,53
K3WF13_Pythium_ultimum
0,98
Fcan01_16775_PA
0,98
A0A0F4IBP8_Streptomyces_sp.
0,56
A0A0F0H387_Streptomyces_sp.
1
A0A024YX28_Streptomyces_sp.
0,83
E1IFK3_Oscillochloris_trichoides
0,44
F4PFI2_Batrachochytrium_dendrobatidis
0,99
F4PFI3_Batrachochytrium_dendrobatidis
0,87
Q67RJ0_Symbiobacterium_thermophilum
0,99
A0A098VMF8_Mitosporidium_daphniae
1
A0A075B1H9_Rozella_allomycis
0,91
A0A0E3SE88_Methanosarcina_horonobensis
0,79
D1Z0B9_Methanocella_paludicola
0,99
H8I699_Methanocella_conradii
1
Q0W057_Methanocella_arvoryzae
0,92
B6YXN2_Thermococcus_onnurineus
0,92
W4XN23_Strongylocentrotus_purpuratus
0,25
W4YUN9_Strongylocentrotus_purpuratus
1
W4XN24_Strongylocentrotus_purpuratus
0,98
W4XWI7_Strongylocentrotus_purpuratus
1
W4XWI8_Strongylocentrotus_purpuratus
1
1
A0A094BMR0_Pseudogymnoascus_sp.
0,91
F2E033_Hordeum_vulgare
M0STI6_Musa_acuminata
0,92
T1HVG7_Rhodnius_prolixus
1
A0A0A9XIF0_Lygus_hesperus
0,39
T1JW54_Tetranychus_urticae
1
G6DAY9_Danaus_plexippus
0,48
F4X7W3_Acromyrmex_echinatior
1
M5W7F1_Prunus_persica
I1L4I9_Glycine_max
K7LES6_Glycine_max
0.5

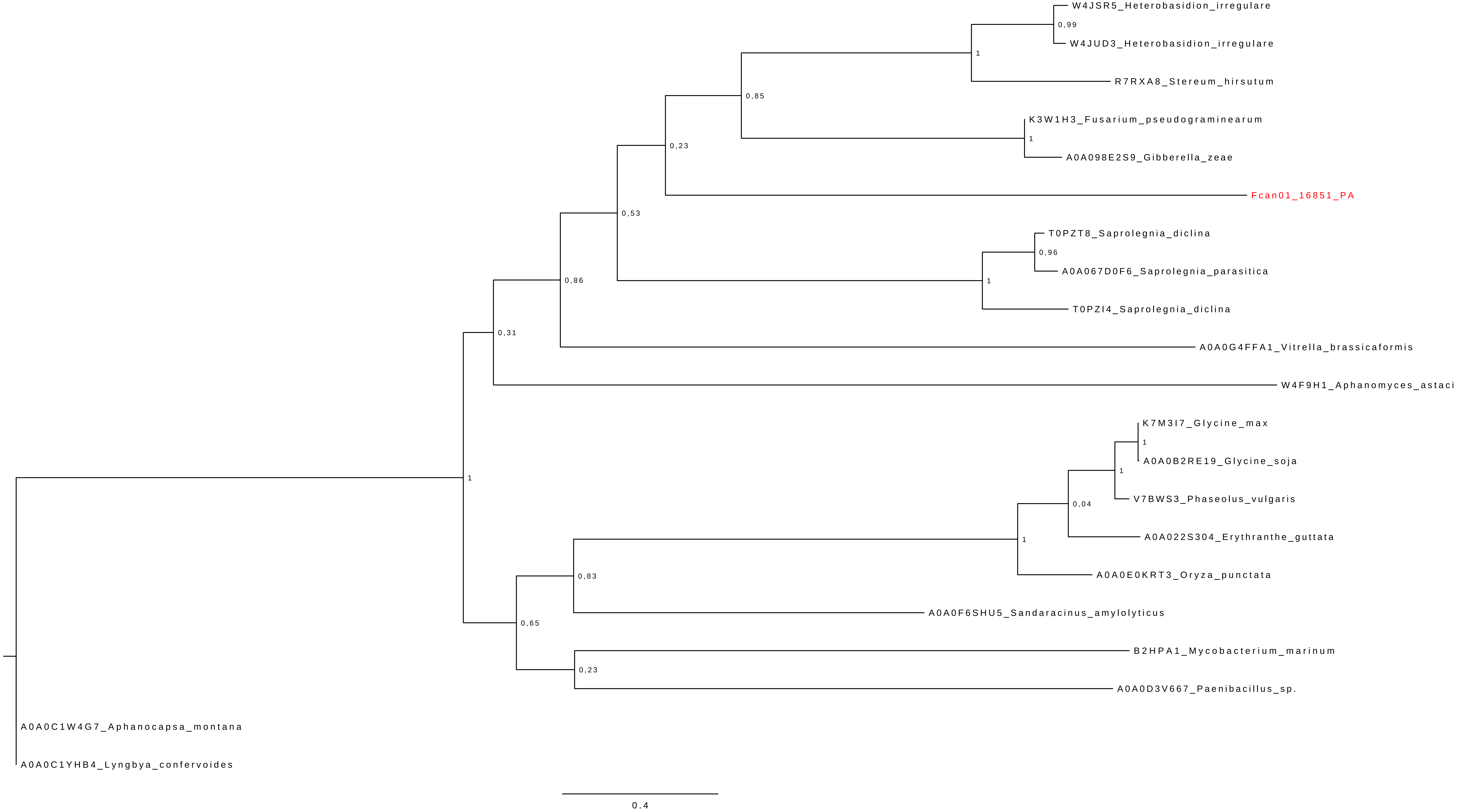

W4JSR5_Heterobasidion_irregulare
0,99
W4JUD3_Heterobasidion_irregulare
1
R7RXA8_Stereum_hirsutum
0,85
K3W1H3_Fusarium_pseudograminearum
1
A0A098E2S9_Gibberella_zeae
0,23
Fcan01_16851_PA
0,53
T0PZT8_Saprolegnia_diclina
0,96
A0A067D0F6_Saprolegnia_parasitica
0,86
1
T0PZI4_Saprolegnia_diclina
0,31
A0A0G4FFA1_Vitrella_brassicaformis
W4F9H1_Aphanomyces_astaci
K7M3I7_Glycine_max
1
A0A0B2RE19_Glycine_soja
1
1
V7BWS3_Phaseolus_vulgaris
0,04
A0A022S304_Erythranthe_guttata
1
A0A0E0KRT3_Oryza_punctata
0,83
A0A0F6SHU5_Sandaracinus_amylolyticus
0,65
B2HPA1_Mycobacterium_marinum
0,23
A0A0D3V667_Paenibacillus_sp.
A0A0C1W4G7_Aphanocapsa_montana
A0A0C1YHB4_Lyngbya_confervoides
0.4

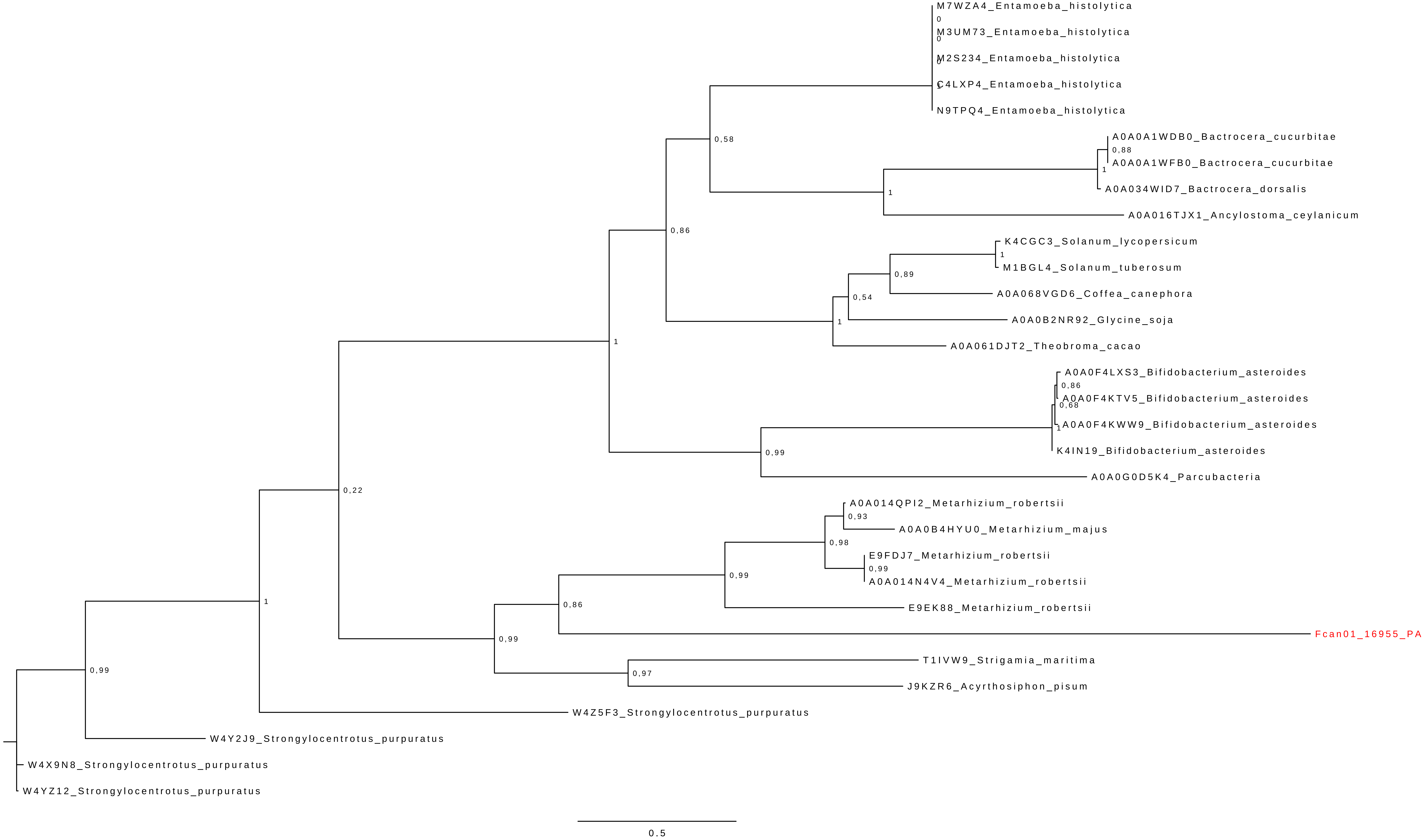
M7WZA4_Entamoeba_histolytica
M3UM73_Entamoeba_histolytica
M2S234_Entamoeba_histolytica
C4LXP4_Entamoeba_histolytica
N9TPQ4_Entamoeba_histolytica
A0A0A1WDB0_Bactrocera_cucurbitae
A0A0A1WFB0_Bactrocera_cucurbitae
A0A034WID7_Bactrocera_dorsalis
A0A016TJX1_Ancylostoma_ceylanicum
K4CGC3_Solanum_lycopersicum
M1BGL4_Solanum_tuberosum
A0A068VGD6_Coffea_canephora
A0A0B2NR92_Glycine_soja
A0A061DJT2_Theobroma_cacao
A0A0F4LXS3_Bifidobacterium_asteroides
A0A0F4KTV5_Bifidobacterium_asteroides
A0A0F4KWW9_Bifidobacterium_asteroides
K4IN19_Bifidobacterium_asteroides
A0A0G0D5K4_Parcubacteria
A0A014QPI2_Metarhizium_robertsii
A0A0B4HYU0_Metarhizium_majus
E9FDJ7_Metarhizium_robertsii
A0A014N4V4_Metarhizium_robertsii
E9EK88_Metarhizium_robertsii
Fcan01_16955_PA
T1IVW9_Strigamia_maritima
J9KZR6_Acyrthosiphon_pisum
W4Z5F3_Strongylocentrotus_purpuratus
W4Y2J9_Strongylocentrotus_purpuratus
W4X9N8_Strongylocentrotus_purpuratus
W4YZ12_Strongylocentrotus_purpuratus
0,58
0,88
1
1
0,86
1
0,89
0,54
1
1
0,86
0,68
1
0,99
0,22
0,93
0,98
0,99
0,99
0,86
1
0,99
0,97
0,99
0.5

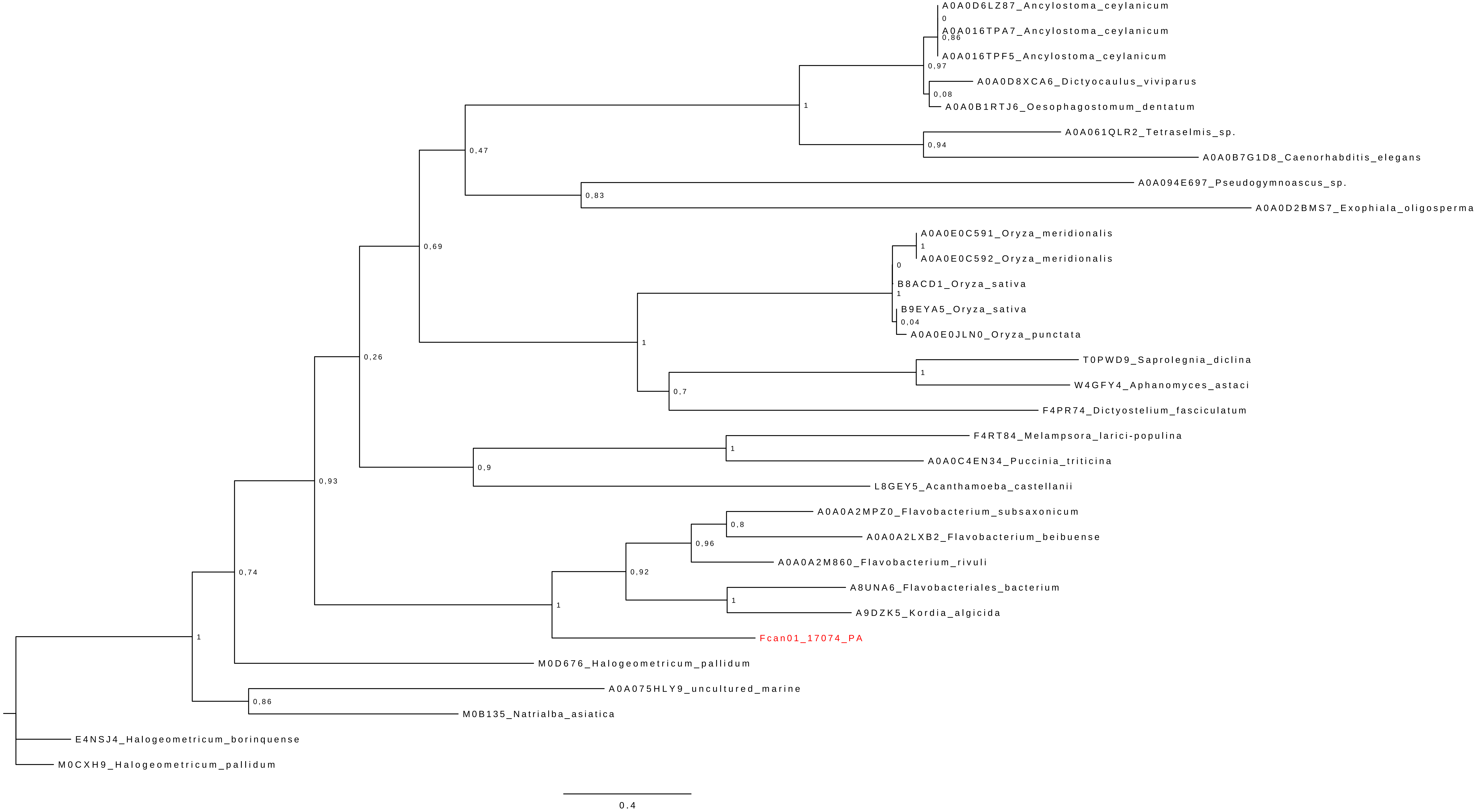
A0A0D6LZ87_Ancylostoma_ceylanicum
A0A016TPA7_Ancylostoma_ceylanicum
A0A016TPF5_Ancylostoma_ceylanicum
A0A0D8XCA6_Dictyocaulus_viviparus
A0A0B1RTJ6_Oesophagostomum_dentatum
A0A061QLR2_Tetraselmis_sp.
A0A0B7G1D8_Caenorhabditis_elegans
A0A094E697_Pseudogymnoascus_sp.
A0A0D2BMS7_Exophiala_oligosperma
A0A0E0C591_Oryza_meridionalis
A0A0E0C592_Oryza_meridionalis
B8ACD1_Oryza_sativa
B9EYA5_Oryza_sativa
A0A0E0JLN0_Oryza_punctata
T0PWD9_Saprolegnia_diclina
W4GFY4_Aphanomyces_astaci
F4PR74_Dictyostelium_fasciculatum
F4RT84_Melampsora_larici-populina
A0A0C4EN34_Puccinia_triticina
L8GEY5_Acanthamoeba_castellanii
A0A0A2MPZ0_Flavobacterium_subsaxonicum
A0A0A2LXB2_Flavobacterium_beibuense
A0A0A2M860_Flavobacterium_rivuli
A8UNA6_Flavobacteriales_bacterium
A9DZK5_Kordia_algicida
Fcan01_17074_PA
M0D676_Halogeometricum_pallidum
A0A075HLY9_uncultured_marine
M0B135_Natrialba_asiatica
E4NSJ4_Halogeometricum_borinquense
M0CXH9_Halogeometricum_pallidum
0.4

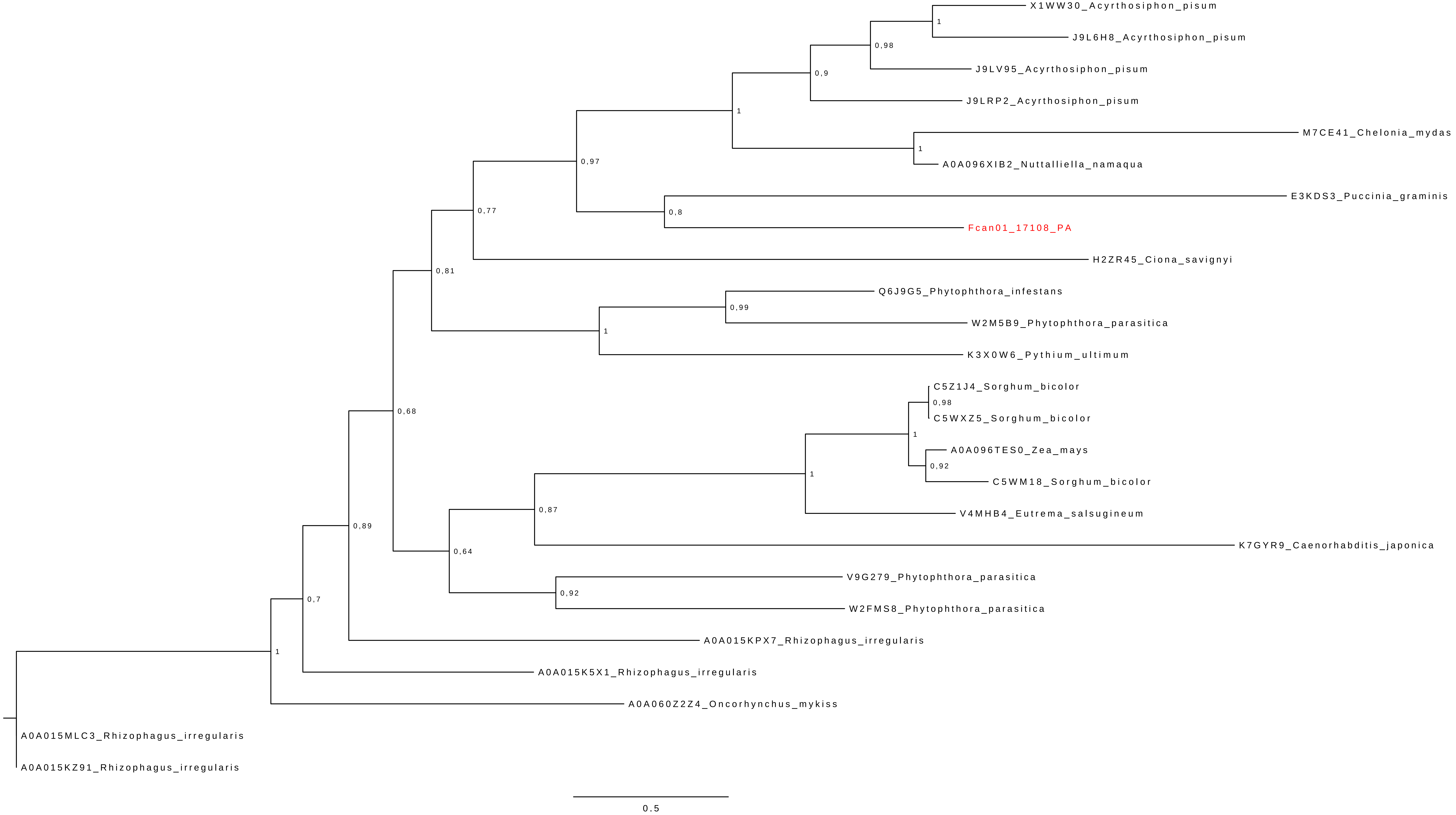
X1WW30_Acyrthosiphon_pisum
1
J9L6H8_Acyrthosiphon_pisum
0,98
J9LV95_Acyrthosiphon_pisum
0,9
J9LRP2_Acyrthosiphon_pisum
1
M7CE41_Chelonia_mydas
1
A0A096XIB2_Nuttalliella_namaqua
0,97
E3KDS3_Puccinia_graminis
0,8
Fcan01_17108_PA
0,77
H2ZR45_Ciona_savignyi
0,81
Q6J9G5_Phytophthora_infestans
0,99
W2M5B9_Phytophthora_parasitica
1
K3X0W6_Pythium_ultimum
C5Z1J4_Sorghum_bicolor
0,98
C5WXZ5_Sorghum_bicolor
1
A0A096TES0_Zea_mays
0,92
C5WM18_Sorghum_bicolor
1
V4MHB4_Eutrema_salsugineum
0,68
0,87
K7GYR9_Caenorhabditis_japonica
0,64
V9G279_Phytophthora_parasitica
0,92
W2FMS8_Phytophthora_parasitica
0,89
A0A015KPX7_Rhizophagus_irregularis
0,7
A0A015K5X1_Rhizophagus_irregularis
1
A0A060Z2Z4_Oncorhynchus_mykiss
A0A015MLC3_Rhizophagus_irregularis
A0A015KZ91_Rhizophagus_irregularis
0.5

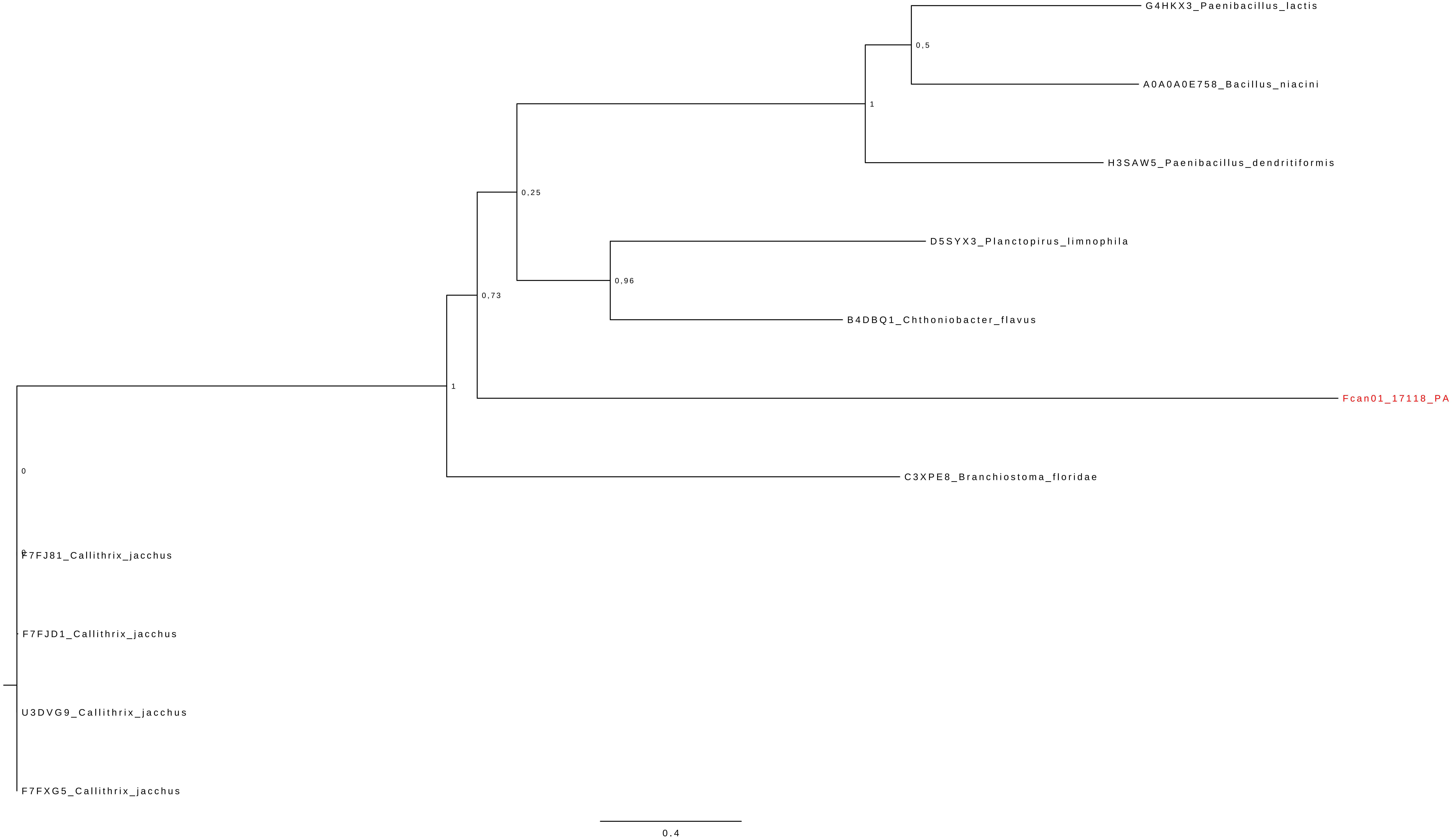
G4HKX3_Paenibacillus_lactis
0,5
A0A0A0E758_Bacillus_niacini
1
H3SAW5_Paenibacillus_dendritiformis
0,25
D5SYX3_Planctopirus_limnophila
0,96
B4DBQ1_Chthoniobacter_flavus
0,73
1
Fcan01_17118_PA
C3XPE8_Branchiostoma_floridae
0
F7FJ81_Callithrix_jacchus
F7FJD1_Callithrix_jacchus
U3DVG9_Callithrix_jacchus
F7FXG5_Callithrix_jacchus
0.4

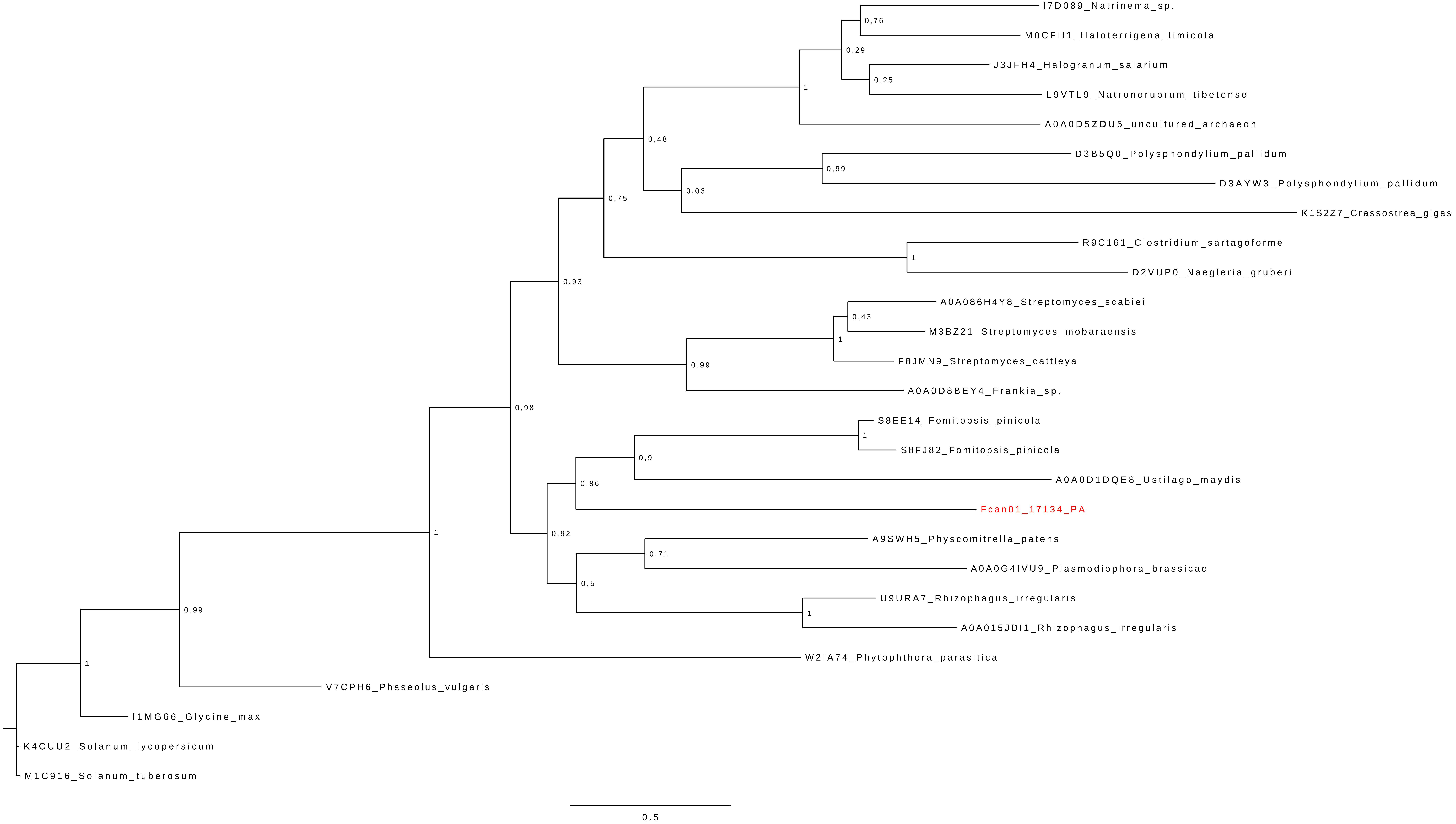
I7D089_Natrinema_sp.
M0CFH1_Haloterrigena_limicola
J3JFH4_Halogranum_salarium
L9VTL9_Natronorubrum_tibetense
A0A0D5ZDU5_uncultured_archaeon
D3B5Q0_Polysphondylium_pallidum
D3AYW3_Polysphondylium_pallidum
K1S2Z7_Crassostrea_gigas
R9C161_Clostridium_sartagoforme
D2VUP0_Naegleria_gruberi
A0A086H4Y8_Streptomyces_scabiei
M3BZ21_Streptomyces_mobaraensis
F8JMN9_Streptomyces_cattleya
A0A0D8BEY4_Frankia_sp.
S8EE14_Fomitopsis_pinicola
S8FJ82_Fomitopsis_pinicola
A0A0D1DQE8_Ustilago_maydis
Fcan01_17134_PA
A9SWH5_Physcomitrella_patens
A0A0G4IVU9_Plasmodiophora_brassicae
U9URA7_Rhizophagus_irregularis
A0A015JDI1_Rhizophagus_irregularis
W2IA74_Phytophthora_parasitica
V7CPH6_Phaseolus_vulgaris
I1MG66_Glycine_max
K4CUU2_Solanum_lycopersicum
M1C916_Solanum_tuberosum
0,76
0,29
0,25
1
0,48
0,99
0,03
0,75
1
0,93
0,43
1
0,99
0,98
1
0,9
0,86
0,92
0,71
0,5
1
1
0,99
1
0.5

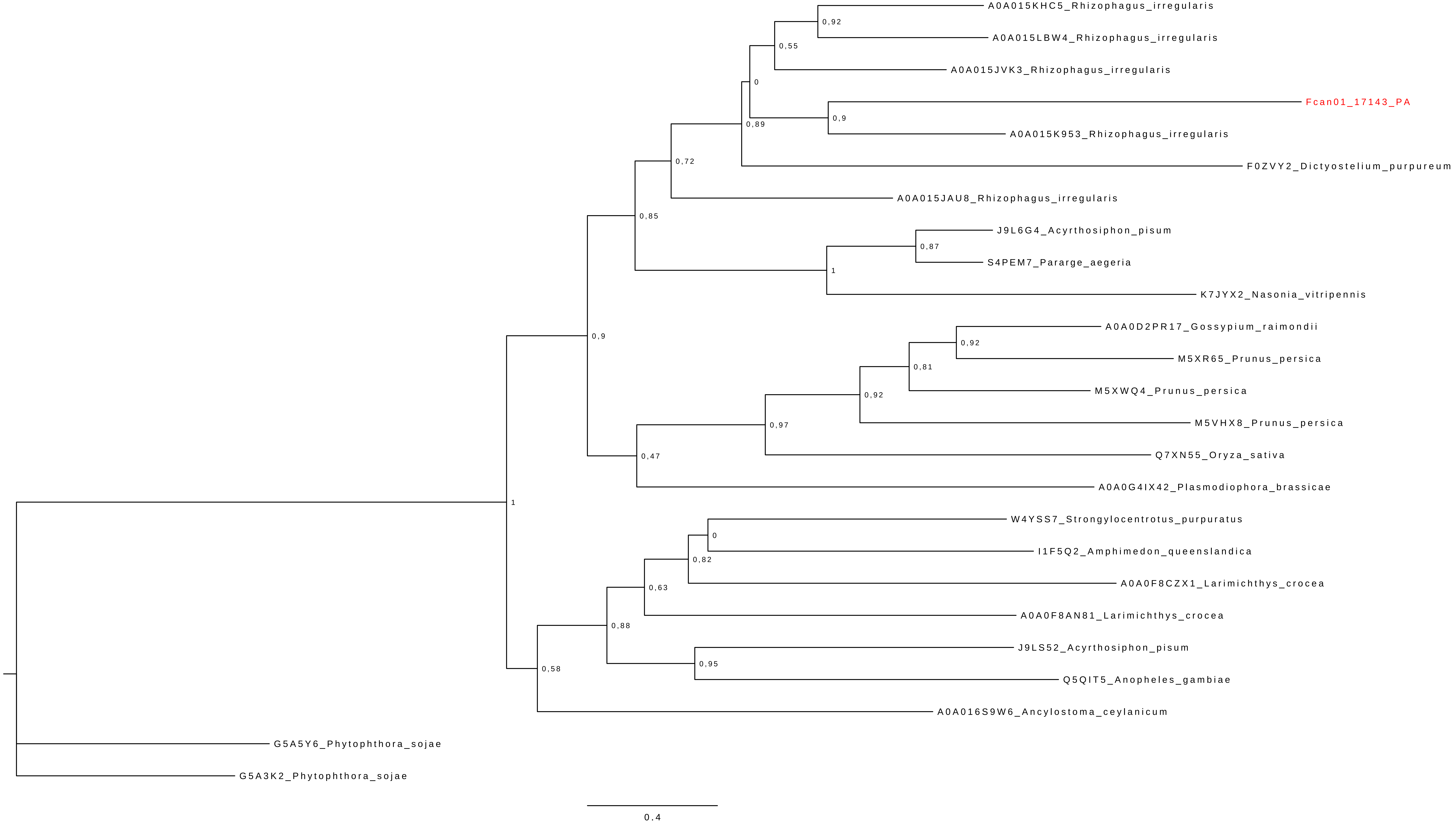
A0A015KHC5_Rhizophagus_irregularis
0,92
A0A015LBW4_Rhizophagus_irregularis
0,55
A0A015JVK3_Rhizophagus_irregularis
0
Fcan01_17143_PA
0,9
A0A015K953_Rhizophagus_irregularis
0,89
F0ZVY2_Dictyostelium_purpureum
0,72
A0A015JAU8_Rhizophagus_irregularis
0,85
J9L6G4_Acyrthosiphon_pisum
0,87
S4PEM7_Pararge_aegeria
1
K7JYX2_Nasonia_vitripennis
0,9
A0A0D2PR17_Gossypium_raimondii
0,92
M5XR65_Prunus_persica
0,81
M5XWQ4_Prunus_persica
0,92
M5VHX8_Prunus_persica
0,97
Q7XN55_Oryza_sativa
0,47
A0A0G4IX42_Plasmodiophora_brassicae
1
W4YSS7_Strongylocentrotus_purpuratus
0
I1F5Q2_Amphimedon_queenslandica
0,82
A0A0F8CZX1_Larimichthys_crocea
0,63
A0A0F8AN81_Larimichthys_crocea
0,88
J9LS52_Acyrthosiphon_pisum
0,95
Q5QIT5_Anopheles_gambiae
0,58
A0A016S9W6_Ancylostoma_ceylanicum
G5A5Y6_Phytophthora_sojae
G5A3K2_Phytophthora_sojae
0.4

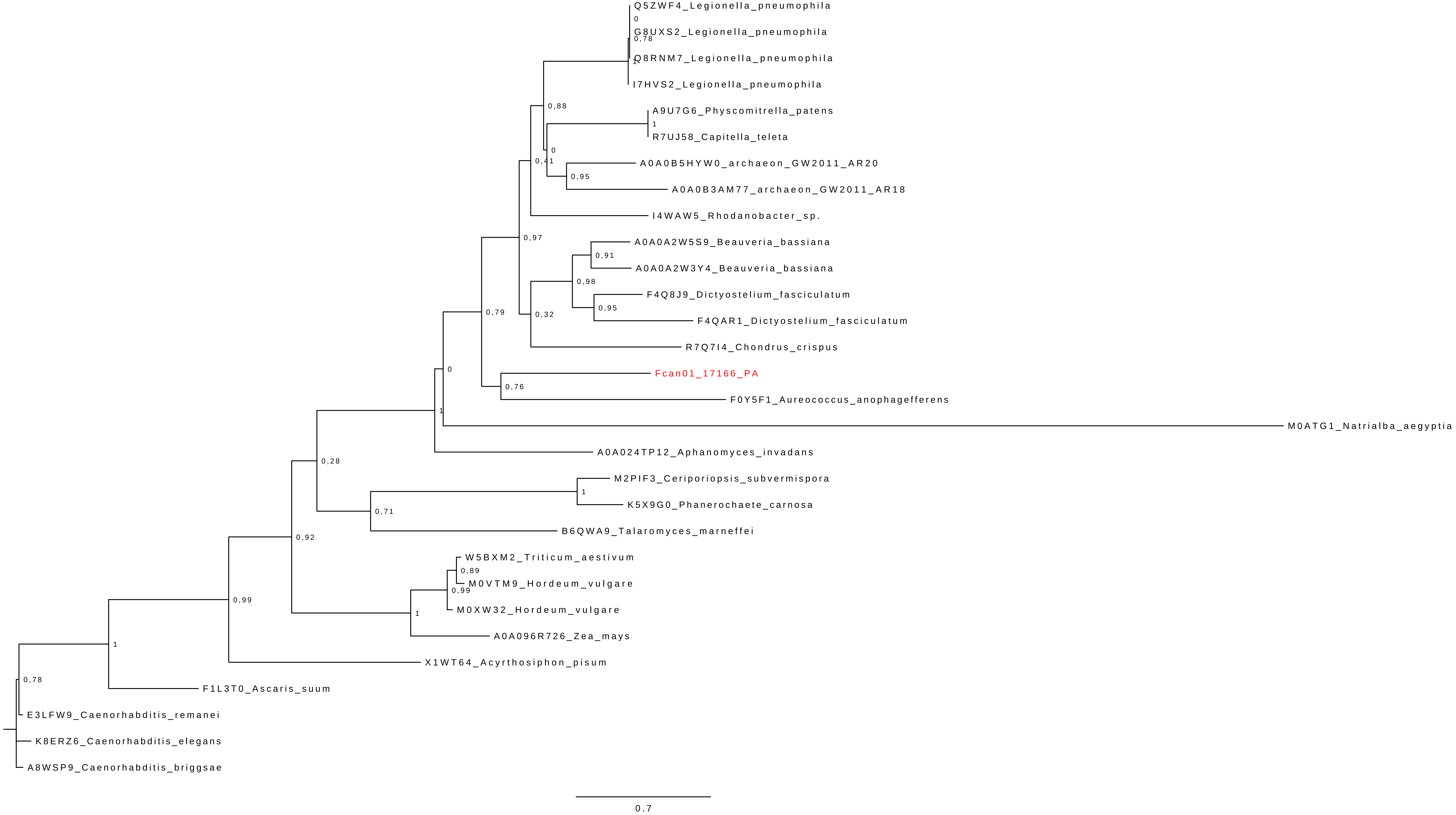

Q5ZWF4_Legionella_pneumophila
0
G8UXS2_Legionella_pneumophila
0,78
Q8RNM7_Legionella_pneumophila
1
I7HVS2_Legionella_pneumophila
0,88
A9U7G6_Physcomitrella_patens
1
R7UJ58_Capitella_teleta
0
0,41
A0A0B5HYW0_archaeon_GW2011_AR20
0,95
A0A0B3AM77_archaeon_GW2011_AR18
I4WAW5_Rhodanobacter_sp.
0,97
A0A0A2W5S9_Beauveria_bassiana
0,91
A0A0A2W3Y4_Beauveria_bassiana
0,98
F4Q8J9_Dictyostelium_fasciculatum
0,95
F4QAR1_Dictyostelium_fasciculatum
0,79
0,32
R7Q7I4_Chondrus_crispus
0
Fcan01_17166_PA
0,76
F0Y5F1_Aureococcus_anophagefferens
1
M0ATG1_Natrialba_aegyptia
A0A024TP12_Aphanomyces_invadans
0,28
M2PIF3_Ceriporiopsis_subvermispora
1
K5X9G0_Phanerochaete_carnosa
0,71
B6QWA9_Talaromyces_marneffei
0,92
W5BXM2_Triticum_aestivum
0,89
M0VTM9_Hordeum_vulgare
0,99
0,99
M0XW32_Hordeum_vulgare
1
A0A096R726_Zea_mays
1
X1WT64_Acyrthosiphon_pisum
0,78
F1L3T0_Ascaris_suum
E3LFW9_Caenorhabditis_remanei
K8ERZ6_Caenorhabditis_elegans
A8WSP9_Caenorhabditis_briggsae
0.7

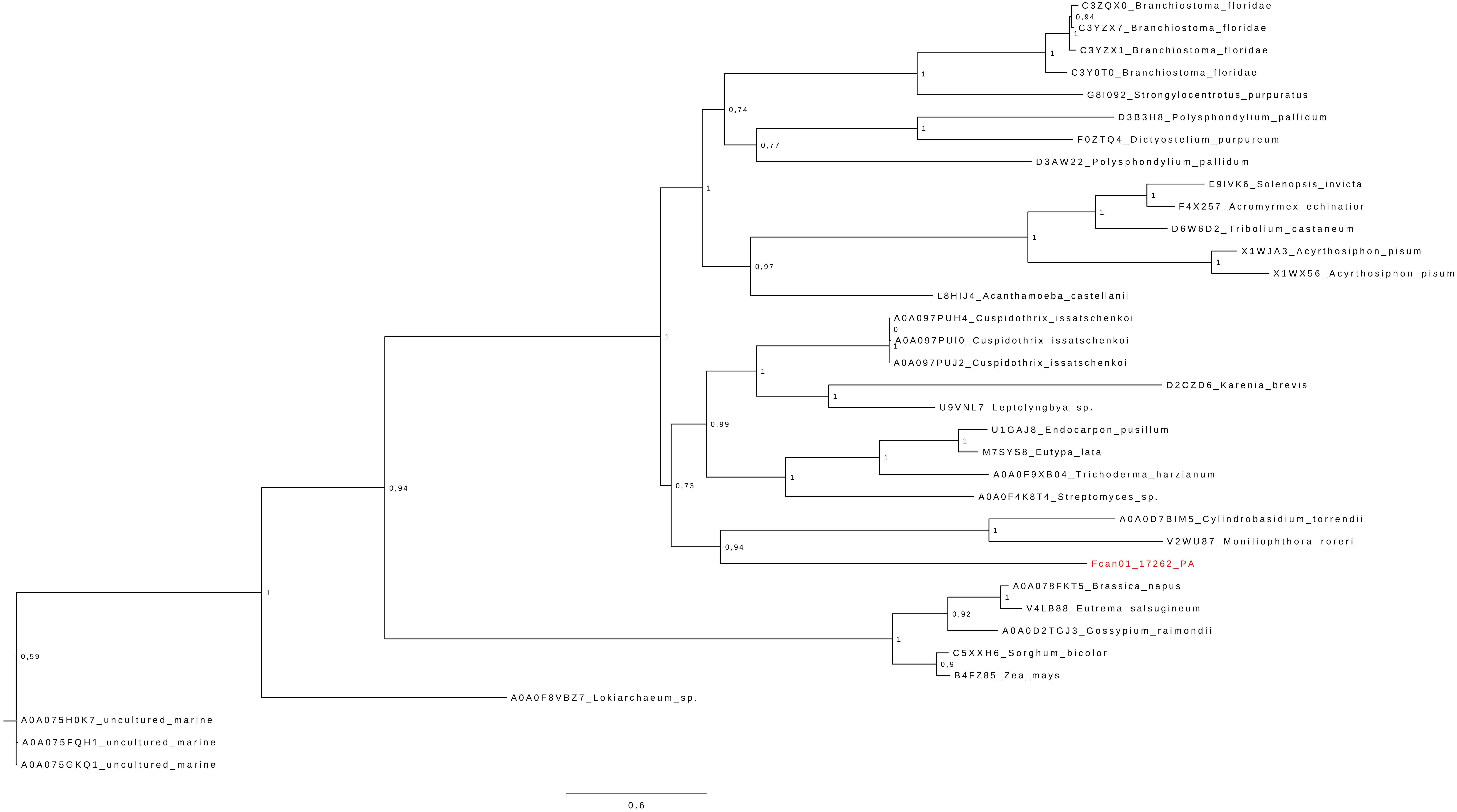
C3ZQX0_Branchiostoma_floridae
0,94
C3YZX7_Branchiostoma_floridae
1
C3YZX1_Branchiostoma_floridae
1
C3Y0T0_Branchiostoma_floridae
1
G8I092_Strongylocentrotus_purpuratus
0,74
D3B3H8_Polysphondylium_pallidum
1
F0ZTQ4_Dictyostelium_purpureum
0,77
D3AW22_Polysphondylium_pallidum
1
E9IVK6_Solenopsis_invicta
1
F4X257_Acromyrmex_echinatior
1
D6W6D2_Tribolium_castaneum
1
X1WJA3_Acyrthosiphon_pisum
1
X1WX56_Acyrthosiphon_pisum
0,97
L8HIJ4_Acanthamoeba_castellanii
A0A097PUH4_Cuspidothrix_issatschenkoi
0
A0A097PUI0_Cuspidothrix_issatschenkoi
1
A0A097PUJ2_Cuspidothrix_issatschenkoi
1
D2CZD6_Karenia_brevis
1
U9VNL7_Leptolyngbya_sp.
0,99
U1GAJ8_Endocarpon_pusillum
1
M7SYS8_Eutypa_lata
1
A0A0F9XB04_Trichoderma_harzianum
1
A0A0F4K8T4_Streptomyces_sp.
0,73
0,94
A0A0D7BIM5_Cylindrobasidium_torrendii
1
V2WU87_Moniliophthora_roreri
0,94
Fcan01_17262_PA
A0A078FKT5_Brassica_napus
1
V4LB88_Eutrema_salsugineum
0,92
A0A0D2TGJ3_Gossypium_raimondii
1
C5XXH6_Sorghum_bicolor
0,9
B4FZ85_Zea_mays
1
0,59
A0A0F8VBZ7_Lokiarchaeum_sp.
A0A075H0K7_uncultured_marine
A0A075FQH1_uncultured_marine
A0A075GKQ1_uncultured_marine
0.6

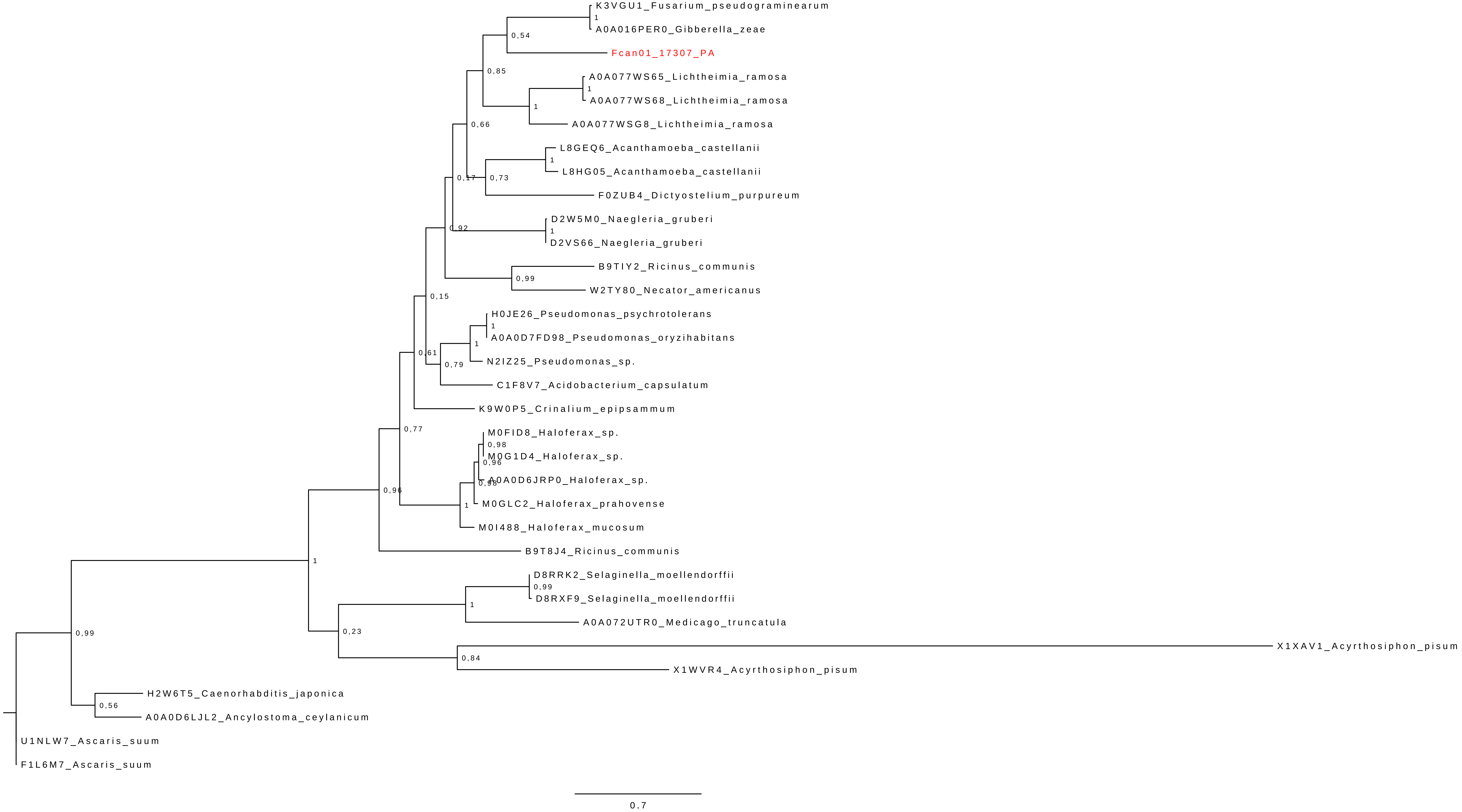
K3VGU1_Fusarium_pseudograminearum
A0A016PER0_Gibberella_zeae
Fcan01_17307_PA
A0A077WS65_Lichtheimia_ramosa
A0A077WS68_Lichtheimia_ramosa
A0A077WSG8_Lichtheimia_ramosa
L8GEQ6_Acanthamoeba_castellanii
L8HG05_Acanthamoeba_castellanii
F0ZUB4_Dictyostelium_purpureum
D2W5M0_Naegleria_gruberi
D2VS66_Naegleria_gruberi
B9TIY2_Ricinus_communis
W2TY80_Necator_americanus
H0JE26_Pseudomonas_psychrotolerans
A0A0D7FD98_Pseudomonas_oryzihabitans
N2IZ25_Pseudomonas_sp.
C1F8V7_Acidobacterium_capsulatum
K9W0P5_Crinalium_epipsammum
M0FID8_Haloferax_sp.
M0G1D4_Haloferax_sp.
A0A0D6JRP0_Haloferax_sp.
M0GLC2_Haloferax_prahovense
M0I488_Haloferax_mucosum
B9T8J4_Ricinus_communis
D8RRK2_Selaginella_moellendorffii
D8RXF9_Selaginella_moellendorffii
A0A072UTR0_Medicago_truncatula
X1XAV1_Acyrthosiphon_pisum
X1WVR4_Acyrthosiphon_pisum
H2W6T5_Caenorhabditis_japonica
A0A0D6LJL2_Ancylostoma_ceylanicum
U1NLW7_Ascaris_suum
F1L6M7_Ascaris_suum
1
0,54
0,85
1
1
0,66
1
0,17
0,73
0,92
1
0,99
0,15
1
1
0,61
0,79
0,77
0,98
0,96
0,98
0,96
1
1
0,99
1
0,23
0,84
0,99
0,56
0.7

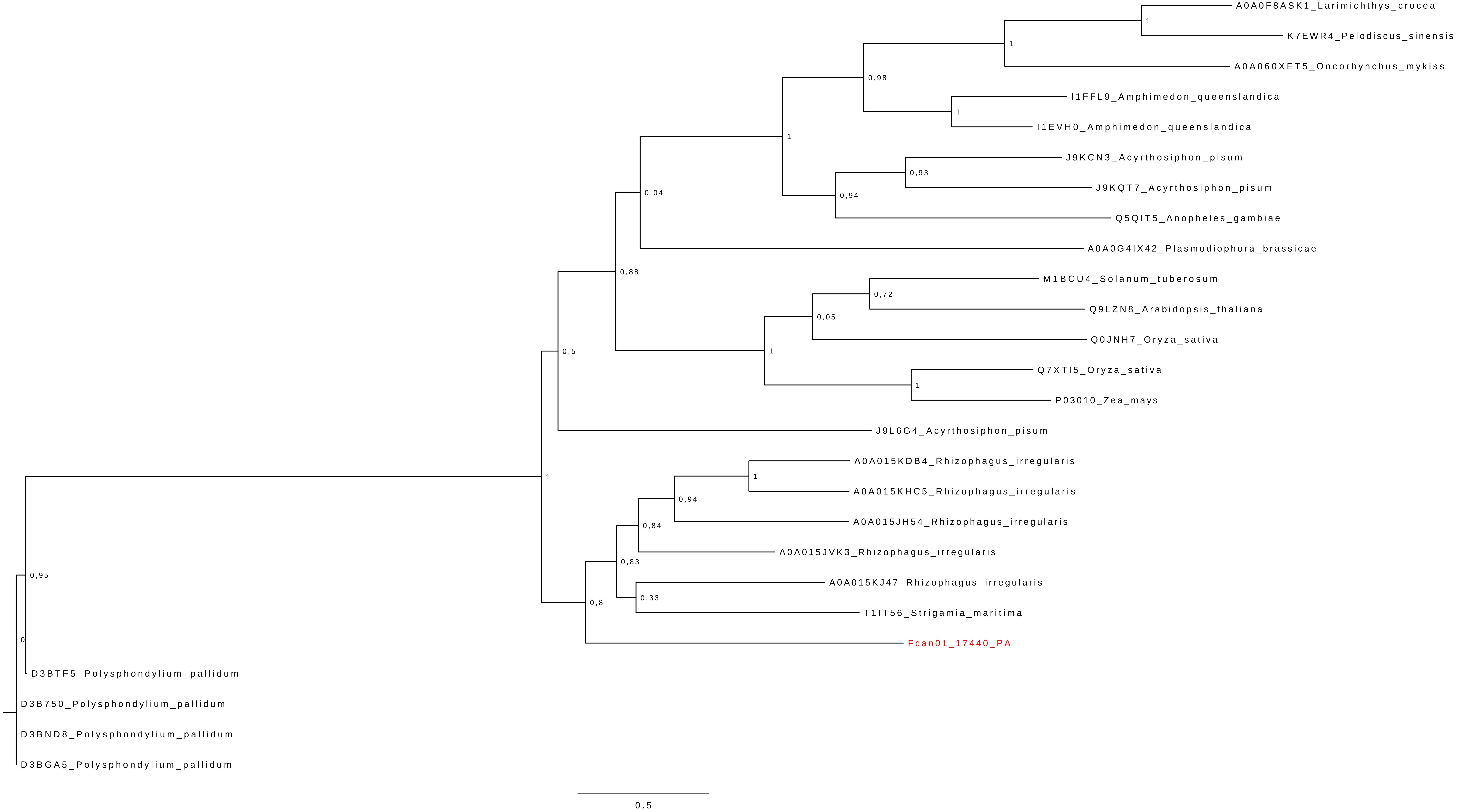
A0A0F8ASK1_Larimichthys_crocea
K7EWR4_Pelodiscus_sinensis
A0A060XET5_Oncorhynchus_mykiss
I1FFL9_Amphimedon_queenslandica
I1EVH0_Amphimedon_queenslandica
J9KCN3_Acyrthosiphon_pisum
J9KQT7_Acyrthosiphon_pisum
Q5QIT5_Anopheles_gambiae
A0A0G4IX42_Plasmodiophora_brassicae
M1BCU4_Solanum_tuberosum
Q9LZN8_Arabidopsis_thaliana
Q0JNH7_Oryza_sativa
Q7XTI5_Oryza_sativa
P03010_Zea_mays
J9L6G4_Acyrthosiphon_pisum
A0A015KDB4_Rhizophagus_irregularis
A0A015KHC5_Rhizophagus_irregularis
A0A015JH54_Rhizophagus_irregularis
A0A015JVK3_Rhizophagus_irregularis
A0A015KJ47_Rhizophagus_irregularis
T1IT56_Strigamia_maritima
Fcan01_17440_PA
D3BTF5_Polysphondylium_pallidum
D3B750_Polysphondylium_pallidum
D3BND8_Polysphondylium_pallidum
D3BGA5_Polysphondylium_pallidum
1
1
0,98
1
1
0,93
0,94
0,04
0,88
0,72
0,05
1
1
0,5
1
1
0,94
0,84
0,83
0,33
0,8
0,95
0
0.5

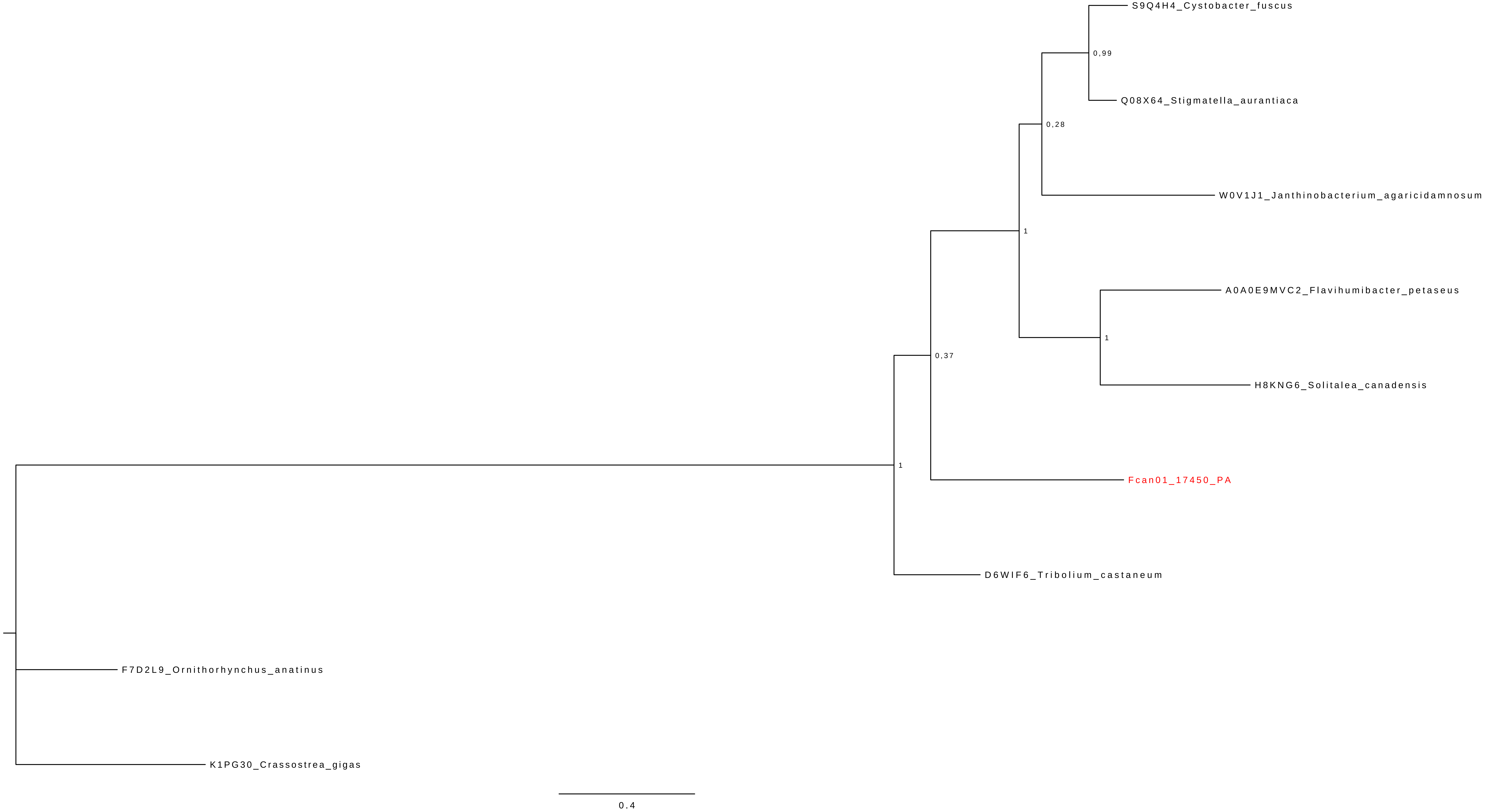
S9Q4H4_Cystobacter_fuscus
0,99
Q08X64_Stigmatella_aurantiaca
0,28
W0V1J1_Janthinobacterium_agaricidamnosum
1
A0A0E9MVC2_Flavihumibacter_petaseus
1
H8KNG6_Solitalea_canadensis
0,37
1
Fcan01_17450_PA
D6WIF6_Tribolium_castaneum
F7D2L9_Ornithorhynchus_anatinus
K1PG30_Crassostrea_gigas
0.4

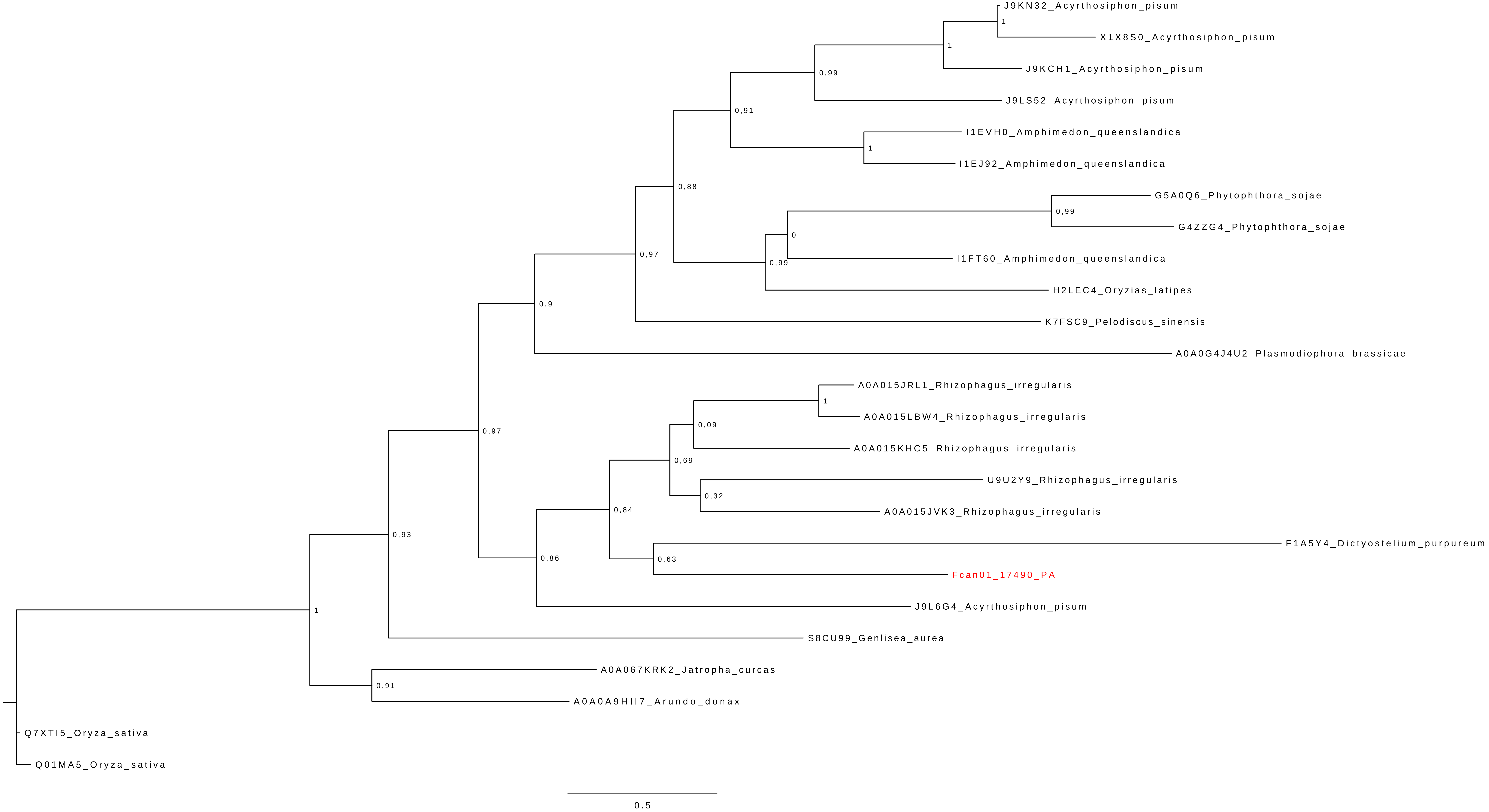
J9KN32_Acyrthosiphon_pisum
X1X8S0_Acyrthosiphon_pisum
J9KCH1_Acyrthosiphon_pisum
J9LS52_Acyrthosiphon_pisum
I1EVH0_Amphimedon_queenslandica
I1EJ92_Amphimedon_queenslandica
G5A0Q6_Phytophthora_sojae
G4ZZG4_Phytophthora_sojae
I1FT60_Amphimedon_queenslandica
H2LEC4_Oryzias_latipes
K7FSC9_Pelodiscus_sinensis
A0A0G4J4U2_Plasmodiophora_brassicae
A0A015JRL1_Rhizophagus_irregularis
A0A015LBW4_Rhizophagus_irregularis
A0A015KHC5_Rhizophagus_irregularis
U9U2Y9_Rhizophagus_irregularis
A0A015JVK3_Rhizophagus_irregularis
F1A5Y4_Dictyostelium_purpureum
Fcan01_17490_PA
J9L6G4_Acyrthosiphon_pisum
S8CU99_Genlisea_aurea
A0A067KRK2_Jatropha_curcas
A0A0A9HII7_Arundo_donax
Q7XTI5_Oryza_sativa
Q01MA5_Oryza_sativa
1
1
0,99
0,91
1
0,88
0,99
0
0,99
0,97
0,9
1
0,09
0,69
0,32
0,84
0,63
0,86
0,97
0,93
1
0,91
0.5

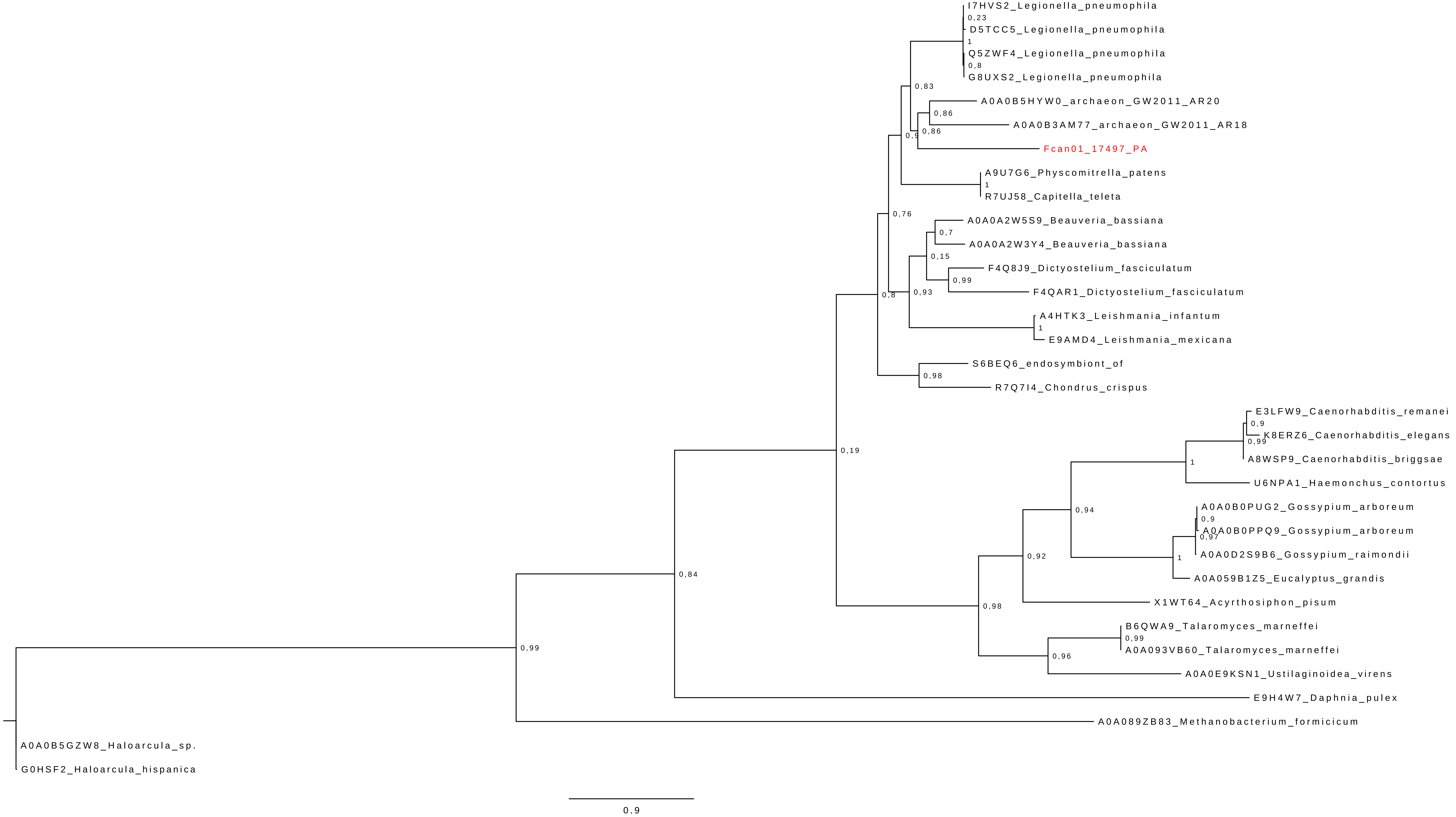

I7HVS2_Legionella_pneumophila
0,23
D5TCC5_Legionella_pneumophila
1
Q5ZWF4_Legionella_pneumophila
0,8
G8UXS2_Legionella_pneumophila
0,83
A0A0B5HYW0_archaeon_GW2011_AR20
0,86
A0A0B3AM77_archaeon_GW2011_AR18
0,86
Fcan01_17497_PA
A9U7G6_Physcomitrella_patens
1
R7UJ58_Capitella_teleta
0,76
A0A0A2W5S9_Beauveria_bassiana
0,7
A0A0A2W3Y4_Beauveria_bassiana
0,15
F4Q8J9_Dictyostelium_fasciculatum
0,99
F4QAR1_Dictyostelium_fasciculatum
0,8
0,93
A4HTK3_Leishmania_infantum
1
E9AMD4_Leishmania_mexicana
S6BEQ6_endosymbiont_of
0,98
R7Q7I4_Chondrus_crispus
E3LFW9_Caenorhabditis_remanei
0,9
K8ERZ6_Caenorhabditis_elegans
0,99
0,19
A8WSP9_Caenorhabditis_briggsae
1
U6NPA1_Haemonchus_contortus
0,94
A0A0B0PUG2_Gossypium_arboreum
0,9
A0A0B0PPQ9_Gossypium_arboreum
0,97
0,92
A0A0D2S9B6_Gossypium_raimondii
1
0,84
A0A059B1Z5_Eucalyptus_grandis
0,98
X1WT64_Acyrthosiphon_pisum
B6QWA9_Talaromyces_marneffei
0,99
0,99
A0A093VB60_Talaromyces_marneffei
0,96
A0A0E9KSN1_Ustilaginoidea_virens
E9H4W7_Daphnia_pulex
A0A089ZB83_Methanobacterium_formicicum
A0A0B5GZW8_Haloarcula_sp.
G0HSF2_Haloarcula_hispanica
0.9

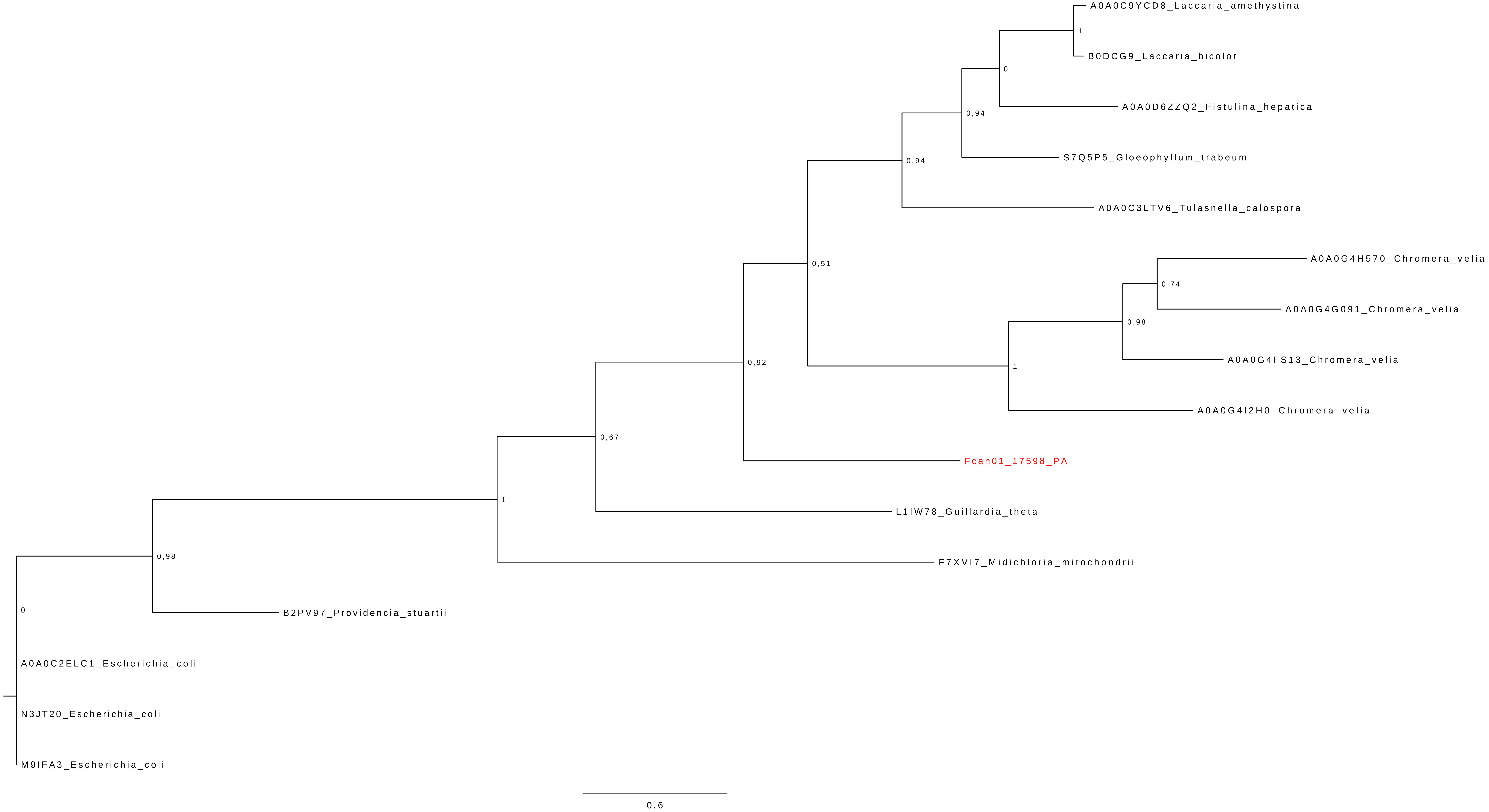
A0A0C9YCD8_Laccaria_amethystina
1
B0DCG9_Laccaria_bicolor
0
A0A0D6ZZQ2_Fistulina_hepatica
0,94
S7Q5P5_Gloeophyllum_trabeum
0,94
A0A0C3LTV6_Tulasnella_calospora
0,51
A0A0G4H570_Chromera_velia
0,74
A0A0G4G091_Chromera_velia
0,98
A0A0G4FS13_Chromera_velia
1
A0A0G4I2H0_Chromera_velia
0,92
Fcan01_17598_PA
0,67
L1IW78_Guillardia_theta
1
F7XVI7_Midichloria_mitochondrii
0,98
B2PV97_Providencia_stuartii
0
A0A0C2ELC1_Escherichia_coli
N3JT20_Escherichia_coli
M9IFA3_Escherichia_coli
0.6

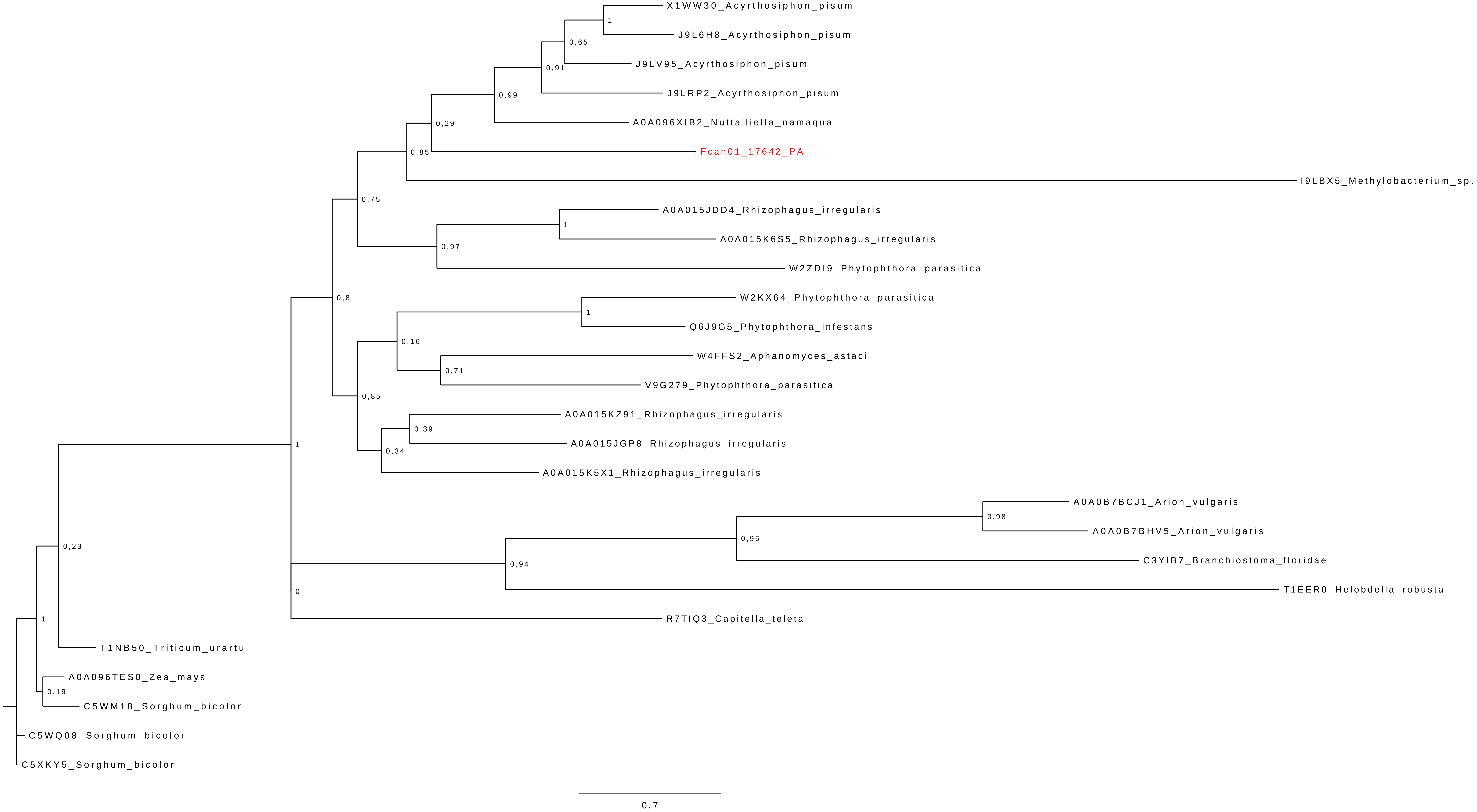
X1WW30_Acyrthosiphon_pisum
J9L6H8_Acyrthosiphon_pisum
J9LV95_Acyrthosiphon_pisum
J9LRP2_Acyrthosiphon_pisum
A0A096XIB2_Nuttalliella_namaqua
Fcan01_17642_PA
I9LBX5_Methylobacterium_sp.
A0A015JDD4_Rhizophagus_irregularis
A0A015K6S5_Rhizophagus_irregularis
W2ZDI9_Phytophthora_parasitica
W2KX64_Phytophthora_parasitica
Q6J9G5_Phytophthora_infestans
W4FFS2_Aphanomyces_astaci
V9G279_Phytophthora_parasitica
A0A015KZ91_Rhizophagus_irregularis
A0A015JGP8_Rhizophagus_irregularis
A0A015K5X1_Rhizophagus_irregularis
A0A0B7BCJ1_Arion_vulgaris
A0A0B7BHV5_Arion_vulgaris
C3YIB7_Branchiostoma_floridae
T1EER0_Helobdella_robusta
R7TIQ3_Capitella_teleta
T1NB50_Triticum_urartu
A0A096TES0_Zea_mays
C5WM18_Sorghum_bicolor
C5WQ08_Sorghum_bicolor
C5XKY5_Sorghum_bicolor
1
0,65
0,91
0,99
0,29
0,85
0,75
1
0,97
0,8
1
0,16
0,71
0,85
0,39
0,34
1
0,98
0,95
0,94
0
0,23
1
0,19
0.7

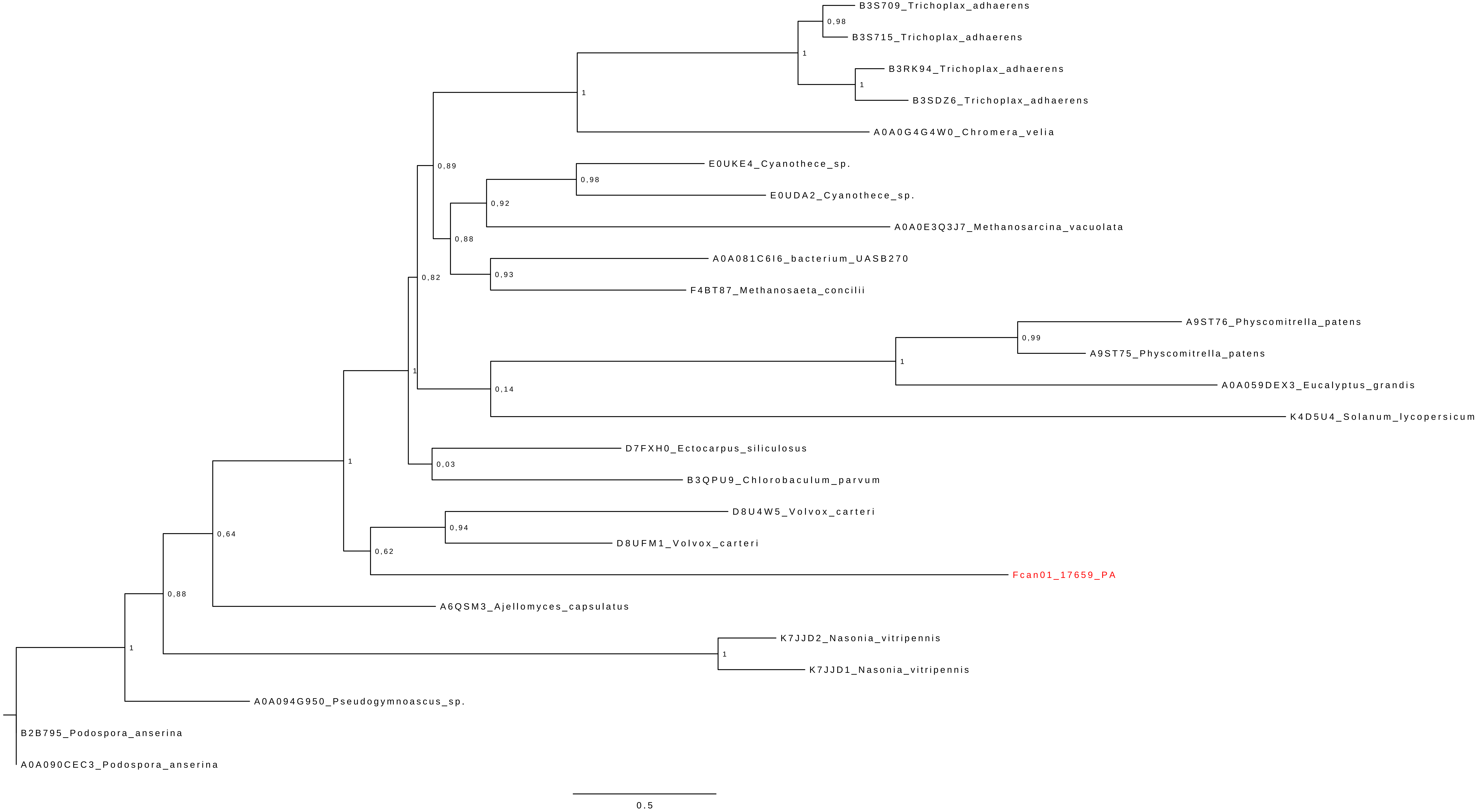
B3S709_Trichoplax_adhaerens
0,98
B3S715_Trichoplax_adhaerens
1
B3RK94_Trichoplax_adhaerens
1
B3SDZ6_Trichoplax_adhaerens
1
A0A0G4G4W0_Chromera_velia
0,89
E0UKE4_Cyanothece_sp.
0,98
E0UDA2_Cyanothece_sp.
0,92
A0A0E3Q3J7_Methanosarcina_vacuolata
0,88
A0A081C6I6_bacterium_UASB270
0,82
0,93
F4BT87_Methanosaeta_concilii
A9ST76_Physcomitrella_patens
0,99
A9ST75_Physcomitrella_patens
1
1
A0A059DEX3_Eucalyptus_grandis
0,14
K4D5U4_Solanum_lycopersicum
D7FXH0_Ectocarpus_siliculosus
1
0,03
B3QPU9_Chlorobaculum_parvum
D8U4W5_Volvox_carteri
0,94
0,64
D8UFM1_Volvox_carteri
0,62
Fcan01_17659_PA
0,88
A6QSM3_Ajellomyces_capsulatus
K7JJD2_Nasonia_vitripennis
1
1
K7JJD1_Nasonia_vitripennis
A0A094G950_Pseudogymnoascus_sp.
B2B795_Podospora_anserina
A0A090CEC3_Podospora_anserina
0.5

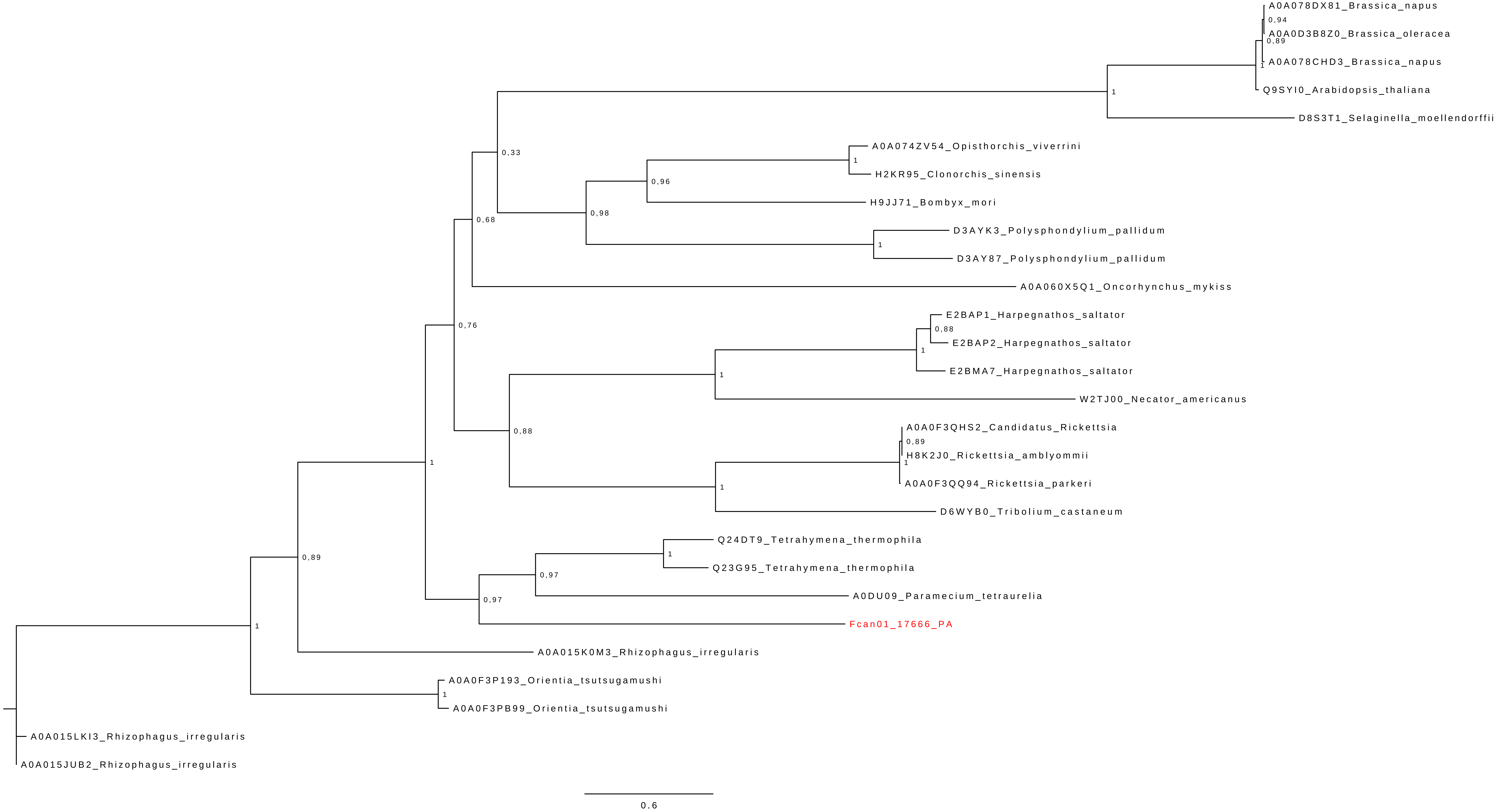
A0A078DX81_Brassica_napus
A0A0D3B8Z0_Brassica_oleracea
A0A078CHD3_Brassica_napus
Q9SYI0_Arabidopsis_thaliana
D8S3T1_Selaginella_moellendorffii
A0A074ZV54_Opisthorchis_viverrini
H2KR95_Clonorchis_sinensis
H9JJ71_Bombyx_mori
D3AYK3_Polysphondylium_pallidum
D3AY87_Polysphondylium_pallidum
A0A060X5Q1_Oncorhynchus_mykiss
E2BAP1_Harpegnathos_saltator
E2BAP2_Harpegnathos_saltator
E2BMA7_Harpegnathos_saltator
W2TJ00_Necator_americanus
A0A0F3QHS2_Candidatus_Rickettsia
H8K2J0_Rickettsia_amblyommii
A0A0F3QQ94_Rickettsia_parkeri
D6WYB0_Tribolium_castaneum
Q24DT9_Tetrahymena_thermophila
Q23G95_Tetrahymena_thermophila
A0DU09_Paramecium_tetraurelia
Fcan01_17666_PA
A0A015K0M3_Rhizophagus_irregularis
A0A0F3P193_Orientia_tsutsugamushi
A0A0F3PB99_Orientia_tsutsugamushi
A0A015LKI3_Rhizophagus_irregularis
A0A015JUB2_Rhizophagus_irregularis
0.6

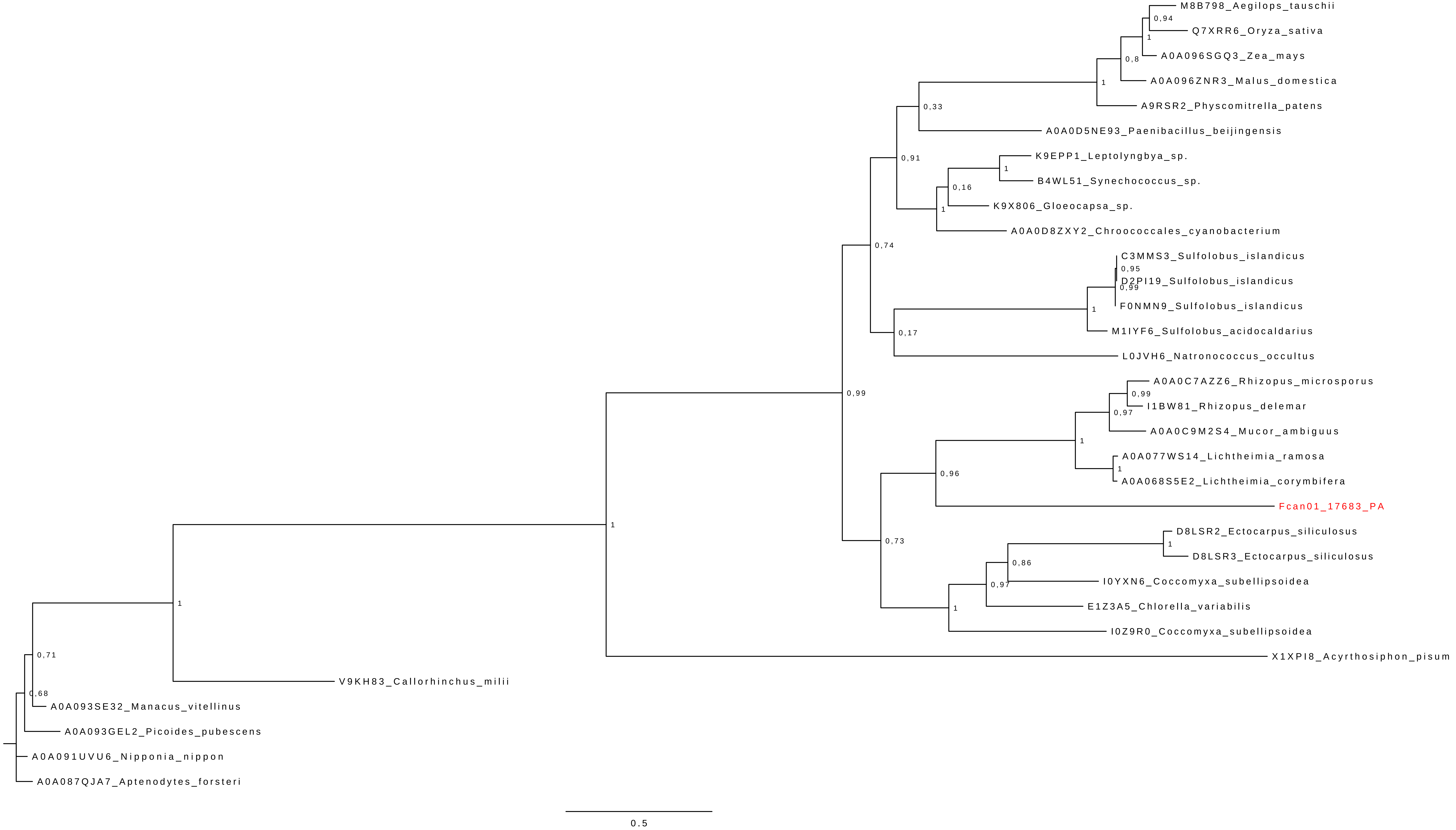
M8B798_Aegilops_tauschii
0,94
Q7XRR6_Oryza_sativa
1
A0A096SGQ3_Zea_mays
0,8
A0A096ZNR3_Malus_domestica
1
A9RSR2_Physcomitrella_patens
0,33
A0A0D5NE93_Paenibacillus_beijingensis
0,91
K9EPP1_Leptolyngbya_sp.
1
B4WL51_Synechococcus_sp.
0,16
K9X806_Gloeocapsa_sp.
1
A0A0D8ZXY2_Chroococcales_cyanobacterium
0,74
C3MMS3_Sulfolobus_islandicus
0,95
D2PI19_Sulfolobus_islandicus
0,99
F0NMN9_Sulfolobus_islandicus
1
M1IYF6_Sulfolobus_acidocaldarius
0,17
L0JVH6_Natronococcus_occultus
0,99
A0A0C7AZZ6_Rhizopus_microsporus
0,99
I1BW81_Rhizopus_delemar
0,97
A0A0C9M2S4_Mucor_ambiguus
1
A0A077WS14_Lichtheimia_ramosa
1
A0A068S5E2_Lichtheimia_corymbifera
0,96
Fcan01_17683_PA
1
D8LSR2_Ectocarpus_siliculosus
0,73
1
D8LSR3_Ectocarpus_siliculosus
0,86
I0YXN6_Coccomyxa_subellipsoidea
0,97
E1Z3A5_Chlorella_variabilis
1
I0Z9R0_Coccomyxa_subellipsoidea
X1XPI8_Acyrthosiphon_pisum
1
0,71
V9KH83_Callorhinchus_milii
0,68
A0A093SE32_Manacus_vitellinus
A0A093GEL2_Picoides_pubescens
A0A091UVU6_Nipponia_nippon
A0A087QJA7_Aptenodytes_forsteri
0.5

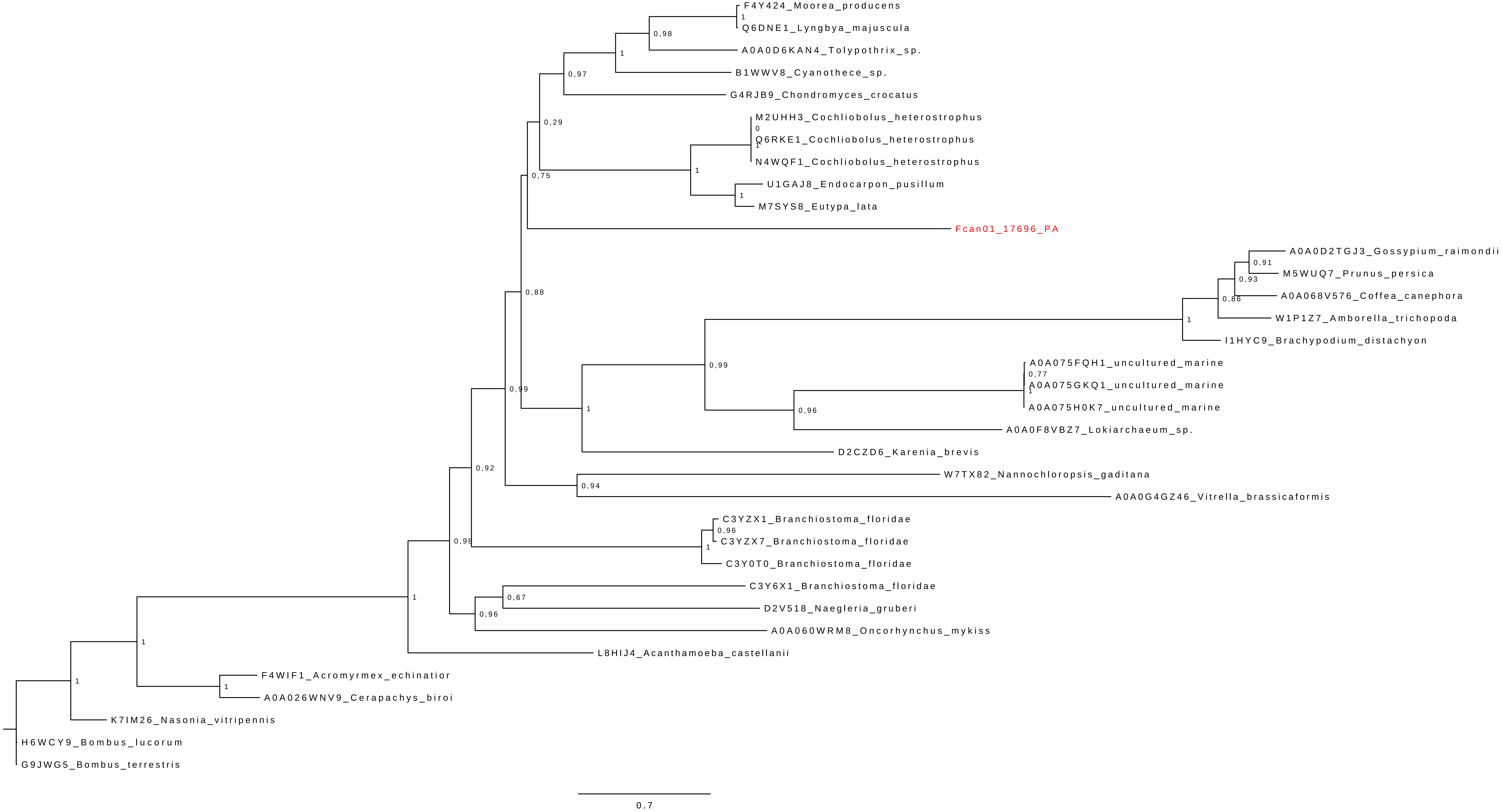
F4Y424_Moorea_producens
Q6DNE1_Lyngbya_majuscula
A0A0D6KAN4_Tolypothrix_sp.
B1WWV8_Cyanothece_sp.
G4RJB9_Chondromyces_crocatus
M2UHH3_Cochliobolus_heterostrophus
Q6RKE1_Cochliobolus_heterostrophus
N4WQF1_Cochliobolus_heterostrophus
U1GAJ8_Endocarpon_pusillum
M7SYS8_Eutypa_lata
Fcan01_17696_PA
A0A0D2TGJ3_Gossypium_raimondii
M5WUQ7_Prunus_persica
A0A068V576_Coffea_canephora
W1P1Z7_Amborella_trichopoda
I1HYC9_Brachypodium_distachyon
A0A075FQH1_uncultured_marine
A0A075GKQ1_uncultured_marine
A0A075H0K7_uncultured_marine
A0A0F8VBZ7_Lokiarchaeum_sp.
D2CZD6_Karenia_brevis
W7TX82_Nannochloropsis_gaditana
A0A0G4GZ46_Vitrella_brassicaformis
C3YZX1_Branchiostoma_floridae
C3YZX7_Branchiostoma_floridae
C3Y0T0_Branchiostoma_floridae
C3Y6X1_Branchiostoma_floridae
D2V518_Naegleria_gruberi
A0A060WRM8_Oncorhynchus_mykiss
L8HIJ4_Acanthamoeba_castellanii
F4WIF1_Acromyrmex_echinatior
A0A026WNV9_Cerapachys_biroi
K7IM26_Nasonia_vitripennis
H6WCY9_Bombus_lucorum
G9JWG5_Bombus_terrestris
0.7

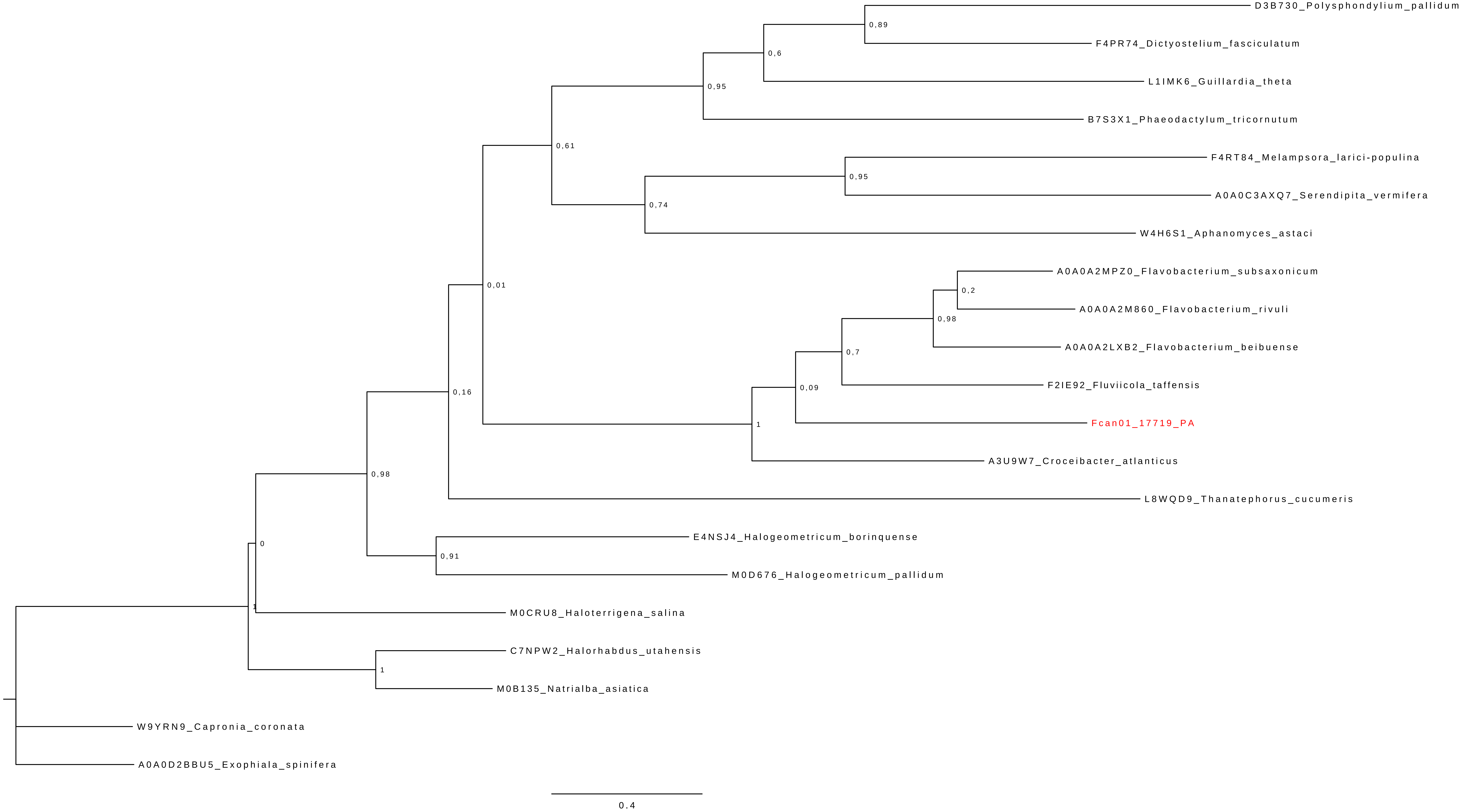
D3B730_Polysphondylium_pallidum
0,89
F4PR74_Dictyostelium_fasciculatum
0,6
L1IMK6_Guillardia_theta
0,95
B7S3X1_Phaeodactylum_tricornutum
0,61
F4RT84_Melampsora_larici-populina
0,95
A0A0C3AXQ7_Serendipita_vermifera
0,74
W4H6S1_Aphanomyces_astaci
A0A0A2MPZ0_Flavobacterium_subsaxonicum
0,01
0,2
A0A0A2M860_Flavobacterium_rivuli
0,98
A0A0A2LXB2_Flavobacterium_beibuense
0,7
F2IE92_Fluviicola_taffensis
0,09
0,16
Fcan01_17719_PA
1
A3U9W7_Croceibacter_atlanticus
0,98
L8WQD9_Thanatephorus_cucumeris
E4NSJ4_Halogeometricum_borinquense
0
0,91
M0D676_Halogeometricum_pallidum
1
M0CRU8_Haloterrigena_salina
C7NPW2_Halorhabdus_utahensis
1
M0B135_Natrialba_asiatica
W9YRN9_Capronia_coronata
A0A0D2BBU5_Exophiala_spinifera
0.4

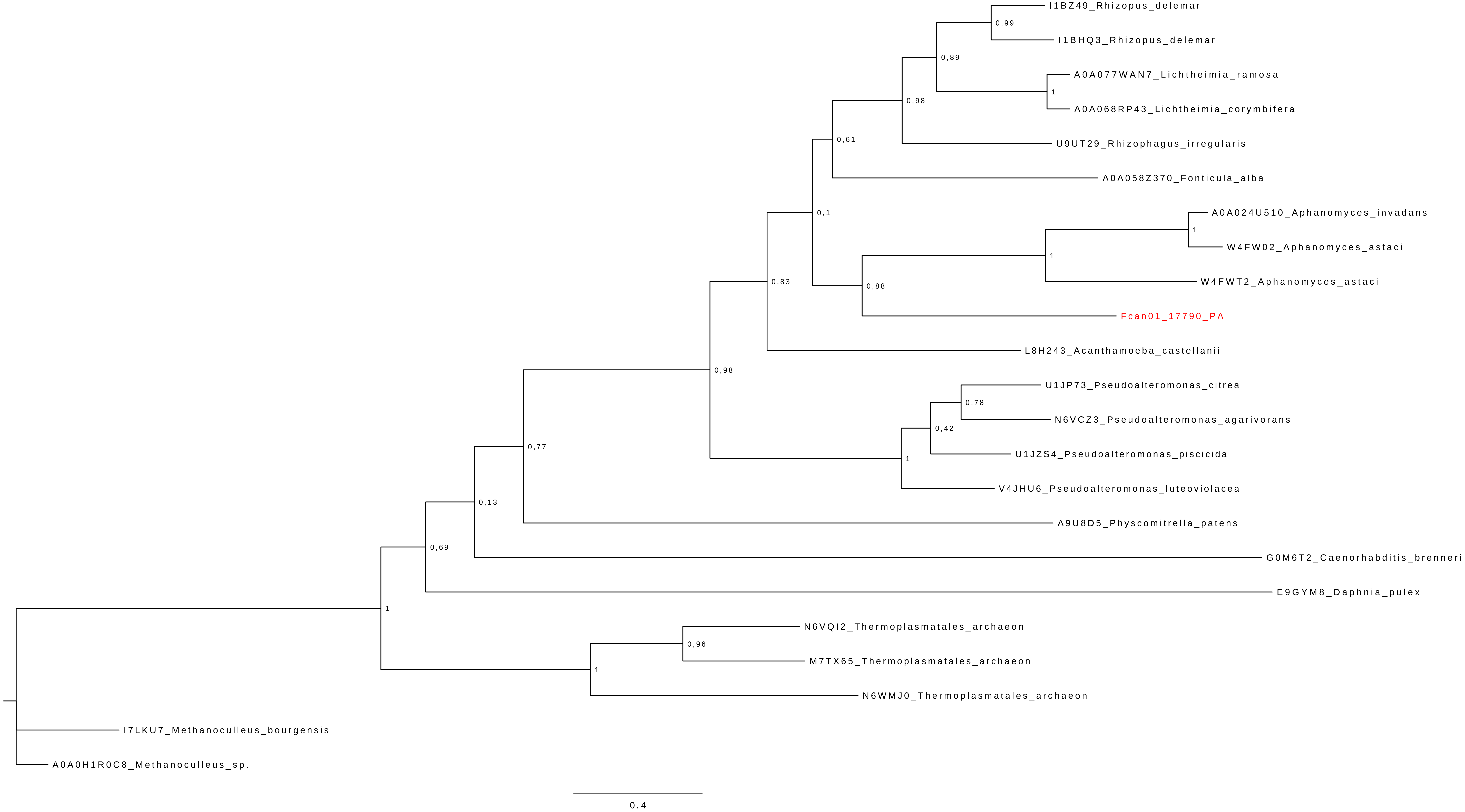
I1BZ49_Rhizopus_delemar
0,99
I1BHQ3_Rhizopus_delemar
0,89
A0A077WAN7_Lichtheimia_ramosa
1
A0A068RP43_Lichtheimia_corymbifera
0,98
U9UT29_Rhizophagus_irregularis
0,61
A0A058Z370_Fonticula_alba
0,1
A0A024U510_Aphanomyces_invadans
1
W4FW02_Aphanomyces_astaci
1
W4FWT2_Aphanomyces_astaci
0,83
0,88
Fcan01_17790_PA
L8H243_Acanthamoeba_castellanii
0,98
U1JP73_Pseudoalteromonas_citrea
0,78
N6VCZ3_Pseudoalteromonas_agarivorans
0,42
U1JZS4_Pseudoalteromonas_piscicida
1
V4JHU6_Pseudoalteromonas_luteoviolacea
0,77
A9U8D5_Physcomitrella_patens
0,13
G0M6T2_Caenorhabditis_brenneri
0,69
E9GYM8_Daphnia_pulex
1
N6VQI2_Thermoplasmatales_archaeon
0,96
M7TX65_Thermoplasmatales_archaeon
1
N6WMJ0_Thermoplasmatales_archaeon
I7LKU7_Methanoculleus_bourgensis
A0A0H1R0C8_Methanoculleus_sp.
0.4

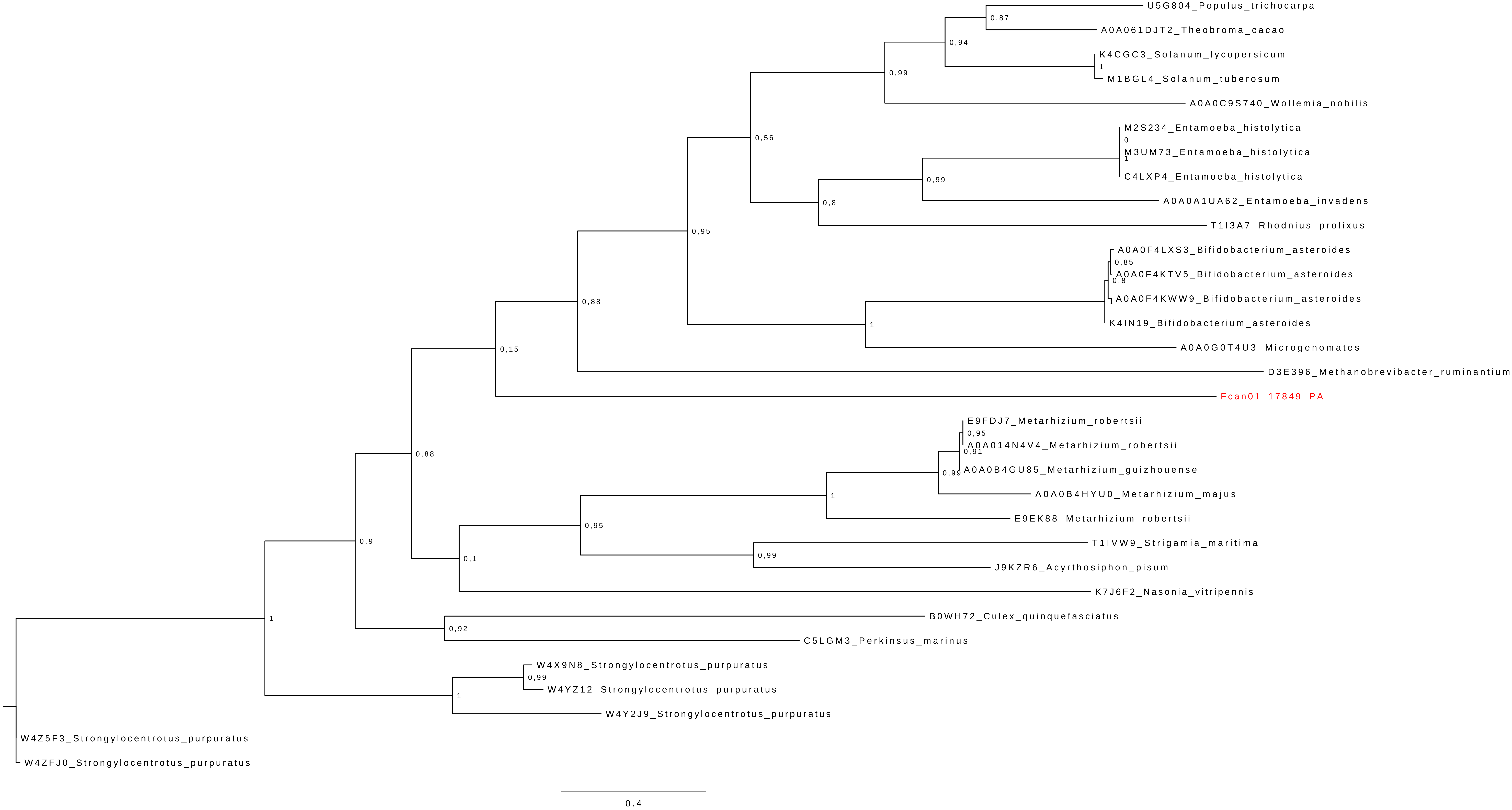
U5G804_Populus_trichocarpa
0,87
A0A061DJT2_Theobroma_cacao
0,94
K4CGC3_Solanum_lycopersicum
1
M1BGL4_Solanum_tuberosum
0,99
A0A0C9S740_Wollemia_nobilis
0,56
M2S234_Entamoeba_histolytica
0
M3UM73_Entamoeba_histolytica
1
C4LXP4_Entamoeba_histolytica
0,99
A0A0A1UA62_Entamoeba_invadens
0,8
T1I3A7_Rhodnius_prolixus
0,95
A0A0F4LXS3_Bifidobacterium_asteroides
0,85
A0A0F4KTV5_Bifidobacterium_asteroides
0,8
A0A0F4KWW9_Bifidobacterium_asteroides
1
K4IN19_Bifidobacterium_asteroides
1
A0A0G0T4U3_Microgenomates
0,88
D3E396_Methanobrevibacter_ruminantium
0,15
Fcan01_17849_PA
E9FDJ7_Metarhizium_robertsii
0,95
A0A014N4V4_Metarhizium_robertsii
0,91
A0A0B4GU85_Metarhizium_guizhouense
0,99
A0A0B4HYU0_Metarhizium_majus
1
E9EK88_Metarhizium_robertsii
0,88
T1IVW9_Strigamia_maritima
0,95
0,99
J9KZR6_Acyrthosiphon_pisum
0,1
K7J6F2_Nasonia_vitripennis
0,9
B0WH72_Culex_quinquefasciatus
0,92
C5LGM3_Perkinsus_marinus
1
W4X9N8_Strongylocentrotus_purpuratus
0,99
W4YZ12_Strongylocentrotus_purpuratus
1
W4Y2J9_Strongylocentrotus_purpuratus
W4Z5F3_Strongylocentrotus_purpuratus
W4ZFJ0_Strongylocentrotus_purpuratus
0.4

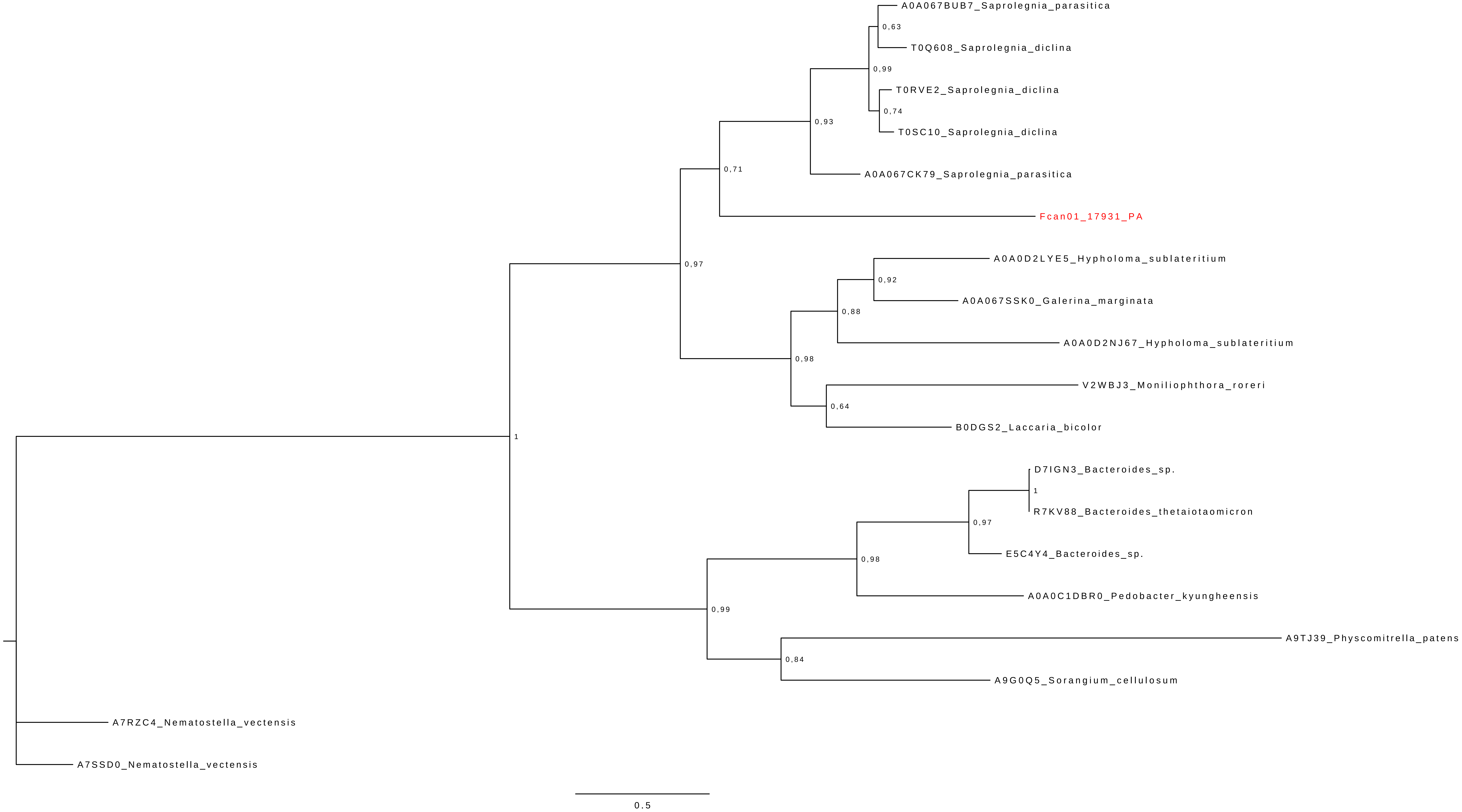
A0A067BUB7_Saprolegnia_parasitica
0,63
T0Q608_Saprolegnia_diclina
0,99
T0RVE2_Saprolegnia_diclina
0,74
T0SC10_Saprolegnia_diclina
0,93
A0A067CK79_Saprolegnia_parasitica
0,71
Fcan01_17931_PA
0,97
A0A0D2LYE5_Hypholoma_sublateritium
0,92
A0A067SSK0_Galerina_marginata
0,88
A0A0D2NJ67_Hypholoma_sublateritium
0,98
V2WBJ3_Moniliophthora_roreri
0,64
B0DGS2_Laccaria_bicolor
1
D7IGN3_Bacteroides_sp.
1
R7KV88_Bacteroides_thetaiotaomicron
0,97
E5C4Y4_Bacteroides_sp.
0,98
A0A0C1DBR0_Pedobacter_kyungheensis
0,99
A9TJ39_Physcomitrella_patens
0,84
A9G0Q5_Sorangium_cellulosum
A7RZC4_Nematostella_vectensis
A7SSD0_Nematostella_vectensis
0.5

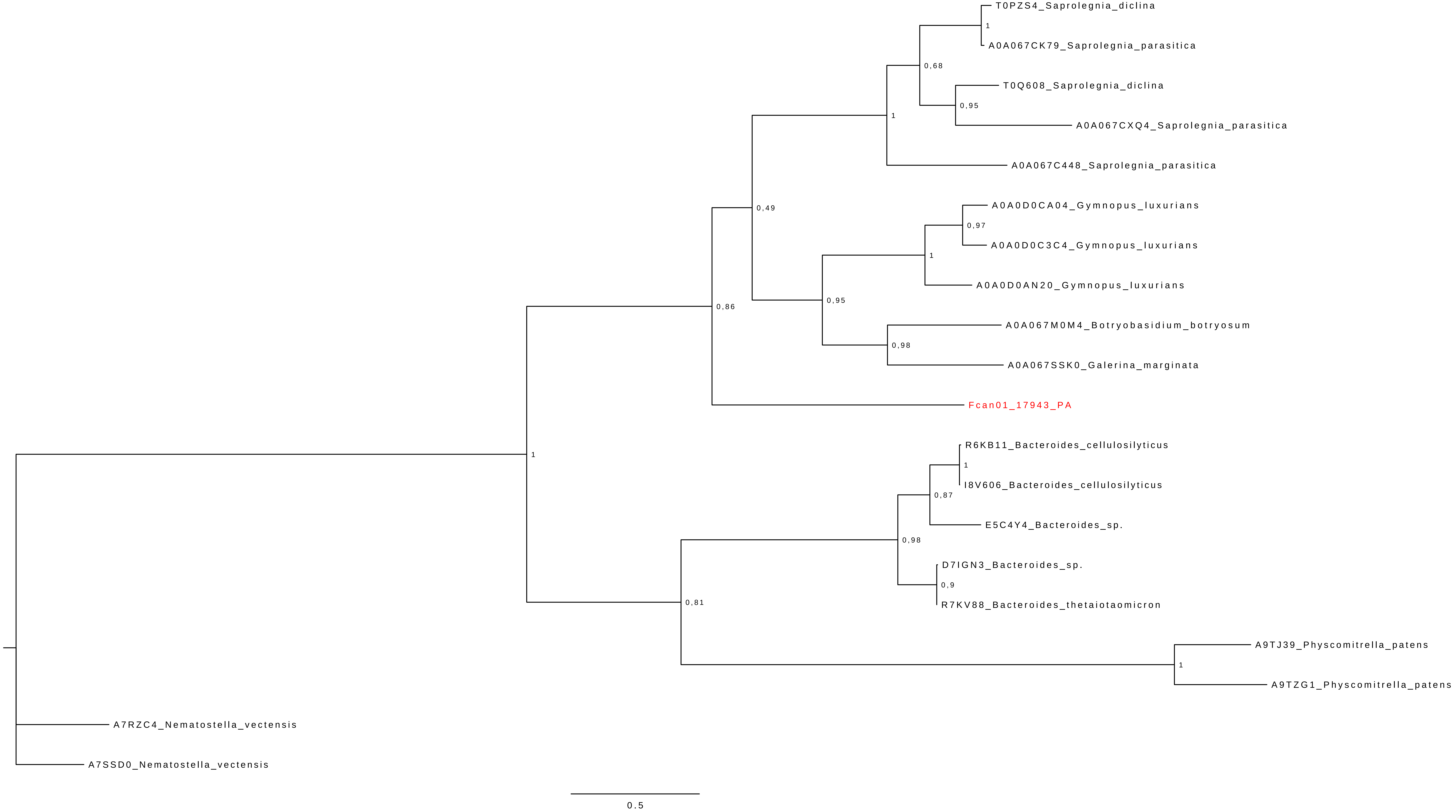
T0PZS4_Saprolegnia_diclina
1
A0A067CK79_Saprolegnia_parasitica
0,68
T0Q608_Saprolegnia_diclina
0,95
A0A067CXQ4_Saprolegnia_parasitica
1
A0A067C448_Saprolegnia_parasitica
0,49
A0A0D0CA04_Gymnopus_luxurians
0,97
A0A0D0C3C4_Gymnopus_luxurians
1
A0A0D0AN20_Gymnopus_luxurians
0,86
0,95
A0A067M0M4_Botryobasidium_botryosum
0,98
A0A067SSK0_Galerina_marginata
Fcan01_17943_PA
R6KB11_Bacteroides_cellulosilyticus
1
1
I8V606_Bacteroides_cellulosilyticus
0,87
E5C4Y4_Bacteroides_sp.
0,98
D7IGN3_Bacteroides_sp.
0,9
0,81
R7KV88_Bacteroides_thetaiotaomicron
A9TJ39_Physcomitrella_patens
1
A9TZG1_Physcomitrella_patens
A7RZC4_Nematostella_vectensis
A7SSD0_Nematostella_vectensis
0.5

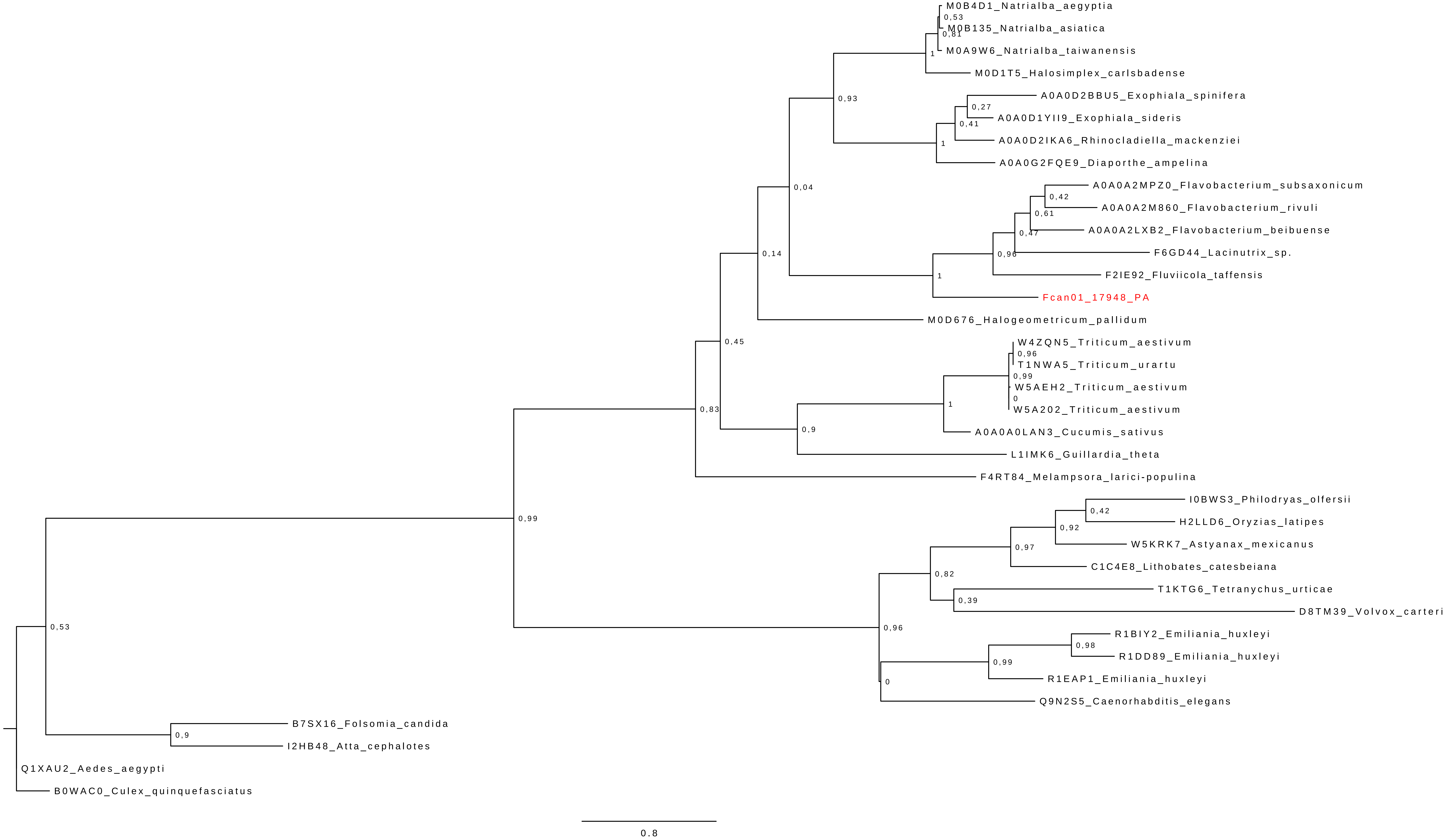
M0B4D1_Natrialba_aegyptia
M0B135_Natrialba_asiatica
M0A9W6_Natrialba_taiwanensis
M0D1T5_Halosimplex_carlsbadense
A0A0D2BBU5_Exophiala_spinifera
A0A0D1YII9_Exophiala_sideris
A0A0D2IKA6_Rhinocladiella_mackenziei
A0A0G2FQE9_Diaporthe_ampelina
A0A0A2MPZ0_Flavobacterium_subsaxonicum
A0A0A2M860_Flavobacterium_rivuli
A0A0A2LXB2_Flavobacterium_beibuense
F6GD44_Lacinutrix_sp.
F2IE92_Fluviicola_taffensis
Fcan01_17948_PA
M0D676_Halogeometricum_pallidum
W4ZQN5_Triticum_aestivum
T1NWA5_Triticum_urartu
W5AEH2_Triticum_aestivum
W5A202_Triticum_aestivum
A0A0A0LAN3_Cucumis_sativus
L1IMK6_Guillardia_theta
F4RT84_Melampsora_larici-populina
I0BWS3_Philodryas_olfersii
H2LLD6_Oryzias_latipes
W5KRK7_Astyanax_mexicanus
C1C4E8_Lithobates_catesbeiana
T1KTG6_Tetranychus_urticae
D8TM39_Volvox_carteri
R1BIY2_Emiliania_huxleyi
R1DD89_Emiliania_huxleyi
R1EAP1_Emiliania_huxleyi
Q9N2S5_Caenorhabditis_elegans
B7SX16_Folsomia_candida
I2HB48_Atta_cephalotes
Q1XAU2_Aedes_aegypti
B0WAC0_Culex_quinquefasciatus
0.8

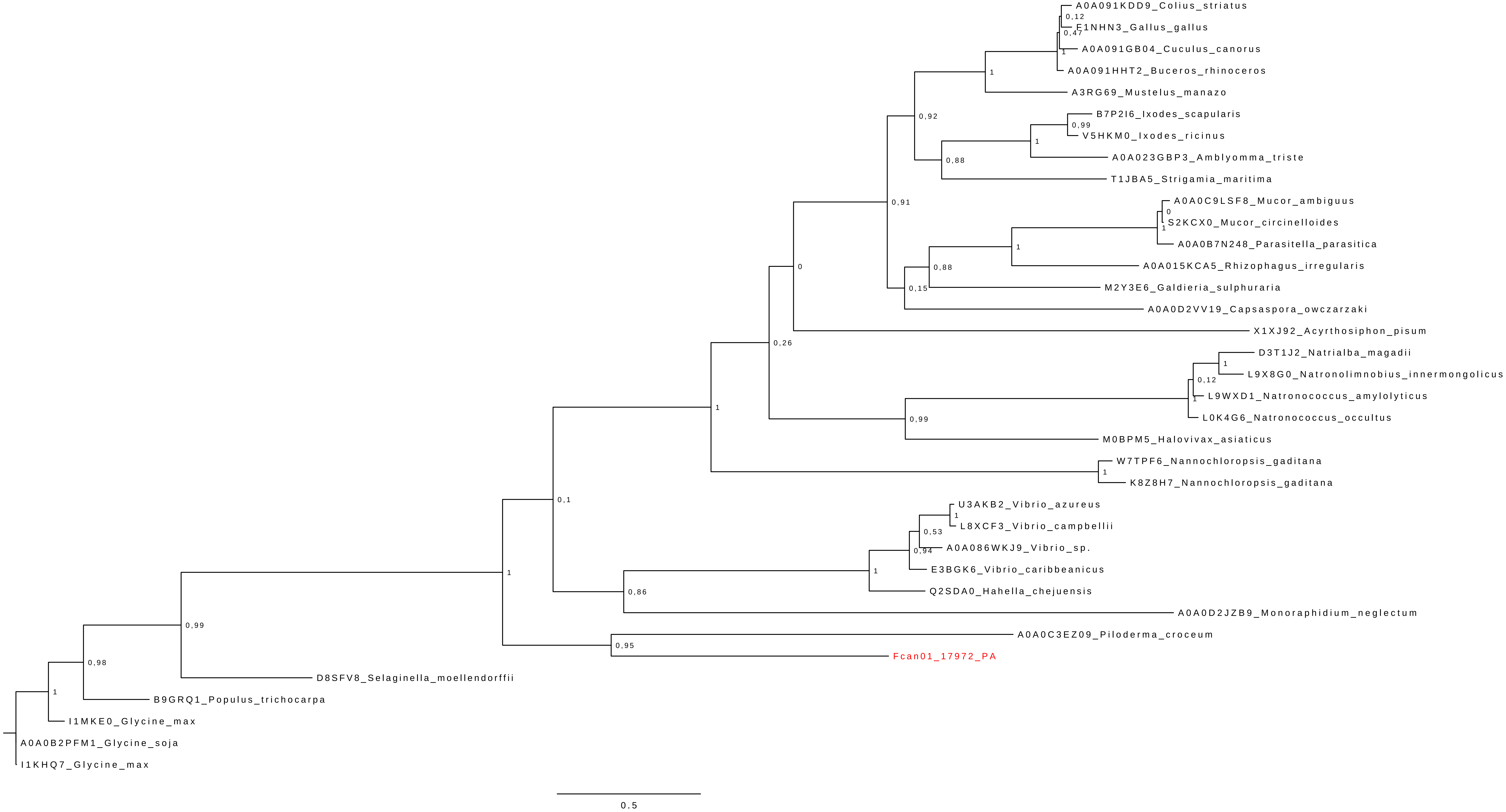
A0A091KDD9_Colius_striatus
F1NHN3_Gallus_gallus
A0A091GB04_Cuculus_canorus
A0A091HHT2_Buceros_rhinoceros
A3RG69_Mustelus_manazo
B7P2I6_Ixodes_scapularis
V5HKM0_Ixodes_ricinus
A0A023GBP3_Amblyomma_triste
T1JBA5_Strigamia_maritima
A0A0C9LSF8_Mucor_ambiguus
S2KCX0_Mucor_circinelloides
A0A0B7N248_Parasitella_parasitica
A0A015KCA5_Rhizophagus_irregularis
M2Y3E6_Galdieria_sulphuraria
A0A0D2VV19_Capsaspora_owczarzaki
X1XJ92_Acyrthosiphon_pisum
D3T1J2_Natrialba_magadii
L9X8G0_Natronolimnobius_innermongolicus
L9WXD1_Natronococcus_amylolyticus
L0K4G6_Natronococcus_occultus
M0BPM5_Halovivax_asiaticus
W7TPF6_Nannochloropsis_gaditana
K8Z8H7_Nannochloropsis_gaditana
U3AKB2_Vibrio_azureus
L8XCF3_Vibrio_campbellii
A0A086WKJ9_Vibrio_sp.
E3BGK6_Vibrio_caribbeanicus
Q2SDA0_Hahella_chejuensis
A0A0D2JZB9_Monoraphidium_neglectum
A0A0C3EZ09_Piloderma_croceum
Fcan01_17972_PA
D8SFV8_Selaginella_moellendorffii
B9GRQ1_Populus_trichocarpa
I1MKE0_Glycine_max
A0A0B2PFM1_Glycine_soja
I1KHQ7_Glycine_max
0.5

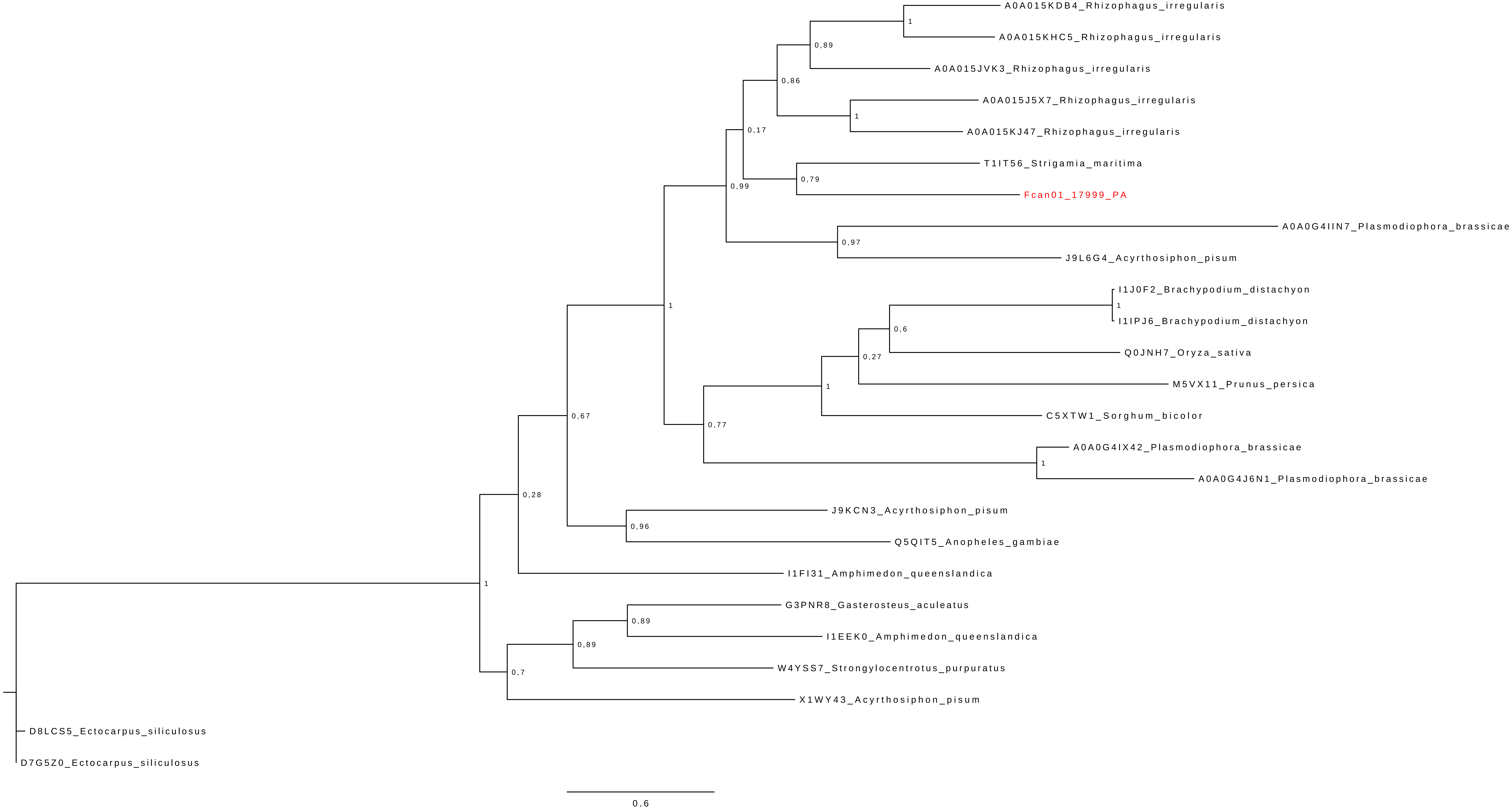
A0A015KDB4_Rhizophagus_irregularis
A0A015KHC5_Rhizophagus_irregularis
A0A015JVK3_Rhizophagus_irregularis
A0A015J5X7_Rhizophagus_irregularis
A0A015KJ47_Rhizophagus_irregularis
T1IT56_Strigamia_maritima
Fcan01_17999_PA
A0A0G4IIN7_Plasmodiophora_brassicae
J9L6G4_Acyrthosiphon_pisum
I1J0F2_Brachypodium_distachyon
I1IPJ6_Brachypodium_distachyon
Q0JNH7_Oryza_sativa
M5VX11_Prunus_persica
C5XTW1_Sorghum_bicolor
A0A0G4IX42_Plasmodiophora_brassicae
A0A0G4J6N1_Plasmodiophora_brassicae
J9KCN3_Acyrthosiphon_pisum
Q5QIT5_Anopheles_gambiae
I1FI31_Amphimedon_queenslandica
G3PNR8_Gasterosteus_aculeatus
I1EEK0_Amphimedon_queenslandica
W4YSS7_Strongylocentrotus_purpuratus
X1WY43_Acyrthosiphon_pisum
D8LCS5_Ectocarpus_siliculosus
D7G5Z0_Ectocarpus_siliculosus
1
0,89
0,86
1
0,17
0,79
0,99
0,97
1
1
0,6
0,27
1
0,67
0,77
1
0,28
0,96
1
0,89
0,89
0,7
0.6

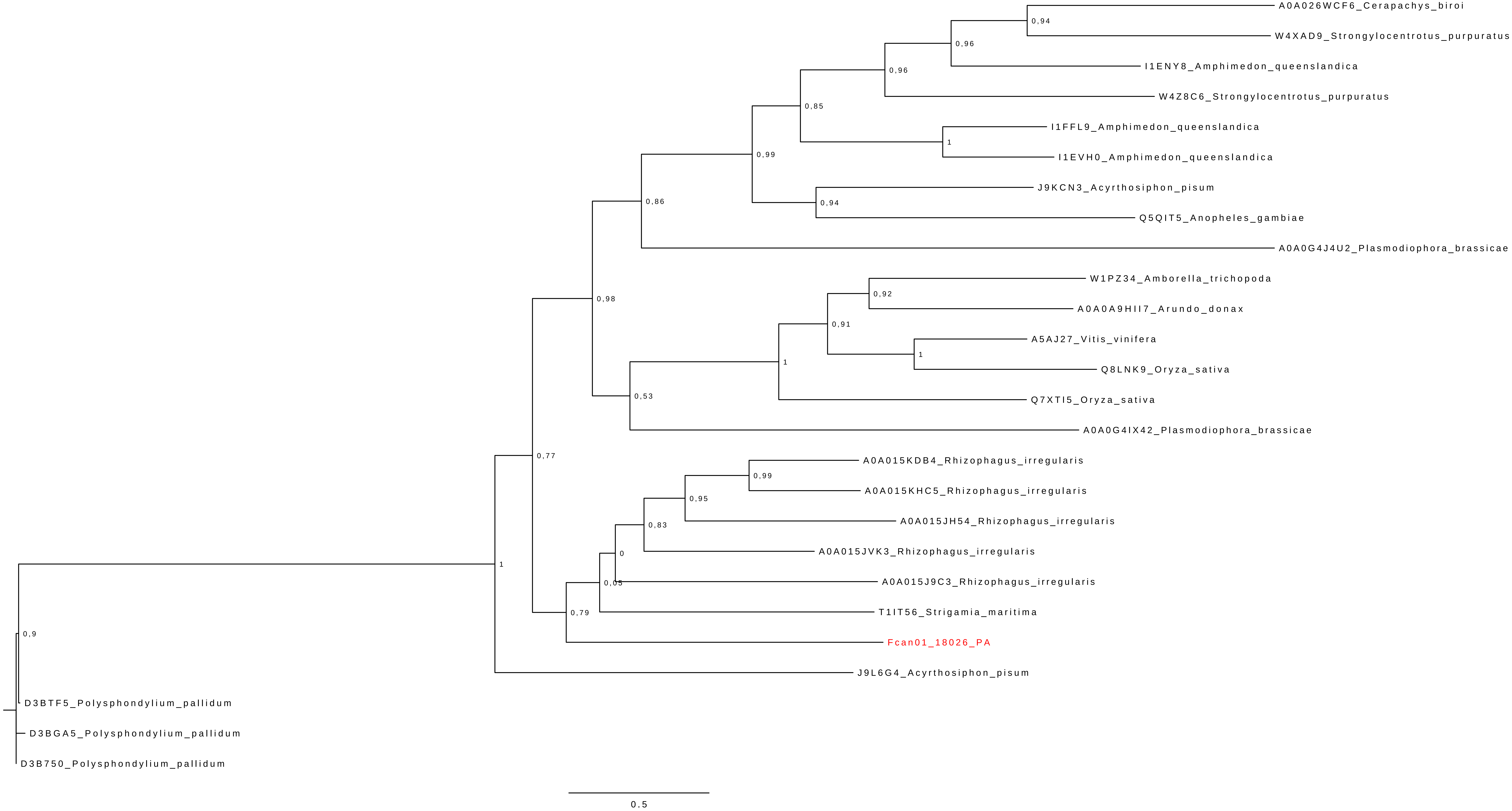
A0A026WCF6_Cerapachys_biroi
0,94
W4XAD9_Strongylocentrotus_purpuratus
0,96
I1ENY8_Amphimedon_queenslandica
0,96
W4Z8C6_Strongylocentrotus_purpuratus
0,85
I1FFL9_Amphimedon_queenslandica
1
I1EVH0_Amphimedon_queenslandica
0,99
J9KCN3_Acyrthosiphon_pisum
0,94
Q5QIT5_Anopheles_gambiae
0,86
A0A0G4J4U2_Plasmodiophora_brassicae
W1PZ34_Amborella_trichopoda
0,92
A0A0A9HII7_Arundo_donax
0,98
0,91
A5AJ27_Vitis_vinifera
1
Q8LNK9_Oryza_sativa
1
Q7XTI5_Oryza_sativa
0,53
A0A0G4IX42_Plasmodiophora_brassicae
A0A015KDB4_Rhizophagus_irregularis
0,77
0,99
A0A015KHC5_Rhizophagus_irregularis
0,95
A0A015JH54_Rhizophagus_irregularis
0,83
A0A015JVK3_Rhizophagus_irregularis
0
1
0,05
A0A015J9C3_Rhizophagus_irregularis
T1IT56_Strigamia_maritima
0,79
Fcan01_18026_PA
J9L6G4_Acyrthosiphon_pisum
0,9
D3BTF5_Polysphondylium_pallidum
D3BGA5_Polysphondylium_pallidum
D3B750_Polysphondylium_pallidum
0.5

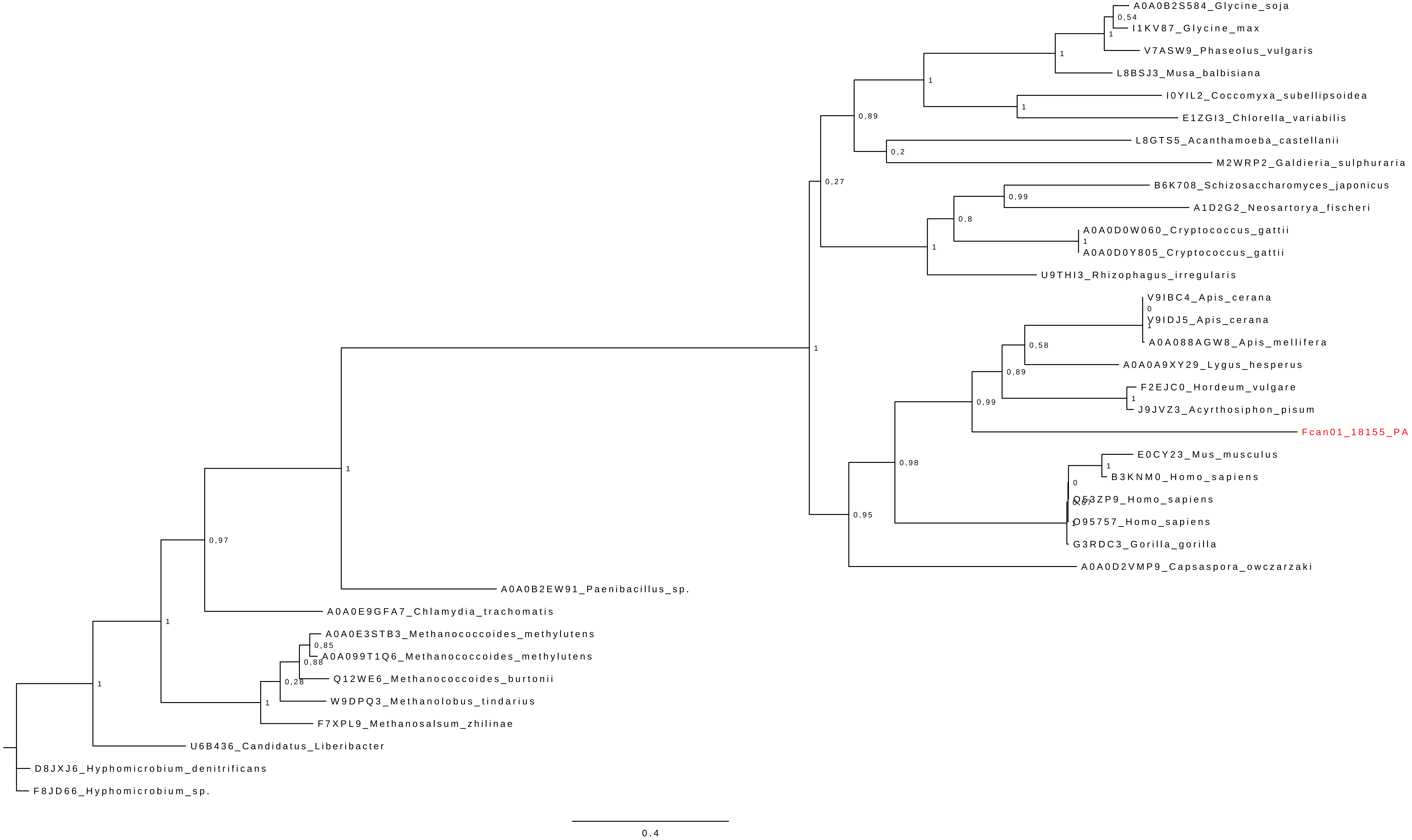
A0A0B2S584_Glycine_soja
I1KV87_Glycine_max
V7ASW9_Phaseolus_vulgaris
L8BSJ3_Musa_balbisiana
I0YIL2_Coccomyxa_subellipsoidea
E1ZGI3_Chlorella_variabilis
L8GTS5_Acanthamoeba_castellanii
M2WRP2_Galdieria_sulphuraria
B6K708_Schizosaccharomyces_japonicus
A1D2G2_Neosartorya_fischeri
A0A0D0W060_Cryptococcus_gattii
A0A0D0Y805_Cryptococcus_gattii
U9THI3_Rhizophagus_irregularis
V9IBC4_Apis_cerana
V9IDJ5_Apis_cerana
A0A088AGW8_Apis_mellifera
A0A0A9XY29_Lygus_hesperus
F2EJC0_Hordeum_vulgare
J9JVZ3_Acyrthosiphon_pisum
Fcan01_18155_PA
E0CY23_Mus_musculus
B3KNM0_Homo_sapiens
Q53ZP9_Homo_sapiens
O95757_Homo_sapiens
G3RDC3_Gorilla_gorilla
A0A0D2VMP9_Capsaspora_owczarzaki
A0A0B2EW91_Paenibacillus_sp.
A0A0E9GFA7_Chlamydia_trachomatis
A0A0E3STB3_Methanococcoides_methylutens
A0A099T1Q6_Methanococcoides_methylutens
Q12WE6_Methanococcoides_burtonii
W9DPQ3_Methanolobus_tindarius
F7XPL9_Methanosalsum_zhilinae
U6B436_Candidatus_Liberibacter
D8JXJ6_Hyphomicrobium_denitrificans
F8JD66_Hyphomicrobium_sp.
0.4

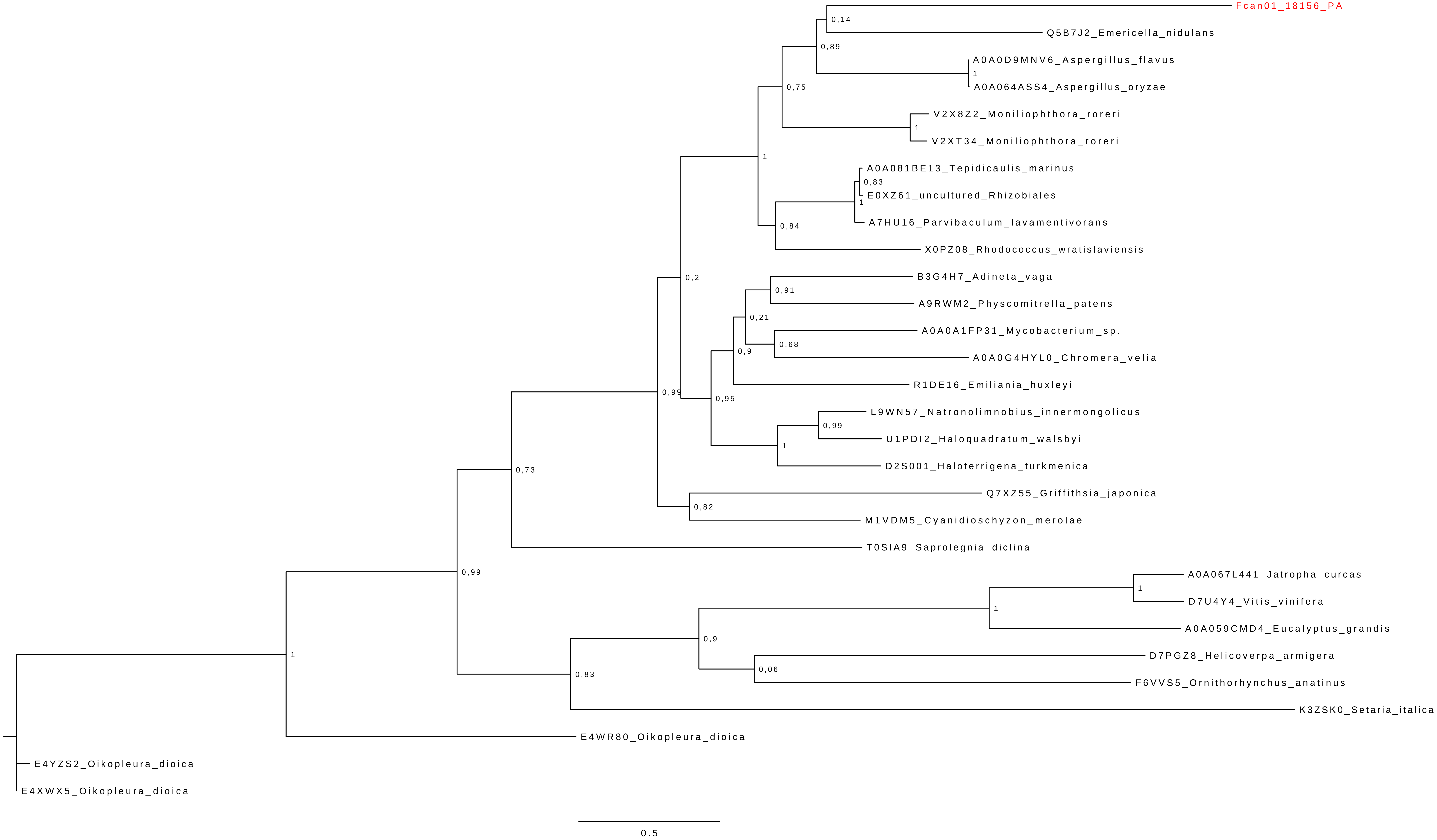

Fcan01_18156_PA
Q5B7J2_Emericella_nidulans
A0A0D9MNV6_Aspergillus_flavus
A0A064ASS4_Aspergillus_oryzae
V2X8Z2_Moniliophthora_roreri
V2XT34_Moniliophthora_roreri
A0A081BE13_Tepidicaulis_marinus
E0XZ61_uncultured_Rhizobiales
A7HU16_Parvibaculum_lavamentivorans
X0PZ08_Rhodococcus_wratislaviensis
B3G4H7_Adineta_vaga
A9RWM2_Physcomitrella_patens
A0A0A1FP31_Mycobacterium_sp.
A0A0G4HYL0_Chromera_velia
R1DE16_Emiliania_huxleyi
L9WN57_Natronolimnobius_innermongolicus
U1PDI2_Haloquadratum_walsbyi
D2S001_Haloterrigena_turkmenica
Q7XZ55_Griffithsia_japonica
M1VDM5_Cyanidioschyzon_merolae
T0SIA9_Saprolegnia_diclina
A0A067L441_Jatropha_curcas
D7U4Y4_Vitis_vinifera
A0A059CMD4_Eucalyptus_grandis
D7PGZ8_Helicoverpa_armigera
F6VVS5_Ornithorhynchus_anatinus
K3ZSK0_Setaria_italica
E4WR80_Oikopleura_dioica
E4YZS2_Oikopleura_dioica
E4XWX5_Oikopleura_dioica
0,14
0,89
1
0,75
1
1
0,83
1
0,84
0,2
0,91
0,21
0,68
0,9
0,99
0,95
0,99
1
0,82
0,73
0,99
1
1
0,9
0,06
0,83
1
0.5

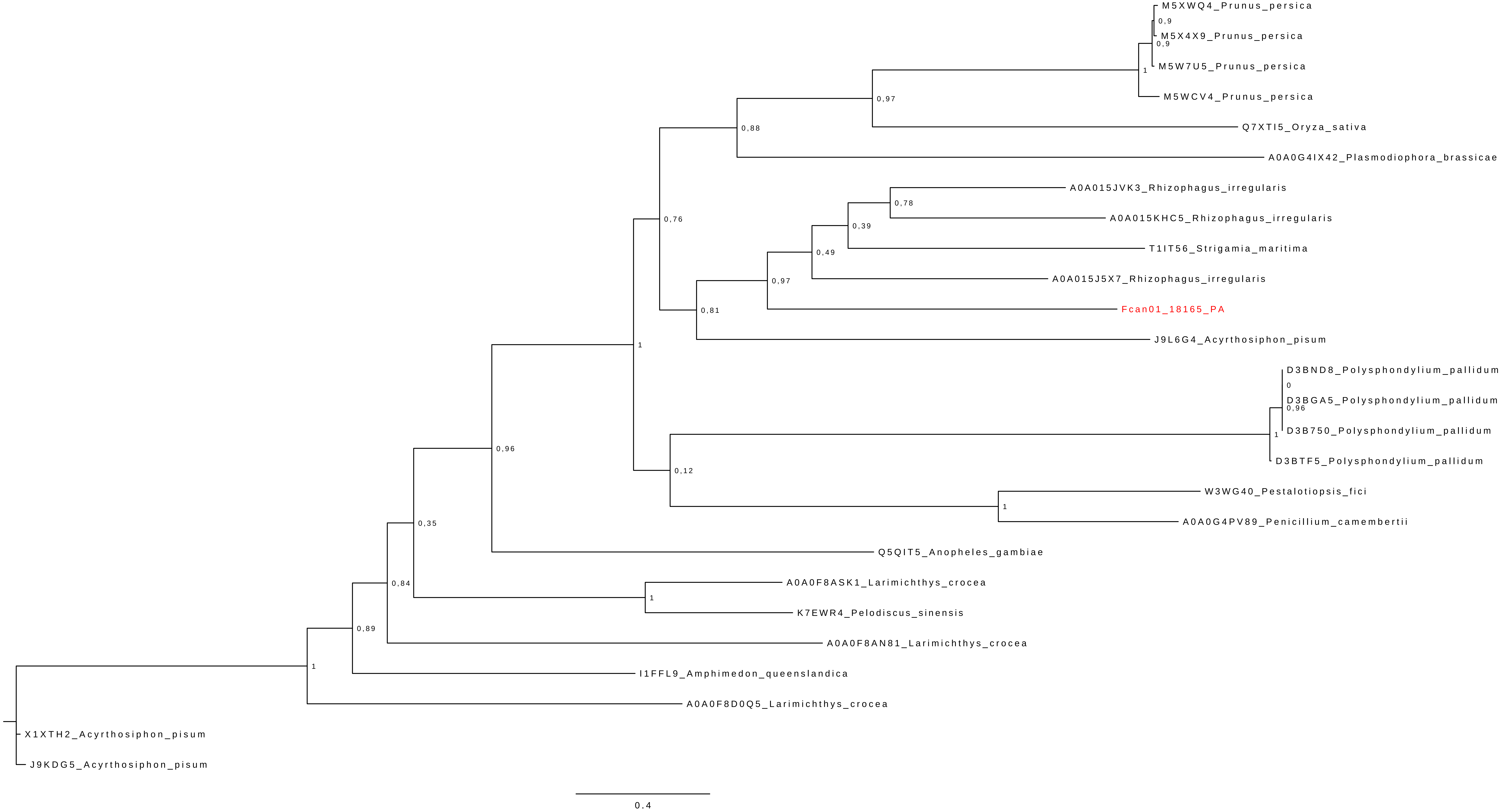
M5XWQ4_Prunus_persica
0,9
M5X4X9_Prunus_persica
0,9
M5W7U5_Prunus_persica
1
M5WCV4_Prunus_persica
0,97
Q7XTI5_Oryza_sativa
0,88
A0A0G4IX42_Plasmodiophora_brassicae
A0A015JVK3_Rhizophagus_irregularis
0,78
A0A015KHC5_Rhizophagus_irregularis
0,39
T1IT56_Strigamia_maritima
0,49
A0A015J5X7_Rhizophagus_irregularis
0,97
Fcan01_18165_PA
0,81
J9L6G4_Acyrthosiphon_pisum
0,76
1
D3BND8_Polysphondylium_pallidum
0
D3BGA5_Polysphondylium_pallidum
0,96
D3B750_Polysphondylium_pallidum
1
D3BTF5_Polysphondylium_pallidum
0,12
W3WG40_Pestalotiopsis_fici
1
A0A0G4PV89_Penicillium_camembertii
0,96
Q5QIT5_Anopheles_gambiae
0,35
A0A0F8ASK1_Larimichthys_crocea
1
K7EWR4_Pelodiscus_sinensis
0,84
A0A0F8AN81_Larimichthys_crocea
0,89
I1FFL9_Amphimedon_queenslandica
1
A0A0F8D0Q5_Larimichthys_crocea
X1XTH2_Acyrthosiphon_pisum
J9KDG5_Acyrthosiphon_pisum
0.4

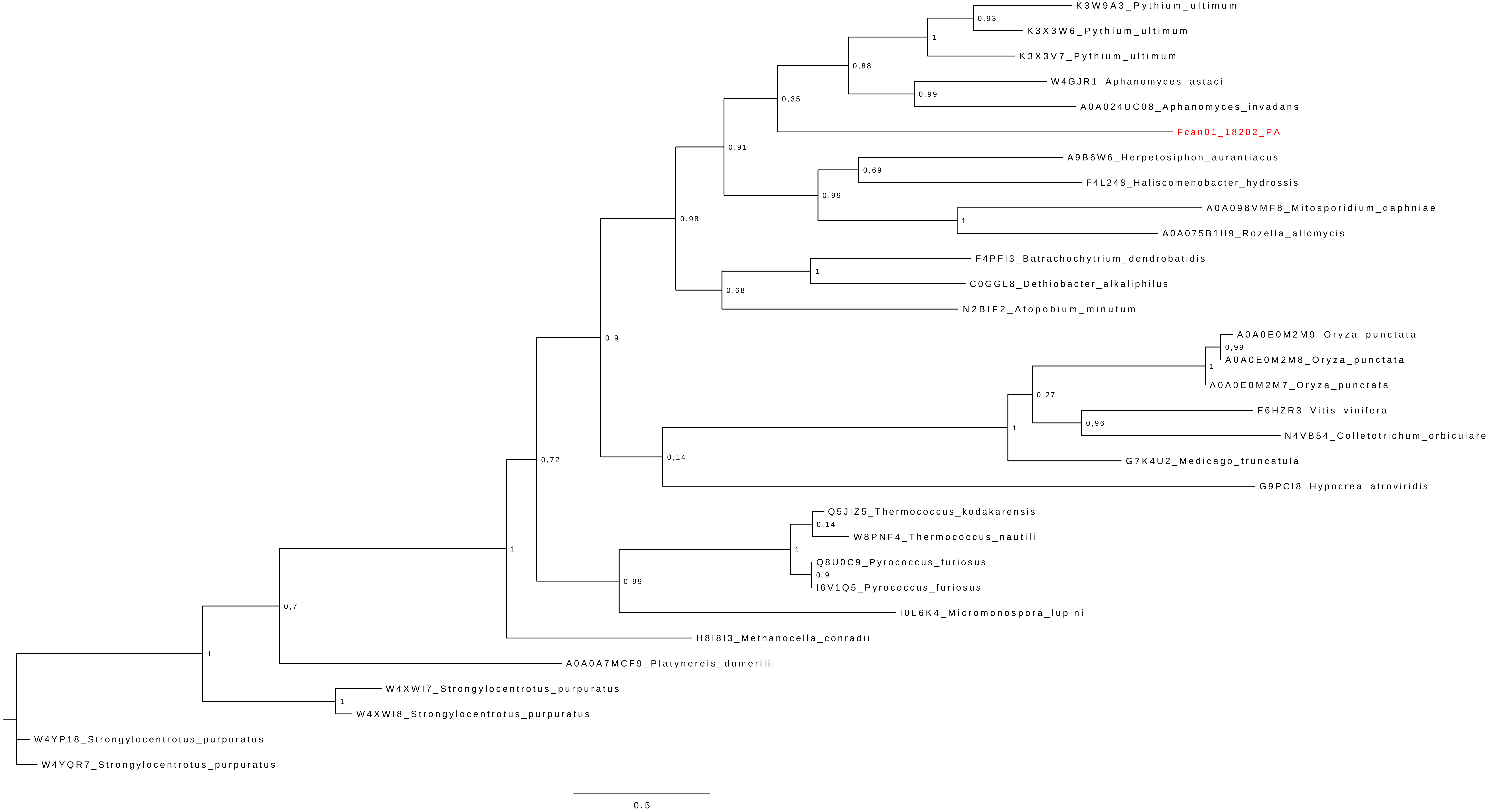
K3W9A3_Pythium_ultimum
0,93
K3X3W6_Pythium_ultimum
1
K3X3V7_Pythium_ultimum
0,88
W4GJR1_Aphanomyces_astaci
0,99
A0A024UC08_Aphanomyces_invadans
0,35
Fcan01_18202_PA
0,91
A9B6W6_Herpetosiphon_aurantiacus
0,69
F4L248_Haliscomenobacter_hydrossis
0,99
A0A098VMF8_Mitosporidium_daphniae
1
A0A075B1H9_Rozella_allomycis
0,98
F4PFI3_Batrachochytrium_dendrobatidis
1
C0GGL8_Dethiobacter_alkaliphilus
0,68
N2BIF2_Atopobium_minutum
0,9
A0A0E0M2M9_Oryza_punctata
0,99
A0A0E0M2M8_Oryza_punctata
1
A0A0E0M2M7_Oryza_punctata
0,27
F6HZR3_Vitis_vinifera
0,96
N4VB54_Colletotrichum_orbiculare
1
G7K4U2_Medicago_truncatula
0,14
G9PCI8_Hypocrea_atroviridis
0,72
Q5JIZ5_Thermococcus_kodakarensis
0,14
W8PNF4_Thermococcus_nautili
1
Q8U0C9_Pyrococcus_furiosus
0,9
I6V1Q5_Pyrococcus_furiosus
0,99
I0L6K4_Micromonospora_lupini
1
H8I8I3_Methanocella_conradii
0,7
A0A0A7MCF9_Platynereis_dumerilii
1
W4XWI7_Strongylocentrotus_purpuratus
1
W4XWI8_Strongylocentrotus_purpuratus
W4YP18_Strongylocentrotus_purpuratus
W4YQR7_Strongylocentrotus_purpuratus
0.5

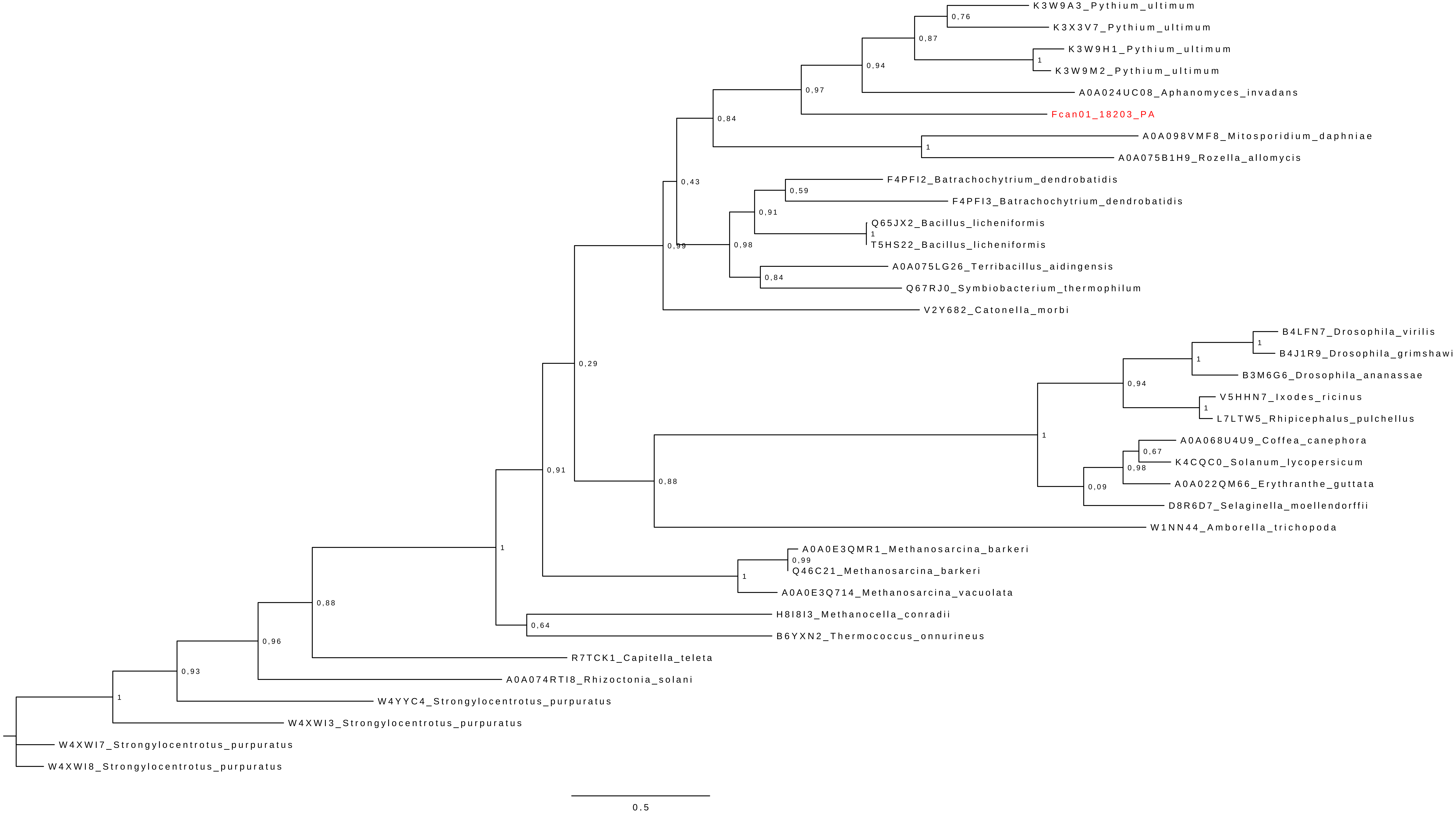
K3W9A3_Pythium_ultimum
K3X3V7_Pythium_ultimum
K3W9H1_Pythium_ultimum
K3W9M2_Pythium_ultimum
A0A024UC08_Aphanomyces_invadans
Fcan01_18203_PA
A0A098VMF8_Mitosporidium_daphniae
A0A075B1H9_Rozella_allomycis
F4PFI2_Batrachochytrium_dendrobatidis
F4PFI3_Batrachochytrium_dendrobatidis
Q65JX2_Bacillus_licheniformis
T5HS22_Bacillus_licheniformis
A0A075LG26_Terribacillus_aidingensis
Q67RJ0_Symbiobacterium_thermophilum
V2Y682_Catonella_morbi
B4LFN7_Drosophila_virilis
B4J1R9_Drosophila_grimshawi
B3M6G6_Drosophila_ananassae
V5HHN7_Ixodes_ricinus
L7LTW5_Rhipicephalus_pulchellus
A0A068U4U9_Coffea_canephora
K4CQC0_Solanum_lycopersicum
A0A022QM66_Erythranthe_guttata
D8R6D7_Selaginella_moellendorffii
W1NN44_Amborella_trichopoda
A0A0E3QMR1_Methanosarcina_barkeri
Q46C21_Methanosarcina_barkeri
A0A0E3Q714_Methanosarcina_vacuolata
H8I8I3_Methanocella_conradii
B6YXN2_Thermococcus_onnurineus
R7TCK1_Capitella_teleta
A0A074RTI8_Rhizoctonia_solani
W4YYC4_Strongylocentrotus_purpuratus
W4XWI3_Strongylocentrotus_purpuratus
W4XWI7_Strongylocentrotus_purpuratus
W4XWI8_Strongylocentrotus_purpuratus
0.5

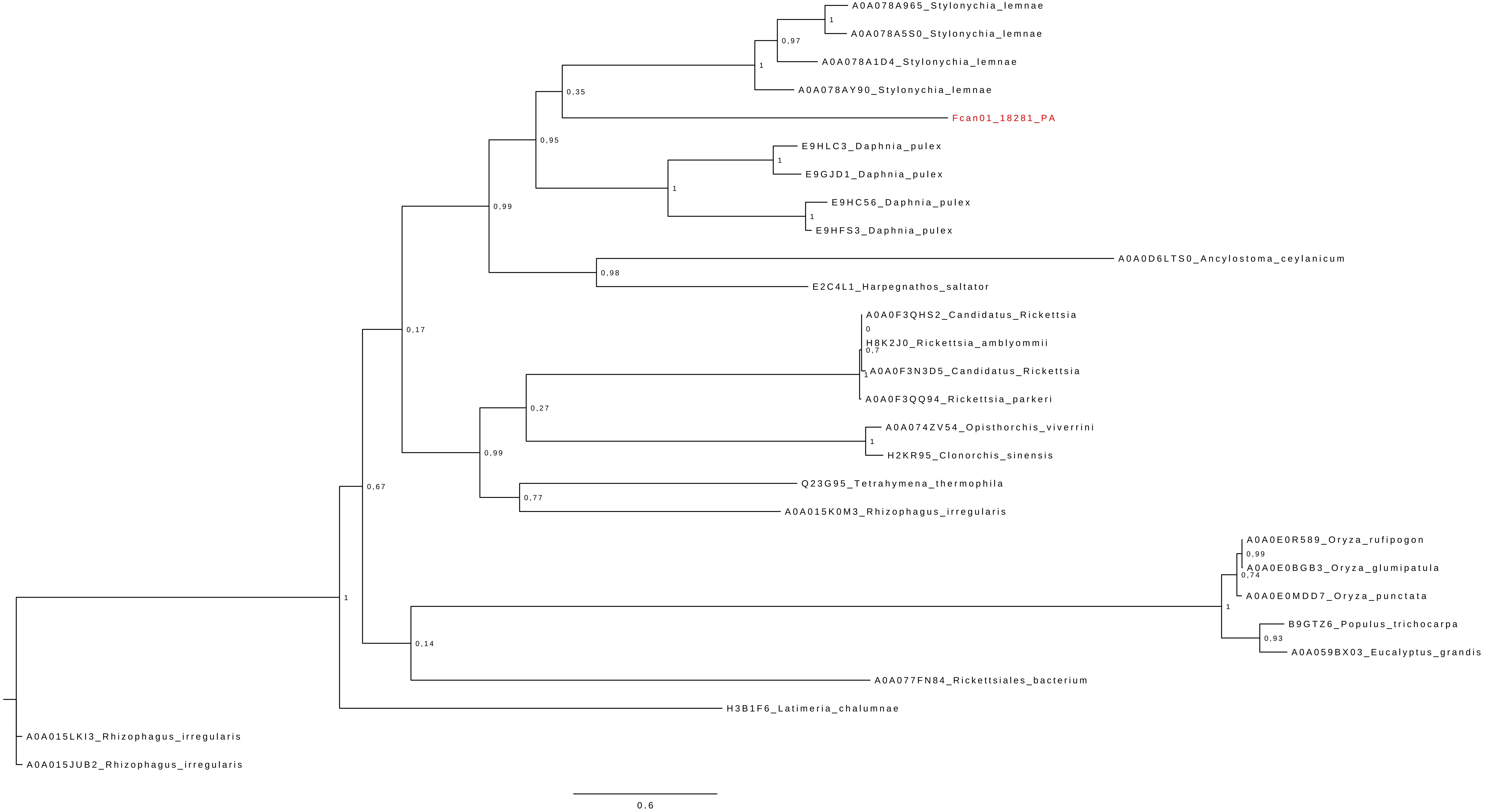
A0A078A965_Stylonychia_lemnae
1
A0A078A5S0_Stylonychia_lemnae
0,97
A0A078A1D4_Stylonychia_lemnae
1
0,35
A0A078AY90_Stylonychia_lemnae
Fcan01_18281_PA
E9HLC3_Daphnia_pulex
0,95
1
E9GJD1_Daphnia_pulex
1
E9HC56_Daphnia_pulex
0,99
1
E9HFS3_Daphnia_pulex
A0A0D6LTS0_Ancylostoma_ceylanicum
0,98
E2C4L1_Harpegnathos_saltator
A0A0F3QHS2_Candidatus_Rickettsia
0
0,17
H8K2J0_Rickettsia_amblyommii
0,7
A0A0F3N3D5_Candidatus_Rickettsia
1
A0A0F3QQ94_Rickettsia_parkeri
0,27
A0A074ZV54_Opisthorchis_viverrini
1
0,99
H2KR95_Clonorchis_sinensis
Q23G95_Tetrahymena_thermophila
0,67
0,77
A0A015K0M3_Rhizophagus_irregularis
A0A0E0R589_Oryza_rufipogon
0,99
A0A0E0BGB3_Oryza_glumipatula
0,74
A0A0E0MDD7_Oryza_punctata
1
1
B9GTZ6_Populus_trichocarpa
0,93
0,14
A0A059BX03_Eucalyptus_grandis
A0A077FN84_Rickettsiales_bacterium
H3B1F6_Latimeria_chalumnae
A0A015LKI3_Rhizophagus_irregularis
A0A015JUB2_Rhizophagus_irregularis
0.6

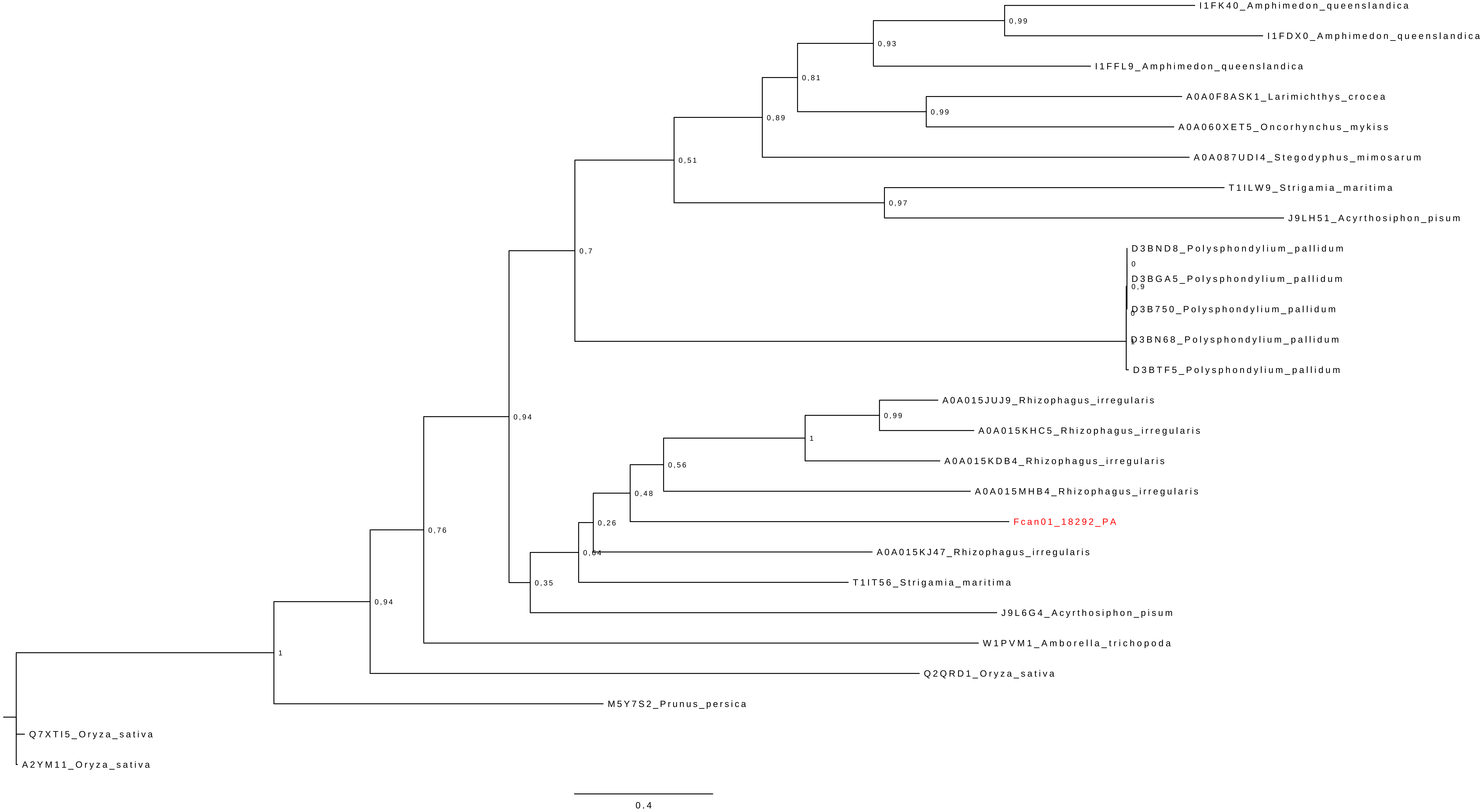
I1FK40_Amphimedon_queenslandica
I1FDX0_Amphimedon_queenslandica
I1FFL9_Amphimedon_queenslandica
A0A0F8ASK1_Larimichthys_crocea
A0A060XET5_Oncorhynchus_mykiss
A0A087UDI4_Stegodyphus_mimosarum
T1ILW9_Strigamia_maritima
J9LH51_Acyrthosiphon_pisum
D3BND8_Polysphondylium_pallidum
D3BGA5_Polysphondylium_pallidum
D3B750_Polysphondylium_pallidum
D3BN68_Polysphondylium_pallidum
D3BTF5_Polysphondylium_pallidum
A0A015JUJ9_Rhizophagus_irregularis
A0A015KHC5_Rhizophagus_irregularis
A0A015KDB4_Rhizophagus_irregularis
A0A015MHB4_Rhizophagus_irregularis
Fcan01_18292_PA
A0A015KJ47_Rhizophagus_irregularis
T1IT56_Strigamia_maritima
J9L6G4_Acyrthosiphon_pisum
W1PVM1_Amborella_trichopoda
Q2QRD1_Oryza_sativa
M5Y7S2_Prunus_persica
Q7XTI5_Oryza_sativa
A2YM11_Oryza_sativa
0,99
0,93
0,81
0,99
0,89
0,51
0,97
0,7
0
0,9
0
1
0,94
0,99
1
0,56
0,48
0,26
0,64
0,35
0,76
0,94
1
0.4

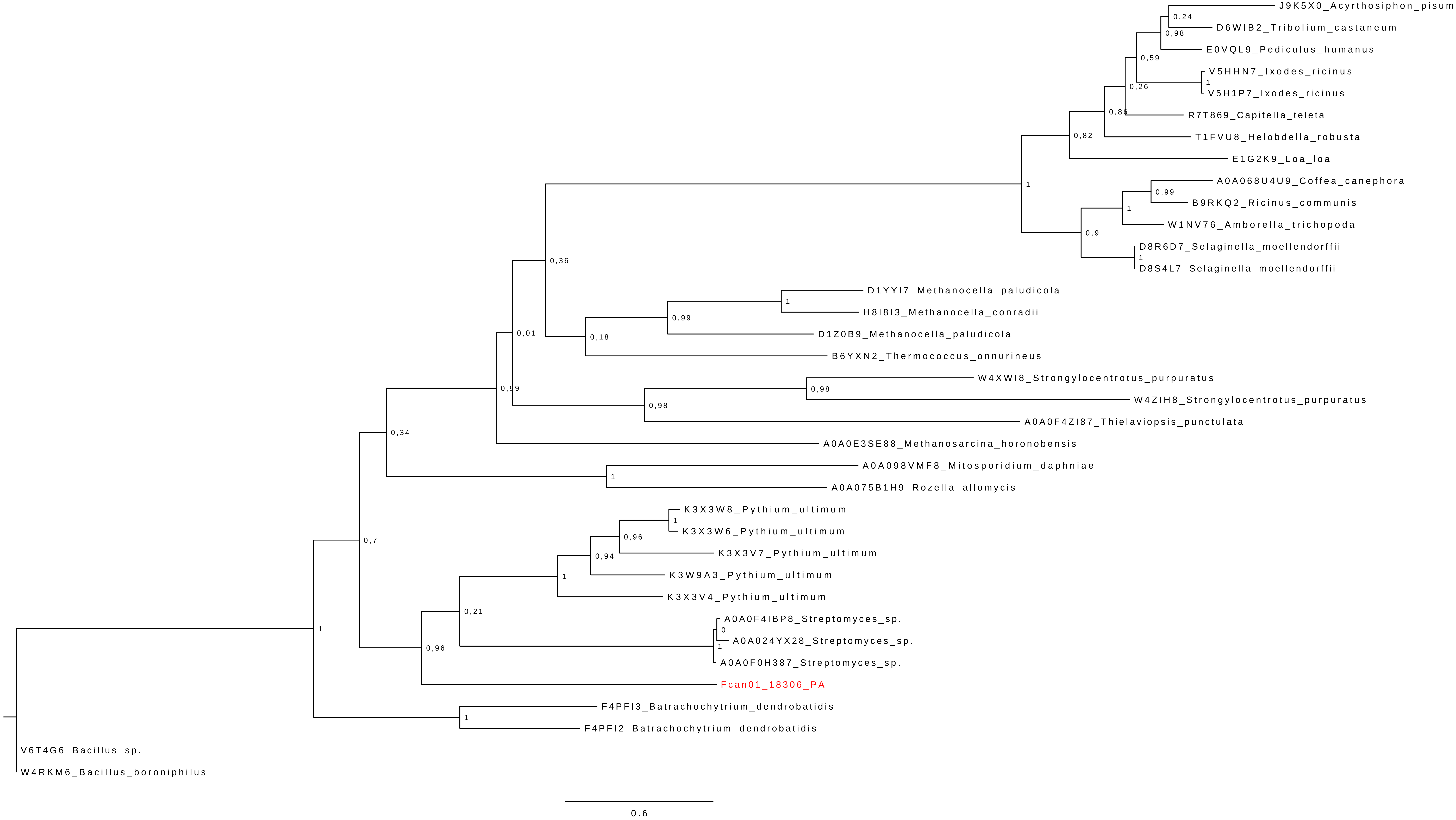
J9K5X0_Acyrthosiphon_pisum
0,24
D6WIB2_Tribolium_castaneum
0,98
E0VQL9_Pediculus_humanus
0,59
V5HHN7_Ixodes_ricinus
1
V5H1P7_Ixodes_ricinus
0,26
R7T869_Capitella_teleta
0,86
T1FVU8_Helobdella_robusta
0,82
E1G2K9_Loa_loa
1
A0A068U4U9_Coffea_canephora
0,99
B9RKQ2_Ricinus_communis
1
W1NV76_Amborella_trichopoda
0,9
D8R6D7_Selaginella_moellendorffii
1
D8S4L7_Selaginella_moellendorffii
0,36
D1YYI7_Methanocella_paludicola
1
H8I8I3_Methanocella_conradii
0,99
D1Z0B9_Methanocella_paludicola
0,18
B6YXN2_Thermococcus_onnurineus
0,01
W4XWI8_Strongylocentrotus_purpuratus
0,98
W4ZIH8_Strongylocentrotus_purpuratus
0,99
0,98
A0A0F4ZI87_Thielaviopsis_punctulata
0,34
A0A0E3SE88_Methanosarcina_horonobensis
A0A098VMF8_Mitosporidium_daphniae
1
A0A075B1H9_Rozella_allomycis
K3X3W8_Pythium_ultimum
1
K3X3W6_Pythium_ultimum
0,96
K3X3V7_Pythium_ultimum
0,94
K3W9A3_Pythium_ultimum
1
K3X3V4_Pythium_ultimum
0,7
0,21
A0A0F4IBP8_Streptomyces_sp.
0
A0A024YX28_Streptomyces_sp.
1
A0A0F0H387_Streptomyces_sp.
1
0,96
Fcan01_18306_PA
F4PFI3_Batrachochytrium_dendrobatidis
1
F4PFI2_Batrachochytrium_dendrobatidis
V6T4G6_Bacillus_sp.
W4RKM6_Bacillus_boroniphilus
0.6

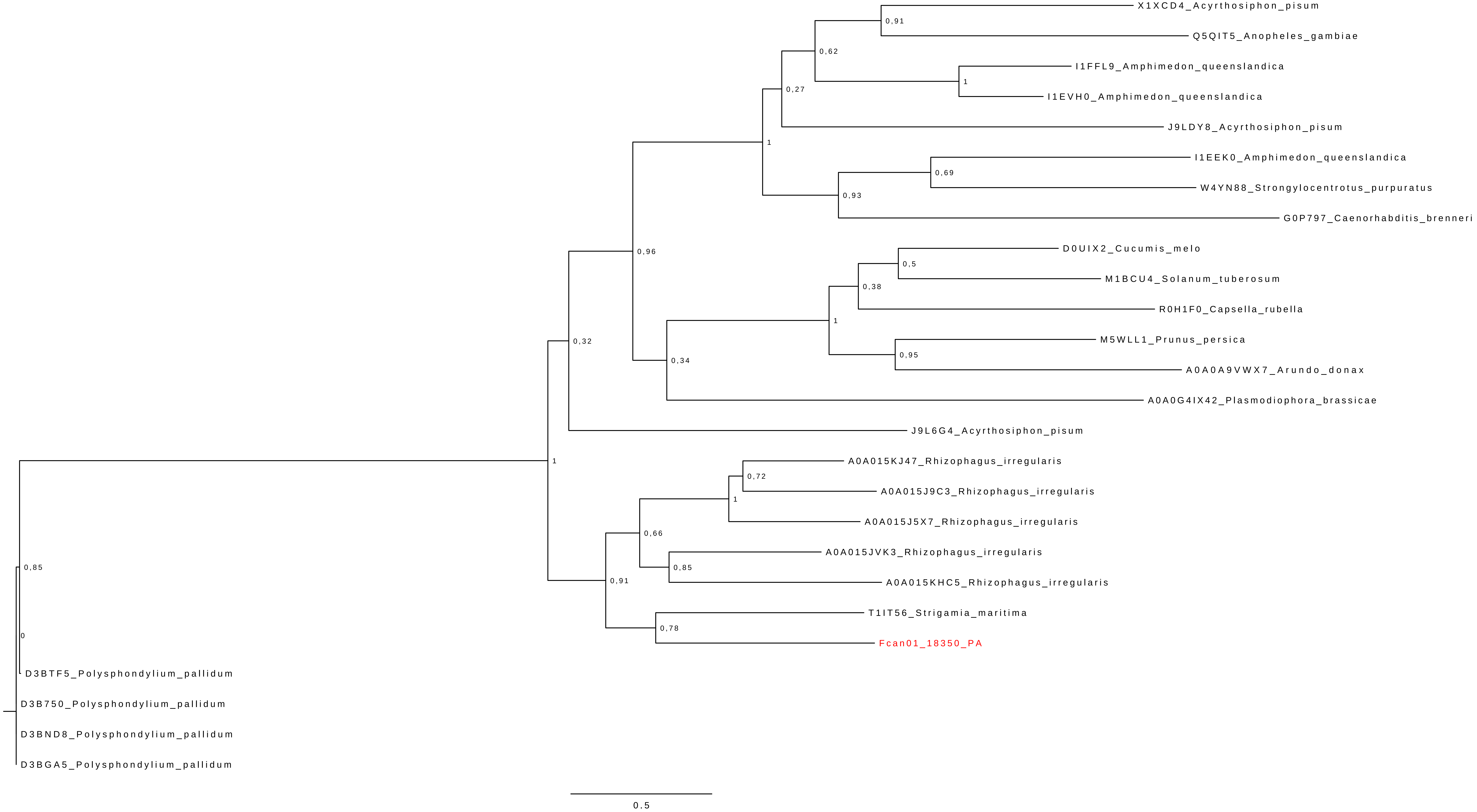
X1XCD4_Acyrthosiphon_pisum
Q5QIT5_Anopheles_gambiae
I1FFL9_Amphimedon_queenslandica
I1EVH0_Amphimedon_queenslandica
J9LDY8_Acyrthosiphon_pisum
I1EEK0_Amphimedon_queenslandica
W4YN88_Strongylocentrotus_purpuratus
G0P797_Caenorhabditis_brenneri
D0UIX2_Cucumis_melo
M1BCU4_Solanum_tuberosum
R0H1F0_Capsella_rubella
M5WLL1_Prunus_persica
A0A0A9VWX7_Arundo_donax
A0A0G4IX42_Plasmodiophora_brassicae
J9L6G4_Acyrthosiphon_pisum
A0A015KJ47_Rhizophagus_irregularis
A0A015J9C3_Rhizophagus_irregularis
A0A015J5X7_Rhizophagus_irregularis
A0A015JVK3_Rhizophagus_irregularis
A0A015KHC5_Rhizophagus_irregularis
T1IT56_Strigamia_maritima
Fcan01_18350_PA
D3BTF5_Polysphondylium_pallidum
D3B750_Polysphondylium_pallidum
D3BND8_Polysphondylium_pallidum
D3BGA5_Polysphondylium_pallidum
0,91
0,62
1
0,27
1
0,69
0,93
0,96
0,5
0,38
1
0,95
0,34
0,32
1
0,72
1
0,66
0,85
0,91
0,78
0,85
0
0.5

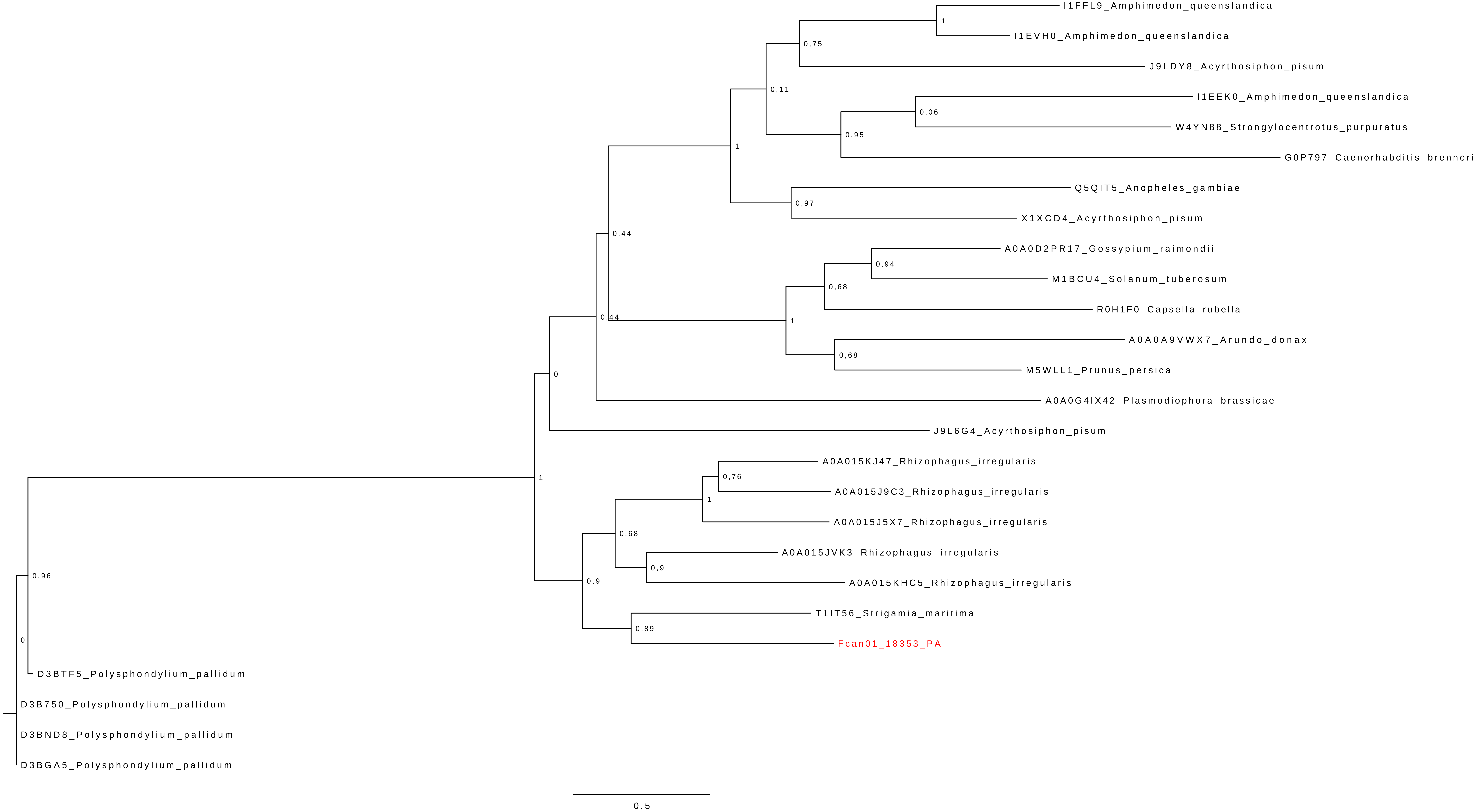
I1FFL9_Amphimedon_queenslandica
I1EVH0_Amphimedon_queenslandica
J9LDY8_Acyrthosiphon_pisum
I1EEK0_Amphimedon_queenslandica
W4YN88_Strongylocentrotus_purpuratus
G0P797_Caenorhabditis_brenneri
Q5QIT5_Anopheles_gambiae
X1XCD4_Acyrthosiphon_pisum
A0A0D2PR17_Gossypium_raimondii
M1BCU4_Solanum_tuberosum
R0H1F0_Capsella_rubella
A0A0A9VWX7_Arundo_donax
M5WLL1_Prunus_persica
A0A0G4IX42_Plasmodiophora_brassicae
J9L6G4_Acyrthosiphon_pisum
A0A015KJ47_Rhizophagus_irregularis
A0A015J9C3_Rhizophagus_irregularis
A0A015J5X7_Rhizophagus_irregularis
A0A015JVK3_Rhizophagus_irregularis
A0A015KHC5_Rhizophagus_irregularis
T1IT56_Strigamia_maritima
Fcan01_18353_PA
D3BTF5_Polysphondylium_pallidum
D3B750_Polysphondylium_pallidum
D3BND8_Polysphondylium_pallidum
D3BGA5_Polysphondylium_pallidum
1
0,75
0,11
0,06
0,95
1
0,97
0,44
0,94
0,68
0,44
1
0,68
0
1
0,76
1
0,68
0,9
0,9
0,89
0,96
0
0.5

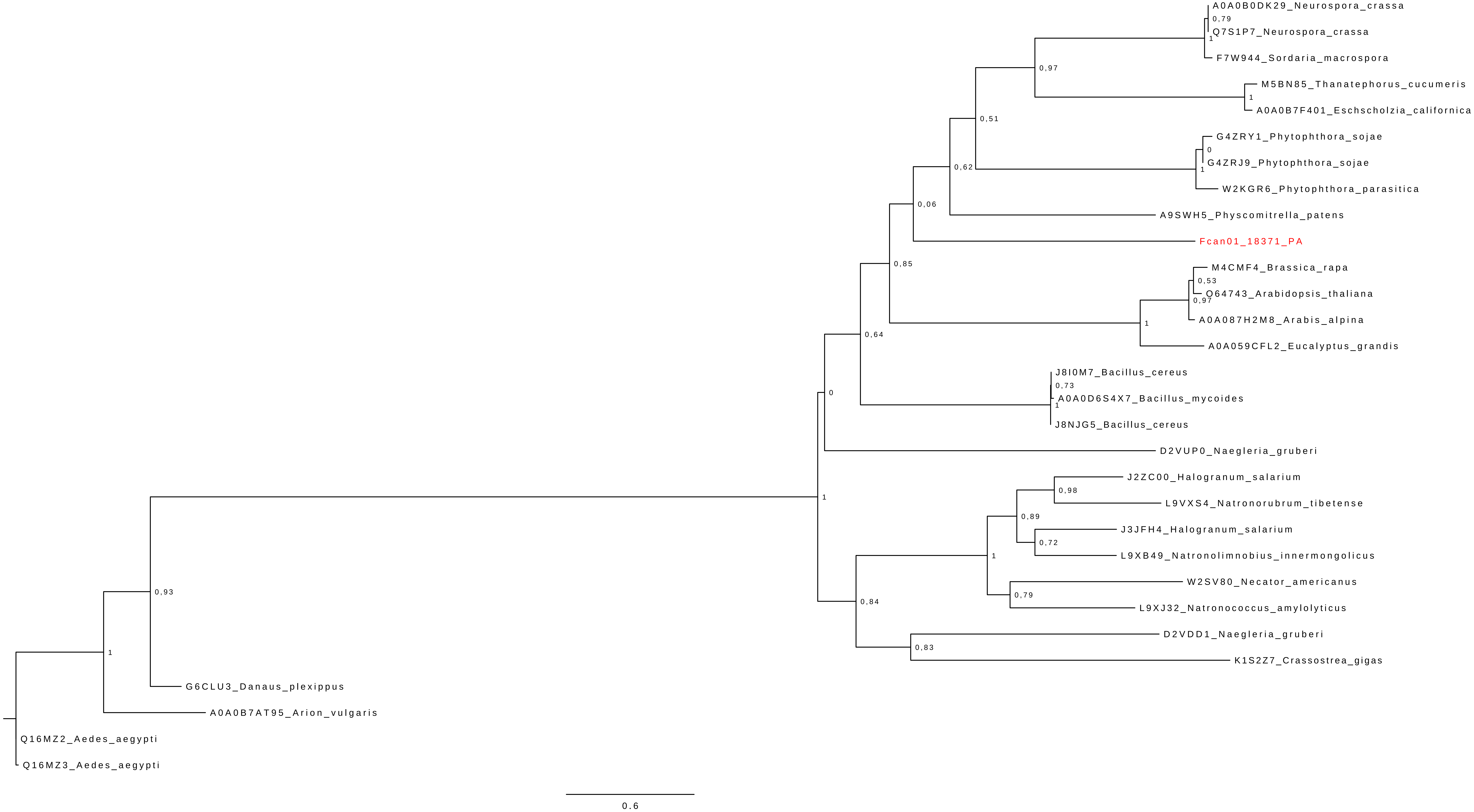
A0A0B0DK29_Neurospora_crassa
0,79
Q7S1P7_Neurospora_crassa
1
F7W944_Sordaria_macrospora
0,97
M5BN85_Thanatephorus_cucumeris
1
A0A0B7F401_Eschscholzia_californica
0,51
G4ZRY1_Phytophthora_sojae
0
G4ZRJ9_Phytophthora_sojae
1
W2KGR6_Phytophthora_parasitica
0,62
A9SWH5_Physcomitrella_patens
0,06
Fcan01_18371_PA
0,85
M4CMF4_Brassica_rapa
0,53
O64743_Arabidopsis_thaliana
0,97
A0A087H2M8_Arabis_alpina
1
A0A059CFL2_Eucalyptus_grandis
0,64
J8I0M7_Bacillus_cereus
0,73
A0A0D6S4X7_Bacillus_mycoides
1
J8NJG5_Bacillus_cereus
0
D2VUP0_Naegleria_gruberi
J2ZC00_Halogranum_salarium
0,98
L9VXS4_Natronorubrum_tibetense
0,89
J3JFH4_Halogranum_salarium
0,72
L9XB49_Natronolimnobius_innermongolicus
1
W2SV80_Necator_americanus
0,79
L9XJ32_Natronococcus_amylolyticus
0,84
D2VDD1_Naegleria_gruberi
0,83
K1S2Z7_Crassostrea_gigas
1
0,93
G6CLU3_Danaus_plexippus
1
A0A0B7AT95_Arion_vulgaris
Q16MZ2_Aedes_aegypti
Q16MZ3_Aedes_aegypti
0.6

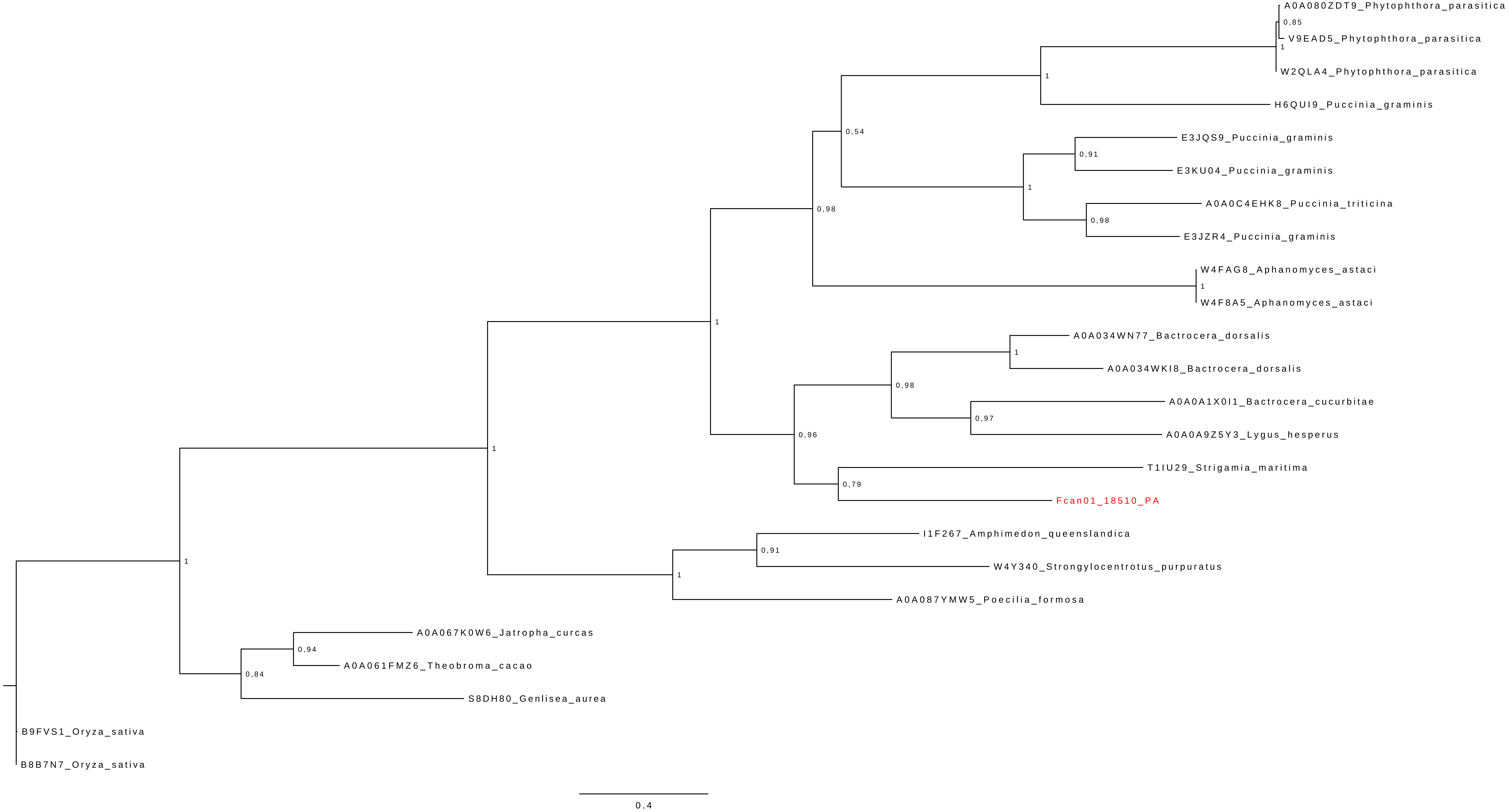
A0A080ZDT9_Phytophthora_parasitica
0,85
V9EAD5_Phytophthora_parasitica
1
W2QLA4_Phytophthora_parasitica
1
H6QUI9_Puccinia_graminis
0,54
E3JQS9_Puccinia_graminis
0,91
E3KU04_Puccinia_graminis
1
A0A0C4EHK8_Puccinia_triticina
0,98
E3JZR4_Puccinia_graminis
0,98
W4FAG8_Aphanomyces_astaci
1
W4F8A5_Aphanomyces_astaci
1
A0A034WN77_Bactrocera_dorsalis
1
A0A034WKI8_Bactrocera_dorsalis
0,98
A0A0A1X0I1_Bactrocera_cucurbitae
0,97
A0A0A9Z5Y3_Lygus_hesperus
0,96
T1IU29_Strigamia_maritima
0,79
Fcan01_18510_PA
1
I1F267_Amphimedon_queenslandica
0,91
W4Y340_Strongylocentrotus_purpuratus
1
A0A087YMW5_Poecilia_formosa
1
A0A067K0W6_Jatropha_curcas
0,94
A0A061FMZ6_Theobroma_cacao
0,84
S8DH80_Genlisea_aurea
B9FVS1_Oryza_sativa
B8B7N7_Oryza_sativa
0.4

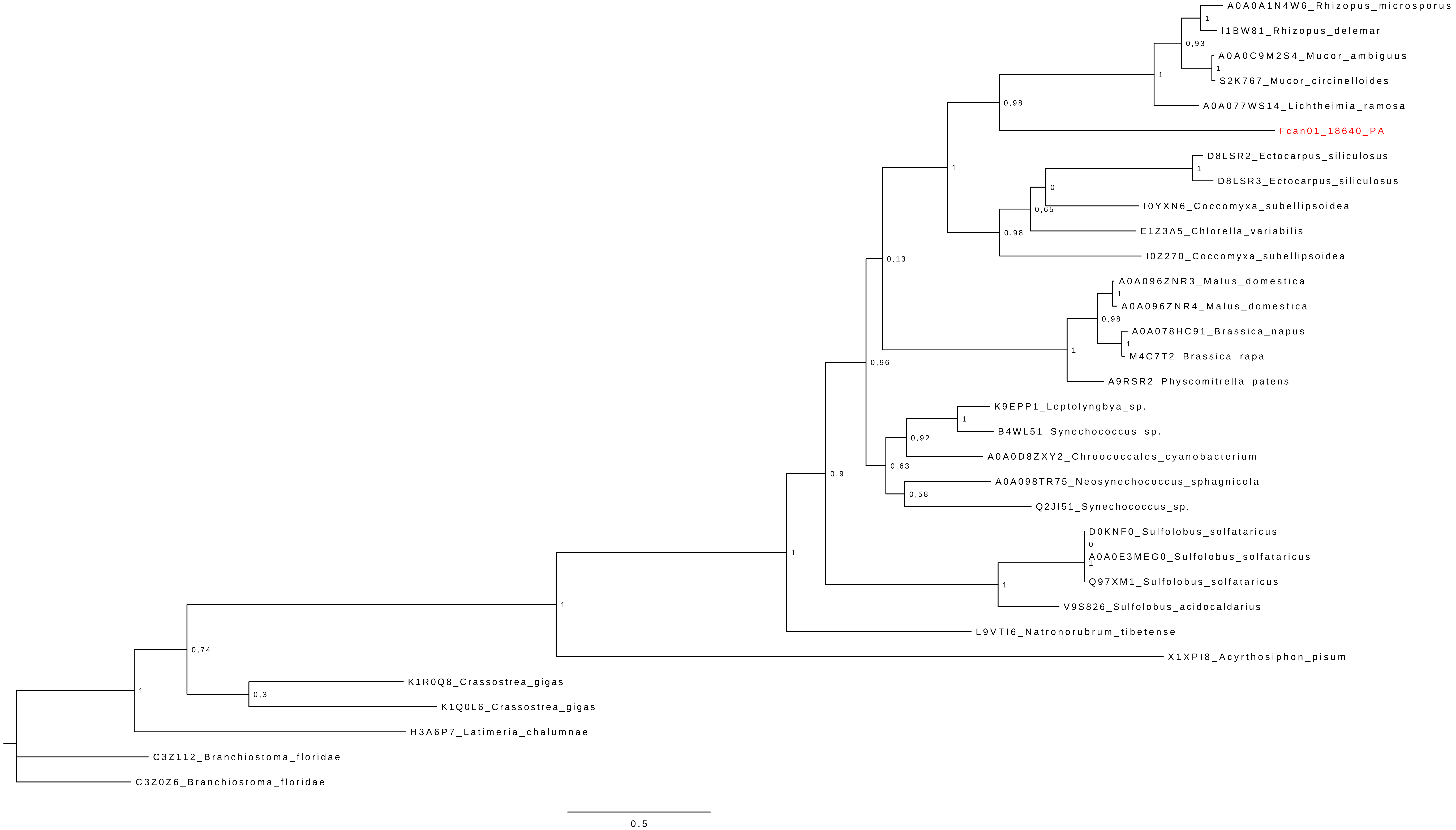
A0A0A1N4W6_Rhizopus_microsporus
I1BW81_Rhizopus_delemar
A0A0C9M2S4_Mucor_ambiguus
S2K767_Mucor_circinelloides
A0A077WS14_Lichtheimia_ramosa
Fcan01_18640_PA
D8LSR2_Ectocarpus_siliculosus
D8LSR3_Ectocarpus_siliculosus
I0YXN6_Coccomyxa_subellipsoidea
E1Z3A5_Chlorella_variabilis
I0Z270_Coccomyxa_subellipsoidea
A0A096ZNR3_Malus_domestica
A0A096ZNR4_Malus_domestica
A0A078HC91_Brassica_napus
M4C7T2_Brassica_rapa
A9RSR2_Physcomitrella_patens
K9EPP1_Leptolyngbya_sp.
B4WL51_Synechococcus_sp.
A0A0D8ZXY2_Chroococcales_cyanobacterium
A0A098TR75_Neosynechococcus_sphagnicola
Q2JI51_Synechococcus_sp.
D0KNF0_Sulfolobus_solfataricus
A0A0E3MEG0_Sulfolobus_solfataricus
Q97XM1_Sulfolobus_solfataricus
V9S826_Sulfolobus_acidocaldarius
L9VTI6_Natronorubrum_tibetense
X1XPI8_Acyrthosiphon_pisum
K1R0Q8_Crassostrea_gigas
K1Q0L6_Crassostrea_gigas
H3A6P7_Latimeria_chalumnae
C3Z112_Branchiostoma_floridae
C3Z0Z6_Branchiostoma_floridae
1
0,93
1
1
0,98
1
0
0,65
0,98
0,13
1
0,98
1
1
0,96
1
0,92
0,63
0,58
0,9
0
1
1
1
1
0,74
0,3
1
0.5

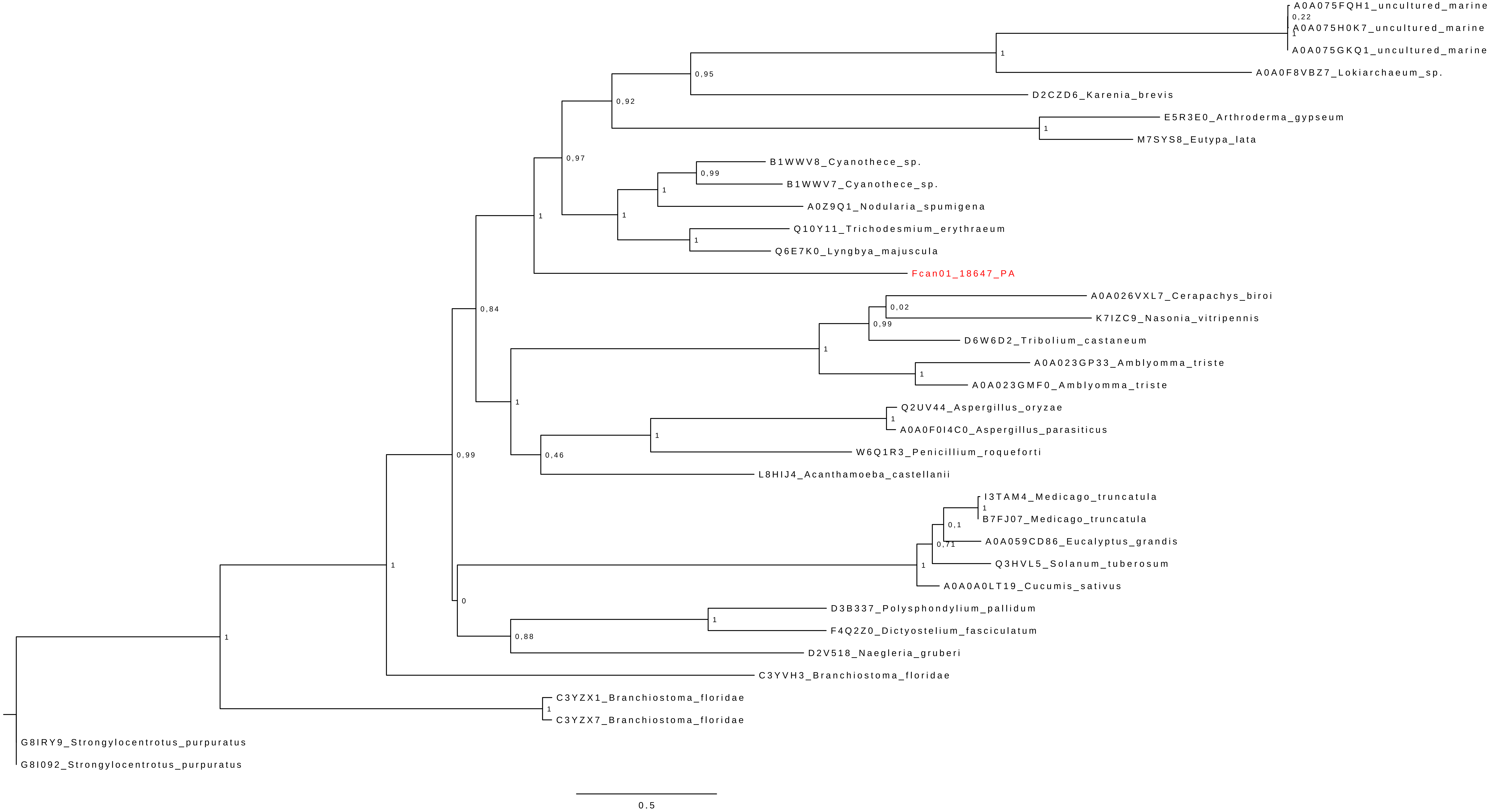
A0A075FQH1_uncultured_marine
A0A075H0K7_uncultured_marine
A0A075GKQ1_uncultured_marine
A0A0F8VBZ7_Lokiarchaeum_sp.
D2CZD6_Karenia_brevis
E5R3E0_Arthroderma_gypseum
M7SYS8_Eutypa_lata
B1WWV8_Cyanothece_sp.
B1WWV7_Cyanothece_sp.
A0Z9Q1_Nodularia_spumigena
Q10Y11_Trichodesmium_erythraeum
Q6E7K0_Lyngbya_majuscula
Fcan01_18647_PA
A0A026VXL7_Cerapachys_biroi
K7IZC9_Nasonia_vitripennis
D6W6D2_Tribolium_castaneum
A0A023GP33_Amblyomma_triste
A0A023GMF0_Amblyomma_triste
Q2UV44_Aspergillus_oryzae
A0A0F0I4C0_Aspergillus_parasiticus
W6Q1R3_Penicillium_roqueforti
L8HIJ4_Acanthamoeba_castellanii
I3TAM4_Medicago_truncatula
B7FJ07_Medicago_truncatula
A0A059CD86_Eucalyptus_grandis
Q3HVL5_Solanum_tuberosum
A0A0A0LT19_Cucumis_sativus
D3B337_Polysphondylium_pallidum
F4Q2Z0_Dictyostelium_fasciculatum
D2V518_Naegleria_gruberi
C3YVH3_Branchiostoma_floridae
C3YZX1_Branchiostoma_floridae
C3YZX7_Branchiostoma_floridae
G8IRY9_Strongylocentrotus_purpuratus
G8I092_Strongylocentrotus_purpuratus
0.5

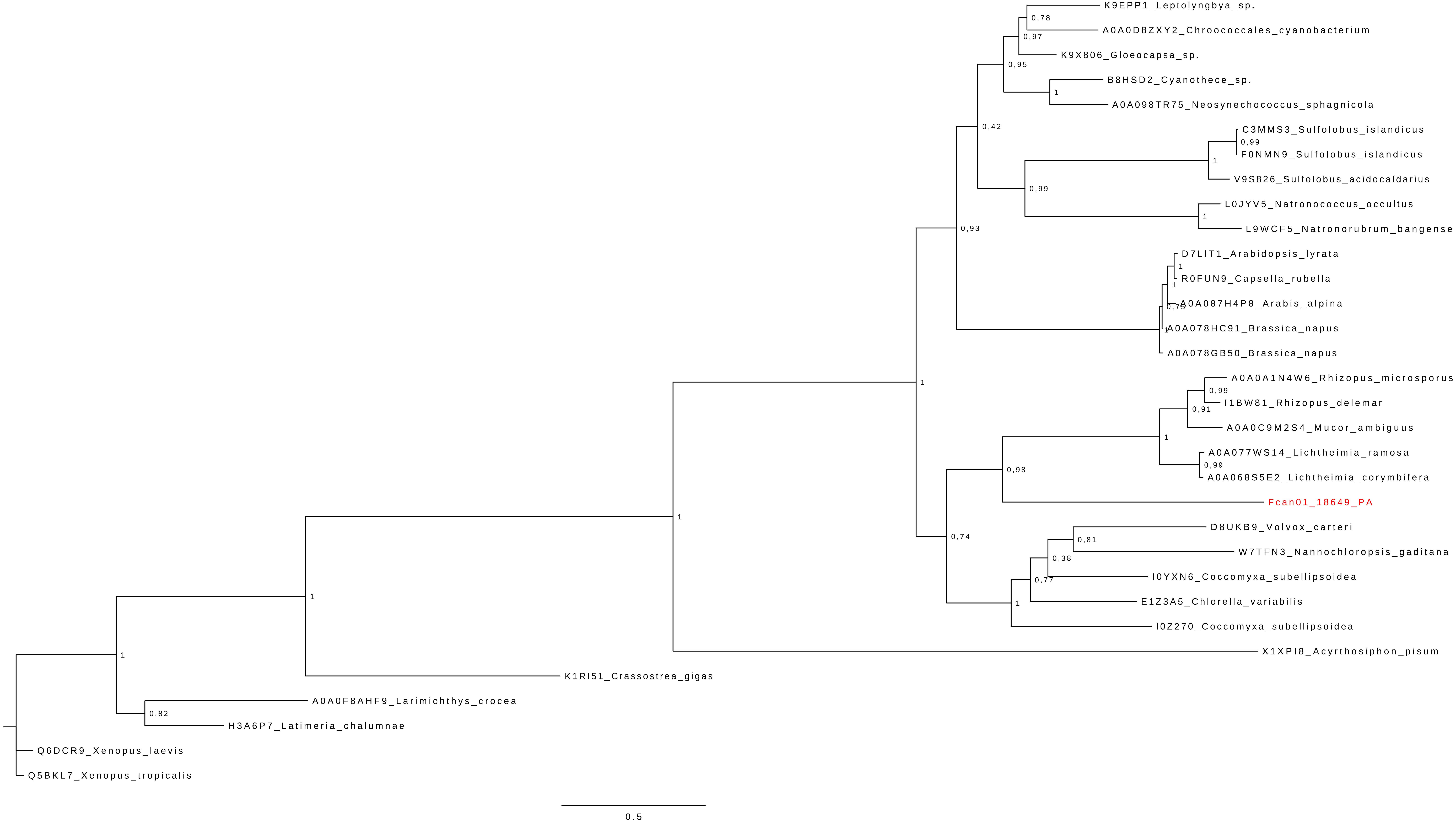
K9EPP1_Leptolyngbya_sp.
A0A0D8ZXY2_Chroococcales_cyanobacterium
K9X806_Gloeocapsa_sp.
B8HSD2_Cyanothece_sp.
A0A098TR75_Neosynechococcus_sphagnicola
C3MMS3_Sulfolobus_islandicus
F0NMN9_Sulfolobus_islandicus
V9S826_Sulfolobus_acidocaldarius
L0JYV5_Natronococcus_occultus
L9WCF5_Natronorubrum_bangense
D7LIT1_Arabidopsis_lyrata
R0FUN9_Capsella_rubella
A0A087H4P8_Arabis_alpina
A0A078HC91_Brassica_napus
A0A078GB50_Brassica_napus
A0A0A1N4W6_Rhizopus_microsporus
I1BW81_Rhizopus_delemar
A0A0C9M2S4_Mucor_ambiguus
A0A077WS14_Lichtheimia_ramosa
A0A068S5E2_Lichtheimia_corymbifera
Fcan01_18649_PA
D8UKB9_Volvox_carteri
W7TFN3_Nannochloropsis_gaditana
I0YXN6_Coccomyxa_subellipsoidea
E1Z3A5_Chlorella_variabilis
I0Z270_Coccomyxa_subellipsoidea
X1XPI8_Acyrthosiphon_pisum
K1RI51_Crassostrea_gigas
A0A0F8AHF9_Larimichthys_crocea
H3A6P7_Latimeria_chalumnae
Q6DCR9_Xenopus_laevis
Q5BKL7_Xenopus_tropicalis
0.5

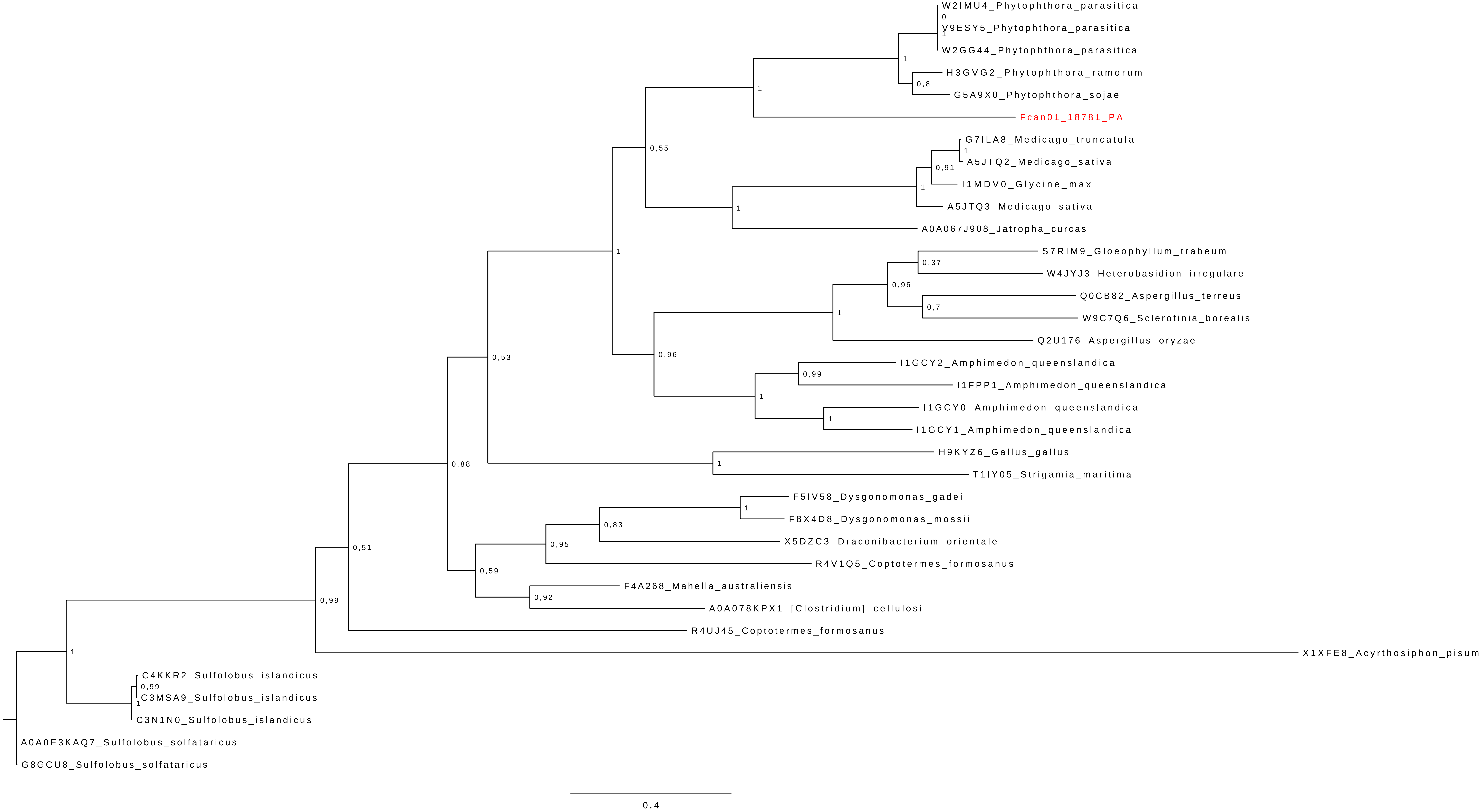
W2IMU4_Phytophthora_parasitica
V9ESY5_Phytophthora_parasitica
W2GG44_Phytophthora_parasitica
H3GVG2_Phytophthora_ramorum
G5A9X0_Phytophthora_sojae
Fcan01_18781_PA
G7ILA8_Medicago_truncatula
A5JTQ2_Medicago_sativa
I1MDV0_Glycine_max
A5JTQ3_Medicago_sativa
A0A067J908_Jatropha_curcas
S7RIM9_Gloeophyllum_trabeum
W4JYJ3_Heterobasidion_irregulare
Q0CB82_Aspergillus_terreus
W9C7Q6_Sclerotinia_borealis
Q2U176_Aspergillus_oryzae
I1GCY2_Amphimedon_queenslandica
I1FPP1_Amphimedon_queenslandica
I1GCY0_Amphimedon_queenslandica
I1GCY1_Amphimedon_queenslandica
H9KYZ6_Gallus_gallus
T1IY05_Strigamia_maritima
F5IV58_Dysgonomonas_gadei
F8X4D8_Dysgonomonas_mossii
X5DZC3_Draconibacterium_orientale
R4V1Q5_Coptotermes_formosanus
F4A268_Mahella_australiensis
A0A078KPX1_[Clostridium]_cellulosi
R4UJ45_Coptotermes_formosanus
X1XFE8_Acyrthosiphon_pisum
C4KKR2_Sulfolobus_islandicus
C3MSA9_Sulfolobus_islandicus
C3N1N0_Sulfolobus_islandicus
A0A0E3KAQ7_Sulfolobus_solfataricus
G8GCU8_Sulfolobus_solfataricus
0,55
0,8
0,91
0,37
0,96
0,7
0,99
0,53
0,88
0,83
0,95
0,59
0,92
0,51
0,99
0.4

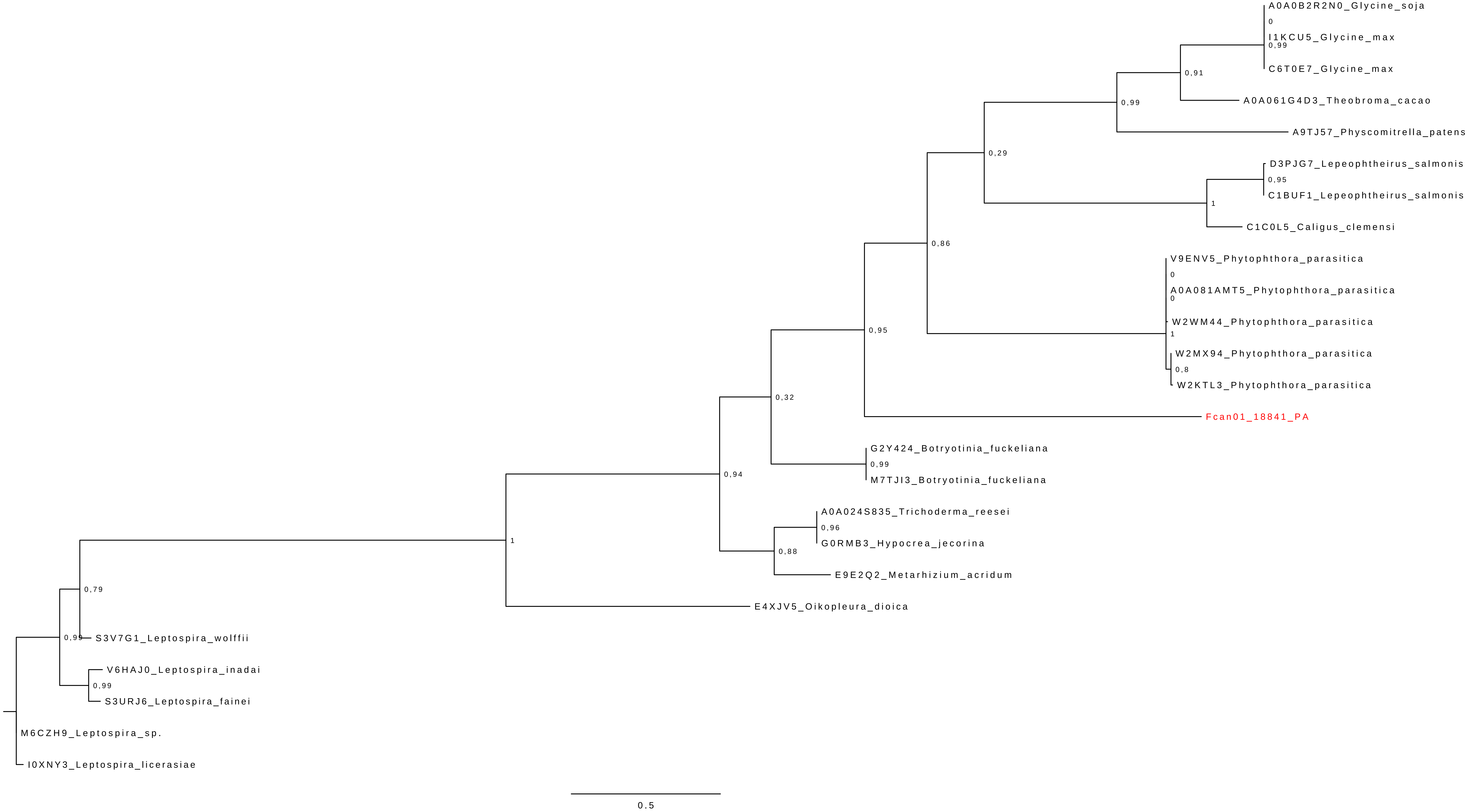

A0A0B2R2N0_Glycine_soja
0
I1KCU5_Glycine_max
0,99
C6T0E7_Glycine_max
0,91
A0A061G4D3_Theobroma_cacao
0,99
A9TJ57_Physcomitrella_patens
0,29
D3PJG7_Lepeophtheirus_salmonis
0,95
C1BUF1_Lepeophtheirus_salmonis
1
C1C0L5_Caligus_clemensi
0,86
V9ENV5_Phytophthora_parasitica
0
A0A081AMT5_Phytophthora_parasitica
0
W2WM44_Phytophthora_parasitica
0,95
1
W2MX94_Phytophthora_parasitica
0,8
W2KTL3_Phytophthora_parasitica
0,32
Fcan01_18841_PA
G2Y424_Botryotinia_fuckeliana
0,99
M7TJI3_Botryotinia_fuckeliana
0,94
A0A024S835_Trichoderma_reesei
0,96
G0RMB3_Hypocrea_jecorina
1
0,88
E9E2Q2_Metarhizium_acridum
0,79
E4XJV5_Oikopleura_dioica
0,99
S3V7G1_Leptospira_wolffii
V6HAJ0_Leptospira_inadai
0,99
S3URJ6_Leptospira_fainei
M6CZH9_Leptospira_sp.
I0XNY3_Leptospira_licerasiae
0.5

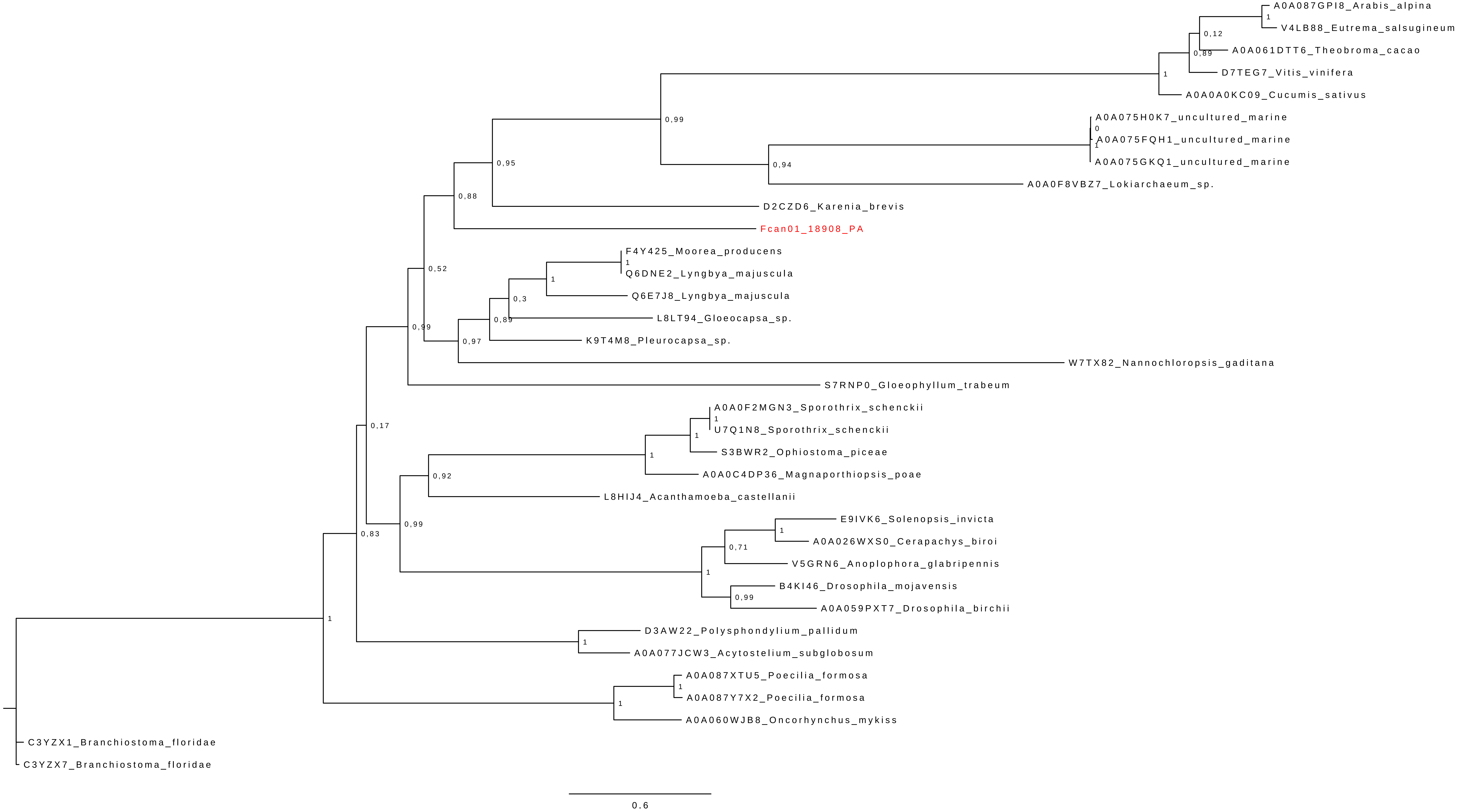

A0A087GPI8_Arabis_alpina
V4LB88_Eutrema_salsugineum
A0A061DTT6_Theobroma_cacao
D7TEG7_Vitis_vinifera
A0A0A0KC09_Cucumis_sativus
A0A075H0K7_uncultured_marine
A0A075FQH1_uncultured_marine
A0A075GKQ1_uncultured_marine
A0A0F8VBZ7_Lokiarchaeum_sp.
D2CZD6_Karenia_brevis
Fcan01_18908_PA
F4Y425_Moorea_producens
Q6DNE2_Lyngbya_majuscula
Q6E7J8_Lyngbya_majuscula
L8LT94_Gloeocapsa_sp.
K9T4M8_Pleurocapsa_sp.
W7TX82_Nannochloropsis_gaditana
S7RNP0_Gloeophyllum_trabeum
A0A0F2MGN3_Sporothrix_schenckii
U7Q1N8_Sporothrix_schenckii
S3BWR2_Ophiostoma_piceae
A0A0C4DP36_Magnaporthiopsis_poae
L8HIJ4_Acanthamoeba_castellanii
E9IVK6_Solenopsis_invicta
A0A026WXS0_Cerapachys_biroi
V5GRN6_Anoplophora_glabripennis
B4KI46_Drosophila_mojavensis
A0A059PXT7_Drosophila_birchii
D3AW22_Polysphondylium_pallidum
A0A077JCW3_Acytostelium_subglobosum
A0A087XTU5_Poecilia_formosa
A0A087Y7X2_Poecilia_formosa
A0A060WJB8_Oncorhynchus_mykiss
C3YZX1_Branchiostoma_floridae
C3YZX7_Branchiostoma_floridae
1
0,12
0,89
1
0
1
0,99
0,94
0,95
0,88
1
1
0,3
0,89
0,97
0,52
0,99
0,17
1
1
1
0,92
1
0,71
1
0,99
0,99
0,83
1
1
1
1
0.6

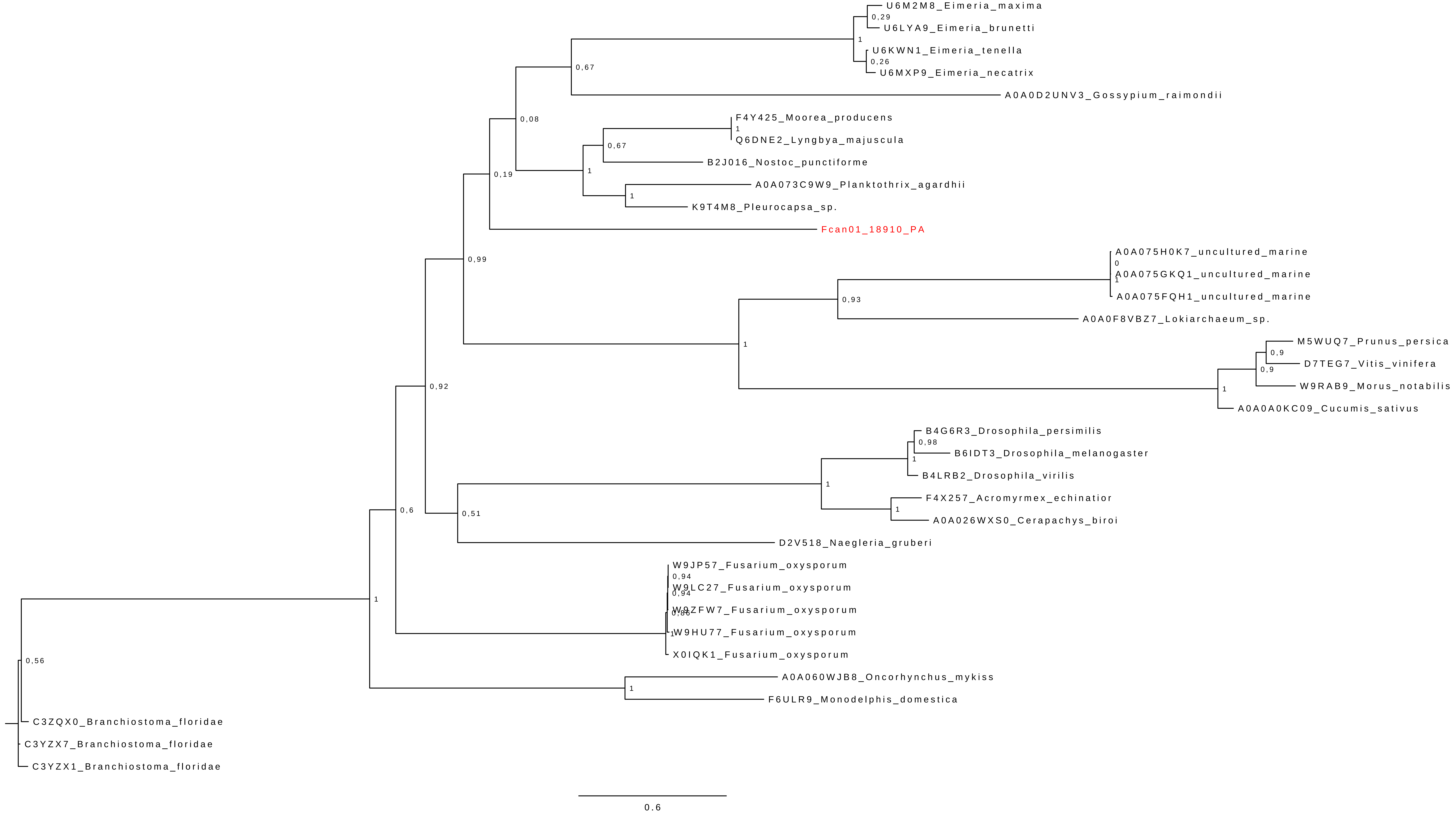
U6M2M8_Eimeria_maxima
U6LYA9_Eimeria_brunetti
U6KWN1_Eimeria_tenella
U6MXP9_Eimeria_necatrix
A0A0D2UNV3_Gossypium_raimondii
F4Y425_Moorea_producens
Q6DNE2_Lyngbya_majuscula
B2J016_Nostoc_punctiforme
A0A073C9W9_Planktothrix_agardhii
K9T4M8_Pleurocapsa_sp.
Fcan01_18910_PA
A0A075H0K7_uncultured_marine
A0A075GKQ1_uncultured_marine
A0A075FQH1_uncultured_marine
A0A0F8VBZ7_Lokiarchaeum_sp.
M5WUQ7_Prunus_persica
D7TEG7_Vitis_vinifera
W9RAB9_Morus_notabilis
A0A0A0KC09_Cucumis_sativus
B4G6R3_Drosophila_persimilis
B6IDT3_Drosophila_melanogaster
B4LRB2_Drosophila_virilis
F4X257_Acromyrmex_echinatior
A0A026WXS0_Cerapachys_biroi
D2V518_Naegleria_gruberi
W9JP57_Fusarium_oxysporum
W9LC27_Fusarium_oxysporum
W9ZFW7_Fusarium_oxysporum
W9HU77_Fusarium_oxysporum
X0IQK1_Fusarium_oxysporum
A0A060WJB8_Oncorhynchus_mykiss
F6ULR9_Monodelphis_domestica
C3ZQX0_Branchiostoma_floridae
C3YZX7_Branchiostoma_floridae
C3YZX1_Branchiostoma_floridae
0.6

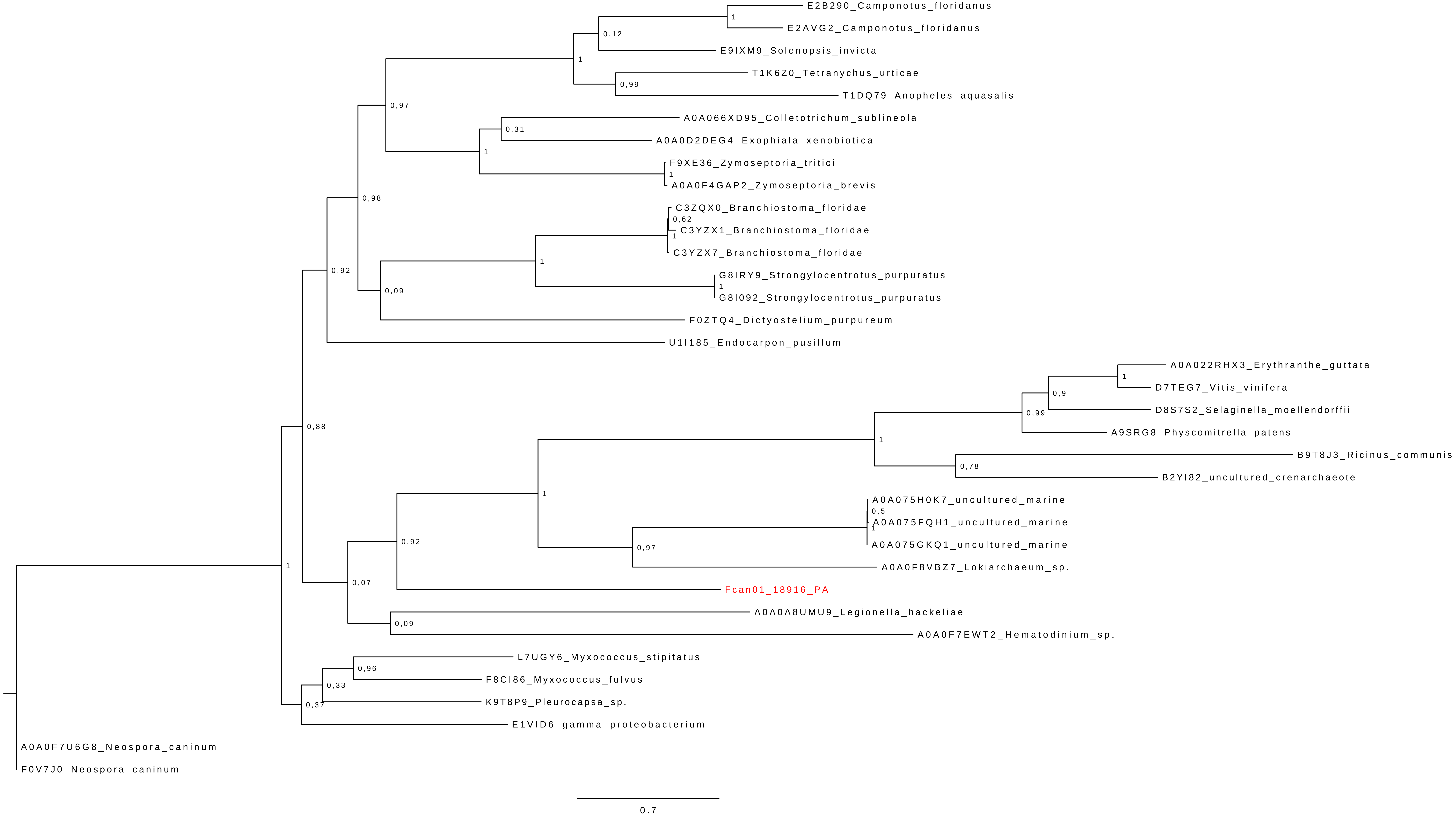
E2B290_Camponotus_floridanus
E2AVG2_Camponotus_floridanus
E9IXM9_Solenopsis_invicta
T1K6Z0_Tetranychus_urticae
T1DQ79_Anopheles_aquasalis
A0A066XD95_Colletotrichum_sublineola
A0A0D2DEG4_Exophiala_xenobiotica
F9XE36_Zymoseptoria_tritici
A0A0F4GAP2_Zymoseptoria_brevis
C3ZQX0_Branchiostoma_floridae
C3YZX1_Branchiostoma_floridae
C3YZX7_Branchiostoma_floridae
G8IRY9_Strongylocentrotus_purpuratus
G8I092_Strongylocentrotus_purpuratus
F0ZTQ4_Dictyostelium_purpureum
U1I185_Endocarpon_pusillum
A0A022RHX3_Erythranthe_guttata
D7TEG7_Vitis_vinifera
D8S7S2_Selaginella_moellendorffii
A9SRG8_Physcomitrella_patens
B9T8J3_Ricinus_communis
B2YI82_uncultured_crenarchaeote
A0A075H0K7_uncultured_marine
A0A075FQH1_uncultured_marine
A0A075GKQ1_uncultured_marine
A0A0F8VBZ7_Lokiarchaeum_sp.
Fcan01_18916_PA
A0A0A8UMU9_Legionella_hackeliae
A0A0F7EWT2_Hematodinium_sp.
L7UGY6_Myxococcus_stipitatus
F8CI86_Myxococcus_fulvus
K9T8P9_Pleurocapsa_sp.
E1VID6_gamma_proteobacterium
A0A0F7U6G8_Neospora_caninum
F0V7J0_Neospora_caninum
0,12
0,99
0,97
0,31
0,98
0,62
0,92
0,09
0,88
0,9
0,99
0,78
0,5
0,97
0,92
0,07
0,09
0,96
0,33
0,37
0.7

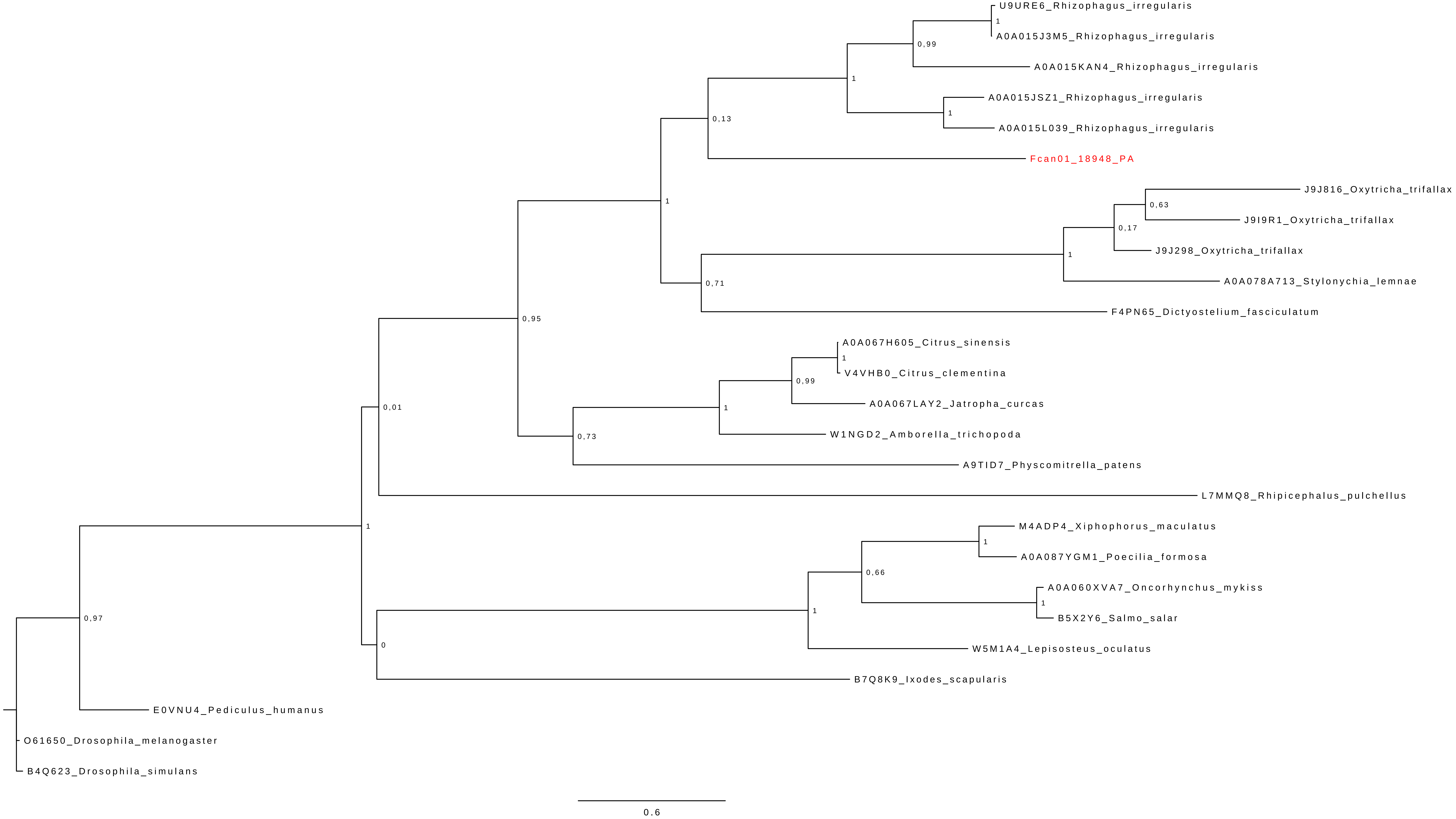
U9URE6_Rhizophagus_irregularis
1
A0A015J3M5_Rhizophagus_irregularis
0,99
A0A015KAN4_Rhizophagus_irregularis
1
A0A015JSZ1_Rhizophagus_irregularis
1
A0A015L039_Rhizophagus_irregularis
0,13
Fcan01_18948_PA
J9J816_Oxytricha_trifallax
0,63
J9I9R1_Oxytricha_trifallax
0,17
J9J298_Oxytricha_trifallax
1
1
A0A078A713_Stylonychia_lemnae
0,71
F4PN65_Dictyostelium_fasciculatum
0,95
A0A067H605_Citrus_sinensis
1
V4VHB0_Citrus_clementina
0,99
A0A067LAY2_Jatropha_curcas
1
0,01
W1NGD2_Amborella_trichopoda
0,73
A9TID7_Physcomitrella_patens
L7MMQ8_Rhipicephalus_pulchellus
M4ADP4_Xiphophorus_maculatus
1
1
A0A087YGM1_Poecilia_formosa
0,66
A0A060XVA7_Oncorhynchus_mykiss
1
1
0,97
B5X2Y6_Salmo_salar
0
W5M1A4_Lepisosteus_oculatus
B7Q8K9_Ixodes_scapularis
E0VNU4_Pediculus_humanus
O61650_Drosophila_melanogaster
B4Q623_Drosophila_simulans
0.6

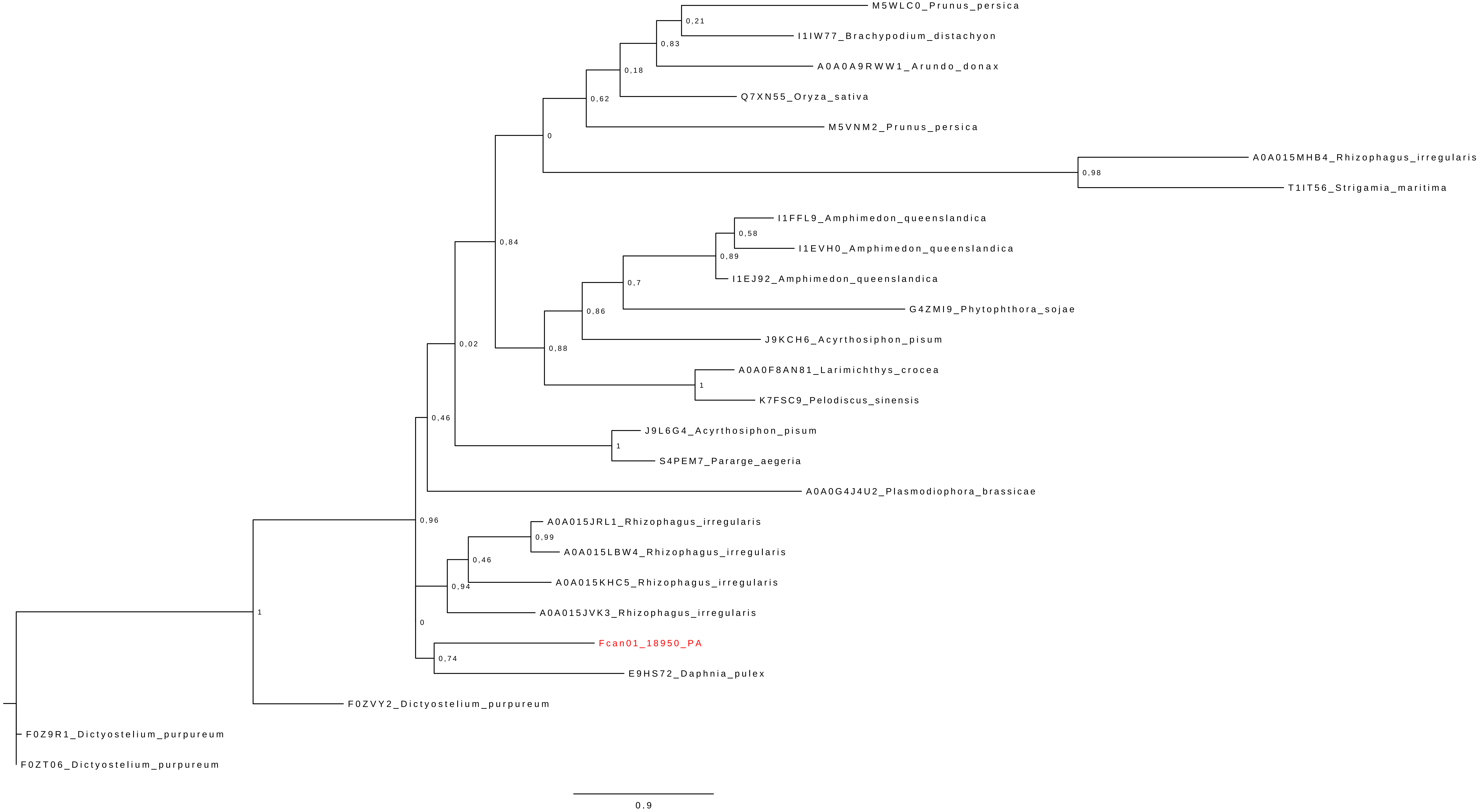

M5WLC0_Prunus_persica
I1IW77_Brachypodium_distachyon
A0A0A9RWW1_Arundo_donax
Q7XN55_Oryza_sativa
M5VNM2_Prunus_persica
A0A015MHB4_Rhizophagus_irregularis
T1IT56_Strigamia_maritima
I1FFL9_Amphimedon_queenslandica
I1EVH0_Amphimedon_queenslandica
I1EJ92_Amphimedon_queenslandica
G4ZMI9_Phytophthora_sojae
J9KCH6_Acyrthosiphon_pisum
A0A0F8AN81_Larimichthys_crocea
K7FSC9_Pelodiscus_sinensis
J9L6G4_Acyrthosiphon_pisum
S4PEM7_Pararge_aegeria
A0A0G4J4U2_Plasmodiophora_brassicae
A0A015JRL1_Rhizophagus_irregularis
A0A015LBW4_Rhizophagus_irregularis
A0A015KHC5_Rhizophagus_irregularis
A0A015JVK3_Rhizophagus_irregularis
Fcan01_18950_PA
E9HS72_Daphnia_pulex
F0ZVY2_Dictyostelium_purpureum
F0Z9R1_Dictyostelium_purpureum
F0ZT06_Dictyostelium_purpureum
0,21
0,83
0,18
0,62
0
0,98
0,84
0,58
0,89
0,7
0,86
0,02
0,88
1
0,46
1
0,96
0,99
0,46
0,94
1
0
0,74
0.9

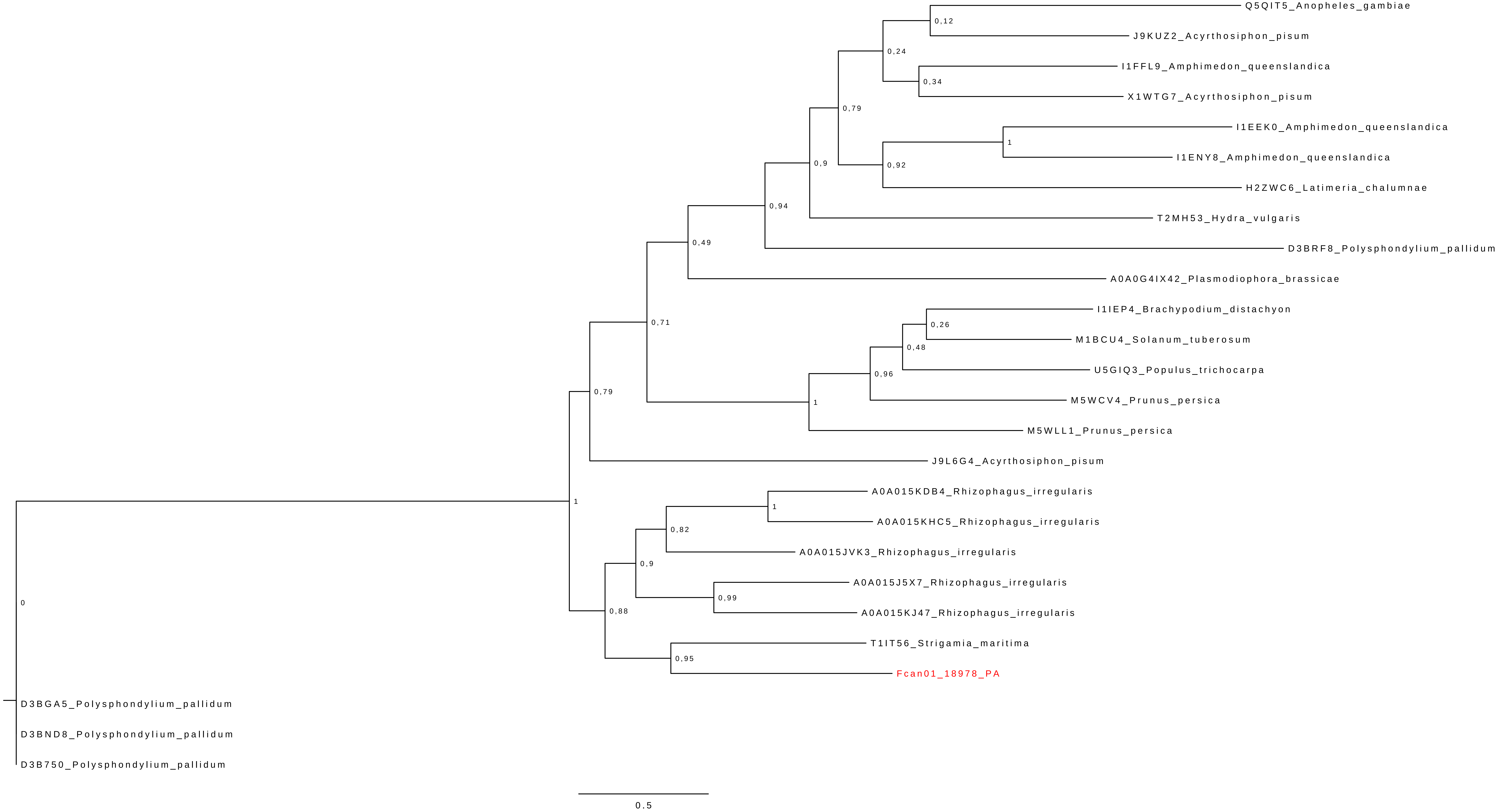
Q5QIT5_Anopheles_gambiae
J9KUZ2_Acyrthosiphon_pisum
I1FFL9_Amphimedon_queenslandica
X1WTG7_Acyrthosiphon_pisum
I1EEK0_Amphimedon_queenslandica
I1ENY8_Amphimedon_queenslandica
H2ZWC6_Latimeria_chalumnae
T2MH53_Hydra_vulgaris
D3BRF8_Polysphondylium_pallidum
A0A0G4IX42_Plasmodiophora_brassicae
I1IEP4_Brachypodium_distachyon
M1BCU4_Solanum_tuberosum
U5GIQ3_Populus_trichocarpa
M5WCV4_Prunus_persica
M5WLL1_Prunus_persica
J9L6G4_Acyrthosiphon_pisum
A0A015KDB4_Rhizophagus_irregularis
A0A015KHC5_Rhizophagus_irregularis
A0A015JVK3_Rhizophagus_irregularis
A0A015J5X7_Rhizophagus_irregularis
A0A015KJ47_Rhizophagus_irregularis
T1IT56_Strigamia_maritima
Fcan01_18978_PA
D3BGA5_Polysphondylium_pallidum
D3BND8_Polysphondylium_pallidum
D3B750_Polysphondylium_pallidum
0,12
0,24
0,34
0,79
1
0,92
0,9
0,94
0,49
0,26
0,48
0,96
1
0,71
0,79
1
1
0,82
0,9
0,99
0,88
0,95
0
0.5

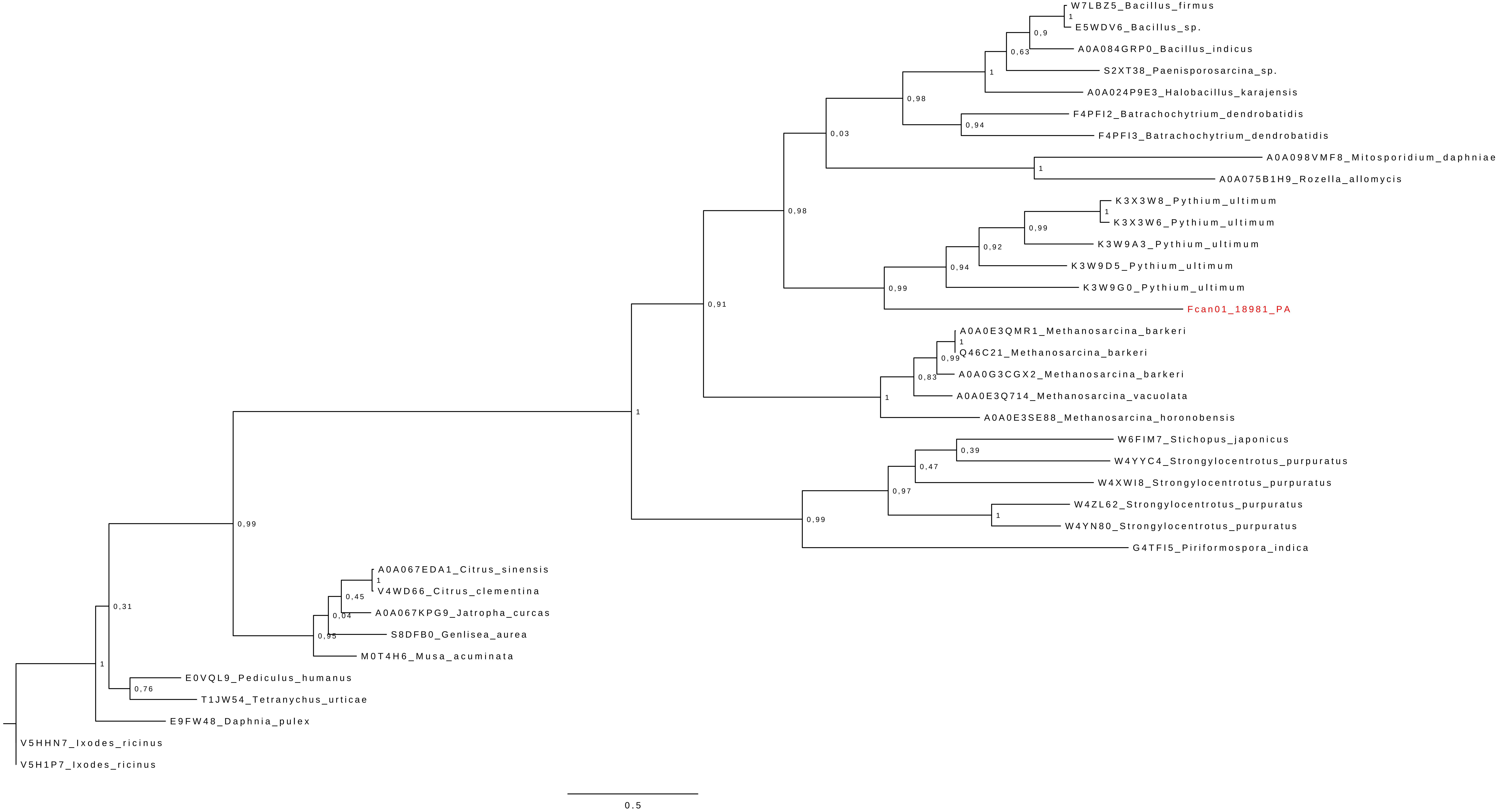
W7LBZ5_Bacillus_firmus
E5WDV6_Bacillus_sp.
A0A084GRP0_Bacillus_indicus
S2XT38_Paenisporosarcina_sp.
A0A024P9E3_Halobacillus_karajensis
F4PFI2_Batrachochytrium_dendrobatidis
F4PFI3_Batrachochytrium_dendrobatidis
A0A098VMF8_Mitosporidium_daphniae
A0A075B1H9_Rozella_allomycis
K3X3W8_Pythium_ultimum
K3X3W6_Pythium_ultimum
K3W9A3_Pythium_ultimum
K3W9D5_Pythium_ultimum
K3W9G0_Pythium_ultimum
Fcan01_18981_PA
A0A0E3QMR1_Methanosarcina_barkeri
Q46C21_Methanosarcina_barkeri
A0A0G3CGX2_Methanosarcina_barkeri
A0A0E3Q714_Methanosarcina_vacuolata
A0A0E3SE88_Methanosarcina_horonobensis
W6FIM7_Stichopus_japonicus
W4YYC4_Strongylocentrotus_purpuratus
W4XWI8_Strongylocentrotus_purpuratus
W4ZL62_Strongylocentrotus_purpuratus
W4YN80_Strongylocentrotus_purpuratus
G4TFI5_Piriformospora_indica
A0A067EDA1_Citrus_sinensis
V4WD66_Citrus_clementina
A0A067KPG9_Jatropha_curcas
S8DFB0_Genlisea_aurea
M0T4H6_Musa_acuminata
E0VQL9_Pediculus_humanus
T1JW54_Tetranychus_urticae
E9FW48_Daphnia_pulex
V5HHN7_Ixodes_ricinus
V5H1P7_Ixodes_ricinus
1
0,9
0,63
1
0,98
0,94
0,03
1
0,98
1
0,99
0,92
0,94
0,99
0,91
1
0,99
0,83
1
1
0,39
0,47
0,97
1
0,99
0,99
1
0,45
0,04
0,95
0,31
1
0,76
0.5

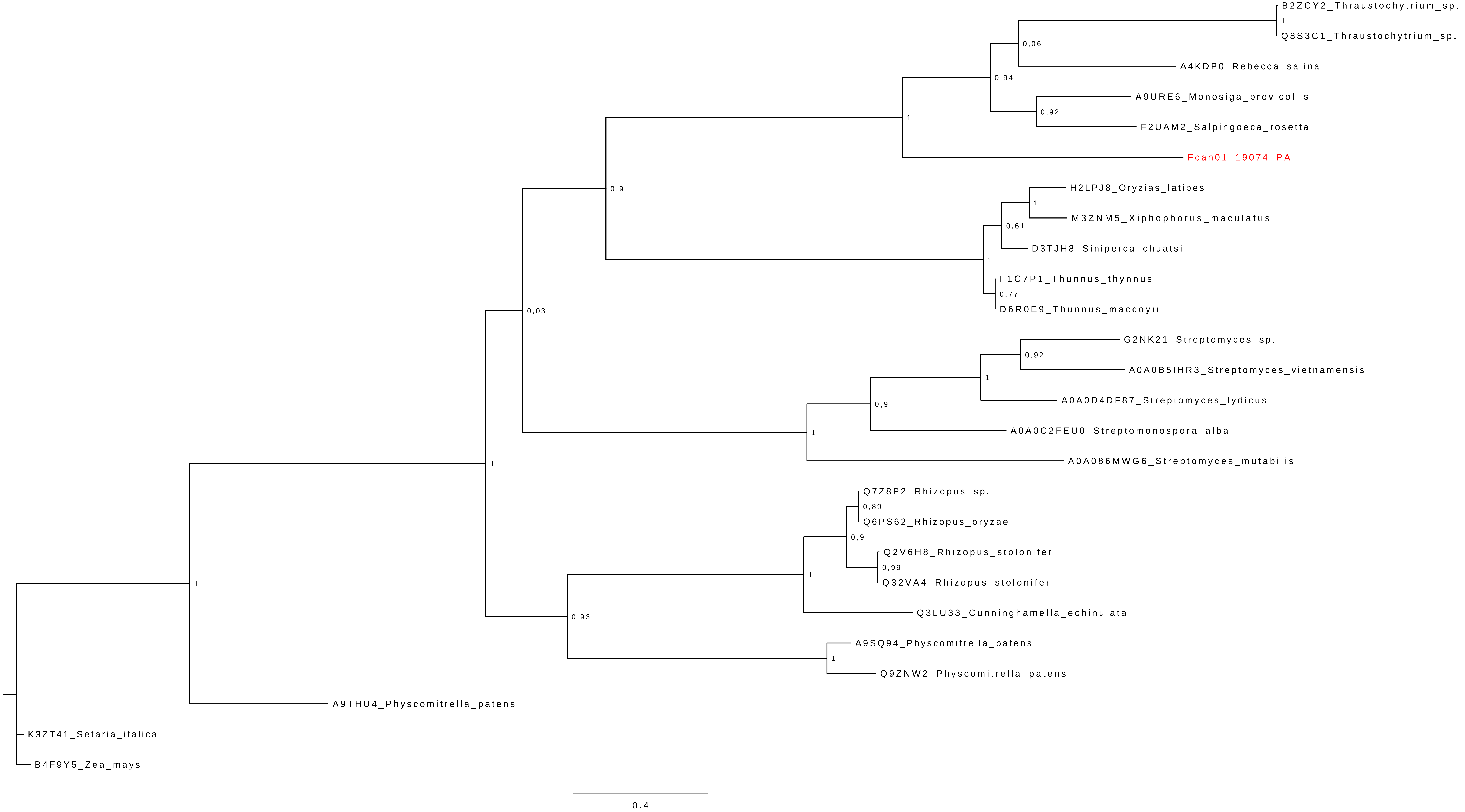
B2ZCY2_Thraustochytrium_sp.
Q8S3C1_Thraustochytrium_sp.
A4KDP0_Rebecca_salina
A9URE6_Monosiga_brevicollis
F2UAM2_Salpingoeca_rosetta
Fcan01_19074_PA
H2LPJ8_Oryzias_latipes
M3ZNM5_Xiphophorus_maculatus
D3TJH8_Siniperca_chuatsi
F1C7P1_Thunnus_thynnus
D6R0E9_Thunnus_maccoyii
G2NK21_Streptomyces_sp.
A0A0B5IHR3_Streptomyces_vietnamensis
A0A0D4DF87_Streptomyces_lydicus
A0A0C2FEU0_Streptomonospora_alba
A0A086MWG6_Streptomyces_mutabilis
Q7Z8P2_Rhizopus_sp.
Q6PS62_Rhizopus_oryzae
Q2V6H8_Rhizopus_stolonifer
Q32VA4_Rhizopus_stolonifer
Q3LU33_Cunninghamella_echinulata
A9SQ94_Physcomitrella_patens
Q9ZNW2_Physcomitrella_patens
A9THU4_Physcomitrella_patens
K3ZT41_Setaria_italica
B4F9Y5_Zea_mays
1
0,06
0,94
0,92
1
0,9
1
0,61
1
0,77
0,03
0,92
1
0,9
1
1
0,89
0,9
0,99
1
0,93
1
1
0.4

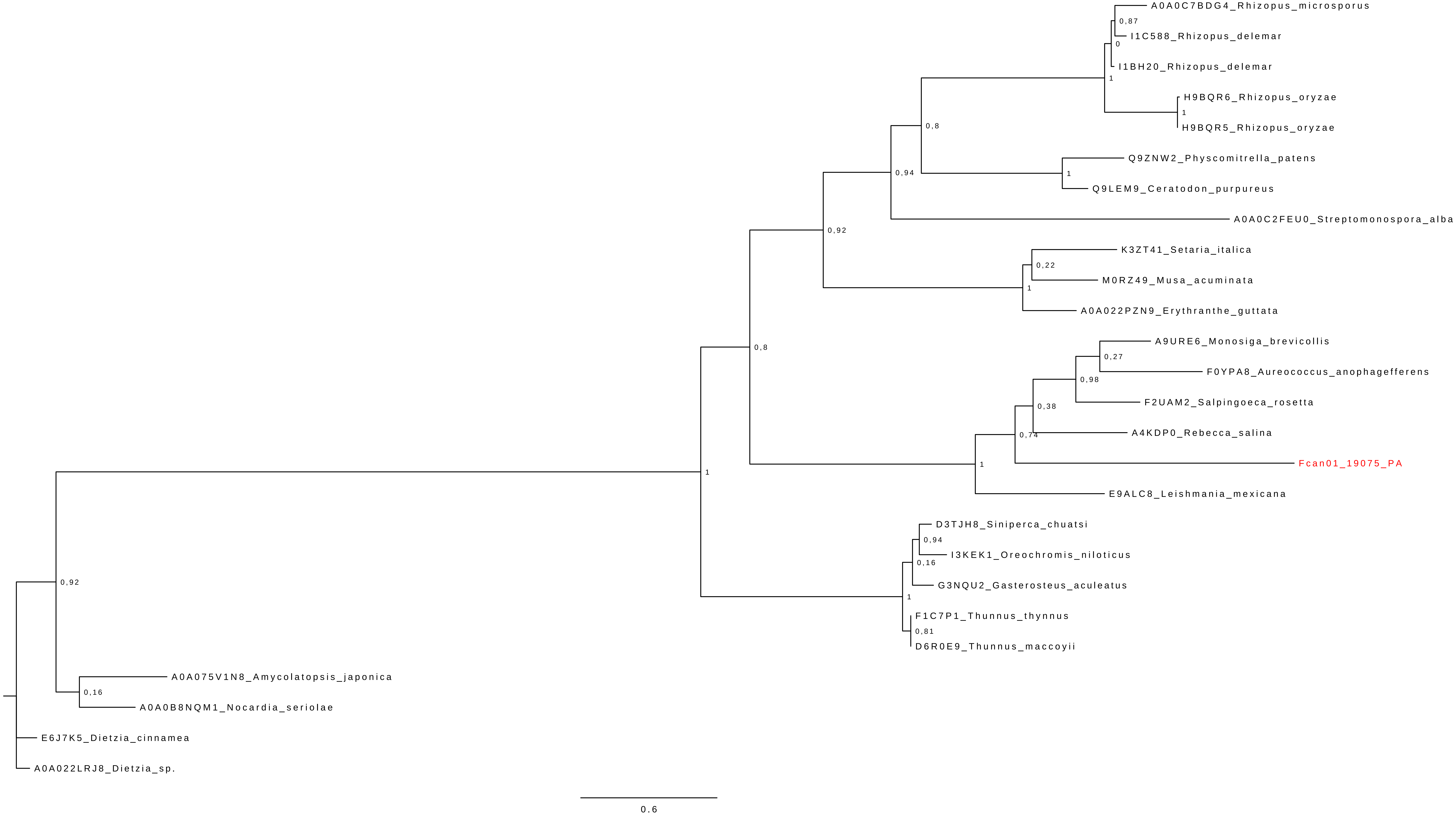
A0A0C7BDG4_Rhizopus_microsporus
0,87
I1C588_Rhizopus_delemar
0
I1BH20_Rhizopus_delemar
1
H9BQR6_Rhizopus_oryzae
1
H9BQR5_Rhizopus_oryzae
0,8
Q9ZNW2_Physcomitrella_patens
0,94
1
Q9LEM9_Ceratodon_purpureus
A0A0C2FEU0_Streptomonospora_alba
0,92
K3ZT41_Setaria_italica
0,22
M0RZ49_Musa_acuminata
1
A0A022PZN9_Erythranthe_guttata
A9URE6_Monosiga_brevicollis
0,8
0,27
F0YPA8_Aureococcus_anophagefferens
0,98
F2UAM2_Salpingoeca_rosetta
0,38
A4KDP0_Rebecca_salina
0,74
1
Fcan01_19075_PA
1
E9ALC8_Leishmania_mexicana
D3TJH8_Siniperca_chuatsi
0,94
I3KEK1_Oreochromis_niloticus
0,16
0,92
G3NQU2_Gasterosteus_aculeatus
1
F1C7P1_Thunnus_thynnus
0,81
D6R0E9_Thunnus_maccoyii
A0A075V1N8_Amycolatopsis_japonica
0,16
A0A0B8NQM1_Nocardia_seriolae
E6J7K5_Dietzia_cinnamea
A0A022LRJ8_Dietzia_sp.
0.6

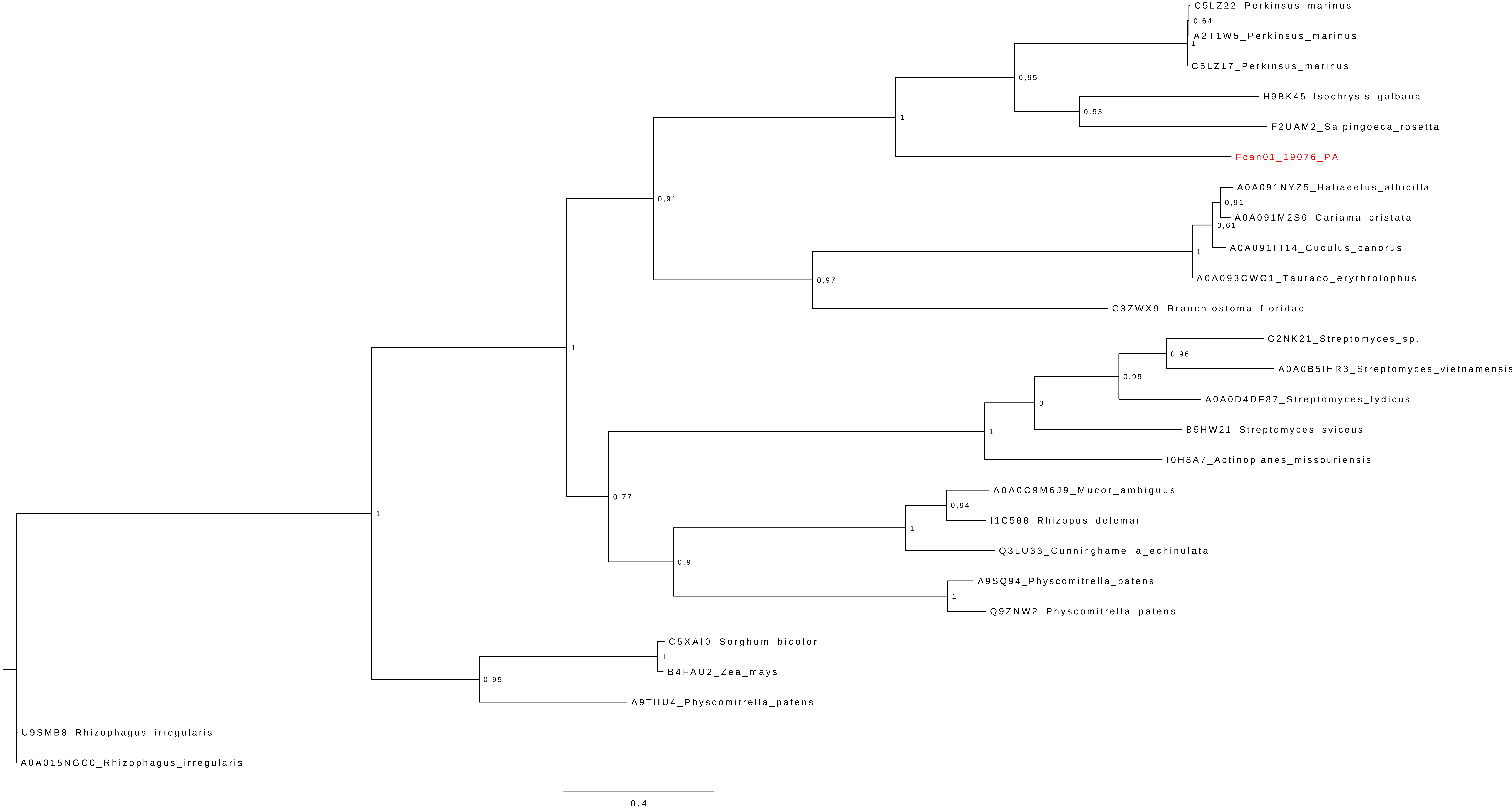

C5LZ22_Perkinsus_marinus
0,64
A2T1W5_Perkinsus_marinus
1
C5LZ17_Perkinsus_marinus
0,95
H9BK45_Isochrysis_galbana
0,93
F2UAM2_Salpingoeca_rosetta
1
Fcan01_19076_PA
0,91
A0A091NYZ5_Haliaeetus_albicilla
0,91
A0A091M2S6_Cariama_cristata
0,61
A0A091FI14_Cuculus_canorus
1
A0A093CWC1_Tauraco_erythrolophus
0,97
C3ZWX9_Branchiostoma_floridae
1
G2NK21_Streptomyces_sp.
0,96
A0A0B5IHR3_Streptomyces_vietnamensis
0,99
A0A0D4DF87_Streptomyces_lydicus
0
B5HW21_Streptomyces_sviceus
1
I0H8A7_Actinoplanes_missouriensis
0,77
A0A0C9M6J9_Mucor_ambiguus
0,94
I1C588_Rhizopus_delemar
1
Q3LU33_Cunninghamella_echinulata
0,9
A9SQ94_Physcomitrella_patens
1
Q9ZNW2_Physcomitrella_patens
1
C5XAI0_Sorghum_bicolor
1
B4FAU2_Zea_mays
0,95
A9THU4_Physcomitrella_patens
U9SMB8_Rhizophagus_irregularis
A0A015NGC0_Rhizophagus_irregularis
0.4

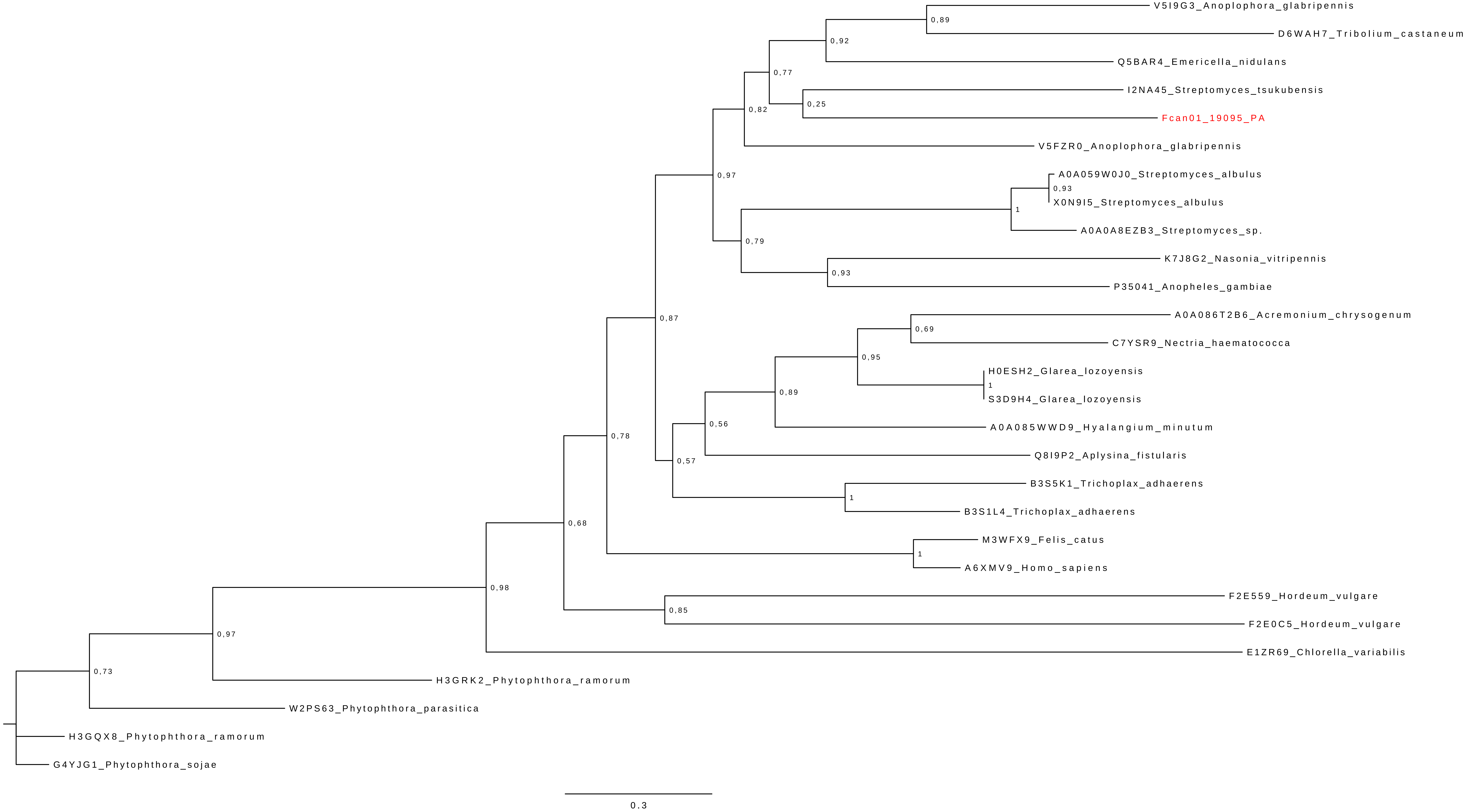
V5I9G3_Anoplophora_glabripennis
0,89
D6WAH7_Tribolium_castaneum
0,92
Q5BAR4_Emericella_nidulans
0,77
I2NA45_Streptomyces_tsukubensis
0,25
Fcan01_19095_PA
0,82
V5FZR0_Anoplophora_glabripennis
0,97
A0A059W0J0_Streptomyces_albulus
0,93
X0N9I5_Streptomyces_albulus
1
A0A0A8EZB3_Streptomyces_sp.
0,79
K7J8G2_Nasonia_vitripennis
0,93
P35041_Anopheles_gambiae
0,87
A0A086T2B6_Acremonium_chrysogenum
0,69
C7YSR9_Nectria_haematococca
0,95
H0ESH2_Glarea_lozoyensis
1
S3D9H4_Glarea_lozoyensis
0,89
A0A085WWD9_Hyalangium_minutum
0,56
Q8I9P2_Aplysina_fistularis
0,78
0,57
B3S5K1_Trichoplax_adhaerens
1
B3S1L4_Trichoplax_adhaerens
0,68
M3WFX9_Felis_catus
1
A6XMV9_Homo_sapiens
0,98
F2E559_Hordeum_vulgare
0,85
F2E0C5_Hordeum_vulgare
E1ZR69_Chlorella_variabilis
0,97
H3GRK2_Phytophthora_ramorum
0,73
W2PS63_Phytophthora_parasitica
H3GQX8_Phytophthora_ramorum
G4YJG1_Phytophthora_sojae
0.3

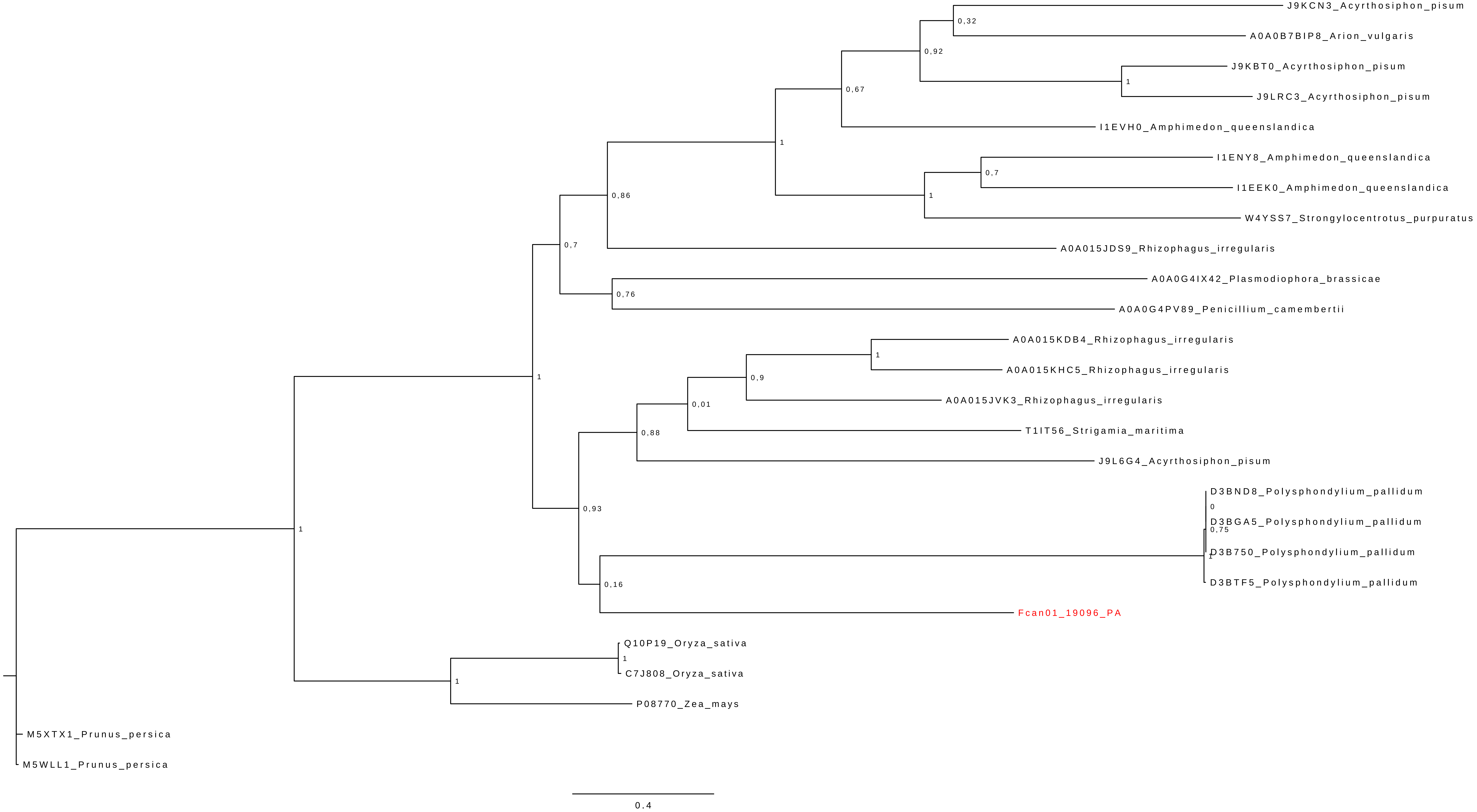

J9KCN3_Acyrthosiphon_pisum
A0A0B7BIP8_Arion_vulgaris
J9KBT0_Acyrthosiphon_pisum
J9LRC3_Acyrthosiphon_pisum
I1EVH0_Amphimedon_queenslandica
I1ENY8_Amphimedon_queenslandica
I1EEK0_Amphimedon_queenslandica
W4YSS7_Strongylocentrotus_purpuratus
A0A015JDS9_Rhizophagus_irregularis
A0A0G4IX42_Plasmodiophora_brassicae
A0A0G4PV89_Penicillium_camembertii
A0A015KDB4_Rhizophagus_irregularis
A0A015KHC5_Rhizophagus_irregularis
A0A015JVK3_Rhizophagus_irregularis
T1IT56_Strigamia_maritima
J9L6G4_Acyrthosiphon_pisum
D3BND8_Polysphondylium_pallidum
D3BGA5_Polysphondylium_pallidum
D3B750_Polysphondylium_pallidum
D3BTF5_Polysphondylium_pallidum
Fcan01_19096_PA
Q10P19_Oryza_sativa
C7J808_Oryza_sativa
P08770_Zea_mays
M5XTX1_Prunus_persica
M5WLL1_Prunus_persica
0.4

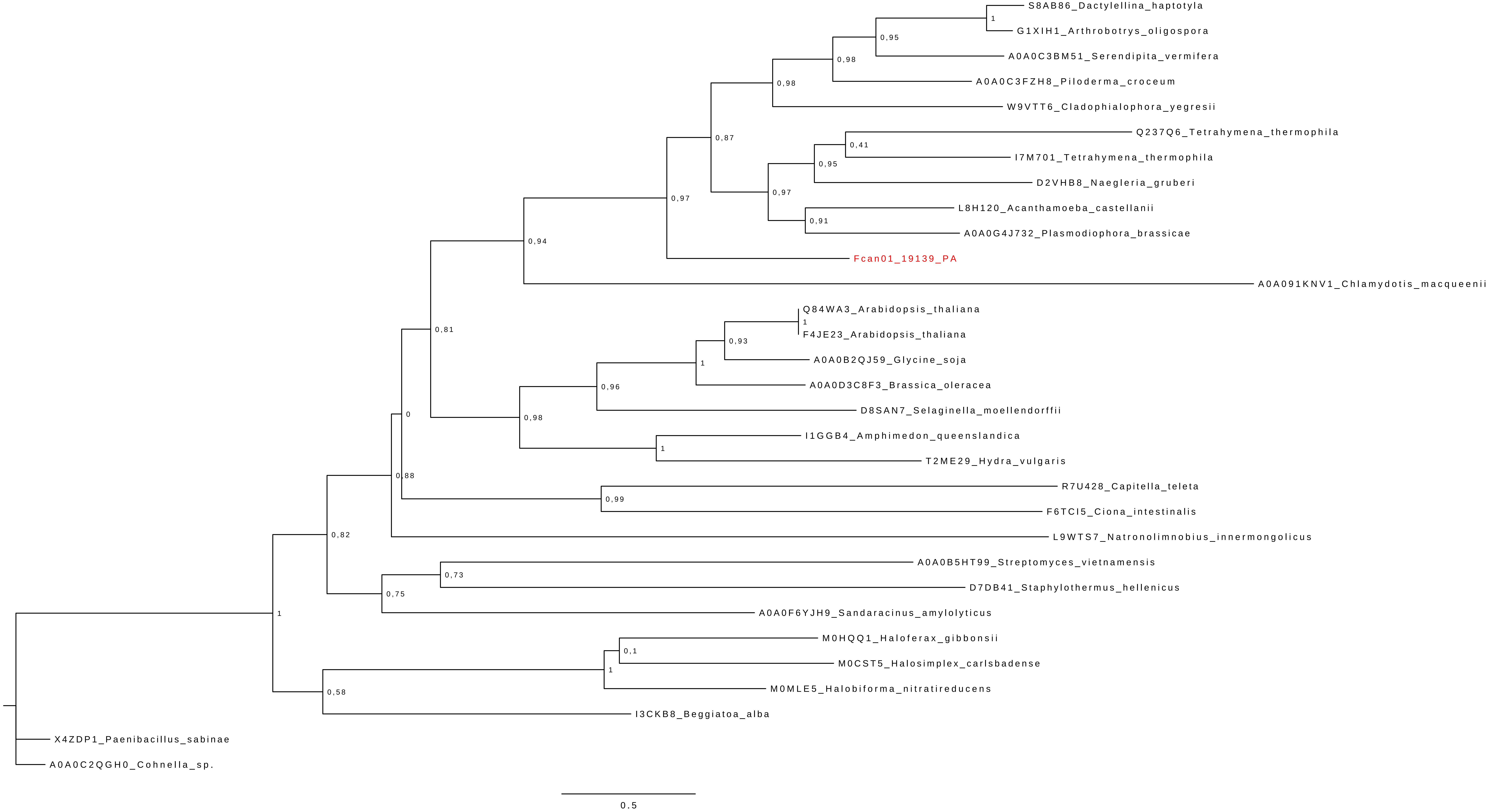
S8AB86_Dactylellina_haptotyla
G1XIH1_Arthrobotrys_oligospora
A0A0C3BM51_Serendipita_vermifera
A0A0C3FZH8_Piloderma_croceum
W9VTT6_Cladophialophora_yegresii
Q237Q6_Tetrahymena_thermophila
I7M701_Tetrahymena_thermophila
D2VHB8_Naegleria_gruberi
L8H120_Acanthamoeba_castellanii
A0A0G4J732_Plasmodiophora_brassicae
Fcan01_19139_PA
A0A091KNV1_Chlamydotis_macqueenii
Q84WA3_Arabidopsis_thaliana
F4JE23_Arabidopsis_thaliana
A0A0B2QJ59_Glycine_soja
A0A0D3C8F3_Brassica_oleracea
D8SAN7_Selaginella_moellendorffii
I1GGB4_Amphimedon_queenslandica
T2ME29_Hydra_vulgaris
R7U428_Capitella_teleta
F6TCI5_Ciona_intestinalis
L9WTS7_Natronolimnobius_innermongolicus
A0A0B5HT99_Streptomyces_vietnamensis
D7DB41_Staphylothermus_hellenicus
A0A0F6YJH9_Sandaracinus_amylolyticus
M0HQQ1_Haloferax_gibbonsii
M0CST5_Halosimplex_carlsbadense
M0MLE5_Halobiforma_nitratireducens
I3CKB8_Beggiatoa_alba
X4ZDP1_Paenibacillus_sabinae
A0A0C2QGH0_Cohnella_sp.
1
0,95
0,98
0,98
0,87
0,41
0,95
0,97
0,91
0,97
0,94
1
0,93
1
0,96
0,98
1
0,81
0
0,88
0,99
0,82
0,73
0,75
1
0,1
1
0,58
0.5

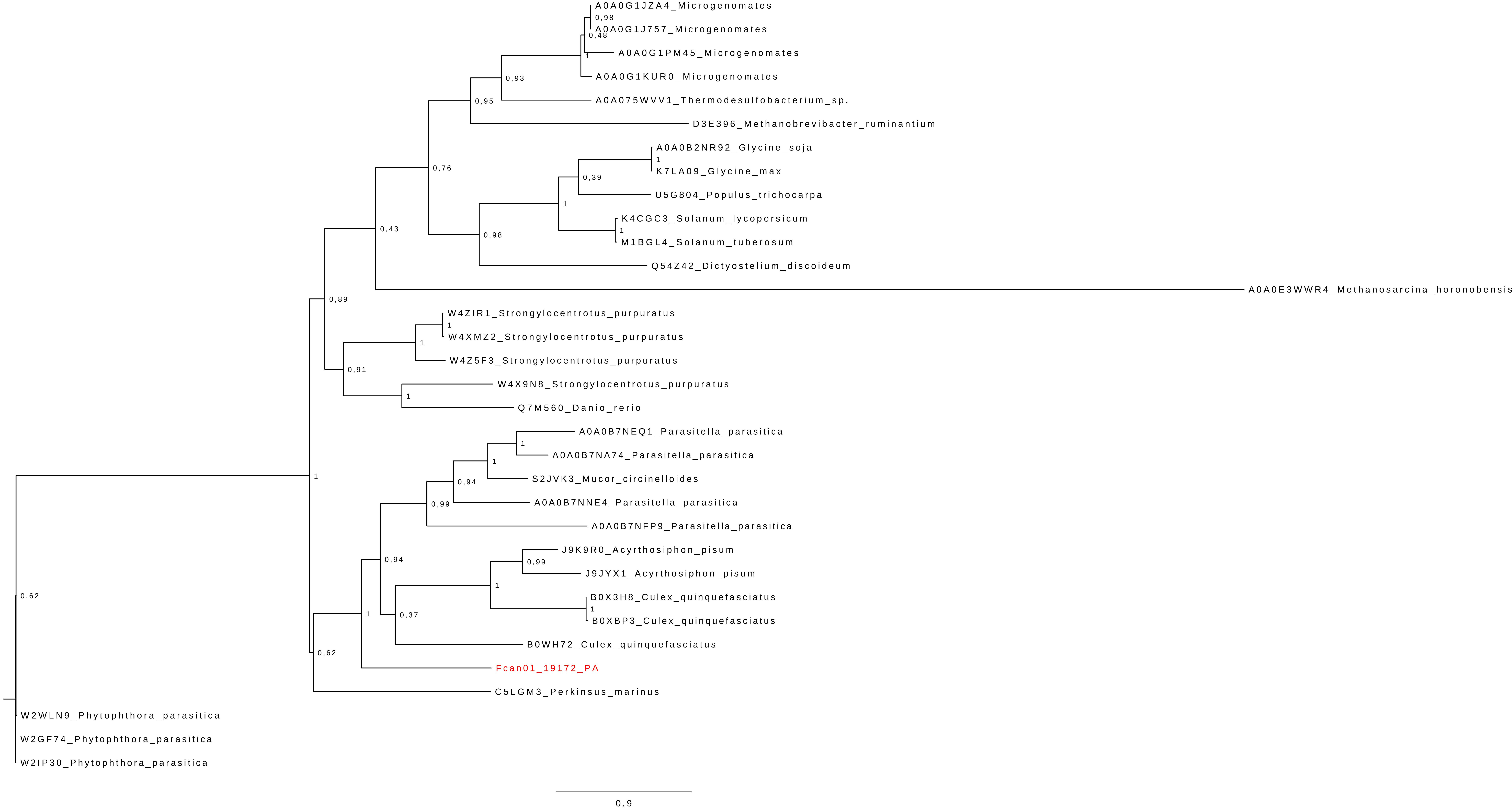
A0A0G1JZA4_Microgenomates
0,98
A0A0G1J757_Microgenomates
0,48
1
A0A0G1PM45_Microgenomates
A0A0G1KUR0_Microgenomates
0,93
A0A075WVV1_Thermodesulfobacterium_sp.
0,95
D3E396_Methanobrevibacter_ruminantium
A0A0B2NR92_Glycine_soja
1
K7LA09_Glycine_max
0,39
U5G804_Populus_trichocarpa
1
K4CGC3_Solanum_lycopersicum
1
M1BGL4_Solanum_tuberosum
0,76
0,98
Q54Z42_Dictyostelium_discoideum
0,43
A0A0E3WWR4_Methanosarcina_horonobensis
0,89
W4ZIR1_Strongylocentrotus_purpuratus
1
W4XMZ2_Strongylocentrotus_purpuratus
1
W4Z5F3_Strongylocentrotus_purpuratus
0,91
W4X9N8_Strongylocentrotus_purpuratus
1
Q7M560_Danio_rerio
A0A0B7NEQ1_Parasitella_parasitica
1
A0A0B7NA74_Parasitella_parasitica
1
S2JVK3_Mucor_circinelloides
0,94
A0A0B7NNE4_Parasitella_parasitica
0,99
A0A0B7NFP9_Parasitella_parasitica
J9K9R0_Acyrthosiphon_pisum
0,99
J9JYX1_Acyrthosiphon_pisum
0,94
1
B0X3H8_Culex_quinquefasciatus
1
B0XBP3_Culex_quinquefasciatus
0,37
B0WH72_Culex_quinquefasciatus
1
0,62
Fcan01_19172_PA
C5LGM3_Perkinsus_marinus
0,62
W2WLN9_Phytophthora_parasitica
W2GF74_Phytophthora_parasitica
W2IP30_Phytophthora_parasitica
0.9

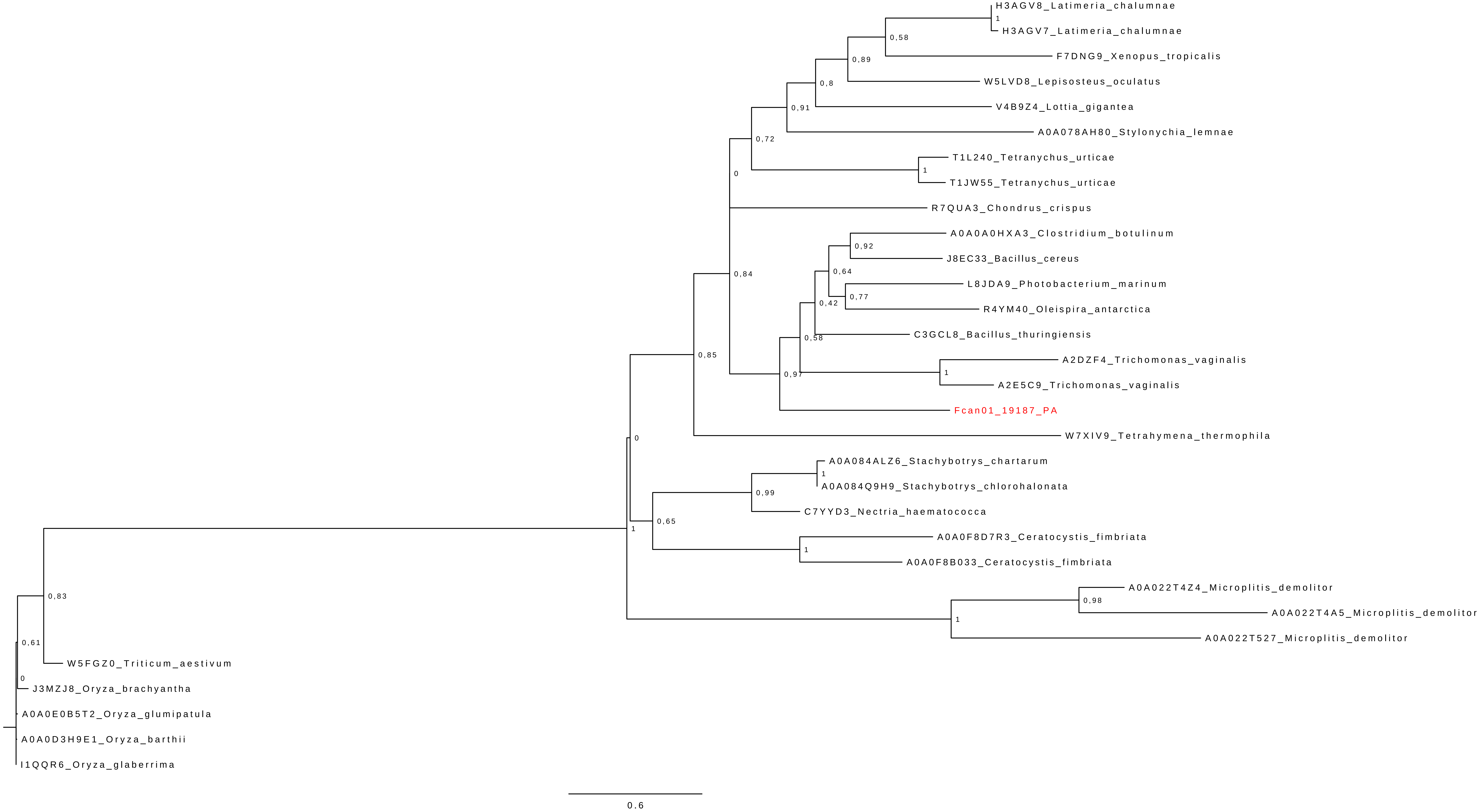
H3AGV8_Latimeria_chalumnae
H3AGV7_Latimeria_chalumnae
F7DNG9_Xenopus_tropicalis
W5LVD8_Lepisosteus_oculatus
V4B9Z4_Lottia_gigantea
A0A078AH80_Stylonychia_lemnae
T1L240_Tetranychus_urticae
T1JW55_Tetranychus_urticae
R7QUA3_Chondrus_crispus
A0A0A0HXA3_Clostridium_botulinum
J8EC33_Bacillus_cereus
L8JDA9_Photobacterium_marinum
R4YM40_Oleispira_antarctica
C3GCL8_Bacillus_thuringiensis
A2DZF4_Trichomonas_vaginalis
A2E5C9_Trichomonas_vaginalis
Fcan01_19187_PA
W7XIV9_Tetrahymena_thermophila
A0A084ALZ6_Stachybotrys_chartarum
A0A084Q9H9_Stachybotrys_chlorohalonata
C7YYD3_Nectria_haematococca
A0A0F8D7R3_Ceratocystis_fimbriata
A0A0F8B033_Ceratocystis_fimbriata
A0A022T4Z4_Microplitis_demolitor
A0A022T4A5_Microplitis_demolitor
A0A022T527_Microplitis_demolitor
W5FGZ0_Triticum_aestivum
J3MZJ8_Oryza_brachyantha
A0A0E0B5T2_Oryza_glumipatula
A0A0D3H9E1_Oryza_barthii
I1QQR6_Oryza_glaberrima
1
0,58
0,89
0,8
0,91
0,72
1
0
0,92
0,64
0,77
0,42
0,58
1
0,97
0,84
0,85
0
1
0,99
0,65
1
1
0,98
1
0,83
0,61
0
0.6

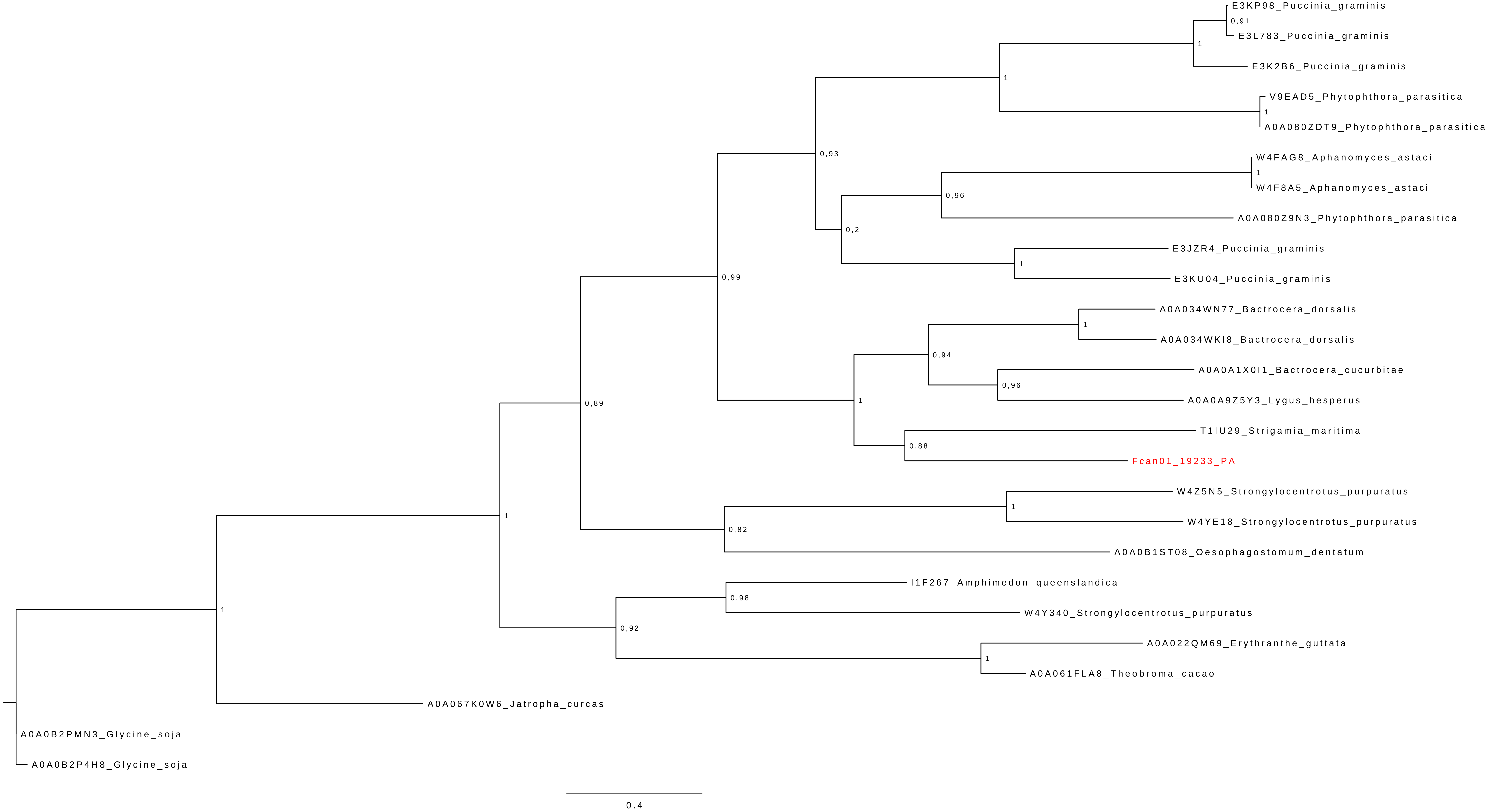
E3KP98_Puccinia_graminis
0,91
E3L783_Puccinia_graminis
1
E3K2B6_Puccinia_graminis
1
V9EAD5_Phytophthora_parasitica
1
A0A080ZDT9_Phytophthora_parasitica
0,93
W4FAG8_Aphanomyces_astaci
1
W4F8A5_Aphanomyces_astaci
0,96
A0A080Z9N3_Phytophthora_parasitica
0,2
E3JZR4_Puccinia_graminis
1
E3KU04_Puccinia_graminis
0,99
A0A034WN77_Bactrocera_dorsalis
1
A0A034WKI8_Bactrocera_dorsalis
0,94
A0A0A1X0I1_Bactrocera_cucurbitae
0,96
A0A0A9Z5Y3_Lygus_hesperus
1
T1IU29_Strigamia_maritima
0,88
Fcan01_19233_PA
0,89
W4Z5N5_Strongylocentrotus_purpuratus
1
W4YE18_Strongylocentrotus_purpuratus
0,82
A0A0B1ST08_Oesophagostomum_dentatum
1
I1F267_Amphimedon_queenslandica
0,98
W4Y340_Strongylocentrotus_purpuratus
0,92
A0A022QM69_Erythranthe_guttata
1
A0A061FLA8_Theobroma_cacao
1
A0A067K0W6_Jatropha_curcas
A0A0B2PMN3_Glycine_soja
A0A0B2P4H8_Glycine_soja
0.4

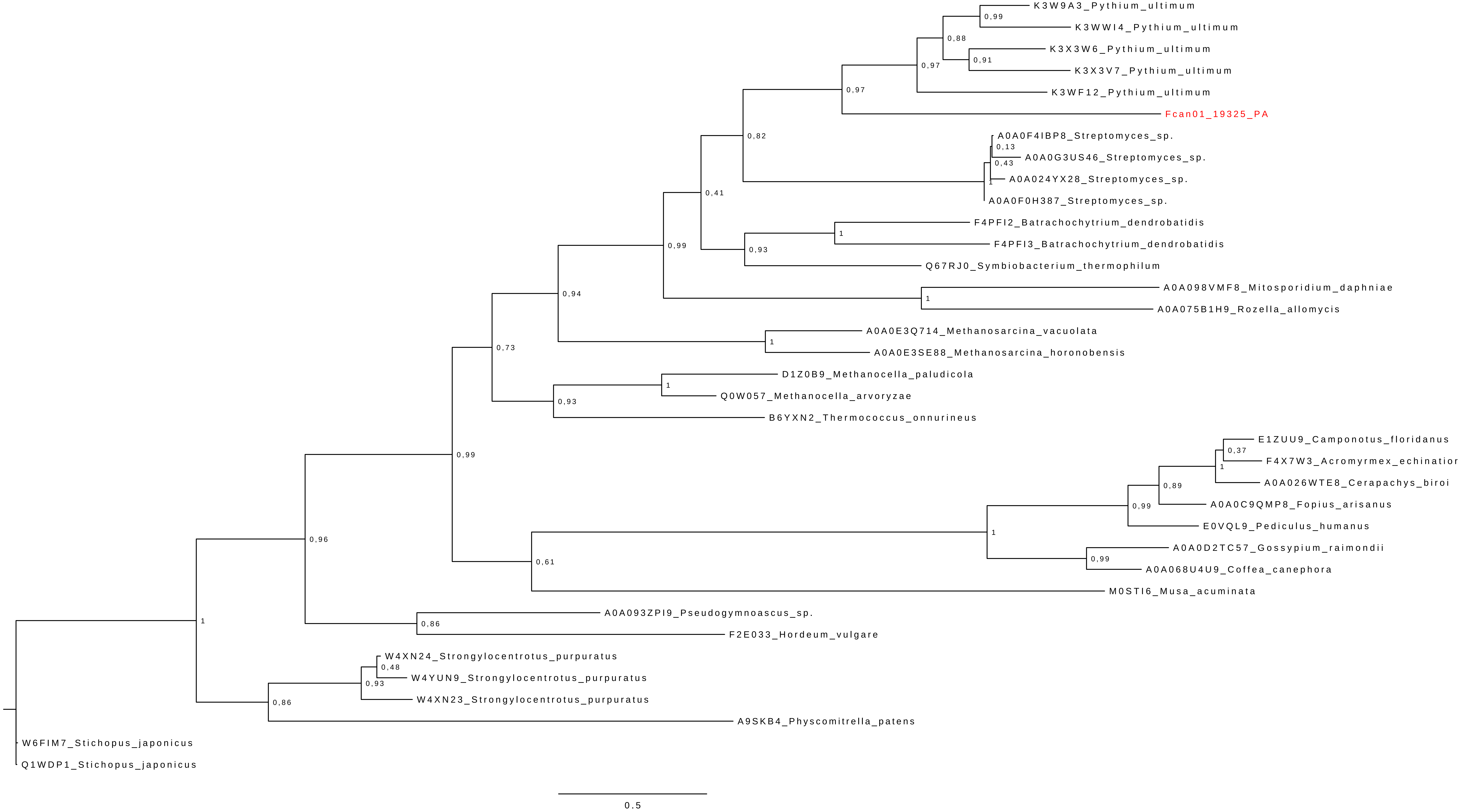
K3W9A3_Pythium_ultimum
K3WWI4_Pythium_ultimum
K3X3W6_Pythium_ultimum
K3X3V7_Pythium_ultimum
K3WF12_Pythium_ultimum
Fcan01_19325_PA
A0A0F4IBP8_Streptomyces_sp.
A0A0G3US46_Streptomyces_sp.
A0A024YX28_Streptomyces_sp.
A0A0F0H387_Streptomyces_sp.
F4PFI2_Batrachochytrium_dendrobatidis
F4PFI3_Batrachochytrium_dendrobatidis
Q67RJ0_Symbiobacterium_thermophilum
A0A098VMF8_Mitosporidium_daphniae
A0A075B1H9_Rozella_allomycis
A0A0E3Q714_Methanosarcina_vacuolata
A0A0E3SE88_Methanosarcina_horonobensis
D1Z0B9_Methanocella_paludicola
Q0W057_Methanocella_arvoryzae
B6YXN2_Thermococcus_onnurineus
E1ZUU9_Camponotus_floridanus
F4X7W3_Acromyrmex_echinatior
A0A026WTE8_Cerapachys_biroi
A0A0C9QMP8_Fopius_arisanus
E0VQL9_Pediculus_humanus
A0A0D2TC57_Gossypium_raimondii
A0A068U4U9_Coffea_canephora
M0STI6_Musa_acuminata
A0A093ZPI9_Pseudogymnoascus_sp.
F2E033_Hordeum_vulgare
W4XN24_Strongylocentrotus_purpuratus
W4YUN9_Strongylocentrotus_purpuratus
W4XN23_Strongylocentrotus_purpuratus
A9SKB4_Physcomitrella_patens
W6FIM7_Stichopus_japonicus
Q1WDP1_Stichopus_japonicus
0.5

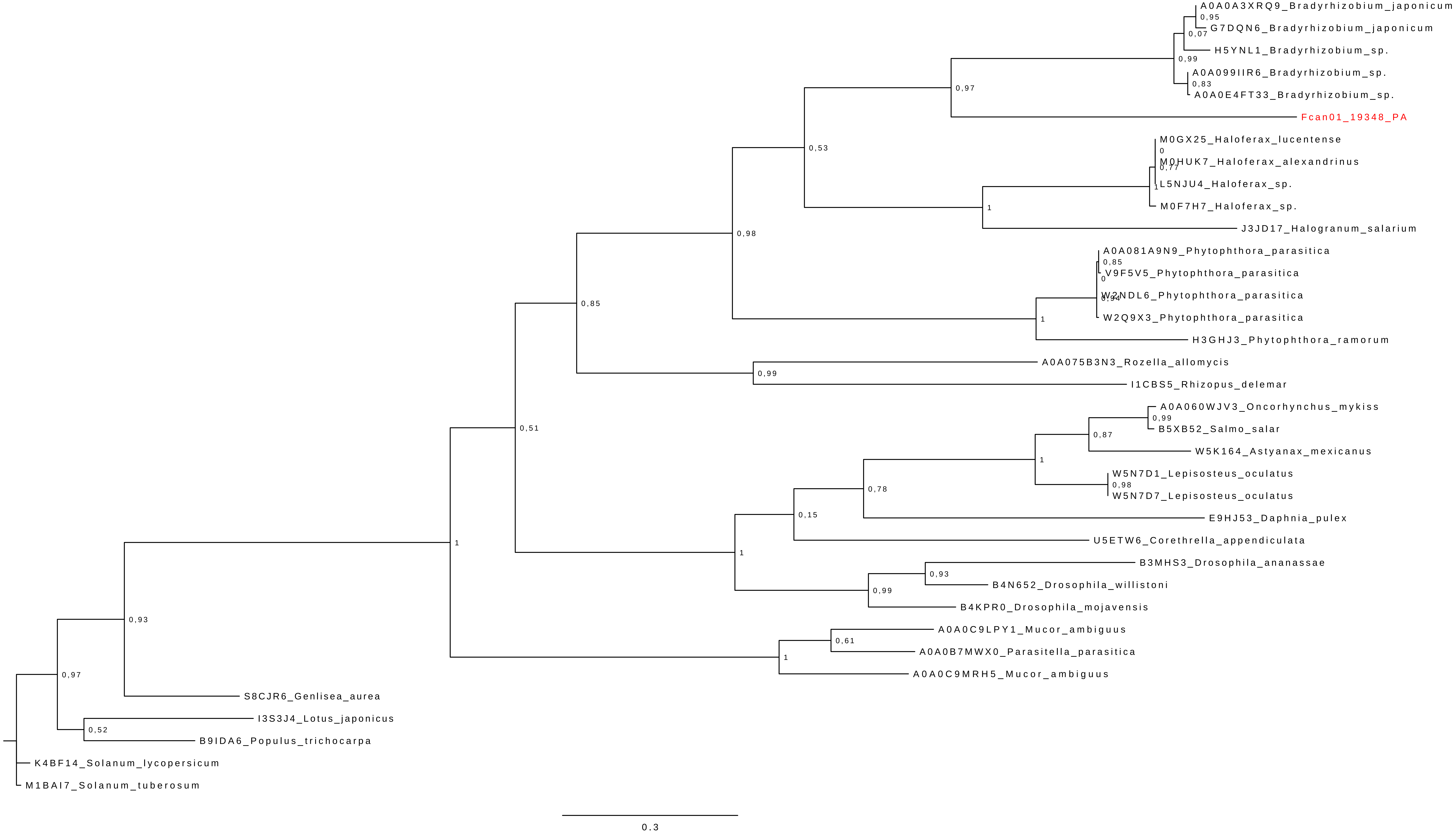
A0A0A3XRQ9_Bradyrhizobium_japonicum
0,95
G7DQN6_Bradyrhizobium_japonicum
0,07
H5YNL1_Bradyrhizobium_sp.
0,99
A0A099IIR6_Bradyrhizobium_sp.
0,83
A0A0E4FT33_Bradyrhizobium_sp.
0,97
Fcan01_19348_PA
0,53
M0GX25_Haloferax_lucentense
0
M0HUK7_Haloferax_alexandrinus
0,77
L5NJU4_Haloferax_sp.
1
M0F7H7_Haloferax_sp.
1
J3JD17_Halogranum_salarium
0,98
A0A081A9N9_Phytophthora_parasitica
0,85
V9F5V5_Phytophthora_parasitica
0
W2NDL6_Phytophthora_parasitica
0,94
W2Q9X3_Phytophthora_parasitica
1
H3GHJ3_Phytophthora_ramorum
0,85
A0A075B3N3_Rozella_allomycis
0,99
I1CBS5_Rhizopus_delemar
A0A060WJV3_Oncorhynchus_mykiss
0,99
B5XB52_Salmo_salar
0,87
W5K164_Astyanax_mexicanus
1
W5N7D1_Lepisosteus_oculatus
0,98
W5N7D7_Lepisosteus_oculatus
0,78
E9HJ53_Daphnia_pulex
0,15
U5ETW6_Corethrella_appendiculata
0,51
1
B3MHS3_Drosophila_ananassae
0,93
B4N652_Drosophila_willistoni
0,99
B4KPR0_Drosophila_mojavensis
A0A0C9LPY1_Mucor_ambiguus
0,61
A0A0B7MWX0_Parasitella_parasitica
1
A0A0C9MRH5_Mucor_ambiguus
1
0,93
S8CJR6_Genlisea_aurea
0,97
I3S3J4_Lotus_japonicus
0,52
B9IDA6_Populus_trichocarpa
K4BF14_Solanum_lycopersicum
M1BAI7_Solanum_tuberosum
0.3

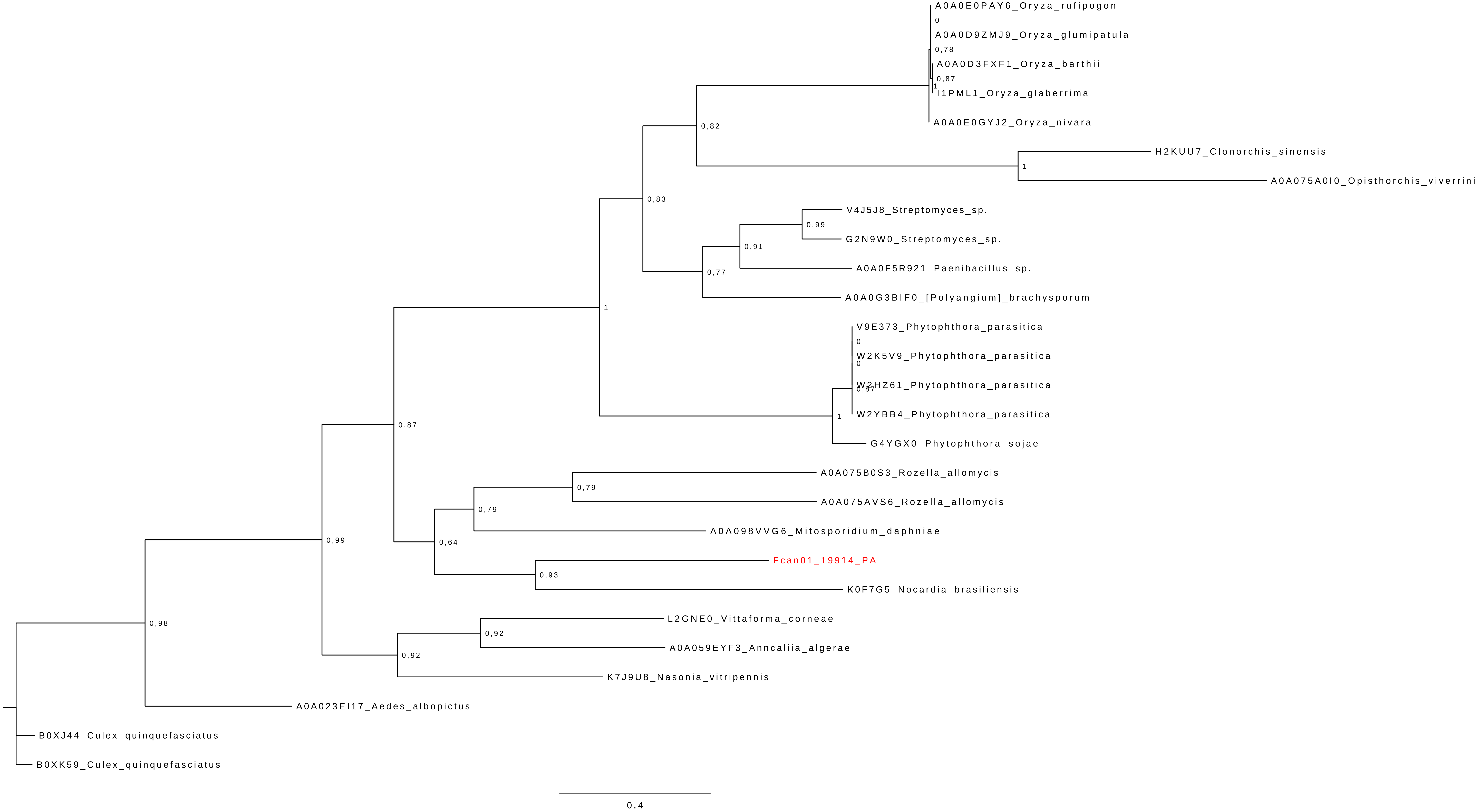
A0A0E0PAY6_Oryza_rufipogon
0
A0A0D9ZMJ9_Oryza_glumipatula
0,78
A0A0D3FXF1_Oryza_barthii
0,87
1
I1PML1_Oryza_glaberrima
A0A0E0GYJ2_Oryza_nivara
0,82
H2KUU7_Clonorchis_sinensis
1
A0A075A0I0_Opisthorchis_viverrini
0,83
V4J5J8_Streptomyces_sp.
0,99
G2N9W0_Streptomyces_sp.
0,91
A0A0F5R921_Paenibacillus_sp.
0,77
A0A0G3BIF0_[Polyangium]_brachysporum
1
V9E373_Phytophthora_parasitica
0
W2K5V9_Phytophthora_parasitica
0
W2HZ61_Phytophthora_parasitica
0,87
W2YBB4_Phytophthora_parasitica
1
G4YGX0_Phytophthora_sojae
0,87
A0A075B0S3_Rozella_allomycis
0,79
A0A075AVS6_Rozella_allomycis
0,79
A0A098VVG6_Mitosporidium_daphniae
0,64
Fcan01_19914_PA
0,93
K0F7G5_Nocardia_brasiliensis
0,99
L2GNE0_Vittaforma_corneae
0,92
A0A059EYF3_Anncaliia_algerae
0,92
K7J9U8_Nasonia_vitripennis
0,98
A0A023EI17_Aedes_albopictus
B0XJ44_Culex_quinquefasciatus
B0XK59_Culex_quinquefasciatus
0.4

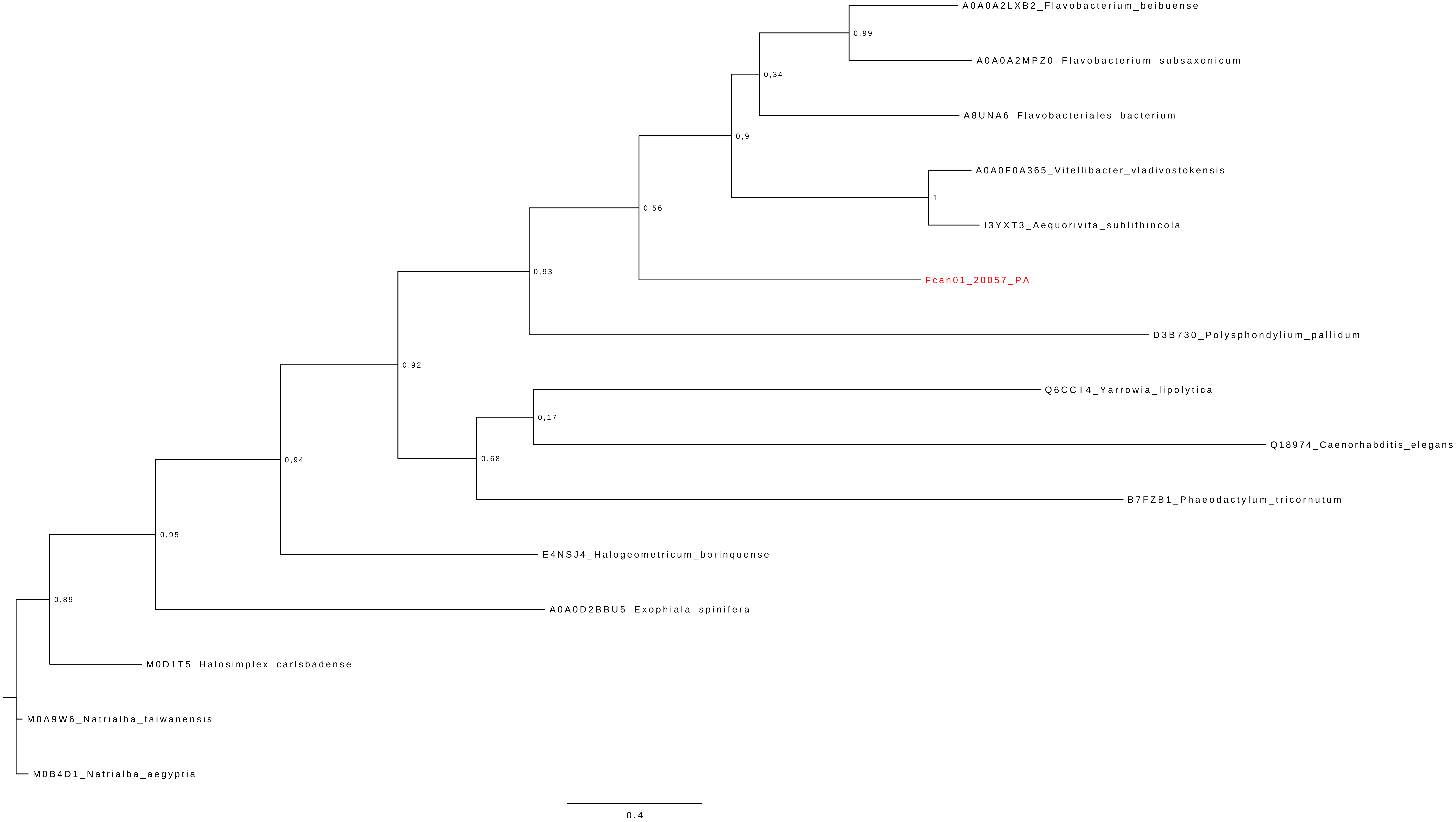
A0A0A2LXB2_Flavobacterium_beibuense
0,99
A0A0A2MPZ0_Flavobacterium_subsaxonicum
0,34
A8UNA6_Flavobacteriales_bacterium
0,9
A0A0F0A365_Vitellibacter_vladivostokensis
1
I3YXT3_Aequorivita_sublithincola
0,56
Fcan01_20057_PA
0,93
D3B730_Polysphondylium_pallidum
0,92
Q6CCT4_Yarrowia_lipolytica
0,17
Q18974_Caenorhabditis_elegans
0,68
B7FZB1_Phaeodactylum_tricornutum
0,94
E4NSJ4_Halogeometricum_borinquense
0,95
A0A0D2BBU5_Exophiala_spinifera
0,89
M0D1T5_Halosimplex_carlsbadense
M0A9W6_Natrialba_taiwanensis
M0B4D1_Natrialba_aegyptia
0.4

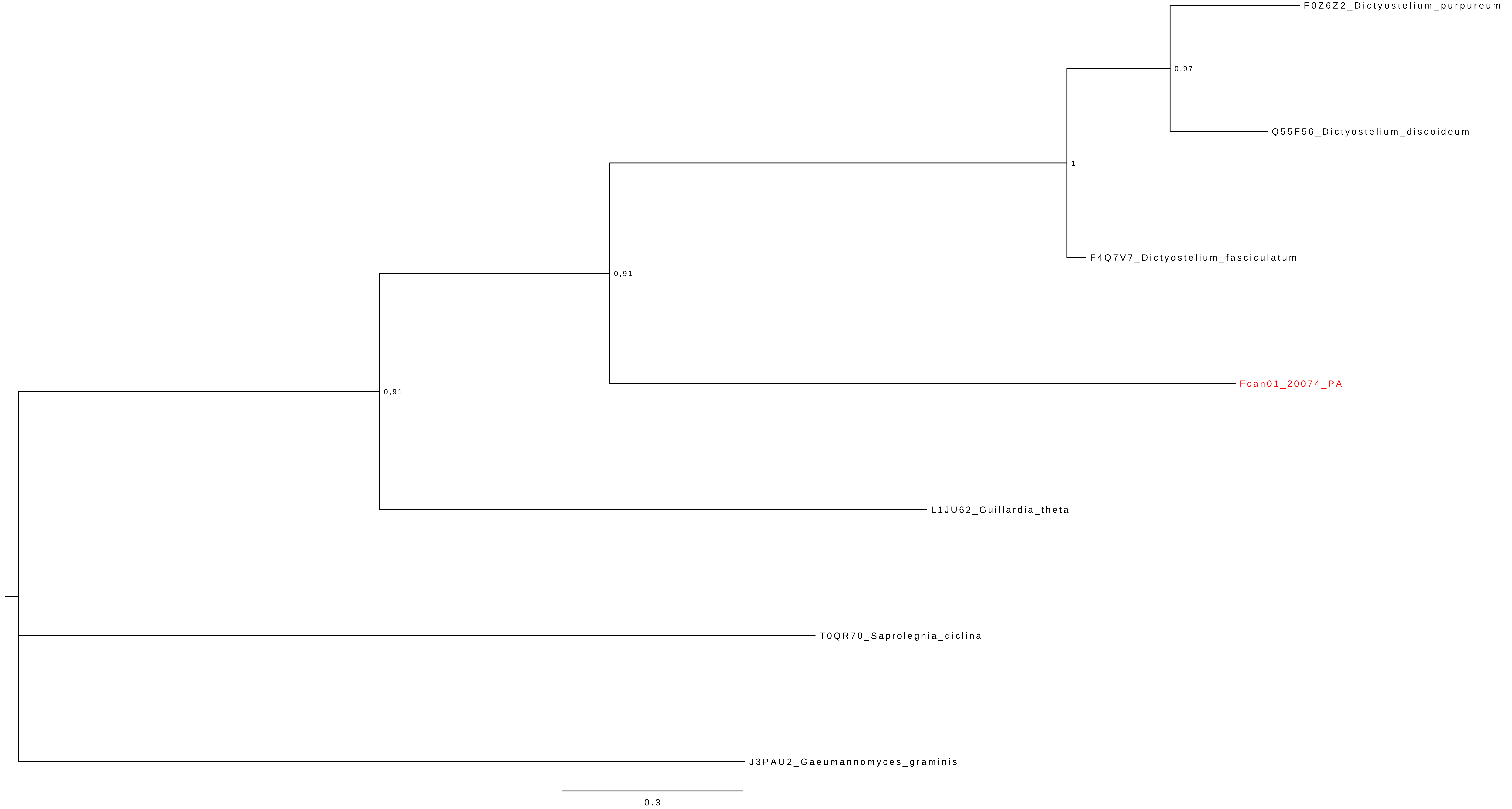
F0Z6Z2_Dictyostelium_purpureum
0,97
Q55F56_Dictyostelium_discoideum
1
F4Q7V7_Dictyostelium_fasciculatum
0,91
Fcan01_20074_PA
0,91
L1JU62_Guillardia_theta
T0QR70_Saprolegnia_diclina
J3PAU2_Gaeumannomyces_graminis
0.3

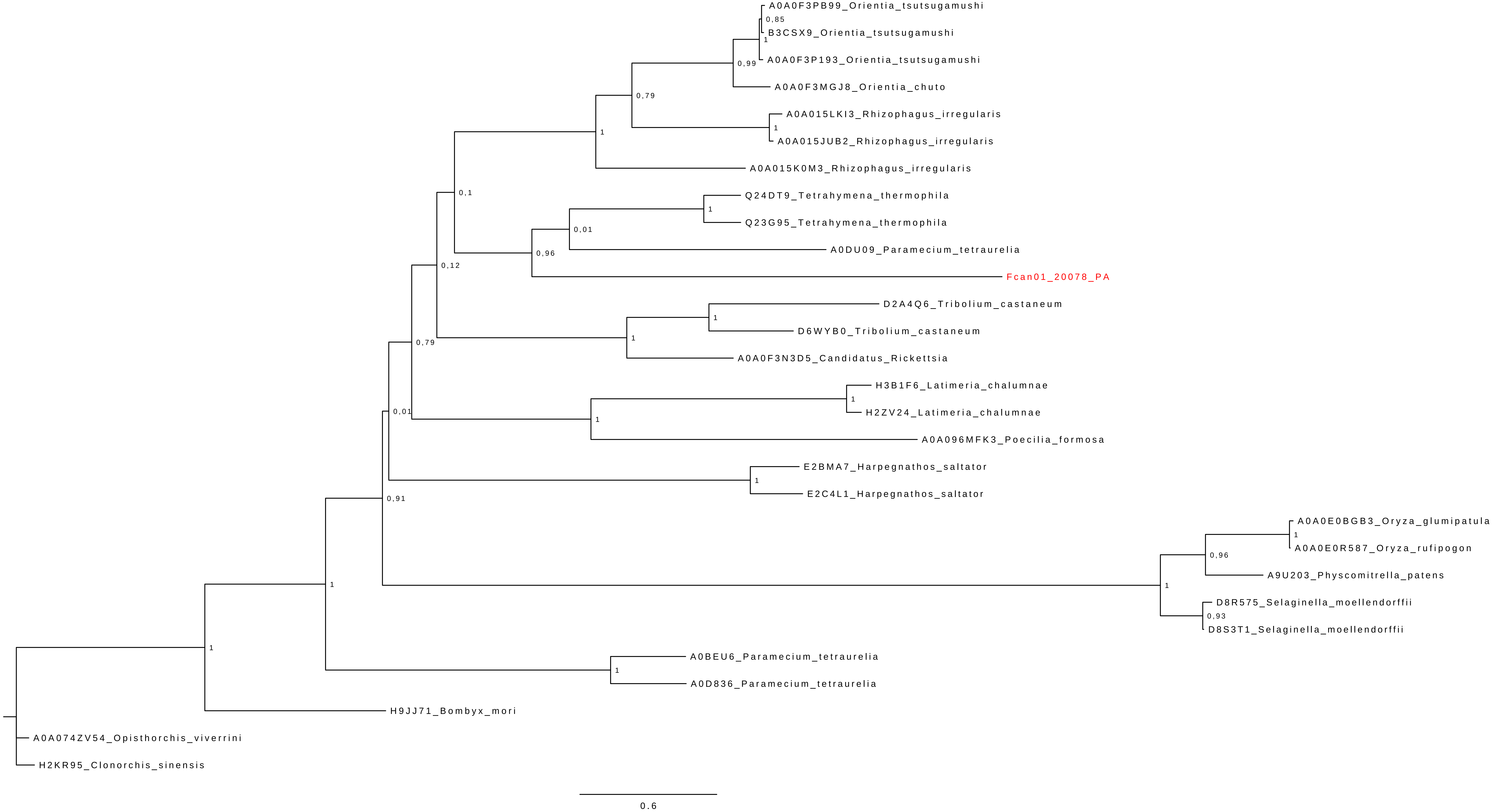
A0A0F3PB99_Orientia_tsutsugamushi
0,85
B3CSX9_Orientia_tsutsugamushi
1
A0A0F3P193_Orientia_tsutsugamushi
0,99
A0A0F3MGJ8_Orientia_chuto
0,79
A0A015LKI3_Rhizophagus_irregularis
1
A0A015JUB2_Rhizophagus_irregularis
1
A0A015K0M3_Rhizophagus_irregularis
0,1
Q24DT9_Tetrahymena_thermophila
1
Q23G95_Tetrahymena_thermophila
0,01
A0DU09_Paramecium_tetraurelia
0,96
Fcan01_20078_PA
0,12
D2A4Q6_Tribolium_castaneum
1
D6WYB0_Tribolium_castaneum
1
A0A0F3N3D5_Candidatus_Rickettsia
0,79
H3B1F6_Latimeria_chalumnae
1
H2ZV24_Latimeria_chalumnae
0,01
1
A0A096MFK3_Poecilia_formosa
E2BMA7_Harpegnathos_saltator
1
E2C4L1_Harpegnathos_saltator
0,91
A0A0E0BGB3_Oryza_glumipatula
1
A0A0E0R587_Oryza_rufipogon
0,96
A9U203_Physcomitrella_patens
1
1
D8R575_Selaginella_moellendorffii
0,93
D8S3T1_Selaginella_moellendorffii
A0BEU6_Paramecium_tetraurelia
1
1
A0D836_Paramecium_tetraurelia
H9JJ71_Bombyx_mori
A0A074ZV54_Opisthorchis_viverrini
H2KR95_Clonorchis_sinensis
0.6

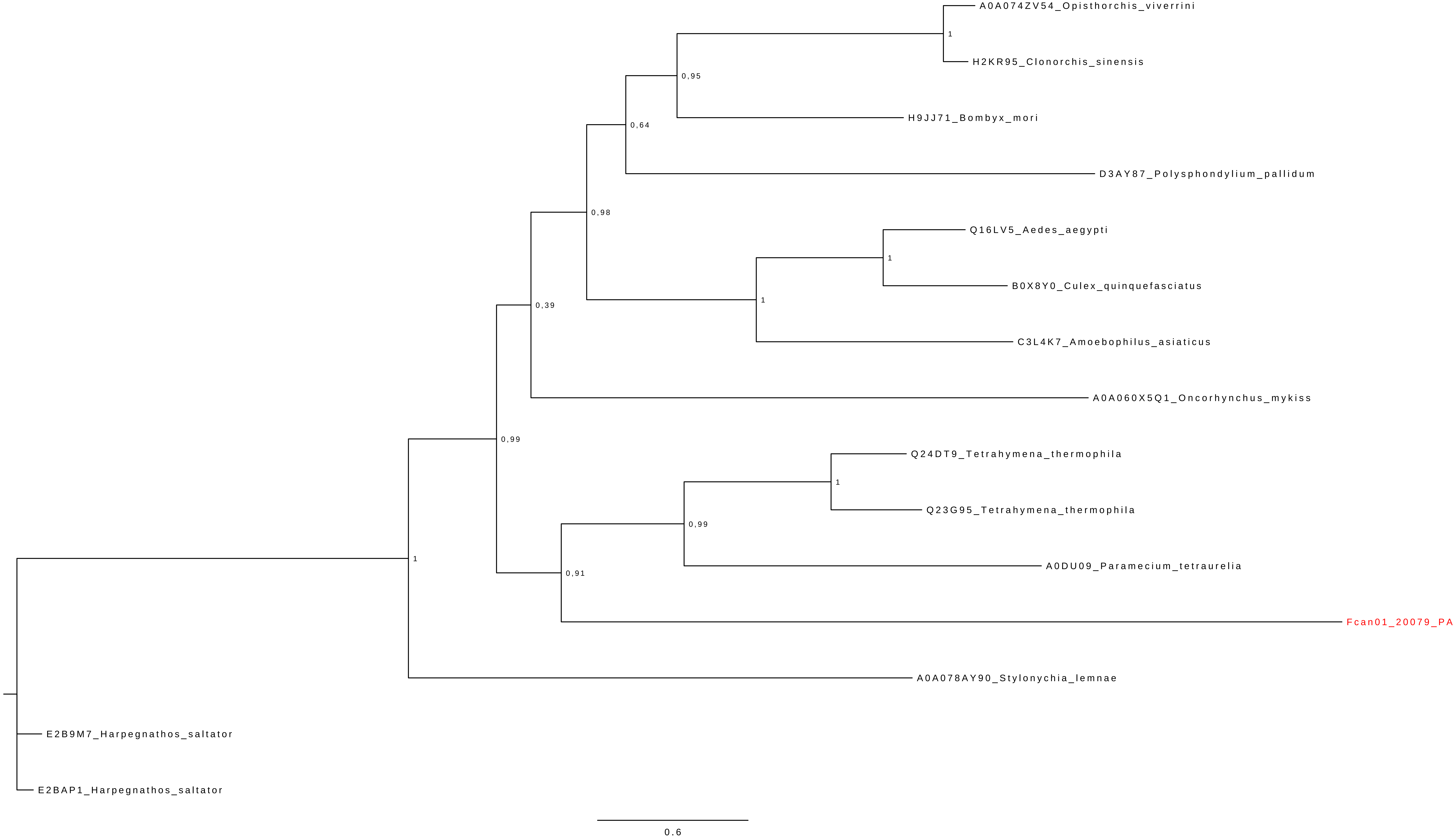
A0A074ZV54_Opisthorchis_viverrini
1
H2KR95_Clonorchis_sinensis
0,95
H9JJ71_Bombyx_mori
0,64
D3AY87_Polysphondylium_pallidum
0,98
Q16LV5_Aedes_aegypti
1
B0X8Y0_Culex_quinquefasciatus
1
C3L4K7_Amoebophilus_asiaticus
0,39
A0A060X5Q1_Oncorhynchus_mykiss
0,99
Q24DT9_Tetrahymena_thermophila
1
Q23G95_Tetrahymena_thermophila
0,99
A0DU09_Paramecium_tetraurelia
0,91
Fcan01_20079_PA
1
A0A078AY90_Stylonychia_lemnae
E2B9M7_Harpegnathos_saltator
E2BAP1_Harpegnathos_saltator
0.6

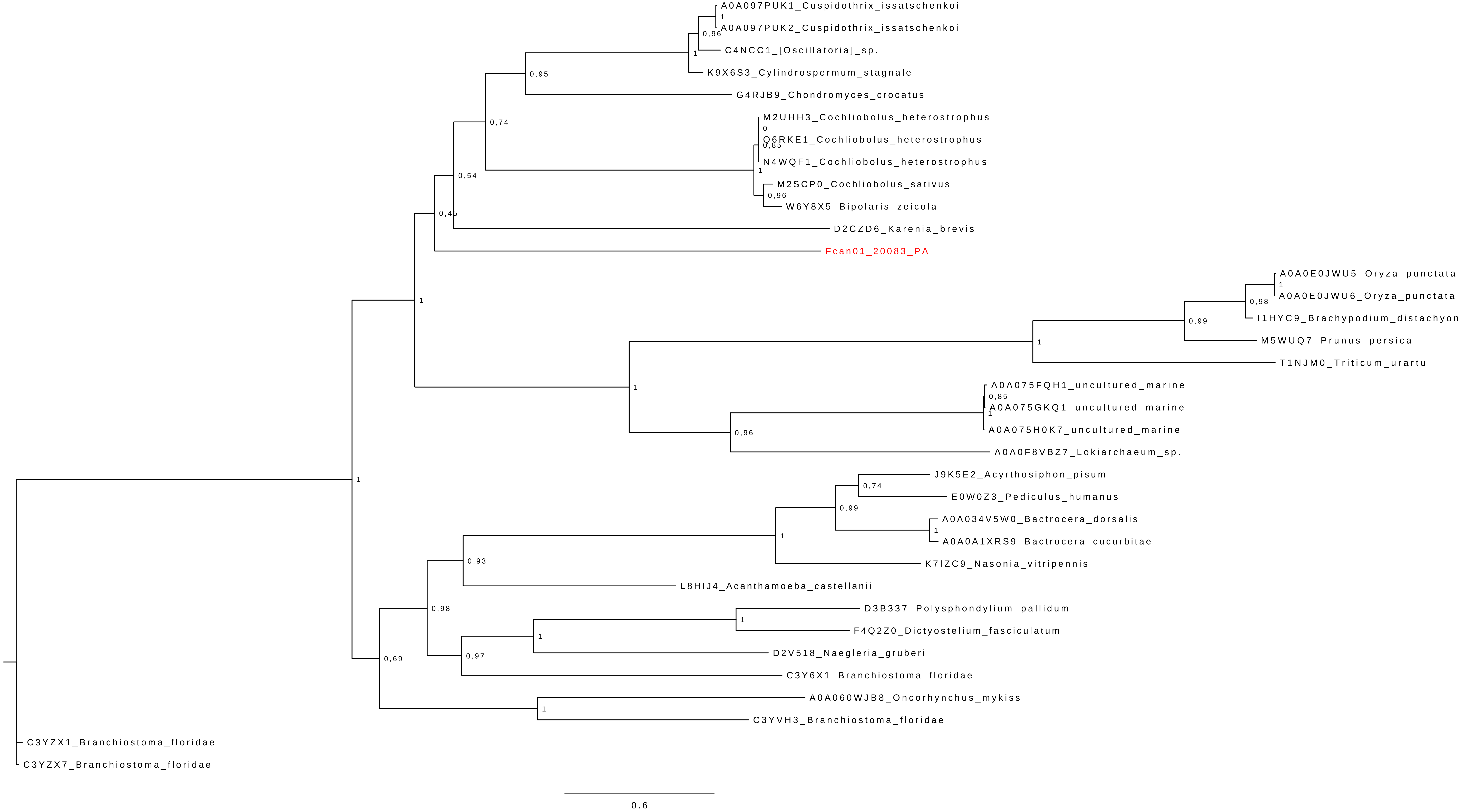
A0A097PUK1_Cuspidothrix_issatschenkoi
1
A0A097PUK2_Cuspidothrix_issatschenkoi
0,96
C4NCC1_[Oscillatoria]_sp.
1
K9X6S3_Cylindrospermum_stagnale
0,95
G4RJB9_Chondromyces_crocatus
0,74
M2UHH3_Cochliobolus_heterostrophus
0
Q6RKE1_Cochliobolus_heterostrophus
0,85
N4WQF1_Cochliobolus_heterostrophus
1
M2SCP0_Cochliobolus_sativus
0,96
W6Y8X5_Bipolaris_zeicola
0,54
0,45
D2CZD6_Karenia_brevis
Fcan01_20083_PA
1
A0A0E0JWU5_Oryza_punctata
1
A0A0E0JWU6_Oryza_punctata
0,98
I1HYC9_Brachypodium_distachyon
0,99
M5WUQ7_Prunus_persica
1
T1NJM0_Triticum_urartu
1
A0A075FQH1_uncultured_marine
0,85
A0A075GKQ1_uncultured_marine
1
A0A075H0K7_uncultured_marine
0,96
A0A0F8VBZ7_Lokiarchaeum_sp.
1
J9K5E2_Acyrthosiphon_pisum
0,74
E0W0Z3_Pediculus_humanus
0,99
A0A034V5W0_Bactrocera_dorsalis
1
A0A0A1XRS9_Bactrocera_cucurbitae
1
K7IZC9_Nasonia_vitripennis
0,93
L8HIJ4_Acanthamoeba_castellanii
0,98
D3B337_Polysphondylium_pallidum
1
F4Q2Z0_Dictyostelium_fasciculatum
1
D2V518_Naegleria_gruberi
0,97
C3Y6X1_Branchiostoma_floridae
0,69
A0A060WJB8_Oncorhynchus_mykiss
1
C3YVH3_Branchiostoma_floridae
C3YZX1_Branchiostoma_floridae
C3YZX7_Branchiostoma_floridae
0.6

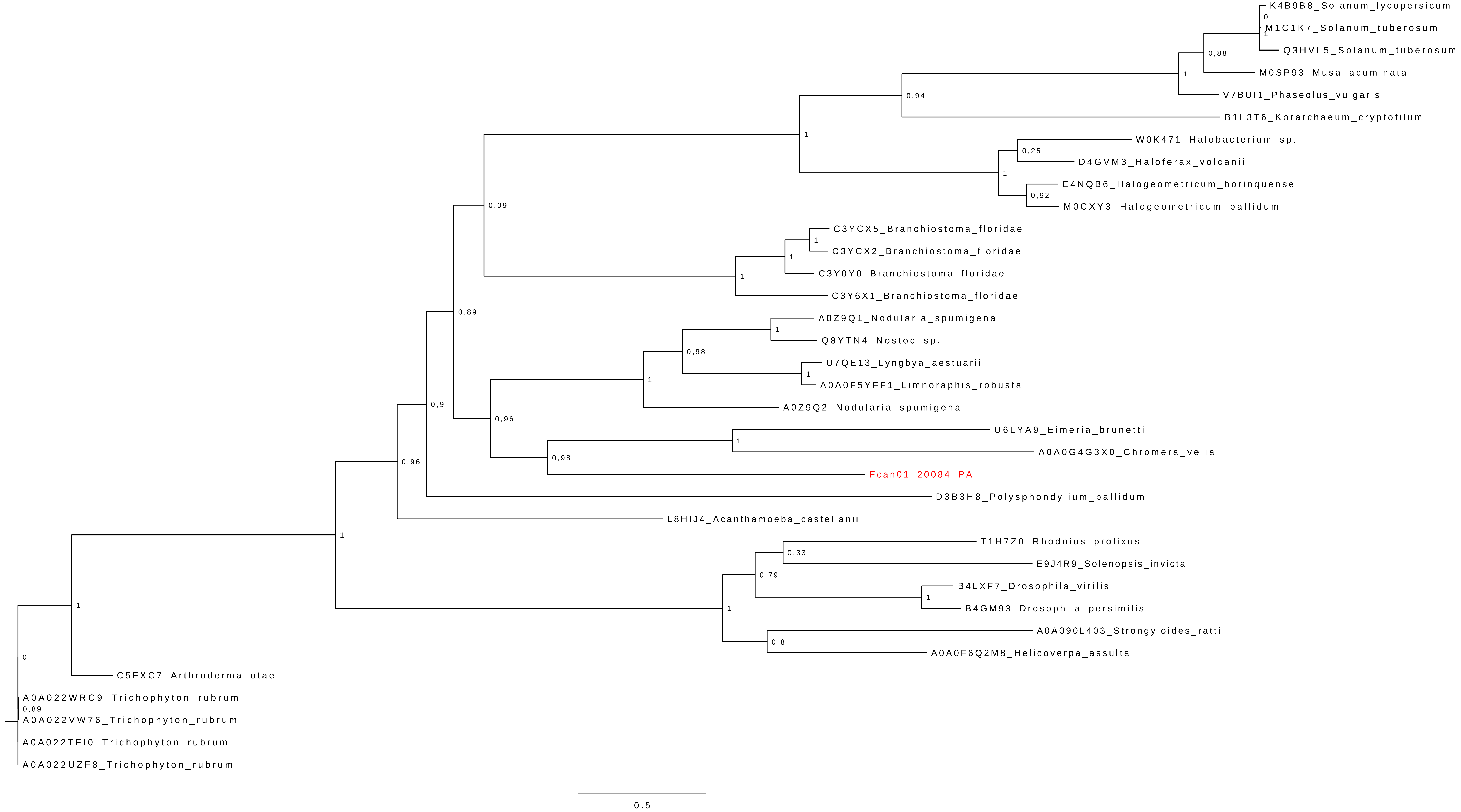
K4B9B8_Solanum_lycopersicum
M1C1K7_Solanum_tuberosum
Q3HVL5_Solanum_tuberosum
M0SP93_Musa_acuminata
V7BUI1_Phaseolus_vulgaris
B1L3T6_Korarchaeum_cryptofilum
W0K471_Halobacterium_sp.
D4GVM3_Haloferax_volcanii
E4NQB6_Halogeometricum_borinquense
M0CXY3_Halogeometricum_pallidum
C3YCX5_Branchiostoma_floridae
C3YCX2_Branchiostoma_floridae
C3Y0Y0_Branchiostoma_floridae
C3Y6X1_Branchiostoma_floridae
A0Z9Q1_Nodularia_spumigena
Q8YTN4_Nostoc_sp.
U7QE13_Lyngbya_aestuarii
A0A0F5YFF1_Limnoraphis_robusta
A0Z9Q2_Nodularia_spumigena
U6LYA9_Eimeria_brunetti
A0A0G4G3X0_Chromera_velia
Fcan01_20084_PA
D3B3H8_Polysphondylium_pallidum
L8HIJ4_Acanthamoeba_castellanii
T1H7Z0_Rhodnius_prolixus
E9J4R9_Solenopsis_invicta
B4LXF7_Drosophila_virilis
B4GM93_Drosophila_persimilis
A0A090L403_Strongyloides_ratti
A0A0F6Q2M8_Helicoverpa_assulta
C5FXC7_Arthroderma_otae
A0A022WRC9_Trichophyton_rubrum
A0A022VW76_Trichophyton_rubrum
A0A022TFI0_Trichophyton_rubrum
A0A022UZF8_Trichophyton_rubrum
0.5

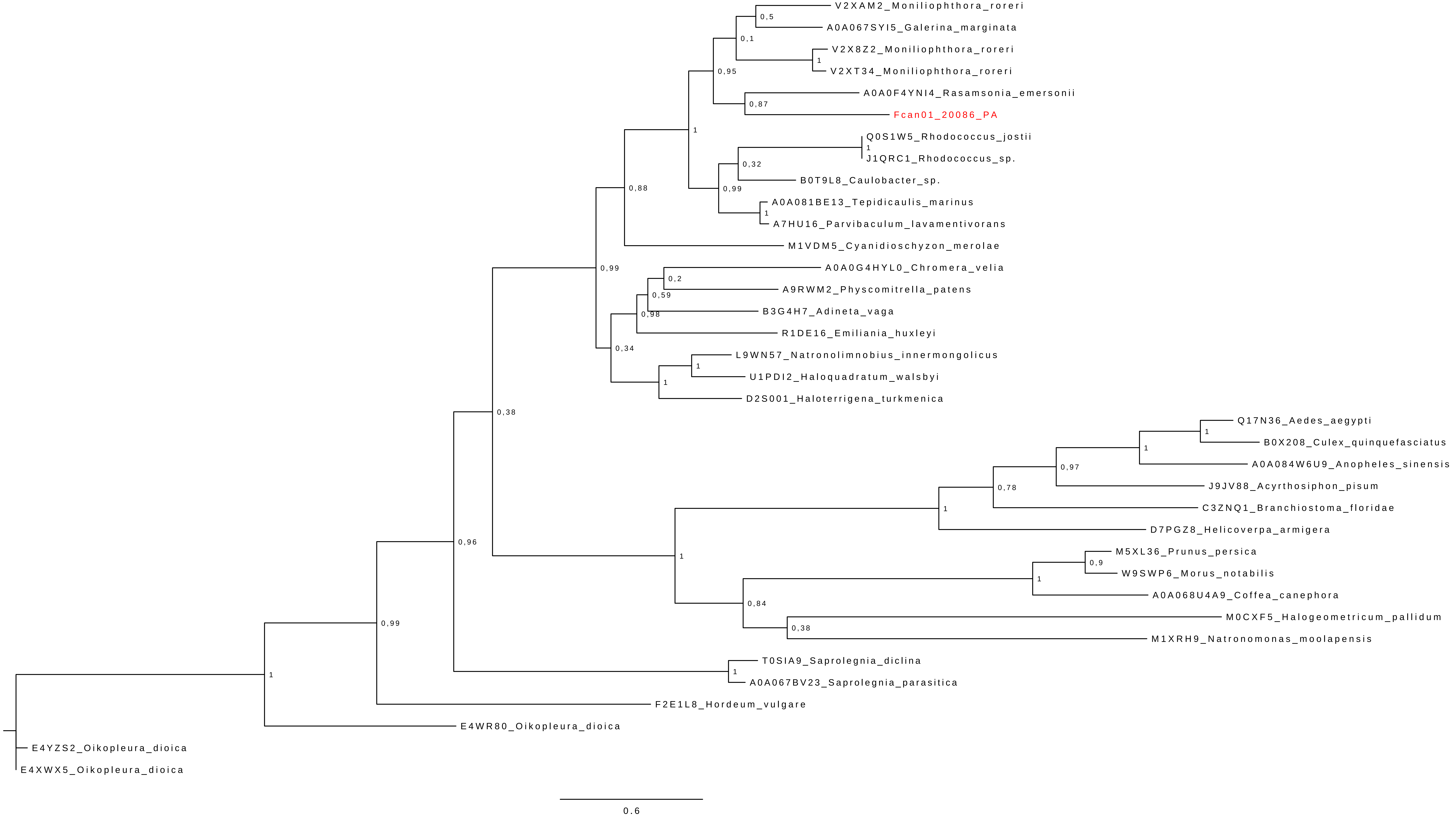
V2XAM2_Moniliophthora_roreri
A0A067SYI5_Galerina_marginata
V2X8Z2_Moniliophthora_roreri
V2XT34_Moniliophthora_roreri
A0A0F4YNI4_Rasamsonia_emersonii
Fcan01_20086_PA
Q0S1W5_Rhodococcus_jostii
J1QRC1_Rhodococcus_sp.
B0T9L8_Caulobacter_sp.
A0A081BE13_Tepidicaulis_marinus
A7HU16_Parvibaculum_lavamentivorans
M1VDM5_Cyanidioschyzon_merolae
A0A0G4HYL0_Chromera_velia
A9RWM2_Physcomitrella_patens
B3G4H7_Adineta_vaga
R1DE16_Emiliania_huxleyi
L9WN57_Natronolimnobius_innermongolicus
U1PDI2_Haloquadratum_walsbyi
D2S001_Haloterrigena_turkmenica
Q17N36_Aedes_aegypti
B0X208_Culex_quinquefasciatus
A0A084W6U9_Anopheles_sinensis
J9JV88_Acyrthosiphon_pisum
C3ZNQ1_Branchiostoma_floridae
D7PGZ8_Helicoverpa_armigera
M5XL36_Prunus_persica
W9SWP6_Morus_notabilis
A0A068U4A9_Coffea_canephora
M0CXF5_Halogeometricum_pallidum
M1XRH9_Natronomonas_moolapensis
T0SIA9_Saprolegnia_diclina
A0A067BV23_Saprolegnia_parasitica
F2E1L8_Hordeum_vulgare
E4WR80_Oikopleura_dioica
E4YZS2_Oikopleura_dioica
E4XWX5_Oikopleura_dioica
0,5
0,1
1
0,95
0,87
1
1
0,32
0,99
1
0,88
0,99
0,2
0,59
0,98
0,34
1
1
0,38
1
1
0,97
0,78
1
0,9
1
1
0,84
0,38
0,96
1
0,99
1
0.6

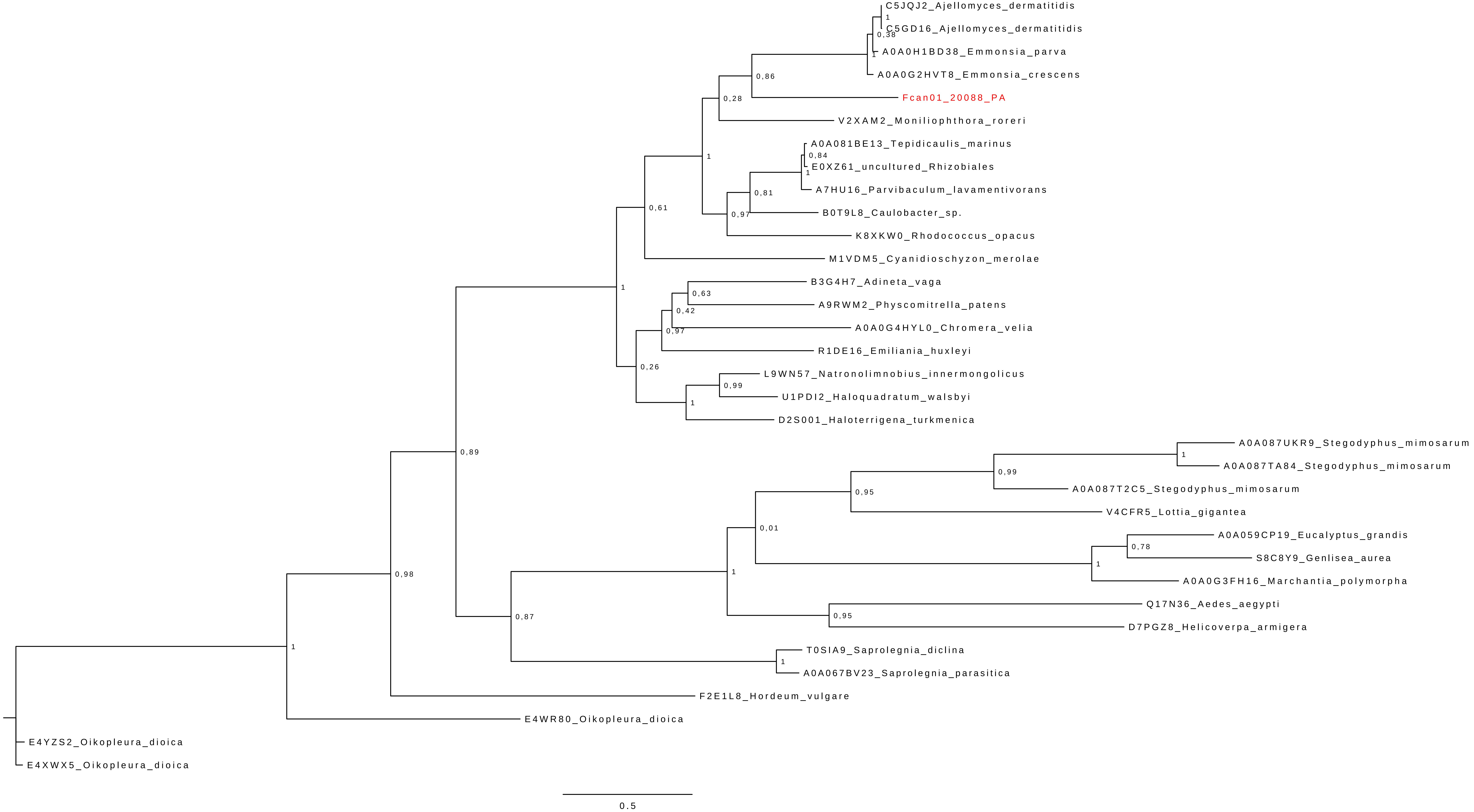
C5JQJ2_Ajellomyces_dermatitidis
1
C5GD16_Ajellomyces_dermatitidis
0,38
A0A0H1BD38_Emmonsia_parva
1
A0A0G2HVT8_Emmonsia_crescens
0,86
Fcan01_20088_PA
0,28
V2XAM2_Moniliophthora_roreri
A0A081BE13_Tepidicaulis_marinus
0,84
E0XZ61_uncultured_Rhizobiales
1
A7HU16_Parvibaculum_lavamentivorans
0,81
B0T9L8_Caulobacter_sp.
0,97
K8XKW0_Rhodococcus_opacus
1
0,61
M1VDM5_Cyanidioschyzon_merolae
1
B3G4H7_Adineta_vaga
0,63
A9RWM2_Physcomitrella_patens
0,42
A0A0G4HYL0_Chromera_velia
0,97
R1DE16_Emiliania_huxleyi
0,26
L9WN57_Natronolimnobius_innermongolicus
0,99
U1PDI2_Haloquadratum_walsbyi
1
D2S001_Haloterrigena_turkmenica
0,89
A0A087UKR9_Stegodyphus_mimosarum
1
A0A087TA84_Stegodyphus_mimosarum
0,99
A0A087T2C5_Stegodyphus_mimosarum
0,95
V4CFR5_Lottia_gigantea
0,01
A0A059CP19_Eucalyptus_grandis
0,78
S8C8Y9_Genlisea_aurea
1
A0A0G3FH16_Marchantia_polymorpha
0,98
1
Q17N36_Aedes_aegypti
0,95
D7PGZ8_Helicoverpa_armigera
0,87
T0SIA9_Saprolegnia_diclina
1
A0A067BV23_Saprolegnia_parasitica
1
F2E1L8_Hordeum_vulgare
E4WR80_Oikopleura_dioica
E4YZS2_Oikopleura_dioica
E4XWX5_Oikopleura_dioica
0.5

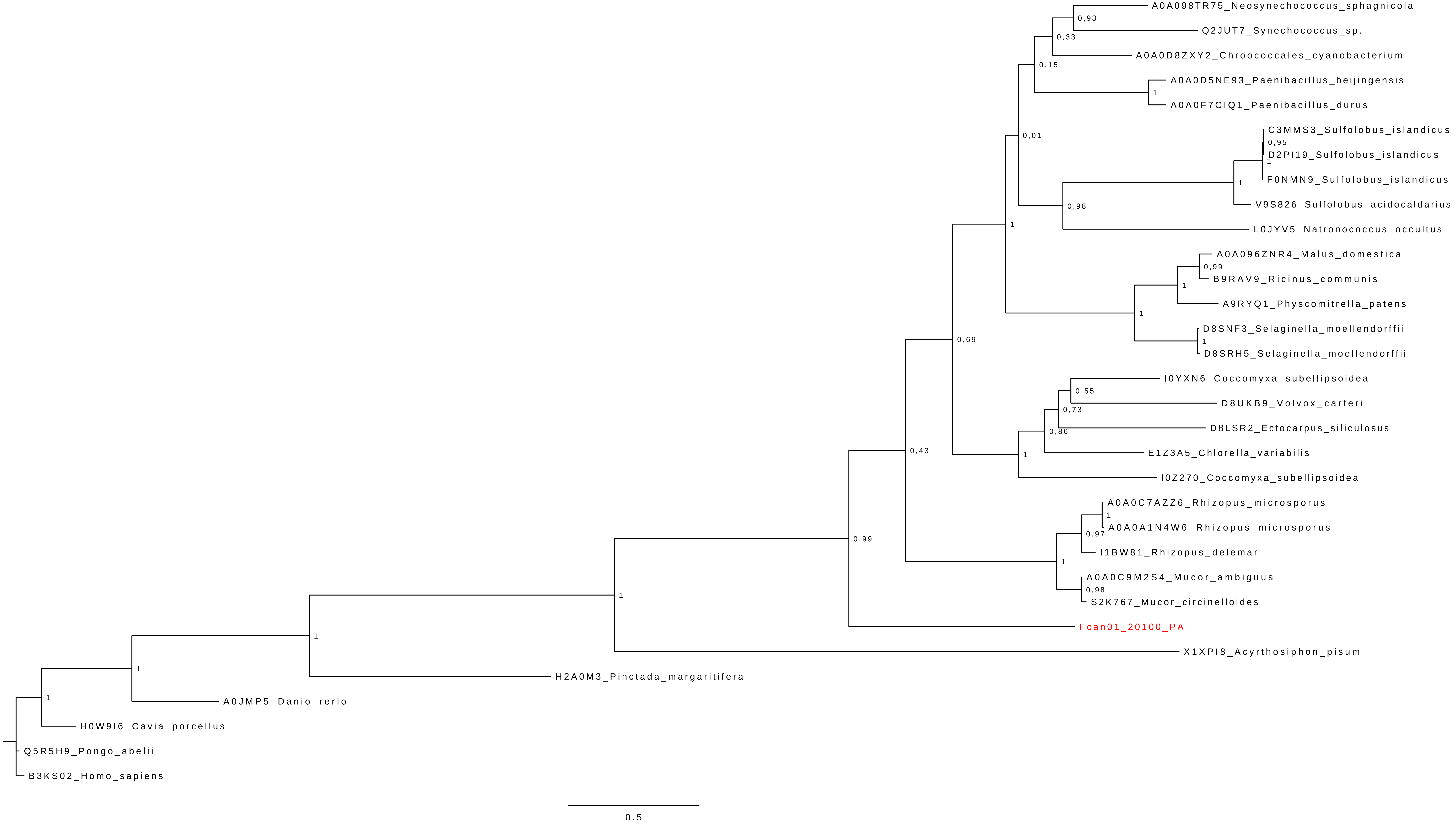

A0A098TR75_Neosynechococcus_sphagnicola
0,93
Q2JUT7_Synechococcus_sp.
0,33
A0A0D8ZXY2_Chroococcales_cyanobacterium
0,15
A0A0D5NE93_Paenibacillus_beijingensis
1
A0A0F7CIQ1_Paenibacillus_durus
0,01
C3MMS3_Sulfolobus_islandicus
0,95
D2PI19_Sulfolobus_islandicus
1
F0NMN9_Sulfolobus_islandicus
1
V9S826_Sulfolobus_acidocaldarius
0,98
L0JYV5_Natronococcus_occultus
1
A0A096ZNR4_Malus_domestica
0,99
B9RAV9_Ricinus_communis
1
A9RYQ1_Physcomitrella_patens
1
D8SNF3_Selaginella_moellendorffii
1
D8SRH5_Selaginella_moellendorffii
0,69
I0YXN6_Coccomyxa_subellipsoidea
0,55
D8UKB9_Volvox_carteri
0,73
D8LSR2_Ectocarpus_siliculosus
0,86
E1Z3A5_Chlorella_variabilis
1
I0Z270_Coccomyxa_subellipsoidea
0,43
A0A0C7AZZ6_Rhizopus_microsporus
1
A0A0A1N4W6_Rhizopus_microsporus
0,97
I1BW81_Rhizopus_delemar
0,99
1
A0A0C9M2S4_Mucor_ambiguus
0,98
S2K767_Mucor_circinelloides
1
Fcan01_20100_PA
X1XPI8_Acyrthosiphon_pisum
1
H2A0M3_Pinctada_margaritifera
1
A0JMP5_Danio_rerio
1
H0W9I6_Cavia_porcellus
Q5R5H9_Pongo_abelii
B3KS02_Homo_sapiens
0.5

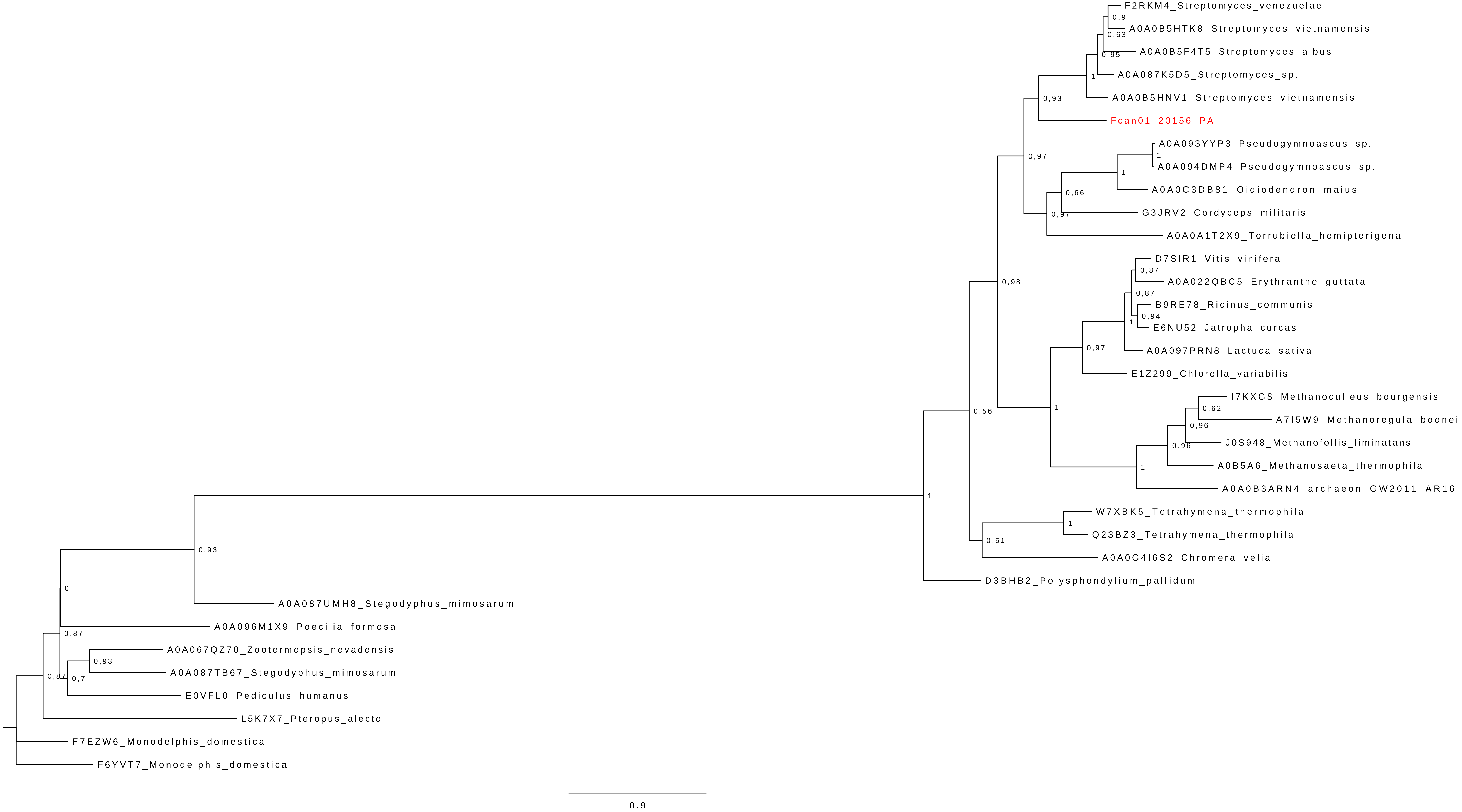
F2RKM4_Streptomyces_venezuelae
0,9
A0A0B5HTK8_Streptomyces_vietnamensis
0,63
A0A0B5F4T5_Streptomyces_albus
0,95
A0A087K5D5_Streptomyces_sp.
1
A0A0B5HNV1_Streptomyces_vietnamensis
0,93
Fcan01_20156_PA
A0A093YYP3_Pseudogymnoascus_sp.
1
A0A094DMP4_Pseudogymnoascus_sp.
0,97
1
A0A0C3DB81_Oidiodendron_maius
0,66
G3JRV2_Cordyceps_militaris
0,97
A0A0A1T2X9_Torrubiella_hemipterigena
D7SIR1_Vitis_vinifera
0,87
A0A022QBC5_Erythranthe_guttata
0,98
0,87
B9RE78_Ricinus_communis
0,94
E6NU52_Jatropha_curcas
1
0,97
A0A097PRN8_Lactuca_sativa
E1Z299_Chlorella_variabilis
I7KXG8_Methanoculleus_bourgensis
0,62
1
A7I5W9_Methanoregula_boonei
0,56
0,96
J0S948_Methanofollis_liminatans
0,96
A0B5A6_Methanosaeta_thermophila
1
A0A0B3ARN4_archaeon_GW2011_AR16
1
W7XBK5_Tetrahymena_thermophila
1
Q23BZ3_Tetrahymena_thermophila
0,51
A0A0G4I6S2_Chromera_velia
D3BHB2_Polysphondylium_pallidum
0,93
0
A0A087UMH8_Stegodyphus_mimosarum
A0A096M1X9_Poecilia_formosa
0,87
A0A067QZ70_Zootermopsis_nevadensis
0,93
A0A087TB67_Stegodyphus_mimosarum
0,87
0,7
E0VFL0_Pediculus_humanus
L5K7X7_Pteropus_alecto
F7EZW6_Monodelphis_domestica
F6YVT7_Monodelphis_domestica
0.9

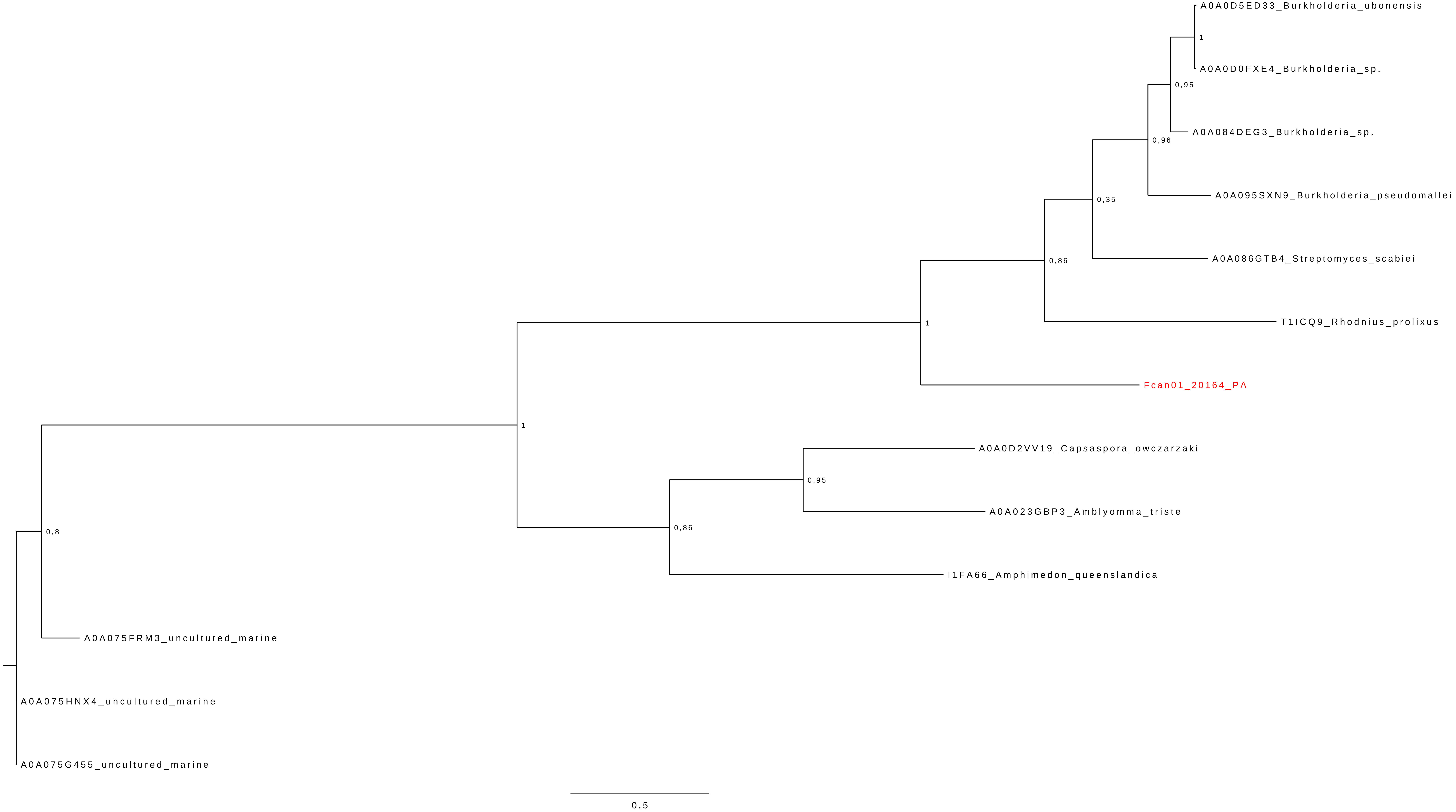
A0A0D5ED33_Burkholderia_ubonensis
1
A0A0D0FXE4_Burkholderia_sp.
0,95
A0A084DEG3_Burkholderia_sp.
0,96
A0A095SXN9_Burkholderia_pseudomallei
0,35
A0A086GTB4_Streptomyces_scabiei
0,86
T1ICQ9_Rhodnius_prolixus
1
Fcan01_20164_PA
1
A0A0D2VV19_Capsaspora_owczarzaki
0,95
A0A023GBP3_Amblyomma_triste
0,86
I1FA66_Amphimedon_queenslandica
0,8
A0A075FRM3_uncultured_marine
A0A075HNX4_uncultured_marine
A0A075G455_uncultured_marine
0.5

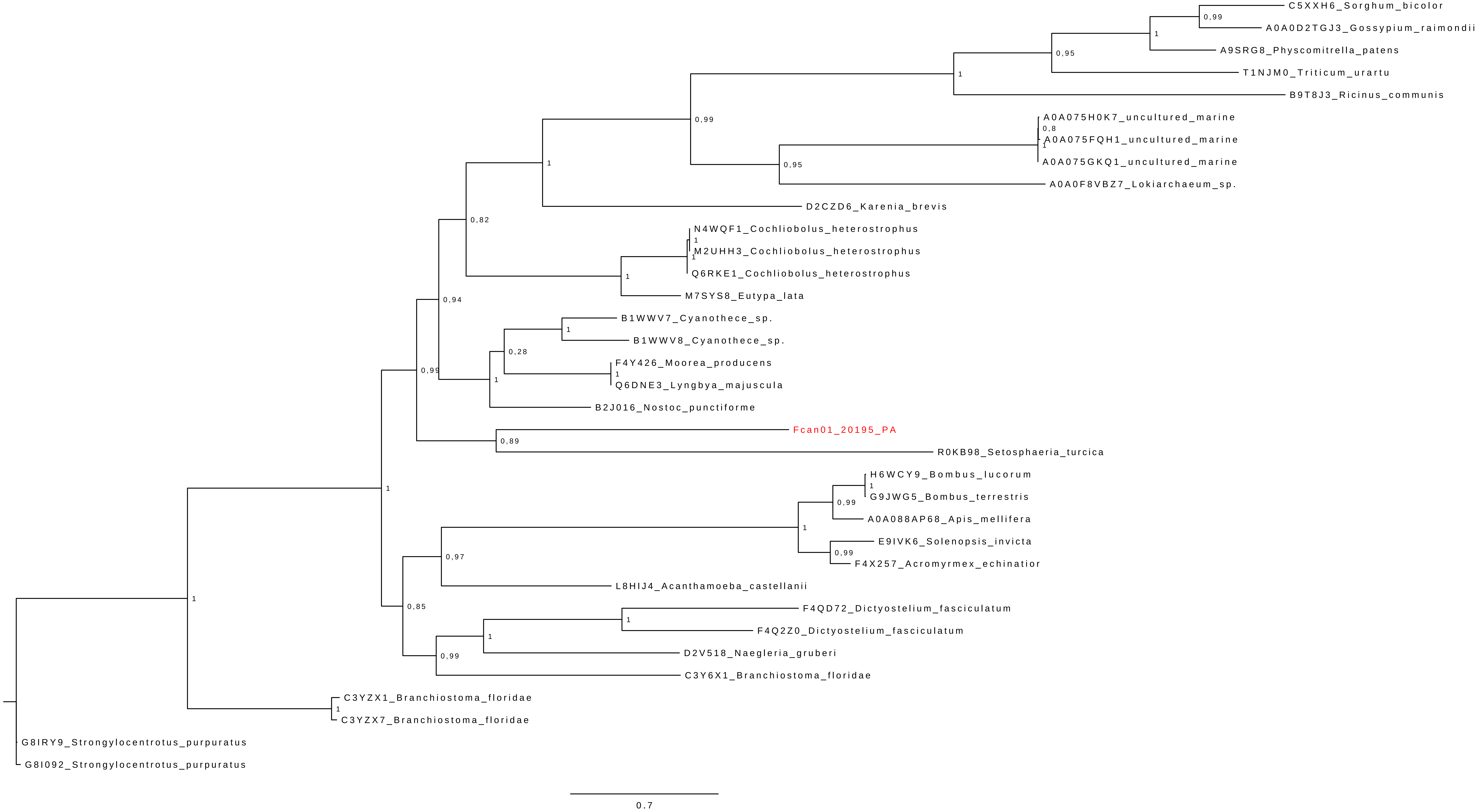
C5XXH6_Sorghum_bicolor
A0A0D2TGJ3_Gossypium_raimondii
A9SRG8_Physcomitrella_patens
T1NJM0_Triticum_urartu
B9T8J3_Ricinus_communis
A0A075H0K7_uncultured_marine
A0A075FQH1_uncultured_marine
A0A075GKQ1_uncultured_marine
A0A0F8VBZ7_Lokiarchaeum_sp.
D2CZD6_Karenia_brevis
N4WQF1_Cochliobolus_heterostrophus
M2UHH3_Cochliobolus_heterostrophus
Q6RKE1_Cochliobolus_heterostrophus
M7SYS8_Eutypa_lata
B1WWV7_Cyanothece_sp.
B1WWV8_Cyanothece_sp.
F4Y426_Moorea_producens
Q6DNE3_Lyngbya_majuscula
B2J016_Nostoc_punctiforme
Fcan01_20195_PA
R0KB98_Setosphaeria_turcica
H6WCY9_Bombus_lucorum
G9JWG5_Bombus_terrestris
A0A088AP68_Apis_mellifera
E9IVK6_Solenopsis_invicta
F4X257_Acromyrmex_echinatior
L8HIJ4_Acanthamoeba_castellanii
F4QD72_Dictyostelium_fasciculatum
F4Q2Z0_Dictyostelium_fasciculatum
D2V518_Naegleria_gruberi
C3Y6X1_Branchiostoma_floridae
C3YZX1_Branchiostoma_floridae
C3YZX7_Branchiostoma_floridae
G8IRY9_Strongylocentrotus_purpuratus
G8I092_Strongylocentrotus_purpuratus
0.7

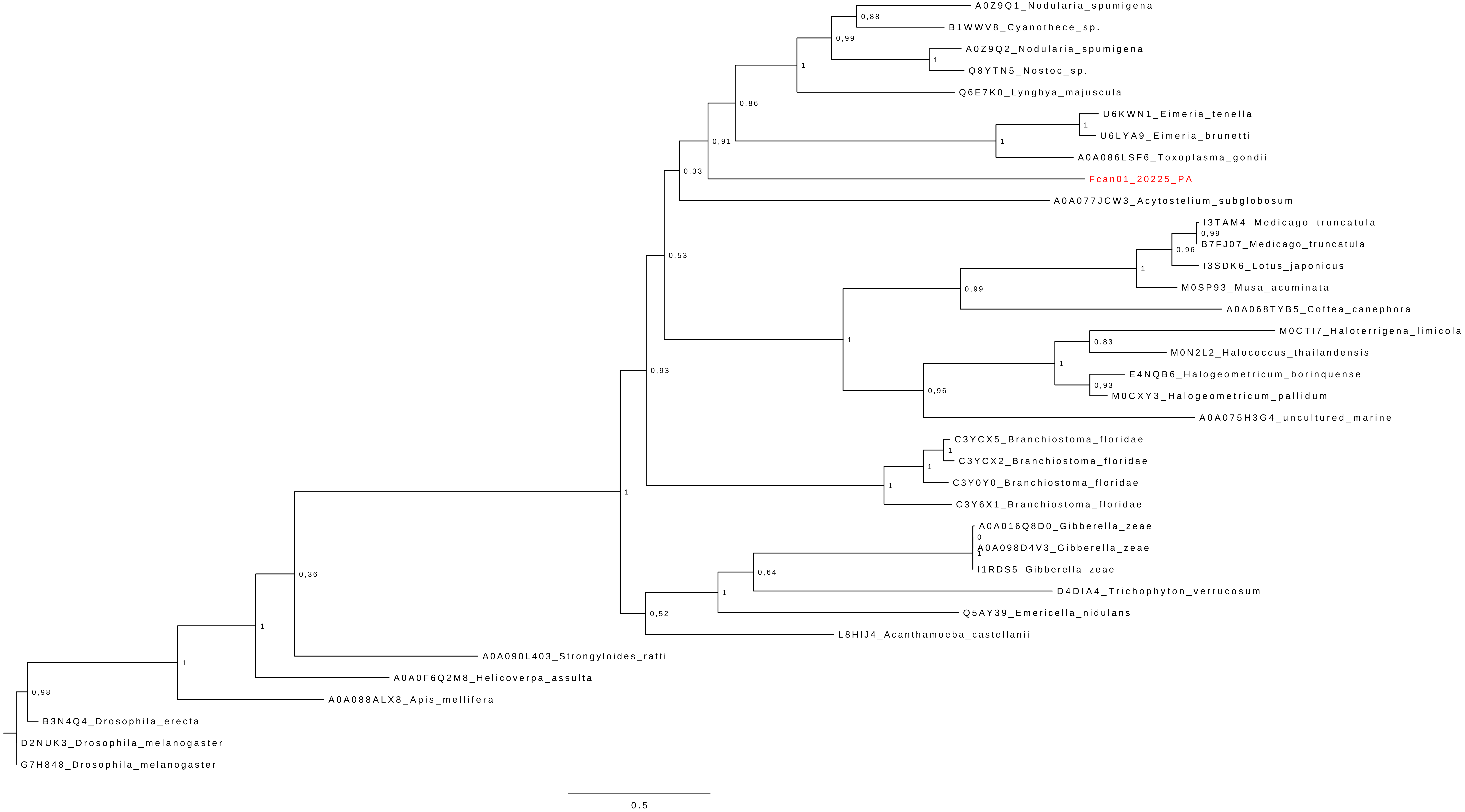
A0Z9Q1_Nodularia_spumigena
B1WWV8_Cyanothece_sp.
A0Z9Q2_Nodularia_spumigena
Q8YTN5_Nostoc_sp.
Q6E7K0_Lyngbya_majuscula
U6KWN1_Eimeria_tenella
U6LYA9_Eimeria_brunetti
A0A086LSF6_Toxoplasma_gondii
Fcan01_20225_PA
A0A077JCW3_Acytostelium_subglobosum
I3TAM4_Medicago_truncatula
B7FJ07_Medicago_truncatula
I3SDK6_Lotus_japonicus
M0SP93_Musa_acuminata
A0A068TYB5_Coffea_canephora
M0CTI7_Haloterrigena_limicola
M0N2L2_Halococcus_thailandensis
E4NQB6_Halogeometricum_borinquense
M0CXY3_Halogeometricum_pallidum
A0A075H3G4_uncultured_marine
C3YCX5_Branchiostoma_floridae
C3YCX2_Branchiostoma_floridae
C3Y0Y0_Branchiostoma_floridae
C3Y6X1_Branchiostoma_floridae
A0A016Q8D0_Gibberella_zeae
A0A098D4V3_Gibberella_zeae
I1RDS5_Gibberella_zeae
D4DIA4_Trichophyton_verrucosum
Q5AY39_Emericella_nidulans
L8HIJ4_Acanthamoeba_castellanii
A0A090L403_Strongyloides_ratti
A0A0F6Q2M8_Helicoverpa_assulta
A0A088ALX8_Apis_mellifera
B3N4Q4_Drosophila_erecta
D2NUK3_Drosophila_melanogaster
G7H848_Drosophila_melanogaster
0.5

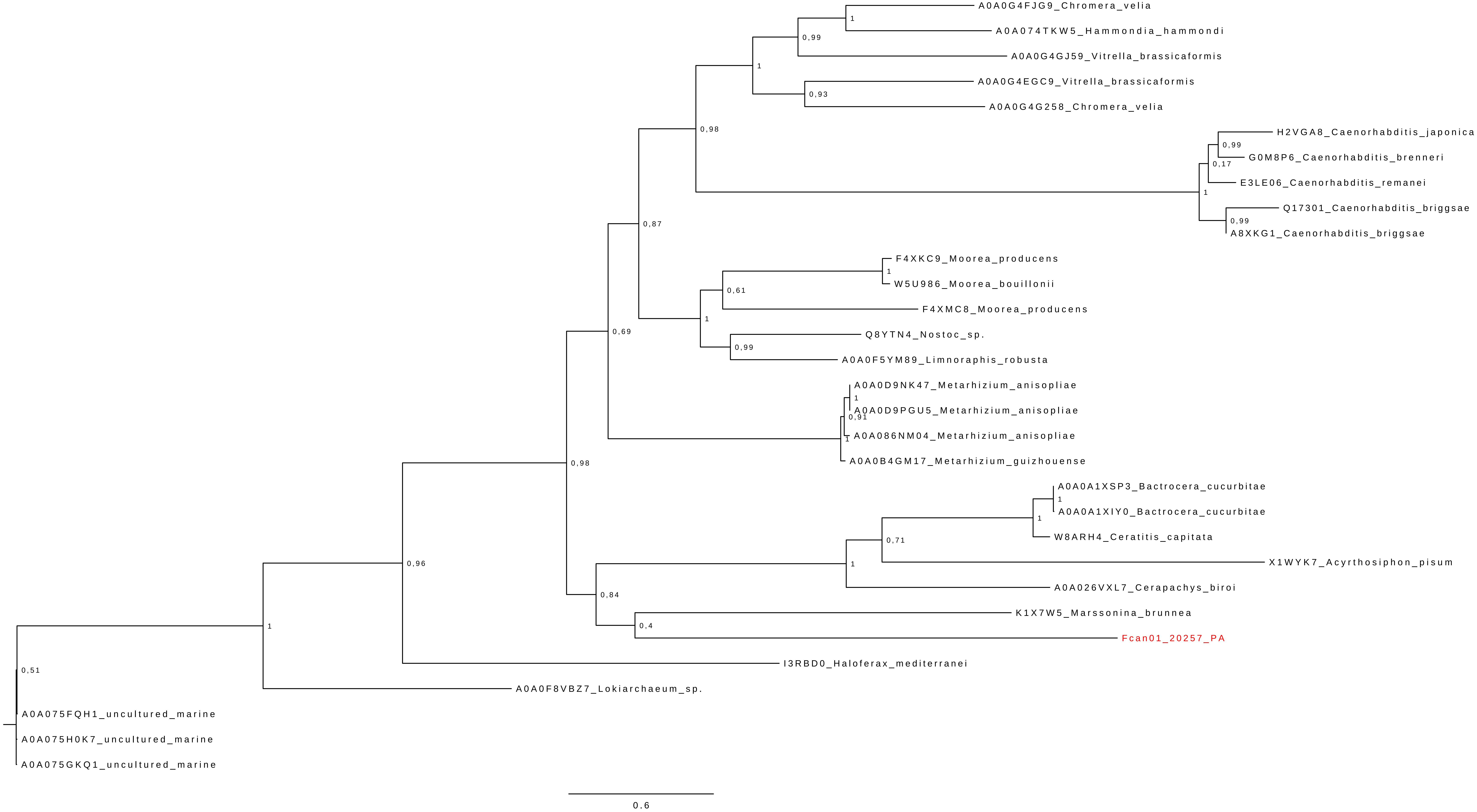
A0A0G4FJG9_Chromera_velia
A0A074TKW5_Hammondia_hammondi
A0A0G4GJ59_Vitrella_brassicaformis
A0A0G4EGC9_Vitrella_brassicaformis
A0A0G4G258_Chromera_velia
H2VGA8_Caenorhabditis_japonica
G0M8P6_Caenorhabditis_brenneri
E3LE06_Caenorhabditis_remanei
Q17301_Caenorhabditis_briggsae
A8XKG1_Caenorhabditis_briggsae
F4XKC9_Moorea_producens
W5U986_Moorea_bouillonii
F4XMC8_Moorea_producens
Q8YTN4_Nostoc_sp.
A0A0F5YM89_Limnoraphis_robusta
A0A0D9NK47_Metarhizium_anisopliae
A0A0D9PGU5_Metarhizium_anisopliae
A0A086NM04_Metarhizium_anisopliae
A0A0B4GM17_Metarhizium_guizhouense
A0A0A1XSP3_Bactrocera_cucurbitae
A0A0A1XIY0_Bactrocera_cucurbitae
W8ARH4_Ceratitis_capitata
X1WYK7_Acyrthosiphon_pisum
A0A026VXL7_Cerapachys_biroi
K1X7W5_Marssonina_brunnea
Fcan01_20257_PA
I3RBD0_Haloferax_mediterranei
A0A0F8VBZ7_Lokiarchaeum_sp.
A0A075FQH1_uncultured_marine
A0A075H0K7_uncultured_marine
A0A075GKQ1_uncultured_marine
1
0,99
1
0,93
0,98
0,99
0,17
1
0,99
0,87
1
0,61
1
0,99
0,69
1
0,91
1
0,98
1
1
0,71
1
0,84
0,4
0,96
1
0,51
0.6

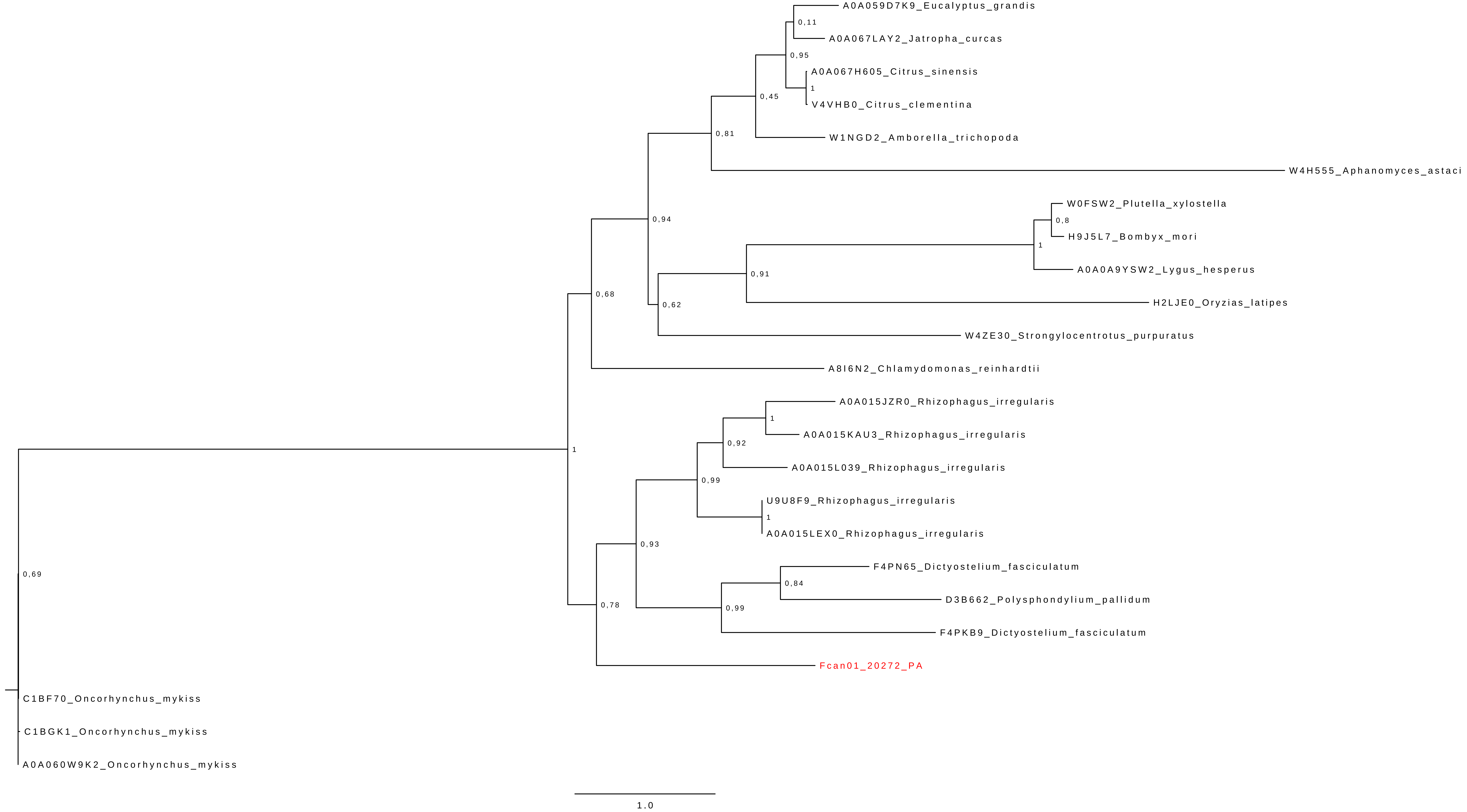
A0A059D7K9_Eucalyptus_grandis
0,11
A0A067LAY2_Jatropha_curcas
0,95
A0A067H605_Citrus_sinensis
1
V4VHB0_Citrus_clementina
0,45
W1NGD2_Amborella_trichopoda
0,81
W4H555_Aphanomyces_astaci
W0FSW2_Plutella_xylostella
0,8
H9J5L7_Bombyx_mori
1
A0A0A9YSW2_Lygus_hesperus
0,94
0,91
H2LJE0_Oryzias_latipes
0,62
W4ZE30_Strongylocentrotus_purpuratus
0,68
A8I6N2_Chlamydomonas_reinhardtii
A0A015JZR0_Rhizophagus_irregularis
1
A0A015KAU3_Rhizophagus_irregularis
0,92
A0A015L039_Rhizophagus_irregularis
1
0,99
U9U8F9_Rhizophagus_irregularis
1
A0A015LEX0_Rhizophagus_irregularis
0,93
F4PN65_Dictyostelium_fasciculatum
0,84
D3B662_Polysphondylium_pallidum
0,78
0,99
F4PKB9_Dictyostelium_fasciculatum
Fcan01_20272_PA
0,69
C1BF70_Oncorhynchus_mykiss
C1BGK1_Oncorhynchus_mykiss
A0A060W9K2_Oncorhynchus_mykiss
1.0

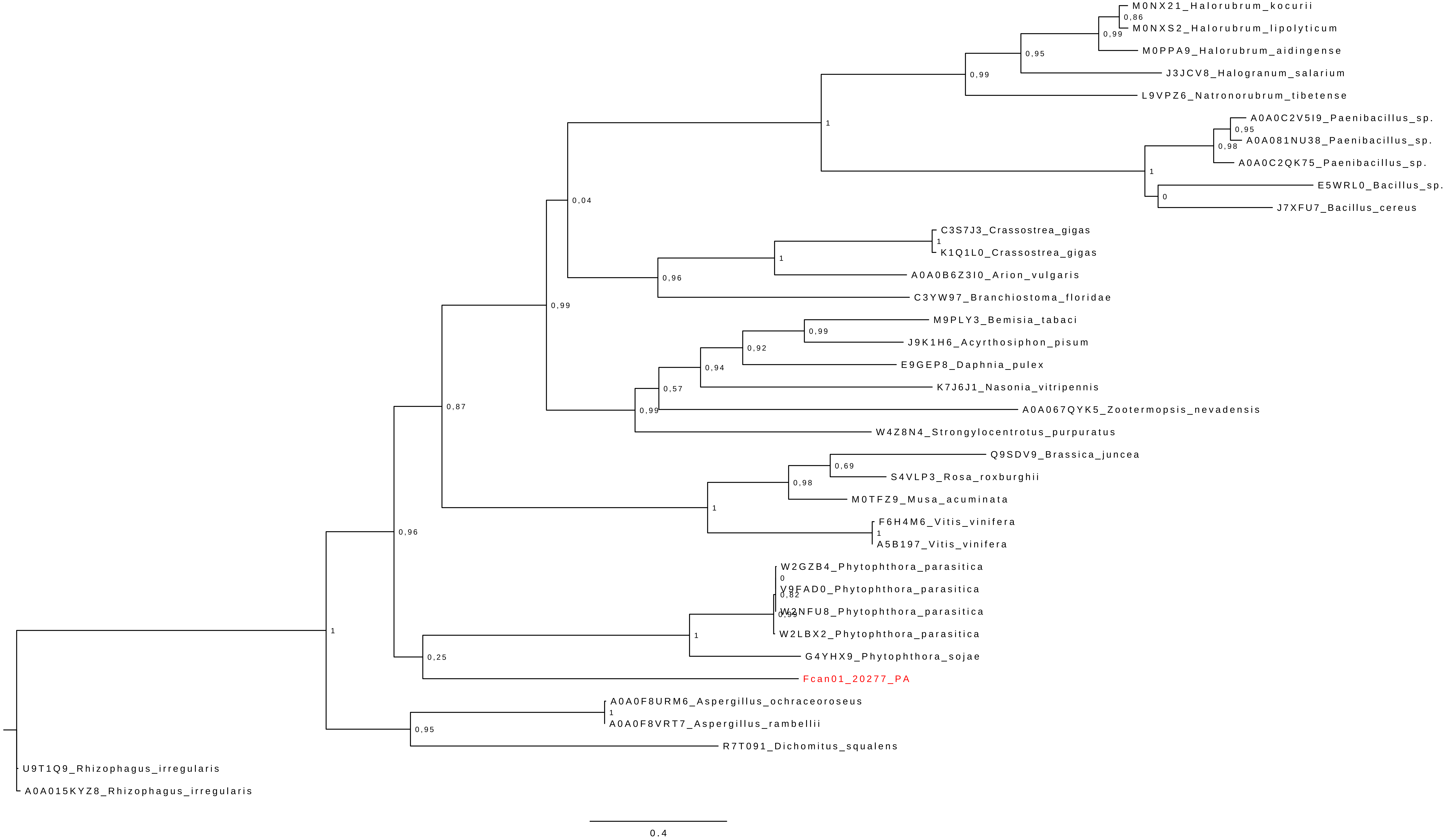
M0NX21_Halorubrum_kocurii
M0NXS2_Halorubrum_lipolyticum
M0PPA9_Halorubrum_aidingense
J3JCV8_Halogranum_salarium
L9VPZ6_Natronorubrum_tibetense
A0A0C2V5I9_Paenibacillus_sp.
A0A081NU38_Paenibacillus_sp.
A0A0C2QK75_Paenibacillus_sp.
E5WRL0_Bacillus_sp.
J7XFU7_Bacillus_cereus
C3S7J3_Crassostrea_gigas
K1Q1L0_Crassostrea_gigas
A0A0B6Z3I0_Arion_vulgaris
C3YW97_Branchiostoma_floridae
M9PLY3_Bemisia_tabaci
J9K1H6_Acyrthosiphon_pisum
E9GEP8_Daphnia_pulex
K7J6J1_Nasonia_vitripennis
A0A067QYK5_Zootermopsis_nevadensis
W4Z8N4_Strongylocentrotus_purpuratus
Q9SDV9_Brassica_juncea
S4VLP3_Rosa_roxburghii
M0TFZ9_Musa_acuminata
F6H4M6_Vitis_vinifera
A5B197_Vitis_vinifera
W2GZB4_Phytophthora_parasitica
V9FAD0_Phytophthora_parasitica
W2NFU8_Phytophthora_parasitica
W2LBX2_Phytophthora_parasitica
G4YHX9_Phytophthora_sojae
Fcan01_20277_PA
A0A0F8URM6_Aspergillus_ochraceoroseus
A0A0F8VRT7_Aspergillus_rambellii
R7T091_Dichomitus_squalens
U9T1Q9_Rhizophagus_irregularis
A0A015KYZ8_Rhizophagus_irregularis
0.4

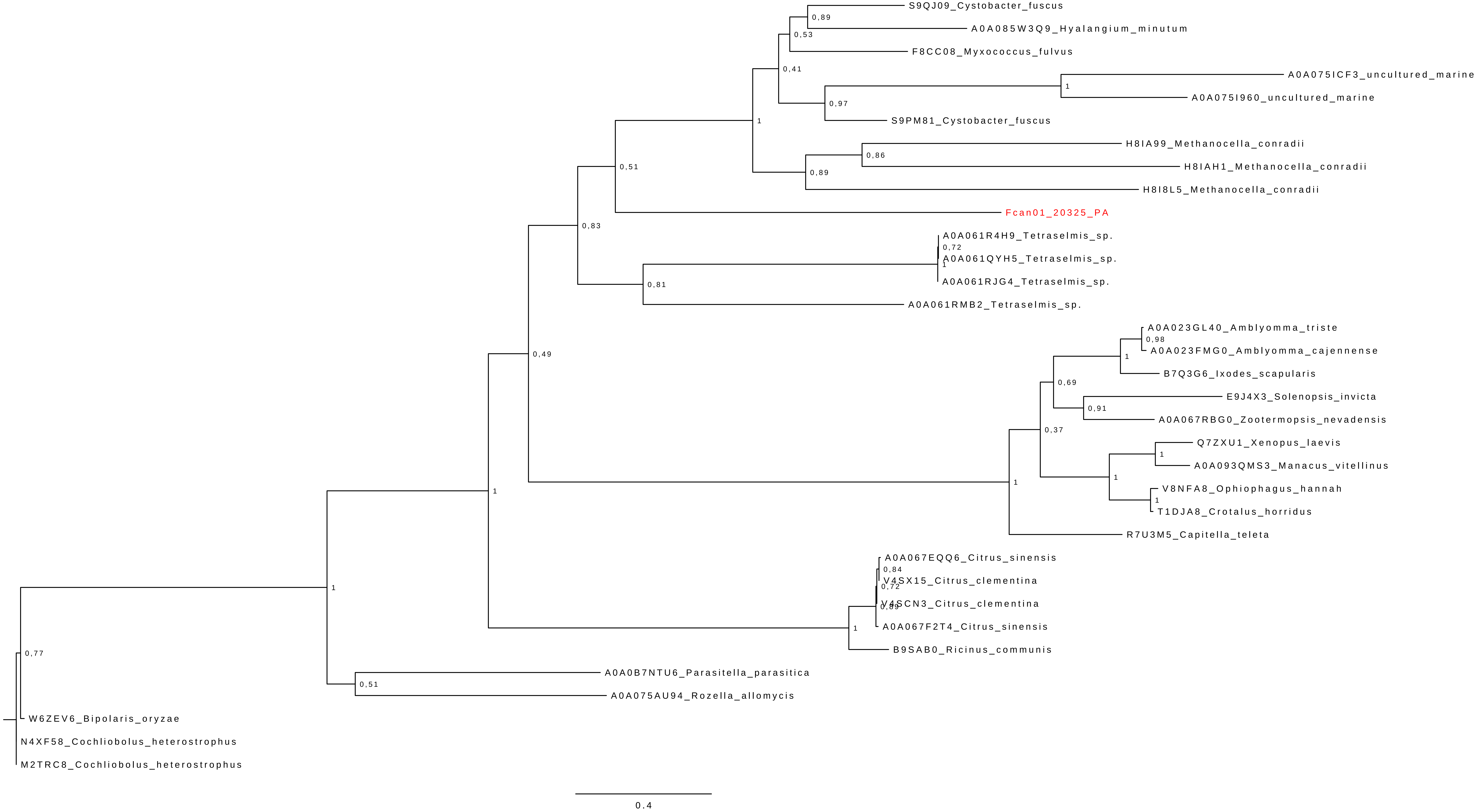
S9QJ09_Cystobacter_fuscus
0,89
A0A085W3Q9_Hyalangium_minutum
0,53
F8CC08_Myxococcus_fulvus
0,41
A0A075ICF3_uncultured_marine
1
A0A075I960_uncultured_marine
0,97
S9PM81_Cystobacter_fuscus
1
H8IA99_Methanocella_conradii
0,86
H8IAH1_Methanocella_conradii
0,89
H8I8L5_Methanocella_conradii
0,51
Fcan01_20325_PA
0,83
A0A061R4H9_Tetraselmis_sp.
0,72
A0A061QYH5_Tetraselmis_sp.
1
A0A061RJG4_Tetraselmis_sp.
0,81
A0A061RMB2_Tetraselmis_sp.
A0A023GL40_Amblyomma_triste
0,98
A0A023FMG0_Amblyomma_cajennense
1
B7Q3G6_Ixodes_scapularis
0,49
0,69
E9J4X3_Solenopsis_invicta
0,91
A0A067RBG0_Zootermopsis_nevadensis
0,37
Q7ZXU1_Xenopus_laevis
1
A0A093QMS3_Manacus_vitellinus
1
V8NFA8_Ophiophagus_hannah
1
1
T1DJA8_Crotalus_horridus
R7U3M5_Capitella_teleta
A0A067EQQ6_Citrus_sinensis
0,84
V4SX15_Citrus_clementina
0,72
V4SCN3_Citrus_clementina
A0A067F2T4_Citrus_sinensis
1
1
B9SAB0_Ricinus_communis
A0A0B7NTU6_Parasitella_parasitica
0,51
A0A075AU94_Rozella_allomycis
0,77
W6ZEV6_Bipolaris_oryzae
N4XF58_Cochliobolus_heterostrophus
M2TRC8_Cochliobolus_heterostrophus
0.4

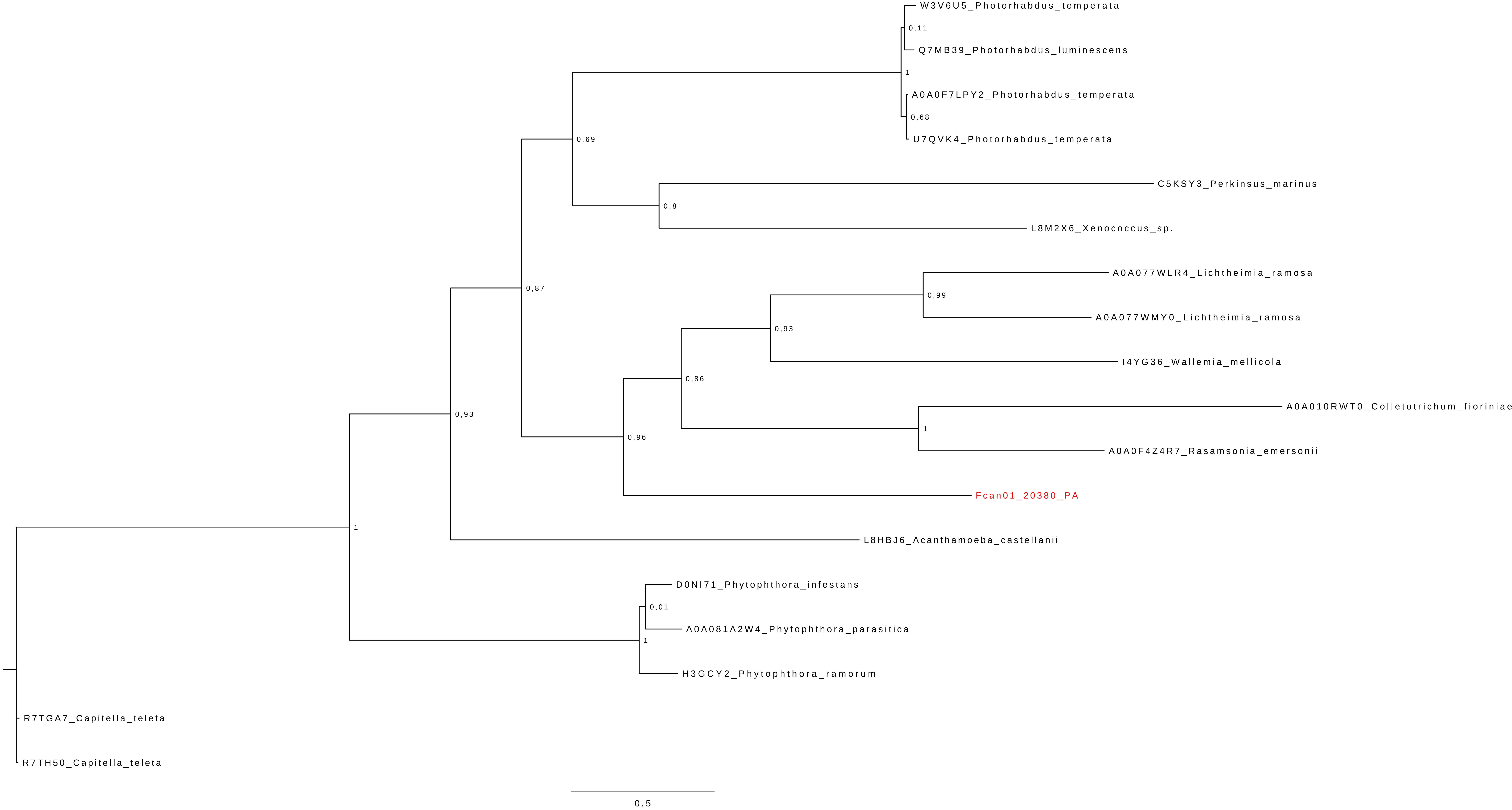
W3V6U5_Photorhabdus_temperata
0,11
Q7MB39_Photorhabdus_luminescens
1
A0A0F7LPY2_Photorhabdus_temperata
0,68
U7QVK4_Photorhabdus_temperata
0,69
C5KSY3_Perkinsus_marinus
0,8
L8M2X6_Xenococcus_sp.
A0A077WLR4_Lichtheimia_ramosa
0,99
A0A077WMY0_Lichtheimia_ramosa
0,87
0,93
I4YG36_Wallemia_mellicola
0,86
A0A010RWT0_Colletotrichum_fioriniae
0,93
1
0,96
A0A0F4Z4R7_Rasamsonia_emersonii
Fcan01_20380_PA
1
L8HBJ6_Acanthamoeba_castellanii
D0NI71_Phytophthora_infestans
0,01
A0A081A2W4_Phytophthora_parasitica
1
H3GCY2_Phytophthora_ramorum
R7TGA7_Capitella_teleta
R7TH50_Capitella_teleta
0.5

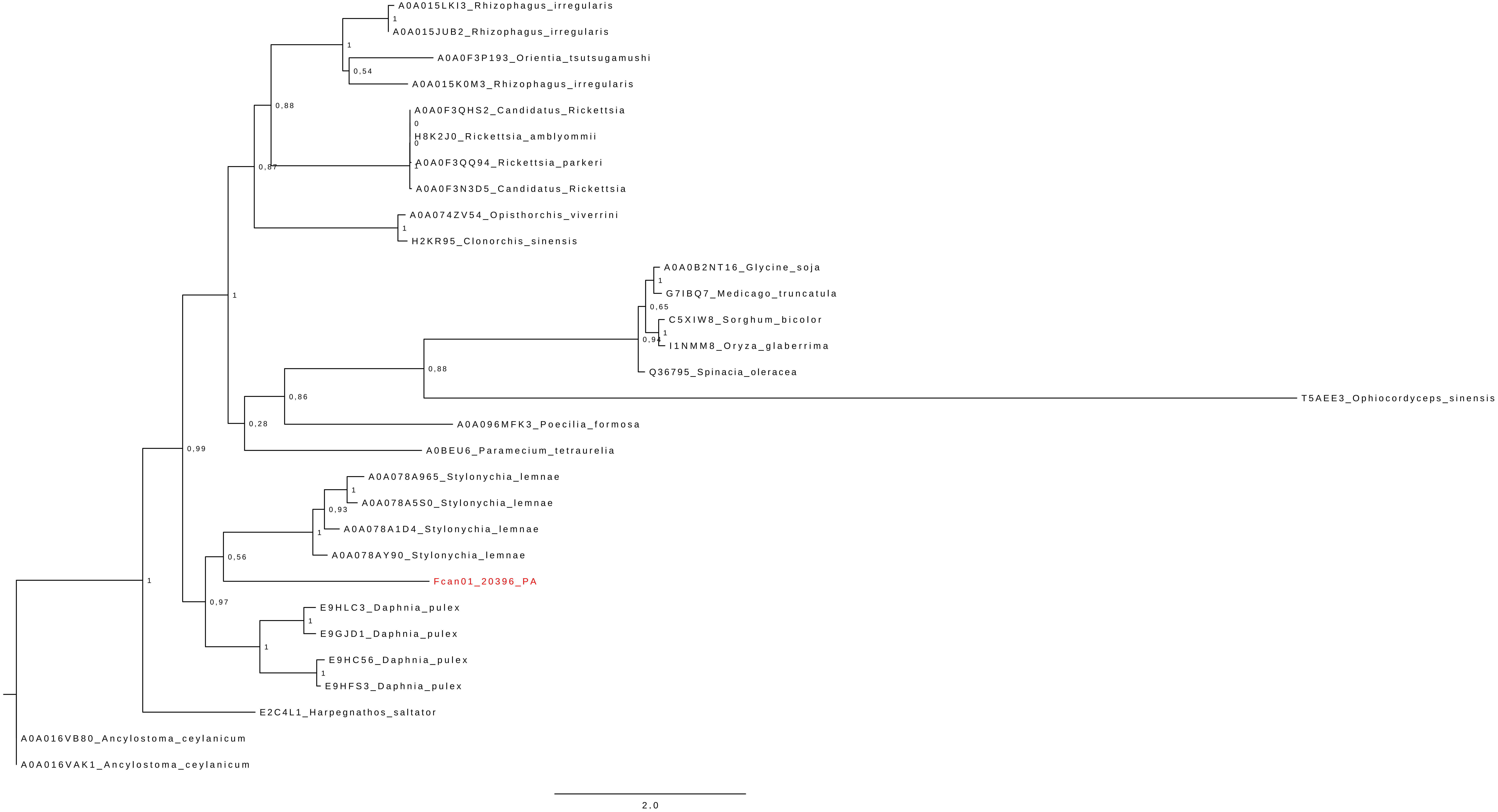
A0A015LKI3_Rhizophagus_irregularis
1
A0A015JUB2_Rhizophagus_irregularis
1
A0A0F3P193_Orientia_tsutsugamushi
0,54
A0A015K0M3_Rhizophagus_irregularis
0,88
A0A0F3QHS2_Candidatus_Rickettsia
0
H8K2J0_Rickettsia_amblyommii
0
A0A0F3QQ94_Rickettsia_parkeri
0,87
1
A0A0F3N3D5_Candidatus_Rickettsia
A0A074ZV54_Opisthorchis_viverrini
1
H2KR95_Clonorchis_sinensis
A0A0B2NT16_Glycine_soja
1
G7IBQ7_Medicago_truncatula
1
0,65
C5XIW8_Sorghum_bicolor
0,94
1
I1NMM8_Oryza_glaberrima
0,88
Q36795_Spinacia_oleracea
0,86
T5AEE3_Ophiocordyceps_sinensis
A0A096MFK3_Poecilia_formosa
0,28
A0BEU6_Paramecium_tetraurelia
0,99
A0A078A965_Stylonychia_lemnae
1
A0A078A5S0_Stylonychia_lemnae
0,93
A0A078A1D4_Stylonychia_lemnae
1
0,56
A0A078AY90_Stylonychia_lemnae
1
Fcan01_20396_PA
0,97
E9HLC3_Daphnia_pulex
1
E9GJD1_Daphnia_pulex
1
E9HC56_Daphnia_pulex
1
E9HFS3_Daphnia_pulex
E2C4L1_Harpegnathos_saltator
A0A016VB80_Ancylostoma_ceylanicum
A0A016VAK1_Ancylostoma_ceylanicum
2.0

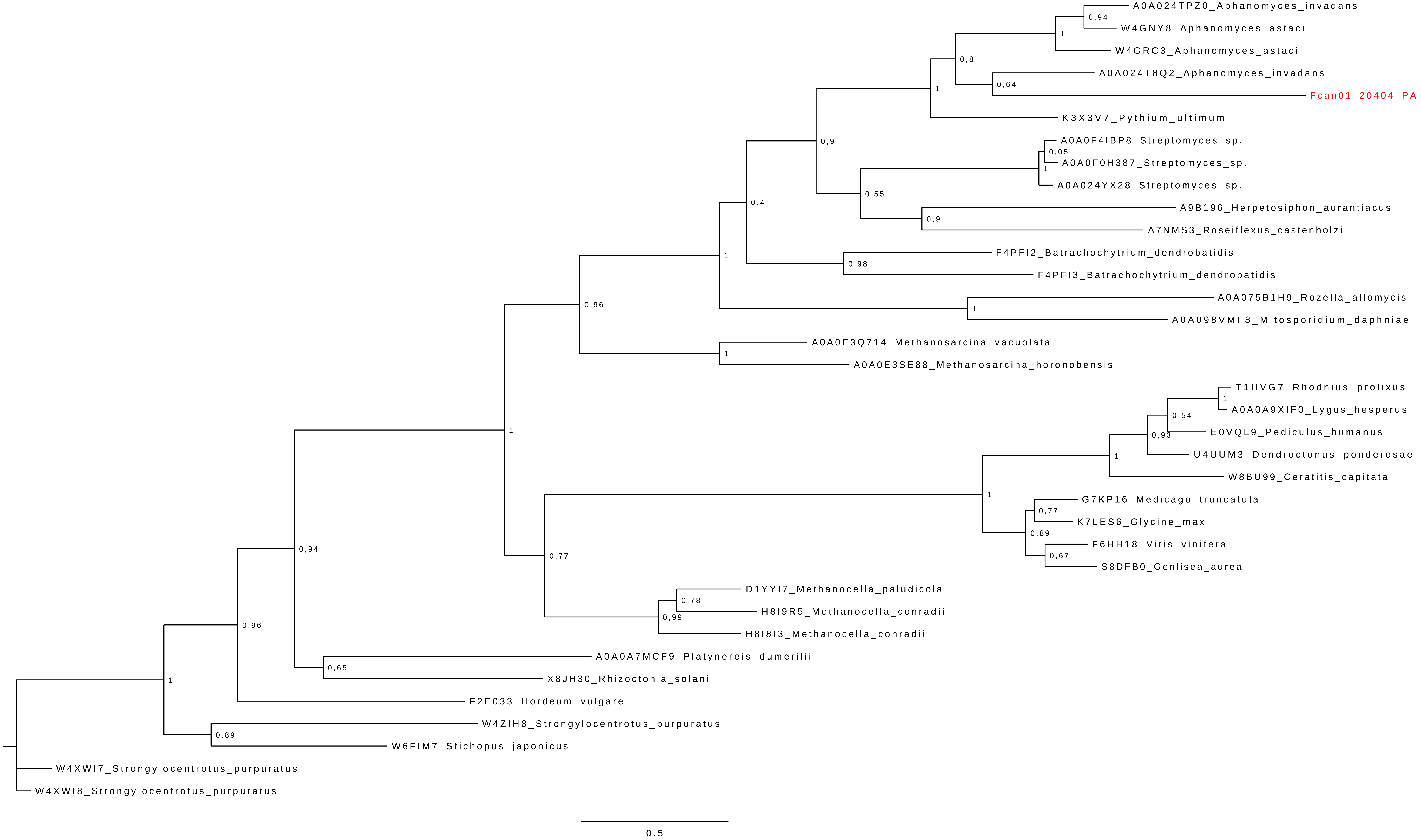
A0A024TPZ0_Aphanomyces_invadans
0,94
W4GNY8_Aphanomyces_astaci
1
W4GRC3_Aphanomyces_astaci
0,8
A0A024T8Q2_Aphanomyces_invadans
0,64
Fcan01_20404_PA
1
K3X3V7_Pythium_ultimum
0,9
A0A0F4IBP8_Streptomyces_sp.
0,05
A0A0F0H387_Streptomyces_sp.
1
A0A024YX28_Streptomyces_sp.
0,55
A9B196_Herpetosiphon_aurantiacus
0,9
A7NMS3_Roseiflexus_castenholzii
0,4
F4PFI2_Batrachochytrium_dendrobatidis
0,98
F4PFI3_Batrachochytrium_dendrobatidis
1
A0A075B1H9_Rozella_allomycis
1
A0A098VMF8_Mitosporidium_daphniae
0,96
A0A0E3Q714_Methanosarcina_vacuolata
1
A0A0E3SE88_Methanosarcina_horonobensis
T1HVG7_Rhodnius_prolixus
1
A0A0A9XIF0_Lygus_hesperus
0,54
E0VQL9_Pediculus_humanus
0,93
U4UUM3_Dendroctonus_ponderosae
1
W8BU99_Ceratitis_capitata
1
1
G7KP16_Medicago_truncatula
0,77
K7LES6_Glycine_max
0,89
F6HH18_Vitis_vinifera
0,67
S8DFB0_Genlisea_aurea
0,77
D1YYI7_Methanocella_paludicola
0,78
H8I9R5_Methanocella_conradii
0,99
H8I8I3_Methanocella_conradii
0,94
A0A0A7MCF9_Platynereis_dumerilii
0,65
X8JH30_Rhizoctonia_solani
0,96
F2E033_Hordeum_vulgare
1
W4ZIH8_Strongylocentrotus_purpuratus
0,89
W6FIM7_Stichopus_japonicus
W4XWI7_Strongylocentrotus_purpuratus
W4XWI8_Strongylocentrotus_purpuratus
0.5

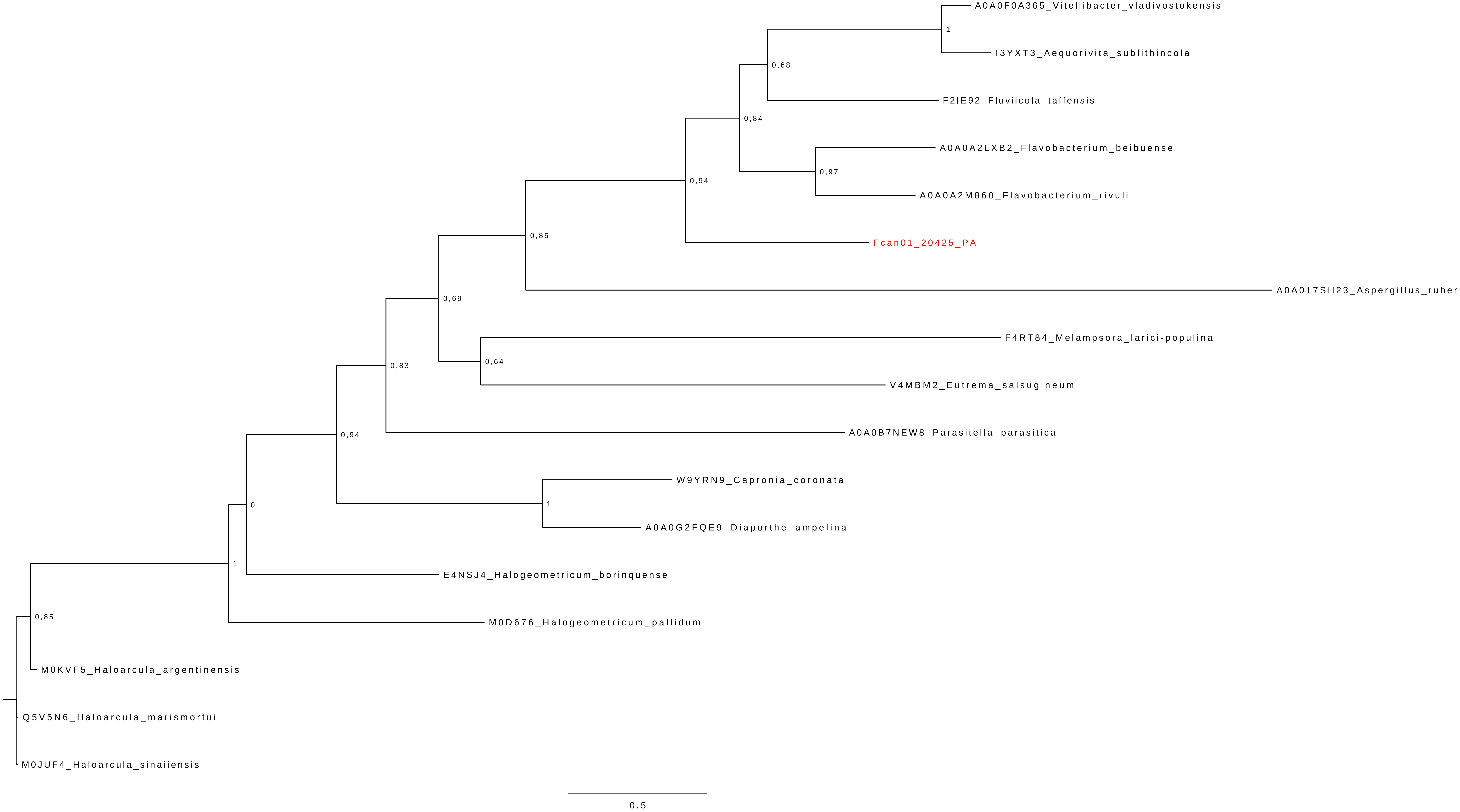

A0A0F0A365_Vitellibacter_vladivostokensis
1
I3YXT3_Aequorivita_sublithincola
0,68
F2IE92_Fluviicola_taffensis
0,84
A0A0A2LXB2_Flavobacterium_beibuense
0,97
A0A0A2M860_Flavobacterium_rivuli
0,94
Fcan01_20425_PA
0,85
A0A017SH23_Aspergillus_ruber
0,69
F4RT84_Melampsora_larici-populina
0,64
V4MBM2_Eutrema_salsugineum
0,83
A0A0B7NEW8_Parasitella_parasitica
0,94
W9YRN9_Capronia_coronata
1
A0A0G2FQE9_Diaporthe_ampelina
0
E4NSJ4_Halogeometricum_borinquense
1
M0D676_Halogeometricum_pallidum
0,85
M0KVF5_Haloarcula_argentinensis
Q5V5N6_Haloarcula_marismortui
M0JUF4_Haloarcula_sinaiiensis
0.5

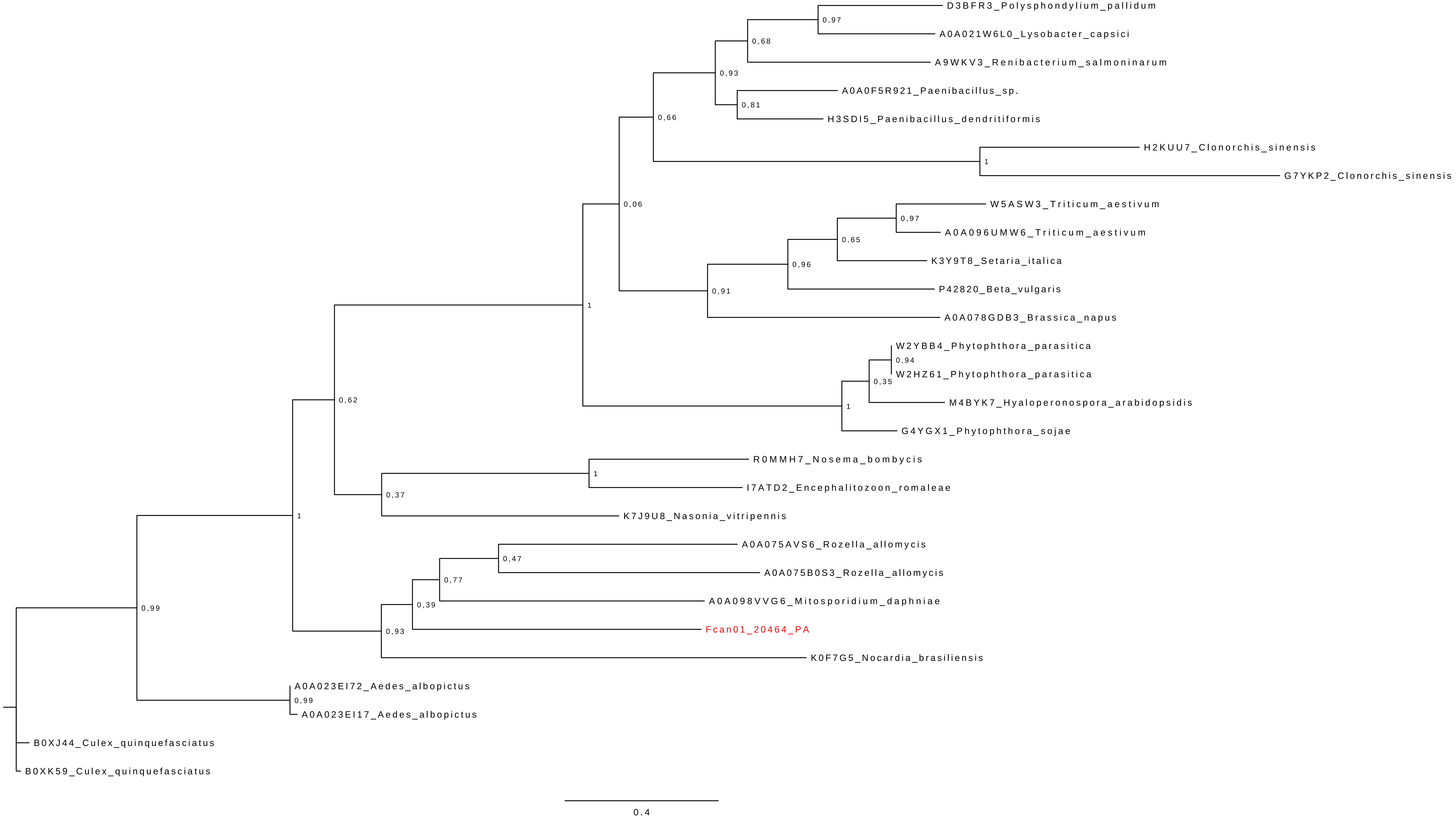
D3BFR3_Polysphondylium_pallidum
0,97
A0A021W6L0_Lysobacter_capsici
0,68
A9WKV3_Renibacterium_salmoninarum
0,93
A0A0F5R921_Paenibacillus_sp.
0,81
H3SDI5_Paenibacillus_dendritiformis
0,66
H2KUU7_Clonorchis_sinensis
1
G7YKP2_Clonorchis_sinensis
0,06
W5ASW3_Triticum_aestivum
0,97
A0A096UMW6_Triticum_aestivum
0,65
K3Y9T8_Setaria_italica
0,96
P42820_Beta_vulgaris
0,91
A0A078GDB3_Brassica_napus
1
W2YBB4_Phytophthora_parasitica
0,94
W2HZ61_Phytophthora_parasitica
0,35
M4BYK7_Hyaloperonospora_arabidopsidis
1
G4YGX1_Phytophthora_sojae
0,62
R0MMH7_Nosema_bombycis
1
I7ATD2_Encephalitozoon_romaleae
0,37
K7J9U8_Nasonia_vitripennis
1
A0A075AVS6_Rozella_allomycis
0,47
A0A075B0S3_Rozella_allomycis
0,77
A0A098VVG6_Mitosporidium_daphniae
0,39
Fcan01_20464_PA
0,93
K0F7G5_Nocardia_brasiliensis
0,99
A0A023EI72_Aedes_albopictus
0,99
A0A023EI17_Aedes_albopictus
B0XJ44_Culex_quinquefasciatus
B0XK59_Culex_quinquefasciatus
0.4

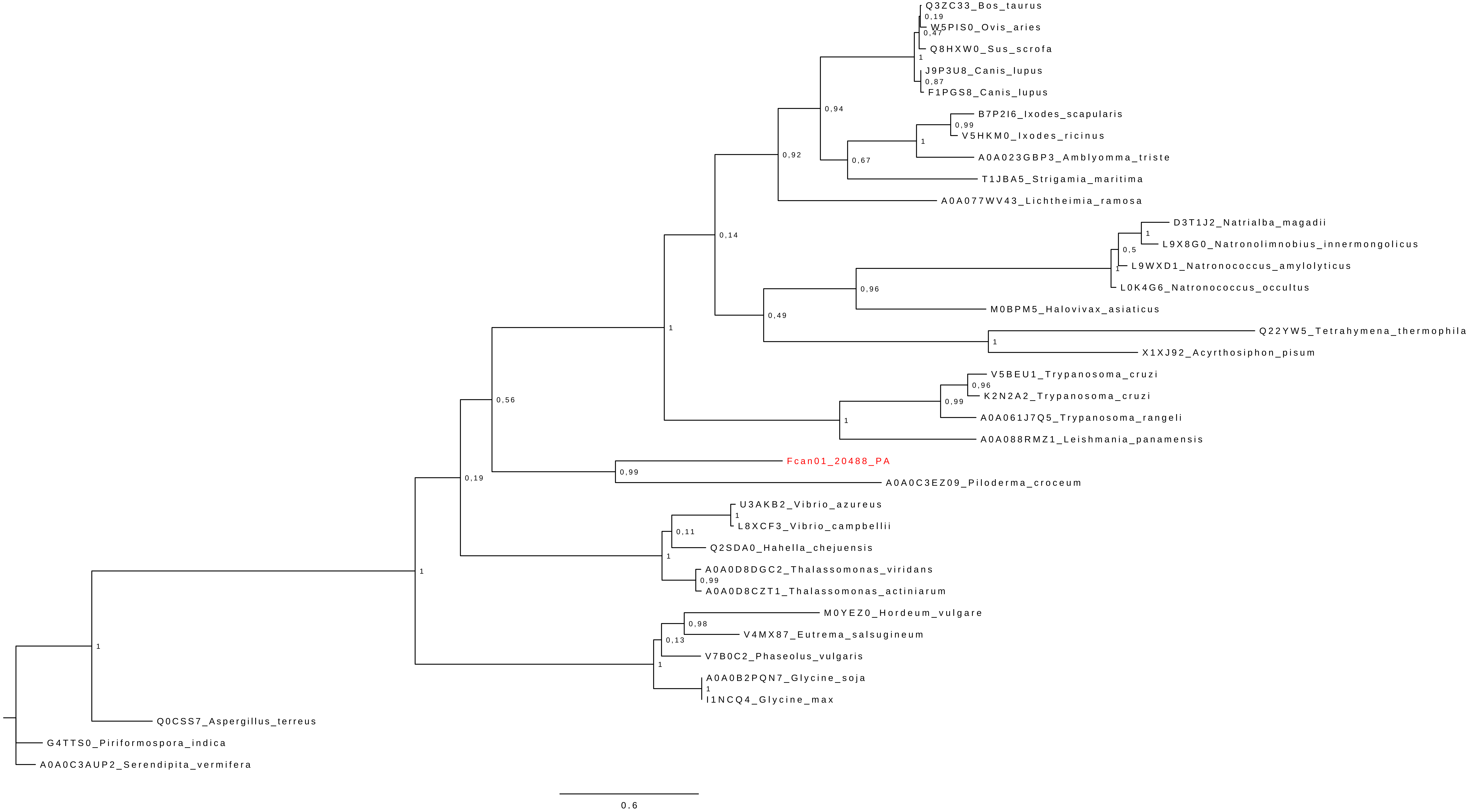
Q3ZC33_Bos_taurus
0,19
W5PIS0_Ovis_aries
0,47
Q8HXW0_Sus_scrofa
1
J9P3U8_Canis_lupus
0,87
F1PGS8_Canis_lupus
0,94
B7P2I6_Ixodes_scapularis
0,99
V5HKM0_Ixodes_ricinus
1
0,92
A0A023GBP3_Amblyomma_triste
0,67
T1JBA5_Strigamia_maritima
A0A077WV43_Lichtheimia_ramosa
D3T1J2_Natrialba_magadii
0,14
1
L9X8G0_Natronolimnobius_innermongolicus
0,5
L9WXD1_Natronococcus_amylolyticus
1
L0K4G6_Natronococcus_occultus
0,96
M0BPM5_Halovivax_asiaticus
0,49
1
Q22YW5_Tetrahymena_thermophila
1
X1XJ92_Acyrthosiphon_pisum
V5BEU1_Trypanosoma_cruzi
0,96
K2N2A2_Trypanosoma_cruzi
0,56
0,99
A0A061J7Q5_Trypanosoma_rangeli
1
A0A088RMZ1_Leishmania_panamensis
Fcan01_20488_PA
0,99
A0A0C3EZ09_Piloderma_croceum
0,19
U3AKB2_Vibrio_azureus
1
L8XCF3_Vibrio_campbellii
0,11
Q2SDA0_Hahella_chejuensis
1
1
A0A0D8DGC2_Thalassomonas_viridans
0,99
A0A0D8CZT1_Thalassomonas_actiniarum
M0YEZ0_Hordeum_vulgare
0,98
V4MX87_Eutrema_salsugineum
0,13
V7B0C2_Phaseolus_vulgaris
1
1
A0A0B2PQN7_Glycine_soja
1
I1NCQ4_Glycine_max
Q0CSS7_Aspergillus_terreus
G4TTS0_Piriformospora_indica
A0A0C3AUP2_Serendipita_vermifera
0.6

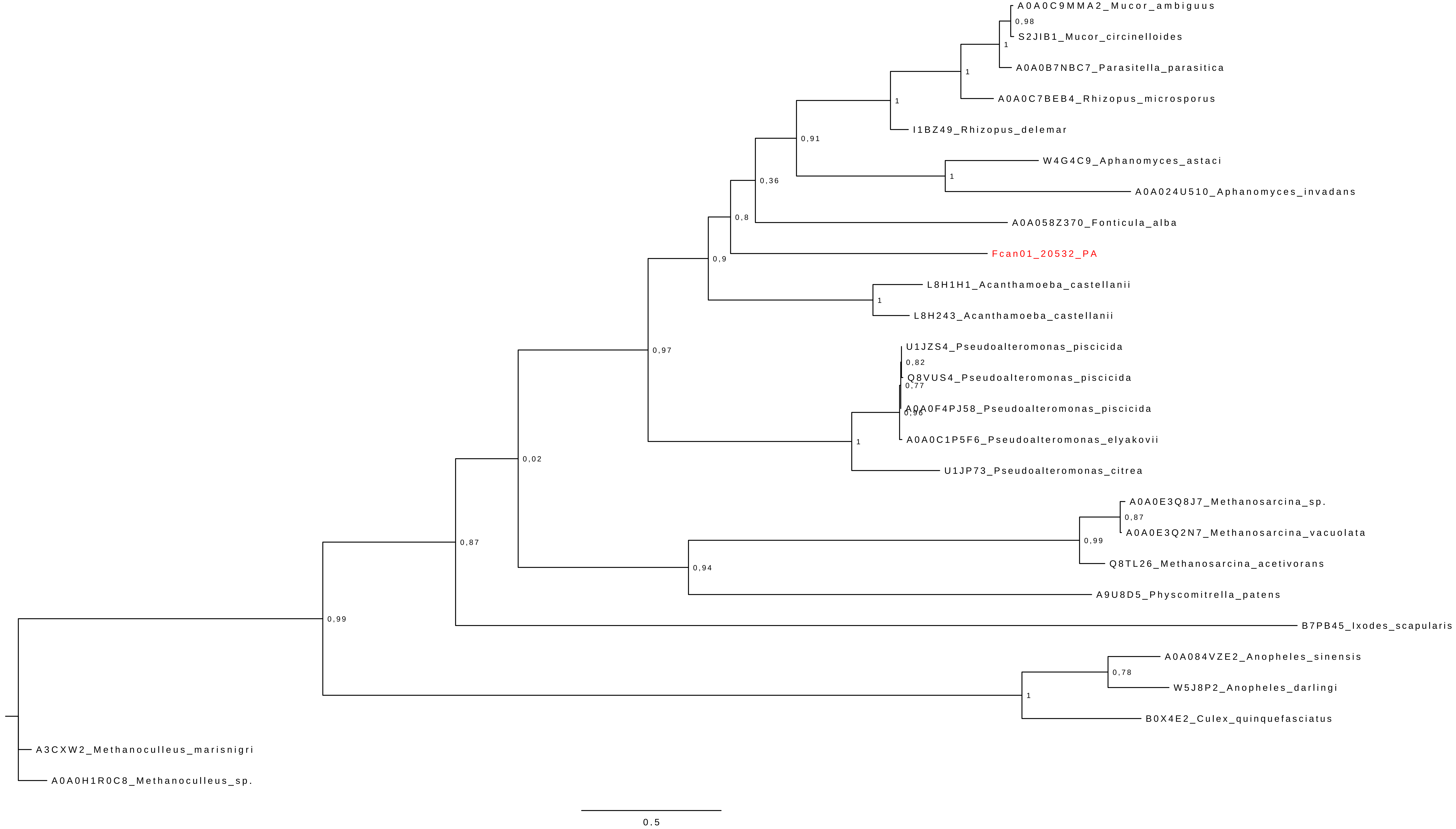
A0A0C9MMA2_Mucor_ambiguus
0,98
S2JIB1_Mucor_circinelloides
1
A0A0B7NBC7_Parasitella_parasitica
1
A0A0C7BEB4_Rhizopus_microsporus
1
I1BZ49_Rhizopus_delemar
0,91
W4G4C9_Aphanomyces_astaci
1
A0A024U510_Aphanomyces_invadans
0,36
A0A058Z370_Fonticula_alba
0,8
Fcan01_20532_PA
0,9
L8H1H1_Acanthamoeba_castellanii
1
L8H243_Acanthamoeba_castellanii
0,97
U1JZS4_Pseudoalteromonas_piscicida
0,82
Q8VUS4_Pseudoalteromonas_piscicida
0,77
A0A0F4PJ58_Pseudoalteromonas_piscicida
0,96
A0A0C1P5F6_Pseudoalteromonas_elyakovii
1
U1JP73_Pseudoalteromonas_citrea
0,02
A0A0E3Q8J7_Methanosarcina_sp.
0,87
A0A0E3Q2N7_Methanosarcina_vacuolata
0,99
Q8TL26_Methanosarcina_acetivorans
0,94
A9U8D5_Physcomitrella_patens
0,87
B7PB45_Ixodes_scapularis
0,99
A0A084VZE2_Anopheles_sinensis
0,78
W5J8P2_Anopheles_darlingi
1
B0X4E2_Culex_quinquefasciatus
A3CXW2_Methanoculleus_marisnigri
A0A0H1R0C8_Methanoculleus_sp.
0.5

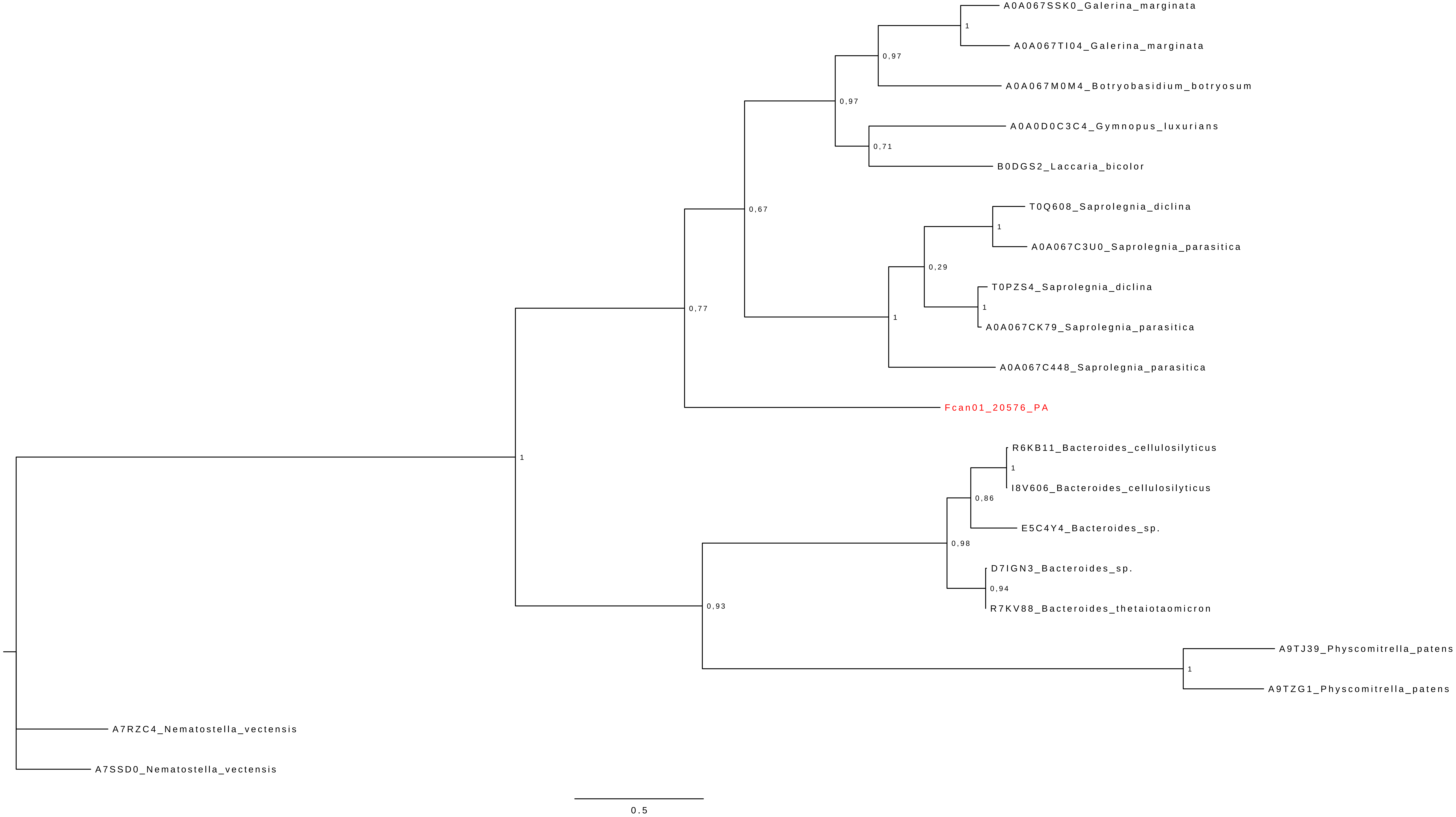
A0A067SSK0_Galerina_marginata
A0A067TI04_Galerina_marginata
A0A067M0M4_Botryobasidium_botryosum
A0A0D0C3C4_Gymnopus_luxurians
B0DGS2_Laccaria_bicolor
T0Q608_Saprolegnia_diclina
A0A067C3U0_Saprolegnia_parasitica
T0PZS4_Saprolegnia_diclina
A0A067CK79_Saprolegnia_parasitica
A0A067C448_Saprolegnia_parasitica
Fcan01_20576_PA
R6KB11_Bacteroides_cellulosilyticus
I8V606_Bacteroides_cellulosilyticus
E5C4Y4_Bacteroides_sp.
D7IGN3_Bacteroides_sp.
R7KV88_Bacteroides_thetaiotaomicron
A9TJ39_Physcomitrella_patens
A9TZG1_Physcomitrella_patens
A7RZC4_Nematostella_vectensis
A7SSD0_Nematostella_vectensis
1
0,97
0,97
0,71
0,67
1
0,29
1
1
0,77
1
1
0,86
0,98
0,94
0,93
1
0.5

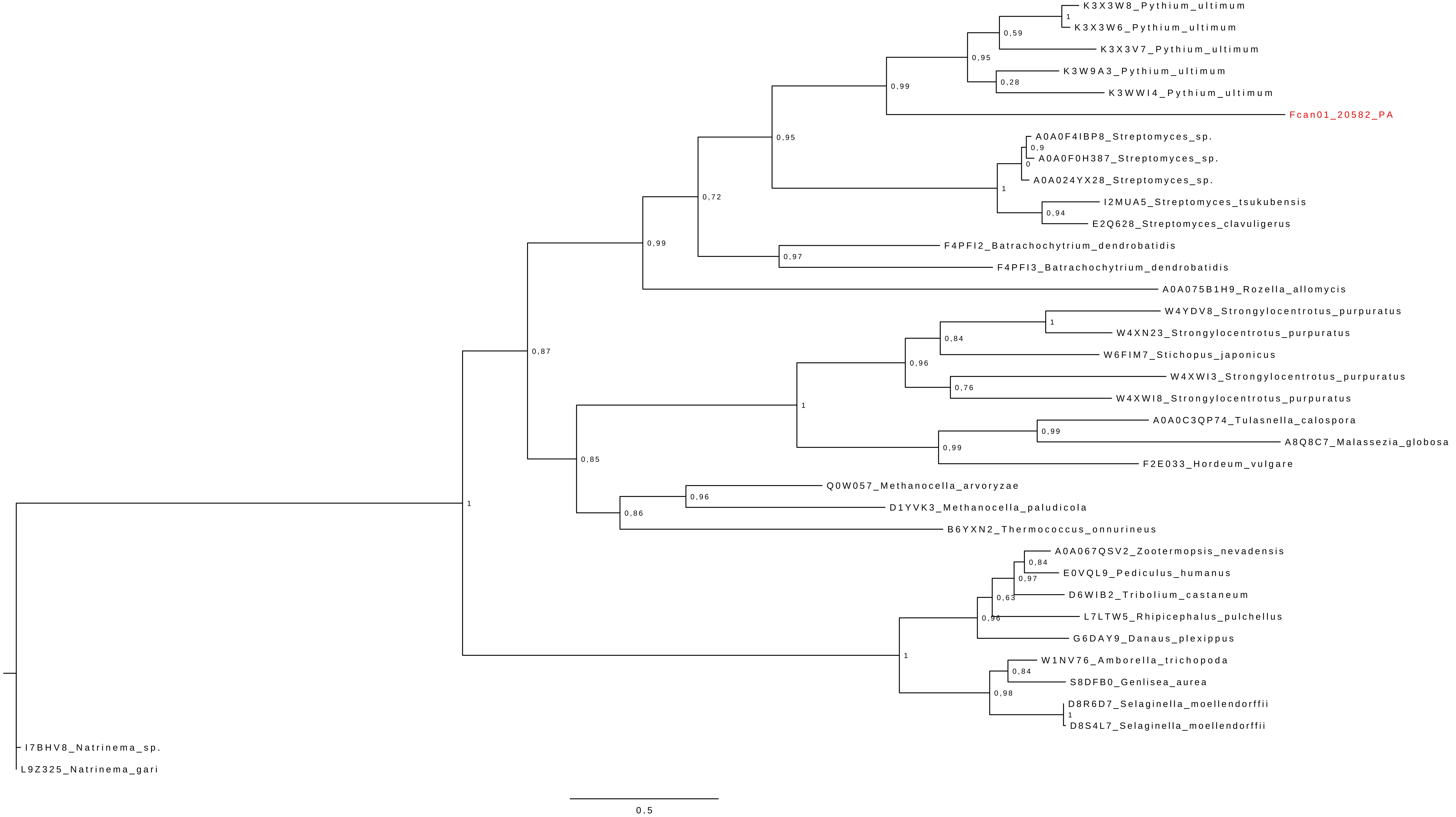

K3X3W8_Pythium_ultimum
K3X3W6_Pythium_ultimum
K3X3V7_Pythium_ultimum
K3W9A3_Pythium_ultimum
K3WWI4_Pythium_ultimum
Fcan01_20582_PA
A0A0F4IBP8_Streptomyces_sp.
A0A0F0H387_Streptomyces_sp.
A0A024YX28_Streptomyces_sp.
I2MUA5_Streptomyces_tsukubensis
E2Q628_Streptomyces_clavuligerus
F4PFI2_Batrachochytrium_dendrobatidis
F4PFI3_Batrachochytrium_dendrobatidis
A0A075B1H9_Rozella_allomycis
W4YDV8_Strongylocentrotus_purpuratus
W4XN23_Strongylocentrotus_purpuratus
W6FIM7_Stichopus_japonicus
W4XWI3_Strongylocentrotus_purpuratus
W4XWI8_Strongylocentrotus_purpuratus
A0A0C3QP74_Tulasnella_calospora
A8Q8C7_Malassezia_globosa
F2E033_Hordeum_vulgare
Q0W057_Methanocella_arvoryzae
D1YVK3_Methanocella_paludicola
B6YXN2_Thermococcus_onnurineus
A0A067QSV2_Zootermopsis_nevadensis
E0VQL9_Pediculus_humanus
D6WIB2_Tribolium_castaneum
L7LTW5_Rhipicephalus_pulchellus
G6DAY9_Danaus_plexippus
W1NV76_Amborella_trichopoda
S8DFB0_Genlisea_aurea
D8R6D7_Selaginella_moellendorffii
D8S4L7_Selaginella_moellendorffii
I7BHV8_Natrinema_sp.
L9Z325_Natrinema_gari
0.5

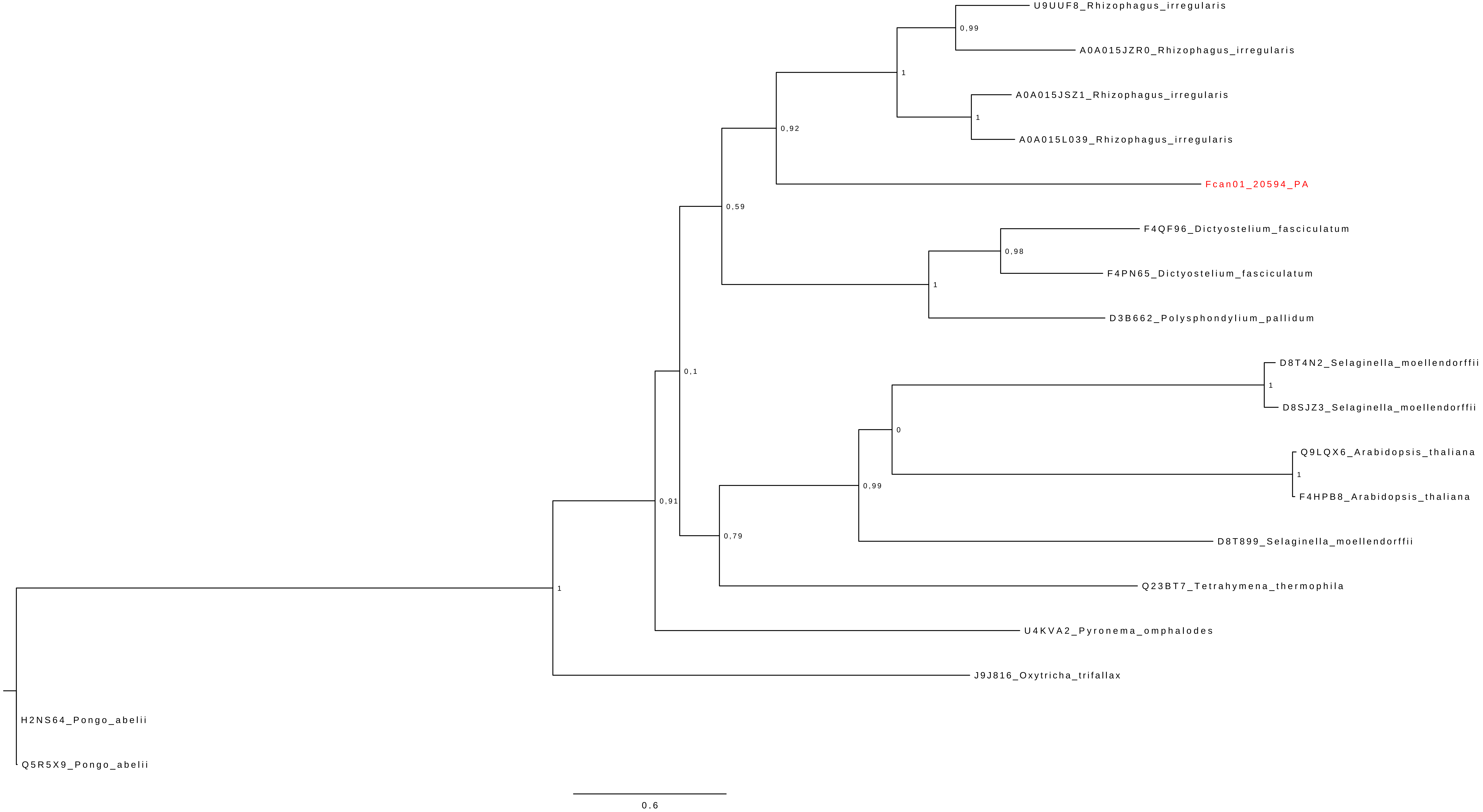
U9UUF8_Rhizophagus_irregularis
0,99
A0A015JZR0_Rhizophagus_irregularis
1
A0A015JSZ1_Rhizophagus_irregularis
1
A0A015L039_Rhizophagus_irregularis
0,92
Fcan01_20594_PA
0,59
F4QF96_Dictyostelium_fasciculatum
0,98
F4PN65_Dictyostelium_fasciculatum
1
D3B662_Polysphondylium_pallidum
D8T4N2_Selaginella_moellendorffii
0,1
1
D8SJZ3_Selaginella_moellendorffii
0
Q9LQX6_Arabidopsis_thaliana
1
F4HPB8_Arabidopsis_thaliana
0,99
0,91
D8T899_Selaginella_moellendorffii
0,79
Q23BT7_Tetrahymena_thermophila
1
U4KVA2_Pyronema_omphalodes
J9J816_Oxytricha_trifallax
H2NS64_Pongo_abelii
Q5R5X9_Pongo_abelii
0.6

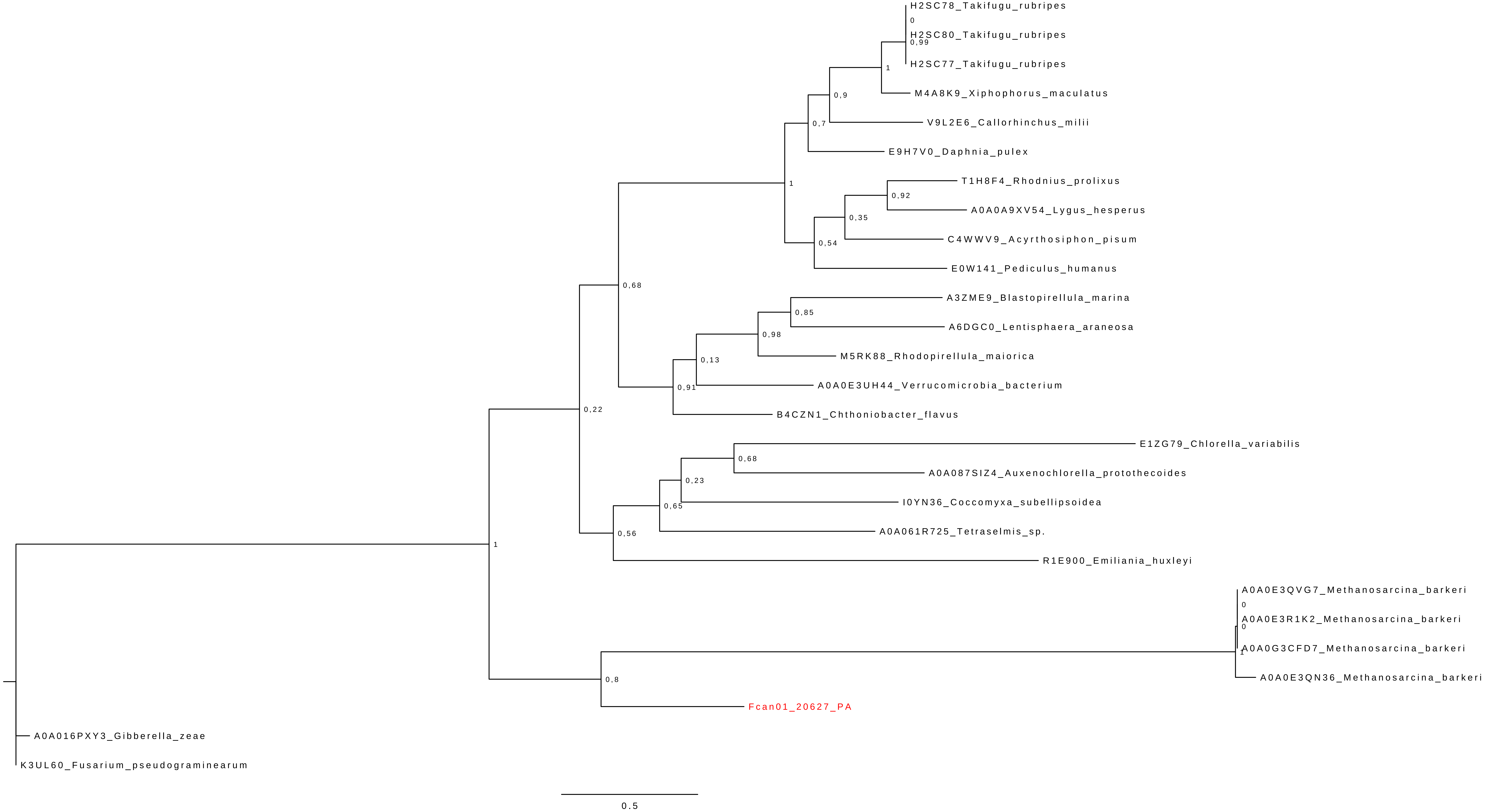

H2SC78_Takifugu_rubripes
0
H2SC80_Takifugu_rubripes
0,99
H2SC77_Takifugu_rubripes
1
M4A8K9_Xiphophorus_maculatus
0,9
V9L2E6_Callorhinchus_milii
0,7
E9H7V0_Daphnia_pulex
1
T1H8F4_Rhodnius_prolixus
0,92
A0A0A9XV54_Lygus_hesperus
0,35
C4WWV9_Acyrthosiphon_pisum
0,54
E0W141_Pediculus_humanus
0,68
A3ZME9_Blastopirellula_marina
0,85
A6DGC0_Lentisphaera_araneosa
0,98
M5RK88_Rhodopirellula_maiorica
0,13
A0A0E3UH44_Verrucomicrobia_bacterium
0,91
B4CZN1_Chthoniobacter_flavus
0,22
E1ZG79_Chlorella_variabilis
0,68
A0A087SIZ4_Auxenochlorella_protothecoides
0,23
I0YN36_Coccomyxa_subellipsoidea
0,65
A0A061R725_Tetraselmis_sp.
0,56
R1E900_Emiliania_huxleyi
1
A0A0E3QVG7_Methanosarcina_barkeri
0
A0A0E3R1K2_Methanosarcina_barkeri
0
A0A0G3CFD7_Methanosarcina_barkeri
1
A0A0E3QN36_Methanosarcina_barkeri
0,8
Fcan01_20627_PA
A0A016PXY3_Gibberella_zeae
K3UL60_Fusarium_pseudograminearum
0.5

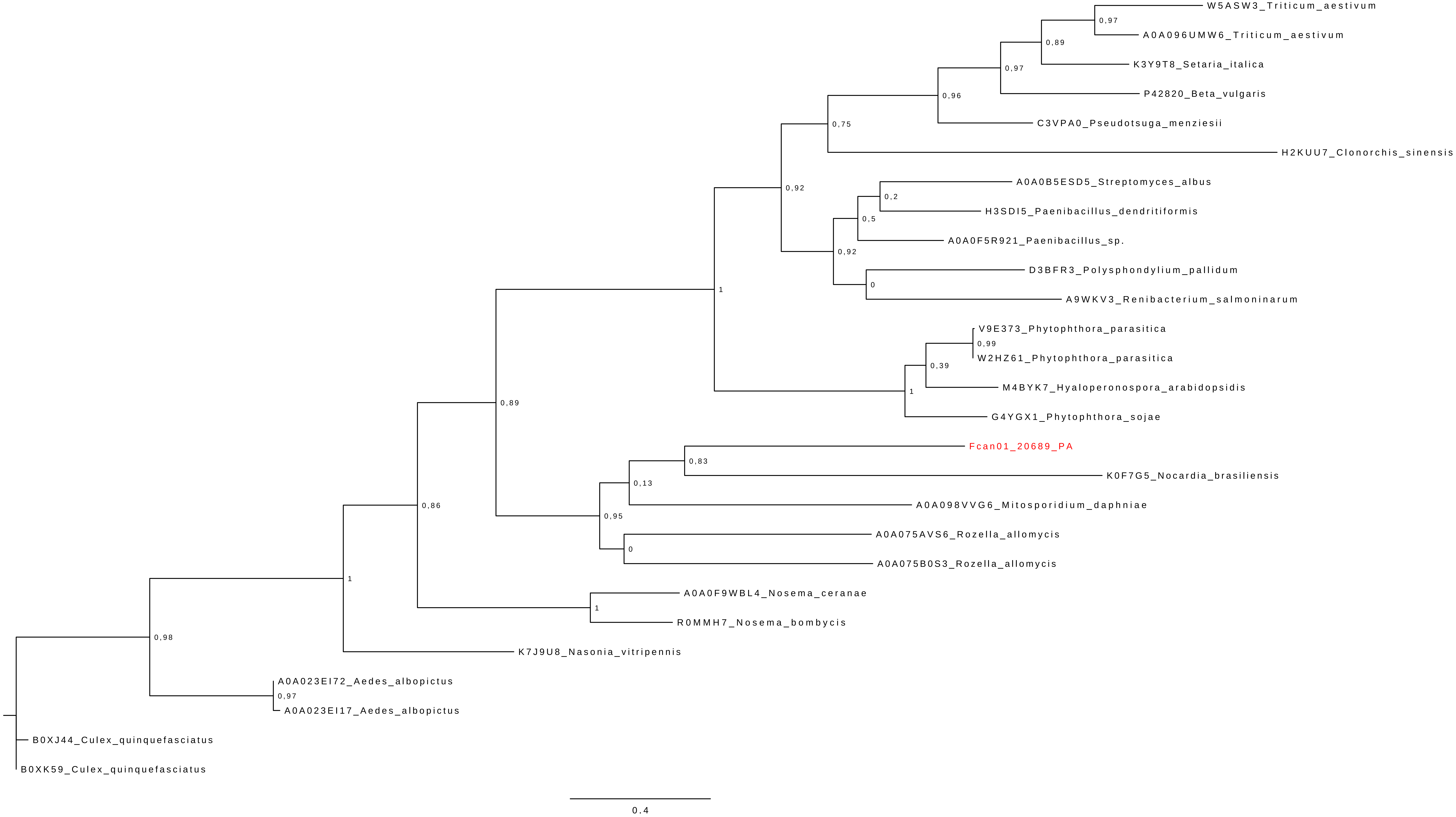

W5ASW3_Triticum_aestivum
0,97
A0A096UMW6_Triticum_aestivum
0,89
K3Y9T8_Setaria_italica
0,97
P42820_Beta_vulgaris
0,96
C3VPA0_Pseudotsuga_menziesii
0,75
H2KUU7_Clonorchis_sinensis
A0A0B5ESD5_Streptomyces_albus
0,92
0,2
H3SDI5_Paenibacillus_dendritiformis
0,5
A0A0F5R921_Paenibacillus_sp.
0,92
D3BFR3_Polysphondylium_pallidum
1
0
A9WKV3_Renibacterium_salmoninarum
V9E373_Phytophthora_parasitica
0,99
W2HZ61_Phytophthora_parasitica
0,39
M4BYK7_Hyaloperonospora_arabidopsidis
1
G4YGX1_Phytophthora_sojae
0,89
Fcan01_20689_PA
0,83
K0F7G5_Nocardia_brasiliensis
0,13
A0A098VVG6_Mitosporidium_daphniae
0,86
0,95
A0A075AVS6_Rozella_allomycis
0
A0A075B0S3_Rozella_allomycis
1
A0A0F9WBL4_Nosema_ceranae
1
R0MMH7_Nosema_bombycis
0,98
K7J9U8_Nasonia_vitripennis
A0A023EI72_Aedes_albopictus
0,97
A0A023EI17_Aedes_albopictus
B0XJ44_Culex_quinquefasciatus
B0XK59_Culex_quinquefasciatus
0.4

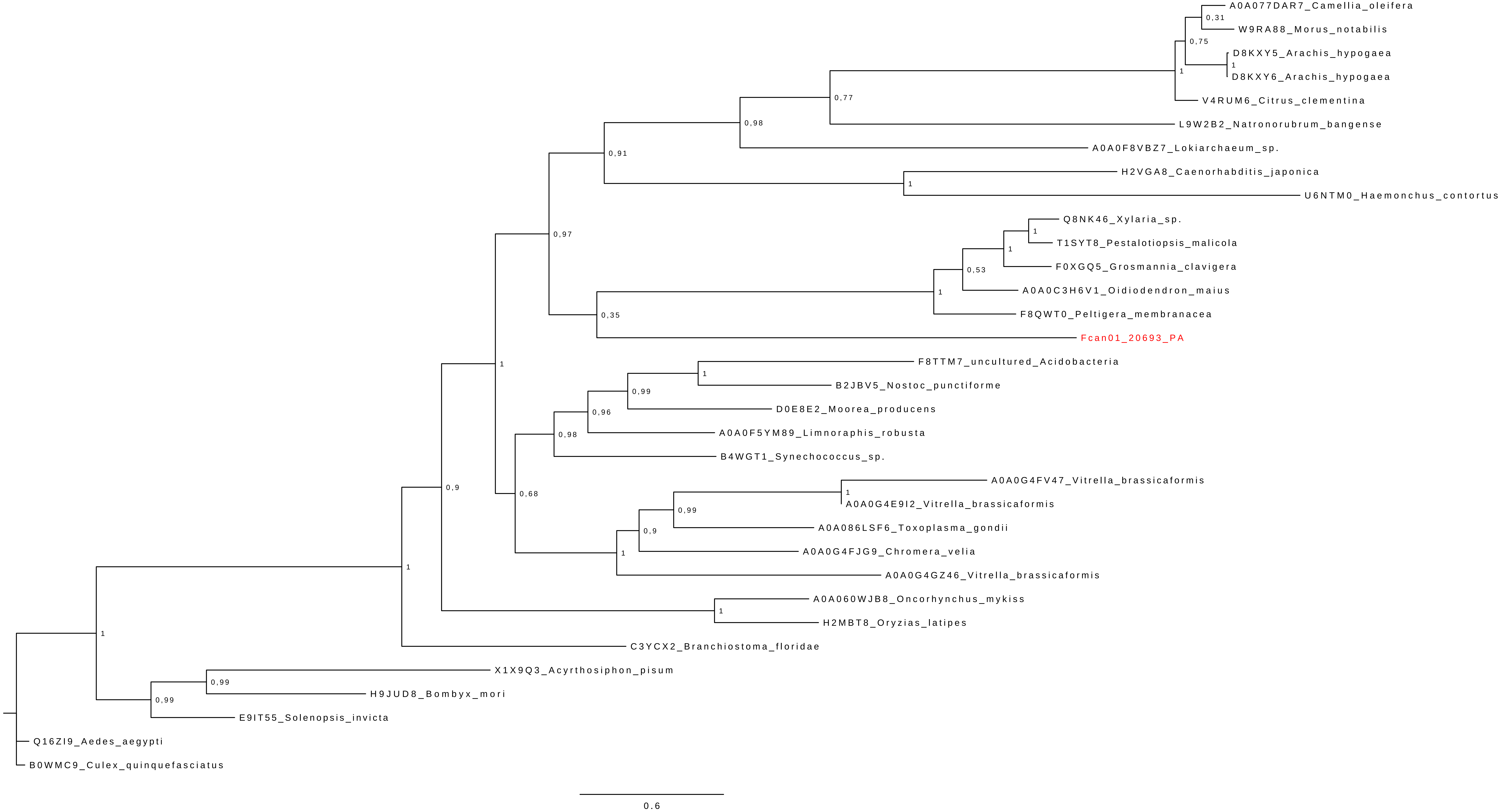
A0A077DAR7_Camellia_oleifera
0,31
W9RA88_Morus_notabilis
0,75
D8KXY5_Arachis_hypogaea
1
D8KXY6_Arachis_hypogaea
1
V4RUM6_Citrus_clementina
0,77
L9W2B2_Natronorubrum_bangense
0,98
A0A0F8VBZ7_Lokiarchaeum_sp.
0,91
H2VGA8_Caenorhabditis_japonica
1
U6NTM0_Haemonchus_contortus
Q8NK46_Xylaria_sp.
1
T1SYT8_Pestalotiopsis_malicola
1
F0XGQ5_Grosmannia_clavigera
0,53
A0A0C3H6V1_Oidiodendron_maius
1
F8QWT0_Peltigera_membranacea
0,35
Fcan01_20693_PA
0,97
F8TTM7_uncultured_Acidobacteria
1
B2JBV5_Nostoc_punctiforme
0,99
D0E8E2_Moorea_producens
0,96
A0A0F5YM89_Limnoraphis_robusta
0,98
B4WGT1_Synechococcus_sp.
1
A0A0G4FV47_Vitrella_brassicaformis
1
A0A0G4E9I2_Vitrella_brassicaformis
0,99
A0A086LSF6_Toxoplasma_gondii
0,9
A0A0G4FJG9_Chromera_velia
1
A0A0G4GZ46_Vitrella_brassicaformis
0,68
0,9
A0A060WJB8_Oncorhynchus_mykiss
1
H2MBT8_Oryzias_latipes
1
C3YCX2_Branchiostoma_floridae
1
X1X9Q3_Acyrthosiphon_pisum
0,99
H9JUD8_Bombyx_mori
0,99
E9IT55_Solenopsis_invicta
Q16ZI9_Aedes_aegypti
B0WMC9_Culex_quinquefasciatus
0.6

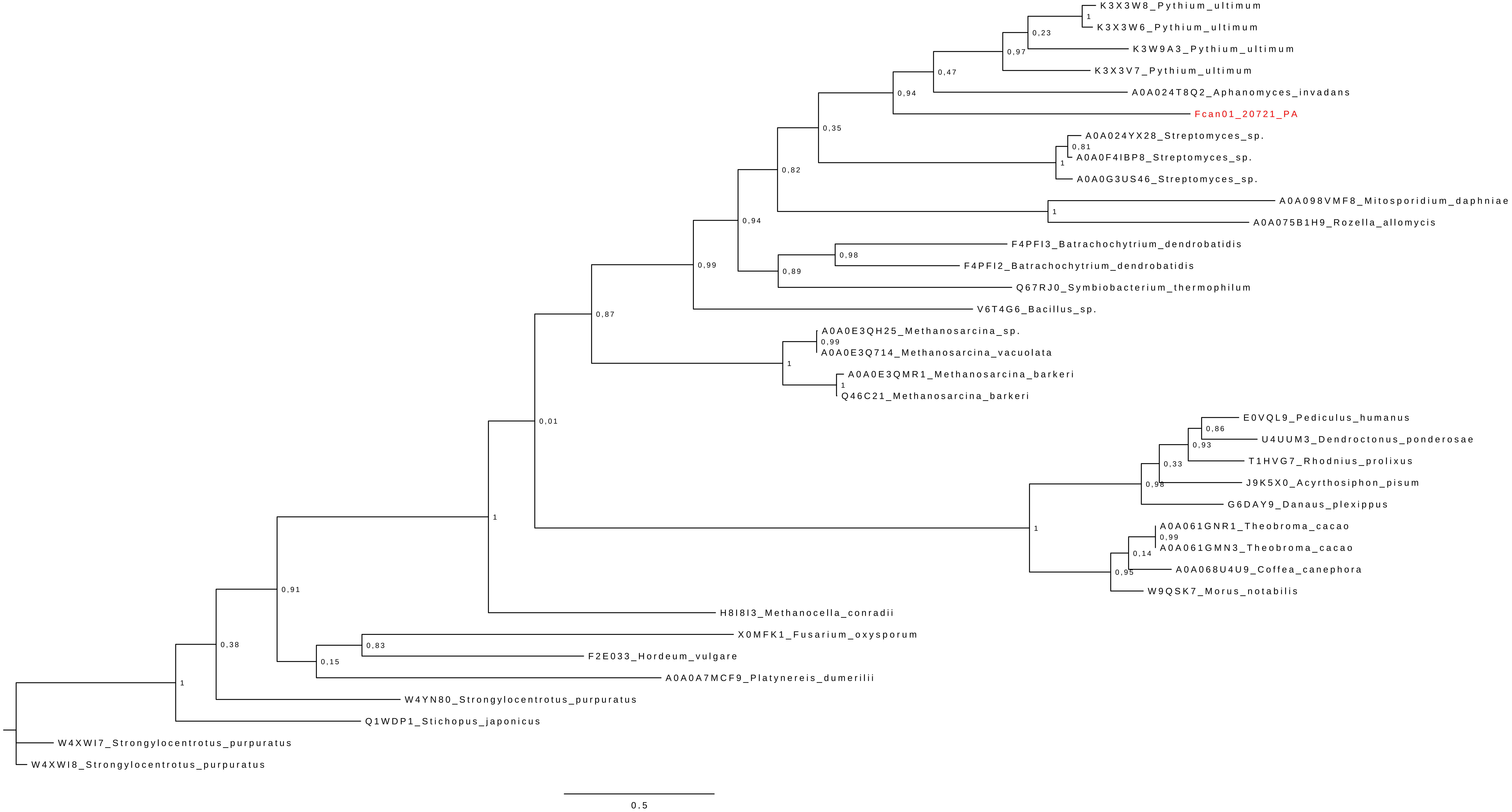
K3X3W8_Pythium_ultimum
K3X3W6_Pythium_ultimum
K3W9A3_Pythium_ultimum
K3X3V7_Pythium_ultimum
A0A024T8Q2_Aphanomyces_invadans
Fcan01_20721_PA
A0A024YX28_Streptomyces_sp.
A0A0F4IBP8_Streptomyces_sp.
A0A0G3US46_Streptomyces_sp.
A0A098VMF8_Mitosporidium_daphniae
A0A075B1H9_Rozella_allomycis
F4PFI3_Batrachochytrium_dendrobatidis
F4PFI2_Batrachochytrium_dendrobatidis
Q67RJ0_Symbiobacterium_thermophilum
V6T4G6_Bacillus_sp.
A0A0E3QH25_Methanosarcina_sp.
A0A0E3Q714_Methanosarcina_vacuolata
A0A0E3QMR1_Methanosarcina_barkeri
Q46C21_Methanosarcina_barkeri
E0VQL9_Pediculus_humanus
U4UUM3_Dendroctonus_ponderosae
T1HVG7_Rhodnius_prolixus
J9K5X0_Acyrthosiphon_pisum
G6DAY9_Danaus_plexippus
A0A061GNR1_Theobroma_cacao
A0A061GMN3_Theobroma_cacao
A0A068U4U9_Coffea_canephora
W9QSK7_Morus_notabilis
H8I8I3_Methanocella_conradii
X0MFK1_Fusarium_oxysporum
F2E033_Hordeum_vulgare
A0A0A7MCF9_Platynereis_dumerilii
W4YN80_Strongylocentrotus_purpuratus
Q1WDP1_Stichopus_japonicus
W4XWI7_Strongylocentrotus_purpuratus
W4XWI8_Strongylocentrotus_purpuratus
0.5

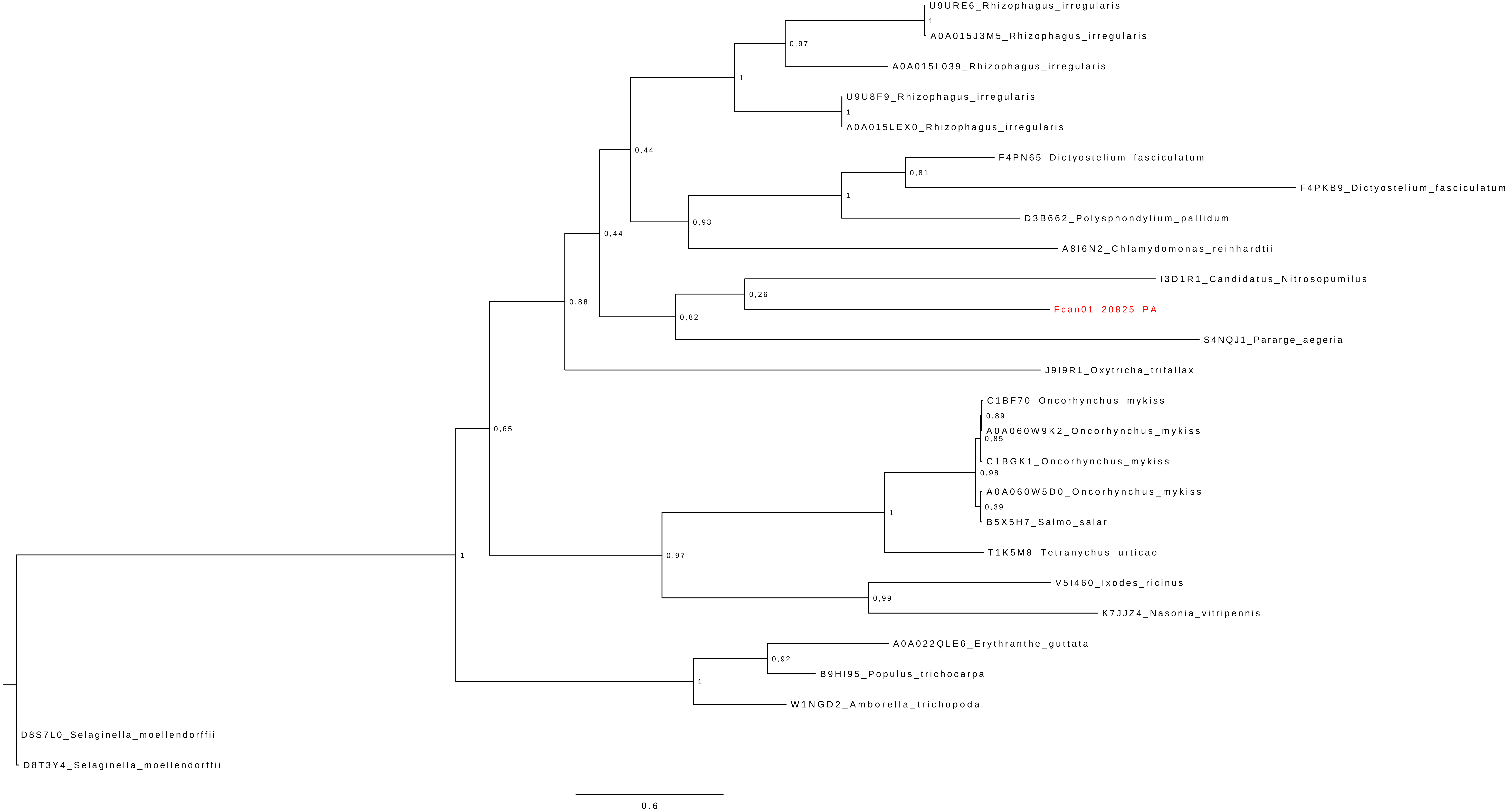
U9URE6_Rhizophagus_irregularis
1
A0A015J3M5_Rhizophagus_irregularis
0,97
A0A015L039_Rhizophagus_irregularis
1
U9U8F9_Rhizophagus_irregularis
1
A0A015LEX0_Rhizophagus_irregularis
0,44
F4PN65_Dictyostelium_fasciculatum
0,81
F4PKB9_Dictyostelium_fasciculatum
1
D3B662_Polysphondylium_pallidum
0,93
A8I6N2_Chlamydomonas_reinhardtii
0,44
I3D1R1_Candidatus_Nitrosopumilus
0,26
Fcan01_20825_PA
0,82
S4NQJ1_Pararge_aegeria
0,88
J9I9R1_Oxytricha_trifallax
C1BF70_Oncorhynchus_mykiss
0,89
A0A060W9K2_Oncorhynchus_mykiss
0,85
C1BGK1_Oncorhynchus_mykiss
0,98
A0A060W5D0_Oncorhynchus_mykiss
0,39
B5X5H7_Salmo_salar
1
T1K5M8_Tetranychus_urticae
0,65
0,97
V5I460_Ixodes_ricinus
0,99
K7JJZ4_Nasonia_vitripennis
1
A0A022QLE6_Erythranthe_guttata
0,92
B9HI95_Populus_trichocarpa
1
W1NGD2_Amborella_trichopoda
D8S7L0_Selaginella_moellendorffii
D8T3Y4_Selaginella_moellendorffii
0.6

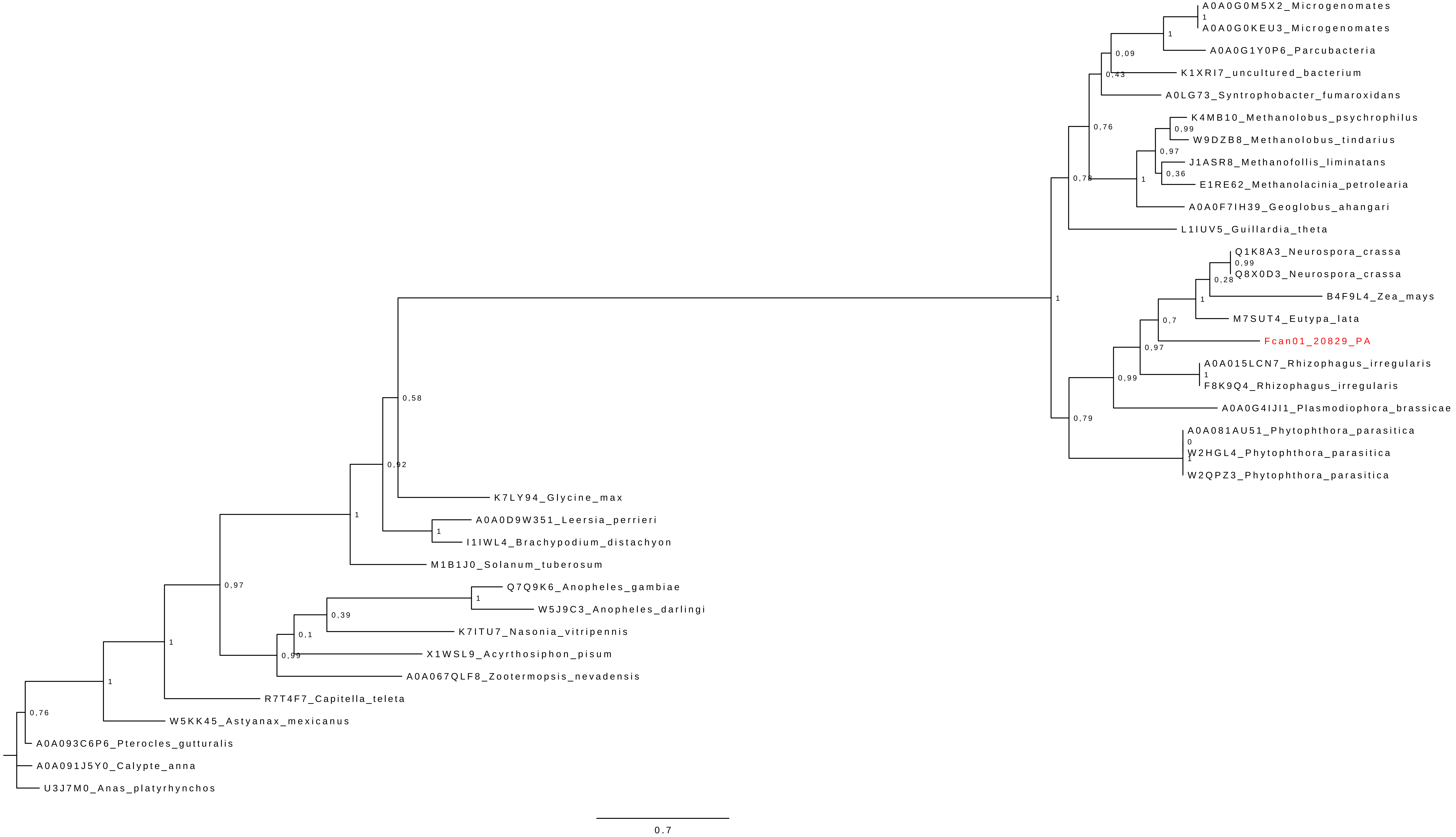

A0A0G0M5X2_Microgenomates
A0A0G0KEU3_Microgenomates
A0A0G1Y0P6_Parcubacteria
K1XRI7_uncultured_bacterium
A0LG73_Syntrophobacter_fumaroxidans
K4MB10_Methanolobus_psychrophilus
W9DZB8_Methanolobus_tindarius
J1ASR8_Methanofollis_liminatans
E1RE62_Methanolacinia_petrolearia
A0A0F7IH39_Geoglobus_ahangari
L1IUV5_Guillardia_theta
Q1K8A3_Neurospora_crassa
Q8X0D3_Neurospora_crassa
B4F9L4_Zea_mays
M7SUT4_Eutypa_lata
Fcan01_20829_PA
A0A015LCN7_Rhizophagus_irregularis
F8K9Q4_Rhizophagus_irregularis
A0A0G4IJI1_Plasmodiophora_brassicae
A0A081AU51_Phytophthora_parasitica
W2HGL4_Phytophthora_parasitica
W2QPZ3_Phytophthora_parasitica
K7LY94_Glycine_max
A0A0D9W351_Leersia_perrieri
I1IWL4_Brachypodium_distachyon
M1B1J0_Solanum_tuberosum
Q7Q9K6_Anopheles_gambiae
W5J9C3_Anopheles_darlingi
K7ITU7_Nasonia_vitripennis
X1WSL9_Acyrthosiphon_pisum
A0A067QLF8_Zootermopsis_nevadensis
R7T4F7_Capitella_teleta
W5KK45_Astyanax_mexicanus
A0A093C6P6_Pterocles_gutturalis
A0A091J5Y0_Calypte_anna
U3J7M0_Anas_platyrhynchos
0.7

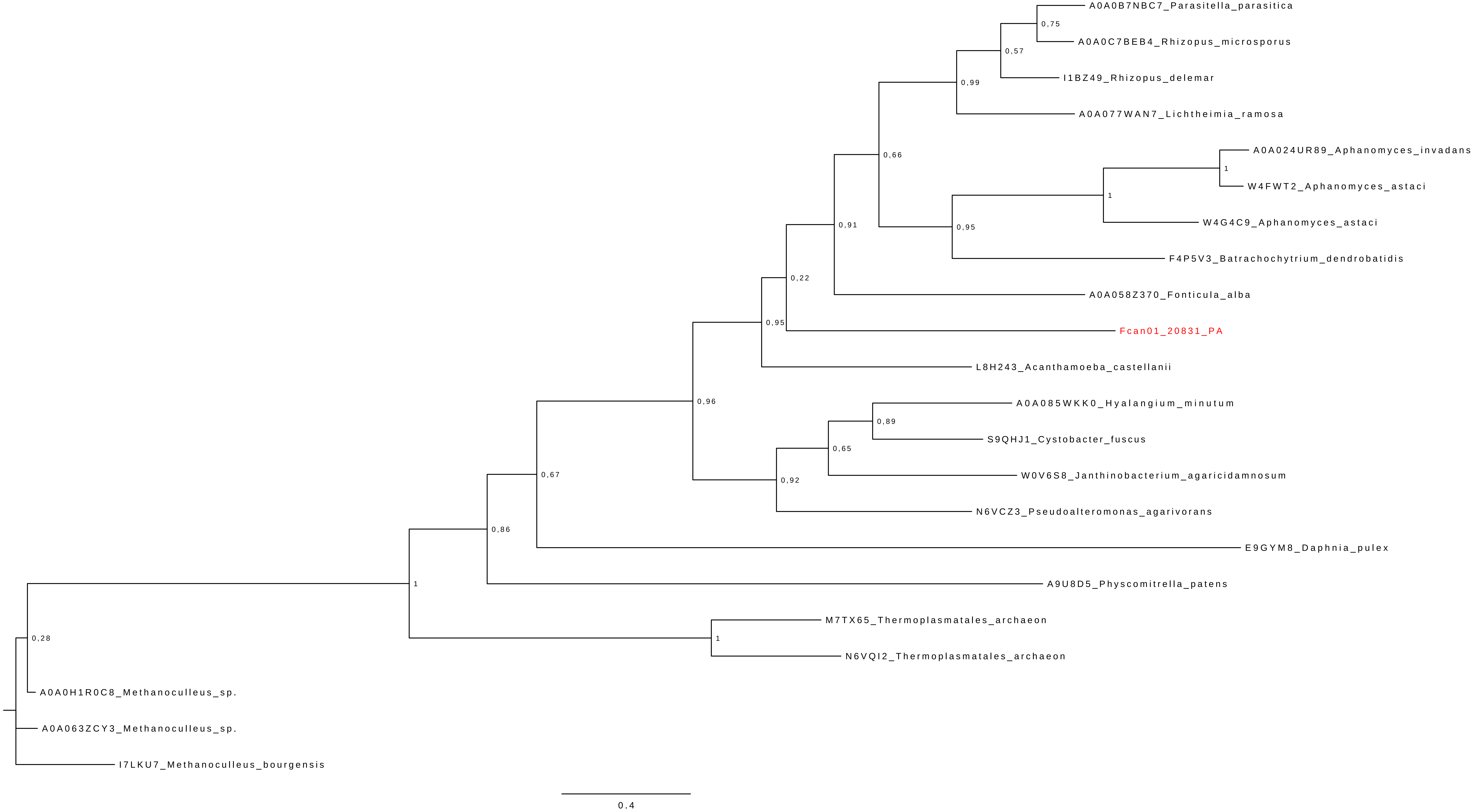

A0A0B7NBC7_Parasitella_parasitica
0,75
A0A0C7BEB4_Rhizopus_microsporus
0,57
I1BZ49_Rhizopus_delemar
0,99
A0A077WAN7_Lichtheimia_ramosa
A0A024UR89_Aphanomyces_invadans
1
W4FWT2_Aphanomyces_astaci
1
0,66
W4G4C9_Aphanomyces_astaci
0,95
F4P5V3_Batrachochytrium_dendrobatidis
0,91
A0A058Z370_Fonticula_alba
0,22
Fcan01_20831_PA
0,95
L8H243_Acanthamoeba_castellanii
0,96
A0A085WKK0_Hyalangium_minutum
0,89
S9QHJ1_Cystobacter_fuscus
0,65
W0V6S8_Janthinobacterium_agaricidamnosum
0,67
0,92
N6VCZ3_Pseudoalteromonas_agarivorans
0,86
E9GYM8_Daphnia_pulex
1
A9U8D5_Physcomitrella_patens
M7TX65_Thermoplasmatales_archaeon
0,28
1
N6VQI2_Thermoplasmatales_archaeon
A0A0H1R0C8_Methanoculleus_sp.
A0A063ZCY3_Methanoculleus_sp.
I7LKU7_Methanoculleus_bourgensis
0.4

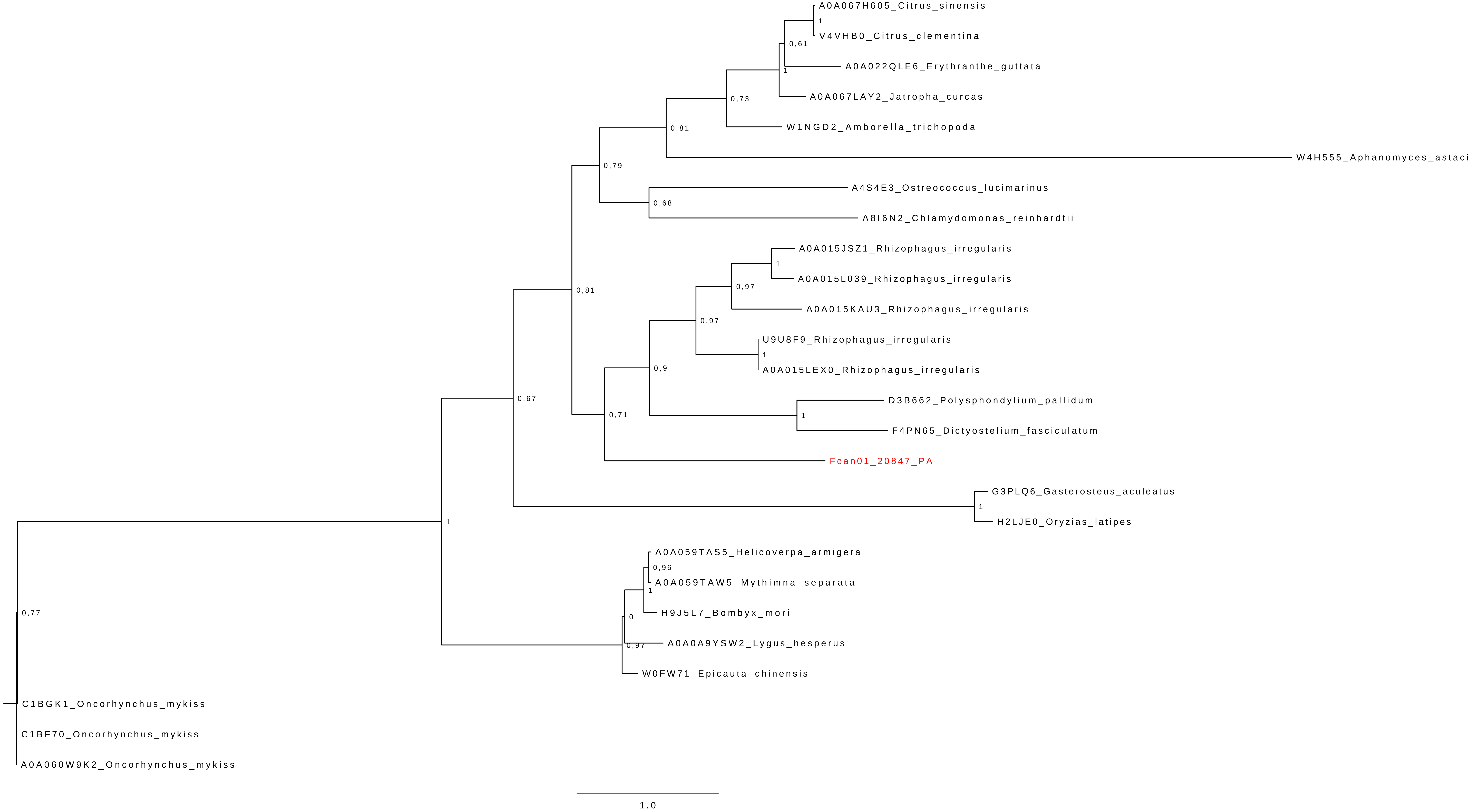
A0A067H605_Citrus_sinensis
1
V4VHB0_Citrus_clementina
0,61
A0A022QLE6_Erythranthe_guttata
1
A0A067LAY2_Jatropha_curcas
0,73
W1NGD2_Amborella_trichopoda
0,81
W4H555_Aphanomyces_astaci
0,79
A4S4E3_Ostreococcus_lucimarinus
0,68
A8I6N2_Chlamydomonas_reinhardtii
A0A015JSZ1_Rhizophagus_irregularis
1
A0A015L039_Rhizophagus_irregularis
0,97
0,81
A0A015KAU3_Rhizophagus_irregularis
0,97
U9U8F9_Rhizophagus_irregularis
1
A0A015LEX0_Rhizophagus_irregularis
0,9
D3B662_Polysphondylium_pallidum
0,67
1
0,71
F4PN65_Dictyostelium_fasciculatum
Fcan01_20847_PA
G3PLQ6_Gasterosteus_aculeatus
1
1
H2LJE0_Oryzias_latipes
A0A059TAS5_Helicoverpa_armigera
0,96
A0A059TAW5_Mythimna_separata
1
0,77
H9J5L7_Bombyx_mori
0
A0A0A9YSW2_Lygus_hesperus
0,97
W0FW71_Epicauta_chinensis
C1BGK1_Oncorhynchus_mykiss
C1BF70_Oncorhynchus_mykiss
A0A060W9K2_Oncorhynchus_mykiss
1.0

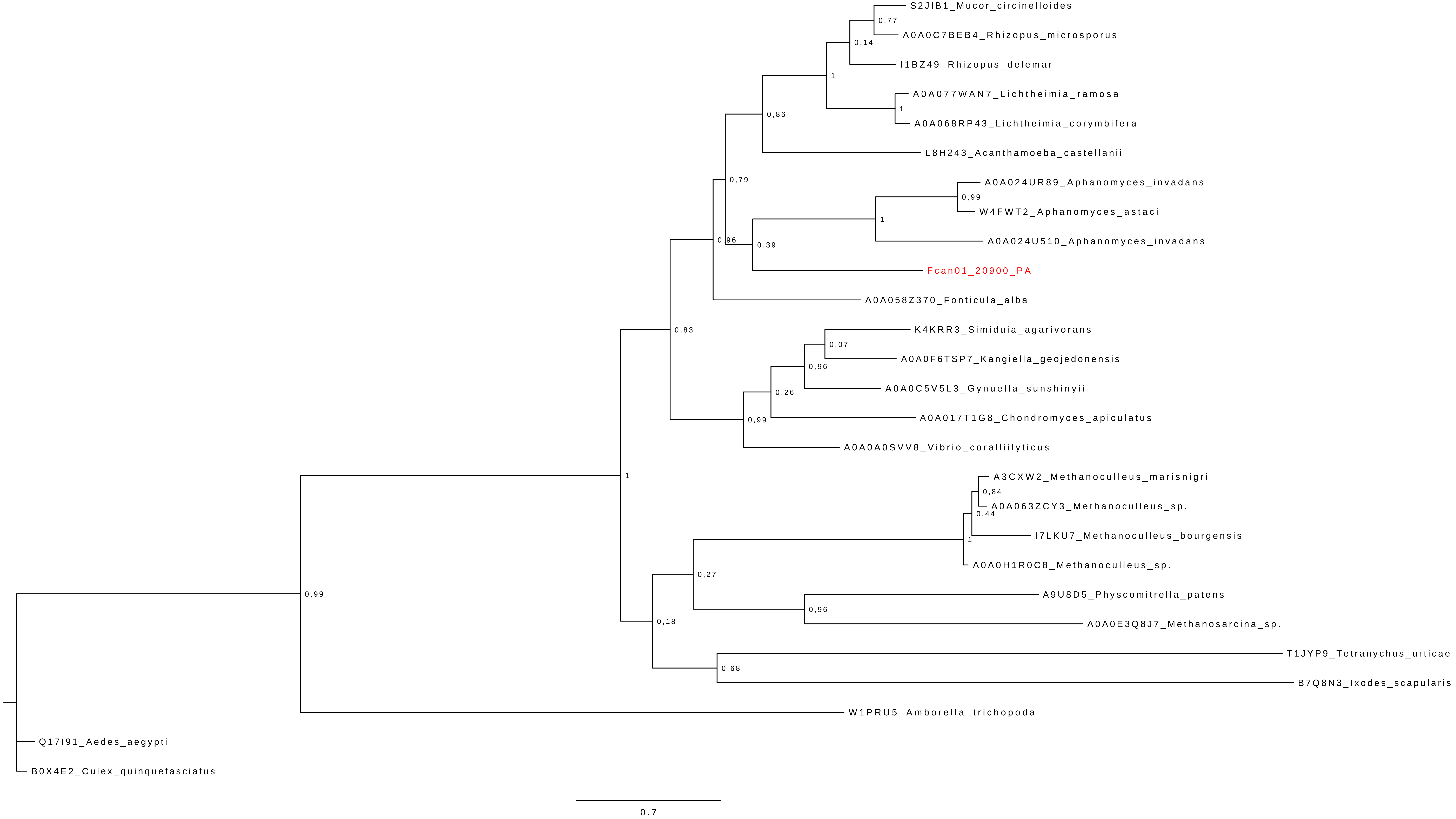
S2JIB1_Mucor_circinelloides
0,77
A0A0C7BEB4_Rhizopus_microsporus
0,14
I1BZ49_Rhizopus_delemar
1
A0A077WAN7_Lichtheimia_ramosa
1
A0A068RP43_Lichtheimia_corymbifera
0,86
L8H243_Acanthamoeba_castellanii
0,79
A0A024UR89_Aphanomyces_invadans
0,99
W4FWT2_Aphanomyces_astaci
1
0,96
0,39
A0A024U510_Aphanomyces_invadans
Fcan01_20900_PA
A0A058Z370_Fonticula_alba
0,83
K4KRR3_Simiduia_agarivorans
0,07
A0A0F6TSP7_Kangiella_geojedonensis
0,96
A0A0C5V5L3_Gynuella_sunshinyii
0,26
A0A017T1G8_Chondromyces_apiculatus
0,99
A0A0A0SVV8_Vibrio_coralliilyticus
1
A3CXW2_Methanoculleus_marisnigri
0,84
A0A063ZCY3_Methanoculleus_sp.
0,44
I7LKU7_Methanoculleus_bourgensis
1
A0A0H1R0C8_Methanoculleus_sp.
0,27
A9U8D5_Physcomitrella_patens
0,96
A0A0E3Q8J7_Methanosarcina_sp.
0,99
0,18
T1JYP9_Tetranychus_urticae
0,68
B7Q8N3_Ixodes_scapularis
W1PRU5_Amborella_trichopoda
Q17I91_Aedes_aegypti
B0X4E2_Culex_quinquefasciatus
0.7

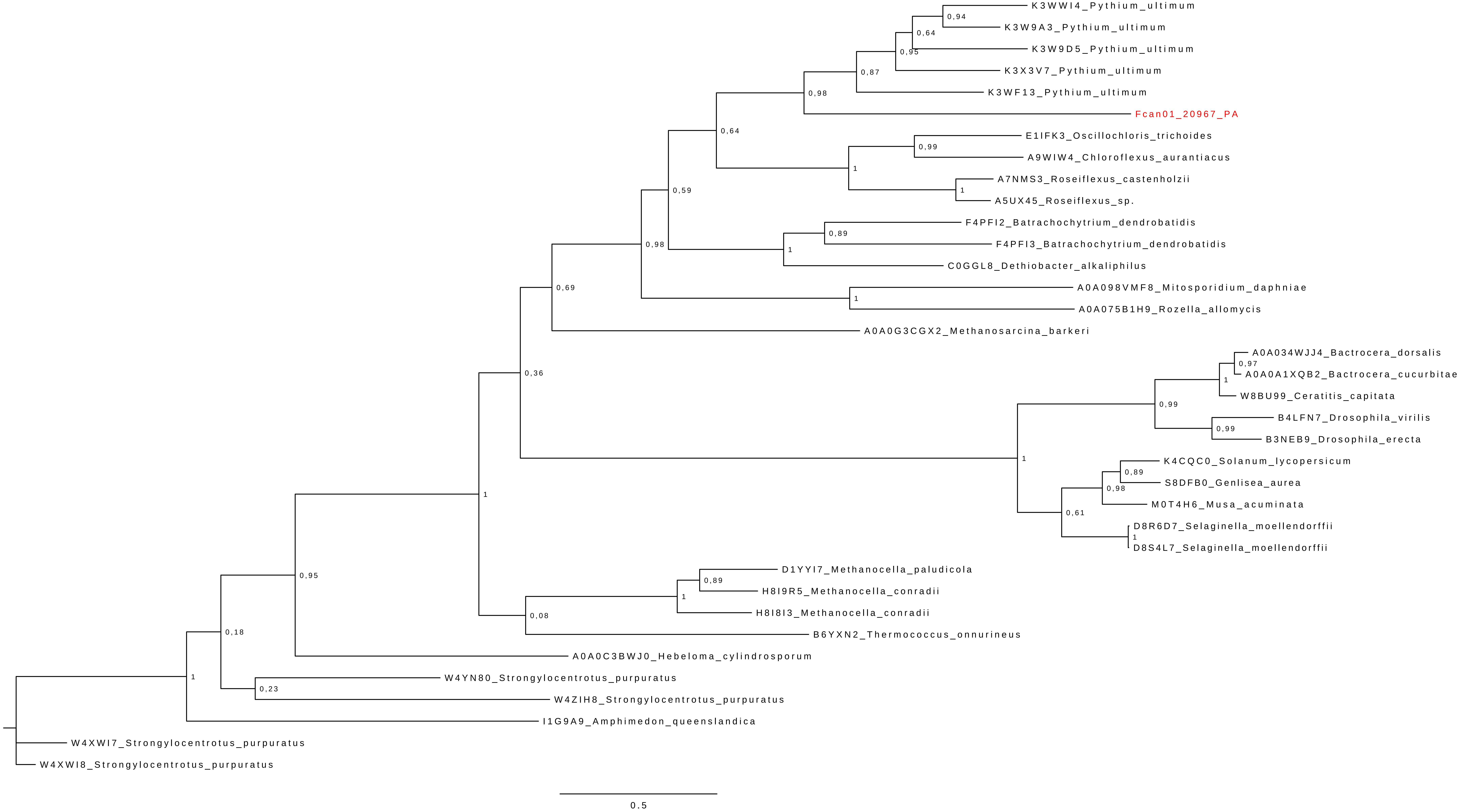

K3WWI4_Pythium_ultimum
K3W9A3_Pythium_ultimum
K3W9D5_Pythium_ultimum
K3X3V7_Pythium_ultimum
K3WF13_Pythium_ultimum
Fcan01_20967_PA
E1IFK3_Oscillochloris_trichoides
A9WIW4_Chloroflexus_aurantiacus
A7NMS3_Roseiflexus_castenholzii
A5UX45_Roseiflexus_sp.
F4PFI2_Batrachochytrium_dendrobatidis
F4PFI3_Batrachochytrium_dendrobatidis
C0GGL8_Dethiobacter_alkaliphilus
A0A098VMF8_Mitosporidium_daphniae
A0A075B1H9_Rozella_allomycis
A0A0G3CGX2_Methanosarcina_barkeri
A0A034WJJ4_Bactrocera_dorsalis
A0A0A1XQB2_Bactrocera_cucurbitae
W8BU99_Ceratitis_capitata
B4LFN7_Drosophila_virilis
B3NEB9_Drosophila_erecta
K4CQC0_Solanum_lycopersicum
S8DFB0_Genlisea_aurea
M0T4H6_Musa_acuminata
D8R6D7_Selaginella_moellendorffii
D8S4L7_Selaginella_moellendorffii
D1YYI7_Methanocella_paludicola
H8I9R5_Methanocella_conradii
H8I8I3_Methanocella_conradii
B6YXN2_Thermococcus_onnurineus
A0A0C3BWJ0_Hebeloma_cylindrosporum
W4YN80_Strongylocentrotus_purpuratus
W4ZIH8_Strongylocentrotus_purpuratus
I1G9A9_Amphimedon_queenslandica
W4XWI7_Strongylocentrotus_purpuratus
W4XWI8_Strongylocentrotus_purpuratus
0.5

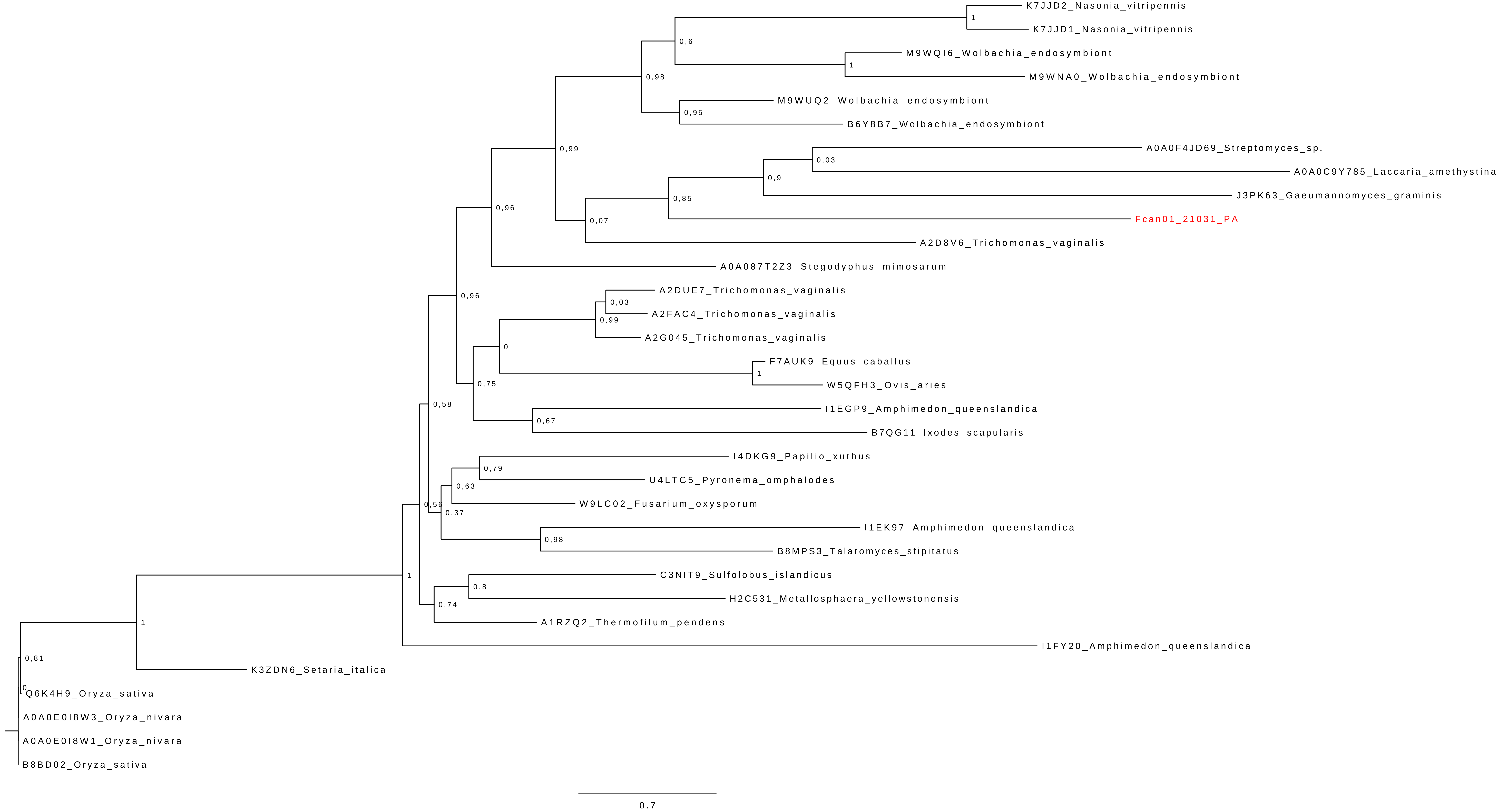
K7JJD2_Nasonia_vitripennis
K7JJD1_Nasonia_vitripennis
M9WQI6_Wolbachia_endosymbiont
M9WNA0_Wolbachia_endosymbiont
M9WUQ2_Wolbachia_endosymbiont
B6Y8B7_Wolbachia_endosymbiont
A0A0F4JD69_Streptomyces_sp.
A0A0C9Y785_Laccaria_amethystina
J3PK63_Gaeumannomyces_graminis
Fcan01_21031_PA
A2D8V6_Trichomonas_vaginalis
A0A087T2Z3_Stegodyphus_mimosarum
A2DUE7_Trichomonas_vaginalis
A2FAC4_Trichomonas_vaginalis
A2G045_Trichomonas_vaginalis
F7AUK9_Equus_caballus
W5QFH3_Ovis_aries
I1EGP9_Amphimedon_queenslandica
B7QG11_Ixodes_scapularis
I4DKG9_Papilio_xuthus
U4LTC5_Pyronema_omphalodes
W9LC02_Fusarium_oxysporum
I1EK97_Amphimedon_queenslandica
B8MPS3_Talaromyces_stipitatus
C3NIT9_Sulfolobus_islandicus
H2C531_Metallosphaera_yellowstonensis
A1RZQ2_Thermofilum_pendens
I1FY20_Amphimedon_queenslandica
K3ZDN6_Setaria_italica
Q6K4H9_Oryza_sativa
A0A0E0I8W3_Oryza_nivara
A0A0E0I8W1_Oryza_nivara
B8BD02_Oryza_sativa
0.7

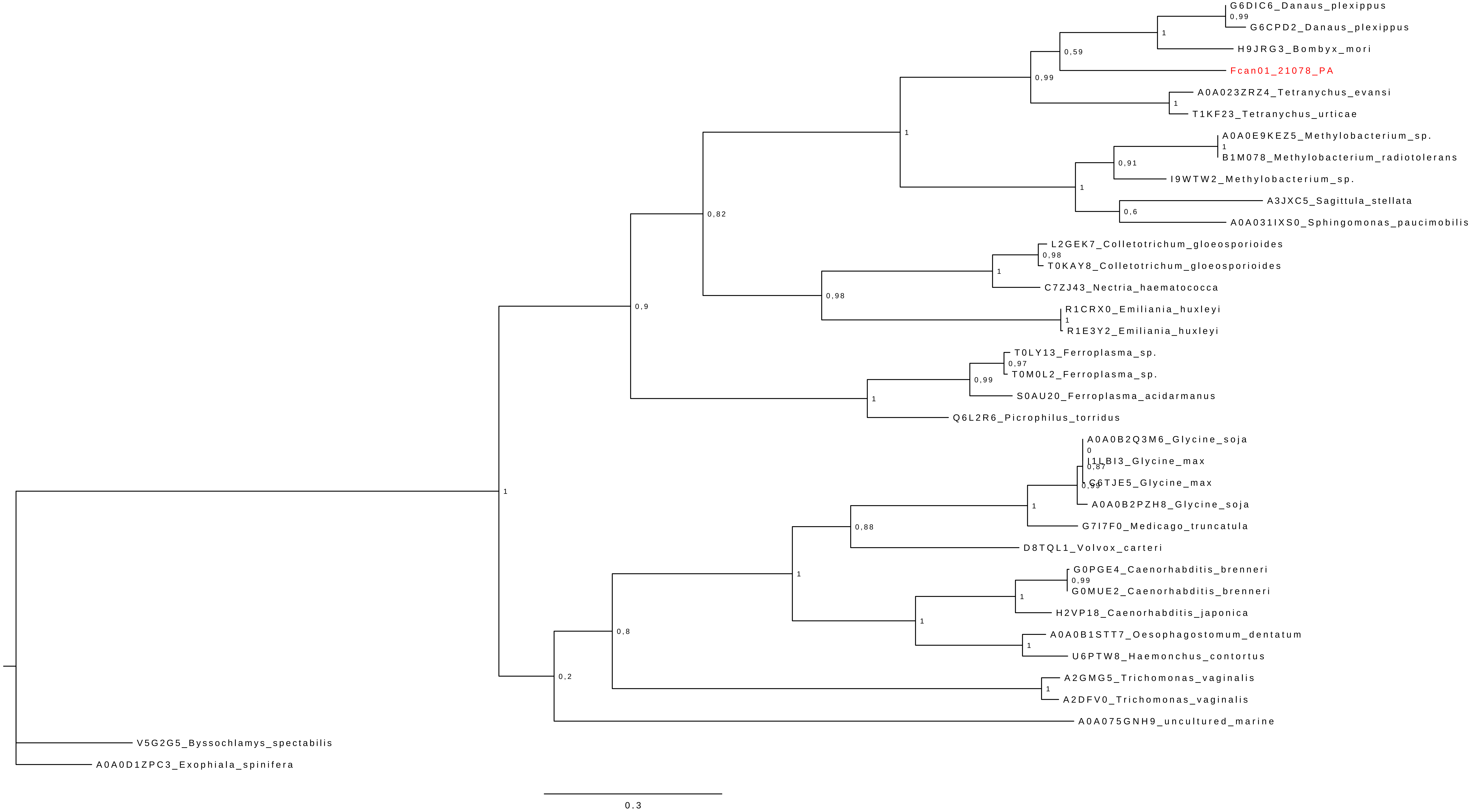
G6DIC6_Danaus_plexippus
0,99
G6CPD2_Danaus_plexippus
1
H9JRG3_Bombyx_mori
0,59
Fcan01_21078_PA
0,99
A0A023ZRZ4_Tetranychus_evansi
1
T1KF23_Tetranychus_urticae
1
A0A0E9KEZ5_Methylobacterium_sp.
1
B1M078_Methylobacterium_radiotolerans
0,91
I9WTW2_Methylobacterium_sp.
1
A3JXC5_Sagittula_stellata
0,6
A0A031IXS0_Sphingomonas_paucimobilis
0,82
L2GEK7_Colletotrichum_gloeosporioides
0,98
T0KAY8_Colletotrichum_gloeosporioides
1
C7ZJ43_Nectria_haematococca
0,98
R1CRX0_Emiliania_huxleyi
1
R1E3Y2_Emiliania_huxleyi
0,9
T0LY13_Ferroplasma_sp.
0,97
T0M0L2_Ferroplasma_sp.
0,99
S0AU20_Ferroplasma_acidarmanus
1
Q6L2R6_Picrophilus_torridus
A0A0B2Q3M6_Glycine_soja
0
I1LBI3_Glycine_max
0,87
C6TJE5_Glycine_max
0,99
A0A0B2PZH8_Glycine_soja
1
G7I7F0_Medicago_truncatula
0,88
D8TQL1_Volvox_carteri
1
G0PGE4_Caenorhabditis_brenneri
0,99
G0MUE2_Caenorhabditis_brenneri
1
H2VP18_Caenorhabditis_japonica
1
A0A0B1STT7_Oesophagostomum_dentatum
1
U6PTW8_Haemonchus_contortus
0,8
A2GMG5_Trichomonas_vaginalis
1
A2DFV0_Trichomonas_vaginalis
0,2
A0A075GNH9_uncultured_marine
1
V5G2G5_Byssochlamys_spectabilis
A0A0D1ZPC3_Exophiala_spinifera
0.3

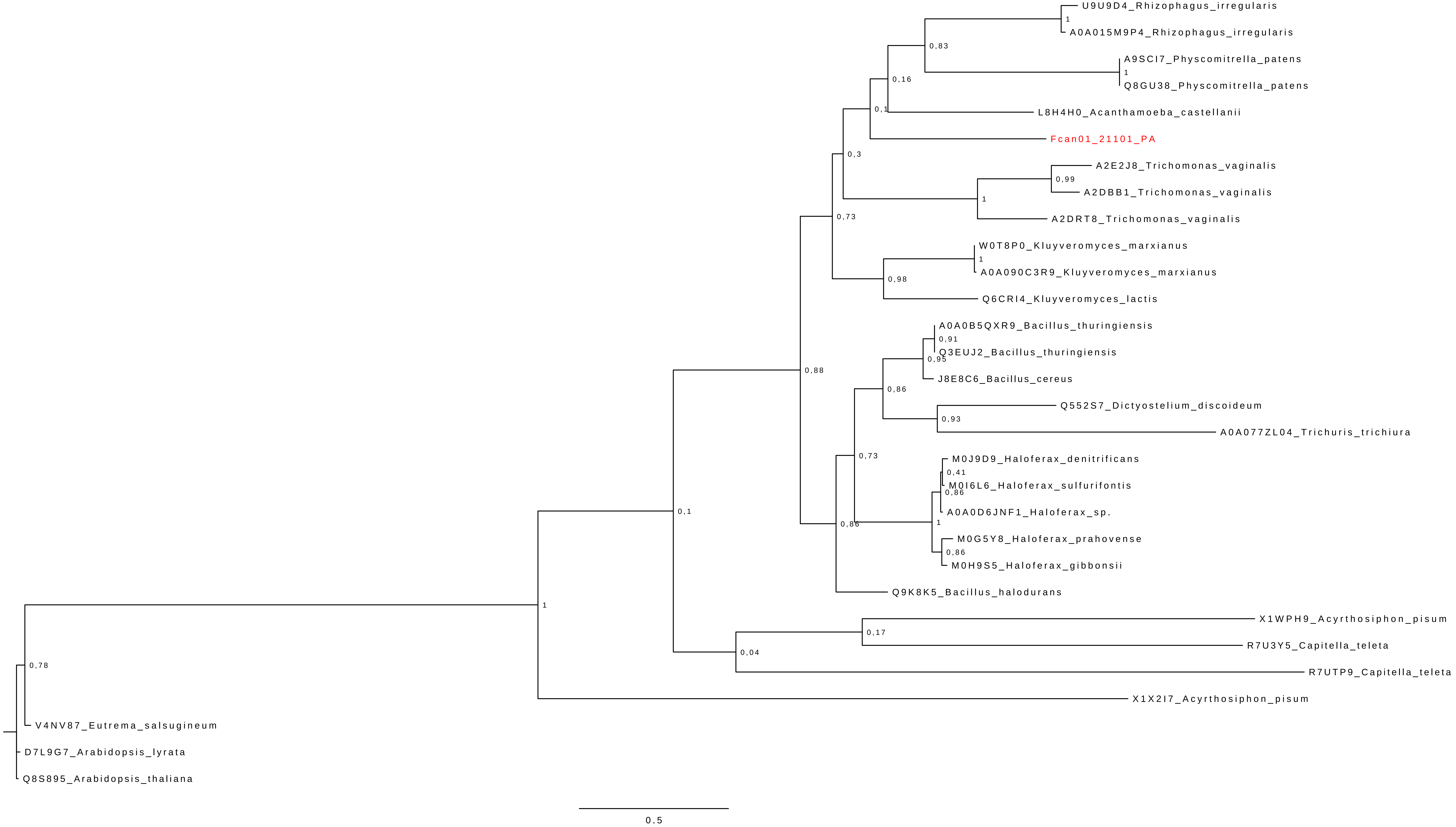
U9U9D4_Rhizophagus_irregularis
A0A015M9P4_Rhizophagus_irregularis
A9SCI7_Physcomitrella_patens
Q8GU38_Physcomitrella_patens
L8H4H0_Acanthamoeba_castellanii
Fcan01_21101_PA
A2E2J8_Trichomonas_vaginalis
A2DBB1_Trichomonas_vaginalis
A2DRT8_Trichomonas_vaginalis
W0T8P0_Kluyveromyces_marxianus
A0A090C3R9_Kluyveromyces_marxianus
Q6CRI4_Kluyveromyces_lactis
A0A0B5QXR9_Bacillus_thuringiensis
Q3EUJ2_Bacillus_thuringiensis
J8E8C6_Bacillus_cereus
Q552S7_Dictyostelium_discoideum
A0A077ZL04_Trichuris_trichiura
M0J9D9_Haloferax_denitrificans
M0I6L6_Haloferax_sulfurifontis
A0A0D6JNF1_Haloferax_sp.
M0G5Y8_Haloferax_prahovense
M0H9S5_Haloferax_gibbonsii
Q9K8K5_Bacillus_halodurans
X1WPH9_Acyrthosiphon_pisum
R7U3Y5_Capitella_teleta
R7UTP9_Capitella_teleta
X1X2I7_Acyrthosiphon_pisum
V4NV87_Eutrema_salsugineum
D7L9G7_Arabidopsis_lyrata
Q8S895_Arabidopsis_thaliana
0.5

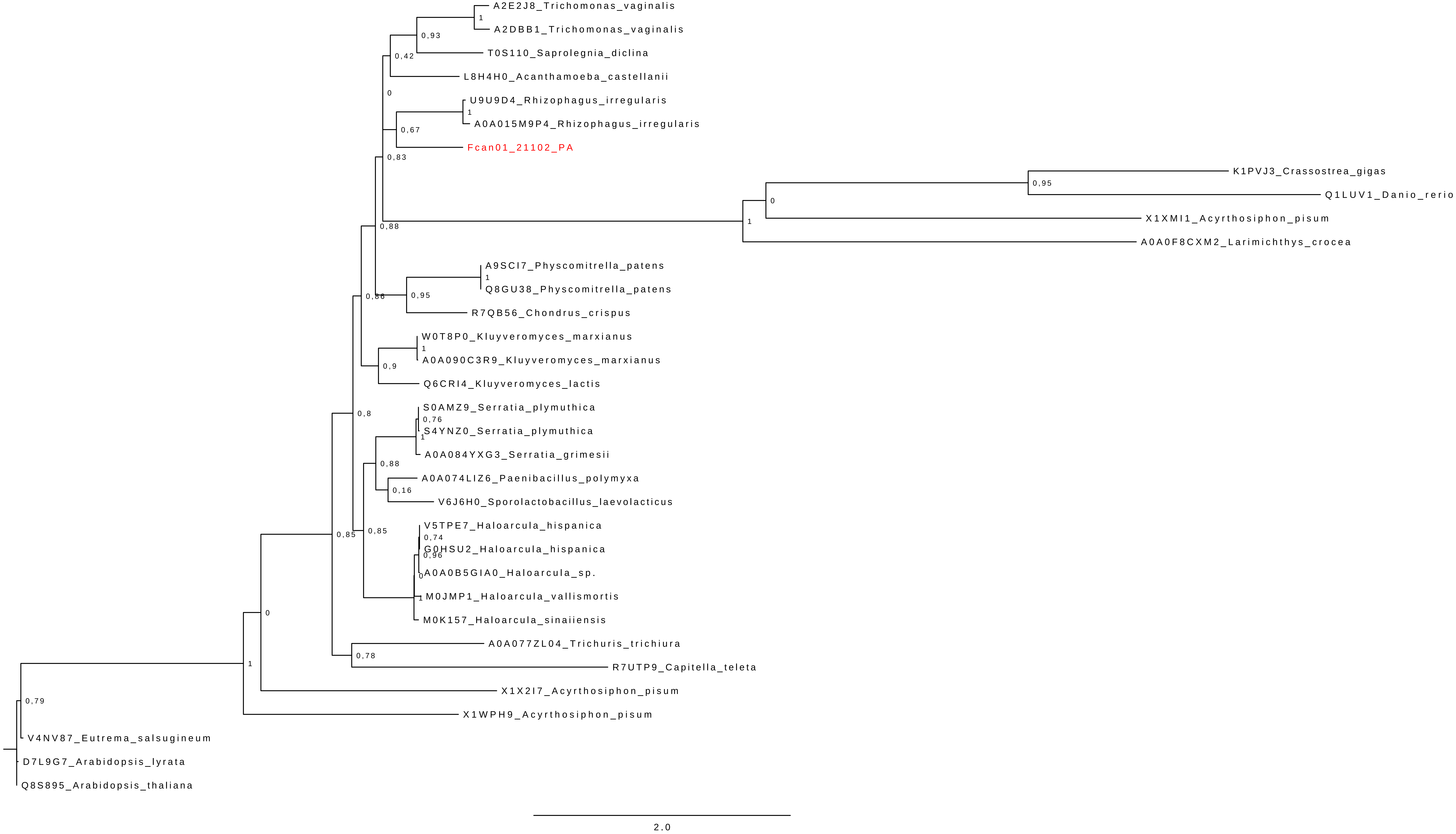
A2E2J8_Trichomonas_vaginalis
A2DBB1_Trichomonas_vaginalis
T0S110_Saprolegnia_diclina
L8H4H0_Acanthamoeba_castellanii
U9U9D4_Rhizophagus_irregularis
A0A015M9P4_Rhizophagus_irregularis
Fcan01_21102_PA
K1PVJ3_Crassostrea_gigas
Q1LUV1_Danio_rerio
X1XMI1_Acyrthosiphon_pisum
A0A0F8CXM2_Larimichthys_crocea
A9SCI7_Physcomitrella_patens
Q8GU38_Physcomitrella_patens
R7QB56_Chondrus_crispus
W0T8P0_Kluyveromyces_marxianus
A0A090C3R9_Kluyveromyces_marxianus
Q6CRI4_Kluyveromyces_lactis
S0AMZ9_Serratia_plymuthica
S4YNZ0_Serratia_plymuthica
A0A084YXG3_Serratia_grimesii
A0A074LIZ6_Paenibacillus_polymyxa
V6J6H0_Sporolactobacillus_laevolacticus
V5TPE7_Haloarcula_hispanica
G0HSU2_Haloarcula_hispanica
A0A0B5GIA0_Haloarcula_sp.
M0JMP1_Haloarcula_vallismortis
M0K157_Haloarcula_sinaiiensis
A0A077ZL04_Trichuris_trichiura
R7UTP9_Capitella_teleta
X1X2I7_Acyrthosiphon_pisum
X1WPH9_Acyrthosiphon_pisum
V4NV87_Eutrema_salsugineum
D7L9G7_Arabidopsis_lyrata
Q8S895_Arabidopsis_thaliana
2.0

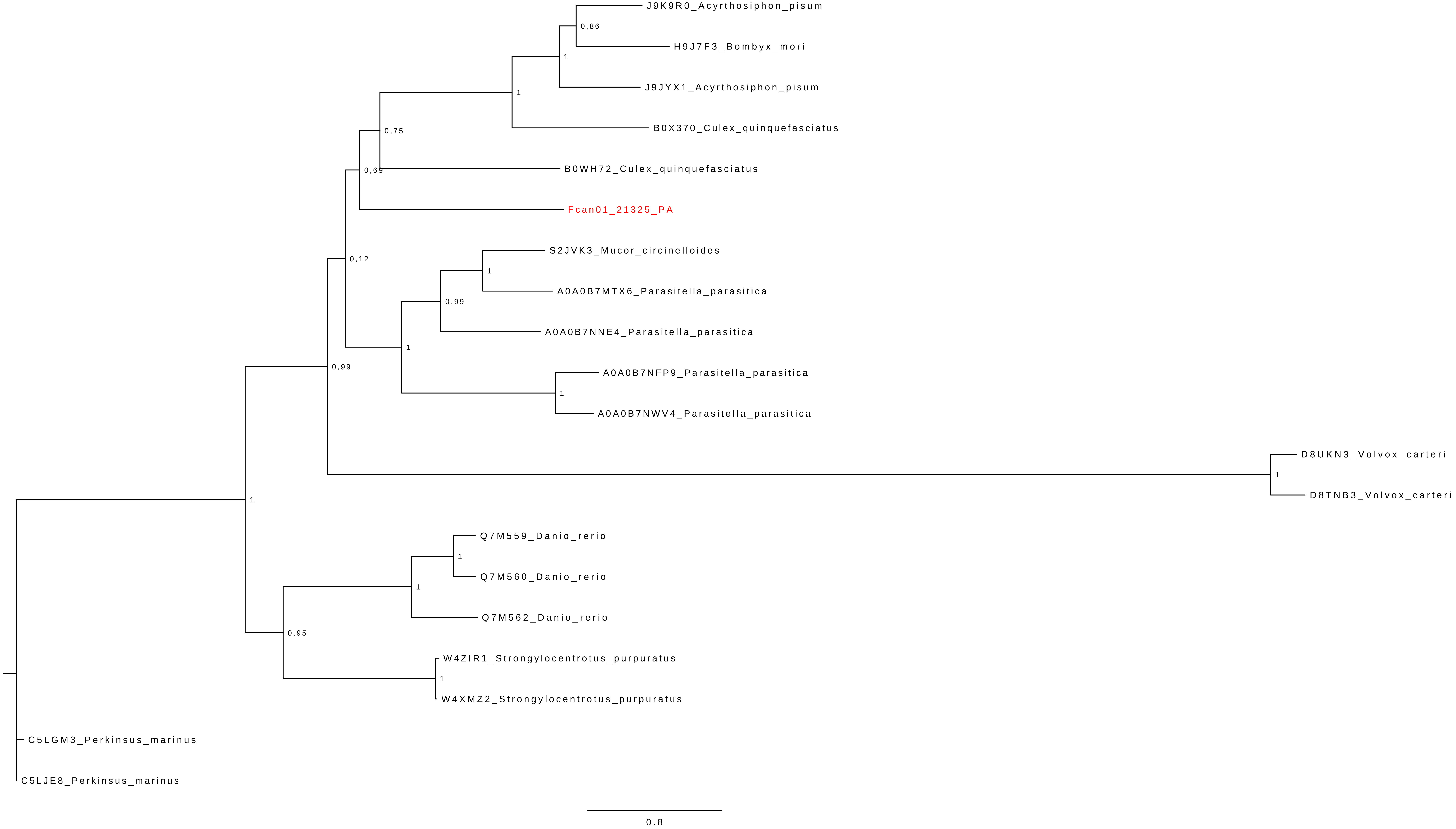

J9K9R0_Acyrthosiphon_pisum
0,86
H9J7F3_Bombyx_mori
1
J9JYX1_Acyrthosiphon_pisum
1
B0X370_Culex_quinquefasciatus
0,75
B0WH72_Culex_quinquefasciatus
0,69
Fcan01_21325_PA
S2JVK3_Mucor_circinelloides
0,12
1
A0A0B7MTX6_Parasitella_parasitica
0,99
A0A0B7NNE4_Parasitella_parasitica
1
A0A0B7NFP9_Parasitella_parasitica
0,99
1
A0A0B7NWV4_Parasitella_parasitica
D8UKN3_Volvox_carteri
1
1
D8TNB3_Volvox_carteri
Q7M559_Danio_rerio
1
Q7M560_Danio_rerio
1
Q7M562_Danio_rerio
0,95
W4ZIR1_Strongylocentrotus_purpuratus
1
W4XMZ2_Strongylocentrotus_purpuratus
C5LGM3_Perkinsus_marinus
C5LJE8_Perkinsus_marinus
0.8

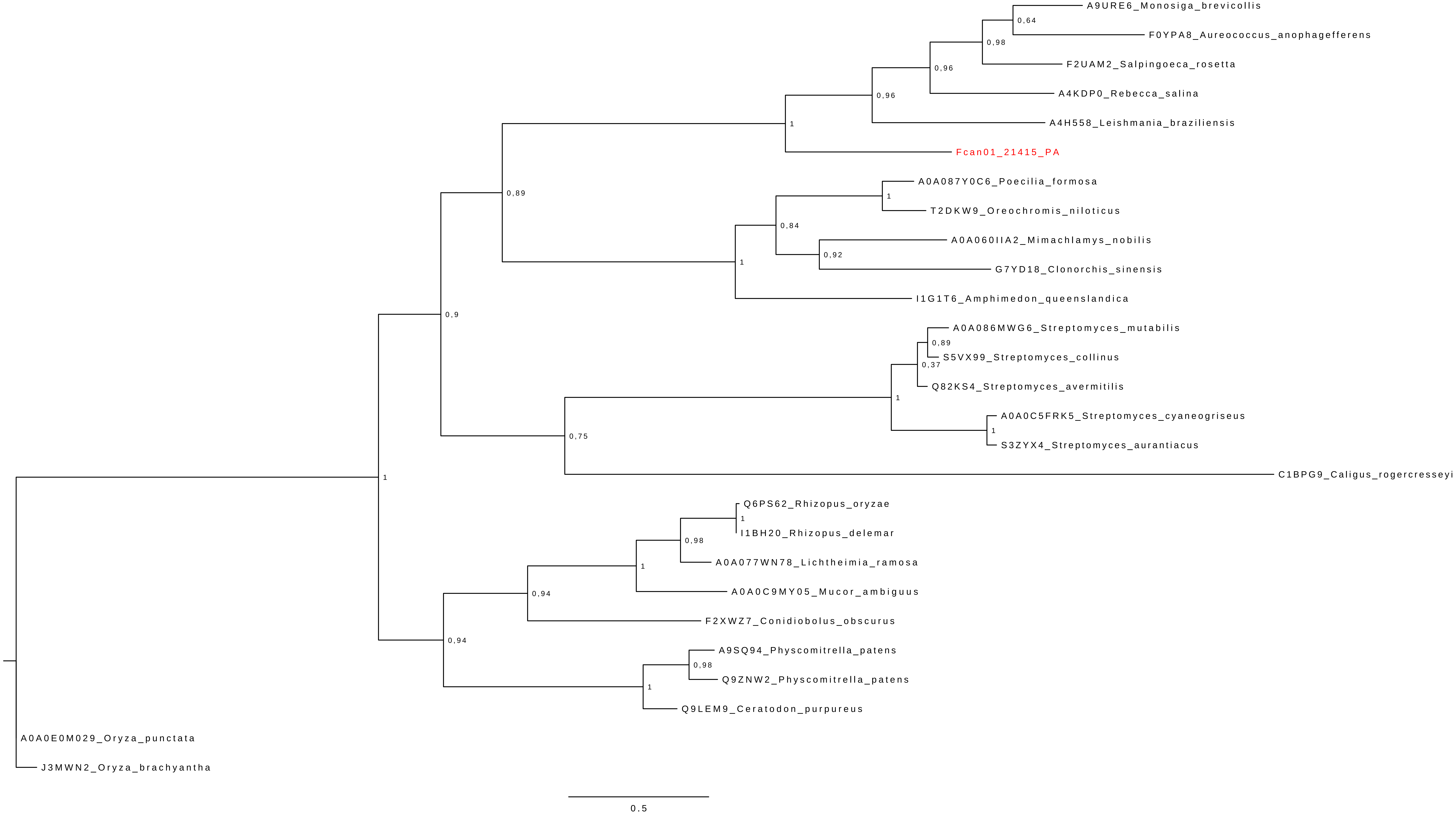
A9URE6_Monosiga_brevicollis
0,64
F0YPA8_Aureococcus_anophagefferens
0,98
F2UAM2_Salpingoeca_rosetta
0,96
A4KDP0_Rebecca_salina
0,96
A4H558_Leishmania_braziliensis
1
Fcan01_21415_PA
A0A087Y0C6_Poecilia_formosa
1
T2DKW9_Oreochromis_niloticus
0,89
0,84
A0A060IIA2_Mimachlamys_nobilis
0,92
G7YD18_Clonorchis_sinensis
1
I1G1T6_Amphimedon_queenslandica
0,9
A0A086MWG6_Streptomyces_mutabilis
0,89
S5VX99_Streptomyces_collinus
0,37
Q82KS4_Streptomyces_avermitilis
1
A0A0C5FRK5_Streptomyces_cyaneogriseus
1
0,75
S3ZYX4_Streptomyces_aurantiacus
C1BPG9_Caligus_rogercresseyi
1
Q6PS62_Rhizopus_oryzae
1
I1BH20_Rhizopus_delemar
0,98
A0A077WN78_Lichtheimia_ramosa
1
A0A0C9MY05_Mucor_ambiguus
0,94
F2XWZ7_Conidiobolus_obscurus
0,94
A9SQ94_Physcomitrella_patens
0,98
Q9ZNW2_Physcomitrella_patens
1
Q9LEM9_Ceratodon_purpureus
A0A0E0M029_Oryza_punctata
J3MWN2_Oryza_brachyantha
0.5

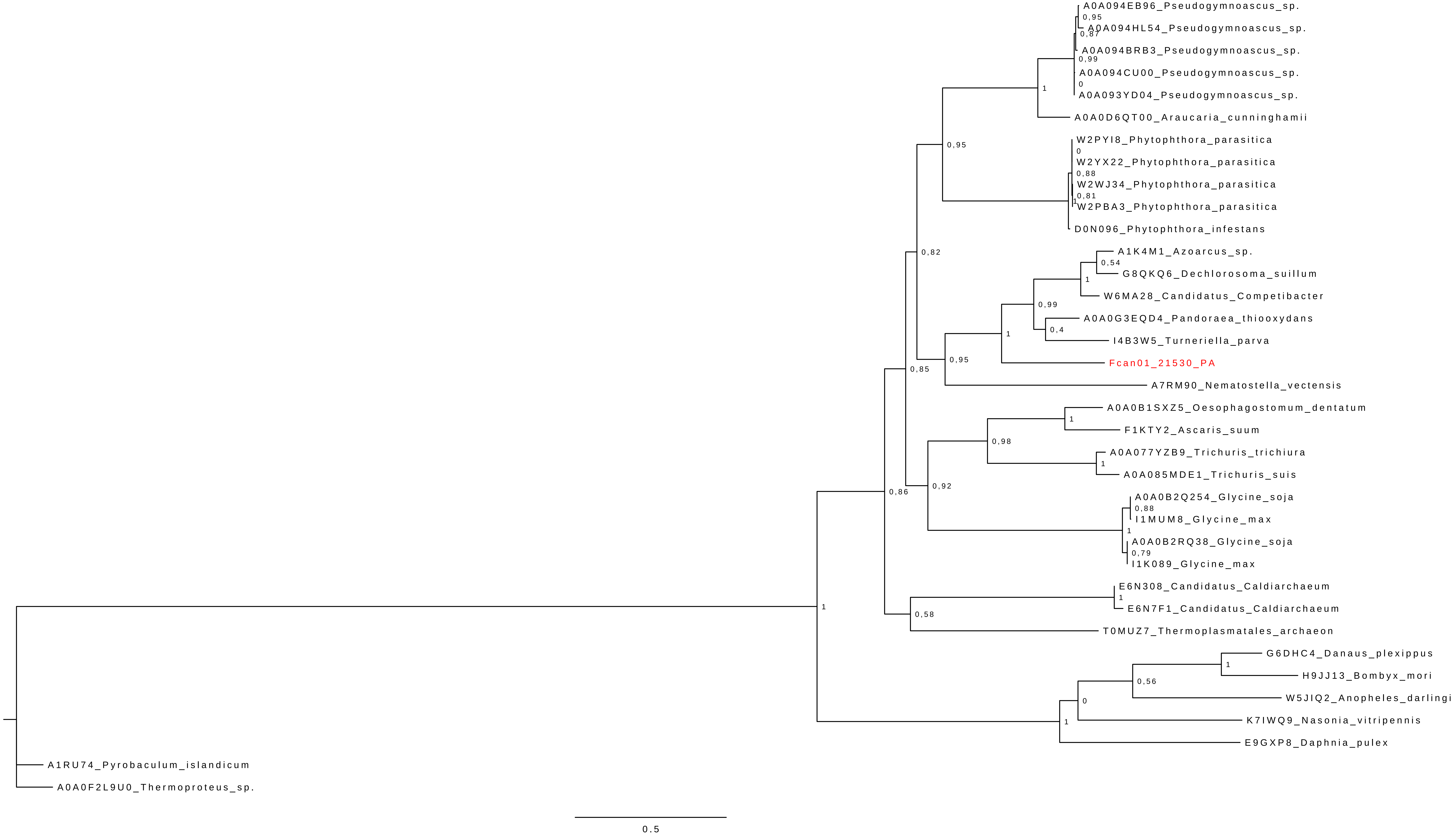
A0A094EB96_Pseudogymnoascus_sp.
A0A094HL54_Pseudogymnoascus_sp.
A0A094BRB3_Pseudogymnoascus_sp.
A0A094CU00_Pseudogymnoascus_sp.
A0A093YD04_Pseudogymnoascus_sp.
A0A0D6QT00_Araucaria_cunninghamii
W2PYI8_Phytophthora_parasitica
W2YX22_Phytophthora_parasitica
W2WJ34_Phytophthora_parasitica
W2PBA3_Phytophthora_parasitica
D0N096_Phytophthora_infestans
A1K4M1_Azoarcus_sp.
G8QKQ6_Dechlorosoma_suillum
W6MA28_Candidatus_Competibacter
A0A0G3EQD4_Pandoraea_thiooxydans
I4B3W5_Turneriella_parva
Fcan01_21530_PA
A7RM90_Nematostella_vectensis
A0A0B1SXZ5_Oesophagostomum_dentatum
F1KTY2_Ascaris_suum
A0A077YZB9_Trichuris_trichiura
A0A085MDE1_Trichuris_suis
A0A0B2Q254_Glycine_soja
I1MUM8_Glycine_max
A0A0B2RQ38_Glycine_soja
I1K089_Glycine_max
E6N308_Candidatus_Caldiarchaeum
E6N7F1_Candidatus_Caldiarchaeum
T0MUZ7_Thermoplasmatales_archaeon
G6DHC4_Danaus_plexippus
H9JJ13_Bombyx_mori
W5JIQ2_Anopheles_darlingi
K7IWQ9_Nasonia_vitripennis
E9GXP8_Daphnia_pulex
A1RU74_Pyrobaculum_islandicum
A0A0F2L9U0_Thermoproteus_sp.
0.5

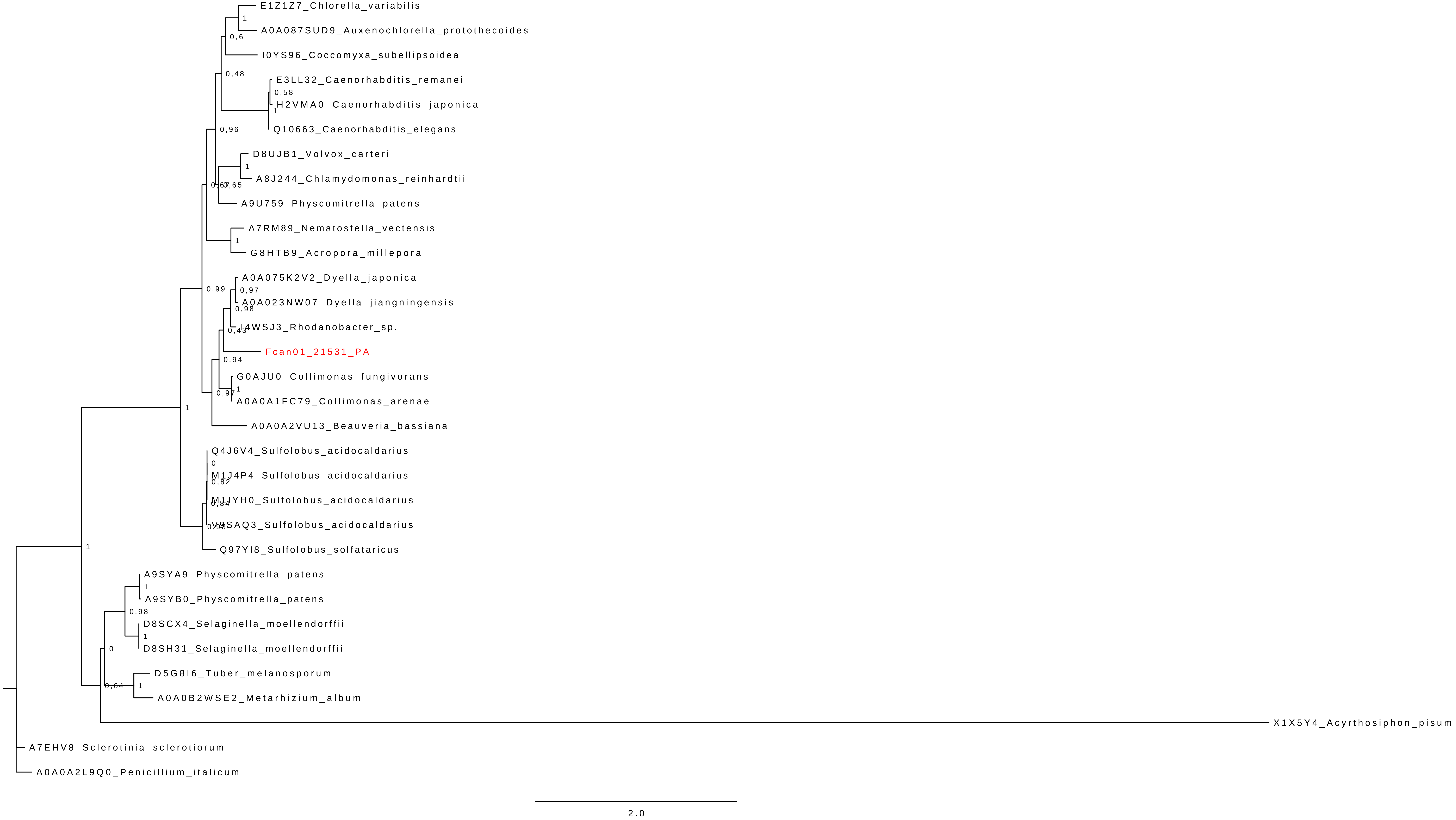
E1Z1Z7_Chlorella_variabilis
A0A087SUD9_Auxenochlorella_protothecoides
I0YS96_Coccomyxa_subellipsoidea
E3LL32_Caenorhabditis_remanei
H2VMA0_Caenorhabditis_japonica
Q10663_Caenorhabditis_elegans
D8UJB1_Volvox_carteri
A8J244_Chlamydomonas_reinhardtii
A9U759_Physcomitrella_patens
A7RM89_Nematostella_vectensis
G8HTB9_Acropora_millepora
A0A075K2V2_Dyella_japonica
A0A023NW07_Dyella_jiangningensis
I4WSJ3_Rhodanobacter_sp.
Fcan01_21531_PA
G0AJU0_Collimonas_fungivorans
A0A0A1FC79_Collimonas_arenae
A0A0A2VU13_Beauveria_bassiana
Q4J6V4_Sulfolobus_acidocaldarius
M1J4P4_Sulfolobus_acidocaldarius
M1IYH0_Sulfolobus_acidocaldarius
V9SAQ3_Sulfolobus_acidocaldarius
Q97YI8_Sulfolobus_solfataricus
A9SYA9_Physcomitrella_patens
A9SYB0_Physcomitrella_patens
D8SCX4_Selaginella_moellendorffii
D8SH31_Selaginella_moellendorffii
D5G8I6_Tuber_melanosporum
A0A0B2WSE2_Metarhizium_album
X1X5Y4_Acyrthosiphon_pisum
A7EHV8_Sclerotinia_sclerotiorum
A0A0A2L9Q0_Penicillium_italicum
2.0

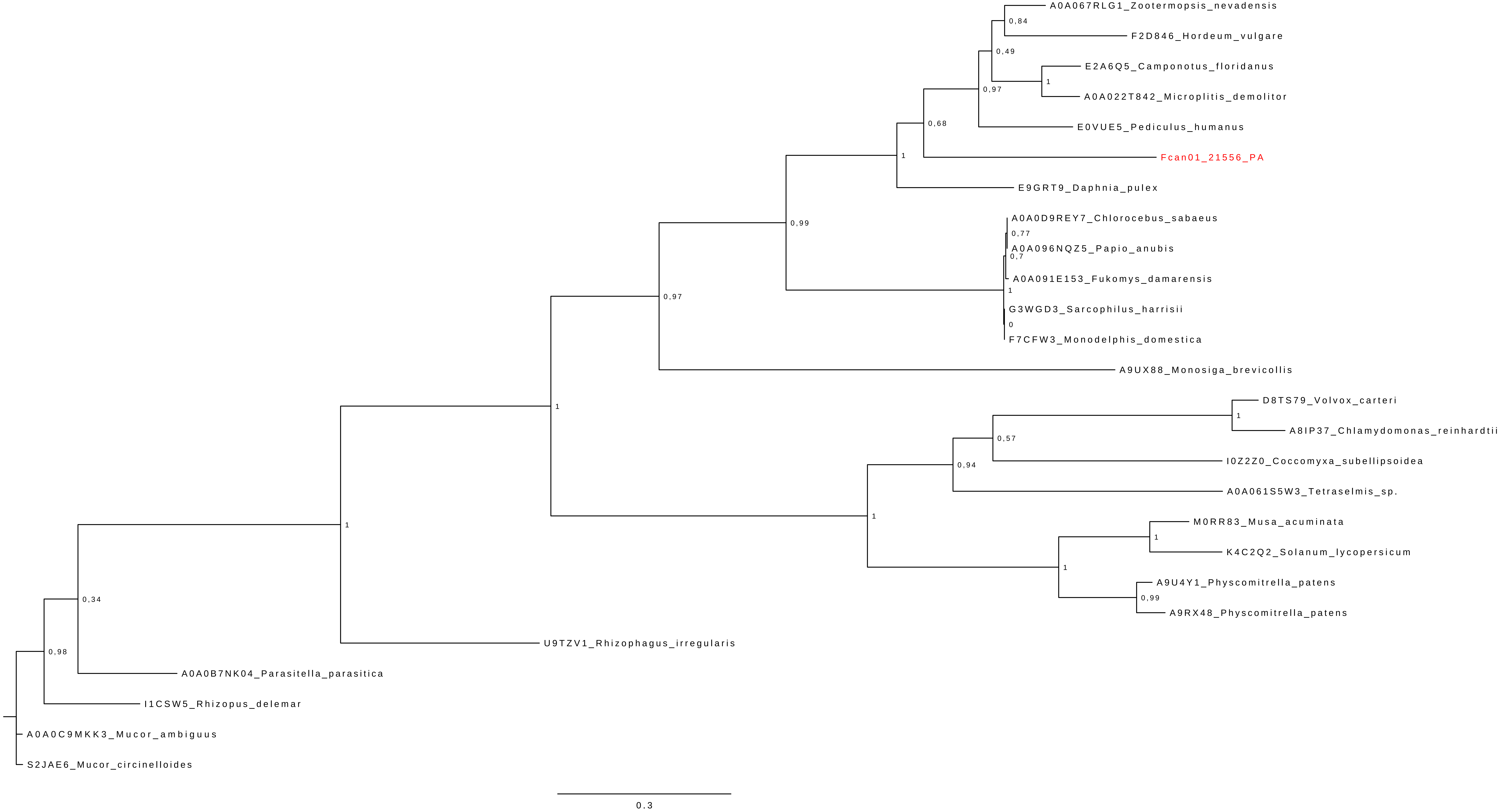

A0A067RLG1_Zootermopsis_nevadensis
0,84
F2D846_Hordeum_vulgare
0,49
E2A6Q5_Camponotus_floridanus
1
A0A022T842_Microplitis_demolitor
0,97
E0VUE5_Pediculus_humanus
0,68
Fcan01_21556_PA
1
E9GRT9_Daphnia_pulex
A0A0D9REY7_Chlorocebus_sabaeus
0,77
A0A096NQZ5_Papio_anubis
0,7
0,99
A0A091E153_Fukomys_damarensis
1
0,97
G3WGD3_Sarcophilus_harrisii
0
F7CFW3_Monodelphis_domestica
A9UX88_Monosiga_brevicollis
D8TS79_Volvox_carteri
1
1
A8IP37_Chlamydomonas_reinhardtii
0,57
I0Z2Z0_Coccomyxa_subellipsoidea
0,94
A0A061S5W3_Tetraselmis_sp.
1
M0RR83_Musa_acuminata
1
1
K4C2Q2_Solanum_lycopersicum
1
A9U4Y1_Physcomitrella_patens
0,99
A9RX48_Physcomitrella_patens
0,34
U9TZV1_Rhizophagus_irregularis
0,98
A0A0B7NK04_Parasitella_parasitica
I1CSW5_Rhizopus_delemar
A0A0C9MKK3_Mucor_ambiguus
S2JAE6_Mucor_circinelloides
0.3

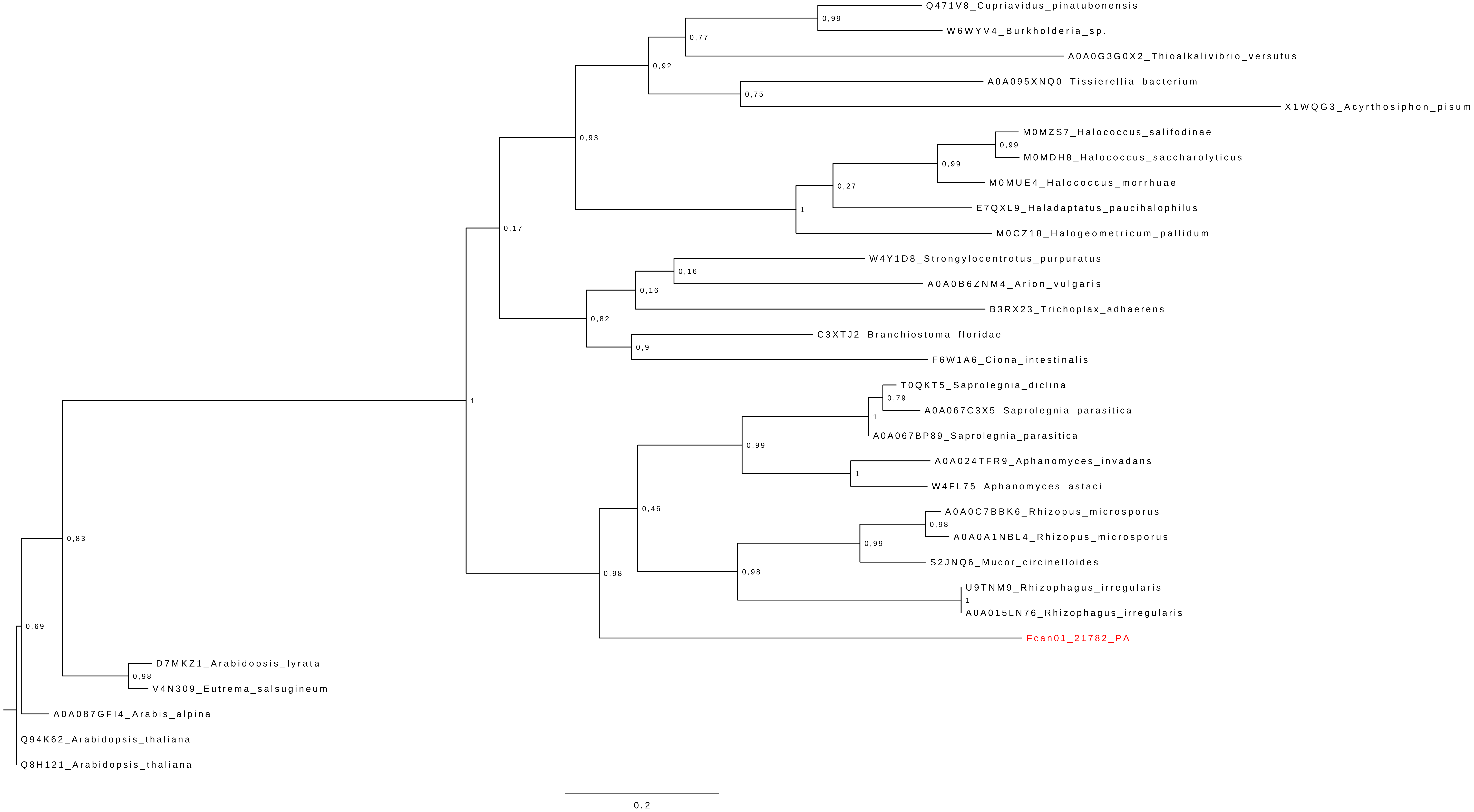
Q471V8_Cupriavidus_pinatubonensis
0,99
W6WYV4_Burkholderia_sp.
0,77
A0A0G3G0X2_Thioalkalivibrio_versutus
0,92
A0A095XNQ0_Tissierellia_bacterium
0,75
X1WQG3_Acyrthosiphon_pisum
0,93
M0MZS7_Halococcus_salifodinae
0,99
M0MDH8_Halococcus_saccharolyticus
0,99
M0MUE4_Halococcus_morrhuae
0,27
E7QXL9_Haladaptatus_paucihalophilus
1
M0CZ18_Halogeometricum_pallidum
0,17
W4Y1D8_Strongylocentrotus_purpuratus
0,16
A0A0B6ZNM4_Arion_vulgaris
0,16
B3RX23_Trichoplax_adhaerens
0,82
C3XTJ2_Branchiostoma_floridae
0,9
F6W1A6_Ciona_intestinalis
T0QKT5_Saprolegnia_diclina
0,79
A0A067C3X5_Saprolegnia_parasitica
1
1
A0A067BP89_Saprolegnia_parasitica
0,99
A0A024TFR9_Aphanomyces_invadans
1
W4FL75_Aphanomyces_astaci
0,46
A0A0C7BBK6_Rhizopus_microsporus
0,98
A0A0A1NBL4_Rhizopus_microsporus
0,99
S2JNQ6_Mucor_circinelloides
0,98
0,98
U9TNM9_Rhizophagus_irregularis
1
A0A015LN76_Rhizophagus_irregularis
Fcan01_21782_PA
0,83
0,69
D7MKZ1_Arabidopsis_lyrata
0,98
V4N309_Eutrema_salsugineum
A0A087GFI4_Arabis_alpina
Q94K62_Arabidopsis_thaliana
Q8H121_Arabidopsis_thaliana
0.2

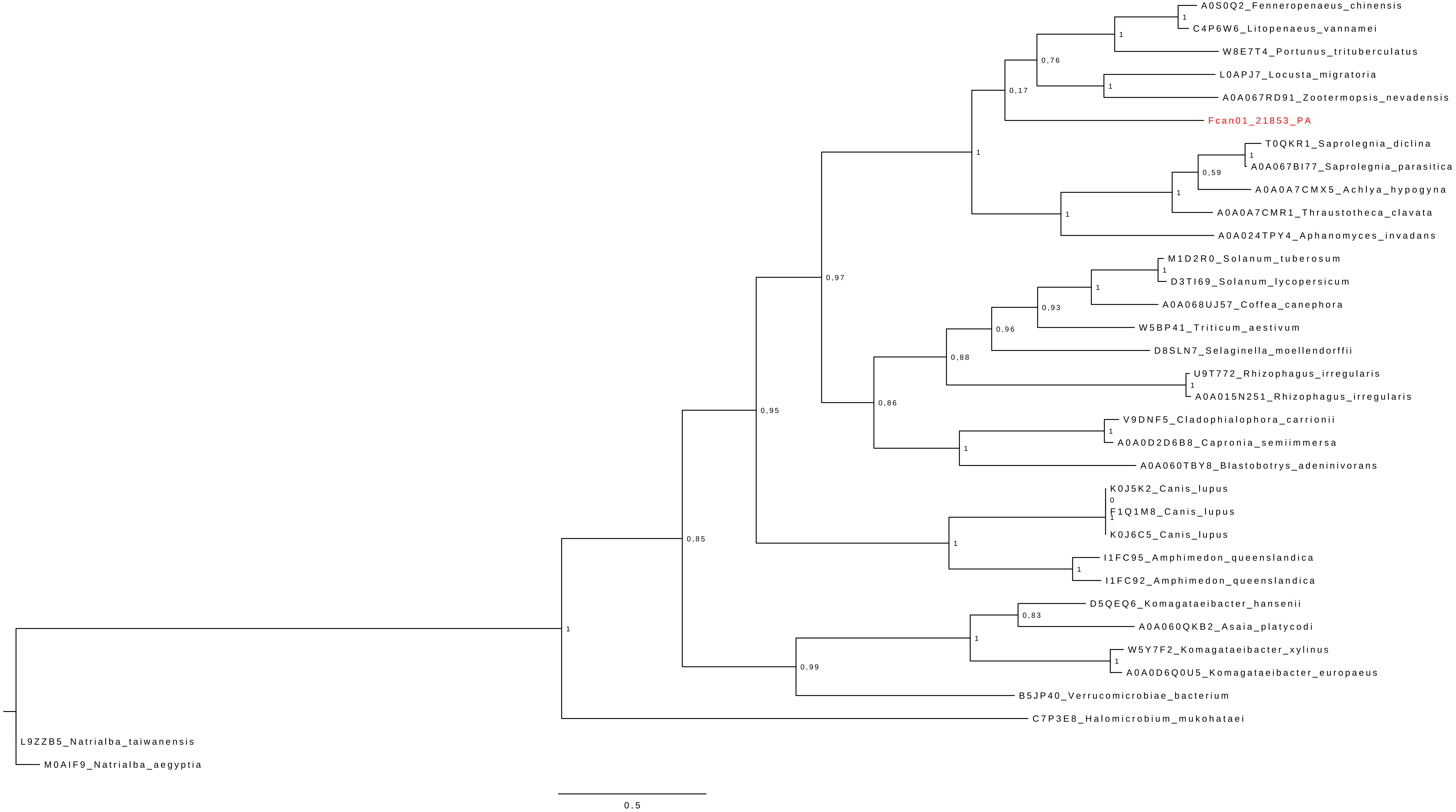
A0S0Q2_Fenneropenaeus_chinensis
C4P6W6_Litopenaeus_vannamei
W8E7T4_Portunus_trituberculatus
L0APJ7_Locusta_migratoria
A0A067RD91_Zootermopsis_nevadensis
Fcan01_21853_PA
T0QKR1_Saprolegnia_diclina
A0A067BI77_Saprolegnia_parasitica
A0A0A7CMX5_Achlya_hypogyna
A0A0A7CMR1_Thraustotheca_clavata
A0A024TPY4_Aphanomyces_invadans
M1D2R0_Solanum_tuberosum
D3TI69_Solanum_lycopersicum
A0A068UJ57_Coffea_canephora
W5BP41_Triticum_aestivum
D8SLN7_Selaginella_moellendorffii
U9T772_Rhizophagus_irregularis
A0A015N251_Rhizophagus_irregularis
V9DNF5_Cladophialophora_carrionii
A0A0D2D6B8_Capronia_semiimmersa
A0A060TBY8_Blastobotrys_adeninivorans
K0J5K2_Canis_lupus
F1Q1M8_Canis_lupus
K0J6C5_Canis_lupus
I1FC95_Amphimedon_queenslandica
I1FC92_Amphimedon_queenslandica
D5QEQ6_Komagataeibacter_hansenii
A0A060QKB2_Asaia_platycodi
W5Y7F2_Komagataeibacter_xylinus
A0A0D6Q0U5_Komagataeibacter_europaeus
B5JP40_Verrucomicrobiae_bacterium
C7P3E8_Halomicrobium_mukohataei
L9ZZB5_Natrialba_taiwanensis
M0AIF9_Natrialba_aegyptia
0,76
0,17
0,59
0,97
0,93
0,96
0,88
0,86
0,95
0,85
0,83
0,99
0.5

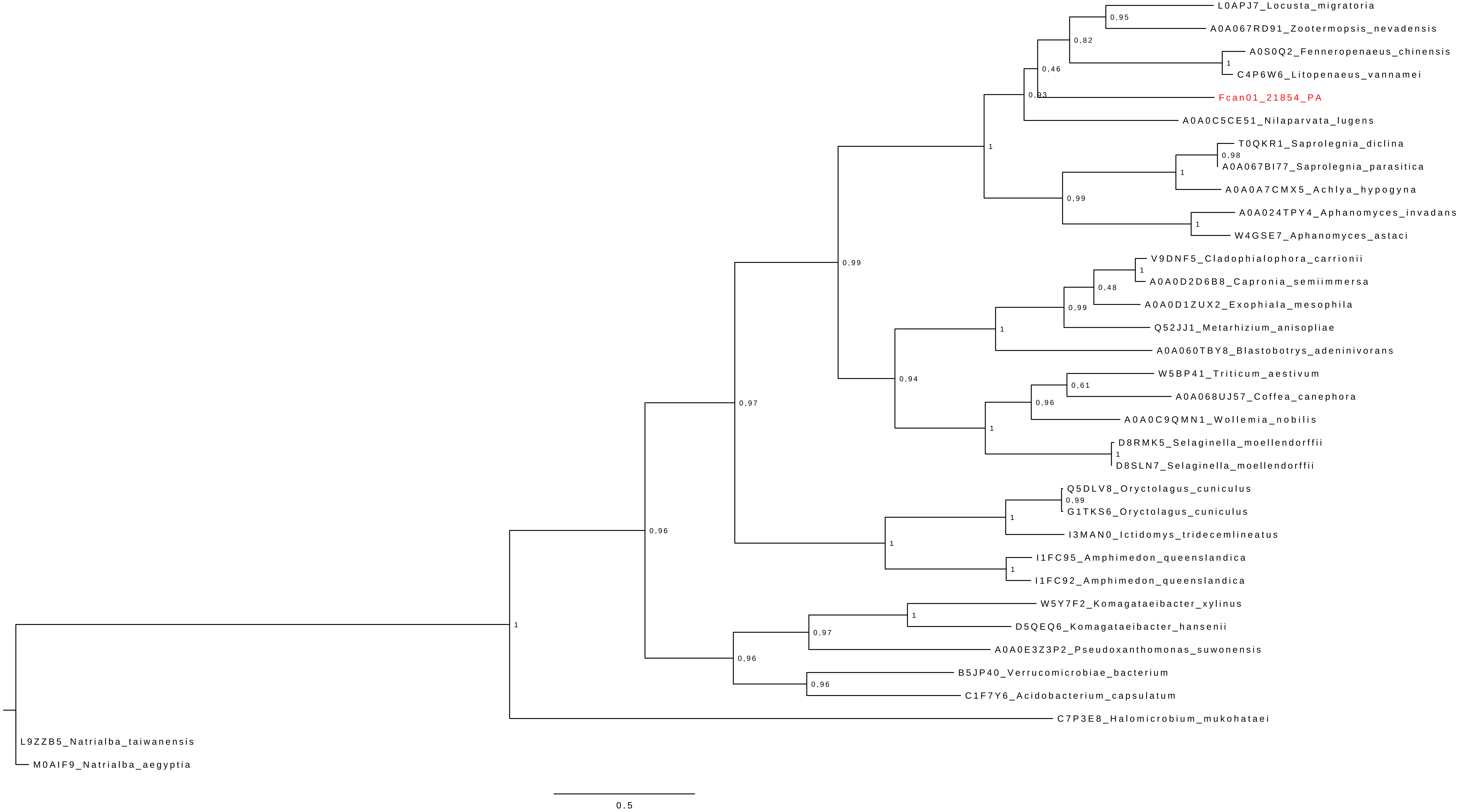
L0APJ7_Locusta_migratoria
0,95
A0A067RD91_Zootermopsis_nevadensis
0,82
A0S0Q2_Fenneropenaeus_chinensis
1
C4P6W6_Litopenaeus_vannamei
0,46
0,93
Fcan01_21854_PA
A0A0C5CE51_Nilaparvata_lugens
T0QKR1_Saprolegnia_diclina
0,98
A0A067BI77_Saprolegnia_parasitica
1
1
A0A0A7CMX5_Achlya_hypogyna
0,99
A0A024TPY4_Aphanomyces_invadans
1
W4GSE7_Aphanomyces_astaci
0,99
V9DNF5_Cladophialophora_carrionii
1
A0A0D2D6B8_Capronia_semiimmersa
0,48
A0A0D1ZUX2_Exophiala_mesophila
0,99
Q52JJ1_Metarhizium_anisopliae
1
A0A060TBY8_Blastobotrys_adeninivorans
W5BP41_Triticum_aestivum
0,61
A0A068UJ57_Coffea_canephora
0,94
0,96
A0A0C9QMN1_Wollemia_nobilis
1
D8RMK5_Selaginella_moellendorffii
1
D8SLN7_Selaginella_moellendorffii
0,97
Q5DLV8_Oryctolagus_cuniculus
0,99
G1TKS6_Oryctolagus_cuniculus
1
I3MAN0_Ictidomys_tridecemlineatus
1
I1FC95_Amphimedon_queenslandica
1
I1FC92_Amphimedon_queenslandica
0,96
W5Y7F2_Komagataeibacter_xylinus
1
D5QEQ6_Komagataeibacter_hansenii
0,97
A0A0E3Z3P2_Pseudoxanthomonas_suwonensis
0,96
B5JP40_Verrucomicrobiae_bacterium
0,96
C1F7Y6_Acidobacterium_capsulatum
1
C7P3E8_Halomicrobium_mukohataei
L9ZZB5_Natrialba_taiwanensis
M0AIF9_Natrialba_aegyptia
0.5

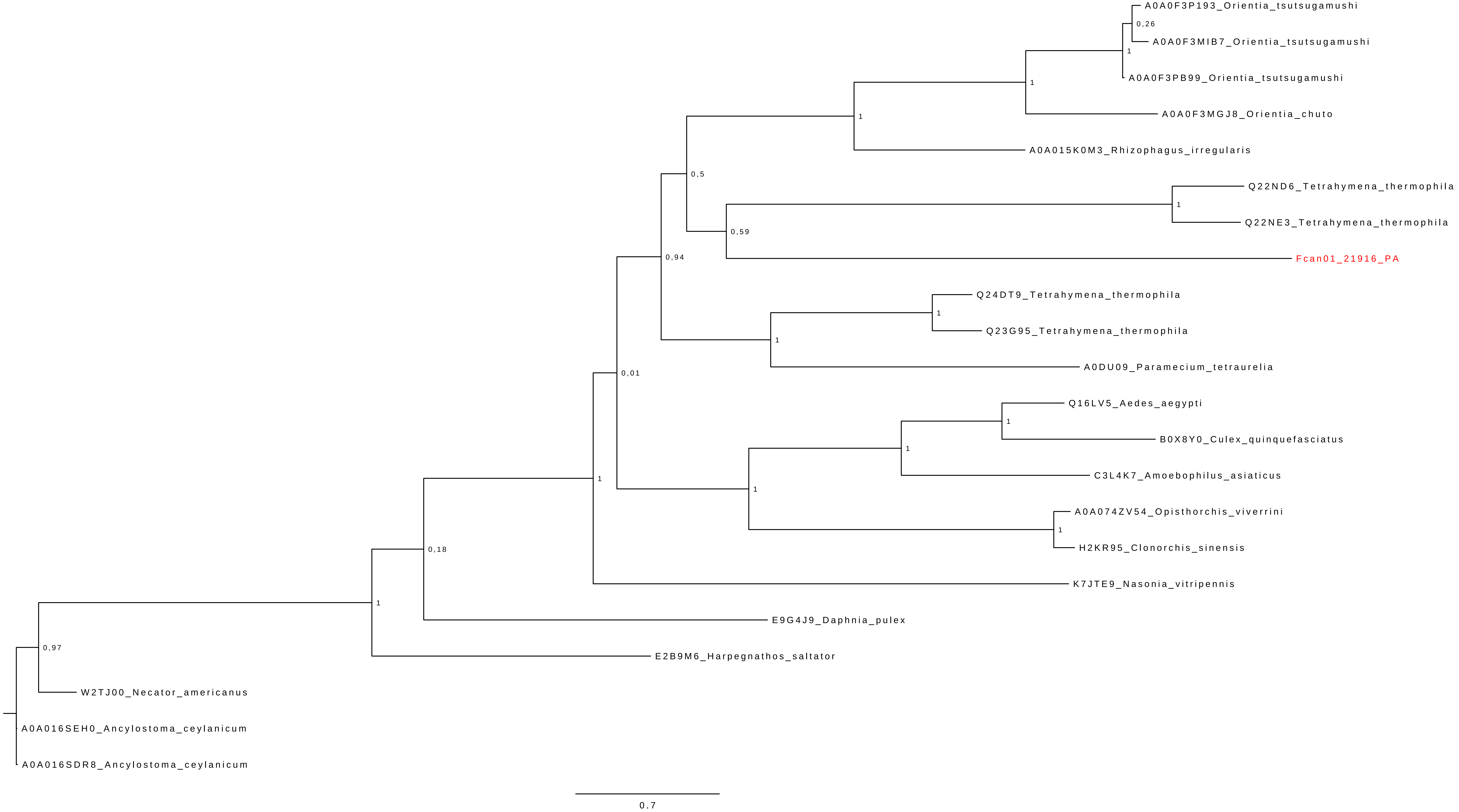
A0A0F3P193_Orientia_tsutsugamushi
0,26
A0A0F3MIB7_Orientia_tsutsugamushi
1
A0A0F3PB99_Orientia_tsutsugamushi
1
A0A0F3MGJ8_Orientia_chuto
1
A0A015K0M3_Rhizophagus_irregularis
0,5
Q22ND6_Tetrahymena_thermophila
1
Q22NE3_Tetrahymena_thermophila
0,59
Fcan01_21916_PA
0,94
Q24DT9_Tetrahymena_thermophila
1
Q23G95_Tetrahymena_thermophila
1
A0DU09_Paramecium_tetraurelia
0,01
Q16LV5_Aedes_aegypti
1
B0X8Y0_Culex_quinquefasciatus
1
C3L4K7_Amoebophilus_asiaticus
1
1
A0A074ZV54_Opisthorchis_viverrini
1
H2KR95_Clonorchis_sinensis
K7JTE9_Nasonia_vitripennis
0,18
1
E9G4J9_Daphnia_pulex
0,97
E2B9M6_Harpegnathos_saltator
W2TJ00_Necator_americanus
A0A016SEH0_Ancylostoma_ceylanicum
A0A016SDR8_Ancylostoma_ceylanicum
0.7

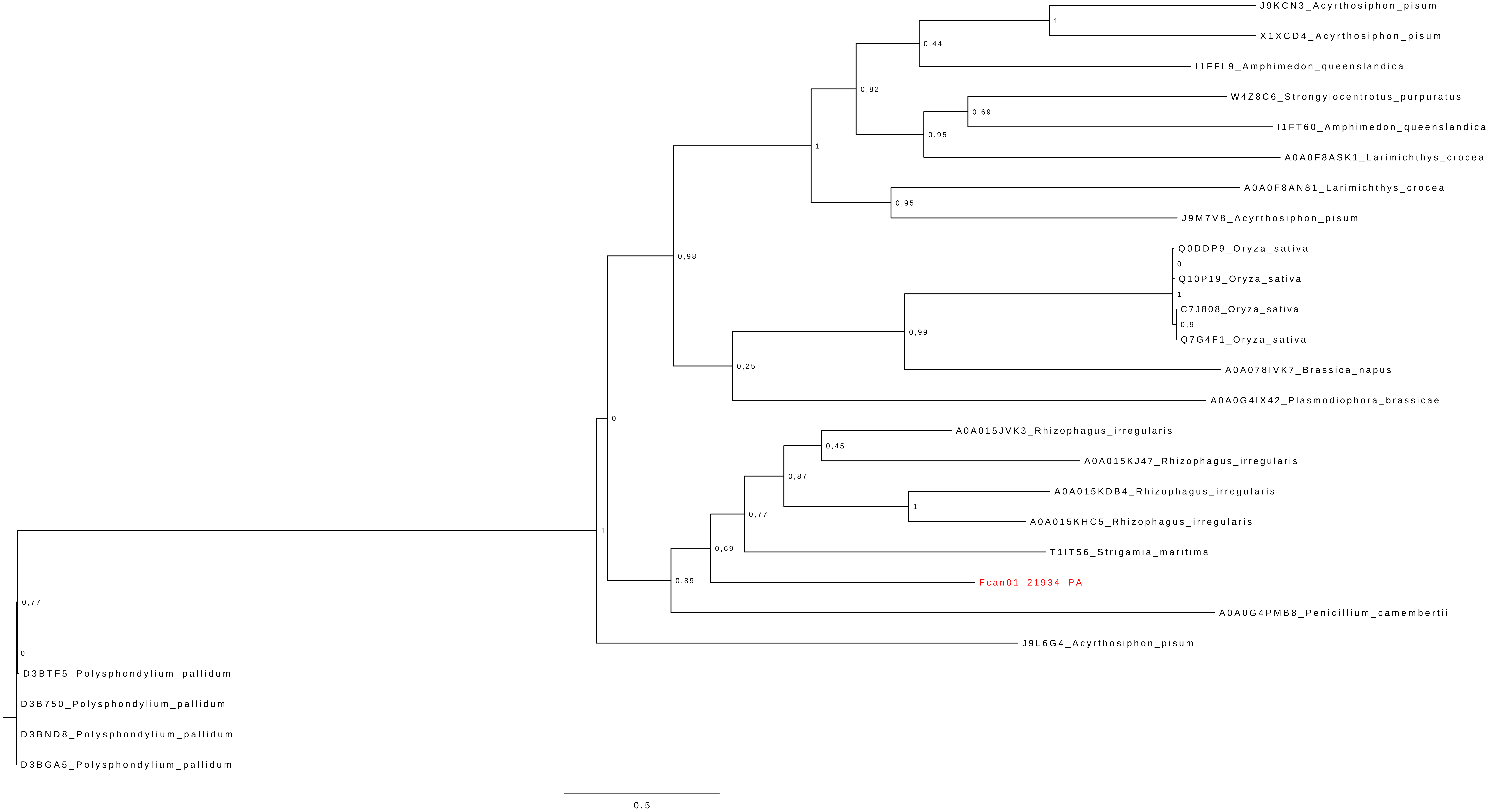
J9KCN3_Acyrthosiphon_pisum
X1XCD4_Acyrthosiphon_pisum
I1FFL9_Amphimedon_queenslandica
W4Z8C6_Strongylocentrotus_purpuratus
I1FT60_Amphimedon_queenslandica
A0A0F8ASK1_Larimichthys_crocea
A0A0F8AN81_Larimichthys_crocea
J9M7V8_Acyrthosiphon_pisum
Q0DDP9_Oryza_sativa
Q10P19_Oryza_sativa
C7J808_Oryza_sativa
Q7G4F1_Oryza_sativa
A0A078IVK7_Brassica_napus
A0A0G4IX42_Plasmodiophora_brassicae
A0A015JVK3_Rhizophagus_irregularis
A0A015KJ47_Rhizophagus_irregularis
A0A015KDB4_Rhizophagus_irregularis
A0A015KHC5_Rhizophagus_irregularis
T1IT56_Strigamia_maritima
Fcan01_21934_PA
A0A0G4PMB8_Penicillium_camembertii
J9L6G4_Acyrthosiphon_pisum
D3BTF5_Polysphondylium_pallidum
D3B750_Polysphondylium_pallidum
D3BND8_Polysphondylium_pallidum
D3BGA5_Polysphondylium_pallidum
1
0,44
0,82
0,69
0,95
1
0,95
0,98
0
1
0,9
0,99
0,25
0
0,45
0,87
1
0,77
0,69
0,89
1
0,77
0
0.5

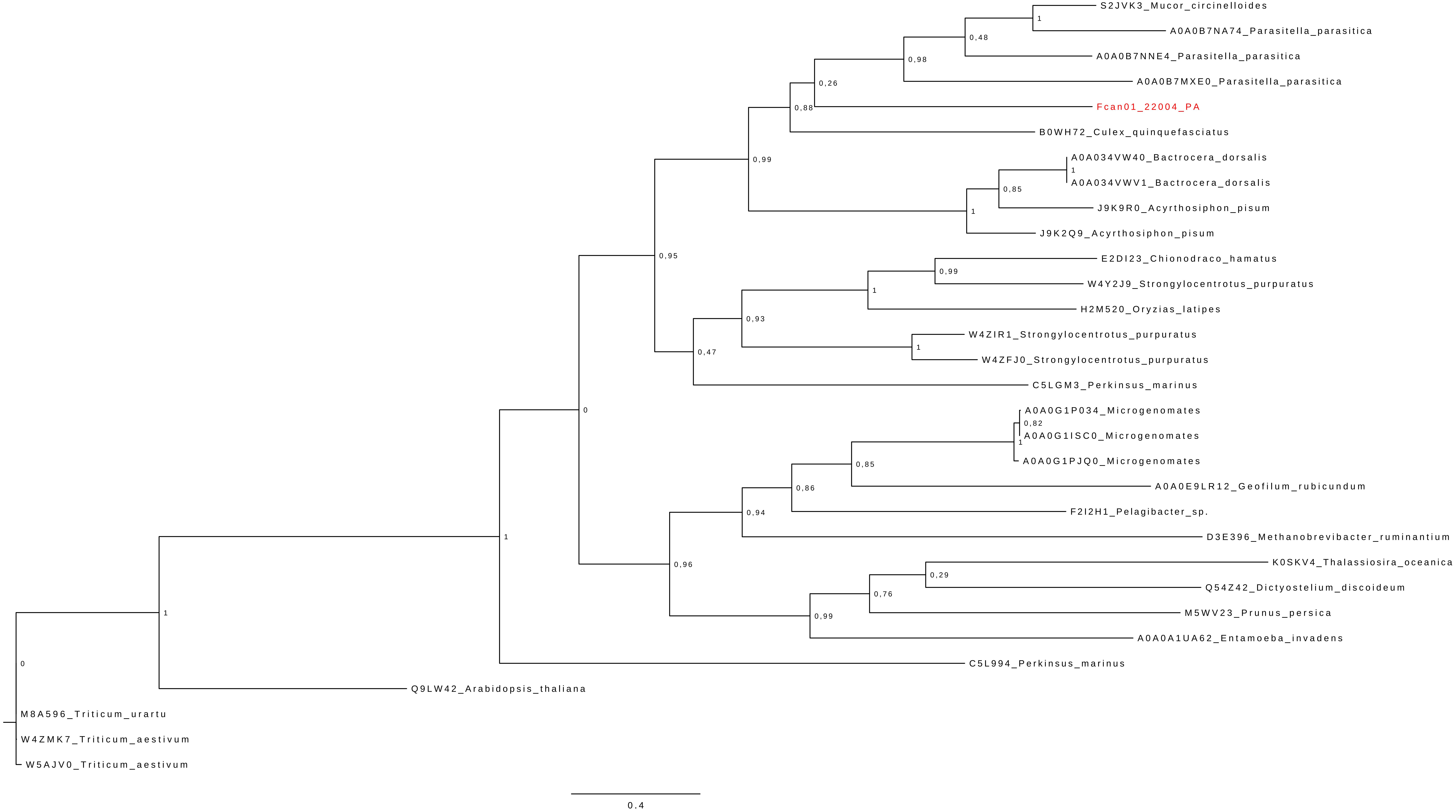
S2JVK3_Mucor_circinelloides
A0A0B7NA74_Parasitella_parasitica
A0A0B7NNE4_Parasitella_parasitica
A0A0B7MXE0_Parasitella_parasitica
Fcan01_22004_PA
B0WH72_Culex_quinquefasciatus
A0A034VW40_Bactrocera_dorsalis
A0A034VWV1_Bactrocera_dorsalis
J9K9R0_Acyrthosiphon_pisum
J9K2Q9_Acyrthosiphon_pisum
E2DI23_Chionodraco_hamatus
W4Y2J9_Strongylocentrotus_purpuratus
H2M520_Oryzias_latipes
W4ZIR1_Strongylocentrotus_purpuratus
W4ZFJ0_Strongylocentrotus_purpuratus
C5LGM3_Perkinsus_marinus
A0A0G1P034_Microgenomates
A0A0G1ISC0_Microgenomates
A0A0G1PJQ0_Microgenomates
A0A0E9LR12_Geofilum_rubicundum
F2I2H1_Pelagibacter_sp.
D3E396_Methanobrevibacter_ruminantium
K0SKV4_Thalassiosira_oceanica
Q54Z42_Dictyostelium_discoideum
M5WV23_Prunus_persica
A0A0A1UA62_Entamoeba_invadens
C5L994_Perkinsus_marinus
Q9LW42_Arabidopsis_thaliana
M8A596_Triticum_urartu
W4ZMK7_Triticum_aestivum
W5AJV0_Triticum_aestivum
1
0,48
0,98
0,26
0,88
0,99
1
0,85
1
0,95
0,99
1
0,93
1
0,47
0
0,82
1
0,85
0,86
0,94
1
0,96
0,29
0,76
0,99
1
0
0.4

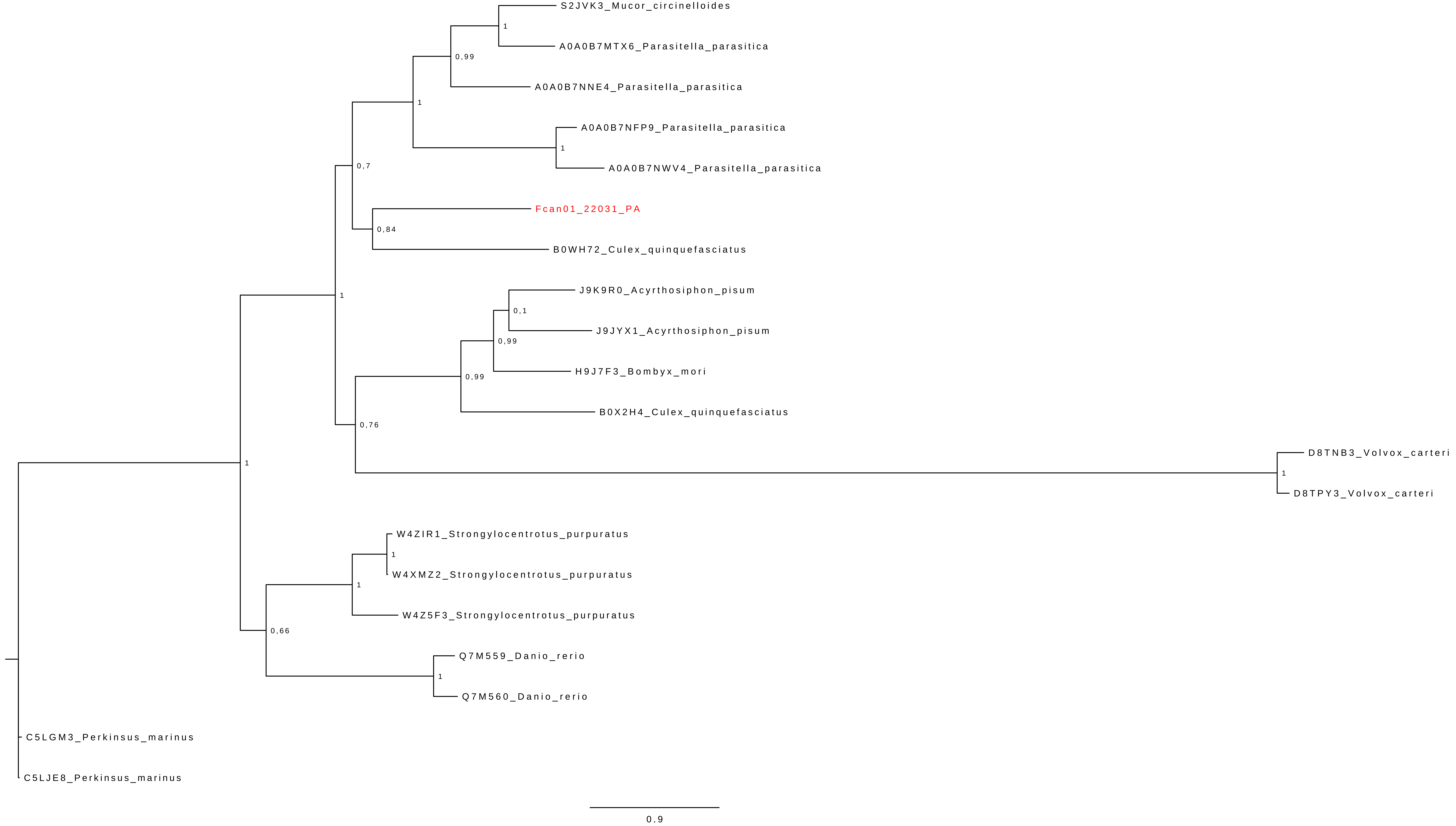
S2JVK3_Mucor_circinelloides
A0A0B7MTX6_Parasitella_parasitica
A0A0B7NNE4_Parasitella_parasitica
A0A0B7NFP9_Parasitella_parasitica
A0A0B7NWV4_Parasitella_parasitica
Fcan01_22031_PA
B0WH72_Culex_quinquefasciatus
J9K9R0_Acyrthosiphon_pisum
J9JYX1_Acyrthosiphon_pisum
H9J7F3_Bombyx_mori
B0X2H4_Culex_quinquefasciatus
D8TNB3_Volvox_carteri
D8TPY3_Volvox_carteri
W4ZIR1_Strongylocentrotus_purpuratus
W4XMZ2_Strongylocentrotus_purpuratus
W4Z5F3_Strongylocentrotus_purpuratus
Q7M559_Danio_rerio
Q7M560_Danio_rerio
C5LGM3_Perkinsus_marinus
C5LJE8_Perkinsus_marinus
1
0,99
1
1
0,7
0,84
1
0,1
0,99
0,99
0,76
1
1
1
1
0,66
1
0.9

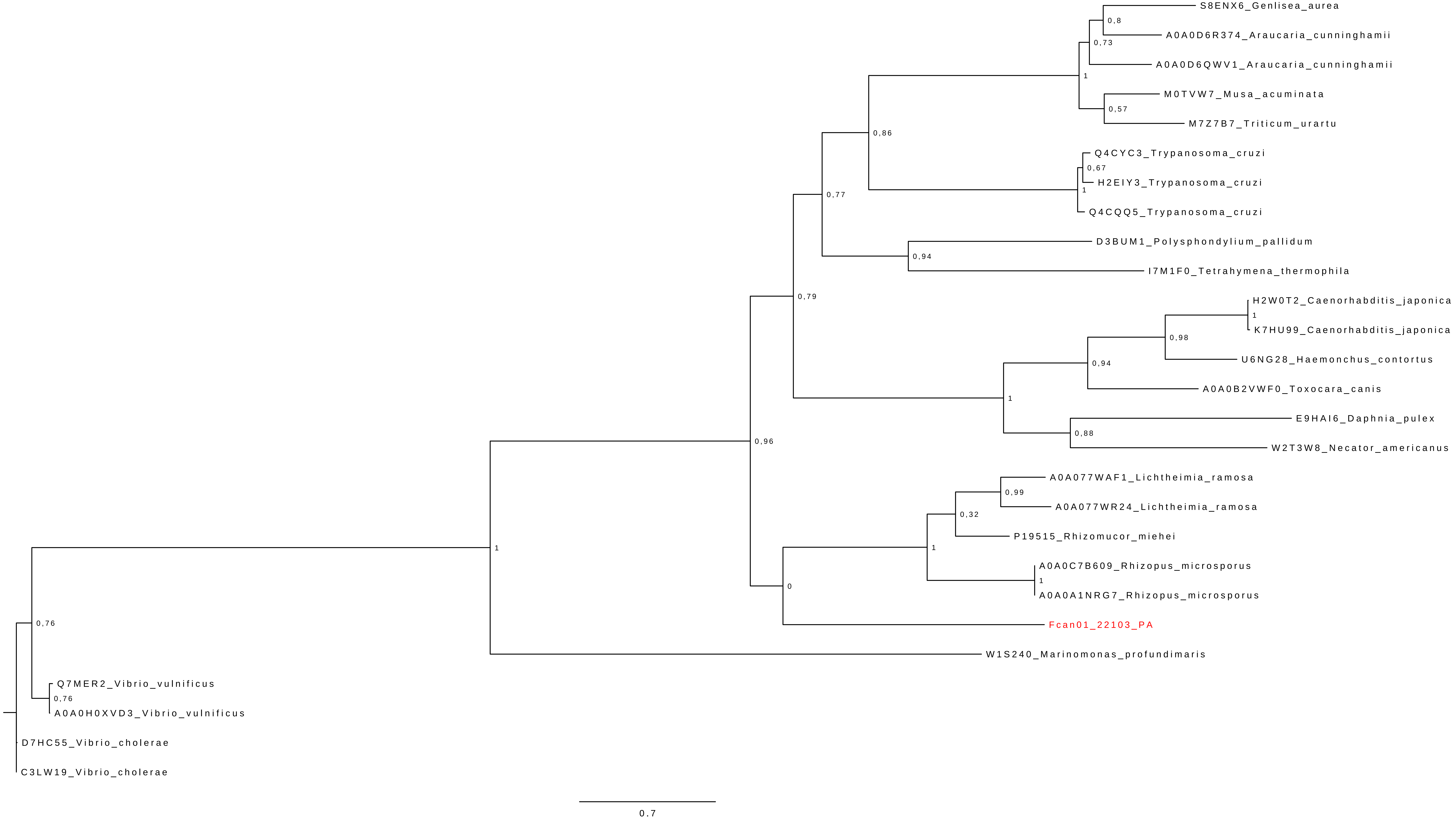
S8ENX6_Genlisea_aurea
0,8
A0A0D6R374_Araucaria_cunninghamii
0,73
A0A0D6QWV1_Araucaria_cunninghamii
1
M0TVW7_Musa_acuminata
0,57
M7Z7B7_Triticum_urartu
0,86
Q4CYC3_Trypanosoma_cruzi
0,67
H2EIY3_Trypanosoma_cruzi
1
Q4CQQ5_Trypanosoma_cruzi
0,77
D3BUM1_Polysphondylium_pallidum
0,94
I7M1F0_Tetrahymena_thermophila
0,79
H2W0T2_Caenorhabditis_japonica
1
K7HU99_Caenorhabditis_japonica
0,98
U6NG28_Haemonchus_contortus
0,94
A0A0B2VWF0_Toxocara_canis
1
E9HAI6_Daphnia_pulex
0,88
W2T3W8_Necator_americanus
0,96
A0A077WAF1_Lichtheimia_ramosa
0,99
A0A077WR24_Lichtheimia_ramosa
0,32
P19515_Rhizomucor_miehei
1
A0A0C7B609_Rhizopus_microsporus
1
A0A0A1NRG7_Rhizopus_microsporus
0
Fcan01_22103_PA
1
W1S240_Marinomonas_profundimaris
0,76
Q7MER2_Vibrio_vulnificus
0,76
A0A0H0XVD3_Vibrio_vulnificus
D7HC55_Vibrio_cholerae
C3LW19_Vibrio_cholerae
0.7

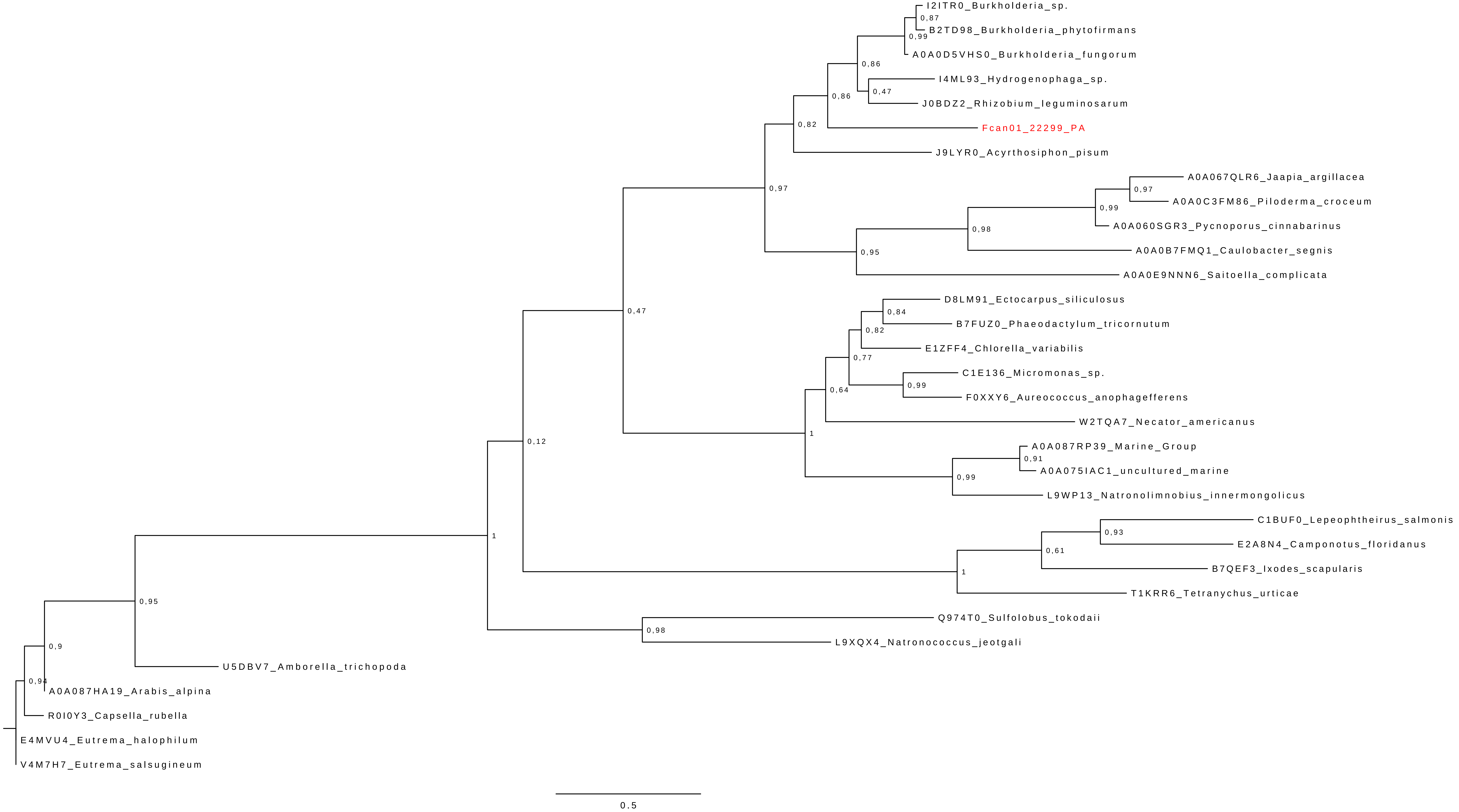

I2ITR0_Burkholderia_sp.
B2TD98_Burkholderia_phytofirmans
A0A0D5VHS0_Burkholderia_fungorum
I4ML93_Hydrogenophaga_sp.
J0BDZ2_Rhizobium_leguminosarum
Fcan01_22299_PA
J9LYR0_Acyrthosiphon_pisum
A0A067QLR6_Jaapia_argillacea
A0A0C3FM86_Piloderma_croceum
A0A060SGR3_Pycnoporus_cinnabarinus
A0A0B7FMQ1_Caulobacter_segnis
A0A0E9NNN6_Saitoella_complicata
D8LM91_Ectocarpus_siliculosus
B7FUZ0_Phaeodactylum_tricornutum
E1ZFF4_Chlorella_variabilis
C1E136_Micromonas_sp.
F0XXY6_Aureococcus_anophagefferens
W2TQA7_Necator_americanus
A0A087RP39_Marine_Group
A0A075IAC1_uncultured_marine
L9WP13_Natronolimnobius_innermongolicus
C1BUF0_Lepeophtheirus_salmonis
E2A8N4_Camponotus_floridanus
B7QEF3_Ixodes_scapularis
T1KRR6_Tetranychus_urticae
Q974T0_Sulfolobus_tokodaii
L9XQX4_Natronococcus_jeotgali
U5DBV7_Amborella_trichopoda
A0A087HA19_Arabis_alpina
R0I0Y3_Capsella_rubella
E4MVU4_Eutrema_halophilum
V4M7H7_Eutrema_salsugineum
0,87
0,99
0,86
0,47
0,86
0,82
0,97
0,97
0,99
0,98
0,95
0,47
0,84
0,82
0,77
0,99
0,64
1
0,91
0,99
0,12
0,93
0,61
1
1
0,98
0,95
0,9
0,94
0.5

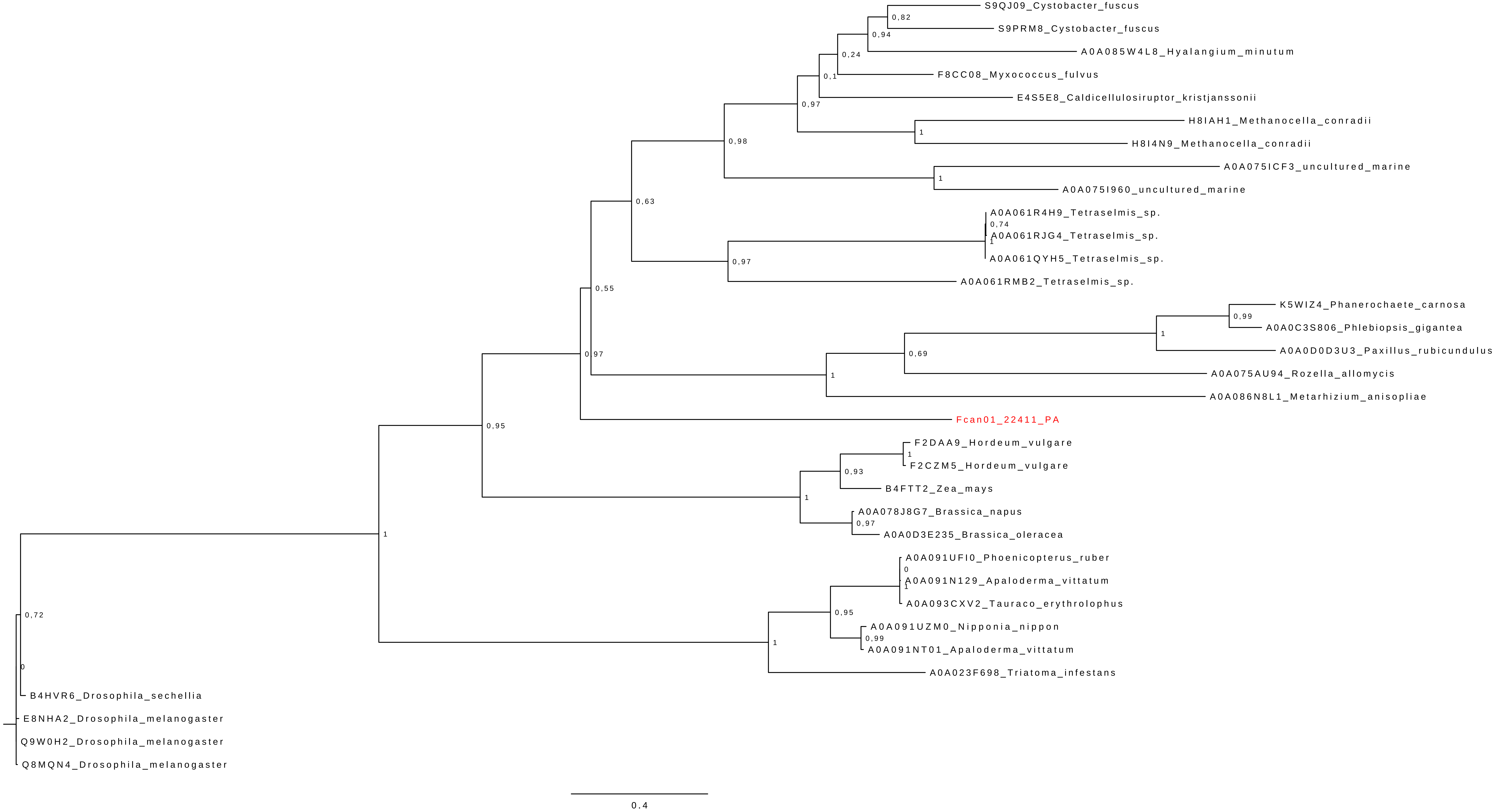
S9QJ09_Cystobacter_fuscus
0,82
S9PRM8_Cystobacter_fuscus
0,94
A0A085W4L8_Hyalangium_minutum
0,24
F8CC08_Myxococcus_fulvus
0,1
E4S5E8_Caldicellulosiruptor_kristjanssonii
0,97
H8IAH1_Methanocella_conradii
1
H8I4N9_Methanocella_conradii
0,98
A0A075ICF3_uncultured_marine
1
A0A075I960_uncultured_marine
0,63
A0A061R4H9_Tetraselmis_sp.
0,74
A0A061RJG4_Tetraselmis_sp.
1
A0A061QYH5_Tetraselmis_sp.
0,97
A0A061RMB2_Tetraselmis_sp.
0,55
K5WIZ4_Phanerochaete_carnosa
0,99
A0A0C3S806_Phlebiopsis_gigantea
1
A0A0D0D3U3_Paxillus_rubicundulus
0,69
A0A075AU94_Rozella_allomycis
1
A0A086N8L1_Metarhizium_anisopliae
0,97
Fcan01_22411_PA
0,95
F2DAA9_Hordeum_vulgare
1
F2CZM5_Hordeum_vulgare
0,93
B4FTT2_Zea_mays
1
A0A078J8G7_Brassica_napus
0,97
A0A0D3E235_Brassica_oleracea
1
A0A091UFI0_Phoenicopterus_ruber
0
A0A091N129_Apaloderma_vittatum
1
A0A093CXV2_Tauraco_erythrolophus
0,95
A0A091UZM0_Nipponia_nippon
0,99
A0A091NT01_Apaloderma_vittatum
1
A0A023F698_Triatoma_infestans
0,72
0
B4HVR6_Drosophila_sechellia
E8NHA2_Drosophila_melanogaster
Q9W0H2_Drosophila_melanogaster
Q8MQN4_Drosophila_melanogaster
0.4

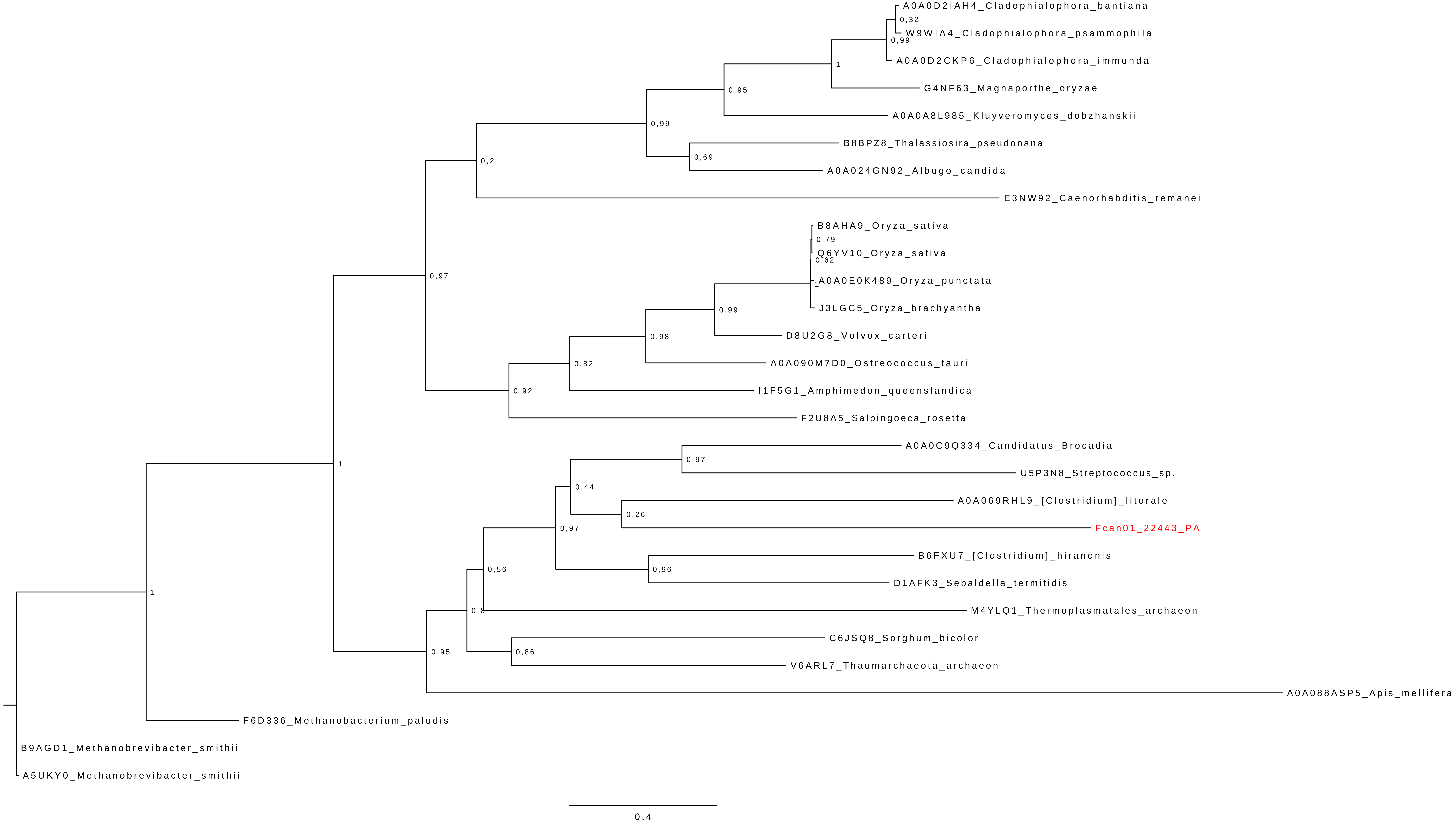
A0A0D2IAH4_Cladophialophora_bantiana
0,32
W9WIA4_Cladophialophora_psammophila
0,99
A0A0D2CKP6_Cladophialophora_immunda
1
G4NF63_Magnaporthe_oryzae
0,95
A0A0A8L985_Kluyveromyces_dobzhanskii
0,99
B8BPZ8_Thalassiosira_pseudonana
0,69
A0A024GN92_Albugo_candida
0,2
E3NW92_Caenorhabditis_remanei
B8AHA9_Oryza_sativa
0,79
Q6YV10_Oryza_sativa
0,62
A0A0E0K489_Oryza_punctata
1
J3LGC5_Oryza_brachyantha
0,99
D8U2G8_Volvox_carteri
0,98
A0A090M7D0_Ostreococcus_tauri
0,82
I1F5G1_Amphimedon_queenslandica
0,92
F2U8A5_Salpingoeca_rosetta
0,97
A0A0C9Q334_Candidatus_Brocadia
0,97
U5P3N8_Streptococcus_sp.
0,44
A0A069RHL9_[Clostridium]_litorale
0,26
Fcan01_22443_PA
0,97
B6FXU7_[Clostridium]_hiranonis
0,96
D1AFK3_Sebaldella_termitidis
0,56
M4YLQ1_Thermoplasmatales_archaeon
0,8
C6JSQ8_Sorghum_bicolor
0,86
V6ARL7_Thaumarchaeota_archaeon
0,95
A0A088ASP5_Apis_mellifera
1
1
F6D336_Methanobacterium_paludis
B9AGD1_Methanobrevibacter_smithii
A5UKY0_Methanobrevibacter_smithii
0.4

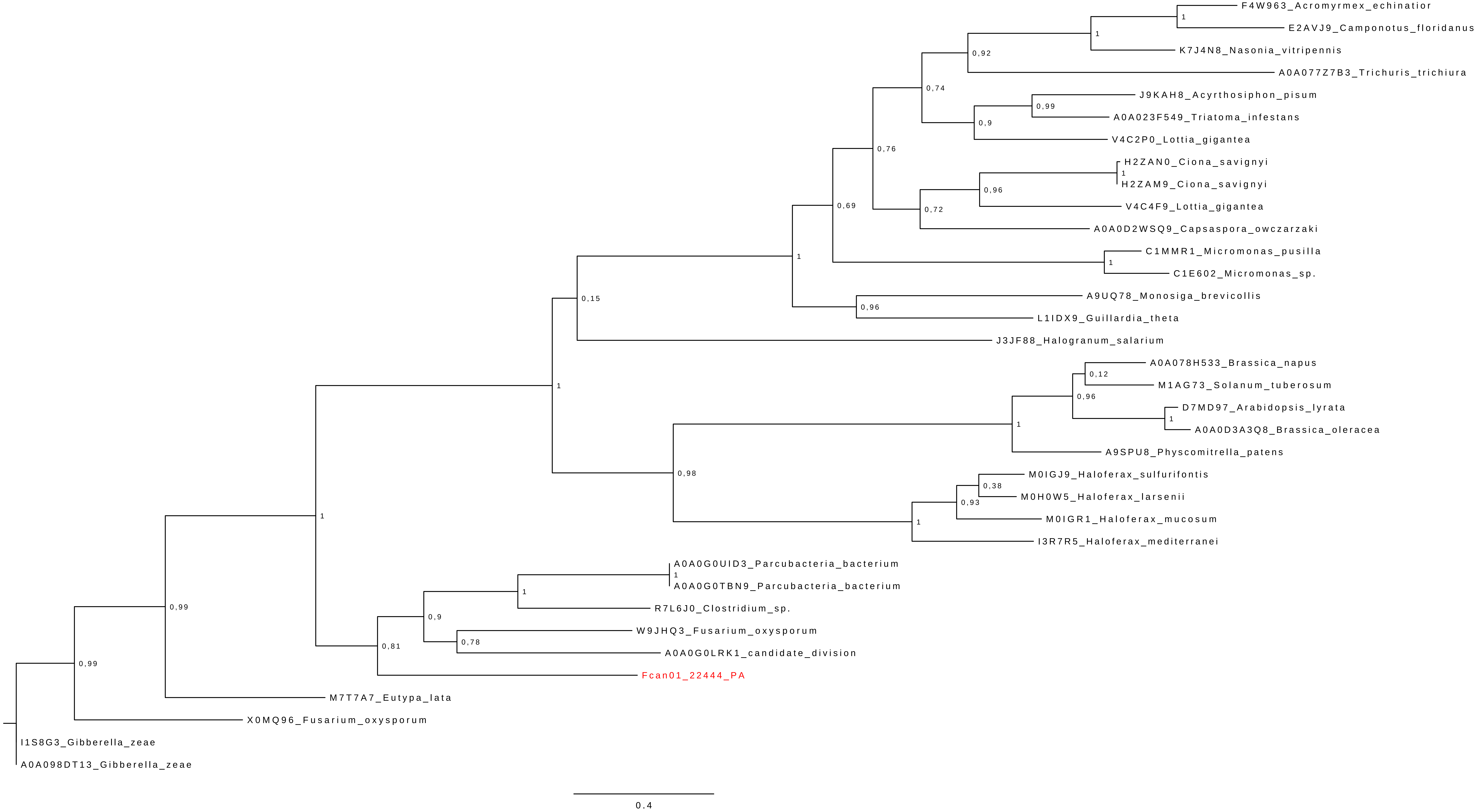
F4W963_Acromyrmex_echinatior
E2AVJ9_Camponotus_floridanus
K7J4N8_Nasonia_vitripennis
A0A077Z7B3_Trichuris_trichiura
J9KAH8_Acyrthosiphon_pisum
A0A023F549_Triatoma_infestans
V4C2P0_Lottia_gigantea
H2ZAN0_Ciona_savignyi
H2ZAM9_Ciona_savignyi
V4C4F9_Lottia_gigantea
A0A0D2WSQ9_Capsaspora_owczarzaki
C1MMR1_Micromonas_pusilla
C1E602_Micromonas_sp.
A9UQ78_Monosiga_brevicollis
L1IDX9_Guillardia_theta
J3JF88_Halogranum_salarium
A0A078H533_Brassica_napus
M1AG73_Solanum_tuberosum
D7MD97_Arabidopsis_lyrata
A0A0D3A3Q8_Brassica_oleracea
A9SPU8_Physcomitrella_patens
M0IGJ9_Haloferax_sulfurifontis
M0H0W5_Haloferax_larsenii
M0IGR1_Haloferax_mucosum
I3R7R5_Haloferax_mediterranei
A0A0G0UID3_Parcubacteria_bacterium
A0A0G0TBN9_Parcubacteria_bacterium
R7L6J0_Clostridium_sp.
W9JHQ3_Fusarium_oxysporum
A0A0G0LRK1_candidate_division
Fcan01_22444_PA
M7T7A7_Eutypa_lata
X0MQ96_Fusarium_oxysporum
I1S8G3_Gibberella_zeae
A0A098DT13_Gibberella_zeae
1
0,92
0,74
0,99
0,9
0,96
0,72
0,76
0,69
0,96
0,15
0,12
0,98
0,38
0,93
0,9
0,78
0,81
0,99
0,99
0.4

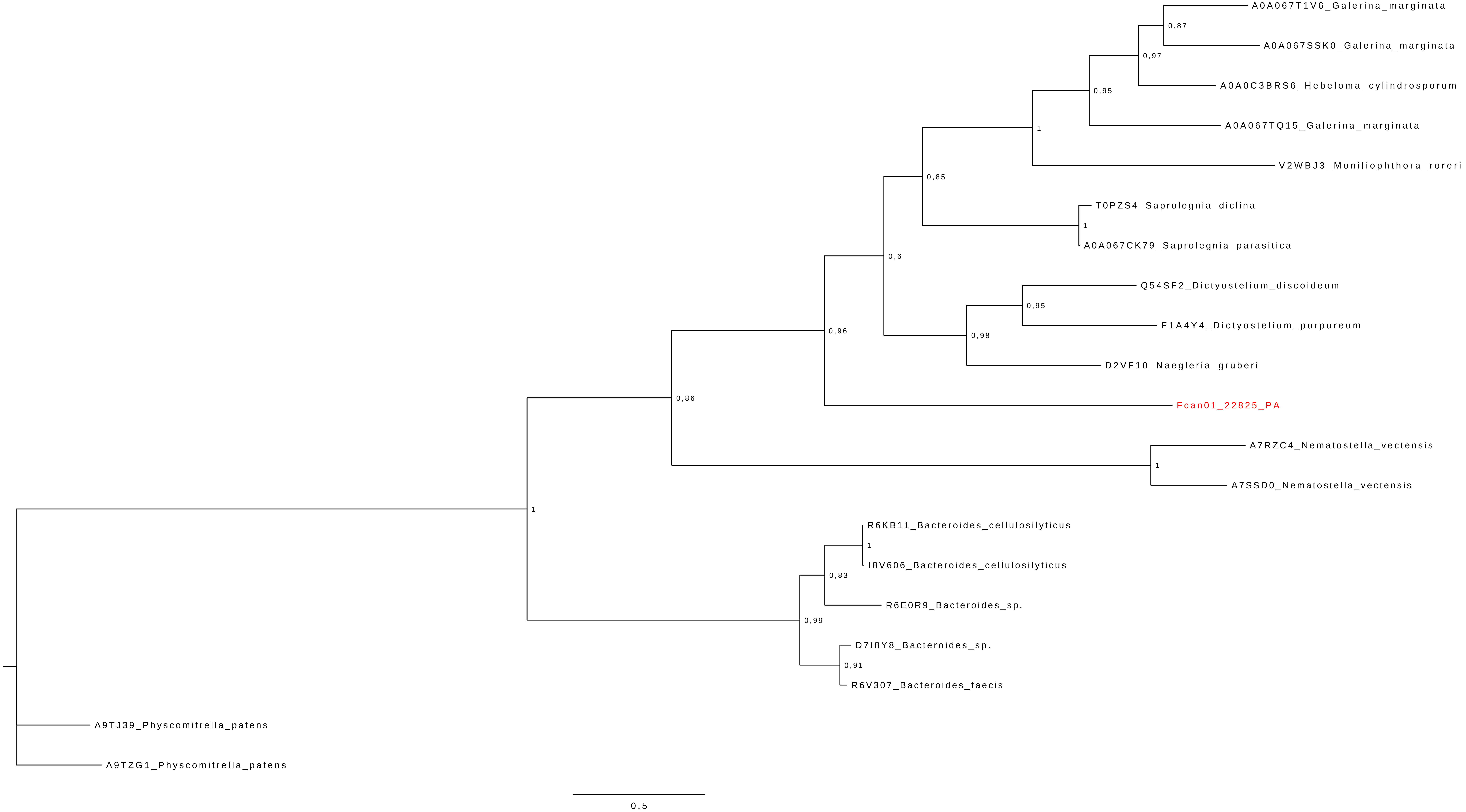
A0A067T1V6_Galerina_marginata
0,87
A0A067SSK0_Galerina_marginata
0,97
A0A0C3BRS6_Hebeloma_cylindrosporum
0,95
A0A067TQ15_Galerina_marginata
1
V2WBJ3_Moniliophthora_roreri
0,85
T0PZS4_Saprolegnia_diclina
1
A0A067CK79_Saprolegnia_parasitica
0,6
Q54SF2_Dictyostelium_discoideum
0,95
F1A4Y4_Dictyostelium_purpureum
0,98
D2VF10_Naegleria_gruberi
0,96
Fcan01_22825_PA
0,86
A7RZC4_Nematostella_vectensis
1
A7SSD0_Nematostella_vectensis
1
R6KB11_Bacteroides_cellulosilyticus
1
I8V606_Bacteroides_cellulosilyticus
0,83
R6E0R9_Bacteroides_sp.
0,99
D7I8Y8_Bacteroides_sp.
0,91
R6V307_Bacteroides_faecis
A9TJ39_Physcomitrella_patens
A9TZG1_Physcomitrella_patens
0.5

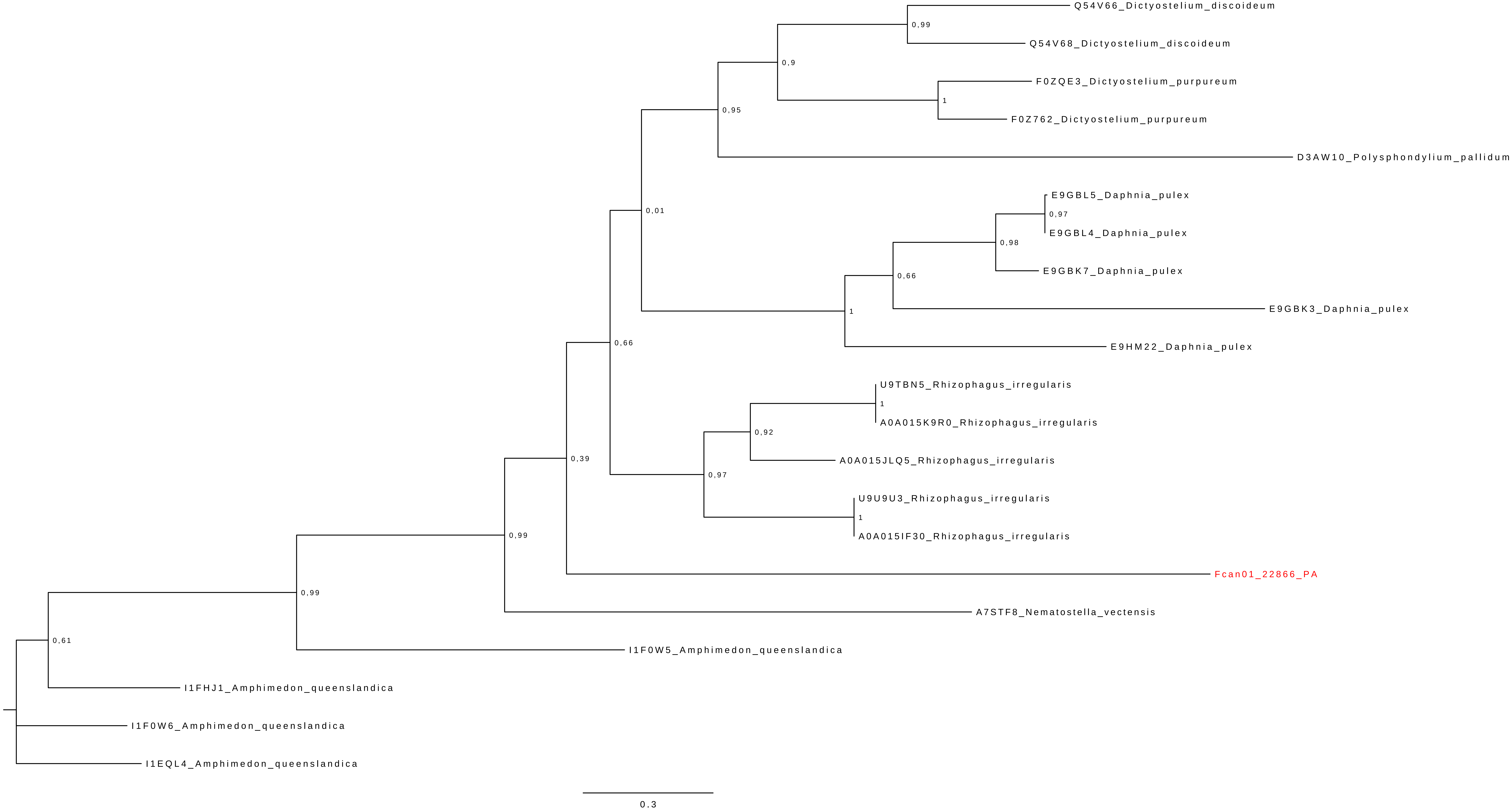
Q54V66_Dictyostelium_discoideum
0,99
Q54V68_Dictyostelium_discoideum
0,9
F0ZQE3_Dictyostelium_purpureum
1
F0Z762_Dictyostelium_purpureum
0,95
D3AW10_Polysphondylium_pallidum
E9GBL5_Daphnia_pulex
0,97
E9GBL4_Daphnia_pulex
0,98
0,01
E9GBK7_Daphnia_pulex
0,66
E9GBK3_Daphnia_pulex
1
0,66
E9HM22_Daphnia_pulex
U9TBN5_Rhizophagus_irregularis
1
A0A015K9R0_Rhizophagus_irregularis
0,92
0,39
A0A015JLQ5_Rhizophagus_irregularis
0,97
U9U9U3_Rhizophagus_irregularis
1
0,99
A0A015IF30_Rhizophagus_irregularis
Fcan01_22866_PA
0,99
A7STF8_Nematostella_vectensis
0,61
I1F0W5_Amphimedon_queenslandica
I1FHJ1_Amphimedon_queenslandica
I1F0W6_Amphimedon_queenslandica
I1EQL4_Amphimedon_queenslandica
0.3

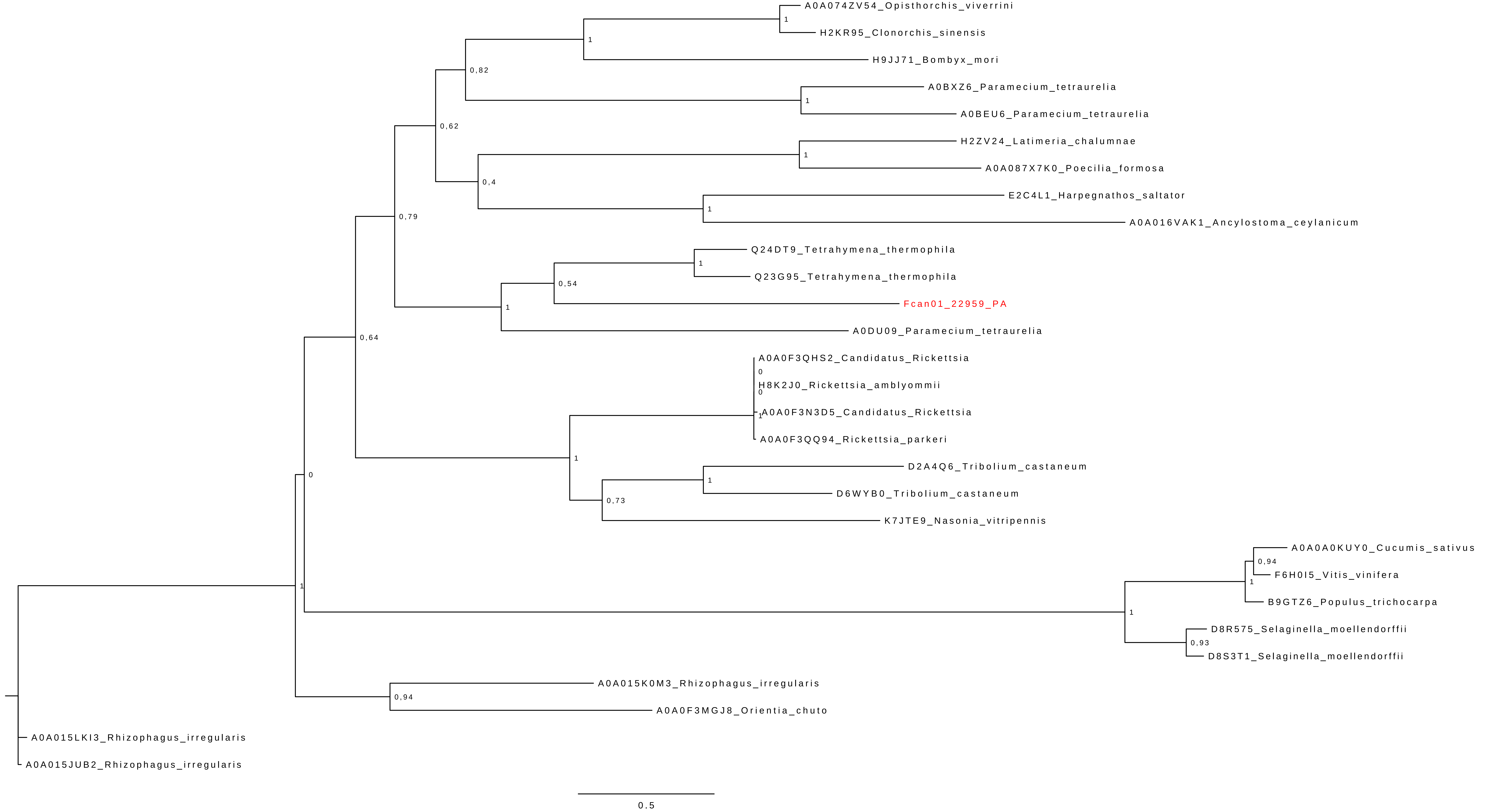
A0A074ZV54_Opisthorchis_viverrini
H2KR95_Clonorchis_sinensis
H9JJ71_Bombyx_mori
A0BXZ6_Paramecium_tetraurelia
A0BEU6_Paramecium_tetraurelia
H2ZV24_Latimeria_chalumnae
A0A087X7K0_Poecilia_formosa
E2C4L1_Harpegnathos_saltator
A0A016VAK1_Ancylostoma_ceylanicum
Q24DT9_Tetrahymena_thermophila
Q23G95_Tetrahymena_thermophila
Fcan01_22959_PA
A0DU09_Paramecium_tetraurelia
A0A0F3QHS2_Candidatus_Rickettsia
H8K2J0_Rickettsia_amblyommii
A0A0F3N3D5_Candidatus_Rickettsia
A0A0F3QQ94_Rickettsia_parkeri
D2A4Q6_Tribolium_castaneum
D6WYB0_Tribolium_castaneum
K7JTE9_Nasonia_vitripennis
A0A0A0KUY0_Cucumis_sativus
F6H0I5_Vitis_vinifera
B9GTZ6_Populus_trichocarpa
D8R575_Selaginella_moellendorffii
D8S3T1_Selaginella_moellendorffii
A0A015K0M3_Rhizophagus_irregularis
A0A0F3MGJ8_Orientia_chuto
A0A015LKI3_Rhizophagus_irregularis
A0A015JUB2_Rhizophagus_irregularis
1
1
0,82
1
0,62
1
0,4
1
0,79
1
0,54
1
0,64
0
0
1
1
1
0,73
0
0,94
1
1
0,93
1
0,94
0.5

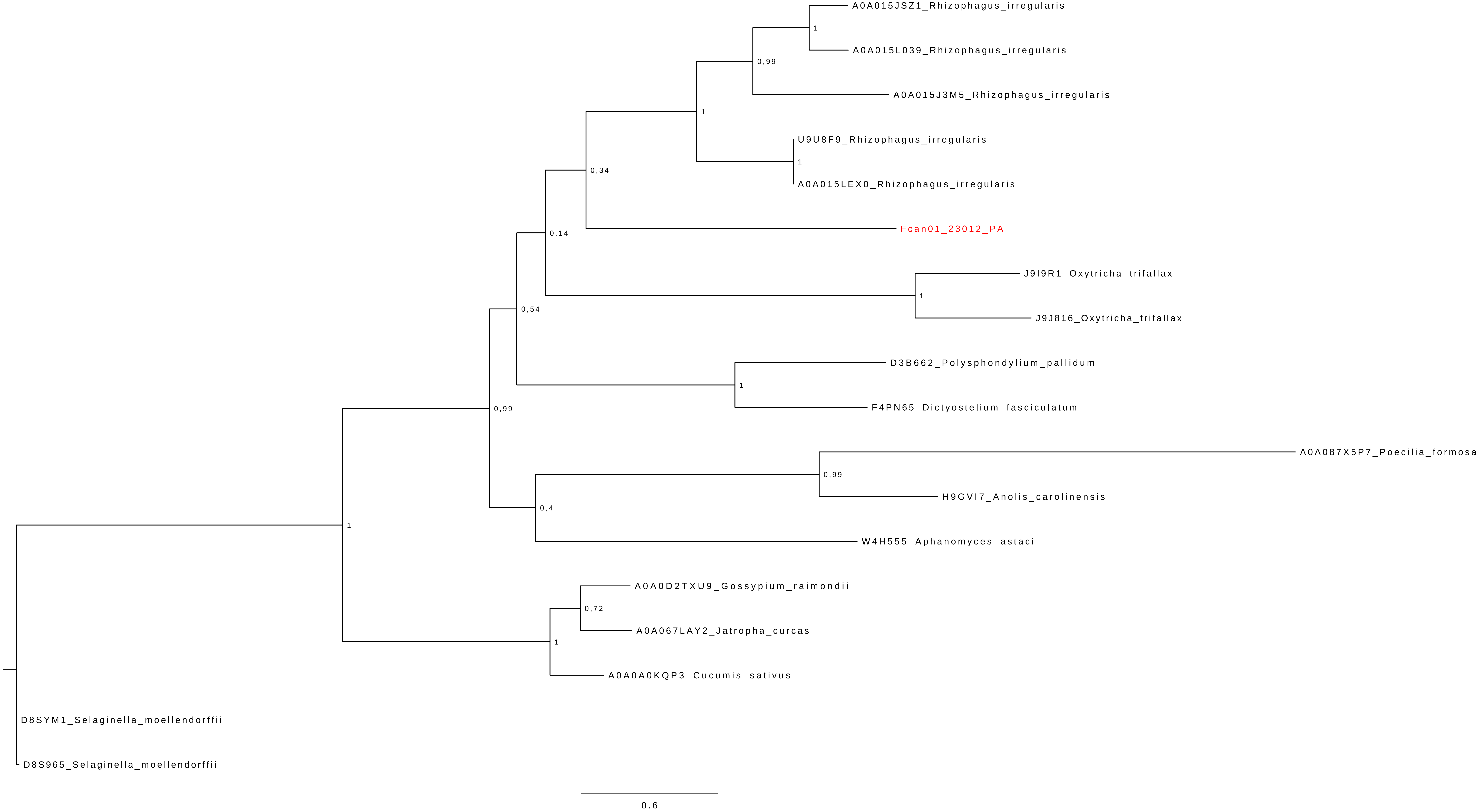

A0A015JSZ1_Rhizophagus_irregularis
1
A0A015L039_Rhizophagus_irregularis
0,99
A0A015J3M5_Rhizophagus_irregularis
1
U9U8F9_Rhizophagus_irregularis
1
A0A015LEX0_Rhizophagus_irregularis
0,34
Fcan01_23012_PA
0,14
J9I9R1_Oxytricha_trifallax
1
J9J816_Oxytricha_trifallax
0,54
D3B662_Polysphondylium_pallidum
1
F4PN65_Dictyostelium_fasciculatum
0,99
A0A087X5P7_Poecilia_formosa
0,99
H9GVI7_Anolis_carolinensis
0,4
W4H555_Aphanomyces_astaci
1
A0A0D2TXU9_Gossypium_raimondii
0,72
A0A067LAY2_Jatropha_curcas
1
A0A0A0KQP3_Cucumis_sativus
D8SYM1_Selaginella_moellendorffii
D8S965_Selaginella_moellendorffii
0.6

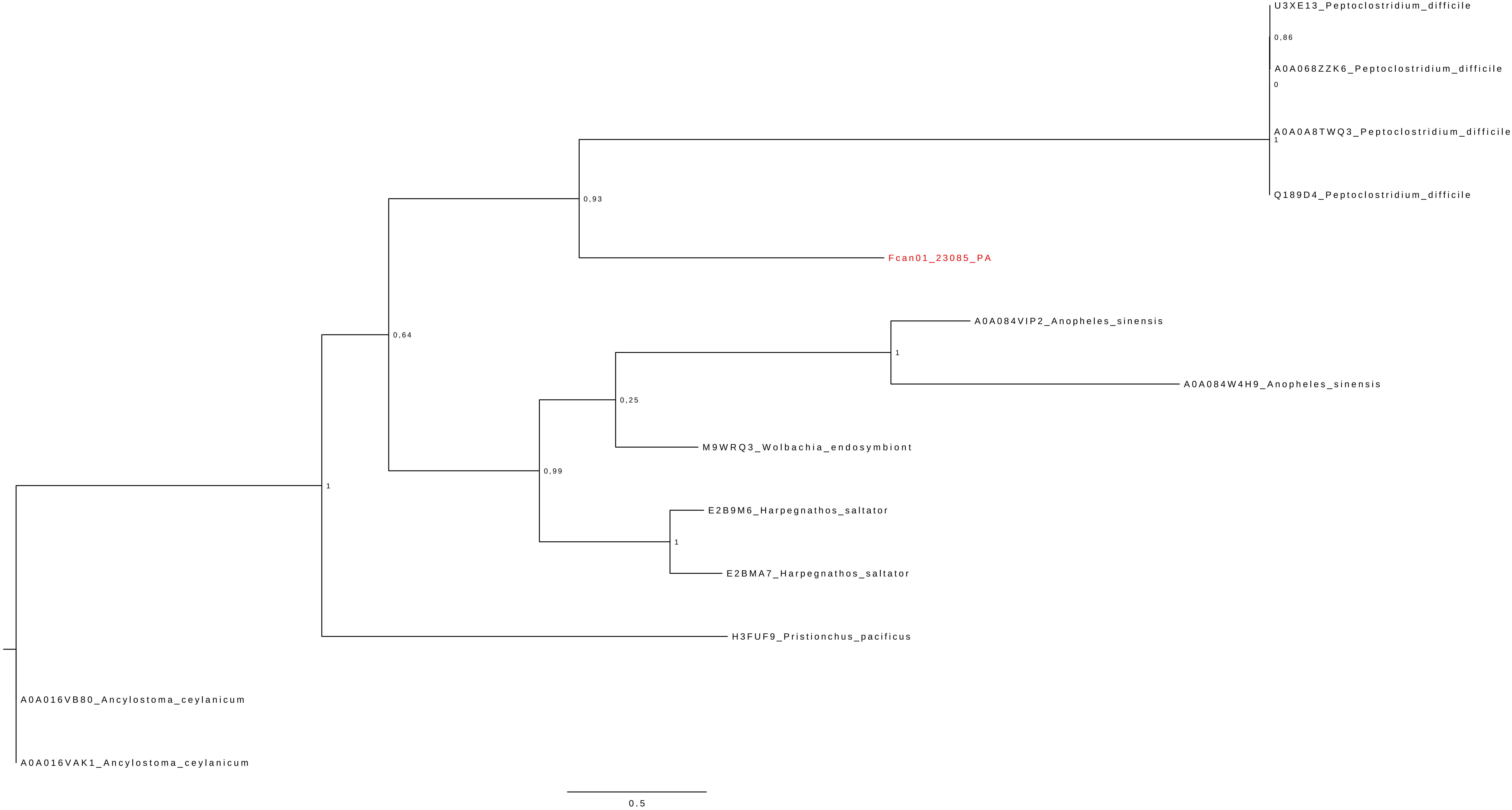
U3XE13_Peptoclostridium_difficile
0,86
A0A068ZZK6_Peptoclostridium_difficile
0
A0A0A8TWQ3_Peptoclostridium_difficile
1
Q189D4_Peptoclostridium_difficile
0,93
Fcan01_23085_PA
A0A084VIP2_Anopheles_sinensis
1
A0A084W4H9_Anopheles_sinensis
0,64
0,25
M9WRQ3_Wolbachia_endosymbiont
0,99
E2B9M6_Harpegnathos_saltator
1
E2BMA7_Harpegnathos_saltator
1
H3FUF9_Pristionchus_pacificus
A0A016VB80_Ancylostoma_ceylanicum
A0A016VAK1_Ancylostoma_ceylanicum
0.5

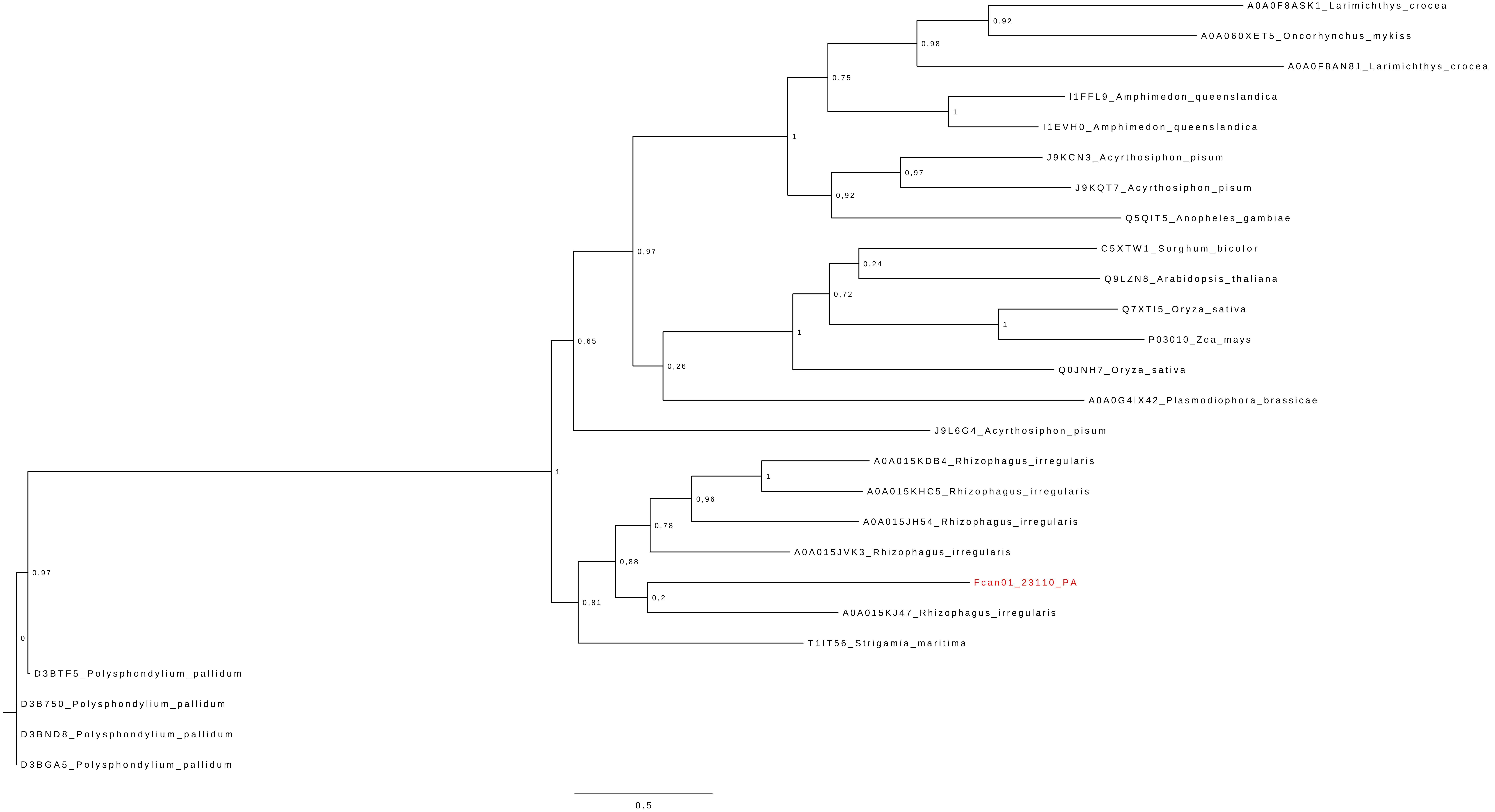
A0A0F8ASK1_Larimichthys_crocea
0,92
A0A060XET5_Oncorhynchus_mykiss
0,98
A0A0F8AN81_Larimichthys_crocea
0,75
I1FFL9_Amphimedon_queenslandica
1
I1EVH0_Amphimedon_queenslandica
1
J9KCN3_Acyrthosiphon_pisum
0,97
J9KQT7_Acyrthosiphon_pisum
0,92
Q5QIT5_Anopheles_gambiae
0,97
C5XTW1_Sorghum_bicolor
0,24
Q9LZN8_Arabidopsis_thaliana
0,72
Q7XTI5_Oryza_sativa
1
P03010_Zea_mays
0,65
1
Q0JNH7_Oryza_sativa
0,26
A0A0G4IX42_Plasmodiophora_brassicae
J9L6G4_Acyrthosiphon_pisum
A0A015KDB4_Rhizophagus_irregularis
1
1
A0A015KHC5_Rhizophagus_irregularis
0,96
A0A015JH54_Rhizophagus_irregularis
0,78
A0A015JVK3_Rhizophagus_irregularis
0,97
0,88
Fcan01_23110_PA
0,2
0,81
A0A015KJ47_Rhizophagus_irregularis
T1IT56_Strigamia_maritima
0
D3BTF5_Polysphondylium_pallidum
D3B750_Polysphondylium_pallidum
D3BND8_Polysphondylium_pallidum
D3BGA5_Polysphondylium_pallidum
0.5

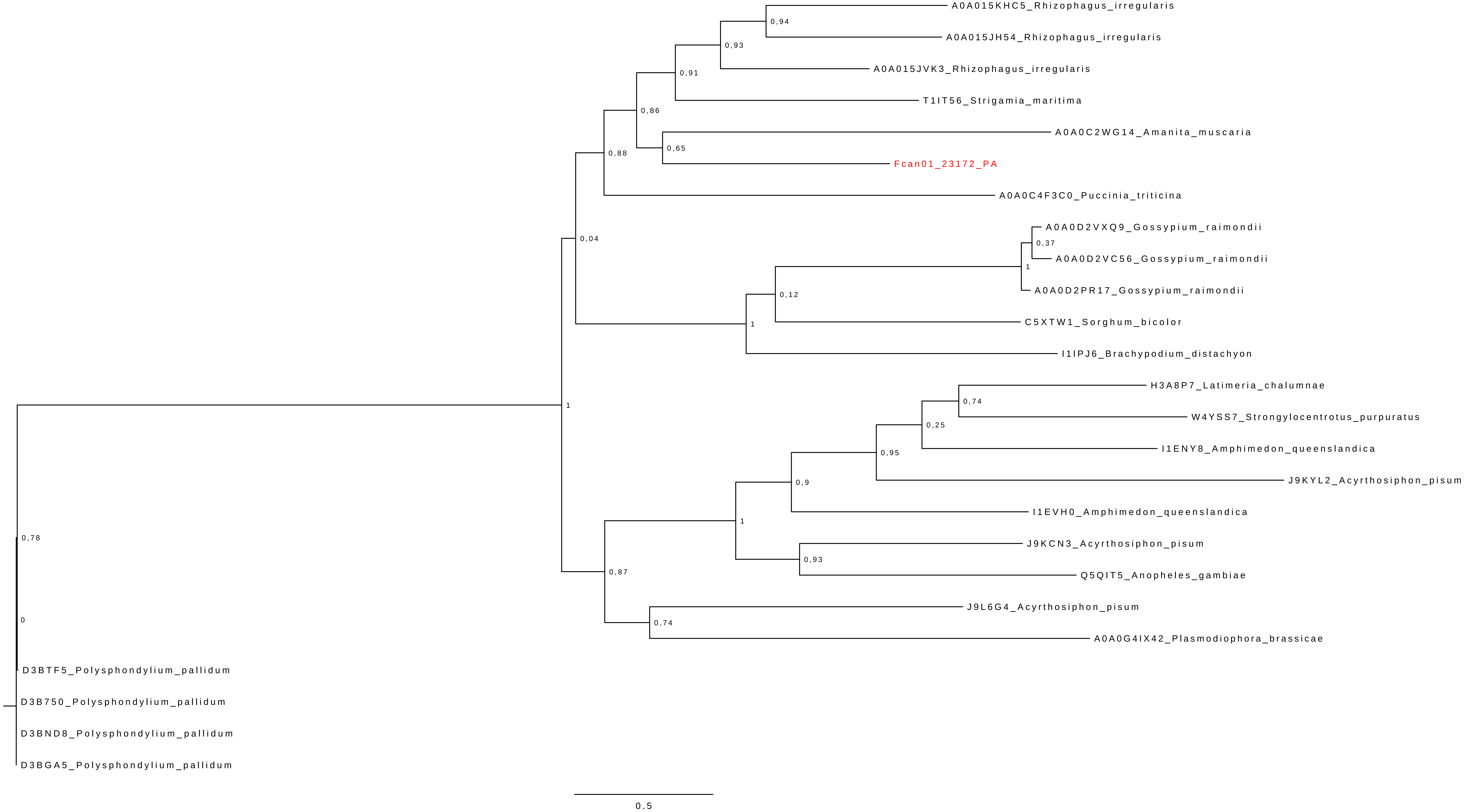
A0A015KHC5_Rhizophagus_irregularis
A0A015JH54_Rhizophagus_irregularis
A0A015JVK3_Rhizophagus_irregularis
T1IT56_Strigamia_maritima
A0A0C2WG14_Amanita_muscaria
Fcan01_23172_PA
A0A0C4F3C0_Puccinia_triticina
A0A0D2VXQ9_Gossypium_raimondii
A0A0D2VC56_Gossypium_raimondii
A0A0D2PR17_Gossypium_raimondii
C5XTW1_Sorghum_bicolor
I1IPJ6_Brachypodium_distachyon
H3A8P7_Latimeria_chalumnae
W4YSS7_Strongylocentrotus_purpuratus
I1ENY8_Amphimedon_queenslandica
J9KYL2_Acyrthosiphon_pisum
I1EVH0_Amphimedon_queenslandica
J9KCN3_Acyrthosiphon_pisum
Q5QIT5_Anopheles_gambiae
J9L6G4_Acyrthosiphon_pisum
A0A0G4IX42_Plasmodiophora_brassicae
D3BTF5_Polysphondylium_pallidum
D3B750_Polysphondylium_pallidum
D3BND8_Polysphondylium_pallidum
D3BGA5_Polysphondylium_pallidum
0,94
0,93
0,91
0,86
0,65
0,88
0,04
0,37
1
0,12
1
1
0,74
0,25
0,95
0,9
1
0,93
0,87
0,74
0,78
0
0.5

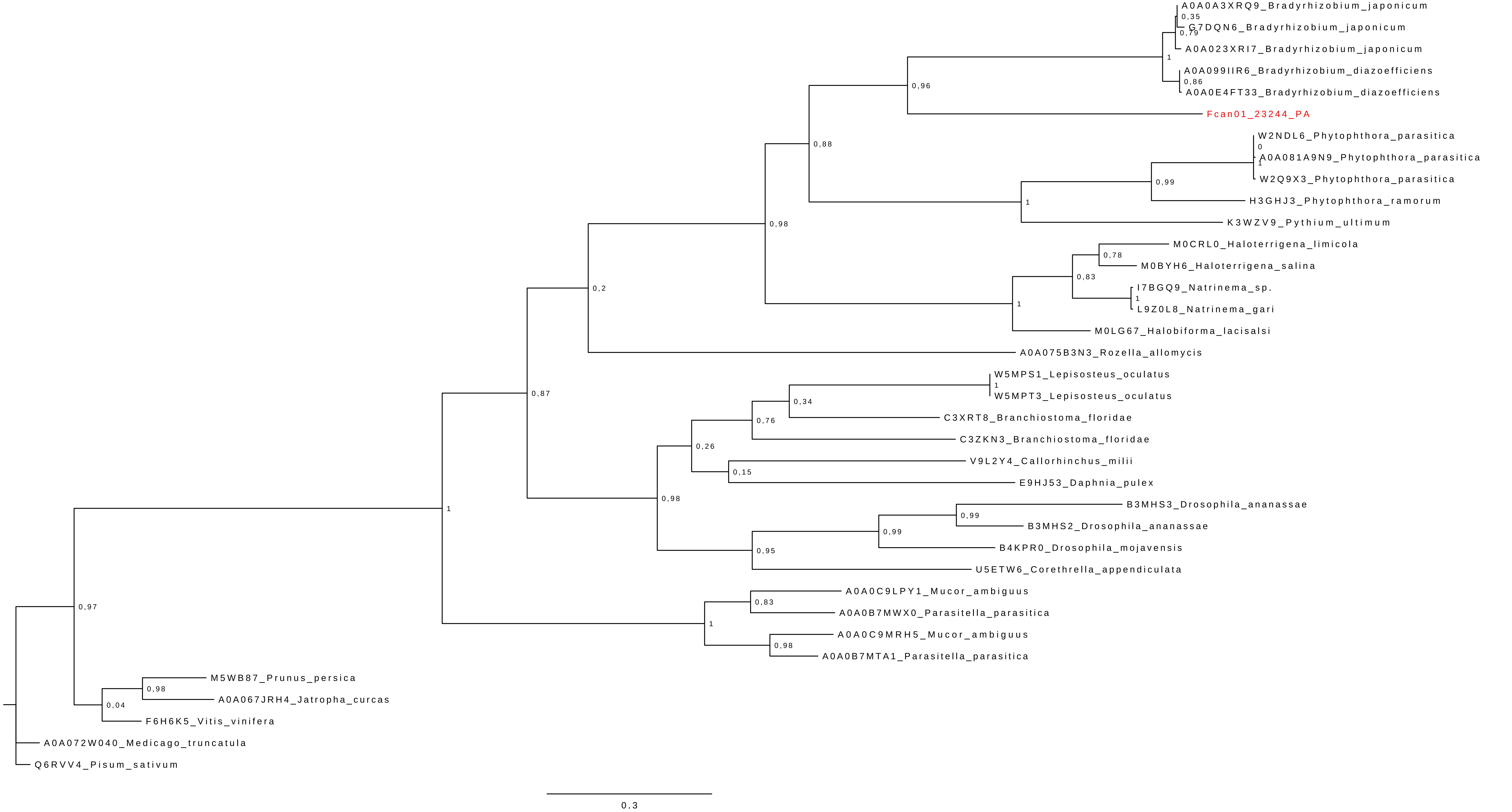

A0A0A3XRQ9_Bradyrhizobium_japonicum
0,35
G7DQN6_Bradyrhizobium_japonicum
0,79
A0A023XRI7_Bradyrhizobium_japonicum
1
A0A099IIR6_Bradyrhizobium_diazoefficiens
0,86
A0A0E4FT33_Bradyrhizobium_diazoefficiens
0,96
Fcan01_23244_PA
W2NDL6_Phytophthora_parasitica
0
A0A081A9N9_Phytophthora_parasitica
1
W2Q9X3_Phytophthora_parasitica
0,88
0,99
H3GHJ3_Phytophthora_ramorum
1
K3WZV9_Pythium_ultimum
0,98
M0CRL0_Haloterrigena_limicola
0,78
M0BYH6_Haloterrigena_salina
0,83
I7BGQ9_Natrinema_sp.
1
L9Z0L8_Natrinema_gari
0,2
1
M0LG67_Halobiforma_lacisalsi
A0A075B3N3_Rozella_allomycis
W5MPS1_Lepisosteus_oculatus
1
W5MPT3_Lepisosteus_oculatus
0,87
0,34
C3XRT8_Branchiostoma_floridae
0,76
C3ZKN3_Branchiostoma_floridae
0,26
V9L2Y4_Callorhinchus_milii
0,15
E9HJ53_Daphnia_pulex
0,98
B3MHS3_Drosophila_ananassae
1
0,99
B3MHS2_Drosophila_ananassae
0,99
B4KPR0_Drosophila_mojavensis
0,95
U5ETW6_Corethrella_appendiculata
A0A0C9LPY1_Mucor_ambiguus
0,83
0,97
A0A0B7MWX0_Parasitella_parasitica
1
A0A0C9MRH5_Mucor_ambiguus
0,98
A0A0B7MTA1_Parasitella_parasitica
M5WB87_Prunus_persica
0,98
A0A067JRH4_Jatropha_curcas
0,04
F6H6K5_Vitis_vinifera
A0A072W040_Medicago_truncatula
Q6RVV4_Pisum_sativum
0.3

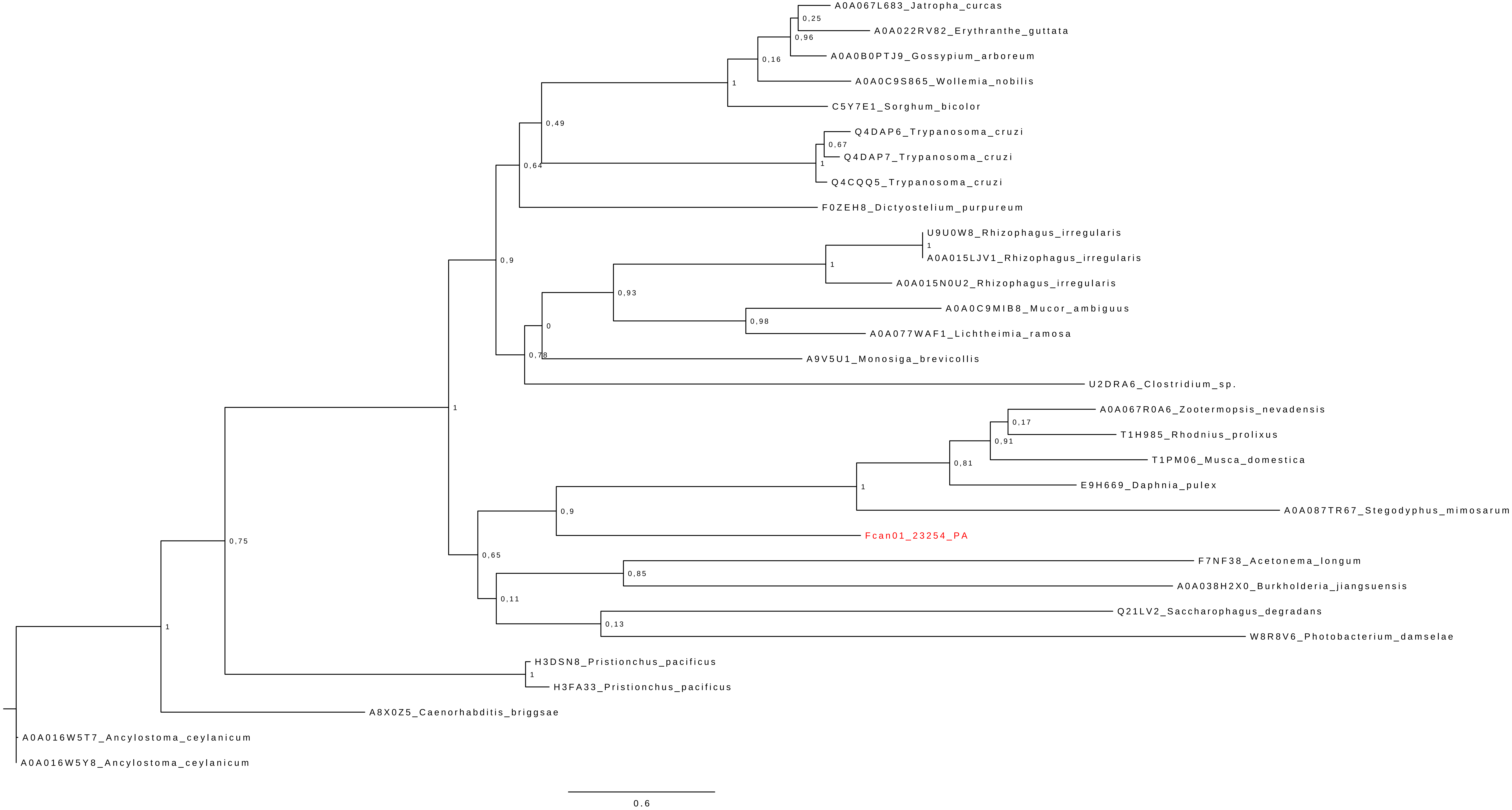
A0A067L683_Jatropha_curcas
0,25
A0A022RV82_Erythranthe_guttata
0,96
A0A0B0PTJ9_Gossypium_arboreum
0,16
A0A0C9S865_Wollemia_nobilis
1
C5Y7E1_Sorghum_bicolor
0,49
Q4DAP6_Trypanosoma_cruzi
0,67
Q4DAP7_Trypanosoma_cruzi
1
0,64
Q4CQQ5_Trypanosoma_cruzi
F0ZEH8_Dictyostelium_purpureum
U9U0W8_Rhizophagus_irregularis
1
A0A015LJV1_Rhizophagus_irregularis
0,9
1
A0A015N0U2_Rhizophagus_irregularis
0,93
A0A0C9MIB8_Mucor_ambiguus
0,98
0
A0A077WAF1_Lichtheimia_ramosa
0,78
A9V5U1_Monosiga_brevicollis
U2DRA6_Clostridium_sp.
1
A0A067R0A6_Zootermopsis_nevadensis
0,17
T1H985_Rhodnius_prolixus
0,91
T1PM06_Musca_domestica
0,81
E9H669_Daphnia_pulex
1
0,9
A0A087TR67_Stegodyphus_mimosarum
0,75
Fcan01_23254_PA
0,65
F7NF38_Acetonema_longum
0,85
A0A038H2X0_Burkholderia_jiangsuensis
0,11
Q21LV2_Saccharophagus_degradans
0,13
1
W8R8V6_Photobacterium_damselae
H3DSN8_Pristionchus_pacificus
1
H3FA33_Pristionchus_pacificus
A8X0Z5_Caenorhabditis_briggsae
A0A016W5T7_Ancylostoma_ceylanicum
A0A016W5Y8_Ancylostoma_ceylanicum
0.6

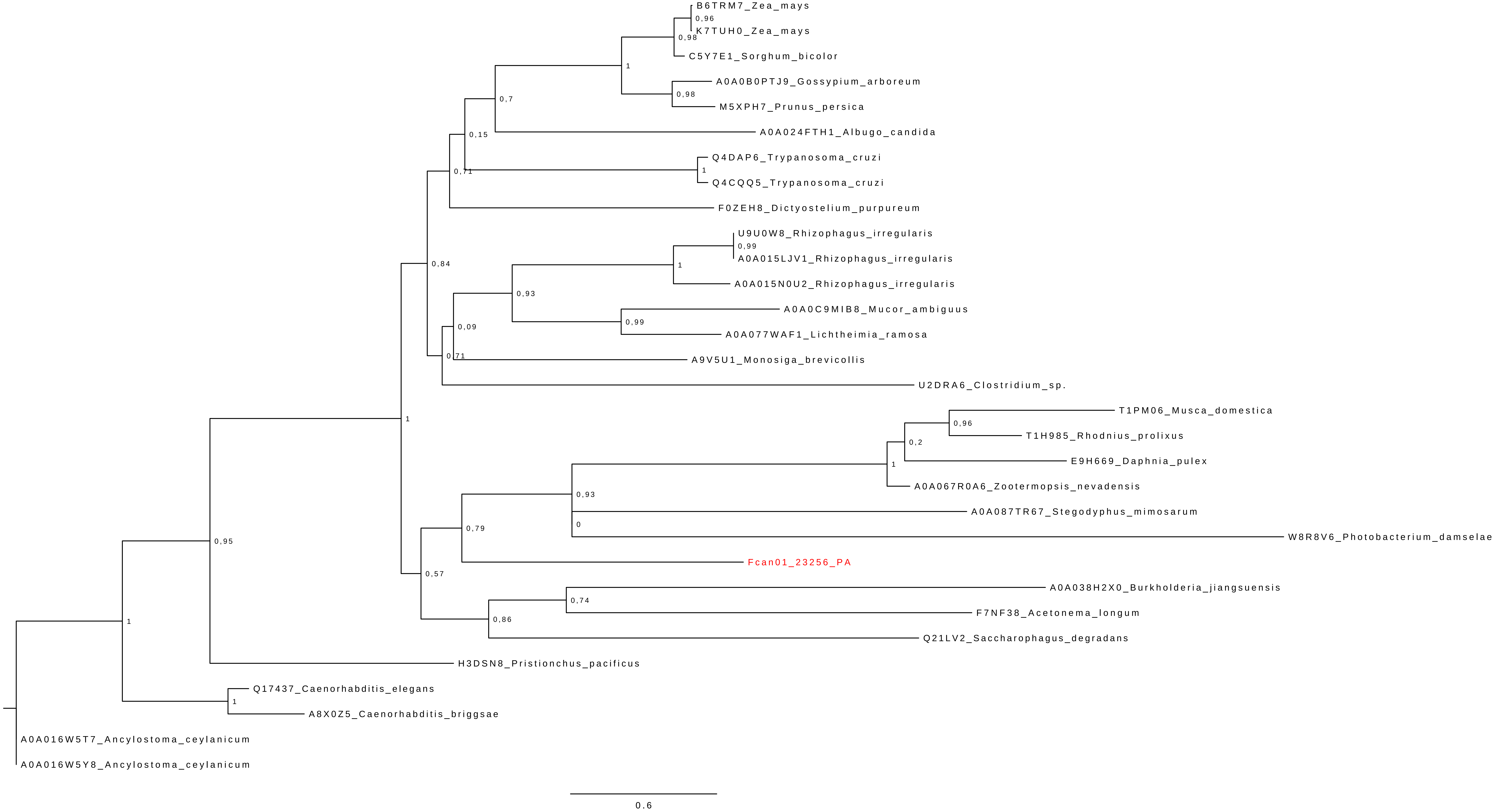
B6TRM7_Zea_mays
0,96
K7TUH0_Zea_mays
0,98
C5Y7E1_Sorghum_bicolor
1
A0A0B0PTJ9_Gossypium_arboreum
0,98
M5XPH7_Prunus_persica
0,7
A0A024FTH1_Albugo_candida
0,15
Q4DAP6_Trypanosoma_cruzi
1
Q4CQQ5_Trypanosoma_cruzi
0,71
F0ZEH8_Dictyostelium_purpureum
U9U0W8_Rhizophagus_irregularis
0,99
A0A015LJV1_Rhizophagus_irregularis
1
A0A015N0U2_Rhizophagus_irregularis
0,84
0,93
A0A0C9MIB8_Mucor_ambiguus
0,99
A0A077WAF1_Lichtheimia_ramosa
0,09
0,71
A9V5U1_Monosiga_brevicollis
U2DRA6_Clostridium_sp.
1
T1PM06_Musca_domestica
0,96
T1H985_Rhodnius_prolixus
0,2
E9H669_Daphnia_pulex
1
A0A067R0A6_Zootermopsis_nevadensis
0,93
A0A087TR67_Stegodyphus_mimosarum
0
W8R8V6_Photobacterium_damselae
0,95
0,79
Fcan01_23256_PA
0,57
A0A038H2X0_Burkholderia_jiangsuensis
0,74
F7NF38_Acetonema_longum
1
0,86
Q21LV2_Saccharophagus_degradans
H3DSN8_Pristionchus_pacificus
Q17437_Caenorhabditis_elegans
1
A8X0Z5_Caenorhabditis_briggsae
A0A016W5T7_Ancylostoma_ceylanicum
A0A016W5Y8_Ancylostoma_ceylanicum
0.6

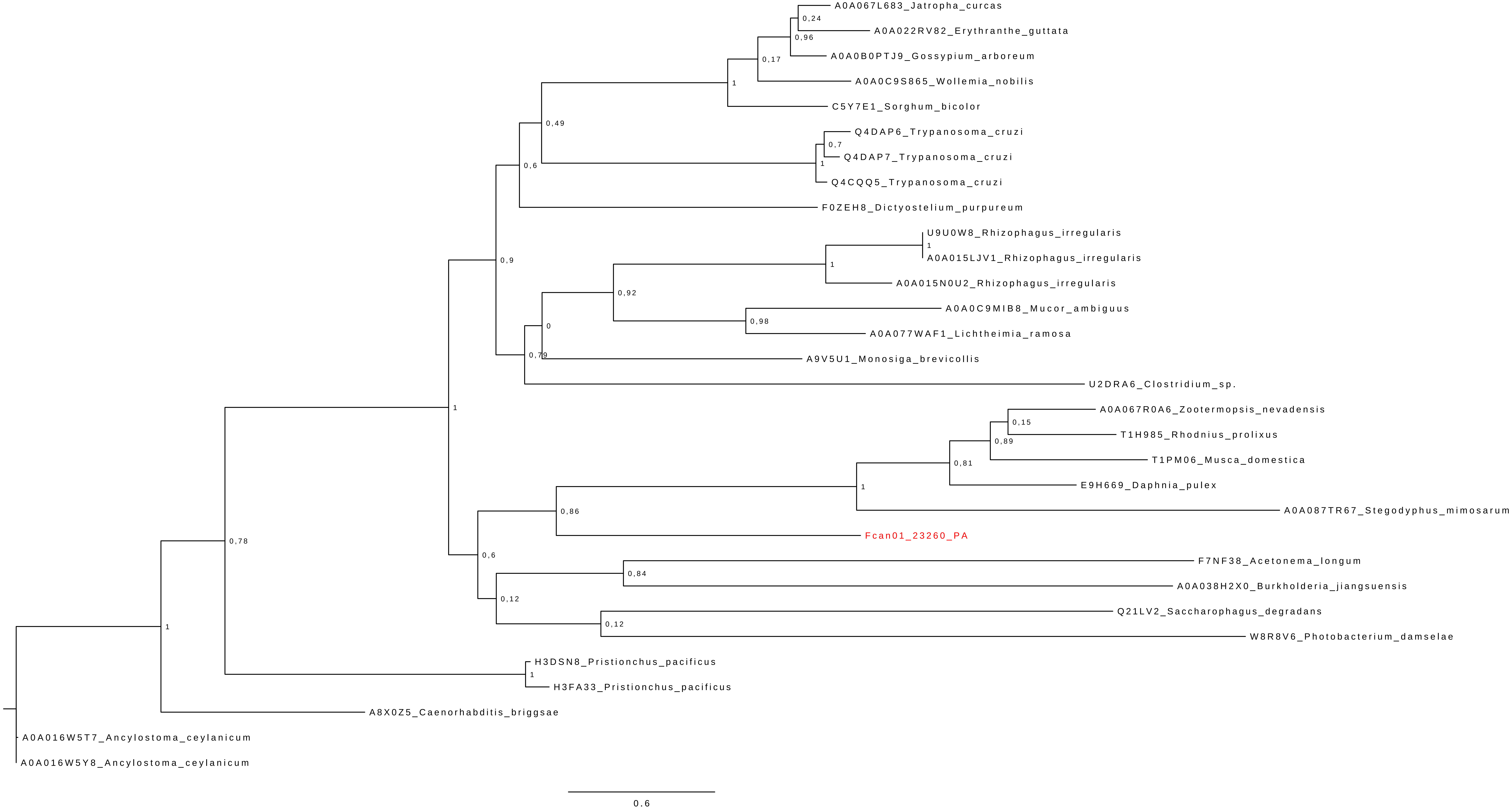
A0A067L683_Jatropha_curcas
A0A022RV82_Erythranthe_guttata
A0A0B0PTJ9_Gossypium_arboreum
A0A0C9S865_Wollemia_nobilis
C5Y7E1_Sorghum_bicolor
Q4DAP6_Trypanosoma_cruzi
Q4DAP7_Trypanosoma_cruzi
Q4CQQ5_Trypanosoma_cruzi
F0ZEH8_Dictyostelium_purpureum
U9U0W8_Rhizophagus_irregularis
A0A015LJV1_Rhizophagus_irregularis
A0A015N0U2_Rhizophagus_irregularis
A0A0C9MIB8_Mucor_ambiguus
A0A077WAF1_Lichtheimia_ramosa
A9V5U1_Monosiga_brevicollis
U2DRA6_Clostridium_sp.
A0A067R0A6_Zootermopsis_nevadensis
T1H985_Rhodnius_prolixus
T1PM06_Musca_domestica
E9H669_Daphnia_pulex
A0A087TR67_Stegodyphus_mimosarum
Fcan01_23260_PA
F7NF38_Acetonema_longum
A0A038H2X0_Burkholderia_jiangsuensis
Q21LV2_Saccharophagus_degradans
W8R8V6_Photobacterium_damselae
H3DSN8_Pristionchus_pacificus
H3FA33_Pristionchus_pacificus
A8X0Z5_Caenorhabditis_briggsae
A0A016W5T7_Ancylostoma_ceylanicum
A0A016W5Y8_Ancylostoma_ceylanicum
0.6

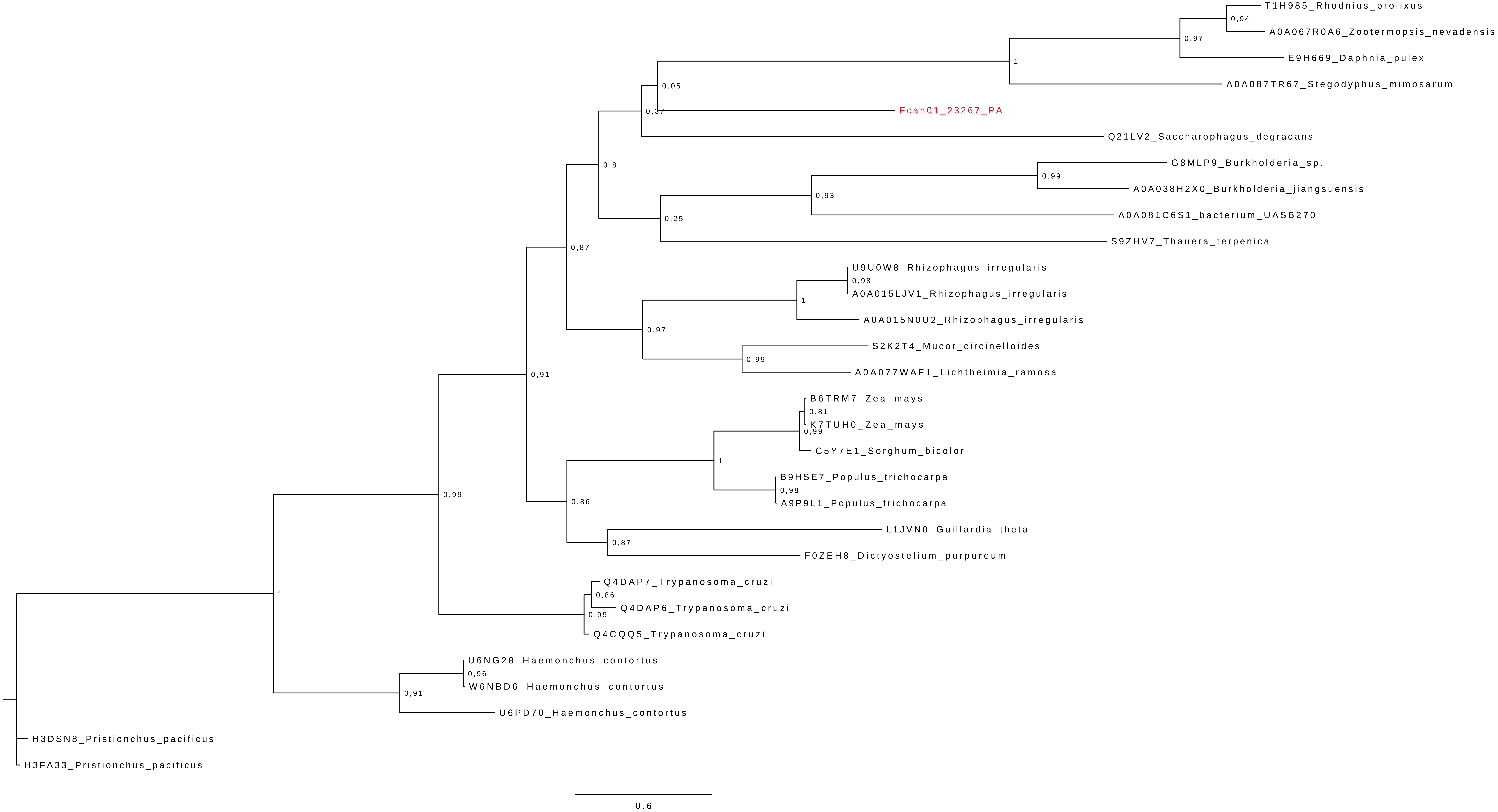
T1H985_Rhodnius_prolixus
0,94
A0A067R0A6_Zootermopsis_nevadensis
0,97
E9H669_Daphnia_pulex
1
A0A087TR67_Stegodyphus_mimosarum
0,05
Fcan01_23267_PA
0,37
Q21LV2_Saccharophagus_degradans
0,8
G8MLP9_Burkholderia_sp.
0,99
A0A038H2X0_Burkholderia_jiangsuensis
0,93
A0A081C6S1_bacterium_UASB270
0,25
S9ZHV7_Thauera_terpenica
0,87
U9U0W8_Rhizophagus_irregularis
0,98
A0A015LJV1_Rhizophagus_irregularis
1
A0A015N0U2_Rhizophagus_irregularis
0,97
S2K2T4_Mucor_circinelloides
0,99
A0A077WAF1_Lichtheimia_ramosa
0,91
B6TRM7_Zea_mays
0,81
K7TUH0_Zea_mays
0,99
C5Y7E1_Sorghum_bicolor
1
B9HSE7_Populus_trichocarpa
0,98
A9P9L1_Populus_trichocarpa
0,86
L1JVN0_Guillardia_theta
0,87
F0ZEH8_Dictyostelium_purpureum
0,99
Q4DAP7_Trypanosoma_cruzi
0,86
Q4DAP6_Trypanosoma_cruzi
0,99
Q4CQQ5_Trypanosoma_cruzi
1
U6NG28_Haemonchus_contortus
0,96
W6NBD6_Haemonchus_contortus
0,91
U6PD70_Haemonchus_contortus
H3DSN8_Pristionchus_pacificus
H3FA33_Pristionchus_pacificus
0.6

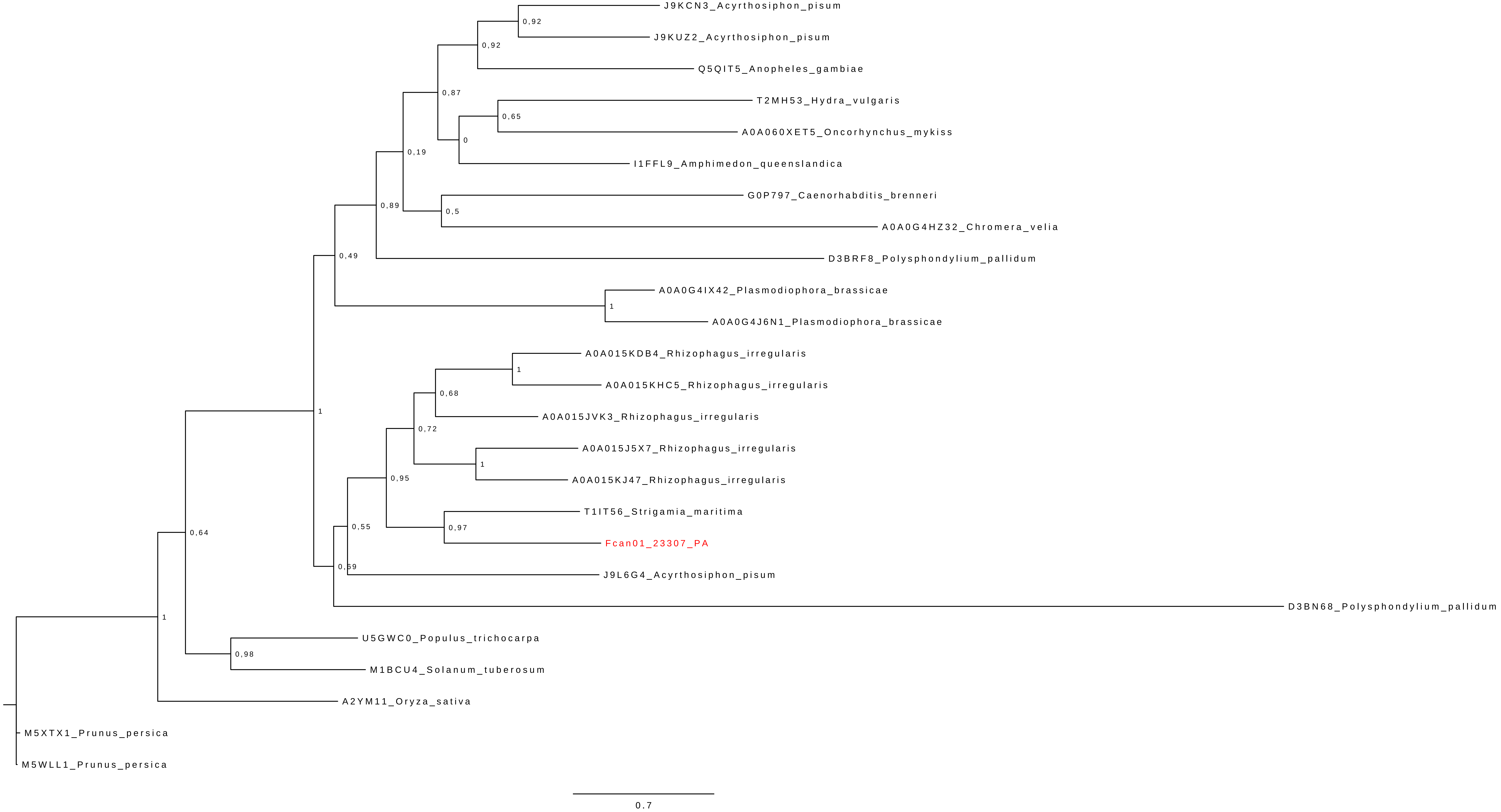
J9KCN3_Acyrthosiphon_pisum
0,92
J9KUZ2_Acyrthosiphon_pisum
0,92
Q5QIT5_Anopheles_gambiae
0,87
T2MH53_Hydra_vulgaris
0,65
A0A060XET5_Oncorhynchus_mykiss
0
I1FFL9_Amphimedon_queenslandica
0,19
G0P797_Caenorhabditis_brenneri
0,89
0,5
A0A0G4HZ32_Chromera_velia
0,49
D3BRF8_Polysphondylium_pallidum
A0A0G4IX42_Plasmodiophora_brassicae
1
A0A0G4J6N1_Plasmodiophora_brassicae
A0A015KDB4_Rhizophagus_irregularis
1
A0A015KHC5_Rhizophagus_irregularis
0,68
1
A0A015JVK3_Rhizophagus_irregularis
0,72
A0A015J5X7_Rhizophagus_irregularis
1
0,95
A0A015KJ47_Rhizophagus_irregularis
T1IT56_Strigamia_maritima
0,64
0,55
0,97
Fcan01_23307_PA
0,69
J9L6G4_Acyrthosiphon_pisum
D3BN68_Polysphondylium_pallidum
1
U5GWC0_Populus_trichocarpa
0,98
M1BCU4_Solanum_tuberosum
A2YM11_Oryza_sativa
M5XTX1_Prunus_persica
M5WLL1_Prunus_persica
0.7

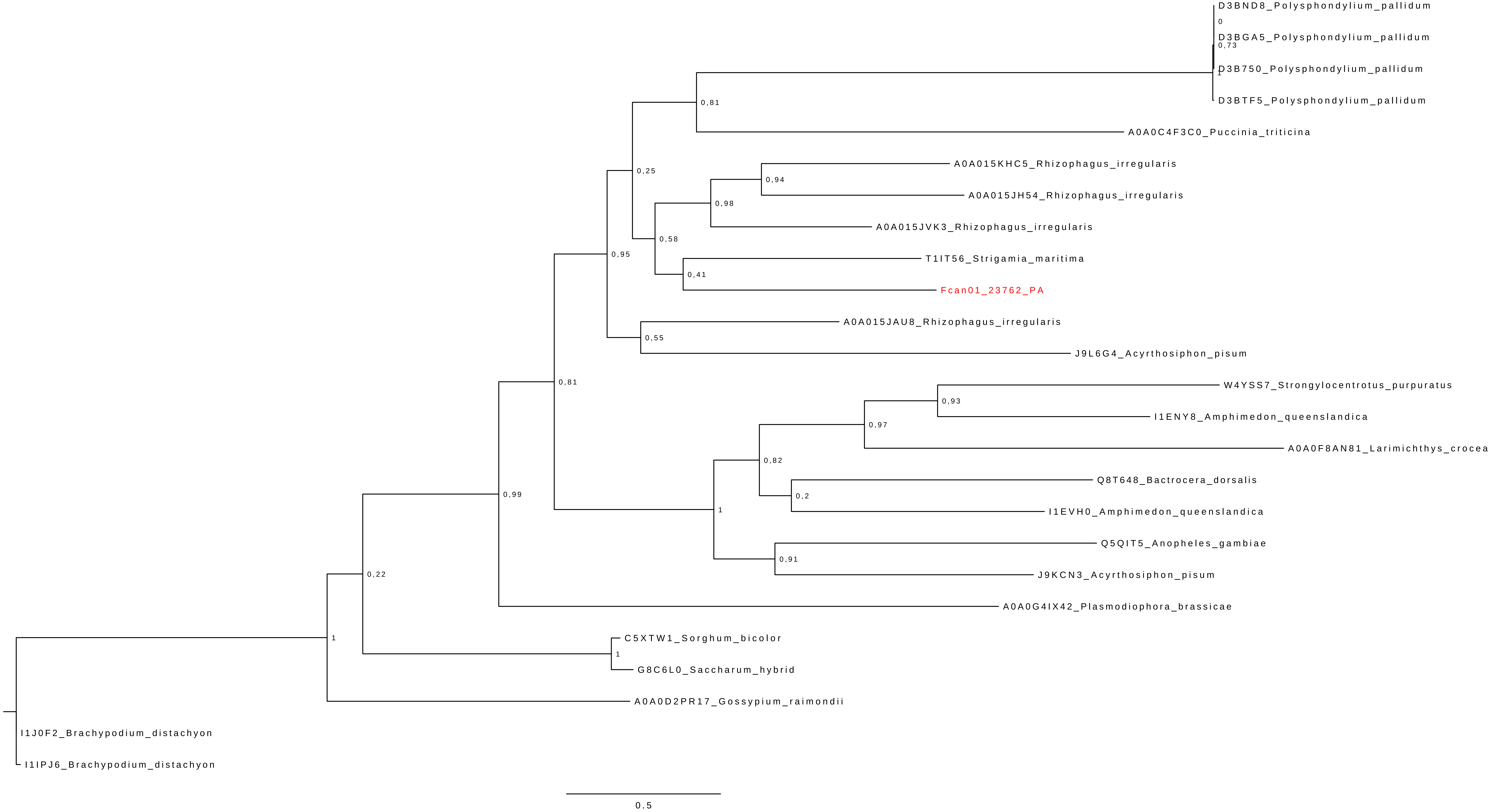
D3BND8_Polysphondylium_pallidum
0
D3BGA5_Polysphondylium_pallidum
0,73
D3B750_Polysphondylium_pallidum
1
D3BTF5_Polysphondylium_pallidum
0,81
A0A0C4F3C0_Puccinia_triticina
A0A015KHC5_Rhizophagus_irregularis
0,94
A0A015JH54_Rhizophagus_irregularis
0,25
0,98
A0A015JVK3_Rhizophagus_irregularis
0,58
T1IT56_Strigamia_maritima
0,95
0,41
Fcan01_23762_PA
A0A015JAU8_Rhizophagus_irregularis
0,55
J9L6G4_Acyrthosiphon_pisum
0,81
W4YSS7_Strongylocentrotus_purpuratus
0,93
I1ENY8_Amphimedon_queenslandica
0,97
A0A0F8AN81_Larimichthys_crocea
0,82
Q8T648_Bactrocera_dorsalis
0,99
0,2
I1EVH0_Amphimedon_queenslandica
1
Q5QIT5_Anopheles_gambiae
0,91
0,22
J9KCN3_Acyrthosiphon_pisum
A0A0G4IX42_Plasmodiophora_brassicae
C5XTW1_Sorghum_bicolor
1
1
G8C6L0_Saccharum_hybrid
A0A0D2PR17_Gossypium_raimondii
I1J0F2_Brachypodium_distachyon
I1IPJ6_Brachypodium_distachyon
0.5

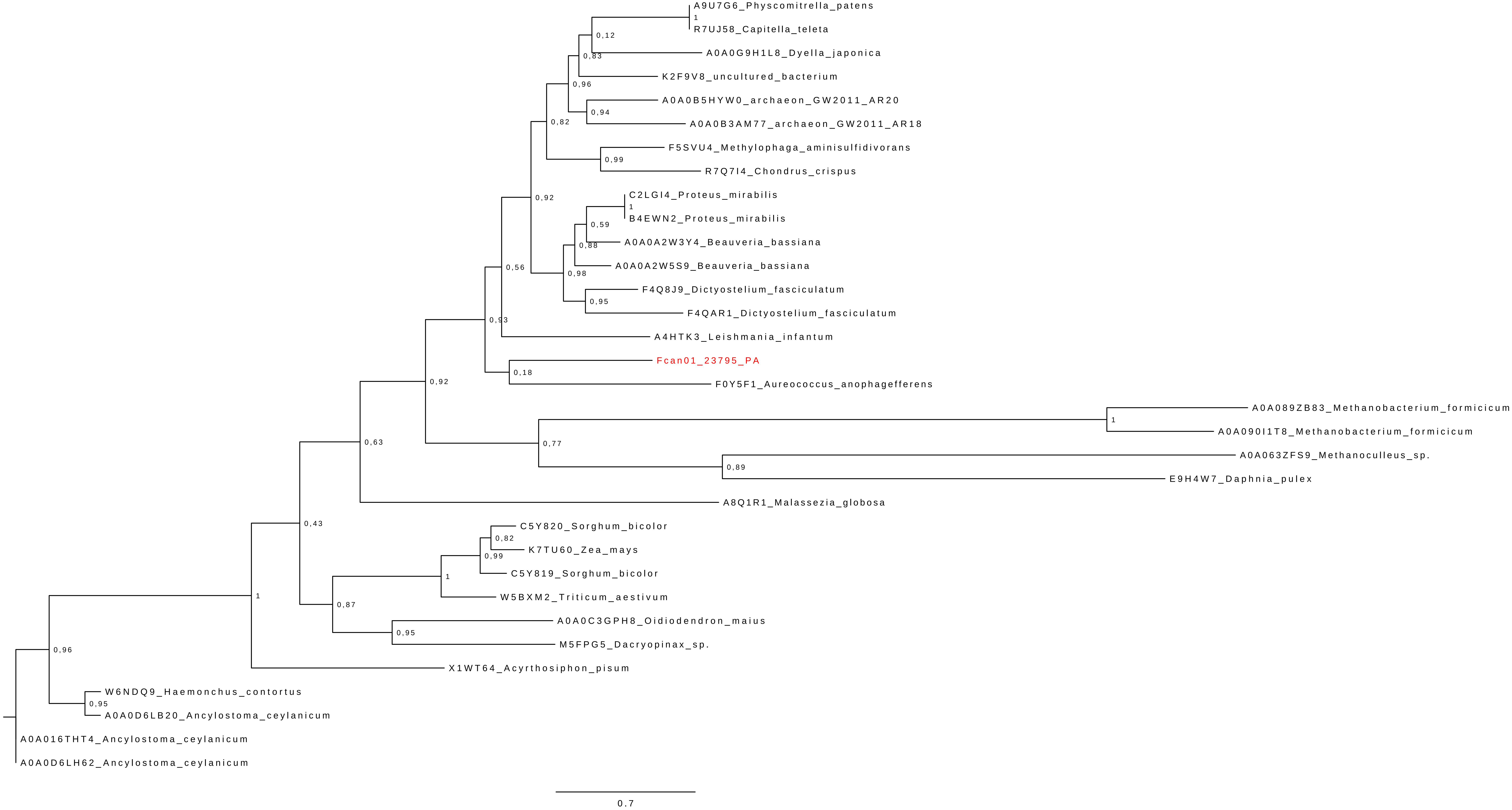
A9U7G6_Physcomitrella_patens
1
R7UJ58_Capitella_teleta
0,12
A0A0G9H1L8_Dyella_japonica
0,83
K2F9V8_uncultured_bacterium
0,96
A0A0B5HYW0_archaeon_GW2011_AR20
0,94
A0A0B3AM77_archaeon_GW2011_AR18
0,82
F5SVU4_Methylophaga_aminisulfidivorans
0,99
R7Q7I4_Chondrus_crispus
0,92
C2LGI4_Proteus_mirabilis
1
B4EWN2_Proteus_mirabilis
0,59
A0A0A2W3Y4_Beauveria_bassiana
0,88
A0A0A2W5S9_Beauveria_bassiana
0,56
0,98
F4Q8J9_Dictyostelium_fasciculatum
0,95
F4QAR1_Dictyostelium_fasciculatum
0,93
A4HTK3_Leishmania_infantum
Fcan01_23795_PA
0,18
F0Y5F1_Aureococcus_anophagefferens
0,92
A0A089ZB83_Methanobacterium_formicicum
1
A0A090I1T8_Methanobacterium_formicicum
0,63
0,77
A0A063ZFS9_Methanoculleus_sp.
0,89
E9H4W7_Daphnia_pulex
A8Q1R1_Malassezia_globosa
0,43
C5Y820_Sorghum_bicolor
0,82
K7TU60_Zea_mays
0,99
C5Y819_Sorghum_bicolor
1
W5BXM2_Triticum_aestivum
1
0,87
A0A0C3GPH8_Oidiodendron_maius
0,95
M5FPG5_Dacryopinax_sp.
0,96
X1WT64_Acyrthosiphon_pisum
W6NDQ9_Haemonchus_contortus
0,95
A0A0D6LB20_Ancylostoma_ceylanicum
A0A016THT4_Ancylostoma_ceylanicum
A0A0D6LH62_Ancylostoma_ceylanicum
0.7

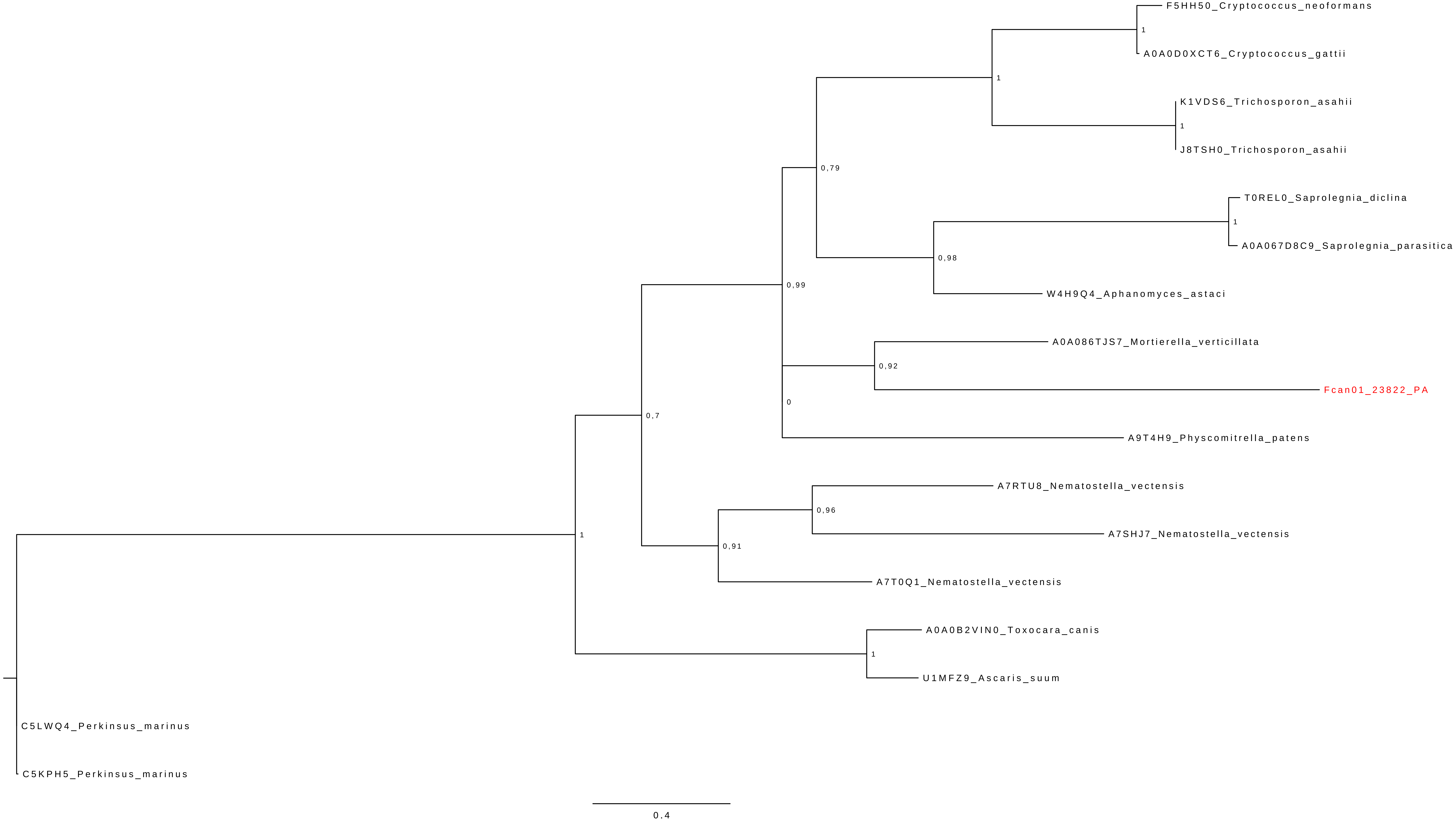
F5HH50_Cryptococcus_neoformans
1
A0A0D0XCT6_Cryptococcus_gattii
1
K1VDS6_Trichosporon_asahii
1
J8TSH0_Trichosporon_asahii
0,79
T0REL0_Saprolegnia_diclina
1
A0A067D8C9_Saprolegnia_parasitica
0,98
W4H9Q4_Aphanomyces_astaci
0,99
A0A086TJS7_Mortierella_verticillata
0,92
Fcan01_23822_PA
0
0,7
A9T4H9_Physcomitrella_patens
A7RTU8_Nematostella_vectensis
0,96
A7SHJ7_Nematostella_vectensis
1
0,91
A7T0Q1_Nematostella_vectensis
A0A0B2VIN0_Toxocara_canis
1
U1MFZ9_Ascaris_suum
C5LWQ4_Perkinsus_marinus
C5KPH5_Perkinsus_marinus
0.4

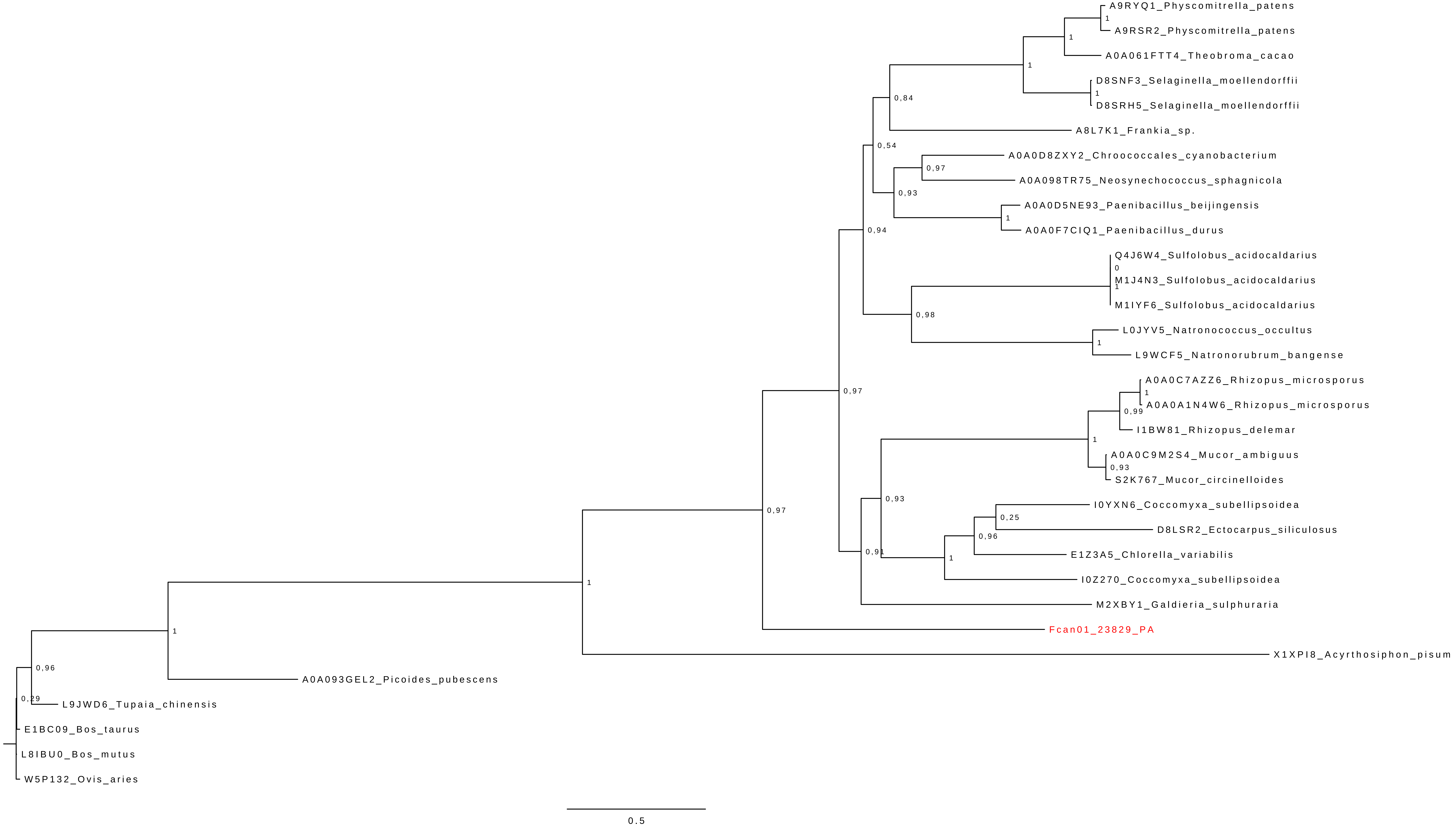
A9RYQ1_Physcomitrella_patens
A9RSR2_Physcomitrella_patens
A0A061FTT4_Theobroma_cacao
D8SNF3_Selaginella_moellendorffii
D8SRH5_Selaginella_moellendorffii
A8L7K1_Frankia_sp.
A0A0D8ZXY2_Chroococcales_cyanobacterium
A0A098TR75_Neosynechococcus_sphagnicola
A0A0D5NE93_Paenibacillus_beijingensis
A0A0F7CIQ1_Paenibacillus_durus
Q4J6W4_Sulfolobus_acidocaldarius
M1J4N3_Sulfolobus_acidocaldarius
M1IYF6_Sulfolobus_acidocaldarius
L0JYV5_Natronococcus_occultus
L9WCF5_Natronorubrum_bangense
A0A0C7AZZ6_Rhizopus_microsporus
A0A0A1N4W6_Rhizopus_microsporus
I1BW81_Rhizopus_delemar
A0A0C9M2S4_Mucor_ambiguus
S2K767_Mucor_circinelloides
I0YXN6_Coccomyxa_subellipsoidea
D8LSR2_Ectocarpus_siliculosus
E1Z3A5_Chlorella_variabilis
I0Z270_Coccomyxa_subellipsoidea
M2XBY1_Galdieria_sulphuraria
Fcan01_23829_PA
X1XPI8_Acyrthosiphon_pisum
A0A093GEL2_Picoides_pubescens
L9JWD6_Tupaia_chinensis
E1BC09_Bos_taurus
L8IBU0_Bos_mutus
W5P132_Ovis_aries
1
1
1
0,84
0,54
0,97
0,93
1
0,94
0
1
0,98
1
0,97
1
0,99
1
0,93
0,93
0,25
0,96
1
0,91
0,97
1
1
0,96
0,29
0.5

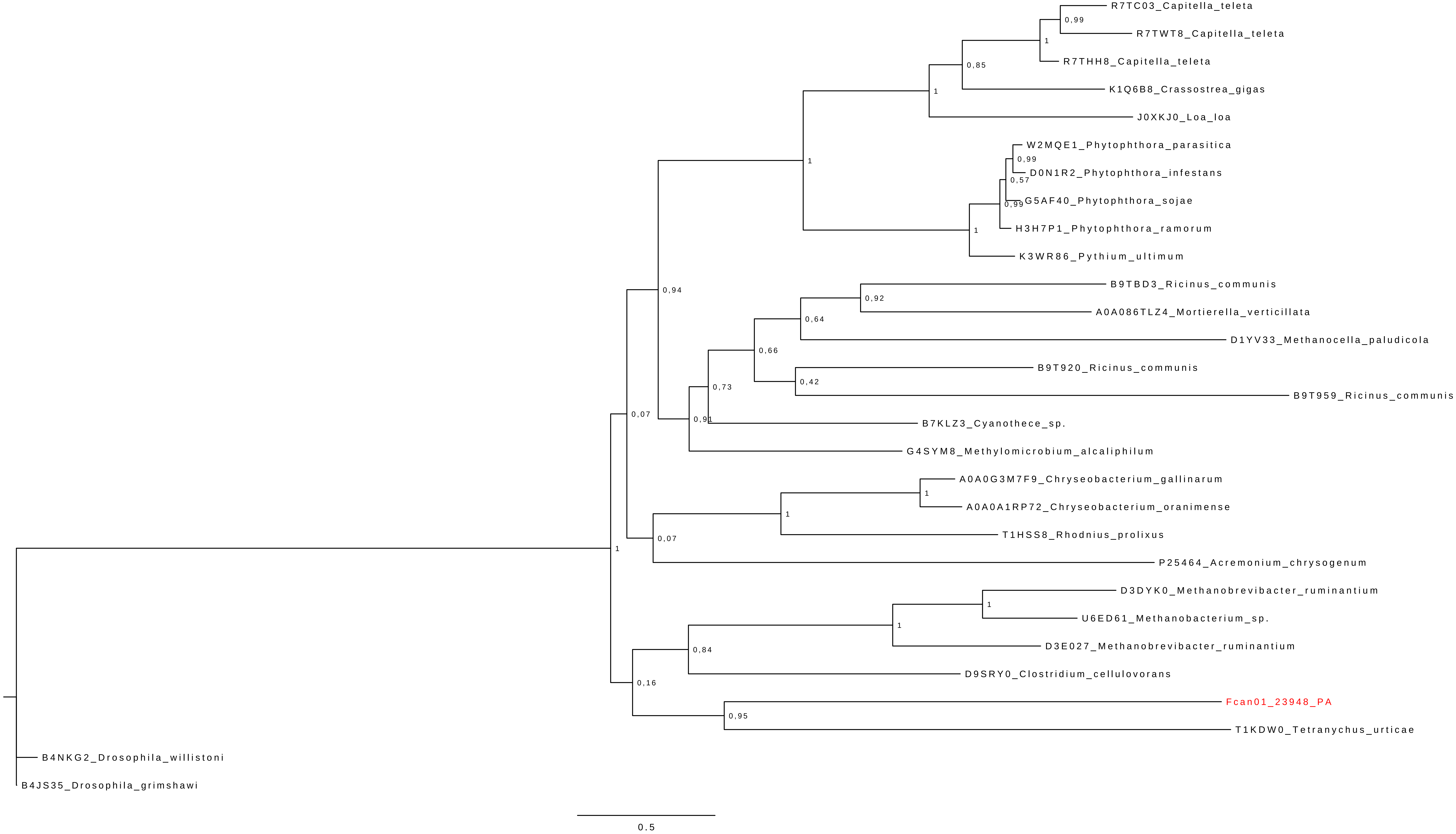
R7TC03_Capitella_teleta
R7TWT8_Capitella_teleta
R7THH8_Capitella_teleta
K1Q6B8_Crassostrea_gigas
J0XKJ0_Loa_loa
W2MQE1_Phytophthora_parasitica
D0N1R2_Phytophthora_infestans
G5AF40_Phytophthora_sojae
H3H7P1_Phytophthora_ramorum
K3WR86_Pythium_ultimum
B9TBD3_Ricinus_communis
A0A086TLZ4_Mortierella_verticillata
D1YV33_Methanocella_paludicola
B9T920_Ricinus_communis
B9T959_Ricinus_communis
B7KLZ3_Cyanothece_sp.
G4SYM8_Methylomicrobium_alcaliphilum
A0A0G3M7F9_Chryseobacterium_gallinarum
A0A0A1RP72_Chryseobacterium_oranimense
T1HSS8_Rhodnius_prolixus
P25464_Acremonium_chrysogenum
D3DYK0_Methanobrevibacter_ruminantium
U6ED61_Methanobacterium_sp.
D3E027_Methanobrevibacter_ruminantium
D9SRY0_Clostridium_cellulovorans
Fcan01_23948_PA
T1KDW0_Tetranychus_urticae
B4NKG2_Drosophila_willistoni
B4JS35_Drosophila_grimshawi
0,99
1
0,85
1
1
0,99
0,57
0,99
1
0,94
0,92
0,64
0,66
0,42
0,73
0,91
0,07
1
1
0,07
1
1
1
0,84
0,16
0,95
0.5

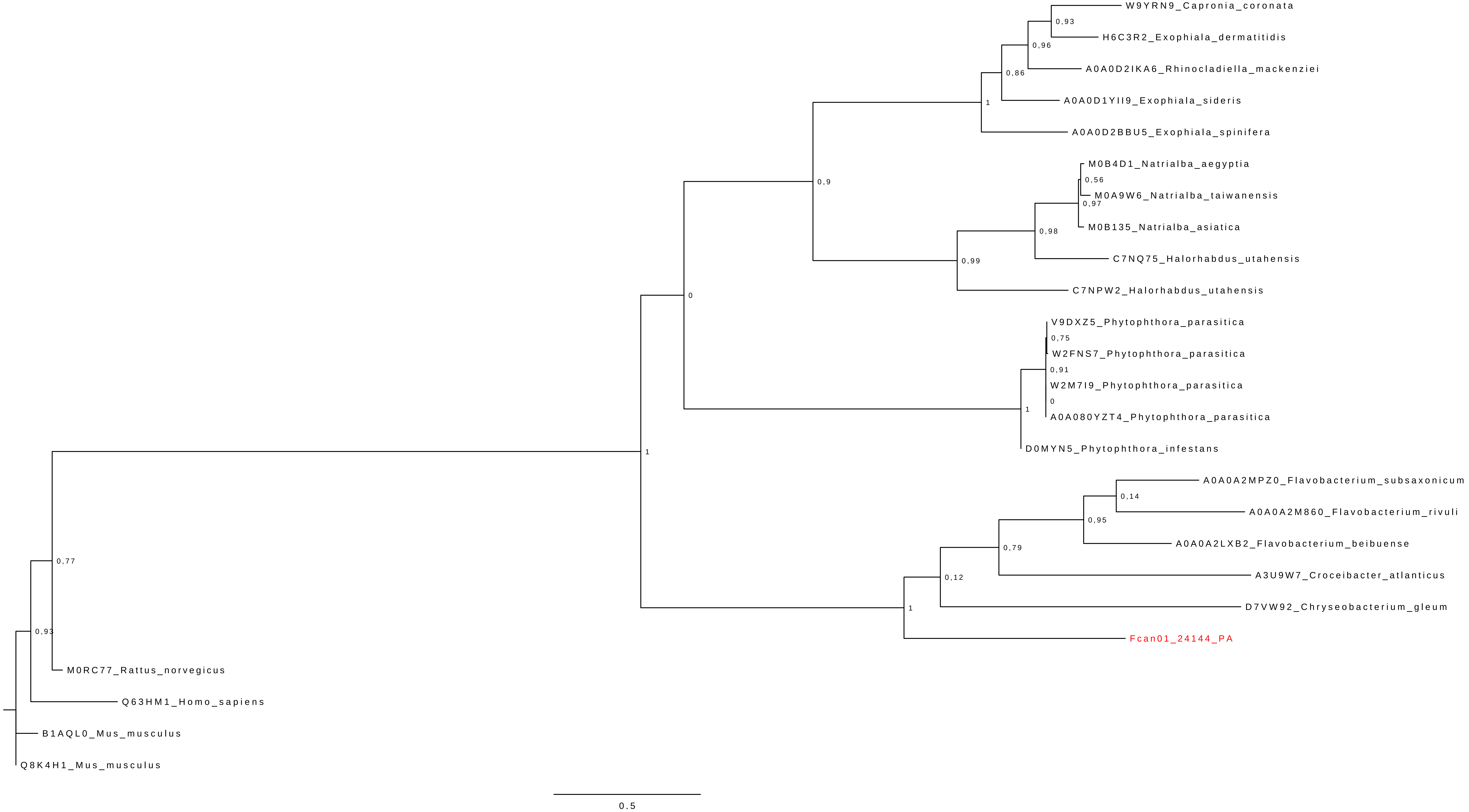

W9YRN9_Capronia_coronata
0,93
H6C3R2_Exophiala_dermatitidis
0,96
A0A0D2IKA6_Rhinocladiella_mackenziei
0,86
A0A0D1YII9_Exophiala_sideris
1
A0A0D2BBU5_Exophiala_spinifera
M0B4D1_Natrialba_aegyptia
0,56
M0A9W6_Natrialba_taiwanensis
0,97
M0B135_Natrialba_asiatica
0,98
0,9
C7NQ75_Halorhabdus_utahensis
0,99
C7NPW2_Halorhabdus_utahensis
0
V9DXZ5_Phytophthora_parasitica
0,75
W2FNS7_Phytophthora_parasitica
0,91
W2M7I9_Phytophthora_parasitica
0
A0A080YZT4_Phytophthora_parasitica
1
D0MYN5_Phytophthora_infestans
1
A0A0A2MPZ0_Flavobacterium_subsaxonicum
0,14
A0A0A2M860_Flavobacterium_rivuli
0,95
A0A0A2LXB2_Flavobacterium_beibuense
0,79
A3U9W7_Croceibacter_atlanticus
0,12
D7VW92_Chryseobacterium_gleum
1
Fcan01_24144_PA
0,77
0,93
M0RC77_Rattus_norvegicus
Q63HM1_Homo_sapiens
B1AQL0_Mus_musculus
Q8K4H1_Mus_musculus
0.5

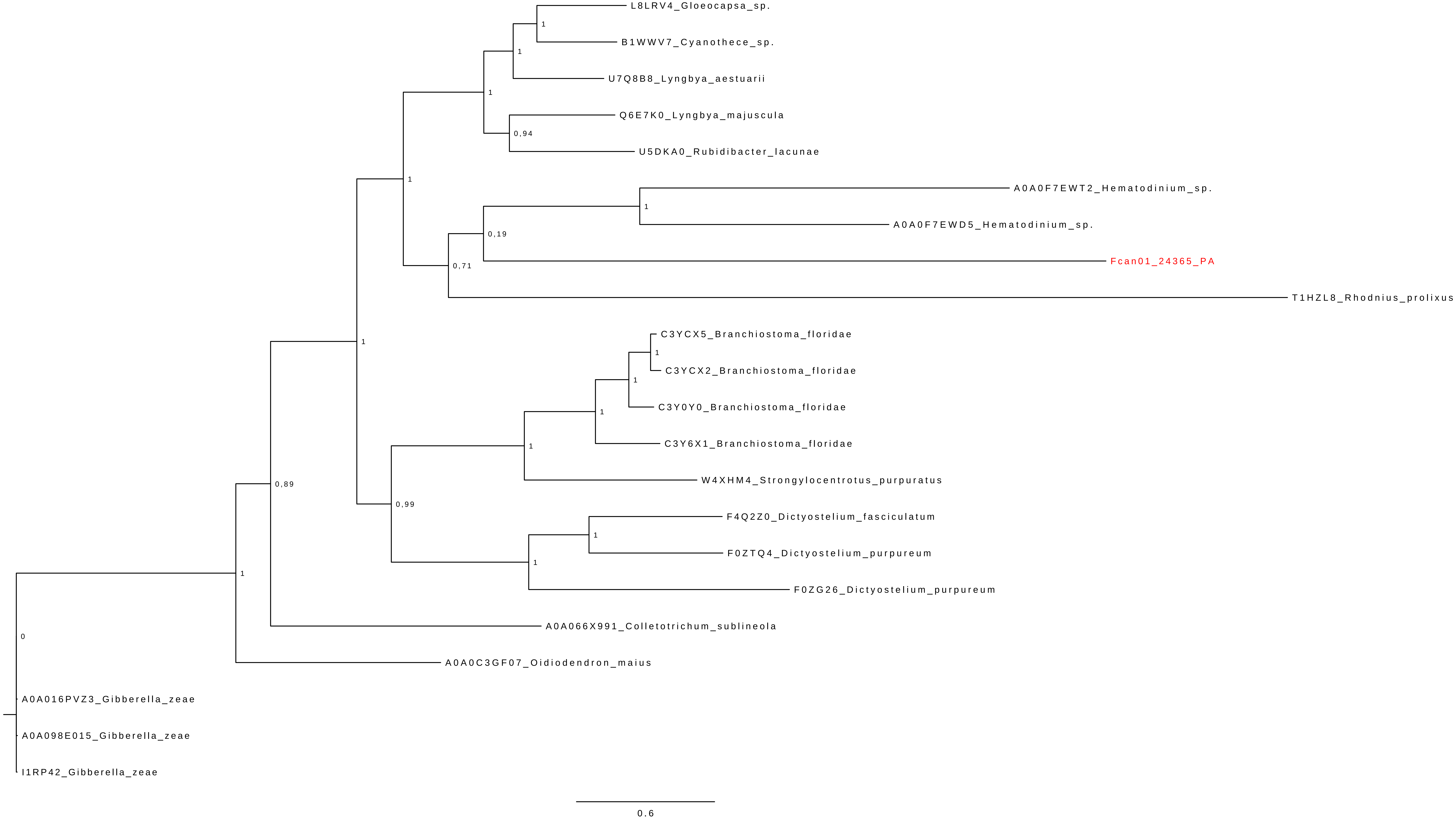

L8LRV4_Gloeocapsa_sp.
B1WWV7_Cyanothece_sp.
U7Q8B8_Lyngbya_aestuarii
Q6E7K0_Lyngbya_majuscula
U5DKA0_Rubidibacter_lacunae
A0A0F7EWT2_Hematodinium_sp.
A0A0F7EWD5_Hematodinium_sp.
Fcan01_24365_PA
T1HZL8_Rhodnius_prolixus
C3YCX5_Branchiostoma_floridae
C3YCX2_Branchiostoma_floridae
C3Y0Y0_Branchiostoma_floridae
C3Y6X1_Branchiostoma_floridae
W4XHM4_Strongylocentrotus_purpuratus
F4Q2Z0_Dictyostelium_fasciculatum
F0ZTQ4_Dictyostelium_purpureum
F0ZG26_Dictyostelium_purpureum
A0A066X991_Colletotrichum_sublineola
A0A0C3GF07_Oidiodendron_maius
A0A016PVZ3_Gibberella_zeae
A0A098E015_Gibberella_zeae
I1RP42_Gibberella_zeae
1
1
1
0,94
1
1
0,19
0,71
1
1
1
1
1
0,99
1
1
0,89
1
0
0.6

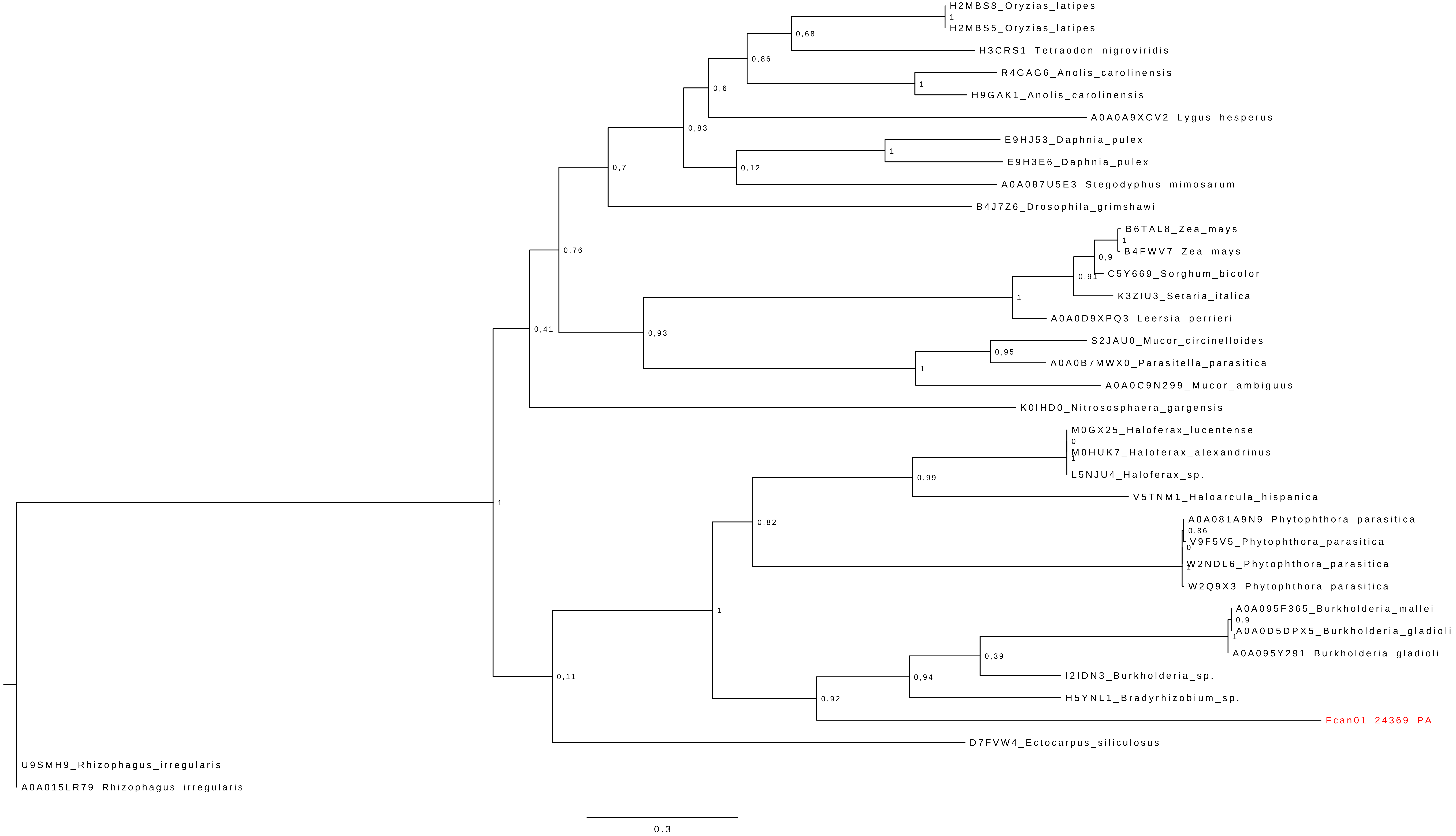
H2MBS8_Oryzias_latipes
H2MBS5_Oryzias_latipes
H3CRS1_Tetraodon_nigroviridis
R4GAG6_Anolis_carolinensis
H9GAK1_Anolis_carolinensis
A0A0A9XCV2_Lygus_hesperus
E9HJ53_Daphnia_pulex
E9H3E6_Daphnia_pulex
A0A087U5E3_Stegodyphus_mimosarum
B4J7Z6_Drosophila_grimshawi
B6TAL8_Zea_mays
B4FWV7_Zea_mays
C5Y669_Sorghum_bicolor
K3ZIU3_Setaria_italica
A0A0D9XPQ3_Leersia_perrieri
S2JAU0_Mucor_circinelloides
A0A0B7MWX0_Parasitella_parasitica
A0A0C9N299_Mucor_ambiguus
K0IHD0_Nitrososphaera_gargensis
M0GX25_Haloferax_lucentense
M0HUK7_Haloferax_alexandrinus
L5NJU4_Haloferax_sp.
V5TNM1_Haloarcula_hispanica
A0A081A9N9_Phytophthora_parasitica
V9F5V5_Phytophthora_parasitica
W2NDL6_Phytophthora_parasitica
W2Q9X3_Phytophthora_parasitica
A0A095F365_Burkholderia_mallei
A0A0D5DPX5_Burkholderia_gladioli
A0A095Y291_Burkholderia_gladioli
I2IDN3_Burkholderia_sp.
H5YNL1_Bradyrhizobium_sp.
Fcan01_24369_PA
D7FVW4_Ectocarpus_siliculosus
U9SMH9_Rhizophagus_irregularis
A0A015LR79_Rhizophagus_irregularis
1
0,68
0,86
1
0,6
0,83
1
0,12
0,7
1
0,9
0,91
1
0,95
1
0,93
0,76
0,41
0
1
0,99
0,86
0
1
0,82
0,9
1
0,39
0,94
0,92
1
0,11
1
0.3

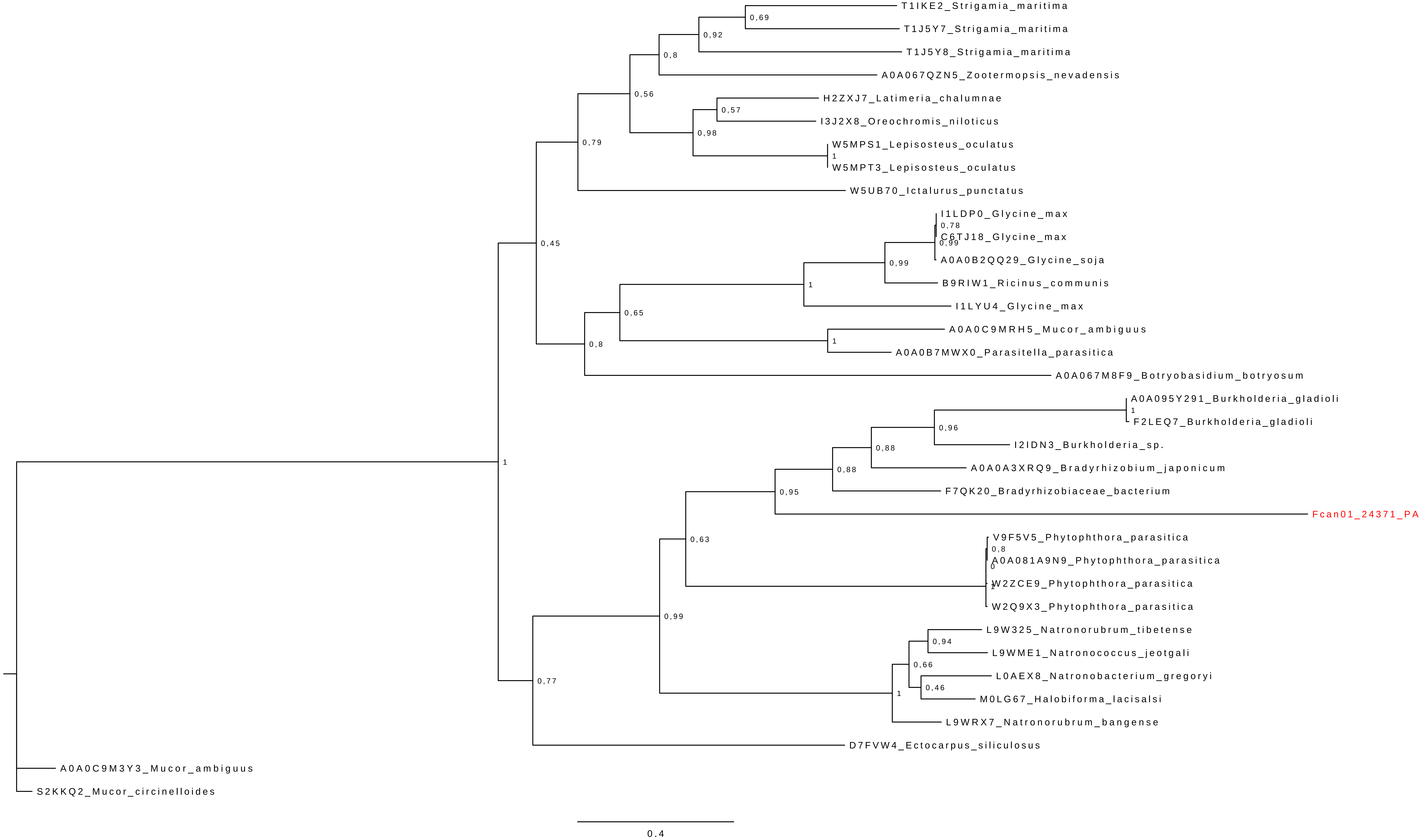
T1IKE2_Strigamia_maritima
T1J5Y7_Strigamia_maritima
T1J5Y8_Strigamia_maritima
A0A067QZN5_Zootermopsis_nevadensis
H2ZXJ7_Latimeria_chalumnae
I3J2X8_Oreochromis_niloticus
W5MPS1_Lepisosteus_oculatus
W5MPT3_Lepisosteus_oculatus
W5UB70_Ictalurus_punctatus
I1LDP0_Glycine_max
C6TJ18_Glycine_max
A0A0B2QQ29_Glycine_soja
B9RIW1_Ricinus_communis
I1LYU4_Glycine_max
A0A0C9MRH5_Mucor_ambiguus
A0A0B7MWX0_Parasitella_parasitica
A0A067M8F9_Botryobasidium_botryosum
A0A095Y291_Burkholderia_gladioli
F2LEQ7_Burkholderia_gladioli
I2IDN3_Burkholderia_sp.
A0A0A3XRQ9_Bradyrhizobium_japonicum
F7QK20_Bradyrhizobiaceae_bacterium
Fcan01_24371_PA
V9F5V5_Phytophthora_parasitica
A0A081A9N9_Phytophthora_parasitica
W2ZCE9_Phytophthora_parasitica
W2Q9X3_Phytophthora_parasitica
L9W325_Natronorubrum_tibetense
L9WME1_Natronococcus_jeotgali
L0AEX8_Natronobacterium_gregoryi
M0LG67_Halobiforma_lacisalsi
L9WRX7_Natronorubrum_bangense
D7FVW4_Ectocarpus_siliculosus
A0A0C9M3Y3_Mucor_ambiguus
S2KKQ2_Mucor_circinelloides
0.4

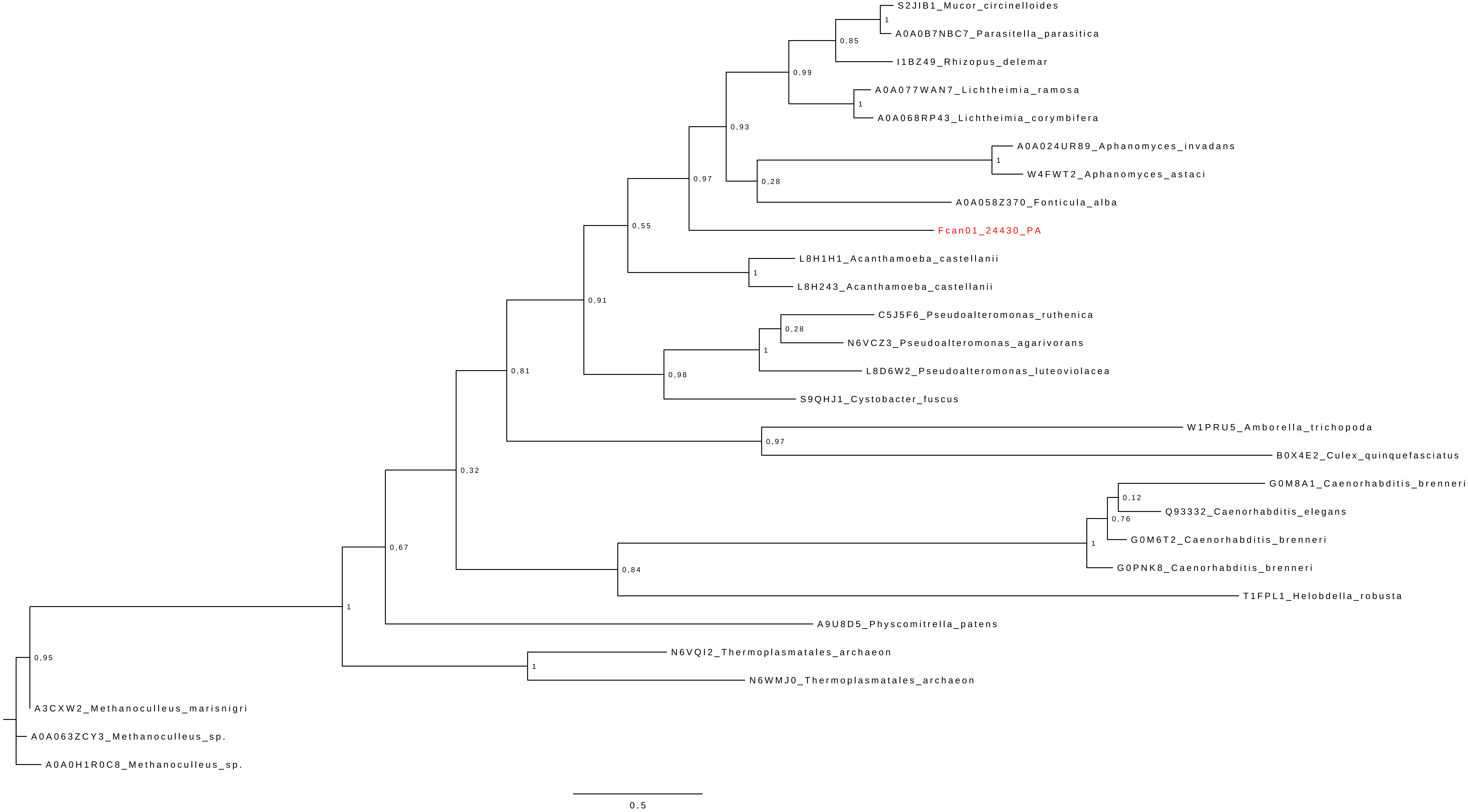

S2JIB1_Mucor_circinelloides
A0A0B7NBC7_Parasitella_parasitica
I1BZ49_Rhizopus_delemar
A0A077WAN7_Lichtheimia_ramosa
A0A068RP43_Lichtheimia_corymbifera
A0A024UR89_Aphanomyces_invadans
W4FWT2_Aphanomyces_astaci
A0A058Z370_Fonticula_alba
Fcan01_24430_PA
L8H1H1_Acanthamoeba_castellanii
L8H243_Acanthamoeba_castellanii
C5J5F6_Pseudoalteromonas_ruthenica
N6VCZ3_Pseudoalteromonas_agarivorans
L8D6W2_Pseudoalteromonas_luteoviolacea
S9QHJ1_Cystobacter_fuscus
W1PRU5_Amborella_trichopoda
B0X4E2_Culex_quinquefasciatus
G0M8A1_Caenorhabditis_brenneri
Q93332_Caenorhabditis_elegans
G0M6T2_Caenorhabditis_brenneri
G0PNK8_Caenorhabditis_brenneri
T1FPL1_Helobdella_robusta
A9U8D5_Physcomitrella_patens
N6VQI2_Thermoplasmatales_archaeon
N6WMJ0_Thermoplasmatales_archaeon
A3CXW2_Methanoculleus_marisnigri
A0A063ZCY3_Methanoculleus_sp.
A0A0H1R0C8_Methanoculleus_sp.
1
0,85
0,99
1
0,93
1
0,28
0,97
0,55
1
0,91
0,28
1
0,98
0,81
0,97
0,32
0,12
0,76
1
0,84
0,67
1
1
0,95
0.5

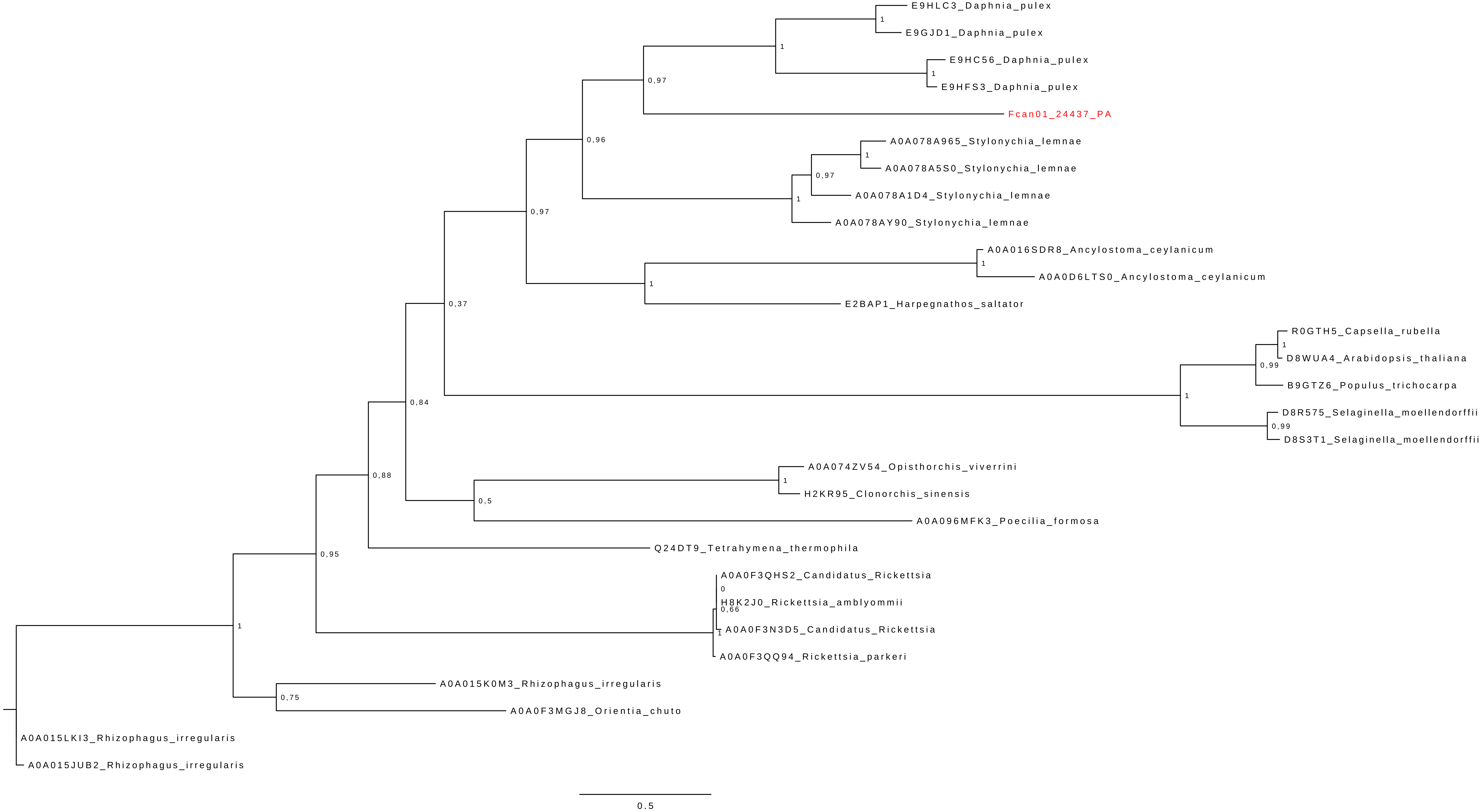
E9HLC3_Daphnia_pulex
E9GJD1_Daphnia_pulex
E9HC56_Daphnia_pulex
E9HFS3_Daphnia_pulex
Fcan01_24437_PA
A0A078A965_Stylonychia_lemnae
A0A078A5S0_Stylonychia_lemnae
A0A078A1D4_Stylonychia_lemnae
A0A078AY90_Stylonychia_lemnae
A0A016SDR8_Ancylostoma_ceylanicum
A0A0D6LTS0_Ancylostoma_ceylanicum
E2BAP1_Harpegnathos_saltator
R0GTH5_Capsella_rubella
D8WUA4_Arabidopsis_thaliana
B9GTZ6_Populus_trichocarpa
D8R575_Selaginella_moellendorffii
D8S3T1_Selaginella_moellendorffii
A0A074ZV54_Opisthorchis_viverrini
H2KR95_Clonorchis_sinensis
A0A096MFK3_Poecilia_formosa
Q24DT9_Tetrahymena_thermophila
A0A0F3QHS2_Candidatus_Rickettsia
H8K2J0_Rickettsia_amblyommii
A0A0F3N3D5_Candidatus_Rickettsia
A0A0F3QQ94_Rickettsia_parkeri
A0A015K0M3_Rhizophagus_irregularis
A0A0F3MGJ8_Orientia_chuto
A0A015LKI3_Rhizophagus_irregularis
A0A015JUB2_Rhizophagus_irregularis
0,97
0,96
0,97
0,37
0,84
0,88
0,95
0,99
0,5
0,66
0,75
0
1
0.5

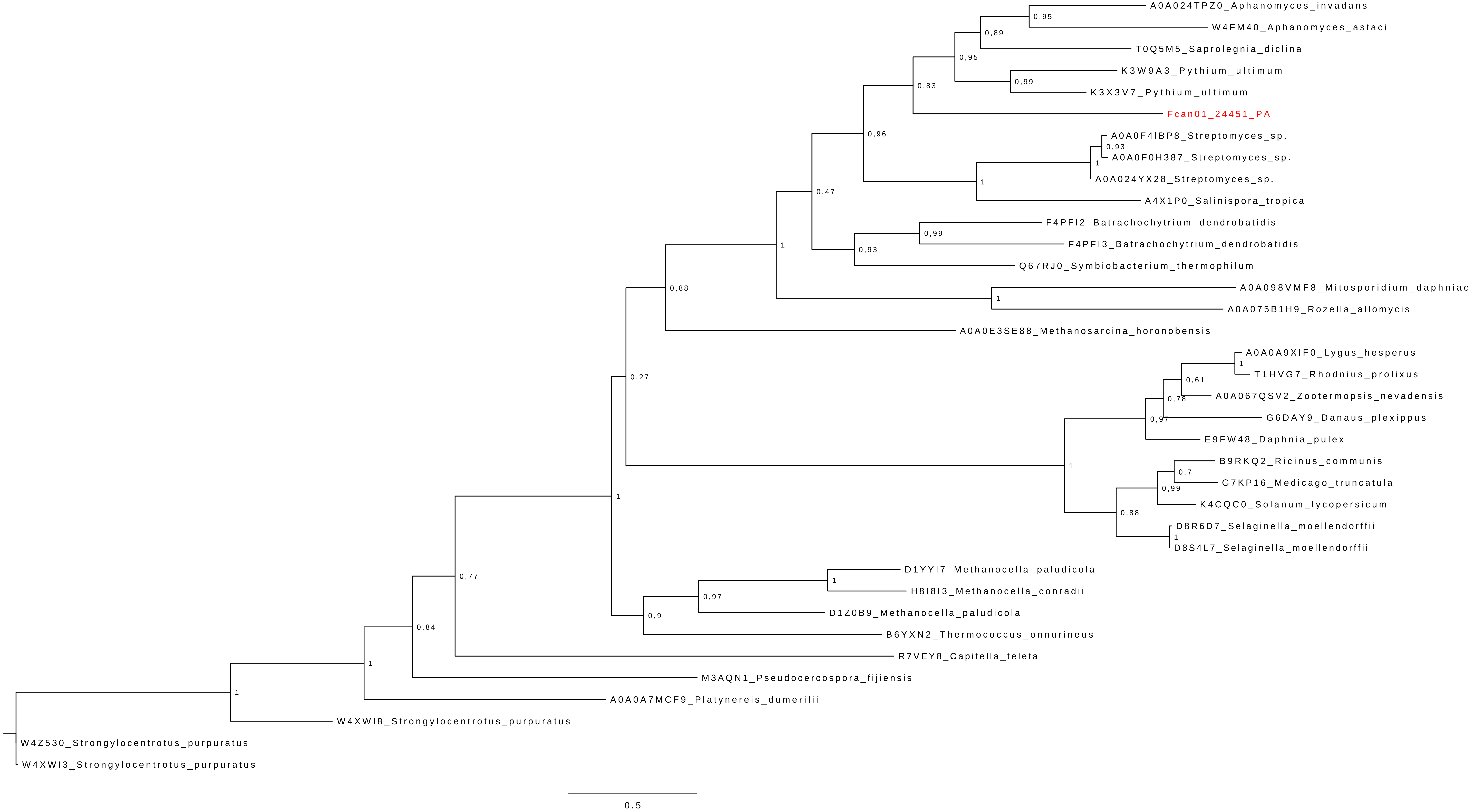
A0A024TPZ0_Aphanomyces_invadans
W4FM40_Aphanomyces_astaci
T0Q5M5_Saprolegnia_diclina
K3W9A3_Pythium_ultimum
K3X3V7_Pythium_ultimum
Fcan01_24451_PA
A0A0F4IBP8_Streptomyces_sp.
A0A0F0H387_Streptomyces_sp.
A0A024YX28_Streptomyces_sp.
A4X1P0_Salinispora_tropica
F4PFI2_Batrachochytrium_dendrobatidis
F4PFI3_Batrachochytrium_dendrobatidis
Q67RJ0_Symbiobacterium_thermophilum
A0A098VMF8_Mitosporidium_daphniae
A0A075B1H9_Rozella_allomycis
A0A0E3SE88_Methanosarcina_horonobensis
A0A0A9XIF0_Lygus_hesperus
T1HVG7_Rhodnius_prolixus
A0A067QSV2_Zootermopsis_nevadensis
G6DAY9_Danaus_plexippus
E9FW48_Daphnia_pulex
B9RKQ2_Ricinus_communis
G7KP16_Medicago_truncatula
K4CQC0_Solanum_lycopersicum
D8R6D7_Selaginella_moellendorffii
D8S4L7_Selaginella_moellendorffii
D1YYI7_Methanocella_paludicola
H8I8I3_Methanocella_conradii
D1Z0B9_Methanocella_paludicola
B6YXN2_Thermococcus_onnurineus
R7VEY8_Capitella_teleta
M3AQN1_Pseudocercospora_fijiensis
A0A0A7MCF9_Platynereis_dumerilii
W4XWI8_Strongylocentrotus_purpuratus
W4Z530_Strongylocentrotus_purpuratus
W4XWI3_Strongylocentrotus_purpuratus
0.5

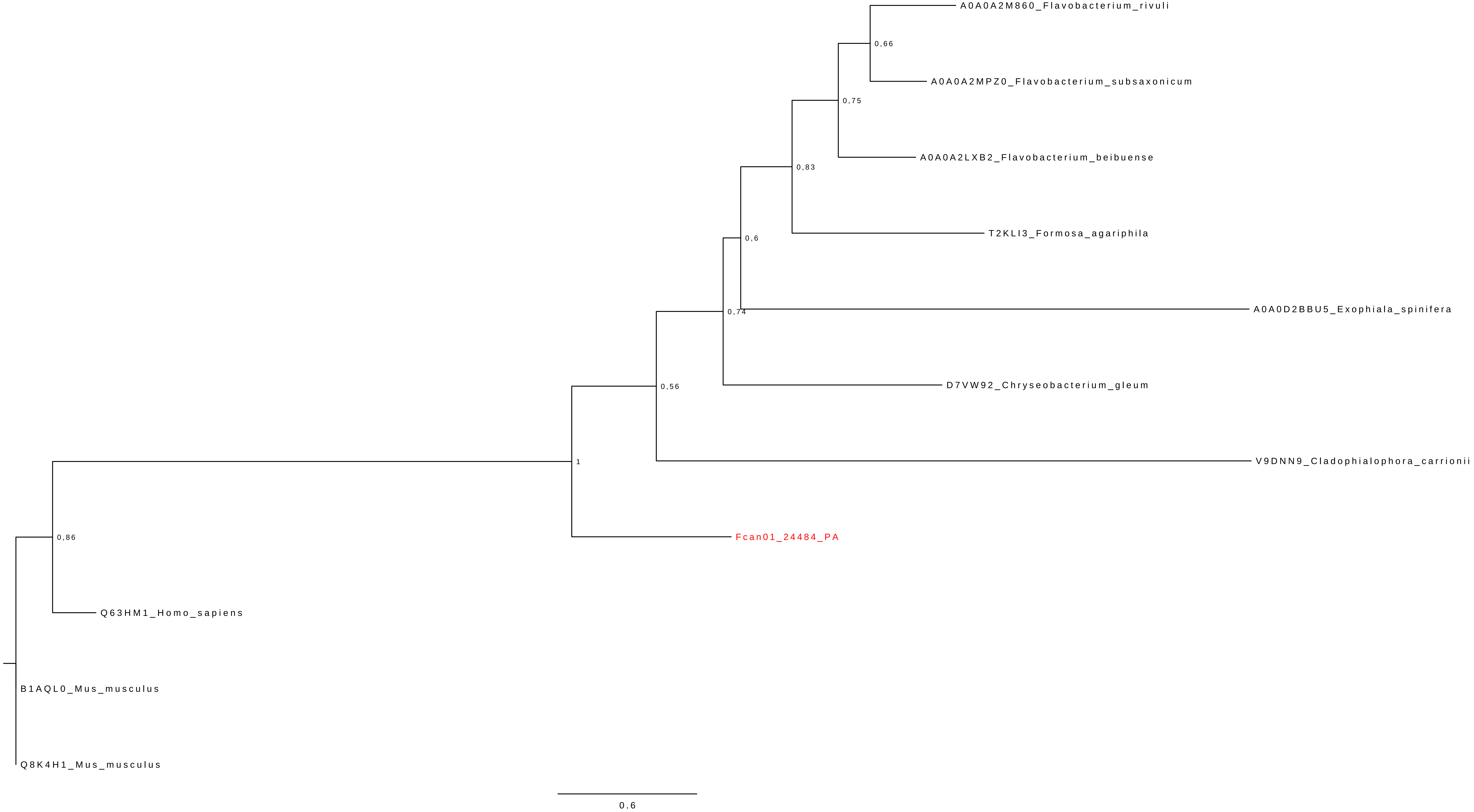

A0A0A2M860_Flavobacterium_rivuli
0,66
A0A0A2MPZ0_Flavobacterium_subsaxonicum
0,75
A0A0A2LXB2_Flavobacterium_beibuense
0,83
T2KLI3_Formosa_agariphila
0,6
A0A0D2BBU5_Exophiala_spinifera
0,74
D7VW92_Chryseobacterium_gleum
0,56
1
V9DNN9_Cladophialophora_carrionii
Fcan01_24484_PA
0,86
Q63HM1_Homo_sapiens
B1AQL0_Mus_musculus
Q8K4H1_Mus_musculus
0.6

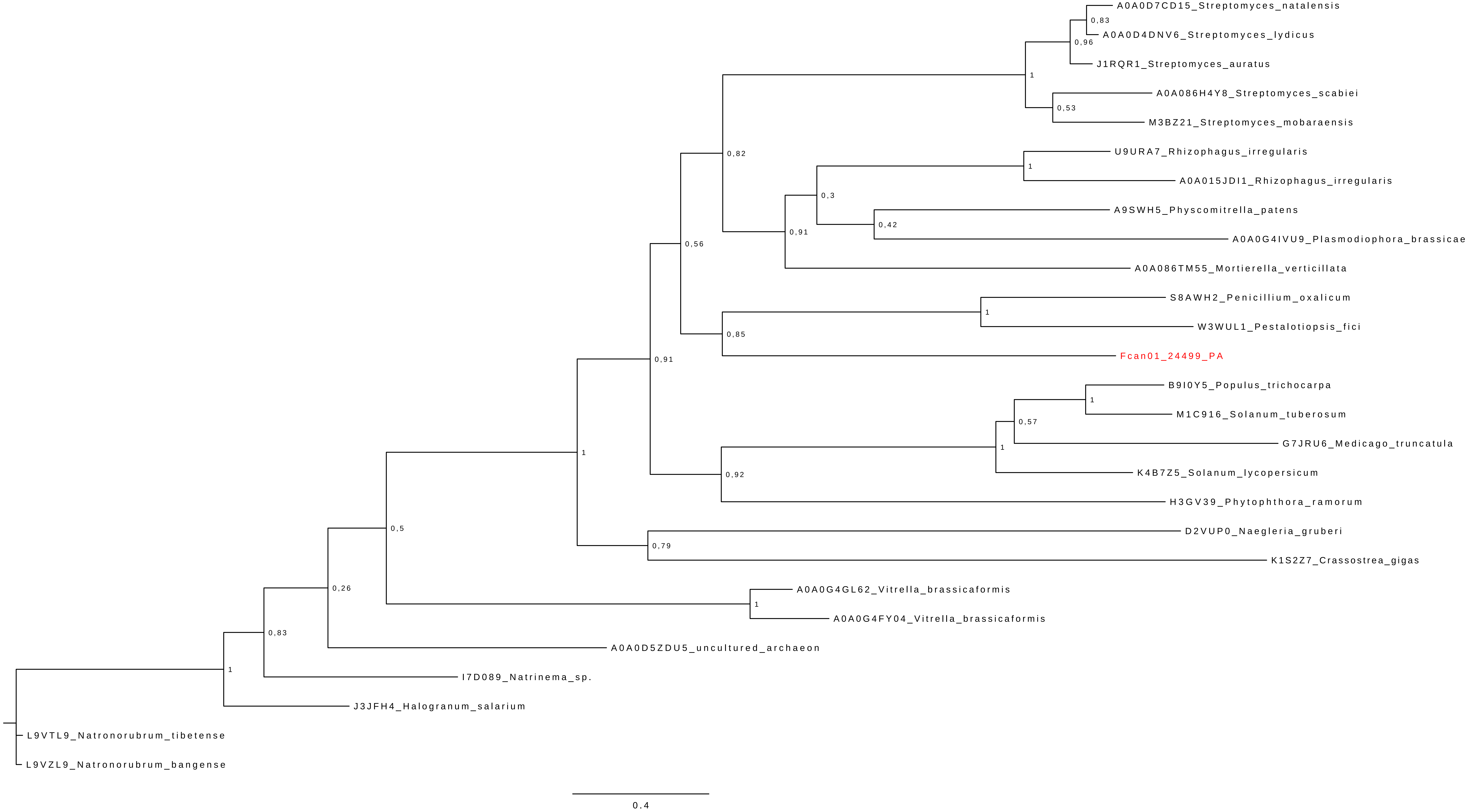
A0A0D7CD15_Streptomyces_natalensis
0,83
A0A0D4DNV6_Streptomyces_lydicus
0,96
J1RQR1_Streptomyces_auratus
1
A0A086H4Y8_Streptomyces_scabiei
0,53
M3BZ21_Streptomyces_mobaraensis
U9URA7_Rhizophagus_irregularis
0,82
1
A0A015JDI1_Rhizophagus_irregularis
0,3
A9SWH5_Physcomitrella_patens
0,42
0,56
0,91
A0A0G4IVU9_Plasmodiophora_brassicae
A0A086TM55_Mortierella_verticillata
S8AWH2_Penicillium_oxalicum
1
W3WUL1_Pestalotiopsis_fici
0,85
Fcan01_24499_PA
0,91
B9I0Y5_Populus_trichocarpa
1
M1C916_Solanum_tuberosum
0,57
G7JRU6_Medicago_truncatula
1
1
K4B7Z5_Solanum_lycopersicum
0,92
H3GV39_Phytophthora_ramorum
D2VUP0_Naegleria_gruberi
0,5
0,79
K1S2Z7_Crassostrea_gigas
A0A0G4GL62_Vitrella_brassicaformis
0,26
1
A0A0G4FY04_Vitrella_brassicaformis
0,83
A0A0D5ZDU5_uncultured_archaeon
1
I7D089_Natrinema_sp.
J3JFH4_Halogranum_salarium
L9VTL9_Natronorubrum_tibetense
L9VZL9_Natronorubrum_bangense
0.4

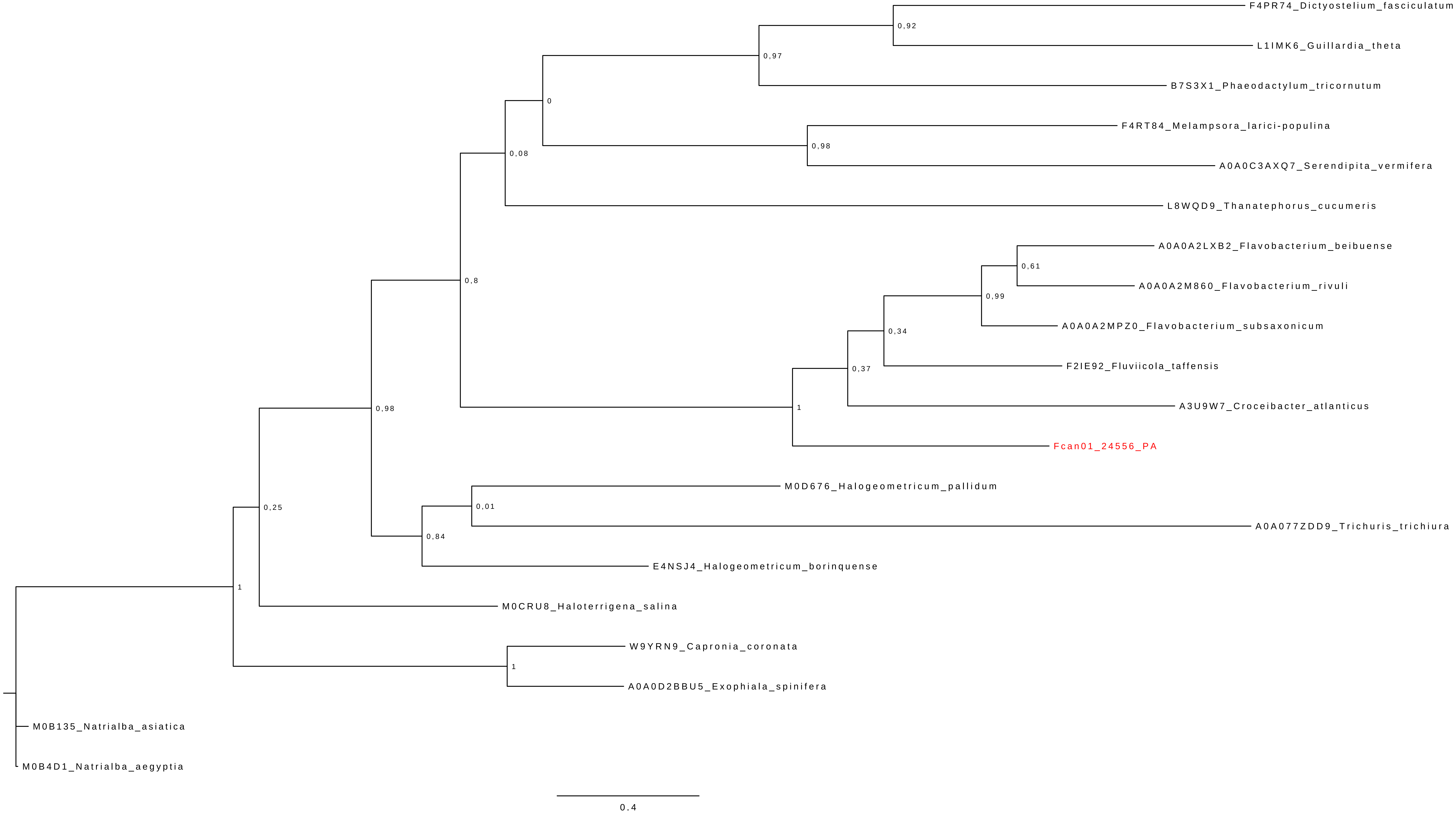
F4PR74_Dictyostelium_fasciculatum
0,92
L1IMK6_Guillardia_theta
0,97
B7S3X1_Phaeodactylum_tricornutum
0
F4RT84_Melampsora_larici-populina
0,98
A0A0C3AXQ7_Serendipita_vermifera
0,08
L8WQD9_Thanatephorus_cucumeris
A0A0A2LXB2_Flavobacterium_beibuense
0,61
A0A0A2M860_Flavobacterium_rivuli
0,99
A0A0A2MPZ0_Flavobacterium_subsaxonicum
0,34
F2IE92_Fluviicola_taffensis
0,37
0,8
A3U9W7_Croceibacter_atlanticus
1
0,98
Fcan01_24556_PA
M0D676_Halogeometricum_pallidum
0,25
0,01
A0A077ZDD9_Trichuris_trichiura
0,84
E4NSJ4_Halogeometricum_borinquense
1
M0CRU8_Haloterrigena_salina
W9YRN9_Capronia_coronata
1
A0A0D2BBU5_Exophiala_spinifera
M0B135_Natrialba_asiatica
M0B4D1_Natrialba_aegyptia
0.4

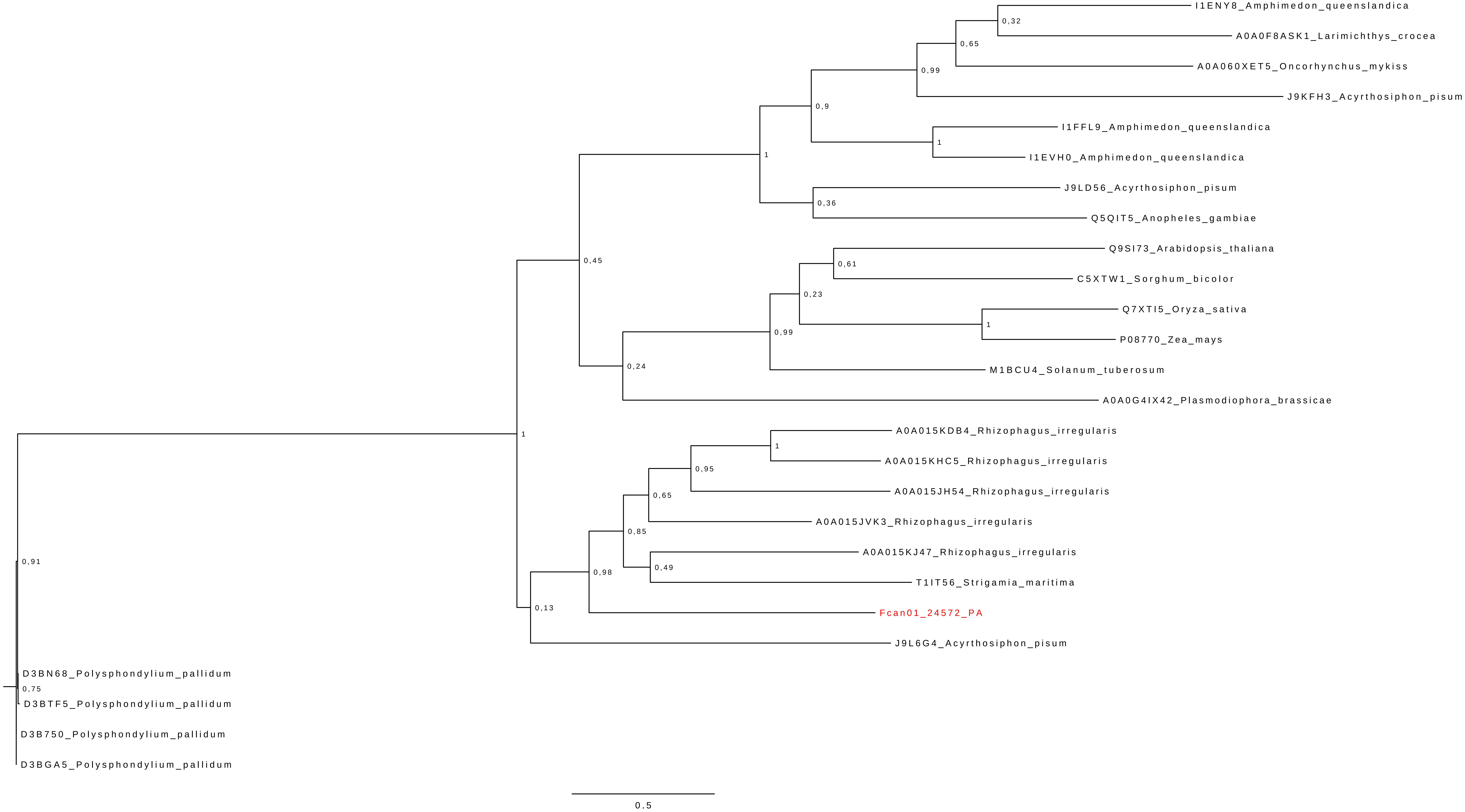
I1ENY8_Amphimedon_queenslandica
0,32
A0A0F8ASK1_Larimichthys_crocea
0,65
A0A060XET5_Oncorhynchus_mykiss
0,99
J9KFH3_Acyrthosiphon_pisum
0,9
I1FFL9_Amphimedon_queenslandica
1
I1EVH0_Amphimedon_queenslandica
1
J9LD56_Acyrthosiphon_pisum
0,36
Q5QIT5_Anopheles_gambiae
0,45
Q9SI73_Arabidopsis_thaliana
0,61
C5XTW1_Sorghum_bicolor
0,23
Q7XTI5_Oryza_sativa
1
P08770_Zea_mays
0,99
M1BCU4_Solanum_tuberosum
0,24
A0A0G4IX42_Plasmodiophora_brassicae
1
A0A015KDB4_Rhizophagus_irregularis
1
A0A015KHC5_Rhizophagus_irregularis
0,95
A0A015JH54_Rhizophagus_irregularis
0,65
A0A015JVK3_Rhizophagus_irregularis
0,85
A0A015KJ47_Rhizophagus_irregularis
0,49
T1IT56_Strigamia_maritima
0,98
Fcan01_24572_PA
0,13
J9L6G4_Acyrthosiphon_pisum
0,91
D3BN68_Polysphondylium_pallidum
0,75
D3BTF5_Polysphondylium_pallidum
D3B750_Polysphondylium_pallidum
D3BGA5_Polysphondylium_pallidum
0.5

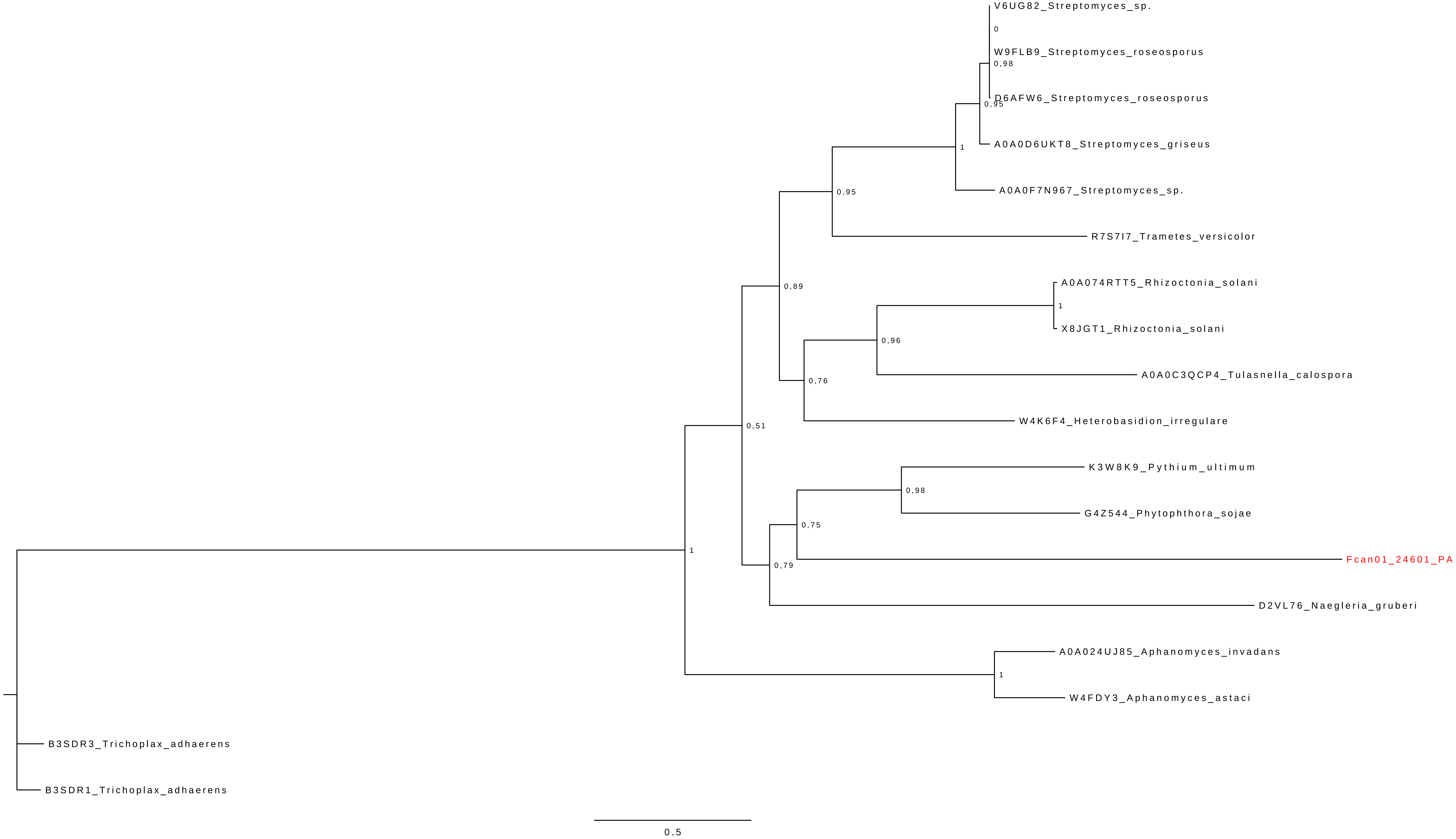
V6UG82_Streptomyces_sp.
0
W9FLB9_Streptomyces_roseosporus
0,98
D6AFW6_Streptomyces_roseosporus
0,95
A0A0D6UKT8_Streptomyces_griseus
1
A0A0F7N967_Streptomyces_sp.
0,95
R7S7I7_Trametes_versicolor
A0A074RTT5_Rhizoctonia_solani
0,89
1
X8JGT1_Rhizoctonia_solani
0,96
A0A0C3QCP4_Tulasnella_calospora
0,76
W4K6F4_Heterobasidion_irregulare
0,51
K3W8K9_Pythium_ultimum
0,98
G4Z544_Phytophthora_sojae
0,75
1
Fcan01_24601_PA
0,79
D2VL76_Naegleria_gruberi
A0A024UJ85_Aphanomyces_invadans
1
W4FDY3_Aphanomyces_astaci
B3SDR3_Trichoplax_adhaerens
B3SDR1_Trichoplax_adhaerens
0.5

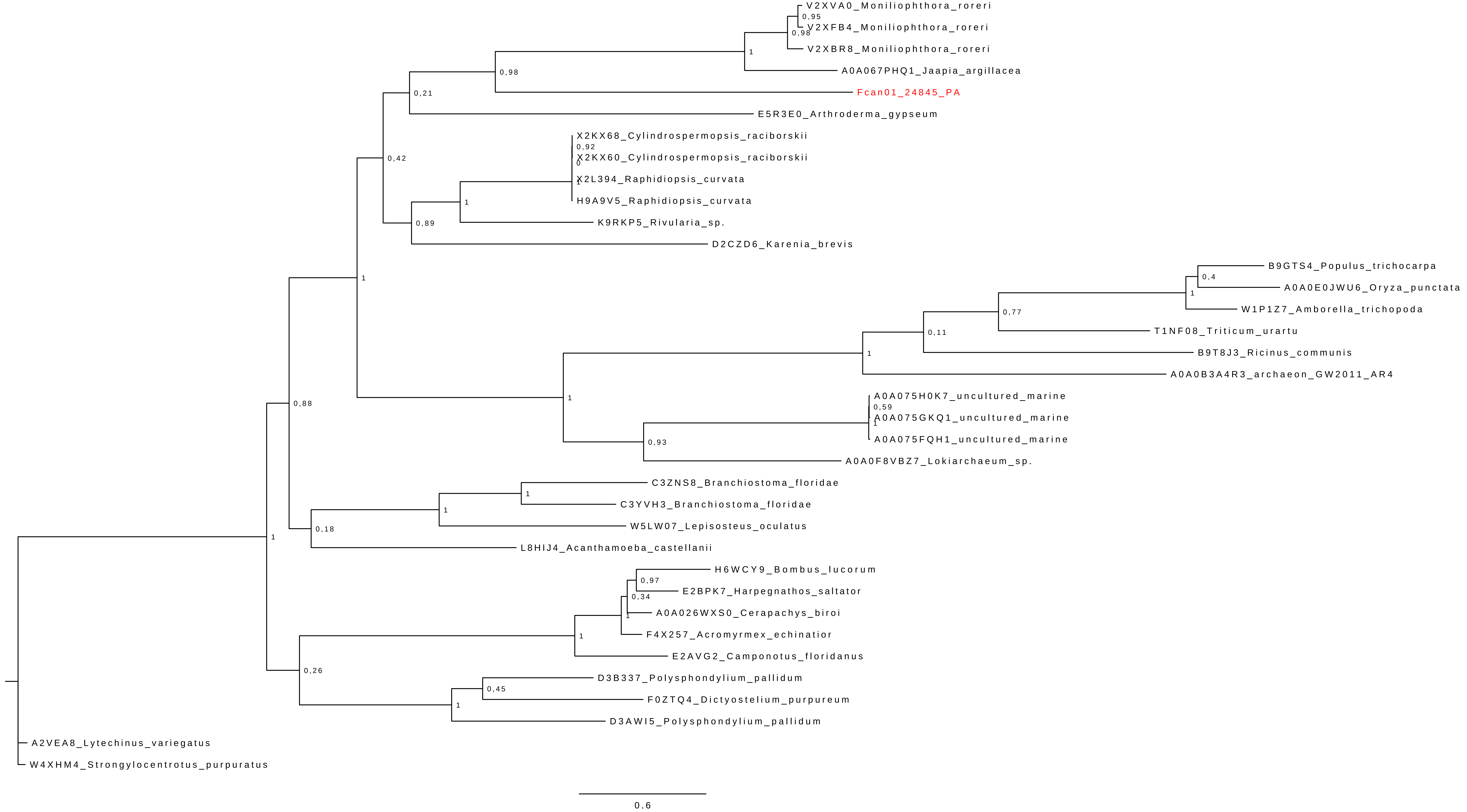
V2XVA0_Moniliophthora_roreri
V2XFB4_Moniliophthora_roreri
V2XBR8_Moniliophthora_roreri
A0A067PHQ1_Jaapia_argillacea
Fcan01_24845_PA
E5R3E0_Arthroderma_gypseum
X2KX68_Cylindrospermopsis_raciborskii
X2KX60_Cylindrospermopsis_raciborskii
X2L394_Raphidiopsis_curvata
H9A9V5_Raphidiopsis_curvata
K9RKP5_Rivularia_sp.
D2CZD6_Karenia_brevis
B9GTS4_Populus_trichocarpa
A0A0E0JWU6_Oryza_punctata
W1P1Z7_Amborella_trichopoda
T1NF08_Triticum_urartu
B9T8J3_Ricinus_communis
A0A0B3A4R3_archaeon_GW2011_AR4
A0A075H0K7_uncultured_marine
A0A075GKQ1_uncultured_marine
A0A075FQH1_uncultured_marine
A0A0F8VBZ7_Lokiarchaeum_sp.
C3ZNS8_Branchiostoma_floridae
C3YVH3_Branchiostoma_floridae
W5LW07_Lepisosteus_oculatus
L8HIJ4_Acanthamoeba_castellanii
H6WCY9_Bombus_lucorum
E2BPK7_Harpegnathos_saltator
A0A026WXS0_Cerapachys_biroi
F4X257_Acromyrmex_echinatior
E2AVG2_Camponotus_floridanus
D3B337_Polysphondylium_pallidum
F0ZTQ4_Dictyostelium_purpureum
D3AWI5_Polysphondylium_pallidum
A2VEA8_Lytechinus_variegatus
W4XHM4_Strongylocentrotus_purpuratus
0.6

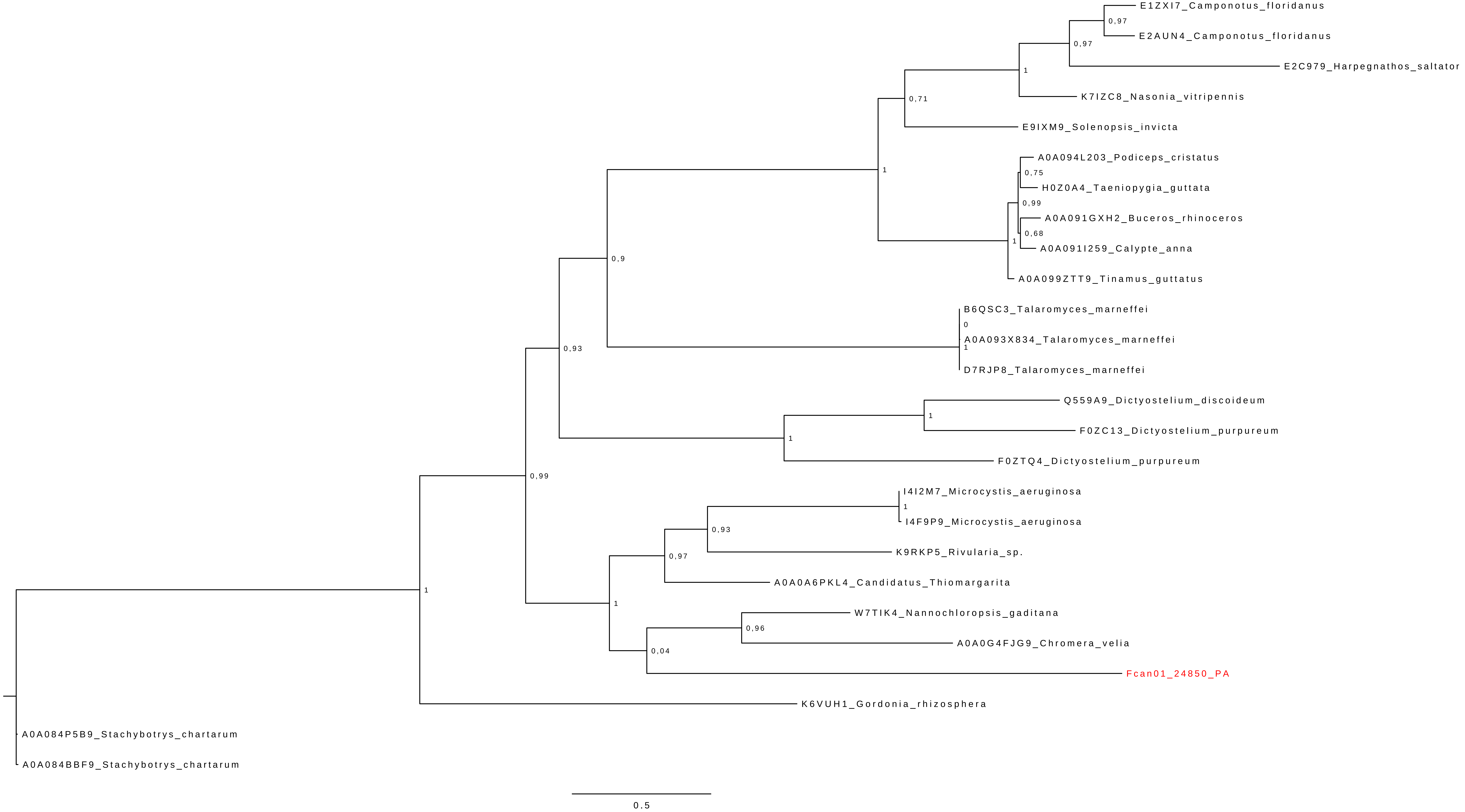
E1ZXI7_Camponotus_floridanus
0,97
E2AUN4_Camponotus_floridanus
0,97
E2C979_Harpegnathos_saltator
1
K7IZC8_Nasonia_vitripennis
0,71
E9IXM9_Solenopsis_invicta
A0A094L203_Podiceps_cristatus
0,75
H0Z0A4_Taeniopygia_guttata
0,99
A0A091GXH2_Buceros_rhinoceros
0,68
A0A091I259_Calypte_anna
1
A0A099ZTT9_Tinamus_guttatus
1
0,9
B6QSC3_Talaromyces_marneffei
0
A0A093X834_Talaromyces_marneffei
1
D7RJP8_Talaromyces_marneffei
0,93
Q559A9_Dictyostelium_discoideum
1
F0ZC13_Dictyostelium_purpureum
1
F0ZTQ4_Dictyostelium_purpureum
0,99
I4I2M7_Microcystis_aeruginosa
1
I4F9P9_Microcystis_aeruginosa
0,93
K9RKP5_Rivularia_sp.
0,97
A0A0A6PKL4_Candidatus_Thiomargarita
1
W7TIK4_Nannochloropsis_gaditana
0,96
A0A0G4FJG9_Chromera_velia
0,04
Fcan01_24850_PA
1
K6VUH1_Gordonia_rhizosphera
A0A084P5B9_Stachybotrys_chartarum
A0A084BBF9_Stachybotrys_chartarum
0.5

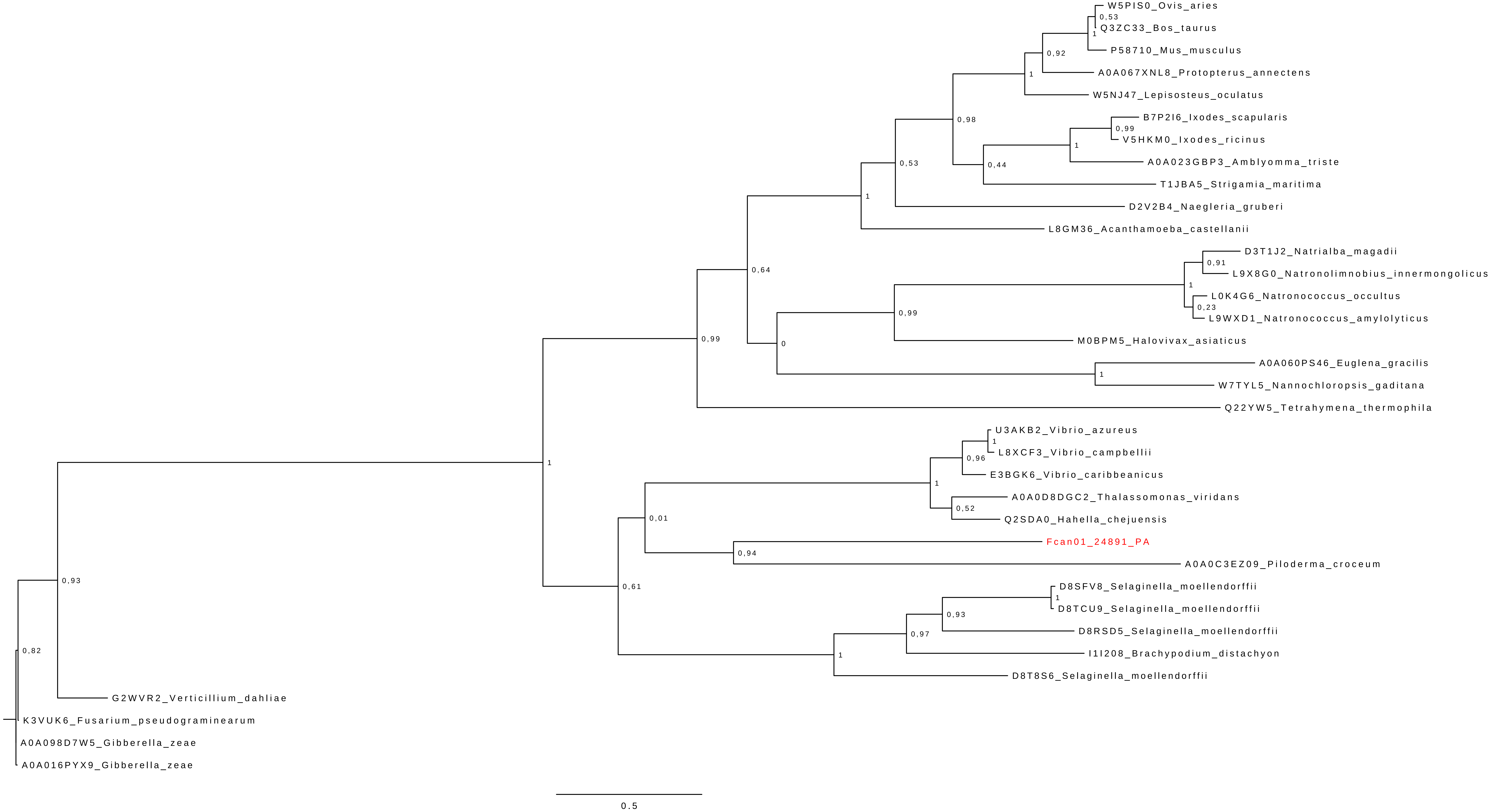
W5PIS0_Ovis_aries
0,53
Q3ZC33_Bos_taurus
1
P58710_Mus_musculus
0,92
A0A067XNL8_Protopterus_annectens
1
W5NJ47_Lepisosteus_oculatus
0,98
B7P2I6_Ixodes_scapularis
0,99
V5HKM0_Ixodes_ricinus
1
A0A023GBP3_Amblyomma_triste
0,44
T1JBA5_Strigamia_maritima
0,53
D2V2B4_Naegleria_gruberi
1
L8GM36_Acanthamoeba_castellanii
0,64
D3T1J2_Natrialba_magadii
0,91
L9X8G0_Natronolimnobius_innermongolicus
1
L0K4G6_Natronococcus_occultus
0,23
L9WXD1_Natronococcus_amylolyticus
0,99
M0BPM5_Halovivax_asiaticus
0
A0A060PS46_Euglena_gracilis
1
W7TYL5_Nannochloropsis_gaditana
0,99
Q22YW5_Tetrahymena_thermophila
1
U3AKB2_Vibrio_azureus
1
L8XCF3_Vibrio_campbellii
0,96
E3BGK6_Vibrio_caribbeanicus
1
A0A0D8DGC2_Thalassomonas_viridans
0,52
Q2SDA0_Hahella_chejuensis
0,01
Fcan01_24891_PA
0,94
A0A0C3EZ09_Piloderma_croceum
0,61
D8SFV8_Selaginella_moellendorffii
1
D8TCU9_Selaginella_moellendorffii
0,93
D8RSD5_Selaginella_moellendorffii
0,97
I1I208_Brachypodium_distachyon
1
D8T8S6_Selaginella_moellendorffii
0,93
0,82
G2WVR2_Verticillium_dahliae
K3VUK6_Fusarium_pseudograminearum
A0A098D7W5_Gibberella_zeae
A0A016PYX9_Gibberella_zeae
0.5

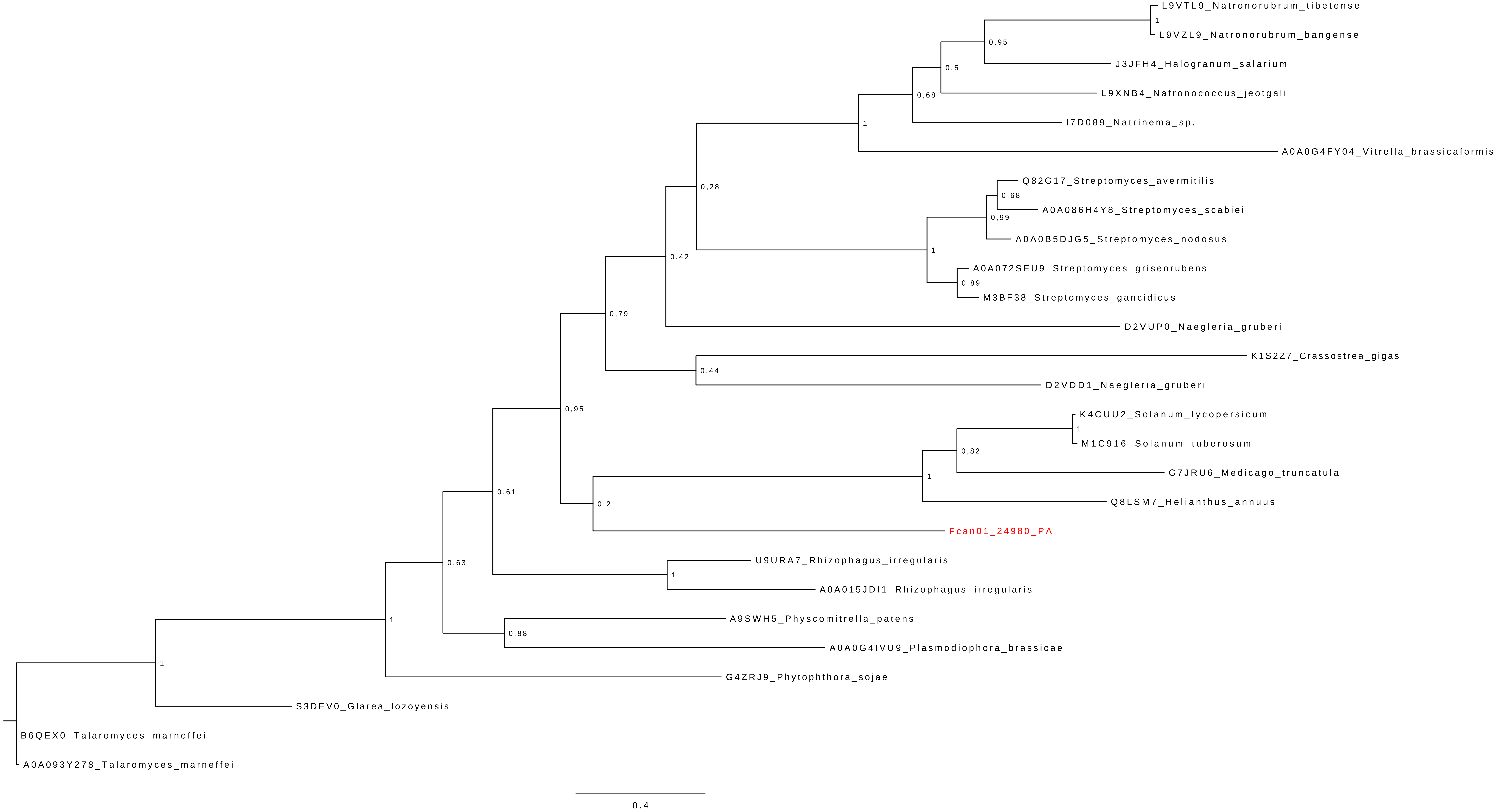
L9VTL9_Natronorubrum_tibetense
L9VZL9_Natronorubrum_bangense
J3JFH4_Halogranum_salarium
L9XNB4_Natronococcus_jeotgali
I7D089_Natrinema_sp.
A0A0G4FY04_Vitrella_brassicaformis
Q82G17_Streptomyces_avermitilis
A0A086H4Y8_Streptomyces_scabiei
A0A0B5DJG5_Streptomyces_nodosus
A0A072SEU9_Streptomyces_griseorubens
M3BF38_Streptomyces_gancidicus
D2VUP0_Naegleria_gruberi
K1S2Z7_Crassostrea_gigas
D2VDD1_Naegleria_gruberi
K4CUU2_Solanum_lycopersicum
M1C916_Solanum_tuberosum
G7JRU6_Medicago_truncatula
Q8LSM7_Helianthus_annuus
Fcan01_24980_PA
U9URA7_Rhizophagus_irregularis
A0A015JDI1_Rhizophagus_irregularis
A9SWH5_Physcomitrella_patens
A0A0G4IVU9_Plasmodiophora_brassicae
G4ZRJ9_Phytophthora_sojae
S3DEV0_Glarea_lozoyensis
B6QEX0_Talaromyces_marneffei
A0A093Y278_Talaromyces_marneffei
1
0,95
0,5
0,68
1
0,28
0,68
0,99
1
0,89
0,42
0,79
0,44
0,95
1
0,82
1
0,2
0,61
1
0,63
0,88
1
1
0.4

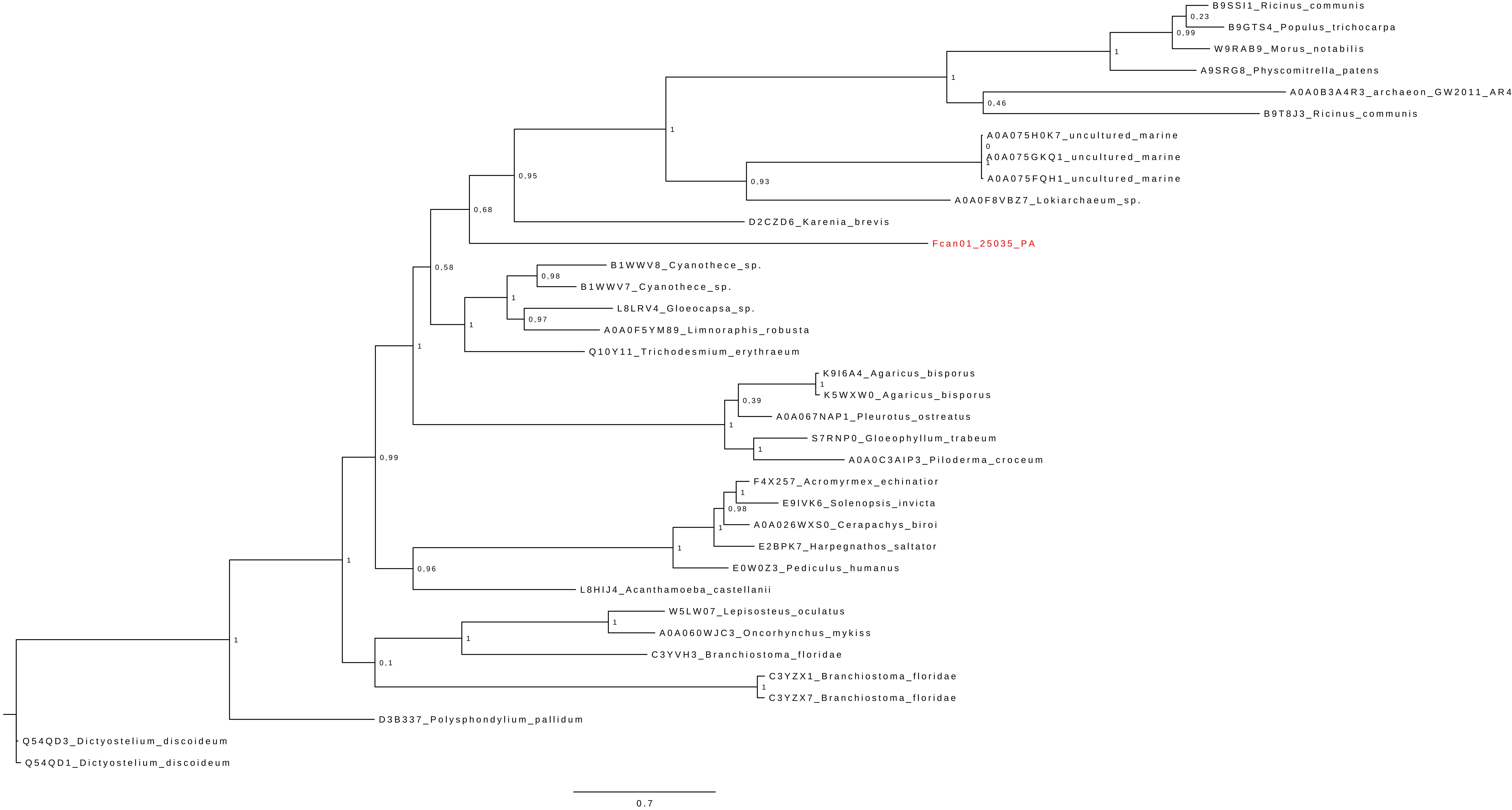
B9SSI1_Ricinus_communis
B9GTS4_Populus_trichocarpa
W9RAB9_Morus_notabilis
A9SRG8_Physcomitrella_patens
A0A0B3A4R3_archaeon_GW2011_AR4
B9T8J3_Ricinus_communis
A0A075H0K7_uncultured_marine
A0A075GKQ1_uncultured_marine
A0A075FQH1_uncultured_marine
A0A0F8VBZ7_Lokiarchaeum_sp.
D2CZD6_Karenia_brevis
Fcan01_25035_PA
B1WWV8_Cyanothece_sp.
B1WWV7_Cyanothece_sp.
L8LRV4_Gloeocapsa_sp.
A0A0F5YM89_Limnoraphis_robusta
Q10Y11_Trichodesmium_erythraeum
K9I6A4_Agaricus_bisporus
K5WXW0_Agaricus_bisporus
A0A067NAP1_Pleurotus_ostreatus
S7RNP0_Gloeophyllum_trabeum
A0A0C3AIP3_Piloderma_croceum
F4X257_Acromyrmex_echinatior
E9IVK6_Solenopsis_invicta
A0A026WXS0_Cerapachys_biroi
E2BPK7_Harpegnathos_saltator
E0W0Z3_Pediculus_humanus
L8HIJ4_Acanthamoeba_castellanii
W5LW07_Lepisosteus_oculatus
A0A060WJC3_Oncorhynchus_mykiss
C3YVH3_Branchiostoma_floridae
C3YZX1_Branchiostoma_floridae
C3YZX7_Branchiostoma_floridae
D3B337_Polysphondylium_pallidum
Q54QD3_Dictyostelium_discoideum
Q54QD1_Dictyostelium_discoideum
0,23
0,99
1
1
0,46
0
1
0,93
1
0,95
0,68
0,58
0,98
1
0,97
1
1
1
0,39
1
1
1
0,98
1
1
0,96
0,99
1
1
0,1
1
1
1
0.7

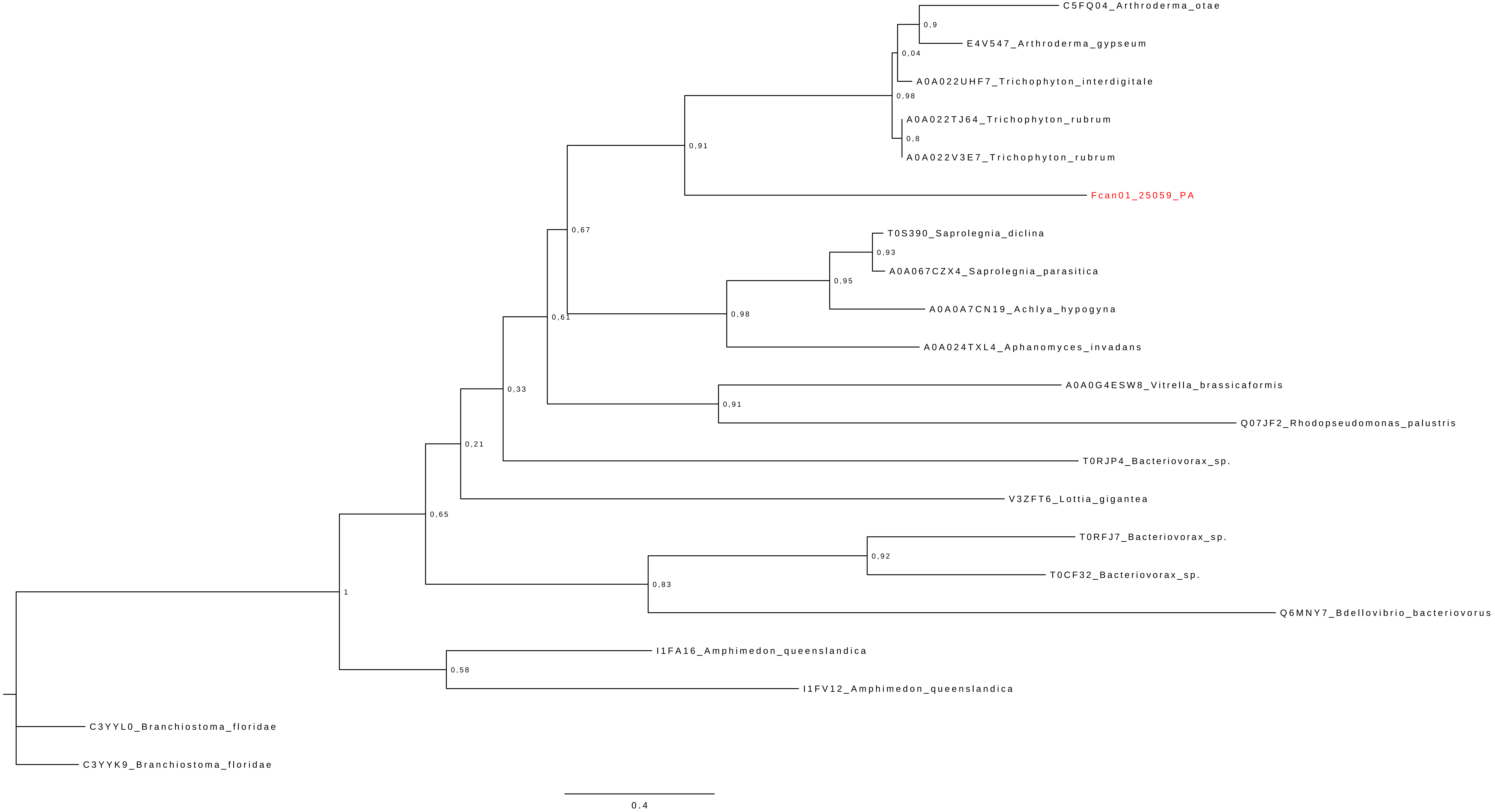
C5FQ04_Arthroderma_otae
0,9
E4V547_Arthroderma_gypseum
0,04
A0A022UHF7_Trichophyton_interdigitale
0,98
A0A022TJ64_Trichophyton_rubrum
0,8
A0A022V3E7_Trichophyton_rubrum
0,91
Fcan01_25059_PA
0,67
T0S390_Saprolegnia_diclina
0,93
A0A067CZX4_Saprolegnia_parasitica
0,95
A0A0A7CN19_Achlya_hypogyna
0,61
0,98
A0A024TXL4_Aphanomyces_invadans
A0A0G4ESW8_Vitrella_brassicaformis
0,33
0,91
Q07JF2_Rhodopseudomonas_palustris
0,21
T0RJP4_Bacteriovorax_sp.
V3ZFT6_Lottia_gigantea
0,65
T0RFJ7_Bacteriovorax_sp.
0,92
T0CF32_Bacteriovorax_sp.
0,83
1
Q6MNY7_Bdellovibrio_bacteriovorus
I1FA16_Amphimedon_queenslandica
0,58
I1FV12_Amphimedon_queenslandica
C3YYL0_Branchiostoma_floridae
C3YYK9_Branchiostoma_floridae
0.4

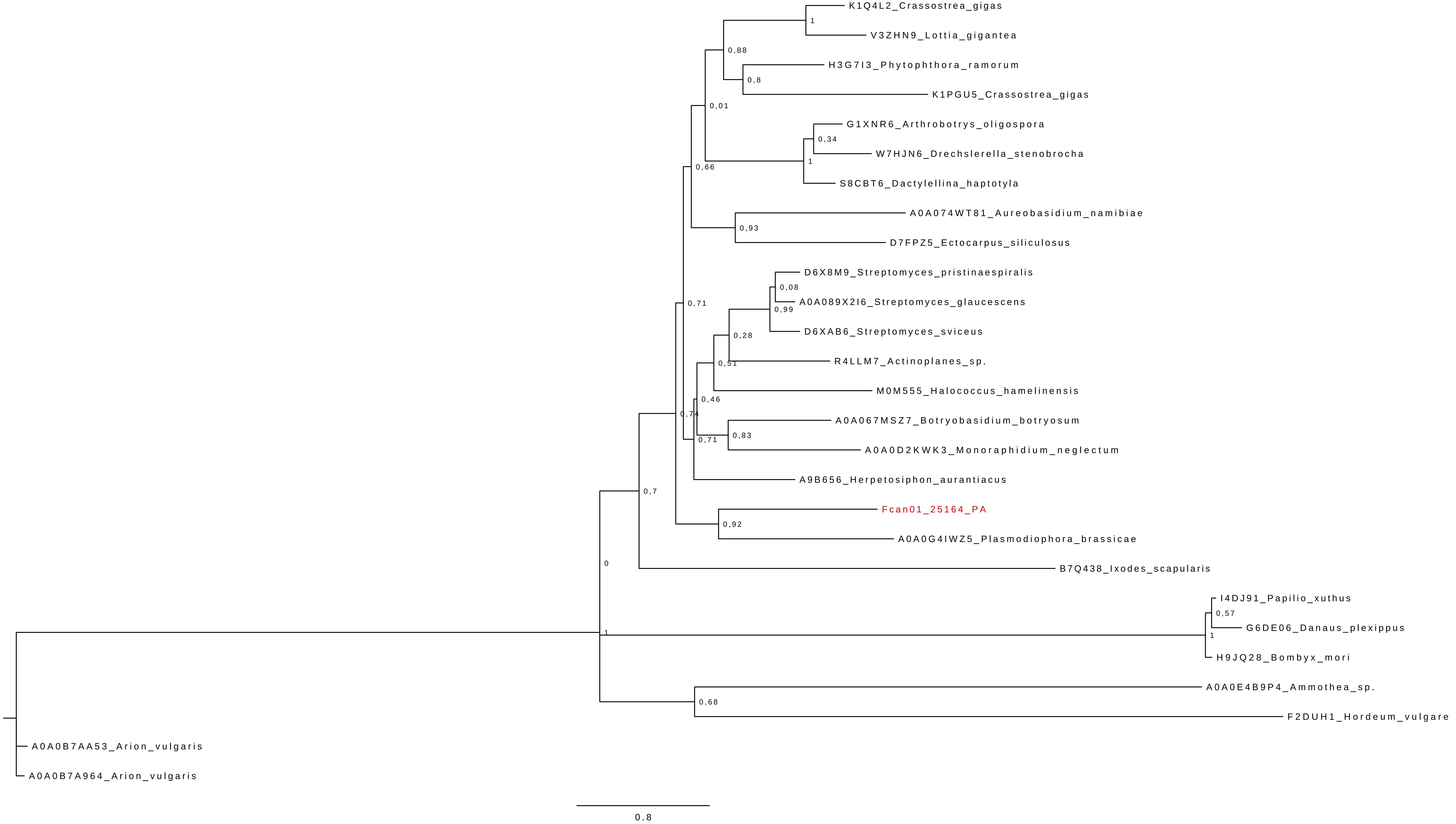
K1Q4L2_Crassostrea_gigas
V3ZHN9_Lottia_gigantea
H3G7I3_Phytophthora_ramorum
K1PGU5_Crassostrea_gigas
G1XNR6_Arthrobotrys_oligospora
W7HJN6_Drechslerella_stenobrocha
S8CBT6_Dactylellina_haptotyla
A0A074WT81_Aureobasidium_namibiae
D7FPZ5_Ectocarpus_siliculosus
D6X8M9_Streptomyces_pristinaespiralis
A0A089X2I6_Streptomyces_glaucescens
D6XAB6_Streptomyces_sviceus
R4LLM7_Actinoplanes_sp.
M0M555_Halococcus_hamelinensis
A0A067MSZ7_Botryobasidium_botryosum
A0A0D2KWK3_Monoraphidium_neglectum
A9B656_Herpetosiphon_aurantiacus
Fcan01_25164_PA
A0A0G4IWZ5_Plasmodiophora_brassicae
B7Q438_Ixodes_scapularis
I4DJ91_Papilio_xuthus
G6DE06_Danaus_plexippus
H9JQ28_Bombyx_mori
A0A0E4B9P4_Ammothea_sp.
F2DUH1_Hordeum_vulgare
A0A0B7AA53_Arion_vulgaris
A0A0B7A964_Arion_vulgaris
1
0,88
0,8
0,01
0,34
1
0,66
0,93
0,08
0,71
0,99
0,28
0,51
0,46
0,74
0,71
0,83
0,7
0,92
0
0,57
1
1
0,68
0.8

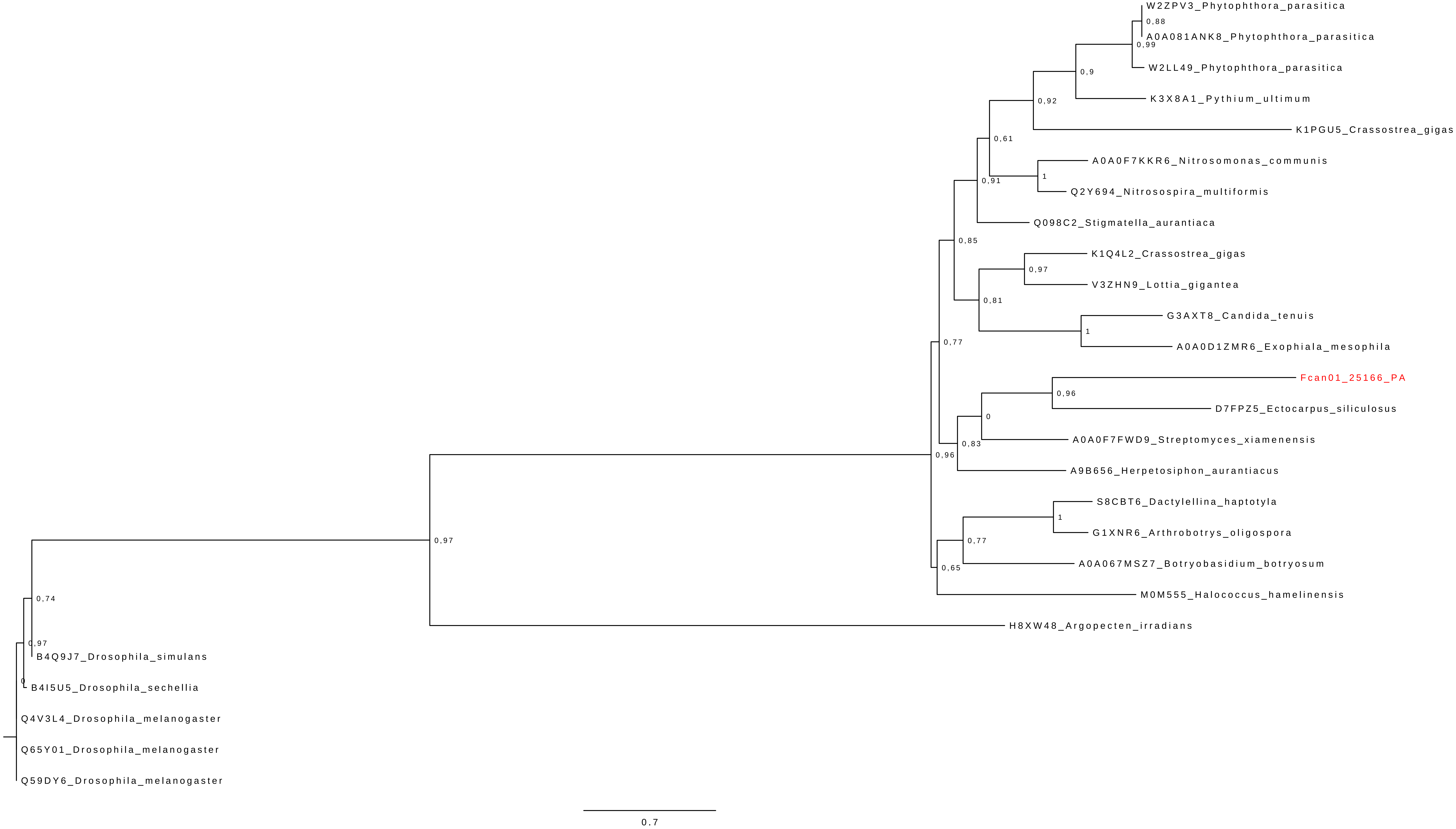
W2ZPV3_Phytophthora_parasitica
0,88
A0A081ANK8_Phytophthora_parasitica
0,99
W2LL49_Phytophthora_parasitica
0,9
K3X8A1_Pythium_ultimum
0,92
K1PGU5_Crassostrea_gigas
0,61
A0A0F7KKR6_Nitrosomonas_communis
1
0,91
Q2Y694_Nitrosospira_multiformis
Q098C2_Stigmatella_aurantiaca
0,85
K1Q4L2_Crassostrea_gigas
0,97
V3ZHN9_Lottia_gigantea
0,81
G3AXT8_Candida_tenuis
1
A0A0D1ZMR6_Exophiala_mesophila
0,77
Fcan01_25166_PA
0,96
D7FPZ5_Ectocarpus_siliculosus
0
A0A0F7FWD9_Streptomyces_xiamenensis
0,83
0,96
A9B656_Herpetosiphon_aurantiacus
S8CBT6_Dactylellina_haptotyla
1
G1XNR6_Arthrobotrys_oligospora
0,97
0,77
A0A067MSZ7_Botryobasidium_botryosum
0,65
M0M555_Halococcus_hamelinensis
0,74
H8XW48_Argopecten_irradians
0,97
B4Q9J7_Drosophila_simulans
0
B4I5U5_Drosophila_sechellia
Q4V3L4_Drosophila_melanogaster
Q65Y01_Drosophila_melanogaster
Q59DY6_Drosophila_melanogaster
0.7

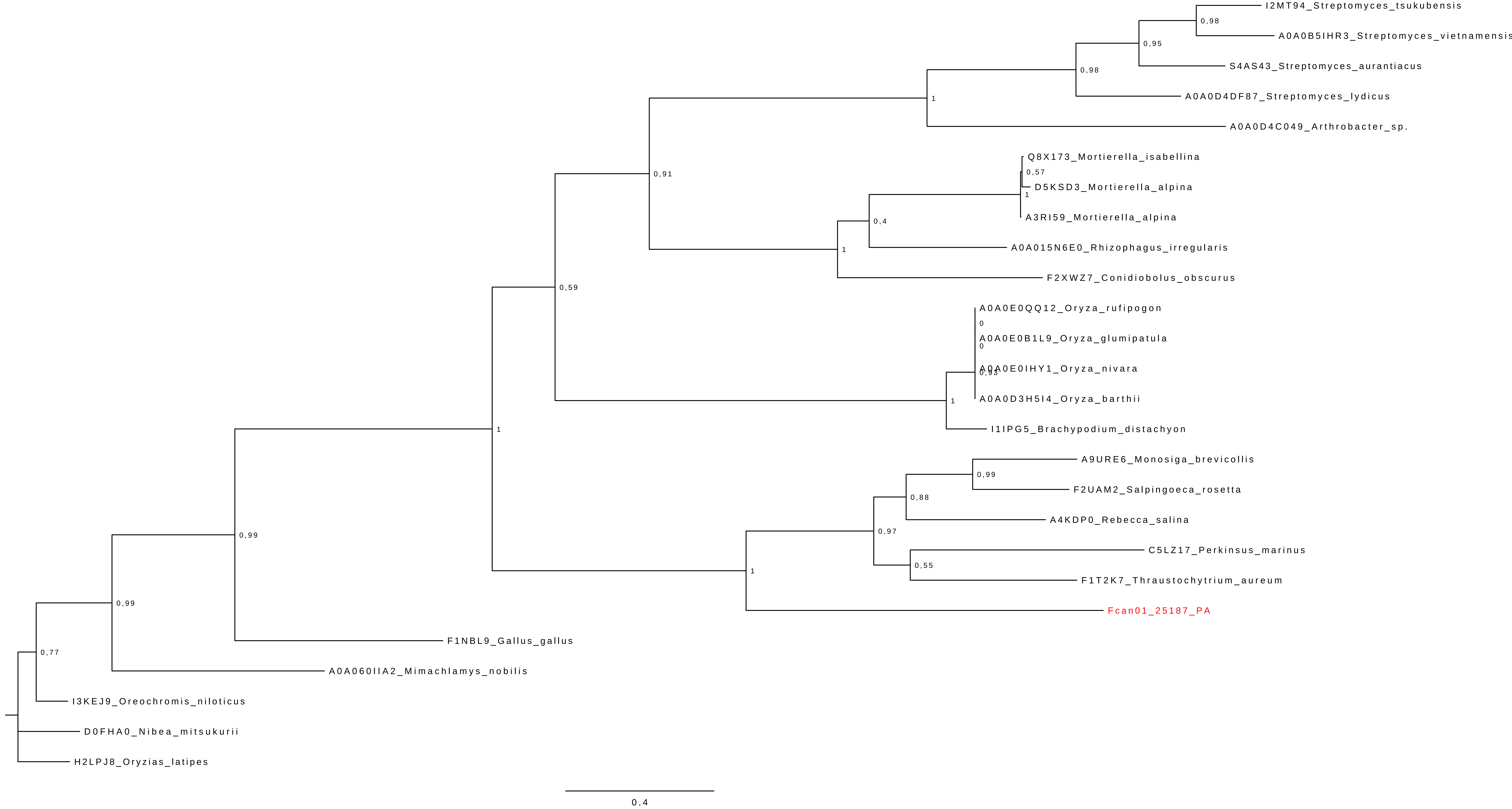
I2MT94_Streptomyces_tsukubensis
0,98
A0A0B5IHR3_Streptomyces_vietnamensis
0,95
S4AS43_Streptomyces_aurantiacus
0,98
A0A0D4DF87_Streptomyces_lydicus
1
A0A0D4C049_Arthrobacter_sp.
Q8X173_Mortierella_isabellina
0,57
D5KSD3_Mortierella_alpina
1
0,91
A3RI59_Mortierella_alpina
0,4
A0A015N6E0_Rhizophagus_irregularis
1
F2XWZ7_Conidiobolus_obscurus
0,59
A0A0E0QQ12_Oryza_rufipogon
0
A0A0E0B1L9_Oryza_glumipatula
0
A0A0E0IHY1_Oryza_nivara
A0A0D3H5I4_Oryza_barthii
1
I1IPG5_Brachypodium_distachyon
1
A9URE6_Monosiga_brevicollis
0,99
F2UAM2_Salpingoeca_rosetta
0,88
A4KDP0_Rebecca_salina
0,97
0,99
C5LZ17_Perkinsus_marinus
0,55
F1T2K7_Thraustochytrium_aureum
1
0,99
Fcan01_25187_PA
F1NBL9_Gallus_gallus
0,77
A0A060IIA2_Mimachlamys_nobilis
I3KEJ9_Oreochromis_niloticus
D0FHA0_Nibea_mitsukurii
H2LPJ8_Oryzias_latipes
0.4

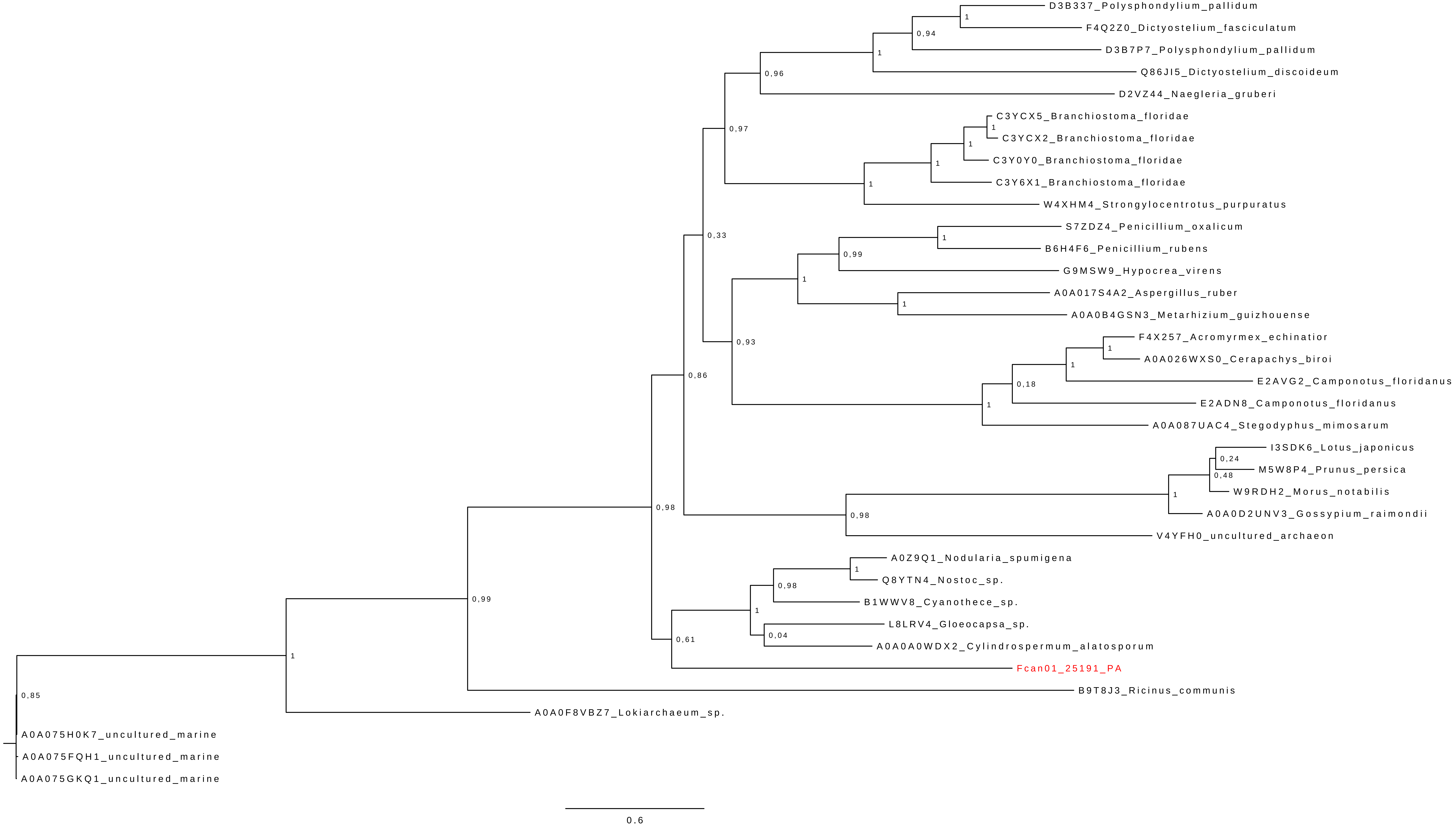
D3B337_Polysphondylium_pallidum
F4Q2Z0_Dictyostelium_fasciculatum
D3B7P7_Polysphondylium_pallidum
Q86JI5_Dictyostelium_discoideum
D2VZ44_Naegleria_gruberi
C3YCX5_Branchiostoma_floridae
C3YCX2_Branchiostoma_floridae
C3Y0Y0_Branchiostoma_floridae
C3Y6X1_Branchiostoma_floridae
W4XHM4_Strongylocentrotus_purpuratus
S7ZDZ4_Penicillium_oxalicum
B6H4F6_Penicillium_rubens
G9MSW9_Hypocrea_virens
A0A017S4A2_Aspergillus_ruber
A0A0B4GSN3_Metarhizium_guizhouense
F4X257_Acromyrmex_echinatior
A0A026WXS0_Cerapachys_biroi
E2AVG2_Camponotus_floridanus
E2ADN8_Camponotus_floridanus
A0A087UAC4_Stegodyphus_mimosarum
I3SDK6_Lotus_japonicus
M5W8P4_Prunus_persica
W9RDH2_Morus_notabilis
A0A0D2UNV3_Gossypium_raimondii
V4YFH0_uncultured_archaeon
A0Z9Q1_Nodularia_spumigena
Q8YTN4_Nostoc_sp.
B1WWV8_Cyanothece_sp.
L8LRV4_Gloeocapsa_sp.
A0A0A0WDX2_Cylindrospermum_alatosporum
Fcan01_25191_PA
B9T8J3_Ricinus_communis
A0A0F8VBZ7_Lokiarchaeum_sp.
A0A075H0K7_uncultured_marine
A0A075FQH1_uncultured_marine
A0A075GKQ1_uncultured_marine
1
0,94
1
0,96
1
1
1
0,97
1
0,33
1
0,99
1
1
0,93
1
1
0,18
1
0,86
0,24
0,48
1
0,98
0,98
1
0,98
1
0,04
0,61
0,99
1
0,85
0.6

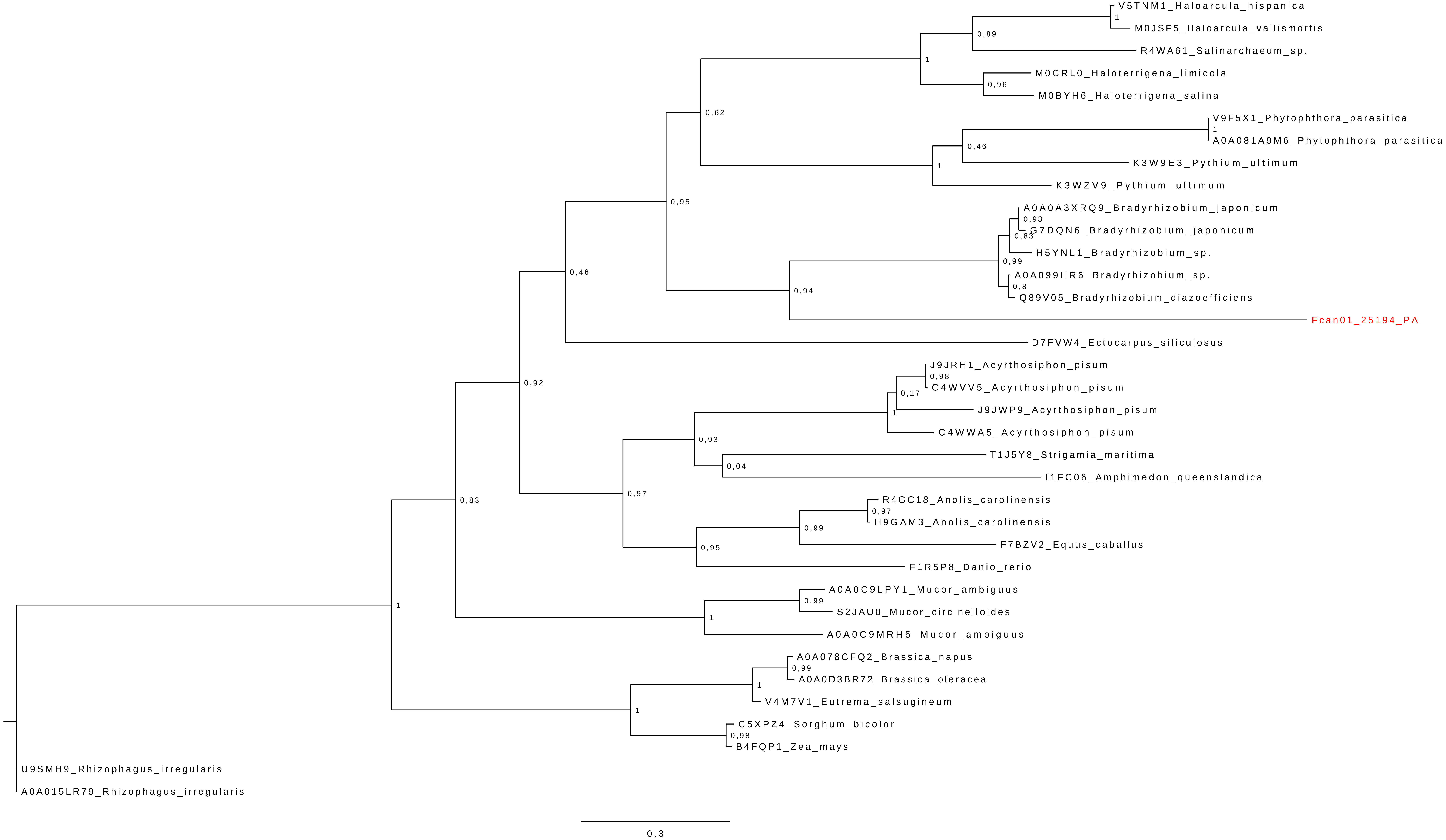
V5TNM1_Haloarcula_hispanica
M0JSF5_Haloarcula_vallismortis
R4WA61_Salinarchaeum_sp.
M0CRL0_Haloterrigena_limicola
M0BYH6_Haloterrigena_salina
V9F5X1_Phytophthora_parasitica
A0A081A9M6_Phytophthora_parasitica
K3W9E3_Pythium_ultimum
K3WZV9_Pythium_ultimum
A0A0A3XRQ9_Bradyrhizobium_japonicum
G7DQN6_Bradyrhizobium_japonicum
H5YNL1_Bradyrhizobium_sp.
A0A099IIR6_Bradyrhizobium_sp.
Q89V05_Bradyrhizobium_diazoefficiens
Fcan01_25194_PA
D7FVW4_Ectocarpus_siliculosus
J9JRH1_Acyrthosiphon_pisum
C4WVV5_Acyrthosiphon_pisum
J9JWP9_Acyrthosiphon_pisum
C4WWA5_Acyrthosiphon_pisum
T1J5Y8_Strigamia_maritima
I1FC06_Amphimedon_queenslandica
R4GC18_Anolis_carolinensis
H9GAM3_Anolis_carolinensis
F7BZV2_Equus_caballus
F1R5P8_Danio_rerio
A0A0C9LPY1_Mucor_ambiguus
S2JAU0_Mucor_circinelloides
A0A0C9MRH5_Mucor_ambiguus
A0A078CFQ2_Brassica_napus
A0A0D3BR72_Brassica_oleracea
V4M7V1_Eutrema_salsugineum
C5XPZ4_Sorghum_bicolor
B4FQP1_Zea_mays
U9SMH9_Rhizophagus_irregularis
A0A015LR79_Rhizophagus_irregularis
0.3

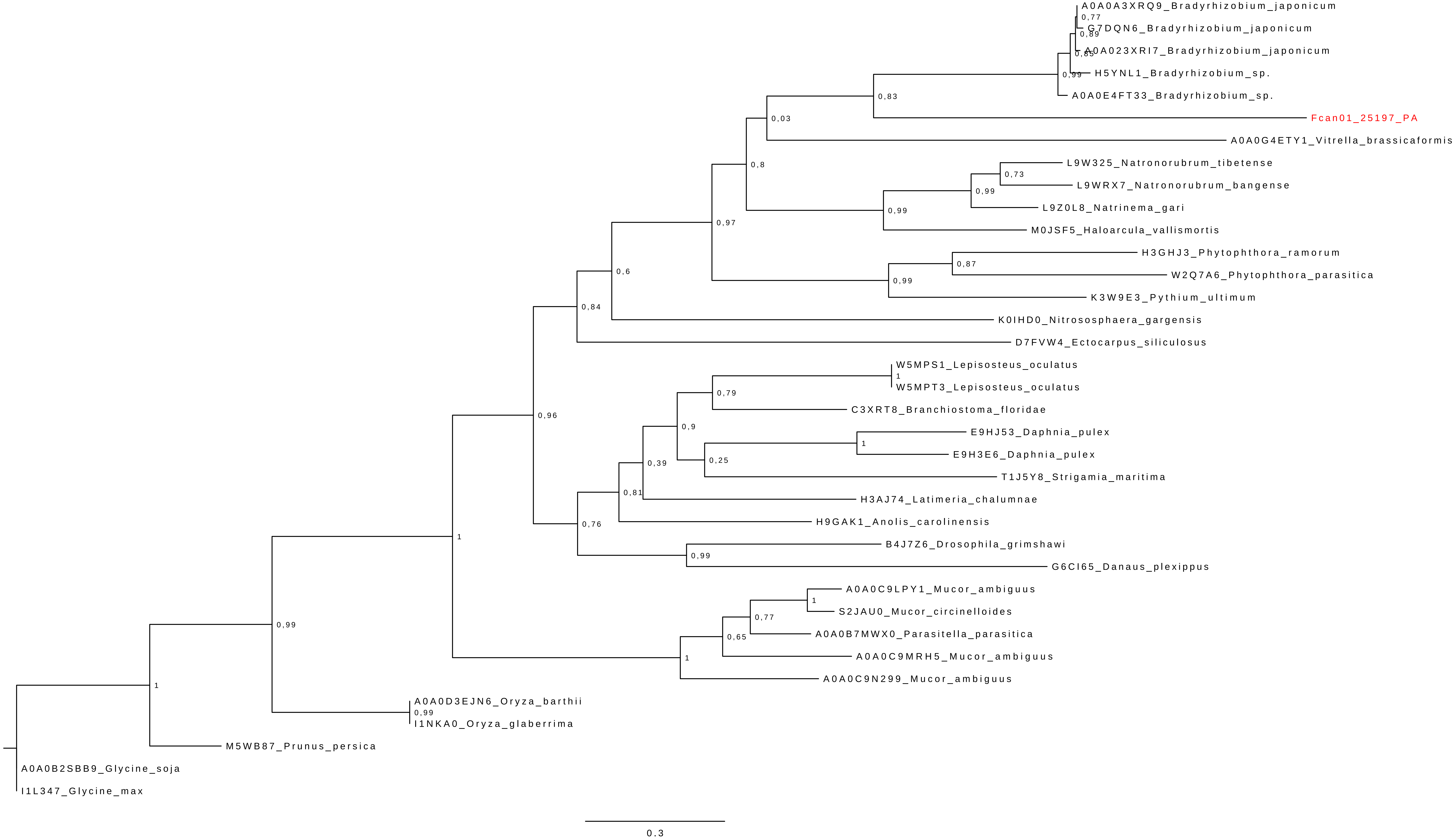
A0A0A3XRQ9_Bradyrhizobium_japonicum
G7DQN6_Bradyrhizobium_japonicum
A0A023XRI7_Bradyrhizobium_japonicum
H5YNL1_Bradyrhizobium_sp.
A0A0E4FT33_Bradyrhizobium_sp.
Fcan01_25197_PA
A0A0G4ETY1_Vitrella_brassicaformis
L9W325_Natronorubrum_tibetense
L9WRX7_Natronorubrum_bangense
L9Z0L8_Natrinema_gari
M0JSF5_Haloarcula_vallismortis
H3GHJ3_Phytophthora_ramorum
W2Q7A6_Phytophthora_parasitica
K3W9E3_Pythium_ultimum
K0IHD0_Nitrososphaera_gargensis
D7FVW4_Ectocarpus_siliculosus
W5MPS1_Lepisosteus_oculatus
W5MPT3_Lepisosteus_oculatus
C3XRT8_Branchiostoma_floridae
E9HJ53_Daphnia_pulex
E9H3E6_Daphnia_pulex
T1J5Y8_Strigamia_maritima
H3AJ74_Latimeria_chalumnae
H9GAK1_Anolis_carolinensis
B4J7Z6_Drosophila_grimshawi
G6CI65_Danaus_plexippus
A0A0C9LPY1_Mucor_ambiguus
S2JAU0_Mucor_circinelloides
A0A0B7MWX0_Parasitella_parasitica
A0A0C9MRH5_Mucor_ambiguus
A0A0C9N299_Mucor_ambiguus
A0A0D3EJN6_Oryza_barthii
I1NKA0_Oryza_glaberrima
M5WB87_Prunus_persica
A0A0B2SBB9_Glycine_soja
I1L347_Glycine_max
0.3

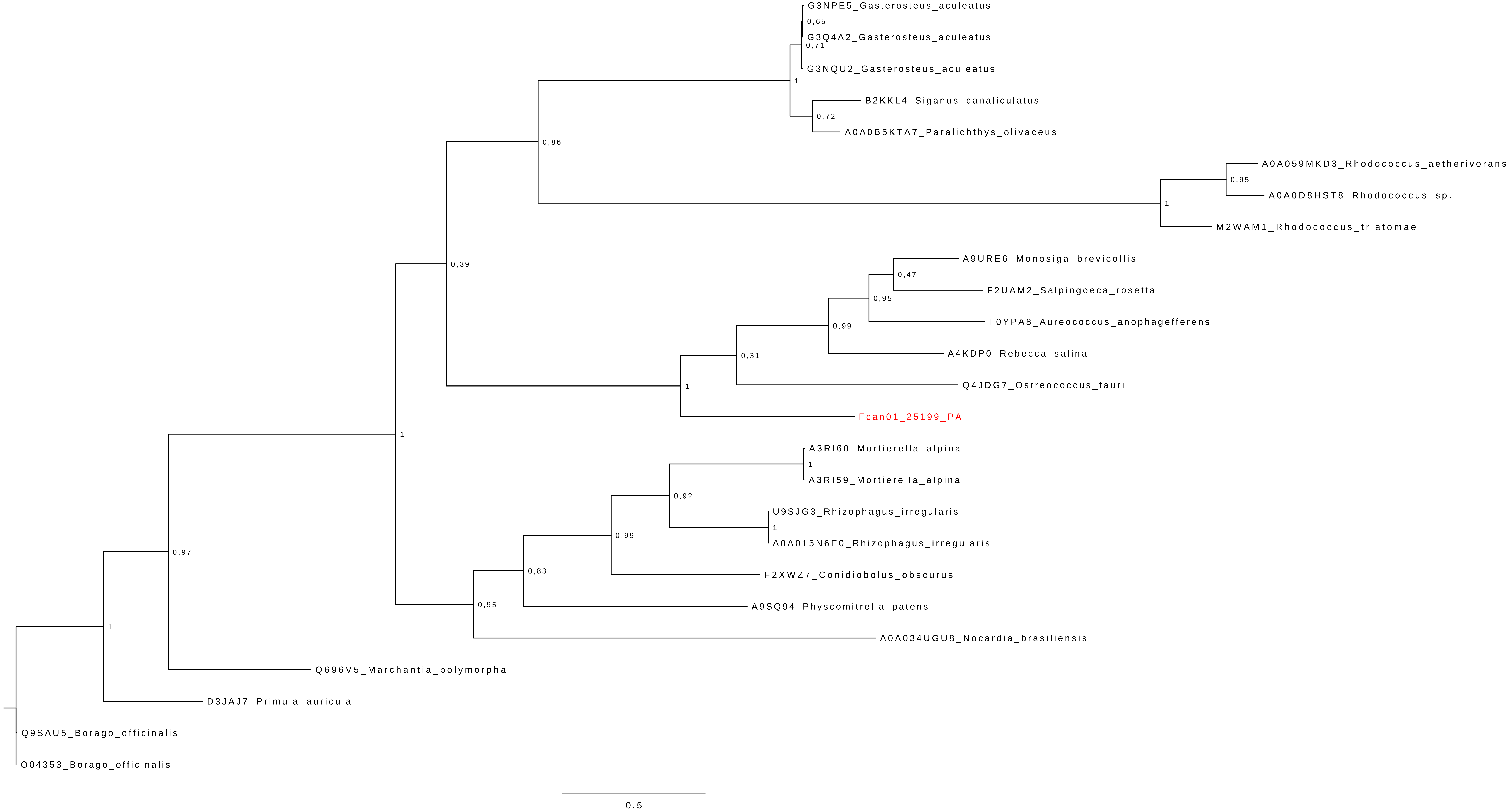
G3NPE5_Gasterosteus_aculeatus
0,65
G3Q4A2_Gasterosteus_aculeatus
0,71
G3NQU2_Gasterosteus_aculeatus
1
B2KKL4_Siganus_canaliculatus
0,72
A0A0B5KTA7_Paralichthys_olivaceus
0,86
A0A059MKD3_Rhodococcus_aetherivorans
0,95
A0A0D8HST8_Rhodococcus_sp.
1
M2WAM1_Rhodococcus_triatomae
A9URE6_Monosiga_brevicollis
0,47
F2UAM2_Salpingoeca_rosetta
0,95
F0YPA8_Aureococcus_anophagefferens
0,99
A4KDP0_Rebecca_salina
0,31
Q4JDG7_Ostreococcus_tauri
0,39
1
Fcan01_25199_PA
1
A3RI60_Mortierella_alpina
1
A3RI59_Mortierella_alpina
0,92
U9SJG3_Rhizophagus_irregularis
1
A0A015N6E0_Rhizophagus_irregularis
0,99
F2XWZ7_Conidiobolus_obscurus
0,83
A9SQ94_Physcomitrella_patens
0,95
A0A034UGU8_Nocardia_brasiliensis
0,97
Q696V5_Marchantia_polymorpha
1
D3JAJ7_Primula_auricula
Q9SAU5_Borago_officinalis
O04353_Borago_officinalis
0.5

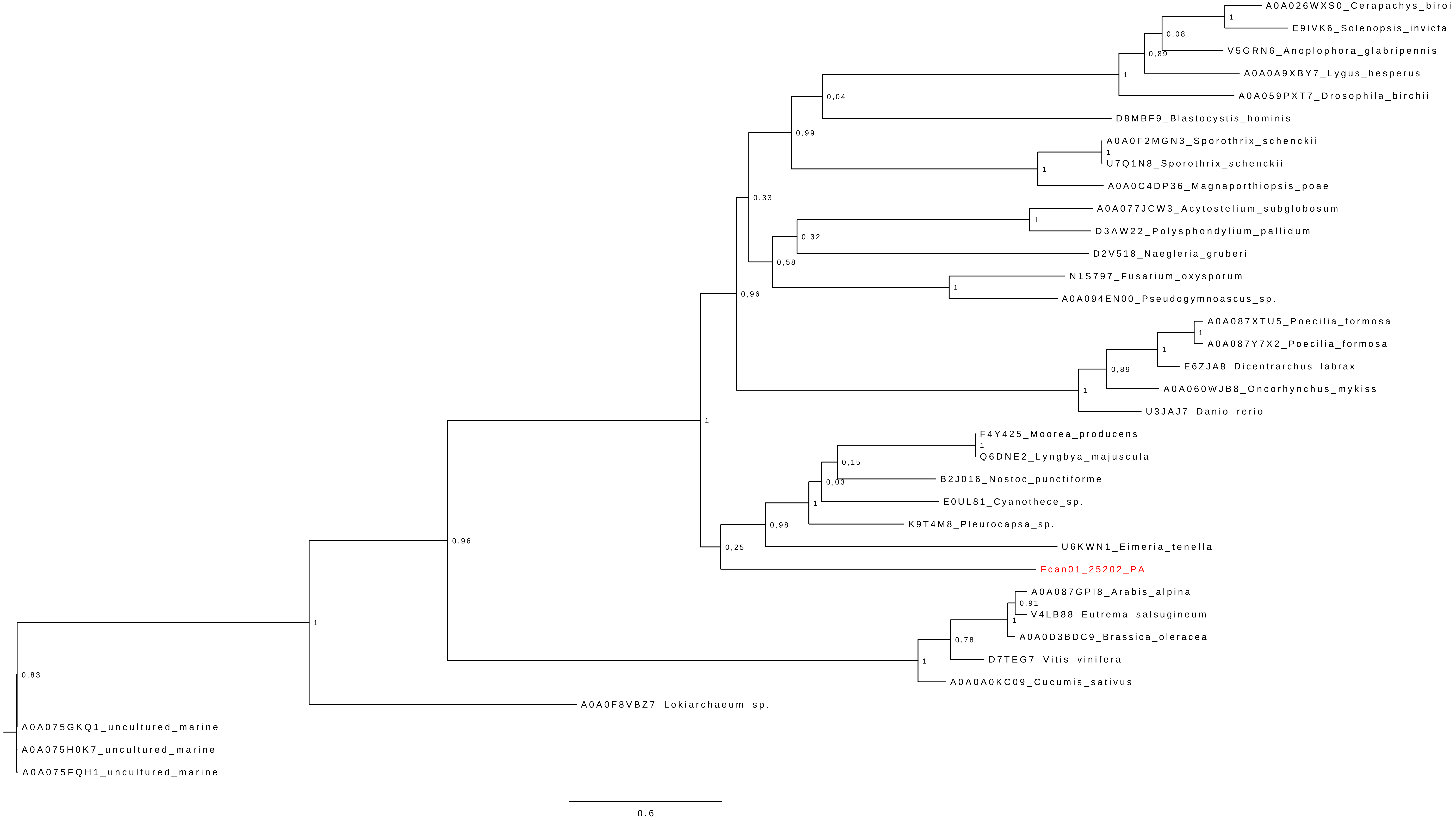
A0A026WXS0_Cerapachys_biroi
E9IVK6_Solenopsis_invicta
V5GRN6_Anoplophora_glabripennis
A0A0A9XBY7_Lygus_hesperus
A0A059PXT7_Drosophila_birchii
D8MBF9_Blastocystis_hominis
A0A0F2MGN3_Sporothrix_schenckii
U7Q1N8_Sporothrix_schenckii
A0A0C4DP36_Magnaporthiopsis_poae
A0A077JCW3_Acytostelium_subglobosum
D3AW22_Polysphondylium_pallidum
D2V518_Naegleria_gruberi
N1S797_Fusarium_oxysporum
A0A094EN00_Pseudogymnoascus_sp.
A0A087XTU5_Poecilia_formosa
A0A087Y7X2_Poecilia_formosa
E6ZJA8_Dicentrarchus_labrax
A0A060WJB8_Oncorhynchus_mykiss
U3JAJ7_Danio_rerio
F4Y425_Moorea_producens
Q6DNE2_Lyngbya_majuscula
B2J016_Nostoc_punctiforme
E0UL81_Cyanothece_sp.
K9T4M8_Pleurocapsa_sp.
U6KWN1_Eimeria_tenella
Fcan01_25202_PA
A0A087GPI8_Arabis_alpina
V4LB88_Eutrema_salsugineum
A0A0D3BDC9_Brassica_oleracea
D7TEG7_Vitis_vinifera
A0A0A0KC09_Cucumis_sativus
A0A0F8VBZ7_Lokiarchaeum_sp.
A0A075GKQ1_uncultured_marine
A0A075H0K7_uncultured_marine
A0A075FQH1_uncultured_marine
1
0,08
0,89
1
0,04
0,99
1
1
0,33
1
0,32
0,58
1
0,96
1
1
0,89
1
1
1
0,15
0,03
1
0,98
0,25
0,96
0,91
1
0,78
1
1
0,83
0.6

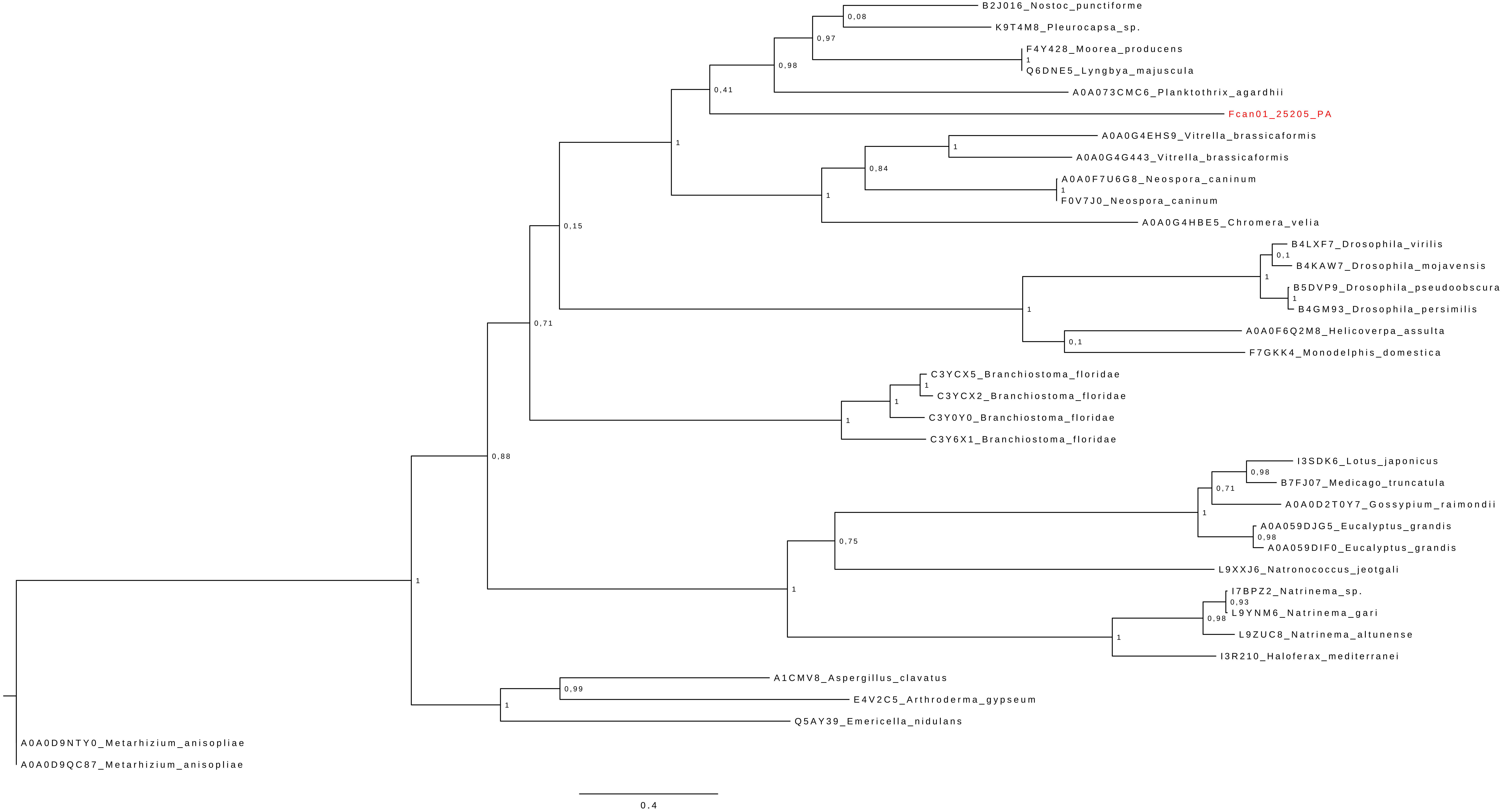

B2J016_Nostoc_punctiforme
0,08
K9T4M8_Pleurocapsa_sp.
0,97
F4Y428_Moorea_producens
1
Q6DNE5_Lyngbya_majuscula
0,98
0,41
A0A073CMC6_Planktothrix_agardhii
Fcan01_25205_PA
1
A0A0G4EHS9_Vitrella_brassicaformis
1
A0A0G4G443_Vitrella_brassicaformis
0,84
A0A0F7U6G8_Neospora_caninum
1
F0V7J0_Neospora_caninum
1
A0A0G4HBE5_Chromera_velia
0,15
B4LXF7_Drosophila_virilis
0,1
B4KAW7_Drosophila_mojavensis
1
B5DVP9_Drosophila_pseudoobscura
1
B4GM93_Drosophila_persimilis
1
A0A0F6Q2M8_Helicoverpa_assulta
0,1
F7GKK4_Monodelphis_domestica
0,71
C3YCX5_Branchiostoma_floridae
1
C3YCX2_Branchiostoma_floridae
1
C3Y0Y0_Branchiostoma_floridae
1
C3Y6X1_Branchiostoma_floridae
0,88
I3SDK6_Lotus_japonicus
0,98
B7FJ07_Medicago_truncatula
0,71
A0A0D2T0Y7_Gossypium_raimondii
1
A0A059DJG5_Eucalyptus_grandis
0,98
A0A059DIF0_Eucalyptus_grandis
0,75
L9XXJ6_Natronococcus_jeotgali
1
1
I7BPZ2_Natrinema_sp.
0,93
L9YNM6_Natrinema_gari
0,98
L9ZUC8_Natrinema_altunense
1
I3R210_Haloferax_mediterranei
A1CMV8_Aspergillus_clavatus
0,99
E4V2C5_Arthroderma_gypseum
1
Q5AY39_Emericella_nidulans
A0A0D9NTY0_Metarhizium_anisopliae
A0A0D9QC87_Metarhizium_anisopliae
0.4

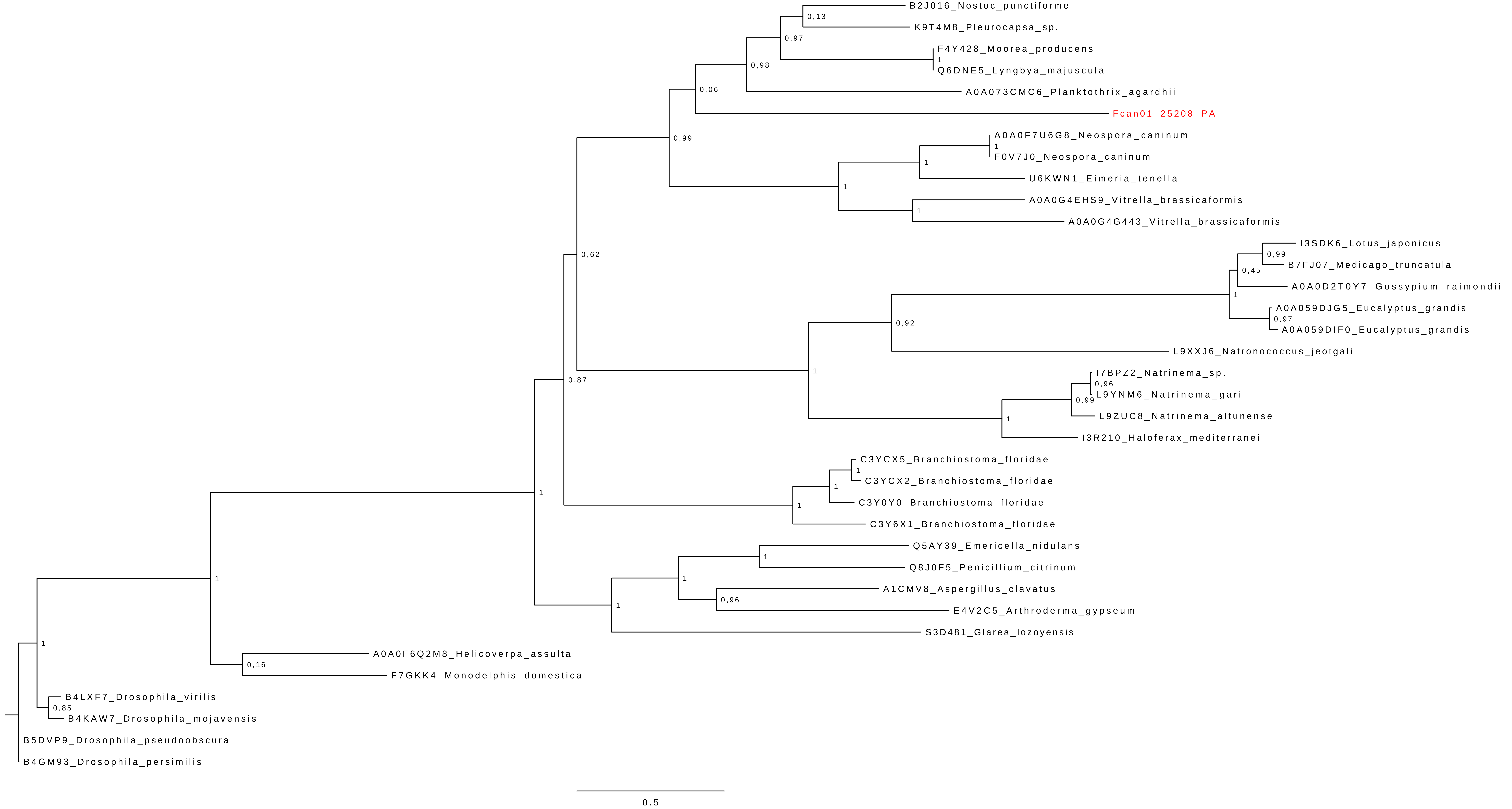
B2J016_Nostoc_punctiforme
K9T4M8_Pleurocapsa_sp.
F4Y428_Moorea_producens
Q6DNE5_Lyngbya_majuscula
A0A073CMC6_Planktothrix_agardhii
Fcan01_25208_PA
A0A0F7U6G8_Neospora_caninum
F0V7J0_Neospora_caninum
U6KWN1_Eimeria_tenella
A0A0G4EHS9_Vitrella_brassicaformis
A0A0G4G443_Vitrella_brassicaformis
I3SDK6_Lotus_japonicus
B7FJ07_Medicago_truncatula
A0A0D2T0Y7_Gossypium_raimondii
A0A059DJG5_Eucalyptus_grandis
A0A059DIF0_Eucalyptus_grandis
L9XXJ6_Natronococcus_jeotgali
I7BPZ2_Natrinema_sp.
L9YNM6_Natrinema_gari
L9ZUC8_Natrinema_altunense
I3R210_Haloferax_mediterranei
C3YCX5_Branchiostoma_floridae
C3YCX2_Branchiostoma_floridae
C3Y0Y0_Branchiostoma_floridae
C3Y6X1_Branchiostoma_floridae
Q5AY39_Emericella_nidulans
Q8J0F5_Penicillium_citrinum
A1CMV8_Aspergillus_clavatus
E4V2C5_Arthroderma_gypseum
S3D481_Glarea_lozoyensis
A0A0F6Q2M8_Helicoverpa_assulta
F7GKK4_Monodelphis_domestica
B4LXF7_Drosophila_virilis
B4KAW7_Drosophila_mojavensis
B5DVP9_Drosophila_pseudoobscura
B4GM93_Drosophila_persimilis
0.5

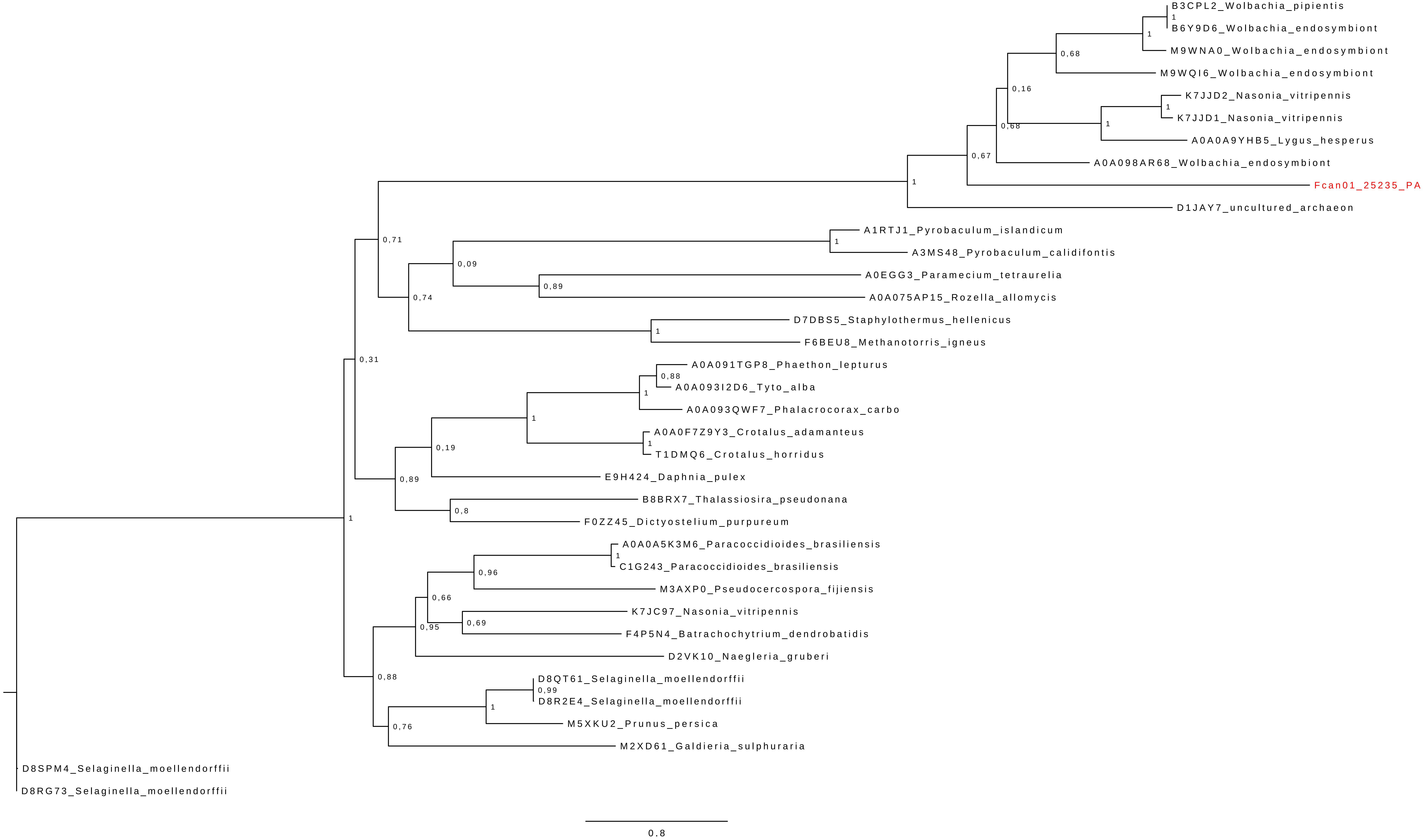
B3CPL2_Wolbachia_pipientis
B6Y9D6_Wolbachia_endosymbiont
M9WNA0_Wolbachia_endosymbiont
M9WQI6_Wolbachia_endosymbiont
K7JJD2_Nasonia_vitripennis
K7JJD1_Nasonia_vitripennis
A0A0A9YHB5_Lygus_hesperus
A0A098AR68_Wolbachia_endosymbiont
Fcan01_25235_PA
D1JAY7_uncultured_archaeon
A1RTJ1_Pyrobaculum_islandicum
A3MS48_Pyrobaculum_calidifontis
A0EGG3_Paramecium_tetraurelia
A0A075AP15_Rozella_allomycis
D7DBS5_Staphylothermus_hellenicus
F6BEU8_Methanotorris_igneus
A0A091TGP8_Phaethon_lepturus
A0A093I2D6_Tyto_alba
A0A093QWF7_Phalacrocorax_carbo
A0A0F7Z9Y3_Crotalus_adamanteus
T1DMQ6_Crotalus_horridus
E9H424_Daphnia_pulex
B8BRX7_Thalassiosira_pseudonana
F0ZZ45_Dictyostelium_purpureum
A0A0A5K3M6_Paracoccidioides_brasiliensis
C1G243_Paracoccidioides_brasiliensis
M3AXP0_Pseudocercospora_fijiensis
K7JC97_Nasonia_vitripennis
F4P5N4_Batrachochytrium_dendrobatidis
D2VK10_Naegleria_gruberi
D8QT61_Selaginella_moellendorffii
D8R2E4_Selaginella_moellendorffii
M5XKU2_Prunus_persica
M2XD61_Galdieria_sulphuraria
D8SPM4_Selaginella_moellendorffii
D8RG73_Selaginella_moellendorffii
1
1
0,68
0,16
1
1
0,68
0,67
1
0,71
1
0,09
0,89
0,74
1
0,31
0,88
1
1
1
0,19
0,89
0,8
1
1
0,96
0,66
0,69
0,95
0,88
0,99
1
0,76
0.8

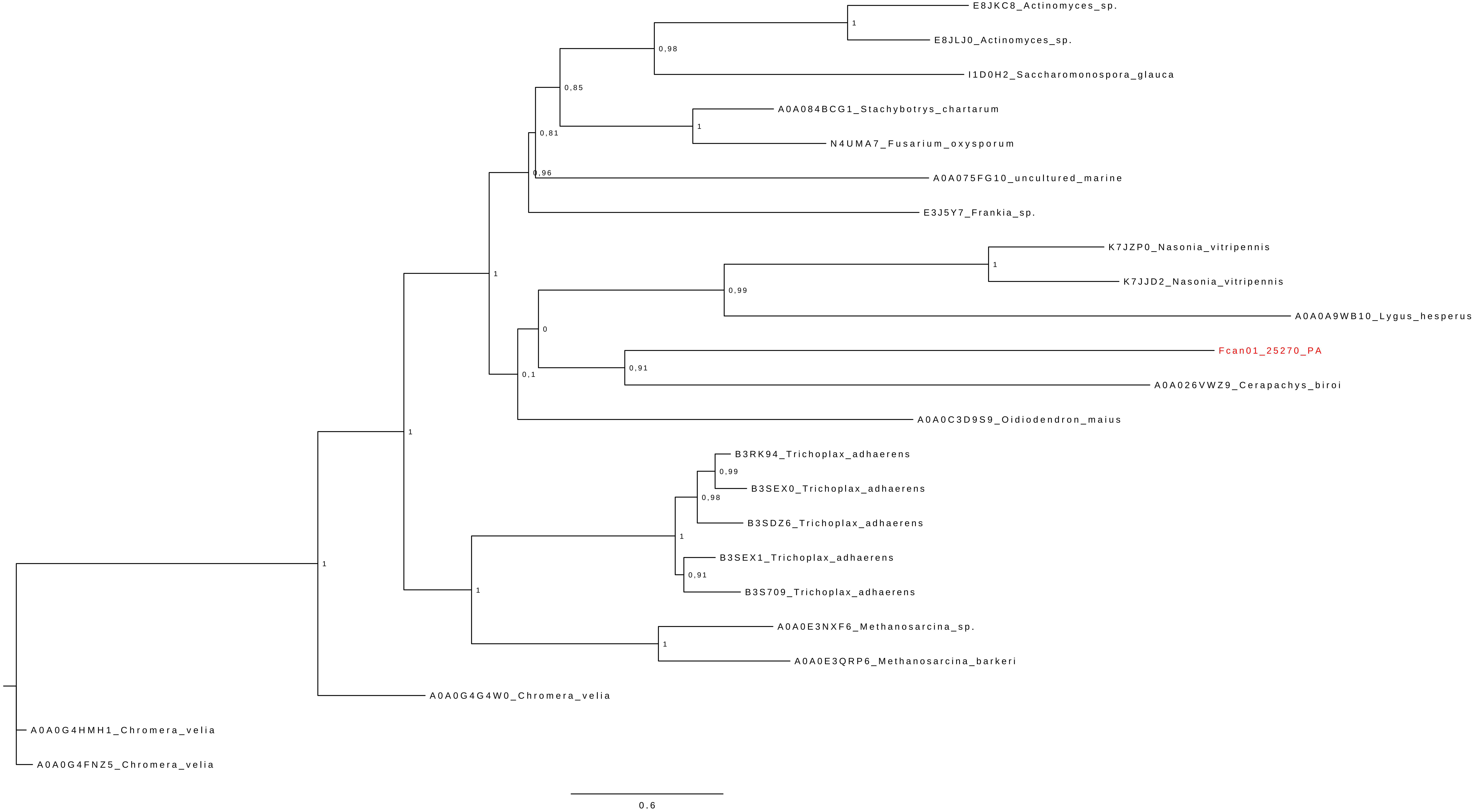

E8JKC8_Actinomyces_sp.
1
E8JLJ0_Actinomyces_sp.
0,98
I1D0H2_Saccharomonospora_glauca
0,85
A0A084BCG1_Stachybotrys_chartarum
1
0,81
N4UMA7_Fusarium_oxysporum
0,96
A0A075FG10_uncultured_marine
E3J5Y7_Frankia_sp.
K7JZP0_Nasonia_vitripennis
1
K7JJD2_Nasonia_vitripennis
0,99
A0A0A9WB10_Lygus_hesperus
1
0
Fcan01_25270_PA
0,91
A0A026VWZ9_Cerapachys_biroi
0,1
A0A0C3D9S9_Oidiodendron_maius
1
B3RK94_Trichoplax_adhaerens
0,99
B3SEX0_Trichoplax_adhaerens
0,98
B3SDZ6_Trichoplax_adhaerens
1
B3SEX1_Trichoplax_adhaerens
1
0,91
B3S709_Trichoplax_adhaerens
1
A0A0E3NXF6_Methanosarcina_sp.
1
A0A0E3QRP6_Methanosarcina_barkeri
A0A0G4G4W0_Chromera_velia
A0A0G4HMH1_Chromera_velia
A0A0G4FNZ5_Chromera_velia
0.6

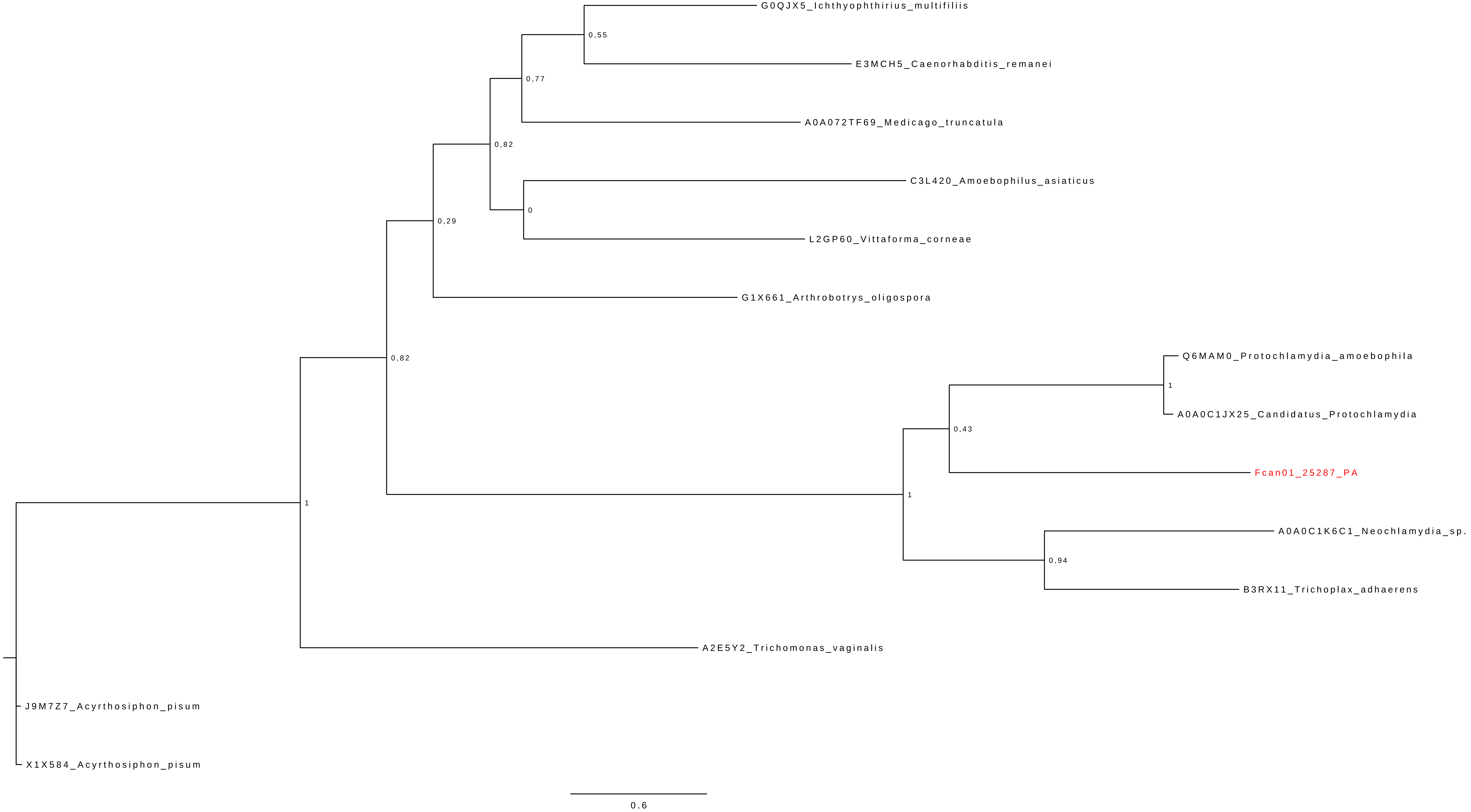

G0QJX5_Ichthyophthirius_multifiliis
0,55
E3MCH5_Caenorhabditis_remanei
0,77
A0A072TF69_Medicago_truncatula
0,82
C3L420_Amoebophilus_asiaticus
0
L2GP60_Vittaforma_corneae
0,29
G1X661_Arthrobotrys_oligospora
0,82
Q6MAM0_Protochlamydia_amoebophila
1
A0A0C1JX25_Candidatus_Protochlamydia
0,43
Fcan01_25287_PA
1
1
A0A0C1K6C1_Neochlamydia_sp.
0,94
B3RX11_Trichoplax_adhaerens
A2E5Y2_Trichomonas_vaginalis
J9M7Z7_Acyrthosiphon_pisum
X1X584_Acyrthosiphon_pisum
0.6

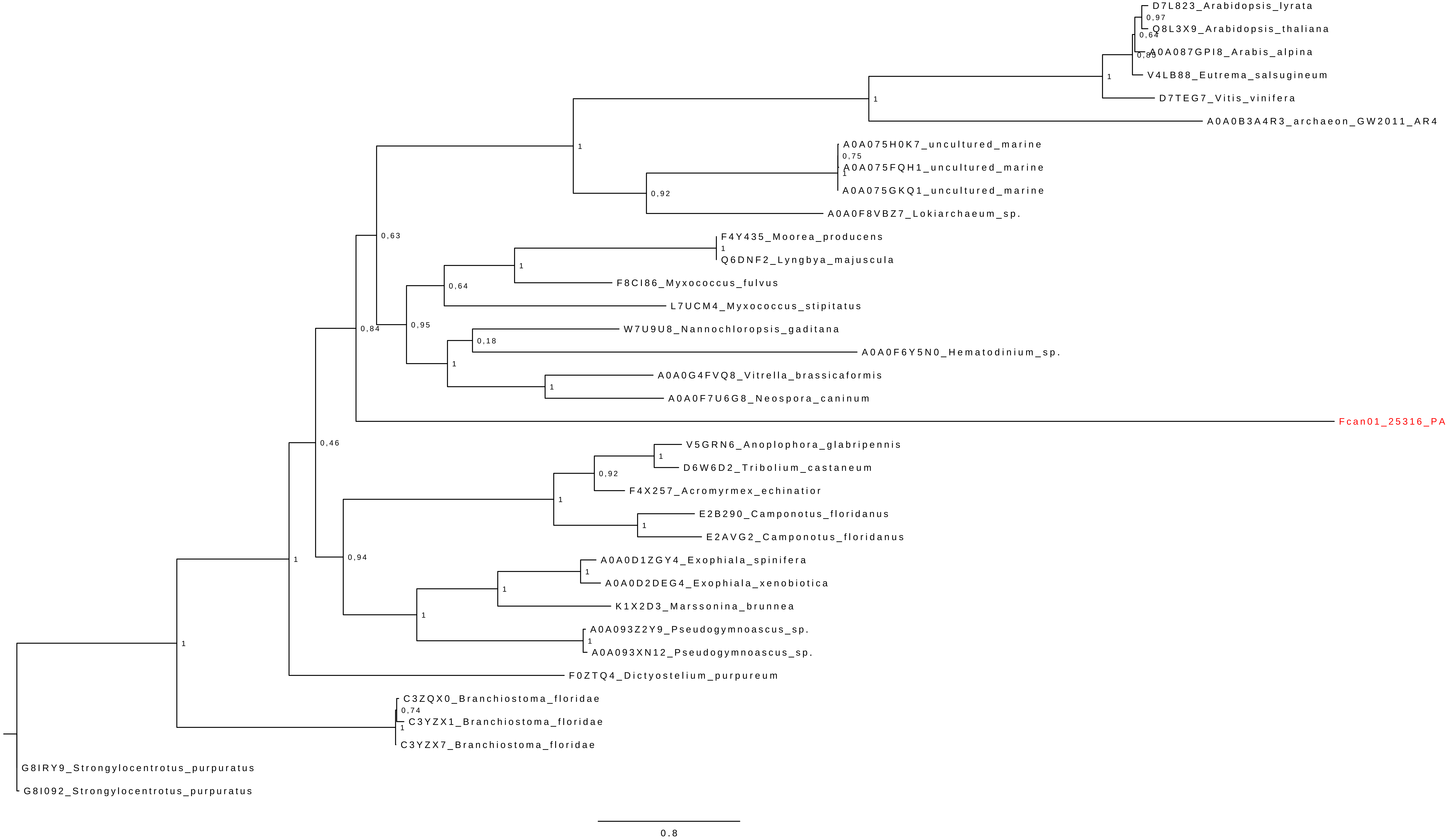
D7L823_Arabidopsis_lyrata
0,97
Q8L3X9_Arabidopsis_thaliana
0,64
A0A087GPI8_Arabis_alpina
0,89
V4LB88_Eutrema_salsugineum
1
D7TEG7_Vitis_vinifera
1
A0A0B3A4R3_archaeon_GW2011_AR4
1
A0A075H0K7_uncultured_marine
0,75
A0A075FQH1_uncultured_marine
1
A0A075GKQ1_uncultured_marine
0,92
A0A0F8VBZ7_Lokiarchaeum_sp.
0,63
F4Y435_Moorea_producens
1
Q6DNF2_Lyngbya_majuscula
1
F8CI86_Myxococcus_fulvus
0,64
L7UCM4_Myxococcus_stipitatus
0,84
0,95
W7U9U8_Nannochloropsis_gaditana
0,18
A0A0F6Y5N0_Hematodinium_sp.
1
A0A0G4FVQ8_Vitrella_brassicaformis
1
A0A0F7U6G8_Neospora_caninum
Fcan01_25316_PA
0,46
V5GRN6_Anoplophora_glabripennis
1
D6W6D2_Tribolium_castaneum
0,92
F4X257_Acromyrmex_echinatior
1
E2B290_Camponotus_floridanus
1
E2AVG2_Camponotus_floridanus
1
0,94
A0A0D1ZGY4_Exophiala_spinifera
1
A0A0D2DEG4_Exophiala_xenobiotica
1
K1X2D3_Marssonina_brunnea
1
A0A093Z2Y9_Pseudogymnoascus_sp.
1
A0A093XN12_Pseudogymnoascus_sp.
F0ZTQ4_Dictyostelium_purpureum
1
C3ZQX0_Branchiostoma_floridae
0,74
C3YZX1_Branchiostoma_floridae
1
C3YZX7_Branchiostoma_floridae
G8IRY9_Strongylocentrotus_purpuratus
G8I092_Strongylocentrotus_purpuratus
0.8

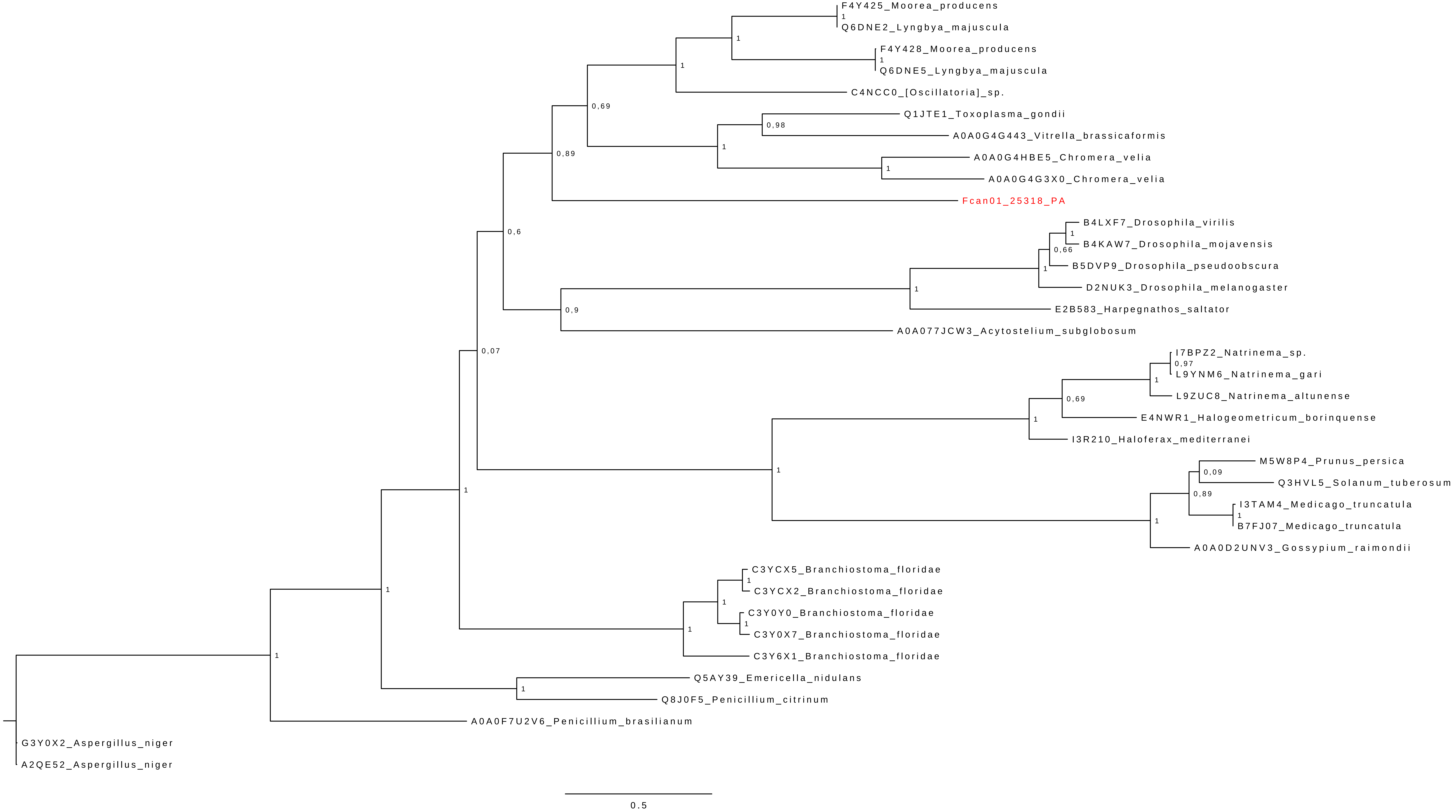
F4Y425_Moorea_producens
Q6DNE2_Lyngbya_majuscula
F4Y428_Moorea_producens
Q6DNE5_Lyngbya_majuscula
C4NCC0_[Oscillatoria]_sp.
Q1JTE1_Toxoplasma_gondii
A0A0G4G443_Vitrella_brassicaformis
A0A0G4HBE5_Chromera_velia
A0A0G4G3X0_Chromera_velia
Fcan01_25318_PA
B4LXF7_Drosophila_virilis
B4KAW7_Drosophila_mojavensis
B5DVP9_Drosophila_pseudoobscura
D2NUK3_Drosophila_melanogaster
E2B583_Harpegnathos_saltator
A0A077JCW3_Acytostelium_subglobosum
I7BPZ2_Natrinema_sp.
L9YNM6_Natrinema_gari
L9ZUC8_Natrinema_altunense
E4NWR1_Halogeometricum_borinquense
I3R210_Haloferax_mediterranei
M5W8P4_Prunus_persica
Q3HVL5_Solanum_tuberosum
I3TAM4_Medicago_truncatula
B7FJ07_Medicago_truncatula
A0A0D2UNV3_Gossypium_raimondii
C3YCX5_Branchiostoma_floridae
C3YCX2_Branchiostoma_floridae
C3Y0Y0_Branchiostoma_floridae
C3Y0X7_Branchiostoma_floridae
C3Y6X1_Branchiostoma_floridae
Q5AY39_Emericella_nidulans
Q8J0F5_Penicillium_citrinum
A0A0F7U2V6_Penicillium_brasilianum
G3Y0X2_Aspergillus_niger
A2QE52_Aspergillus_niger
0.5

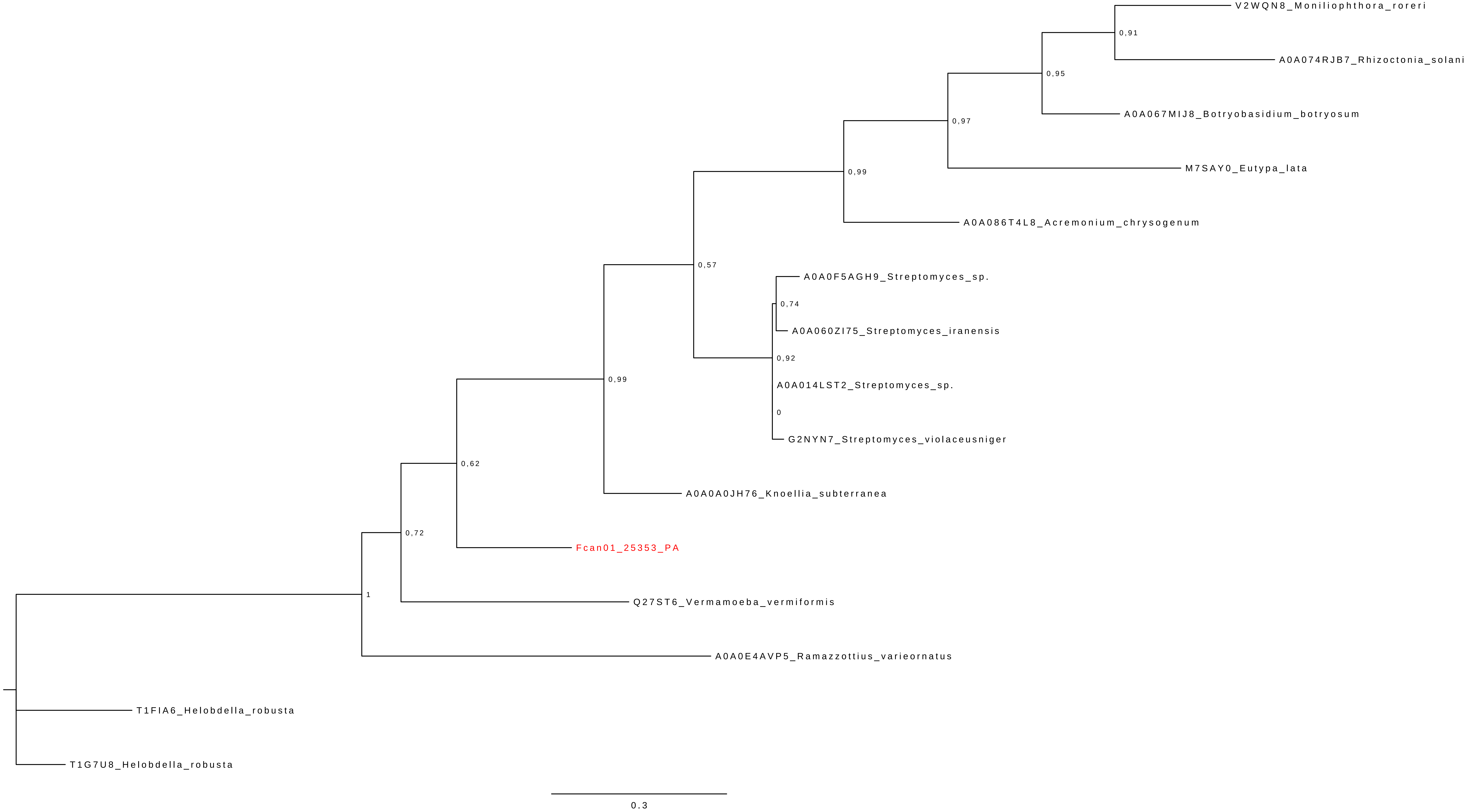
V2WQN8_Moniliophthora_roreri
0,91
A0A074RJB7_Rhizoctonia_solani
0,95
A0A067MIJ8_Botryobasidium_botryosum
0,97
M7SAY0_Eutypa_lata
0,99
A0A086T4L8_Acremonium_chrysogenum
0,57
A0A0F5AGH9_Streptomyces_sp.
0,74
A0A060ZI75_Streptomyces_iranensis
0,92
A0A014LST2_Streptomyces_sp.
0
G2NYN7_Streptomyces_violaceusniger
0,99
A0A0A0JH76_Knoellia_subterranea
0,62
Fcan01_25353_PA
0,72
Q27ST6_Vermamoeba_vermiformis
1
A0A0E4AVP5_Ramazzottius_varieornatus
T1FIA6_Helobdella_robusta
T1G7U8_Helobdella_robusta
0.3

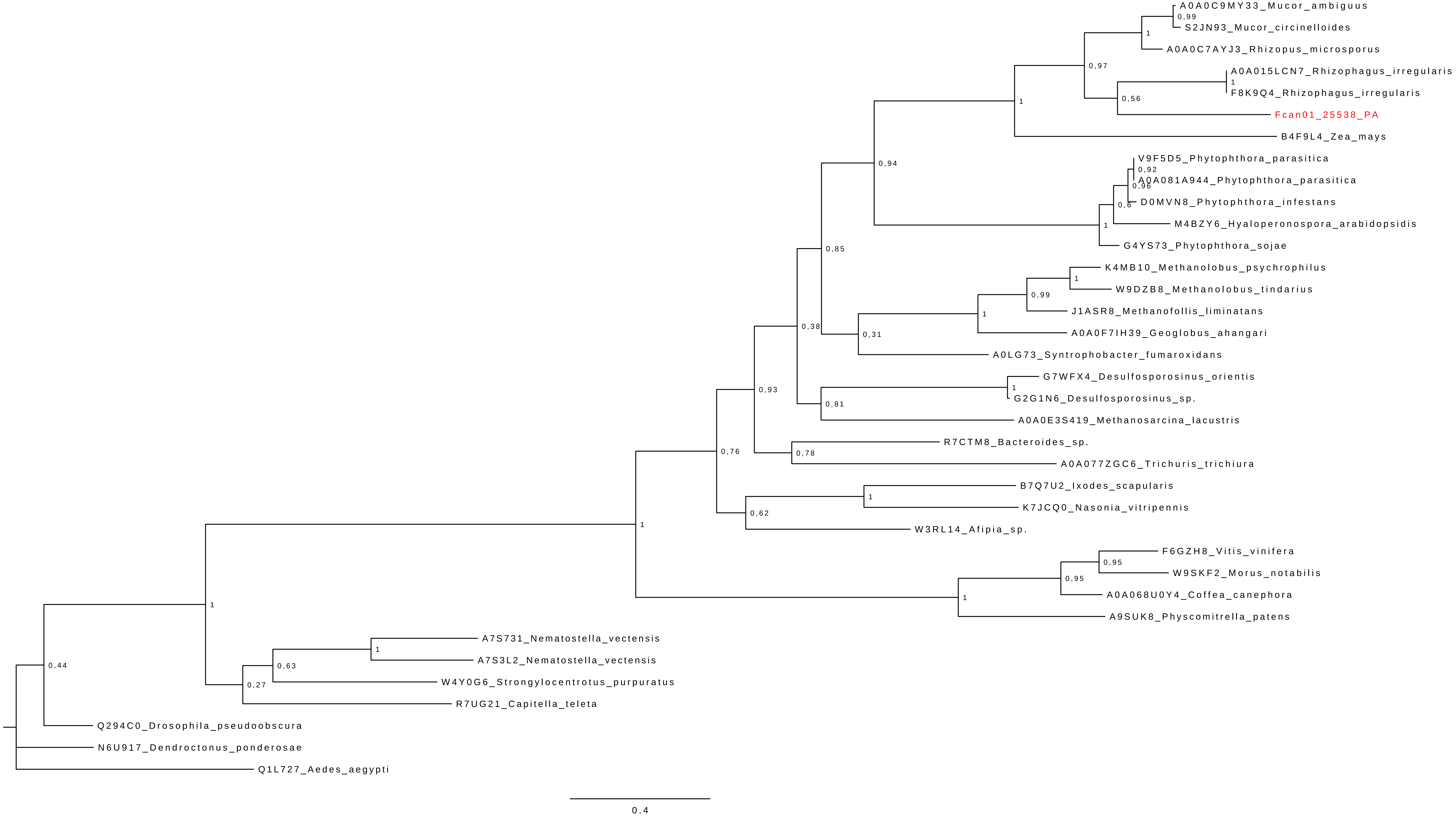
A0A0C9MY33_Mucor_ambiguus
0,99
S2JN93_Mucor_circinelloides
1
A0A0C7AYJ3_Rhizopus_microsporus
0,97
A0A015LCN7_Rhizophagus_irregularis
1
F8K9Q4_Rhizophagus_irregularis
0,56
1
Fcan01_25538_PA
B4F9L4_Zea_mays
0,94
V9F5D5_Phytophthora_parasitica
0,92
A0A081A944_Phytophthora_parasitica
0,96
D0MVN8_Phytophthora_infestans
0,6
M4BZY6_Hyaloperonospora_arabidopsidis
1
G4YS73_Phytophthora_sojae
0,85
K4MB10_Methanolobus_psychrophilus
1
W9DZB8_Methanolobus_tindarius
0,99
J1ASR8_Methanofollis_liminatans
1
A0A0F7IH39_Geoglobus_ahangari
0,38
0,31
A0LG73_Syntrophobacter_fumaroxidans
G7WFX4_Desulfosporosinus_orientis
1
G2G1N6_Desulfosporosinus_sp.
0,93
0,81
A0A0E3S419_Methanosarcina_lacustris
R7CTM8_Bacteroides_sp.
0,76
0,78
A0A077ZGC6_Trichuris_trichiura
B7Q7U2_Ixodes_scapularis
1
K7JCQ0_Nasonia_vitripennis
0,62
1
W3RL14_Afipia_sp.
F6GZH8_Vitis_vinifera
0,95
W9SKF2_Morus_notabilis
0,95
A0A068U0Y4_Coffea_canephora
1
A9SUK8_Physcomitrella_patens
1
A7S731_Nematostella_vectensis
1
A7S3L2_Nematostella_vectensis
0,63
W4Y0G6_Strongylocentrotus_purpuratus
0,44
0,27
R7UG21_Capitella_teleta
Q294C0_Drosophila_pseudoobscura
N6U917_Dendroctonus_ponderosae
Q1L727_Aedes_aegypti
0.4

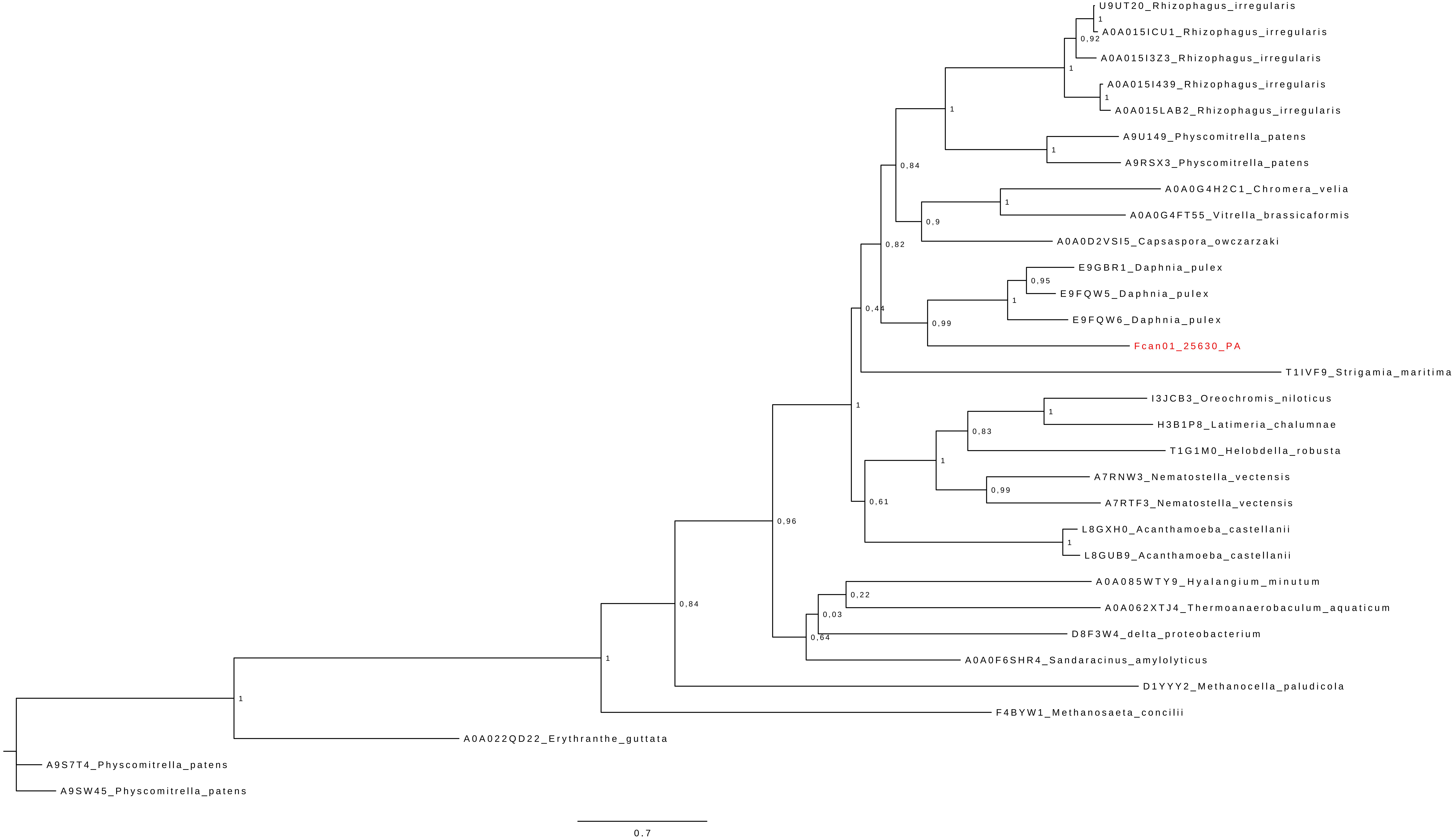
U9UT20_Rhizophagus_irregularis
A0A015ICU1_Rhizophagus_irregularis
A0A015I3Z3_Rhizophagus_irregularis
A0A015I439_Rhizophagus_irregularis
A0A015LAB2_Rhizophagus_irregularis
A9U149_Physcomitrella_patens
A9RSX3_Physcomitrella_patens
A0A0G4H2C1_Chromera_velia
A0A0G4FT55_Vitrella_brassicaformis
A0A0D2VSI5_Capsaspora_owczarzaki
E9GBR1_Daphnia_pulex
E9FQW5_Daphnia_pulex
E9FQW6_Daphnia_pulex
Fcan01_25630_PA
T1IVF9_Strigamia_maritima
I3JCB3_Oreochromis_niloticus
H3B1P8_Latimeria_chalumnae
T1G1M0_Helobdella_robusta
A7RNW3_Nematostella_vectensis
A7RTF3_Nematostella_vectensis
L8GXH0_Acanthamoeba_castellanii
L8GUB9_Acanthamoeba_castellanii
A0A085WTY9_Hyalangium_minutum
A0A062XTJ4_Thermoanaerobaculum_aquaticum
D8F3W4_delta_proteobacterium
A0A0F6SHR4_Sandaracinus_amylolyticus
D1YYY2_Methanocella_paludicola
F4BYW1_Methanosaeta_concilii
A0A022QD22_Erythranthe_guttata
A9S7T4_Physcomitrella_patens
A9SW45_Physcomitrella_patens
0.7

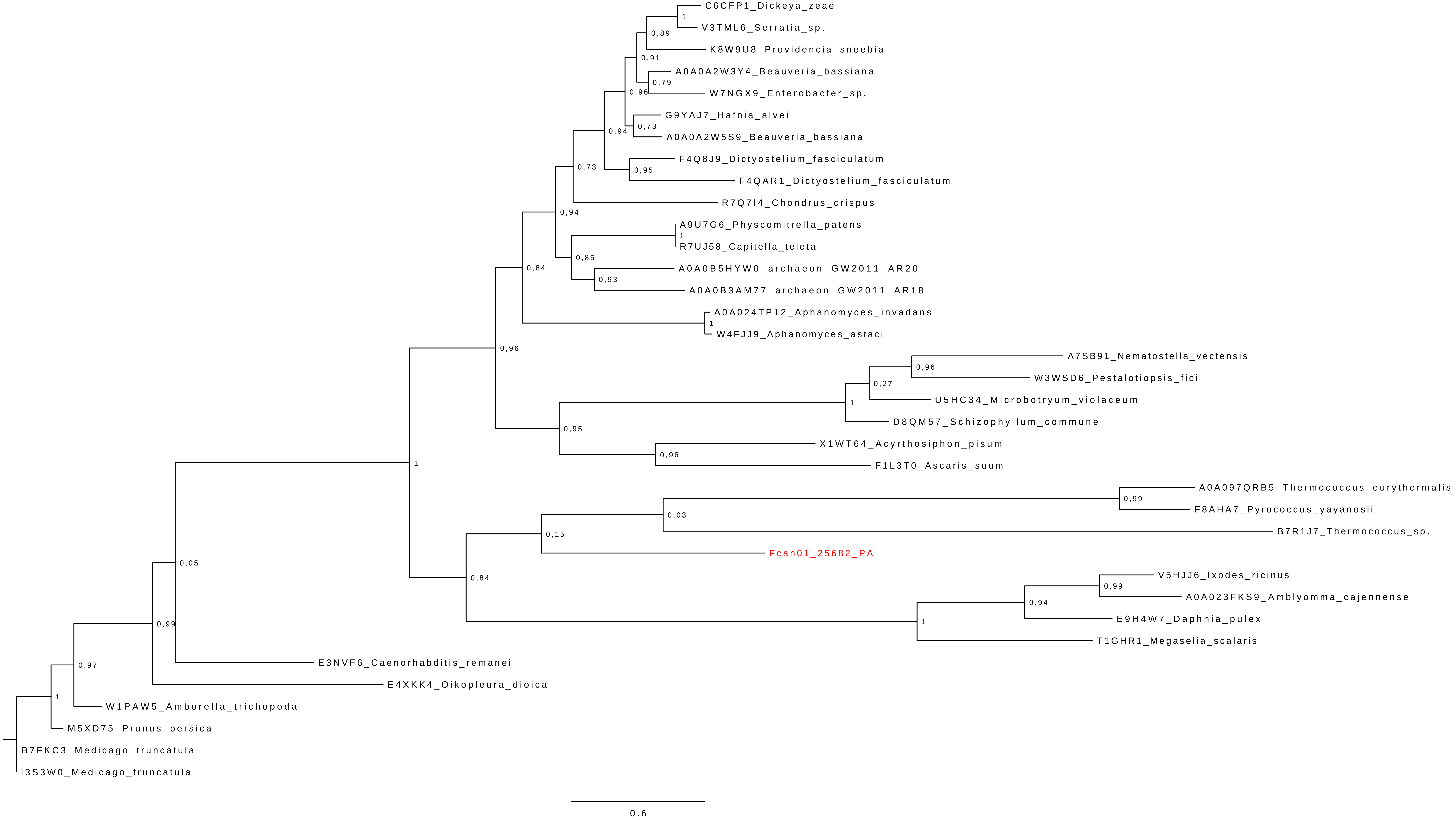
C6CFP1_Dickeya_zeae
V3TML6_Serratia_sp.
K8W9U8_Providencia_sneebia
A0A0A2W3Y4_Beauveria_bassiana
W7NGX9_Enterobacter_sp.
G9YAJ7_Hafnia_alvei
A0A0A2W5S9_Beauveria_bassiana
F4Q8J9_Dictyostelium_fasciculatum
F4QAR1_Dictyostelium_fasciculatum
R7Q7I4_Chondrus_crispus
A9U7G6_Physcomitrella_patens
R7UJ58_Capitella_teleta
A0A0B5HYW0_archaeon_GW2011_AR20
A0A0B3AM77_archaeon_GW2011_AR18
A0A024TP12_Aphanomyces_invadans
W4FJJ9_Aphanomyces_astaci
A7SB91_Nematostella_vectensis
W3WSD6_Pestalotiopsis_fici
U5HC34_Microbotryum_violaceum
D8QM57_Schizophyllum_commune
X1WT64_Acyrthosiphon_pisum
F1L3T0_Ascaris_suum
A0A097QRB5_Thermococcus_eurythermalis
F8AHA7_Pyrococcus_yayanosii
B7R1J7_Thermococcus_sp.
Fcan01_25682_PA
V5HJJ6_Ixodes_ricinus
A0A023FKS9_Amblyomma_cajennense
E9H4W7_Daphnia_pulex
T1GHR1_Megaselia_scalaris
E3NVF6_Caenorhabditis_remanei
E4XKK4_Oikopleura_dioica
W1PAW5_Amborella_trichopoda
M5XD75_Prunus_persica
B7FKC3_Medicago_truncatula
I3S3W0_Medicago_truncatula
1
0,89
0,91
0,79
0,96
0,73
0,94
0,95
0,73
1
0,94
0,85
0,93
0,84
1
0,96
0,96
0,27
1
0,95
0,96
1
0,99
0,03
0,15
0,05
0,84
0,99
0,94
1
0,99
0,97
1
0.6

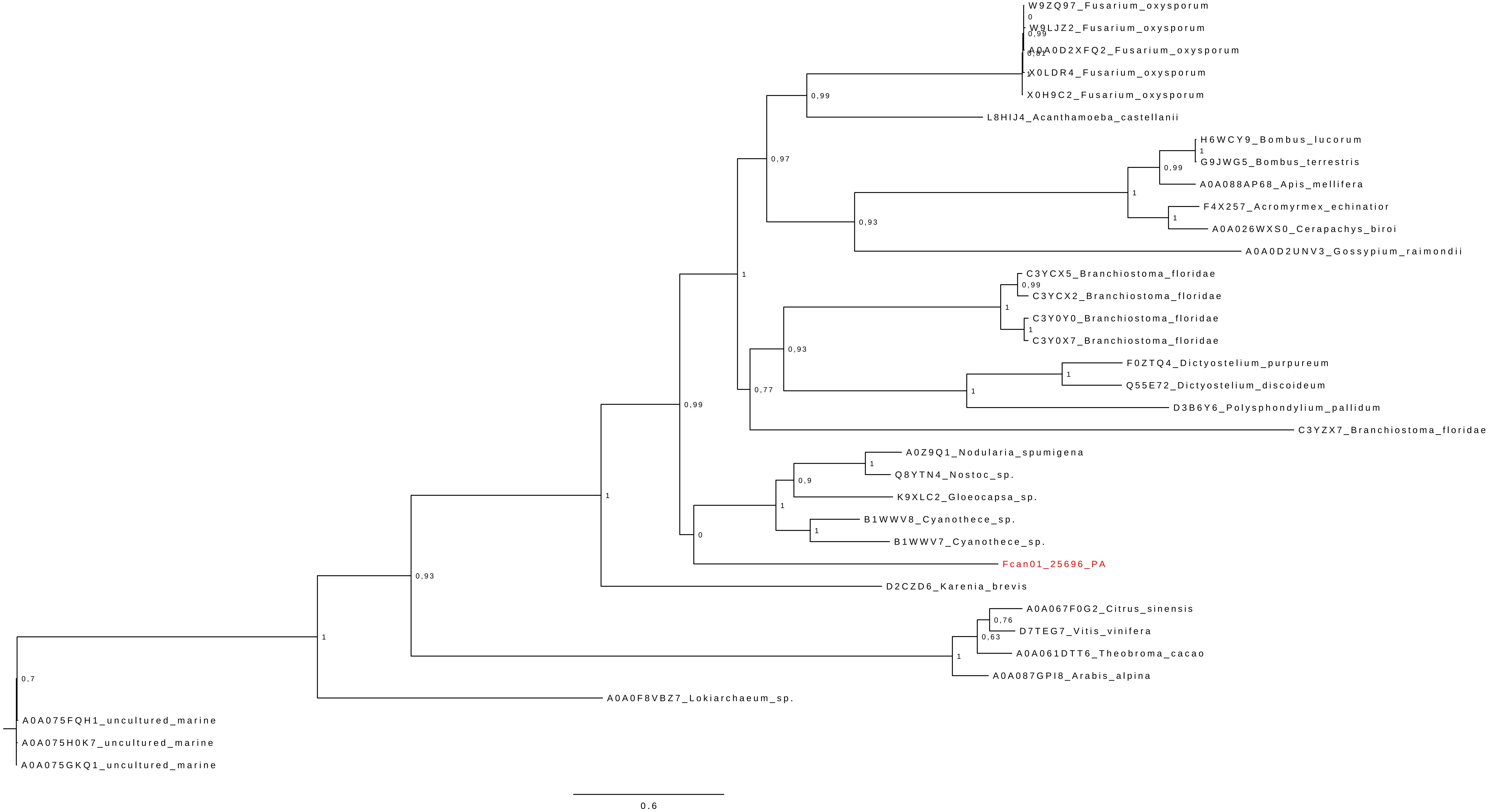
W9ZQ97_Fusarium_oxysporum
W9LJZ2_Fusarium_oxysporum
A0A0D2XFQ2_Fusarium_oxysporum
X0LDR4_Fusarium_oxysporum
X0H9C2_Fusarium_oxysporum
L8HIJ4_Acanthamoeba_castellanii
H6WCY9_Bombus_lucorum
G9JWG5_Bombus_terrestris
A0A088AP68_Apis_mellifera
F4X257_Acromyrmex_echinatior
A0A026WXS0_Cerapachys_biroi
A0A0D2UNV3_Gossypium_raimondii
C3YCX5_Branchiostoma_floridae
C3YCX2_Branchiostoma_floridae
C3Y0Y0_Branchiostoma_floridae
C3Y0X7_Branchiostoma_floridae
F0ZTQ4_Dictyostelium_purpureum
Q55E72_Dictyostelium_discoideum
D3B6Y6_Polysphondylium_pallidum
C3YZX7_Branchiostoma_floridae
A0Z9Q1_Nodularia_spumigena
Q8YTN4_Nostoc_sp.
K9XLC2_Gloeocapsa_sp.
B1WWV8_Cyanothece_sp.
B1WWV7_Cyanothece_sp.
Fcan01_25696_PA
D2CZD6_Karenia_brevis
A0A067F0G2_Citrus_sinensis
D7TEG7_Vitis_vinifera
A0A061DTT6_Theobroma_cacao
A0A087GPI8_Arabis_alpina
A0A0F8VBZ7_Lokiarchaeum_sp.
A0A075FQH1_uncultured_marine
A0A075H0K7_uncultured_marine
A0A075GKQ1_uncultured_marine
0
0,99
0,81
1
0,99
0,97
1
0,99
1
0,93
1
0,99
1
1
0,93
1
1
0,77
0,99
1
0,9
1
1
0
1
0,76
0,63
1
0,93
1
0,7
0.6

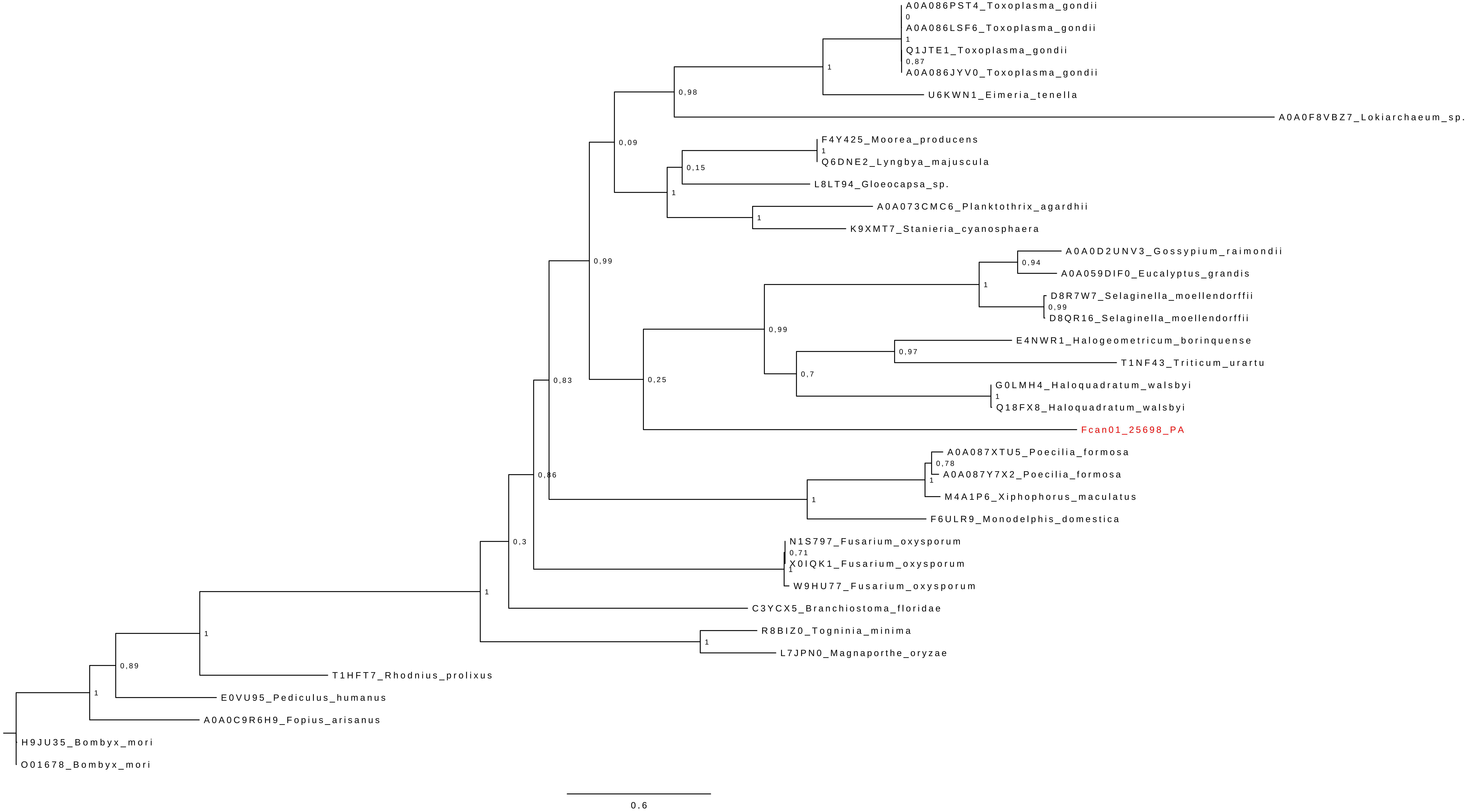
A0A086PST4_Toxoplasma_gondii
0
A0A086LSF6_Toxoplasma_gondii
1
Q1JTE1_Toxoplasma_gondii
0,87
A0A086JYV0_Toxoplasma_gondii
1
U6KWN1_Eimeria_tenella
0,98
A0A0F8VBZ7_Lokiarchaeum_sp.
0,09
F4Y425_Moorea_producens
1
Q6DNE2_Lyngbya_majuscula
0,15
L8LT94_Gloeocapsa_sp.
1
A0A073CMC6_Planktothrix_agardhii
1
K9XMT7_Stanieria_cyanosphaera
0,99
A0A0D2UNV3_Gossypium_raimondii
0,94
A0A059DIF0_Eucalyptus_grandis
1
D8R7W7_Selaginella_moellendorffii
0,99
D8QR16_Selaginella_moellendorffii
0,99
E4NWR1_Halogeometricum_borinquense
0,97
T1NF43_Triticum_urartu
0,7
G0LMH4_Haloquadratum_walsbyi
1
Q18FX8_Haloquadratum_walsbyi
0,83
0,25
Fcan01_25698_PA
A0A087XTU5_Poecilia_formosa
0,78
A0A087Y7X2_Poecilia_formosa
0,86
1
M4A1P6_Xiphophorus_maculatus
1
F6ULR9_Monodelphis_domestica
N1S797_Fusarium_oxysporum
0,71
X0IQK1_Fusarium_oxysporum
0,3
1
W9HU77_Fusarium_oxysporum
1
C3YCX5_Branchiostoma_floridae
R8BIZ0_Togninia_minima
1
L7JPN0_Magnaporthe_oryzae
1
T1HFT7_Rhodnius_prolixus
0,89
1
E0VU95_Pediculus_humanus
A0A0C9R6H9_Fopius_arisanus
H9JU35_Bombyx_mori
O01678_Bombyx_mori
0.6

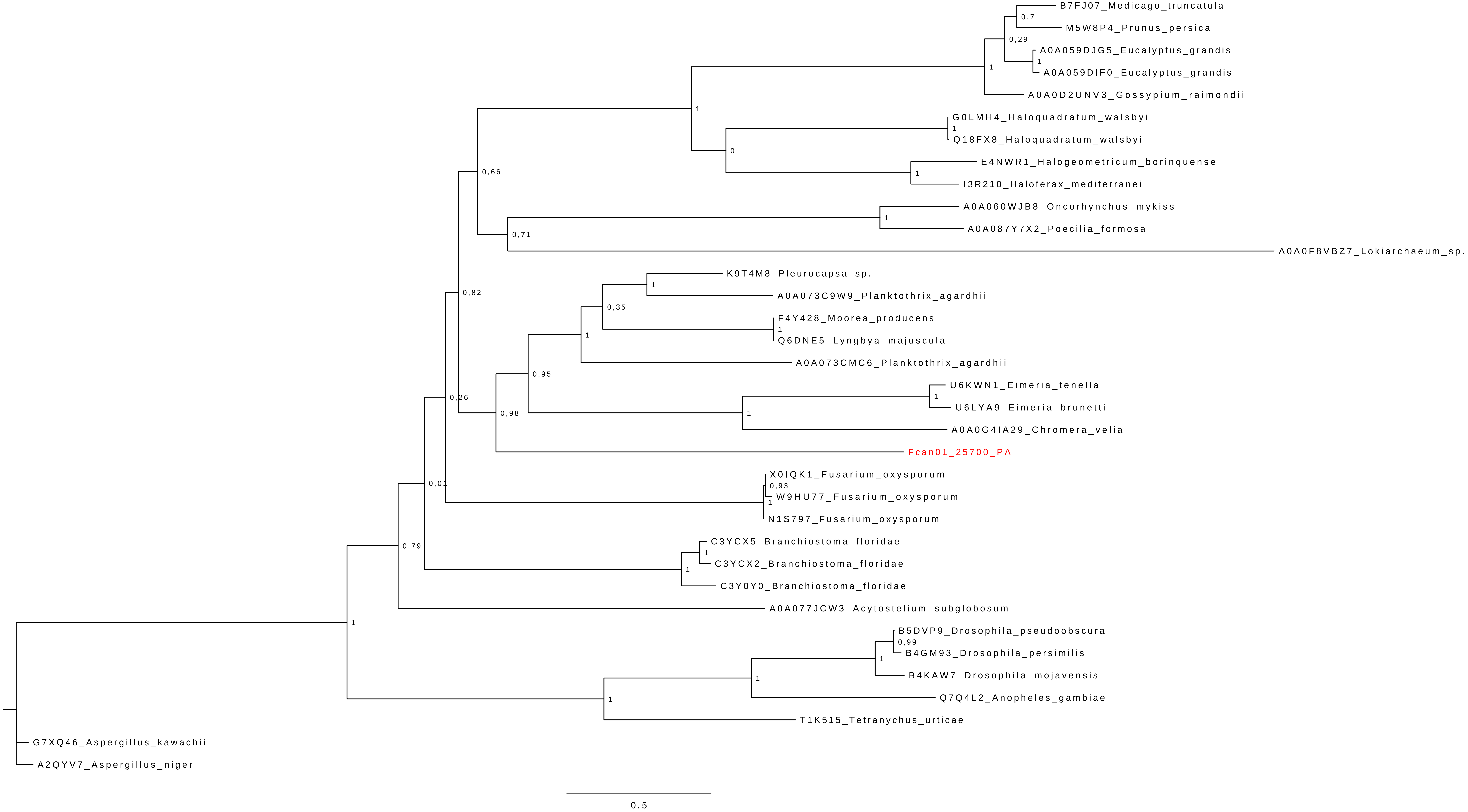

B7FJ07_Medicago_truncatula
M5W8P4_Prunus_persica
A0A059DJG5_Eucalyptus_grandis
A0A059DIF0_Eucalyptus_grandis
A0A0D2UNV3_Gossypium_raimondii
G0LMH4_Haloquadratum_walsbyi
Q18FX8_Haloquadratum_walsbyi
E4NWR1_Halogeometricum_borinquense
I3R210_Haloferax_mediterranei
A0A060WJB8_Oncorhynchus_mykiss
A0A087Y7X2_Poecilia_formosa
A0A0F8VBZ7_Lokiarchaeum_sp.
K9T4M8_Pleurocapsa_sp.
A0A073C9W9_Planktothrix_agardhii
F4Y428_Moorea_producens
Q6DNE5_Lyngbya_majuscula
A0A073CMC6_Planktothrix_agardhii
U6KWN1_Eimeria_tenella
U6LYA9_Eimeria_brunetti
A0A0G4IA29_Chromera_velia
Fcan01_25700_PA
X0IQK1_Fusarium_oxysporum
W9HU77_Fusarium_oxysporum
N1S797_Fusarium_oxysporum
C3YCX5_Branchiostoma_floridae
C3YCX2_Branchiostoma_floridae
C3Y0Y0_Branchiostoma_floridae
A0A077JCW3_Acytostelium_subglobosum
B5DVP9_Drosophila_pseudoobscura
B4GM93_Drosophila_persimilis
B4KAW7_Drosophila_mojavensis
Q7Q4L2_Anopheles_gambiae
T1K515_Tetranychus_urticae
G7XQ46_Aspergillus_kawachii
A2QYV7_Aspergillus_niger
0,7
0,29
1
0,66
0,71
0,82
0,35
0,95
0,26
0,98
0,01
0,93
0,79
0,99
0
0.5

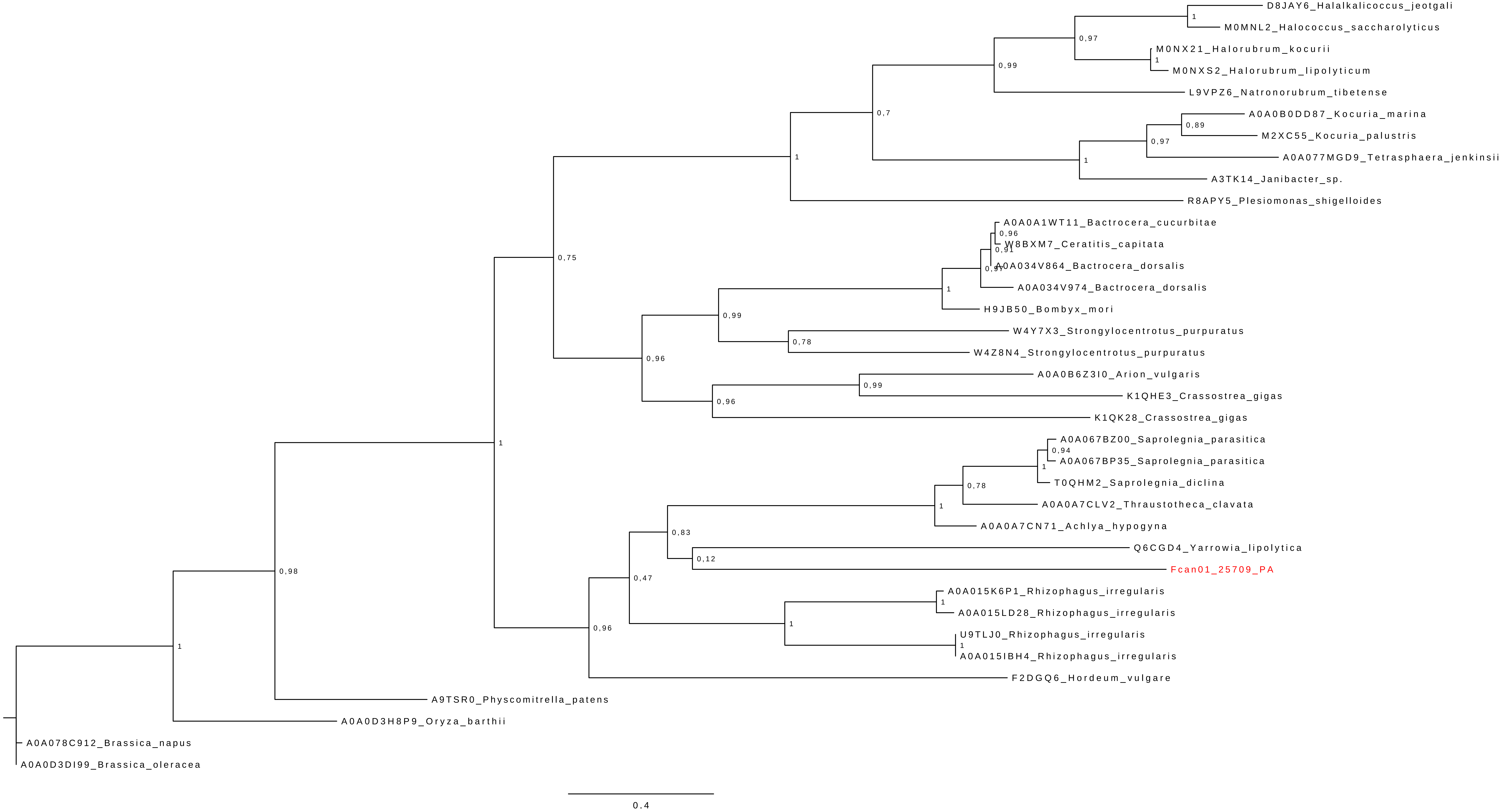
D8JAY6_Halalkalicoccus_jeotgali
M0MNL2_Halococcus_saccharolyticus
M0NX21_Halorubrum_kocurii
M0NXS2_Halorubrum_lipolyticum
L9VPZ6_Natronorubrum_tibetense
A0A0B0DD87_Kocuria_marina
M2XC55_Kocuria_palustris
A0A077MGD9_Tetrasphaera_jenkinsii
A3TK14_Janibacter_sp.
R8APY5_Plesiomonas_shigelloides
A0A0A1WT11_Bactrocera_cucurbitae
W8BXM7_Ceratitis_capitata
A0A034V864_Bactrocera_dorsalis
A0A034V974_Bactrocera_dorsalis
H9JB50_Bombyx_mori
W4Y7X3_Strongylocentrotus_purpuratus
W4Z8N4_Strongylocentrotus_purpuratus
A0A0B6Z3I0_Arion_vulgaris
K1QHE3_Crassostrea_gigas
K1QK28_Crassostrea_gigas
A0A067BZ00_Saprolegnia_parasitica
A0A067BP35_Saprolegnia_parasitica
T0QHM2_Saprolegnia_diclina
A0A0A7CLV2_Thraustotheca_clavata
A0A0A7CN71_Achlya_hypogyna
Q6CGD4_Yarrowia_lipolytica
Fcan01_25709_PA
A0A015K6P1_Rhizophagus_irregularis
A0A015LD28_Rhizophagus_irregularis
U9TLJ0_Rhizophagus_irregularis
A0A015IBH4_Rhizophagus_irregularis
F2DGQ6_Hordeum_vulgare
A9TSR0_Physcomitrella_patens
A0A0D3H8P9_Oryza_barthii
A0A078C912_Brassica_napus
A0A0D3DI99_Brassica_oleracea
0.4

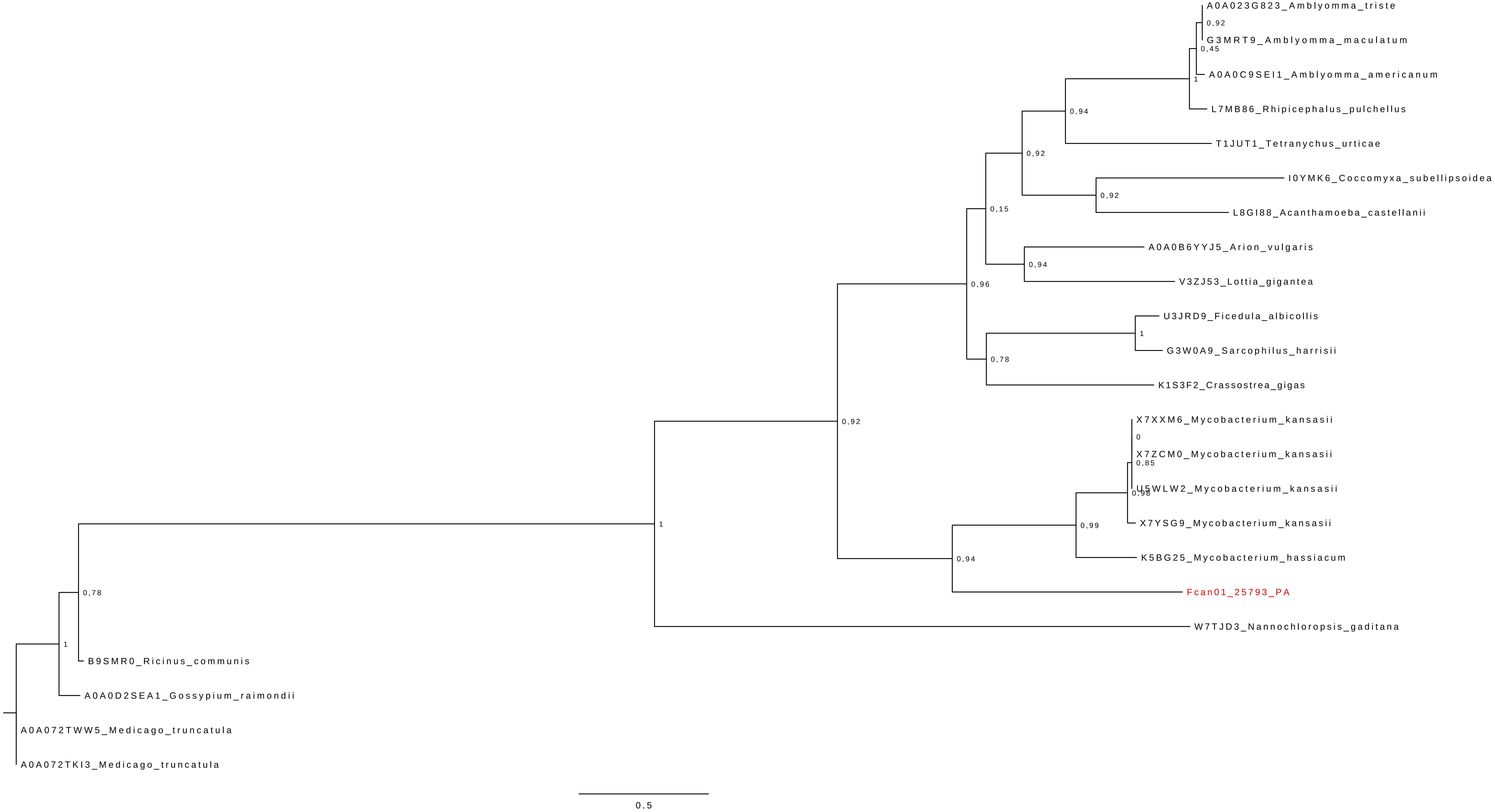
A0A023G823_Amblyomma_triste
0,92
G3MRT9_Amblyomma_maculatum
0,45
A0A0C9SEI1_Amblyomma_americanum
1
L7MB86_Rhipicephalus_pulchellus
0,94
T1JUT1_Tetranychus_urticae
0,92
I0YMK6_Coccomyxa_subellipsoidea
0,92
L8GI88_Acanthamoeba_castellanii
0,15
A0A0B6YYJ5_Arion_vulgaris
0,94
V3ZJ53_Lottia_gigantea
0,96
U3JRD9_Ficedula_albicollis
1
G3W0A9_Sarcophilus_harrisii
0,78
K1S3F2_Crassostrea_gigas
0,92
X7XXM6_Mycobacterium_kansasii
0
X7ZCM0_Mycobacterium_kansasii
0,85
U5WLW2_Mycobacterium_kansasii
0,98
X7YSG9_Mycobacterium_kansasii
0,99
K5BG25_Mycobacterium_hassiacum
0,94
Fcan01_25793_PA
1
W7TJD3_Nannochloropsis_gaditana
0,78
1
B9SMR0_Ricinus_communis
A0A0D2SEA1_Gossypium_raimondii
A0A072TWW5_Medicago_truncatula
A0A072TKI3_Medicago_truncatula
0.5

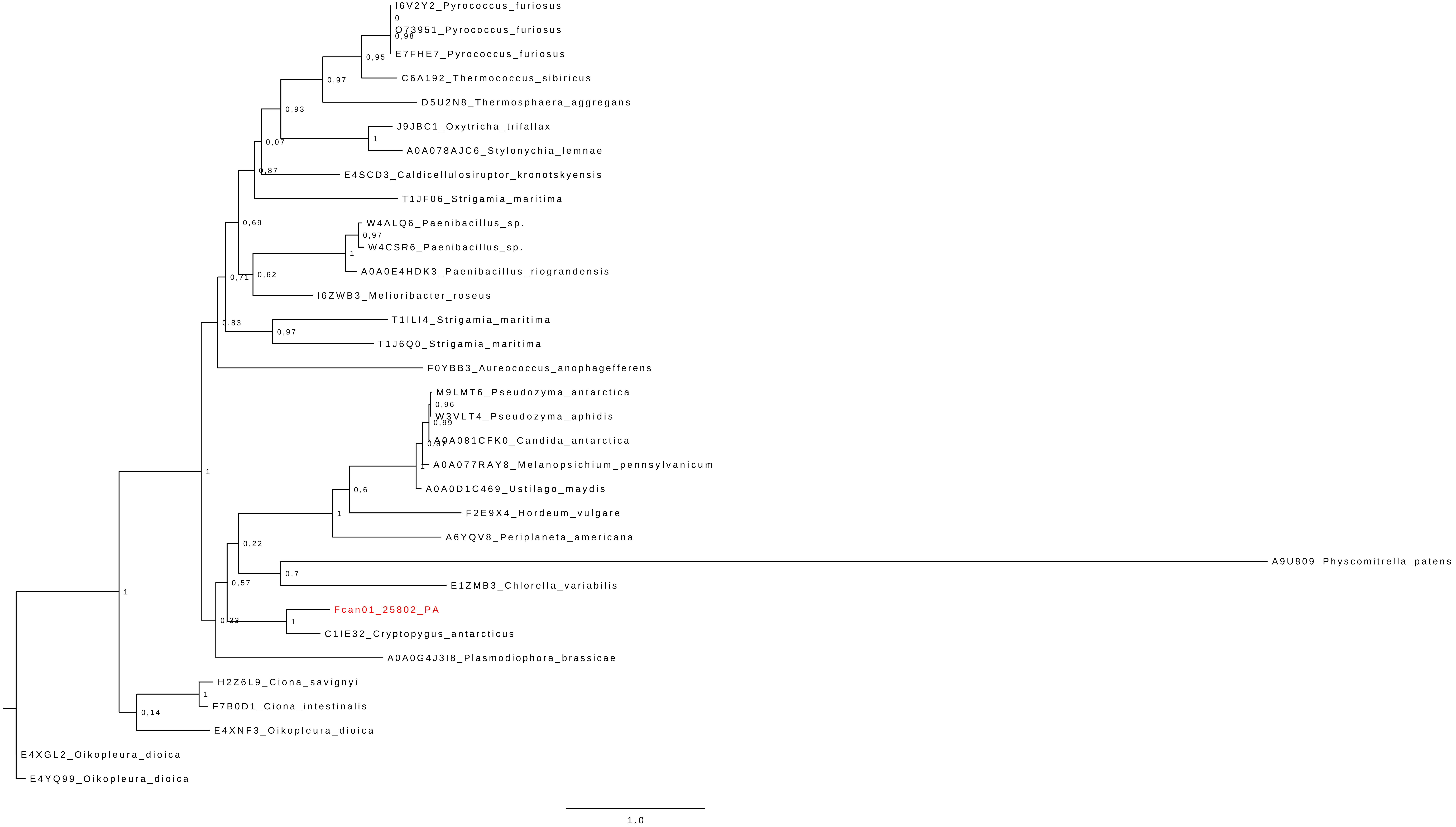
I6V2Y2_Pyrococcus_furiosus
O73951_Pyrococcus_furiosus
E7FHE7_Pyrococcus_furiosus
C6A192_Thermococcus_sibiricus
D5U2N8_Thermosphaera_aggregans
J9JBC1_Oxytricha_trifallax
A0A078AJC6_Stylonychia_lemnae
E4SCD3_Caldicellulosiruptor_kronotskyensis
T1JF06_Strigamia_maritima
W4ALQ6_Paenibacillus_sp.
W4CSR6_Paenibacillus_sp.
A0A0E4HDK3_Paenibacillus_riograndensis
I6ZWB3_Melioribacter_roseus
T1ILI4_Strigamia_maritima
T1J6Q0_Strigamia_maritima
F0YBB3_Aureococcus_anophagefferens
M9LMT6_Pseudozyma_antarctica
W3VLT4_Pseudozyma_aphidis
A0A081CFK0_Candida_antarctica
A0A077RAY8_Melanopsichium_pennsylvanicum
A0A0D1C469_Ustilago_maydis
F2E9X4_Hordeum_vulgare
A6YQV8_Periplaneta_americana
A9U809_Physcomitrella_patens
E1ZMB3_Chlorella_variabilis
Fcan01_25802_PA
C1IE32_Cryptopygus_antarcticus
A0A0G4J3I8_Plasmodiophora_brassicae
H2Z6L9_Ciona_savignyi
F7B0D1_Ciona_intestinalis
E4XNF3_Oikopleura_dioica
E4XGL2_Oikopleura_dioica
E4YQ99_Oikopleura_dioica
1.0

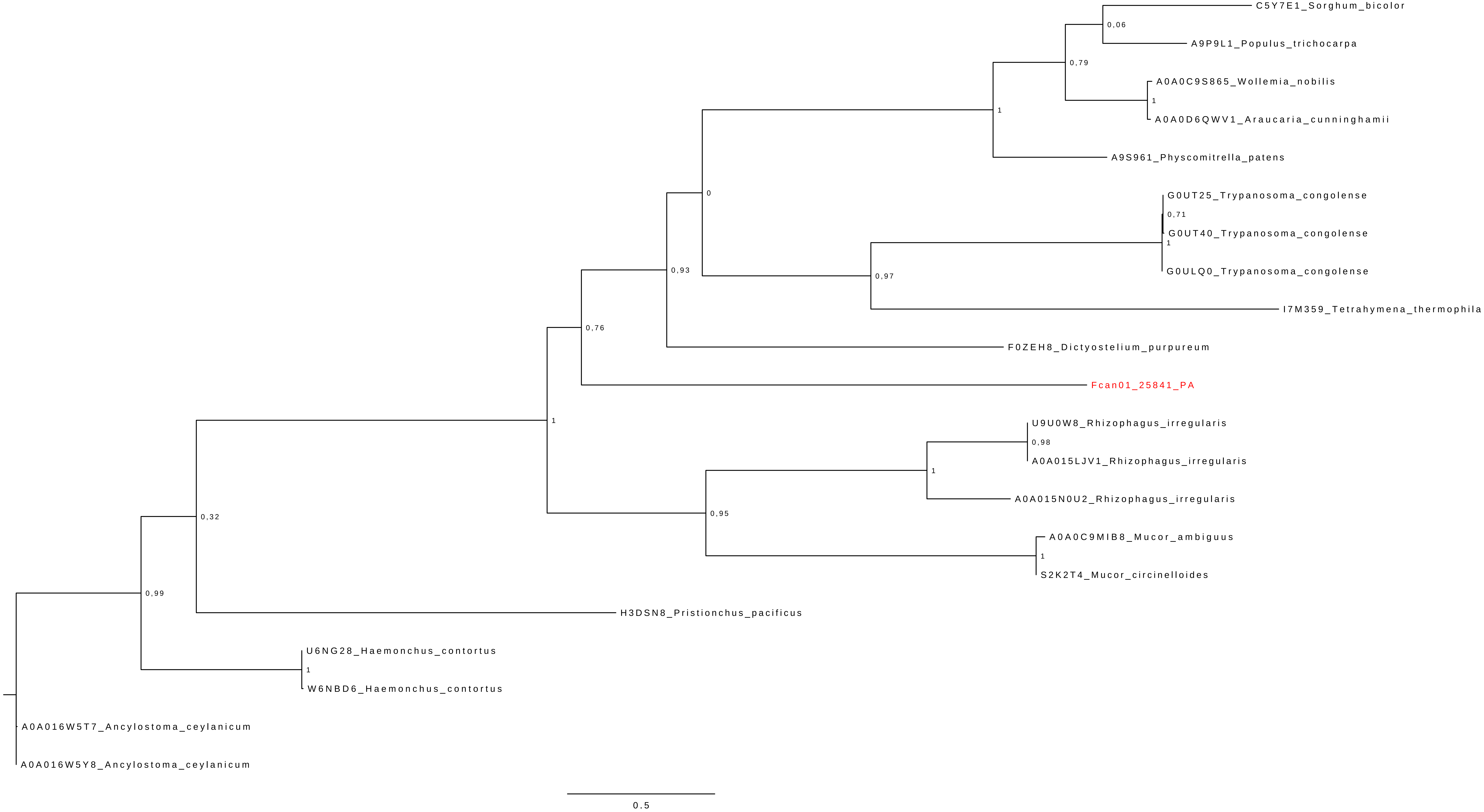
C5Y7E1_Sorghum_bicolor
0,06
A9P9L1_Populus_trichocarpa
0,79
A0A0C9S865_Wollemia_nobilis
1
A0A0D6QWV1_Araucaria_cunninghamii
1
A9S961_Physcomitrella_patens
0
G0UT25_Trypanosoma_congolense
0,71
G0UT40_Trypanosoma_congolense
1
G0ULQ0_Trypanosoma_congolense
0,93
0,97
I7M359_Tetrahymena_thermophila
0,76
F0ZEH8_Dictyostelium_purpureum
Fcan01_25841_PA
1
U9U0W8_Rhizophagus_irregularis
0,98
A0A015LJV1_Rhizophagus_irregularis
1
A0A015N0U2_Rhizophagus_irregularis
0,95
A0A0C9MIB8_Mucor_ambiguus
1
S2K2T4_Mucor_circinelloides
0,32
0,99
H3DSN8_Pristionchus_pacificus
U6NG28_Haemonchus_contortus
1
W6NBD6_Haemonchus_contortus
A0A016W5T7_Ancylostoma_ceylanicum
A0A016W5Y8_Ancylostoma_ceylanicum
0.5

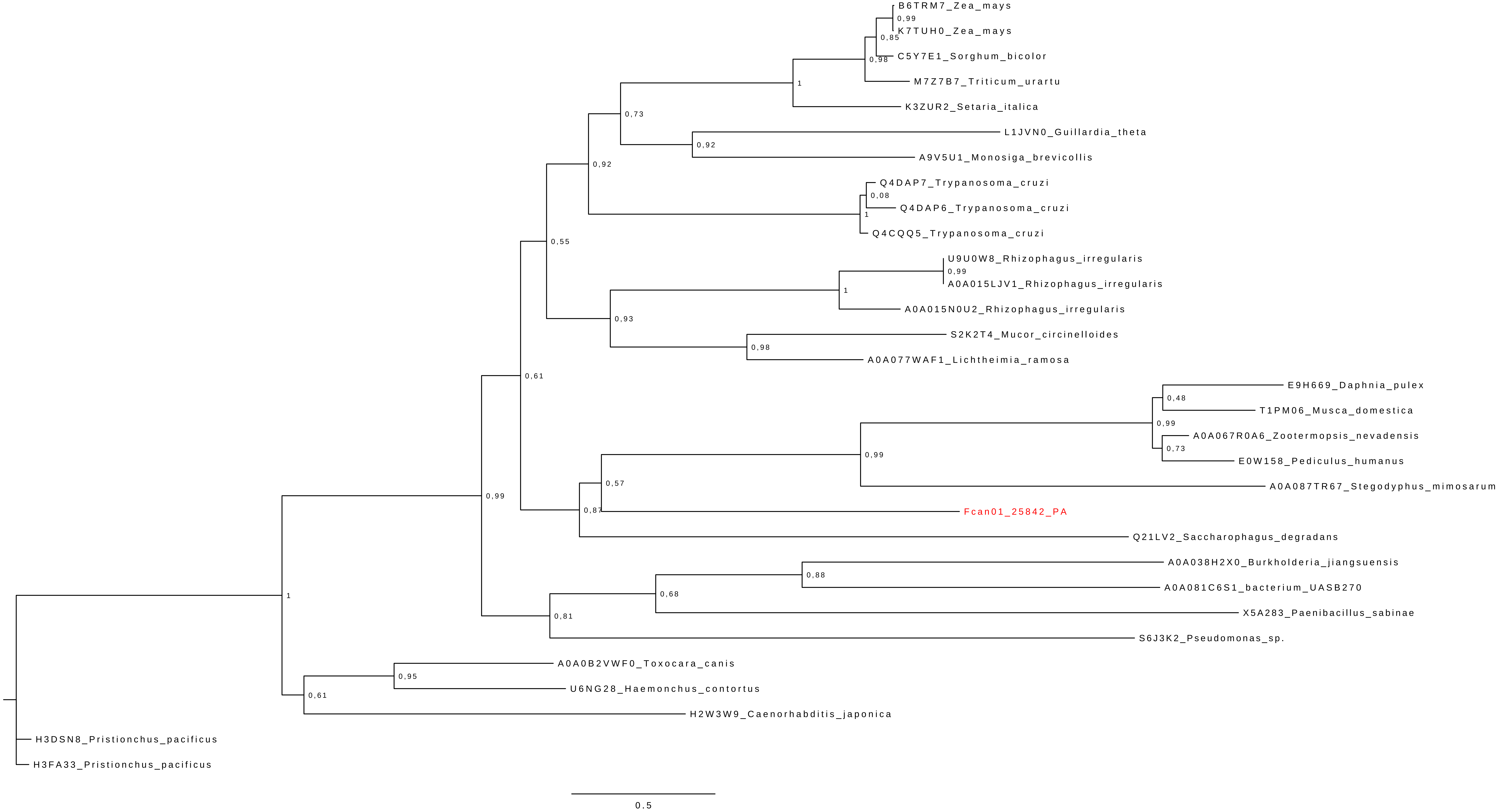
B6TRM7_Zea_mays
0,99
K7TUH0_Zea_mays
0,85
C5Y7E1_Sorghum_bicolor
0,98
M7Z7B7_Triticum_urartu
1
K3ZUR2_Setaria_italica
0,73
L1JVN0_Guillardia_theta
0,92
A9V5U1_Monosiga_brevicollis
0,92
Q4DAP7_Trypanosoma_cruzi
0,08
Q4DAP6_Trypanosoma_cruzi
1
Q4CQQ5_Trypanosoma_cruzi
0,55
U9U0W8_Rhizophagus_irregularis
0,99
A0A015LJV1_Rhizophagus_irregularis
1
A0A015N0U2_Rhizophagus_irregularis
0,93
S2K2T4_Mucor_circinelloides
0,98
A0A077WAF1_Lichtheimia_ramosa
0,61
E9H669_Daphnia_pulex
0,48
T1PM06_Musca_domestica
0,99
A0A067R0A6_Zootermopsis_nevadensis
0,73
E0W158_Pediculus_humanus
0,99
A0A087TR67_Stegodyphus_mimosarum
0,57
Fcan01_25842_PA
0,87
Q21LV2_Saccharophagus_degradans
0,99
A0A038H2X0_Burkholderia_jiangsuensis
0,88
A0A081C6S1_bacterium_UASB270
0,68
X5A283_Paenibacillus_sabinae
0,81
S6J3K2_Pseudomonas_sp.
1
A0A0B2VWF0_Toxocara_canis
0,95
U6NG28_Haemonchus_contortus
0,61
H2W3W9_Caenorhabditis_japonica
H3DSN8_Pristionchus_pacificus
H3FA33_Pristionchus_pacificus
0.5

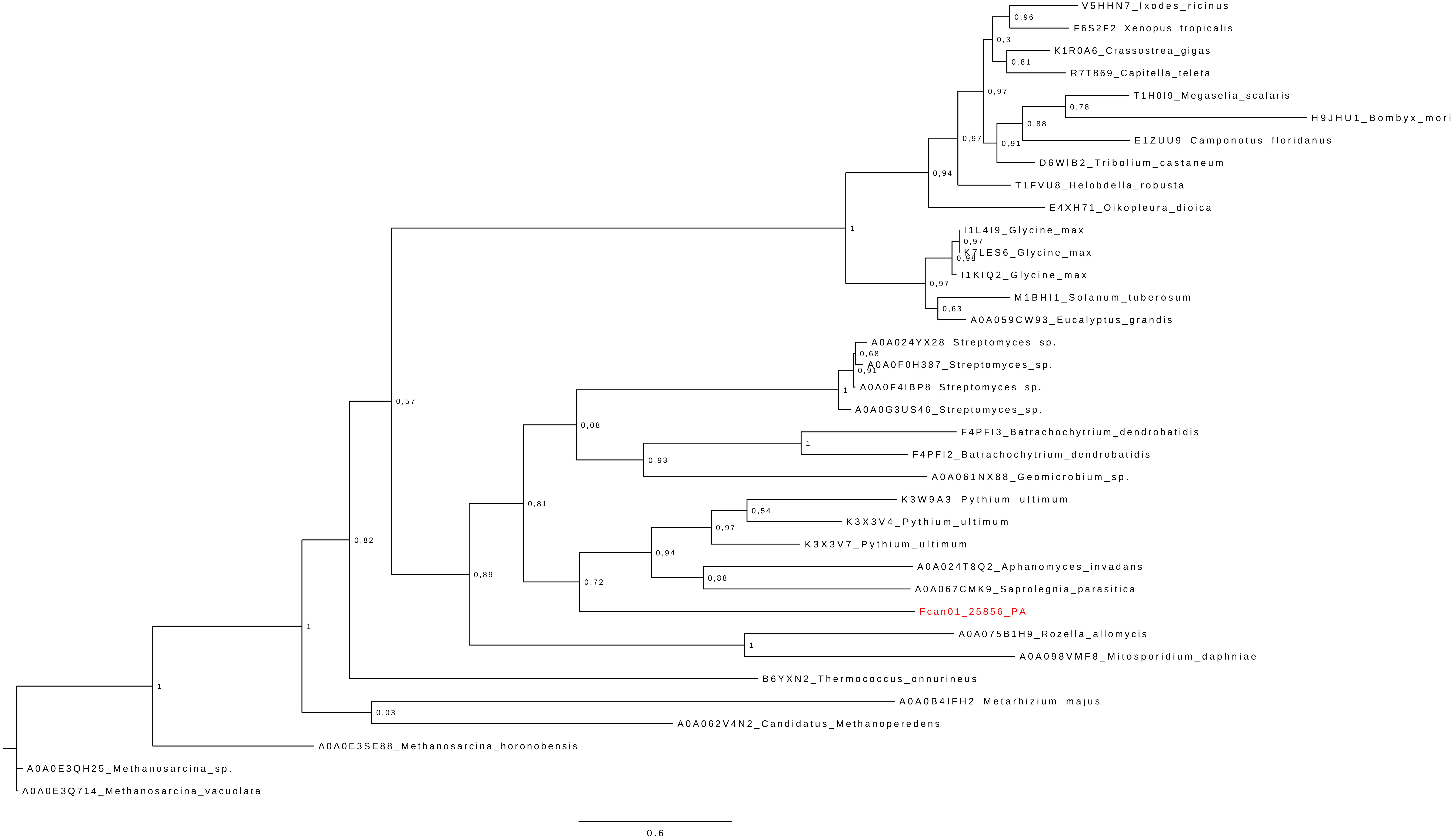
V5HHN7_Ixodes_ricinus
0,96
F6S2F2_Xenopus_tropicalis
0,3
K1R0A6_Crassostrea_gigas
0,81
R7T869_Capitella_teleta
0,97
T1H0I9_Megaselia_scalaris
0,78
H9JHU1_Bombyx_mori
0,88
E1ZUU9_Camponotus_floridanus
0,97
0,91
D6WIB2_Tribolium_castaneum
T1FVU8_Helobdella_robusta
0,94
E4XH71_Oikopleura_dioica
1
I1L4I9_Glycine_max
0,97
K7LES6_Glycine_max
0,98
I1KIQ2_Glycine_max
0,97
M1BHI1_Solanum_tuberosum
0,63
A0A059CW93_Eucalyptus_grandis
A0A024YX28_Streptomyces_sp.
0,68
A0A0F0H387_Streptomyces_sp.
0,91
A0A0F4IBP8_Streptomyces_sp.
1
A0A0G3US46_Streptomyces_sp.
0,57
0,08
F4PFI3_Batrachochytrium_dendrobatidis
1
F4PFI2_Batrachochytrium_dendrobatidis
0,93
A0A061NX88_Geomicrobium_sp.
0,81
K3W9A3_Pythium_ultimum
0,54
K3X3V4_Pythium_ultimum
0,97
K3X3V7_Pythium_ultimum
0,82
0,94
A0A024T8Q2_Aphanomyces_invadans
0,88
0,89
A0A067CMK9_Saprolegnia_parasitica
0,72
Fcan01_25856_PA
A0A075B1H9_Rozella_allomycis
1
1
A0A098VMF8_Mitosporidium_daphniae
B6YXN2_Thermococcus_onnurineus
1
A0A0B4IFH2_Metarhizium_majus
0,03
A0A062V4N2_Candidatus_Methanoperedens
A0A0E3SE88_Methanosarcina_horonobensis
A0A0E3QH25_Methanosarcina_sp.
A0A0E3Q714_Methanosarcina_vacuolata
0.6

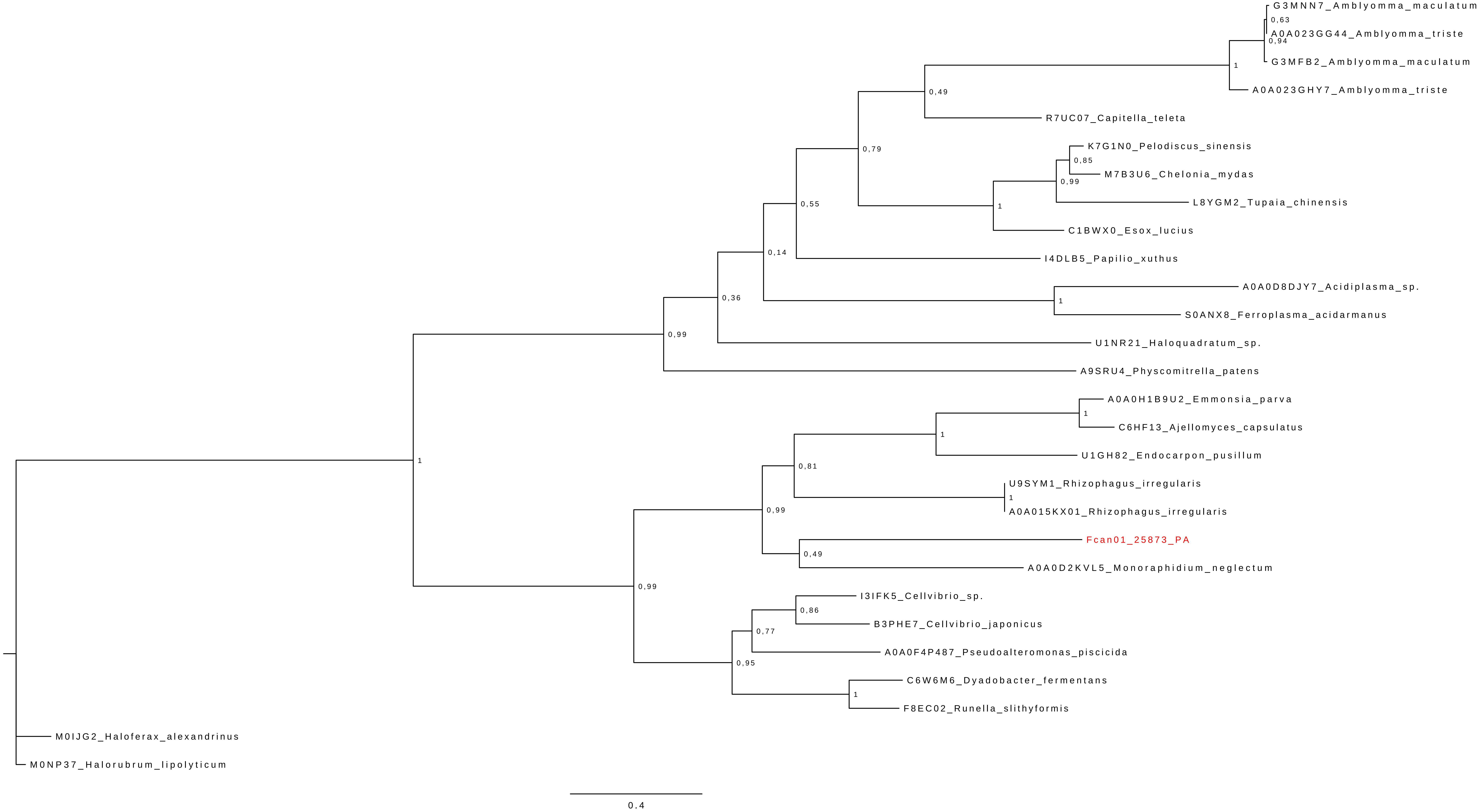
G3MNN7_Amblyomma_maculatum
0,63
A0A023GG44_Amblyomma_triste
0,94
G3MFB2_Amblyomma_maculatum
1
A0A023GHY7_Amblyomma_triste
0,49
R7UC07_Capitella_teleta
0,79
K7G1N0_Pelodiscus_sinensis
0,85
M7B3U6_Chelonia_mydas
0,99
L8YGM2_Tupaia_chinensis
1
C1BWX0_Esox_lucius
0,55
I4DLB5_Papilio_xuthus
0,14
A0A0D8DJY7_Acidiplasma_sp.
1
S0ANX8_Ferroplasma_acidarmanus
0,36
U1NR21_Haloquadratum_sp.
0,99
A9SRU4_Physcomitrella_patens
A0A0H1B9U2_Emmonsia_parva
1
C6HF13_Ajellomyces_capsulatus
1
U1GH82_Endocarpon_pusillum
0,81
U9SYM1_Rhizophagus_irregularis
1
A0A015KX01_Rhizophagus_irregularis
0,99
Fcan01_25873_PA
0,49
A0A0D2KVL5_Monoraphidium_neglectum
0,99
I3IFK5_Cellvibrio_sp.
0,86
B3PHE7_Cellvibrio_japonicus
0,77
A0A0F4P487_Pseudoalteromonas_piscicida
0,95
C6W6M6_Dyadobacter_fermentans
1
F8EC02_Runella_slithyformis
M0IJG2_Haloferax_alexandrinus
M0NP37_Halorubrum_lipolyticum
0.4

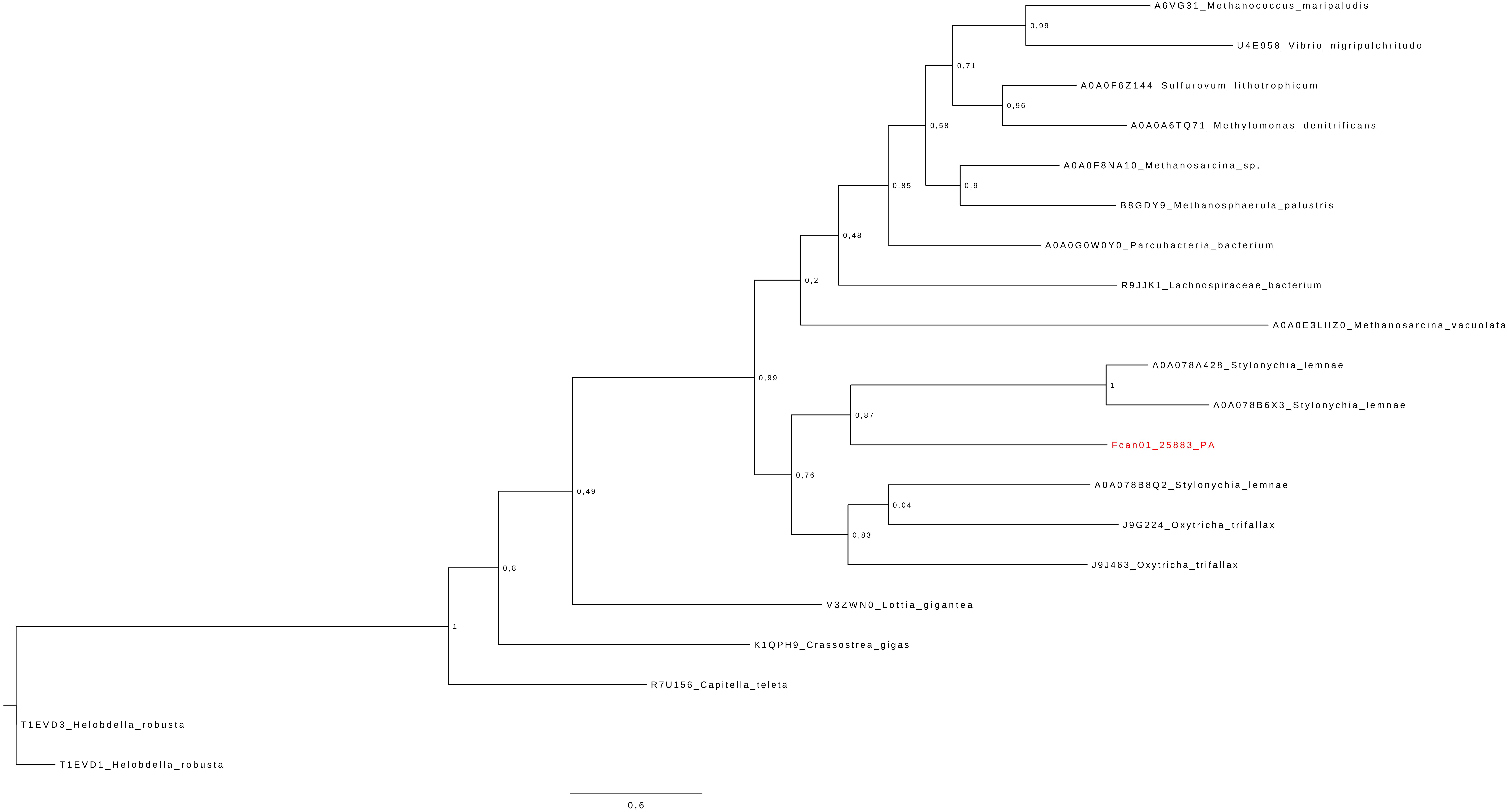
A6VG31_Methanococcus_maripaludis
0,99
U4E958_Vibrio_nigripulchritudo
0,71
A0A0F6Z144_Sulfurovum_lithotrophicum
0,96
A0A0A6TQ71_Methylomonas_denitrificans
0,58
A0A0F8NA10_Methanosarcina_sp.
0,9
B8GDY9_Methanosphaerula_palustris
0,85
A0A0G0W0Y0_Parcubacteria_bacterium
0,48
R9JJK1_Lachnospiraceae_bacterium
0,2
A0A0E3LHZ0_Methanosarcina_vacuolata
A0A078A428_Stylonychia_lemnae
1
A0A078B6X3_Stylonychia_lemnae
0,99
0,87
Fcan01_25883_PA
0,76
A0A078B8Q2_Stylonychia_lemnae
0,04
J9G224_Oxytricha_trifallax
0,83
J9J463_Oxytricha_trifallax
0,49
V3ZWN0_Lottia_gigantea
0,8
K1QPH9_Crassostrea_gigas
1
R7U156_Capitella_teleta
T1EVD3_Helobdella_robusta
T1EVD1_Helobdella_robusta
0.6

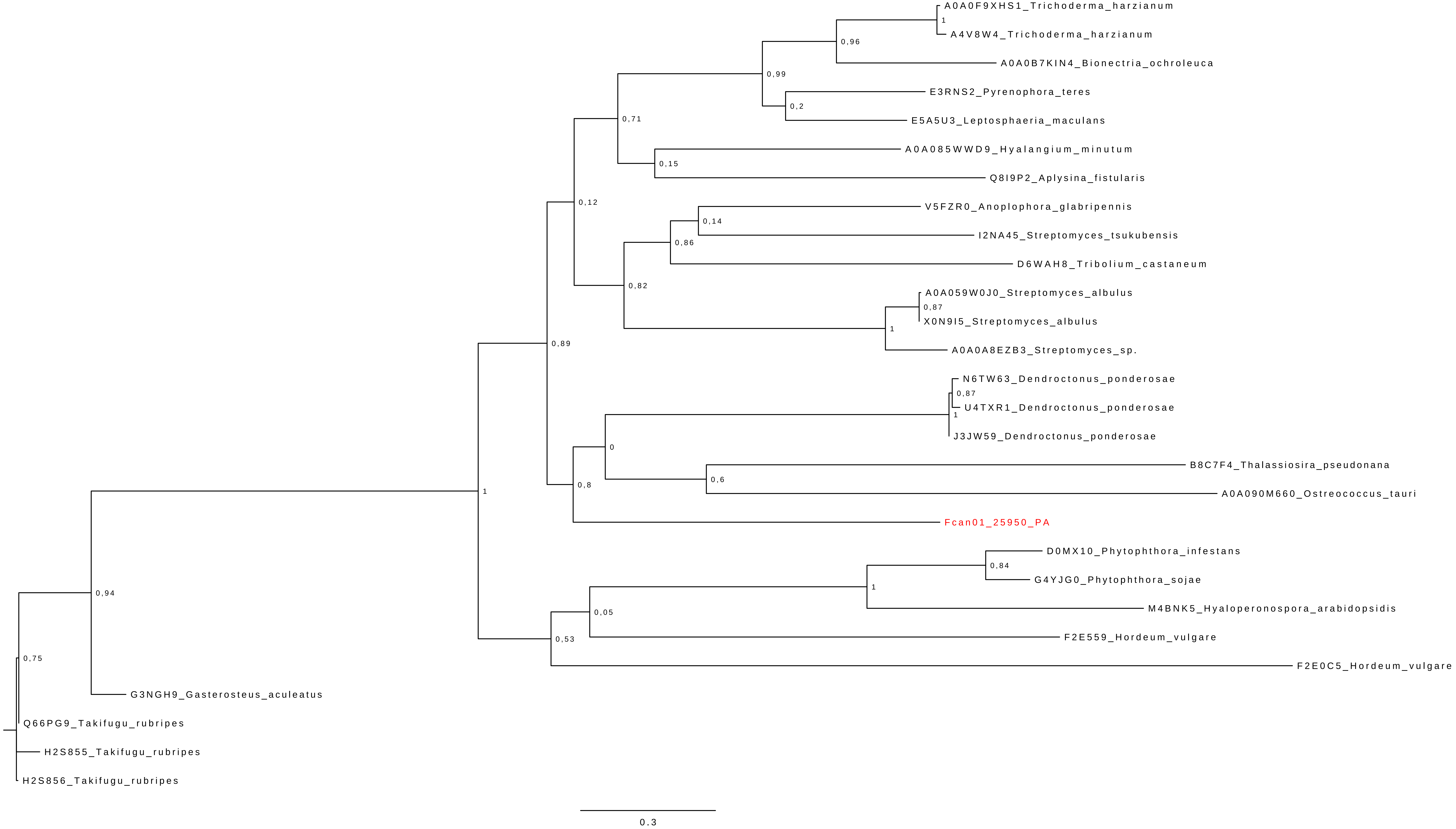

A0A0F9XHS1_Trichoderma_harzianum
1
A4V8W4_Trichoderma_harzianum
0,96
A0A0B7KIN4_Bionectria_ochroleuca
0,99
E3RNS2_Pyrenophora_teres
0,2
E5A5U3_Leptosphaeria_maculans
0,71
A0A085WWD9_Hyalangium_minutum
0,15
Q8I9P2_Aplysina_fistularis
0,12
V5FZR0_Anoplophora_glabripennis
0,14
I2NA45_Streptomyces_tsukubensis
0,86
D6WAH8_Tribolium_castaneum
0,82
A0A059W0J0_Streptomyces_albulus
0,87
X0N9I5_Streptomyces_albulus
1
A0A0A8EZB3_Streptomyces_sp.
0,89
N6TW63_Dendroctonus_ponderosae
0,87
U4TXR1_Dendroctonus_ponderosae
1
J3JW59_Dendroctonus_ponderosae
0
B8C7F4_Thalassiosira_pseudonana
0,6
A0A090M660_Ostreococcus_tauri
0,8
Fcan01_25950_PA
1
D0MX10_Phytophthora_infestans
0,84
G4YJG0_Phytophthora_sojae
1
M4BNK5_Hyaloperonospora_arabidopsidis
0,05
F2E559_Hordeum_vulgare
0,53
F2E0C5_Hordeum_vulgare
0,94
0,75
G3NGH9_Gasterosteus_aculeatus
Q66PG9_Takifugu_rubripes
H2S855_Takifugu_rubripes
H2S856_Takifugu_rubripes
0.3

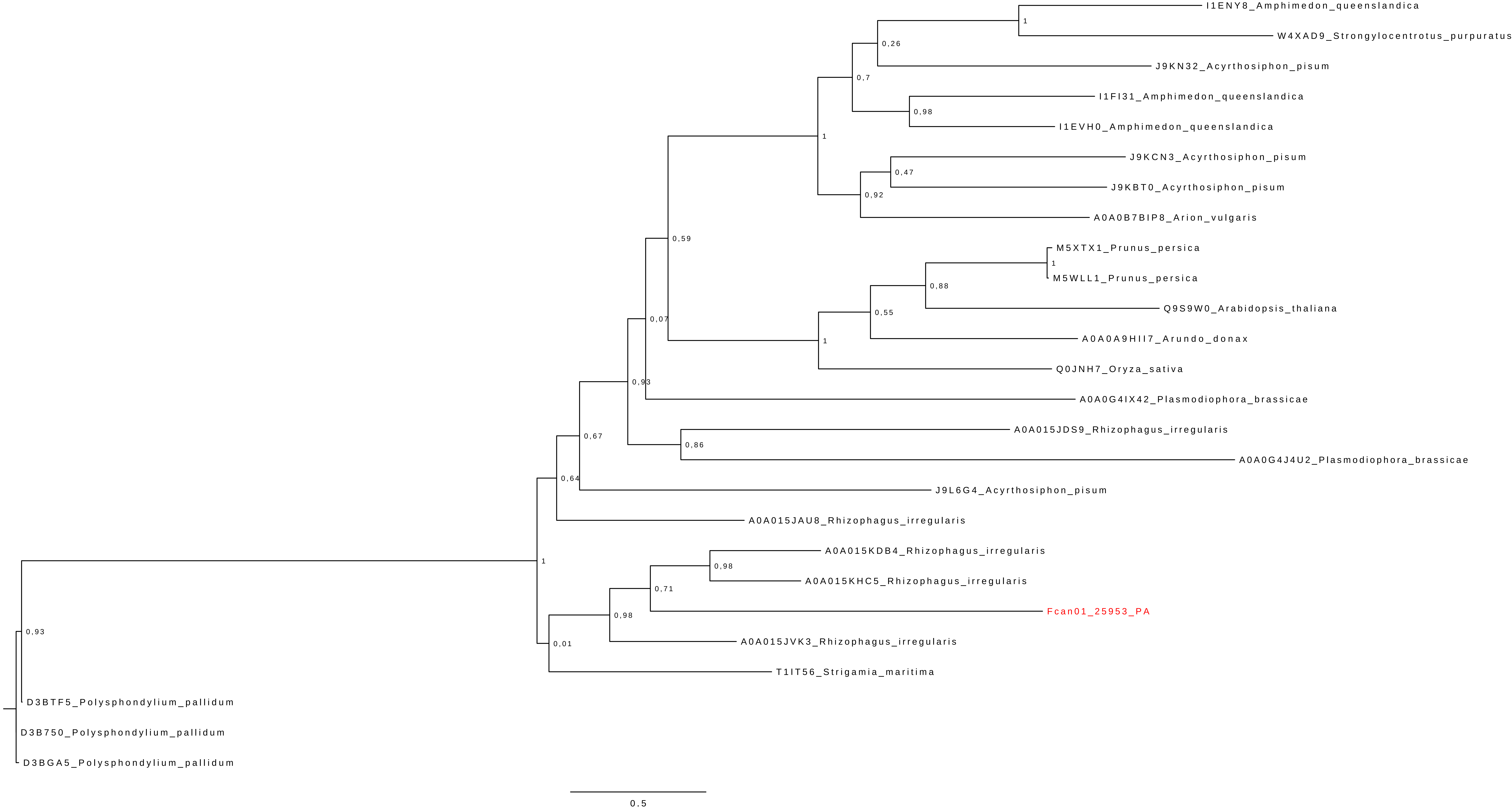
I1ENY8_Amphimedon_queenslandica
W4XAD9_Strongylocentrotus_purpuratus
J9KN32_Acyrthosiphon_pisum
I1FI31_Amphimedon_queenslandica
I1EVH0_Amphimedon_queenslandica
J9KCN3_Acyrthosiphon_pisum
J9KBT0_Acyrthosiphon_pisum
A0A0B7BIP8_Arion_vulgaris
M5XTX1_Prunus_persica
M5WLL1_Prunus_persica
Q9S9W0_Arabidopsis_thaliana
A0A0A9HII7_Arundo_donax
Q0JNH7_Oryza_sativa
A0A0G4IX42_Plasmodiophora_brassicae
A0A015JDS9_Rhizophagus_irregularis
A0A0G4J4U2_Plasmodiophora_brassicae
J9L6G4_Acyrthosiphon_pisum
A0A015JAU8_Rhizophagus_irregularis
A0A015KDB4_Rhizophagus_irregularis
A0A015KHC5_Rhizophagus_irregularis
Fcan01_25953_PA
A0A015JVK3_Rhizophagus_irregularis
T1IT56_Strigamia_maritima
D3BTF5_Polysphondylium_pallidum
D3B750_Polysphondylium_pallidum
D3BGA5_Polysphondylium_pallidum
1
0,26
0,7
0,98
1
0,47
0,92
0,59
1
0,88
0,55
0,07
1
0,93
0,86
0,67
0,64
1
0,98
0,71
0,98
0,01
0,93
0.5

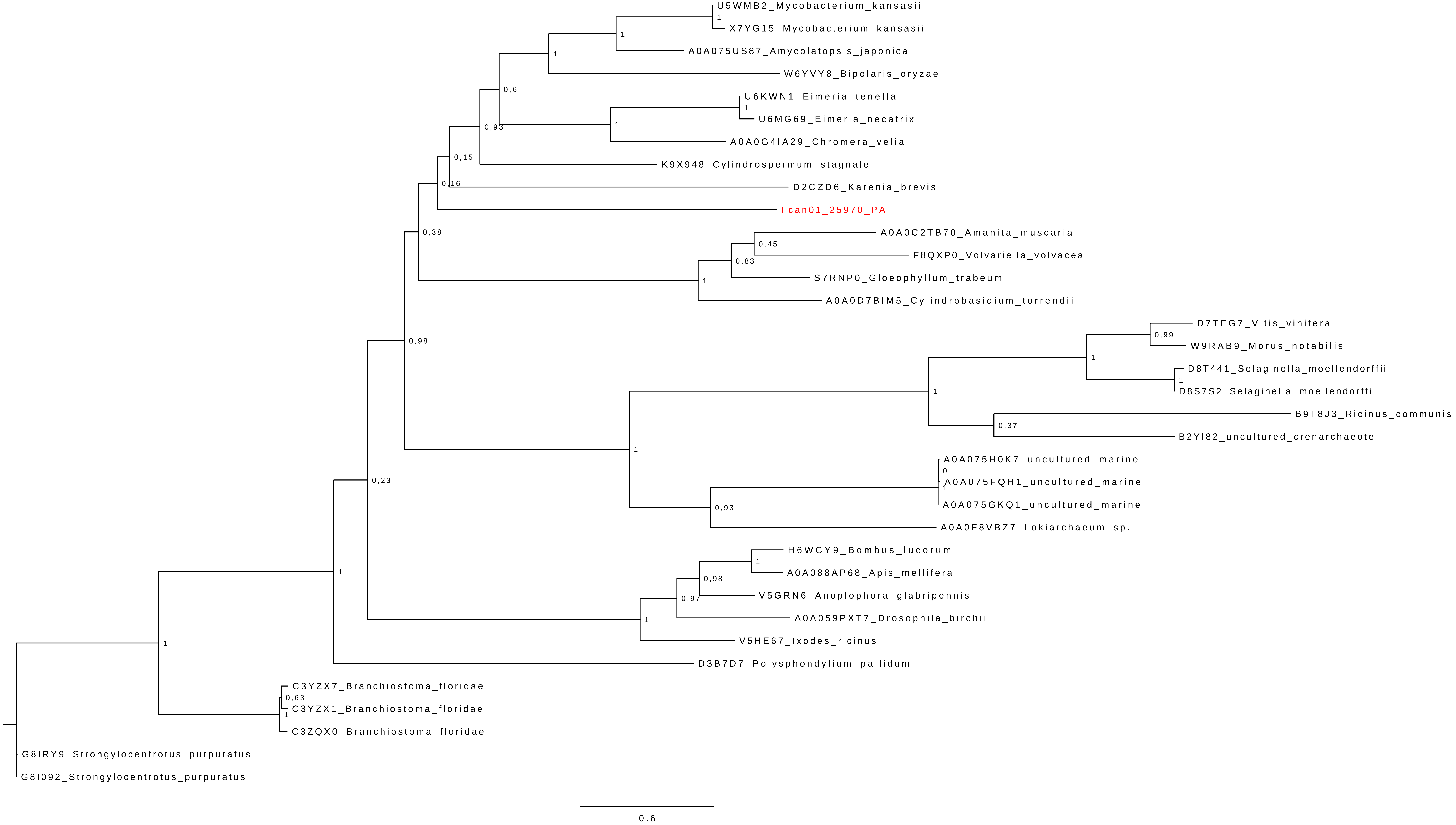
U5WMB2_Mycobacterium_kansasii
X7YG15_Mycobacterium_kansasii
A0A075US87_Amycolatopsis_japonica
W6YVY8_Bipolaris_oryzae
U6KWN1_Eimeria_tenella
U6MG69_Eimeria_necatrix
A0A0G4IA29_Chromera_velia
K9X948_Cylindrospermum_stagnale
D2CZD6_Karenia_brevis
Fcan01_25970_PA
A0A0C2TB70_Amanita_muscaria
F8QXP0_Volvariella_volvacea
S7RNP0_Gloeophyllum_trabeum
A0A0D7BIM5_Cylindrobasidium_torrendii
D7TEG7_Vitis_vinifera
W9RAB9_Morus_notabilis
D8T441_Selaginella_moellendorffii
D8S7S2_Selaginella_moellendorffii
B9T8J3_Ricinus_communis
B2YI82_uncultured_crenarchaeote
A0A075H0K7_uncultured_marine
A0A075FQH1_uncultured_marine
A0A075GKQ1_uncultured_marine
A0A0F8VBZ7_Lokiarchaeum_sp.
H6WCY9_Bombus_lucorum
A0A088AP68_Apis_mellifera
V5GRN6_Anoplophora_glabripennis
A0A059PXT7_Drosophila_birchii
V5HE67_Ixodes_ricinus
D3B7D7_Polysphondylium_pallidum
C3YZX7_Branchiostoma_floridae
C3YZX1_Branchiostoma_floridae
C3ZQX0_Branchiostoma_floridae
G8IRY9_Strongylocentrotus_purpuratus
G8I092_Strongylocentrotus_purpuratus
1
1
1
0,6
1
1
0,93
0,15
0,16
0,38
0,45
0,83
1
0,98
0,99
1
1
1
0,37
1
0
1
0,93
1
0,98
0,97
1
0,23
1
0,63
1
1
0.6

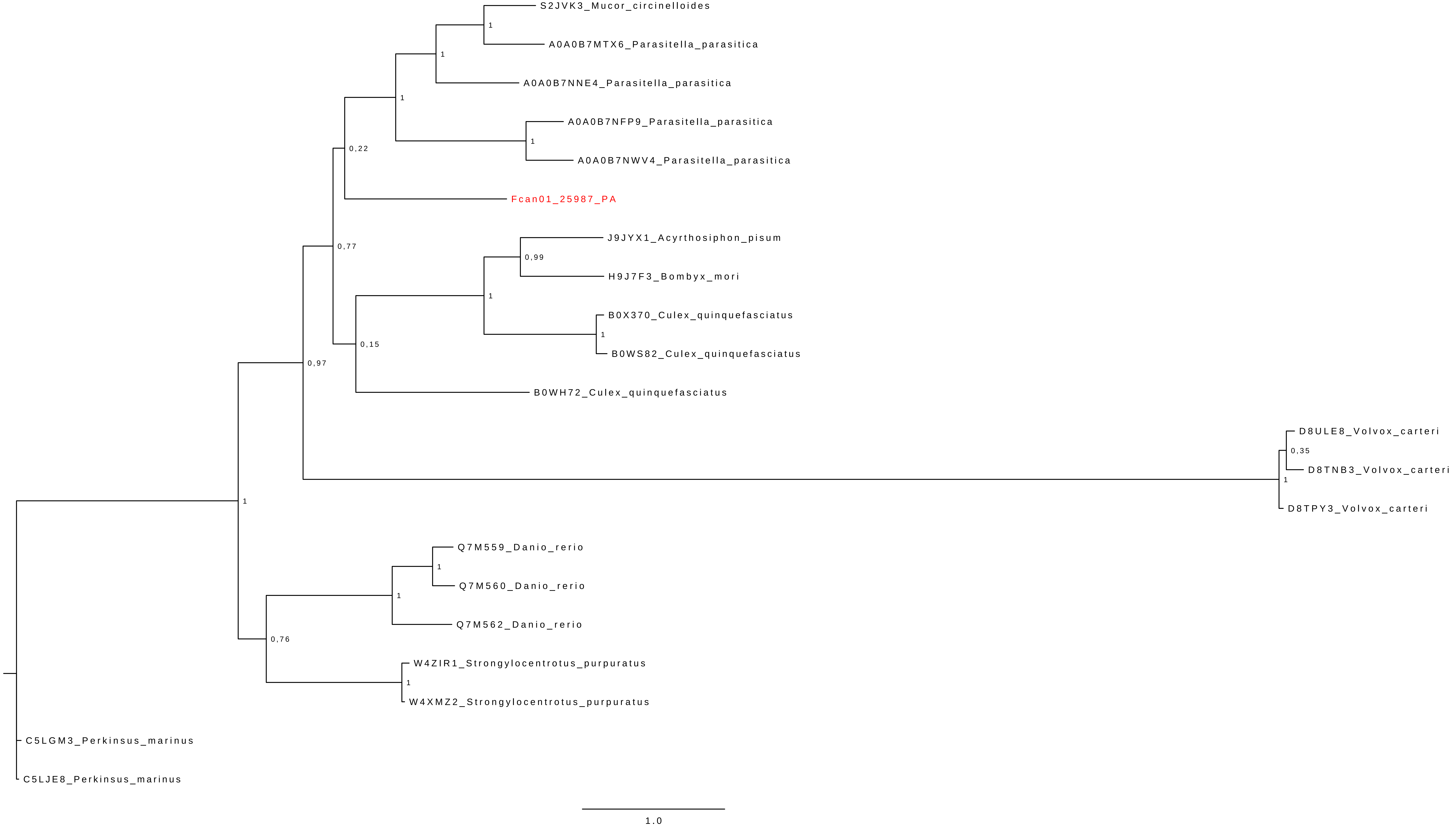
S2JVK3_Mucor_circinelloides
A0A0B7MTX6_Parasitella_parasitica
A0A0B7NNE4_Parasitella_parasitica
A0A0B7NFP9_Parasitella_parasitica
A0A0B7NWV4_Parasitella_parasitica
Fcan01_25987_PA
J9JYX1_Acyrthosiphon_pisum
H9J7F3_Bombyx_mori
B0X370_Culex_quinquefasciatus
B0WS82_Culex_quinquefasciatus
B0WH72_Culex_quinquefasciatus
D8ULE8_Volvox_carteri
D8TNB3_Volvox_carteri
D8TPY3_Volvox_carteri
Q7M559_Danio_rerio
Q7M560_Danio_rerio
Q7M562_Danio_rerio
W4ZIR1_Strongylocentrotus_purpuratus
W4XMZ2_Strongylocentrotus_purpuratus
C5LGM3_Perkinsus_marinus
C5LJE8_Perkinsus_marinus
1
1
1
0,22
1
0,77
0,99
1
1
0,15
0,97
0,35
1
1
1
1
0,76
1
1.0

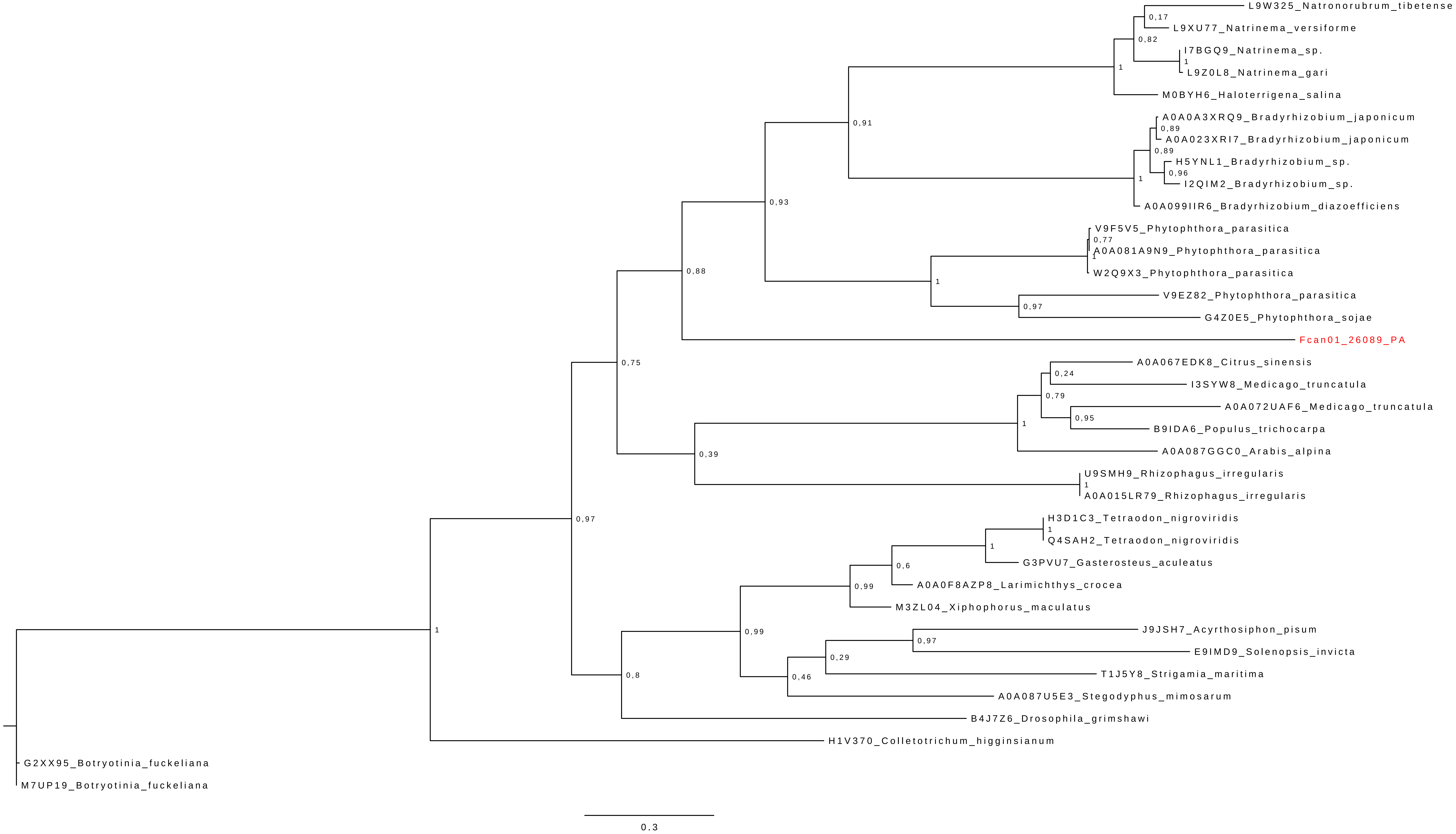

L9W325_Natronorubrum_tibetense
L9XU77_Natrinema_versiforme
I7BGQ9_Natrinema_sp.
L9Z0L8_Natrinema_gari
M0BYH6_Haloterrigena_salina
A0A0A3XRQ9_Bradyrhizobium_japonicum
A0A023XRI7_Bradyrhizobium_japonicum
H5YNL1_Bradyrhizobium_sp.
I2QIM2_Bradyrhizobium_sp.
A0A099IIR6_Bradyrhizobium_diazoefficiens
V9F5V5_Phytophthora_parasitica
A0A081A9N9_Phytophthora_parasitica
W2Q9X3_Phytophthora_parasitica
V9EZ82_Phytophthora_parasitica
G4Z0E5_Phytophthora_sojae
Fcan01_26089_PA
A0A067EDK8_Citrus_sinensis
I3SYW8_Medicago_truncatula
A0A072UAF6_Medicago_truncatula
B9IDA6_Populus_trichocarpa
A0A087GGC0_Arabis_alpina
U9SMH9_Rhizophagus_irregularis
A0A015LR79_Rhizophagus_irregularis
H3D1C3_Tetraodon_nigroviridis
Q4SAH2_Tetraodon_nigroviridis
G3PVU7_Gasterosteus_aculeatus
A0A0F8AZP8_Larimichthys_crocea
M3ZL04_Xiphophorus_maculatus
J9JSH7_Acyrthosiphon_pisum
E9IMD9_Solenopsis_invicta
T1J5Y8_Strigamia_maritima
A0A087U5E3_Stegodyphus_mimosarum
B4J7Z6_Drosophila_grimshawi
H1V370_Colletotrichum_higginsianum
G2XX95_Botryotinia_fuckeliana
M7UP19_Botryotinia_fuckeliana
0.3

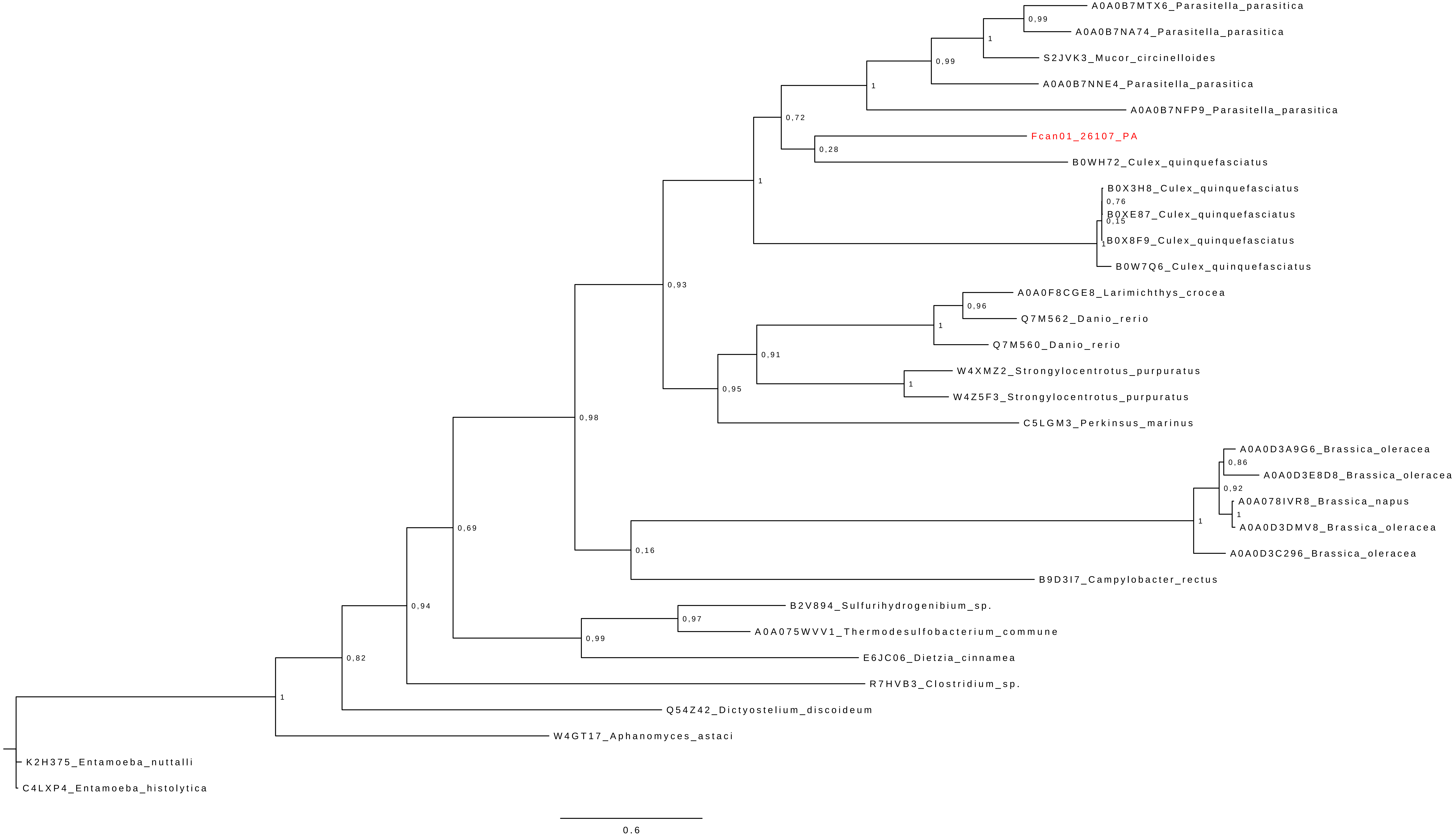
A0A0B7MTX6_Parasitella_parasitica
0,99
A0A0B7NA74_Parasitella_parasitica
1
S2JVK3_Mucor_circinelloides
0,99
A0A0B7NNE4_Parasitella_parasitica
1
A0A0B7NFP9_Parasitella_parasitica
0,72
Fcan01_26107_PA
0,28
B0WH72_Culex_quinquefasciatus
1
B0X3H8_Culex_quinquefasciatus
0,76
B0XE87_Culex_quinquefasciatus
0,15
B0X8F9_Culex_quinquefasciatus
1
B0W7Q6_Culex_quinquefasciatus
0,93
A0A0F8CGE8_Larimichthys_crocea
0,96
Q7M562_Danio_rerio
1
Q7M560_Danio_rerio
0,91
W4XMZ2_Strongylocentrotus_purpuratus
1
W4Z5F3_Strongylocentrotus_purpuratus
0,95
C5LGM3_Perkinsus_marinus
0,98
A0A0D3A9G6_Brassica_oleracea
0,86
A0A0D3E8D8_Brassica_oleracea
0,92
A0A078IVR8_Brassica_napus
1
A0A0D3DMV8_Brassica_oleracea
1
A0A0D3C296_Brassica_oleracea
0,16
B9D3I7_Campylobacter_rectus
0,69
B2V894_Sulfurihydrogenibium_sp.
0,97
A0A075WVV1_Thermodesulfobacterium_commune
0,99
E6JC06_Dietzia_cinnamea
0,94
R7HVB3_Clostridium_sp.
0,82
Q54Z42_Dictyostelium_discoideum
1
W4GT17_Aphanomyces_astaci
K2H375_Entamoeba_nuttalli
C4LXP4_Entamoeba_histolytica
0.6

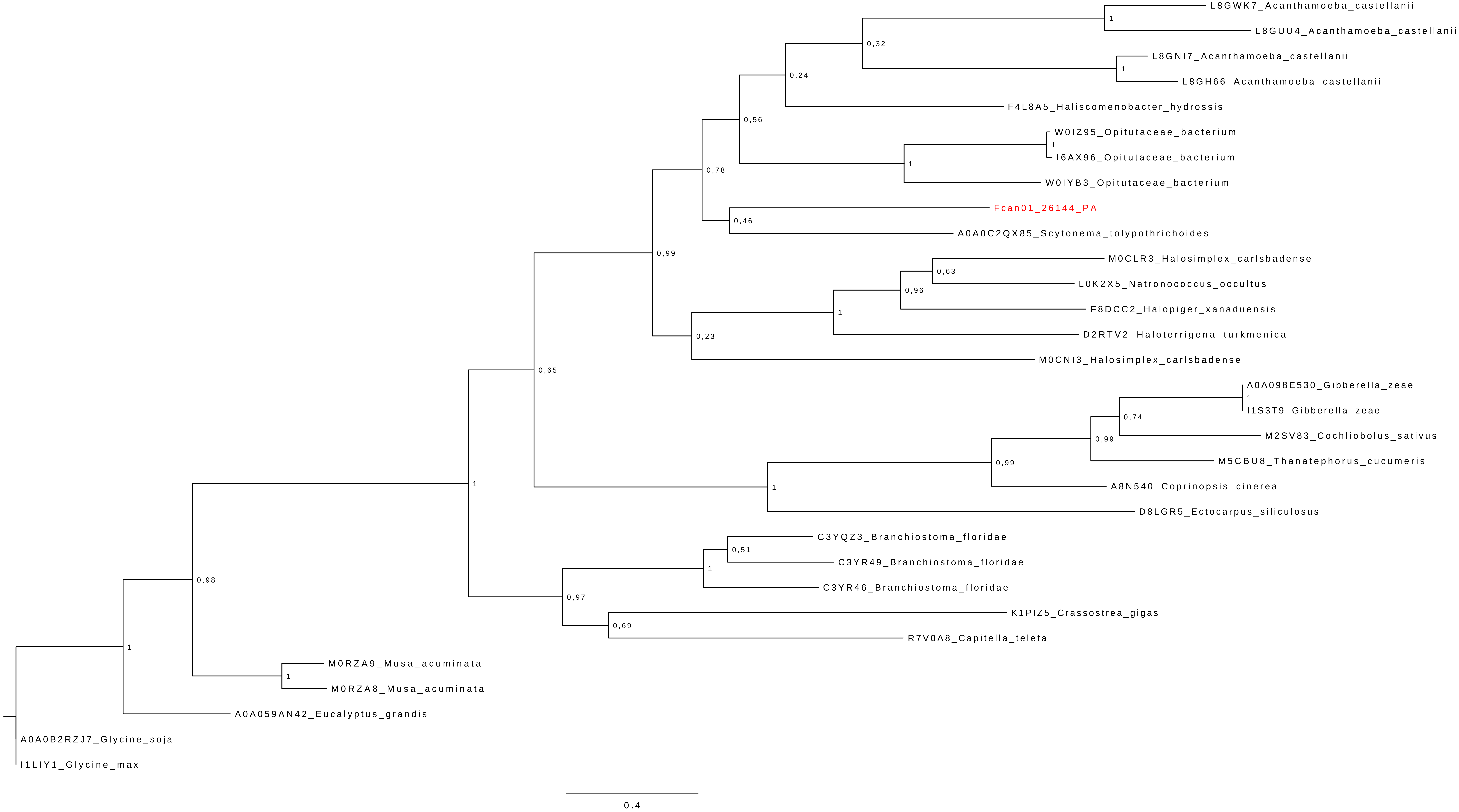
L8GWK7_Acanthamoeba_castellanii
L8GUU4_Acanthamoeba_castellanii
L8GNI7_Acanthamoeba_castellanii
L8GH66_Acanthamoeba_castellanii
F4L8A5_Haliscomenobacter_hydrossis
W0IZ95_Opitutaceae_bacterium
I6AX96_Opitutaceae_bacterium
W0IYB3_Opitutaceae_bacterium
Fcan01_26144_PA
A0A0C2QX85_Scytonema_tolypothrichoides
M0CLR3_Halosimplex_carlsbadense
L0K2X5_Natronococcus_occultus
F8DCC2_Halopiger_xanaduensis
D2RTV2_Haloterrigena_turkmenica
M0CNI3_Halosimplex_carlsbadense
A0A098E530_Gibberella_zeae
I1S3T9_Gibberella_zeae
M2SV83_Cochliobolus_sativus
M5CBU8_Thanatephorus_cucumeris
A8N540_Coprinopsis_cinerea
D8LGR5_Ectocarpus_siliculosus
C3YQZ3_Branchiostoma_floridae
C3YR49_Branchiostoma_floridae
C3YR46_Branchiostoma_floridae
K1PIZ5_Crassostrea_gigas
R7V0A8_Capitella_teleta
M0RZA9_Musa_acuminata
M0RZA8_Musa_acuminata
A0A059AN42_Eucalyptus_grandis
A0A0B2RZJ7_Glycine_soja
I1LIY1_Glycine_max
0.4

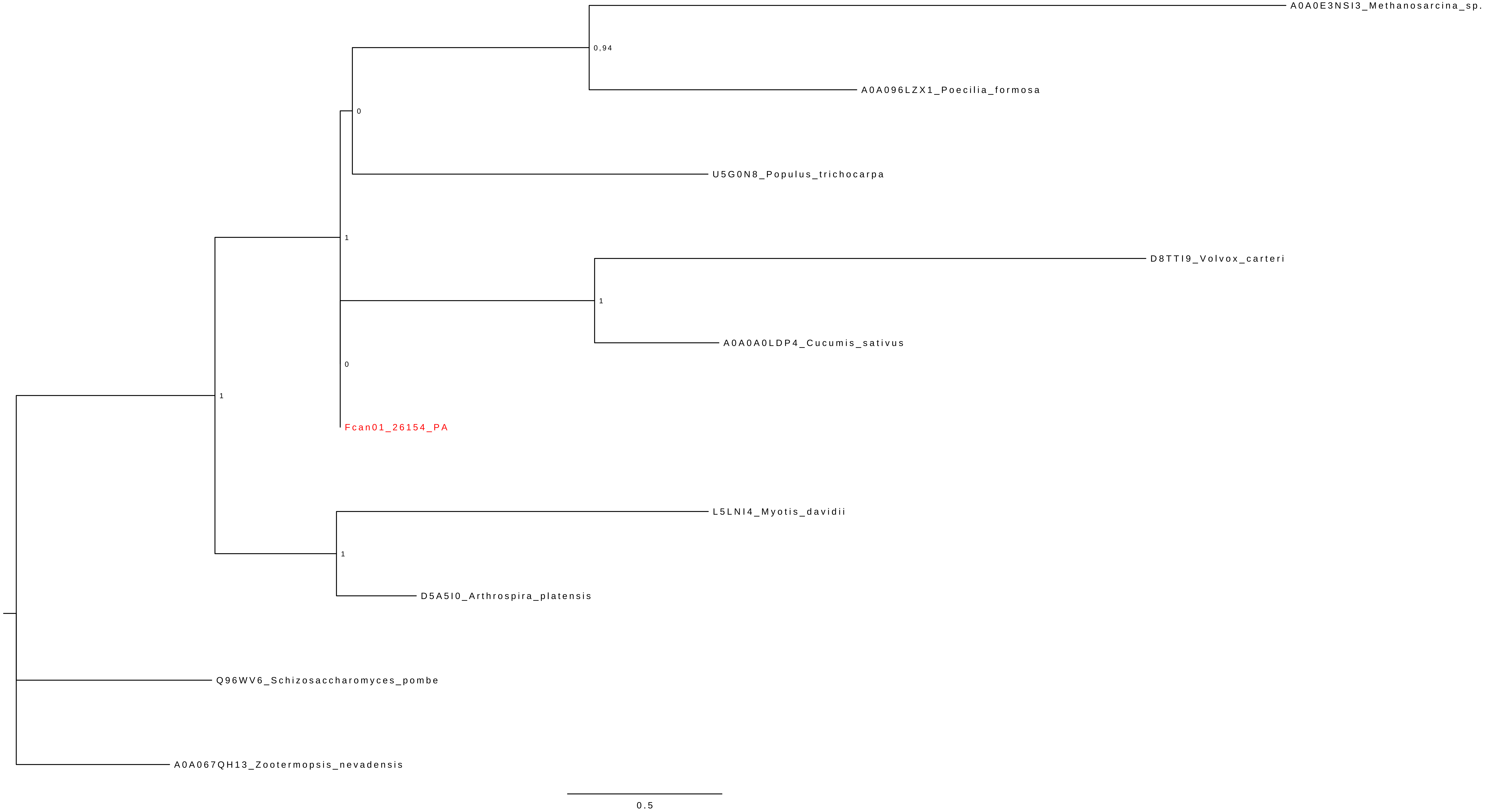
A0A0E3NSI3_Methanosarcina_sp.
0,94
A0A096LZX1_Poecilia_formosa
0
U5G0N8_Populus_trichocarpa
1
D8TTI9_Volvox_carteri
1
A0A0A0LDP4_Cucumis_sativus
0
1
Fcan01_26154_PA
L5LNI4_Myotis_davidii
1
D5A5I0_Arthrospira_platensis
Q96WV6_Schizosaccharomyces_pombe
A0A067QH13_Zootermopsis_nevadensis
0.5

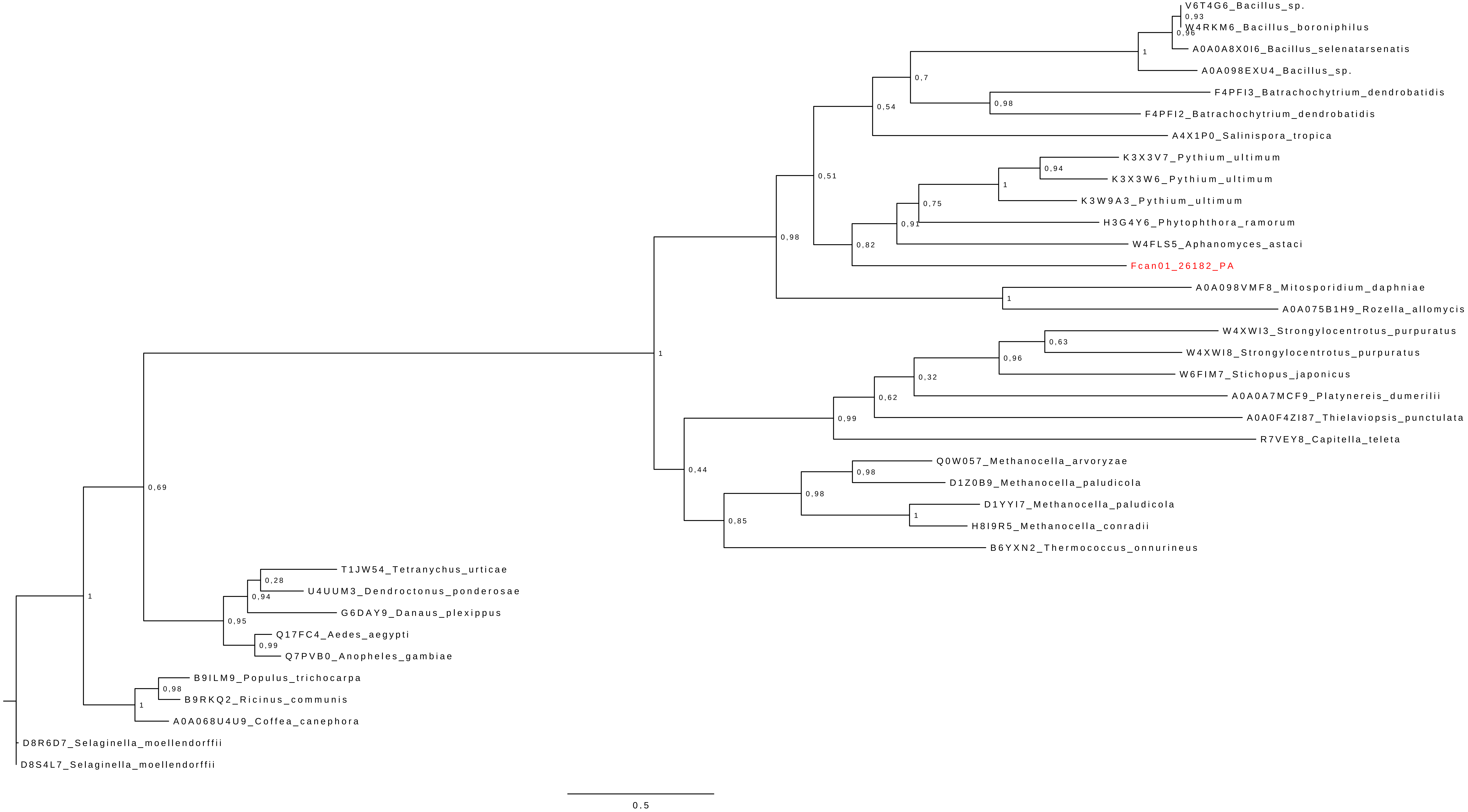
V6T4G6_Bacillus_sp.
W4RKM6_Bacillus_boroniphilus
A0A0A8X0I6_Bacillus_selenatarsenatis
A0A098EXU4_Bacillus_sp.
F4PFI3_Batrachochytrium_dendrobatidis
F4PFI2_Batrachochytrium_dendrobatidis
A4X1P0_Salinispora_tropica
K3X3V7_Pythium_ultimum
K3X3W6_Pythium_ultimum
K3W9A3_Pythium_ultimum
H3G4Y6_Phytophthora_ramorum
W4FLS5_Aphanomyces_astaci
Fcan01_26182_PA
A0A098VMF8_Mitosporidium_daphniae
A0A075B1H9_Rozella_allomycis
W4XWI3_Strongylocentrotus_purpuratus
W4XWI8_Strongylocentrotus_purpuratus
W6FIM7_Stichopus_japonicus
A0A0A7MCF9_Platynereis_dumerilii
A0A0F4ZI87_Thielaviopsis_punctulata
R7VEY8_Capitella_teleta
Q0W057_Methanocella_arvoryzae
D1Z0B9_Methanocella_paludicola
D1YYI7_Methanocella_paludicola
H8I9R5_Methanocella_conradii
B6YXN2_Thermococcus_onnurineus
T1JW54_Tetranychus_urticae
U4UUM3_Dendroctonus_ponderosae
G6DAY9_Danaus_plexippus
Q17FC4_Aedes_aegypti
Q7PVB0_Anopheles_gambiae
B9ILM9_Populus_trichocarpa
B9RKQ2_Ricinus_communis
A0A068U4U9_Coffea_canephora
D8R6D7_Selaginella_moellendorffii
D8S4L7_Selaginella_moellendorffii
0.5

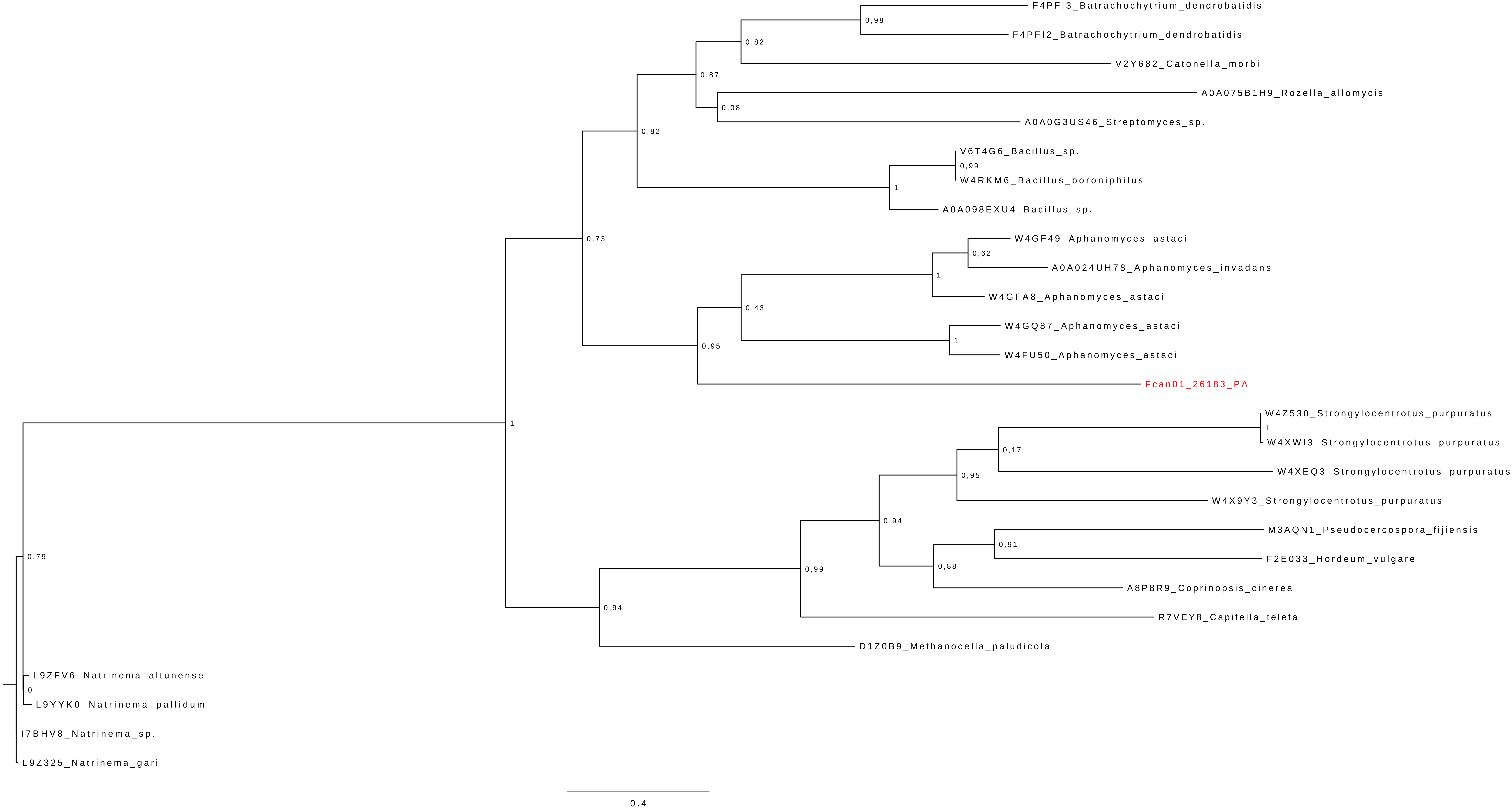
F4PFI3_Batrachochytrium_dendrobatidis
0,98
F4PFI2_Batrachochytrium_dendrobatidis
0,82
V2Y682_Catonella_morbi
0,87
A0A075B1H9_Rozella_allomycis
0,08
A0A0G3US46_Streptomyces_sp.
0,82
V6T4G6_Bacillus_sp.
0,99
W4RKM6_Bacillus_boroniphilus
1
A0A098EXU4_Bacillus_sp.
0,73
W4GF49_Aphanomyces_astaci
0,62
A0A024UH78_Aphanomyces_invadans
1
W4GFA8_Aphanomyces_astaci
0,43
W4GQ87_Aphanomyces_astaci
1
W4FU50_Aphanomyces_astaci
0,95
Fcan01_26183_PA
1
W4Z530_Strongylocentrotus_purpuratus
1
W4XWI3_Strongylocentrotus_purpuratus
0,17
W4XEQ3_Strongylocentrotus_purpuratus
0,95
W4X9Y3_Strongylocentrotus_purpuratus
0,94
M3AQN1_Pseudocercospora_fijiensis
0,91
F2E033_Hordeum_vulgare
0,88
A8P8R9_Coprinopsis_cinerea
0,99
R7VEY8_Capitella_teleta
0,94
D1Z0B9_Methanocella_paludicola
0,79
L9ZFV6_Natrinema_altunense
0
L9YYK0_Natrinema_pallidum
I7BHV8_Natrinema_sp.
L9Z325_Natrinema_gari
0.4

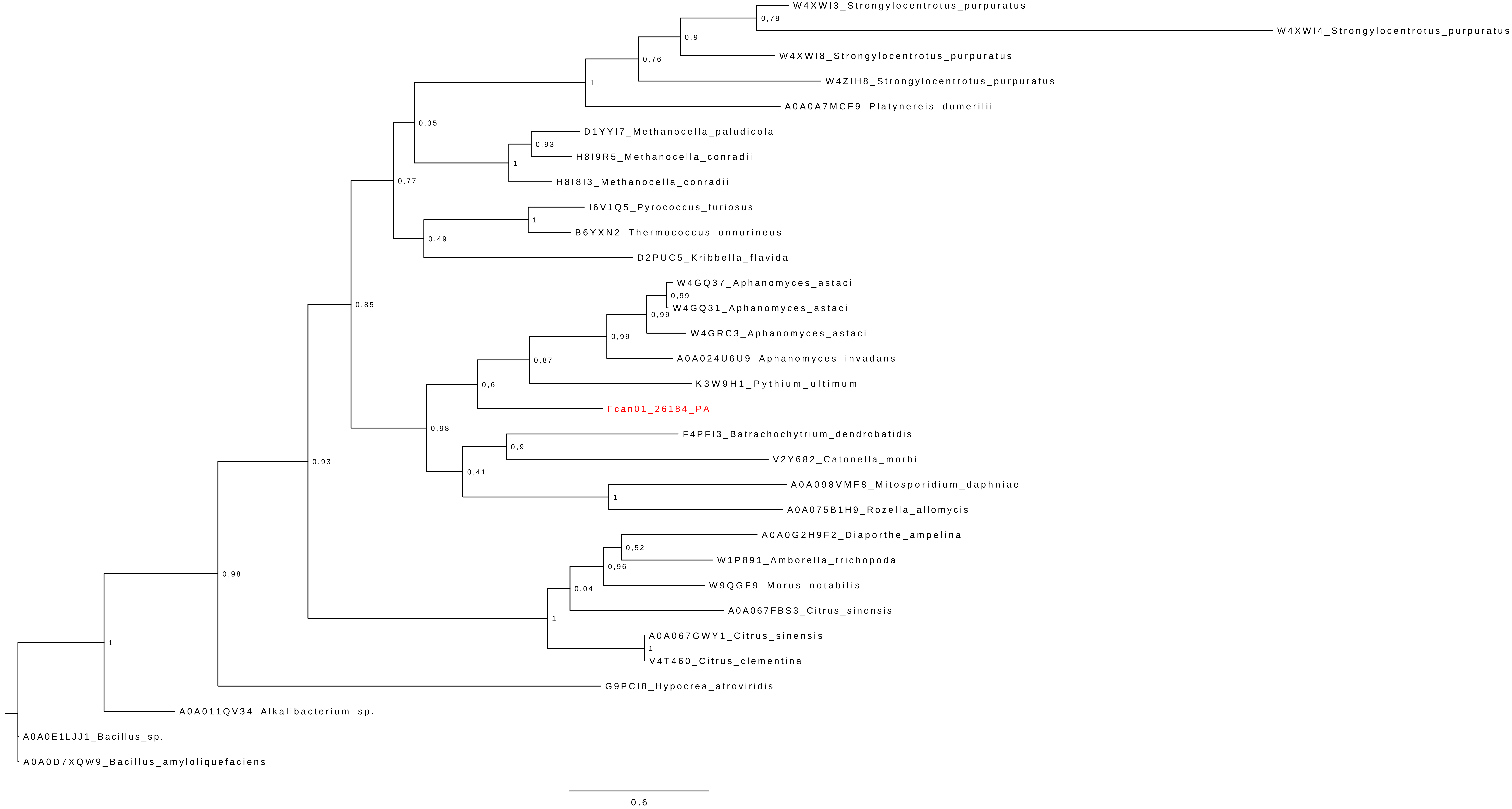
W4XWI3_Strongylocentrotus_purpuratus
0,78
W4XWI4_Strongylocentrotus_purpuratus
0,9
W4XWI8_Strongylocentrotus_purpuratus
0,76
W4ZIH8_Strongylocentrotus_purpuratus
1
A0A0A7MCF9_Platynereis_dumerilii
0,35
D1YYI7_Methanocella_paludicola
0,93
H8I9R5_Methanocella_conradii
1
H8I8I3_Methanocella_conradii
0,77
I6V1Q5_Pyrococcus_furiosus
1
B6YXN2_Thermococcus_onnurineus
0,49
D2PUC5_Kribbella_flavida
W4GQ37_Aphanomyces_astaci
0,99
W4GQ31_Aphanomyces_astaci
0,99
0,85
W4GRC3_Aphanomyces_astaci
0,99
A0A024U6U9_Aphanomyces_invadans
0,87
K3W9H1_Pythium_ultimum
0,6
Fcan01_26184_PA
0,98
F4PFI3_Batrachochytrium_dendrobatidis
0,9
V2Y682_Catonella_morbi
0,93
0,41
A0A098VMF8_Mitosporidium_daphniae
1
A0A075B1H9_Rozella_allomycis
A0A0G2H9F2_Diaporthe_ampelina
0,52
W1P891_Amborella_trichopoda
0,96
0,98
W9QGF9_Morus_notabilis
0,04
A0A067FBS3_Citrus_sinensis
1
A0A067GWY1_Citrus_sinensis
1
1
V4T460_Citrus_clementina
G9PCI8_Hypocrea_atroviridis
A0A011QV34_Alkalibacterium_sp.
A0A0E1LJJ1_Bacillus_sp.
A0A0D7XQW9_Bacillus_amyloliquefaciens
0.6

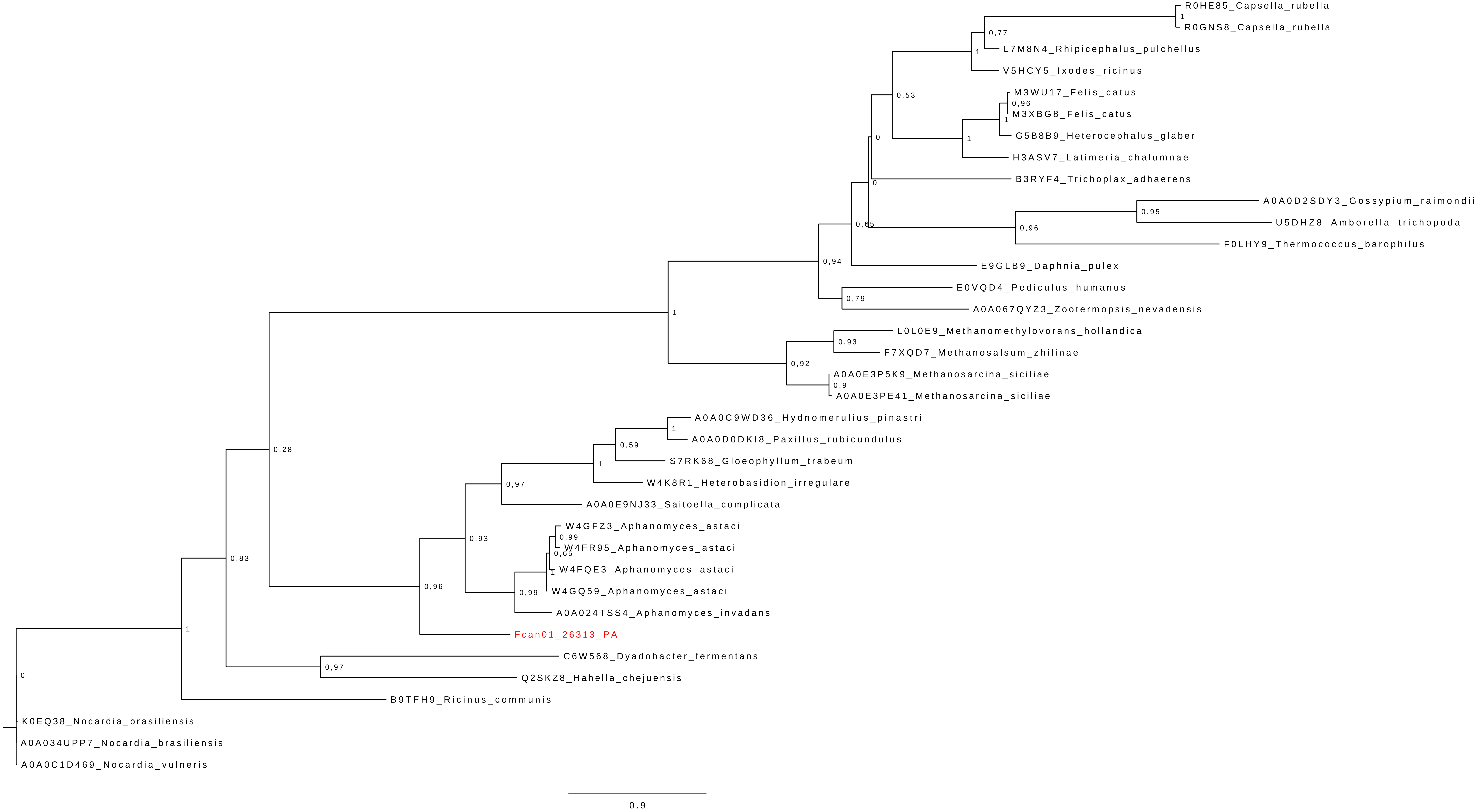
R0HE85_Capsella_rubella
R0GNS8_Capsella_rubella
L7M8N4_Rhipicephalus_pulchellus
V5HCY5_Ixodes_ricinus
M3WU17_Felis_catus
M3XBG8_Felis_catus
G5B8B9_Heterocephalus_glaber
H3ASV7_Latimeria_chalumnae
B3RYF4_Trichoplax_adhaerens
A0A0D2SDY3_Gossypium_raimondii
U5DHZ8_Amborella_trichopoda
F0LHY9_Thermococcus_barophilus
E9GLB9_Daphnia_pulex
E0VQD4_Pediculus_humanus
A0A067QYZ3_Zootermopsis_nevadensis
L0L0E9_Methanomethylovorans_hollandica
F7XQD7_Methanosalsum_zhilinae
A0A0E3P5K9_Methanosarcina_siciliae
A0A0E3PE41_Methanosarcina_siciliae
A0A0C9WD36_Hydnomerulius_pinastri
A0A0D0DKI8_Paxillus_rubicundulus
S7RK68_Gloeophyllum_trabeum
W4K8R1_Heterobasidion_irregulare
A0A0E9NJ33_Saitoella_complicata
W4GFZ3_Aphanomyces_astaci
W4FR95_Aphanomyces_astaci
W4FQE3_Aphanomyces_astaci
W4GQ59_Aphanomyces_astaci
A0A024TSS4_Aphanomyces_invadans
Fcan01_26313_PA
C6W568_Dyadobacter_fermentans
Q2SKZ8_Hahella_chejuensis
B9TFH9_Ricinus_communis
K0EQ38_Nocardia_brasiliensis
A0A034UPP7_Nocardia_brasiliensis
A0A0C1D469_Nocardia_vulneris
1
0,77
1
0,96
1
1
0
0
0,53
0,95
0,96
0,65
0,94
0,79
1
0,93
0,92
0,9
1
0,59
1
0,97
0,99
0,65
1
0,93
0,99
0,96
0,28
0,83
1
0,97
0
0.9

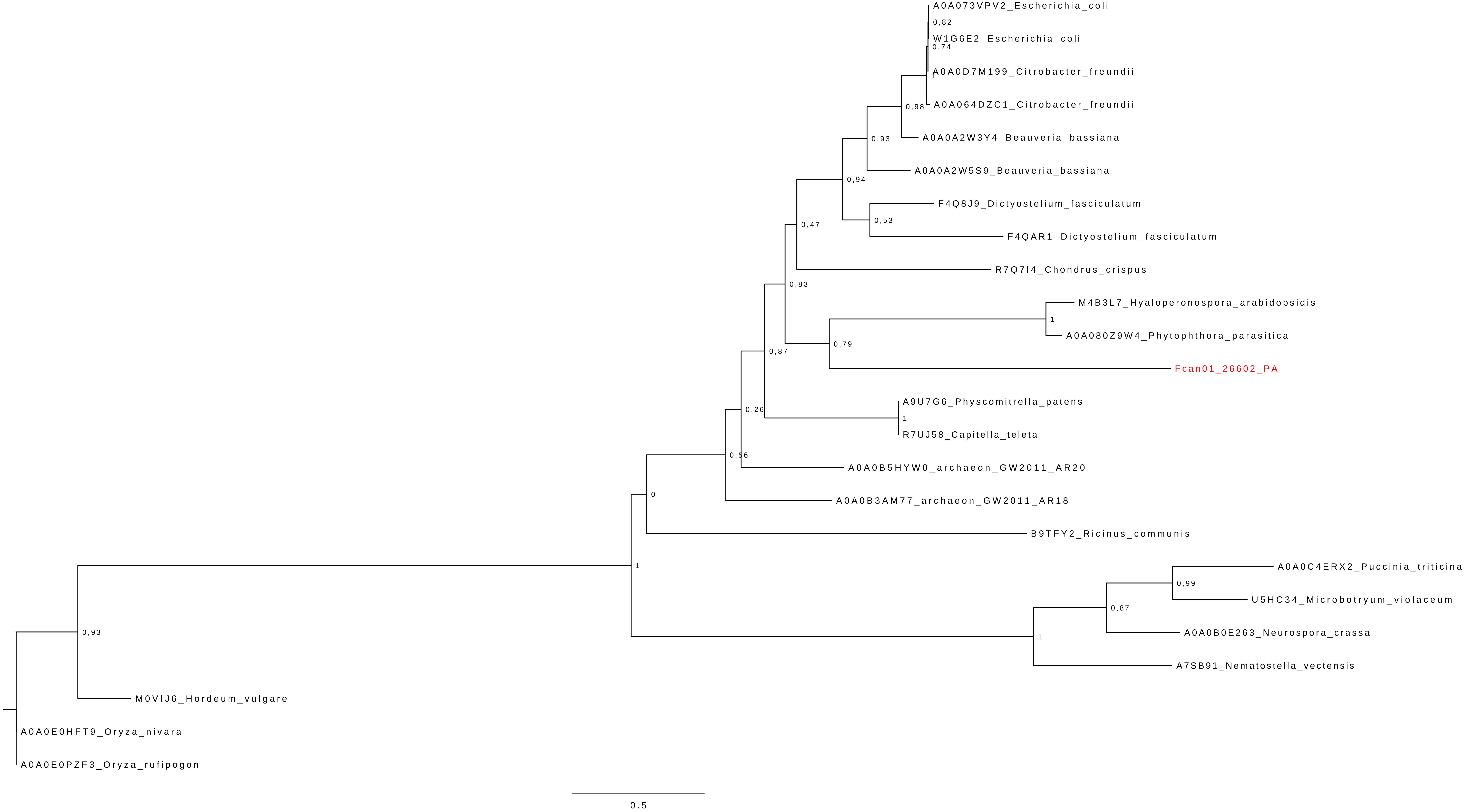
A0A073VPV2_Escherichia_coli
0,82
W1G6E2_Escherichia_coli
0,74
A0A0D7M199_Citrobacter_freundii
1
A0A064DZC1_Citrobacter_freundii
0,98
A0A0A2W3Y4_Beauveria_bassiana
0,93
A0A0A2W5S9_Beauveria_bassiana
0,94
F4Q8J9_Dictyostelium_fasciculatum
0,53
F4QAR1_Dictyostelium_fasciculatum
0,47
R7Q7I4_Chondrus_crispus
0,83
M4B3L7_Hyaloperonospora_arabidopsidis
1
A0A080Z9W4_Phytophthora_parasitica
0,79
Fcan01_26602_PA
0,87
A9U7G6_Physcomitrella_patens
1
R7UJ58_Capitella_teleta
0,26
0,56
A0A0B5HYW0_archaeon_GW2011_AR20
A0A0B3AM77_archaeon_GW2011_AR18
0
B9TFY2_Ricinus_communis
1
A0A0C4ERX2_Puccinia_triticina
0,99
U5HC34_Microbotryum_violaceum
0,87
A0A0B0E263_Neurospora_crassa
1
A7SB91_Nematostella_vectensis
0,93
M0VIJ6_Hordeum_vulgare
A0A0E0HFT9_Oryza_nivara
A0A0E0PZF3_Oryza_rufipogon
0.5

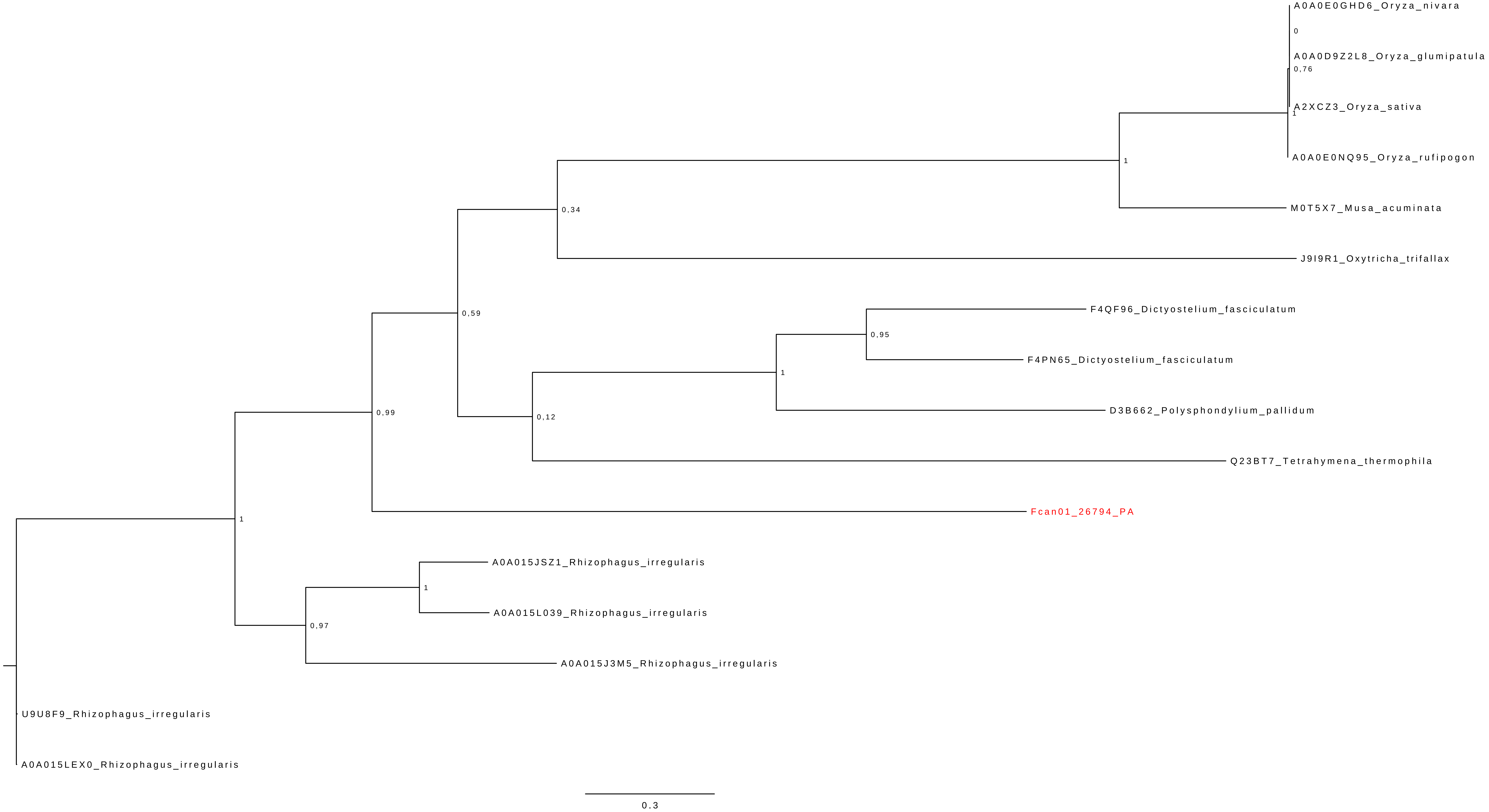
A0A0E0GHD6_Oryza_nivara
0
A0A0D9Z2L8_Oryza_glumipatula
0,76
A2XCZ3_Oryza_sativa
1
A0A0E0NQ95_Oryza_rufipogon
1
M0T5X7_Musa_acuminata
0,34
J9I9R1_Oxytricha_trifallax
F4QF96_Dictyostelium_fasciculatum
0,95
F4PN65_Dictyostelium_fasciculatum
1
D3B662_Polysphondylium_pallidum
0,59
0,12
Q23BT7_Tetrahymena_thermophila
0,99
Fcan01_26794_PA
1
A0A015JSZ1_Rhizophagus_irregularis
1
A0A015L039_Rhizophagus_irregularis
0,97
A0A015J3M5_Rhizophagus_irregularis
U9U8F9_Rhizophagus_irregularis
A0A015LEX0_Rhizophagus_irregularis
0.3

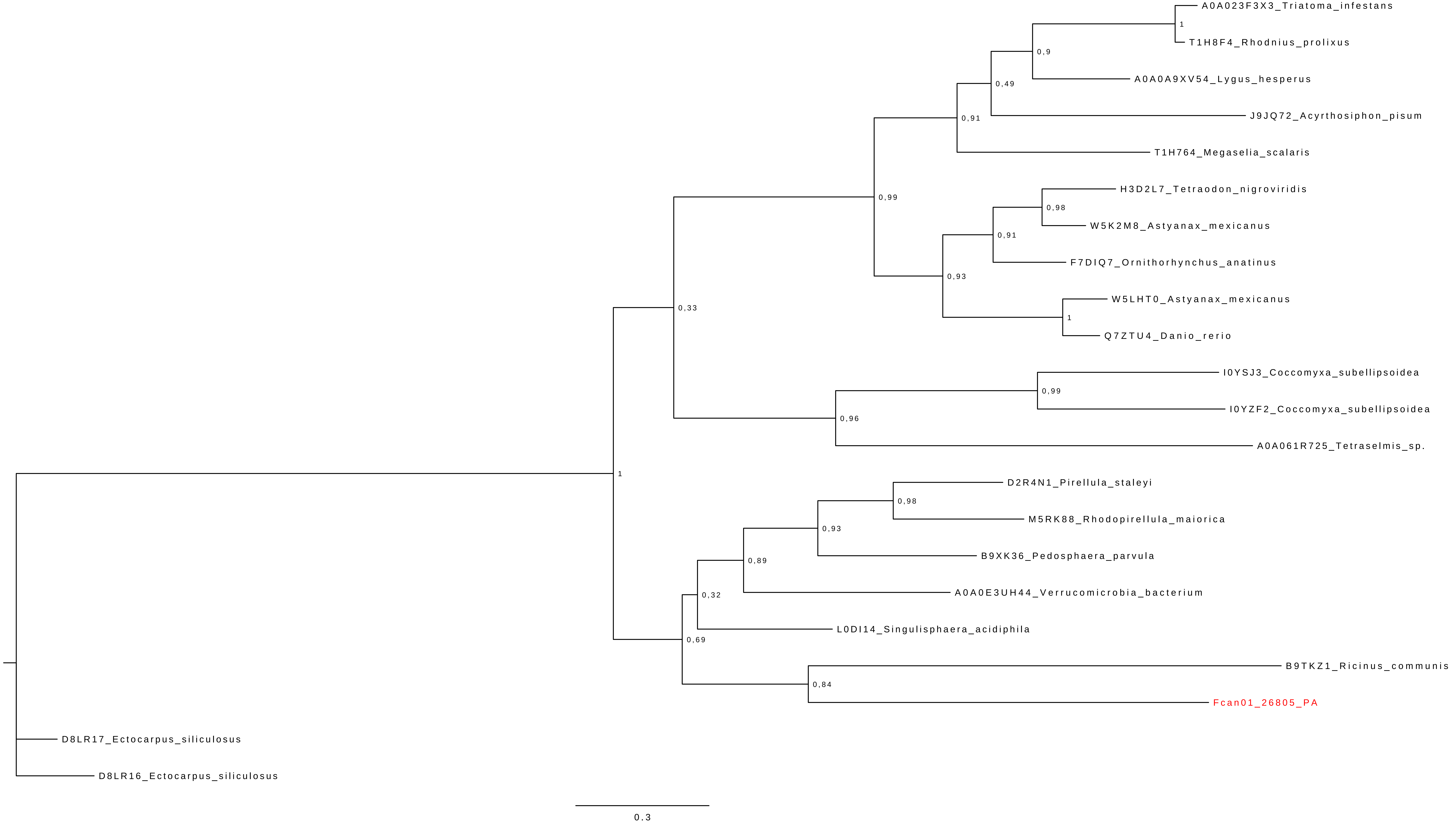
A0A023F3X3_Triatoma_infestans
1
T1H8F4_Rhodnius_prolixus
0,9
A0A0A9XV54_Lygus_hesperus
0,49
J9JQ72_Acyrthosiphon_pisum
0,91
T1H764_Megaselia_scalaris
H3D2L7_Tetraodon_nigroviridis
0,99
0,98
W5K2M8_Astyanax_mexicanus
0,91
F7DIQ7_Ornithorhynchus_anatinus
0,93
W5LHT0_Astyanax_mexicanus
0,33
1
Q7ZTU4_Danio_rerio
I0YSJ3_Coccomyxa_subellipsoidea
0,99
I0YZF2_Coccomyxa_subellipsoidea
0,96
A0A061R725_Tetraselmis_sp.
1
D2R4N1_Pirellula_staleyi
0,98
M5RK88_Rhodopirellula_maiorica
0,93
B9XK36_Pedosphaera_parvula
0,89
0,32
A0A0E3UH44_Verrucomicrobia_bacterium
L0DI14_Singulisphaera_acidiphila
0,69
B9TKZ1_Ricinus_communis
0,84
Fcan01_26805_PA
D8LR17_Ectocarpus_siliculosus
D8LR16_Ectocarpus_siliculosus
0.3

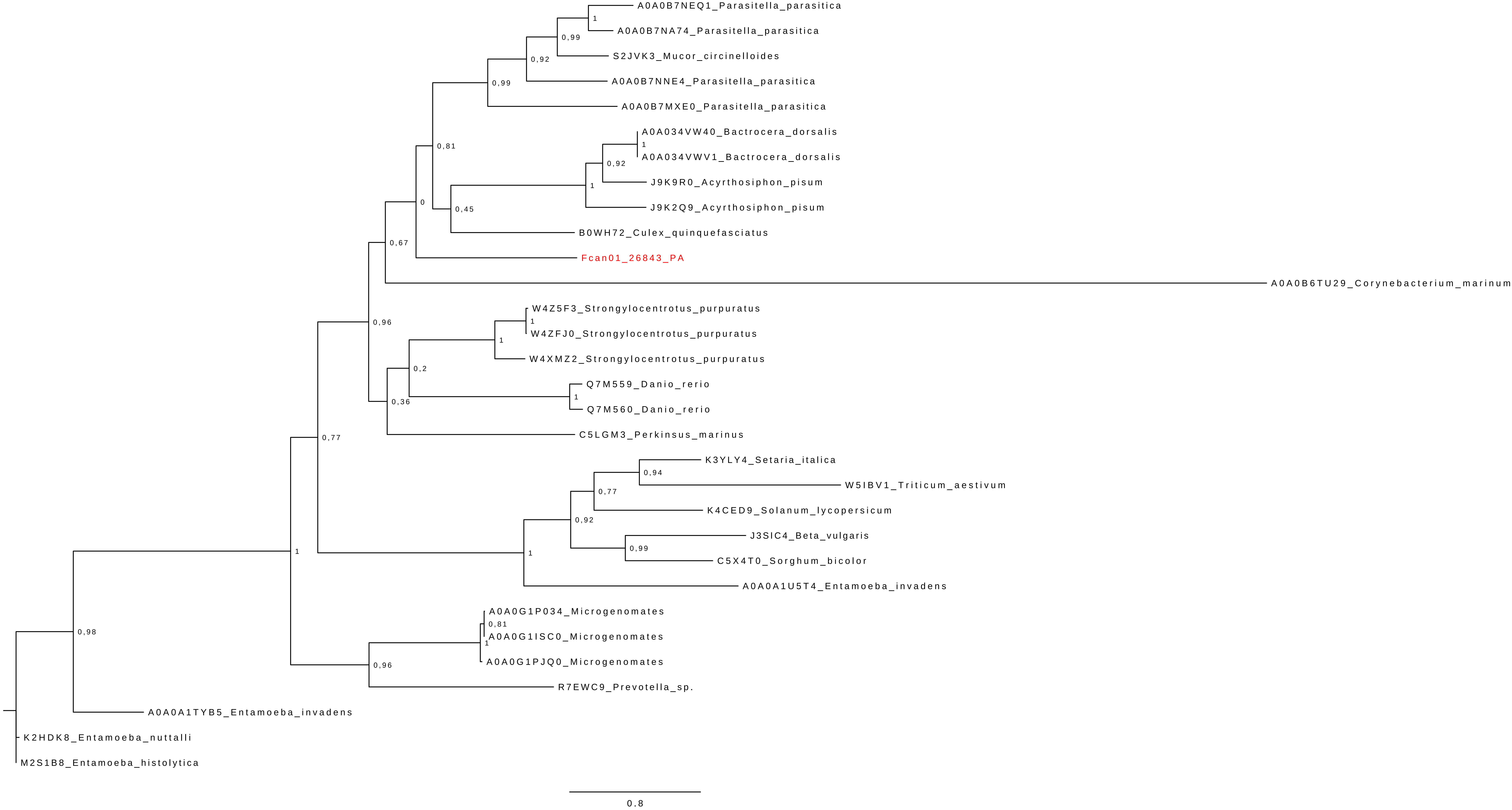
A0A0B7NEQ1_Parasitella_parasitica
1
A0A0B7NA74_Parasitella_parasitica
0,99
S2JVK3_Mucor_circinelloides
0,92
A0A0B7NNE4_Parasitella_parasitica
0,99
A0A0B7MXE0_Parasitella_parasitica
A0A034VW40_Bactrocera_dorsalis
1
A0A034VWV1_Bactrocera_dorsalis
0,81
0,92
J9K9R0_Acyrthosiphon_pisum
1
0
0,45
J9K2Q9_Acyrthosiphon_pisum
B0WH72_Culex_quinquefasciatus
0,67
Fcan01_26843_PA
A0A0B6TU29_Corynebacterium_marinum
W4Z5F3_Strongylocentrotus_purpuratus
0,96
1
W4ZFJ0_Strongylocentrotus_purpuratus
1
W4XMZ2_Strongylocentrotus_purpuratus
0,2
Q7M559_Danio_rerio
1
0,36
Q7M560_Danio_rerio
0,77
C5LGM3_Perkinsus_marinus
K3YLY4_Setaria_italica
0,94
W5IBV1_Triticum_aestivum
0,77
K4CED9_Solanum_lycopersicum
0,92
J3SIC4_Beta_vulgaris
1
1
0,99
C5X4T0_Sorghum_bicolor
A0A0A1U5T4_Entamoeba_invadens
A0A0G1P034_Microgenomates
0,81
A0A0G1ISC0_Microgenomates
0,98
1
0,96
A0A0G1PJQ0_Microgenomates
R7EWC9_Prevotella_sp.
A0A0A1TYB5_Entamoeba_invadens
K2HDK8_Entamoeba_nuttalli
M2S1B8_Entamoeba_histolytica
0.8

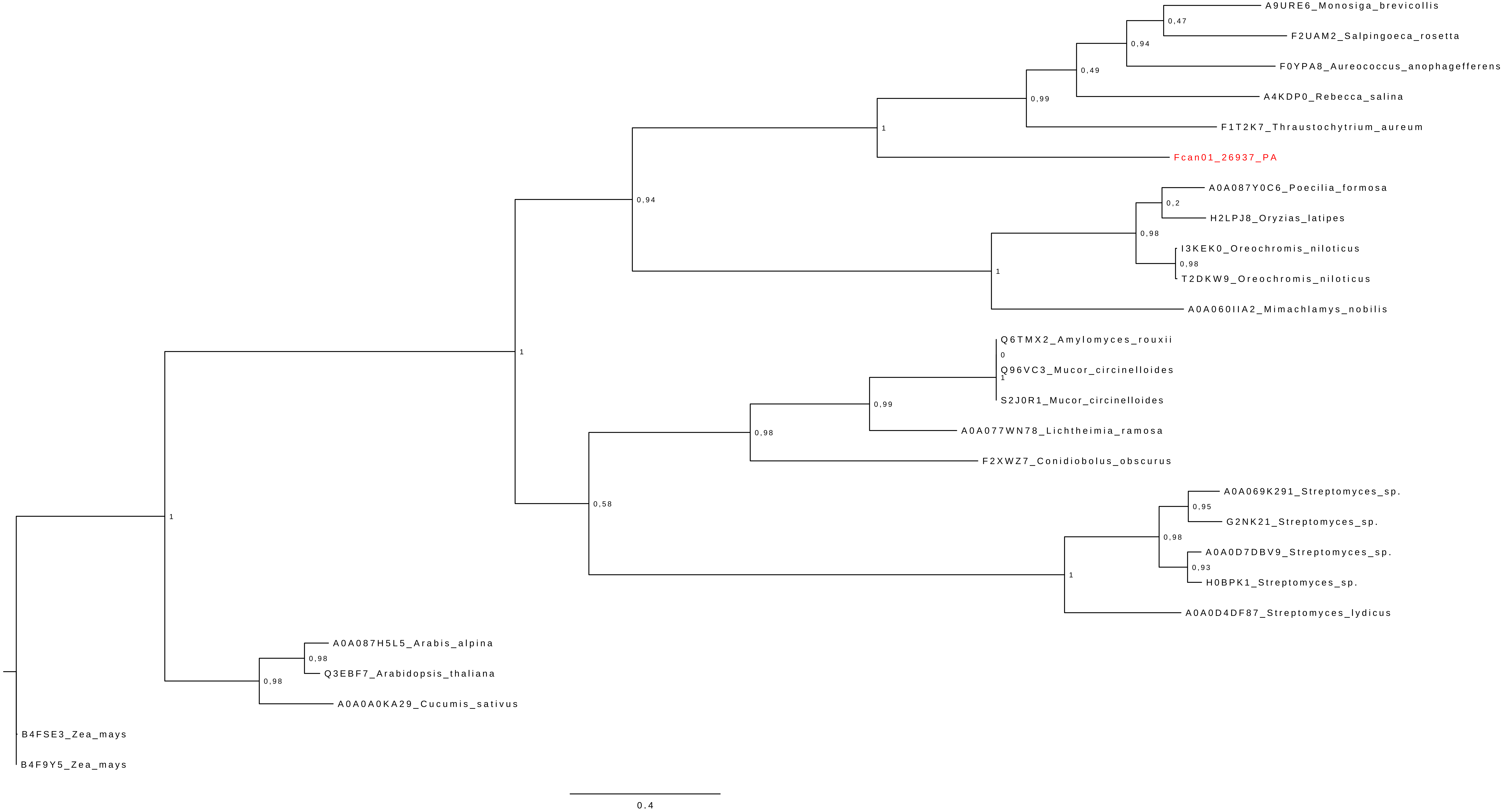

A9URE6_Monosiga_brevicollis
0,47
F2UAM2_Salpingoeca_rosetta
0,94
F0YPA8_Aureococcus_anophagefferens
0,49
A4KDP0_Rebecca_salina
0,99
F1T2K7_Thraustochytrium_aureum
1
Fcan01_26937_PA
0,94
A0A087Y0C6_Poecilia_formosa
0,2
H2LPJ8_Oryzias_latipes
0,98
I3KEK0_Oreochromis_niloticus
0,98
T2DKW9_Oreochromis_niloticus
1
A0A060IIA2_Mimachlamys_nobilis
1
Q6TMX2_Amylomyces_rouxii
0
Q96VC3_Mucor_circinelloides
1
S2J0R1_Mucor_circinelloides
0,99
A0A077WN78_Lichtheimia_ramosa
0,98
F2XWZ7_Conidiobolus_obscurus
A0A069K291_Streptomyces_sp.
0,95
G2NK21_Streptomyces_sp.
0,58
0,98
A0A0D7DBV9_Streptomyces_sp.
0,93
H0BPK1_Streptomyces_sp.
1
A0A0D4DF87_Streptomyces_lydicus
1
A0A087H5L5_Arabis_alpina
0,98
Q3EBF7_Arabidopsis_thaliana
0,98
A0A0A0KA29_Cucumis_sativus
B4FSE3_Zea_mays
B4F9Y5_Zea_mays
0.4

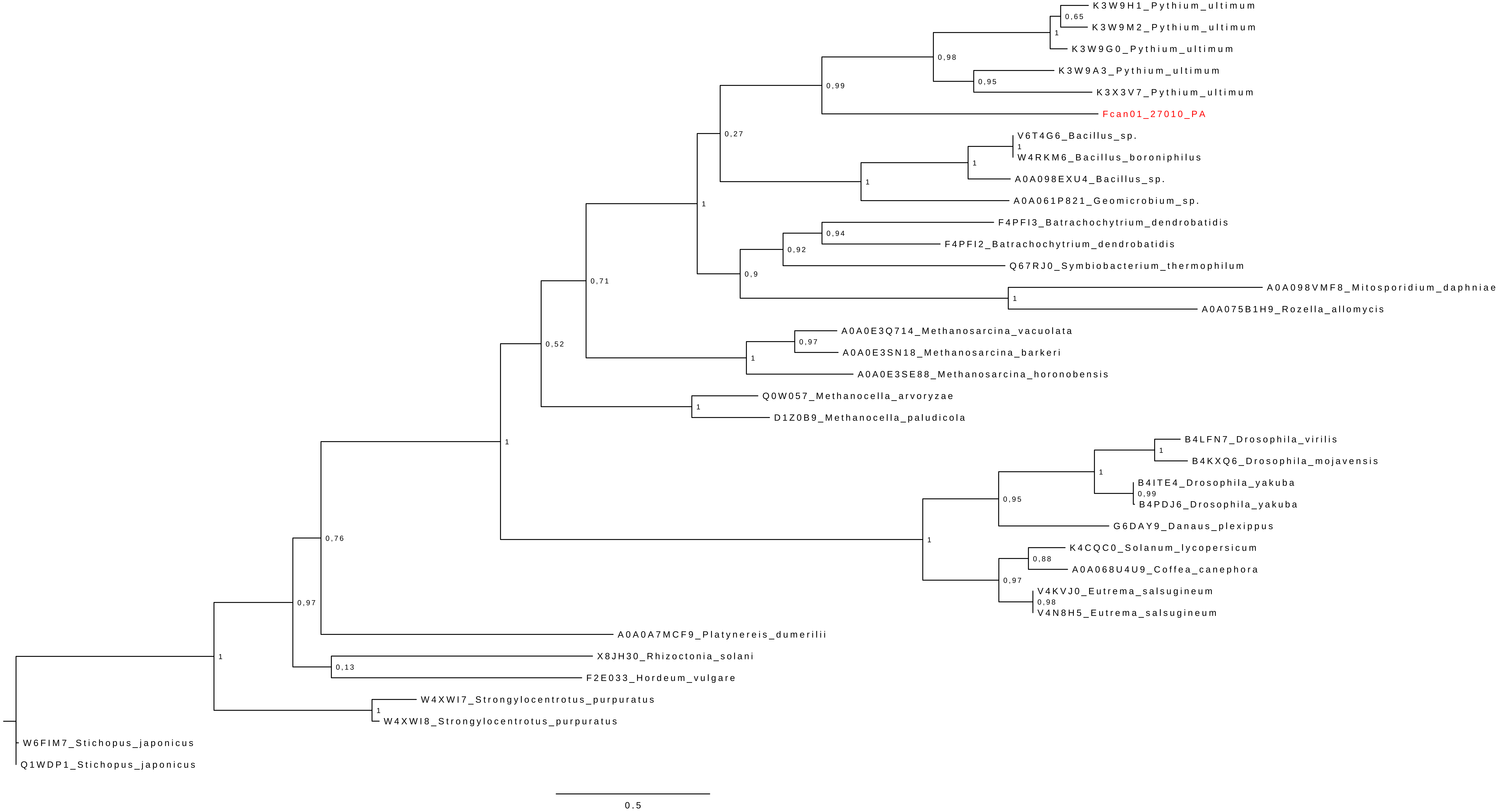
K3W9H1_Pythium_ultimum
0,65
K3W9M2_Pythium_ultimum
1
K3W9G0_Pythium_ultimum
0,98
K3W9A3_Pythium_ultimum
0,95
K3X3V7_Pythium_ultimum
0,99
Fcan01_27010_PA
0,27
V6T4G6_Bacillus_sp.
1
W4RKM6_Bacillus_boroniphilus
1
A0A098EXU4_Bacillus_sp.
1
A0A061P821_Geomicrobium_sp.
1
F4PFI3_Batrachochytrium_dendrobatidis
0,94
F4PFI2_Batrachochytrium_dendrobatidis
0,92
Q67RJ0_Symbiobacterium_thermophilum
0,9
A0A098VMF8_Mitosporidium_daphniae
1
A0A075B1H9_Rozella_allomycis
0,71
A0A0E3Q714_Methanosarcina_vacuolata
0,97
A0A0E3SN18_Methanosarcina_barkeri
1
A0A0E3SE88_Methanosarcina_horonobensis
0,52
Q0W057_Methanocella_arvoryzae
1
D1Z0B9_Methanocella_paludicola
1
B4LFN7_Drosophila_virilis
1
B4KXQ6_Drosophila_mojavensis
1
B4ITE4_Drosophila_yakuba
0,99
B4PDJ6_Drosophila_yakuba
0,95
G6DAY9_Danaus_plexippus
1
K4CQC0_Solanum_lycopersicum
0,88
A0A068U4U9_Coffea_canephora
0,97
V4KVJ0_Eutrema_salsugineum
0,98
V4N8H5_Eutrema_salsugineum
0,76
A0A0A7MCF9_Platynereis_dumerilii
0,97
X8JH30_Rhizoctonia_solani
0,13
F2E033_Hordeum_vulgare
1
W4XWI7_Strongylocentrotus_purpuratus
1
W4XWI8_Strongylocentrotus_purpuratus
W6FIM7_Stichopus_japonicus
Q1WDP1_Stichopus_japonicus
0.5

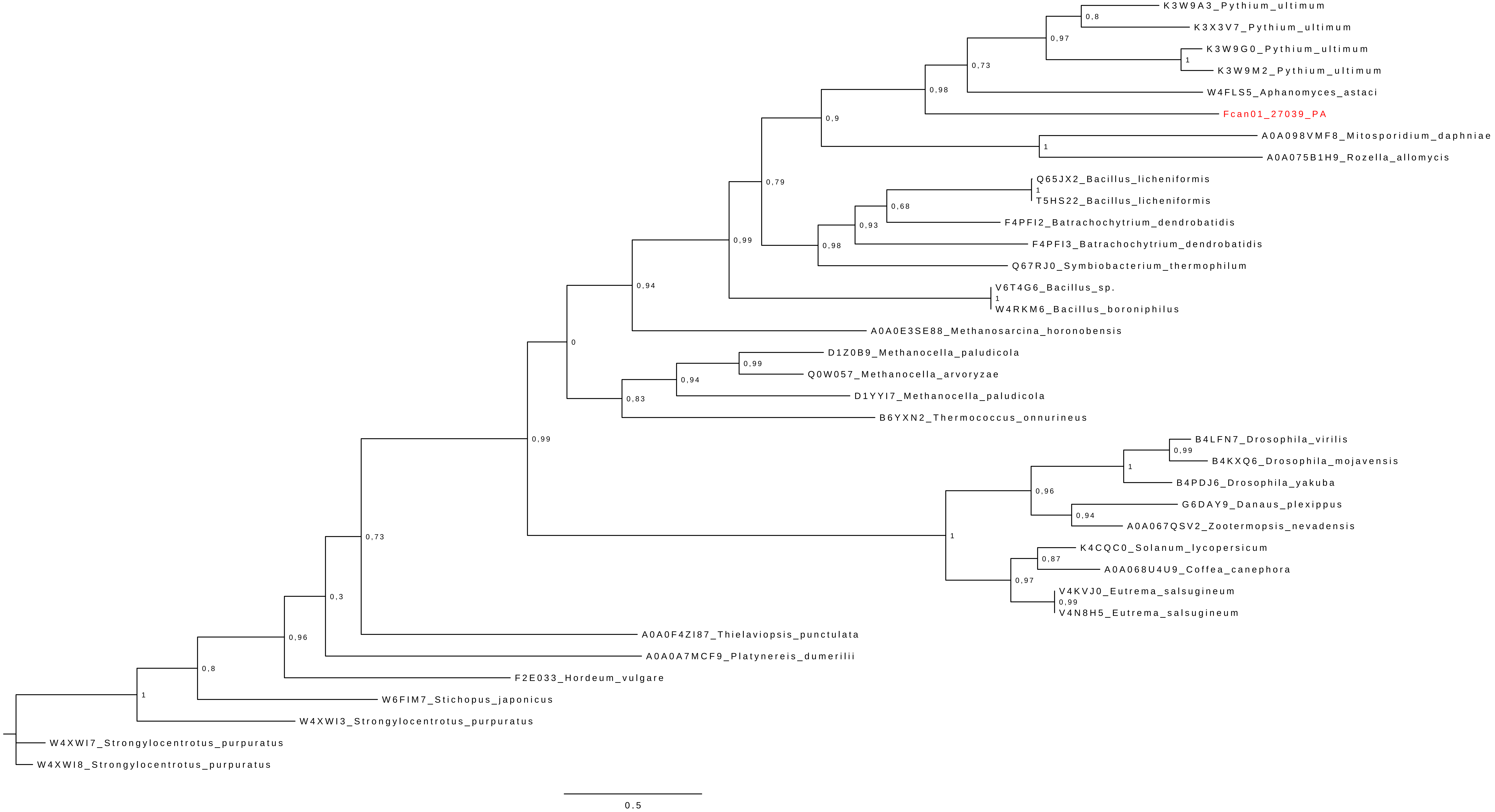
K3W9A3_Pythium_ultimum
0,8
K3X3V7_Pythium_ultimum
0,97
K3W9G0_Pythium_ultimum
1
K3W9M2_Pythium_ultimum
0,73
W4FLS5_Aphanomyces_astaci
0,98
Fcan01_27039_PA
0,9
A0A098VMF8_Mitosporidium_daphniae
1
A0A075B1H9_Rozella_allomycis
0,79
Q65JX2_Bacillus_licheniformis
1
T5HS22_Bacillus_licheniformis
0,68
F4PFI2_Batrachochytrium_dendrobatidis
0,93
F4PFI3_Batrachochytrium_dendrobatidis
0,98
Q67RJ0_Symbiobacterium_thermophilum
0,99
V6T4G6_Bacillus_sp.
1
W4RKM6_Bacillus_boroniphilus
0,94
A0A0E3SE88_Methanosarcina_horonobensis
0
D1Z0B9_Methanocella_paludicola
0,99
Q0W057_Methanocella_arvoryzae
0,94
D1YYI7_Methanocella_paludicola
0,83
B6YXN2_Thermococcus_onnurineus
0,99
B4LFN7_Drosophila_virilis
0,99
B4KXQ6_Drosophila_mojavensis
1
B4PDJ6_Drosophila_yakuba
0,96
G6DAY9_Danaus_plexippus
0,94
A0A067QSV2_Zootermopsis_nevadensis
1
K4CQC0_Solanum_lycopersicum
0,87
A0A068U4U9_Coffea_canephora
0,97
V4KVJ0_Eutrema_salsugineum
0,99
V4N8H5_Eutrema_salsugineum
0,73
A0A0F4ZI87_Thielaviopsis_punctulata
0,3
A0A0A7MCF9_Platynereis_dumerilii
0,96
F2E033_Hordeum_vulgare
0,8
W6FIM7_Stichopus_japonicus
1
W4XWI3_Strongylocentrotus_purpuratus
W4XWI7_Strongylocentrotus_purpuratus
W4XWI8_Strongylocentrotus_purpuratus
0.5

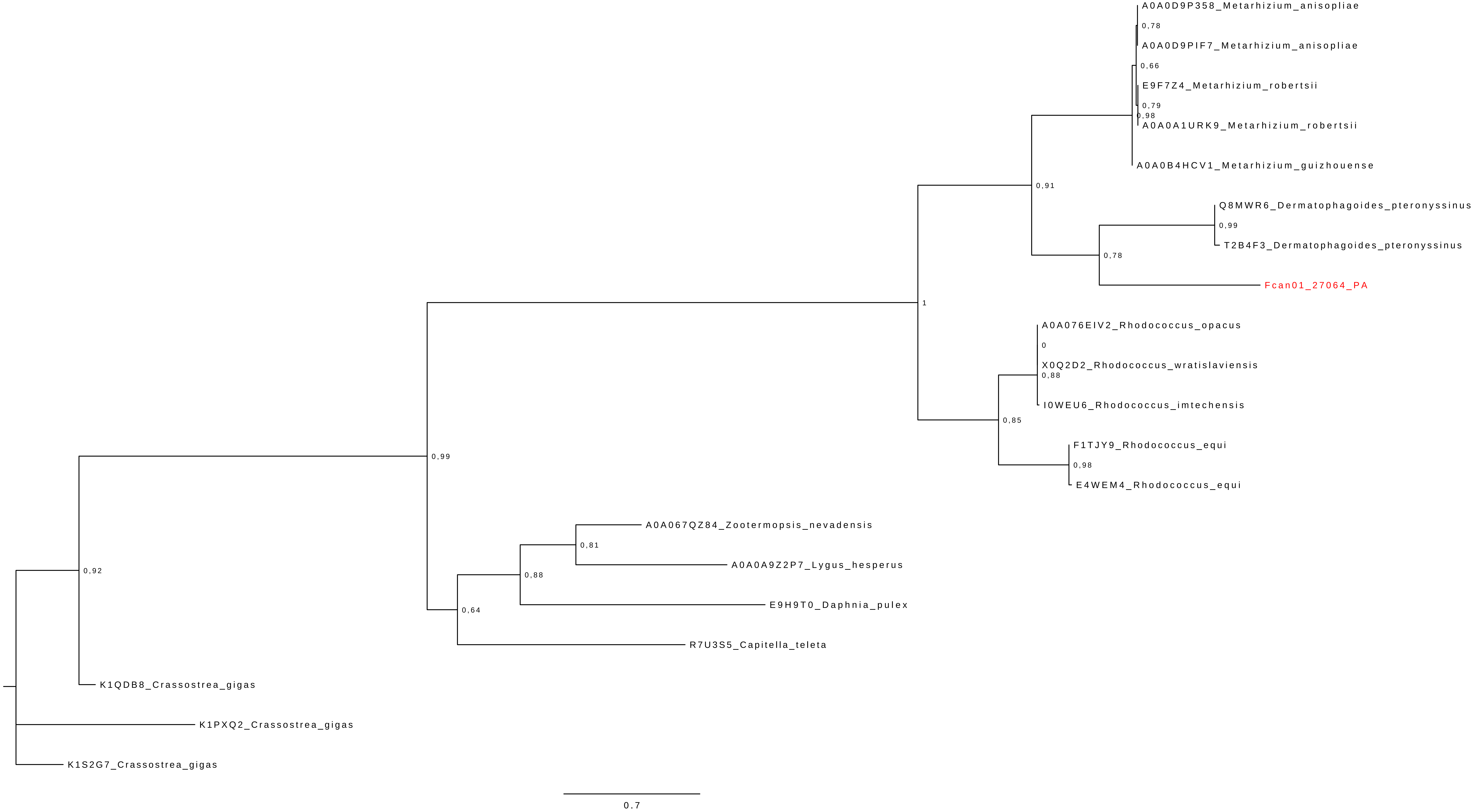
A0A0D9P358_Metarhizium_anisopliae
0,78
A0A0D9PIF7_Metarhizium_anisopliae
0,66
E9F7Z4_Metarhizium_robertsii
0,79
0,98
A0A0A1URK9_Metarhizium_robertsii
A0A0B4HCV1_Metarhizium_guizhouense
0,91
Q8MWR6_Dermatophagoides_pteronyssinus
0,99
T2B4F3_Dermatophagoides_pteronyssinus
0,78
Fcan01_27064_PA
1
A0A076EIV2_Rhodococcus_opacus
0
X0Q2D2_Rhodococcus_wratislaviensis
0,88
I0WEU6_Rhodococcus_imtechensis
0,85
F1TJY9_Rhodococcus_equi
0,98
E4WEM4_Rhodococcus_equi
0,99
A0A067QZ84_Zootermopsis_nevadensis
0,81
A0A0A9Z2P7_Lygus_hesperus
0,88
E9H9T0_Daphnia_pulex
0,64
R7U3S5_Capitella_teleta
0,92
K1QDB8_Crassostrea_gigas
K1PXQ2_Crassostrea_gigas
K1S2G7_Crassostrea_gigas
0.7

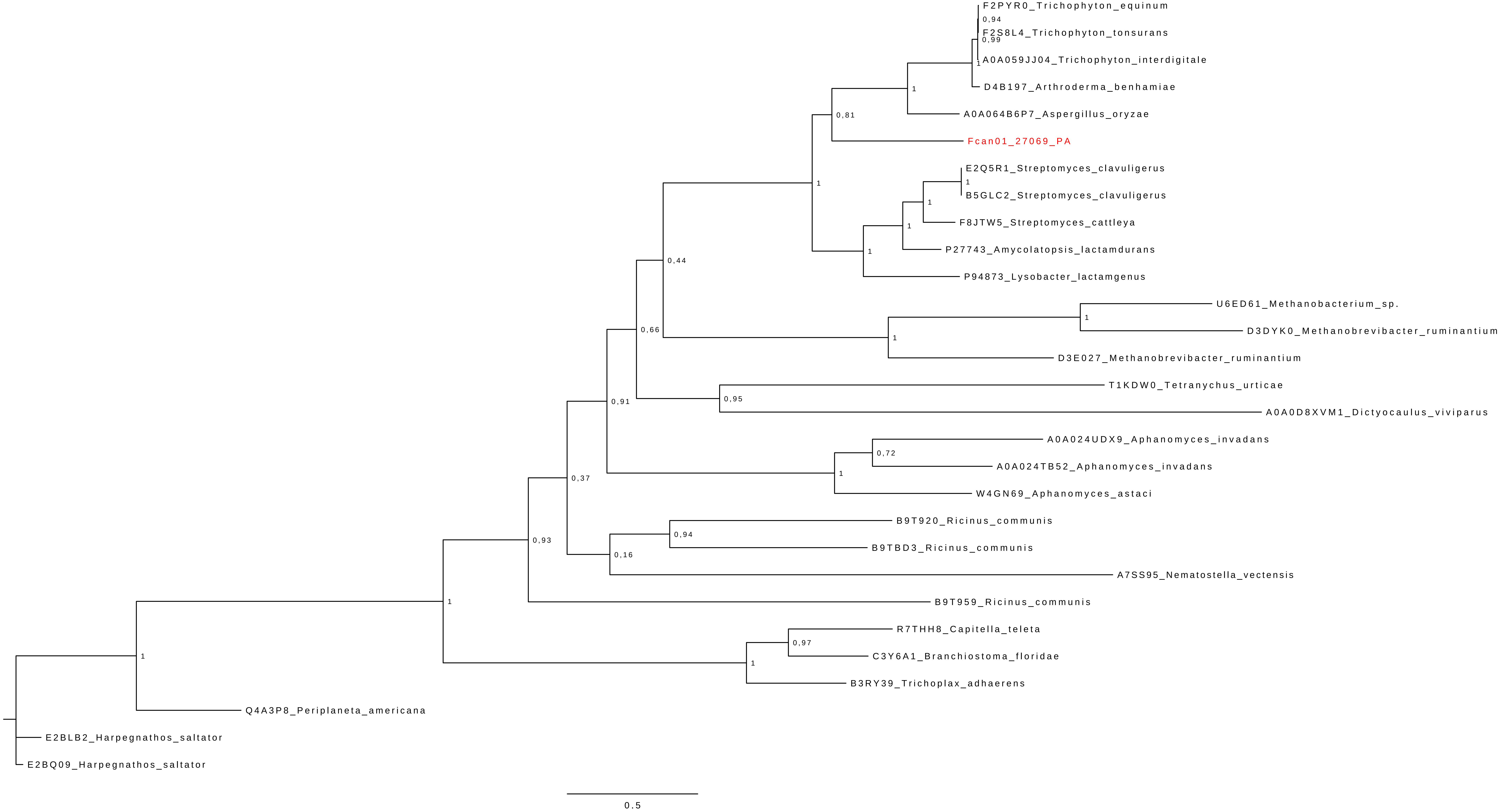

F2PYR0_Trichophyton_equinum
0,94
F2S8L4_Trichophyton_tonsurans
0,99
A0A059JJ04_Trichophyton_interdigitale
1
D4B197_Arthroderma_benhamiae
1
A0A064B6P7_Aspergillus_oryzae
0,81
Fcan01_27069_PA
E2Q5R1_Streptomyces_clavuligerus
1
B5GLC2_Streptomyces_clavuligerus
1
F8JTW5_Streptomyces_cattleya
1
P27743_Amycolatopsis_lactamdurans
1
P94873_Lysobacter_lactamgenus
1
U6ED61_Methanobacterium_sp.
1
D3DYK0_Methanobrevibacter_ruminantium
1
D3E027_Methanobrevibacter_ruminantium
0,44
0,66
T1KDW0_Tetranychus_urticae
0,95
A0A0D8XVM1_Dictyocaulus_viviparus
0,91
A0A024UDX9_Aphanomyces_invadans
0,72
A0A024TB52_Aphanomyces_invadans
1
W4GN69_Aphanomyces_astaci
0,37
B9T920_Ricinus_communis
0,94
B9TBD3_Ricinus_communis
0,16
A7SS95_Nematostella_vectensis
0,93
B9T959_Ricinus_communis
1
R7THH8_Capitella_teleta
0,97
C3Y6A1_Branchiostoma_floridae
1
B3RY39_Trichoplax_adhaerens
1
Q4A3P8_Periplaneta_americana
E2BLB2_Harpegnathos_saltator
E2BQ09_Harpegnathos_saltator
0.5

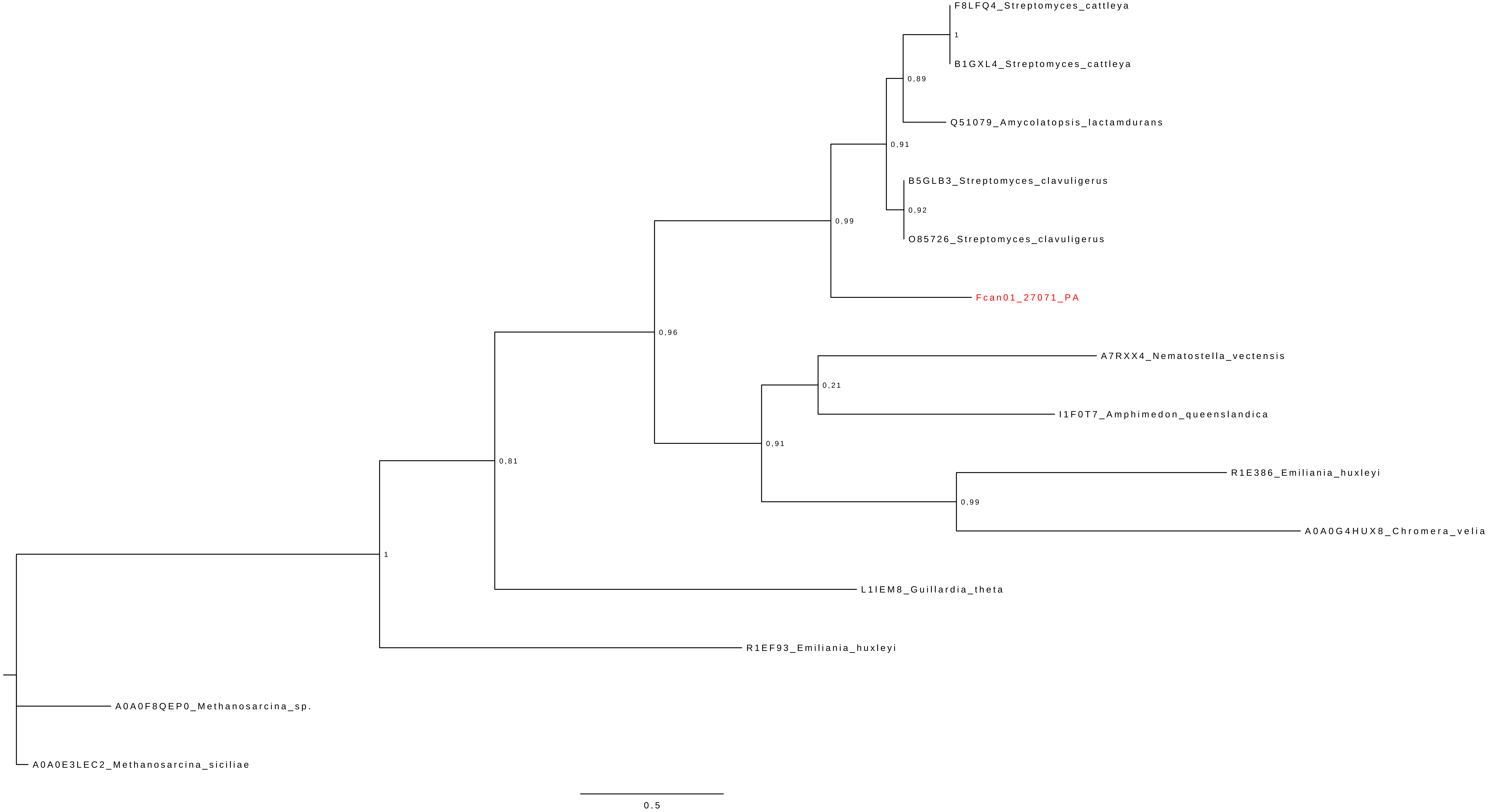

F8LFQ4_Streptomyces_cattleya
1
B1GXL4_Streptomyces_cattleya
0,89
Q51079_Amycolatopsis_lactamdurans
0,91
B5GLB3_Streptomyces_clavuligerus
0,92
O85726_Streptomyces_clavuligerus
0,99
Fcan01_27071_PA
0,96
A7RXX4_Nematostella_vectensis
0,21
I1F0T7_Amphimedon_queenslandica
0,91
R1E386_Emiliania_huxleyi
0,99
A0A0G4HUX8_Chromera_velia
0,81
1
L1IEM8_Guillardia_theta
R1EF93_Emiliania_huxleyi
A0A0F8QEP0_Methanosarcina_sp.
A0A0E3LEC2_Methanosarcina_siciliae
0.5

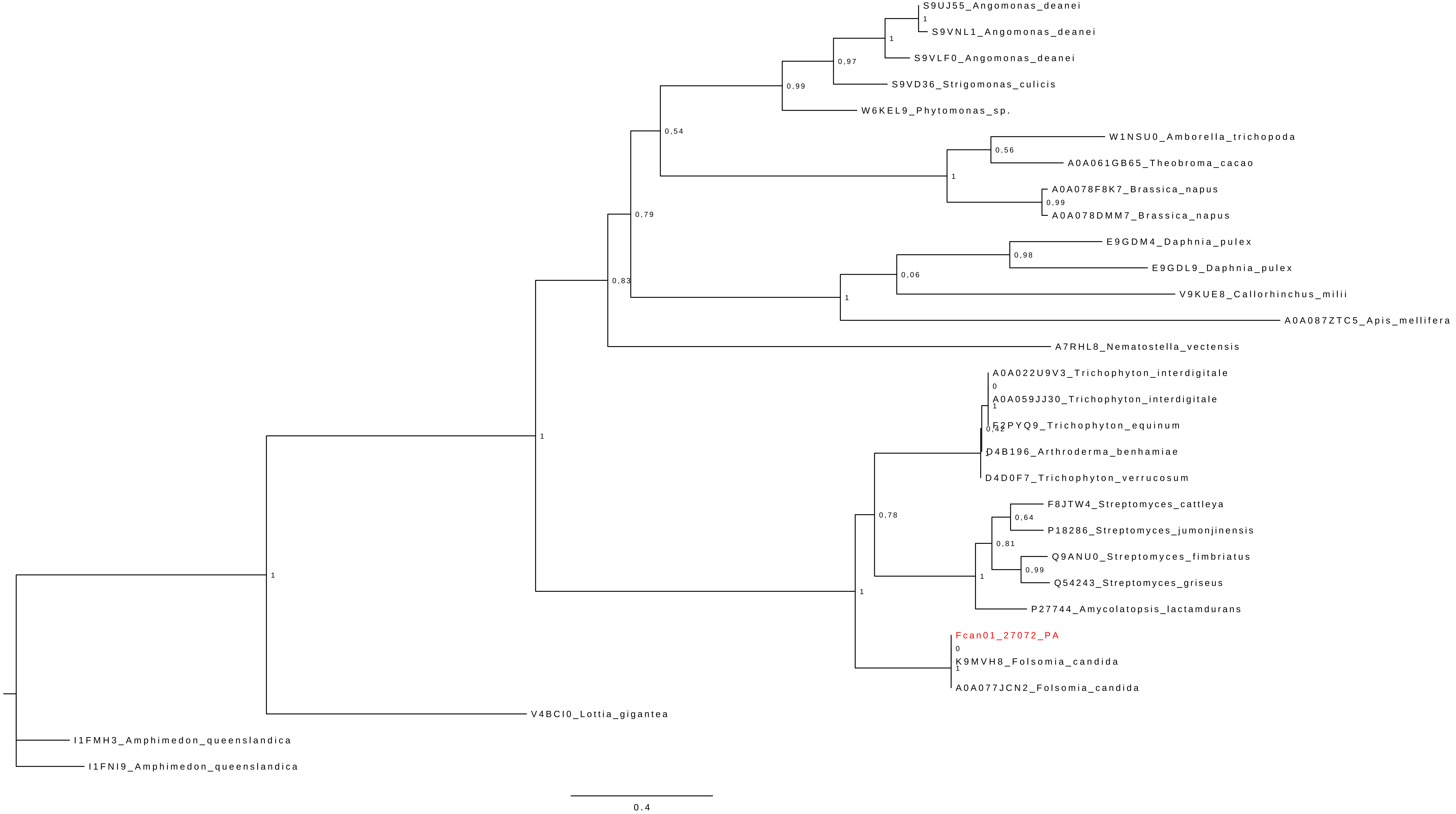
S9UJ55_Angomonas_deanei
S9VNL1_Angomonas_deanei
S9VLF0_Angomonas_deanei
S9VD36_Strigomonas_culicis
W6KEL9_Phytomonas_sp.
W1NSU0_Amborella_trichopoda
A0A061GB65_Theobroma_cacao
A0A078F8K7_Brassica_napus
A0A078DMM7_Brassica_napus
E9GDM4_Daphnia_pulex
E9GDL9_Daphnia_pulex
V9KUE8_Callorhinchus_milii
A0A087ZTC5_Apis_mellifera
A7RHL8_Nematostella_vectensis
A0A022U9V3_Trichophyton_interdigitale
A0A059JJ30_Trichophyton_interdigitale
F2PYQ9_Trichophyton_equinum
D4B196_Arthroderma_benhamiae
D4D0F7_Trichophyton_verrucosum
F8JTW4_Streptomyces_cattleya
P18286_Streptomyces_jumonjinensis
Q9ANU0_Streptomyces_fimbriatus
Q54243_Streptomyces_griseus
P27744_Amycolatopsis_lactamdurans
Fcan01_27072_PA
K9MVH8_Folsomia_candida
A0A077JCN2_Folsomia_candida
V4BCI0_Lottia_gigantea
I1FMH3_Amphimedon_queenslandica
I1FNI9_Amphimedon_queenslandica
0.4

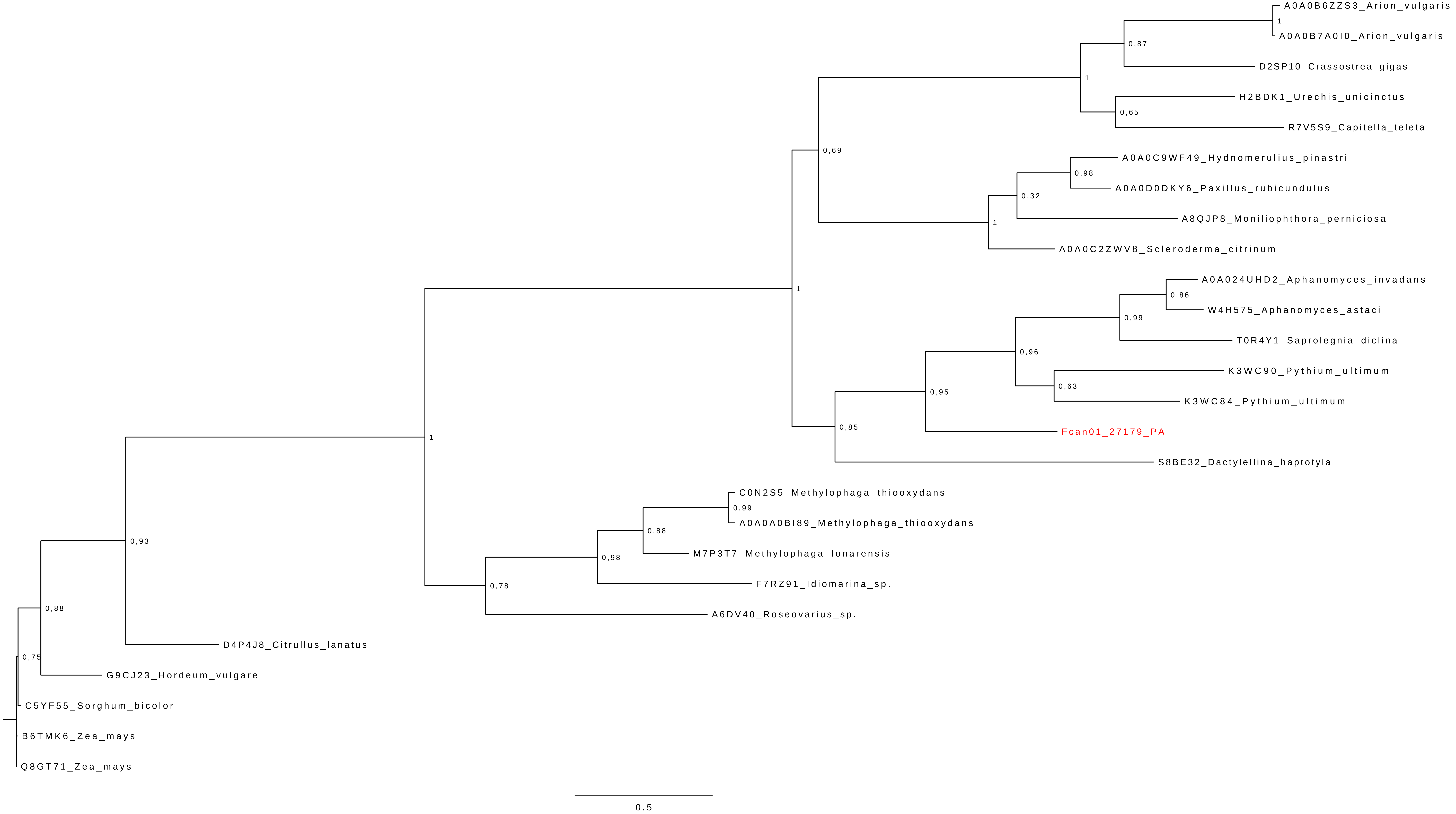
A0A0B6ZZS3_Arion_vulgaris
1
A0A0B7A0I0_Arion_vulgaris
0,87
D2SP10_Crassostrea_gigas
1
H2BDK1_Urechis_unicinctus
0,65
R7V5S9_Capitella_teleta
0,69
A0A0C9WF49_Hydnomerulius_pinastri
0,98
A0A0D0DKY6_Paxillus_rubicundulus
0,32
A8QJP8_Moniliophthora_perniciosa
1
A0A0C2ZWV8_Scleroderma_citrinum
1
A0A024UHD2_Aphanomyces_invadans
0,86
W4H575_Aphanomyces_astaci
0,99
T0R4Y1_Saprolegnia_diclina
0,96
K3WC90_Pythium_ultimum
0,63
K3WC84_Pythium_ultimum
0,95
0,85
Fcan01_27179_PA
S8BE32_Dactylellina_haptotyla
1
C0N2S5_Methylophaga_thiooxydans
0,99
A0A0A0BI89_Methylophaga_thiooxydans
0,88
M7P3T7_Methylophaga_lonarensis
0,98
F7RZ91_Idiomarina_sp.
0,78
A6DV40_Roseovarius_sp.
0,93
D4P4J8_Citrullus_lanatus
0,88
0,75
G9CJ23_Hordeum_vulgare
C5YF55_Sorghum_bicolor
B6TMK6_Zea_mays
Q8GT71_Zea_mays
0.5

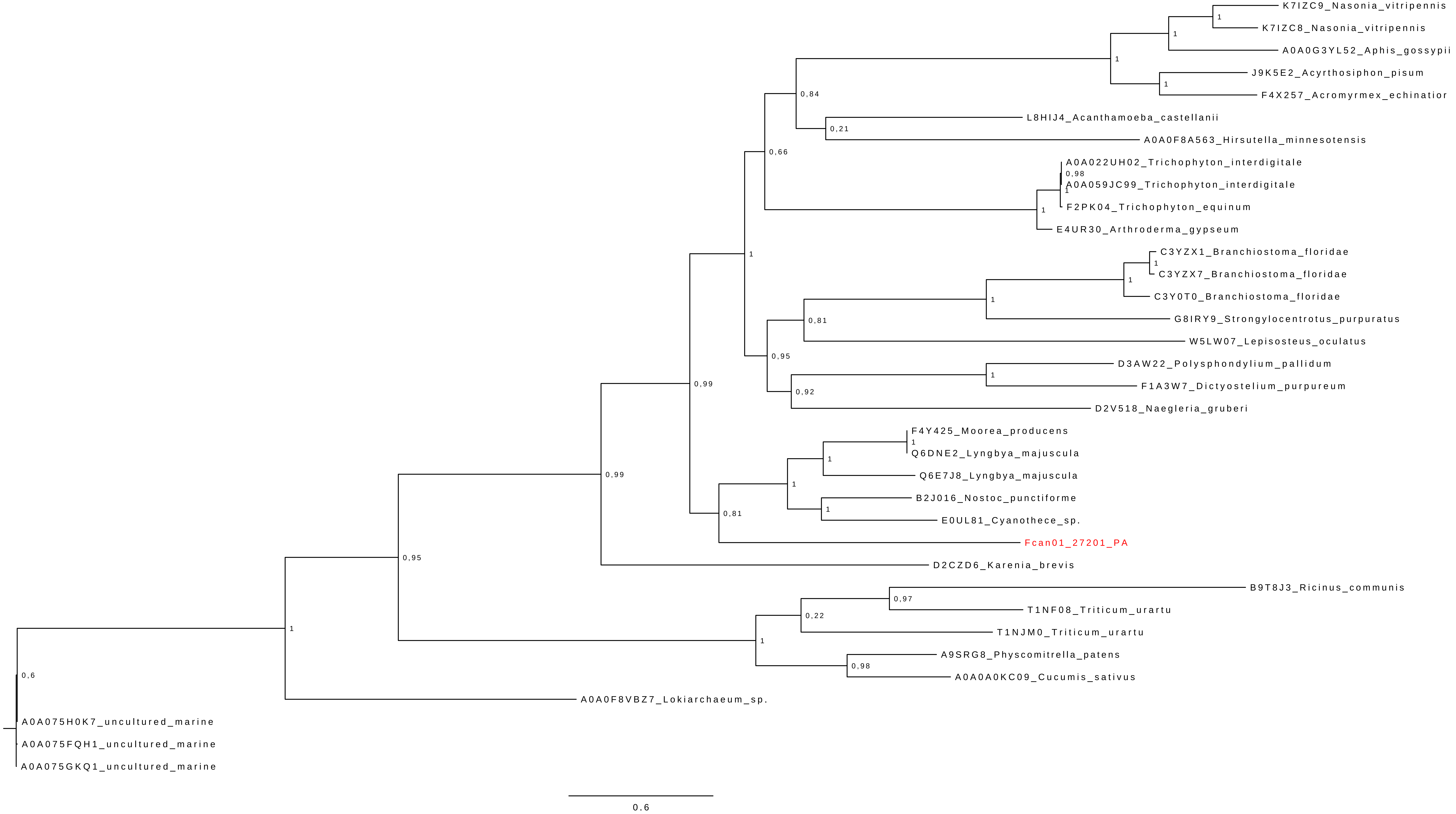

K7IZC9_Nasonia_vitripennis
K7IZC8_Nasonia_vitripennis
A0A0G3YL52_Aphis_gossypii
J9K5E2_Acyrthosiphon_pisum
F4X257_Acromyrmex_echinatior
L8HIJ4_Acanthamoeba_castellanii
A0A0F8A563_Hirsutella_minnesotensis
A0A022UH02_Trichophyton_interdigitale
A0A059JC99_Trichophyton_interdigitale
F2PK04_Trichophyton_equinum
E4UR30_Arthroderma_gypseum
C3YZX1_Branchiostoma_floridae
C3YZX7_Branchiostoma_floridae
C3Y0T0_Branchiostoma_floridae
G8IRY9_Strongylocentrotus_purpuratus
W5LW07_Lepisosteus_oculatus
D3AW22_Polysphondylium_pallidum
F1A3W7_Dictyostelium_purpureum
D2V518_Naegleria_gruberi
F4Y425_Moorea_producens
Q6DNE2_Lyngbya_majuscula
Q6E7J8_Lyngbya_majuscula
B2J016_Nostoc_punctiforme
E0UL81_Cyanothece_sp.
Fcan01_27201_PA
D2CZD6_Karenia_brevis
B9T8J3_Ricinus_communis
T1NF08_Triticum_urartu
T1NJM0_Triticum_urartu
A9SRG8_Physcomitrella_patens
A0A0A0KC09_Cucumis_sativus
A0A0F8VBZ7_Lokiarchaeum_sp.
A0A075H0K7_uncultured_marine
A0A075FQH1_uncultured_marine
A0A075GKQ1_uncultured_marine
0,84
0,21
0,66
0,98
0,81
0,95
0,92
0,99
0,99
0,81
0,95
0,97
0,22
0,98
0,6
0.6

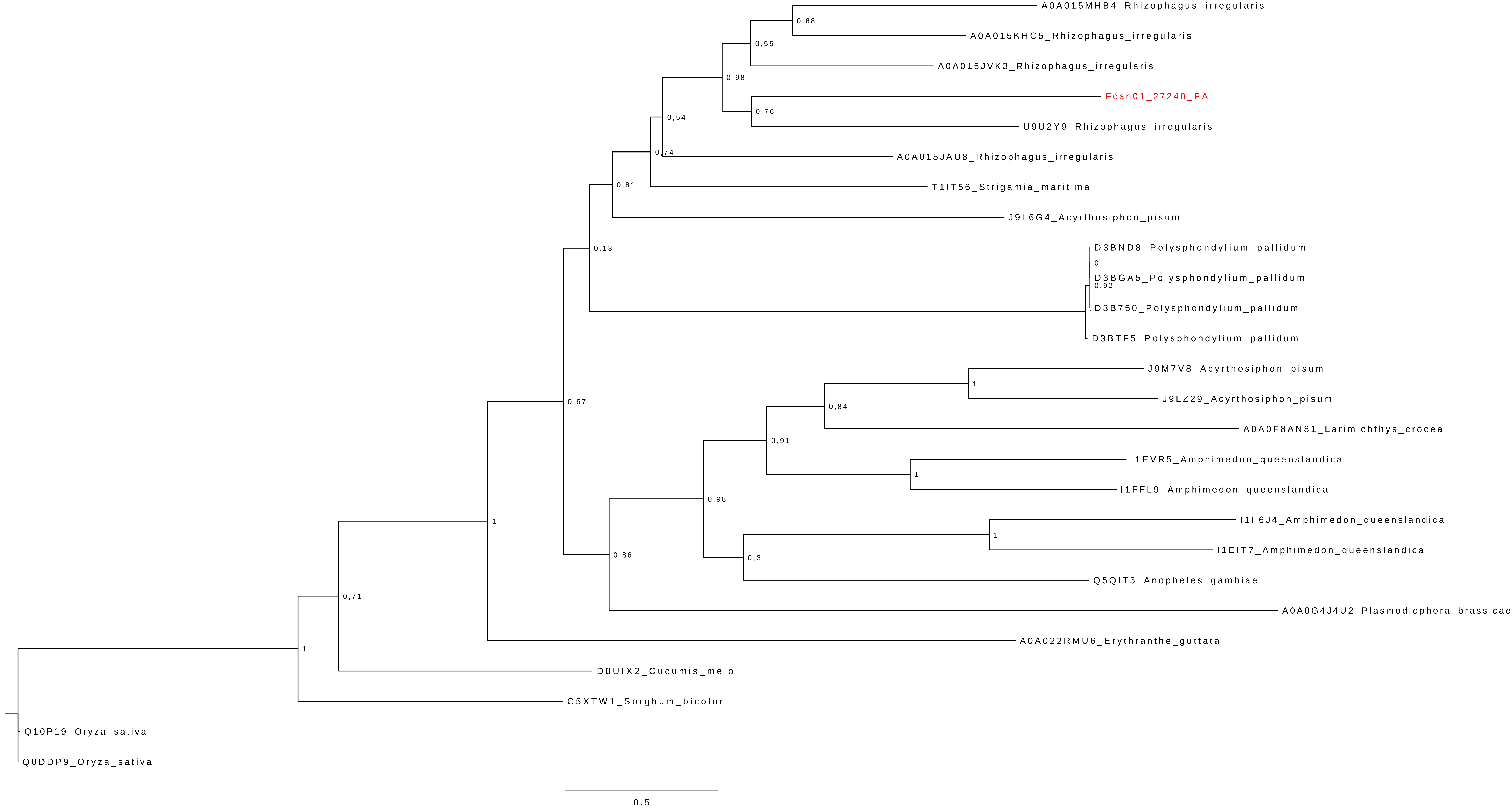
A0A015MHB4_Rhizophagus_irregularis
0,88
A0A015KHC5_Rhizophagus_irregularis
0,55
A0A015JVK3_Rhizophagus_irregularis
0,98
Fcan01_27248_PA
0,76
U9U2Y9_Rhizophagus_irregularis
0,54
0,74
A0A015JAU8_Rhizophagus_irregularis
0,81
T1IT56_Strigamia_maritima
J9L6G4_Acyrthosiphon_pisum
0,13
D3BND8_Polysphondylium_pallidum
0
D3BGA5_Polysphondylium_pallidum
0,92
D3B750_Polysphondylium_pallidum
1
D3BTF5_Polysphondylium_pallidum
J9M7V8_Acyrthosiphon_pisum
1
J9LZ29_Acyrthosiphon_pisum
0,67
0,84
A0A0F8AN81_Larimichthys_crocea
0,91
I1EVR5_Amphimedon_queenslandica
1
I1FFL9_Amphimedon_queenslandica
0,98
I1F6J4_Amphimedon_queenslandica
1
1
I1EIT7_Amphimedon_queenslandica
0,86
0,3
Q5QIT5_Anopheles_gambiae
0,71
A0A0G4J4U2_Plasmodiophora_brassicae
A0A022RMU6_Erythranthe_guttata
1
D0UIX2_Cucumis_melo
C5XTW1_Sorghum_bicolor
Q10P19_Oryza_sativa
Q0DDP9_Oryza_sativa
0.5

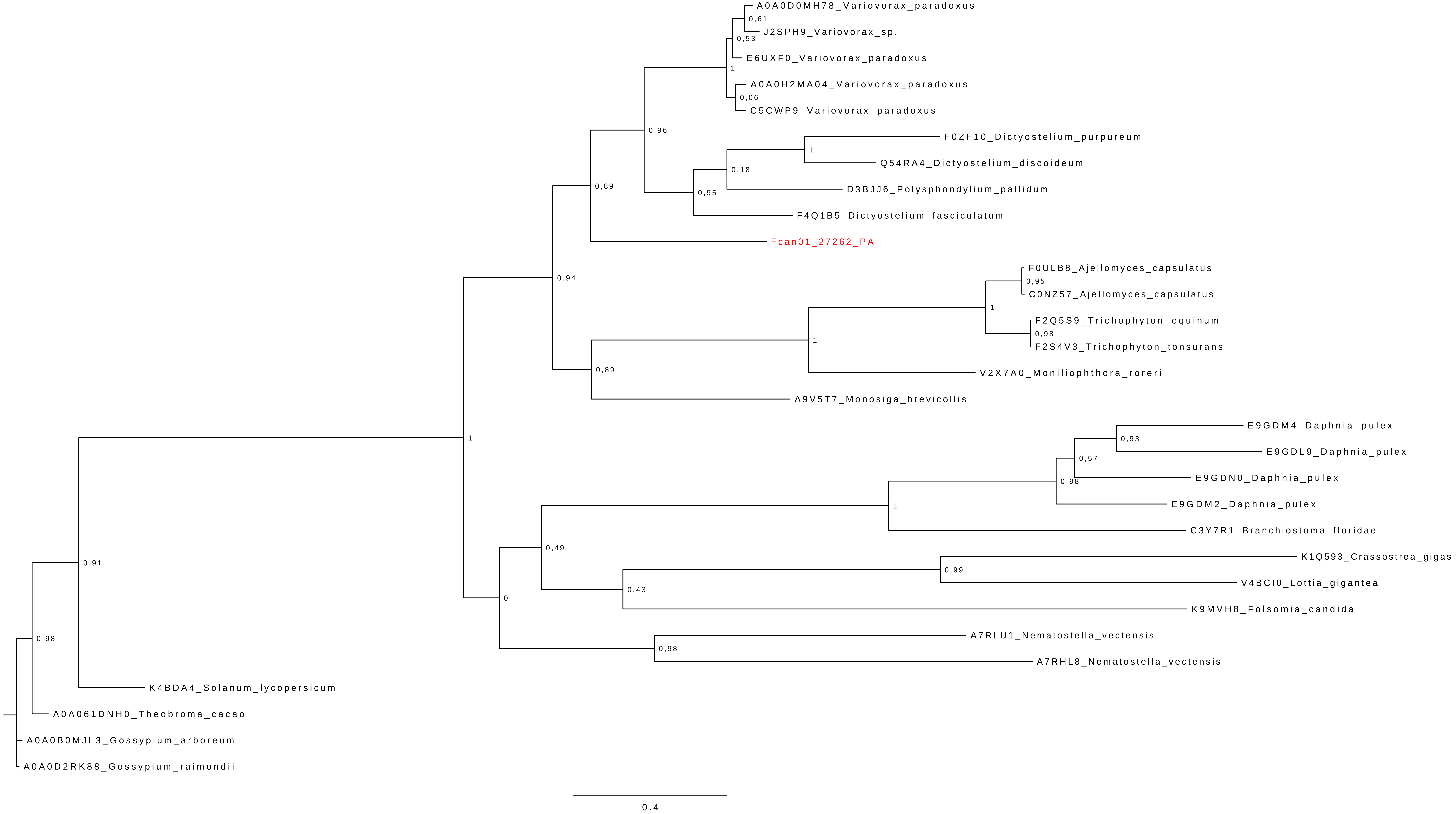
A0A0D0MH78_Variovorax_paradoxus
J2SPH9_Variovorax_sp.
E6UXF0_Variovorax_paradoxus
A0A0H2MA04_Variovorax_paradoxus
C5CWP9_Variovorax_paradoxus
F0ZF10_Dictyostelium_purpureum
Q54RA4_Dictyostelium_discoideum
D3BJJ6_Polysphondylium_pallidum
F4Q1B5_Dictyostelium_fasciculatum
Fcan01_27262_PA
F0ULB8_Ajellomyces_capsulatus
C0NZ57_Ajellomyces_capsulatus
F2Q5S9_Trichophyton_equinum
F2S4V3_Trichophyton_tonsurans
V2X7A0_Moniliophthora_roreri
A9V5T7_Monosiga_brevicollis
E9GDM4_Daphnia_pulex
E9GDL9_Daphnia_pulex
E9GDN0_Daphnia_pulex
E9GDM2_Daphnia_pulex
C3Y7R1_Branchiostoma_floridae
K1Q593_Crassostrea_gigas
V4BCI0_Lottia_gigantea
K9MVH8_Folsomia_candida
A7RLU1_Nematostella_vectensis
A7RHL8_Nematostella_vectensis
K4BDA4_Solanum_lycopersicum
A0A061DNH0_Theobroma_cacao
A0A0B0MJL3_Gossypium_arboreum
A0A0D2RK88_Gossypium_raimondii
0.4

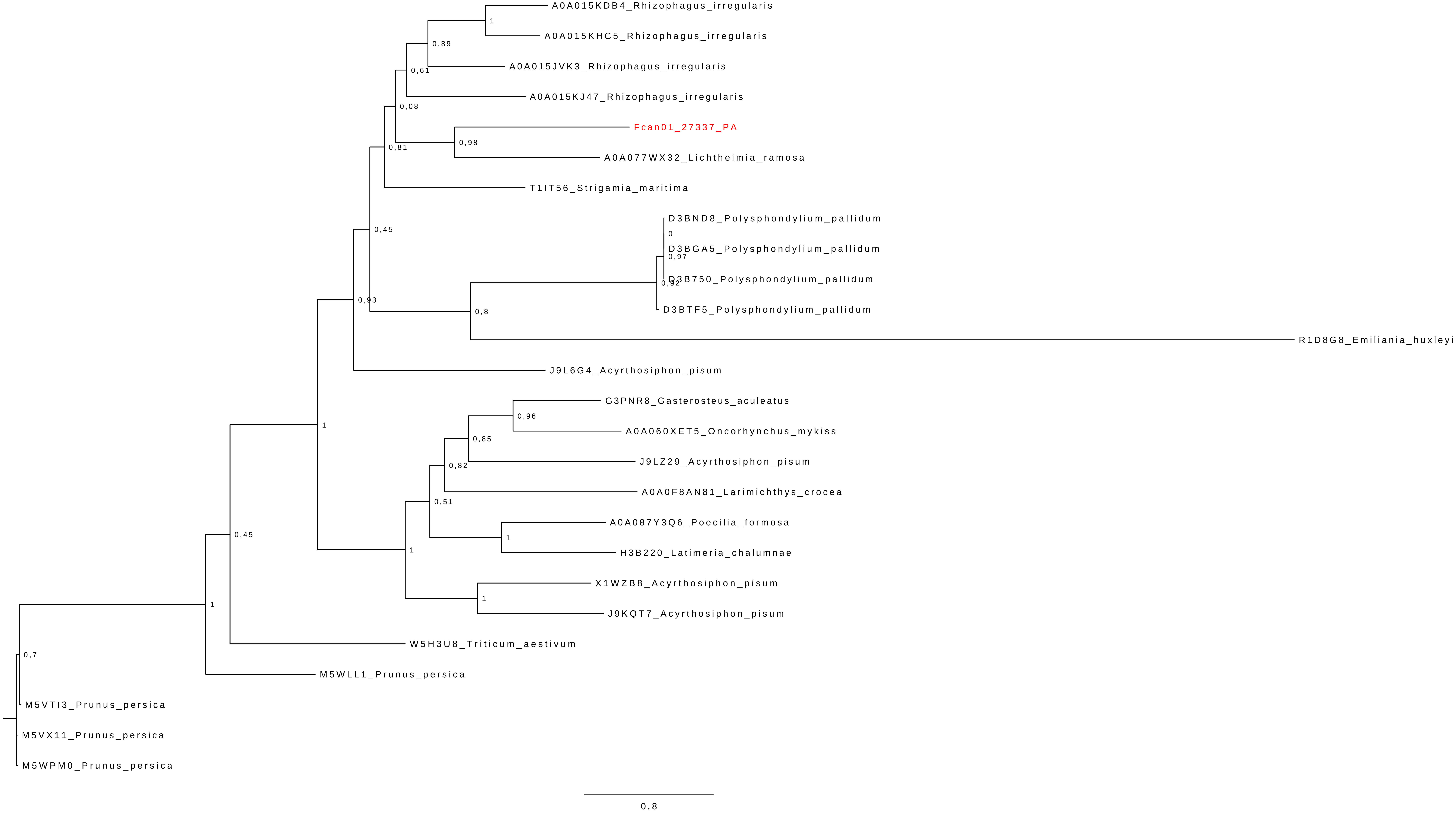
A0A015KDB4_Rhizophagus_irregularis
1
A0A015KHC5_Rhizophagus_irregularis
0,89
A0A015JVK3_Rhizophagus_irregularis
0,61
A0A015KJ47_Rhizophagus_irregularis
0,08
Fcan01_27337_PA
0,98
0,81
A0A077WX32_Lichtheimia_ramosa
T1IT56_Strigamia_maritima
D3BND8_Polysphondylium_pallidum
0
D3BGA5_Polysphondylium_pallidum
0,97
D3B750_Polysphondylium_pallidum
0,92
0,45
D3BTF5_Polysphondylium_pallidum
0,93
0,8
R1D8G8_Emiliania_huxleyi
J9L6G4_Acyrthosiphon_pisum
G3PNR8_Gasterosteus_aculeatus
0,96
1
A0A060XET5_Oncorhynchus_mykiss
0,85
J9LZ29_Acyrthosiphon_pisum
0,82
A0A0F8AN81_Larimichthys_crocea
0,51
A0A087Y3Q6_Poecilia_formosa
0,45
1
1
H3B220_Latimeria_chalumnae
X1WZB8_Acyrthosiphon_pisum
1
1
J9KQT7_Acyrthosiphon_pisum
W5H3U8_Triticum_aestivum
0,7
M5WLL1_Prunus_persica
M5VTI3_Prunus_persica
M5VX11_Prunus_persica
M5WPM0_Prunus_persica
0.8

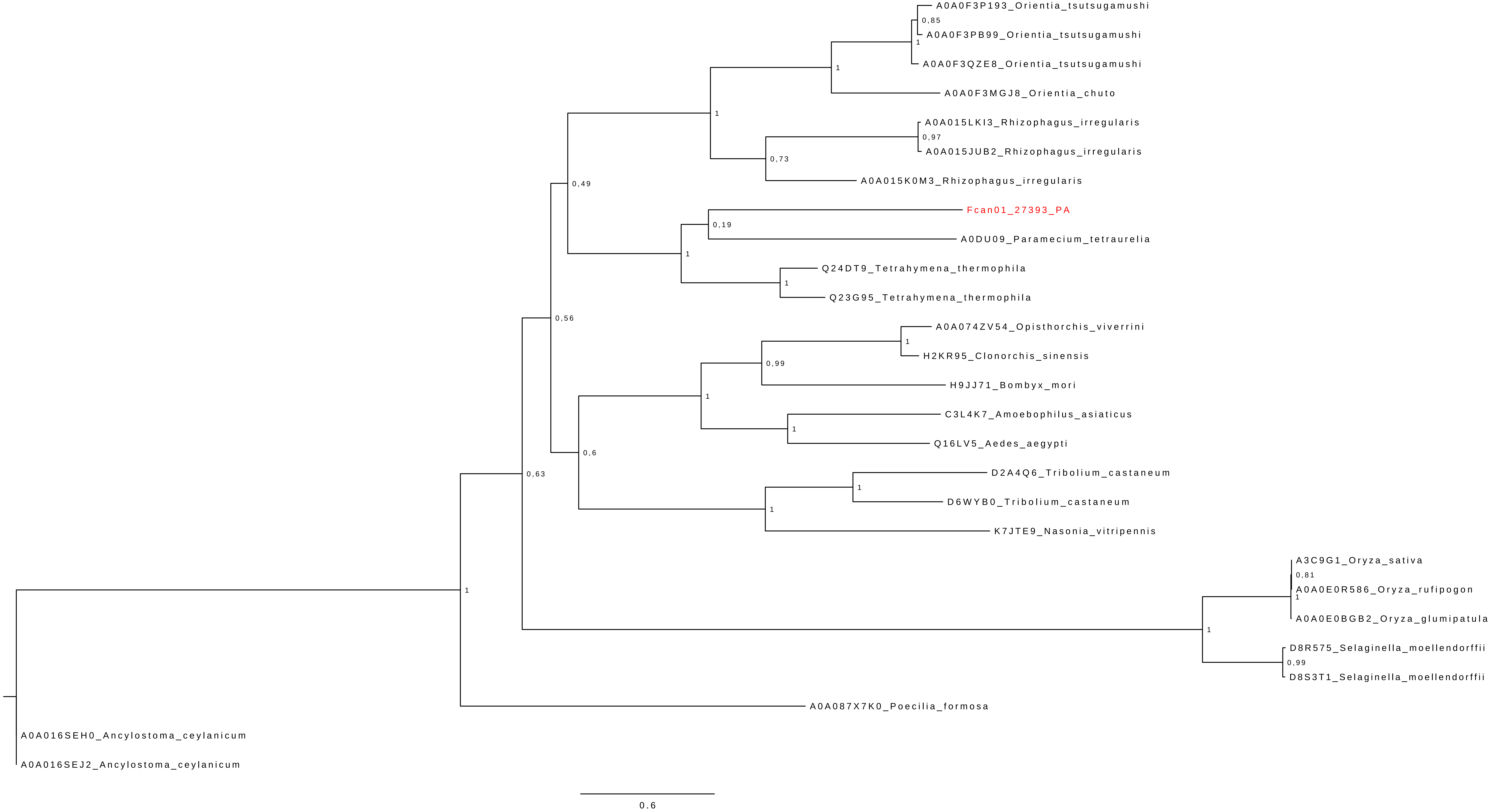
A0A0F3P193_Orientia_tsutsugamushi
0,85
A0A0F3PB99_Orientia_tsutsugamushi
1
A0A0F3QZE8_Orientia_tsutsugamushi
1
A0A0F3MGJ8_Orientia_chuto
1
A0A015LKI3_Rhizophagus_irregularis
0,97
A0A015JUB2_Rhizophagus_irregularis
0,73
A0A015K0M3_Rhizophagus_irregularis
0,49
Fcan01_27393_PA
0,19
A0DU09_Paramecium_tetraurelia
1
Q24DT9_Tetrahymena_thermophila
1
Q23G95_Tetrahymena_thermophila
0,56
A0A074ZV54_Opisthorchis_viverrini
1
H2KR95_Clonorchis_sinensis
0,99
H9JJ71_Bombyx_mori
1
C3L4K7_Amoebophilus_asiaticus
1
Q16LV5_Aedes_aegypti
0,6
D2A4Q6_Tribolium_castaneum
1
D6WYB0_Tribolium_castaneum
1
K7JTE9_Nasonia_vitripennis
0,63
A3C9G1_Oryza_sativa
0,81
A0A0E0R586_Oryza_rufipogon
1
A0A0E0BGB2_Oryza_glumipatula
1
1
D8R575_Selaginella_moellendorffii
0,99
D8S3T1_Selaginella_moellendorffii
A0A087X7K0_Poecilia_formosa
A0A016SEH0_Ancylostoma_ceylanicum
A0A016SEJ2_Ancylostoma_ceylanicum
0.6

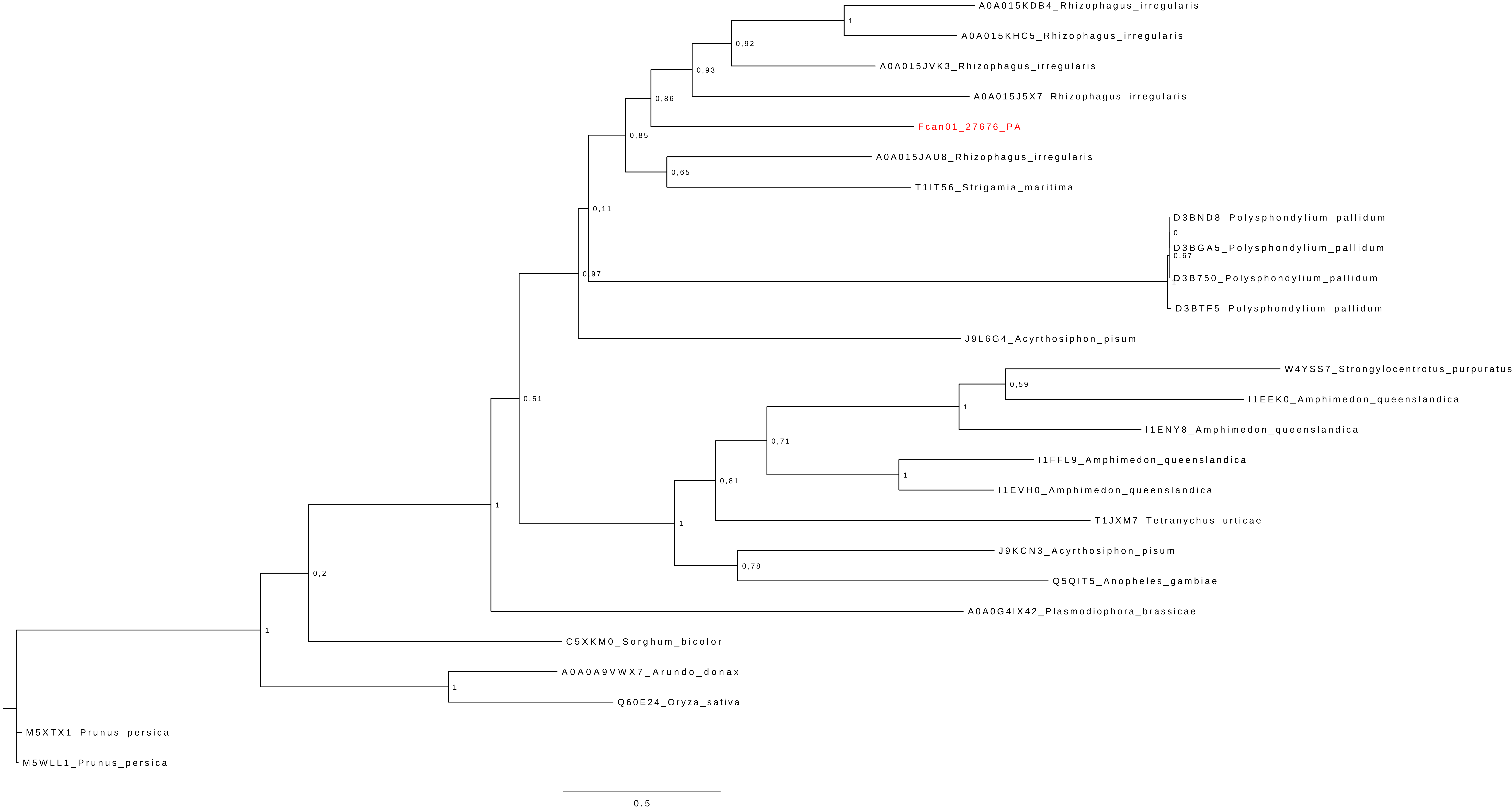
A0A015KDB4_Rhizophagus_irregularis
A0A015KHC5_Rhizophagus_irregularis
A0A015JVK3_Rhizophagus_irregularis
A0A015J5X7_Rhizophagus_irregularis
Fcan01_27676_PA
A0A015JAU8_Rhizophagus_irregularis
T1IT56_Strigamia_maritima
D3BND8_Polysphondylium_pallidum
D3BGA5_Polysphondylium_pallidum
D3B750_Polysphondylium_pallidum
D3BTF5_Polysphondylium_pallidum
J9L6G4_Acyrthosiphon_pisum
W4YSS7_Strongylocentrotus_purpuratus
I1EEK0_Amphimedon_queenslandica
I1ENY8_Amphimedon_queenslandica
I1FFL9_Amphimedon_queenslandica
I1EVH0_Amphimedon_queenslandica
T1JXM7_Tetranychus_urticae
J9KCN3_Acyrthosiphon_pisum
Q5QIT5_Anopheles_gambiae
A0A0G4IX42_Plasmodiophora_brassicae
C5XKM0_Sorghum_bicolor
A0A0A9VWX7_Arundo_donax
Q60E24_Oryza_sativa
M5XTX1_Prunus_persica
M5WLL1_Prunus_persica
1
0,92
0,93
0,86
0,85
0,65
0,11
0
0,67
1
0,97
0,59
1
0,71
1
0,81
0,51
1
1
0,78
0,2
1
1
0.5

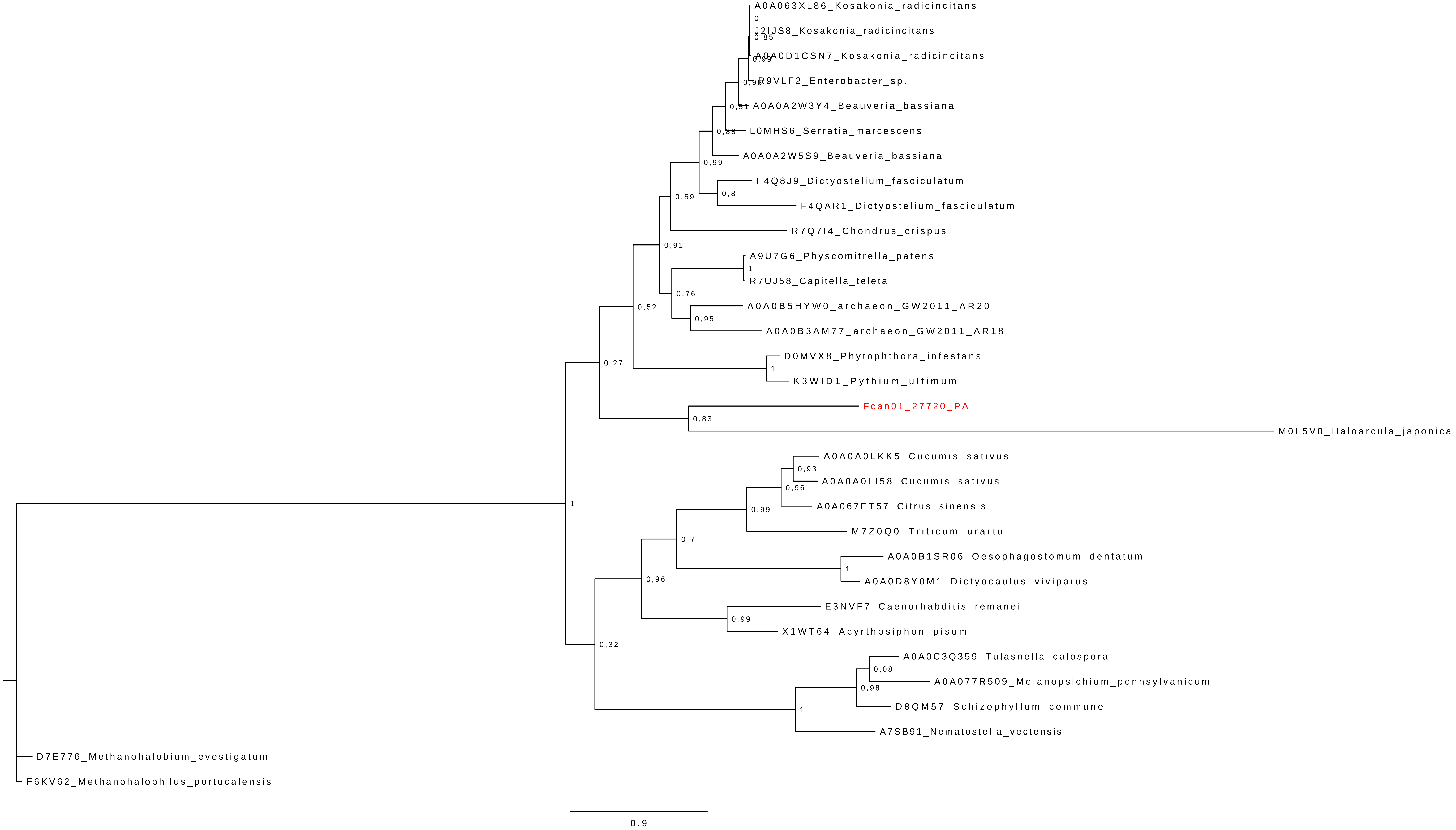
A0A063XL86_Kosakonia_radicincitans
J2IJS8_Kosakonia_radicincitans
A0A0D1CSN7_Kosakonia_radicincitans
R9VLF2_Enterobacter_sp.
A0A0A2W3Y4_Beauveria_bassiana
L0MHS6_Serratia_marcescens
A0A0A2W5S9_Beauveria_bassiana
F4Q8J9_Dictyostelium_fasciculatum
F4QAR1_Dictyostelium_fasciculatum
R7Q7I4_Chondrus_crispus
A9U7G6_Physcomitrella_patens
R7UJ58_Capitella_teleta
A0A0B5HYW0_archaeon_GW2011_AR20
A0A0B3AM77_archaeon_GW2011_AR18
D0MVX8_Phytophthora_infestans
K3WID1_Pythium_ultimum
Fcan01_27720_PA
M0L5V0_Haloarcula_japonica
A0A0A0LKK5_Cucumis_sativus
A0A0A0LI58_Cucumis_sativus
A0A067ET57_Citrus_sinensis
M7Z0Q0_Triticum_urartu
A0A0B1SR06_Oesophagostomum_dentatum
A0A0D8Y0M1_Dictyocaulus_viviparus
E3NVF7_Caenorhabditis_remanei
X1WT64_Acyrthosiphon_pisum
A0A0C3Q359_Tulasnella_calospora
A0A077R509_Melanopsichium_pennsylvanicum
D8QM57_Schizophyllum_commune
A7SB91_Nematostella_vectensis
D7E776_Methanohalobium_evestigatum
F6KV62_Methanohalophilus_portucalensis
0.9

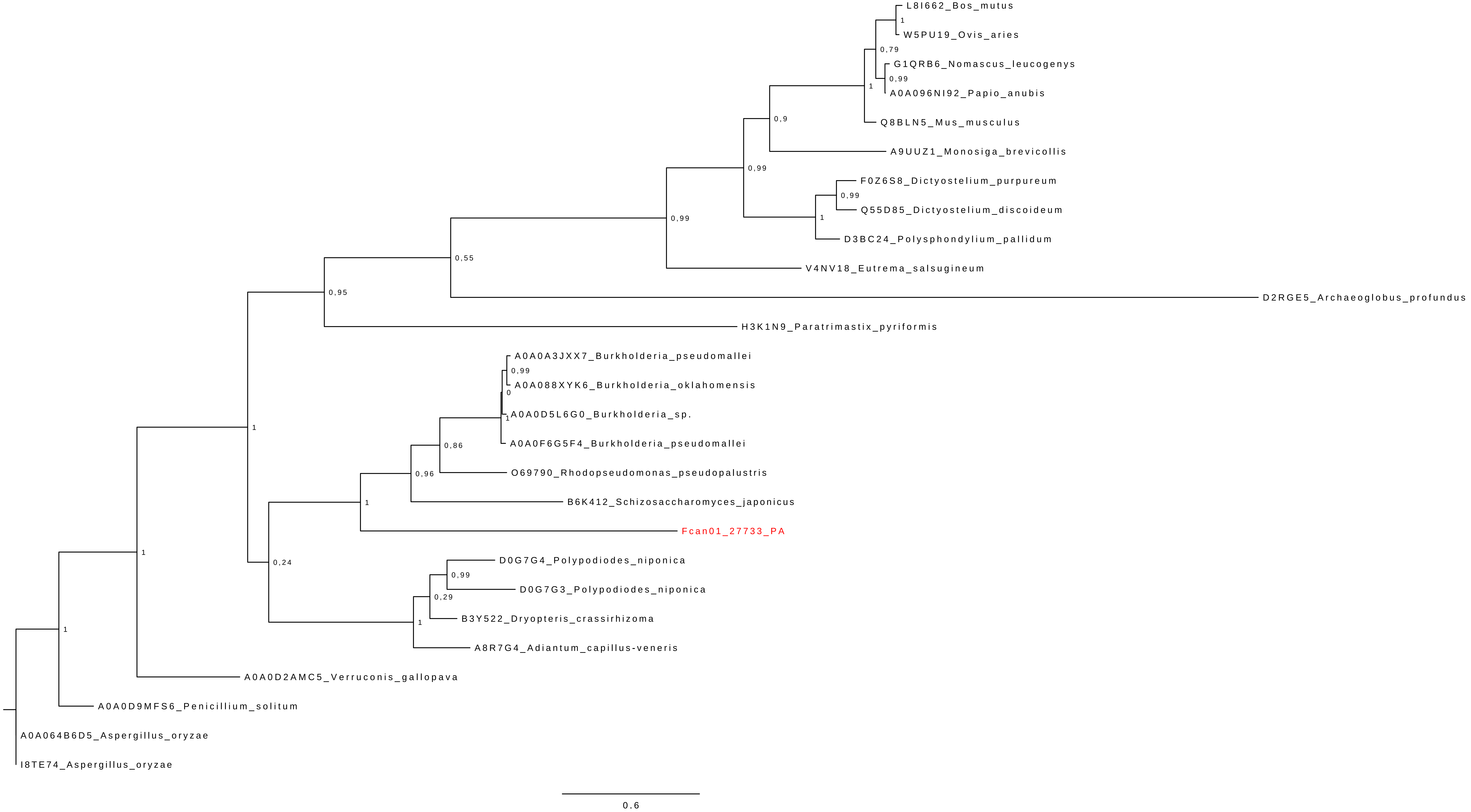
L8I662_Bos_mutus
W5PU19_Ovis_aries
G1QRB6_Nomascus_leucogenys
A0A096NI92_Papio_anubis
Q8BLN5_Mus_musculus
A9UUZ1_Monosiga_brevicollis
F0Z6S8_Dictyostelium_purpureum
Q55D85_Dictyostelium_discoideum
D3BC24_Polysphondylium_pallidum
V4NV18_Eutrema_salsugineum
D2RGE5_Archaeoglobus_profundus
H3K1N9_Paratrimastix_pyriformis
A0A0A3JXX7_Burkholderia_pseudomallei
A0A088XYK6_Burkholderia_oklahomensis
A0A0D5L6G0_Burkholderia_sp.
A0A0F6G5F4_Burkholderia_pseudomallei
O69790_Rhodopseudomonas_pseudopalustris
B6K412_Schizosaccharomyces_japonicus
Fcan01_27733_PA
D0G7G4_Polypodiodes_niponica
D0G7G3_Polypodiodes_niponica
B3Y522_Dryopteris_crassirhizoma
A8R7G4_Adiantum_capillus-veneris
A0A0D2AMC5_Verruconis_gallopava
A0A0D9MFS6_Penicillium_solitum
A0A064B6D5_Aspergillus_oryzae
I8TE74_Aspergillus_oryzae
0.6

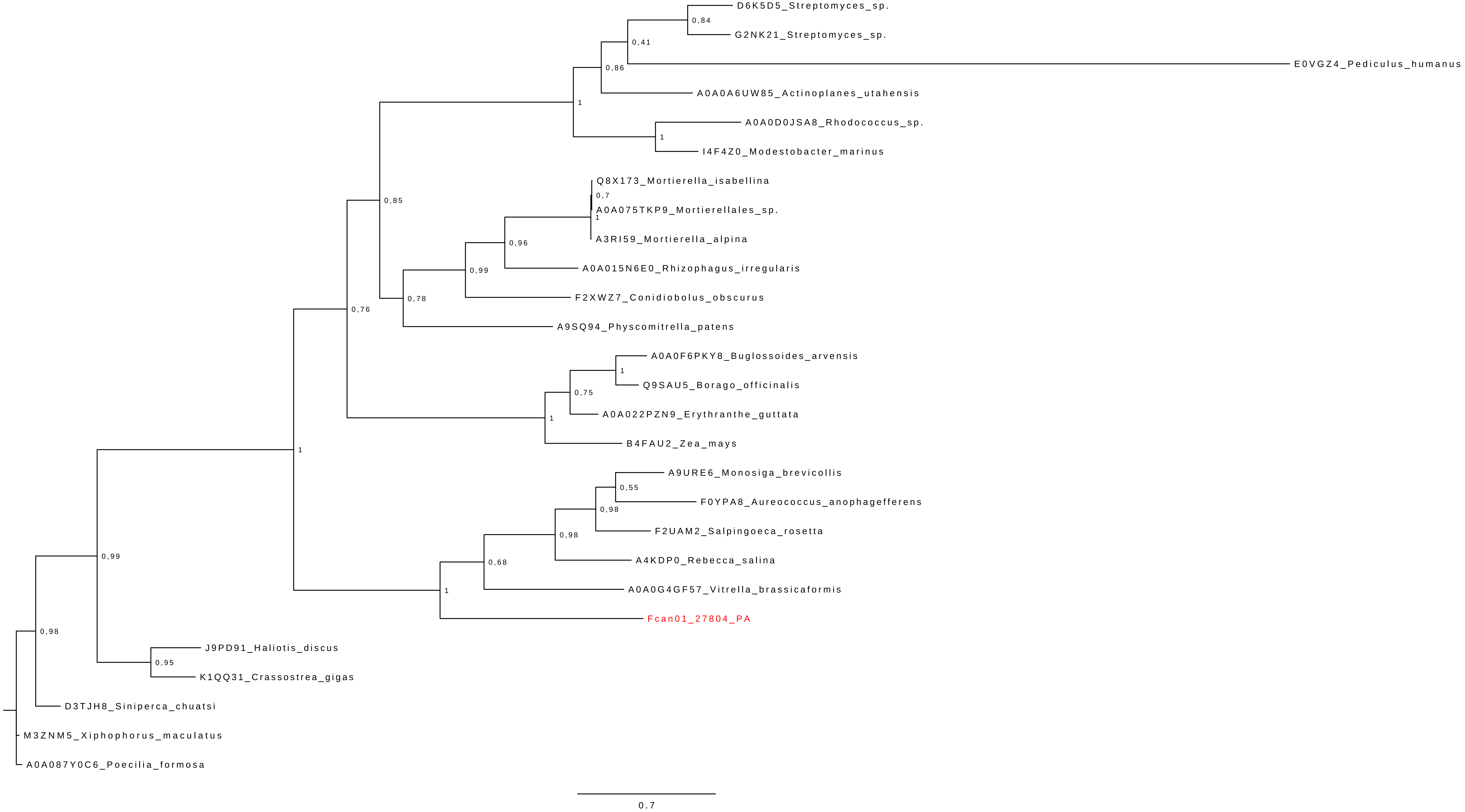
D6K5D5_Streptomyces_sp.
0,84
G2NK21_Streptomyces_sp.
0,41
E0VGZ4_Pediculus_humanus
0,86
A0A0A6UW85_Actinoplanes_utahensis
1
A0A0D0JSA8_Rhodococcus_sp.
1
I4F4Z0_Modestobacter_marinus
Q8X173_Mortierella_isabellina
0,7
A0A075TKP9_Mortierellales_sp.
1
A3RI59_Mortierella_alpina
0,85
0,96
A0A015N6E0_Rhizophagus_irregularis
0,99
F2XWZ7_Conidiobolus_obscurus
0,78
0,76
A9SQ94_Physcomitrella_patens
A0A0F6PKY8_Buglossoides_arvensis
1
Q9SAU5_Borago_officinalis
0,75
A0A022PZN9_Erythranthe_guttata
1
B4FAU2_Zea_mays
1
A9URE6_Monosiga_brevicollis
0,55
F0YPA8_Aureococcus_anophagefferens
0,98
F2UAM2_Salpingoeca_rosetta
0,98
A4KDP0_Rebecca_salina
0,68
A0A0G4GF57_Vitrella_brassicaformis
0,99
1
Fcan01_27804_PA
0,98
J9PD91_Haliotis_discus
0,95
K1QQ31_Crassostrea_gigas
D3TJH8_Siniperca_chuatsi
M3ZNM5_Xiphophorus_maculatus
A0A087Y0C6_Poecilia_formosa
0.7

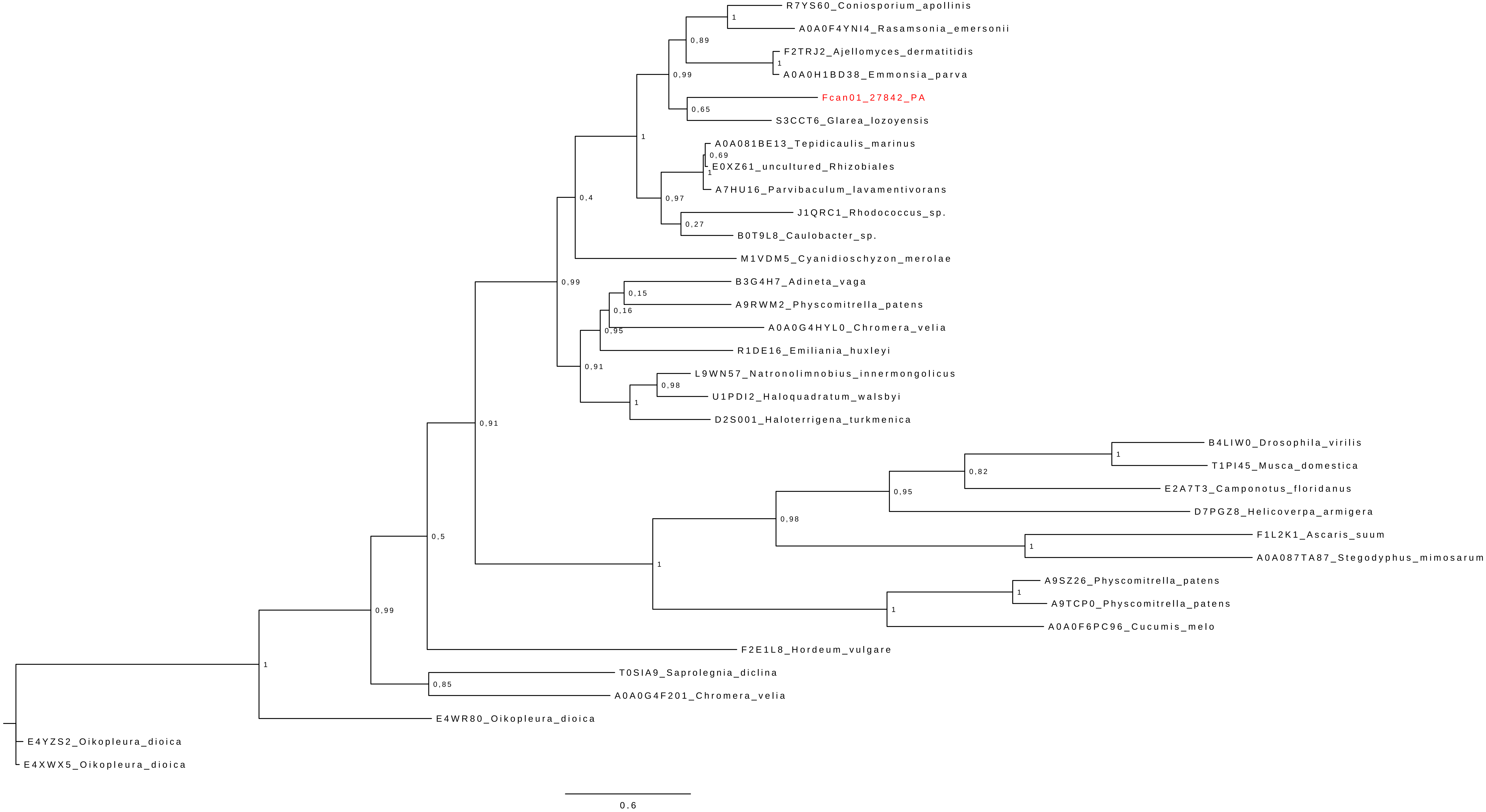
R7YS60_Coniosporium_apollinis
A0A0F4YNI4_Rasamsonia_emersonii
F2TRJ2_Ajellomyces_dermatitidis
A0A0H1BD38_Emmonsia_parva
Fcan01_27842_PA
S3CCT6_Glarea_lozoyensis
A0A081BE13_Tepidicaulis_marinus
E0XZ61_uncultured_Rhizobiales
A7HU16_Parvibaculum_lavamentivorans
J1QRC1_Rhodococcus_sp.
B0T9L8_Caulobacter_sp.
M1VDM5_Cyanidioschyzon_merolae
B3G4H7_Adineta_vaga
A9RWM2_Physcomitrella_patens
A0A0G4HYL0_Chromera_velia
R1DE16_Emiliania_huxleyi
L9WN57_Natronolimnobius_innermongolicus
U1PDI2_Haloquadratum_walsbyi
D2S001_Haloterrigena_turkmenica
B4LIW0_Drosophila_virilis
T1PI45_Musca_domestica
E2A7T3_Camponotus_floridanus
D7PGZ8_Helicoverpa_armigera
F1L2K1_Ascaris_suum
A0A087TA87_Stegodyphus_mimosarum
A9SZ26_Physcomitrella_patens
A9TCP0_Physcomitrella_patens
A0A0F6PC96_Cucumis_melo
F2E1L8_Hordeum_vulgare
T0SIA9_Saprolegnia_diclina
A0A0G4F201_Chromera_velia
E4WR80_Oikopleura_dioica
E4YZS2_Oikopleura_dioica
E4XWX5_Oikopleura_dioica
1
0,89
1
0,99
0,65
1
0,69
1
0,97
0,27
0,4
0,99
0,15
0,16
0,95
0,91
0,98
1
0,91
1
0,82
0,95
0,98
1
1
1
1
0,5
0,99
0,85
1
0.6

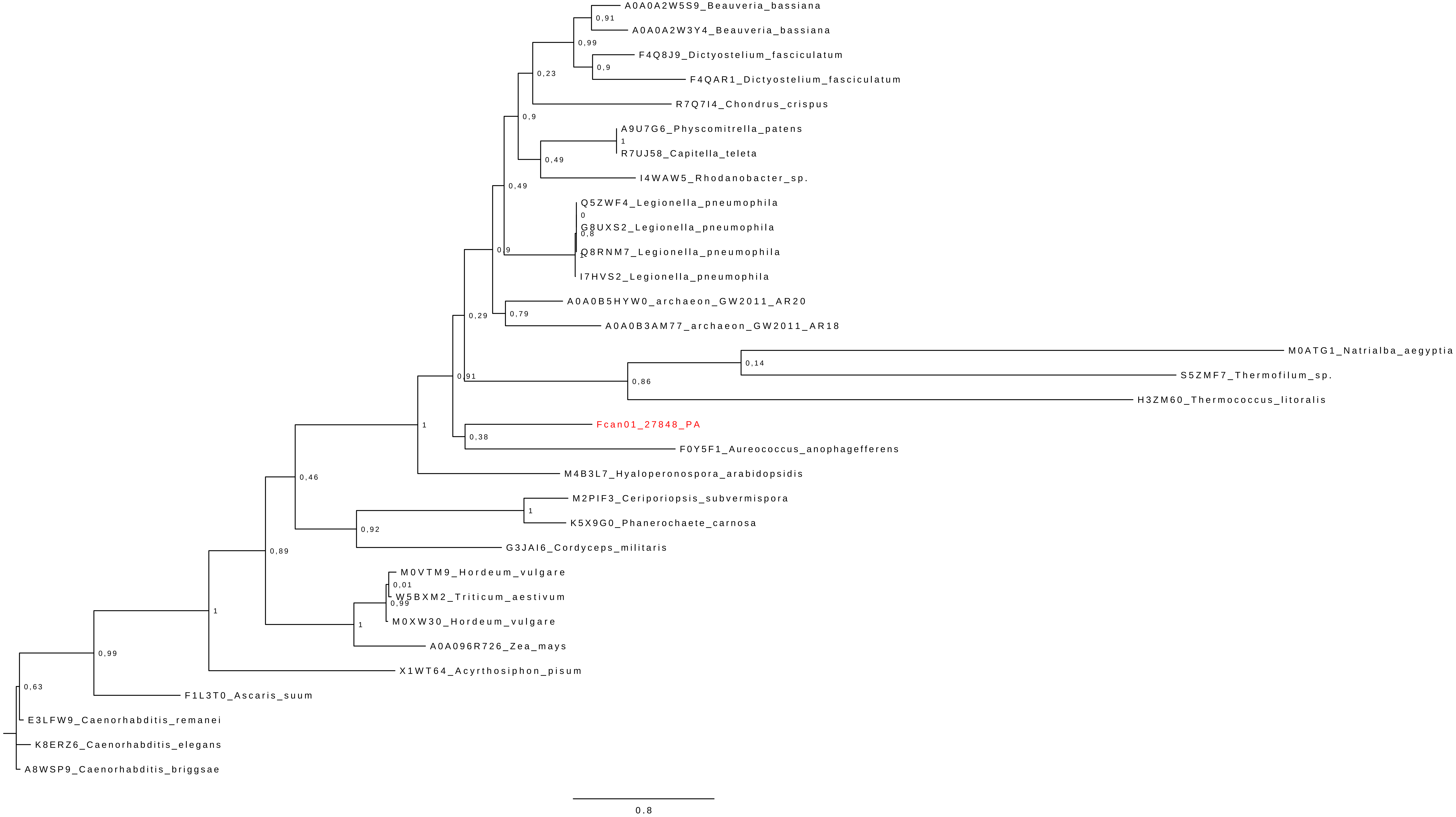
A0A0A2W5S9_Beauveria_bassiana
A0A0A2W3Y4_Beauveria_bassiana
F4Q8J9_Dictyostelium_fasciculatum
F4QAR1_Dictyostelium_fasciculatum
R7Q7I4_Chondrus_crispus
A9U7G6_Physcomitrella_patens
R7UJ58_Capitella_teleta
I4WAW5_Rhodanobacter_sp.
Q5ZWF4_Legionella_pneumophila
G8UXS2_Legionella_pneumophila
Q8RNM7_Legionella_pneumophila
I7HVS2_Legionella_pneumophila
A0A0B5HYW0_archaeon_GW2011_AR20
A0A0B3AM77_archaeon_GW2011_AR18
M0ATG1_Natrialba_aegyptia
S5ZMF7_Thermofilum_sp.
H3ZM60_Thermococcus_litoralis
Fcan01_27848_PA
F0Y5F1_Aureococcus_anophagefferens
M4B3L7_Hyaloperonospora_arabidopsidis
M2PIF3_Ceriporiopsis_subvermispora
K5X9G0_Phanerochaete_carnosa
G3JAI6_Cordyceps_militaris
M0VTM9_Hordeum_vulgare
W5BXM2_Triticum_aestivum
M0XW30_Hordeum_vulgare
A0A096R726_Zea_mays
X1WT64_Acyrthosiphon_pisum
F1L3T0_Ascaris_suum
E3LFW9_Caenorhabditis_remanei
K8ERZ6_Caenorhabditis_elegans
A8WSP9_Caenorhabditis_briggsae
0.8

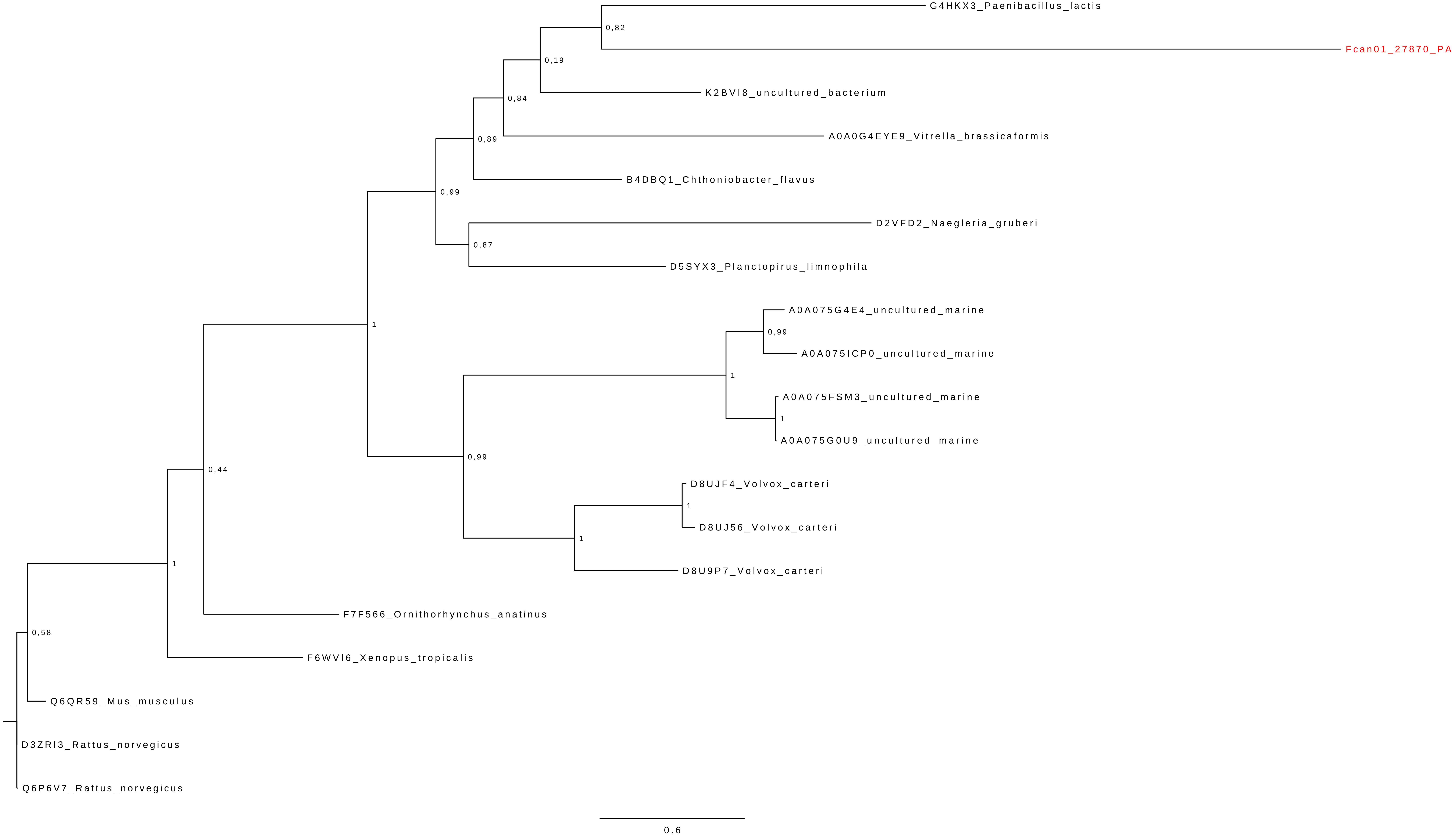
G4HKX3_Paenibacillus_lactis
0,82
Fcan01_27870_PA
0,19
K2BVI8_uncultured_bacterium
0,84
A0A0G4EYE9_Vitrella_brassicaformis
0,89
B4DBQ1_Chthoniobacter_flavus
0,99
D2VFD2_Naegleria_gruberi
0,87
D5SYX3_Planctopirus_limnophila
1
A0A075G4E4_uncultured_marine
0,99
A0A075ICP0_uncultured_marine
1
A0A075FSM3_uncultured_marine
1
A0A075G0U9_uncultured_marine
0,99
0,44
D8UJF4_Volvox_carteri
1
D8UJ56_Volvox_carteri
1
D8U9P7_Volvox_carteri
1
F7F566_Ornithorhynchus_anatinus
0,58
F6WVI6_Xenopus_tropicalis
Q6QR59_Mus_musculus
D3ZRI3_Rattus_norvegicus
Q6P6V7_Rattus_norvegicus
0.6

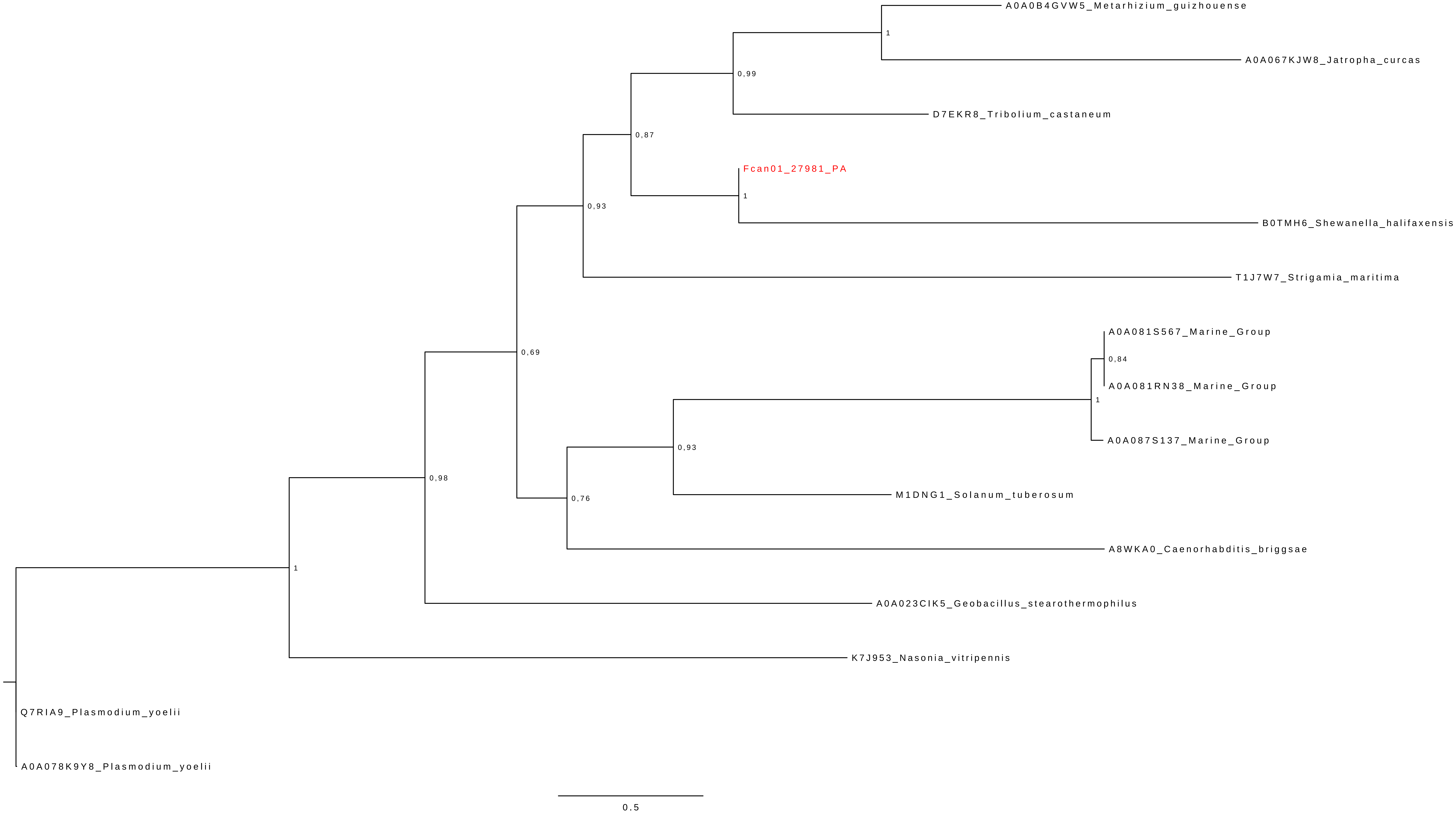

A0A0B4GVW5_Metarhizium_guizhouense
1
A0A067KJW8_Jatropha_curcas
0,99
D7EKR8_Tribolium_castaneum
0,87
Fcan01_27981_PA
1
B0TMH6_Shewanella_halifaxensis
0,93
T1J7W7_Strigamia_maritima
A0A081S567_Marine_Group
0,84
A0A081RN38_Marine_Group
1
A0A087S137_Marine_Group
0,69
0,93
M1DNG1_Solanum_tuberosum
0,98
0,76
A8WKA0_Caenorhabditis_briggsae
1
A0A023CIK5_Geobacillus_stearothermophilus
K7J953_Nasonia_vitripennis
Q7RIA9_Plasmodium_yoelii
A0A078K9Y8_Plasmodium_yoelii
0.5

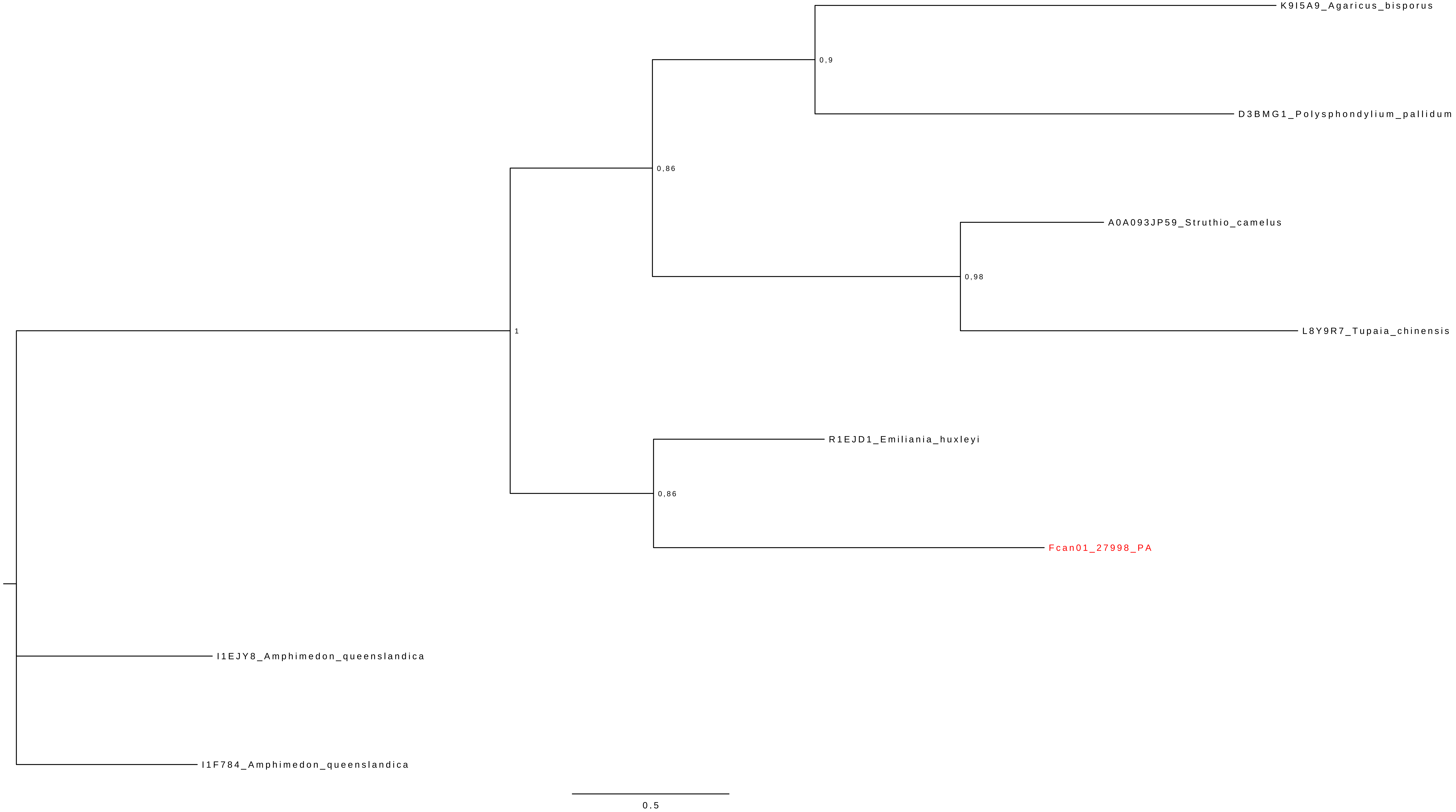
K9I5A9_Agaricus_bisporus
0,9
D3BMG1_Polysphondylium_pallidum
0,86
A0A093JP59_Struthio_camelus
0,98
L8Y9R7_Tupaia_chinensis
1
R1EJD1_Emiliania_huxleyi
0,86
Fcan01_27998_PA
I1EJY8_Amphimedon_queenslandica
I1F784_Amphimedon_queenslandica
0.5

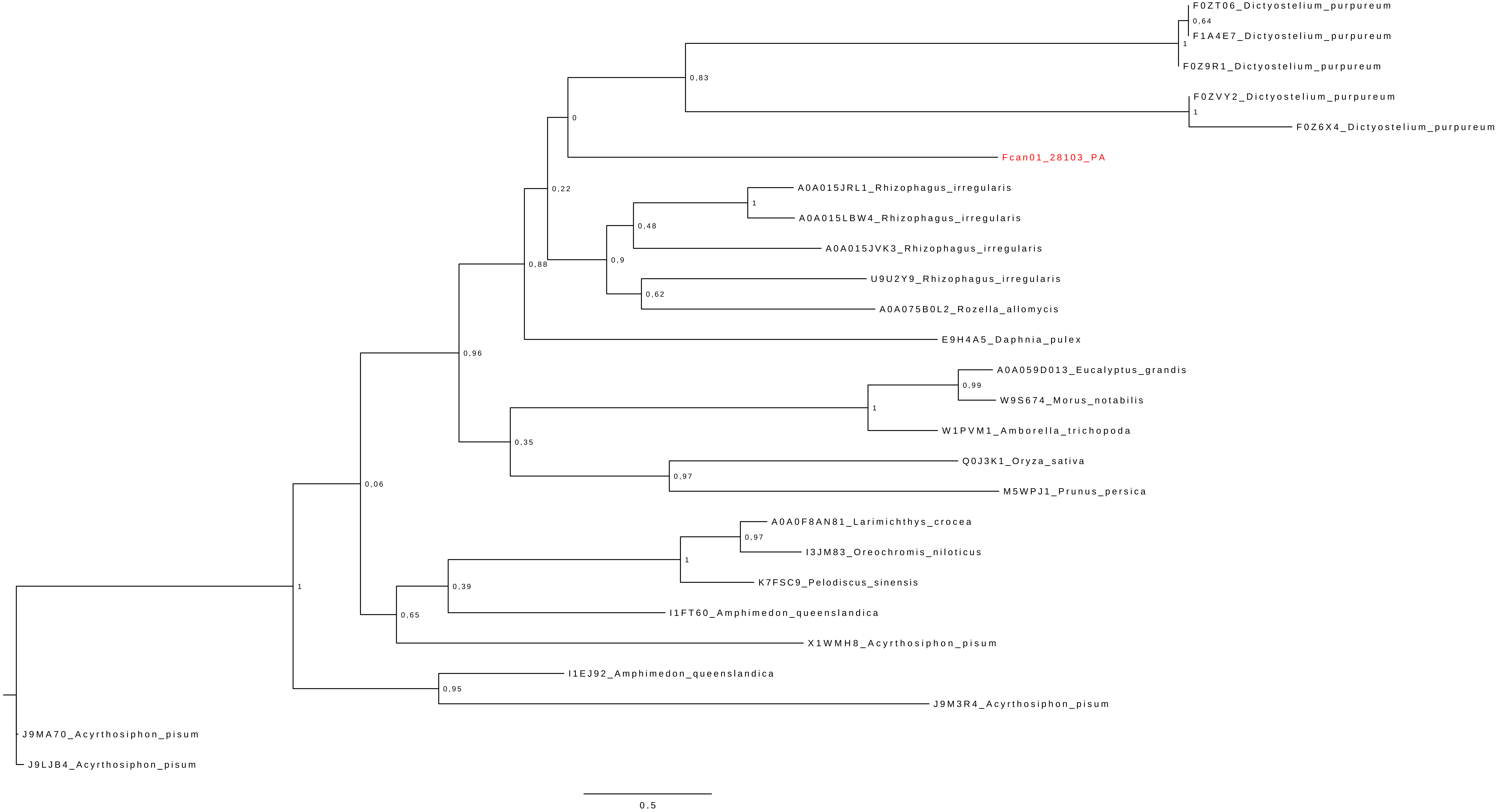
F0ZT06_Dictyostelium_purpureum
0,64
F1A4E7_Dictyostelium_purpureum
1
F0Z9R1_Dictyostelium_purpureum
0,83
F0ZVY2_Dictyostelium_purpureum
1
F0Z6X4_Dictyostelium_purpureum
0
Fcan01_28103_PA
0,22
A0A015JRL1_Rhizophagus_irregularis
1
A0A015LBW4_Rhizophagus_irregularis
0,48
A0A015JVK3_Rhizophagus_irregularis
0,9
0,88
U9U2Y9_Rhizophagus_irregularis
0,62
A0A075B0L2_Rozella_allomycis
E9H4A5_Daphnia_pulex
0,96
A0A059D013_Eucalyptus_grandis
0,99
W9S674_Morus_notabilis
1
W1PVM1_Amborella_trichopoda
0,35
Q0J3K1_Oryza_sativa
0,97
M5WPJ1_Prunus_persica
0,06
A0A0F8AN81_Larimichthys_crocea
0,97
I3JM83_Oreochromis_niloticus
1
K7FSC9_Pelodiscus_sinensis
0,39
1
I1FT60_Amphimedon_queenslandica
0,65
X1WMH8_Acyrthosiphon_pisum
I1EJ92_Amphimedon_queenslandica
0,95
J9M3R4_Acyrthosiphon_pisum
J9MA70_Acyrthosiphon_pisum
J9LJB4_Acyrthosiphon_pisum
0.5

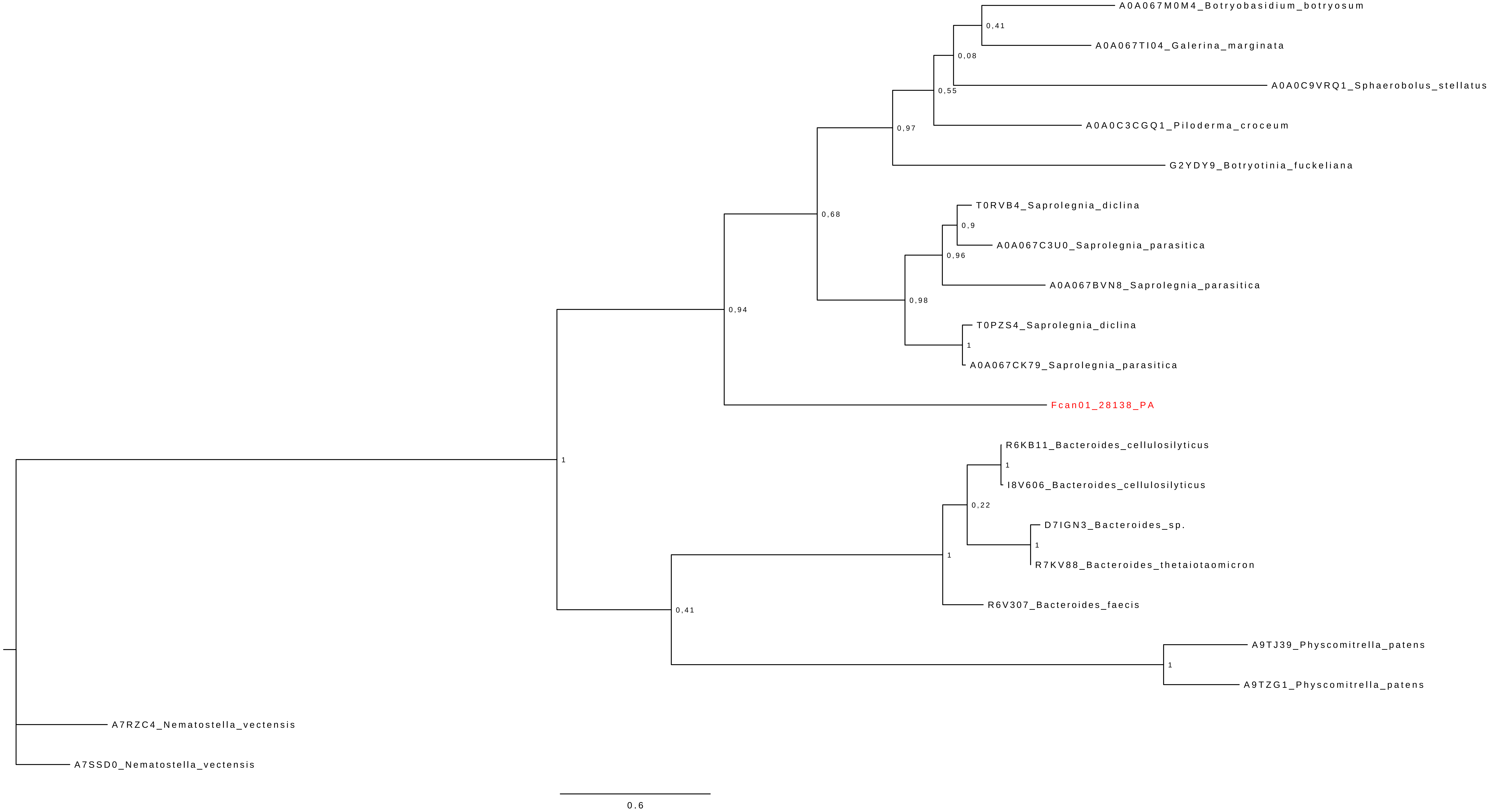
A0A067M0M4_Botryobasidium_botryosum
0,41
A0A067TI04_Galerina_marginata
0,08
A0A0C9VRQ1_Sphaerobolus_stellatus
0,55
A0A0C3CGQ1_Piloderma_croceum
0,97
G2YDY9_Botryotinia_fuckeliana
T0RVB4_Saprolegnia_diclina
0,68
0,9
A0A067C3U0_Saprolegnia_parasitica
0,96
A0A067BVN8_Saprolegnia_parasitica
0,94
0,98
T0PZS4_Saprolegnia_diclina
1
A0A067CK79_Saprolegnia_parasitica
Fcan01_28138_PA
R6KB11_Bacteroides_cellulosilyticus
1
1
I8V606_Bacteroides_cellulosilyticus
0,22
D7IGN3_Bacteroides_sp.
1
1
R7KV88_Bacteroides_thetaiotaomicron
R6V307_Bacteroides_faecis
0,41
A9TJ39_Physcomitrella_patens
1
A9TZG1_Physcomitrella_patens
A7RZC4_Nematostella_vectensis
A7SSD0_Nematostella_vectensis
0.6

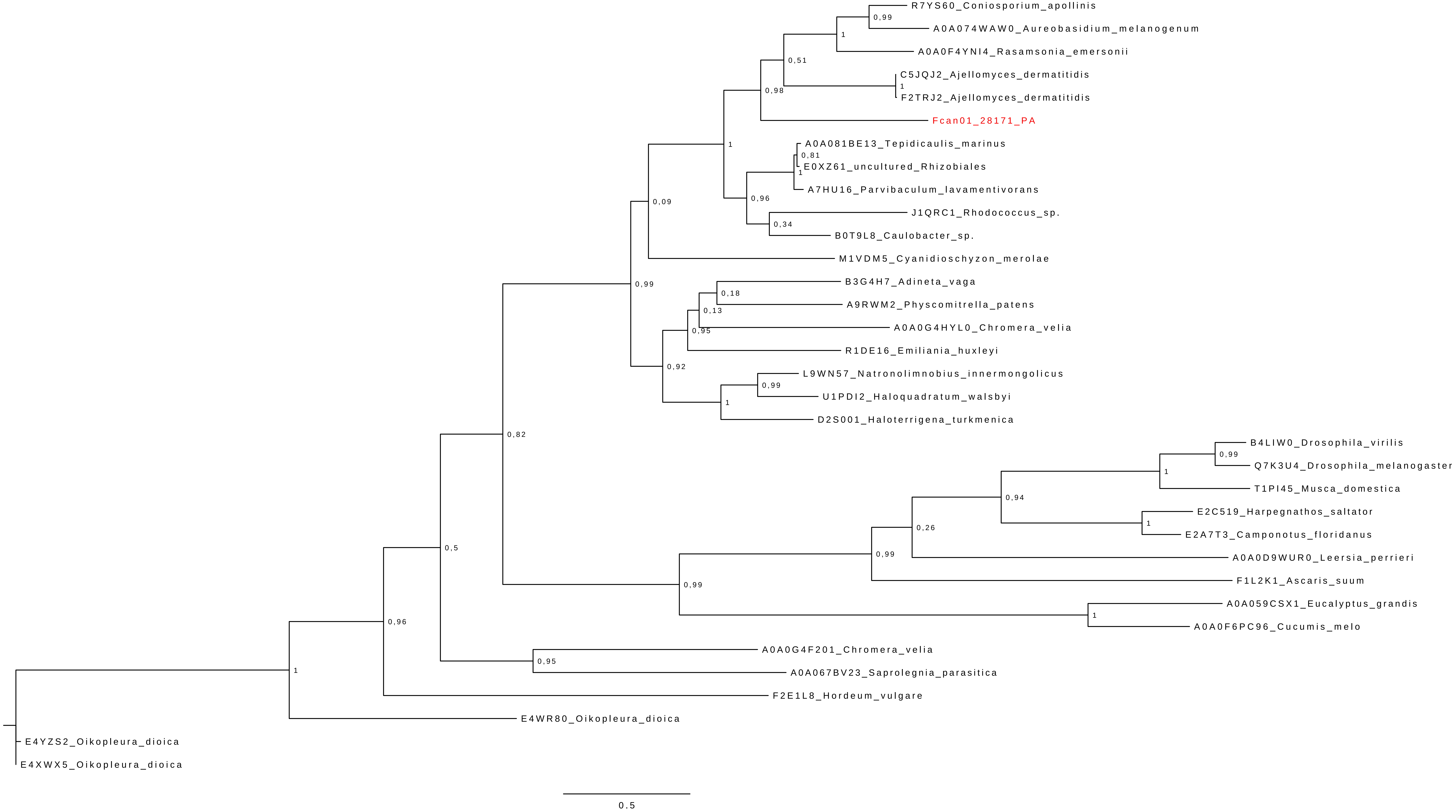
R7YS60_Coniosporium_apollinis
0,99
A0A074WAW0_Aureobasidium_melanogenum
1
A0A0F4YNI4_Rasamsonia_emersonii
0,51
C5JQJ2_Ajellomyces_dermatitidis
1
F2TRJ2_Ajellomyces_dermatitidis
0,98
Fcan01_28171_PA
1
A0A081BE13_Tepidicaulis_marinus
0,81
E0XZ61_uncultured_Rhizobiales
1
A7HU16_Parvibaculum_lavamentivorans
0,09
0,96
J1QRC1_Rhodococcus_sp.
0,34
B0T9L8_Caulobacter_sp.
M1VDM5_Cyanidioschyzon_merolae
0,99
B3G4H7_Adineta_vaga
0,18
A9RWM2_Physcomitrella_patens
0,13
A0A0G4HYL0_Chromera_velia
0,95
R1DE16_Emiliania_huxleyi
0,92
L9WN57_Natronolimnobius_innermongolicus
0,99
U1PDI2_Haloquadratum_walsbyi
1
D2S001_Haloterrigena_turkmenica
0,82
B4LIW0_Drosophila_virilis
0,99
Q7K3U4_Drosophila_melanogaster
1
T1PI45_Musca_domestica
0,94
E2C519_Harpegnathos_saltator
1
E2A7T3_Camponotus_floridanus
0,26
A0A0D9WUR0_Leersia_perrieri
0,99
F1L2K1_Ascaris_suum
0,5
0,99
A0A059CSX1_Eucalyptus_grandis
1
A0A0F6PC96_Cucumis_melo
0,96
A0A0G4F201_Chromera_velia
0,95
A0A067BV23_Saprolegnia_parasitica
1
F2E1L8_Hordeum_vulgare
E4WR80_Oikopleura_dioica
E4YZS2_Oikopleura_dioica
E4XWX5_Oikopleura_dioica
0.5

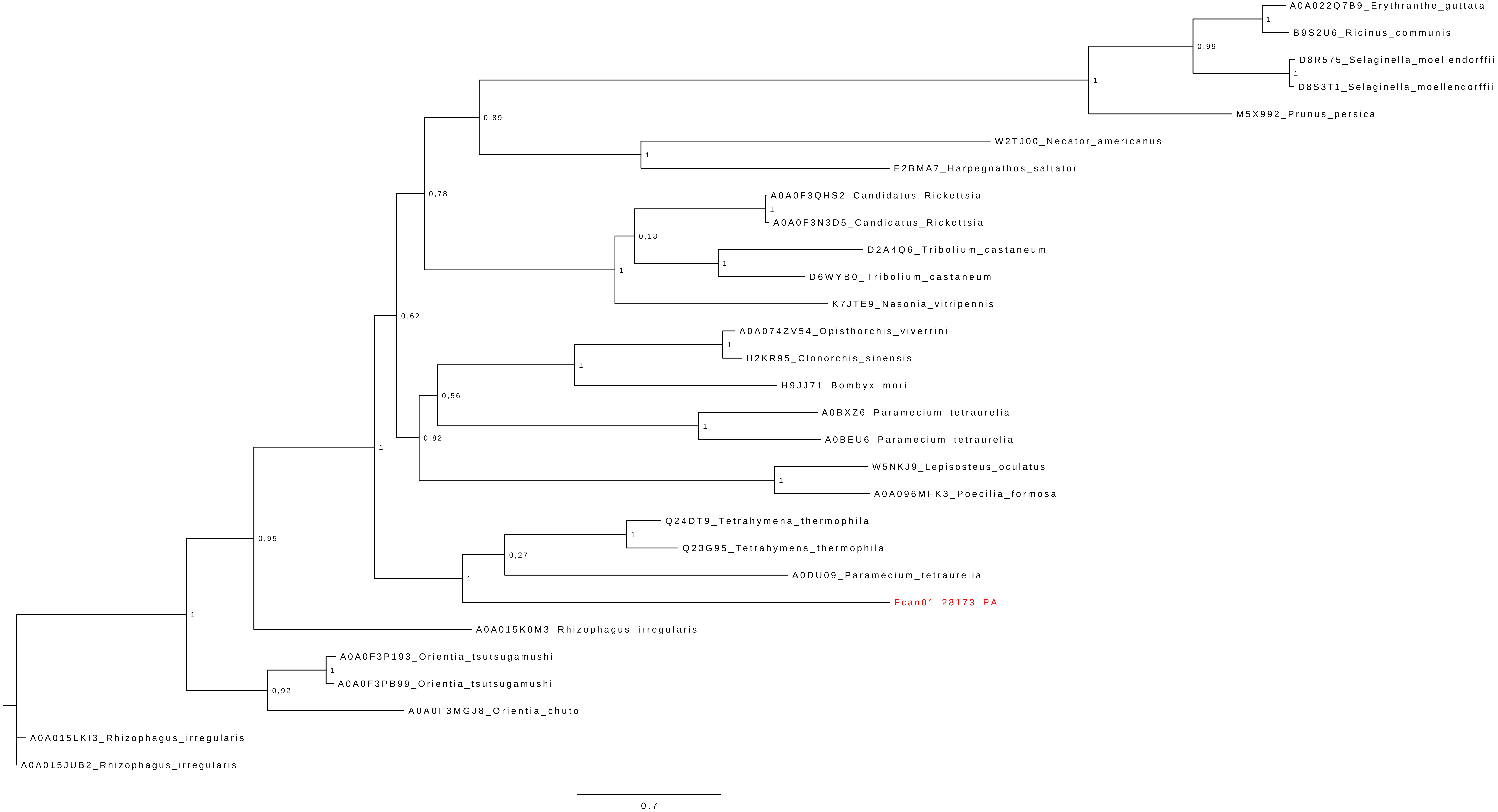
A0A022Q7B9_Erythranthe_guttata
B9S2U6_Ricinus_communis
D8R575_Selaginella_moellendorffii
D8S3T1_Selaginella_moellendorffii
M5X992_Prunus_persica
W2TJ00_Necator_americanus
E2BMA7_Harpegnathos_saltator
A0A0F3QHS2_Candidatus_Rickettsia
A0A0F3N3D5_Candidatus_Rickettsia
D2A4Q6_Tribolium_castaneum
D6WYB0_Tribolium_castaneum
K7JTE9_Nasonia_vitripennis
A0A074ZV54_Opisthorchis_viverrini
H2KR95_Clonorchis_sinensis
H9JJ71_Bombyx_mori
A0BXZ6_Paramecium_tetraurelia
A0BEU6_Paramecium_tetraurelia
W5NKJ9_Lepisosteus_oculatus
A0A096MFK3_Poecilia_formosa
Q24DT9_Tetrahymena_thermophila
Q23G95_Tetrahymena_thermophila
A0DU09_Paramecium_tetraurelia
Fcan01_28173_PA
A0A015K0M3_Rhizophagus_irregularis
A0A0F3P193_Orientia_tsutsugamushi
A0A0F3PB99_Orientia_tsutsugamushi
A0A0F3MGJ8_Orientia_chuto
A0A015LKI3_Rhizophagus_irregularis
A0A015JUB2_Rhizophagus_irregularis
0,99
0,89
0,78
0,18
0,62
0,56
0,82
0,27
0,95
0,92
0.7

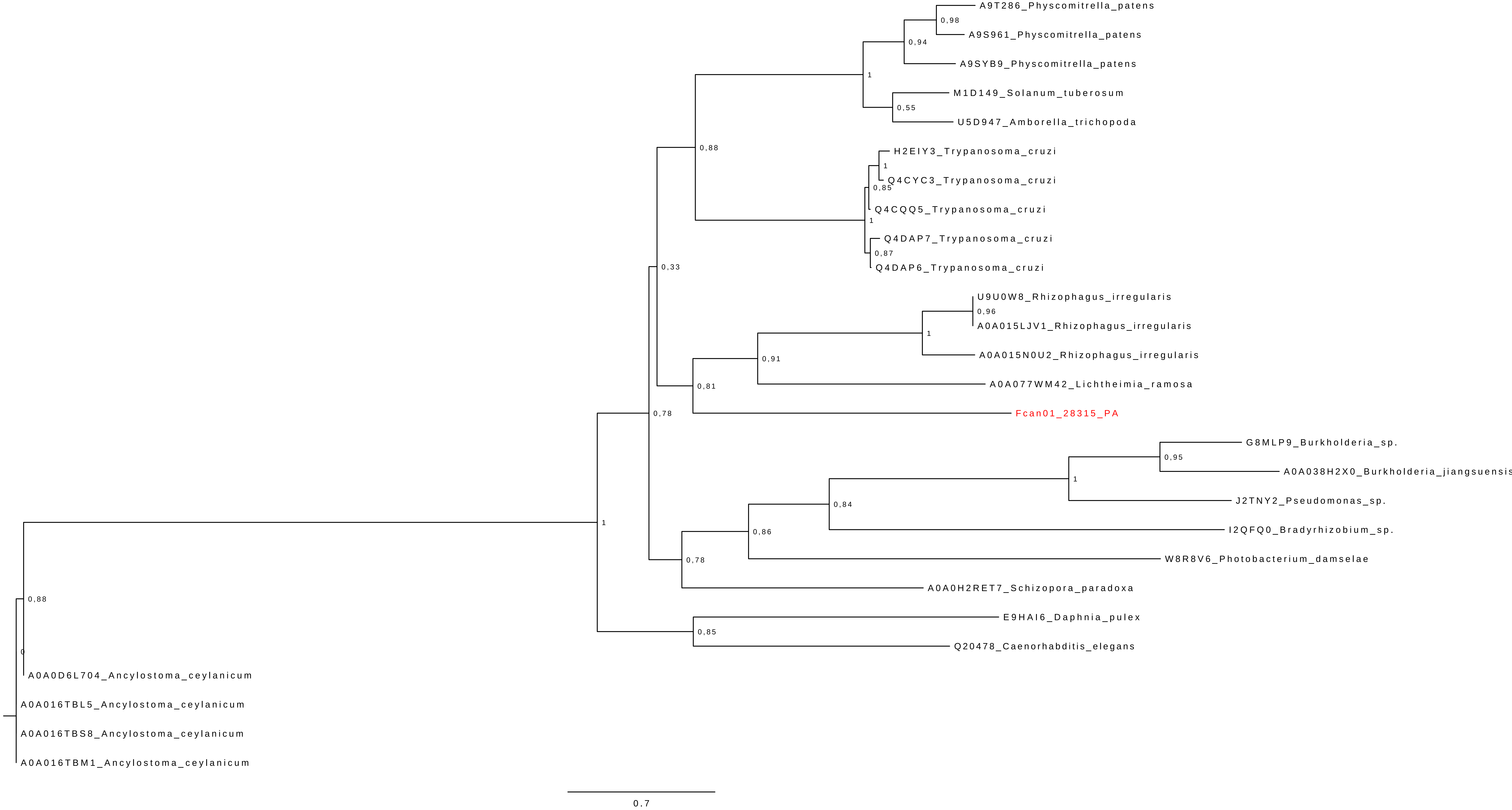

A9T286_Physcomitrella_patens
0,98
A9S961_Physcomitrella_patens
0,94
A9SYB9_Physcomitrella_patens
1
M1D149_Solanum_tuberosum
0,55
U5D947_Amborella_trichopoda
0,88
H2EIY3_Trypanosoma_cruzi
1
Q4CYC3_Trypanosoma_cruzi
0,85
Q4CQQ5_Trypanosoma_cruzi
1
Q4DAP7_Trypanosoma_cruzi
0,87
Q4DAP6_Trypanosoma_cruzi
0,33
U9U0W8_Rhizophagus_irregularis
0,96
A0A015LJV1_Rhizophagus_irregularis
1
A0A015N0U2_Rhizophagus_irregularis
0,91
A0A077WM42_Lichtheimia_ramosa
0,81
Fcan01_28315_PA
0,78
G8MLP9_Burkholderia_sp.
0,95
A0A038H2X0_Burkholderia_jiangsuensis
1
J2TNY2_Pseudomonas_sp.
0,84
I2QFQ0_Bradyrhizobium_sp.
1
0,86
W8R8V6_Photobacterium_damselae
0,78
A0A0H2RET7_Schizopora_paradoxa
0,88
E9HAI6_Daphnia_pulex
0,85
Q20478_Caenorhabditis_elegans
0
A0A0D6L704_Ancylostoma_ceylanicum
A0A016TBL5_Ancylostoma_ceylanicum
A0A016TBS8_Ancylostoma_ceylanicum
A0A016TBM1_Ancylostoma_ceylanicum
0.7

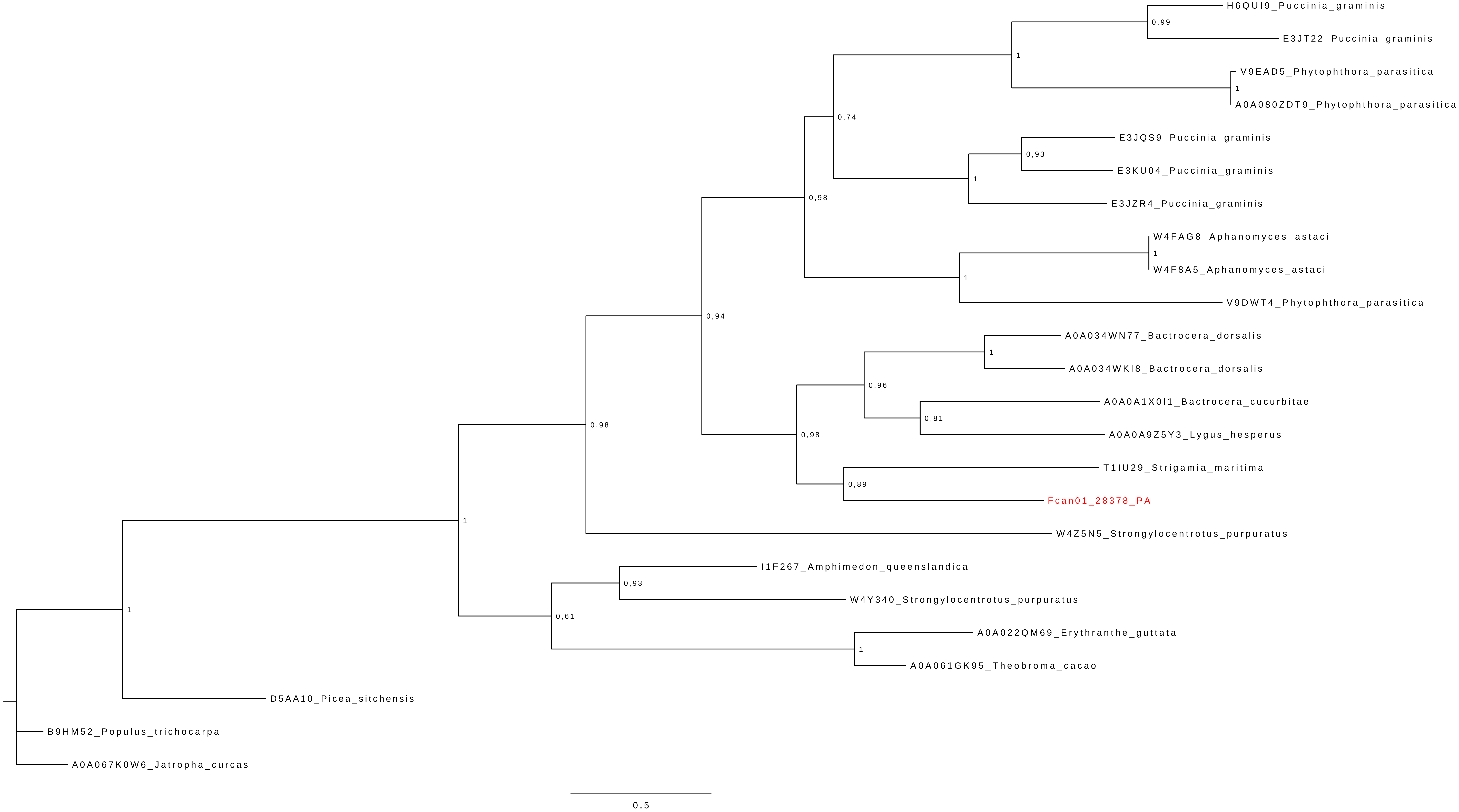
H6QUI9_Puccinia_graminis
0,99
E3JT22_Puccinia_graminis
1
V9EAD5_Phytophthora_parasitica
1
A0A080ZDT9_Phytophthora_parasitica
0,74
E3JQS9_Puccinia_graminis
0,93
E3KU04_Puccinia_graminis
1
E3JZR4_Puccinia_graminis
0,98
W4FAG8_Aphanomyces_astaci
1
W4F8A5_Aphanomyces_astaci
1
V9DWT4_Phytophthora_parasitica
0,94
A0A034WN77_Bactrocera_dorsalis
1
A0A034WKI8_Bactrocera_dorsalis
0,96
A0A0A1X0I1_Bactrocera_cucurbitae
0,81
A0A0A9Z5Y3_Lygus_hesperus
0,98
0,98
T1IU29_Strigamia_maritima
0,89
Fcan01_28378_PA
W4Z5N5_Strongylocentrotus_purpuratus
1
I1F267_Amphimedon_queenslandica
0,93
W4Y340_Strongylocentrotus_purpuratus
1
0,61
A0A022QM69_Erythranthe_guttata
1
A0A061GK95_Theobroma_cacao
D5AA10_Picea_sitchensis
B9HM52_Populus_trichocarpa
A0A067K0W6_Jatropha_curcas
0.5

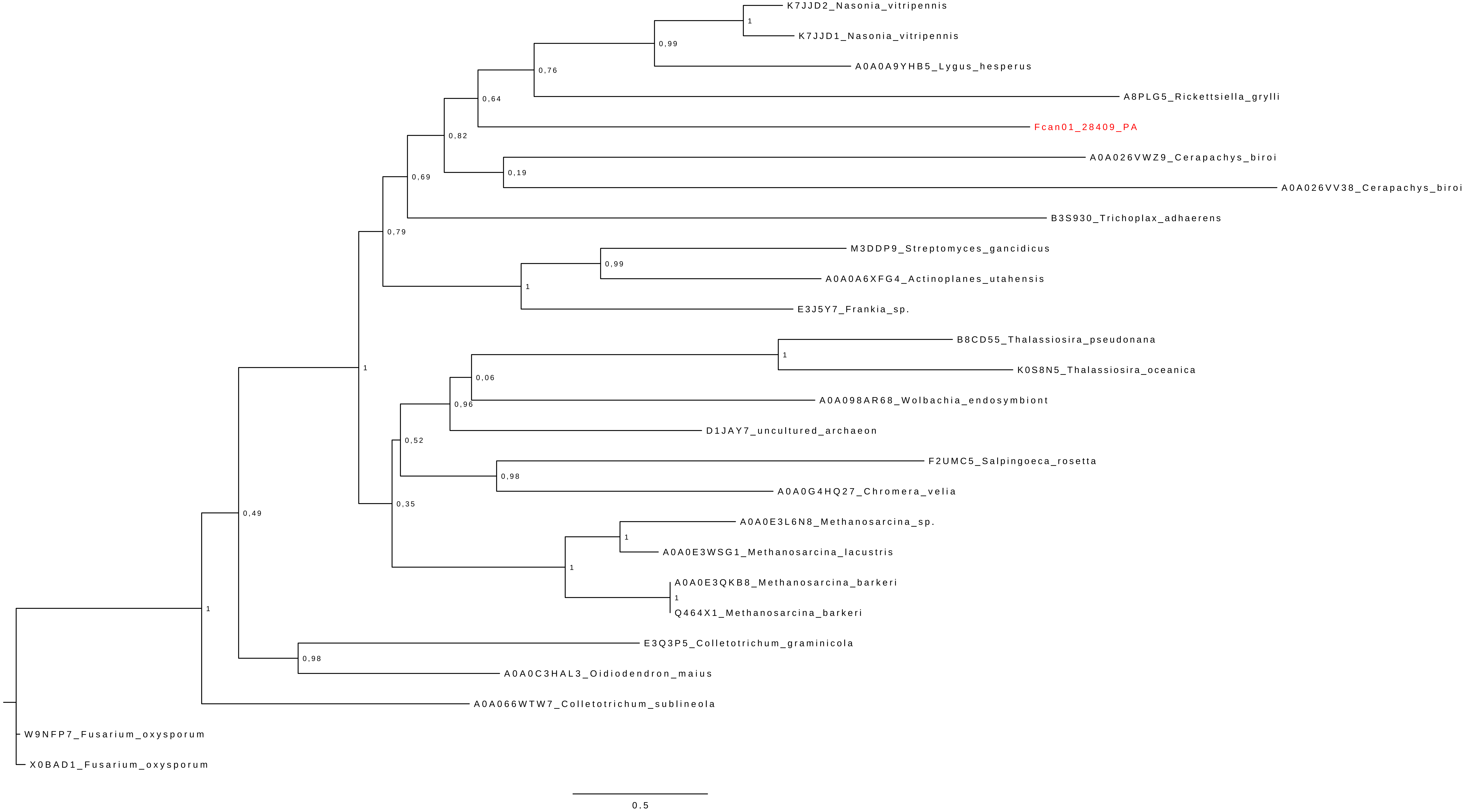
K7JJD2_Nasonia_vitripennis
1
K7JJD1_Nasonia_vitripennis
0,99
A0A0A9YHB5_Lygus_hesperus
0,76
A8PLG5_Rickettsiella_grylli
0,64
Fcan01_28409_PA
0,82
A0A026VWZ9_Cerapachys_biroi
0,19
A0A026VV38_Cerapachys_biroi
0,69
B3S930_Trichoplax_adhaerens
0,79
M3DDP9_Streptomyces_gancidicus
0,99
A0A0A6XFG4_Actinoplanes_utahensis
1
E3J5Y7_Frankia_sp.
B8CD55_Thalassiosira_pseudonana
1
K0S8N5_Thalassiosira_oceanica
1
0,06
A0A098AR68_Wolbachia_endosymbiont
0,96
D1JAY7_uncultured_archaeon
0,52
F2UMC5_Salpingoeca_rosetta
0,98
A0A0G4HQ27_Chromera_velia
0,49
0,35
A0A0E3L6N8_Methanosarcina_sp.
1
A0A0E3WSG1_Methanosarcina_lacustris
1
A0A0E3QKB8_Methanosarcina_barkeri
1
1
Q464X1_Methanosarcina_barkeri
E3Q3P5_Colletotrichum_graminicola
0,98
A0A0C3HAL3_Oidiodendron_maius
A0A066WTW7_Colletotrichum_sublineola
W9NFP7_Fusarium_oxysporum
X0BAD1_Fusarium_oxysporum
0.5

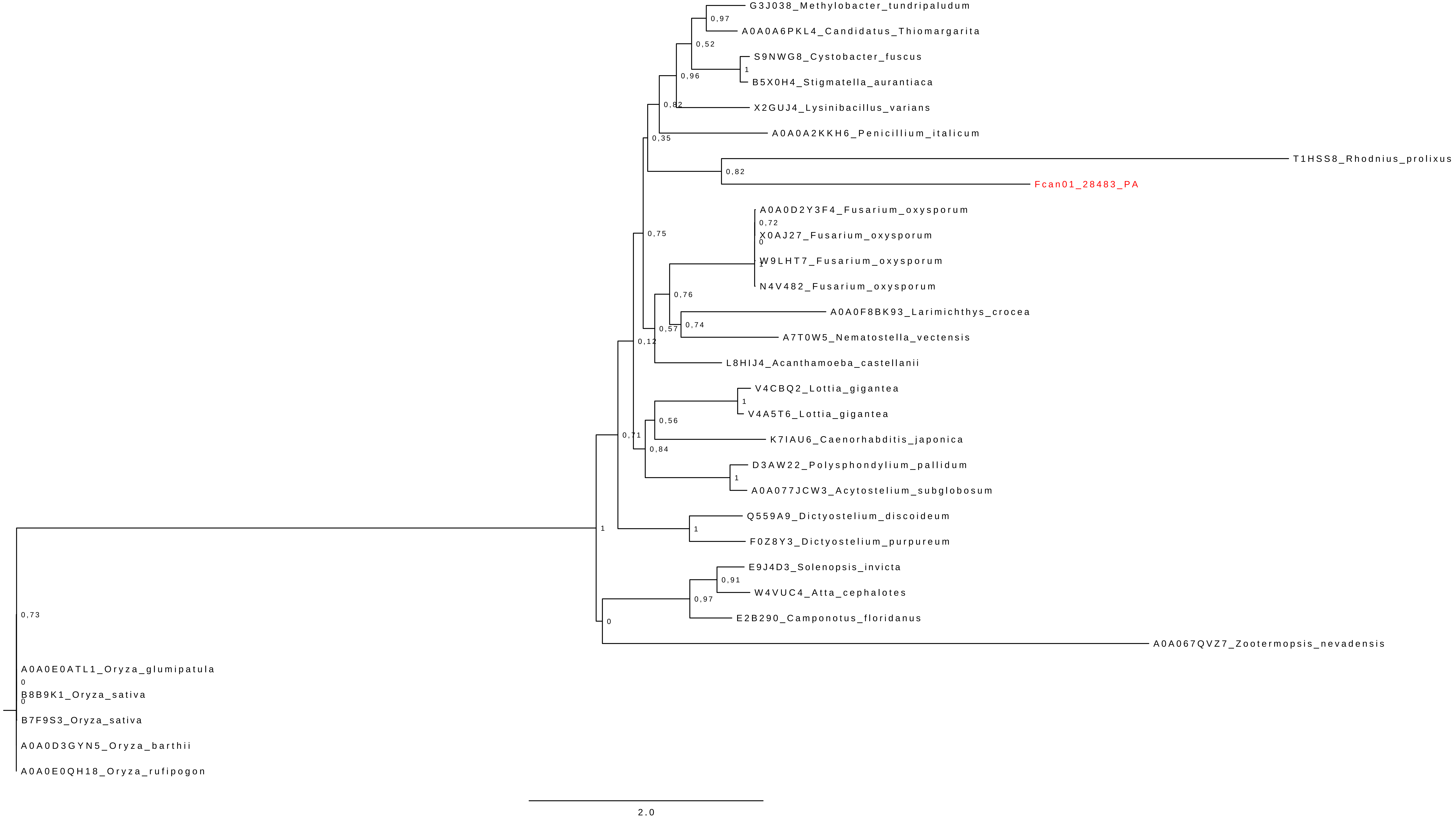

G3J038_Methylobacter_tundripaludum
0,97
A0A0A6PKL4_Candidatus_Thiomargarita
0,52
S9NWG8_Cystobacter_fuscus
1
B5X0H4_Stigmatella_aurantiaca
0,96
0,82
X2GUJ4_Lysinibacillus_varians
A0A0A2KKH6_Penicillium_italicum
0,35
T1HSS8_Rhodnius_prolixus
0,82
Fcan01_28483_PA
A0A0D2Y3F4_Fusarium_oxysporum
0,72
X0AJ27_Fusarium_oxysporum
0
W9LHT7_Fusarium_oxysporum
N4V482_Fusarium_oxysporum
0,75
0,76
A0A0F8BK93_Larimichthys_crocea
0,74
0,57
A7T0W5_Nematostella_vectensis
0,12
L8HIJ4_Acanthamoeba_castellanii
V4CBQ2_Lottia_gigantea
1
V4A5T6_Lottia_gigantea
0,56
0,71
K7IAU6_Caenorhabditis_japonica
0,84
D3AW22_Polysphondylium_pallidum
1
A0A077JCW3_Acytostelium_subglobosum
Q559A9_Dictyostelium_discoideum
1
1
F0Z8Y3_Dictyostelium_purpureum
E9J4D3_Solenopsis_invicta
0,91
W4VUC4_Atta_cephalotes
0,97
E2B290_Camponotus_floridanus
0
A0A067QVZ7_Zootermopsis_nevadensis
0,73
A0A0E0ATL1_Oryza_glumipatula
0
B8B9K1_Oryza_sativa
0
B7F9S3_Oryza_sativa
A0A0D3GYN5_Oryza_barthii
A0A0E0QH18_Oryza_rufipogon
2.0

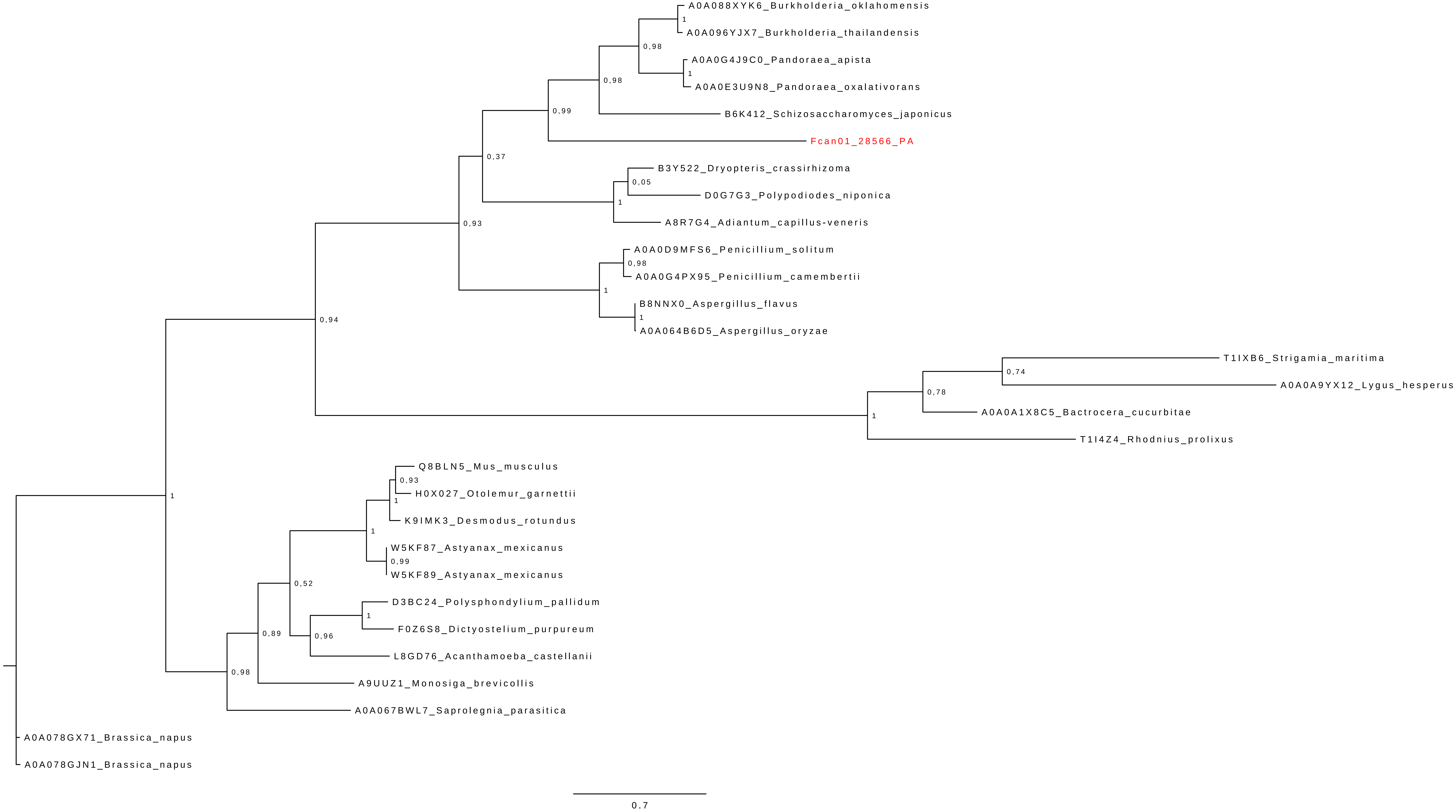
A0A088XYK6_Burkholderia_oklahomensis
1
A0A096YJX7_Burkholderia_thailandensis
0,98
A0A0G4J9C0_Pandoraea_apista
1
A0A0E3U9N8_Pandoraea_oxalativorans
0,98
B6K412_Schizosaccharomyces_japonicus
0,99
Fcan01_28566_PA
0,37
B3Y522_Dryopteris_crassirhizoma
0,05
D0G7G3_Polypodiodes_niponica
1
A8R7G4_Adiantum_capillus-veneris
0,93
A0A0D9MFS6_Penicillium_solitum
0,98
A0A0G4PX95_Penicillium_camembertii
1
B8NNX0_Aspergillus_flavus
1
A0A064B6D5_Aspergillus_oryzae
0,94
T1IXB6_Strigamia_maritima
0,74
A0A0A9YX12_Lygus_hesperus
0,78
A0A0A1X8C5_Bactrocera_cucurbitae
1
T1I4Z4_Rhodnius_prolixus
Q8BLN5_Mus_musculus
0,93
H0X027_Otolemur_garnettii
1
K9IMK3_Desmodus_rotundus
1
1
W5KF87_Astyanax_mexicanus
0,99
W5KF89_Astyanax_mexicanus
0,52
D3BC24_Polysphondylium_pallidum
1
F0Z6S8_Dictyostelium_purpureum
0,89
0,96
L8GD76_Acanthamoeba_castellanii
0,98
A9UUZ1_Monosiga_brevicollis
A0A067BWL7_Saprolegnia_parasitica
A0A078GX71_Brassica_napus
A0A078GJN1_Brassica_napus
0.7

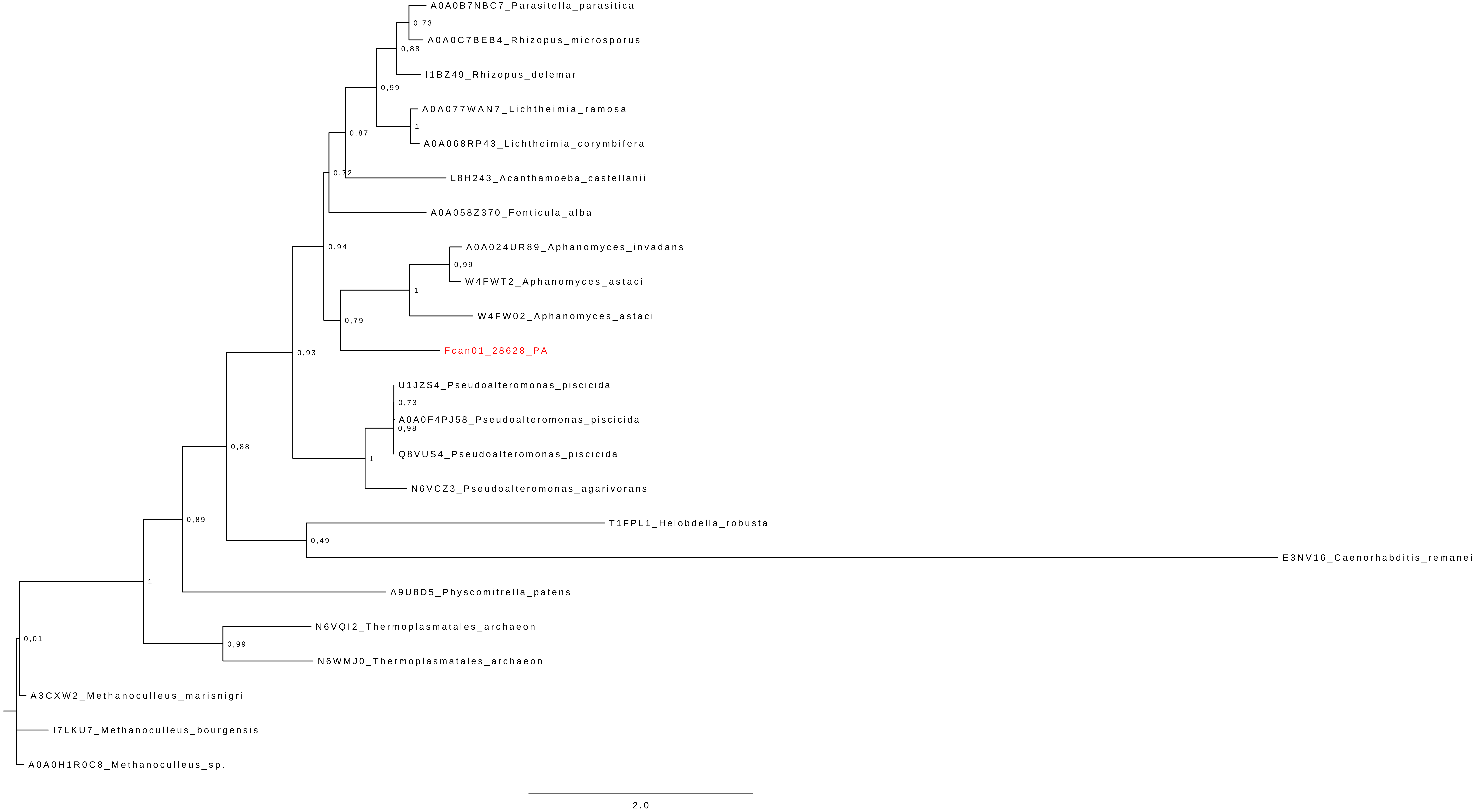
A0A0B7NBC7_Parasitella_parasitica
0,73
A0A0C7BEB4_Rhizopus_microsporus
0,88
I1BZ49_Rhizopus_delemar
0,99
A0A077WAN7_Lichtheimia_ramosa
1
A0A068RP43_Lichtheimia_corymbifera
0,87
L8H243_Acanthamoeba_castellanii
0,72
A0A058Z370_Fonticula_alba
0,94
A0A024UR89_Aphanomyces_invadans
0,99
W4FWT2_Aphanomyces_astaci
1
W4FW02_Aphanomyces_astaci
0,79
Fcan01_28628_PA
0,93
U1JZS4_Pseudoalteromonas_piscicida
0,73
A0A0F4PJ58_Pseudoalteromonas_piscicida
0,98
Q8VUS4_Pseudoalteromonas_piscicida
1
N6VCZ3_Pseudoalteromonas_agarivorans
0,88
T1FPL1_Helobdella_robusta
0,89
0,49
E3NV16_Caenorhabditis_remanei
1
A9U8D5_Physcomitrella_patens
N6VQI2_Thermoplasmatales_archaeon
0,01
0,99
N6WMJ0_Thermoplasmatales_archaeon
A3CXW2_Methanoculleus_marisnigri
I7LKU7_Methanoculleus_bourgensis
A0A0H1R0C8_Methanoculleus_sp.
2.0

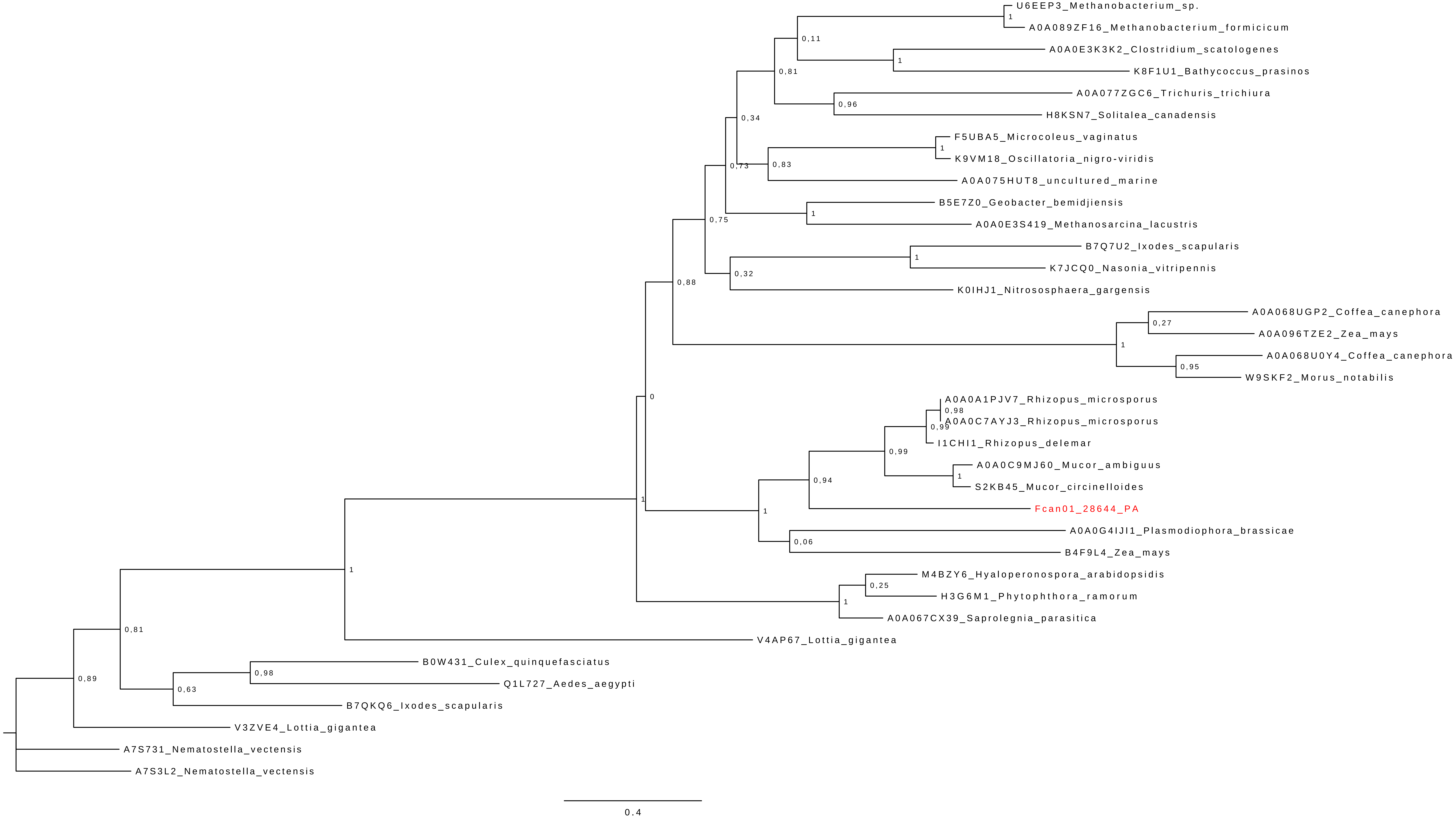
U6EEP3_Methanobacterium_sp.
A0A089ZF16_Methanobacterium_formicicum
A0A0E3K3K2_Clostridium_scatologenes
K8F1U1_Bathycoccus_prasinos
A0A077ZGC6_Trichuris_trichiura
H8KSN7_Solitalea_canadensis
F5UBA5_Microcoleus_vaginatus
K9VM18_Oscillatoria_nigro-viridis
A0A075HUT8_uncultured_marine
B5E7Z0_Geobacter_bemidjiensis
A0A0E3S419_Methanosarcina_lacustris
B7Q7U2_Ixodes_scapularis
K7JCQ0_Nasonia_vitripennis
K0IHJ1_Nitrososphaera_gargensis
A0A068UGP2_Coffea_canephora
A0A096TZE2_Zea_mays
A0A068U0Y4_Coffea_canephora
W9SKF2_Morus_notabilis
A0A0A1PJV7_Rhizopus_microsporus
A0A0C7AYJ3_Rhizopus_microsporus
I1CHI1_Rhizopus_delemar
A0A0C9MJ60_Mucor_ambiguus
S2KB45_Mucor_circinelloides
Fcan01_28644_PA
A0A0G4IJI1_Plasmodiophora_brassicae
B4F9L4_Zea_mays
M4BZY6_Hyaloperonospora_arabidopsidis
H3G6M1_Phytophthora_ramorum
A0A067CX39_Saprolegnia_parasitica
V4AP67_Lottia_gigantea
B0W431_Culex_quinquefasciatus
Q1L727_Aedes_aegypti
B7QKQ6_Ixodes_scapularis
V3ZVE4_Lottia_gigantea
A7S731_Nematostella_vectensis
A7S3L2_Nematostella_vectensis
0.4

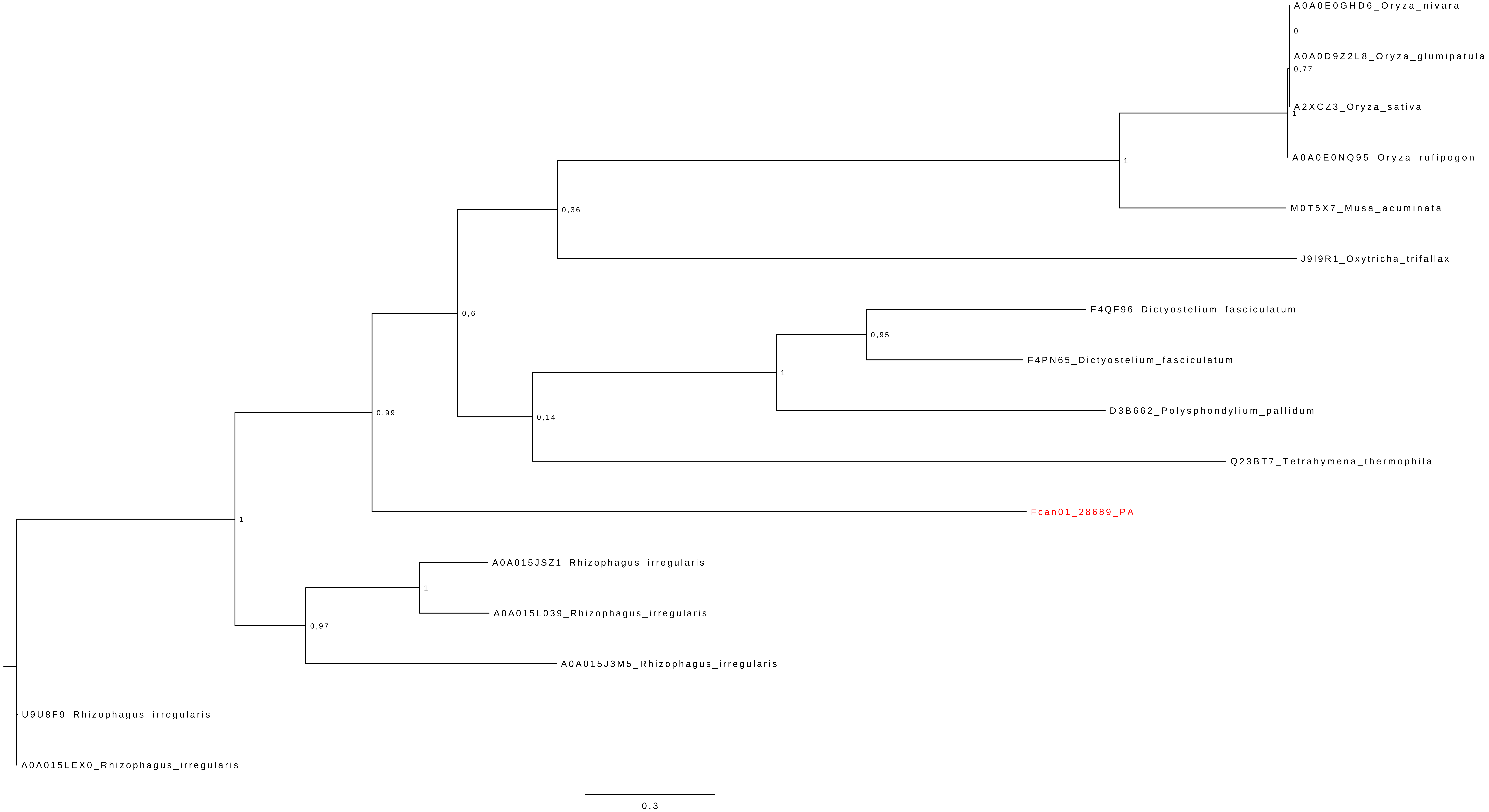
A0A0E0GHD6_Oryza_nivara
0
A0A0D9Z2L8_Oryza_glumipatula
0,77
A2XCZ3_Oryza_sativa
1
A0A0E0NQ95_Oryza_rufipogon
1
M0T5X7_Musa_acuminata
0,36
J9I9R1_Oxytricha_trifallax
F4QF96_Dictyostelium_fasciculatum
0,95
F4PN65_Dictyostelium_fasciculatum
0,6
1
D3B662_Polysphondylium_pallidum
0,99
0,14
Q23BT7_Tetrahymena_thermophila
Fcan01_28689_PA
1
A0A015JSZ1_Rhizophagus_irregularis
1
A0A015L039_Rhizophagus_irregularis
0,97
A0A015J3M5_Rhizophagus_irregularis
U9U8F9_Rhizophagus_irregularis
A0A015LEX0_Rhizophagus_irregularis
0.3
